# Phase I Clinical Trial of a Recombinant Blood Stage Vaccine Candidate for *Plasmodium falciparum* Malaria Based on MSP1 and EBA175

**DOI:** 10.1371/journal.pone.0117820

**Published:** 2015-04-30

**Authors:** Chetan E. Chitnis, Paushali Mukherjee, Shantanu Mehta, Syed Shams Yazdani, Shikha Dhawan, Ahmad Rushdi Shakri, Rukmini Bharadwaj, Puneet Kumar Gupta, Dhiraj Hans, Suman Mazumdar, Bijender Singh, Sanjeev Kumar, Gaurav Pandey, Varsha Parulekar, Nathalie Imbault, Preethi Shivyogi, Girish Godbole, Krishna Mohan, Odile Leroy, Kavita Singh, Virander S. Chauhan

**Affiliations:** 1 International Centre for Genetic Engineering and Biotechnology (ICGEB), New Delhi, India; 2 Malaria Vaccine Development Program (MVDP), New Delhi, India; 3 European Vaccine Initiative (EVI), Heidelberg, Germany; 4 Bharat Biotech International Ltd. (BBIL), Hyderabad, India; 5 Lotus Laboratories, Bangalore, India; 6 Diagnosearch Laboratory Services, Mumbai, India; Aeras, UNITED STATES

## Abstract

**Background:**

A phase I randomised, controlled, single blind, dose escalation trial was conducted to evaluate safety and immunogenicity of JAIVAC-1, a recombinant blood stage vaccine candidate against *Plasmodium falciparum* malaria, composed of a physical mixture of two recombinant proteins, PfMSP-1_19_, the 19 kD conserved, C-terminal region of PfMSP-1 and PfF2 the receptor-binding F2 domain of EBA175.

**Method:**

Healthy malaria naïve Indian male subjects aged 18–45 years were recruited from the volunteer database of study site. Fifteen subjects in each cohort, randomised in a ratio of 2:1 and meeting the protocol specific eligibility criteria, were vaccinated either with three doses (10μg, 25μg and 50μg of each antigen) of JAIVAC-1 formulated with adjuvant Montanide ISA 720 or with standard dosage of Hepatitis B vaccine. Each subject received the assigned vaccine in the deltoid muscle of the upper arms on Day 0, Day 28 and Day 180.

**Results:**

JAIVAC-1 was well tolerated and no serious adverse event was observed. All JAIVAC-1 subjects sero-converted for PfF2 but elicited poor immune response to PfMSP-1_19_. Dose-response relationship was observed between vaccine dose of PfF2 and antibody response. The antibodies against PfF2 were predominantly of IgG1 and IgG3 isotype. Sera from JAIVAC-1 subjects reacted with late schizonts in a punctate pattern in immunofluorescence assays. Purified IgG from JAIVAC-1 sera displayed significant growth inhibitory activity against *Plasmodium falciparum* CAMP strain.

**Conclusion:**

Antigen PfF2 should be retained as a component of a recombinant malaria vaccine but PfMSP-1_19_ construct needs to be optimised to improve its immunogenicity.

**Trial Registration:**

Clinical Trial Registry, India CTRI/2010/091/000301

## Introduction

In 2010, malaria caused an estimated 219 million clinical malaria cases, which resulted in ~660,000 deaths worldwide. Most of the deaths were caused by *Plasmodium (P*.*) falciparum* infections [1]. Various control measures have helped reduce the number of malaria cases but many of the tools employed such as drugs and insecticides are vulnerable to development of resistance. The availability of an effective vaccine is a critical tool for sustainable control and eventual elimination of malaria from endemic regions [2].

During the blood stage of its life cycle, *Plasmodium* merozoites invade and multiply within host erythrocytes. Parasite proteins that mediate erythrocyte binding and invasion are considered attractive candidates for blood stage malaria vaccines since antibodies directed against such parasite ligands may block erythrocyte invasion, limit parasite multiplication and thereby provide protection against malaria [3]. The erythrocyte binding antigen 175 kDa (EBA-175) is one of the high-affinity ligands that binds sialic acid residues of glycophorin A on the red cell surface to mediate invasion [4]. The amino-terminal, conserved, cysteine-rich region of EBA-175, referred to as PfF2, contains receptor-binding sites for glycophorin A [4–6]. Antibodies directed against the PfF2 region block binding of EBA-175 to erythrocytes and inhibit parasite growth *in vitro* [6]. Merozoite surface protein-1 (195 kD MSP-1) is also thought to play an important role in RBC invasion [7]. PfMSP-1 contains a C-terminal, conserved cysteine-rich region, referred to as PfMSP-1_19_ that is retained on the surface of merozoites during invasion while rest of PfMSP-1 is proteolytically cleaved and shed [8]. Naturally acquired antibodies against PfMSP-1_19_ that inhibit erythrocyte invasion by preventing the proteolytic processing of PfMSP-1 are associated with protection against clinical malaria [9–12].

Previous clinical studies to evaluate vaccine potential of PfMSP-1 have tested constructs based on PfMSP-1_19_ as well as larger C-terminal constructs based on a 42 kD C-terminal fragment (PfMSP-1_42_). A Phase I trial with recombinant PfMSP-1_19_ fused to T-helper (T_h_) epitopes from tetanus toxoid formulated with alhydrogel demonstrated generation of specific antibodies although the trial was discontinued due to hypersensitivity reactions in some of the subjects [13]. Other trials have utilized a larger C-terminal fragment, MSP-1_42_ that exhibits greater immunogenicity [14]. Recombinant PfMSP-1_42_ formulated with AS02A elicited high antibody titers but failed to reduce parasite densities and overall incidence of clinical malaria episodes in young children in Western Kenya [15]. In another approach immunization with recombinant chimpanzee adenovirus (ChAd63) and modified vaccina virus (MVA) based vectors designed to deliver PfMSP-1_42_ in a heterologous prime-boost immunization regime induced PfMSP1-specific antibody responses [16, 17]. The receptor-binding domain EBA175 has also been tested in a Phase I clinical trial. Recombinant EBA175 binding domain formulated with aluminum phosphate was immunogenic in humans and elicited invasion inhibitory antibodies [18].

We have conducted a phase I clinical trial with blood stage malaria vaccine, JAIVAC-1, which is composed of a mixture of recombinant PfF2, the binding domain of EBA175 from *P*. *falciparum* CAMP strain, and PfMSP-1_19_, the C-terminal conserved region of PfMSP-1 derived from *P*. *falciparum* FVO strain, formulated with the adjuvant Montanide ISA720. Montanide 720 is a biodegradable, water-in-oil emulsion composed of mineral oil mixed with surfactant mannide mono-oleate in a 1:1 ratio [19–21]. It exerts adjuvant effects by several mechanisms including the slow release of antigen, recruitment and stimulation of antigen presenting cells, and diffusion of oil droplets to draining lymph nodes [22]. Pre-clinical immunogenicity studies have demonstrated that JAIVAC-1 formulated with Montanide ISA720 elicits balanced antibody responses against both PfF2 and PfMSP-1_19_ and exhibits significant growth inhibitory activity against *P*. *falciparum* [unpublished data]. Here, we report the safety and immunogenicity results of a first-in-man phase I clinical trial conducted in healthy young Indian adults with JAIVAC-1 formulated with Montanide ISA720.

## Materials and Methods

The protocol, pre-screening information sheet and informed consent form, main subject information sheet and informed consent form, final analysis listings, subject diary card and supporting CONSORT checklist are available as supporting information: see S1 Protocol, S1 Pre-screening Information Sheet and Informed Consent Form, S1 Main Subject Information Sheet and Informed Consent Form, S1 Final Analysis Listings, S1 Subject Diary Card and S1 CONSORT checklist.

### Ethics, Monitoring and Approvals

The study was approved by the “Institutional Ethics Committee (IEC) of International Centre for Genetic Engineering and Biotechnology (ICGEB), New Delhi” and the “Independent Ethics Committee Consultants, Bangalore”. The clinical trial was conducted in compliance with ICH-GCP guidelines, the Declaration of Helsinki and the Ethical and Regulatory standards of India.

Since India is a malaria endemic country, the subjects were pre-screened for the presence of anti-malarial antibodies (anti-PfMSP-1_19_ and anti-PfF2) after consenting on a pre-screening consent form. Subsequently, a study specific screening consent was obtained before participation. Both the consents were written and voluntary. Both pre-screening and screening consent forms were approved by the Ethics Committees. The study was approved by Drug Controller General, India. (application #F.No. 12-02/Diagno/09-BD).

An independent Data Safety Monitoring Board (DSMB) was appointed for the study. The role of DSMB was to periodically review the safety data and grant approvals for dose escalation. The meetings were convened telephonically at the following time-points:

- Prior to subject enrolment in Cohort 1 to review the study protocol, investigator’s brochure and also to review and approve the DSMB charter
- Review of 14-day safety data after first vaccination of Cohort 1 and recommend further dose escalation
- Review of 14-day safety data after first vaccination of Cohort 2 and recommend further dose escalation
- Review of 28-day safety data after first vaccination for all the three cohorts
- Review of 28-day safety data (including 14-day solicited AE data) after second vaccination for all the three cohorts
- Review of 28-day safety data (including 14-day solicited AE data) after third vaccination for all the three cohorts
- Review of general safety data post completion of all the safety follow-ups for the entire study population

The DSMB was authorised to stop the study for discussions if 50% of participants in a particular group were found to have developed a Grade 3 adverse event that was related to vaccination and which persisted at Grade 3 for > 48 hours during the 14 follow-up days after vaccination. The DSMB was empowered to put on hold any subsequent vaccination of that group pending discussion with the investigator and the sponsor. Based on its review, the DSMB was permitted to recommend one of the following actions after due considerations.

- Proceed to the next dose level or stop the dose escalation.
- Pause or stop enrollment with reasons
- Discontinue the study (with provisions for orderly discontinuation in accordance with good medical practice) along with reasons for discontinuation
- Monitor for additional safety end-points along with rationale for the suggested end-points

The trial is registered with Clinical Trial Registry-India (Registration No.: CTRI/2010/091/000301); url: http://ctri.nic.in/Clinicaltrials/pmaindet2.php?trialid=1507&EncHid=&userName=malaria

### Study Vaccines

JAIVAC-1 was the investigational vaccine used while recombinant Hepatitis B vaccine (Gene Vac-B) provided by Serum Institute of India was used as the control vaccine.

Clinical grade recombinant PfF2 and PfMSP-1_19_ were produced, and 62.5μg of each antigen was vialed and lyophilised under cGMP (current Good Manufacturing Practice) at Bharat Biotech International Limited (BBIL), Hyderabad, India. Lot no. BRD/JV/FFL/7003 was used for the study. This lot was released by the Central Research Institute, Kasauli, India. Adjuvant Montanide ISA 720 was supplied by Seppic Inc., France.

JAIVAC-1 was reconstituted under laminar flow hood just before immunisation. The lyophilised product was reconstituted with 0.20 ml of sterile water and 0.42 ml of Montanide ISA 720. The entire contents of the reconstituted and formulated vaccine (0.62 ml) in the vial were drawn into a 2 ml latex free syringe using a 22G needle and was emulsified by mixing up and down twenty five times. One up and down = 1 stroke and 25 such strokes were performed with the plunger to obtain the desired emulsion. The particles in the formulation were homogenous as they displayed a monomodal population with average particle size < 5 microns in diameter as previously described [23].

Volumes of 0.10 ml (10μg each antigen), 0.25 ml (25μg each antigen) and 0.50 ml (50μg each antigen) of the ready to use emulsion were administered by the intramuscular route using a 22G needle attached to a 1ml tuberculin syringe. On reconstitution and emulsification, the three different dosages of JAIVAC-1 vaccine intended for clinical use contained approximately the following concentrations of sterile water and Montanide ISA 720:

- Cohort 1: 0.03 ml sterile water and 0.07 ml of Montanide ISA 720.
- Cohort 2: 0.08 ml sterile water and 0.17 ml of Montanide ISA 720.
- Cohort 3: 0.16 ml sterile water and 0.03 ml of Montanide ISA 720.

Single dose 1 ml adult vials of Gene Vac-B (recombinant DNA Hepatitis B vaccine, I.P), a ready to use suspension from Serum Institute of India was used as control vaccine. The vaccine contains purified surface antigen of the HBsAg virus obtained by culturing genetically engineered *Hansenula polymorpha* yeast cells. The vaccine contains Aluminium hydroxide as adjuvant and thiomersal as preservative.

### Study Design

The clinical trial was a randomised, controlled, single blind, dose escalating, safety and immunogenicity phase I study. Each subject was administered three vaccinations by intramuscular route on Days 0, 28 and 180 in alternate arms in the deltoid region. Subjects were followed up for one year after first immunisation. The three doses of JAIVAC-1 tested contained 10μg (Dosage A, Cohort 1), 25μg (Dosage B, Cohort 2) and 50μg (Dosage C, Cohort 3) of each antigen.

### Study Subjects

The subjects were healthy Indian males aged 18 to 45 years recruited from the volunteer database of healthy subjects available with the study site, Lotus Laboratories, Bangalore. After obtaining pre-screening informed consent, their sera were tested for presence of anti-PfMSP-1_19_ and anti-PfF2 antibodies by ELISA. Subjects whose sera were negative for recognition of both the antigens and signed the screening informed consent form underwent standard screening tests. Subjects were excluded if medical history, physical examination and laboratory examinations revealed any acute or chronic medical condition/disorder. In addition, subjects were not enrolled if they had previously been immunised with Hepatitis B vaccine; had prior history of malaria, had history of hypersensitivity to any of the components of the study vaccines, if they had used any investigational or non-registered drug or vaccine or any gamma globulin three months prior or had plans to use it during the study period; or had history of chronic use of immunosuppressants or other immune modifying drugs six months prior to the study. Subjects were not enrolled if the haematology and biochemistry parameters were not within protocol defined reference ranges and if found positive for viral serology.

### Study Objectives

The primary objectives of the study were to evaluate the safety of JAIVAC-1 and to compare with Hepatitis B vaccine. The secondary objectives were to measure antibody responses to the component antigens of JAIVAC-1 by Enzyme Linked Immunosorbent Assay (ELISA) and test specific recognition of native antigens in the parasite by Immunofluorescence Assay (IFA). As an exploratory objective, the quality of humoral immune response was assessed by measuring the subclass of IgG antibodies by ELISA and the functional activity of such antibodies was tested by Growth Inhibition Assay (GIA).

### Study Endpoints

The primary endpoint was assessment of safety of the study vaccines. The safety profile was evaluated by monitoring 1) immediate reactogenicity every 30 minutes for 3 hours post each vaccination 2) local and systemic solicited events through recordings in the subject diary cards and visits to the clinical research unit (on day of immunization and day 7 and 14 post-immunization) during 14 day follow up period 3) unsolicited events through recordings in the subject diary cards and visits to the clinical research unit (on day of immunization and day 7, 14 and 28 post-immunization) during 28 day follow up period 4) Laboratory derangements on day 28 post each immunization and on days 180 and 365) Serious Adverse Events were looked for the entire study duration from the first dose of vaccine.

The secondary endpoints were to measure the IgG responses against PfMSP-1_19_ and PfF2 by ELISA and IFA at baseline, 28 days after each immunization, on the day of third immunization and at the end of study visit.

The exploratory measures included measurement of sub-classes of IgG antibodies against PfF2 in individuals who had received 25μg and 50μg of JAIVAC-1 and determination of the Growth Inhibitory effects of the antibodies elicited in subjects enrolled in Cohort 3. IgG subtypes were determined at baseline, 28 days after each immunization, on the day of third immunization and at the last study visit and GIA was performed at baseline and 28 days after third immunization.

### Assessment of Clinical and Biological Safety

After each immunisation, vital signs were recorded every 30 minutes for three hours. Pain at the injection site, swelling, redness, induration, limitation of arm motion abduction at shoulder, fever, headache, malaise, myalgia, arthralgia, nausea and vomiting were recorded by the subject in his diary card for 14 days. The investigators assessed local and systemic signs and symptoms, including the presence of nodules and any enlargement of draining lymph nodes 14 days after each vaccination. Unsolicited adverse events were recorded by the subject in his diary card for 28 days after each vaccination.

Biological safety was evaluated at protocol defined time-points by performing haematological and biochemical investigations

The grading scales utilised for severity ranking of solicited and unsolicited Adverse Events is given in Table 1.

**Table 1:** Scoring of severity of solicited and unsolicited adverse events.

	Score/Grade
**Pain at injection site**	
Absent	0
Painful on touch	1
Painful when limb is moved	2
Spontaneously painful or painful at rest	3
**Swelling, Redness, Induration**	
Absent	0
Longest diameter: <25 mm	1
Longest diameter: ≥ 25 mm to < 50mm	2
Longest diameter: ≥50mm	3
**Limitation of arm motion abduction at shoulder**	
Angle of voluntary arm abduction is 180°	0
Angle of voluntary arm abduction is > 90° but < 120°	1
Angle of voluntary arm abduction is > 30° but < 90°	2
Angle of voluntary arm abduction is ≤30°	3
**Fever (axillary temperature measured once daily even in the absence of signs)**	
<38°C	0
38°C to <39°C	1
39°C to <40°C	2
≥40°C	3
**Headache**, **Malaise, Myalgia, Arthralgia, Nausea and Vomiting**	
Absent	0
Present, but easily tolerated	1
Discomforting enough to interfere with normal activities	2
Disabling, prevents normal daily activity, requires bed rest and treatment	3
**Unsolicited Adverse Event Characteristics**	
Sign or symptom present, but easily tolerated	1
Discomforting enough to interfere with normal activities	2
Disabling, incapacitating with inability to perform usual activity	3

### Assessment of anti-PfMSP-119 and anti-PfF2 antibodies and IgG subtypes by ELISA

Assessment of anti-PfF2 and anti-PfMSP-1_19_ antibodies was done on sera collected on Days 0, 28, 56, 180, 208 and 365 by ELISA. Exploratory analysis to measure the quality of the humoral immune response by IgG subtyping using ELISA was originally planned for all three cohorts on Days 0, 28, 56, 180, 208 and 365 for both the antigens. But since PfMSP-1_19_ gave poor immune response, IgG subtyping of antibodies elicited against PfMSP-1_19_ was not performed. Furthermore PfF2 was significantly immunogenic at higher doses (Cohort 2 and 3 compared to Cohort 1) hence IgG subtyping of antibodies against PfF2 was performed on sera samples for subjects immunized with JAIVAC-1 vaccine in Cohorts 2 and 3. IgG subtyping was not performed for subjects immunized with Hepatitis B vaccine as the control subjects had no antibody response against both *P*. *falciparum* antigens.

In brief, ELISA plates were coated with 5μg/ml of PfMSP-1_19_ or 2μg/ml of PfF2. After washing, serially diluted test serum samples and reference standard sera were added to the plates. Horseradish peroxidase (HRP) conjugated rabbit anti-human IgG or biotin–conjugated mouse anti–human isotype–specific IgG and avidin-peroxide conjugate were added to the plate followed by substrate o-phenylenediamine substrate solution. The plates were read at 492 nm using Versamax ELISA reader. Reference standard hyper-immune sera which gave an optical density (OD) of 1.0 in our standardised assay were included as controls. Duplicates of the serially diluted standard reference hyper-immune sera were included on each test plate in order to generate a standard curve. The standard curve was fitted to a four-parameter hyperbolic function, which was used to convert the absorbances of individual test sera into antibody units using the ADAMSEL software. IgG subtype data was reported as the OD at 492 nm (OD_492_). The relative abundance (percent) of each IgG subclass was estimated based on relative mean OD values.

### Assessment of anti-parasite Immunofluoroscence Assay (IFA)

IFA was used to test recognition of native antigens (PfMSP-1_19_ and PfF2) in *P*. *falciparum* schizonts and merozoites. In brief, synchronised *P*. *falciparum* 3D7 were fixed in ice cold methanol on 12 well immunofluoroscence slides and stored at -80°C until use. Test sera were added at various dilutions starting at 1:5. Anti-PfMSP-1_19_ and anti-PfF2 mice sera and reference hyper-immune human sera were used as controls at 1:400 dilution. Naïve human sera and pre-immune mice sera were used as negative controls. Alexa Fluor 488 conjugated goat anti-human IgG or Alexa Fluor 488 conjugated goat anti-mouse IgG secondary antibodies were used. Slides were mounted with a drop of Antifade with DAPI (4’-6-Diamidno-2-phenylindole, a nuclear stain) and viewed using a Nikon TE 2000U fluorescence microscope at 100X magnification using an oil immersion objective lens. End-point IFA titre was determined as the last dilution at which fluorescence signal was observed.

### P. falciparum Growth Inhibition Assay (GIA)

The ability of purified IgG from subject sera to inhibit *in vitro* growth of *P*. *falciparum* 3D7 and CAMP parasite strains was determined by measuring levels of parasite lactate dehydrogenase (pLDH) as described previously [24]. GIA against three parasite strains (*P*.*falciparum* 3D7, *P*.*falciparum* CAMP and *P*.*falciparum* FVO) was planned for sera samples collected on Days 0, 56, 208 and 365. But since PfF2 showed highest antibody titres in Cohort 3, therefore, GIA was performed only for samples of Days 0 and 208. Anti-JAIVAC-1-induced IgG were tested in GIA against PfF2 homologous parasite strain, CAMP, and PfF2 heterologous parasite strain, 3D7. *P*. *falciparum* CAMP is dependent on sialic acid residues on glycophorin A for erythrocyte invasion but *P*. *falciparum* 3D7 invades neuraminidase-treated erythrocytes indicating that it can use sialic acid-independent invasion pathways. GIA was performed with PfF2 heterologous strain 3D7 to check whether the antibodies display any potential for growth inhibition of *P*. *falciparum* strains that use alternative invasion pathways. Since antibody responses to PfMSP1_19_ were poor, GIA with *P*.*falciparum* FVO strain, which is homologous to PfMSP1_19_, was not performed. IgGs purified from sera samples of two randomly chosen Hepatitis B vaccine recipients from Cohort 3 were tested for GIA activity as negative controls. The percent GIA activity of purified IgG was calculated as follows: 100 − (OD_650_ of infected RBCs with test IgG − OD_650_ of normal RBCs only)/(OD_650_ of infected RBCs without any IgG−OD_650_ of normal RBCs only)×100.

### Randomisation

Forty-five subjects who were found eligible after screening assessments were sequentially enrolled into three dose escalating cohorts. Within each Cohort, 15 subjects each were randomised in a 2:1 ratio to receive either JAIVAC-1 or Hepatitis B vaccine. Each subject was assigned a unique, cohort specific, three-digit randomisation number.

Randomization List was generated by the Biostatistics department of DiagnoSearch Life Sciences (the CRO) using Block Randomization method. Six blocks were used for each Cohort. Of these six blocks, three blocks were used to randomize 15 subjects in a cohort and another three to generate 15 additional randomization numbers. In each of the three blocks, two blocks were of size six and one block was of size three.

Printed copies of the randomization lists were provided to Bilcare Ltd. (Vendor for IP packaging) for labelling of vaccine packs and correctly assigning a randomization number; to the site for counter checking the correct assignment of the vaccine to a randomization number and for preparation of vaccine according to the protocol using vaccine packs; to the two sponsors, ICGEB and EVI. A printed copy along with a soft copy of the randomization list was maintained by the biostatistics department of DiagnoSearch.

### Blinding

This clinical trial was designed as a single-blind study. The subjects were blinded to the study treatment while the investigators, site staff, and data analysts were aware of the identity of the vaccine.

The investigator designated appropriately qualified personnel as the “vaccine manager” and the “vaccinator”. The “vaccine manager” was a qualified and trained pharmacist who was dedicated to vaccine preparation while the “vaccinator” was an independent physician/nurse, who was responsible for administration of the assigned vaccine to the individual subject as indicated in the randomisation list. Though the “vaccinator” was aware of the vaccine being administered to the subject, he/she was not directly involved in evaluation of AEs following vaccination and follow up of subjects.

Appropriate measures were taken to ensure subject blinding during vaccine administration. The immunization within a Cohort was staggered over three days so that subjects did not get a chance to interact with each other. The vaccine preparation area and the vaccine administration area were physically separated because the study and control vaccines could be distinguished by their appearance and the syringe barrels were covered with opaque tape to ensure subject blinding. Adherence to randomisation list was verified by the study monitor by checking the randomisation list against the study vaccine administration records at each study visit. Post-vaccination there was minimal contact between subjects within a Cohort. Subjects left the study site after three hours of assessment of immediate reactogenicity and did not meet each other for the next 28 days (except for 24 hour housing of the first three subjects of Cohort 1). The adverse events were recorded in the subject diary cards. Further, being a dose escalating study, subjects belonging to different cohorts never met.

While performing the immunogenicity assessments the scientists involved with ELISA and IFA were also blinded.

### Data Analysis

This was a safety and immunogenicity study, and the sample size of 45 subjects was taken based on previous clinical trials with malaria vaccine candidates and to demonstrate the safety and tolerability profile of the investigational vaccine in comparison to the control vaccine. No statistical hypothesis was formulated for this study and only descriptive statistics was performed for all parameters.

Categorical variables are summarized by vaccine groups as frequency, percentages and 95% confidence interval (CI). Continuous Variables other than titres and concentrations are summarized by vaccine groups as Mean, Standard Error of Mean (SEM), Median, Minimum, Maximum, and Inter-Quartile Range. The proportion of subjects who received three doses without experiencing grade 3 AEs is estimated by Exact Binomial Proportion (Proportion and 95% CI). One interim analysis was performed for data obtained till 28 days after the third dose of the vaccine for each cohort.

The humoral response to the vaccine antigens PfMSP-1_19_ and PfF2 was assessed by measuring the primary antibody response, booster response and immune-persistence. The Geometric Mean Titers (GMTs) of the antibodies is used for measuring the humoral response along with 95% Confidence Interval (CI). For calculating GMTs the titers are log transformed using base 10. The Log transformed titers are summarised as N, Mean, Median, SEM, Maximum, Minimum and Inter-Quartile Range. Statistical analysis is performed to compare the difference between groups at each time point using the SAS software package. The immune response data is analysed by Wilcoxon test for paired comparison, Kruskal-Wallis test for multiple group comparisons or Fisher’s exact test to compare categorical variables as appropriate. Data correlation is performed using a nonparametric Spearman’s Rank Correlation.

## Results

### Subject Disposition

A total of 210 subjects consented to participate in the study at pre-screening. Seventy three were assessed as eligible while 45 were enrolled. The first subject was enrolled in August 2010 and the Last Subject Completed the protocol specified Last Visit i.e. day 365 after first vaccination in November 2011. The study was stopped after the last subject successfully completed the last follow up visit.

**Fig 1:**
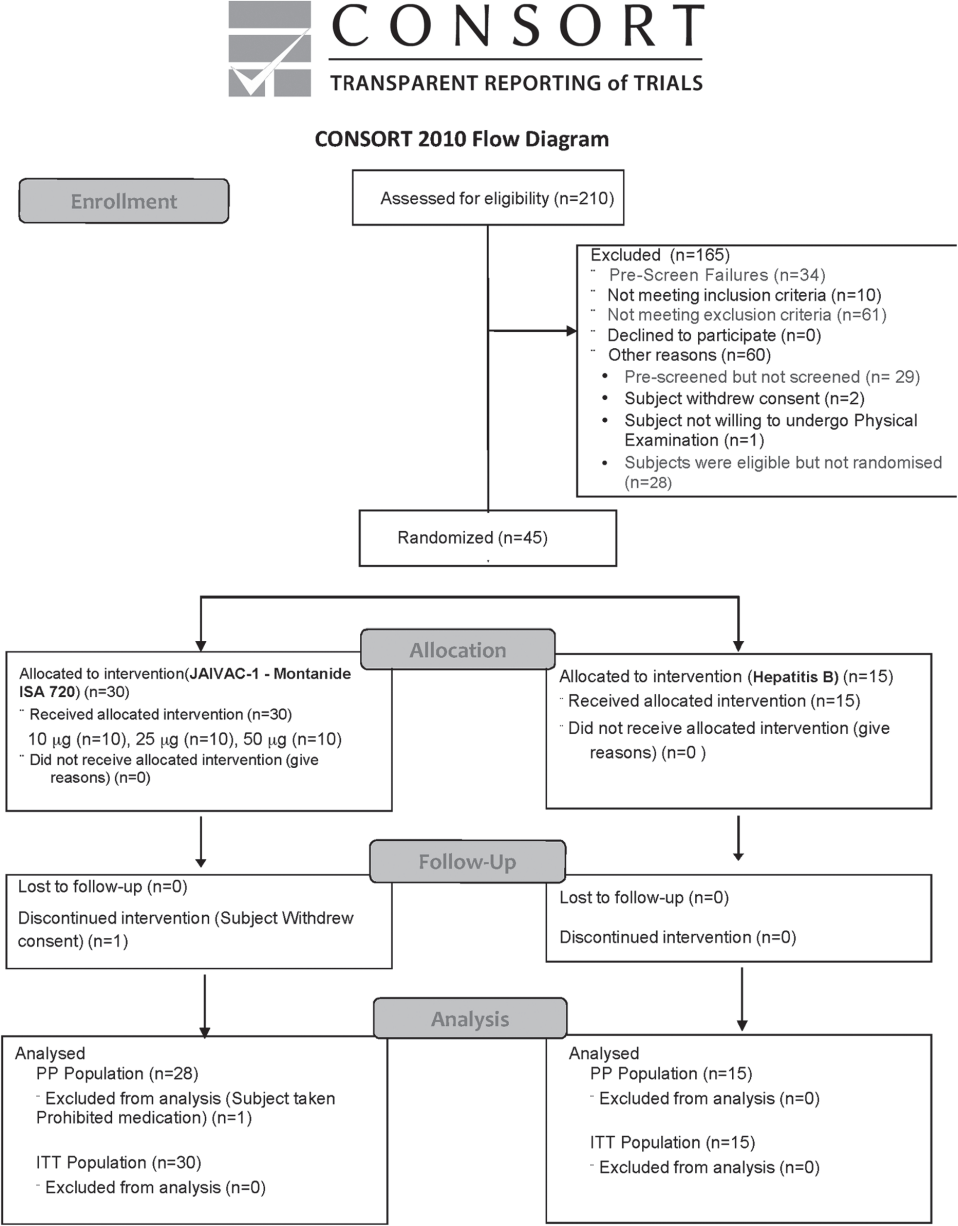
Volunteer Flow Chart (CONSORT diagram).

All the subjects who entered the study satisfied the inclusion criteria and no subject received the wrong vaccine or incorrect dose. A total of 17 protocol deviations were reported in the study of which 9 were reported in the JAIVAC-1 group and 8 in the Hepatitis B group. Among the reported deviations, a single deviation qualified as major while all others were minor protocol deviations. Most minor deviations were categorised as visit window deviations or missed procedures. The major deviation was reported in subject S279 of Cohort 3, immunised with JAIVAC-1 vaccine, who received a prohibited vaccine (tetanus toxoid injection) after the third dose of the study vaccine. This Subject was excluded from the Per-protocol (PP) population but was followed up for safety till end of study. Another subject, S281 withdrew consent after receiving two doses of JAIVAC-1 (Fig 1).

Therefore, the Intention to treat (ITT) population includes all 45 subjects (received at least one dose of either of the study vaccines) while the PP population (subset of the ITT who completed the study without any major protocol violations) includes 43 subjects.

### Clinical and Biological Safety

The baseline demographic characteristics of both groups were similar (Table 2). Vital signs remained normal within first three hours post vaccination for all study subjects.

**Table 2:** Baseline Demographics by Study Groups.

			JAIVAC-1 Montanide ISA 720			Hepatitis B
Characteristic	Classification	Statistic	Dosage A (0.1 ml) (N = 10)	Dosage B (0.25 ml) (N = 10)	Dosage C (0.5 ml) (N = 10)	All (1 ml) (N = 15)
*Age (years)	All	N (Missing)	10 (0)	10 (0)	10 (0)	15 (0)
		Mean (SEM)	26.80 (1.43)	28.94 (1.99)	27.80 (2.18)	26.72 (1.15)
		Median	26.67	27.15	25.94	25.43
		Range (Min, Max)	13.92 (19.58, 33.50)	20.04 (19.60, 39.64)	21.74 (18.59, 40.33)	15.46 (21.83, 37.29)
	ANOVA	p-value	0.7863			
Height (cm)	All	N (Missing)	10 (0)	10 (0)	10 (0)	15 (0)
		Mean (SEM)	170.21 (2.03)	168.42 (2.09)	167.32 (2.00)	168.50 (1.58)
		Median	168.10	169.55	168.05	169.90
		Range (Min, Max)	21.90 (160.60, 182.50)	19.90 (157.40, 177.30)	22.70 (158.10, 180.80)	23.30 (160.00, 183.30)
	ANOVA	p-value	0.7863			
Weight (kg)	All	N (Missing)	10 (0)	10 (0)	10 (0)	15 (0)
		Mean (SEM)	64.93 (3.34)	63.51 (3.28)	65.51 (3.00)	62.46 (1.38)
		Median	67.20	62.00	64.85	63.10
		Range (Min, Max)	31.90 (50.50, 82.40)	32.40 (50.30, 82.70)	32.10 (52.90, 85.00)	19.70 (50.50, 70.20)
	ANOVA	p-value	0.7863			

Note

1)*Age (in years) is calculated from the DOB, as the difference between the date the Pre-screening ICF is signed and the DOB.

2) Percentages are based on Number of randomised subjects in each study group.

3) Between group comparison for quantitative variables is performed using ANOVA and qualitative variables is performed using Fisher’s Exact Test

Pain at the injection site and limitation of arm motion abduction were the frequently reported local solicited adverse events. Other reported events included swelling, redness and induration. Pain at injection site was reported by twenty eight subjects who received JAIVAC-1 and five subjects in Hepatitis B group (Table 3). Grade III pain was reported in six participants who had received JAIVAC-1 (Table 3). Pain at injection site resolved between 24 to 60 hours in JAIVAC-1 recipients whereas it lasted for 66 to 108 hours in participants who received Hepatitis B. Limitation of abduction lasted one to seven days in the JAIVAC-1 group while it resolved in 72 hours in Hepatitis B group.

**Table 3:** Number of subjects (percentage) experiencing Solicited Adverse events by study arms and intensity levels.

Study Arm (N):	JAIVAC-1 (10μg) (N = 10)				JAIVAC-1 (25μg) (N = 10)				JAIVAC-1 (50μg) (N = 10)				Hepatitis B (N = 15)			
Event/ Intensity Grade	Anyn (%)	In (%)	IIn (%)	IIIn (%)	Anyn (%)	In (%)	IIn (%)	IIIn (%)	Anyn (%)	In (%)	IIn (%)	IIIn (%)	Anyn (%)	In (%)	IIn (%)	IIIn (%)
**Local Solicited AEs**	9 (90.00)	2 (20.00)	5 (50.00)	2 (20.00)	10 (100.00)	3 (30.00)	6 (60.00)	1 (10.00)	9 (90.00)	5 (50.00)	1 (10.00)	3 (30.00)	5 (33.33)	3 (20.00)	2 (13.33)	0 (0.00)
Pain at injection site	9 (90.00)	2 (20.00)	5 (50.00)	2 (20.00)	10 (100.00)	3 (30.00)	6 (60.00)	1 (10.00)	9 (90.00)	5 (50.00)	1 (10.00)	3 (30.00)	5 (33.33)	3 (20.00)	2 (13.33)	0 (0.00)
Limitation of arm motion abduction at shoulder	1 (10.00)	1 (10.00)	0 (0.00)	0 (0.00)	0 (0.00)	0 (0.00)	0 (0.00)	0 (0.00)	4 (40.00)	3 (30.00)	1 (10.00)	0 (0.00)	1 (6.67)	1 (6.67)	0 (0.00)	0 (0.00)
Swelling	0 (0.00)	0 (0.00)	0 (0.00)	0 (0.00)	0 (0.00)	0 (0.00)	0 (0.00)	0 (0.00)	2 (20.00)	2 (20.00)	0 (0.00)	0 (0.00)	1 (6.67)	1 (6.67)	0 (0.00)	0 (0.00)
Redness	1 (10.00)	1 (10.00)	0 (0.00)	0 (0.00)	0 (0.00)	0 (0.00)	0 (0.00)	0 (0.00)	1 (10.00)	1 (10.00)	0 (0.00)	0 (0.00)	0 (0.00)	0 (0.00)	0 (0.00)	0 (0.00)
Induration	1 (10.00)	1 (10.00)	0 (0.00)	0 (0.00)	0 (0.00)	0 (0.00)	0 (0.00)	0 (0.00)	0 (0.00)	0 (0.00)	0 (0.00)	0 (0.00)	1 (6.67)	1 (6.67)	0 (0.00)	0 (0.00)
**Systemic Solicited AEs**	6 (60.00)	3 (30.00)	3 (30.00)	0 (0.00)	1 (10.00)	1 (10.00)	0 (0.00)	0 (0.00)	3 (30.00)	1 (10.00)	1 (10.00)	1 (10.00)	2 (13.33)	2 (13.33)	0 (0.00)	0 (0.00)
Fever	0 (0.00)	0 (0.00)	0 (0.00)	0 (0.00)	0 (0.00)	0 (0.00)	0 (0.00)	0 (0.00)	0 (0.00)	0 (0.00)	0 (0.00)	0 (0.00)	1 (6.67)	1 (6.67)	0 (0.00)	0 (0.00)
Headache	4 (40.00)	3 (30.00)	1 (10.00)	0 (0.00)	0 (0.00)	0 (0.00)	0 (0.00)	0 (0.00)	2 (20.00)	1 (10.00)	1 (10.00)	0 (0.00)	2 (13.33)	2 (13.33)	0 (0.00)	0 (0.00)
Malaise	1 (10.00)	0 (0.00)	1 (10.00)	0 (0.00)	0 (0.00)	0 (0.00)	0 (0.00)	0 (0.00)	1 (10.00)	0 (0.00)	0 (0.00)	1 (10.00)	0 (0.00)	0 (0.00)	0 (0.00)	0 (0.00)
Myalgia	2 (20.00)	0 (0.00)	2 (20.00)	0 (0.00)	1 (10.00)	1 (10.00)	0 (0.00)	0 (0.00)	1 (10.00)	1 (10.00)	0 (0.00)	0 (0.00)	0 (0.00)	0 (0.00)	0 (0.00)	0 (0.00)
Arthralgia	0 (0.00)	0 (0.00)	0 (0.00)	0 (0.00)	0 (0.00)	0 (0.00)	0 (0.00)	0 (0.00)	0 (0.00)	0 (0.00)	0 (0.00)	0 (0.00)	0 (0.00)	0 (0.00)	0 (0.00)	0 (0.00)
Nausea	1 (10.00)	0 (0.00)	1 (10.00)	0 (0.00)	0 (0.00)	0 (0.00)	0 (0.00)	0 (0.00)	0 (0.00)	0 (0.00)	0 (0.00)	0 (0.00)	0 (0.00)	0 (0.00)	0 (0.00)	0 (0.00)
Vomiting	0 (0.00)	0 (0.00)	0 (0.00)	0 (0.00)	0 (0.00)	0 (0.00)	0 (0.00)	0 (0.00)	0 (0.00)	0 (0.00)	0 (0.00)	0 (0.00)	0 (0.00)	0 (0.00)	0 (0.00)	0 (0.00)

N = no. of subjects vaccinated in each cohort should be considered as the denominator.

n = number of subjects who have experienced the respective event at least once after administration of doses of vaccine during study.

Each subject is counted at most once for each solicited event and the maximum intensity level is considered.

Headache, myalgia, malaise, nausea and fever were the systemic solicited adverse events reported by the subjects. Majority of events occurred within 24 to 72 hours post vaccination. Except one Grade III event of Malaise in Cohort 3, rest of the systemic adverse events were of either grade I or II intensity. All adverse events resolved without any sequealae.

No serious adverse event was reported during the study.

### **Assessment of antibody responses to PfF2 and PfMSP-1**_*19*_

The titers of antigen-specific antibodies were expressed as the reciprocal dilution of serum that yields an OD of 1.0. At Day 0, the geometric mean of baseline antibody responses with specificity to PfF2 as well as to PfMSP-1_19_ was ≤ 10. The geometric mean antibody titers specific to PfF2 and PfMSP-1_19_ at different time points after vaccination with 10μg, 25μg and 50μg JAIVAC-1 and Hepatitis B are shown in Table 4 and Table 5, respectively.

**Table 4:** Geometric Mean Titre (GMT) at 95% Cl for PfF2-specific antibodies elicited after vaccination with 10μg, 25μg and 50μg JAIVAC-1 and Hepatitis B vaccine.

Days/Dosage	JAIVAC-1 Dosage 10μg	JAIVAC-1 Dosage 25μg	JAIVAC-1 Dosage 50μg	Hepatitis B Dosage 1ml
	n = 10	n = 10	n = 10	n = 15
0	10.0 (—,—)^a^	10.5 (9.7, 11.3)	12.3 (9.9, 15.3)	10.2 (9.8, 10.7)
28	11.4 (9.1, 14.2))	12.4 (9.7, 15.9)	66.8 (41.1, 108.7)	10.3 (9.7. 11.0)
56	62.7 (27.7, 141.7)	106.7 (46.3, 245.8)	635.0 (290.4, 1388.8)	10.3 (9.7, 10.8)
180	14.4 (10.4, 20.0)	19.1 (12.8, 28.6)	66.9 (29.3, 153.0)	10.1 (9.9, 10.3)
208	88.7 (37.0, 212.5)	254.1 (136.2, 473.9)	709.4 (256.7, 1960.6)	13.0 (7.7, 21.8)
365	46.6 (30.5, 71.3)	81.4 (58.0, 114.2)	132.0 (67.4, 258.6)	15.4 (11.2, 21.1)

^a^Lower Limit of Quantitation determined during method validation of ELISA was 10IU (International Unit). Antibody titre ≥10 IU against PfMSP1_19_ or PfF2 were considered in actual for analysis purpose while values < 10IU were reported as 10 during calculations involving logs.

**Table 5:** Geometric Mean Titre (GMT) at 95% Cl PfMSP-1_19_-specific antibodies elicited after vaccination with 10μg, 25μg and 50μg JAIVAC-1 and Hepatitis B vaccine.

Days/Dosage	JAIVAC-1 Dosage 10μg	JAIVAC-1 Dosage 25μg	JAIVAC-1 Dosage 50μg	Hepatitis B Dosage 1ml
	n = 10	n = 10	n = 10	n = 15
0	10 (—,—)^a^	10 (—,—)^a^	10 (—,—)^a^	10 (—,—)^a^
28	13.5 (6.8, 26.8)	10 (—,—)^a^	11.6 (8.3, 16.3)	10 (—,—)^a^
56	14.1 (6.5, 30.9)	12.0 (9.3, 15.4)	14.4 (8.7, 23.6)	10 (—,—)^a^
180	13.7 (6.7, 27.8)	10 (—,—)^a^	10.3 (9.8, 10.8)	10 (—,—)^a^
208	18 (7.4, 43.8)	24.5 (12.8, 46.8)	33.0 (15.2, 71.5)	11.6 (8.4, 16.2)
365	26.8 (14.0, 51.3)	16.1 (12.0, 21.6)	10.8 (9.6, 12.2)	15.1 (10.7, 21.2)

^a^Lower Limit of Quantitation determined during method validation of ELISA was 10IU (International Unit). Antibody titre ≥10 IU against PfMSP1_19_ or PfF2 were considered in actual for analysis purpose while values < 10IU were reported as 10 during calculations involving logs.

**Fig 2:**
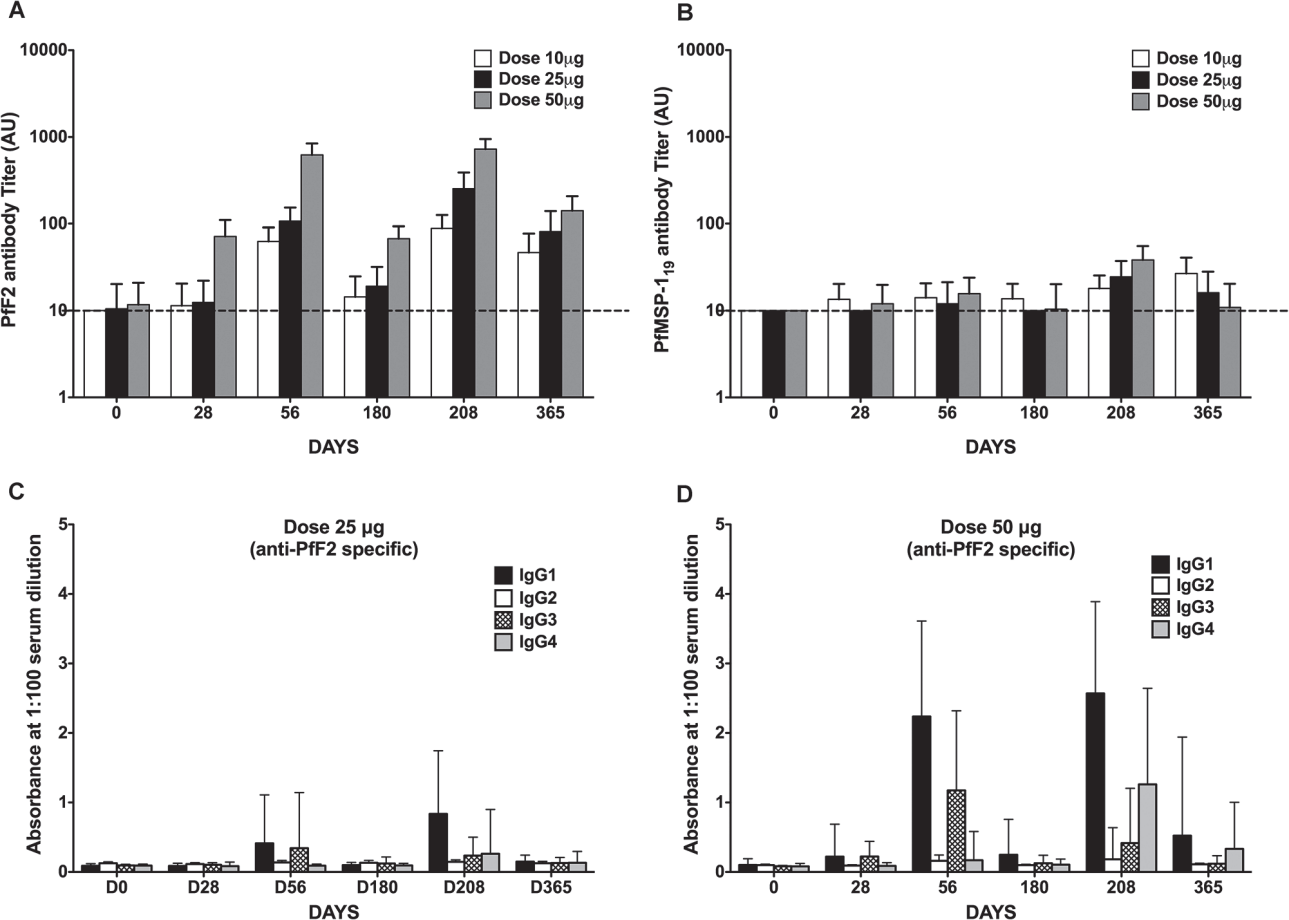
Antibodies to PfF2 and PfMSP-1_19_ after vaccination with JAIVAC-1. Anti-PfF2 (A) and PfMSP-1_19_ (B) antibody levels measured by ELISA. Geometric mean antibody levels (AU) to recombinant PfF2 and PfMSP-1_19_ measured by ELISA in sera collected from Day 0 to Day 365 in 10μg, 25μg, and 50μg JAIVAC-1/Montanide ISA720 and Hepatitis B vaccine recipients. Study participants were immunized on Days 0, 28 and 180 and sera were collected on Days 0, 28, 56, 180, 208 and 365. Antibody levels measured by ELISA were expressed as Geometric Mean Titres (GMT) for the 10 subjects. Anti-PfF2 specific IgG subclass profiles in the sera of individuals vaccinated with 25μg (C) and 50μg (D) JAIVAC-1 as measured by ELISA are shown.

The geometric mean titer (GMT) for recognition of PfF2 by sera collected at Day 208 was higher in subjects who had received 10μg (*p* < 0.05), 25μg (*p* < 0.01) or 50μg (*p* < 0.001) of JAIVAC-1 vaccine compared to pre-immune sera (Fig 2A). Sera from Hepatitis B controls did not recognise PfF2. Pair-wise comparisons of the anti-PfF2 GMT responses at Day 208 between the groups revealed that antibody titre showed a dose dependent increase in 25μg and 50μg dose groups compared to the 10μg dose group (10μg vs 25μg, *p* = 0.0690; 10μg vs 50μg, *p* = 0.0123) (Fig 2A). Following the increase in response at Day 56, PfF2 antibody titer decayed by Day 180. Day 180 immunization boosted antibody responses with titres comparable to those achieved on day 56. Antibody titres of sera collected on Day 365, six months after third immunization, showed a significant decline.

The predominant PfF2 specific IgG subtype response was IgG1 and IgG3 for both 25μg and 50μg doses (Fig 2C and 2D). Twenty-eight days after the booster administration (at Day 208), there was an increase in IgG4 subclass and a decrease in IgG3 subclass response to PfF2. At Day 365, the concentration of all IgG subclasses in cohort 2 declined to very low levels whereas in cohort 3, there was a fall in IgG1, IgG3 and IgG4 concentrations from the Day 208 values but these subclasses were still detectable.

The response elicited by PfMSP-1_19_ was significantly lower than that induced by PfF2 in all doses groups. (Fig 2B).

### Vaccine responders 28 days after completion of the vaccination schedule (Day 208)

A vaccine responder was defined as a subject showing greater than or equal to 2-fold increase in antibody titer to both the antigens, PfMSP-1_19_ and PfF2.

A 2-fold response to PfF2 and PfMSP-1_19_ was observed in 28 and 12 subjects respectively of the PP population of 43. Twelve subjects showed 2-fold response to both antigens and were therefore considered as vaccine responders (Table 6).

**Table 6:** Number of responders to antigen PfF2 and PfMSP-1_19_ in 10μg, 25μg and 50μg JAIVAC-1/Montanide ISA720 and Hepatitis B vaccine recipients.

Dose	≥ 2-fold Responders			≥ 4-fold Responders		
	PfF2	PfMSP-1_19_	PfF2 and PfMSP-1_19_	PfF2	PfMSP-1_19_	PfF2 and PfMSP-1_19_
DOSE 10μg	9	2	2	7	2	1
DOSE 25μg	10	5	5	10	4	4
DOSE 50μg	9	5	5	9	4	4
HEP B	1	1	1	1	1	1

### Kinetics of Ab response as measured by IFA

**Fig 3:**
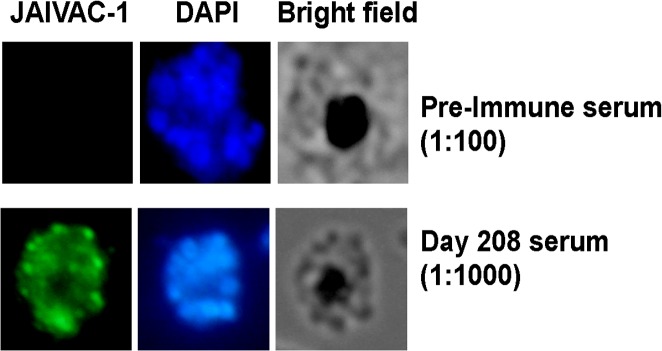
Recognition of native antigens in blood stage *P*. *falciparum* schizonts. Serum from one representative subject immunised with JAIVAC-1 was used to detect native PfMSP1 and EBA 175 in *P*. *falciparum* schizonts by Immunofluoroscence Assay (IFA). The punctate staining at the apical end of merozoites in late-stage schizonts reflects the known distribution of EBA 175.

As seen in Fig 3, JAIVAC-1 vaccinated individuals recognised native antigens on the surface of *P*. *falciparum* parasite. Sera with high anti-PfF2 titres reacted with late schizonts in a punctate pattern, reminiscent of the pattern of EBA175 localisation at the apical end of merozoites. When titres to both PfF2 and PfMSP-1_19_ were high in the sera, a strong apical staining of PfF2 tended to predominate, although a uniform staining of the PfMSP-1 merozoite circumference staining was also evident. Pre-immune sera showed no reactivity to the parasite (Fig 3).

**Fig 4:**
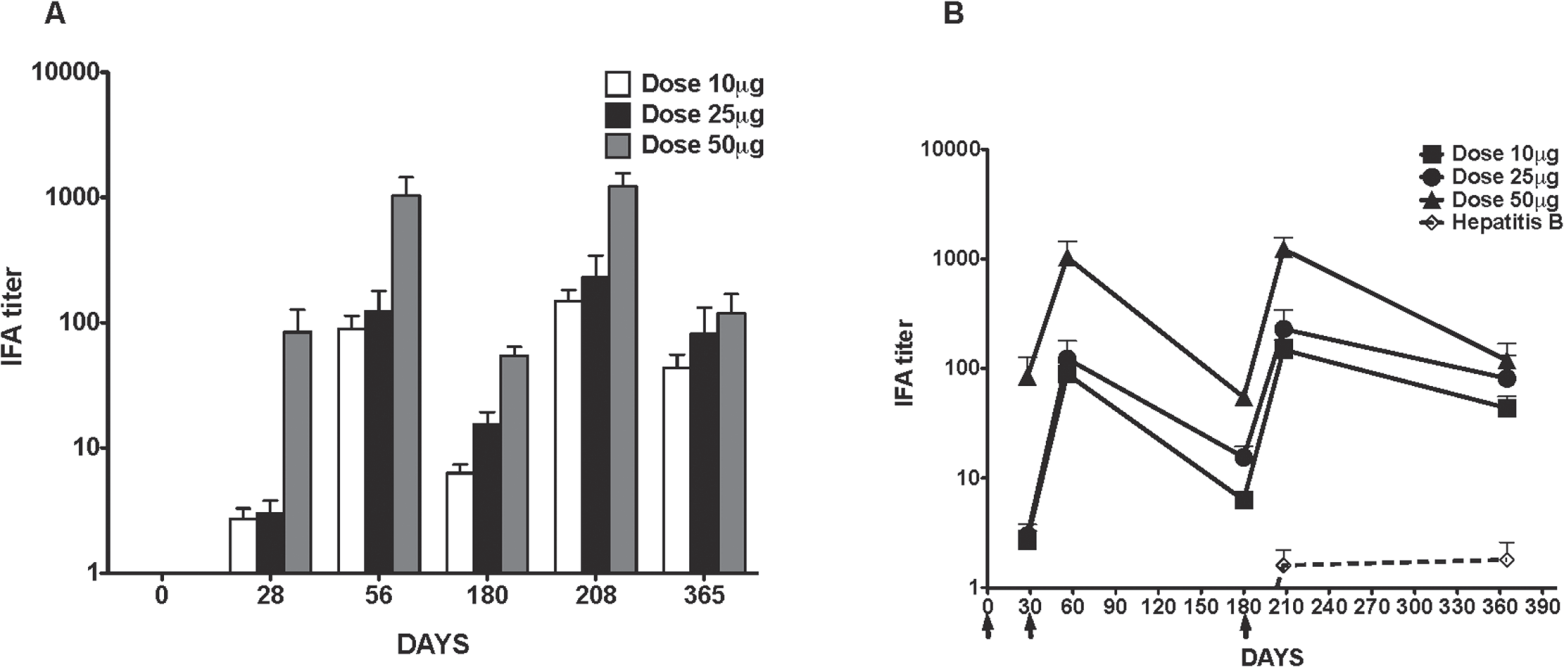
IFA titer. Dose-dependence (A) and kinetics (B) of antibody response as measured by IFA using sera from subjects who received 10μg, 25μg and 50μg of JAIVAC-1 and Hepatitis B vaccine. The arrows indicate days of immunisation.

All JAIVAC-1 subjects developed detectable antibody reactivity to malaria parasites (Fig 4A & 4B). An increased antibody response was observed at Day 56 after two doses of vaccine, followed by a decline in titer by Day 180. Responses were marginally boosted 28 days post third immunisation in comparison to the Day 56 titres. At the end of one year of follow-up (Day 365) IFA titer remained significantly elevated, but was much lower compared to titers of Days 56 and 208. The dose of vaccine did not alter the kinetics of the response with similar patterns observed for the all doses. In sera from the Hepatitis B group, there were no detectable antibodies against PfF2 or PfMSP-1_19_. At Day 208 there was a marginal rise which persisted till Day 365 (Fig 4A & 4B).

### Correlation between antibody response measured by ELISA and IFA

There is a significant positive correlation between anti-PfF2 ELISA titres and IFA titres at Day 208 in vaccination cohorts 10μg (r = 0.83, p = 0.003), 25μg (r = 0.93, p = 0.00009) and 50μg (r = 0.86, p = 0.006). In case of PfMSP-1_19_, this correlation was not observed.

### Growth Inhibition Assays

**Fig 5:**
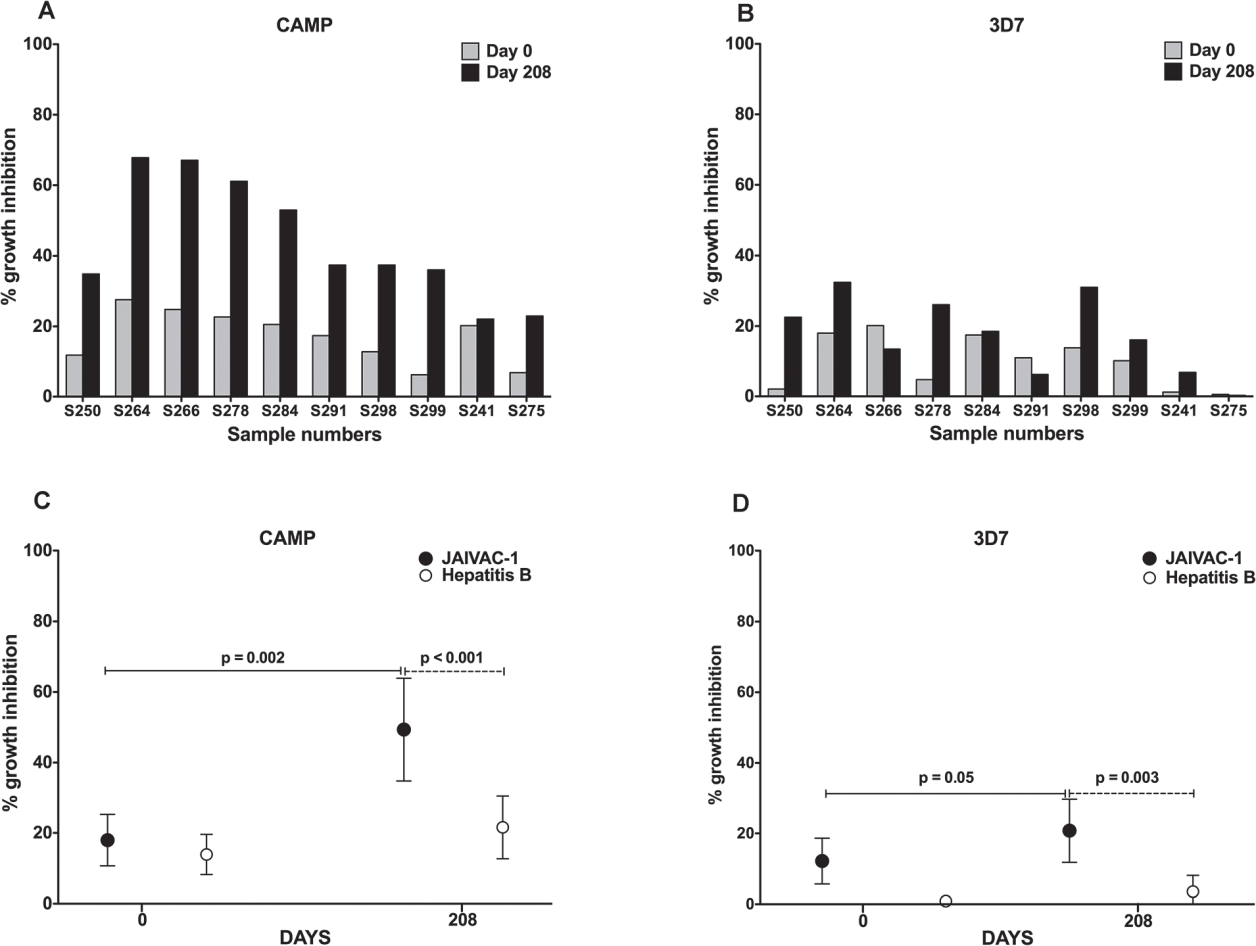
Vaccine induced in vitro Growth Inhibitory Activity (GIA). The percent growth inhibition in cultures was measured by pLDH using two different strains of *P*. *falciparum* (CAMP and 3D7). (A) and (B) represent the percent growth inhibition of *P*. *falciparum* CAMP and 3D7 parasite strains after addition of 10 mg/ml purified IgG from sera of Day 0 and Day 208 of 50μg of JAIVAC-1 vaccine and Hepatitis vaccine group. (C) and (D) represent the average percent growth inhibition of—*P*. *falciparum* parasite strains CAMP and 3D7 with Day 0 and Day 208 sera from individuals immunized with 50μg of JAIVAC-1 and Hepatitis vaccine.

IgGs purified from sera collected on Day 208 from ten subjects of Cohort 3 were tested for GIA activity against *P*. *falciparum* CAMP and 3D7 strains. Of these 10 subjects, eight had received JAIVAC-1 vaccine while two had received Hepatitis B vaccine. Samples from subjects S279 and S281 of Cohort 3 were not analysed since they had been excluded from PP analysis. IgG purified from pre-immune sera collected from the same individuals were used as negative controls. The average GIA was around 49.35% ± 14.54% (34.89% to 67.83%) against the CAMP strain and around 20.79% ± 8.97% (6.23% to 32.43%) against the 3D7 strain. IgG purified from Day 208 sera from JAIVAC-1 group showed significant inhibition of parasite growth (CAMP) compared to antibodies purified from Day 208 sera obtained from Hepatitis B vaccinated individuals (p < 0.001) (Fig 5A, 5B, 5C and 5D).

## Discussion

JAIVAC-1, a *P*. *falciparum* malaria vaccine candidate formulated with Montanide ISA 720 was generally well tolerated by healthy Indian adult male subjects. No serious adverse event was observed and no subject was withdrawn from study on account of safety concerns. Immunogenicity data shows that all three doses of JAIVAC-1 (10μg, 25μg and 50μg) elicited antibodies against PfF2 but failed to elicit significant antibody responses against PfMSP-1_19_.

### Safety and Reactogenicity

Prior Phase I and II clinical trials with Montanide ISA 720 adjuvanted vaccines have frequently reported delayed local injection site reactogenicity like nodule formation, induration, and tenderness [20, 21, 25–33]. It has been reported that reactogenicity of Montanide ISA 720 can vary. Combination B vaccine (proteins RESA, MSP1 and MSP2) formulated with Montanide ISA 720 was highly reactogenic in Australian adults but was very well tolerated in adults and children in Papua New Guinea [21, 31, 33–36]. In this study the maximum antigen dose was limited to 50μg of each antigen and an extended immunization schedule of 0, 1and 6 months was followed, which may explain the absence of delayed reactogenicity. As with other Montanide adjuvanted vaccines, pain at injection site was the most common local solicited event. Additional local reactogenicity symptoms were observed but did not appear to increase with increasing dose of JAIVAC-1.

Therefore, although JAIVAC-1 was more reactogenic in comparison to Hepatitis B vaccine, it had a good safety profile when compared with the pattern of adverse events reported in literature for recombinant vaccines formulated with Montanide ISA720 [20, 21, 25–33].

### Immunogenicity

Montanide ISA720 has been repeatedly used in pre-clinical and clinical trials of different malaria vaccine candidates and in particular of *P*. *falciparum* AMA-1 [20, 21, 25–33, 37–39]. Montanide ISA 720 adjuvant formulation is highly immunogenic and is known to induce robust antigen-specific humoral and cellular responses. Immunisation with JAIVAC-1 formulated with Montanide ISA720 elicited high levels of antibodies against PfF2 with response peaking a month after the third immunisation. However, the antibody response elicited against PfMSP-1_19_ was poor. In the previous clinical studies, Keitel et al had produced two recombinant MSP-1_19_ antigens derived from *P*. *falciparum* 3D7 and FVO strains in *Saccharomyces cerevisiae* fused to tetanus toxoid T-helper epitopes P30 and P2 [13] They noted no clear dose-response relationship for the FVO MSP-1_19_ vaccine groups but had reported a dose-response relationship for the 3D7 MSP-1_19_ vaccine groups. Although both vaccines were immunogenic, several subjects in both the groups experienced apparent hypersensitivity reactions after receiving the third dose. In JAIVAC-1 vaccine, PfMSP-1_19_ was derived from FVO *P*. *falciparum* strains and did not induce significant PfMSP-1_19_ antibody responses.

A positive dose-response for antibody responses against PfF2 was observed; higher JAIVAC-1 vaccine dose resulted in significantly higher antibody response against PfF2. The number of vaccine responders in the 25μg and 50μg dose groups increased significantly after three vaccinations. The ant-PfF2 and anti- PfMSP-1_19_ antibodies induced by JAIVAC-1 vaccine recognise the native parasite protein in *P*. *falciparum* schizonts as studied by IFA. The pattern of recognition was similar to the known punctate localisation pattern of EBA175 at the apical end of merozoites. The anti-PfF2 ELISA titers correlated with IFA titers. Given the poor recognition of PfMSP-1_19_ by IFA it is not surprising that the pattern of recognition appears to match the pattern of distribution of EBA-175 in schizonts/merozoites. The poor immunogenicity of PfMSP-1_19_ is unlikely to be due to dominance of PfF2 as this was not observed in pre-clinical studies [unpublished data]. Inclusion of widely recognized T-helper epitopes in the recombinant PfMSP-1_19_ construct may be required to improve immunogenicity of this candidate antigen. Antibodies generated by JAIVAC-1 vaccine had substantial parasite growth inhibitory activity. Invasion of erythrocytes by the *P*. *falciparum* CAMP strain is dependent on the presence of sialic acids on glycophorin A, while 3D7 parasites invade erythrocytes via alternate pathways that do not involve sialic acids [40]. Purified IgG from the sera of JAIVAC-1 vaccinated (50μg dose) individuals inhibited invasion of the CAMP strain (homologous for the PfF2 immunogen tested here). The reduced inhibition of invasion of 3D7 parasite could be due to the ability of 3D7 parasites to invade erythrocytes via sialic acid independent pathway(s). Efficient inhibition of erythrocyte invasion by parasites such as 3D7 may require the addition of other parasite ligands to the antigen combination in the vaccine. In conclusion, in this phase I clinical trial, JAIVAC-1 vaccine demonstrated a good safety profile and was able to induce significant antibody responses in malaria-naive individuals. Additionally, the functional properties of these antibodies were demonstrated by their ability to partially inhibit parasite growth *in vitro*. These observations provide support for the inclusion of PfF2 as a component of a multicomponent vaccine for *P*. *falciparum* malaria. However, the problem of poor immunogenicity of PfMSP-1_19_ needs to be addressed. Addition of widely recognised T-helper epitopes to recombinant PfMSP-1_19_ may provide T cell help and boost antibody responses against PfMSP-1_19_.

## Supporting Information

S1 CONSORT Checklist (DOC)

S1 Final Analysis Listings (PDF)

S1 Pre-screening Information Sheet and Informed Consent Form (PDF)

S1 Main Subject Information Sheet and Informed Consent Form (PDF)

S1 Protocol A Phase I, Randomised, Controlled, Dose-escalating, Single blind Clinical trial to evaluate the safety and immunogenicity of JAIVAC-1 vaccine (PfMSP-1_19_ and PfF2) formulated with Montanide ISA 720 in Healthy Indian Male Subjects between 18 to 45 years of age. (PDF)

S1 Subject Diary Card (PDF)
