# Correction: Phase I Clinical Trial of a Recombinant Blood Stage Vaccine Candidate for *Plasmodium falciparum* Malaria Based on MSP1 and EBA175

**DOI:** 10.1371/journal.pone.0137816

**Published:** 2015-09-02

**Authors:** Chetan E. Chitnis, Paushali Mukherjee, Shantanu Mehta, Syed Shams Yazdani, Shikha Dhawan, Ahmad Rushdi Shakri, Rukmini Bhardwaj, Puneet Kumar Gupta, Dhiraj Hans, Suman Mazumdar, Bijender Singh, Sanjeev Kumar, Gaurav Pandey, Varsha Parulekar, Nathalie Imbault, Preethi Shivyogi, Girish Godbole, Krishna Mohan, Odile Leroy, Kavita Singh, Virander S. Chauhan

The seventh author’s name is spelled incorrectly. The correct name is: Rukmini Bhardwaj.
