# Supplementary material for: Phase I Clinical Trial of a Recombinant Blood Stage Vaccine Candidate for Plasmodium falciparum Malaria Based on MSP1 and EBA175
Source: PLoS One. 2015 Apr 30;10(4):e0117820. doi: 10.1371/journal.pone.0117820 (PMC4415778; doi:10.1371/journal.pone.0117820)
Supplement: S1 Pre-screening Information Sheet and Informed Consent Form — (PDF) [file pone.0117820.s003.pdf]

PRE-SCREENING INFORMATION SHEET AND INFORMED CONSENT FORM

**Clinical Trial Identifier**	JAIVAC-1_1_09
**Version, Date**	Final Version # 1.2 dated 15 June 2010
**Protocol Title**	A Phase I, Randomised, Controlled, Dose-Escalating, Single-Blind Clinical Trial to Evaluate the Safety and Immunogenicity of JAIVAC-1 Vaccine (PfMSP-$1_{19}$and PfF2) formulated with Montanide ISA720 in Healthy Indian Male Subjects between 18 to 45 Years of Age
**Name of the Investigator**	Dr. Girish Godbole
**Name of the Study Site**	Lotus Labs Pvt Ltd

"A new vaccine under study against *Plasmodium falciparum* malaria in Indian male subjects in the age group of 18 to 45 years"

You are invited to know if you can participate in a research study testing a new vaccine for malaria. Please read this information carefully. Make sure that you understand it to your complete satisfaction before signing your name at the end. Please ask questions if you do not understand any of the information.

This research study will be conducted by the Investigator (Dr. Girish Godbole) at the Lotus Labs Pvt. Ltd. in Bangalore and sponsored by [European Vaccine Initiative (EVI, UniversitätsKlinikum Heidelberg - Germany) and International Centre for Genetic Engineering and Biotechnology (ICGEB, New Delhi, India)]. This study will be conducted to find out whether it is safe to administer a new malaria vaccine in humans. A vaccine is any preparation intended to protect against disease by stimulating the production of substances called antibodies.

To participate in the study you must be aged 18 to 45 years and must be in good health as judged by your investigator. You should not have known previous record or history of malaria or anti-malaria antibodies in your blood.

After you sign this form, your demographic details (date of birth, race, etc.) will be recorded by the investigator.

The very first step is to know if you have already had malaria in your life therefore approximately 5 ml (approximately 1 teaspoon) of your blood will be taken to allow the team to check if you have the so-called anti-malaria antibodies.

If you do not have anti-malaria antibodies you will be invited to a second visit where the study will be further explained and more tests will be performed to know if you can participate in the proposed study.

You must be honest with your doctor about your health, now and in the past, or it may not be safe for you to participate in the study.

PRE-SCREENING CONSENT FORM TO PARTICIPATE IN THE JAIVAC-1_1_09 STUDY

By signing this document, I freely give my consent to the medical staff of Lotus Labs Pvt. Ltd. to examine me for possible participation in a research study testing a potential malaria vaccine, which is a recombinant product. I also give consent for providing finger prints for biometric identification.

- I understand that approximately 5 ml of blood sample will be collected to test if it contains anti-malarial antibodies by a test called ELISA. If such antibodies are present in my blood, I will be excluded from the study.
- I also understand that if no antibodies are shown in my blood then I will be again explained of all the procedures and other tests which will be done to decide my eligibility into the study after giving written consent for the same.

I understand that giving false, incomplete, or misleading information about my medical history could have very serious consequences. I understand that in the event I am not selected for inclusion in the study after taking this sample for analysis, I will not receive any compensation from Lotus Labs Pvt Ltd and/or Sponsors (European Vaccine Initiative (EVI) and International Center for Genetic Engineering and Biotechnology (ICGEB)) of the vaccine.

Subject's Name (PRINT):______________________________________

Subject Initials: ___ ___ ___

Subject's date of birth, Age : __ __ / __ __ __ / __ __ __ __, ________

Screening Number: _____________________

Sign and Date:____________________________________

Physician Name: _____________________________________

Sign and Date _:_____________________________________
