# Supplementary material for: Phase I Clinical Trial of a Recombinant Blood Stage Vaccine Candidate for Plasmodium falciparum Malaria Based on MSP1 and EBA175
Source: PLoS One. 2015 Apr 30;10(4):e0117820. doi: 10.1371/journal.pone.0117820 (PMC4415778; doi:10.1371/journal.pone.0117820)
Supplement: S1 Main Subject Information Sheet and Informed Consent Form — (PDF) [file pone.0117820.s004.pdf]

MAIN SUBJECT INFORMATION SHEET AND INFORMED CONSENT FORM

**Clinical Trial Identifier**	JAIVAC-1_1_09
**Version, Date**	Final, Version # 1.6, 15 November 2010
**Protocol Title**	A Phase I, Randomised, Controlled, Dose-Escalating, Single-Blind Clinical Trial to Evaluate the Safety and Immunogenicity of JAIVAC-1 Vaccine (PfMSP-1_{19} and PfF2) formulated with Montanide ISA 720 in Healthy Indian Male Subjects between 18 to 45 Years of Age
**Name of the Investigator**	Dr. Preethi Shivyogi
**Name of the Study Site**	Lotus Labs Pvt. Ltd.

"A new vaccine under study against *Plasmodium falciparum* malaria in Indian male subjects in the age group of 18 to 45 years"

You are invited to participate in a research study testing a new vaccine for malaria. Please read this information carefully. Make sure that you understand it to your complete satisfaction before signing your name at the end. Please ask questions if you do not understand any of the information.

INTRODUCTION

You are being asked to take part in a research study which is being conducted by the Investigator (Dr. Preethi Shivyogi) and sponsored by [European Vaccine Initiative (EVI, Universitäts Klinikum Heidelberg - Germany) and International Centre for Genetic Engineering and Biotechnology (ICGEB, New Delhi, India)]. This study is being conducted to find out whether it is safe to administer a new malaria vaccine in humans. A vaccine is any preparation intended to protect against disease by stimulating the production of substances called antibodies. The knowledge gained from this study would be beneficial to those who are at increased risk of getting malaria. This research study involves an experimental vaccine called the JAIVAC-1 – Montanide ISA 720 vaccine. "Experimental" means the vaccine has not been approved for public use by any Authority that regulates new medicines, or other government health authorities such as the Drug Controller General of India (DCGI) in India.

You must be honest with your doctor about your health, now and in the past, or it may not be safe for you to participate in the study. This subject information sheet and informed consent form explain why we are doing the study, the possible benefits and risks of participating in this study, and what you will be asked to do during the study. This consent also explains your rights as a subject involved in a study.

IF YOU HAVE ANY QUESTIONS (CONTACT PERSONNEL)

If you have any questions about this research, the details of the study, or your rights during this study if you choose to participate, following persons are available to answer your questions. His/Her/Their contact information is listed below:

Contact the following person for research or injury-related queries:

Name: Dr. Preethi Shivyogi<br>Role: Investigator	Daytime contact number: +91 9880020530<br>24x7 contact number: +91 9880020530
Name: Dr.James John<br>Role: Co-Investigator	Daytime contact number: +91 9845162433<br>24x7 contact number: +91 9845162433

Name: Dr. Veena B. M. Daytime contact number: +91 9880762781
Role: Co-Investigator 24x7 contact number: +91 9880762781

Contact the following person for subject (your) rights-related queries:

Name: Dr. Manorama Thomas Daytime contact number: +91 8025534621
Role: Member of Ethics Committee

YOUR PARTICIPATION IS VOLUNTARY

Before you learn about the study, it is important that you know the following:

- Your participation is entirely voluntary (you decide whether or not to join the study)
- You may decide not to take part or to withdraw from the study at any time without losing the benefits of your routine medical care.

WHY THIS STUDY IS BEING DONE (STUDY PURPOSE)

This study is a phase I study being done in humans for the first time. Malaria is a major public health problem in many regions like India, Africa, Asia, the Middle East and Central and South America. Malaria is caused by the malaria parasite, which spreads via the bite of infected mosquitoes. Of the various species of the malarial parasite, *Plasmodium falciparum* (transmitted by the Anopheles mosquito) causes the most severe disease and majority of complications and deaths due to malaria. If not treated, malaria can become life-threatening by compromising the blood supply to vital organs. In many parts of the world, the parasite has developed drug resistance and does not respond to a number of malaria medicines. Thus, there is a need to develop a vaccine, which can provide protection against *Plasmodium falciparum* malaria. At present, there are no vaccines available against malaria anywhere in the world; although several vaccines are in the testing stage.

The new vaccine, JAIVAC-1 – Montanide ISA 720 is an experimental vaccine, which may confer protection against *Plasmodium falciparum* malaria. This vaccine has undergone testing in animals and was found to be generally well tolerated and safe to be administered in humans. When administered in animals, the vaccine did not cause any observable harm except mild local reaction at the site of injection that subsided after three-six (3-6) days of administration of the vaccine. The present study is designed to test JAIVAC-1 – Montanide ISA 720 in healthy human male subjects in the age group of 18 to 45 in India and hence you are being asked to participate.

NUMBER OF SUBJECTS AND STUDY CENTERS IN THE STUDY

A total of 45 healthy male adult subjects will participate in this study. The study will be done at only one site in India i.e. Lotus Labs Pvt. Ltd.

WHO CAN BE IN THIS STUDY (STUDY POPULATION)

To participate in the study you must be a male subject in the age group of 18 to 45 years and must be in good health as judged by your investigator. You should not have known previous record or history of malaria or antibodies to the malarial antigens in your blood. You should not have prior history of vaccination with Hepatitis B vaccine. Certain other blood tests like haematology (haemoglobin, cell counts etc.), tests for biochemical substances, HIV, Hepatitis B and C need to be normal to participate in this study. All participating male subjects along with their female partners (as applicable) must agree to use an effective method of birth control while they are in the study.

HOW THIS STUDY WILL BE DONE (STUDY PROCEDURES AND CONDUCT)

The duration of your participation will be approximately 379 days (approximately 13 months) from the day you sign this consent document.

Study entry procedures:

- You must sign an informed consent form before any study entry tests are done.
- After you sign this form, you will be questioned regarding current signs and symptoms of any illness, recent medical history and any chronic medical conditions or drugs you may be taking.
- Physical examination will be done and height, weight and vital signs (body temperature, heart rate, respiratory rate and blood pressure) will be recorded. ECG will be done only at the time of screening (Visit 2).
- Blood sample will be collected for HIV, Hepatitis B and Hepatitis C testing. If you test positive, you will not be included in the study; however you will be appropriately counselled and managed by your investigator as per site policies.
- Blood samples will also be collected for laboratory tests such as like haematology (haemoglobin, cell counts etc.), tests for biochemical substances like potassium, sodium, fasting blood glucose, and special test for evidence of certain blood disorder will also be done. If any of these tests are abnormal, you may not be enrolled in the study. At the discretion of the Investigator, additional laboratory testing maybe performed to assist in identifying any other conditions that may affect your eligibility.
- Your urine will be collected for urinalysis to test for drugs of abuse (e.g. opioids, cocaine etc.).
- All participating male subjects along with their female partners (as applicable) must agree to use an effective method of birth control while you are in the study. Various birth control measures will be discussed with you as a part of birth counselling.

Study conduct:

- In this study, three (3) different dosages of the malaria vaccine (i.e. dosage A - 10+10μg each antigen, dosage B - 25+25 μg each antigen and dosage C - 50+50μg each antigen) will be tested in 30 subjects in a stepped-up fashion while 15 subjects will receive the control vaccine i.e. Hepatitis B vaccine. Subjects will be enrolled gradually such that the 15 subjects at each dosage will be enrolled over a three-day period.
- All the three (3) groups will not start at the same time. Dosing will begin with the dosage A If you are amongst the first three (3) subjects of the first dose, you will be asked to stay at the site for 24 hrs after dosing. If you belong to the subsequent subjects being enrolled, you may or may not be asked to stay at the site for 24 hrs after dosing based on the safety data. In case you are not asked to stay at the site for 24 hrs after dosing, you will be observed at the study site for 3 hours after dosing for any adverse events.
- Data (from day of vaccination to Day 14) obtained from the first 15 subjects will be reviewed by the investigator, an independent safety committee and sponsor and then based on their decision, further dose-escalation to dosage B will be done. Similarly, after review of data obtained from 15 subjects at dosage B, decision to move to the next and last dosage C will be taken.
- **Randomisation:** If you are eligible for study entry, you will be one of the 15 subjects who are assigned to a particular dosage (A or B or C). At that dosage, 10 subjects will receive the experimental vaccine (JAIVAC-1 – Montanide ISA 720 vaccine) and 5 subjects will receive the control vaccine (Hepatitis B). This will be decided as per the randomisation scheme generated by the computer. Thus, at that dosage, you will have a 66% chance of being treated with the experimental vaccine and a 34% chance of receiving the control vaccine.

- **Blinding:** This is a single blind study, which means that only you will not know whether you will be given the experimental vaccine or the control vaccine. However; your investigator and the site team will be aware of the vaccine you are receiving.
- **Dosing:** As per your assigned scheme, you will receive three (3) doses of the study vaccine or the control vaccine. The assigned vaccine will be administered on Days 0, 28 and 180 as an intramuscular injection in the muscle located in the upper and outer part of your dominant or non-dominant arm (as applicable).
- **Follow-up Period:** There will be a follow up period till one (1) year from the first administration and throughout the study you will be followed up for safety. It is very important that you come to all the follow up visits to help collect information for the study to help figure out if the assigned study vaccine is safe and working.

Visit procedures:

- In total, the study would require you to pay 14 visits to the clinic. During the first visit i.e. the pre-screening visit, once you sign the screening consent form, your blood sample will be collected for testing for anti-malarial antibodies.
- Further, if no such anti-malarial antibodies are present in your blood, you will be asked to sign the main informed consent form at the screening visit (Visit 2). Only after signing this main informed consent form, further protocol-defined study procedures will be initiated.
- At Visit 2, you will be assessed for eligibility to take part in the study (as explained above). If you are found eligible you will be randomised into the study and will have to complete Visits 3 through 14 as per the visit schedule. During the study there are certain responsibilities that you will have to fulfil. You are requested to co-operate and fulfil your responsibilities to ensure smooth conduct of the study.
- The importance of strict adherence to the birth control measures in order to avoid pregnancy in your female partner, if any, during the study duration will be emphasised to you.
- Blood samples will be collected throughout the study on Days 28, 56, 180, 208 and 365 for haematology assessments like haemoglobin, cell counts etc as well as biochemical substances like potassium, sodium, fasting blood glucose etc. At the discretion of the Investigator, additional laboratory testing maybe performed to assist in identifying any other conditions that may affect your safety.
- Urine analysis to test for drugs of abuse (benzodiazepines, opioids, amphetamine, cocaine, and THC) will be done during the study on Days 28, 180 and 365. In case, you test positive for the same during the study, you will asked to stop participating in the study.
- On Days 0, 28 and 180, after administration of the assigned study vaccine, you will be monitored for any changes in vital signs (including temperature, pulse, respiratory rate and blood pressure) every 30 minutes for 3 hours post-vaccination. Appropriate medical treatment will be readily available at the study site to manage any adverse event with special emphasis on allergic/anaphylactic reactions.
- A pre-printed diary card will be given to you on the days of vaccine administration (i.e. on Days 0, 28 and 180). Thus, you will receive three (3) separate diary cards for the three (3) doses of the vaccine. You have to record two (2) types of solicited reactions i.e. local solicited adverse events and systemic solicited adverse events. The local solicited reactions are those that occur at the injection-site like pain, swelling, redness and limitation of arm motion at shoulder while the systemic solicited reactions include record of body

temperature and symptoms like headache, malaise, muscle pain, joint pain, nausea and vomiting.

- You will be explained and given detailed instructions about how to record various symptoms/signs in the diary cards for 28 days following every dose of the vaccine. You will be asked to record your temperature every evening for 14 days following each vaccination with the help of a thermometer given to you. A ruler will be given to you to record any swelling or redness at the injection site and the details of how to measure the same will be explained to you by the investigator or study staff. These diary cards will be given to you on the days of vaccine administration (Days 0, 28 and 180) and will be collected 28 days after each vaccination i.e. respectively on Days 28, 56 and 208.
- In addition, all solicited signs and symptoms will also be independently assessed by your investigator 14 days after each vaccination (Days 14, 42 and 194). Also, on Days 28, 56 and 208, the investigator will collect your diary cards and record theirs information into the data collection document.
- It is also important for you to report on the diary card any other diseases apart from the ones mentioned above and that occurred during the first 28 days following each vaccination as well as any serious events (e.g. leading to hospitalisation, etc.) throughout the study period.
- Blood samples will be collected on Days 0, 28, 56, 180, 208 and 365 for serology assessments. Any remaining samples, which are not utilised, may be used later on for other investigations on malaria. The Sponsor will seek permission from the Independent Ethics Committee and applicable laws before starting such kind of research. The remaining samples will never be used for any other purpose other than malaria research. The remaining samples will be kept for 15 years.

ANTICIPATED BENEFITS

The investigational vaccine, JAIVAC-1 could be beneficial to those subjects who are at increased risk of getting malaria and may possibly offer protection against developing malaria while the reference vaccine, Hepatitis B will offer protections against Hepatitis B.

POSSIBLE RISKS AND / OR DISCOMFORTS

Study Vaccine Related Risks: In general, side effects observed after immunisation with malaria vaccines where Montanide ISA 720 was used include local reactions at the injection site like mild to moderate pain, swelling, tenderness and redness and general reactions like nausea, fever, headache and fatigue. Till date, JAIVAC-1 – Montanide ISA 720 has not been tested in humans. However, it has been tested in animals and is found to be generally safe and well tolerated. This vaccine did not cause any severe side effects in animals except for a mild local reaction (redness), which subsided within three-six days of administration.

Administration of the control vaccine Hepatitis B vaccine is associated with mild reactions, which are generally confined to the first few days after vaccine administration. The most common reactions observed with Hepatitis B are mild soreness, redness and hardening at injection site as well as fatigue, fever, malaise and flu-like symptoms. Less common systemic effects include nausea, vomiting, diarrhoea, abdominal pain, abnormal liver function tests, joint pain, muscle pain, itching and skin rashes.

Study Procedures and Risks: Blood samples will be collected for laboratory measurements several times during the course of the study. The amount of blood drawn during each of these visits will vary from approximately 5-30 ml (6 teaspoons). The total amount of blood collected for various

laboratory measurements during the course of the study will be up to approximately 160 ml, which is much less than a blood donation (which is around 350 ml). If the screening period exceed 14 days, approximately 10 ml of blood may be drawn to re-assess the safety parameters for entering in the study. When blood samples are taken, there are possibilities of local bruising and slight pain. Urine will be collected for certain tests explained above and is not risky. Rarely, fainting or infection may also occur. Electrocardiogram will also be done once at the second visit. The electrocardiogram is a routine procedure done to assess the cardiac activity and is painless and not risky. During the study, it is important that you report all symptoms including any new or unusual symptoms to your doctor immediately or during your visit to the study center.

NEW FINDINGS

During the course of the study, your doctor will keep you informed in a timely manner of any significant new information that pertains to your safety or that may affect your willingness to continue participation in this study.

ALTERNATIVES TO BEING IN THE STUDY

Before you decide to take part in this study, your doctor will talk with you about possible alternatives to being in this study, which can include not being in the study or participating in a similar other study.

LEAVING THE STUDY

If you decide to stop being in this study at any time, please contact your doctor who will discuss what treatment you should receive. Except for participation in this study, your decision to leave the study will not affect your future care or treatment by doctors or by this institution. If you leave the study early for any reason, after receiving either of the assigned study vaccines, you will be encouraged to undergo End of Study Visit procedure and continue follow-up procedures including safety monitoring.

REASONS WHY YOU MAY BE TAKEN OFF THE STUDY WITHOUT YOUR CONSENT

You may be taken off the study without your consent at any time for reasons that may include the following:

- Your doctor decides it is unsafe for you to continue in the study
- You need a treatment that is not allowed to be taken during this study
- You are unable to make it to the required study visits or to do all the tests as instructed or follow the instructions
- The Sponsors, the Indian regulatory agency and/or the Independent Ethics Committee at this clinic may also stop the study at any time if required.

If you are discontinued from the study, your doctor will contact you to discuss your continued care. In all cases; where you have received either of the study vaccines, the doctors will want to continue to evaluate you for safety assessments for one year following first vaccination.

CONFIDENTIALITY

The results of this research study will be given to the Sponsor. Your medical and research records may be provided to the Sponsor, the Sponsor's representatives, regulatory agencies, and the ethics committee. The absolute confidentiality of study-related documents may not be guaranteed because of the need to give information to these parties. You will be identified by a code number throughout the study. All personal information made available for inspection will be handled in strictest confidence. Data and information obtained from this study may also be shared with others including researchers who are investigating malarial vaccines. However, your participation in this study or medical information will not be publicly disclosed in a manner that could be identified to

you as an individual. By signing this form, you agree not to restrict the use of any data or results that arise from this study provided such a use is only for scientific purpose.

COMPENSATION

You will not be paid for participating in this research study. However, you will receive 29,500 INR or 30,000 INR (as applicable) as compensation towards cost incurred for your travel to the site during the study. All clinical and professional services, diagnostics, and lab work that are required as part of this study are covered by the Sponsors.

If you decide to participate in the study, you will have to undergo all the procedures explained earlier and provide the study related information to the study doctor and strictly follow the instructions of study doctors. If you follow the directions of the doctors in charge of this study, and you are physically injured because of any substance or procedure improperly given to you under the plan for this study, the sponsors will pay for the treatment. If you require immediate medical care and are not in the clinic at that time, you should seek immediate medical treatment (take your informed consent form with you). Your study doctor will take care of you or help you get the care you need.

By providing medical or other assistance, neither the Sponsors nor the researchers are in any way stating or acknowledging that they are legally responsible for the injury. There is no compensation available for injury resulting from your personal conduct or participation in activities outside the scope of the study protocol.

Non-Waiver of Legal Rights: By your agreement to participate in the research study described in this document and by signing this consent form, you also have been told that you are not waiving any of your legal rights. You also affirm that you have read this consent form in its entirety, and you understand that you will receive a signed copy for your records.

You will be given a copy of this consent form.

MAIN INFORMED CONSENT FORM TO PARTICIPATE IN

THE JAIVAC-1_1_09 STUDY

**Subject's Name (PRINT):**______________________________________<br>**Subject Initials:** ___ ___ ___<br>**Subject's date of birth, Age :** __ __ / __ __ __ / __ __ __ __, ________<br>**Screening Number:** _____________________	**Subject's Initials in box**
I confirm that I have read and understood the Subject Information Sheet dated Final, Version # 1.6, 15 November 2010 for the above study and have had the opportunity to ask questions.	[ ]
I understand that my participation in the study is voluntary and that I am free to withdraw at any time, without giving any reason, without my medical care or legal rights being affected.	[ ]
I understand that the Sponsors of the clinical trial, others working on the Sponsor's behalf, the Independent Ethics Committee and the regulatory authorities will not need my permission to look at my health records both in respect of the current study and any further research that may be conducted in relation to it, even if I withdraw from the trial. I agree to this access. However, I understand that my identity will not be revealed in any information released to third parties or published.	[ ]
I, being an adult subject of reproductive potential agree to use one of the specified and medically-acceptable birth control measures throughout the duration of the study	[ ]
I agree not to restrict the use of any data or results that arise from this study provided such a use is only for scientific purpose(s)	[ ]
I agree to take part in the above study.	[ ]

**Subject's Name (PRINT)**	**Signature**	**Date**
**Name of the person conducting consent (PRINT)**	**Signature**	**Date**

INFORMED CONSENT FORM FOR HIV TESTING IN JAIVAC-1_1_09 STUDY

Screening Number: ____________________

Please read the section below and indicate your agreement by checking the Yes box to the right of the section, completing all blanks, and signing on the line below.

1. I understand that my blood will be tested for HIV after I have been counselled and that my HIV test result will be told to me and kept confidential. ☐ Yes ☐ No

**Subject's Name (PRINT)**	**Date and Signature**
______________________	______________________
**Name of the person conducting consent (PRINT)**	**Date and Signature**
______________________	______________________
