# Supplementary material for: Phase I Clinical Trial of a Recombinant Blood Stage Vaccine Candidate for Plasmodium falciparum Malaria Based on MSP1 and EBA175
Source: PLoS One. 2015 Apr 30;10(4):e0117820. doi: 10.1371/journal.pone.0117820 (PMC4415778; doi:10.1371/journal.pone.0117820)
Supplement: S1 Protocol — (PDF) [file pone.0117820.s005.pdf]

TITLE PAGE

CLINICAL STUDY PROTOCOL

PROTOCOL NO. JAIVAC-1_1_09

A Phase I, Randomised, Controlled, Dose-Escalating, Single-Blind Clinical Trial to Evaluate the Safety and Immunogenicity of JAIVAC-1 Vaccine (PfMSP-1_{19} and PfF2) formulated with Montanide ISA 720 in Healthy Indian Male Subjects between 18 to 45 Years of Age

**Protocol Version**	Final, version # 1.5, 15 November 2010
**Investigational Vaccine**	JAIVAC-1 Vaccine (PfMSP-$1_{19}$and PfF2) formulated with Montanide ISA 720
**Indication**	Recombinant vaccine for *Plasmodium falciparum* malaria
**Clinical Development Phase**	Phase I (first study in humans)
**Amendment(s) with date(s)**	Amendment 1 - Final, version# 1.1, 091030<br>Amendment 2 - Final, version # 1.2, 100212<br>Amendment 3 - Final, version # 1.3, 100506<br>Amendment 4 - Final, version # 1.4, 100614<br>Amendment 5 - Final, version # 1.5, 101115

Information contained in this publication is the property of EVI and ICGEB and is confidential and will not be disclosed to third parties without written authorization from EVI and ICGEB. No part of this document may be reproduced, stored in a retrieval system or transmitted in any form or by any means -electronic, mechanical, recording or otherwise - without the prior authorization of EVI and ICGEB

**Sponsor(s)**	**European Vaccine Initiative (EVI)**<br>UniversitätsKlinikum Heidelberg<br>Im Neuenheimer Feld – 307<br>69120 Heidelberg - Germany<br>**International Centre for Genetic Engineering and Biotechnology (ICGEB)**<br>Aruna Asaf Ali Marg<br>New Delhi-110067, India
**Contract Research Organisation**	**DiagnoSearch Life Sciences Pvt. Ltd. (DLS)**<br>101, First Floor, B.G. House,<br>Orchard Avenue, Hiranandani Business Park,<br>Powai, Mumbai 400076,<br>Maharashtra, India
**Study Site**	**Lotus Labs Pvt. Ltd.**<br>100 ft. Road, 3rd Block, Koramangala<br>Bangalore 560034, India
**Principal Investigator (s)**	**Dr. Preethi Shivyogi**<br>Lotus Labs Pvt Ltd<br>100 ft. Road, 3rd Block, Koramangala<br>Bangalore 560034, India
**Author(s)**	**Supriya Desai, MD**<br>Associate Director, Medical Affairs and Safety Management<br>**Varsha Parulekar, MSc**<br>Manager, Biostatistics<br>**DiagnoSearch Life Sciences Pvt. Ltd. (DLS)**

Information contained in this publication is the property of EVI and ICGEB and is confidential and will not be disclosed to third parties without written authorization from EVI and ICGEB. No part of this document may be reproduced, stored in a retrieval system or transmitted in any form or by any means -electronic, mechanical, recording or otherwise - without the prior authorization of EVI and ICGEB

Co-Author(s)

Nathalie Imbault

Quality Assurance and External Relations Manager

European Vaccine Initiative (EVI)

Chetan Chitnis

Staff Research Scientist

Syed Shams Yazdani

Staff Research Scientist

Shikha Dhawan

Project Manager, Malaria Vaccines

International Centre for Genetic Engineering and Biotechnology (ICGEB)

Information contained in this publication is the property of EVI and ICGEB and is confidential and will not be disclosed to third parties without written authorization from EVI and ICGEB. No part of this document may be reproduced, stored in a retrieval system or transmitted in any form or by any means -electronic, mechanical, recording or otherwise - without the prior authorization of EVI and ICGEB

CLINICAL STUDY PROTOCOL

JAIVAC-1_1_09

PROTOCOL APPROVAL PAGE

**Trial Identifier**	JAIVAC-1_1_09
**Protocol version, date**	Final, Version # 1.5, 15 November 2010
**Protocol Title**	A Phase I, Randomised, Controlled, Dose-escalating, Single-blind Clinical Trial to Evaluate the Safety and Immunogenicity of the JAIVAC-1 Vaccine (PfMSP-1_{19} and PfF2) formulated with Montanide ISA 720 in healthy Indian adults between 18 to 45 years of age.

Prepared by: DiagnoSearch Life Sciences Pvt. Ltd. (DLS)

Supriya Desai, MD<br>Associate Director, Medical Affairs and Safety Management	SCDesai<br>15 NOV 2010
Varsha Parulekar, MSc<br>Manager, Biostatistics	Parulekar<br>15/11/2010

Approved by: European Vaccine Initiative (EVI) and International Centre for Genetic Engineering and Biotechnology (ICGEB)

Dr Egeruan Babatunde Imoukhuede<br>Director of Clinical and Regulatory Affairs	[illegible]<br>15/11/2010
Dr. Chetan Chitnis<br>Staff Research Scientist	[illegible]<br>15/11/2010

GCP Statement: *The clinical trial will be conducted in compliance with the protocol, ICH-GCP and the applicable regulatory requirement(s)*

Confidentiality Statement: *The information contained in this document is confidential and the property of European Vaccine Initiative and International Centre for Genetic Engineering and Biotechnology. It is intended for the use of clinical investigators and it is understood that the information will not be disclosed to others than the investigator(s) and his/her/their staff, applicable IEC(s)/IRB(s) and regulatory authorities without prior written approval from European Vaccine Initiative and International Centre for Genetic Engineering and Biotechnology except to the extent necessary to obtain informed consent from those persons to whom the candidate vaccine may be administered. Further dissemination may only be made with the express written information of European Vaccine Initiative and International Centre for Genetic Engineering and Biotechnology.*

Information contained in this publication is the property of EVI and ICGEB and is confidential and will not be disclosed to third parties without written authorization from EVI and ICGEB. No part of this document may be reproduced, stored in a retrieval system or transmitted in any form or by any means -electronic, mechanical, recording or otherwise - without the prior authorization of EVI and ICGEB

CLINICAL STUDY PROTOCOL

JAIVAC-1_1_09

INVESTIGATOR'S SIGNATURE PAGE

"By my signature below, I, Dr. Preethi Shivyogi, hereby confirm that I have read this protocol and I understand it. I will conduct the study described in Protocol No. JAIVAC-1_1_09 (Final, Version # 1.5, 15 November 2010) in compliance with ethical principles stated in the latest version of the Declaration of Helsinki, the principles and guidelines of Good Clinical Practices, ICH guidelines and applicable regulatory guidelines and laws and as per the version of such protocol agreed to by the applicable regulatory authority (ies) and approved by all reviewing IRBs/IECs. I will accept overseeing of the study by European Vaccine Initiative and International Centre for Genetic Engineering and Biotechnology designated monitor. I will abide by the communication and publication plan set forth in my agreement with European Vaccine Initiative and International Centre for Genetic Engineering and Biotechnology. I will promptly submit the protocol to applicable independent ethical committee(s) or institutional review board(s)."

**Principal Investigator (Name and Title):**	**Signature and Date:**
DR. PREETHI SHIVYOGI	[signature] 16·NOV·2010
**Institution (address and phone number, or stamp):** LOTUS LABS PRIVATE LIMITED 100 feet Road, 3rd BLOCK, KORAMANGALA BANGALORE - 560034 INDIA	
**Sponsor Representative (Name and Title):** DR. CHETAN CHITNIS STAFF RESEARCH SCIENTIST, ICGEB	**Signature and Date:** [signature] 18/11/10
DR EGERUAN BABATUNDE IMOUKHUEDE DIR, CLINICAL AND REGULATORY AFFAIRS	[signature] 20/11/10

Information contained in this publication is the property of EVI and ICGEB and is confidential and will not be disclosed to third parties without written authorization from EVI and ICGEB. No part of this document may be reproduced, stored in a retrieval system or transmitted in any form or by any means -electronic, mechanical, recording or otherwise - without the prior authorization of EVI and ICGEB

STUDY SYNOPSIS

**Clinical Trial Identifier:**	JAIVAC-1_1_09
**Study Title:**	A Phase I, Randomised, Controlled, Dose-escalating, Single-blind Clinical Trial to Evaluate the Safety and Immunogenicity of the JAIVAC-1 Vaccine (PfMSP-1_{19} and PfF2) formulated with Montanide ISA 720 in healthy Indian male subjects between 18 to 45 years of age.
**Sponsors (Legal Names):**	1. European Vaccine Initiative (EVI), Heidelberg, Germany. International Centre for Genetic Engineering and Biotechnology (ICGEB), New Delhi, India
**Biological Evaluator(s):**	Paushali Mukherjee, ICGEB
**Contract Research Organisation:**	DiagnoSearch Life Sciences Pvt. Ltd. (DLS), Mumbai, Maharashtra, India
**Study Site:**	Lotus Labs Pvt. Ltd.<br>100 ft. Road, 3rd Block, Koramangala<br>Bangalore 560034, India
**Principal Investigator:**	Dr. Preethi Shivyogi<br>Lotus Labs Pvt Ltd<br>100 ft. Road, 3rd Block, Koramangala<br>Bangalore 560034, India
**Clinical Development Phase:**	Phase I (first study in humans)
**Name of the Investigational Vaccine:**	JAIVAC-1 *Plasmodium falciparum* malaria vaccine formulated with Montanide ISA 720 (JAIVAC-1 – Montanide ISA 720)
**Active Ingredient(s) of the Investigational Vaccine:**	PfMSP-1_{19} and PfF2

Information contained in this publication is the property of EVI and ICGEB and is confidential and will not be disclosed to third parties without written authorization from EVI and ICGEB. No part of this document may be reproduced, stored in a retrieval system or transmitted in any form or by any means -electronic, mechanical, recording or otherwise - without the prior authorization of EVI and ICGEB

**Dose(s) of the Investigational Vaccine:**	The three (3) dosages of antigen of the investigational vaccine to be evaluated in the present study include:<br>▪ 10 µg of each antigen (PfMSP-1_{19} and PfF2), 0.1 ml of the reconstituted and emulsified (JAIVAC-1 – Montanide ISA 720) vaccine (**Dosage A**)<br>▪ 25 µg of each antigen (PfMSP-1_{19} and PfF2), 0.25 ml of the reconstituted and emulsified (JAIVAC-1 – Montanide ISA 720) vaccine (**Dosage B**)<br>▪ 50 µg of each antigen (PfMSP-1_{19} and PfF2), 0.5 ml of the reconstituted and emulsified (JAIVAC-1 – Montanide ISA 720) vaccine (**Dosage C**)
**Name of the Adjuvant:**	Montanide ISA 720
**Name of the Comparator Vaccine:**	Hepatitis B vaccine
**Vaccination Schedule:**	All subjects will receive three (3) doses of the assigned investigational vaccine or control vaccine on Days 0, 28 and 180 by intramuscular route. Follow-up duration is 365 days/ one (1) year after the first administration of the vaccine.
**Study Indication:**	Recombinant vaccine for *Plasmodium falciparum* malaria
**Primary Objective(s):**	▪ To evaluate the safety of three different dosages of JAIVAC-1 malaria vaccine (PfMSP-1_{19} and PfF2) formulated with Montanide ISA 720 as adjuvant in healthy Indian male subjects between 18 to 45 years of age<br>▪ To compare the safety of three different dosages of JAIVAC-1 vaccine (PfMSP-1_{19} and PfF2) formulated with Montanide ISA 720 as adjuvant to the safety of the control vaccine (Hepatitis B vaccine) in healthy Indian male subjects between 18 to 45 years of age
**Secondary Objective(s):**	▪ To assess the humoral response to the vaccine antigens PfMSP-1_{19} and PfF2 by measuring the IgG antibody response by Enzyme Linked Immunosorbent Assay (ELISA) in healthy Indian male subjects between 18 to 45 years of age<br>▪ To assess the humoral response to the vaccine antigens PfMSP-1_{19} and PfF2 by measuring the IgG antibody response by Immunofluorescence Assay (IFA) in healthy Indian male subjects between 18 to 45 years of age

Information contained in this publication is the property of EVI and ICGEB and is confidential and will not be disclosed to third parties without written authorization from EVI and ICGEB. No part of this document may be reproduced, stored in a retrieval system or transmitted in any form or by any means -electronic, mechanical, recording or otherwise - without the prior authorization of EVI and ICGEB

**Exploratory Objective(s):**	▪ To assess the quality of the humoral immune response (IgG antibody) to the vaccine antigens in healthy Indian male subjects by measuring the following:<br>o Subclasses of IgG antibodies - IgG1, IgG2, IgG3, IgG4 by ELISA<br>o The ability of the IgG antibodies to block homologous and heterologous parasite growth *in vitro* by a *P. falciparum* blood-stage growth inhibition assay (GIA) against three parasite strains

Information contained in this publication is the property of EVI and ICGEB and is confidential and will not be disclosed to third parties without written authorization from EVI and ICGEB. No part of this document may be reproduced, stored in a retrieval system or transmitted in any form or by any means -electronic, mechanical, recording or otherwise - without the prior authorization of EVI and ICGEB

Study Design:

The present study is designed as a randomised, controlled, single-blind and dose-escalating clinical study for the assessment of the safety and immunogenicity of JAIVAC-1 malaria vaccine (PfMSP-1_{19} and PfF2) formulated with Montanide ISA 720 as adjuvant in healthy Indian male subjects between 18 to 45 years of age. This is a dose-escalating study and involves testing of three (3) different dosages of JAIVAC-1 malaria vaccine (PfMSP-1_{19} and PfF2) formulated with Montanide ISA 720 as adjuvant with the intramuscular administration of the investigational vaccine as a 3-dose schedule on Days 0, 28 and 180.

30 subjects will receive JAIVAC-1—Montanide ISA720 (10 subjects at each dosage cohort) and 15 subjects will receive control vaccine, Hepatitis B vaccine (5 subjects at each dosage cohort). Dosing of the JAIVAC-1—Montanide ISA720 vaccine arms will begin with Cohort 1 (i.e. 0.1 ml of investigational vaccine administered by intramuscular route – Dosage A), followed by Cohort 2 (i.e. 0.25 ml of investigational vaccine – Dosage B) and finally Cohort 3 (i.e. 0.5 ml of investigational vaccine – Dosage C). Each cohort will be staggered into sub-cohorts such that the subjects in each cohort will be enrolled over a three-day period. The first three (3) subjects of the first dose (Cohort 1) will be kept under observation for 24 hrs following vaccination. The decision of the 24-hour housing for the remaining subjects will be jointly taken by the Principal Investigator and Medical and Safety Monitor, DLS.

An independent Data Safety Monitoring Board (DSMB) will be appointed for this study. Dose escalation to the next higher dosage cohort of JAIVAC-1 – Montanide ISA 720 will occur after the safety data from the first dose of vaccine through Day 14 day for all 15 subjects of the previous dosage cohort has been reviewed by the DSMB comprising of three (3) independent clinicians with relevant expertise in the field of vaccines development and evaluation of vaccine safety along with the designated Local Safety Monitor (who would be a designated personnel from the investigational site but independent of the study team).

Thus, for each dosage cohort, each subject will receive a total of three (3) doses of either of the study or control vaccines by intramuscular route, one each on Day 0, Day 28 and Day 180 respectively.

Information contained in this publication is the property of EVI and ICGEB and is confidential and will not be disclosed to third parties without written authorization from EVI and ICGEB. No part of this document may be reproduced, stored in a retrieval system or transmitted in any form or by any means -electronic, mechanical, recording or otherwise - without the prior authorization of EVI and ICGEB

Dosing Cohorts:

There will be three (3) cohorts in this study such that each cohort will consist of 15 eligible subjects randomised in a ratio of 2:1 as follows:

Cohort 1

**Test Arm 1**	10 subjects shall receive dosage A of investigational vaccine (0.1 ml of JAIVAC-1 vaccine containing 10µg PfMSP-1_{19} and 10µg PfF2 formulated with adjuvant Montanide ISA 720) by intramuscular route on Days 0, 28 and 180
**Control Arm 1**	5 subjects shall receive control vaccine (1 ml of Hepatitis B vaccine) by intramuscular route on Days 0, 28 and 180

Cohort 2

**Test Arm 2**	10 subjects shall receive dosage B of investigational vaccine (0.25 ml of JAIVAC-1 vaccine containing 25µg PfMSP-1_{19} and 25µg PfF2 formulated with adjuvant Montanide ISA 720) by intramuscular route on Days 0, 28 and 180
**Control Arm 2**	5 subjects shall receive control vaccine (1 ml of Hepatitis B vaccine) by intramuscular route on Days 0, 28 and 180

Cohort 3

**Test Arm 3**	10 subjects shall receive dosage C of investigational vaccine (0.5 ml of JAIVAC-1 vaccine containing 50µg PfMSP-1_{19} and 50µg PfF2 formulated with adjuvant Montanide ISA 720) by intramuscular route on Days 0, 28 and 180
**Control Arm 3**	5 subjects shall receive control vaccine (1 ml of Hepatitis B vaccine) by intramuscular route on Days 0, 28 and 180

Information contained in this publication is the property of EVI and ICGEB and is confidential and will not be disclosed to third parties without written authorization from EVI and ICGEB. No part of this document may be reproduced, stored in a retrieval system or transmitted in any form or by any means -electronic, mechanical, recording or otherwise - without the prior authorization of EVI and ICGEB

**Number of Subjects:**	A sufficient number of subjects will be screened to enrol a total number of 45 subjects in the study.<br>• 30 subjects will receive the investigational vaccine (i.e. JAIVAC-1 – Montanide ISA 720) with ten (10) subjects in each dosage cohort<br>and<br>• 15 subjects will receive the control vaccine (i.e. Hepatitis B vaccine) with five (5) subjects in each dosage cohort.<br>Subjects who are discontinued from the study following administration of the first dose of the assigned vaccine will be considered as drop-outs and will not be subsequently replaced.
**Inclusion Criteria**	1. Male subject aged 18 to 45 years (both inclusive)<br>2. Subject with general good health based on the medical history and clinical examination<br>3. Subject must be willing to sign the Informed Consent Form<br>4. Subject must be reachable by phone during the entire study period (12 months)<br>5. Subject must be capable and willing to complete and return diary cards and to attend all follow-up visits<br>6. Male subject must agree to use one of the following medically-acceptable birth control measures throughout the duration of the study (birth control counselling and measures will be provided by sites as required)<br>• Double barrier method (e.g. condom with spermicidal jelly) used for the entire study period<br>Or<br>• Should be Surgically sterile (vasectomy)

Information contained in this publication is the property of EVI and ICGEB and is confidential and will not be disclosed to third parties without written authorization from EVI and ICGEB. No part of this document may be reproduced, stored in a retrieval system or transmitted in any form or by any means -electronic, mechanical, recording or otherwise - without the prior authorization of EVI and ICGEB

Exclusion Criteria:

1. Subject with evidence of IgG antibodies against PfMSP-1_{19} and PfF2 as measured by ELISA
2. Subject with prior history of immunisation with Hepatitis B vaccine
3. Subject with known history of malaria
4. Subject with history of allergic reactions, hypersensitivity or anaphylaxis to any of the components of the study vaccines (JAIVAC-1 – Montanide ISA 720 malaria vaccine or Hepatitis B vaccine) (including adjuvant or peptide) or with history of serious allergic reactions to any substance, requiring hospitalisation or emergency medical care
5. Subject with previous vaccination with any other malaria candidate vaccines
6. Subject with use of an investigational or non-registered drug or vaccine other than the study vaccines within three (3) months preceding the first study vaccination, or planned use during the entire clinical trial period
7. Subject, who receives any vaccination or gamma globulin during the three-month period prior to the first vaccination
8. Subject with chronic administration (defined as more than 14 days) of immuno-suppressants or other immune-modifying drugs within six months prior to the first vaccination. This includes any dose level of oral steroids or inhaled steroids, but not topical steroids
9. Subjects will be excluded if AST > 40 IU/L, ALT > 41 IU/L, γ GT > 71 IU/L, Total Bilirubin > 1.2 mg/ dL, Indirect Bilirubin > 1.2 mg/ dL, Direct Bilirubin > 0.4 mg/dL, Serum Creatinine > 1.2 mg/ dL(Appendix B and B-1).
10. Subjects will be excluded in case of out of range values for the following parameters: Hemoglobin 13 to 18 g/ dL, RBC count 4.0 to 7.0 × 10E6/µL TLC 4.0 to 11.0 × 10E3/µL, platelet count 150 to 500 × 10E3/µL, Neutrophils 40 to 75 % or Eosinophils > 10 %, Sodium 136 to 145 mEq/L, Potassium 3.5 to 5.1 mEq/L, Random Blood Glucose 45 to 130 mg/dl and Alkaline Phosphatase 40 to 129 U/L (Appendix B and B-1)
11. Subjects with other clinically significant abnormal laboratory values based on the normal reference range (Refer Appendix B and B1) apart from the laboratory parameters listed above.

Information contained in this publication is the property of EVI and ICGEB and is confidential and will not be disclosed to third parties without written authorization from EVI and ICGEB. No part of this document may be reproduced, stored in a retrieval system or transmitted in any form or by any means -electronic, mechanical, recording or otherwise - without the prior authorization of EVI and ICGEB

12. Subject with positive HIV, HBV or HCV test
13. Subject with positive direct Coomb's test
14. Subject with a peripheral blood film/smear with features of haemolysis or any other haematological disorder
15. Subject unable to give written informed consent
16. Subject unable to be closely followed for social, geographic or psychological reasons
17. Subject with previous history of drug or alcohol abuse interfering with normal social function during a period of one year prior to enrolment in the study or positive urine drug test
18. Subject with an abnormal 12-lead ECG at screening associated with relevant clinical symptoms/signs suggestive of cardiac pathology (including conduction disturbances)
19. Subject with an abnormal Chest X-Ray associated with relevant clinical symptoms/signs of respiratory pathology at screening/ anytime in the past 6 months
20. Subject with known history of congenital/hereditary immunodeficiency disorders
21. Subject with acute or chronic pulmonary, reticulo-endothelial, cardiovascular, hepatic, renal or neurological condition, or any other clinical findings that in the opinion of the clinical investigator, may increase the risk of participating in the study
22. Subject with any other condition that in the opinion of the clinical investigator would jeopardise the safety or rights of a participant in the trial or would render the participant unable to comply with the protocol

Information contained in this publication is the property of EVI and ICGEB and is confidential and will not be disclosed to third parties without written authorization from EVI and ICGEB. No part of this document may be reproduced, stored in a retrieval system or transmitted in any form or by any means -electronic, mechanical, recording or otherwise - without the prior authorization of EVI and ICGEB

**Planned duration of Study Participation/Study period:**	The total duration of each subject's participation (excluding allowed window periods) will be approximately 386 days which includes:<br>• Pre-Screening Period of 7 days<br>• Screening period of 14 days<br>• Vaccination period of 180 days (refer window period by visit)<br>• Follow-up period till 365 days (1 year) after the first vaccination (window period of +/- 7 days)<br>The approximately, total study duration (476 days) includes enrolment period (90 days) and duration of participation of individual subjects (386 days).

Information contained in this publication is the property of EVI and ICGEB and is confidential and will not be disclosed to third parties without written authorization from EVI and ICGEB. No part of this document may be reproduced, stored in a retrieval system or transmitted in any form or by any means -electronic, mechanical, recording or otherwise - without the prior authorization of EVI and ICGEB

**Primary Endpoint(s)**	The safety profile will be assessed on the basis of the following criteria<br>▪ Immediate reactogenicity (any event occurring within the first three (3) hours after each vaccination, with emphasis on allergic reactions)<br>▪ Local and systemic reactogenicity (any event occurring from three (3) hours post vaccination on Day 0 till Day 14 after each dose)<br>▪ Any unsolicited adverse events 28 days after each vaccination<br>▪ Any Serious Adverse Event (SAE) occurring from the first dose of vaccine till the last follow-up visit.<br>▪ Biological safety, 28 days after each vaccination, in reference with the baseline before the first dose, by measuring the following parameters:<br>• Haematology: RBC Count, Haemoglobin*, Haematocrit/Packed Cell Volume (PCV), MCV (Mean Corpuscular Volume), MCH (Mean Corpuscular Haemoglobin), MCHC (Mean Corpuscular Haemoglobin Concentration) on Days -14, 28, 56, 180, 208, 365<br>• Platelet Count and Total Leukocyte Count (TLC) along with Differential Leukocyte Count (DLC) on Days -14, 28, 56, 180, 208, 365.<br>*If the haemoglobin drops below 13 gm/dL, then a direct Coomb's test and a peripheral blood smear/film will be prepared and examined for evidence of possible haemolysis<br>• Serum Chemistry: Potassium, Sodium, AST, ALT, Direct, Indirect and Total Bilirubin, Alkaline Phosphatase, Gamma Glutamyl Transpeptidase (γGT), Creatinine, and Random blood glucose on Days -14, 28, 56, 180, 208, 365<br>The Investigator will be responsible for causality assessment i.e. assessment of the relationship of the AE to either of the assigned the study vaccines, using the following definitions: related or not related.

Information contained in this publication is the property of EVI and ICGEB and is confidential and will not be disclosed to third parties without written authorization from EVI and ICGEB. No part of this document may be reproduced, stored in a retrieval system or transmitted in any form or by any means -electronic, mechanical, recording or otherwise - without the prior authorization of EVI and ICGEB

**Secondary Endpoint(s)**	▪ The humoral response to the candidate vaccine antigen will be assessed (quantitative assessment) by measuring the level of IgG antibodies developed against PfMSP-1_{19} and PfF2 by ELISA on Days 0, 28, 56, 180, 208 and 365<br>▪ The humoral response to the candidate vaccine antigen will be assessed qualitatively to verify the ability of the IgG antibodies developed against PfMSP-1_{19} and PfF2 to recognise the native protein on late stage *P. falciparum* schizonts and merozoites *in vitro* by Immunoflouresecence Assay (IFA) on Days 0, 28, 56, 180, 208 and 365
**Exploratory Endpoint(s)**	The quality of the humoral immune response will be additionally assessed by measuring the following:<br>▪ IgG1, IgG2, IgG3, IgG4 subclasses by ELISA on samples obtained at Days 0, 28, 56, 180, 208 and 365<br>▪ The ability of the IgG antibodies to block homologous and heterologous parasite growth in vitro by a *P. falciparum* blood-stage Growth Inhibition Assay (GIA) against three parasite strains on samples obtained at Days 0, 56, 208 and 365
**Statistical Methods:**	The analysis shall be descriptive as the sample size does not allow any comparison between groups. Categorical variables will be summarised by vaccine groups as frequency, percentages and 95% confidence interval. Continuous variables other than titers and concentrations will be summarised by vaccine groups as Mean, SEM, Median, Minimum, Maximum, inter-quartile range/range. The proportion of subjects that received three (3) doses without experiencing grade 3 adverse events will be estimated by Exact Binomial Proportion (Proportion and 95% confidence interval).

Information contained in this publication is the property of EVI and ICGEB and is confidential and will not be disclosed to third parties without written authorization from EVI and ICGEB. No part of this document may be reproduced, stored in a retrieval system or transmitted in any form or by any means -electronic, mechanical, recording or otherwise - without the prior authorization of EVI and ICGEB

OVERALL STUDY DESIGN

JAIVAC-1_1_09
STUDY DESIGN

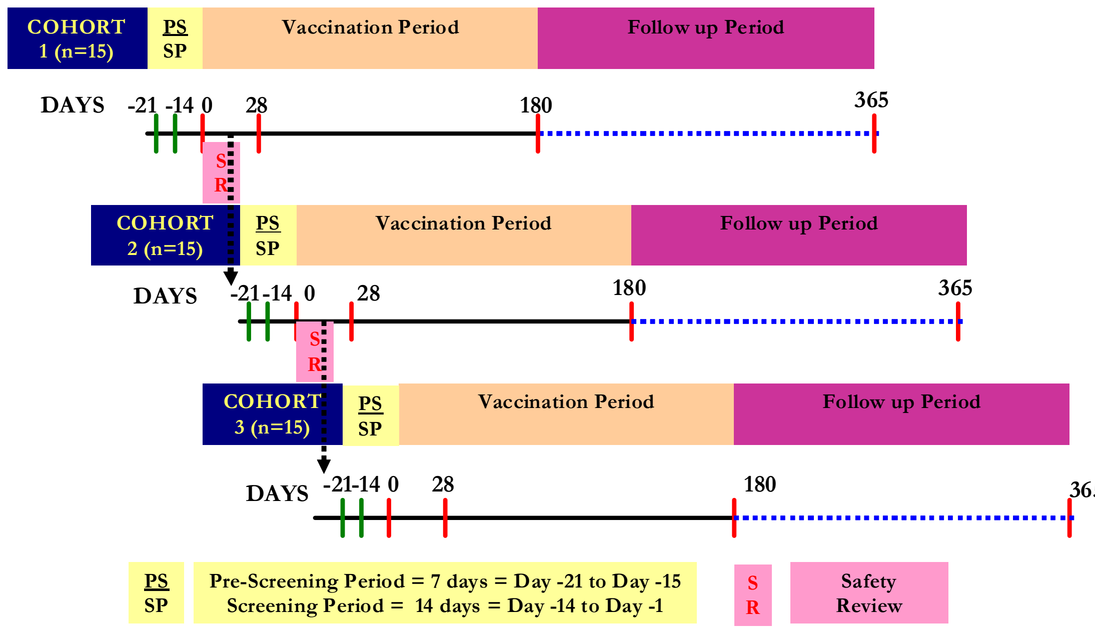

FOOTNOTES:

The defined study period for the JAIVAC-1_1_09 study consists of four (4) distinct study periods; a pre-screening period of seven (7) days, a screening period of 14 days, a vaccination period of 180 days and a follow-up period till 365 days from the first dose of the assigned study vaccine.

Each cohort will consist of 15 subjects. Dosing of the JAIVAC-1 – Montanide ISA 720 investigational vaccine arms will begin with Cohort 1 (10µg PfMSP-1_{19} + 10µg PfF2 i.e. Dosage A), followed by Cohort 2 (25µg PfMSP-1_{19} + 25µg PfF2 i.e. Dosage B) and finally Cohort 3 (50µg PfMSP-1_{19} + 50µg PfF2 i.e. Dosage C) as per the common vaccine administration schedule (administered as a 3-dose schedule on Days 0, 28 and 180 by intramuscular route) at each dosage. Each cohort will be staggered into sub-cohorts such that the subjects in each cohort will be enrolled over a three-day period. The first three (3) subjects of the first dose (Cohort 1) will be kept under observation for 24 hrs following vaccination. The decision of the 24-hour housing for the remaining subjects will be jointly taken by the Principal Investigator and Medical and Safety Monitor, DLS.

Dose escalation to the next higher dosage cohort of JAIVAC-1 – Montanide ISA 720 will occur after review of the appropriately monitored and verified safety data obtained from the first dose of the vaccine through Day 14 for all 15 subjects in the previous dosage cohort. The "Safety Review" will be undertaken by the DSMB comprising of three (3) independent clinicians with relevant expertise along with the designated Local Safety Monitor (who would be a designated personnel from the investigational site but independent of the study team).The decision to begin the next cohort will be taken in the next 7 days. Thus, the next dosage cohort may begin at the earliest at 21 days after the 15th subject enrolled in the given dosage cohort has been administered the first dose of the JAIVAC-1 – Montanide ISA 720.

Information contained in this publication is the property of EVI and ICGEB and is confidential and will not be disclosed to third parties without written authorization from EVI and ICGEB. No part of this document may be reproduced, stored in a retrieval system or transmitted in any form or by any means -electronic, mechanical, recording or otherwise - without the prior authorization of EVI and ICGEB

VISIT SCHEDULE

TABLE 1: PROTOCOL JAIVAC-1_1_09 - SCHEDULE OF VISITS AND PROCEDURES

Visit	1	2	3	4	5	6	7	8	9	10	11	12	13	14
Day	*Day -21 to Day -15*	*-14 to Day -1*	*0*	*7*	*14*	*28*	*35*	*42*	*56*	*180*	*187*	*194*	*208*	*365*
Window Period [1]			*± 1 day*	*± 1 day*	*± 1 day*	*± 1 day*	*± 1 day*	*± 1 day*	*± 1 day*	*± 1 day*	*± 1 day*	*± 1 day*	*± 1 day*	*± 7 days*
Study Period [2]	Pre-Screening Period	Screening Period	Vaccination Period								Follow up Period			
Vaccination			Dose 1			Dose 2				Dose 3				
Informed Consent[3]	*x*	*x*												
Screening Number[4]	*x*	*x*												
Demographics[5]	*x*													
ELISA for Anti-malaria Antibodies[6]	*x*													
Medical History		*x*												
Concomitant Medications		*x*	*x*	*x*	*x*	*x*	*x*	*X*	*x*	*x*	*x*	*x*	*x*	*X*
Physical Examination		*x*	*x*	*x*	*x*	*x*	*x*	*X*	*x*	*x*	*x*	*x*	*x*	*X*
Vital Signs (TPR and BP)[7]		*x*	*x*	*x*	*x*	*x*	*x*	*X*	*x*	*x*	*x*	*x*	*x*	*X*
12-Lead Electrocardiogram and X-Ray Chest[7]		*x*												
Birth Control Counselling[8]		*x*	*x*			*x*				*x*				
Haematology[9]		*x*				*x*			*x*	*x*			*x*	*x*
Serum Chemistry[10]		*x*				*x*			*x*	*x*			*x*	*x*
HIV, HBV, HCV Tests		*x*												
Direct Coomb's Test[11]		*x*				*x*			*x*	*x*			*x*	*x*
Peripheral Blood Smear [12]		*x*				*x*			*x*	*x*			*x*	*x*
Urine Drug Screen[13]		*x*				*x*				*x*				*x*
Check Inclusion Criteria		*x*	*x*											
Check Exclusion Criteria		*x*	*x*											
Randomisation number[14]			*x*											
Distribute Diary Cards[15]			*x*			*x*				*x*				
Collect Diary Cards [15]						*x*			*x*				*x*	
Review Diary Cards[15]				*x*	*x*		*x*	*X*			*x*	*x*		
Transcribe Diary Cards [15]				*x*	*x*	*x*	*x*	*X*	*x*		*x*	*x*	*x*	
Record Immediate Reactogenicity[16]			*x*			*x*				*x*				
Record Solicited AEs[17]			*x*	*x*	*x*	*x*	*x*	*X*		*x*	*x*	*x*		
Record Unsolicited AEs			*x*	*x*	*x*	*x*	*x*	*X*	*x*	*x*	*x*	*x*	*x*	
Record SAEs			*x*	*x*	*x*	*x*	*x*	*X*	*x*	*x*	*x*	*x*	*x*	*x*
Serology (ELISA and IFA)[18]			*x*			*x*			*x*	*x*			*x*	*x*
Serology (IgG subclasses)[19]			*x*			*x*			*x*	*x*			*x*	*x*
Serology (GIA)[19]			*x*						*x*				*x*	*x*
Check Discontinuation Criteria				*x*	*x*	*x*	*x*	*X*	*x*	*x*	*x*	*x*	*x*	

Please refer to the footnotes below.

Information contained in this publication is the property of EVI and ICGEB and is confidential and will not be disclosed to third parties without written authorization from EVI and ICGEB. No part of this document may be reproduced, stored in a retrieval system or transmitted in any form or by any means -electronic, mechanical, recording or otherwise - without the prior authorization of EVI and ICGEB

Following are the footnotes for TABLE 1: PROTOCOL JAIVAC-1_1_09 - SCHEDULE OF VISITS AND PROCEDURES

1. **Window Period:** A window period of specific number of days has been assigned for each visit. (*Refer Table 1 for details*). In case a subject cannot come on a scheduled day he can be called anytime in the window period for that particular visit but for subsequent visits he should be urged to adhere to the original visit schedule.
2. **Study Period:** The study consists of four (4) distinct study periods: A pre-screening period of 7 days, a screening period of 14 days, a vaccination period of 180 days and a follow-up period till 365 days from the first dose of the assigned study vaccines.
3. **Informed Consent:** There are two (2) discreet informed consent documents for this study. Following the signing of the pre-screening informed consent, blood sample for testing for anti-malarial antibodies will be taken. Only if the subject tests negative for anti-malarial antibodies, he will be administered the main informed consent form. Following the signing of the main informed consent, further study-related procedures will be undertaken.
4. **Screening number:** Following the signing of the pre-screening informed consent, each subject will be assigned a unique screening number. This same number will be used to identify the subject both during the pre-screening as well as the screening period.
5. **Demographics:** At the pre-screening Visit (Visit 1), each subject shall be required to provide demographic information (including date of birth, height, weight, gender, and race).
6. **ELISA for Anti-malarial Antibodies:** Blood sample drawn in the pre-screening period will be used for ELISA test for anti-malarial antibodies. Subjects with evidence of IgG antibodies against PfMSP-1_{19} and PfF2 as measured by ELISA at pre-screening visit (Visit 1) will be not be further considered for the study.
7. **Vital Signs and 12-lead ECG:** General examination including vital signs i.e. temperature, pulse, respiratory rate (TPR) and blood pressure (supine BP) will be performed at every visit. One 12-lead ECG and one X-Ray Chest will be done at the screening visit (Visit 2).
8. **Birth Control Couselling:** The importance of strict adherence to the birth control measures during the study duration will be emphasised at these visits to the subjects.
9. **Haematology:** Blood samples will be collected for haematology assessments (including RBC Count, haemoglobin, haematocrit, MCV, MCH, MCHC, platelet count, TLC and DLC) at screening visit (any time from D-14 through D-1), D28, D56, D180, D208 and D365. Haematology assessments done at Screening Visit (Visit 2) will be considered as screening laboratory assessments for eligibility as well as baseline assessments for safety end-points since haematology assessments will not be done on Day 0 (pre-vaccination). Blood samples for haematology will be collected prior to vaccination on Days 28 and 180.
10. **Serum Chemistry:** Blood samples will be collected for serum chemistry assessments (including potassium, sodium, AST, ALT, direct, indirect and total bilirubin, alkaline phosphatase, γGT, creatinine, random blood glucose) during screening period (D-14 through D-1), D28, D56, D180, D208 and D365. Serum chemistry assessments done at Screening Visit (Visit 2) will be considered as screening laboratory assessments for eligibility as well as baseline assessments for safety end-points since serum chemistry assessments will not be done on Day 0 (pre-vaccination). Blood samples for serum chemistry will be collected prior to vaccination on Days 28 and 180.

Information contained in this publication is the property of EVI and ICGEB and is confidential and will not be disclosed to third parties without written authorization from EVI and ICGEB. No part of this document may be reproduced, stored in a retrieval system or transmitted in any form or by any means -electronic, mechanical, recording or otherwise - without the prior authorization of EVI and ICGEB

11. **Direct Coomb's Test:** Direct Coomb's test will be undertaken at Screening Visit (Visit 2) and additionally will be done during the entire study period if the haemoglobin falls below 13 gm/dL
12. **Peripheral Blood Smear (PBS):** PBS examination will be undertaken for evidence of haemolysis or any haematological disorder at Screening Visit (Visit 2) and additionally during the entire study period along with Coomb's test if the haemoglobin falls below 13 gm/dL
13. **Urine Drug Screen:** Urinalysis to test for drugs of abuse (benzodiazepines, opioids, amphetamine, cocaine, and THC) will be undertaken once at the Screening Visit (Visit 2) and subsequently on D28, D180 and D365. If the subject tests positive during screening period, he will be excluded from the study. If the subject tests positive on D28, D180 or D365, he will be immediately discontinued from the study.
14. **Randomisation Number:** Subjects, who fulfill all the eligibility criteria, will be randomised into the study with the assignment of a unique randomisation number.
15. **Diary Cards:** A pre-printed diary card will be given to the subject with detailed completion instructions on the day of vaccine administration and will be collected on Day 28 after each dose of vaccine. This diary card will be used for the record of solicited AEs (Day 0 through Day 14) and unsolicited AEs (Day 0 through Day 28). Thus three (3) such diary cards will be given to each subject. The Investigator will collect, review and transcribe the data from diary cards to CRF at pre-defined time-points.
16. **Record Immediate Reactogenicity:** Following vaccination on Days 0, 28 and 180, all subjects will be monitored at the site every 30 minutes till 3 hours for any immediate reactions with special emphasis on allergic/anaphylactic reactions. Vital signs will be done after the subject has been resting quietly for at least 5 minutes. BP will be taken in the supine position.
17. **Record Solicited AEs:** Each subject will record solicited AEs from vaccination through Day 14 after each dose on the diary card. These solicited AEs will be reviewed and transcribed from diary cards to CRF by the Investigator on Days 7, 14, 35, 42, 187 and 194. In addition, solicited AEs will be independently assessed by the investigator Days 14, 42 and 194 and the data directly entered in the CRF.
18. **Serology (ELISA and IFA):** Blood samples will be collected for serology assessment (ELISA and IFA) on Days 0, 28, 56, 180, 208 and 365. On Days 0, 28 and 180, blood samples will be collected prior to vaccination.
19. **Serology (IgG subclasses and GIA):** Blood samples will be collected for serology assessment (IgG subclasses by ELISA) on Days 0, 28, 56, 180, 208 and 365 and for GIA on Days 0, 56, 208 and 365 only. On Days 0, 28 and 180, blood samples will be collected prior to vaccination.

Information contained in this publication is the property of EVI and ICGEB and is confidential and will not be disclosed to third parties without written authorization from EVI and ICGEB. No part of this document may be reproduced, stored in a retrieval system or transmitted in any form or by any means -electronic, mechanical, recording or otherwise - without the prior authorization of EVI and ICGEB

TABLE OF CONTENTS

TITLE PAGE....1
PROTOCOL APPROVAL PAGE....4
INVESTIGATOR'S SIGNATURE PAGE....5
STUDY SYNOPSIS....6
TABLE OF CONTENTS....21
1 LIST OF ABBREVIATIONS....26
2 INTRODUCTION....26
2.1 BURDEN OF MALARIA....26
2.2 MALARIAL VACCINES AND RATIONALE OF THE STUDY....26
2.3 JAIVAC-1 MALARIA VACCINE (PFMSP-1_{19} AND PFF2)....26
2.4 SUMMARY OF PRE-CLINICAL AND CLINICAL STUDIES WITH JAIVAC-1 MALARIA VACCINE....26
3 STUDY OBJECTIVES....26
3.1 PRIMARY OBJECTIVE(S)....26
3.2 SECONDARY OBJECTIVE(S)....26
3.3 EXPLORATORY OBJECTIVE(S)....26
4 STUDY DESIGN....26
4.1 OVERALL STUDY DESIGN....26
4.2 DISCUSSION OF OVERALL STUDY DESIGN....26
4.3 PLANNED DURATION OF STUDY PARTICIPATION/STUDY PERIOD....26
4.4 RANDOMISATION....26
4.5 BLINDING....26
4.6 DEFINITION OF STUDY ENDPOINTS....26
4.6.1 Primary Endpoint(s)....26
4.6.2 Secondary Endpoint(s)....26
4.6.3 Exploratory Endpoint(s)....26
5 STUDY POPULATION....26
5.1 STUDY SITE(S)....26
5.2 PRINCIPAL INVESTIGATOR(S)....26
5.3 NUMBER OF SUBJECTS....26

Information contained in this publication is the property of EVI and ICGEB and is confidential and will not be disclosed to third parties without written authorization from EVI and ICGEB. No part of this document may be reproduced, stored in a retrieval system or transmitted in any form or by any means -electronic, mechanical, recording or otherwise - without the prior authorization of EVI and ICGEB

5.4 Target Population 26
5.4.1 Inclusion Criteria *26*
5.4.2 Exclusion Criteria *26*
5.4.3 Violation of Entry Criteria *26*
5.4.4 Discontinuation Criteria for a Subject during the Study *26*
5.5 Premature Termination of the Clinical Trial at a Specific Site / All Sites 26
5.6 Prohibited Prior/Concomitant Medications or Vaccines 26
6 STUDY VACCINES 26
6.1 Preparation and Administration of Study Vaccines 26
6.1.1 The Investigational Vaccine *26*
6.1.1.1 Composition of the Investigational Vaccine intended for Clinical Use 26
6.1.1.2 Preparation of the Investigational Vaccine 26
6.1.1.3 Administration of the Investigational Vaccine 26
6.1.2 The Control Vaccine *26*
6.1.2.1 Composition of the Control Vaccine 26
6.1.2.2 Preparation of a Single Dose of the Control Vaccine 26
6.1.2.3 Administration of the Control Vaccine 26
6.2 Packaging, Labelling and Storage of Study Vaccines 26
6.3 Clinical Study Supply, Dispensing and Accountability 26
6.4 Method of Assigning Subjects to Vaccination Groups 26
6.4.1 Subject Numbering *26*
6.4.2 Screening Number *26*
6.4.3 Randomisation Number *26*
6.4.4 Adherence to Randomisation List *26*
6.4.5 Emergency Unblinding *26*
7 STUDY CONDUCT, ASSESSMENTS AND VISIT PROCEDURES 26
7.1 General Aspects 26
7.2 Safety Assessments 26
7.2.1 Assessment of Immediate Reactogenicity *26*
7.2.2 Assessment of Local and Systemic Reactogenicity (Solicited AEs) *26*
7.2.2.1 Solicited AEs by Subject Diary 26
7.2.2.2 Solicited AEs by Investigator's Assessment 26
7.2.2.3 Assessment of Severity of Solicited Adverse Events 26
7.2.3 Assessment of Unsolicited Adverse Events *26*
7.2.4 Assessment of Any Serious Adverse Event (SAE) *26*
7.2.5 Assessment of Severity for all Unsolicited Adverse Events (including any Serious Adverse Events) *26*

Information contained in this publication is the property of EVI and ICGEB and is confidential and will not be disclosed to third parties without written authorization from EVI and ICGEB. No part of this document may be reproduced, stored in a retrieval system or transmitted in any form or by any means -electronic, mechanical, recording or otherwise - without the prior authorization of EVI and ICGEB

7.2.6 Other Clinical Safety Assessments *26*
7.2.7 Laboratory Safety Assessments *26*
7.3 IMMUNOGENICITY ASSESSMENTS 26
7.3.1 Anti-PfMSP-1$_{19}$ and Anti-PfF2 IgG antibodies (ELISA) *26*
7.3.2 Anti-PfMSP-1$_{19}$ and Anti-PfF2 IgG antibodies (IFA) *26*
7.3.3 Exploratory Efficacy End-points *26*
7.4 VISIT SCHEDULE AND PROCEDURES 26
7.4.1 Visit Schedule *26*
7.4.2 Overview of Study Procedures and Assessments *26*
7.4.3 Visit-wise Study Procedures and Assessments *26*
7.4.3.1 Visit 1 (Day -21 to Day -15), Pre-Screening Visit 26
7.4.3.2 Visit 2 (Day -14 to Day -1), Screening Visit 26
7.4.3.3 Visit 3 (Day 0, First Dose of the Vaccine) 26
7.4.3.4 Visit 4 (Day 7) 26
7.4.3.5 Visit 5 (Day 14) 26
7.4.3.6 Visit 6 (Day 28, Second Dose of the Vaccine) 26
7.4.3.7 Visit 7 (Day 35) 26
7.4.3.8 Visit 8 (Day 42) 26
7.4.3.9 Visit 9 (Day 56) 26
7.4.3.10 Visit 10 (Day 180, Third Dose of the Vaccine) 26
7.4.3.11 Visit 11 (Day 187) 26
7.4.3.12 Visit 12 (Day 194) 26
7.4.3.13 Visit 13 (Day 208) 26
7.4.3.14 Visit 14 (Day 365, End of Study Visit) 26

8 SAFETY MONITORING 26

8.1 INTRODUCTION 26
8.2 DATA SAFETY MONITORING BOARD 26
8.3 POST-VACCINATION REACTIONS 26
8.4 ADVERSE EVENTS 26
8.4.1 Definition of an Adverse Event *26*
8.4.2 Reporting of Adverse Events *26*
8.4.3 Documentation of Adverse Events *26*
8.4.4 Assessment of Severity of Adverse Events *26*
8.4.5 Assessment of Causality of Adverse Events *26*
8.4.6 Assessment of Expectedness of Adverse Event *26*
8.4.7 Follow-up of Adverse Events and Assessment of Outcome *26*
8.5 SERIOUS ADVERSE EVENTS 26

Information contained in this publication is the property of EVI and ICGEB and is confidential and will not be disclosed to third parties without written authorization from EVI and ICGEB. No part of this document may be reproduced, stored in a retrieval system or transmitted in any form or by any means -electronic, mechanical, recording or otherwise - without the prior authorization of EVI and ICGEB

8.5.1 Definition of a Serious Adverse Event ... 26
8.5.2 Reporting Serious Adverse Events ... 26
8.5.3 Processing and Regulatory Submission of SAE Reports ... 26
8.5.4 Assessment of Severity, Causality and Expectedness of Adverse Events ... 26
8.5.5 Follow-up of Serious Adverse Events and Assessment of Outcome ... 26
8.6 PREGNANCY REPORTING ... 26

9 QUALITY CONTROL AND QUALITY ASSURANCE ... 26

9.1 INTRODUCTION ... 26
9.2 PRE-STUDY DOCUMENTATION ... 26
9.3 MONITORING PROCEDURES ... 26
9.4 RECORDING OF DATA ... 26
9.5 AUDITS AND INSPECTIONS ... 26
9.6 STUDY AND SITE CLOSURE ... 26
9.7 DATA PROTECTION ... 26

10 DATA MANAGEMENT AND PROCESSING ... 26

10.1 DATA COLLECTION ... 26
10.2 DATA MANAGEMENT PROCEDURES ... 26
10.3 DATA VERIFICATION PROCEDURES ... 26
10.4 CODING ... 26
10.5 PROCEDURES FOR ANALYSIS OF CONSISTENCY AND MEDICAL PLAUSIBILITY ... 26
10.6 DATA TO BE RECORDED DIRECTLY INTO THE CRF ... 26

11 STATISTICAL CONSIDERATIONS AND ANALYSIS PLAN ... 26

11.1 STUDY CONDUCT CONSIDERATIONS ... 26
11.1.1 Sample Size ... 26
11.1.2 Randomisation Procedures ... 26
11.1.3 Blinding Procedures ... 26
11.2 TIMING OF ANALYSIS ... 26
11.3 DEFINITION OF ANALYSIS SETS ... 26
11.3.1 Intention-To-Treat Population (ITT Population) ... 26
11.3.2 Per Protocol Population (PP Population) ... 26
11.4 ASSESSMENT ENDPOINTS ... 26
11.5 ANALYSIS METHODS ... 26
11.5.1 Statistical Model, Hypothesis Testing and Tests ... 26
11.5.1.1 Analysis of Demographics and Other Baseline Characteristics ... 26

Information contained in this publication is the property of EVI and ICGEB and is confidential and will not be disclosed to third parties without written authorization from EVI and ICGEB. No part of this document may be reproduced, stored in a retrieval system or transmitted in any form or by any means -electronic, mechanical, recording or otherwise - without the prior authorization of EVI and ICGEB

11.5.1.2 Analysis of Reactogenicity 26
11.5.1.3 Analysis of Overall Safety 26
11.5.1.4 Analysis of Immunogenicity 26

12 REGULATORY AND ETHICAL REQUIREMENTS 26

12.1 REGULATORY AUTHORITY APPROVAL 26
12.2 INSTITUTIONAL REVIEW BOARDS/ETHICS COMMITTEES 26
12.2.1 Institutional Review Board 26
12.2.2 Independent Ethics Committees 26
12.3 SUBJECT INFORMATION AND INFORMED CONSENT 26
12.4 SUBJECT CONFIDENTIALITY 26
12.5 NOTIFICATION OF PRIMARY CARE PHYSICIAN 26
12.6 RECORD RETENTION 26

13 ADMINISTRATIVE MATTERS 26

13.1 PROTOCOL AMENDMENTS 26
13.2 FINANCING AND INSURANCE 26
13.3 CONFIDENTIALITY AND PUBLICATION 26

14 REFERENCES 26

15 APPENDICES 26

APPENDIX A: WORLD MEDICAL ASSOCIATION DECLARATION OF HELSINKI 26

APPENDIX B: JAIVAC_1_1_09 LABORATORY REFERENCE RANGES 26

APPENDIX B-1: JAIVAC_1_1_09 ACCEPTABLE LIMITS FOR 'OUT OF RANGE' LABORATORY PARAMETER(S) AT SCREENING/BASELINE 26

APPENDIX C: SERIOUS ADVERSE EVENT MANAGEMENT FLOWCHART 26

APPENDIX D: PACKAGE INSERT FOR THE CONTROL VACCINE - GENE VAC-B™ (RECOMBINANT HEPATITIS - B VACCINE, I.P.) 26

Information contained in this publication is the property of EVI and ICGEB and is confidential and will not be disclosed to third parties without written authorization from EVI and ICGEB. No part of this document may be reproduced, stored in a retrieval system or transmitted in any form or by any means -electronic, mechanical, recording or otherwise - without the prior authorization of EVI and ICGEB

1 LIST OF ABBREVIATIONS

AE	Adverse Event
ALT	Alanine Aminotransferase
AST	Aspartate Aminotransferase
BBIL	Bharat Biotech International Limited
b.i.d	bis in die/twice a day
CRF	Case Report Form
CRO	Contract Research Organization
DCGI	Drug Controller General of India
DLC	Differential Leukocyte Count
DLS	DiagnoSearch LifeSciences
DNA	Deoxyribonucleic acid
DSMB	Data Safety Monitoring Board
EBA	Erythrocyte Binding Antigen
EC	Ethics Committee
ECG	Electrocardiogram
ELISA	Enzyme Linked Immunosorbent Assay
EVI	European Vaccine Initiative
FDA	Food and Drug Administration
γGT	Gamma Glutamyl Transpeptidase

Information contained in this publication is the property of EVI and ICGEB and is confidential and will not be disclosed to third parties without written authorization from EVI and ICGEB. No part of this document may be reproduced, stored in a retrieval system or transmitted in any form or by any means -electronic, mechanical, recording or otherwise - without the prior authorization of EVI and ICGEB

GCP	Good Clinical Practice
GIA	Growth Inhibition Assay
GMP	Good Manufacturing Practice
HBV	Hepatitis B Virus
HCV	Hepatitis C Virus
HIV	Human Immunodeficiency Virus
ICGEB	International Centre for Genetic Engineering and Biotechnology
ICH	International Conference on Harmonization
IEC	Independent Ethics Committee
IFA	Immunofluorescence Assay
IgG	Immunoglobulin G
IP	Investigational Product
IRB	Institutional Review Board
ITT	Intention To Treat
IUD	Intrauterine Device
MCH	Mean Corpuscular Haemoglobin
MCHC	Mean Corpuscular Haemoglobin Concentration
MCV	Mean Corpuscular Volume
MedDRA	Medical Dictionary for Regulatory Activities
NCR	No Carbon Required
o.d.	omnia die/Once a Day

Information contained in this publication is the property of EVI and ICGEB and is confidential and will not be disclosed to third parties without written authorization from EVI and ICGEB. No part of this document may be reproduced, stored in a retrieval system or transmitted in any form or by any means -electronic, mechanical, recording or otherwise - without the prior authorization of EVI and ICGEB

PCV	Packed Cell Volume
p.o.	per os/By mouth/Orally
PfF2	Region F2 of *Plasmodium falciparum* EBA175 Protein expressed heterologously in *E.coli*
PfMSP-1_{19}	C-terminal 19-kD domain of *Plasmodium falciparum* Merozoite Surface protein-1 expressed heterologously in *E.coli*
PPP	Per Protocol Population
PRF	Pregnancy Report Form
RBC	Red Blood Cell
SAE	Serious Adverse Event
SIIL	Serum Institute of India Limited
SmPC	Summary of Product Characteristics
SOP	Standard Operating Procedures
SUSAR	Suspected Unexpected Serious Adverse Reaction
THC	Tetrahydrocannabinol
TLC	Total Leukocyte Count
ULN	Upper Limit of Normal
UN	United Nation
WHO DD	World Health Organization Drug Dictionary

Information contained in this publication is the property of EVI and ICGEB and is confidential and will not be disclosed to third parties without written authorization from EVI and ICGEB. No part of this document may be reproduced, stored in a retrieval system or transmitted in any form or by any means -electronic, mechanical, recording or otherwise - without the prior authorization of EVI and ICGEB

2 INTRODUCTION

2.1 Burden of Malaria

Malaria is an acute infectious disease, which is caused by plasmodium parasites that are transmitted by female anopheline mosquitoes. Malaria remains a major public health problem in many regions of the tropical world. Forty- percent of the world's population live in malaria endemic regions of Africa, Asia, the Middle East and Central and South America. As per the World Health Organisation's World Malaria Report 2008, 247 million people were infected with malaria in 2006, and 881,000 died of the disease in the same year **(1).**

Around 60% of the cases of clinical malaria and over 80% of the deaths occur in sub-Saharan Africa. Of the one million Africans who die from malaria each year, most are children under five years of age. In addition to acute disease episodes and deaths, malaria also contributes significantly to anaemia in children and pregnant women, adverse birth outcomes such as spontaneous abortion, stillbirth, premature delivery and low birth weight and overall child mortality. The disease is estimated to be responsible for an estimated average annual reduction of 1.3% in economic growth for those countries with the highest burden. Of the four species of *Plasmodium* that infect humans; *P. falciparum, P. vivax, P. malariae and P. ovale; P. falciparum* causes the most severe disease and is responsible for majority of deaths attributable to malaria. *P. falciparum*is most prevalent in sub-Saharan Africa, and in certain areas of South-East Asia and the Western Pacific.

All the clinical symptoms of malaria including severe complications like anaemia, cerebral malaria, placental malaria and renal failure are associated with the blood-stage of *P. falciparum* infection. Unfortunately, there is an increasing level of resistance of the malaria parasite, particularly *P. falciparum*, to several of the known anti-malarial drugs. Given the rapid spread of chloroquine resistance, there is an urgent need to develop vaccines that provide protection against *P. falciparum* malaria **(2)-4).**

2.2 Malarial Vaccines and Rationale of the Study

An ideal malaria vaccine is one that would prevent infection at the first instance and if this is not possible, should at least decrease the intensity of infection and prevent disease. The complex life cycle of the parasite involving human and vector mosquitoes as well as immune evasion mechanisms such as allelic diversity and antigenic variation makes the development of vaccines for malaria difficult. Parasite proteins that play essential roles in parasite survival and contain conserved epitopes with limited polymorphism are likely to be good candidates for malaria vaccine development. Identification of such candidates from different stages of the malaria parasite life cycle may allow the development of multi-stage, multi-component vaccines that target different stages of the parasite life cycle **(5)-6).**

All the clinical symptoms of malaria are attributed to the blood-stage of the parasite's life cycle. In the blood-stage, *P. falciparum* invades and multiplies within host erythrocytes. Each cycle of blood-stage development involves red cell invasion, parasite multiplication, red cell rupture, egress of next generation merozoites and re-invasion of red cells. Erythrocyte invasion requires specific receptor-ligand interactions. Parasite proteins that bind erythrocyte receptors to mediate invasion are attractive candidates for malaria vaccine development since antibodies directed against such

Information contained in this publication is the property of EVI and ICGEB and is confidential and will not be disclosed to third parties without written authorization from EVI and ICGEB. No part of this document may be reproduced, stored in a retrieval system or transmitted in any form or by any means -electronic, mechanical, recording or otherwise - without the prior authorization of EVI and ICGEB

parasite ligands may block erythrocyte invasion, limit parasite multiplication and provide protection against malaria **(7)**.

2.3 JAIVAC-1 Malaria Vaccine (PfMSP-1_{19} and PfF2)

The candidate vaccine, JAIVAC-1 (PfMSP-1_{19} and PfF2) is a multi-antigen vaccine intended to be used for *P. falciparum* malaria. The active ingredients in the vaccine are PfMSP-1_{19} and PfF2. Recombinant PfMSP-1_{19} is a 19 kD protein representing C-terminal, cysteine rich region of PfMSP-1 (PfMSP-1_{19}). PfMSP-1 is the most abundant protein on the surface of invasive blood stage merozoites. PfMSP-1_{19} is retained on the surface of merozoites during invasion while rest of PfMSP-1 is proteolytically cleaved and shed. Recombinant PfF2 is a protein of 42 kD representing domain F2 (PfF2) of the 175 kD *P. falciparum* erythrocyte binding antigen (EBA-175), which binds sialic acid residues on glycophorin A as receptors to mediate erythrocyte invasion. The amino-terminal, conserved, cysteine-rich region of EBA-175, referred to as PfF2, contains receptor-binding sites for glycophorin A **(2), 3), 7)**. EVI (European Vaccine Initiative) in partnership with ICGEB (International Centre for Genetic Engineering and Biotechnology, New Delhi,) has undertaken development and GMP production of the multi-antigen malaria vaccine, JAIVAC-1 (*P.falciparum* PfF2 and PfMSP-1_{19}) formulated with Montanide ISA 720 (adjuvant). The production of the JAIVAC-1 vaccine has been undertaken by Bharat Biotech International Limited (BBIL), Hyderabad, India, while the adjuvant Montanide ISA 720 will be manufactured by Seppic Inc., France. Production of three consistency batches of the vaccine was completed in December 2007 and pre-clinical toxicology studies have been completed. A seroprevalence study was also undertaken in December 2008 to determine prevalence rates for antibodies against PfF2 and PfMSP-1_{19} in residents of Mumbai and Ahmedabad. No clinical trials for the evaluation of the safety and immunogenicity of the JAIVAC-1 vaccine have been initiated or completed till date.

2.4 Summary of Pre-clinical and Clinical Studies with JAIVAC-1 Malaria Vaccine

The candidate vaccine, JAIVAC-1 is a multi-antigen vaccine for P. falciparum malaria composed of two recombinant antigens; PfMSP-1_{19} and PfF2, which is formulated with the adjuvant Montanide ISA 720 prior to administration. The vaccine, developed by EVI and ICGEB and manufactured by BBIL is supplied in single-dose vial of lyophilised product (vaccine), along with vials of adjuvant Montanide ISA 720 manufactured by Seppic Inc, France.

The final solution for intramuscular injection is to be prepared by mixing 2 parts of Montanide ISA 720 with 1 part of lyophilised JAIVAC-1 antigens reconstituted in water (vol/vol). The vaccine is to be emulsified using a 2 ml latex free syringe and a 22 G needle by mixing up and down twenty five times. One up and down = 1 stroke and 25 such strokes are to be performed with the plunger to obtain the desired emulsion. After 25 strokes the emulsion is ready for use **(8)**. The final ready-to-use emulsion is to be administered by intramuscular route on Days 0, 28 and 180 (as planned for the proposed Phase I clinical trial) with follow-up duration of one year after the first administration of the vaccine.

Preclinical Experience with JAIVAC-1

JAIVAC-1 formulated with Montanide ISA 720 was tested in acute toxicity studies in Swiss Albino mice and Sprague Dawley rats. No observable toxicity at and up to the dose of 50 µl /animal (280

Information contained in this publication is the property of EVI and ICGEB and is confidential and will not be disclosed to third parties without written authorization from EVI and ICGEB. No part of this document may be reproduced, stored in a retrieval system or transmitted in any form or by any means -electronic, mechanical, recording or otherwise - without the prior authorization of EVI and ICGEB

x human dose) was observed following single intramuscular administration in mice, except mild local reaction at the site of injection that subsided after three days of immunisation. In rats, doses upto 100 µl (10 µg each antigen) per animal of JAIVAC-1—Montanide ISA 720 (80x human dose) did not cause any observable toxicity, except mild local reaction at the site of injection that subsided after three to six days of immunisation. Two repeated dose 71 Day intramuscular toxicity studies of JAIVAC-1—Montanide ISA 720 were performed in rat and rabbits. Findings from these studies revealed that JAIVAC-1—Montanide ISA 720 did not cause any observable toxicity at and up to the dose of 0.5 ml/animal, except local inflammatory reaction at the site of injection.

Seroprevalence Studies

A study to determine prevalence rates of antibodies against *P. falciparum* antigens PfF2 and PfMSP-1_{19} was conducted in residents of Ahmedabad and Mumbai. Hundred individuals from both sites were tested for the presence of serum antibodies against PfF2 and PfMSP-1_{19}. In Ahmedabad, 28% were seropositive for PfF2 and 10% were seropositive for PfMSP-1_{19}. Three percent were seropositive for both the antigens. Sixty five out of 100 were sero-negative for both antigens. In Mumbai, 19% were seropositive for PfF2 and 15% seropositive for PfMSP-1_{19}. Five percent were seropositive for both antigens. Seventy one out of 100 were sero-negative for both antigens **(9)**.

Clinical Experience with JAIVAC-1 – Montanide ISA 720

No clinical trials for the evaluation of the safety and immunogenicity of the JAIVAC-1 – Montanide ISA 720 vaccine have been initiated or completed till date. The clinical development plan for JAIVAC-1—Montanide ISA 720 malaria vaccine includes a Phase I study in male healthy volunteers in India and Phase II and III studies to assess efficacy, safety and immunogenicity in the target population living in malaria endemic regions of India and Africa. The first clinical study is a Phase I randomised, controlled, dose escalating, single-blind clinical trial for the assessment of the safety and immunogenicity of JAIVAC-1 formulated in Montanide ISA 720 in healthy Indian male subjects between 18 to 45 years of age; proposed study period being February 2010 to April 2011.

Information contained in this publication is the property of EVI and ICGEB and is confidential and will not be disclosed to third parties without written authorization from EVI and ICGEB. No part of this document may be reproduced, stored in a retrieval system or transmitted in any form or by any means -electronic, mechanical, recording or otherwise - without the prior authorization of EVI and ICGEB

3 STUDY OBJECTIVES

3.1 Primary Objective(s)

- To evaluate the safety of three (3) different dosages of JAIVAC-1 malaria vaccine (PfMSP-1_{19} and PfF2) formulated with Montanide ISA720 as adjuvant in healthy Indian male subjects between 18 to 45 years of age
- To compare the safety of three (3) different dosages of JAIVAC-1 vaccine (PfMSP-1_{19} and PfF2) formulated with Montanide ISA720 as adjuvant to the safety of the control vaccine (Hepatitis B vaccine) in healthy Indian male subjects between 18 to 45 years of age

3.2 Secondary Objective(s)

- To assess the humoral response to the vaccine antigens PfMSP-1_{19} and PfF2 by measuring the IgG antibody response by ELISA in healthy Indian male subjects between 18 to 45 years of age
- To assess the humoral response to the vaccine antigens PfMSP-1_{19} and PfF2 by measuring the IgG antibody response by IFA in healthy Indian male subjects between 18 to 45 years of age

3.3 Exploratory Objective(s)

- To assess the quality of the humoral immune response (IgG antibody) to the vaccine antigens in healthy Indian male subjects by measuring the following:
    - Subclasses of IgG antibodies - IgG1, IgG2, IgG3, IgG4 by ELISA
    - The ability of the IgG antibodies to block homologous and heterologous parasite growth in vitro by a *P. falciparum* blood-stage GIA against three parasite strains

Information contained in this publication is the property of EVI and ICGEB and is confidential and will not be disclosed to third parties without written authorization from EVI and ICGEB. No part of this document may be reproduced, stored in a retrieval system or transmitted in any form or by any means -electronic, mechanical, recording or otherwise - without the prior authorization of EVI and ICGEB

4 STUDY DESIGN

4.1 Overall Study Design

JAIVAC-1_1_09
STUDY DESIGN

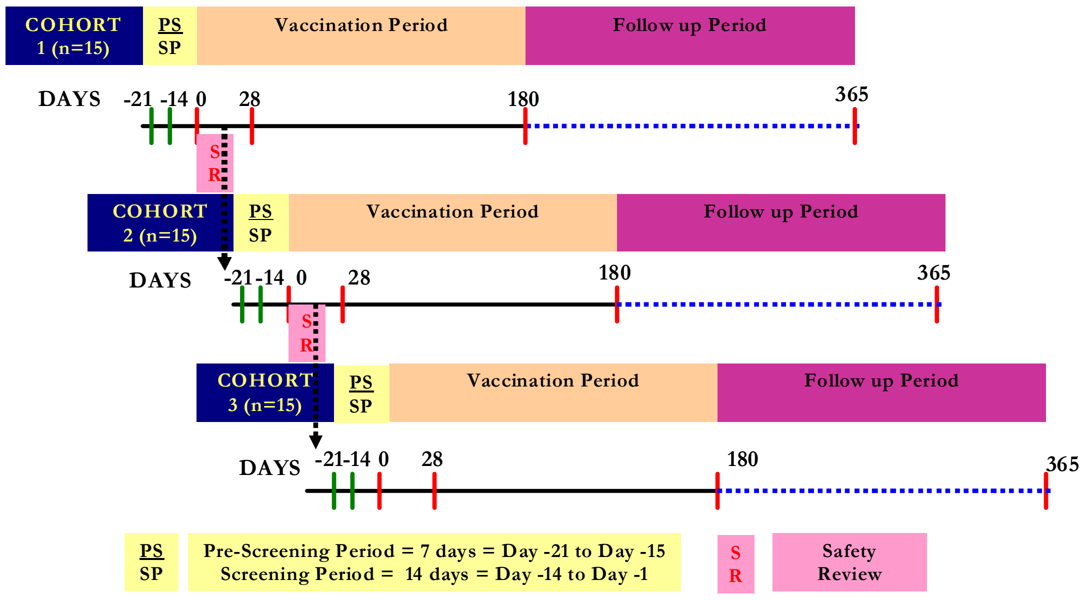

FOOTNOTES:

The defined study period for the JAIVAC-1_1_09 study consists of four (4) distinct study periods; a pre-screening period of seven (7) days, a screening period of 14 days, a vaccination period of 180 days and a follow-up period till 365 days from the first dose of the assigned study vaccine. Each cohort will consist of 15 subjects. Dosing of the JAIVAC-1 – Montanide ISA 720 investigational vaccine arms will begin with Cohort 1 (10µg PfMSP-1$_{19}$ + 10µg PfF2 i.e. Dosage A), followed by Cohort 2 (25µg PfMSP-1$_{19}$ + 25µg PfF2 i.e. Dosage B) and finally Cohort 3 (50µg PfMSP-1$_{19}$ + 50µg PfF2 i.e. Dosage C) as per the common vaccine administration schedule (administered as a 3-dose schedule on Days 0, 28 and 180 by intramuscular route) at each dosage. Each cohort will be staggered into sub-cohorts such that the subjects in each cohort will be enrolled over a three day period. The first three (3) subjects of the first dose (Cohort 1) will be kept under observation for 24 hrs following vaccination. The decision of the 24-hour housing for the remaining subjects will be jointly taken by the Principal Investigator and Medical and Safety Monitor, DLS.

Dose escalation to the next higher dosage cohort of JAIVAC-1 – Montanide ISA 720 will occur after review of the appropriately monitored and verified safety data obtained from the first dose of the vaccine through Day 14 for all 15 subjects in the previous dosage cohort. The "Safety Review" will be undertaken by the DSMB comprising of three (3) independent clinicians with relevant expertise along with the designated Local Safety Monitor (who would be a designated personnel from the investigational site but independent of the study team).The decision to begin the next cohort will be taken in the next 7 days. Thus, the next dosage cohort may begin at the earliest at 21 days after the 15th subject enrolled in the given dosage cohort has been administered the first dose of the JAIVAC-1 – Montanide ISA 720.

Information contained in this publication is the property of EVI and ICGEB and is confidential and will not be disclosed to third parties without written authorization from EVI and ICGEB. No part of this document may be reproduced, stored in a retrieval system or transmitted in any form or by any means -electronic, mechanical, recording or otherwise - without the prior authorization of EVI and ICGEB

4.2 Discussion of Overall Study Design

The present study is designed as a randomised, controlled single-blind and dose-escalating clinical study for the assessment of the safety and immunogenicity of JAIVAC-1 – Montanide ISA 720 in healthy human Indian male subjects between 18 to 45 years of age. This is a dose-escalating study and involves testing of three (3) different dosages of JAIVAC-1 malaria vaccine (PfMSP-1_{19} and PfF2) formulated with Montanide ISA 720 as adjuvant with the intramuscular administration of the investigational vaccine as a 3-dose schedule on Days 0, 28 and 180.

30 subjects will receive JAIVAC-1 – Montanide ISA 720(10 subjects in each cohort) and 15 subjects will receive control vaccine, Hepatitis B vaccine (5 subjects in each cohort). There will be three (3) cohorts in this study such that each cohort will consist of 15 eligible subjects randomised in a ratio of 2:1 as follows:

Cohort 1

**Test Arm 1**	10 subjects shall receive dosage A of investigational vaccine (0.1 ml of JAIVAC-1 vaccine containing 10μg PfMSP-1_{19} and 10μg PfF2 formulated with adjuvant Montanide ISA 720) by intramuscular route on Days 0, 28 and 180
**Control Arm 1**	5 subjects shall receive control vaccine (1 ml of Hepatitis B vaccine) by intramuscular route on Days 0, 28 and 180

Cohort 2

**Test Arm 2**	10 subjects shall receive dosage B of investigational vaccine (0.25 ml of JAIVAC-1 vaccine containing 25μg PfMSP-1_{19} and 25μg PfF2 formulated with adjuvant Montanide ISA 720) by intramuscular route on Days 0, 28 and 180
**Control Arm 2**	5 subjects shall receive control vaccine (1 ml of Hepatitis B vaccine) by intramuscular route on Days 0, 28 and 180

Cohort 3

**Test Arm 3**	10 subjects shall receive dosage C of investigational vaccine (0.5 ml of JAIVAC-1 vaccine containing 50μg PfMSP-1_{19} and 50μg PfF2 formulated with adjuvant Montanide ISA 720) by intramuscular route on Days 0, 28 and 180
**Control Arm 3**	5 subjects shall receive control vaccine (1 ml of Hepatitis B vaccine) by intramuscular route on Days 0, 28 and 180

Dosing of the JAIVAC-1 – Montanide ISA 720 investigational vaccine arms will begin with Cohort 1 (i.e. 0.1 ml of investigational vaccine administered by intramuscular route as a 3-dose schedule, followed by Cohort 2 (i.e. 0.25 ml of investigational vaccine) and finally Cohort 3 (i.e. 0.5 ml of investigational vaccine). Each cohort will be staggered into sub-cohorts such that the subjects in each cohort will be enrolled over a three-day period. The first three (3) subjects of the first dose (Cohort 1) will be kept under observation for 24 hrs following vaccination. The decision of the 24-hour housing for the remaining subjects will be jointly taken by the Principal Investigator and Medical and Safety Monitor, DLS.

Information contained in this publication is the property of EVI and ICGEB and is confidential and will not be disclosed to third parties without written authorization from EVI and ICGEB. No part of this document may be reproduced, stored in a retrieval system or transmitted in any form or by any means -electronic, mechanical, recording or otherwise - without the prior authorization of EVI and ICGEB

Dose escalation to a higher dosage of JAIVAC-1 – Montanide ISA 720 will occur after review of safety data by the DSMB through Day 14 for all subjects in a cohort. Thus, in each cohort, each subject will receive a total of three (3) doses of either of the study or control vaccines by intramuscular route, one each on Day 0, Day 28 and Day 180 respectively.

4.3 Planned Duration of Study Participation/Study Period

The total duration of each subject's participation (excluding allowed window periods) will be approximately 386 which include:

- Pre-screening period of 7 days
- Screening period of 14 days
- Vaccination period of 180 days (refer window period by visit)
- Follow-up period till 365 days (1 year) after the first vaccination (window period of +/- 7 days)

The approximately, total study duration (476 days) includes enrolment period (90 days) and duration of participation of individual subjects (386 days).

4.4 Randomisation

A total of 45 healthy subjects in the age group of 18 to 45 years will be enrolled in the study. There will be three (3) cohorts in this study such that each cohort will consist of 15 eligible subjects randomised in a ratio of 2:1 to receive the particular dosage of investigational vaccine i.e. JAIVAC-1 – Montanide ISA 720 (10 subjects) and the control vaccine i.e. Hepatitis B vaccine (5 subjects) respectively, both administered as a 3-dose schedule. Thus, overall 30 subjects will receive JAIVAC-1 – Montanide ISA 720 (10 subjects for each cohort) and 15 subjects will receive control vaccine, Hepatitis B vaccine (5 subjects for each cohort).

Subjects will be randomised to study cohort using a reproducible, computer-generated permuted block randomisation schedule which will be cohort specific to ensure allocation ratio 2: 1 between investigational and control vaccine. Randomisation list will be generated and sent to DLS pharmacist for labelling and packaging purpose.

The randomisation number will be a three-digit subject number and will be cohort specific. The first digit identifies cohort as 1 (Cohort 1), 2 (Cohort 2) or 3 (Cohort 3), and the next two digits identify the subject within cohort and will be assigned sequentially as per the enrolment. Thus, study subjects in Cohort 1 will get randomisation numbers like 101, 102 and so on sequentially, study subjects in Cohort 2 will get randomisation numbers like 201, 202 and so on sequentially and study subjects in Cohort 3 will get randomisation numbers like 301, 302 and so on sequentially.

4.5 Blinding

This clinical trial is designed as a single-blind study. Single blind means that the subjects taking part in the study will not know whether they have been assigned to the investigational vaccine arm or the control. The Investigators, investigator staff, persons performing the assessments, and data analysts will remain unblinded to the identity of the vaccine.

Information contained in this publication is the property of EVI and ICGEB and is confidential and will not be disclosed to third parties without written authorization from EVI and ICGEB. No part of this document may be reproduced, stored in a retrieval system or transmitted in any form or by any means -electronic, mechanical, recording or otherwise - without the prior authorization of EVI and ICGEB

4.6 Definition of Study Endpoints

4.6.1 Primary Endpoint(s)

The safety profile will be assessed on the basis of the following criteria

- Immediate reactogenicity (any event occurring within the first three (3) hours recorded every 30 minutes after each vaccination, with emphasis on allergic reactions)
- Local and systemic reactogenicity (solicited adverse events occurring from three (3) hours post-vaccination on Day 0 till Day 14 after each dose)
- Any unsolicited adverse events 28 days after each vaccination
- Any Serious Adverse Event (SAE) occurring from the first dose of vaccine till the last follow-up visit.
- Biological safety, 28 days after each vaccination, in reference with the baseline before the first dose, by measuring the following parameters:
    - Haematology: RBC count, Haemoglobin*, Haematocrit/Packed Cell Volume (PCV), MCV (Mean Corpuscular Volume), MCH (Mean Corpuscular Haemoglobin), MCHC (Mean Corpuscular Haemoglobin Concentration), Platelet Count and Total Leukocyte Count (TLC) along with Differential Leukocyte Count (DLC) on Days -14, 28, 56, 180, 208, 365.

      *If the haemoglobin drops below 13 gm/dL, then a direct Coomb's test and a peripheral blood smear/film will be prepared and examined for evidence of possible haemolysis.
    - Serum Chemistry: Potassium, Sodium, AST, ALT, Direct, Indirect and Total Bilirubin, Alkaline Phosphatase, Gamma Glutamyl Transpeptidase (γGT), Creatinine, and Random blood glucose on Days -14, 28, 56, 180, 208, 365.

The Investigator will be responsible for causality assessment i.e. assessment of the relationship of the AE to the assigned study vaccines, using the following definitions: related or not related.

4.6.2 Secondary Endpoint(s)

- The humoral response to the candidate vaccine antigen will be assessed (quantitative assessment) by measuring the level of IgG antibodies developed against PfMSP-1_{19} and PfF2 by ELISA on Days 0, 28, 56, 180, 208 and 365
- The humoral response to the candidate vaccine antigen will be assessed (qualitative assessment) to verify the ability of the IgG antibodies developed against PfMSP-1_{19} and PfF2 to recognise the native proteins, namely, PfMSP1 and EBA175 in late stage *P. falciparum* schizonts and merozoites by IFA on Days 0, 28, 56, 180, 208 and 365.

4.6.3 Exploratory Endpoint(s)

- The quality of the humoral immune response will be additionally assessed by measuring the following:

Information contained in this publication is the property of EVI and ICGEB and is confidential and will not be disclosed to third parties without written authorization from EVI and ICGEB. No part of this document may be reproduced, stored in a retrieval system or transmitted in any form or by any means -electronic, mechanical, recording or otherwise - without the prior authorization of EVI and ICGEB

- IgG1, IgG2, IgG3, IgG4 subclasses by ELISA on samples obtained at Days 0, 28, 56, 180, 208 and 365.
- The ability of the IgG antibodies to block homologous and heterologous parasite growth *in vitro* by a *P. falciparum* blood-stage growth inhibition assay (GIA) against three parasite strains on samples obtained at Days 0, 56, 208 and 365

Information contained in this publication is the property of EVI and ICGEB and is confidential and will not be disclosed to third parties without written authorization from EVI and ICGEB. No part of this document may be reproduced, stored in a retrieval system or transmitted in any form or by any means -electronic, mechanical, recording or otherwise - without the prior authorization of EVI and ICGEB

5 STUDY POPULATION

5.1 Study Site(s)

Lotus Labs Pvt Ltd

100 ft. Road, 3rd Block, Koramangala

Bangalore 560034, India

5.2 Principal Investigator(s)

Dr. Preethi Shivyogi

Lotus Labs Pvt Ltd

100 ft. Road, 3rd Block, Koramangala

Bangalore 560034, India

5.3 Number of Subjects

- A sufficient number of subjects will be screened to enrol a total number of 45 subjects in the study.
- 30 subjects will receive the investigational vaccine (i.e. JAIVAC-1—Montanide ISA 720) with 10 subjects in each cohort and
- 15 subjects will receive the control vaccine (i.e. Hepatitis B vaccine) with 5 subjects in each cohort.

Subjects who are discontinued from the study prior to the first vaccination will be replaced. However, subjects who are discontinued from the study after the first vaccination will be considered as drop-outs and will not be subsequently replaced.

5.4 Target Population

The study will be performed in healthy male Indian volunteers aged 18 to 45 years (both inclusive). The Investigator must ensure that all subjects who meet the inclusion and exclusion criteria during screening period (D-14 through D-1) and / at baseline (Day 0) (as applicable) are offered enrolment in the study. No additional exclusions can be applied by the Investigator, in order that the study population will be representative of all eligible subjects.

5.4.1 Inclusion Criteria

To be eligible to participate in the study, the subjects should fulfil all of the following inclusion criteria at the time of the Screening/Baseline Visit:

1. Male subject aged 18 to 45 years (both inclusive)
2. Subject with general good health based on the medical history and clinical examination

Information contained in this publication is the property of EVI and ICGEB and is confidential and will not be disclosed to third parties without written authorization from EVI and ICGEB. No part of this document may be reproduced, stored in a retrieval system or transmitted in any form or by any means -electronic, mechanical, recording or otherwise - without the prior authorization of EVI and ICGEB

3. Subject must be willing to sign the Informed Consent Form
4. Subject must be reachable by phone during the entire study period (12 months)
5. Subject must be capable and willing to complete and return diary cards and to attend all follow-up visits
6. Male subject must agree to use one of the following medically-acceptable birth control measures throughout the duration of the study (birth control counselling and measures will be provided by sites as required)

   Double barrier method (e.g. condom with spermicidal jelly) used for the entire study period

   Or

   Should be Surgically sterile (vasectomy)

5.4.2 Exclusion Criteria

The subjects will be excluded from study entry, if any one or more of the following criteria exist at the time of the Screening/Baseline Visit:

1. Subject with evidence of IgG antibodies against PfMSP-1_{19} and PfF2 as measured by ELISA
2. Subject with prior history of immunisation with Hepatitis B vaccine
3. Subject with known history of malaria
4. Subject with history of allergic reactions, hypersensitivity or anaphylaxis to any of the components of the study vaccines (JAIVAC-1 – Montanide ISA 720 malaria vaccine or Hepatitis B vaccine) (including adjuvant or peptide) or with history of serious allergic reactions to any substance, requiring hospitalisation or emergency medical care
5. Subject with previous vaccination with any other malaria candidate vaccines
6. Subject with use of an investigational or non-registered drug or vaccine other than the study vaccines within three (3) months preceding the first study vaccination, or planned use during the entire clinical trial period
7. Subject, who receives any vaccination or gamma globulin during the three-month period prior to the first vaccination
8. Subject with chronic administration (defined as more than 14 days) of immuno-suppressants or other immune-modifying drugs within six months prior to the first vaccination. This includes any dose level of oral steroids or inhaled steroids, but not topical steroids
9. Subjects will be excluded if AST > 40 IU/L, ALT > 41 IU/L, γ GT > 71 IU/L, Total Bilirubin > 1.2 mg/ dL, Indirect Bilirubin > 1.2 mg/ dL, Direct Bilirubin > 0.4 mg/dL, Serum Creatinine > 1.2 mg/ dL (Appendix B and B-1).
10. Subjects will be excluded in case of out of range values for the following parameters: Hemoglobin 13 to 18 g/ dL, RBC count 4.0 to 7.0 × 10E6/µL TLC 4.0 to 11.0 ×

Information contained in this publication is the property of EVI and ICGEB and is confidential and will not be disclosed to third parties without written authorization from EVI and ICGEB. No part of this document may be reproduced, stored in a retrieval system or transmitted in any form or by any means -electronic, mechanical, recording or otherwise - without the prior authorization of EVI and ICGEB

10E3/μL, platelet count 150 to 500 × 10E3/μL, Neutrophils 40 to 75 % or Eosinophils > 10 %, Sodium 136 to 145 mEq/L, Potassium 3.5 to 5.1 mEq/L, Random Blood Glucose 45 to 130 mg/dl and Alkaline Phosphatase 40 to 129 U/L (Appendix B and B-1).

11. Subjects with other clinically significant abnormal laboratory values based on the normal reference range (Refer Appendix B and B1) apart from the laboratory parameters listed above.
12. Subject with positive HIV, HBV or HCV test
13. Subject with positive direct Coomb's test
14. Subject with a peripheral blood film/smear with features of haemolysis or any other haematological disorder
15. Subject unable to give written informed consent
16. Subject unable to be closely followed for social, geographic or psychological reasons
17. Subject with previous history of drug or alcohol abuse interfering with normal social function during a period of one year prior to enrolment in the study or positive urine drug test
18. Subject with an abnormal 12-lead ECG at screening associated with relevant clinical symptoms/signs suggestive of cardiac pathology (including conduction disturbances)
19. Subject with an abnormal Chest X-Ray associated with relevant clinical symptoms/signs of respiratory pathology at screening/ anytime in the past 6 months
20. Subject with known history of congenital/hereditary immunodeficiency disorders
21. Subject with acute or chronic pulmonary, reticulo-endothelial, cardiovascular, hepatic, renal or neurological condition, or any other clinical findings that in the opinion of the clinical investigator, may increase the risk of participating in the study
22. Subject with any other condition that in the opinion of the clinical investigator would jeopardise the safety or rights of a participant in the trial or would render the participant unable to comply with the protocol

5.4.3 Violation of Entry Criteria

Adherence to the inclusion and exclusion criteria by the Investigator and subjects is required. Any questions concerning the inclusion or exclusion criteria must be discussed with the Medical and Safety Monitor prior to subject enrolment.

Additional evaluations with respect to safety may be added based upon the judgement of the investigator.

If a subject is enrolled in apparent conformity with the protocol entry criteria by the Investigator but is later found to be wrongly enrolled (e.g. at the time of the monitoring visit), it will immediately be brought to the notice of the designated Medical and Safety Monitor. Further continuation of such wrongly enrolled subjects may be possible, subject to the final decision taken by the designated Medical and Safety Monitor in consultation with the Sponsor (as required).

Information contained in this publication is the property of EVI and ICGEB and is confidential and will not be disclosed to third parties without written authorization from EVI and ICGEB. No part of this document may be reproduced, stored in a retrieval system or transmitted in any form or by any means -electronic, mechanical, recording or otherwise - without the prior authorization of EVI and ICGEB

5.4.4 Discontinuation Criteria for a Subject during the Study

A discontinuation occurs when an enrolled subject ceases participation in the study, regardless of the circumstances, prior to completion of the protocol. Subjects may voluntarily withdraw from the study for any reason at any time or, if necessary, the Investigator or Sponsor may withdraw a subject from the study to protect the health of that subject. Subjects will be considered withdrawn if they state an intention to withdraw, or fail to return for visits, or become lost to follow up for any other reason.

Thus, a study subject may be discontinued from participation in the study if:

- Subject withdraws consent
- Any adverse event (AE), laboratory abnormality, inter-current illness, or other medical condition or situation occurs such that continued participation in the study would not be in the best interest of the subject
- Non-compliance with protocol requirements (e.g. use of restricted medication, urine drug screen positive, not following dosing procedures, failure to make scheduled study visits in a timely fashion)
- Wrongly enrolled subject may be discontinued from the study based on the decision taken by the designated Medical and Safety Monitor in consultation with the Sponsor (as required).
- Any other reason (as applicable) as adjudged by the Principal Investigator

The Investigator must manage the premature discontinuation /withdrawal of a study subject as follows:

- The Investigator shall determine/verify the primary reason for a subject's discontinuation/premature withdrawal from the study and record this information on the CRF.
- For subjects who are lost to follow-up (i.e., those subjects whose status is unclear because they fail to appear for study visits without stating an intention to withdraw), the Investigator should show "due diligence" by documenting in the source documents steps taken to contact the subject, e.g., dates of telephone calls, registered letters, etc.
- Discontinuation must be reported to designated Medical and Safety Monitor and Sponsor Representative without delay if it is due to an adverse event.
- If a subject terminates prematurely from the study early, all efforts will be made to complete D365 procedures for the given subject.

All subjects who receive one dose of the vaccine will be included in the safety analysis.

Subjects who are prematurely withdrawn from the study after receiving the first (1st) vaccination will not be replaced by an equal number of newly enrolled subjects.

5.5 Premature Termination of the Clinical Trial at a Specific Site /All Sites

Premature termination of the clinical trial may occur because of a regulatory authority decision, change in the opinion of the Independent Ethics Committee or vaccine safety problems at the

Information contained in this publication is the property of EVI and ICGEB and is confidential and will not be disclosed to third parties without written authorization from EVI and ICGEB. No part of this document may be reproduced, stored in a retrieval system or transmitted in any form or by any means -electronic, mechanical, recording or otherwise - without the prior authorization of EVI and ICGEB

discretion of EVI/ICGEB. Recruitment at a centre may be stopped for reasons of particularly low enrolment, protocol violations, or inadequate data recording.

The study may end formally at the site for the following reasons:

- The study has been completed (all the subjects have completed their participation in the study)
- Poor enrolment rates
- Poor site performance
- IEC/IRB decision, Investigator /site/ decision etc

The Investigator also must provide follow-up medical care for all subjects who are prematurely withdrawn from the study, or must refer them for appropriate ongoing care (as required).

5.6 Prohibited Prior/Concomitant Medications or Vaccines

Following prior medications/vaccines are prohibited at study entry:

- Use of any malaria candidate vaccines at any time preceding the first study vaccination
- Use of any investigational or non-registered drug or vaccine within three (3) months preceding the first study vaccination and during the entire clinical trial period (including the follow-up period up to six months after the 3rd vaccination)
- Use of any approved vaccination or gamma globulin within three (3) months preceding the first study vaccination and during the entire clinical trial period (including the follow-up period up to six months after the 3rd vaccination)
- Chronic use (defined as use for 14 days or more) of any of the following drugs within six (6) months preceding the first study vaccination and during the entire trial period (including the follow-up period up to six months after the 3rd vaccination)
  - Immunosuppressants
  - Immune-modifying drugs
  - Oral/inhaled/systemic steroids (any dose level); *only topical steroids are permitted.*

Information contained in this publication is the property of EVI and ICGEB and is confidential and will not be disclosed to third parties without written authorization from EVI and ICGEB. No part of this document may be reproduced, stored in a retrieval system or transmitted in any form or by any means -electronic, mechanical, recording or otherwise - without the prior authorization of EVI and ICGEB

6 STUDY VACCINES

Two (2) vaccines will be used in the study, namely the investigational vaccine (JAIVAC-1 – Montanide ISA 720 malaria vaccine) and the control vaccine (Hepatitis B vaccine), both being referred together as the "study vaccines". The vaccine under development, JAIVAC-1 malaria vaccine (PfMSP-1_{19} and PfF2) will be referred to as the investigational vaccine; it has been developed by ICGEB and EVI and manufactured and supplied by Bharat Biotech International Ltd., Hyderabad, India. The quality control standards and requirements for the investigational vaccine as well as the adjuvant are described in separate release protocols and the required approvals for the clinical use of the investigational vaccine as well as the adjuvant will be obtained prior to start of the study.

The control vaccine used in this study is Gene Vac-B™ i.e. Recombinant Hepatitis-B Vaccine, I.P. and will be procured from Serum Institute of India Limited (SIIL), Pune, India and the respective prescribing information is provided in Package Insert provided with the vaccine (*Refer Appendix D*).

6.1 Preparation and Administration of Study Vaccines

Both the investigational and control vaccines will be administered intramuscularly in the non-dominant deltoid for the first and third administration. The second administration will be performed in the dominant deltoid. Since this is a single-blind study, care will be taken that the identity of the assigned vaccine is not disclosed even inadvertently to the subject.

6.1.1 The Investigational Vaccine

One batch of the investigational vaccine (JAIVAC-1) i.e. lot no. BRD/JV/FFL/7003 will be used for the entire study. The shelf-life of the investigational vaccine JAIVAC-1 is under evaluation (Refer Annexure D.1 of the Investigator's Brochure). The clinical batch i.e. lot no. BRD/JV/FFL/7003 to be used in the clinical trial will be released by the Central Research Institute, Kasauli, India.

6.1.1.1 Composition of the Investigational Vaccine intended for Clinical Use

JAIVAC-1 vaccine is composed of a physical mixture of the two (2) antigens PfMSP-1_{19} and PfF2 (62.5 μg each), which is lyophilised for long term storage. JAIVAC-1 vaccine will be formulated with the adjuvant Montanide ISA 720 just prior to administration of the final vaccine. The lyophilised JAIVAC-1 antigens as well as the sterile water and Montanide ISA 720 (used for reconstitution and formulation of the vaccine respectively) should be stored at a temperature of +2 to +8 °C. **The vials should not be frozen.**

6.1.1.2 Preparation of the Investigational Vaccine

A. Reconstitution

The reconstitution of the JAIVAC-1 vaccine will be undertaken under laminar flow hood.

- The mixture of the lyophilised malaria antigens in the vial will be first reconstituted in 0.2 ml of sterile water. 0.425 ml of Montanide ISA 720 will then be added to the reconstituted combination antigens in the same vial.

Information contained in this publication is the property of EVI and ICGEB and is confidential and will not be disclosed to third parties without written authorization from EVI and ICGEB. No part of this document may be reproduced, stored in a retrieval system or transmitted in any form or by any means -electronic, mechanical, recording or otherwise - without the prior authorization of EVI and ICGEB

- The entire contents of the reconstituted and formulated vaccine (0.625 ml) in the vial is to be then drawn into a 2 ml latex free syringe using a 22 G needle and is to be emulsified by mixing up and down twenty five times.
- One up and down = 1 stroke and 25 such strokes are to be performed with the plunger to obtain the desired emulsion.
- After 25 strokes, the emulsion is ready for use **(8)**.
- The ready-to-use emulsion will be used immediately after reconstitution and will be administered by the intramuscular route in the required dose (0.1, 0.25 or 0.5 ml) using an appropriate sized syringe and needle, taking care to expel any air bubbles as mentioned on the vaccine label.
- The reconstituted JAIVAC-1 upon formulation with Montanide ISA720 is stable up to 48 hrs at room temperature (Refer Annex D.2 of Investigator's Brochure). However, it is recommended to use the reconstituted vaccine as soon as possible for vaccination with recording of the time of vaccine reconstitution and volunteer vaccination.

On reconstitution, each 0.5 ml of the JAIVAC-1 – Montanide ISA 720 vaccine intended for clinical use contains:

**Constituents**	**Quantity**
PfMSP-1_{19} (Antigen)	50 μg
PfF2 (Antigen)	50 μg
Sucrose (for lyophilisation)	15 mg
Maltose (for lyophilisation)	25 mg
Citrate-Phosphate (Buffer)	10 mM
Tris-Acetate (Buffer)	4 mM
Sterile Water	0.160 ml
Montanide ISA 720 (Adjuvant)	0.340 ml

B. Precautions

- Please note that after 10 strokes, the formulation becomes very viscous and requires extra force to perform the strokes.
- All precautions should be taken to avoid introducing air bubbles.

6.1.1.3 Administration of the Investigational Vaccine

A. Dosage of the investigational vaccine

0.1 ml (Dosage A), 0.25 ml (Dosage B) or 0.5 ml (Dosage C) of the reconstituted and emulsified JAIVAC-1 – Montanide ISA 720 vaccine (composed of a physical mixture of the two antigens PfMSP-1_{19} and PfF2 (50 μg each/0.5 ml) shall be used in the planned dose-escalating Phase I study in healthy human volunteers.

B. Route of administration

The vaccine will be administered intramuscularly in the non-dominant deltoid for the first and third administrations while the second administration will be performed in the dominant deltoid.

C. Vaccination Schedule

Information contained in this publication is the property of EVI and ICGEB and is confidential and will not be disclosed to third parties without written authorization from EVI and ICGEB. No part of this document may be reproduced, stored in a retrieval system or transmitted in any form or by any means -electronic, mechanical, recording or otherwise - without the prior authorization of EVI and ICGEB

The vaccine will be administered as per a 3-dose vaccination schedule on Days 0, 28 and 180

6.1.2 The Control Vaccine

One batch of the control vaccine (Gene Vac-B™ i.e. Recombinant Hepatitis - B Vaccine, I.P. by SIIL, Pune, India) will be used for the entire study.

6.1.2.1 Composition of the Control Vaccine

Gene Vac-B™ (Recombinant Hepatitis-B Vaccine, I.P.) is a non infectious recombinant DNA Hepatitis B Vaccine. It contains purified surface antigen of the virus obtained by culturing genetically-engineered*Hansenula polymorpha* yeast cells having the surface antigen gene of the Hepatitis B virus. The Hepatitis-B surface antigen (HBsAg) expressed in the cells of *Hansenula polymorpha* is purified through several chemical steps and formulated as a suspension of the antigen adsorbed on aluminium hydroxide and thiomersal is added as preservative. The vaccine does not contain any material of human or animal origin.

Each ml of Gene Vac-B™; produced in *Hansenula Polymorpha* (yeast) contains:

Constituents	Quantity
Purified Hepatitis B surface antigen	20 mcg
Adsorbed on Aluminium hydroxide (Al+++)	≤ 1.25 mg
Preservative: Thiomersal	≤ 0.01%

6.1.2.2 Preparation of a Single Dose of the Control Vaccine

Gene Vac-B™ (Recombinant Hepatitis-B Vaccine, I.P.) is available as a ready-to-use suspension as single and multiple-dose presentations for paediatric and adult use. The single-dose adult vial of 1.0 ml will be used for this study.

A. Precautions and Warnings

Because of the period of latency of hepatitis B infection, it is possible for unrecognised infection to be present at the time of immunisation. The vaccine may not prevent hepatitis B infection in such cases. The vaccine will not prevent infection caused by other agents such as hepatitis A, hepatitis C and hepatitis E and other pathogens known to infect the liver. The immune response to Hepatitis B vaccines is related to age. In general, people over 40 years of age respond less well. In haemodialysis patients and persons with an impaired immune system, adequate anti-HBs antibody titres may not be obtained after the primary immunisation course and such patients may therefore require administration of additional doses of vaccine (*Please refer to Appendix D for dosage recommendations for immunocompromised persons*). As with all injectable vaccines, appropriate medication (e.g. adrenaline) should always be readily available for treatment in case of rare anaphylactic reactions following the administration of the vaccine.

Gene Vac-B™ should not be administered in the gluteal muscle or intradermally since this may result in a lower immune response. Gene Vac-B™ may be used to complete a primary immunisation course started either with plasma-derived or with other genetically-engineered Hepatitis B vaccines, or as a booster dose in subjects who have previously received a primary immunisation course with plasma-derived or with other genetically-engineered hepatitis B vaccines.

B. Adverse Reactions

Information contained in this publication is the property of EVI and ICGEB and is confidential and will not be disclosed to third parties without written authorization from EVI and ICGEB. No part of this document may be reproduced, stored in a retrieval system or transmitted in any form or by any means -electronic, mechanical, recording or otherwise - without the prior authorization of EVI and ICGEB

The undesirable events are temporally related to the administration of Hepatitis B Vaccine. They are usually mild and confined to the first few days of the vaccination. The most common reactions are mild soreness, erythema, induration, fatigue, fever, malaise, influenza-like symptoms. Less common systemic reactions include nausea, vomiting, diarrhoea, abdominal pain, abnormal liver function tests, arthralgia, myalgia, rash, pruritus, urticaria, liver function. *Please refer to Appendix D for additional details of the control vaccine.*

6.1.2.3 Administration of the Control Vaccine

A. Dosage of the Control Vaccine

1 ml of the ready-to-use suspension of the control vaccine (Gene Vac-B™ i.e. Recombinant Hepatitis-B Vaccine, I.P. by SIIL, Pune, India) consisting of 20 mcg dose (recommended for male subjects) shall be used for vaccination.

B. Route of Administration

The vaccine will be administered intramuscularly in the non-dominant deltoid for the first and third administrations while the second administration will be performed in the dominant deltoid.

C. Vaccination Schedule

The vaccine will be administered as per a 3-dose vaccination schedule on Days 0, 28 and 180

6.2 Packaging, Labelling and Storage of Study Vaccines

DLS/designated facility will receive the investigational vaccine from BBIL under controlled conditions and the record of the temperature during transit as well during transient storage will be maintained by DLS/designated facility, as applicable. Similarly, the control vaccine will be received from Serum Institute of India and the temperature records both for in-transit status and during transient storage will be maintained by DLS/designated facility.

Study vaccines (both investigational and control vaccines) will be packed in individual boxes per subject called as "vaccine packs". A total of 45 such boxes will be prepared, and numbered from 1 to 45, according to the randomisation list. In addition, two (2) packs will be prepared (one each for the study and control vaccine) and utilised for site training. These two (2) trial packs will be separately labelled as "FOR TRAINING PURPOSE ONLY, NOT FOR CLINICAL TRIAL USE" and labelled accordingly. Extra supply of study vaccines of 20% will be included as contingency in case of any breakage during preparation.

There will be two kind of vaccine packs: 1) The primary vaccination pack will contain two (2) doses of the assigned vaccine (study/control) requested for the first two vaccination at D0 and D28, 2) The booster pack will contain one (1) dose of the assigned vaccine (study/control) requested for the booster vaccination at D180, along with the required quantity of adjuvant and WFI, as applicable. The syringes and needles of the required specifications for reconstitution and vaccine administration for assigned vaccine will be available at the site and used by the site team as required.

Information contained in this publication is the property of EVI and ICGEB and is confidential and will not be disclosed to third parties without written authorization from EVI and ICGEB. No part of this document may be reproduced, stored in a retrieval system or transmitted in any form or by any means -electronic, mechanical, recording or otherwise - without the prior authorization of EVI and ICGEB

DLS/ designated party will be responsible for appropriate packaging and labelling of the vaccine packs which will consist of the respective study vaccine vials (investigational/control vaccines) and adjuvant vial and WFI for the test vaccine. A data logger will be placed in the box during shipment from DLS to study site. A print-out of the data logger will be retained in the Site Master File at study site. Should there be any deviation in the temperature during transit; the same will be communicated immediately by study site to DLS and Sponsor and accordingly corrective action will be instituted, as applicable.

A two-piece, perforated tear-off, unblinded label will be attached to each of the vaccine vials. Vial labels may contain the following information: Protocol number, Subject's randomisation number, dosage and administration instructions, storage instructions and "For Clinical Study Use Only". The study vaccines must be received by a designated person at the study site, handled and stored in a safe, locked, and secure place with no access to any unauthorised personnel. The Investigator at the study site is responsible for ensuring that all study vaccines are stored in a locked, secure location, with access limited to the Investigator and his/her designee(s).

Upon receipt, all study vaccines will be stored according to the instructions specified on the vaccine labels. They must be kept between +2°C and +8°C and **must not be frozen**. Storage temperature should be monitored twice daily. Access to a back-up refrigerator in case of power failure/breakdown is necessary. Clinical supplies are to be dispensed only in accordance with the protocol. The Investigator must maintain an accurate record of the shipment and dispensing of study vaccines in a vaccine accountability ledger. Monitoring of vaccine accountability will be performed by the study monitor during site visits and at the completion of the clinical trial.

At the end of the study (after the database has been locked) all unused study vaccines may be returned to EVI/ICGEB or designee. Empty or partially used vials of study vaccines may be may be returned to EVI/ICGEB or designee.

6.3 Clinical Study Supply, Dispensing and Accountability

All study vaccines will be provided by DLS/designated party to the study site. The Investigator will acknowledge receipt of the study vaccines indicating shipment content and condition. All study vaccines used in the study will be accounted for in the "Study Vaccines Accountability Record" provided by DLS/available at study site", which indicates the date of administration to the subject.

Temperature (between +2°C and +8°C) will be monitored during the shipment. The Investigator will check the temperature record and store it in the Site TMF. The Investigator shall inform the Sponsors immediately of any shipment temperature out of the range. The Investigator must maintain an inventory record of study vaccine received and administered. At the end of the clinical trial, all study vaccines will be returned to EVI/ICGEB or designee, including study vaccines used and/or not administered to subjects. A shipment log identifying each vial will be included in the shipment. In case a vial of vaccine is broken or unusable, the vaccine manager should replace it with a replacement vaccine vial. Although the Sponsor need not be notified immediately in these cases, documentation of the use of the replacement vial and reason for using it must be recorded by the Investigator on the vaccine administration page of the CRF and on the "Study Vaccines Accountability Record".

At the conclusion of the clinical trial and as appropriate during the course of the clinical trial, the Investigator will return all used and unused study vaccines, packaging, labels, and a copy of the

Information contained in this publication is the property of EVI and ICGEB and is confidential and will not be disclosed to third parties without written authorization from EVI and ICGEB. No part of this document may be reproduced, stored in a retrieval system or transmitted in any form or by any means -electronic, mechanical, recording or otherwise - without the prior authorization of EVI and ICGEB

completed vaccine accountability ledger to the monitor or to DLS/appropriate designee at the address provided in the Investigator folder at each site.

6.4 Method of Assigning Subjects to Vaccination Groups

6.4.1 Subject Numbering

Each subject is usually uniquely identified in the study by a combination of his/her centre number and subject number. Upon signing pre-screening informed consent form, the subject will be assigned a screening number. Once assigned to a subject, a subject screening number will not be reused. Only if the subject tests negative for anti-malarial antibodies, he will be administered the main informed consent form. Following the signing of the main informed consent, further study-related procedures will be undertaken. Similarly, the subject will be assigned a randomisation number once he fulfils all the eligibility criteria. If the subject fails to be randomised for any reason, the reason for not being randomised will be entered on the appropriate pages of the CRF.

6.4.2 Screening Number

The pre-screening informed consent form will be administered to the subject following which the subject will be assigned a unique screening number. This number will be alphanumeric of length 4. The first digit of this number will be S, and the next three digits identify the subject and will be assigned sequentially, with 001 corresponding to the first subject pre-screened/screened. Thus, study subjects will get screening numbers like S001, S002 and so on. The informed consent process for the main study using the main informed consent form will be conducted at the clinic following which the same unique screening number assigned to the subject will be used. Thus, the same number will be used to identify the subject both during the pre-screening as well as the screening period.

6.4.3 Randomisation Number

All subjects eligible after screening will undergo a complete physical examination and evaluation of medical history. A complete review of inclusion/exclusion criteria (including laboratory assessments) will be conducted. Provided all the inclusion criteria and no exclusion criteria are met, subject will be enrolled into the study and will be assigned a unique randomisation number.

The randomisation number will be a three-digit subject number and will be cohort specific. The first digit identifies cohort as 1 (Cohort 1), 2 (Cohort 2) or 3 (Cohort 3), and the next two digits identify the subject within cohort and will be assigned sequentially as per the enrolment. Thus, study subjects in Cohort 1 will get randomisation numbers like 101, 102 and so on sequentially, study subjects in Cohort 2 will get randomisation numbers like 201, 202 and so on sequentially and study subjects in Cohort 3 will get randomisation numbers like 301, 302 and so on sequentially.

6.4.4 Adherence to Randomisation List

The Investigator will designate separate appropriately qualified personnel as the "vaccine manager" and the "vaccinator" respectively. The vaccine manager will be a qualified and trained individual, who will be exclusively dedicated to vaccine preparation while the "vaccinator" will be an independent physician/nurse, who will be responsible for administration of the assigned vaccine as indicated in the randomisation list for the individual subject.

Information contained in this publication is the property of EVI and ICGEB and is confidential and will not be disclosed to third parties without written authorization from EVI and ICGEB. No part of this document may be reproduced, stored in a retrieval system or transmitted in any form or by any means -electronic, mechanical, recording or otherwise - without the prior authorization of EVI and ICGEB

Only the subject will be blinded during the conduct of this single-blind study. Since the study and control vaccines can be distinguished by their appearance, the vaccine preparation area and the vaccine administration area will be physically separated. Also, the syringe barrels will be covered with opaque tape to ensure subject blinding.

Adherence to randomisation list will be verified by the study monitor by checking the randomisation list against the study vaccine administration records at each study visit.

6.4.5 Emergency Unblinding

The present study is designed as a single-blind study. Hence, only the subjects taking part in the study will not know whether they have been assigned to the investigational vaccine or the control vaccine. The investigators, investigator staff, persons performing the assessments, and data analysts will remain unblinded to the identity of the vaccine and hence unblinding per se is not applicable for this study.

Information contained in this publication is the property of EVI and ICGEB and is confidential and will not be disclosed to third parties without written authorization from EVI and ICGEB. No part of this document may be reproduced, stored in a retrieval system or transmitted in any form or by any means -electronic, mechanical, recording or otherwise - without the prior authorization of EVI and ICGEB

7 STUDY CONDUCT, ASSESSMENTS AND VISIT PROCEDURES

7.1 General Aspects

This is a phase I, randomised, controlled, dose-escalating, single-blind clinical study for assessment of the safety and immunogenicity of the JAIVAC-1 – Montanide ISA 720 in healthy Indian male subjects between 18 to 45 years of age.

There are two (2) discreet informed consent documents for this study. Following the signing of the pre-screening informed consent form, each subject will be assigned a unique screening number. Thereafter, the required blood sample for testing for anti-malarial antibodies by ELISA will be drawn. Only if the subject tests negative for anti-malarial antibodies, he will be further administered the main informed consent form.

Following the signing of the main informed consent for the actual study participation, the relevant study related activities and screening procedures will be undertaken. The subject's demographic data, medical history and concomitant medication history will be recorded at screening/baseline. All subjects included in screening will undergo a detailed physical examination (including vital signs) as well as all protocol-defined laboratory assessments for the screening period (D-14 through D-1).

Informed consent process will include counselling about birth control measures for male subjects by the Investigator team. Results of HIV, Hepatitis B, and Hepatitis C virus infection will be reported in a confidential manner to the study subject only and will not be reported to anyone outside of the study.

A complete review of inclusion/exclusion criteria will be conducted and subjects in whom all inclusion criteria and no exclusion criteria are met, will be enrolled in the study if in good health as judged by physical examination and medical history and will be given a unique randomisation number.

The protocol-defined screening period is 14 days. In case, a subject exceeds this defined screening period (D-14 through D-1), then the subject will have to be re-screened keeping the same screening number. This subject will undergo all the screening procedures again prior to eligibility check and study entry; however the previously done pre-screening procedures and the pre-screening as well as main informed consents will be considered valid at the time of this re-screening. Apart from this, no retest for any abnormal laboratory parameters reported during the screening period will be allowed.

There will be three (3) cohorts in this study corresponding to the three (3) dosages of the investigational vaccine being evaluated. Following enrolment, the first 15 eligible subjects will be randomised and assigned to either the investigational vaccine arm (JAIVAC-1 – Montanide ISA 720 vaccine, n= 10 subjects) or the control vaccine arm (Hepatitis B vaccine, n= 5 subjects) of the Cohort 1 in a ratio of 2:1 respectively, both administered as a 3-dose schedule. Subsequent 15 subjects will be assigned to Cohort 2 and the last 15 subjects will ultimately be a part of Cohort 3 in a similar randomisation ratio of 2:1.

Information contained in this publication is the property of EVI and ICGEB and is confidential and will not be disclosed to third parties without written authorization from EVI and ICGEB. No part of this document may be reproduced, stored in a retrieval system or transmitted in any form or by any means -electronic, mechanical, recording or otherwise - without the prior authorization of EVI and ICGEB

Thus, dosing of the JAIVAC-1 – Montanide ISA 720 vaccine will begin with Cohort 1 (i.e. 0.1 ml (10 µg each antigen) of investigational vaccine administered by intramuscular route as a 3-dose schedule, followed by Cohort 2 (i.e. 0.25 ml (25 µg each antigen) of investigational vaccine) and finally Cohort 3 (i.e. 0.5 ml (50 µg each antigen) of investigational vaccine). Each cohort will be staggered into sub-cohorts such that the subjects in each cohort will be enrolled over a three day period. The first three (3) subjects of the first dose (Cohort 1) will be kept under observation for 24 hrs following vaccination. The decision of the 24-hour housing for the remaining subjects will be jointly taken by the Principal Investigator and Medical and Safety Monitor, DLS.

Dose escalation to a higher dosage of JAIVAC-1 – Montanide ISA 720 will occur after review of safety data by the DSMB through Day 14 after the first vaccination for all subjects in a cohort. In each cohort, each subject will receive a total of three (3) doses of either of the study or control vaccines by intramuscular route, one each on Day 0, Day 28 and Day 180 respectively. Thus, overall 30 subjects will receive JAIVAC-1 – Montanide ISA 720 (10 subjects for each cohort) and 15 subjects will receive control vaccine, Hepatitis B vaccine (5 subjects for each cohort) as follows:

Cohort 1

**Test Arm 1**	10 subjects shall receive dosage A of investigational vaccine (0.1 ml of JAIVAC-1 containing 10µg PfMSP-1_{19} and 10µg PfF2 formulated with adjuvant Montanide ISA 720) by intramuscular route on Days 0, 28 and 180
**Control Arm 1**	5 subjects shall receive control vaccine (1 ml of Hepatitis B vaccine) by intramuscular route on Days 0, 28 and 180

Cohort 2

**Test Arm 2**	10 subjects shall receive dosage B of investigational vaccine (0.25 ml of JAIVAC-1 containing 25µg PfMSP-1_{19} and 25µg PfF2 formulated with adjuvant Montanide ISA 720) by intramuscular route on Days 0, 28 and 180
**Control Arm 2**	5 subjects shall receive control vaccine (1 ml of Hepatitis B vaccine) by intramuscular route on Days 0, 28 and 180

Cohort 3

**Test Arm 3**	10 subjects shall receive dosage C of investigational vaccine (0.5 ml of JAIVAC-1 containing 50µg PfMSP-1_{19} and 50µg PfF2 formulated with adjuvant Montanide ISA 720) by intramuscular route on Days 0, 28 and 180
**Control Arm 3**	5 subjects shall receive control vaccine (1 ml of Hepatitis B vaccine) by intramuscular route on Days 0, 28 and 180

Subjects, who discontinue participation before completing the study, and those who are prematurely withdrawn from the study for any reason, should be scheduled for a visit as soon as possible, at which time all of the assessments listed for the final visit will be performed.

7.2 Safety Assessments

Safety assessment will begin with detailed medical history, record of concomitant medications, detailed physical examination and measurement of vital signs at each visit. The specific safety assessments for this study will include the following:

Clinical Safety Assessments

Information contained in this publication is the property of EVI and ICGEB and is confidential and will not be disclosed to third parties without written authorization from EVI and ICGEB. No part of this document may be reproduced, stored in a retrieval system or transmitted in any form or by any means -electronic, mechanical, recording or otherwise - without the prior authorization of EVI and ICGEB

- Immediate reactogenicity (reactions recorded every 30 minutes till the first 3 hours after each vaccination, with emphasis on allergic /anaphylactic reactions)
- Local and systemic solicited reactogenicity measured from Day 0 (3 hours after vaccination to Day 14 after each vaccination
- Any unsolicited adverse event up to 28 days after each vaccination
- Any Serious Adverse Event (SAE) occurring from the inclusion throughout the study till one-year (Day 365) after the first vaccination

Laboratory Safety Assessments

- Laboratory assessments 28 days after each vaccination, in reference with the baseline before the first dose, by measuring the haematological and biochemical parameters.

7.2.1 Assessment of Immediate Reactogenicity

Vital signs (including temperature, pulse, respiratory rate and blood pressure) will be recorded prior to each dose of the vaccine. After the administration of each dose of the assigned study vaccine, the subject will be monitored for any changes in vital signs every 30 minutes till 3 hours post-vaccination (with a window period of +/- 5 minutes for post-dose vital signs). Vital signs will be done after the subject has been resting quietly for at least 5 minutes. BP will be taken in the supine position. Appropriate medical treatment will be readily available at the study site to manage any adverse event with special emphasis on allergic/ anaphylactic reactions.

7.2.2 Assessment of Local and Systemic Reactogenicity (Solicited AEs)

Local and systemic reactogenicity will be assessed for a period of 14 days following each vaccination i.e. from Days 0 to 14 for the first dose, Day 28 to 42 for the second dose and Day 180 to 194 for the third dose of the assigned study vaccine. This assessment of local and systemic signs and symptoms will be done based both on the subject diary card and investigator assessment as follows:

- For the first 14 days the subjects will be asked to record the local and systemic signs and symptoms (Solicited AEs) in their diary cards (pre-printed).
- In addition, 14 days after each vaccination (i.e. on Days 14, 42 and 194), the investigator will do an independent assessment of the local and systemic signs and symptoms.

7.2.2.1 Solicited AEs by Subject Diary

During the first 14 days (2 weeks) following each dose of the assigned study vaccine (Days 0 to 14, Day 28 to 42 and Day 180 to 194), subjects will be asked to record local and general signs and symptoms (Solicited AEs) using pre-printed diary cards, thermometer, and small ruler.

Solicited AEs to be reported by the subject are pre-printed on the diary cards and consist of the following:

- Local Reactogenicity (at the injection site) - pain, swelling, redness, induration and limitation of arm motion abduction at shoulder

Information contained in this publication is the property of EVI and ICGEB and is confidential and will not be disclosed to third parties without written authorization from EVI and ICGEB. No part of this document may be reproduced, stored in a retrieval system or transmitted in any form or by any means -electronic, mechanical, recording or otherwise - without the prior authorization of EVI and ICGEB

- Systemic Reactogenicity (systemic signs) - fever (axillary temperature) or systemic symptoms - headache, malaise, myalgia, arthralgia, nausea and vomiting.
- The investigator will review the subject diary cards and transcribe the data on solicited AEs on to the CRF on Days 7, 14, 35, 42, 187 and 194.
- The above-mentioned local and systemic symptoms and signs will be graded on the pre-printed diary card by the subject or the investigator as specified in the diary card instructions and based on Table 2: Scoring of Severity of Local Solicited Adverse Events and Table 3: Scoring of Severity of Systemic Solicited Adverse Events.

7.2.2.2 Solicited AEs by Investigator's Assessment

The Investigator will do an independent assessment of the local and systemic signs and symptoms 14 days after each vaccination (i.e. on Days 14, 42 and 194),

- During these visits, the investigator will perform a complete physical examination (including general examination for temperature, lymphadenopathy and any other local or systemic signs and symptoms).
- In addition, the investigator will specifically check for the presence of a nodule **(10)** at the injection site. The size and outcome of the nodule (if any) will be recorded on the CRF pages, as well as the size, localisation and outcome of the local lymph nodes.
- The investigator will independently evaluate the subjects for solicited AEs on Days 14, 42 and 194.
- The above-mentioned local symptoms and signs (solicited AEs) will be graded on the CRF by the Investigator on Days 7, 14, 35, 42, 187 and 194 as per Table 2: Scoring of Severity of Local Solicited Adverse Events and Table 3: Scoring for Severity of Systemic Solicited Adverse Events

7.2.2.3 Assessment of Severity of Solicited Adverse Events

Criteria for severity ranking for solicited AEs are given in Table 2 **(11)-13)** and Table 3 **(11), 14)** below. For events measured with continuous variables such as fever or diameters, the severity grades are provided for completeness. The investigator will report the highest measured value and the corresponding severity grade in the CRF.

Information contained in this publication is the property of EVI and ICGEB and is confidential and will not be disclosed to third parties without written authorization from EVI and ICGEB. No part of this document may be reproduced, stored in a retrieval system or transmitted in any form or by any means -electronic, mechanical, recording or otherwise - without the prior authorization of EVI and ICGEB

Table 2: Scoring of Severity of Local Solicited Adverse Events	
**Pain at injection site**	**Score/Grade**
Absent	**0**
Painful on touch	**1**
Painful when limb is moved	**2**
Spontaneously painful or painful at rest	**3**
**Swelling, Redness, Induration**	**Score/Grade**
Absent	**0**
Longest diameter: <25 mm	**1**
Longest diameter: ≥ 25 mm to < 50mm	**2**
Longest diameter: ≥ 50mm	**3**
**Limitation of arm motion abduction at shoulder**	**Score/Grade**
Angle of voluntary arm abduction is 180°	**0**
Angle of voluntary arm abduction is > 90° but < 120°	**1**
Angle of voluntary arm abduction is > 30° but < 90°	**2**
Angle of voluntary arm abduction is ≤ 30°	**3**

Information contained in this publication is the property of EVI and ICGEB and is confidential and will not be disclosed to third parties without written authorization from EVI and ICGEB. No part of this document may be reproduced, stored in a retrieval system or transmitted in any form or by any means -electronic, mechanical, recording or otherwise - without the prior authorization of EVI and ICGEB

Table 3: Scoring for Severity of Systemic Solicited Adverse Events	
**Fever (axillary temperature measured once daily even in the absence of signs)**	**Score/Grade**
<38°C	**0**
38°C to <39°C	**1**
39°C to <40°C	**2**
≥40°C	**3**
**Headache, Malaise, Myalgia, Arthralgia, Nausea and Vomiting**	**Score/Grade**
Absent	**0**
Present, but easily tolerated	**1**
Discomforting enough to interfere with normal activities	**2**
Disabling, prevents normal daily activity, requires bed rest and treatment	**3**

7.2.3 Assessment of Unsolicited Adverse Events

- During the first 28 days after each dose of the assigned vaccine, the subject will record any unsolicited AEs in the subject diary card.
- These unsolicited AEs will be reviewed and transcribed onto the Adverse Event CRF pages by the Investigator, using appropriate medical terminology and appropriate scoring for severity of AE (as applicable).

7.2.4 Assessment of Any Serious Adverse Event (SAE)

- During the entire study period (from study inclusion till last follow-up visit i.e. till Day 365), the Investigator will record and report all Serious Adverse Events (SAEs); whether or not related to the vaccination.

7.2.5 Assessment of Severity for all Unsolicited Adverse Events (including any Serious Adverse Events)

The Investigator will record, report and grade the following unsolicited AEs on the Adverse Event CRF pages, using medical language and as per Table 4: Scoring for severity of systemic unsolicited Adverse Events:

Information contained in this publication is the property of EVI and ICGEB and is confidential and will not be disclosed to third parties without written authorization from EVI and ICGEB. No part of this document may be reproduced, stored in a retrieval system or transmitted in any form or by any means -electronic, mechanical, recording or otherwise - without the prior authorization of EVI and ICGEB

- All unsolicited adverse events that occurred during the first 28 days following each dose of the vaccine, and
- All Serious Adverse Events during the entire study period (from study inclusion till last follow-up visit)

Table 4: Scoring for severity of systemic unsolicited Adverse Events

Unsolicited Adverse Event Characteristics	Score/Grade
Sign or symptom present, but easily tolerated	**1**
Discomforting enough to interfere with normal activities	**2**
Disabling, incapacitating with inability to perform usual activity	**3**

7.2.6 Other Clinical Safety Assessments

- During the entire study period (from study inclusion till last follow-up visit), the Investigator will also record details of all the concomitant medications taken.
- During each visit the investigator will perform a complete physical examination (including general examination for temperature, lymphadenopathy and other local or systemic signs and symptoms).
- In addition, on Days 14, 42 and 194, the investigator will specifically check for the presence of a nodule at the injection site. The size, localisation and outcome of the lymph nodes will be recorded on the CRF pages, as well as the size and outcome of the nodule if any.

7.2.7 Laboratory Safety Assessments

Laboratory assessments will be done to assess biological safety at 28 days after each vaccination in reference with the baseline before the first dose, by measuring the following:

- Haematology: RBC count, Haemoglobin*, Haematocrit/Packed Cell Volume (PCV), MCV (Mean Corpuscular Volume), MCH (Mean Corpuscular Haemoglobin), MCHC (Mean Corpuscular Haemoglobin Concentration), Platelet Count and Total Leukocyte Count (TLC) along with Differential Leukocyte Count (DLC) on Days -14, 28, 56, 180, 208, 365.

  *If the haemoglobin drops below 13 gm/dL, then a direct Coomb's test and a peripheral blood smear/film will be prepared and examined for evidence of possible haemolysis

Information contained in this publication is the property of EVI and ICGEB and is confidential and will not be disclosed to third parties without written authorization from EVI and ICGEB. No part of this document may be reproduced, stored in a retrieval system or transmitted in any form or by any means -electronic, mechanical, recording or otherwise - without the prior authorization of EVI and ICGEB

- Serum Chemistry: Potassium, Sodium, AST, ALT, Direct, Indirect and Total Bilirubin, Alkaline Phosphatase, Gamma Glutamyl Transpeptidase (γGT), Creatinine, and Random blood glucose on Days -14, 28, 56, 180, 208, 365

- The total amount of blood sample required for these assessments across all visits would be approximately 60 ml.
- For all the laboratory evaluations, the investigator will receive the results/reports as per specified timelines from receipt of specimen at DLS (provided acceptable specimens and/or requisition forms have been obtained).
- The Sponsor's Medical Monitor will be alerted about any value significantly outside the normal range (Refer to the DLS Laboratory Manual for details).
- The screening (D-14 through D-1) and post-vaccination safety laboratory evaluations will be performed at the Central Laboratory at DLS, Mumbai, India.

<table>
<tr><th colspan="7">TABLE 5: SCHEDULE OF SAFETY LABORATORY EVALUATIONS</th></tr>
<tr><th>PARAMETERS</th><th colspan="6">VOLUME OF BLOOD</th></tr>
<tr><th>VISITS</th><th>V2</th><th>V6</th><th>V9</th><th>V10</th><th>V13</th><th>V14</th></tr>
<tr><th>DAYS</th><th>D-14 to D-1</th><th>D28</th><th>D56</th><th>D180</th><th>D208</th><th>D365</th></tr>
<tr><td>Haematology: Haemoglobin, haematocrit (PCV), MCV, MCH, MCHC, RBC count, TLC, DLC, Platelet count</td><td>2 ml</td><td>2 ml</td><td>2 ml</td><td>2 ml</td><td>2 ml</td><td>2 ml</td></tr>
<tr><td>Direct Coomb's Test (Peripheral Smear Test, as required)</td><td>2 ml</td><td>2 ml</td><td>2 ml</td><td>2 ml</td><td>2 ml</td><td>2 ml</td></tr>
<tr><td>Serum Chemistry: Random blood glucose, sodium, potassium</td><td rowspan="3">5 ml</td><td rowspan="2">3.5 ml</td><td rowspan="2">3.5 ml</td><td rowspan="2">3.5 ml</td><td rowspan="2">3.5 ml</td><td rowspan="2">3.5 ml</td></tr>
<tr><td>Organ function tests:
Hepatic: Direct, indirect and total bilirubin, alanine-amino transferase (ALT/SGPT), aspartate-amino-transferase (AST/SGOT), alkaline Phosphatase and γGT
Renal: Serum creatinine</td></tr>
<tr><td>Viral serology tests: HIV, HBs Ag and HCV</td><td>---</td><td>---</td><td>---</td><td>---</td><td>---</td></tr>
</table>

Information contained in this publication is the property of EVI and ICGEB and is confidential and will not be disclosed to third parties without written authorization from EVI and ICGEB. No part of this document may be reproduced, stored in a retrieval system or transmitted in any form or by any means -electronic, mechanical, recording or otherwise - without the prior authorization of EVI and ICGEB

7.3 Immunogenicity Assessments

The secondary immunogenicity endpoints of the present study will be assessed as follows:

- The humoral response to the candidate vaccine antigen will be assessed (quantitative assessment) by measuring the level of IgG antibodies developed against PfMSP-1_{19} and PfF2 by ELISA on Days 0, 28, 56, 180, 208 and 365
- The humoral response to the candidate vaccine antigen will be assessed (qualitative assessment) to verify the ability of the IgG antibodies developed against PfMSP-1_{19} and PfF2 to recognise the native protein on late stage *P. falciparum* schizonts and merozoites in vitro by IFA on Days 0, 28, 56, 180, 208 and 365.

The exploratory immunogenicity endpoints (for assessment of the quality of the humoral immune response) of the present study will be assessed as follows:

- IgG1, IgG2, IgG3, IgG4 subclasses by ELISA on samples obtained at Days 0, 28, 56, 180, 208 and 365.
- The ability of the IgG antibodies to block homologous and heterologous parasite growth in vitro by a *P. falciparum* blood-stage growth inhibition assay (GIA) against three parasite strains on samples obtained at D0, 56, 208 and 365

The immunogenicity laboratory evaluations will be performed at ICGEB, New Delhi, India.

<table>
<tr><th colspan="8">TABLE 6: SCHEDULE OF IMMUNOGENICITY LABORATORY EVALUATIONS</th></tr>
<tr><th>PARAMETERS</th><th colspan="7">VOLUME OF BLOOD</th></tr>
<tr><th>VISITS</th><th>V1</th><th>V3</th><th>V6</th><th>V9</th><th>V10</th><th>V13</th><th>V14</th></tr>
<tr><th>DAYS</th><th>D-21 to D-14</th><th>D0</th><th>D28</th><th>D56</th><th>D180</th><th>D208</th><th>D365</th></tr>
<tr><td>Serology (ELISA): quantitative assessment- IgG antibodies developed against PfMSP-1_{19} and PfF2 (including screening ELISA)</td><td>4 ml</td><td rowspan="4">18 ml</td><td rowspan="3">6 ml</td><td rowspan="4">18 ml</td><td rowspan="3">6 ml</td><td rowspan="4">18 ml</td><td rowspan="4">18 ml</td></tr>
<tr><td>Serology (IFA): qualitative assessment- IgG antibodies developed against PfMSP-1_{19} and PfF2</td><td>---</td></tr>
<tr><td>Serology (IgG subclasses by ELISA): IgG1, IgG2, IgG3, IgG4 subclasses</td><td>---</td></tr>
<tr><td>Serology (GIA)</td><td>---</td><td>---</td><td>---</td></tr>
</table>

Information contained in this publication is the property of EVI and ICGEB and is confidential and will not be disclosed to third parties without written authorization from EVI and ICGEB. No part of this document may be reproduced, stored in a retrieval system or transmitted in any form or by any means -electronic, mechanical, recording or otherwise - without the prior authorization of EVI and ICGEB

Approximately 4 ml of whole blood will be drawn at Visit 1 and 6 ml of whole blood at Visits 6 and 10. Approximately 18 ml of whole blood will be drawn to provide about 8-9 ml of serum for serology efficacy assessments at Visits 3, 9, 13 and 14. Thus, approximately 100 ml of whole blood sample will be drawn across all visits. The serum samples will be stored frozen at -70ºC (with a temperature variation of +/- 10ºC) before batch shipment to the laboratory for analysis. The measurements will be performed at ICGEB.

7.3.1 Anti-PfMSP-1_{19} and Anti-PfF2 IgG antibodies (ELISA)

ELISA will be used to measure total levels of antibody (specific IgG concentrations) to *Plasmodium falciparum* antigens PfMSP-1_{19} and PfF2. The concentration of specific IgG antibodies in human sera is determined relative to a human standard reference serum pool using the procedure developed earlier **(15)**. The ELISA plate will be coated with either PfMSP-1_{19} or PfF2 antigen and will be incubated with human sera at various dilutions. Anti-human IgG conjugated with horseradish peroxidase will be added to the ELISA plate and colour will develop following addition of the substrate for the peroxidase. The analyses will be performed at ICGEB, New Delhi, India.

7.3.2 Anti-PfMSP-1_{19} and Anti-PfF2 IgG antibodies (IFA)

An IFA will be employed to verify that the antibodies elicited by the vaccine recognise the native protein in late stage *P. falciparum* schizonts and merozoites. Cultured parasite at schizont and merozoite stages will be used to prepare IFA slides. Slides will be incubated with human sera collected at D0, 28, 56, 180, 208 and 365 and further incubated with fluorescein isothiocyanate labelled secondary antibody. The bound antibodies will be analyzed using fluorescence microscope **(15)**. The analyses will be performed at ICGEB, New Delhi, India.

7.3.3 Exploratory Efficacy End-points

The quality of the humoral immune response will be assessed by measuring:

- IgG1, IgG2, IgG3, IgG4 subclasses by ELISA on samples obtained at D0, 28, 56, 180, 208 and 365. Assay will be performed on ELISA plate coated with either PfMSP-1_{19} or PfF2. Human sera obtained at various intervals will be added to the ELISA plate and bound antibodies will be detected by anti-human IgG subtype specific antibody **(15)**. The analyses will be performed at ICGEB, New Delhi, India.
- The ability to block parasite growth *in vitro* by a *P. falciparum* blood-stage growth inhibition assay (GIA) against three parasite strains on samples obtained at D0, 56, 208 and 365. GIA will be performed with purified IgG obtained from human sera with the synchronized parasite at trophozoite stage. *P. falciparum* schizonts stage cultures will be incubated along with test and control sera in a 96 well plate and growth of the parasite will be monitored by colorimetric lactate dehydrogenase assay **(16)**. The analyses will be performed at ICGEB, New Delhi, India.

Additional tests on available samples may be performed if deemed necessary by the sponsors based on findings in the present study or in other studies, which necessitate further investigation of the immunogenicity of the vaccine.

Information contained in this publication is the property of EVI and ICGEB and is confidential and will not be disclosed to third parties without written authorization from EVI and ICGEB. No part of this document may be reproduced, stored in a retrieval system or transmitted in any form or by any means -electronic, mechanical, recording or otherwise - without the prior authorization of EVI and ICGEB

7.4 Visit Schedule and Procedures

All eligible subjects will be asked to attend a total of 14 visits (Visit 1 to Visit 14).

Table 7 lists all of the assessments and indicates with an "x" against the visits at which the assessments are performed.

The subjects will be instructed to contact the investigator immediately any time after the vaccination, should they manifest with any signs or symptoms they perceive as serious.

Subjects, who discontinue the study vaccine before completing the study, and those who prematurely withdraw from the study for any reason, should be scheduled for a visit as soon as possible, at which time all of the assessments listed for the final visit (Day 365) will be performed.

If they refuse to return for these assessments or are unable to do so, every effort will be made to contact them or a knowledgeable informant by telephone to determine their health status.

Information contained in this publication is the property of EVI and ICGEB and is confidential and will not be disclosed to third parties without written authorization from EVI and ICGEB. No part of this document may be reproduced, stored in a retrieval system or transmitted in any form or by any means -electronic, mechanical, recording or otherwise - without the prior authorization of EVI and ICGEB

7.4.1 Visit Schedule

TABLE 7: PROTOCOL JAIVAC-1_1_09 - SCHEDULE OF VISITS AND PROCEDURES

Visit	1	2	3	4	5	6	7	8	9	10	11	12	13	14
Day	*Day -21 to Day -15*	*-14 to Day -1*	*0*	*7*	*14*	*28*	*35*	*42*	*56*	*180*	*187*	*194*	*208*	*365*
Window Period [1]			*± 1 Day*	*± 1 day*	*± 1 day*	*± 1 day*	*± 1 day*	*± 1 day*	*± 1 day*	*± 1 day*	*± 1 day*	*± 1 day*	*± 1 day*	*± 7 days*
Study Period [2]	Pre-Screening Period	Screening Period	Vaccination Period								Follow up Period			
Vaccination			Dose 1			Dose 2				Dose 3				
Informed Consent[3]	*x*	*x*												
Screening Number[4]	*x*	*x*												
Demographics[5]	*x*													
ELISA for Anti-malaria Antibodies[6]	*x*													
Medical History		*x*												
Concomitant Medications		*x*	*X*	*x*	*X*	*X*	*x*	*x*	*x*	*x*	*x*	*x*	*x*	*x*
Physical Examination		*x*	*X*	*x*	*X*	*X*	*x*	*x*	*x*	*x*	*x*	*x*	*x*	*x*
Vital Signs (TPR and BP)[7]		*x*	*X*	*x*	*X*	*X*	*x*	*x*	*x*	*x*	*x*	*x*	*x*	*x*
12-Lead Electrocardiogram and X-Ray Chest[7]		*x*												
Birth Control Couselling[8]		*x*	*X*			*X*				*x*				
Haematology[9]		*x*				*X*			*x*	*x*			*x*	*x*
Serum Chemistry[10]		*x*				*X*			*x*	*x*			*x*	*x*
HIV, HBV, HCV Tests		*x*												
Direct Coomb's Test[11]		*x*				*X*			*x*	*x*			*x*	*x*
Peripheral Blood Smear [12]		*x*				*X*			*x*	*x*			*x*	*x*
Urine Drug Screen[13]		*x*				*X*				*x*				*x*
Check Inclusion Criteria		*x*	*X*											
Check Exclusion Criteria		*x*	*X*											
Randomisation number[14]			*X*											
Distribute Diary Cards[15]			*X*			*X*				*x*				
Collect Diary Cards [15]						*X*			*x*				*x*	
Review Diary Cards[15]				*x*	*x*		*x*	*x*			*x*	*x*		
Transcribe Diary Cards [15]				*x*	*x*	*X*	*x*	*x*	*x*		*x*	*x*	*x*	
Record Immediate Reactogenicity[16]			*X*			*X*				*x*				
Record Solicited AEs[17]			*X*	*x*	*x*	*X*	*x*	*x*		*x*	*x*	*x*		
Record Unsolicited AEs			*X*	*x*	*x*	*X*	*x*	*x*	*x*	*x*	*x*	*x*	*x*	
Record SAEs			*X*	*x*	*x*	*X*	*x*	*x*	*x*	*x*	*x*	*x*	*x*	*x*
Serology (ELISA and IFA)[18]			*X*			*X*			*x*	*x*			*x*	*x*
Serology (IgG subclasses)[19]			*X*			*X*			*x*	*x*			*x*	*x*
Serology (GIA)[19]			*X*						*x*				*x*	*x*
Check Discontinuation Criteria				*x*	*x*	*X*	*x*	*x*	*x*	*x*	*x*	*x*	*x*	

Please refer to the footnotes below.

Information contained in this publication is the property of EVI and ICGEB and is confidential and will not be disclosed to third parties without written authorization from EVI and ICGEB. No part of this document may be reproduced, stored in a retrieval system or transmitted in any form or by any means -electronic, mechanical, recording or otherwise - without the prior authorization of EVI and ICGEB

Following are the footnotes for TABLE 7: PROTOCOL JAIVAC-1_1_09 - SCHEDULE OF VISITS AND PROCEDURES

1. *Window Period: A window period of specific number of days has been assigned for each visit. (Refer Table 1 for details). In case a subject cannot come on a scheduled day he can be called anytime in the window period for that particular visit but for subsequent visits he should be urged to adhere to the original visit schedule.*
2. *Study Period: The study consists of four (4) distinct study periods: A pre-screening period of 7 days, a screening period of 14 days, a vaccination period of 180 days and a follow-up period till 365 days from the first dose of the assigned study vaccines.*
3. *Informed Consent: There are two (2) discreet informed consent documents for this study. Following the signing of the pre-screening informed consent, blood sample for testing for anti-malarial antibodies will be taken. Only if the subject tests negative for anti-malarial antibodies, he will be administered the main informed consent form. Following the signing of the main informed consent, further study-related procedures will be undertaken.*
4. *Screening number: Following the signing of the pre-screening informed consent, each subject will be assigned a unique screening number. This same number will be used to identify the subject both during the pre-screening as well as the screening period.*
5. *Demographics: At the pre-screening Visit (Visit 1), each subject shall be required to provide demographic information (including date of birth, height, weight, gender, and race).*
6. *ELISA for Anti-malarial Antibodies: Blood sample drawn in the pre-screening period will be used for ELISA test for anti-malarial antibodies. Subjects with evidence of IgG antibodies against PfMSP-119 and PfF2 as measured by ELISA at pre-screening visit (Visit 1) will be not be further considered for the study.*
7. *Vital Signs and 12-lead ECG: General examination including vital signs i.e. temperature, pulse, respiratory rate (TPR) and blood pressure (supine BP) will be performed at every visit. One 12-lead ECG and one X-Ray Chest will be done at the screening visit (Visit 2).*
8. *Birth Control Couselling: The importance of strict adherence to the birth control measures during the study duration will be emphasised at these visits to the male subjects.*
9. *Haematology: Blood samples will be collected for haematology assessments (including RBC Count, haemoglobin, haematocrit, MCV, MCH, MCHC, platelet count, TLC and DLC) at screening visit (any time from D-14 through D-1), D28, D56, D180, D208 and D365. Haematology assessments done at Screening Visit (Visit 2) will be considered as screening laboratory assessments for eligibility as well as baseline assessments for safety end-points since haematology assessments will not be done on Day 0 (pre-vaccination). Blood samples for haematology will be collected prior to vaccination on Days 28 and 180.*
10. *Serum Chemistry: Blood samples will be collected for serum chemistry assessments (including potassium, sodium, AST, ALT, direct, indirect and total bilirubin, alkaline phosphatase, γGT, creatinine, random blood glucose) during screening period (D-14 through D-1), D28, D56, D180, D208 and D365. Serum chemistry assessments done at Screening Visit (Visit 2) will be considered as screening laboratory assessments for eligibility as well as baseline assessments for safety end-points since serum chemistry assessments will not be done on Day 0 (pre-vaccination). Blood samples for serum chemistry will be collected prior to vaccination on Days 28 and 180.*
11. *Direct Coomb's Test: Direct Coomb's test will be undertaken at Screening Visit (Visit 2) and additionally will be done during the entire study period if the haemoglobin falls below* 13 gm/dL

Information contained in this publication is the property of EVI and ICGEB and is confidential and will not be disclosed to third parties without written authorization from EVI and ICGEB. No part of this document may be reproduced, stored in a retrieval system or transmitted in any form or by any means -electronic, mechanical, recording or otherwise - without the prior authorization of EVI and ICGEB

12. *Peripheral Blood Smear (PBS): PBS examination will be undertaken for evidence of haemolysis or any haematological disorder at Screening Visit (Visit 2) and additionally during the entire study period along with Coomb's test if the haemoglobin falls below* 13 gm/dL
13. *Urine Drug Screen: Urinalysis to test for drugs of abuse (benzodiazepines, opioids, amphetamine, cocaine, and THC) will be undertaken once at the Screening Visit (Visit 2) and subsequently on D28, D180 and D365. If the subject tests positive during screening period, he will be excluded from the study. If the subject tests positive on D28, D180 or D365, he will be immediately discontinued from the study.*
14. *Randomisation Number: Subjects, who fulfill all the eligibility criteria, will be randomised into the study with the assignment of a unique randomisation number.*
15. *Diary Cards: A pre-printed diary card will be given to the subject with detailed completion instructions on the day of vaccine administration and will be collected on Day 28 after each dose of vaccine. This diary card will be used for the record of solicited AEs (Day 0 through Day 14) and unsolicited AEs (Day 0 through Day 28). Thus three (3) such diary cards will be given to each subject. The Investigator will collect, review and transcribe the data from diary cards to CRF at pre-defined time-points.*
16. *Record Immediate Reactogenicity: Following vaccination on Days 0, 28 and 180, all subjects will be monitored at the site every 30 minutes till 3 hours for any immediate reactions with special emphasis on allergic/anaphylactic reactions. Vital signs will be done after the subject has been resting quietly for at least 5 minutes. BP will be taken in the supine position.*
17. *Record Solicited AEs: Each subject will record solicited AEs from vaccination through Day 14 after each dose on the diary card. These solicited AEs will be reviewed and transcribed from diary cards to CRF by the Investigator on Days 7, 14, 35, 42, 187 and 194. In addition, solicited AEs will be independently assessed by the investigator Days 14, 42 and 194 and the data directly entered in the CRF.*
18. *Serology (ELISA and IFA): Blood samples will be collected for serology assessment (ELISA and IFA) on Days 0, 28, 56, 180, 208 and 365. On Days 0, 28 and 180, blood samples will be collected prior to vaccination.*
19. *Serology (IgG subclasses and GIA): Blood samples will be collected for serology assessment (IgG subclasses by ELISA) on Days 0, 28, 56, 180, 208 and 365 and for GIA on Days 0, 56, 208 and 365 only. On Days 0, 28 and 180, blood samples will be collected prior to vaccination.*

Information contained in this publication is the property of EVI and ICGEB and is confidential and will not be disclosed to third parties without written authorization from EVI and ICGEB. No part of this document may be reproduced, stored in a retrieval system or transmitted in any form or by any means -electronic, mechanical, recording or otherwise - without the prior authorization of EVI and ICGEB

7.4.2 Overview of Study Procedures and Assessments

The following assessments will be undertaken during the study:

Informed Consent

There are two (2) discreet informed consent documents for this study. At Visit 1, the pre-screening informed consent will be administered prior to taking blood sample for testing for anti-malarial antibodies. At Visit 2, before undertaking any study related activities, the main informed written consent will be taken from each and every subject. Only after the subject understands and willingly signs the informed consent form, all other study related activities would begin.

Demographics

At Visit 1, each subject shall be required to provide demographic information (including date of birth, height, weight, gender, and race).

ELISA for anti-malarial antibodies

During pre-screening period (D-21 through D-15), a blood sample will be collected for assessment of anti-malarial antibody titres by ELISA. Subjects without evidence of IgG antibodies against PfMSP-1_{19} and PfF2 as measured by ELISA will be further administered the main informed consent.

Medical History

At Visit 2, all subjects will be questioned regarding current signs and symptoms, their active and past medical history and other relevant chronic medical conditions and the same will be recorded as a part of medical history.

Concomitant Medications

A concomitant medications history will be sought at every visit including screening visit (at any time in the screening period of D-14 through D-1 and those subjects who have medicated themselves with any of the prohibited medications during the study period may be terminated from the study.

Physical Examination/Vital Signs/12-lead ECG

A general physical examination including vital signs (temperature, pulse rate, respiratory rate and blood pressure) will be performed at each visit. 12-lead ECG will be done only once at any time in the screening period of D-14 through D-1. Vital signs will be recorded after the subject has been resting quietly for at least five (5) minutes. BP will be recorded in the supine position.

Birth Control Counselling

All male subjects participating in the study will be counselled at the pre-defined visits (including screening visit) regarding the various birth control options described in the protocol. The importance of strict adherence to the birth control measures in order to avoid pregnancy in the female partner during the study duration will be emphasised.

Haematology

Blood samples will be collected on Days -14, 28, 56, 180, 208 and 365 for haematology assessments like RBC Count, Haemoglobin*, Haematocrit/Packed Cell Volume (PCV), MCV

Information contained in this publication is the property of EVI and ICGEB and is confidential and will not be disclosed to third parties without written authorization from EVI and ICGEB. No part of this document may be reproduced, stored in a retrieval system or transmitted in any form or by any means -electronic, mechanical, recording or otherwise - without the prior authorization of EVI and ICGEB

(Mean Corpuscular Volume), MCH (Mean Corpuscular Haemoglobin), MCHC (Mean Corpuscular Haemoglobin Concentration), Platelet Count and Total Leukocyte Count (TLC) along with Differential Leukocyte Count (DLC). **If the haemoglobin drops below 13 gm/dL, then a direct Coomb's test and a peripheral blood smear/film will be prepared and examined for evidence of possible haemolysis*

Serum Chemistry

Blood samples will be collected on Days -14, 28, 56, 180, 208 and 365 for serum chemistry assessments which include potassium, sodium, AST, ALT, direct, indirect and total bilirubin, alkaline phosphatase, γGT, creatinine and random blood glucose. At the discretion of the Investigator, additional laboratory testing maybe performed to assist in identifying any other conditions that may affect the subject's eligibility.

HIV, HBV and HCV Tests

Blood samples will be collected at Visit 2 for HIV, HBV and HCV testing. Those who test positive will not be included in the study and appropriately counselled and managed as per applicable policies.

Direct Coombs Test and Peripheral Blood Smear

Test for Direct Coomb's test and peripheral blood smear/film for evidence of haemolysis will be done at screening and subsequently when haemoglobin drops to less than 13 gm/dL

Urine Drug Screen

Urinalysis to test for drugs of abuse (benzodiazepines, opioids, amphetamine, cocaine, and THC) will be undertaken at Visits 2, 6, 10 and 14

Randomisation Number

A subject will be randomised into the study and assigned a unique randomisation number only after completion of an eligibility check of a subject's inclusion/exclusion criteria by the Investigator. The subject will be recognised by the randomisation number for all study-related assessments and procedures.

Diary Cards

Distribution: Pre-printed diary cards will be given to subject on the days of vaccine administration (Days 0, 28 and 180) and will be collected on Day 28 following each vaccination. Detailed instructions to fill the diary cards will be given to the subjects at the time of dispensing the diary. These diary cards will be used for the record of solicited AEs (local and systemic reactogenicity) till Day 14 and unsolicited AEs till Day 28 after each vaccination by the subject. Thus three (3) such diary cards will be given to each subject.

Review and Transcription: 7 and 14 days after each vaccination, i.e. on Days 7, 14, 35, 42, 187 and 194, the investigator will review the subject diary card and transcribe the local and systemic reactogenicity data and the unsolicited AEs from the subject diary cards into the CRF.

Collection: Pre-printed diary cards given to the subject on the days of vaccine administration (Days 0, 28 and 180) will be collected 28 days after each vaccination i.e. respectively on Days 28, 56 and 208.

Record Immediate Reactogenicity

Information contained in this publication is the property of EVI and ICGEB and is confidential and will not be disclosed to third parties without written authorization from EVI and ICGEB. No part of this document may be reproduced, stored in a retrieval system or transmitted in any form or by any means -electronic, mechanical, recording or otherwise - without the prior authorization of EVI and ICGEB

On Day 0, 28 and 180, after administration of the assigned study vaccine, the subject will be monitored for any changes in vital signs (including temperature, pulse, respiratory rate and blood pressure) every 30 minutes till 3 hours post-vaccination. Appropriate medical treatment will be readily available at the study site to manage any adverse event with special emphasis on allergic/anaphylactic reactions. Vital signs will be assessed after the subject has been resting quietly for at least five (5) minutes. BP will be taken in the supine position.

Record Solicited AEs (Local and Systemic Reactogenicity)

Subjects will record solicited AEs for 14 days following each vaccination. The investigator will transcribe the solicited AEs data from the diary cards into the CRF on Days 7, 14, 35, 42, 187 and 194. All solicited AEs will also be independently assessed by the investigator 14 days after each vaccination (Days 14, 42 and 194).

Record Unsolicited AEs and SAEs

Subjects will record unsolicited AEs for 28 days following each vaccination in the diary cards. The Investigator will report all unsolicited adverse events that occur during the first 28 days following each dose of the vaccine and all SAEs during the entire study period (from study inclusion till last follow-up visit i.e. Day 365)

Serology Assessments

Blood samples will be collected on Days 0, 28, 56, 180, 208 and 365 for assessment of:

- IgG antibodies developed against PfMSP-1_{19} and PfF2 by ELISA
- Ability of the IgG antibodies developed against PfMSP-1_{19} and PfF2 to recognise the native protein on late stage *P. falciparum* schizonts and merozoites *in vitro* by IFA
- IgG1, IgG2, IgG3, IgG4 subclasses by ELISA

Blood samples will be collected on Days 0, 56, 208 and 365 for assessment of ability to block parasite growth *in vitro* by a *P. falciparum* blood-stage growth inhibition assay (GIA) against three (3) parasite strains

Check Discontinuation Criteria

At Visits 4, 5, 6, 7, 8, 9, 10, 11, 12 and 13 the Investigator will check for development of the subject discontinuation criteria per section 5.4.4.

7.4.3 Visit-wise Study Procedures and Assessments

7.4.3.1 Visit 1 (Day -21 to Day -15), Pre-Screening Visit

- Pre-Screening Informed consent
- Assignment of screening number
- Demographics
- ELISA for anti-malarial antibodies

7.4.3.2 Visit 2 (Day -14 to Day -1), Screening Visit

- Main Informed consent

Information contained in this publication is the property of EVI and ICGEB and is confidential and will not be disclosed to third parties without written authorization from EVI and ICGEB. No part of this document may be reproduced, stored in a retrieval system or transmitted in any form or by any means -electronic, mechanical, recording or otherwise - without the prior authorization of EVI and ICGEB

- Medical history/concomitant medications
- Physical examination/vital signs/12-lead ECG
- Birth control counselling
- Haematology (*direct Coomb's test and peripheral blood smear*)
- Serum chemistry
- HIV, HBV, HCV tests
- Urine drug screening for drugs of abuse
- Check inclusion criteria
- Check exclusion criteria

7.4.3.3 Visit 3 (Day 0, First Dose of the Vaccine)

- Concomitant medications
- Physical examination/vital signs
- Birth control counselling
- Check inclusion criteria
- Check exclusion criteria
- Assignment of randomisation number
- Distribution of first diary card by Investigator
- Vaccine administration (first dose)
- Record of immediate reactogenicity (first dose)
- Record of solicited and unsolicited AEs/SAEs by subject in diary card
- Serology (ELISA, IFA, IgG subclases and GIA)

7.4.3.4 Visit 4 (Day 7)

- Concomitant medications
- Physical examination/vital signs
- Review of first diary card by Investigator
- Transcription of data from diary card to CRF by Investigator (solicited and unsolicited AEs)
- Record of any SAEs by Investigator
- Check discontinuation criteria

7.4.3.5 Visit 5 (Day 14)

- Concomitant medications

Information contained in this publication is the property of EVI and ICGEB and is confidential and will not be disclosed to third parties without written authorization from EVI and ICGEB. No part of this document may be reproduced, stored in a retrieval system or transmitted in any form or by any means -electronic, mechanical, recording or otherwise - without the prior authorization of EVI and ICGEB

- Physical examination/vital signs
- Review of first diary card by Investigator
- Transcription of data from diary card to CRF by Investigator (solicited and unsolicited AEs)
- Independent assessment by Investigator and direct record in CRF (solicited and unsolicited AEs)
- Record of any SAEs by Investigator
- Check discontinuation criteria

7.4.3.6 Visit 6 (Day 28, Second Dose of the Vaccine)

- Concomitant medications
- Physical examination/vital signs
- Birth control counselling
- Haematology (*direct Coomb's test and peripheral blood smear if required*)
- Serum chemistry
- Urine drug screening for drugs of abuse
- Collection and review of first diary card by Investigator (unsolicited AEs)
- Transcription of data from diary card to CRF by Investigator (unsolicited AEs)
- Distribution of second diary card by Investigator
- Vaccine administration (second dose)
- Record of immediate reactogenicity (second dose)
- Record of any SAEs by Investigator
- Serology (ELISA, IFA and IgG subclasses)
- Check discontinuation criteria

7.4.3.7 Visit 7 (Day 35)

- Concomitant medications
- Physical examination/vital signs
- Review of second diary card by Investigator
- Transcription of data from diary card to CRF by Investigator (solicited and unsolicited AEs)
- Record of any SAEs by Investigator
- Check discontinuation criteria

Information contained in this publication is the property of EVI and ICGEB and is confidential and will not be disclosed to third parties without written authorization from EVI and ICGEB. No part of this document may be reproduced, stored in a retrieval system or transmitted in any form or by any means -electronic, mechanical, recording or otherwise - without the prior authorization of EVI and ICGEB

7.4.3.8 Visit 8 (Day 42)

- Concomitant medications
- Physical examination/vital signs
- Review of second diary card by Investigator for second dose (solicited and unsolicited AEs)
- Transcription of data from diary cards to CRF by Investigator for second dose (solicited and unsolicited AEs)
- Independent assessment by Investigator and direct record in CRF for second dose (solicited and unsolicited AEs)
- Record of any SAEs by Investigator
- Check discontinuation criteria

7.4.3.9 Visit 9 (Day 56)

- Concomitant medications
- Physical examination/vital signs
- Haematology *(direct Coomb's test and peripheral blood smear if required)*
- Serum Chemistry
- Collection and review of second diary card by Investigator (unsolicited AEs)
- Transcription of data from diary card to CRF by Investigator (unsolicited AEs)
- Record of any SAEs by Investigator
- Serology (ELISA, IFA, IgG subclasses and GIA)
- Check discontinuation criteria

7.4.3.10 Visit 10 (Day 180, Third Dose of the Vaccine)

- Concomitant medications
- Physical examination/vital signs
- Birth control counselling
- Haematology (*direct Coomb's test and peripheral blood smear if required*)
- Serum chemistry
- Urine drug screening for drugs of abuse
- Distribution of third diary card by Investigator
- Vaccine administration (third dose)
- Record of immediate reactogenicity (third dose)
- Record of any SAEs by Investigator

Information contained in this publication is the property of EVI and ICGEB and is confidential and will not be disclosed to third parties without written authorization from EVI and ICGEB. No part of this document may be reproduced, stored in a retrieval system or transmitted in any form or by any means -electronic, mechanical, recording or otherwise - without the prior authorization of EVI and ICGEB

- Serology (ELISA, IFA and IgG subclasses)
- Check discontinuation criteria

7.4.3.11 Visit 11 (Day 187)

- Concomitant medications
- Physical examination/vital signs
- Review of third diary card by Investigator
- Transcription of data from diary card to CRF by Investigator (solicited and unsolicited AEs)
- Record of any SAEs by Investigator
- Check discontinuation criteria

7.4.3.12 Visit 12 (Day 194)

- Concomitant medications
- Physical examination/vital signs
- Review of third diary card by Investigator for third dose (solicited and unsolicited AEs)
- Transcription of data from diary cards to CRF by Investigator for third dose (solicited and unsolicited AEs)
- Independent assessment by Investigator and direct record in CRF for third dose (solicited and unsolicited AEs)
- Record of any SAEs by Investigator
- Check discontinuation criteria

7.4.3.13 Visit 13 (Day 208)

- Concomitant medications
- Physical examination/vital signs
- Collection and review of third diary card by Investigator (unsolicited AEs)
- Transcription of data from diary card to CRF by Investigator (unsolicited AEs)
- Haematology (*Direct Coomb's test and peripheral blood smear if required*)
- Serum chemistry
- Record of any SAEs by Investigator
- Serology (ELISA, IFA, IgG subclasses and GIA)
- Check discontinuation criteria

7.4.3.14 Visit 14 (Day 365, End of Study Visit)

- Concomitant medications

Information contained in this publication is the property of EVI and ICGEB and is confidential and will not be disclosed to third parties without written authorization from EVI and ICGEB. No part of this document may be reproduced, stored in a retrieval system or transmitted in any form or by any means -electronic, mechanical, recording or otherwise - without the prior authorization of EVI and ICGEB

- Physical examination/vital signs
- Haematology (*direct Coomb's test and peripheral blood smear if required*)
- Serum chemistry
- Urine drug screening for drugs of abuse
- Record any SAEs by the Investigator
- Serology (ELISA, IFA, IgG subclasses and GIA)

Information contained in this publication is the property of EVI and ICGEB and is confidential and will not be disclosed to third parties without written authorization from EVI and ICGEB. No part of this document may be reproduced, stored in a retrieval system or transmitted in any form or by any means -electronic, mechanical, recording or otherwise - without the prior authorization of EVI and ICGEB

8 SAFETY MONITORING

8.1 Introduction

The collection, recording, assessment and reporting of adverse events (AE) and serious adverse events (SAE) represent the core activities for the safety evaluation. These activities are under the direct responsibility of the Investigator.

The recording of adverse events is an important aspect of study documentation as the evaluation of the safety of three (3) intramuscular injections of the investigational vaccine (JAIVAC-1 – Montanide ISA 720 malaria vaccine) in comparison with that of the control vaccine (Hepatitis B vaccine) is the primary objective of this study. The procedure for evaluation of immediate reactogenicity, solicited adverse events and unsolicited adverse events is described Section 7. The investigator is responsible for the detection and documentation of events meeting the definition of an adverse event (AE) or a serious adverse event (SAE) as provided in Sections 8.4 and 8.5 of this protocol. This includes the evaluation of its seriousness, its severity, and the causal relationship to the investigational vaccine and/or concomitant therapy.

At each visit, the investigator will assess whether any AE including laboratory abnormalities has occurred. The investigator shall inquire for unsolicited AEs with non-suggestive questions to the subject at each study visit. The investigator shall attempt to establish a diagnosis of the event based on signs, symptoms and/or other clinical information. The diagnosis shall be recorded as the AE and/or SAE and not the individual symptoms.

If a laboratory abnormality or other abnormal assessment meets the definition of an AE or SAE, the Adverse Event or Serious Adverse Event Form shall be completed as appropriate. A diagnosis, if known or clinical signs and symptoms if diagnosis is unknown, rather than the laboratory abnormality shall be reported. If no diagnosis is known and clinical signs and symptoms are not present, then the abnormal finding shall be recorded. The subject shall be observed and monitored carefully until the AE resolves, the condition stabilises or its cause is identified completely. The investigator is responsible for ensuring that follow-up includes any supplemental investigations that may be indicated to elucidate the nature and/or the cause of the event.

8.2 Data Safety Monitoring Board

An independent Data Safety Monitoring Board (DSMB) will be set up prior to initiating the study comprising of three (3) independent clinicians with relevant expertise in the field of vaccines development and evaluation of vaccine safety along with the designated Local Safety Monitor (who would be a designated personnel from the investigational site but independent of the study team). Dose escalation to the next higher dosage cohort of JAIVAC-1 – Montanide ISA 720 will occur after the safety data from the first dose of vaccine through Day 14 day for all 15 subjects of the previous dosage cohort has been reviewed by the DSMB.

8.3 Post-vaccination Reactions

The procedure for evaluation of post-vaccination reactions including immediate reactogenicity, solicited AEs (local and systemic reactogenicity recorded till 14 days after vaccination) and unsolicited AEs till 28 days after vaccination as described in details in Section 7.

Information contained in this publication is the property of EVI and ICGEB and is confidential and will not be disclosed to third parties without written authorization from EVI and ICGEB. No part of this document may be reproduced, stored in a retrieval system or transmitted in any form or by any means -electronic, mechanical, recording or otherwise - without the prior authorization of EVI and ICGEB

8.4 Adverse Events

8.4.1 Definition of an Adverse Event

Adverse Event (AE) is defined as any untoward medical occurrence in a subject or clinical investigation subject administered a pharmaceutical product and which does not necessarily have a causal relationship with this treatment. An AE can therefore be any unfavourable and unintended sign (including an abnormal laboratory finding), symptom, or disease temporally associated with the use of the Medicinal (investigational) product (ICH GCP definition).

8.4.2 Reporting of Adverse Events

To ensure subject safety, every AE, regardless of suspected causality, occurring after the subject has received the first dose of the vaccine till 28 days after the subject has received each dose of the vaccine will be reported. Information about all adverse events, whether volunteered by the subject, discovered by investigator questioning, or detected through physical examination, laboratory test or other means, will be collected and recorded on the Adverse Event Case Report Form and followed as appropriate. Thus, AEs will be collected through both solicited and unsolicited means and subsequently coded in tabular form using the latest version of MedDRA Adverse Event Dictionary. The subjects will be encouraged to report signs, symptoms, and any changes in health to the Investigator. Severity of each AE will be determined by the staff based on observation and questioning of the subjects. The Investigator will judge the relationship of the event to the study treatments.

Adverse events occurring before the subject has signed the informed consent as well as those which occur in the screening period are recorded on the Medical History. Abnormal laboratory values or test results constitute adverse events if they are not a part of any specific diagnosis, induce clinical signs or symptoms, require any action to be taken or any intervention to be made, and are considered as clinically significant. Such clinically significant abnormal lab values will be recorded on the Adverse Events Case Report Form under the signs, symptoms or diagnosis associated with them and will be followed till resolution or stabilization of the same.

8.4.3 Documentation of Adverse Events

Whenever possible, AEs should be documented in terms of a diagnosis or syndrome rather than multiple symptoms that are clearly manifestations of the same diagnosis/syndrome. If a diagnosis can not be obtained then enter each sign or symptoms as separate events. Pre-existing conditions or signs and/or symptoms (including any which are not recognised at study entry but are recognised during the study period) present in a subject prior to Visit 2 should be recorded in the Medical History form in the subject's CRF.

AEs that occur after subject is enrolled in the study, but prior to first vaccination, will be documented in the medical history and pre-existing conditions sections of the CRF. Hospitalisation for either elective surgery related to a pre-existing condition which did not increase in severity or frequency following initiation of the study, or for routine clinical procedures (including hospitalisation for "social" reasons) that are not the result of an AE, must be recorded on the Adverse Event page of the CRF. The relationship to vaccination will be checked "No". These AEs are not considered as SAEs.

Information contained in this publication is the property of EVI and ICGEB and is confidential and will not be disclosed to third parties without written authorization from EVI and ICGEB. No part of this document may be reproduced, stored in a retrieval system or transmitted in any form or by any means -electronic, mechanical, recording or otherwise - without the prior authorization of EVI and ICGEB

As far as possible, each adverse event will be described by:

1. Duration of AE (start and end dates)
2. Severity of AE (as per scoring/grading system)
3. Relationship to the study vaccine (related, unrelated)
4. Action(s) taken and, as relevant, the outcome.

8.4.4 Assessment of Severity of Adverse Events

The following three-point rating scale will be used for rating the severity of all unsolicited AEs/SAEs (apart from the ones covered in Section 7.2):

Grade 1: Awareness of signs or symptoms, no interference with daily activities

Grade 2: Symptoms cause discomfort with some interference with daily activities

Grade 3: Disabling, the subject is unable to conduct a range of daily activities

8.4.5 Assessment of Causality of Adverse Events

Assessment of causality Adverse Event (related or not related) will be done by the Investigator. The following scale will be used for rating the causal relationship of the AE to the investigational product (vaccine):

Causality Assessment: Related

The adverse event is clearly related to the vaccine i.e. an event that follows a reasonable temporal sequence from administration of the vaccine, follows a known or expected response pattern to the suspected vaccine, that is confirmed by improvement on stopping and reappearance of the event on repeated exposure and that could not be reasonably explained by the known characteristics of the subject s clinical state.

Causality Assessment: Not Related

The adverse event is clearly not related to the vaccine i.e. another cause of the event is most plausible; and/or a clinically plausible temporal sequence is inconsistent with the onset of the event and the vaccine administration; and/or a causal relationship is considered biologically implausible.

8.4.6 Assessment of Expectedness of Adverse Event

An unexpected AE is an AE, the nature or severity of which is not consistent with the applicable product (vaccine) information, e.g. investigator's brochure for an unapproved investigational product (vaccine), or package insert/summary of product characteristics for an approved product (vaccine). Assessment of expectedness of Adverse Event will be done by the Sponsor.

8.4.7 Follow-up of Adverse Events and Assessment of Outcome

The Investigator should follow-up a subject with an AE until the event has subsided / resolved or until the condition has stabilised. In addition, all serious adverse events and those non-serious events assessed by the Investigator as related to the investigational vaccine should continue to be

Information contained in this publication is the property of EVI and ICGEB and is confidential and will not be disclosed to third parties without written authorization from EVI and ICGEB. No part of this document may be reproduced, stored in a retrieval system or transmitted in any form or by any means -electronic, mechanical, recording or otherwise - without the prior authorization of EVI and ICGEB

followed even after the subject's participation in the clinical trial is over. Such events should be followed until they resolve or until the Investigator assesses them as "chronic" or "stable. Follow-up reports must be submitted to the Medical and Safety Monitor using SAE Report Form for serious adverse events or Data Clarification Request Form for non-serious events.

8.5 Serious Adverse Events

8.5.1 Definition of a Serious Adverse Event

Serious Adverse Event (SAE) is defined as any untoward medical occurrence that at any dose:

- results in death,

- is life-threatening,

- requires subject hospitalisation or prolongation of existing hospitalisation,

- results in persistent or significant disability/incapacity, or

- is a congenital anomaly/birth defect

(ICH GCP definition)

Life threatening AE: An AE is life threatening if the subject was at immediate risk of death from the event as it occurred; i.e. it does not include a reaction that if it had occurred in a more serious form it might have caused death. For example, drug-induced hepatitis that resolves without evidence of hepatic failure would not be considered life threatening even though drug-induced hepatitis could be fatal.

Hospitalisation: In general, hospitalisation signifies that the subject has been detained at the hospital or emergency ward (usually involving at least an overnight stay) for observation and/or treatment that would not have been appropriate in the physician's office or out-subject setting. When in doubt as to whether hospitalisation' occurred or was necessary, the AE should be considered serious. Hospitalisation for elective surgery or routine clinical procedures that are not the result of an AE (e.g., elective surgery for a pre-existing condition) need not be considered AEs. If anything untoward is reported during the procedure, that occurrence must be reported as an AE, either 'serious' or 'nonserious' according to the usual criteria.

Disabling/incapacitating AE: An AE is incapacitating or disabling if it results in a substantial and/or permanent disruption of the subject s ability to carry out normal every-day activities.

Medically important condition: Medical and scientific judgment should be exercised in deciding whether expedited reporting is appropriate in other situations, such as important medical events that may not be immediately life-threatening or result in death or hospitalisation, but may jeopardise the subject or may require intervention to prevent one of the other outcomes listed in the definition above. These should also usually be considered serious. Examples of such events are intensive treatment in an emergency room or at home for allergic bronchospasm, blood dyscrasias or convulsions that do not result in hospitalisation, or development of drug dependency or drug abuse.

Information contained in this publication is the property of EVI and ICGEB and is confidential and will not be disclosed to third parties without written authorization from EVI and ICGEB. No part of this document may be reproduced, stored in a retrieval system or transmitted in any form or by any means -electronic, mechanical, recording or otherwise - without the prior authorization of EVI and ICGEB

8.5.2 Reporting Serious Adverse Events

To ensure subject safety, every SAE, regardless of suspected causality, occurring after the subject has received the first dose of the vaccine till Day 365 (end of study) must be reported by the Principal Investigator/designee to the Sponsor and DLS within 24 hours of the knowledge of its occurrence. Recurrent episodes, complications, or progression of the initial SAE must be reported as follow-up to the original episode, regardless of when the event occurs. This report must be submitted within 24 hours of the investigator receiving the follow-up information. An SAE that is considered completely unrelated to a previously reported one should be reported separately as a new event.

Every SAE will be reported to the Medical and Safety Monitor at DLS as well as the Sponsor Representatives (both ICGEB and EVI) by telephone/e-mail within 24 hours from observation, regardless of the treatment group or suspected relationship to study vaccines. In addition, for each SAE, the investigator must assess the relationship to the study vaccine, complete the SAE Report Form and send the completed, signed form by e-mail/fax as soon as possible. The telephone and fax number of the Medical and Safety Monitor at DLS will be listed in the investigator folder provided to the study site. The original copy of the SAE Report Form and the fax confirmation sheet must be kept with the case report form documentation at the study site. The initial report should be followed by a full written summary (using SAE Report Form) detailing relevant aspects of the AE, in sufficient detail to allow for a complete medical assessment of the case and independent determination of possible causality. In general, this information includes copies of relevant hospital case records, autopsy reports and other documents where applicable. The initial and follow-up reports shall identify the trial subjects by the unique randomisation number assigned to each subject.

8.5.3 Processing and Regulatory Submission of SAE Reports

All SAE reports will be processed, evaluated and reported to the Sponsor, IEC(s)/IRB(s) and regulatory agencies as per applicable timelines.

Every serious adverse event must be reported by the Investigator to the Medical and Safety Monitor, DLS and the Sponsor representatives (ICGEB/EVI) within 24 hours of the knowledge of SAE.

The Medical and Safety Monitor will send the following to the Sponsor's Contact Person; preferably by e-mail or fax:

- Reports for death or life-threatening SAEs not later than one (1) business day from Date of Receipt from the Investigator
- Reports for other SAEs not later than two (2) business days from the Date of Receipt from the Investigator
- Reports on pregnancy cases in female partner of male subjects (Pregnancy Reports), as applicable not later than two (2) business days from the Date of Receipt from the Investigator

Information contained in this publication is the property of EVI and ICGEB and is confidential and will not be disclosed to third parties without written authorization from EVI and ICGEB. No part of this document may be reproduced, stored in a retrieval system or transmitted in any form or by any means -electronic, mechanical, recording or otherwise - without the prior authorization of EVI and ICGEB

In addition, the following SAE Reporting timelines will be followed:

- The Principal Investigator will send the SAE report to the site EC within 7 working days from the day of awareness of occurrence of the SAE
- The ICGEB Representative will send the SAE report to the ICGEB IRB within 7 working days from the day of awareness of occurrence of the SAE
- DLS will send the SAE Reports for unexpected and related SAEs, which are deaths/life-threatening SAEs occurring at study site to the DCGI within 7 calendar days from the day of awareness of occurrence of the SAE with a follow-up report sent within the next 8 calendar days.
- DLS will send the SAE Reports for all other SAEs occurring at study site to the DCGI within 14 calendar days from the day of awareness of occurrence of the SAE

Contact Information

The Sponsor's Contact Person(s):

1. European Vaccine Initiative (EVI)

Name: Dr. Egeruan Babatunde Imoukhuede

Designation: Director of Clinical and Regulatory Affairs

Tel: +44 208 674 8318

Email: babatunde.imoukhuedei@emvi.org

2. International Centre for Genetic Engineering and Biotechnology (ICGEB)

Name: Dr. Chetan Chitnis

Designation: Staff Research Scientist

Tel: +91 98104 00155

E-mail: cchitnis@icgeb.res.in

The CRO's Contact Person(s)/Medical and Safety Monitor:

DiagnoSearch Life Sciences Pvt. Ltd. (DLS)

Name: Dr. Supriya Desai

Designation: Associate Director, Medical Affairs and Safety Management

Tel: +91 99207 51248

E-mail: supriya.desai@diagnosearch.com

8.5.4 Assessment of Severity, Causality and Expectedness of Adverse Events

Assessment of severity of all SAEs will follow the same criteria as for Adverse Event (related or not related) and will be done by the Investigator as described in Section 8.4.4

Information contained in this publication is the property of EVI and ICGEB and is confidential and will not be disclosed to third parties without written authorization from EVI and ICGEB. No part of this document may be reproduced, stored in a retrieval system or transmitted in any form or by any means -electronic, mechanical, recording or otherwise - without the prior authorization of EVI and ICGEB

Assessment of causality for SAEs will follow the same criteria as for Adverse Event (related or not related) and will be done by the Investigator as described in Section 8.4.5

Assessment of expectedness will be done by the Sponsor as described in Section 8.4.6 by checking against the Investigator's Brochure (for the investigational vaccine) and package insert/SmPC included in Trial Master File for the control vaccine information.

8.5.5 Follow-up of Serious Adverse Events and Assessment of Outcome

Follow-up information is sent to the sponsors contact person and the Clinical Trial Monitor using a new SAE Report Form stating that this is a follow-up to the previously reported SAE and giving the date of the original report. The follow-up information should describe whether the event has resolved or continues, if and how it was treated, whether the blind was broken or not, and whether the subject continued or withdrew from study participation.

8.6 Pregnancy Reporting

To ensure subject safety, each pregnancy occurring in the female partner of a male subject, enrolled in the study must be reported to the Sponsor within 24 hours of learning of its occurrence. The pregnancy should be followed up to determine outcome, including spontaneous or voluntary termination, details of the birth and the presence or absence of any birth defects, congenital abnormalities, or maternal and/or newborn complications.

Any pregnancy occurring in the female partner of a male subject that occurs during study participation should be recorded using a Pregnancy Report Form (PRF) and reported by the investigator to the Medical and Safety Monitor at DLS and the Sponsor within 24 hours of learning of its occurrence. The pregnancy should be followed up to determine outcome, including spontaneous or voluntary termination, details of birth, and the presence or absence of any birth defects, congenital abnormalities or maternal and newborn complications. The outcome of the pregnancy should be reported on the same Pregnancy Report Form (PRF) and should include an assessment of the possible relationship to the study vaccine of any pregnancy outcome.

Information contained in this publication is the property of EVI and ICGEB and is confidential and will not be disclosed to third parties without written authorization from EVI and ICGEB. No part of this document may be reproduced, stored in a retrieval system or transmitted in any form or by any means -electronic, mechanical, recording or otherwise - without the prior authorization of EVI and ICGEB

9 QUALITY CONTROL AND QUALITY ASSURANCE

9.1 Introduction

To ensure accurate, complete, and reliable data, EVI/ICGEB/DLS must:

- Provide instructional material to the study site, as appropriate.
- Sponsor a start-up training session to instruct the investigators and study coordinators. This session will give instruction regarding all sections of the protocol, the completion of the clinical report forms, and study procedures.
- Make periodic visits to the study site to ensure that the protocol is adhered to.
- Be available at all times for consultation and in contact with the study-site personnel by mail, telephone, and/or fax.
- Review and evaluate clinical report data and will use standard computer edits to detect errors in data collection.

To ensure accurate, complete, and reliable data, the investigator will keep records of laboratory tests, clinical notes, and subject's medical records in the subject's files as original source documents for the study. Quality Assurance Audits / inspections by regulatory bodies or EVI/ICGEB or an appointed agent may be done by visiting the investigator site and periodically assessing the progress of the clinical trial, review CRFs along with original source data with site personnel, adverse event management, study vaccine management and correspondence. Periodically the investigator sites may be inspected by the regulatory authorities, and/or audited by IEC/IRB, EVI/ICGEB or the agents appointed by EVI/ICGEB.

9.2 Pre-study Documentation

Prior to enrolment of subjects at study site, specific regulatory documents must be available, along with IEC approvals, other IRB required approvals and curriculum vitae for investigator and study staff. EVI/ICGEB/DLS will inform the investigator regarding which documents need to be provided according to the applicable regulatory requirements.

9.3 Monitoring Procedures

The sponsor should ensure that it is specified in the protocol or other written agreement that the investigator(s)/institution(s) will permit clinical trial-related monitoring, audits, IRB/IEC review, and regulatory inspection(s), providing direct access to source data/documents. Qualified and appropriately trained (including training on study specific issues) clinical study monitors designated by the sponsors will carefully monitor all aspects of the study. A representative of the sponsor may accompany this person. Study monitors will periodically contact the site and perform on-site visits. The extent, nature and frequency of site visits will be based on such considerations as study objectives, study design and complexity, and enrolment rate. Periodicity and nature of monitoring activities will be described in the Monitoring Plan.

Information contained in this publication is the property of EVI and ICGEB and is confidential and will not be disclosed to third parties without written authorization from EVI and ICGEB. No part of this document may be reproduced, stored in a retrieval system or transmitted in any form or by any means -electronic, mechanical, recording or otherwise - without the prior authorization of EVI and ICGEB

During study visits and contacts, the monitor will:

- Check and assess the progress of the study
- Review study data collected
- Perform source data verification
- Identify any issues and address their resolution

This will be done in order to verify that:

- The data are authentic, accurate and complete
- The safety and rights of subjects are being protected
- The study is conducted in accordance with the approved protocol (and any subsequent amendment), ICH-GCP and all applicable regulatory requirements.

Monitoring will be conducted according to ICH- GCP and Sponsors (and/or designee) standard operating procedures (SOPs). The individuals responsible for monitoring the study will have access to all records necessary to ensure the integrity/validity of the recorded data and will periodically review the progress of the study. The investigator agrees to allow the study monitor direct access to all relevant documents and to assign his/her time and the time of his/her staff to the monitor to discuss findings and any relevant issues. The monitor will also contact the site prior to the start of the study to discuss the protocol and data collection procedures with site personnel.

9.4 Recording of Data

In compliance with good clinical practice / ICH guidelines, the medical records and related documents should be clearly marked to permit easy identification of participation by an individual in this study. The investigator must record all data with respect to study procedures, study vaccine administration, concomitant medications, laboratory data, adverse events and efficacy ratings on the CRF. The investigator must, as a minimum, sign the final CRF to certify the correctness and completeness of all the data captured. All corrections on the CRF and on source data must be made in such a way that does not obscure the original entry and maintain the transparency. In order to provide the Data Management Centre and Sponsor with accurate, complete, and legible case reports, the following will be ensured:

- The original data entry will remain legible
- There will be no erasures, write-overs or use of correction fluid or tape
- Each error will be corrected separately.
- Errors will be corrected by placing one line through the error.
- The correct entry will appear next to the error and the Investigator or the designated and authorised site team member (as per the on the Site Delegation /Signature Log) making the change will initial and date each correction.
- Wherever possible, a reason for the correction should be given.
- Changes to CRFs previously signed by the Investigator will be initialled and dated by the Investigator after the change is made.

Information contained in this publication is the property of EVI and ICGEB and is confidential and will not be disclosed to third parties without written authorization from EVI and ICGEB. No part of this document may be reproduced, stored in a retrieval system or transmitted in any form or by any means -electronic, mechanical, recording or otherwise - without the prior authorization of EVI and ICGEB

- Changes made to the CRFs via data clarification forms issued by Sponsor or its designee will be signed by the Investigator.

9.5 Audits and Inspections

EVI/ICGEB and/or DLS will visit the investigator site periodically to assess the progress of the clinical trial, review CRFs along with original source data with site personnel, adverse event management, study vaccine management and correspondence. Periodically the investigator sites may be reviewed or inspected by the regulatory authorities and the IEC/IRB. This may occur at any time from start to after conclusion of the study. The investigator agrees to allow the auditor direct access to all relevant documents and to assign his/her time and the time of his/her staff to the auditor to discuss findings and any relevant issues. National and foreign Regulatory agencies may conduct a regulatory inspection of this study. If a regulatory authority requests an inspection, the investigator must inform the sponsors or its designee immediately about this request. The investigator agrees to allow the inspector(s) direct access to all relevant documents and to assign his/her time and the time of his/her staff to the inspector(s) to discuss findings and any relevant issues.

9.6 Study and Site Closure

Premature termination of the clinical trial may occur because of a regulatory authority decision, change in the opinion of the IEC/IRB or vaccine safety issues at the discretion of EVI/ICGEB. Recruitment at a centre may be stopped for reasons of particularly low enrolment, protocol violations, or inadequate data recording. The investigator also must provide follow-up medical care for all subjects who are prematurely withdrawn from the study, or must refer them for appropriate ongoing care. Once the study ends formally at the site either because the study has been completed (all the subjects have completed their participation in the study) or because of other reasons such as poor enrolment rates, poor site performance or IEC/IRB decision, investigator /site/ decision etc, the following close out activities will be done:

- "End of Study" letter will be sent to the investigator
- Instructions for retention of study documents at the site will be given to the investigators
- Unused CRF's, non vaccine supplies and equipment should be returned to EVI/ICGEB or designee or destroyed at site as per instructions provided by the sponsor.
- All study vaccines should be accounted for and the unused supplies returned to EVI/ICGEB or designee or destroyed at the site as per instructions.
- Investigator should send a letter to the IEC/IRB stating that the study has been closed.
- The study monitor will further guide the investigators regarding the detailed close out activities.

9.7 Data Protection

Medical information about individual subjects obtained during the course of this study is confidential and may not be disclosed to third parties, except authorised monitors, sponsors, auditors or inspectors. Confidentiality will be ensured by the use of subject number for the

Information contained in this publication is the property of EVI and ICGEB and is confidential and will not be disclosed to third parties without written authorization from EVI and ICGEB. No part of this document may be reproduced, stored in a retrieval system or transmitted in any form or by any means -electronic, mechanical, recording or otherwise - without the prior authorization of EVI and ICGEB

identification of each subject; which will also be used for subject data in the subject files at the site and for the CRFs.

10 DATA MANAGEMENT AND PROCESSING

10.1 Data Collection

The CRF will be supplied as 2-part no carbon required (NCR) paper. All data must be entered legibly, in English, as described in the CRF completion guidelines. An explanation must be provided for any missing data. All the completed CRFs will be checked for completeness and accuracy by the study monitor. One NCR copy will be retained at the site and the original (white) copy will be sent to the Data Management Centre by the study monitor. The investigator must sign and date the CRFs, attesting to his responsibility for the quality of all data recorded and that the data represent a complete and accurate record of each subject's participation into the study.

10.2 Data Management Procedures

Data from the CRFs are entered into the study database by Data Management staff using double data entry system. Concomitant medications entered into the database will be coded using the WHO Drug Dictionary. Medical history/current medical conditions and adverse events will be coded using the Medical dictionary for regulatory activities (latest version of MedDRA) terminology. At the conclusion of the study, the occurrence of any protocol violations will be determined.

Sponsor / sponsor designee will perform all statistical analyses. The Sponsor (EVI and ICGEB), designated Medical Writers and the designated Statisticians will be responsible for the interpretation of study results and appropriate conduct of an internal review process for both the final study report and any study report and study-related material to be authorised for publication by EVI/ICGEB.

10.3 Data Verification Procedures

After data have been entered in the study database, they will be checked systematically by the staff of the data management center according to a pre-specified data validation plan. For this process, error messages printed by a validation program and database listings for medical in-house review will be used. For errors or missing data, Query Forms will be created and sent to the study monitor for clarification at the investigational site. After answering, one copy of the signed Query Form will be added to the CRF copy at the study site, while the signed original query form will be transferred to the data management center for entering into the study database. After these actions have been completed and the database has been declared to be complete and accurate, it will be locked and made available for data analysis.

10.4 Coding

All medical verbatim terms will be coded by a medical doctor according to latest version of MedDRA Adverse Event Dictionary. (Adverse events and medical history) and the WHO Drug Dictionary enhanced version (concomitant medication).

Information contained in this publication is the property of EVI and ICGEB and is confidential and will not be disclosed to third parties without written authorization from EVI and ICGEB. No part of this document may be reproduced, stored in a retrieval system or transmitted in any form or by any means -electronic, mechanical, recording or otherwise - without the prior authorization of EVI and ICGEB

10.5 Procedures for Analysis of Consistency and Medical Plausibility

All listings of the database will be reviewed and discussed prior assessment of consistency and medical plausibility. After resolution of all issues, the Statistical Analysis Plan (SAP) will be finalised and the database will be locked after resolution of all remaining queries. An audit trail will be kept of all subsequent changes to the data.

10.6 Data to be recorded directly into the CRF

All the relevant data will be recorded in the source notes as well as the relevant sections of the CRF. This will include; weight, height and vital signs like temperature, pulse, respiratory rate and blood pressure as well as physical examination findings. For solicited as well as unsolicited adverse events, the diary card will be considered as source data.

Information contained in this publication is the property of EVI and ICGEB and is confidential and will not be disclosed to third parties without written authorization from EVI and ICGEB. No part of this document may be reproduced, stored in a retrieval system or transmitted in any form or by any means -electronic, mechanical, recording or otherwise - without the prior authorization of EVI and ICGEB

11 STATISTICAL CONSIDERATIONS AND ANALYSIS PLAN

This section describes an outline of the planned statistical methods. Prior to study completion a "Statistical Analysis Plan" will be produced as a separate document, which will contain full details of all planned analyses. The Sponsor or the Sponsor designee will perform all statistical analyses. The interpretation of study results will be the responsibility of the Sponsor (EVI and ICGEB), designated Medical Writers and the designated Statistician; all will also be responsible for the appropriate conduct of an internal review process for the interim analysis, the final statistical and the clinical study report, any other relevant study report and study-related material to be authorised for publication by EVI/ICGEB. Additional exploratory analyses of the data will be performed as deemed appropriate.

11.1 Study Conduct Considerations

11.1.1 Sample Size

Since this is a proof of safety and immunogenicity study, true sample size calculations have not been performed and the sample size is approximately 45 healthy male subjects. Therefore no statistical hypothesis will be formulated for this study and only descriptive statistics will be provided for all parameters. The data will be described between vaccines, and within vaccines using the different strata.

A sufficient number of subjects will be screened to enrol a total number of 45 subjects in the study.

- 30 subjects will receive the investigational vaccine (i.e. JAIVAC-1 – Montanide ISA 720 malaria vaccine) with 10 subjects in each cohort and
- 15 subjects will receive the control vaccine (i.e. Hepatitis B vaccine) with five (5) subjects in each cohort.

Subjects who are discontinued from the study following enrolment and after the first (1st) vaccination (drop-outs) will not be subsequently replaced.

11.1.2 Randomisation Procedures

A total of 45 healthy subjects in the age group of 18 to 45 years will be enrolled and vaccinated in the study. There will be three (3) cohorts in this study such that each cohort will consist of 15 eligible subjects randomised in a ratio of 2:1 to receive the particular dose-level of antigen of investigational vaccine i.e. JAIVAC-1 – Montanide ISA 720 vaccine (10 subjects) and the control vaccine i.e. Hepatitis B vaccine (5 subjects) respectively, both administered as a 3-dose schedule. Thus, overall 30 subjects will receive JAIVAC-1 – Montanide ISA 720 (10 subjects for each cohort) and 15 subjects will receive control vaccine, Hepatitis B vaccine (5 subjects for each cohort) as follows:

Cohort 1

**Test Arm 1**	10 subjects shall receive dosage A of investigational vaccine (0.1 ml of JAIVAC-1 vaccine containing 10μg $PfMSP1_{19}$ and 10μg PfF2 formulated

Information contained in this publication is the property of EVI and ICGEB and is confidential and will not be disclosed to third parties without written authorization from EVI and ICGEB. No part of this document may be reproduced, stored in a retrieval system or transmitted in any form or by any means -electronic, mechanical, recording or otherwise - without the prior authorization of EVI and ICGEB

	with adjuvant Montanide ISA720) by intramuscular route on Days 0, 28 and 180
**Control Arm 1**	5 subjects shall receive control vaccine (1 ml of Hepatitis B vaccine) by intramuscular route on Days 0, 28 and 180

Cohort 2

**Test Arm 2**	10 subjects shall receive dosage B of investigational vaccine (0.25 ml of JAIVAC-1 vaccine containing 25µg PfMSP1_{19} and 25µg PfF2 formulated with adjuvant Montanide ISA720) by intramuscular route on Days 0, 28 and 180
**Control Arm 2**	5 subjects shall receive control vaccine (1 ml of Hepatitis B vaccine) by intramuscular route on Days 0, 28 and 180

Cohort 3

**Test Arm 3**	10 subjects shall receive dosage C of investigational vaccine (0.5 ml of JAIVAC-1 vaccine containing 50µg PfMSP1_{19} and 50µg PfF2 formulated with adjuvant Montanide ISA720) by intramuscular route on Days 0, 28 and 180
**Control Arm 3**	5 subjects shall receive control vaccine (1 ml of Hepatitis B vaccine) by intramuscular route on Days 0, 28 and 180

Each cohort will be staggered into sub-cohorts such that the subjects in each cohort will be enrolled over a three-day period. The first three (3) subjects of the first dose (Cohort 1) will be kept under observation for 24 hrs following vaccination. Dose escalation to a higher dosage of JAIVAC-1 – Montanide ISA 720 will occur after review of safety data by the DSMB through Day 14 after the first vaccination for all subjects in a cohort. In each cohort, each subject will receive a total of three (3) doses of either of the study or control vaccines by intramuscular route, one each on Day 0, Day 28 and Day 180 respectively.

11.1.3 Blinding Procedures

This clinical trial is designed as a single-blind study. Single blind means that the subjects taking part in the study will not know whether they have been assigned to the investigational vaccine arm or the control arm. However, unlike a double blind study, here the Investigators, investigator staff, persons performing the assessments, and data analysts will not remain blind to the identity of the vaccine.

The Sponsor /designee will provide lists of participant numbers with vaccine group assignments to the site Investigator at each of the sites (i.e., unblinded list or randomisation list). The Investigator or his/her designee will use these lists to sequentially assign participants into study groups as they are enrolled. The site Investigator will work with the site staff member and design a strategy whereby this information is not inadvertently disclosed to the study subjects. The vaccine group assignment numbers will be entered on the CRF or source documents.

To facilitate auditing of vaccine administration, the subject number of the participant receiving a vaccine dose with date of injection/vaccination will be reported on to the vaccine box and vaccine

Information contained in this publication is the property of EVI and ICGEB and is confidential and will not be disclosed to third parties without written authorization from EVI and ICGEB. No part of this document may be reproduced, stored in a retrieval system or transmitted in any form or by any means -electronic, mechanical, recording or otherwise - without the prior authorization of EVI and ICGEB

vial. All vaccine boxes (containing used vials) will be saved for audit by the site and once the vaccine accountability is done these will be returned to sponsor or destroyed at site maintaining appropriate documentation. Study statisticians will be unblinded to study group identification for the purposes of performing the primary and secondary statistical analyses.

11.2 Timing of Analysis

The data collected will be analysed at the end of the study i.e. after the last subject/last visit has been completed and the data has been locked. An interim analysis will be undertaken for data obtained till 28 days after the third dose of the vaccine for each dose-level (cohort). The details of the same will be stated in the SAP.

11.3 Definition of Analysis Sets

The Intention-To-Treat (ITT) population data set and the Per Protocol (PP) population data set will be used for the summarisation and descriptive analysis of the safety (primary endpoints) and immunogenicity data (secondary endpoints) as follows:

11.3.1 Intention-To-Treat Population (ITT Population)

The ITT population will include all randomised subjects who received at least one dose of either of the assigned study vaccines. This population will be used for reactogenicity, overall safety and immunogenicity analysis.

11.3.2 Per Protocol Population (PP Population)

The "Per Protocol" (PP) set includes the subjects from the ITT set who are compliant with the protocol. The PP analysis set will include all subjects who:

1. Received the randomised vaccine via intramuscular route
2. Met all eligibility (inclusion/exclusion) criteria; and
3. Developed no premature discontinuation criteria during the follow-up visits post vaccination
4. Did not take any prohibited concomitant medication during the vaccine period (up to Day 208)

This population will be used for reactogenicity and immunogenicity analysis.

11.4 Assessment Endpoints

In Section 8 of this protocol, all clinical and biological endpoints are defined for evaluation of reactogenicity, safety, immunogenicity, at defined visits after the vaccination (assessments during the vaccination period and during the follow-up period). A detailed description of these variables will be provided in the SAP.

Information contained in this publication is the property of EVI and ICGEB and is confidential and will not be disclosed to third parties without written authorization from EVI and ICGEB. No part of this document may be reproduced, stored in a retrieval system or transmitted in any form or by any means -electronic, mechanical, recording or otherwise - without the prior authorization of EVI and ICGEB

11.5 Analysis Methods

11.5.1 Statistical Model, Hypothesis Testing and Tests

No statistical hypothesis can be set up for the safety and immunogenicity data as sample size is limited. The analysis shall be descriptive as the sample size does not allow any comparison between groups. Categorical Variables will be summarised by vaccine groups as frequency, percentages and 95% confidence interval. Continuous Variables other than titers and Concentrations will be summarised by vaccine groups as Mean, SEM, Median, Minimum, Maximum, inter-quartile range/range. The proportion of subjects that received three doses without experiencing grade 3 adverse events will be estimated by Exact Binomial proportion (Proportion and 95% confidence interval).

11.5.1.1 Analysis of Demographics and Other Baseline Characteristics

Demographic characteristics (age, race etc.) will be tabulated by vaccine group on the ITT data set. Quantitative demographic variable age will be described as number of subjects, mean, SEM, minimum, median, maximum, and range. Qualitative demographic variables will be described by number of subjects and percentage by each vaccine group. Group comparisons will be performed to confirm whether the vaccine groups are similar with regard to demographic characteristics. Clinical laboratory results will be tabulated by vaccine group using both quantitative (raw values) and qualitative parameters (relative positions regarding normal ranges in use in the laboratory). The investigator will check the biological parameters (haematology and serum chemistry) and the vital signs. The clinically significant abnormalities will be described. Concomitant diseases at the first visit will be tabulated by vaccine group using the latest version of MedDRA Adverse Event Dictionary.

11.5.1.2 Analysis of Reactogenicity

The primary analysis objectives are to assess the reactogenicity of vaccine administration. Primary analyses will evaluate immediate reactogenicity (every 30 minutes up to 3 hours post-vaccination) and delayed reactogenicity (from Day 0 and Day 14 based on solicited adverse events defined in section 7.2.2).

Binomial proportion for local, systemic reactions and solicited AEs will be calculated along with 95% confidence interval by vaccine group.

11.5.1.3 Analysis of Overall Safety

The safety analysis will include all randomised subjects who have received at least one dose of either of the assigned study vaccines. The overall percentage of subjects with at least one reported adverse event / serious adverse event, with date of onset up to 28 days after vaccination will be tabulated with 95% confidence intervals, by type of adverse event; by severity; and by causality. These events will be summarised and classified by System Organ Class and preferred term MedDRA. They will be displayed by vaccine group as both frequencies and percentages on the ITT data set. All reported adverse events that start post-vaccination would be tabulated. If a given disease is already reported as ongoing at the first visit on the medical history pages, it will be counted and tabulated as a vaccine emergent adverse event only if it worsens after the vaccination with the study vaccines. For each vaccine group vital sign parameters will be summarised visit wise as number of subjects, mean, SEM, median minimum, maximum and range. The changes in vital

Information contained in this publication is the property of EVI and ICGEB and is confidential and will not be disclosed to third parties without written authorization from EVI and ICGEB. No part of this document may be reproduced, stored in a retrieval system or transmitted in any form or by any means -electronic, mechanical, recording or otherwise - without the prior authorization of EVI and ICGEB

signs for every 30 minutes till 3 hours post-vaccination from pre-vaccination will be calculated and tabulated for each vaccine group.

The investigator will check the biological parameters (haematology and serum chemistry). Newly occurring or worsening lab abnormalities will be assessed and reported by the investigator as adverse events as applicable. Clinical laboratory results will be tabulated by vaccine group at each time point as both quantitative parameters (raw values) and qualitative parameters (relative positions regarding normal ranges in use in the laboratory). Serious adverse events and discontinuation due to adverse event(s) will be described in detail by vaccine group.

11.5.1.4 Analysis of Immunogenicity

Immunogenicity endpoints will be analysed at Day 28 and Day 56 compared to baseline (Day 0), and Day 56 compared to Day 28 to define the primary response to the vaccine, at Day 208 compared to Day 56 to describe the antibody levels in response to the booster dose and further at Day 180 compared to Day 56 to describe the antibody persistence of the primary vaccination, and Day 365 compared to Day 208 to describe the antibody persistence. GMTs and GMCs will be presented along with 95% Confidence Intervals.

A vaccine responder will be defined as a subject with sera reactivity by ELISA against both antigens (PfMSP-1_{19} and PfF2) greater than background reactivity (average plus three standard deviations) of pre-vaccinated sera **(17)**.

Information contained in this publication is the property of EVI and ICGEB and is confidential and will not be disclosed to third parties without written authorization from EVI and ICGEB. No part of this document may be reproduced, stored in a retrieval system or transmitted in any form or by any means -electronic, mechanical, recording or otherwise - without the prior authorization of EVI and ICGEB

12 REGULATORY AND ETHICAL REQUIREMENTS

This clinical study is designed and shall be implemented and reported in accordance with the ICH Harmonised Tripartite Guidelines for Good Clinical Practice (ICH-GCP), the ethical principles laid down in the Declaration of Helsinki and as per the applicable local regulations of the country where the study takes place.

12.1 Regulatory Authority Approval

Regulatory notification for this study will be sent to the responsible authority, the DCGI, according to the applicable country-specific laws and regulations of India. DiagnoSearch Life Sciences (DLS) will obtain the necessary approval from the DCGI on behalf of the Sponsor (EVI/ICGEB) prior to initiation of the study.

12.2 Institutional Review Boards/Ethics Committees

12.2.1 Institutional Review Board

The Sponsor's Institutional Review Board (ICGEB-IRB) will review and give approval to the present study protocol before its implementation by the study site.

12.2.2 Independent Ethics Committees

Study-related documents (including the protocol, subject information sheet, informed consent form or any other relevant document must be reviewed and approved by a properly constituted IRB/IEC/Research Ethics Board before study start. It is the investigator's responsibility to ensure that this protocol is reviewed and approved by the local IEC responsible for the study site. Where necessary, the sponsors will provide summaries of toxicological, clinical and pharmacological data to support this application. The IEC must also review and approve the Subject Information Sheet, Informed Consent Form and any other written information to be provided to the subject.

Written IEC approval shall be obtained and sent by the Investigator or his/her designee to the Sponsor prior to the start of the study. The IEC approval letter must identify all documents approved and list the study site, the study investigator, protocol number, date, version and title, Subject Information Sheet and Informed Consent Form number, date and version and the date of IEC approval. A list of IEC members shall be attached to the approval letter.

No deviations from, or changes to, the protocol shall be implemented without prior written IRB/IEC approvals of an appropriate amendment, except when necessary to eliminate immediate hazards to the subjects or when the change(s) involves only logistical or administrative aspects of the study (e.g., change of monitor(s), telephone number(s)). The investigator shall provide to the sponsors a statement from the IEC confirming the IEC is organised and operates according to ICH-GCP and applicable laws and regulations.

Prior to submitting to the IEC/IRB, the investigator is also required to sign a protocol signature page confirming his/her agreement to conduct the study in accordance with these documents and all of the instructions and procedures found in this protocol and to give access to all relevant data and records to monitors, auditors, Clinical Quality Assurance representatives, designated agents of, IRBs/IECs, and regulatory authorities as required. If an inspection of the clinical site is requested

Information contained in this publication is the property of EVI and ICGEB and is confidential and will not be disclosed to third parties without written authorization from EVI and ICGEB. No part of this document may be reproduced, stored in a retrieval system or transmitted in any form or by any means -electronic, mechanical, recording or otherwise - without the prior authorization of EVI and ICGEB

by a regulatory authority, the investigator must inform immediately that this request has been made.

12.3 Subject Information and Informed Consent

There are two (2) discreet informed consent documents for this study. Following the signing of the pre-screening informed consent form, the pre-screening blood sample for testing for anti-malarial antibodies will be drawn. Eligible subjects may only be included in the study after providing written (witnessed, where required by law or regulation), IRB/IEC-approved informed consent in a language understood by the subject. Following the signing of the main informed consent for the actual study participation, the relevant study related activities and screening procedures (i.e., all of the procedures described in the protocol) will be undertaken. The process of obtaining informed consent (both pre-screening and main consent) will be documented in the subject's source documents.

The Sponsor or its designee will provide the proposed informed consent forms that comply with the ICH-GCP guideline and regulatory requirements and are considered appropriate for this study to the study investigator in two separate documents. Any changes to the proposed consent form suggested by the investigator must be agreed to by the Sponsor before submission to the IRB/IEC, and a copy of the final approved version must be provided to the study monitor after IRB/IEC approval.

The investigator or his/her designee will inform the subject of all aspects pertaining to the subject's participation in the study. The process for obtaining subject informed consent will be in accordance with all applicable regulatory requirements. Prior to including any subject in the clinical study, his/her free and expressed informed consent must be obtained in writing. The written informed consent must be signed and dated by both the investigator and his/her designee and the subject prior to any study related procedure. The investigator or his/her designee will describe the study protocol to potential subjects. The Subject Information Sheet and the Informed Consent Form may be given to the subject or read to him. The investigator must give the subjects ample opportunity to inquire about details of the study and ask any questions before dating and signing the Informed Consent Form.

Original subject's signed informed consent forms must be kept on file by the investigator for possible inspection by regulatory authorities and Sponsor (or its designees). The subject must receive a copy of the signed and dated informed consent form, and any subsequent updates or amendments. The study monitor will check the documentation of the individual Informed Consent Form during each monitoring visit.

12.4 Subject Confidentiality

Subject names will not be supplied to the sponsor. The subject will be identified by the subject number in the CRF. If a subject's name appears on any other document (e.g. pathologist report), it will be obliterated before the copy of the document is supplied to the sponsor. Study findings stored on a computer will be subject to local data protection laws. The subject and his/her legally acceptable representative(s) will be informed that representatives of the sponsor, IEC or IRB, or regulatory authorities may inspect their medical records to verify the information collected and that all personal information made available for inspection will be handled in the strictest confidence. The investigator or designee will maintain a personal list of subject numbers and subject names to enable records to be found at a later date.

Information contained in this publication is the property of EVI and ICGEB and is confidential and will not be disclosed to third parties without written authorization from EVI and ICGEB. No part of this document may be reproduced, stored in a retrieval system or transmitted in any form or by any means -electronic, mechanical, recording or otherwise - without the prior authorization of EVI and ICGEB

12.5 Notification of Primary Care Physician

If agreed by the subject, the investigator shall notify the subject's primary care physician, if applicable, of the subject's participation in the study.

12.6 Record Retention

In accordance with the applicable regulatory requirements, following closure of the study, the investigator/institution will maintain a copy of all study records in a safe and designated location at the study site. The Sponsor or its designee will inform the investigator/institution of the time period for retaining these records in order to comply with applicable regulatory requirements. Essential documents (the subject screening and enrolment logs, subject files and other source data) shall be retained at the site for at least fifteen (15) years after the completion or discontinuation of the study, and (if longer) until at least two (2) years after the last approval of a marketing application in an ICH region and until there are no pending or contemplated marketing applications in an ICH region or at least two (2) years have elapsed since the formal discontinuation of clinical development of the IP (investigational product). The CRFs shall be retained at DLS for at least fifteen (15) years.

Information contained in this publication is the property of EVI and ICGEB and is confidential and will not be disclosed to third parties without written authorization from EVI and ICGEB. No part of this document may be reproduced, stored in a retrieval system or transmitted in any form or by any means -electronic, mechanical, recording or otherwise - without the prior authorization of EVI and ICGEB

13 ADMINISTRATIVE MATTERS

To comply with ICH-GCP important administrative obligations relating to investigator responsibilities, monitoring, archiving, audits, confidentially and publications must be fulfilled.

13.1 Protocol Amendments

Any significant change in the study protocol will be addressed in a written protocol amendment, which will be signed by the investigator(s) and the sponsor. It is the investigator's responsibility to submit protocol amendments to the relevant IEC and to obtain written approval where required. The approval letter, signed by the IEC chairman, must refer specifically to the investigator, the protocol number, the protocol title, the protocol amendment number, and the date of the protocol amendment. A protocol amendment may be implemented after it has been approved by all the IRBs/IECs and the regulatory authorities. In the case of a protocol change intended to eliminate an apparent immediate hazard to subjects, the change may be implemented immediately. In this case, the change must be documented in an amendment and reported to the IRB/IEC and Regulatory authorities as soon as possible. Amendments affecting only administrative aspects of the study may not require formal IEC /Regulatory Authority approval.

13.2 Financing and Insurance

All details pertaining to financing and insurance related to this study are provided in a separate contract between the Investigator and EVI/ICGEB or its designee.

13.3 Confidentiality and Publication

All proprietary or confidential information communicated to the investigator by or for EVI/ICGEB or its designee, or communicated to the investigator during the course of and/or as a result of the clinical study is the exclusive property of EVI/ICGEB or its designee and the investigator shall ensure that the same shall be kept strictly confidential by him/her and any other person connected with the clinical study and shall not be disclosed, either orally or in written form, by him/her or such person to any third party without the prior written consent of EVI/ICGEB or its designee. The investigator shall communicate the results of the clinical study promptly to EVI/ICGEB or its designee. All rights and interests worldwide in any inventions, know-how, or other intellectual or industrial property rights, which arise during the course of and/or as a result of the clinical study, which is the subject of this protocol or which otherwise arise from the information or materials supplied under this protocol, shall be assigned to, vest in and remain the property of EVI/ICGEB or its designee.

All information concerning the study vaccine supplied by EVI/ICGEB in connection with this study, and not previously published, is considered confidential and proprietary information. This information includes the Investigator's Brochure, the study protocol, CRFs, assay methods, the sponsor's technical methodology and basic scientific data. This confidential information shall remain the sole property of EVI/ICGEB, shall not be disclosed to others without prior written consent from EVI/ICGEB and shall not be used except in the performance of this study. The information developed during the conduct of this clinical study is also considered confidential and will be used by EVI/ICGEB in connection with the development of the study vaccine. To allow

Information contained in this publication is the property of EVI and ICGEB and is confidential and will not be disclosed to third parties without written authorization from EVI and ICGEB. No part of this document may be reproduced, stored in a retrieval system or transmitted in any form or by any means -electronic, mechanical, recording or otherwise - without the prior authorization of EVI and ICGEB

for the use of information derived from this clinical study and to ensure compliance to current regulations, the investigator is obliged to provide EVI/ICGEB with complete test results and all data developed in this study. An agreement on publications and authorship will be reached between the sponsors and the investigators before the study starts.

Information contained in this publication is the property of EVI and ICGEB and is confidential and will not be disclosed to third parties without written authorization from EVI and ICGEB. No part of this document may be reproduced, stored in a retrieval system or transmitted in any form or by any means -electronic, mechanical, recording or otherwise - without the prior authorization of EVI and ICGEB

14 REFERENCES

1) World Malaria Report 2008. http://www.who.int/malaria/wmr2008/

2) Pandey KC, Singh S, Pattnaik P, *et al.* Bacterially expressed and refolded receptor binding domain of *Plasmodium falciparum* EBA-175 elicits invasion inhibitory antibodies. Molecular and Biochemical Parasitology 2002; 123:23-33

3) Pattnaik P, Shakri AR, Singh S, *et al.* Immunogenicity of a recombinant malaria vaccine based on receptor binding domain of *Plasmodium falciparum* EBA-175. Vaccine 2007; 25:806-813

4) World Malaria Report 2005. http://www.rbm.who.int/wmr2005/

5) Malaria Vaccine. The Last word in Malaria Prevention - a Myth or a Reality? http://www.malariasite.com/malaria/malaria_vaccine.htm

6) Brown GV, Reeder JC. Malaria Vaccines. MJA 2002; 177(5):230-231. http://www.mja.com.au/public/issues/177_05_020902/bro10303_fm.html

7) Malaria Vaccine Development Unit, ICGEB. (DATA ON FILE, ICGEB).

8) Formulation of JAIVAC-1 antigens with adjuvant Montanide ISA 720. Malaria vaccine development unit, Standard Operating Procedure. (DATA ON FILE, ICGEB).

9) Prevalence of antibodies against Plasmodium falciparum antigens PfF2 (42 kDa receptor binding domain of P. falciparum EBA 175) and PfMSP-119 (19 kDA C-terminal fragment of P. falciparum Merozoite Surface Protein-1) in residents of Ahmedabad and Mumbai. (DATA ON FILE, ICGEB).

10) Rothstein E, Kohl KS, Ball L, *et al.* Nodule at injection site as an adverse event following immunization: case definition and guidelines for data collection, analysis, and presentation. Vaccine 2004; 22: 575–585

11) Stoute JA, Heppner GD, Mason CJ, et al. Phase 1 safety and immunogenicity trial of malaria vaccine RTS,S/AS02A in adults in a hyperendemic region of Western Kenya. Am. J. Trop. Med. Hyg 2006; 75(1):166-170

12) Kohl KS, Walop W, Gidudu J, *et al.* Induration at or near injection site: Case definition and guidelines for collection, analysis, and presentation of immunization safety data. Vaccine 2007; 25:5839–5857

13) Kohl KS, Walop W, Gidudu J, *et al.* Swelling at or near injection site: Case definition and guidelines for collection, analysis and presentation of immunization safety data. Vaccine 2007; 25:5858–5874

Information contained in this publication is the property of EVI and ICGEB and is confidential and will not be disclosed to third parties without written authorization from EVI and ICGEB. No part of this document may be reproduced, stored in a retrieval system or transmitted in any form or by any means -electronic, mechanical, recording or otherwise - without the prior authorization of EVI and ICGEB

14) Marcy M, Kohl K, Blum M, *et al*. Fever as an adverse event following immunisation. Case definition and guidelines of data collection, analysis, and presentation.1-12.13-3-2003. Brighton Collaboration Fever Working Group

15) Malkin E, Hu J, Li Z, *et al*.. A phase 1 trial of PfCP2.9: an AMA1/MSP1 chimeric recombinant protein vaccine for *Plasmodium falciparum* malaria. Vaccine. 2008; 26(52):6864-73.

16) Hermsen CC, Verhage DF, Telgt DS, *et al*.. Glutamate-rich protein (GLURP) induces antibodies that inhibit in vitro growth of *Plasmodium falciparum* in a phase 1 malaria vaccine trial.Vaccine. 2007; 25(15):2930-40.

17) Hu J, Chen Z, Gu J, *et al*. Safety and immunogenicity of a malaria vaccine, Plasmodium falciparum AMA-1/MSP-1 chimeric protein formulated in Montanide ISA 720 in healthy adults. PLoS One, 2008 Apr 9; 3(4):e1952.

Information contained in this publication is the property of EVI and ICGEB and is confidential and will not be disclosed to third parties without written authorization from EVI and ICGEB. No part of this document may be reproduced, stored in a retrieval system or transmitted in any form or by any means -electronic, mechanical, recording or otherwise - without the prior authorization of EVI and ICGEB

15 APPENDICES

APPENDIX A: WORLD MEDICAL ASSOCIATION DECLARATION OF HELSINKI

Ethical Principles for Medical Research Involving Human Subjects

Adopted by the 18th WMA General Assembly, Helsinki, Finland, June 1964, and amended by the:

29th WMA General Assembly, Tokyo, Japan, October 1975

35th WMA General Assembly, Venice, Italy, October 1983

41st WMA General Assembly, Hong Kong, September 1989

48th WMA General Assembly, Somerset West, Republic of South Africa, October 1996

52nd WMA General Assembly, Edinburgh, Scotland, October 2000

53th WMA General Assembly, Washington 2002 (Note of Clarification on paragraph 29 added)

55th WMA General Assembly, Tokyo 2004 (Note of Clarification on Paragraph 30 added)

59th WMA General Assembly, Seoul, October 2008

A. INTRODUCTION

1. The World Medical Association (WMA) has developed the Declaration of Helsinki as a statement of ethical principles for medical research involving human subjects, includingresearch on identifiable human material and data. The Declaration is intended to be read as a whole and each of its constituent paragraphs should not be applied without consideration of all other relevant paragraphs.

2. Although the Declaration is addressed primarily to physicians, the WMA encourages other participants in medical research involving human subjects to adopt these principles.

3. It is the duty of the physician to promote and safeguard the health of patients, including those who are involved in medical research. The physician's knowledge and conscience are dedicated to the fulfilment of this duty.

4. The Declaration of Geneva of the WMA binds the physician with the words, "The health of my patient will be my first consideration," and the International Code of Medical Ethics declares that, "A physician shall act in the patient's best interest when providing medical care."

Information contained in this publication is the property of EVI and ICGEB and is confidential and will not be disclosed to third parties without written authorization from EVI and ICGEB. No part of this document may be reproduced, stored in a retrieval system or transmitted in any form or by any means -electronic, mechanical, recording or otherwise - without the prior authorization of EVI and ICGEB

5. Medical progress is based on research that ultimately must include studies involving human subjects. Populations that are underrepresented in medical research should be provided appropriate access to participation in research.

6. In medical research involving human subjects, the well-being of the individual research subject must take precedence over all other interests.

7. The primary purpose of medical research involving human subjects is to understand the causes, development and effects of diseases and improve preventive, diagnostic and therapeutic interventions (methods, procedures and treatments). Even the best current interventions must be evaluated continually through research for their safety, effectiveness, efficiency, accessibility and quality.

8. In medical practice and in medical research, most interventions involve risks and burdens.

9. Medical research is subject to ethical standards that promote respect for all human subjects and protect their health and rights. Some research populations are particularly vulnerable and need special protection. These include those who cannot give or refuse consent for themselves and those who may be vulnerable to coercion or undue influence.

10. Physicians should consider the ethical, legal and regulatory norms and standards for research involving human subjects in their own countries as well as applicable international norms and standards. No national or international ethical, legal or regulatory requirement should reduce or eliminate any of the protections for research subjects set forth in this Declaration.

B. PRINCIPLES FOR ALL MEDICAL RESEARCH

11. It is the duty of physicians who participate in medical research to protect the life, health, dignity, integrity, right to self-determination, privacy, and confidentiality of personal information of research subjects.

12. Medical research involving human subjects must conform to generally accepted scientific principles, be based on a thorough knowledge of the scientific literature, other relevant sources of information, and adequate laboratory and, as appropriate, animal experimentation. The welfare of animals used for research must be respected.

13. Appropriate caution must be exercised in the conduct of medical research that may harm the environment.

Information contained in this publication is the property of EVI and ICGEB and is confidential and will not be disclosed to third parties without written authorization from EVI and ICGEB. No part of this document may be reproduced, stored in a retrieval system or transmitted in any form or by any means -electronic, mechanical, recording or otherwise - without the prior authorization of EVI and ICGEB

14. The design and performance of each research study involving human subjects must be clearly described in a research protocol. The protocol should contain a statement of the ethical considerations involved and should indicate how the principles in this Declaration have been addressed. The protocol should include information regarding funding, sponsors, institutional affiliations, other potential conflicts of interest, incentives for subjects and provisions for treating and/or compensating subjects who are harmed as a consequence of participation in the research study. The protocol should describe arrangements for post-study access by study subjects to interventions identified as beneficial in the study or access to other appropriate care or benefits.

15. The research protocol must be submitted for consideration, comment, guidance and approval to a research ethics committee before the study begins. This committee must be independent of the researcher, the sponsor and any other undue influence. It must take into consideration the laws and regulations of the country or countries in which the research is to be performed as well as applicable international norms and standards but these must not be allowed to reduce or eliminate any of the protections for research subjects set forth in this Declaration. The committee must have the right to monitor ongoing studies. The researcher must provide monitoring information to the committee, especially information about any serious adverse events. No change to the protocol may be made without consideration and approval by the committee.

16. Medical research involving human subjects must be conducted only by individuals with the appropriate scientific training and qualifications. Research on patients or healthy volunteers requires the supervision of a competent and appropriately qualified physician or other health care professional. The responsibility for the protection of research subjects must always rest with the physician or other health care professional and never the research subjects, even though they have given consent.

17. Medical research involving a disadvantaged or vulnerable population or community is only justified if the research is responsive to the health needs and priorities of this population or community and if there is a reasonable likelihood that this population or community stands to benefit from the results of the research.

18. Every medical research study involving human subjects must be preceded by careful

assessment of predictable risks and burdens to the individuals and communities involved in the research in comparison with foreseeable benefits to them and to other individuals or communities affected by the condition under investigation.

19. Every clinical trial must be registered in a publicly accessible database before recruitment of the first subject.

20. Physicians may not participate in a research study involving human subjects unless they are confident that the risks involved have been adequately assessed and can be satisfactorily managed.

Information contained in this publication is the property of EVI and ICGEB and is confidential and will not be disclosed to third parties without written authorization from EVI and ICGEB. No part of this document may be reproduced, stored in a retrieval system or transmitted in any form or by any means -electronic, mechanical, recording or otherwise - without the prior authorization of EVI and ICGEB

Physicians must immediately stop a study when the risks are found to outweigh the potential benefits or when there is conclusive proof of positive and beneficial results.

21. Medical research involving human subjects may only be conducted if the importance of the objective outweighs the inherent risks and burdens to the research subjects.

22. Participation by competent individuals as subjects in medical research must be voluntary. Although it may be appropriate to consult family members or community leaders, no competent individual may be enrolled in a research study unless he or she freely agrees.

23. Every precaution must be taken to protect the privacy of research subjects and the confidentiality of their personal information and to minimize the impact of the study on their physical, mental and social integrity.

24. In medical research involving competent human subjects, each potential subject must be adequately informed of the aims, methods, sources of funding, any possible conflicts of interest, institutional affiliations of the researcher, the anticipated benefits and potential risks of the study and the discomfort it may entail, and any other relevant aspects of the study. The potential subject must be informed of the right to refuse to participate in the study or to withdraw consent to participate at any time without reprisal. Special attention should be given to the specific information needs of individual potential subjects as well as to the methods used to deliver the information. After ensuring that the potential subject has understood the information, the physician or another appropriately qualified individual must then seek the potential subject's freely-given informed consent, preferably in writing. If the consent cannot be expressed in writing, the non-written consent must be formally documented and witnessed.

25. For medical research using identifiable human material or data, physicians must normally seek consent for the collection, analysis, storage and/or reuse. There may be situations where consent would be impossible or impractical to obtain for such research or would pose a threat to the validity of the research. In such situations the research may be done only after consideration and approval of a research ethics committee.

26. When seeking informed consent for participation in a research study the physician should be particularly cautious if the potential subject is in a dependent relationship with the physician or may consent under duress. In such situations the informed consent should be sought by an appropriately qualified individual who is completely independent of this relationship.

27. For a potential research subject who is incompetent, the physician must seek informed consent from the legally authorized representative. These individuals must not be included in a research study that has no likelihood of benefit for them unless it is intended to promote the health of the

Information contained in this publication is the property of EVI and ICGEB and is confidential and will not be disclosed to third parties without written authorization from EVI and ICGEB. No part of this document may be reproduced, stored in a retrieval system or transmitted in any form or by any means -electronic, mechanical, recording or otherwise - without the prior authorization of EVI and ICGEB

population represented by the potential subject, the research cannot instead be performed with competent persons, and the research entails only minimal risk and minimal burden.

28. When a potential research subject who is deemed incompetent is able to give assent to

decisions about participation in research, the physician must seek that assent in addition to the consent of the legally authorized representative. The potential subject's dissent should be respected.

29. Research involving subjects who are physically or mentally incapable of giving consent, for example, unconscious patients, may be done only if the physical or mental condition that prevents giving informed consent is a necessary characteristic of the research population. In such circumstances the physician should seek informed consent from the legally authorized representative. If no such representative is available and if the research cannot be delayed, the study may proceed without informed consent provided that the specific reasons for involving subjects with a condition that renders them unable to give informed consent have been stated in the research protocol and the study has been approved by a research ethics committee. Consent to remain in the research should be obtained as soon as possible from the subject or a legally authorized representative.

30. Authors, editors and publishers all have ethical obligations with regard to the publication of the results of research. Authors have a duty to make publicly available the results of their research on human subjects and are accountable for the completeness and accuracy of their reports. They should adhere to accepted guidelines for ethical reporting. Negative and inconclusive as well as positive results should be published or otherwise made publicly available. Sources of funding, institutional affiliations and conflicts of interest should be declared in the publication. Reports of research not in accordance with the principles of this Declaration should not be accepted for publication.

C. ADDITIONAL PRINCIPLES FOR MEDICAL RESEARCH COMBINED WITH MEDICAL CARE

31. The physician may combine medical research with medical care only to the extent that the research is justified by its potential preventive, diagnostic or therapeutic value and if the physician has good reason to believe that participation in the research study will not adversely affect the health of the patients who serve as research subjects.

32. The benefits, risks, burdens and effectiveness of a new intervention must be tested against those of the best current proven intervention, except in the following circumstances:

• The use of placebo, or no treatment, is acceptable in studies where no current proven intervention exists; or

Information contained in this publication is the property of EVI and ICGEB and is confidential and will not be disclosed to third parties without written authorization from EVI and ICGEB. No part of this document may be reproduced, stored in a retrieval system or transmitted in any form or by any means -electronic, mechanical, recording or otherwise - without the prior authorization of EVI and ICGEB

• Where for compelling and scientifically sound methodological reasons the use of placebo is necessary to determine the efficacy or safety of an intervention and the patients who receive placebo or no treatment will not be subject to any risk of serious or irreversible harm. Extreme care must be taken to avoid abuse of this option.

33. At the conclusion of the study, patients entered into the study are entitled to be informed about the outcome of the study and to share any benefits that result from it, for example, access to interventions identified as beneficial in the study or to other appropriate care or benefits.

34. The physician must fully inform the patient which aspects of the care are related to the research. The refusal of a patient to participate in a study or the patient's decision to withdraw from the study must never interfere with the patient-physician relationship.

35. In the treatment of a patient, where proven interventions do not exist or have been ineffective, the physician, after seeking expert advice, with informed consent from the patient or a legally authorized representative, may use an unproven intervention if in the physician's judgement it offers hope of saving life, re-establishing health or alleviating suffering. Where possible, this intervention should be made the object of research, designed to evaluate its safety and efficacy. In all cases, new information should be recorded and, where appropriate, made publicly available.

Information contained in this publication is the property of EVI and ICGEB and is confidential and will not be disclosed to third parties without written authorization from EVI and ICGEB. No part of this document may be reproduced, stored in a retrieval system or transmitted in any form or by any means -electronic, mechanical, recording or otherwise - without the prior authorization of EVI and ICGEB

APPENDIX B: JAIVAC_1_1_09 LABORATORY REFERENCE RANGES

JAIVAC-1_1_09 Laboratory Reference Ranges

Sr #	Analyte	Age Limit				Sex	Units	Ref Lo	Ref Hi
		Age Low	Age Low unit	Age High	Age High Unit				
**Haematology**									
1	Haematocrit	13	y	999	y	Males	%	40.0	54.0
2	Haemoglobin	13	y	999	y	Males	gm / dl	13.5	18.0
3	RBC Count	13	y	999	y	Males	10E6/ul	4.60	6.20
4	MCV	13	y	999	y	Males	fL	**80.0**	**96.0**
5	MCH	13	y	999	y	Males	pg	**27.0**	**31.0**
6	MCHC	13	y	999	y	Males	gm%	**32.0**	**36.0**
7	WBC count	13	y	999	y	Males	10E3/ul	**4.50**	**11.00**
8	% Neutrophils	13	y	999	y	Males	%	**40.0**	**75.0**
9	% Basophils	13	y	999	y	Males	%	**0.0**	**1.0**
10	% Lymphocytes	13	y	999	y	Males	%	**20.0**	**45.0**
11	% Monocytes	13	y	999	y	Males	%	**2.0**	**10.0**
12	% Eosinophils	13	y	999	y	Males	%	**1.0**	**6.0**
13	Platelet count	1	y	999	y	Males	10E3/ul	**150**	**400**
**Serum Chemistry**									
14	Sodium	13	y	999	y	Males	mEq / L	**136**	**145**
15	Potassium	13	y	999	y	Males	mEq / L	**3.5**	**5.1**
16	Random Blood Glucose	1	y	999	y	Males	mg /dl	**45**	**130**
17	Creatinine	16	y	999	y	Males	mg / dl	**0.7**	**1.2**
18	Bilirubin, Total	1	y	999	y	Males	mg / dl	**0.00**	**0.99**
19	Bilirubin, Indirect	1	y	999	y	Males	mg / dl	**0.00**	**0.99**
20	Bilirubin, Direct	1	y	999	y	Males	mg / dl	**0.00**	**0.30**
21	SGOT (AST)	0	y	999	y	Males	U / L	**0**	**40**
22	SGPT (ALT)	0	y	999	y	Males	U / L	**0**	**41**
23	γ-GT	1	y	999	y	Males	U / L	**10**	**71**
24	Alkaline Phosphatase	18	y	999	y	Males	U / L	**40**	**129**
**Serology**									
25	Anti HCV	0	y	999	y	Males	None	Negative	
26	HbsAg	0	y	999	y	Males	None	Negative	
27	Anti-HIV	0	y	999	y	Males	None	Negative	

Information contained in this publication is the property of EVI and ICGEB and is confidential and will not be disclosed to third parties without written authorization from EVI and ICGEB. No part of this document may be reproduced, stored in a retrieval system or transmitted in any form or by any means -electronic, mechanical, recording or otherwise - without the prior authorization of EVI and ICGEB

APPENDIX B-1: JAIVAC_1_1_09 ACCEPTABLE LIMITS FOR 'OUT OF RANGE' LABORATORY PARAMETER(S) AT SCREENING/BASELINE

Sr #	Analyte	Units	Ref Lo	Ref Hi
**Haematology**				
1	Haematocrit	%	**If outside range as per Appendix B, then the value is to be co-related with other relevant lab parameters**	
2	Haemoglobin	gm / dl	**13.0**	**18.0**
3	RBC Count	10E6/ul	**4.0**	**7.0**
4	MCV	fL	**If outside range as per Appendix B, then the value is to be co-related with other relevant lab parameters**	
5	MCH	pg	**If outside range as per Appendix B, then the value is to be co-related with other relevant lab parameters**	
6	MCHC	gm%	**If outside range as per Appendix B, then the value is to be co-related with other relevant lab parameters**	
7	WBC count	10E3/ul	**4.0**	**11.0**
8	% Neutrophils	%	**40.0**	**75.0**
9	% Basophils	%	**If outside range as per Appendix B, then the value is to be co-related with other relevant lab parameters**	
10	% Lymphocytes	%	**If outside range as per Appendix B, then the value is to be co-related with other relevant lab parameters**	
11	% Monocytes	%	**If outside range as per Appendix B, then the value is to be co-related with other relevant lab parameters**	
12	% Eosinophils	%	**0.0**	**10.0**
13	Platelet count	10E3/ul	**150**	**500**
**Serum Chemistry**				
14	Creatinine	mg / dl	**0.0**	**1.2**
15	Bilirubin, Total	mg / dl	**0.0**	**1.2**
16	Bilirubin, Indirect	mg / dl	**0.0**	**1.2**
17	Bilirubin, Direct	mg / dl	**0.0**	**0.4**
18	γ-GT	U / L	**0**	**71**

Information contained in this publication is the property of EVI and ICGEB and is confidential and will not be disclosed to third parties without written authorization from EVI and ICGEB. No part of this document may be reproduced, stored in a retrieval system or transmitted in any form or by any means -electronic, mechanical, recording or otherwise - without the prior authorization of EVI and ICGEB

APPENDIX C: SERIOUS ADVERSE EVENT MANAGEMENT FLOWCHART

Occurrence of a Serious Adverse Event (SAE)

Site becomes aware of the occurrence of the SAE

Within 24 hours

Call site CRA/Sponsor immediately & e-mail/fax the SAE Report Form to DiagnoSearch LifeSciences/ICGEB/EVI
ATTN: Dr. Supriya Desai /Dr. Gandhali Paranjape /Dr. Bhupendra Badhe
Fax: +91 22 6692 5344 /6675 4090
Tel: +91 22 6777 6390/ 6777 6309 / 6777 6330
Cell: +91 99207 51248/ 98332 29010 /9920023320
E-mail:safety@diagnosearch.com

Minimum information required to be filled in the SAE Form (Initial Report):

- Identifiable Patient (protocol ID, site ID, subject ID, age at the time of onset of AE, gender)
- Identifiable Reporter (the first person, who becomes aware of the occurrence of the event)
- Reaction/Adverse Event (AE term, seriousness criteria e.g., hospitalisation)
- Suspect Vaccine (study /control vaccine, relatedness to vaccine (if available)
- Other information may include start date of AE, grade of the AE and description of AE (as per availability at the time of reporting)

Report to the ICGEB IRB by ICGEB Representative within 7 working days

Follow up by DiagnoSearch Safety till the event is closed

Report to the IEC by Site within 7 working days

Obtain and report the following information as per updates:

- Investigator's opinion on relatedness to the test vaccine
- Action taken with the study vaccine, if any - temporary interruption or permanent discontinuation
- Relevant tests/ laboratory data if any
- Associated illness and symptoms
- Relevant history including preexisting medical conditions
- Concomitant medications during event
- Outcome of the event
- Any other significant findings

Closure of the SAE (following receipt of the Final Follow-up SAE Report Form)

- Keep copies of all SAE Report Forms (Initial, Follow-up till Final)
- Keep copies of all the SAE communications (including fax and e-mail)
- Capture all relevant data in source documents (as applicable)
  - Hospital discharge summary
  - Laboratory reports, 12-lead ECG report, X-Ray chest reports, any other tests
  - Death certificate / Autopsy report

Information contained in this publication is the property of EVI and ICGEB and is confidential and will not be disclosed to third parties without written authorization from EVI and ICGEB. No part of this document may be reproduced, stored in a retrieval system or transmitted in any form or by any means -electronic, mechanical, recording or otherwise - without the prior authorization of EVI and ICGEB

APPENDIX D: PACKAGE INSERT FOR THE CONTROL VACCINE - GENE VAC-B™ (RECOMBINANT HEPATITIS - B VACCINE, I.P.)

Information contained in this publication is the property of EVI and ICGEB and is confidential and will not be disclosed to third parties without written authorization from EVI and ICGEB. No part of this document may be reproduced, stored in a retrieval system or transmitted in any form or by any means -electronic, mechanical, recording or otherwise - without the prior authorization of EVI and ICGEB
