# Supplementary material for: Phase I Clinical Trial of a Recombinant Blood Stage Vaccine Candidate for Plasmodium falciparum Malaria Based on MSP1 and EBA175
Source: PLoS One. 2015 Apr 30;10(4):e0117820. doi: 10.1371/journal.pone.0117820 (PMC4415778; doi:10.1371/journal.pone.0117820)
Supplement: S1 Subject Diary Card — (PDF) [file pone.0117820.s006.pdf]

SUBJECT DIARY CARD

Status Page

**Type of document**	Project specific document	**Identifier**	JAIVAC-1_1_09
**Effective date**	16/02/2010	**Version number**	Final
**Review due date**	Not Applicable	**Supersedes version**	None

Name	Designation	Signature and date
Shashikanth Rai<br>Name (Author)	Clinical Data Associate, DiagnoSearch Life Sciences Pvt. Ltd<br>Designation	12/02/2010<br>Signature and date
Vikas Bhavsar<br>Name (Author)	Clinical Data Associate, DiagnoSearch Life Sciences Pvt. Ltd.<br>Designation	12/02/2010.<br>Signature and date
Shilpa Kulkarni<br>Reviewed by: Name	Senior Data Validator , DiagnoSearch Life Sciences Pvt. Ltd<br>Designation	12/02/2010<br>Signature and date
Nathalie Imbault<br>Approved by: Name	Clinical Operation Manager, European Vaccine Initiative<br>Designation	16/02/10<br>Signature and date
Dr. Shantanu Mehta<br>Approved by: Name	Manager- Clinical Research, Malaria Vaccine Development Program<br>Designation	12/02/2010<br>Signature and date
Shikha Dhawan<br>Approved by: Name	Project Manager, Malaria Vaccines, International Centre for Genetic Engineering and Biotechnology<br>Designation	[illegible]<br>Signature and date

Revision History

Version: ---	SOP ID: ---	Document Name: ---
Reason for change from last version: ---		

JAIVAC – 1_1_09

A Phase I, Randomised, Controlled, Dose-escalating, Single-blind Clinical Trial to Evaluate the Safety and Immunogenicity of the JAIVAC-1 Vaccine (PfMSP-119 and PfF2) formulated with Montanide ISA 720 in healthy Indian male subjects between 18 to 45 years of age.

Subject Diary Card ☐

Visit ☐ to Visit ☐

Site Number	0	1		
Screening Number	S			
Randomization Number				

GENERAL INSTRUCTIONS

- Enter data using ball point pen. DO NOT write with pencil.
- The study coordinator will complete the cover page and will also fill the screening number on top of each page from pages 1 to 23 before handing over the diary card to the subject.
- DO NOT overwrite on existing data. If there is any incorrect entry, cancel it with a single line, sign and date it and enter the correct value above the cancelled data.
- DO NOT erase or use correction fluid or make corrections over existing data.
- All dates must be entered in DD/MM/YYYY format. e.g. 04/02/2010
- The diary should be entered every evening at a consistent time.

DIARY INSTRUCTIONS

- The Diary comprises of three tables: Table 1, Table 2 and Table 3.
- Table 1 needs to be entered daily (from page 3 to 17) while Table 2 (from page 19 to 20) and Table 3 (from page 22 to 23) can be entered to capture details of Any other signs or symptoms and Medication respectively, if any, is required, at particular days.
- Check one box with [X] : either Yes or No
- If Yes, only then check one of the response 1, 2 or 3 as applicable and mentioned below:

TABLE 1: (from page 3 to 17)

Date

- The study coordinator will enter the dates corresponding to each “Day” on the top of each page (from page no. 3 to 17), before handing over the diary cards to the subject.

Pain at injection site

1	Painful on touch
2	Painful when limb is moved
3	Spontaneously painful or painful at rest

Swelling or Redness or Induration at injection site

Measure the maximum diameter (in millimeter) with the scale provided

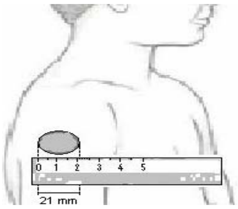

Limitation of arm motion abduction at shoulder

1	Angle of voluntary arm abduction is > 90° but < 120°
2	Angle of voluntary arm abduction is > 30° but < 90°
3	Angle of voluntary arm abduction is ≤ 30°

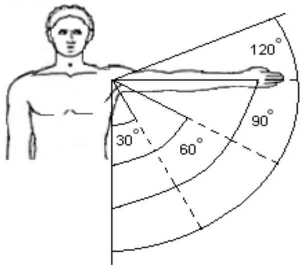

Axillary temperature

Measure the Axillary temperature (in Degree Celsius) with the thermometer provided, every day (at the same time in the evening) and write the value in the table

Headache or Malaise or Muscle pain (Myalgia) **or Joint Pain without swelling** (Arthralgia) **or Nausea or Vomiting**

1	Present, but easily tolerated
2	Discomforting enough to interfere with normal activities
3	Disabling, prevents normal daily activity, requires bed rest and treatment

Any other signs or symptoms?

Yes must be checked if any signs or symptoms (other than the ones listed in Table 1) are observed. The details of the same must be entered in Table 2(from page 19 to 20).

Any medication taken?

Yes must be checked if any medication is taken during the day and details of the same must be entered in Table 3(from page 22 to 23).

Table 1	DAY 0	Date (DD/MM/YYYY)	__ __/__ __/2 0 __ __

**Pain at injection site***	☐ Yes ☐ No	1 2 3
	*(1: Painful on touch, 2:Painful when limb is moved; 3: Painful at Rest)	
**Swelling at injection site**	☐ Yes ☐ No	______ mm
**Redness at injection site**	☐ Yes ☐ No	______ mm
**Induration** (Hardness) **at injection site**	☐ Yes ☐ No	______ mm
**Limitation of arm motion abduction at shoulder** (moving the upper arm up to the side away from the body)	☐ Yes ☐ No	1 2 3
**Axillary Temperature**	_______°C	
**Headache***	☐ Yes ☐ No	1 2 3
**Feeling of general discomfort or uneasiness** (Malaise)*	☐ Yes ☐ No	1 2 3
**Muscle pain** (Myalgia) *	☐ Yes ☐ No	1 2 3
**Joint Pain without swelling** (Arthralgia) *	☐ Yes ☐ No	1 2 3
**Discomfort in the stomach with an urge to vomit** (Nausea)*	☐ Yes ☐ No	1 2 3
**Vomiting***	☐ Yes ☐ No	1 2 3
***(1: Present, but easily tolerated, 2: Discomforting enough to interfere with normal activities; 3: Disabling, prevents normal activities, requires bed rest and treatment)**		
**Any other signs or symptoms?**	☐ Yes ☐ No	If Yes, then complete the details in Table 2
**Any Medication taken?**	☐ Yes ☐ No	If Yes, then complete the details in Table 3

Table 1	DAY 1	Date (DD/MM/YYYY)	__ __/__ __/20__ __

**Pain at injection site***	☐ Yes ☐ No	1 2 3
	*(1: Painful on touch, 2:Painful when limb is moved; 3: Painful at Rest)	
**Swelling at injection site**	☐ Yes ☐ No	______ mm
**Redness at injection site**	☐ Yes ☐ No	______ mm
**Induration** (Hardness) **at injection site**	☐ Yes ☐ No	______ mm
**Limitation of arm motion abduction at shoulder** (moving the upper arm up to the side away from the body)	☐ Yes ☐ No	1 2 3
**Axillary Temperature**	_______°C	
**Headache***	☐ Yes ☐ No	1 2 3
**Feeling of general discomfort or uneasiness** (Malaise)*	☐ Yes ☐ No	1 2 3
**Muscle pain** (Myalgia) *	☐ Yes ☐ No	1 2 3
**Joint Pain without swelling** (Arthralgia) *	☐ Yes ☐ No	1 2 3
**Discomfort in the stomach with an urge to vomit** (Nausea)*	☐ Yes ☐ No	1 2 3
**Vomiting***	☐ Yes ☐ No	1 2 3
***(1: Present, but easily tolerated, 2: Discomforting enough to interfere with normal activities; 3: Disabling, prevents normal activities, requires bed rest and treatment)**		
**Any other signs or symptoms?**	☐ Yes ☐ No	If Yes, then complete the details in Table 2
**Any Medication taken?**	☐ Yes ☐ No	If Yes, then complete the details in Table 3

Table 1	DAY 2	Date (DD/MM/YYYY)	__ __ / __ __ / 2 0 __ __

**Pain at injection site***	☐ Yes ☐ No	1 2 3
	***(1: Painful on touch, 2:Painful when limb is moved; 3: Painful at Rest)**	
**Swelling at injection site**	☐ Yes ☐ No	______ mm
**Redness at injection site**	☐ Yes ☐ No	______ mm
**Induration** (Hardness) **at injection site**	☐ Yes ☐ No	______ mm
**Limitation of arm motion abduction at shoulder** (moving the upper arm up to the side away from the body)	☐ Yes ☐ No	1 2 3
**Axillary Temperature**	________°C	
**Headache***	☐ Yes ☐ No	1 2 3
**Feeling of general discomfort or uneasiness** (Malaise)*	☐ Yes ☐ No	1 2 3
**Muscle pain** (Myalgia) *	☐ Yes ☐ No	1 2 3
**Joint Pain without swelling** (Arthralgia) *	☐ Yes ☐ No	1 2 3
**Discomfort in the stomach with an urge to vomit** (Nausea)*	☐ Yes ☐ No	1 2 3
**Vomiting***	☐ Yes ☐ No	1 2 3
***(1: Present, but easily tolerated, 2: Discomforting enough to interfere with normal activities; 3: Disabling, prevents normal activities, requires bed rest and treatment)**		
**Any other signs or symptoms?**	☐ Yes ☐ No	If Yes, then complete the details in Table 2
**Any Medication taken?**	☐ Yes ☐ No	If Yes, then complete the details in Table 3

Table 1	DAY 3	Date (DD/MM/YYYY)	__ __/__ __/2 0 __ __

**Pain at injection site***	☐ Yes ☐ No	1 2 3
	*(1: Painful on touch, 2:Painful when limb is moved; 3: Painful at Rest)	
**Swelling at injection site**	☐ Yes ☐ No	______ mm
**Redness at injection site**	☐ Yes ☐ No	______ mm
**Induration** (Hardness) **at injection site**	☐ Yes ☐ No	______ mm
**Limitation of arm motion abduction at shoulder** (moving the upper arm up to the side away from the body)	☐ Yes ☐ No	1 2 3
**Axillary Temperature**	_______°C	
**Headache***	☐ Yes ☐ No	1 2 3
**Feeling of general discomfort or uneasiness** (Malaise)*	☐ Yes ☐ No	1 2 3
**Muscle pain** (Myalgia) *	☐ Yes ☐ No	1 2 3
**Joint Pain without swelling** (Arthralgia) *	☐ Yes ☐ No	1 2 3
**Discomfort in the stomach with an urge to vomit** (Nausea)*	☐ Yes ☐ No	1 2 3
**Vomiting***	☐ Yes ☐ No	1 2 3
***(1: Present, but easily tolerated, 2: Discomforting enough to interfere with normal activities; 3: Disabling, prevents normal activities, requires bed rest and treatment)**		
**Any other signs or symptoms?**	☐ Yes ☐ No	If Yes, then complete the details in Table 2
**Any Medication taken?**	☐ Yes ☐ No	If Yes, then complete the details in Table 3

Table 1	DAY 4	Date (DD/MM/YYYY)	__ __/__ __/20__ __

**Pain at injection site***	☐ Yes ☐ No	1 2 3
	*(1: Painful on touch, 2:Painful when limb is moved; 3: Painful at Rest)	
**Swelling at injection site**	☐ Yes ☐ No	_____ mm
**Redness at injection site**	☐ Yes ☐ No	_____ mm
**Induration** (Hardness) **at injection site**	☐ Yes ☐ No	_____ mm
**Limitation of arm motion abduction at shoulder** (moving the upper arm up to the side away from the body)	☐ Yes ☐ No	1 2 3
**Axillary Temperature**	______°C	
**Headache***	☐ Yes ☐ No	1 2 3
**Feeling of general discomfort or uneasiness** (Malaise)*	☐ Yes ☐ No	1 2 3
**Muscle pain** (Myalgia) *	☐ Yes ☐ No	1 2 3
**Joint Pain without swelling** (Arthralgia) *	☐ Yes ☐ No	1 2 3
**Discomfort in the stomach with an urge to vomit** (Nausea)*	☐ Yes ☐ No	1 2 3
**Vomiting***	☐ Yes ☐ No	1 2 3
***(1: Present, but easily tolerated, 2: Discomforting enough to interfere with normal activities; 3: Disabling, prevents normal activities, requires bed rest and treatment)**		
**Any other signs or symptoms?**	☐ Yes ☐ No	If Yes, then complete the details in Table 2
**Any Medication taken?**	☐ Yes ☐ No	If Yes, then complete the details in Table 3

Table 1	DAY 5	Date (DD/MM/YYYY)	__ __/__ __/2 0 __ __

**Pain at injection site***	☐ Yes ☐ No	1 2 3
	*(1: Painful on touch, 2:Painful when limb is moved; 3: Painful at Rest)	
**Swelling at injection site**	☐ Yes ☐ No	_____ mm
**Redness at injection site**	☐ Yes ☐ No	_____ mm
**Induration** (Hardness) **at injection site**	☐ Yes ☐ No	_____ mm
**Limitation of arm motion abduction at shoulder** (moving the upper arm up to the side away from the body)	☐ Yes ☐ No	1 2 3
**Axillary Temperature**	______°C	
**Headache***	☐ Yes ☐ No	1 2 3
**Feeling of general discomfort or uneasiness** (Malaise)*	☐ Yes ☐ No	1 2 3
**Muscle pain** (Myalgia) *	☐ Yes ☐ No	1 2 3
**Joint Pain without swelling** (Arthralgia) *	☐ Yes ☐ No	1 2 3
**Discomfort in the stomach with an urge to vomit** (Nausea)*	☐ Yes ☐ No	1 2 3
**Vomiting***	☐ Yes ☐ No	1 2 3
***(1: Present, but easily tolerated, 2: Discomforting enough to interfere with normal activities; 3: Disabling, prevents normal activities, requires bed rest and treatment)**		
**Any other signs or symptoms?**	☐ Yes ☐ No	If Yes, then complete the details in Table 2
**Any Medication taken?**	☐ Yes ☐ No	If Yes, then complete the details in Table 3

Table 1	DAY 6	Date (DD/MM/YYYY)	__ __ / __ __ / 2 0 __ __

**Pain at injection site***	☐ Yes ☐ No	1 2 3
	***(1: Painful on touch, 2:Painful when limb is moved; 3: Painful at Rest)**	
**Swelling at injection site**	☐ Yes ☐ No	______ mm
**Redness at injection site**	☐ Yes ☐ No	______ mm
**Induration** (Hardness) **at injection site**	☐ Yes ☐ No	______ mm
**Limitation of arm motion abduction at shoulder** (moving the upper arm up to the side away from the body)	☐ Yes ☐ No	1 2 3
**Axillary Temperature**	________°C	
**Headache***	☐ Yes ☐ No	1 2 3
**Feeling of general discomfort or uneasiness** (Malaise)*	☐ Yes ☐ No	1 2 3
**Muscle pain** (Myalgia) *	☐ Yes ☐ No	1 2 3
**Joint Pain without swelling** (Arthralgia) *	☐ Yes ☐ No	1 2 3
**Discomfort in the stomach with an urge to vomit** (Nausea)*	☐ Yes ☐ No	1 2 3
**Vomiting***	☐ Yes ☐ No	1 2 3
***(1: Present, but easily tolerated, 2: Discomforting enough to interfere with normal activities; 3: Disabling, prevents normal activities, requires bed rest and treatment)**		
**Any other signs or symptoms?**	☐ Yes ☐ No	If Yes, then complete the details in Table 2
**Any Medication taken?**	☐ Yes ☐ No	If Yes, then complete the details in Table 3

Table 1	DAY 7	Date (DD/MM/YYYY)	__ __/__ __/2 0 __ __

**Pain at injection site***	☐ Yes ☐ No	1 2 3
	*(1: Painful on touch, 2:Painful when limb is moved; 3: Painful at Rest)	
**Swelling at injection site**	☐ Yes ☐ No	_____ mm
**Redness at injection site**	☐ Yes ☐ No	_____ mm
**Induration** (Hardness) **at injection site**	☐ Yes ☐ No	_____ mm
**Limitation of arm motion abduction at shoulder** (moving the upper arm up to the side away from the body)	☐ Yes ☐ No	1 2 3
**Axillary Temperature**	_______°C	
**Headache***	☐ Yes ☐ No	1 2 3
**Feeling of general discomfort or uneasiness** (Malaise)*	☐ Yes ☐ No	1 2 3
**Muscle pain** (Myalgia) *	☐ Yes ☐ No	1 2 3
**Joint Pain without swelling** (Arthralgia) *	☐ Yes ☐ No	1 2 3
**Discomfort in the stomach with an urge to vomit** (Nausea)*	☐ Yes ☐ No	1 2 3
**Vomiting***	☐ Yes ☐ No	1 2 3
***(1: Present, but easily tolerated, 2: Discomforting enough to interfere with normal activities; 3: Disabling, prevents normal activities, requires bed rest and treatment)**		
**Any other signs or symptoms?**	☐ Yes ☐ No	If Yes, then complete the details in Table 2
**Any Medication taken?**	☐ Yes ☐ No	If Yes, then complete the details in Table 3

Table 1	DAY 8	Date (DD/MM/YYYY)	__ __ / __ __ / 2 0 __ __

**Pain at injection site***	☐ Yes ☐ No	1 2 3
	*(1: Painful on touch, 2:Painful when limb is moved; 3: Painful at Rest)	
**Swelling at injection site**	☐ Yes ☐ No	______ mm
**Redness at injection site**	☐ Yes ☐ No	______ mm
**Induration** (Hardness) **at injection site**	☐ Yes ☐ No	______ mm
**Limitation of arm motion abduction at shoulder** (moving the upper arm up to the side away from the body)	☐ Yes ☐ No	1 2 3
**Axillary Temperature**	_______°C	
**Headache***	☐ Yes ☐ No	1 2 3
**Feeling of general discomfort or uneasiness** (Malaise)*	☐ Yes ☐ No	1 2 3
**Muscle pain** (Myalgia) *	☐ Yes ☐ No	1 2 3
**Joint Pain without swelling** (Arthralgia) *	☐ Yes ☐ No	1 2 3
**Discomfort in the stomach with an urge to vomit** (Nausea)*	☐ Yes ☐ No	1 2 3
**Vomiting***	☐ Yes ☐ No	1 2 3
***(1: Present, but easily tolerated, 2: Discomforting enough to interfere with normal activities; 3: Disabling, prevents normal activities, requires bed rest and treatment)**		
**Any other signs or symptoms?**	☐ Yes ☐ No	If Yes, then complete the details in Table 2
**Any Medication taken?**	☐ Yes ☐ No	If Yes, then complete the details in Table 3

Table 1	DAY 9	Date (DD/MM/YYYY)	__ __/__ __/20__ __

**Pain at injection site***	☐ Yes ☐ No	1 2 3
	***(1: Painful on touch, 2:Painful when limb is moved; 3: Painful at Rest)**	
**Swelling at injection site**	☐ Yes ☐ No	______ mm
**Redness at injection site**	☐ Yes ☐ No	______ mm
**Induration** (Hardness) **at injection site**	☐ Yes ☐ No	______ mm
**Limitation of arm motion abduction at shoulder** (moving the upper arm up to the side away from the body)	☐ Yes ☐ No	1 2 3
**Axillary Temperature**	_______°C	
**Headache***	☐ Yes ☐ No	1 2 3
**Feeling of general discomfort or uneasiness** (Malaise)*	☐ Yes ☐ No	1 2 3
**Muscle pain** (Myalgia) *	☐ Yes ☐ No	1 2 3
**Joint Pain without swelling** (Arthralgia) *	☐ Yes ☐ No	1 2 3
**Discomfort in the stomach with an urge to vomit** (Nausea)*	☐ Yes ☐ No	1 2 3
**Vomiting***	☐ Yes ☐ No	1 2 3
***(1: Present, but easily tolerated, 2: Discomforting enough to interfere with normal activities; 3: Disabling, prevents normal activities, requires bed rest and treatment)**		
**Any other signs or symptoms?**	☐ Yes ☐ No	If Yes, then complete the details in Table 2
**Any Medication taken?**	☐ Yes ☐ No	If Yes, then complete the details in Table 3

Table 1	DAY 10	Date (DD/MM/YYYY)	__ __/__ __/20__ __

**Pain at injection site***	☐ Yes ☐ No	1 2 3
	***(1: Painful on touch, 2:Painful when limb is moved; 3: Painful at Rest)**	
**Swelling at injection site**	☐ Yes ☐ No	_____ **mm**
**Redness at injection site**	☐ Yes ☐ No	_____ **mm**
**Induration** (Hardness) **at injection site**	☐ Yes ☐ No	_____ **mm**
**Limitation of arm motion abduction at shoulder** (moving the upper arm up to the side away from the body)	☐ Yes ☐ No	1 2 3
**Axillary Temperature**	_______°C	
**Headache***	☐ Yes ☐ No	1 2 3
**Feeling of general discomfort or uneasiness** (Malaise)*	☐ Yes ☐ No	1 2 3
**Muscle pain** (Myalgia) *	☐ Yes ☐ No	1 2 3
**Joint Pain without swelling** (Arthralgia) *	☐ Yes ☐ No	1 2 3
**Discomfort in the stomach with an urge to vomit** (Nausea)*	☐ Yes ☐ No	1 2 3
**Vomiting***	☐ Yes ☐ No	1 2 3
***(1: Present, but easily tolerated, 2: Discomforting enough to interfere with normal activities; 3: Disabling, prevents normal activities, requires bed rest and treatment)**		
**Any other signs or symptoms?**	☐ Yes ☐ No	If Yes, then complete the details in Table 2
**Any Medication taken?**	☐ Yes ☐ No	If Yes, then complete the details in Table 3

Table 1	DAY 11	Date (DD/MM/YYYY)	__ __ / __ __ / 2 0 __ __

**Pain at injection site***	☐ **Yes** ☐ **No**	1 2 3
	***(1: Painful on touch, 2:Painful when limb is moved; 3: Painful at Rest)**	
**Swelling at injection site**	☐ **Yes** ☐ **No**	______ **mm**
**Redness at injection site**	☐ **Yes** ☐ **No**	______ **mm**
**Induration** (Hardness) **at injection site**	☐ **Yes** ☐ **No**	______ **mm**
**Limitation of arm motion abduction at shoulder** (moving the upper arm up to the side away from the body)	☐ **Yes** ☐ **No**	1 2 3
**Axillary Temperature**	________°C	
**Headache***	☐ **Yes** ☐ **No**	1 2 3
**Feeling of general discomfort or uneasiness** (Malaise)*	☐ **Yes** ☐ **No**	1 2 3
**Muscle pain** (Myalgia) *	☐ **Yes** ☐ **No**	1 2 3
**Joint Pain without swelling** (Arthralgia) *	☐ **Yes** ☐ **No**	1 2 3
**Discomfort in the stomach with an urge to vomit** (Nausea)*	☐ **Yes** ☐ **No**	1 2 3
**Vomiting***	☐ **Yes** ☐ **No**	1 2 3
***(1: Present, but easily tolerated, 2: Discomforting enough to interfere with normal activities; 3: Disabling, prevents normal activities, requires bed rest and treatment)**		
**Any other signs or symptoms?**	☐ **Yes** ☐ **No**	If Yes, then complete the details in Table 2
**Any Medication taken?**	☐ **Yes** ☐ **No**	If Yes, then complete the details in Table 3

Table 1	DAY 12	Date (DD/MM/YYYY)	__ __/__ __/20__ __

**Pain at injection site***	☐ Yes ☐ No	1 2 3
	*(1: Painful on touch, 2:Painful when limb is moved; 3: Painful at Rest)	
**Swelling at injection site**	☐ Yes ☐ No	______ mm
**Redness at injection site**	☐ Yes ☐ No	______ mm
**Induration** (Hardness) **at injection site**	☐ Yes ☐ No	______ mm
**Limitation of arm motion abduction at shoulder** (moving the upper arm up to the side away from the body)	☐ Yes ☐ No	1 2 3
**Axillary Temperature**	________°C	
**Headache***	☐ Yes ☐ No	1 2 3
**Feeling of general discomfort or uneasiness** (Malaise)*	☐ Yes ☐ No	1 2 3
**Muscle pain** (Myalgia) *	☐ Yes ☐ No	1 2 3
**Joint Pain without swelling** (Arthralgia) *	☐ Yes ☐ No	1 2 3
**Discomfort in the stomach with an urge to vomit** (Nausea)*	☐ Yes ☐ No	1 2 3
**Vomiting***	☐ Yes ☐ No	1 2 3
***(1: Present, but easily tolerated, 2: Discomforting enough to interfere with normal activities; 3: Disabling, prevents normal activities, requires bed rest and treatment)**		
**Any other signs or symptoms?**	☐ Yes ☐ No	If Yes, then complete the details in Table 2
**Any Medication taken?**	☐ Yes ☐ No	If Yes, then complete the details in Table 3

Table 1	DAY 13	Date (DD/MM/YYYY)	__ __/__ __/20__ __

**Pain at injection site***	☐ Yes ☐ No	1 2 3
	*(1: Painful on touch, 2:Painful when limb is moved; 3: Painful at Rest)	
**Swelling at injection site**	☐ Yes ☐ No	_____ mm
**Redness at injection site**	☐ Yes ☐ No	_____ mm
**Induration** (Hardness) **at injection site**	☐ Yes ☐ No	_____ mm
**Limitation of arm motion abduction at shoulder** (moving the upper arm up to the side away from the body)	☐ Yes ☐ No	1 2 3
**Axillary Temperature**	______°C	
**Headache***	☐ Yes ☐ No	1 2 3
**Feeling of general discomfort or uneasiness** (Malaise)*	☐ Yes ☐ No	1 2 3
**Muscle pain** (Myalgia) *	☐ Yes ☐ No	1 2 3
**Joint Pain without swelling** (Arthralgia) *	☐ Yes ☐ No	1 2 3
**Discomfort in the stomach with an urge to vomit** (Nausea)*	☐ Yes ☐ No	1 2 3
**Vomiting***	☐ Yes ☐ No	1 2 3
***(1: Present, but easily tolerated, 2: Discomforting enough to interfere with normal activities; 3: Disabling, prevents normal activities, requires bed rest and treatment)**		
**Any other signs or symptoms?**	☐ Yes ☐ No	If Yes, then complete the details in Table 2
**Any Medication taken?**	☐ Yes ☐ No	If Yes, then complete the details in Table 3

Table 1	DAY 14	Date (DD/MM/YYYY)	__ __/__ __/2 0 __ __

Pain at injection site*	☐ Yes ☐ No	1 2 3
	*(1: Painful on touch, 2:Painful when limb is moved; 3: Painful at Rest)	
Swelling at injection site	☐ Yes ☐ No	______ mm
Redness at injection site	☐ Yes ☐ No	______ mm
Induration (Hardness) at injection site	☐ Yes ☐ No	______ mm
Limitation of arm motion abduction at shoulder (moving the upper arm up to the side away from the body)	☐ Yes ☐ No	1 2 3
Axillary Temperature	_______°C	
Headache*	☐ Yes ☐ No	1 2 3
Feeling of general discomfort or uneasiness (Malaise)*	☐ Yes ☐ No	1 2 3
Muscle pain (Myalgia) *	☐ Yes ☐ No	1 2 3
Joint Pain without swelling (Arthralgia) *	☐ Yes ☐ No	1 2 3
Discomfort in the stomach with an urge to vomit (Nausea)*	☐ Yes ☐ No	1 2 3
Vomiting*	☐ Yes ☐ No	1 2 3
*(1: Present, but easily tolerated, 2: Discomforting enough to interfere with normal activities; 3: Disabling, prevents normal activities, requires bed rest and treatment)		
Any other signs or symptoms?	☐ Yes ☐ No	If Yes, then complete the details in Table 2
Any Medication taken?	☐ Yes ☐ No	If Yes, then complete the details in Table 3

TABLE 2: (from page 19 to 20)

Enter the details of the signs or symptoms observed (other than the ones listed in Table 1).

The details can be entered as mentioned in the example stated below:

E.g.1. If Diarrhea is experienced on 23/01/2010 and stopped on 25/01/2010, medications were taken, then it will be entered as follows:

Sr. No.	Signs or symptoms	Start Date (DD/MM/YYYY) / Stop Date (DD/MM/YYYY)	Ongoing	Any medication taken?	Comments, if any
1	Diarrhea	23 / 01 / 2010<br>25 / 01 / 2010	☐	☒ Yes<br>☐ No	

The details of the medication taken will be entered in Table 3.

E.g.2. If Cough is experienced on 23/01/2010 and is ongoing, then it will be entered as follows:

Sr. No.	Signs or symptoms	Start Date (DD/MM/YYYY) / Stop Date (DD/MM/YYYY)	Ongoing	Any medication taken?	Comments, if any
1	Cough	23 / 01 / 2010<br>__ __ / __ __ / __ __ __ __	☒	☐ Yes<br>☒ No	Dry cough, occurs more at night

Table 2					
**Sr. No.**	**Signs or symptoms**	**Start Date** (DD/MM/YYYY)<br>**Stop Date** (DD/MM/YYYY)	**Ongoing**	**Any medication taken?**	**Comments, if any**
1		__ __/__ __/20__ __<br>__ __/__ __/20__ __	☐	☐ Yes<br>☐ No	
2		__ __/__ __/20__ __<br>__ __/__ __/20__ __	☐	☐ Yes<br>☐ No	
3		__ __/__ __/20__ __<br>__ __/__ __/20__ __	☐	☐ Yes<br>☐ No	
4		__ __/__ __/20__ __<br>__ __/__ __/20__ __	☐	☐ Yes<br>☐ No	
5		__ __/__ __/20__ __<br>__ __/__ __/20__ __	☐	☐ Yes<br>☐ No	
6		__ __/__ __/20__ __<br>__ __/__ __/20__ __	☐	☐ Yes<br>☐ No	
7		__ __/__ __/20__ __<br>__ __/__ __/20__ __	☐	☐ Yes<br>☐ No	
8		__ __/__ __/20__ __<br>__ __/__ __/20__ __	☐	☐ Yes<br>☐ No	
9		__ __/__ __/20__ __<br>__ __/__ __/20__ __	☐	☐ Yes<br>☐ No	
10		__ __/__ __/20__ __<br>__ __/__ __/20__ __	☐	☐ Yes<br>☐ No	

Table 2						
Sr. No.	Signs or symptoms	Start Date (DD/MM/YYYY) / Stop Date (DD/MM/YYYY)	Ongoing	Any medication taken?	Comments, if any
11		__ __/__ __/2 0__ __<br>__ __/__ __/2 0__ __	☐	☐ Yes<br>☐ No	
12		__ __/__ __/2 0__ __<br>__ __/__ __/2 0__ __	☐	☐ Yes<br>☐ No	
13		__ __/__ __/2 0__ __<br>__ __/__ __/2 0__ __	☐	☐ Yes<br>☐ No	
14		__ __/__ __/2 0__ __<br>__ __/__ __/2 0__ __	☐	☐ Yes<br>☐ No	
15		__ __/__ __/2 0__ __<br>__ __/__ __/2 0__ __	☐	☐ Yes<br>☐ No	
16		__ __/__ __/2 0__ __<br>__ __/__ __/2 0__ __	☐	☐ Yes<br>☐ No	
17		__ __/__ __/2 0__ __<br>__ __/__ __/2 0__ __	☐	☐ Yes<br>☐ No	
18		__ __/__ __/2 0__ __<br>__ __/__ __/2 0__ __	☐	☐ Yes<br>☐ No	
19		__ __/__ __/2 0__ __<br>__ __/__ __/2 0__ __	☐	☐ Yes<br>☐ No	
20		__ __/__ __/2 0__ __<br>__ __/__ __/2 0__ __	☐	☐ Yes<br>☐ No	

TABLE 3: (from page 22 to 23)

Enter details of any new medication that you were not already taking at the start of the study.

Details of the medication can be entered as mentioned in the example stated below:

If 2 CROCIN tablets of 500 mg each are taken on 23/01/2010 and 24/01/2010 then it will be entered as follows:

Sr. No.	Name of the medication	Dose (with units)	Frequency	Route	Start Date (DD/MM/YYYY)<br>Stop Date (DD/MM/YYYY)	Reason medication taken
**1**	CROCIN	500 mg	2	Oral	23 / 01 / 2010<br>24 / 01 / 2010	Headache

Table 3	MEDICATIONS TAKEN					
Sr. No.	Name of the medication	Dose (with units)	Frequency	Route	Start Date (DD/MM/YYYY)<br>Stop Date (DD/MM/YYYY)	Reason medication taken
1					__ __ / __ __ / 2 0 __ __<br>__ __ / __ __ / 2 0 __ __	
2					__ __ / __ __ / 2 0 __ __<br>__ __ / __ __ / 2 0 __ __	
3					__ __ / __ __ / 2 0 __ __<br>__ __ / __ __ / 2 0 __ __	
4					__ __ / __ __ / 2 0 __ __<br>__ __ / __ __ / 2 0 __ __	
5					__ __ / __ __ / 2 0 __ __<br>__ __ / __ __ / 2 0 __ __	
6					__ __ / __ __ / 2 0 __ __<br>__ __ / __ __ / 2 0 __ __	
7					__ __ / __ __ / 2 0 __ __<br>__ __ / __ __ / 2 0 __ __	
8					__ __ / __ __ / 2 0 __ __<br>__ __ / __ __ / 2 0 __ __	
9					__ __ / __ __ / 2 0 __ __<br>__ __ / __ __ / 2 0 __ __	
10					__ __ / __ __ / 2 0 __ __<br>__ __ / __ __ / 2 0 __ __	

Units: mcg, meq, mg, Gm, Cap, gtts, inches, units, sprays, mL, bottle

Table 3		MEDICATIONS TAKEN				
Sr. No.	Name of the medication	Dose (with units)	Frequency	Route	Start Date (DD/MM/YYYY)<br>Stop Date (DD/MM/YYYY)	Reason medication taken
11					__ __ / __ __ / 2 0 __ __<br>__ __ / __ __ / 2 0 __ __	
12					__ __ / __ __ / 2 0 __ __<br>__ __ / __ __ / 2 0 __ __	
13					__ __ / __ __ / 2 0 __ __<br>__ __ / __ __ / 2 0 __ __	
14					__ __ / __ __ / 2 0 __ __<br>__ __ / __ __ / 2 0 __ __	
15					__ __ / __ __ / 2 0 __ __<br>__ __ / __ __ / 2 0 __ __	
16					__ __ / __ __ / 2 0 __ __<br>__ __ / __ __ / 2 0 __ __	
17					__ __ / __ __ / 2 0 __ __<br>__ __ / __ __ / 2 0 __ __	
18					__ __ / __ __ / 2 0 __ __<br>__ __ / __ __ / 2 0 __ __	
19					__ __ / __ __ / 2 0 __ __<br>__ __ / __ __ / 2 0 __ __	
20					__ __ / __ __ / 2 0 __ __<br>__ __ / __ __ / 2 0 __ __	

Units: mcg, meq, mg, Gm, Cap, gtts, inches, units, sprays, mL, bottle

Investigator's signature and date: ______________________________
