# Supplementary material for: Phase I Clinical Trial of a Recombinant Blood Stage Vaccine Candidate for Plasmodium falciparum Malaria Based on MSP1 and EBA175
Source: PLoS One. 2015 Apr 30;10(4):e0117820. doi: 10.1371/journal.pone.0117820 (PMC4415778; doi:10.1371/journal.pone.0117820)
Supplement: S1 Final Analysis Listings — (PDF) [file pone.0117820.s002.pdf]

Final Listings: Final Analysis

**Protocol ID**	JAIVAC-1_1_09
**Tile**	A Phase I, Randomised, Controlled, Dose-Escalating, Single-Blind Clinical Trial to Evaluate the Safety and Immunogenicity of JAIVAC-1 Vaccine (PfMSP-1_{19} and PfF2) formulated with Montanide ISA 720 in Healthy Indian Male Subjects between 18 to 45 Years of Age
**Protocol Version**	Final, version # 1.5, 15 November 2010
**Phase**	Phase I (first study in humans)
**Sponsor's Name**	European Vaccine Initiative (EVI);<br>International Centre for Genetic Engineering and Biotechnology (ICGEB)
**Reference**	Case Report Form (Revision 1: 09th August 2010, Revision 1: 12th May 2010, Final: 16th March 2010)<br>Mock TLGs developed using Statistical Analysis Plan (dated 26/09/2011 - approved by an email) & Mock TLGs are finalized using Final signed off SAP dated 20/10/2011

Shashikanth Rai	SAS Programmer<br>DiagnoSearch Life Sciences	06/08/2013
Name (Author)	Designation	Signature and date
Varsha Parulekar	Manager: Biostatistics<br>DiagnoSearch Life Sciences	Parulekar 06/08/2013
Reviewed by: Name	Designation	Signature and date
Dr. Shantanu Mehta	Senior Manager, Clinical Research<br>MVDP	12/08/2013
Approved by: Name	Designation	Signature and date
Dr. Chetan Chitnis	Principal Investigator<br>ICGEB	12/08/2013
Approved by: Name	Designation	Signature and date
Nathalie Imbault	QA Director<br>EVI	Imbault 24/09/2013
Approved by: Name	Designation	Signature and date

Revision History

[illegible]	SOP ID	Document Name
Reason for change:		

Table of Contents

Glossary 5
14.3 SAFETY DATA 6
14.3.2 Listing of Deaths, Other Serious and Significant Adverse Events 6
14.3.2.1: Listing of Deaths (I of II) 6
14.3.2.1: Listing of Deaths (II of II) 7
14.3.2.2: Listing of Serious Adverse Events (Other than Death) (I of II) 8
14.3.2.2: Listing of Serious Adverse Events (Other than Death) (II of II) 9
14.3.2.3: Listing of Significant Adverse Events (Other than SAEs) 10
14.3.4 Abnormal Laboratory Values 11
14.3.4.1: Listing of Abnormal Haematology values by subject 11
14.3.4.2: Listing of Abnormal Serum Chemistry values by subject 29
14.3.4.3: Listing of Abnormal Urine values by subject 36
14.3.5 Abnormal Vital Signs 37
14.3.5.1: Listing of Abnormal Vital Signs by subject 37
14.3.6 Abnormal Physical Examination Results 89
14.3.6.1: Listing of Abnormal Physical Examination Results by subject 89
16.1 Study Information 94
Figure 16.1.1: Study Flowchart (Refer ICH E3 guidelines – ANNEX IV a) 94
Figure 16.1.2: Disposition of Subjects (Refer ICH E3 guidelines – ANNEX IV b) 95
16.1.6: Listing of Subjects receiving test vaccine/investigational product (s) from specific batches, where more than one batch was used 96
16.1.7: Randomisation scheme and code (Subject identification and treatment assigned) 97
16.1.9: Documentation of Statistical Methods 100
16.1.10: Documentation of inter-laboratory standardization methods and quality assurance procedures if used 101
16.2 Subject Data Listings **102**
16.2.1 Discontinued Subjects 102
16.2.1.1: Listing of Subjects who are excluded from the study (Screen Failures) 102
16.2.1.2: Listing of Subjects who Discontinued During the study 105
16.2.1.3: Listing of Subjects who Discontinued During the study due to AEs 106
16.2.2 Protocol Deviations 107
16.2.2.1: Listing of Protocol Deviations during the study 107
16.2.3 Subject excluded from the Immunogenicity analysis 110
16.2.3.1: Listing of Disposition of Subjects 110
16.2.4 Demographic data 118
16.2.4.1: Listing of Demographic data – Baseline Visit 118
16.2.4.2: Demographic data – Other Evaluation Parameters 120
16.2.5 Compliance and/or drug concentration data 122
16.2.5.1: Listing of Vaccine Administration Record for Study Visits 122
16.2.6 Individual efficacy response data 127
16.2.6.1: Listing of Immunogenicity Parameter ELISA Titers for all visits 127
16.2.6.2: Listing of Immunogenicity Parameter by IFA for all Visits 131
16.2.6.3.a: Listing of Immunogenicity Parameter IgG Subclasses against PfMSP-1_{19} by ELISA for all Visits 135
16.2.6.3.b: Listing of Immunogenicity Parameter IgG Subclasses against PfF2 by ELISA for all Visits 136

16.2.6.4: Listing of Immunogenicity Parameter Percent Inhibition by Growth Inhibition Assay (GIA) for all Visits 140
16.2.7 Adverse Events Listings 142
16.2.7.1: Unsolicited Adverse Events Listings (each Subject) (I of II) 142
16.2.7.1: Unsolicited Adverse Events Listings (each Subject) (II of II) 146
16.2.7.2: Serious Adverse Events Listings (each Subject) (I of II) 148
16.2.7.2: Serious Adverse Events Listings (each Subject) (II of II) 149
16.2.8 Listings of Individual Laboratory Measurements by Subject 150
16.2.8.1: Listing of Clinical Laboratory Normal Reference Ranges 150
16.2.8.2: Listing of Clinical Laboratory Evaluations – Haematology 153
16.2.8.3: Listing of Clinical Laboratory Evaluations – Serum Chemistry 274
16.2.8.4: Listing of Clinical Laboratory Evaluations – Urinalysis 377
16.2.8.5: Listing of Direct Coomb's Test 411
16.2.8.6: Listing of Peripheral Blood Smear (PBS) 421
16.4 Individual Subject Data Listings 433
16.4.1: Listing of Individual Accrual Status (I of II) 433
16.4.1: Listing of Individual Accrual Status (II of II) 441
16.4.2: Listing of Subject Eligibility Criteria 449
16.4.3: Listing of Subject Inclusion and Exclusion Criteria Details 455
16.4.4: Listing of Subject Inclusion Criteria 457
16.4.5: Listing of Subject Exclusion Criteria (I of II) 463
16.4.5: Listing of Subject Exclusion Criteria (II of II) 469
16.4.6: Listing of Past Medical and Surgical History 475
16.4.7: Listing of Pre-existing (Current Medical) Conditions 476
16.4.8: Listing of Pregnancy Reporting Conditions (I of III) 477
16.4.8: Listing of Pregnancy Reporting Conditions (II of III) 478
16.4.8: Listing of Pregnancy Reporting Conditions (III of III) 479
16.4.9: Listing of ECG Results 480
16.4.10: Listing of Chest X-Ray – Post Anterior View 482
16.4.11: Listing of Physical Examination Results 484
16.4.12: Listing of Vital Signs 905
16.4.13: Listing of Viral Serology Results 1134
16.4.14: Listing of Safety Assessment Data - Nodule at injection site and Lymph Node 1139
16.4.15: Listing of Safety Assessment Data – Immediate Reactogenicity - Post Immunization Reactions within 3 Hrs Post vaccination (I of II) 1140
16.4.15: Listing of Safety Assessment Data – Immediate Reactogenicity - Post Immunization Reactions from 30 minutes to 3Hrs Post Vaccination (II of II)1145
16.4.16: Listing of Safety Assessment Data – Local Solicited Adverse Events – As per Analysis (I of III) 1284
16.4.16: Listing of Safety Assessment Data – Local Solicited Adverse Events – As per CRF Data (II of III) 1293
16.4.16: Listing of Safety Assessment Data – Local Solicited Adverse Events – As per CRF Data (III of III) 1298
16.4.17: Listing of Safety Assessment Data – Systemic Solicited Adverse Events- As per Analysis (I of III) 1303
16.4.17: Listing of Safety Assessment Data – Systemic Solicited Adverse Events- As per CRF Data (II of III) 1305
16.4.17: Listing of Safety Assessment Data – Systemic Solicited Adverse Events- As per CRF Data (III of III) 1306
16.4.18: Listing of WHO Enhanced Dictionary coding for Prior Concomitant Medications 1307
16.4.19: Listing of WHO Enhanced Dictionary coding for Concomitant Medications 1308
16.4.20: Listing of Study Completion 1317
16.4.21: Listing of Completed Study Visits (I of II) 1319

16.4.21: Listing of Completed Study Visits (II of II) 1321
16.4.22: Listing of Investigator's Comments 1323
16.4.23: Listing of Laboratory Comments 1346

Glossary

Cohort 1	(10 subjects receiving dosage 0.1 ml of JAIVAC-1 vaccine containing 10µg PfMSP-1_{19} and 10µg PfF2 formulated with adjuvant Montanide ISA 720) + (5 subjects receiving 1ml of Hepatitis B vaccine)
Cohort 2	(10 subjects receiving dosage 0.25 ml of JAIVAC-1 vaccine containing 25µg PfMSP-1_{19} and 25µg PfF2 formulated with adjuvant Montanide ISA 720) + (5 subjects receiving 1ml of Hepatitis B vaccine)
Cohort 3	(10 subjects receiving dosage 0.5 ml of JAIVAC-1 vaccine containing 50µg PfMSP-1_{19} and 50µg PfF2 formulated with adjuvant Montanide ISA 720) + (5 subjects receiving 1ml of Hepatitis B vaccine)
Dosage A	0.1 ml of JAIVAC-1 vaccine containing 10µg PfMSP-1_{19} and 10µg PfF2 formulated with adjuvant Montanide ISA 720
Dosage B	0.25 ml of JAIVAC-1 vaccine containing 25µg PfMSP-1_{19} and 25µg PfF2 formulated with adjuvant Montanide ISA 720
Dosage C	0.5 ml of JAIVAC-1 vaccine containing 50µg PfMSP-1_{19} and 50µg PfF2 formulated with adjuvant Montanide ISA 720
Study Vaccine	JAIVAC-1 formulated with Montanide ISA 720
Control Vaccine	Hepatitis B vaccine
Vaccine Group	JAIVAC-1 formulated with Montanide ISA 720 and Hepatitis B vaccine
Study Group	JAIVAC-1 (Dosage A, Dosage B, Dosage C) and Hepatitis B
NA	Not Applicable
CI	Confidence Interval

14.3 SAFETY DATA

14.3.2 Listing of Deaths, Other Serious and Significant Adverse Events

14.3.2.1: Listing of Deaths (I of II)

Subject No.	Randomisation Number	Study Group	Dosage	Dose	Reported Term	Primary SOC	Preferred Term	Lower Level Term	*Preferred Term Modified for Analysis	Serious	Seriousness Criteria	Start Date	Stop Date	Duration Days	Onset Day
.	.	.	.	.	.	.	.	.	.	.	.	.	.	.	.

Note:
1) The version of the MedDRA browser used is version 13.0
2) Seriousness Criteria: 1: Death, 2-Life Threatening, 3-In-Subject Hospitalization OR Prolongation of Existing Hospitalization, 4-Persistent or Significant Disability/ Incapacity, 5-Congenital Anomaly/ Birth Defect, 6-Other
3) Duration Days = Stop Date – Start Date + 1
4) Onset Day = Start Date – Vaccination Date + 1
5) *Adverse Events for which Preferred Term is not available were replaced by Low Level Term (if available), else by verbatim name as entered in the CRF.
6) ** No subject experienced Death

14.3.2.1: Listing of Deaths (II of II)

Subject No.	Randomisation Number	Study Group	Dosage	Dose	Reported Term	Intensity	Relationship	Date of Death	Cause of Death	Autopsy Done	Autopsy Report Attached	Any Medication Given?	Action Taken on Study Vaccine
.	.	.	.	.	.	.	.	.	.	.	.	.	.

Note:
1) The version of the MedDRA browser used is version 13.0
2) Intensity: 1- Sign or symptom present, but easily tolerated, 2- Discomforting enough to interfere with normal activities, 3- Disabling, incapacitating with inability to perform usual activity
3) Relationship: 1-Related, 2-Not Related
4) * No subject experienced Death

14.3.2.2: Listing of Serious Adverse Events (Other than Death) (I of II)

Subject No.	Randomisation Number	Study Group	Dosage	Dose	Reported Term	Primary SOC	Preferred Term	Lower Level Term	*Preferred Term Modified for Analysis	Serious	Seriousness Criteria	Start Date	Stop Date	Duration Days	Onset Day
.	.	.	.	.	.	.	.	.	.	.	.	.	.	.	.

Note:
1) The version of the MedDRA browser used is version 13.0
2) Seriousness Criteria: 1: Death, 2-Life Threatening, 3-In-Subject Hospitalization OR Prolongation of Existing Hospitalization, 4-Persistent or Significant Disability/ Incapacity, 5-Congenital Anomaly/ Birth Defect, 6-Other
3) Duration Days = Stop Date – Start Date + 1
4) Onset Day = Start Date – Vaccination Date + 1
5) Action Taken on Study Vaccine: 1- Permanently discontinued, 2- Treatment period completed, 3- Already stopped before SAE onset, 4- None
6) *Adverse Events for which Preferred Term is not available were replaced by Low Level Term (if available), else by verbatim name as entered in the CRF.
7) **No subject experienced serious adverse event

14.3.2.2: Listing of Serious Adverse Events (Other than Death) (II of II)

Subject No.	Randomisation Number	Study Group	Dosage	Dose	Reported Term	Intensity	Relationship	Outcome	Any Medication Given?	Did SAE abate after study vaccine discontinuation?
.	.	.	.	.	.	.	.	.	.	.

Note:
1) The version of the MedDRA browser used is version 13.0
2) Intensity: 1- Sign or symptom present, but easily tolerated, 2- Discomforting enough to interfere with normal activities, 3- Disabling, incapacitating with inability to perform usual activity
3) Relationship: 1-Related, 2-Not Related
4) Outcome: 1- Death, 2- SAE persisted/ongoing, 3- SAE resolved, 4- Unknown, 5- Other
5) *No subject experienced serious adverse event

14.3.2.3: Listing of Significant Adverse Events (Other than SAEs)

Not Applicable

14.3.4 Abnormal Laboratory Values

14.3.4.1: Listing of Abnormal Haematology values by subject

Subject No.	Randomisation Number	Study Group	Dosage	Dose	Haematology Parameters	Unit	Day	Date of sample Collection	Results	Out of Range	Reference Range	Flag	Clinical Significance
S108	115	Hepatitis B	Hepatitis B		MCHC	gm %	Day -14 to Day -1	18/08/2010	31.6	Yes	32.0 - 36.0	L	NCS
S108	115	Hepatitis B	Hepatitis B	2	% Basophils	%	Day 28	24/09/2010	1.2	Yes	0.0 - 1.0	H	NCS
S108	115	Hepatitis B	Hepatitis B	2	% Eosinophils	%	Day 28	24/09/2010	9.2	Yes	1.0 - 6.0	H	NCS
S108	115	Hepatitis B	Hepatitis B	2	MCHC	gm %	Day 28	24/09/2010	31.7	Yes	32.0 - 36.0	L	NCS
S108	115	Hepatitis B	Hepatitis B	2	% Eosinophils	%	Day 56	22/10/2010	7.8	Yes	1.0 - 6.0	H	NCS
S108	115	Hepatitis B	Hepatitis B	3	% Eosinophils	%	Day 180	23/02/2011	6.3	Yes	1.0 - 6.0	H	NCS
S108	115	Hepatitis B	Hepatitis B	3	Haematocrit	%	Day 180	23/02/2011	39.3	Yes	40.0 - 54.0	L	CS
S108	115	Hepatitis B	Hepatitis B	3	Haemoglobin*	gm / dl	Day 180	23/02/2011	12.5	Yes	13.5 - 18.0	L	CS
S108	115	Hepatitis B	Hepatitis B	3	MCH	pg	Day 180	23/02/2011	26.9	Yes	27.0 - 31.0	L	CS
S108	115	Hepatitis B	Hepatitis B	3	MCHC	gm %	Day 180	23/02/2011	31.8	Yes	32.0 - 36.0	L	CS
S108	115	Hepatitis B	Hepatitis B	3	% Lymphocytes	%	Day 208	23/03/2011	16.7	Yes	20.0 - 45.0	L	NCS
S108	115	Hepatitis B	Hepatitis B	3	Haematocrit	%	Day 208	23/03/2011	38.8	Yes	40.0 - 54.0	L	CS
S108	115	Hepatitis B	Hepatitis B	3	Haemoglobin*	gm / dl	Day 208	23/03/2011	12.7	Yes	13.5 - 18.0	L	CS
S108	115	Hepatitis B	Hepatitis B	3	% Eosinophils	%	Day 365	26/08/2011	7.1	Yes	1.0 - 6.0	H	NCS
S118	114	JAIVAC-1	Dosage A	2	% Basophils	%	Day 28	24/09/2010	1.3	Yes	0.0 - 1.0	H	NCS
S118	114	JAIVAC-1	Dosage A	2	MCHC	gm %	Day 28	24/09/2010	31.9	Yes	32.0 - 36.0	L	NCS
S118	114	JAIVAC-1	Dosage A	2	% Eosinophils	%	Day 56	22/10/2010	6.5	Yes	1.0 - 6.0	H	NCS
S118	114	JAIVAC-1	Dosage A	3	% Basophils	%	Day 365	26/08/2011	1.2	Yes	0.0 - 1.0	H	NCS
S118	114	JAIVAC-1	Dosage A	3	% Eosinophils	%	Day 365	26/08/2011	6.1	Yes	1.0 - 6.0	H	NCS

Subject No.	Randomisation Number	Study Group	Dosage	Dose	Haematology Parameters	Unit	Day	Date of sample Collection	Results	Out of Range	Reference Range	Flag	Clinical Significance
S119	107	Hepatitis B	Hepatitis B	2	% Basophils	%	Day 28	23/09/2010	1.4	Yes	0.0 - 1.0	H	NCS
S119	107	Hepatitis B	Hepatitis B	2	% Lymphocytes	%	Day 28	23/09/2010	47.7	Yes	20.0 - 45.0	H	NCS
S119	107	Hepatitis B	Hepatitis B	2	% Neutrophils	%	Day 28	23/09/2010	39.6	Yes	40.0 - 75.0	L	NCS
S119	107	Hepatitis B	Hepatitis B	2	TLC	10E3/ul	Day 28	23/09/2010	4.41	Yes	4.50 - 11.00	L	NCS
S119	107	Hepatitis B	Hepatitis B	2	% Basophils	%	Day 56	21/10/2010	2.3	Yes	0.0 - 1.0	H	NCS
S119	107	Hepatitis B	Hepatitis B	3	% Lymphocytes	%	Day 208	22/03/2011	47.8	Yes	20.0 - 45.0	H	NCS
S119	107	Hepatitis B	Hepatitis B	3	% Neutrophils	%	Day 208	22/03/2011	39.7	Yes	40.0 - 75.0	L	NCS
S120	104	JAIVAC-1	Dosage A		% Basophils	%	Day -14 to Day -1	18/08/2010	1.4	Yes	0.0 - 1.0	H	NCS
S120	104	JAIVAC-1	Dosage A		% Lymphocytes	%	Day -14 to Day -1	18/08/2010	19.5	Yes	20.0 - 45.0	L	NCS
S120	104	JAIVAC-1	Dosage A	2	% Basophils	%	Day 28	23/09/2010	1.3	Yes	0.0 - 1.0	H	NCS
S120	104	JAIVAC-1	Dosage A	3	% Lymphocytes	%	Day 180	22/02/2011	19.5	Yes	20.0 - 45.0	L	NCS
S120	104	JAIVAC-1	Dosage A	3	% Basophils	%	Day 208	22/03/2011	1.7	Yes	0.0 - 1.0	H	NCS
S120	104	JAIVAC-1	Dosage A	3	% Lymphocytes	%	Day 365	25/08/2011	19	Yes	20.0 - 45.0	L	NCS
S120	104	JAIVAC-1	Dosage A	3	% Neutrophils	%	Day 365	25/08/2011	76	Yes	40.0 - 75.0	H	NCS
S124	106	Hepatitis B	Hepatitis B		% Basophils	%	Day -14 to Day -1	18/08/2010	1.8	Yes	0.0 - 1.0	H	NCS
S124	106	Hepatitis B	Hepatitis B		MCH	pg	Day -14 to Day -1	18/08/2010	25	Yes	27.0 - 31.0	L	NCS
S124	106	Hepatitis B	Hepatitis B		MCHC	gm %	Day -14 to Day -1	18/08/2010	30.4	Yes	32.0 - 36.0	L	NCS
S124	106	Hepatitis B	Hepatitis B	2	% Basophils	%	Day 28	23/09/2010	1.4	Yes	0.0 - 1.0	H	NCS

Subject No.	Randomisation Number	Study Group	Dosage	Dose	Haematology Parameters	Unit	Day	Date of sample Collection	Results	Out of Range	Reference Range	Flag	Clinical Significance
S124	106	Hepatitis B	Hepatitis B	2	Haemoglobin*	gm / dl	Day 28	23/09/2010	13.3	Yes	13.5 - 18.0	L	NCS
S124	106	Hepatitis B	Hepatitis B	2	MCH	pg	Day 28	23/09/2010	24.6	Yes	27.0 - 31.0	L	NCS
S124	106	Hepatitis B	Hepatitis B	2	MCHC	gm %	Day 28	23/09/2010	31.7	Yes	32.0 - 36.0	L	NCS
S124	106	Hepatitis B	Hepatitis B	2	MCV	fL	Day 28	23/09/2010	77.8	Yes	80.0- 96.0	L	NCS
S124	106	Hepatitis B	Hepatitis B	2	% Basophils	%	Day 56	21/10/2010	2	Yes	0.0 - 1.0	H	NCS
S124	106	Hepatitis B	Hepatitis B	2	Haemoglobin*	gm / dl	Day 56	21/10/2010	13.1	Yes	13.5 - 18.0	L	CS
S124	106	Hepatitis B	Hepatitis B	2	MCH	pg	Day 56	21/10/2010	23.6	Yes	27.0 - 31.0	L	CS
S124	106	Hepatitis B	Hepatitis B	2	MCHC	gm %	Day 56	21/10/2010	31.4	Yes	32.0 - 36.0	L	CS
S124	106	Hepatitis B	Hepatitis B	2	MCV	fL	Day 56	21/10/2010	75.2	Yes	80.0- 96.0	L	CS
S124	106	Hepatitis B	Hepatitis B	3	MCH	pg	Day 180	22/02/2011	24.8	Yes	27.0 - 31.0	L	NCS
S124	106	Hepatitis B	Hepatitis B	3	MCHC	gm %	Day 180	22/02/2011	31.7	Yes	32.0 - 36.0	L	NCS
S124	106	Hepatitis B	Hepatitis B	3	MCV	fL	Day 180	22/02/2011	78.2	Yes	80.0- 96.0	L	NCS
S124	106	Hepatitis B	Hepatitis B	3	% Basophils	%	Day 208	22/03/2011	2	Yes	0.0 - 1.0	H	NCS
S124	106	Hepatitis B	Hepatitis B	3	MCH	pg	Day 208	22/03/2011	24.7	Yes	27.0 - 31.0	L	NCS
S124	106	Hepatitis B	Hepatitis B	3	MCHC	gm %	Day 208	22/03/2011	31.4	Yes	32.0 - 36.0	L	NCS
S124	106	Hepatitis B	Hepatitis B	3	MCV	fL	Day 208	22/03/2011	78.7	Yes	80.0- 96.0	L	NCS
S124	106	Hepatitis B	Hepatitis B	3	% Basophils	%	Day 365	25/08/2011	1.7	Yes	0.0 - 1.0	H	NCS
S124	106	Hepatitis B	Hepatitis B	3	MCH	pg	Day 365	25/08/2011	23.7	Yes	27.0 - 31.0	L	NCS
S124	106	Hepatitis B	Hepatitis B	3	MCHC	gm %	Day 365	25/08/2011	31.1	Yes	32.0 - 36.0	L	NCS
S124	106	Hepatitis B	Hepatitis B	3	MCV	fL	Day 365	25/08/2011	76	Yes	80.0- 96.0	L	NCS
S125	102	JAIVAC-1	Dosage A	2	% Basophils	%	Day 28	21/09/2010	1.4	Yes	0.0 - 1.0	H	NCS
S125	102	JAIVAC-1	Dosage A	2	% Basophils	%	Day 56	19/10/2010	2	Yes	0.0 - 1.0	H	NCS
S125	102	JAIVAC-1	Dosage A	3	% Basophils	%	Day 180	21/02/2011	1.1	Yes	0.0 - 1.0	H	NCS
S125	102	JAIVAC-1	Dosage A	3	% Lymphocytes	%	Day 208	21/03/2011	19.3	Yes	20.0 - 45.0	L	NCS
S125	102	JAIVAC-1	Dosage A	3	MCH	pg	Day 365	26/08/2011	31.3	Yes	27.0 - 31.0	H	NCS
S130	105	JAIVAC-1	Dosage A	3	% Eosinophils	%	Day 365	25/08/2011	10.1	Yes	1.0 - 6.0	H	CS

Subject No.	Randomisation Number	Study Group	Dosage	Dose	Haematology Parameters	Unit	Day	Date of sample Collection	Results	Out of Range	Reference Range	Flag	Clinical Significance
S132	108	Hepatitis B	Hepatitis B		% Lymphocytes	%	Day -14 to Day -1	18/08/2010	16.1	Yes	20.0 - 45.0	L	NCS
S132	108	Hepatitis B	Hepatitis B	2	% Basophils	%	Day 28	23/09/2010	1.6	Yes	0.0 - 1.0	H	NCS
S132	108	Hepatitis B	Hepatitis B	2	% Basophils	%	Day 56	21/10/2010	1.7	Yes	0.0 - 1.0	H	NCS
S132	108	Hepatitis B	Hepatitis B	3	% Eosinophils	%	Day 208	22/03/2011	8	Yes	1.0 - 6.0	H	NCS
S132	108	Hepatitis B	Hepatitis B	3	% Eosinophils	%	Day 365	25/08/2011	6.6	Yes	1.0 - 6.0	H	NCS
S136	109	JAIVAC-1	Dosage A		MCHC	gm %	Day -14 to Day -1	19/08/2010	31.7	Yes	32.0 - 36.0	L	NCS
S136	109	JAIVAC-1	Dosage A	2	% Basophils	%	Day 28	23/09/2010	1.4	Yes	0.0 - 1.0	H	NCS
S136	109	JAIVAC-1	Dosage A	2	% Eosinophils	%	Day 28	23/09/2010	6.3	Yes	1.0 - 6.0	H	NCS
S136	109	JAIVAC-1	Dosage A	2	% Eosinophils	%	Day 56	21/10/2010	6.9	Yes	1.0 - 6.0	H	NCS
S136	109	JAIVAC-1	Dosage A	2	Haemoglobin*	gm / dl	Day 56	21/10/2010	13.4	Yes	13.5 - 18.0	L	NCS
S136	109	JAIVAC-1	Dosage A	3	% Basophils	%	Day 208	22/03/2011	1.4	Yes	0.0 - 1.0	H	NCS
S136	109	JAIVAC-1	Dosage A	3	% Eosinophils	%	Day 208	22/03/2011	6.2	Yes	1.0 - 6.0	H	NCS
S136	109	JAIVAC-1	Dosage A	3	MCH	pg	Day 208	22/03/2011	31.4	Yes	27.0 - 31.0	H	NCS
S136	109	JAIVAC-1	Dosage A	3	% Basophils	%	Day 365	25/08/2011	1.1	Yes	0.0 - 1.0	H	NCS
S136	109	JAIVAC-1	Dosage A	3	% Eosinophils	%	Day 365	25/08/2011	6.6	Yes	1.0 - 6.0	H	NCS
S136	109	JAIVAC-1	Dosage A	3	MCH	pg	Day 365	25/08/2011	31.9	Yes	27.0 - 31.0	H	NCS
S143	101	JAIVAC-1	Dosage A	2	% Basophils	%	Day 28	21/09/2010	1.8	Yes	0.0 - 1.0	H	NCS
S143	101	JAIVAC-1	Dosage A	2	TLC	10E3/ul	Day 28	21/09/2010	4	Yes	4.50 - 11.00	L	NCS
S143	101	JAIVAC-1	Dosage A	2	% Basophils	%	Day 56	19/10/2010	1.3	Yes	0.0 - 1.0	H	NCS
S143	101	JAIVAC-1	Dosage A	3	% Basophils	%	Day 180	21/02/2011	1.4	Yes	0.0 - 1.0	H	NCS
S143	101	JAIVAC-1	Dosage A	3	% Lymphocytes	%	Day 180	21/02/2011	16.8	Yes	20.0 - 45.0	L	NCS
S143	101	JAIVAC-1	Dosage A	3	% Basophils	%	Day 208	21/03/2011	1.2	Yes	0.0 - 1.0	H	NCS

Subject No.	Randomisation Number	Study Group	Dosage	Dose	Haematology Parameters	Unit	Day	Date of sample Collection	Results	Out of Range	Reference Range	Flag	Clinical Significance
S143	101	JAIVAC-1	Dosage A	3	TLC	10E3/ul	Day 208	21/03/2011	4.34	Yes	4.50 - 11.00	L	NCS
S143	101	JAIVAC-1	Dosage A	3	% Basophils	%	Day 365	26/08/2011	1.5	Yes	0.0 - 1.0	H	NCS
S144	103	Hepatitis B	Hepatitis B		% Basophils	%	Day -14 to Day -1	19/08/2010	1.3	Yes	0.0 - 1.0	H	NCS
S144	103	Hepatitis B	Hepatitis B	3	% Basophils	%	Day 365	26/08/2011	1.4	Yes	0.0 - 1.0	H	NCS
S145	112	JAIVAC-1	Dosage A	2	% Basophils	%	Day 56	22/10/2010	1.1	Yes	0.0 - 1.0	H	NCS
S145	112	JAIVAC-1	Dosage A	2	% Eosinophils	%	Day 56	22/10/2010	0.9	Yes	1.0 - 6.0	L	NCS
S145	112	JAIVAC-1	Dosage A	2	MCH	pg	Day 56	22/10/2010	31.2	Yes	27.0 - 31.0	H	NCS
S145	112	JAIVAC-1	Dosage A	3	% Lymphocytes	%	Day 180	23/02/2011	14.1	Yes	20.0 - 45.0	L	NCS
S145	112	JAIVAC-1	Dosage A	3	% Neutrophils	%	Day 180	23/02/2011	79.3	Yes	40.0 - 75.0	H	NCS
S148	111	JAIVAC-1	Dosage A		MCH	pg	Day -14 to Day -1	20/08/2010	26.8	Yes	27.0 - 31.0	L	NCS
S148	111	JAIVAC-1	Dosage A		MCHC	gm %	Day -14 to Day -1	20/08/2010	31.3	Yes	32.0 - 36.0	L	NCS
S148	111	JAIVAC-1	Dosage A	2	MCHC	gm %	Day 28	24/09/2010	31.1	Yes	32.0 - 36.0	L	NCS
S148	111	JAIVAC-1	Dosage A	2	Platelet Count	10E3/ul	Day 28	24/09/2010	411	Yes	150 - 400	H	NCS
S148	111	JAIVAC-1	Dosage A	2	% Basophils	%	Day 56	22/10/2010	1.5	Yes	0.0 - 1.0	H	NCS
S148	111	JAIVAC-1	Dosage A	2	Platelet Count	10E3/ul	Day 56	22/10/2010	484	Yes	150 - 400	H	NCS
S148	111	JAIVAC-1	Dosage A	3	% Basophils	%	Day 180	23/02/2011	1.3	Yes	0.0 - 1.0	H	NCS
S148	111	JAIVAC-1	Dosage A	3	% Lymphocytes	%	Day 180	23/02/2011	13.9	Yes	20.0 - 45.0	L	NCS
S148	111	JAIVAC-1	Dosage A	3	% Neutrophils	%	Day 180	23/02/2011	78.1	Yes	40.0 - 75.0	H	NCS
S148	111	JAIVAC-1	Dosage A	3	Platelet Count	10E3/ul	Day 180	23/02/2011	507	Yes	150 - 400	H	NCS
S148	111	JAIVAC-1	Dosage A	3	TLC	10E3/ul	Day 180	23/02/2011	11.82	Yes	4.50 - 11.00	H	NCS
S148	111	JAIVAC-1	Dosage A	3	% Basophils	%	Day 208	23/03/2011	1.4	Yes	0.0 - 1.0	H	NCS

Subject No.	Randomisation Number	Study Group	Dosage	Dose	Haematology Parameters	Unit	Day	Date of sample Collection	Results	Out of Range	Reference Range	Flag	Clinical Significance
S148	111	JAIVAC-1	Dosage A	3	Platelet Count	10E3/ul	Day 208	23/03/2011	415	Yes	150 - 400	H	NCS
S148	111	JAIVAC-1	Dosage A	3	% Basophils	%	Day 365	26/08/2011	2.2	Yes	0.0 - 1.0	H	NCS
S148	111	JAIVAC-1	Dosage A	3	Platelet Count	10E3/ul	Day 365	26/08/2011	463	Yes	150 - 400	H	NCS
S154	113	JAIVAC-1	Dosage A		% Lymphocytes	%	Day -14 to Day -1	19/08/2010	48.8	Yes	20.0 - 45.0	H	NCS
S154	113	JAIVAC-1	Dosage A		MCH	pg	Day -14 to Day -1	19/08/2010	25.2	Yes	27.0 - 31.0	L	NCS
S154	113	JAIVAC-1	Dosage A		MCHC	gm %	Day -14 to Day -1	19/08/2010	31.8	Yes	32.0 - 36.0	L	NCS
S154	113	JAIVAC-1	Dosage A		MCV	fL	Day -14 to Day -1	19/08/2010	79.2	Yes	80.0- 96.0	L	NCS
S154	113	JAIVAC-1	Dosage A	2	MCH	pg	Day 28	24/09/2010	25.1	Yes	27.0 - 31.0	L	NCS
S154	113	JAIVAC-1	Dosage A	2	MCHC	gm %	Day 28	24/09/2010	31.9	Yes	32.0 - 36.0	L	NCS
S154	113	JAIVAC-1	Dosage A	2	MCV	fL	Day 28	24/09/2010	78.5	Yes	80.0- 96.0	L	NCS
S154	113	JAIVAC-1	Dosage A	2	MCH	pg	Day 56	22/10/2010	25.6	Yes	27.0 - 31.0	L	NCS
S154	113	JAIVAC-1	Dosage A	2	MCV	fL	Day 56	22/10/2010	76.8	Yes	80.0- 96.0	L	NCS
S154	113	JAIVAC-1	Dosage A	3	MCH	pg	Day 180	23/02/2011	26	Yes	27.0 - 31.0	L	NCS
S154	113	JAIVAC-1	Dosage A	3	MCV	fL	Day 180	23/02/2011	78.1	Yes	80.0- 96.0	L	NCS
S154	113	JAIVAC-1	Dosage A	3	MCH	pg	Day 208	23/03/2011	25.5	Yes	27.0 - 31.0	L	NCS
S154	113	JAIVAC-1	Dosage A	3	MCV	fL	Day 208	23/03/2011	77.8	Yes	80.0- 96.0	L	NCS
S154	113	JAIVAC-1	Dosage A	3	MCH	pg	Day 365	26/08/2011	26.1	Yes	27.0 - 31.0	L	NCS
S154	113	JAIVAC-1	Dosage A	3	MCV	fL	Day 365	26/08/2011	78.1	Yes	80.0- 96.0	L	NCS
S156	110	JAIVAC-1	Dosage A		% Basophils	%	Day -14 to Day -1	19/08/2010	2.1	Yes	0.0 - 1.0	H	NCS
S156	110	JAIVAC-1	Dosage A	2	% Basophils	%	Day 28	24/09/2010	2.1	Yes	0.0 - 1.0	H	NCS
S156	110	JAIVAC-1	Dosage A	2	% Eosinophils	%	Day 28	24/09/2010	7.8	Yes	1.0 - 6.0	H	NCS
S156	110	JAIVAC-1	Dosage A	2	% Basophils	%	Day 56	22/10/2010	1.9	Yes	0.0 - 1.0	H	NCS
S156	110	JAIVAC-1	Dosage A	2	% Eosinophils	%	Day 56	22/10/2010	6.6	Yes	1.0 - 6.0	H	NCS

Subject No.	Randomisation Number	Study Group	Dosage	Dose	Haematology Parameters	Unit	Day	Date of sample Collection	Results	Out of Range	Reference Range	Flag	Clinical Significance
S156	110	JAIVAC-1	Dosage A	2	MCH	pg	Day 56	22/10/2010	31.8	Yes	27.0 - 31.0	H	NCS
S156	110	JAIVAC-1	Dosage A	3	% Basophils	%	Day 180	23/02/2011	1.3	Yes	0.0 - 1.0	H	NCS
S156	110	JAIVAC-1	Dosage A	3	% Eosinophils	%	Day 180	23/02/2011	9.1	Yes	1.0 - 6.0	H	NCS
S156	110	JAIVAC-1	Dosage A	3	MCH	pg	Day 208	23/03/2011	31.2	Yes	27.0 - 31.0	H	NCS
S156	110	JAIVAC-1	Dosage A	3	% Basophils	%	Day 365	26/08/2011	2.2	Yes	0.0 - 1.0	H	NCS
S156	110	JAIVAC-1	Dosage A	3	MCH	pg	Day 365	26/08/2011	31.8	Yes	27.0 - 31.0	H	NCS
S170	204	Hepatitis B	Hepatitis B		MCH	pg	Day -14 to Day -1	21/09/2010	31.2	Yes	27.0 - 31.0	H	NCS
S170	204	Hepatitis B	Hepatitis B	2	% Basophils	%	Day 28	27/10/2010	1.5	Yes	0.0 - 1.0	H	NCS
S170	204	Hepatitis B	Hepatitis B	2	TLC	10E3/ul	Day 28	27/10/2010	4.43	Yes	4.50 - 11.00	L	NCS
S170	204	Hepatitis B	Hepatitis B	3	% Basophils	%	Day 180	28/03/2011	1.1	Yes	0.0 - 1.0	H	NCS
S170	204	Hepatitis B	Hepatitis B	3	% Basophils	%	Day 208	25/04/2011	1.8	Yes	0.0 - 1.0	H	NCS
S170	204	Hepatitis B	Hepatitis B	3	MCH	pg	Day 208	25/04/2011	31.2	Yes	27.0 - 31.0	H	NCS
S170	204	Hepatitis B	Hepatitis B	3	% Basophils	%	Day 365	28/09/2011	1.2	Yes	0.0 - 1.0	H	NCS
S175	209	JAIVAC-1	Dosage B		% Basophils	%	Day -14 to Day -1	21/09/2010	1.6	Yes	0.0 - 1.0	H	NCS
S175	209	JAIVAC-1	Dosage B		MCH	pg	Day -14 to Day -1	21/09/2010	26.4	Yes	27.0 - 31.0	L	NCS
S175	209	JAIVAC-1	Dosage B	2	MCH	pg	Day 28	27/10/2010	25.6	Yes	27.0 - 31.0	L	NCS
S175	209	JAIVAC-1	Dosage B	2	MCHC	gm %	Day 28	27/10/2010	31.8	Yes	32.0 - 36.0	L	NCS
S175	209	JAIVAC-1	Dosage B	2	MCH	pg	Day 56	24/11/2010	25.8	Yes	27.0 - 31.0	L	NCS
S175	209	JAIVAC-1	Dosage B	2	MCHC	gm %	Day 56	24/11/2010	31.8	Yes	32.0 - 36.0	L	NCS
S175	209	JAIVAC-1	Dosage B	2	RBC count	10E6/ul	Day 56	24/11/2010	6.25	Yes	4.60 - 6.20	H	NCS
S175	209	JAIVAC-1	Dosage B	3	% Basophils	%	Day 180	28/03/2011	1.2	Yes	0.0 - 1.0	H	NCS
S175	209	JAIVAC-1	Dosage B	3	MCH	pg	Day 180	28/03/2011	25.6	Yes	27.0 - 31.0	L	NCS
S175	209	JAIVAC-1	Dosage B	3	MCV	fL	Day 180	28/03/2011	77.2	Yes	80.0- 96.0	L	NCS
S175	209	JAIVAC-1	Dosage B	3	MCH	pg	Day 208	25/04/2011	26.7	Yes	27.0 - 31.0	L	NCS
S175	209	JAIVAC-1	Dosage B	3	% Basophils	%	Day 365	28/09/2011	1.8	Yes	0.0 - 1.0	H	NCS
S175	209	JAIVAC-1	Dosage B	3	MCH	pg	Day 365	28/09/2011	26.4	Yes	27.0 - 31.0	L	NCS

Subject No.	Randomisation Number	Study Group	Dosage	Dose	Haematology Parameters	Unit	Day	Date of sample Collection	Results	Out of Range	Reference Range	Flag	Clinical Significance
S176	205	Hepatitis B	Hepatitis B		% Basophils	%	Day -14 to Day -1	22/09/2010	1.1	Yes	0.0 - 1.0	H	NCS
S176	205	Hepatitis B	Hepatitis B	2	% Basophils	%	Day 28	27/10/2010	1.7	Yes	0.0 - 1.0	H	NCS
S176	205	Hepatitis B	Hepatitis B	2	% Basophils	%	Day 56	24/11/2010	1.5	Yes	0.0 - 1.0	H	NCS
S176	205	Hepatitis B	Hepatitis B	3	TLC	10E3/ul	Day 180	28/03/2011	4.29	Yes	4.50 - 11.00	L	NCS
S176	205	Hepatitis B	Hepatitis B	3	% Basophils	%	Day 208	25/04/2011	1.5	Yes	0.0 - 1.0	H	NCS
S179	207	Hepatitis B	Hepatitis B	3	% Basophils	%	Day 180	28/03/2011	1.1	Yes	0.0 - 1.0	H	NCS
S182	208	JAIVAC-1	Dosage B	2	% Lymphocytes	%	Day 56	24/11/2010	10.4	Yes	20.0 - 45.0	L	NCS
S182	208	JAIVAC-1	Dosage B	2	% Neutrophils	%	Day 56	24/11/2010	79.4	Yes	40.0 - 75.0	H	NCS
S182	208	JAIVAC-1	Dosage B	3	% Basophils	%	Day 208	25/04/2011	1.1	Yes	0.0 - 1.0	H	NCS
S182	208	JAIVAC-1	Dosage B	3	% Basophils	%	Day 365	29/09/2011	2.4	Yes	0.0 - 1.0	H	NCS
S182	208	JAIVAC-1	Dosage B	3	% Lymphocytes	%	Day 365	29/09/2011	16.1	Yes	20.0 - 45.0	L	NCS
S185	202	JAIVAC-1	Dosage B	2	% Basophils	%	Day 28	26/10/2010	1.5	Yes	0.0 - 1.0	H	NCS
S185	202	JAIVAC-1	Dosage B	2	% Basophils	%	Day 56	23/11/2010	1.1	Yes	0.0 - 1.0	H	NCS
S185	202	JAIVAC-1	Dosage B	3	% Basophils	%	Day 365	28/09/2011	1.2	Yes	0.0 - 1.0	H	NCS
S186	201	JAIVAC-1	Dosage B		% Basophils	%	Day -14 to Day -1	21/09/2010	1.5	Yes	0.0 - 1.0	H	NCS
S186	201	JAIVAC-1	Dosage B	2	% Basophils	%	Day 28	26/10/2010	1.3	Yes	0.0 - 1.0	H	NCS
S186	201	JAIVAC-1	Dosage B	2	% Eosinophils	%	Day 28	26/10/2010	7.9	Yes	1.0 - 6.0	H	NCS
S186	201	JAIVAC-1	Dosage B	2	% Basophils	%	Day 56	23/11/2010	1.1	Yes	0.0 - 1.0	H	NCS
S186	201	JAIVAC-1	Dosage B	2	% Eosinophils	%	Day 56	23/11/2010	7.8	Yes	1.0 - 6.0	H	NCS
S186	201	JAIVAC-1	Dosage B	3	% Eosinophils	%	Day 180	28/03/2011	7.9	Yes	1.0 - 6.0	H	NCS
S186	201	JAIVAC-1	Dosage B	3	% Basophils	%	Day 208	25/04/2011	1.2	Yes	0.0 - 1.0	H	NCS
S186	201	JAIVAC-1	Dosage B	3	% Eosinophils	%	Day 208	25/04/2011	7.1	Yes	1.0 - 6.0	H	NCS

Subject No.	Randomisation Number	Study Group	Dosage	Dose	Haematology Parameters	Unit	Day	Date of sample Collection	Results	Out of Range	Reference Range	Flag	Clinical Significance
S186	201	JAIVAC-1	Dosage B	3	% Basophils	%	Day 365	28/09/2011	1.6	Yes	0.0 - 1.0	H	NCS
S186	201	JAIVAC-1	Dosage B	3	% Eosinophils	%	Day 365	28/09/2011	7.3	Yes	1.0 - 6.0	H	NCS
S192	203	JAIVAC-1	Dosage B	2	% Basophils	%	Day 28	26/10/2010	1.1	Yes	0.0 - 1.0	H	NCS
S192	203	JAIVAC-1	Dosage B	2	% Eosinophils	%	Day 56	23/11/2010	13.6	Yes	1.0 - 6.0	H	NCS
S192	203	JAIVAC-1	Dosage B	2	TLC	10E3/ul	Day 56	23/11/2010	4.32	Yes	4.50 - 11.00	L	NCS
S192	203	JAIVAC-1	Dosage B	3	TLC	10E3/ul	Day 180	28/03/2011	4.29	Yes	4.50 - 11.00	L	NCS
S192	203	JAIVAC-1	Dosage B	3	MCH	pg	Day 208	25/04/2011	31.6	Yes	27.0 - 31.0	H	NCS
S192	203	JAIVAC-1	Dosage B	3	% Basophils	%	Day 365	28/09/2011	1.5	Yes	0.0 - 1.0	H	NCS
S192	203	JAIVAC-1	Dosage B	3	MCH	pg	Day 365	28/09/2011	31.3	Yes	27.0 - 31.0	H	NCS
S192	203	JAIVAC-1	Dosage B	3	RBC count	10E6/ul	Day 365	28/09/2011	4.37	Yes	4.60 - 6.20	L	NCS
S193	215	JAIVAC-1	Dosage B		% Basophils	%	Day -14 to Day -1	24/09/2010	1.2	Yes	0.0 - 1.0	H	NCS
S193	215	JAIVAC-1	Dosage B		% Monocytes	%	Day -14 to Day -1	24/09/2010	17.4	Yes	2.0 - 10.0	H	NCS
S193	215	JAIVAC-1	Dosage B	2	% Eosinophils	%	Day 28	28/10/2010	10	Yes	1.0 - 6.0	H	NCS
S193	215	JAIVAC-1	Dosage B	2	% Monocytes	%	Day 28	28/10/2010	11	Yes	2.0 - 10.0	H	NCS
S193	215	JAIVAC-1	Dosage B	2	Platelet Count	10E3/ul	Day 28	28/10/2010	402	Yes	150 - 400	H	NCS
S193	215	JAIVAC-1	Dosage B	2	% Eosinophils	%	Day 56	25/11/2010	12	Yes	1.0 - 6.0	H	NCS
S193	215	JAIVAC-1	Dosage B	2	Platelet Count	10E3/ul	Day 56	25/11/2010	438	Yes	150 - 400	H	NCS
S193	215	JAIVAC-1	Dosage B	3	% Eosinophils	%	Day 180	29/03/2011	17	Yes	1.0 - 6.0	H	NCS
S193	215	JAIVAC-1	Dosage B	3	% Lymphocytes	%	Day 180	29/03/2011	18	Yes	20.0 - 45.0	L	NCS
S193	215	JAIVAC-1	Dosage B	3	% Eosinophils	%	Day 208	26/04/2011	17	Yes	1.0 - 6.0	H	CS

Subject No.	Randomisation Number	Study Group	Dosage	Dose	Haematology Parameters	Unit	Day	Date of sample Collection	Results	Out of Range	Reference Range	Flag	Clinical Significance
S193	215	JAIVAC-1	Dosage B	3	Platelet Count	10E3/ul	Day 208	26/04/2011	461	Yes	150 - 400	H	NCS
S193	215	JAIVAC-1	Dosage B	3	% Basophils	%	Day 365	29/09/2011	1.7	Yes	0.0 - 1.0	H	NCS
S193	215	JAIVAC-1	Dosage B	3	% Eosinophils	%	Day 365	29/09/2011	0.5	Yes	1.0 - 6.0	L	NCS
S193	215	JAIVAC-1	Dosage B	3	% Monocytes	%	Day 365	29/09/2011	11.6	Yes	2.0 - 10.0	H	NCS
S193	215	JAIVAC-1	Dosage B	3	Platelet Count	10E3/ul	Day 365	29/09/2011	424	Yes	150 - 400	H	NCS
S193	215	JAIVAC-1	Dosage B	3	TLC	10E3/ul	Day 365	29/09/2011	11.35	Yes	4.50 - 11.00	H	CS
S195	214	JAIVAC-1	Dosage B		% Basophils	%	Day -14 to Day -1	23/09/2010	1.6	Yes	0.0 - 1.0	H	NCS
S195	214	JAIVAC-1	Dosage B	2	% Basophils	%	Day 28	28/10/2010	2.1	Yes	0.0 - 1.0	H	NCS
S195	214	JAIVAC-1	Dosage B	3	% Basophils	%	Day 180	29/03/2011	1.1	Yes	0.0 - 1.0	H	NCS
S195	214	JAIVAC-1	Dosage B	3	Haematocrit	%	Day 365	29/09/2011	35.4	Yes	40.0 - 54.0	L	CS
S195	214	JAIVAC-1	Dosage B	3	Haemoglobin*	gm / dl	Day 365	29/09/2011	11.8	Yes	13.5 - 18.0	L	CS
S195	214	JAIVAC-1	Dosage B	3	RBC count	10E6/ul	Day 365	29/09/2011	4.16	Yes	4.60 - 6.20	L	NCS
S202	206	JAIVAC-1	Dosage B	3	% Basophils	%	Day 365	29/09/2011	1.4	Yes	0.0 - 1.0	H	NCS
S203	211	Hepatitis B	Hepatitis B		Haematocrit	%	Day -14 to Day -1	24/09/2010	39.9	Yes	40.0 - 54.0	L	NCS
S203	211	Hepatitis B	Hepatitis B		MCH	pg	Day -14 to Day -1	24/09/2010	24.7	Yes	27.0 - 31.0	L	NCS
S203	211	Hepatitis B	Hepatitis B		MCV	fL	Day -14 to Day -1	24/09/2010	73.3	Yes	80.0- 96.0	L	NCS
S203	211	Hepatitis B	Hepatitis B	2	% Basophils	%	Day 28	28/10/2010	2.5	Yes	0.0 - 1.0	H	NCS
S203	211	Hepatitis B	Hepatitis B	2	Haemoglobin*	gm / dl	Day 28	28/10/2010	13	Yes	13.5 - 18.0	L	NCS
S203	211	Hepatitis B	Hepatitis B	2	MCH	pg	Day 28	28/10/2010	23	Yes	27.0 - 31.0	L	NCS
S203	211	Hepatitis B	Hepatitis B	2	MCHC	gm %	Day 28	28/10/2010	31.2	Yes	32.0 - 36.0	L	NCS
S203	211	Hepatitis B	Hepatitis B	2	MCV	fL	Day 28	28/10/2010	73.7	Yes	80.0- 96.0	L	NCS
S203	211	Hepatitis B	Hepatitis B	2	Haemoglobin*	gm / dl	Day 56	25/11/2010	12.5	Yes	13.5 - 18.0	L	CS

Subject No.	Randomisation Number	Study Group	Dosage	Dose	Haematology Parameters	Unit	Day	Date of sample Collection	Results	Out of Range	Reference Range	Flag	Clinical Significance
S203	211	Hepatitis B	Hepatitis B	2	MCH	pg	Day 56	25/11/2010	23.1	Yes	27.0 - 31.0	L	CS
S203	211	Hepatitis B	Hepatitis B	2	MCHC	gm %	Day 56	25/11/2010	31	Yes	32.0 - 36.0	L	CS
S203	211	Hepatitis B	Hepatitis B	2	MCV	fL	Day 56	25/11/2010	74.6	Yes	80.0- 96.0	L	CS
S203	211	Hepatitis B	Hepatitis B	3	% Basophils	%	Day 180	29/03/2011	1.7	Yes	0.0 - 1.0	H	NCS
S203	211	Hepatitis B	Hepatitis B	3	MCH	pg	Day 180	29/03/2011	23.6	Yes	27.0 - 31.0	L	NCS
S203	211	Hepatitis B	Hepatitis B	3	MCV	fL	Day 180	29/03/2011	73.2	Yes	80.0- 96.0	L	NCS
S203	211	Hepatitis B	Hepatitis B	3	% Basophils	%	Day 208	28/04/2011	1.6	Yes	0.0 - 1.0	H	CS
S203	211	Hepatitis B	Hepatitis B	3	MCH	pg	Day 208	28/04/2011	24.4	Yes	27.0 - 31.0	L	NCS
S203	211	Hepatitis B	Hepatitis B	3	MCV	fL	Day 208	28/04/2011	75.5	Yes	80.0- 96.0	L	NCS
S203	211	Hepatitis B	Hepatitis B	3	TLC	10E3/ul	Day 208	28/04/2011	12.56	Yes	4.50 - 11.00	H	CS
S203	211	Hepatitis B	Hepatitis B	3	Haemoglobin*	gm / dl	Day 365	29/09/2011	13.3	Yes	13.5 - 18.0	L	NCS
S203	211	Hepatitis B	Hepatitis B	3	MCH	pg	Day 365	29/09/2011	24.6	Yes	27.0 - 31.0	L	NCS
S203	211	Hepatitis B	Hepatitis B	3	MCV	fL	Day 365	29/09/2011	74.8	Yes	80.0- 96.0	L	NCS
S212	212	JAIVAC-1	Dosage B		% Basophils	%	Day -14 to Day -1	23/09/2010	1.2	Yes	0.0 - 1.0	H	NCS
S212	212	JAIVAC-1	Dosage B	2	% Basophils	%	Day 28	28/10/2010	1.2	Yes	0.0 - 1.0	H	NCS
S212	212	JAIVAC-1	Dosage B	2	% Lymphocytes	%	Day 28	28/10/2010	17.7	Yes	20.0 - 45.0	L	NCS
S212	212	JAIVAC-1	Dosage B	2	Platelet Count	10E3/ul	Day 28	28/10/2010	409	Yes	150 - 400	H	NCS
S212	212	JAIVAC-1	Dosage B	3	% Lymphocytes	%	Day 180	29/03/2011	15.7	Yes	20.0 - 45.0	L	NCS
S212	212	JAIVAC-1	Dosage B	3	% Neutrophils	%	Day 180	29/03/2011	75.1	Yes	40.0 - 75.0	H	NCS
S212	212	JAIVAC-1	Dosage B	3	Haemoglobin*	gm / dl	Day 180	29/03/2011	13.3	Yes	13.5 - 18.0	L	NCS
S212	212	JAIVAC-1	Dosage B	3	MCH	pg	Day 180	29/03/2011	26.3	Yes	27.0 - 31.0	L	NCS
S212	212	JAIVAC-1	Dosage B	3	% Basophils	%	Day 208	19/04/2011	1.7	Yes	0.0 - 1.0	H	NCS
S212	212	JAIVAC-1	Dosage B	3	Haemoglobin*	gm / dl	Day 208	19/04/2011	13.3	Yes	13.5 - 18.0	L	NCS

Subject No.	Randomisation Number	Study Group	Dosage	Dose	Haematology Parameters	Unit	Day	Date of sample Collection	Results	Out of Range	Reference Range	Flag	Clinical Significance
S212	212	JAIVAC-1	Dosage B	3	MCH	pg	Day 208	19/04/2011	26.9	Yes	27.0 - 31.0	L	NCS
S212	212	JAIVAC-1	Dosage B	3	% Basophils	%	Day 365	29/09/2011	2.4	Yes	0.0 - 1.0	H	NCS
S212	212	JAIVAC-1	Dosage B	3	% Lymphocytes	%	Day 365	29/09/2011	19.7	Yes	20.0 - 45.0	L	NCS
S215	213	Hepatitis B	Hepatitis B	3	% Monocytes	%	Day 180	29/03/2011	14	Yes	2.0 - 10.0	H	NCS
S215	213	Hepatitis B	Hepatitis B	3	MCH	pg	Day 208	26/04/2011	31.2	Yes	27.0 - 31.0	H	NCS
S215	213	Hepatitis B	Hepatitis B	3	% Monocytes	%	Day 365	29/09/2011	10.2	Yes	2.0 - 10.0	H	NCS
S215	213	Hepatitis B	Hepatitis B	3	MCH	pg	Day 365	29/09/2011	31.8	Yes	27.0 - 31.0	H	NCS
S233	210	JAIVAC-1	Dosage B		% Basophils	%	Day -14 to Day -1	24/09/2010	1.2	Yes	0.0 - 1.0	H	NCS
S233	210	JAIVAC-1	Dosage B		MCH	pg	Day -14 to Day -1	24/09/2010	23.6	Yes	27.0 - 31.0	L	NCS
S233	210	JAIVAC-1	Dosage B		MCHC	gm %	Day -14 to Day -1	24/09/2010	31.3	Yes	32.0 - 36.0	L	NCS
S233	210	JAIVAC-1	Dosage B		MCV	fL	Day -14 to Day -1	24/09/2010	75.5	Yes	80.0- 96.0	L	NCS
S233	210	JAIVAC-1	Dosage B	2	MCH	pg	Day 28	28/10/2010	23.3	Yes	27.0 - 31.0	L	NCS
S233	210	JAIVAC-1	Dosage B	2	MCHC	gm %	Day 28	28/10/2010	31.9	Yes	32.0 - 36.0	L	NCS
S233	210	JAIVAC-1	Dosage B	2	MCV	fL	Day 28	28/10/2010	73.1	Yes	80.0- 96.0	L	NCS
S233	210	JAIVAC-1	Dosage B	2	MCH	pg	Day 56	25/11/2010	22.8	Yes	27.0 - 31.0	L	NCS
S233	210	JAIVAC-1	Dosage B	2	MCHC	gm %	Day 56	25/11/2010	31.7	Yes	32.0 - 36.0	L	NCS
S233	210	JAIVAC-1	Dosage B	2	MCV	fL	Day 56	25/11/2010	72	Yes	80.0- 96.0	L	NCS
S233	210	JAIVAC-1	Dosage B	2	RBC count	10E6/ul	Day 56	25/11/2010	6.79	Yes	4.60 - 6.20	H	NCS
S233	210	JAIVAC-1	Dosage B	3	Haematocrit	%	Day 180	29/03/2011	37.9	Yes	40.0 - 54.0	L	CS
S233	210	JAIVAC-1	Dosage B	3	Haemoglobin*	gm / dl	Day 180	29/03/2011	11.6	Yes	13.5 - 18.0	L	CS
S233	210	JAIVAC-1	Dosage B	3	MCH	pg	Day 180	29/03/2011	20.2	Yes	27.0 - 31.0	L	CS
S233	210	JAIVAC-1	Dosage B	3	MCHC	gm %	Day 180	29/03/2011	30.6	Yes	32.0 - 36.0	L	CS
S233	210	JAIVAC-1	Dosage B	3	MCV	fL	Day 180	29/03/2011	66	Yes	80.0- 96.0	L	CS
S233	210	JAIVAC-1	Dosage B	3	% Basophils	%	Day 208	27/04/2011	1.2	Yes	0.0 - 1.0	H	NCS

Subject No.	Randomisation Number	Study Group	Dosage	Dose	Haematology Parameters	Unit	Day	Date of sample Collection	Results	Out of Range	Reference Range	Flag	Clinical Significance
S233	210	JAIVAC-1	Dosage B	3	MCH	pg	Day 208	27/04/2011	22	Yes	27.0 - 31.0	L	NCS
S233	210	JAIVAC-1	Dosage B	3	MCHC	gm %	Day 208	27/04/2011	30	Yes	32.0 - 36.0	L	NCS
S233	210	JAIVAC-1	Dosage B	3	MCV	fL	Day 208	27/04/2011	73.2	Yes	80.0- 96.0	L	NCS
S233	210	JAIVAC-1	Dosage B	3	RBC count	10E6/ul	Day 208	27/04/2011	6.45	Yes	4.60 - 6.20	H	NCS
S233	210	JAIVAC-1	Dosage B	3	MCH	pg	Day 365	29/09/2011	24.3	Yes	27.0 - 31.0	L	NCS
S233	210	JAIVAC-1	Dosage B	3	MCV	fL	Day 365	29/09/2011	73.2	Yes	80.0- 96.0	L	NCS
S241	303	Hepatitis B	Hepatitis B		MCH	pg	Day -14 to Day -1	03/11/2010	32.1	Yes	27.0 - 31.0	H	NCS
S241	303	Hepatitis B	Hepatitis B	2	% Basophils	%	Day 28	08/12/2010	2.5	Yes	0.0 - 1.0	H	NCS
S241	303	Hepatitis B	Hepatitis B	2	MCH	pg	Day 28	08/12/2010	31.7	Yes	27.0 - 31.0	H	NCS
S241	303	Hepatitis B	Hepatitis B	2	% Basophils	%	Day 56	05/01/2011	1.8	Yes	0.0 - 1.0	H	NCS
S241	303	Hepatitis B	Hepatitis B	3	% Basophils	%	Day 180	09/05/2011	1.1	Yes	0.0 - 1.0	H	NCS
S241	303	Hepatitis B	Hepatitis B	3	% Basophils	%	Day 208	06/06/2011	1.4	Yes	0.0 - 1.0	H	NCS
S241	303	Hepatitis B	Hepatitis B	3	Haematocrit	%	Day 208	06/06/2011	54.2	Yes	40.0 - 54.0	H	NCS
S241	303	Hepatitis B	Hepatitis B	3	Haemoglobin	gm / dl	Day 208	06/06/2011	18.4	Yes	13.5 - 18.0	H	NCS
S250	311	JAIVAC-1	Dosage C		% Basophils	%	Day -14 to Day -1	03/11/2010	1.4	Yes	0.0 - 1.0	H	NCS
S250	311	JAIVAC-1	Dosage C	2	% Eosinophils	%	Day 28	10/12/2010	6.9	Yes	1.0 - 6.0	H	NCS
S250	311	JAIVAC-1	Dosage C	2	% Basophils	%	Day 56	07/01/2011	1.2	Yes	0.0 - 1.0	H	NCS
S250	311	JAIVAC-1	Dosage C	2	MCH	pg	Day 56	07/01/2011	31.6	Yes	27.0 - 31.0	H	NCS
S250	311	JAIVAC-1	Dosage C	3	% Basophils	%	Day 180	11/05/2011	1.2	Yes	0.0 - 1.0	H	NCS
S250	311	JAIVAC-1	Dosage C	3	% Eosinophils	%	Day 180	11/05/2011	6.3	Yes	1.0 - 6.0	H	NCS
S250	311	JAIVAC-1	Dosage C	3	MCH	pg	Day 180	11/05/2011	32.9	Yes	27.0 - 31.0	H	NCS
S250	311	JAIVAC-1	Dosage C	3	% Basophils	%	Day 208	08/06/2011	1.4	Yes	0.0 - 1.0	H	NCS
S250	311	JAIVAC-1	Dosage C	3	% Eosinophils	%	Day 208	08/06/2011	8.4	Yes	1.0 - 6.0	H	NCS
S250	311	JAIVAC-1	Dosage C	3	MCH	pg	Day 208	08/06/2011	32.6	Yes	27.0 - 31.0	H	NCS
S250	311	JAIVAC-1	Dosage C	3	TLC	10E3/ul	Day 208	08/06/2011	4.47	Yes	4.50 - 11.00	L	NCS

Subject No.	Randomisation Number	Study Group	Dosage	Dose	Haematology Parameters	Unit	Day	Date of sample Collection	Results	Out of Range	Reference Range	Flag	Clinical Significance
S250	311	JAIVAC-1	Dosage C	3	% Basophils	%	Day 365	10/11/2011	1.7	Yes	0.0 - 1.0	H	NCS
S250	311	JAIVAC-1	Dosage C	3	% Eosinophils	%	Day 365	10/11/2011	6.2	Yes	1.0 - 6.0	H	NCS
S263	313	Hepatitis B	Hepatitis B	2	% Basophils	%	Day 28	10/12/2010	1.5	Yes	0.0 - 1.0	H	NCS
S263	313	Hepatitis B	Hepatitis B	2	% Lymphocytes	%	Day 28	10/12/2010	19.9	Yes	20.0 - 45.0	L	NCS
S263	313	Hepatitis B	Hepatitis B	2	% Basophils	%	Day 56	07/01/2011	1.6	Yes	0.0 - 1.0	H	NCS
S263	313	Hepatitis B	Hepatitis B	3	% Lymphocytes	%	Day 180	11/05/2011	12.6	Yes	20.0 - 45.0	L	NCS
S263	313	Hepatitis B	Hepatitis B	3	% Neutrophils	%	Day 180	11/05/2011	80.1	Yes	40.0 - 75.0	H	NCS
S263	313	Hepatitis B	Hepatitis B	3	% Basophils	%	Day 208	08/06/2011	1.1	Yes	0.0 - 1.0	H	NCS
S263	313	Hepatitis B	Hepatitis B	3	% Basophils	%	Day 365	10/11/2011	1.5	Yes	0.0 - 1.0	H	NCS
S264	306	JAIVAC-1	Dosage C		MCH	pg	Day -14 to Day -1	03/11/2010	26.5	Yes	27.0 - 31.0	L	NCS
S264	306	JAIVAC-1	Dosage C	2	MCH	pg	Day 28	09/12/2010	26.1	Yes	27.0 - 31.0	L	NCS
S264	306	JAIVAC-1	Dosage C	2	MCHC	gm %	Day 28	09/12/2010	31.9	Yes	32.0 - 36.0	L	NCS
S264	306	JAIVAC-1	Dosage C	2	MCH	pg	Day 56	06/01/2011	26.6	Yes	27.0 - 31.0	L	NCS
S264	306	JAIVAC-1	Dosage C	3	MCH	pg	Day 180	10/05/2011	25.7	Yes	27.0 - 31.0	L	NCS
S264	306	JAIVAC-1	Dosage C	3	% Eosinophils	%	Day 208	07/06/2011	8	Yes	1.0 - 6.0	H	NCS
S264	306	JAIVAC-1	Dosage C	3	% Lymphocytes	%	Day 208	07/06/2011	50	Yes	20.0 - 45.0	H	NCS
S264	306	JAIVAC-1	Dosage C	3	% Neutrophils	%	Day 208	07/06/2011	38	Yes	40.0 - 75.0	L	NCS
S264	306	JAIVAC-1	Dosage C	3	MCH	pg	Day 208	07/06/2011	25.1	Yes	27.0 - 31.0	L	NCS
S264	306	JAIVAC-1	Dosage C	3	MCHC	gm %	Day 208	07/06/2011	31.5	Yes	32.0 - 36.0	L	NCS
S264	306	JAIVAC-1	Dosage C	3	MCV	fL	Day 208	07/06/2011	79.8	Yes	80.0- 96.0	L	NCS
S264	306	JAIVAC-1	Dosage C	3	% Basophils	%	Day 365	09/11/2011	1.1	Yes	0.0 - 1.0	H	NCS
S264	306	JAIVAC-1	Dosage C	3	% Eosinophils	%	Day 365	09/11/2011	6.6	Yes	1.0 - 6.0	H	NCS

Subject No.	Randomisation Number	Study Group	Dosage	Dose	Haematology Parameters	Unit	Day	Date of sample Collection	Results	Out of Range	Reference Range	Flag	Clinical Significance
S264	306	JAIVAC-1	Dosage C	3	MCH	pg	Day 365	09/11/2011	24.9	Yes	27.0 - 31.0	L	NCS
S264	306	JAIVAC-1	Dosage C	3	MCHC	gm %	Day 365	09/11/2011	31.5	Yes	32.0 - 36.0	L	NCS
S264	306	JAIVAC-1	Dosage C	3	MCV	fL	Day 365	09/11/2011	79.2	Yes	80.0- 96.0	L	NCS
S266	308	JAIVAC-1	Dosage C	2	Platelet Count	10E3/ul	Day 28	09/12/2010	429	Yes	150 - 400	H	NCS
S266	308	JAIVAC-1	Dosage C	2	% Eosinophils	%	Day 56	06/01/2011	0.5	Yes	1.0 - 6.0	L	NCS
S266	308	JAIVAC-1	Dosage C	3	Platelet Count	10E3/ul	Day 180	10/05/2011	412	Yes	150 - 400	H	NCS
S266	308	JAIVAC-1	Dosage C	3	% Basophils	%	Day 208	07/06/2011	1.7	Yes	0.0 - 1.0	H	NCS
S266	308	JAIVAC-1	Dosage C	3	Platelet Count	10E3/ul	Day 208	07/06/2011	409	Yes	150 - 400	H	NCS
S266	308	JAIVAC-1	Dosage C	3	% Basophils	%	Day 365	09/11/2011	2.3	Yes	0.0 - 1.0	H	NCS
S267	309	Hepatitis B	Hepatitis B		% Basophils	%	Day -14 to Day -1	02/11/2010	1.5	Yes	0.0 - 1.0	H	NCS
S267	309	Hepatitis B	Hepatitis B		MCH	pg	Day -14 to Day -1	02/11/2010	31.1	Yes	27.0 - 31.0	H	NCS
S267	309	Hepatitis B	Hepatitis B	2	% Eosinophils	%	Day 28	09/12/2010	8	Yes	1.0 - 6.0	H	NCS
S267	309	Hepatitis B	Hepatitis B	3	% Basophils	%	Day 180	10/05/2011	1.3	Yes	0.0 - 1.0	H	NCS
S267	309	Hepatitis B	Hepatitis B	3	% Eosinophils	%	Day 180	10/05/2011	7.3	Yes	1.0 - 6.0	H	NCS
S267	309	Hepatitis B	Hepatitis B	3	MCH	pg	Day 180	10/05/2011	31.8	Yes	27.0 - 31.0	H	NCS
S267	309	Hepatitis B	Hepatitis B	3	RBC count	10E6/ul	Day 180	10/05/2011	4.48	Yes	4.60 - 6.20	L	NCS
S267	309	Hepatitis B	Hepatitis B	3	MCH	pg	Day 208	07/06/2011	31.9	Yes	27.0 - 31.0	H	NCS
S267	309	Hepatitis B	Hepatitis B	3	RBC count	10E6/ul	Day 365	09/11/2011	4.51	Yes	4.60 - 6.20	L	NCS
S267	309	Hepatitis B	Hepatitis B	3	TLC	10E3/ul	Day 365	09/11/2011	3.44	Yes	4.50 - 11.00	L	CS
S275	302	Hepatitis B	Hepatitis B	2	% Eosinophils	%	Day 56	05/01/2011	8.7	Yes	1.0 - 6.0	H	NCS
S275	302	Hepatitis B	Hepatitis B	3	% Basophils	%	Day 180	09/05/2011	1.2	Yes	0.0 - 1.0	H	NCS
S275	302	Hepatitis B	Hepatitis B	3	% Lymphocytes	%	Day 208	06/06/2011	15.5	Yes	20.0 - 45.0	L	NCS

Subject No.	Randomisation Number	Study Group	Dosage	Dose	Haematology Parameters	Unit	Day	Date of sample Collection	Results	Out of Range	Reference Range	Flag	Clinical Significance
S275	302	Hepatitis B	Hepatitis B	3	% Neutrophils	%	Day 208	06/06/2011	77.2	Yes	40.0 - 75.0	H	NCS
S275	302	Hepatitis B	Hepatitis B	3	MCH	pg	Day 208	06/06/2011	31.1	Yes	27.0 - 31.0	H	NCS
S275	302	Hepatitis B	Hepatitis B	3	% Basophils	%	Day 365	09/11/2011	1.3	Yes	0.0 - 1.0	H	NCS
S278	315	JAIVAC-1	Dosage C		% Basophils	%	Day -14 to Day -1	08/11/2010	1.3	Yes	0.0 - 1.0	H	NCS
S278	315	JAIVAC-1	Dosage C		MCH	pg	Day -14 to Day -1	08/11/2010	32.1	Yes	27.0 - 31.0	H	NCS
S278	315	JAIVAC-1	Dosage C	2	MCH	pg	Day 28	10/12/2010	31.2	Yes	27.0 - 31.0	H	NCS
S278	315	JAIVAC-1	Dosage C	2	% Eosinophils	%	Day 56	07/01/2011	6.4	Yes	1.0 - 6.0	H	NCS
S278	315	JAIVAC-1	Dosage C	3	% Eosinophils	%	Day 180	11/05/2011	6.4	Yes	1.0 - 6.0	H	NCS
S278	315	JAIVAC-1	Dosage C	3	MCH	pg	Day 180	11/05/2011	32	Yes	27.0 - 31.0	H	NCS
S278	315	JAIVAC-1	Dosage C	3	% Basophils	%	Day 208	09/06/2011	1.9	Yes	0.0 - 1.0	H	NCS
S278	315	JAIVAC-1	Dosage C	3	% Eosinophils	%	Day 208	09/06/2011	8	Yes	1.0 - 6.0	H	NCS
S278	315	JAIVAC-1	Dosage C	3	MCH	pg	Day 208	09/06/2011	31.5	Yes	27.0 - 31.0	H	NCS
S278	315	JAIVAC-1	Dosage C	3	% Basophils	%	Day 365	10/11/2011	1.8	Yes	0.0 - 1.0	H	NCS
S278	315	JAIVAC-1	Dosage C	3	MCH	pg	Day 365	10/11/2011	32.1	Yes	27.0 - 31.0	H	NCS
S279	301	JAIVAC-1	Dosage C		MCHC	gm %	Day -14 to Day -1	03/11/2010	31.8	Yes	32.0 - 36.0	L	NCS
S279	301	JAIVAC-1	Dosage C	2	% Basophils	%	Day 28	08/12/2010	1.1	Yes	0.0 - 1.0	H	NCS
S279	301	JAIVAC-1	Dosage C	2	MCHC	gm %	Day 56	05/01/2011	31.9	Yes	32.0 - 36.0	L	NCS
S279	301	JAIVAC-1	Dosage C	3	% Basophils	%	Day 180	09/05/2011	1.4	Yes	0.0 - 1.0	H	NCS
S279	301	JAIVAC-1	Dosage C	3	% Eosinophils	%	Day 180	09/05/2011	6.8	Yes	1.0 - 6.0	H	NCS
S279	301	JAIVAC-1	Dosage C	3	% Basophils	%	Day 208	06/06/2011	1.2	Yes	0.0 - 1.0	H	NCS
S279	301	JAIVAC-1	Dosage C	3	% Basophils	%	Day 365	09/11/2011	1.1	Yes	0.0 - 1.0	H	NCS
S279	301	JAIVAC-1	Dosage C	3	MCHC	gm %	Day 365	09/11/2011	31.7	Yes	32.0 - 36.0	L	NCS
S281	304	JAIVAC-1	Dosage C		MCH	pg	Day -14 to Day -1	03/11/2010	26.4	Yes	27.0 - 31.0	L	NCS
S281	304	JAIVAC-1	Dosage C	2	% Basophils	%	Day 28	09/12/2010	4.1	Yes	0.0 - 1.0	H	NCS

Subject No.	Randomisation Number	Study Group	Dosage	Dose	Haematology Parameters	Unit	Day	Date of sample Collection	Results	Out of Range	Reference Range	Flag	Clinical Significance
S281	304	JAIVAC-1	Dosage C	2	Haemoglobin*	gm / dl	Day 28	09/12/2010	13.1	Yes	13.5 - 18.0	L	NCS
S281	304	JAIVAC-1	Dosage C	2	MCH	pg	Day 28	09/12/2010	25	Yes	27.0 - 31.0	L	NCS
S281	304	JAIVAC-1	Dosage C	2	MCHC	gm %	Day 28	09/12/2010	31.9	Yes	32.0 - 36.0	L	NCS
S281	304	JAIVAC-1	Dosage C	2	MCV	fL	Day 28	09/12/2010	78.2	Yes	80.0- 96.0	L	NCS
S281	304	JAIVAC-1	Dosage C	2	TLC	10E3/ul	Day 28	09/12/2010	3.32	Yes	4.50 - 11.00	L	NCS
S281	304	JAIVAC-1	Dosage C	2	Haematocrit	%	Day 56	06/01/2011	39.2	Yes	40.0 - 54.0	L	NCS
S281	304	JAIVAC-1	Dosage C	2	Haemoglobin*	gm / dl	Day 56	06/01/2011	12.7	Yes	13.5 - 18.0	L	CS
S281	304	JAIVAC-1	Dosage C	2	MCH	pg	Day 56	06/01/2011	24.8	Yes	27.0 - 31.0	L	CS
S281	304	JAIVAC-1	Dosage C	2	MCV	fL	Day 56	06/01/2011	76.2	Yes	80.0- 96.0	L	CS
S281	304	JAIVAC-1	Dosage C		Haematocrit	%	Early Termination	11/05/2011	39.7	Yes	40.0 - 54.0	L	CS
S281	304	JAIVAC-1	Dosage C		Haemoglobin*	gm / dl	Early Termination	11/05/2011	12.4	Yes	13.5 - 18.0	L	CS
S281	304	JAIVAC-1	Dosage C		MCH	pg	Early Termination	11/05/2011	22.9	Yes	27.0 - 31.0	L	CS
S281	304	JAIVAC-1	Dosage C		MCHC	gm %	Early Termination	11/05/2011	31.2	Yes	32.0 - 36.0	L	CS
S281	304	JAIVAC-1	Dosage C		MCV	fL	Early Termination	11/05/2011	73.2	Yes	80.0- 96.0	L	CS
S281	304	JAIVAC-1	Dosage C		TLC	10E3/ul	Early Termination	11/05/2011	4	Yes	4.50 - 11.00	L	NCS
S284	305	JAIVAC-1	Dosage C		% Basophils	%	Day -14 to Day -1	03/11/2010	1.9	Yes	0.0 - 1.0	H	NCS
S284	305	JAIVAC-1	Dosage C	2	% Basophils	%	Day 28	09/12/2010	2.1	Yes	0.0 - 1.0	H	NCS
S284	305	JAIVAC-1	Dosage C	2	% Basophils	%	Day 56	06/01/2011	1.8	Yes	0.0 - 1.0	H	NCS
S284	305	JAIVAC-1	Dosage C	3	MCH	pg	Day 365	09/11/2011	26.9	Yes	27.0 - 31.0	L	NCS
S285	307	Hepatitis B	Hepatitis B		% Basophils	%	Day -14 to Day -1	02/11/2010	1.3	Yes	0.0 - 1.0	H	NCS
S285	307	Hepatitis B	Hepatitis B	2	% Basophils	%	Day 28	09/12/2010	1.7	Yes	0.0 - 1.0	H	NCS
S285	307	Hepatitis B	Hepatitis B	3	% Basophils	%	Day 180	10/05/2011	1.2	Yes	0.0 - 1.0	H	NCS
S285	307	Hepatitis B	Hepatitis B	3	% Basophils	%	Day 208	07/06/2011	2.4	Yes	0.0 - 1.0	H	NCS

Subject No.	Randomisation Number	Study Group	Dosage	Dose	Haematology Parameters	Unit	Day	Date of sample Collection	Results	Out of Range	Reference Range	Flag	Clinical Significance
S291	312	JAIVAC-1	Dosage C	2	% Eosinophils	%	Day 56	07/01/2011	10.7	Yes	1.0 - 6.0	H	NCS
S291	312	JAIVAC-1	Dosage C	3	% Basophils	%	Day 180	11/05/2011	1.2	Yes	0.0 - 1.0	H	NCS
S291	312	JAIVAC-1	Dosage C	3	% Eosinophils	%	Day 180	11/05/2011	8.9	Yes	1.0 - 6.0	H	NCS
S291	312	JAIVAC-1	Dosage C	3	% Basophils	%	Day 208	08/06/2011	1.3	Yes	0.0 - 1.0	H	NCS
S291	312	JAIVAC-1	Dosage C	3	% Eosinophils	%	Day 208	08/06/2011	6.2	Yes	1.0 - 6.0	H	NCS
S291	312	JAIVAC-1	Dosage C	3	% Basophils	%	Day 365	10/11/2011	1.2	Yes	0.0 - 1.0	H	NCS
S298	314	JAIVAC-1	Dosage C		% Basophils	%	Day -14 to Day -1	09/11/2010	1.4	Yes	0.0 - 1.0	H	NCS
S298	314	JAIVAC-1	Dosage C	2	% Basophils	%	Day 28	10/12/2010	1.3	Yes	0.0 - 1.0	H	NCS
S298	314	JAIVAC-1	Dosage C	2	% Eosinophils	%	Day 56	07/01/2011	0.7	Yes	1.0 - 6.0	L	NCS
S298	314	JAIVAC-1	Dosage C	3	% Basophils	%	Day 180	11/05/2011	1.2	Yes	0.0 - 1.0	H	NCS
S298	314	JAIVAC-1	Dosage C	3	% Basophils	%	Day 208	08/06/2011	1.1	Yes	0.0 - 1.0	H	NCS
S298	314	JAIVAC-1	Dosage C	3	% Basophils	%	Day 365	10/11/2011	2.4	Yes	0.0 - 1.0	H	NCS
S299	310	JAIVAC-1	Dosage C	3	% Basophils	%	Day 208	08/06/2011	1.4	Yes	0.0 - 1.0	H	NCS
S299	310	JAIVAC-1	Dosage C	3	% Basophils	%	Day 365	10/11/2011	1.9	Yes	0.0 - 1.0	H	NCS

Note:
1) * If the Haemoglobin drops below the lower limit of the normal range, then a direct Coomb's test and a peripheral blood smear/film will be prepared and examined for evidence of possible haemolysis
2) Flag: L- Below Range, N- Within Range, H- Above Range
3) Clinical Significance: CS-Clinically Significant, NCS-Not Clinically Significant
4) *As per central Laboratory reference range the value is out of range but as per protocol specified reference range related to exclusion criteria the subject is eligible and Randomised and hence CS /NCS not marked.

14.3.4.2: Listing of Abnormal Serum Chemistry values by subject

Subject No.	Randomisation Number	Study Group	Dosage	Dose	Serum Chemistry Parameters	Unit	Day	Date of sample Collection	Results	Out of Range	Reference Range	Flag	Clinical Significance
S108	115	Hepatitis B	Hepatitis B	2	AST	U / L	Day 56	22/10/2010	41	Yes	0 - 40	H	NCS
S108	115	Hepatitis B	Hepatitis B	2	ALT	U / L	Day 56	22/10/2010	43	Yes	0 - 41	H	NCS
S119	107	Hepatitis B	Hepatitis B	3	Potassium	mEq / L	Day 180	22/02/2011	5.2	Yes	3.5 - 5.1	H	NCS
S120	104	JAIVAC-1	Dosage A	3	Direct Bilirubin	mg / dl	Day 208	22/03/2011	0.41	Yes	0.00 - 0.30	H	CS
S120	104	JAIVAC-1	Dosage A	3	Total Bilirubin	mg / dl	Day 208	22/03/2011	1.38	Yes	0.00 - 0.99	H	CS
S124	106	Hepatitis B	Hepatitis B	2	Direct Bilirubin	mg / dl	Day 28	23/09/2010	0.32	Yes	0.00 - 0.30	H	NCS
S124	106	Hepatitis B	Hepatitis B	2	Direct Bilirubin	mg / dl	Day 56	21/10/2010	0.38	Yes	0.00 - 0.30	H	NCS
S124	106	Hepatitis B	Hepatitis B	2	Total Bilirubin	mg / dl	Day 56	21/10/2010	1.18	Yes	0.00 - 0.99	H	NCS
S124	106	Hepatitis B	Hepatitis B	3	Direct Bilirubin	mg / dl	Day 180	22/02/2011	0.31	Yes	0.00 - 0.30	H	NCS
S124	106	Hepatitis B	Hepatitis B	3	Total Bilirubin	mg / dl	Day 180	22/02/2011	1.05	Yes	0.00 - 0.99	H	NCS
S125	102	JAIVAC-1	Dosage A	3	Random blood glucose	mg / dl	Day 180	21/02/2011	149	Yes	45-130	H	CS
S125	102	JAIVAC-1	Dosage A	3	Random blood glucose	mg / dl	Day 365	26/08/2011	160	Yes	45-130	H	CS
S125	102	JAIVAC-1	Dosage A	3	Direct Bilirubin	mg / dl	Day 365	26/08/2011	0.31	Yes	0.00 - 0.30	H	CS
S125	102	JAIVAC-1	Dosage A	3	Total Bilirubin	mg / dl	Day 365	26/08/2011	1	Yes	0.00 - 0.99	H	CS
S130	105	JAIVAC-1	Dosage A	2	Gamma Glutamyl Transpeptidase	U / L	Day 56	21/10/2010	9	Yes	10 - 71	L	NCS
S132	108	Hepatitis B	Hepatitis B	3	Random blood glucose	mg / dl	Day 208	22/03/2011	131	Yes	45-130	H	NCS

Subject No.	Randomisation Number	Study Group	Dosage	Dose	Serum Chemistry Parameters	Unit	Day	Date of sample Collection	Results	Out of Range	Reference Range	Flag	Clinical Significance
S144	103	Hepatitis B	Hepatitis B	2	Gamma Glutamyl Transpeptidase	U / L	Day 28	21/09/2010	6	Yes	10 - 71	L	NCS
S144	103	Hepatitis B	Hepatitis B	2	Gamma Glutamyl Transpeptidase	U / L	Day 56	19/10/2010	8	Yes	10 - 71	L	NCS
S144	103	Hepatitis B	Hepatitis B	3	Gamma Glutamyl Transpeptidase	U / L	Day 180	21/02/2011	6	Yes	10 - 71	L	NCS
S144	103	Hepatitis B	Hepatitis B	3	Gamma Glutamyl Transpeptidase	U / L	Day 208	21/03/2011	8	Yes	10 - 71	L	NCS
S144	103	Hepatitis B	Hepatitis B	3	Gamma Glutamyl Transpeptidase	U / L	Day 365	26/08/2011	8	Yes	10 - 71	L	NCS
S145	112	JAIVAC-1	Dosage A	3	Direct Bilirubin	mg / dl	Day 365	26/08/2011	0.33	Yes	0.00 - 0.30	H	NCS
S156	110	JAIVAC-1	Dosage A	3	Direct Bilirubin	mg / dl	Day 208	23/03/2011	0.34	Yes	0.00 - 0.30	H	NCS
S156	110	JAIVAC-1	Dosage A	3	Total Bilirubin	mg / dl	Day 208	23/03/2011	1.09	Yes	0.00 - 0.99	H	NCS
S175	209	JAIVAC-1	Dosage B	3	Gamma Glutamyl Transpeptidase	U / L	Day 208	25/04/2011	9	Yes	10 - 71	L	NCS
S175	209	JAIVAC-1	Dosage B	3	Gamma Glutamyl Transpeptidase	U / L	Day 365	28/09/2011	9	Yes	10 - 71	L	NCS
S176	205	Hepatitis B	Hepatitis B	2	ALT	U / L	Day 56	24/11/2010	44	Yes	0 - 41	H	NCS
S176	205	Hepatitis B	Hepatitis B	3	ALT	U / L	Day 208	25/04/2011	42	Yes	0 - 41	H	NCS
S176	205	Hepatitis B	Hepatitis B	3	ALT	U / L	Day 365	28/09/2011	44	Yes	0 - 41	H	CS

Subject No.	Randomisation Number	Study Group	Dosage	Dose	Serum Chemistry Parameters	Unit	Day	Date of sample Collection	Results	Out of Range	Reference Range	Flag	Clinical Significance
S179	207	Hepatitis B	Hepatitis B	2	Gamma Glutamyl Transpeptidase	U / L	Day 28	27/10/2010	5	Yes	10 - 71	L	NCS
S179	207	Hepatitis B	Hepatitis B	2	Gamma Glutamyl Transpeptidase	U / L	Day 56	24/11/2010	7	Yes	10 - 71	L	NCS
S179	207	Hepatitis B	Hepatitis B	3	Gamma Glutamyl Transpeptidase	U / L	Day 180	28/03/2011	8	Yes	10 - 71	L	NCS
S179	207	Hepatitis B	Hepatitis B	3	Gamma Glutamyl Transpeptidase	U / L	Day 208	25/04/2011	7	Yes	10 - 71	L	NCS
S179	207	Hepatitis B	Hepatitis B	3	Gamma Glutamyl Transpeptidase	U / L	Day 365	28/09/2011	6	Yes	10 - 71	L	NCS
S182	208	JAIVAC-1	Dosage B	3	Serum creatinine	mg / dl	Day 365	29/09/2011	1.3	Yes	0.7 - 1.2	H	CS
S185	202	JAIVAC-1	Dosage B	2	Direct Bilirubin	mg / dl	Day 56	23/11/2010	0.31	Yes	0.00 - 0.30	H	NCS
S186	201	JAIVAC-1	Dosage B	2	Gamma Glutamyl Transpeptidase	U / L	Day 28	26/10/2010	7	Yes	10 - 71	L	NCS
S186	201	JAIVAC-1	Dosage B	2	Gamma Glutamyl Transpeptidase	U / L	Day 56	23/11/2010	6	Yes	10 - 71	L	NCS
S186	201	JAIVAC-1	Dosage B	3	Gamma Glutamyl Transpeptidase	U / L	Day 180	28/03/2011	7	Yes	10 - 71	L	NCS
S186	201	JAIVAC-1	Dosage B	3	Direct Bilirubin	mg / dl	Day 208	25/04/2011	0.32	Yes	0.00 - 0.30	H	NCS
S186	201	JAIVAC-1	Dosage B	3	Total Bilirubin	mg / dl	Day 208	25/04/2011	1.02	Yes	0.00 - 0.99	H	NCS

Subject No.	Randomisation Number	Study Group	Dosage	Dose	Serum Chemistry Parameters	Unit	Day	Date of sample Collection	Results	Out of Range	Reference Range	Flag	Clinical Significance
S186	201	JAIVAC-1	Dosage B	3	Gamma Glutamyl Transpeptidase	U / L	Day 208	25/04/2011	6	Yes	10 - 71	L	NCS
S186	201	JAIVAC-1	Dosage B	3	Total Bilirubin	mg / dl	Day 365	28/09/2011	1.06	Yes	0.00 - 0.99	H	NCS
S186	201	JAIVAC-1	Dosage B	3	Gamma Glutamyl Transpeptidase	U / L	Day 365	28/09/2011	8	Yes	10 - 71	L	NCS
S192	203	JAIVAC-1	Dosage B	3	Gamma Glutamyl Transpeptidase	U / L	Day 365	28/09/2011	9	Yes	10 - 71	L	NCS
S193	215	JAIVAC-1	Dosage B	2	Serum creatinine	mg / dl	Day 28	28/10/2010	0.6	Yes	0.7 - 1.2	L	NCS
S193	215	JAIVAC-1	Dosage B	3	Potassium	mEq / L	Day 365	29/09/2011	5.2	Yes	3.5 - 5.1	H	CS
S215	213	Hepatitis B	Hepatitis B	2	Direct Bilirubin	mg / dl	Day 28	28/10/2010	0.31	Yes	0.00 - 0.30	H	NCS
S215	213	Hepatitis B	Hepatitis B	2	Total Bilirubin	mg / dl	Day 28	28/10/2010	1.13	Yes	0.00 - 0.99	H	NCS
S215	213	Hepatitis B	Hepatitis B	2	Gamma Glutamyl Transpeptidase	U / L	Day 28	28/10/2010	8	Yes	10 - 71	L	NCS
S215	213	Hepatitis B	Hepatitis B	2	Direct Bilirubin	mg / dl	Day 56	25/11/2010	0.37	Yes	0.00 - 0.30	H	NCS
S215	213	Hepatitis B	Hepatitis B	2	Total Bilirubin	mg / dl	Day 56	25/11/2010	1.06	Yes	0.00 - 0.99	H	NCS
S215	213	Hepatitis B	Hepatitis B	2	Gamma Glutamyl Transpeptidase	U / L	Day 56	25/11/2010	8	Yes	10 - 71	L	NCS
S215	213	Hepatitis B	Hepatitis B	3	Direct Bilirubin	mg / dl	Day 180	29/03/2011	0.44	Yes	0.00 - 0.30	H	CS
S215	213	Hepatitis B	Hepatitis B	3	Indirect Bilirubin	mg / dl	Day 180	29/03/2011	1.08	Yes	0.00 - 0.99	H	CS

Subject No.	Randomisation Number	Study Group	Dosage	Dose	Serum Chemistry Parameters	Unit	Day	Date of sample Collection	Results	Out of Range	Reference Range	Flag	Clinical Significance
S215	213	Hepatitis B	Hepatitis B	3	Total Bilirubin	mg / dl	Day 180	29/03/2011	1.52	Yes	0.00 - 0.99	H	CS
S215	213	Hepatitis B	Hepatitis B	3	Direct Bilirubin	mg / dl	Day 208	26/04/2011	0.39	Yes	0.00 - 0.30	H	CS
S215	213	Hepatitis B	Hepatitis B	3	Total Bilirubin	mg / dl	Day 208	26/04/2011	1.23	Yes	0.00 - 0.99	H	CS
S215	213	Hepatitis B	Hepatitis B	3	Gamma Glutamyl Transpeptidase	U / L	Day 208	26/04/2011	8	Yes	10 - 71	L	NCS
S215	213	Hepatitis B	Hepatitis B	3	ALT	U / L	Day 365	29/09/2011	60	Yes	0 - 41	H	CS
S215	213	Hepatitis B	Hepatitis B	3	Direct Bilirubin	mg / dl	Day 365	29/09/2011	0.34	Yes	0.00 - 0.30	H	NCS
S215	213	Hepatitis B	Hepatitis B	3	Total Bilirubin	mg / dl	Day 365	29/09/2011	1.17	Yes	0.00 - 0.99	H	NCS
S215	213	Hepatitis B	Hepatitis B	3	Gamma Glutamyl Transpeptidase	U / L	Day 365	29/09/2011	9	Yes	10 - 71	L	NCS
S233	210	JAIVAC-1	Dosage B	2	ALT	U / L	Day 56	25/11/2010	47	Yes	0 - 41	H	NCS
S233	210	JAIVAC-1	Dosage B	3	Random blood glucose	mg / dl	Day 208	27/04/2011	133	Yes	45-130	H	NCS
S233	210	JAIVAC-1	Dosage B	3	ALT	U / L	Day 208	27/04/2011	45	Yes	0 - 41	H	NCS
S233	210	JAIVAC-1	Dosage B	3	ALT	U / L	Day 365	29/09/2011	44	Yes	0 - 41	H	CS
S241	303	Hepatitis B	Hepatitis B	2	ALT	U / L	Day 56	05/01/2011	49	Yes	0 - 41	H	NCS
S250	311	JAIVAC-1	Dosage C	3	Gamma Glutamyl Transpeptidase	U / L	Day 180	11/05/2011	74	Yes	10 - 71	H	NCS
S263	313	Hepatitis B	Hepatitis B	2	Potassium	mEq / L	Day 56	07/01/2011	5.2	Yes	3.5 - 5.1	H	NCS
S264	306	JAIVAC-1	Dosage C	3	Random blood glucose	mg / dl	Day 208	07/06/2011	145	Yes	45-130	H	CS
S264	306	JAIVAC-1	Dosage C	3	AST	U / L	Day 208	07/06/2011	59	Yes	0 - 40	H	CS
S264	306	JAIVAC-1	Dosage C	3	ALT	U / L	Day 208	07/06/2011	66	Yes	0 - 41	H	CS

Subject No.	Randomisation Number	Study Group	Dosage	Dose	Serum Chemistry Parameters	Unit	Day	Date of sample Collection	Results	Out of Range	Reference Range	Flag	Clinical Significance
S264	306	JAIVAC-1	Dosage C	3	Gamma Glutamyl Transpeptidase	U / L	Day 208	07/06/2011	76	Yes	10 - 71	H	CS
S264	306	JAIVAC-1	Dosage C	3	Random blood glucose	mg / dl	Day 365	09/11/2011	156	Yes	45-130	H	CS
S264	306	JAIVAC-1	Dosage C	3	AST	U / L	Day 365	09/11/2011	46	Yes	0 - 40	H	CS
S264	306	JAIVAC-1	Dosage C	3	ALT	U / L	Day 365	09/11/2011	68	Yes	0 - 41	H	CS
S266	308	JAIVAC-1	Dosage C	2	Potassium	mEq / L	Day 56	06/01/2011	5.6	Yes	3.5 - 5.1	H	NCS
S267	309	Hepatitis B	Hepatitis B	2	Serum creatinine	mg / dl	Day 28	09/12/2010	1.3	Yes	0.7 - 1.2	H	NCS
S267	309	Hepatitis B	Hepatitis B	2	Serum creatinine	mg / dl	Day 56	06/01/2011	1.4	Yes	0.7 - 1.2	H	NCS
S267	309	Hepatitis B	Hepatitis B	3	Serum creatinine	mg / dl	Day 180	10/05/2011	1.4	Yes	0.7 - 1.2	H	NCS
S267	309	Hepatitis B	Hepatitis B	3	Serum creatinine	mg / dl	Day 208	07/06/2011	1.5	Yes	0.7 - 1.2	H	CS
S267	309	Hepatitis B	Hepatitis B	3	Serum creatinine	mg / dl	Day 365	09/11/2011	1.4	Yes	0.7 - 1.2	H	CS
S275	302	Hepatitis B	Hepatitis B	2	Serum creatinine	mg / dl	Day 28	08/12/2010	1.3	Yes	0.7 - 1.2	H	NCS
S275	302	Hepatitis B	Hepatitis B	3	Potassium	mEq / L	Day 208	06/06/2011	3.4	Yes	3.5 - 5.1	L	NCS
S275	302	Hepatitis B	Hepatitis B	3	Serum creatinine	mg / dl	Day 365	09/11/2011	1.3	Yes	0.7 - 1.2	H	CS
S279	301	JAIVAC-1	Dosage C	2	Sodium	mEq / L	Day 28	08/12/2010	135	Yes	136 - 145	L	NCS
S279	301	JAIVAC-1	Dosage C	2	Serum creatinine	mg / dl	Day 28	08/12/2010	1.4	Yes	0.7 - 1.2	H	NCS
S279	301	JAIVAC-1	Dosage C	3	Random blood glucose	mg / dl	Day 180	09/05/2011	189	Yes	45-130	H	CS
S279	301	JAIVAC-1	Dosage C	3	ALT	U / L	Day 208	06/06/2011	59	Yes	0 - 41	H	NCS
S279	301	JAIVAC-1	Dosage C	3	Serum creatinine	mg / dl	Day 365	09/11/2011	1.3	Yes	0.7 - 1.2	H	CS
S279	301	JAIVAC-1	Dosage C	3	ALT	U / L	Day 365	09/11/2011	61	Yes	0 - 41	H	CS

Subject No.	Randomisation Number	Study Group	Dosage	Dose	Serum Chemistry Parameters	Unit	Day	Date of sample Collection	Results	Out of Range	Reference Range	Flag	Clinical Significance
S281	304	JAIVAC-1	Dosage C		Serum creatinine	mg / dl	Early Termination	11/05/2011	1.3	Yes	0.7 - 1.2	H	NCS
S284	305	JAIVAC-1	Dosage C	3	Serum creatinine	mg / dl	Day 365	09/11/2011	1.3	Yes	0.7 - 1.2	H	CS
S285	307	Hepatitis B	Hepatitis B	3	ALT	U / L	Day 365	09/11/2011	49	Yes	0 - 41	H	CS
S298	314	JAIVAC-1	Dosage C	2	Random blood glucose	mg / dl	Day 28	10/12/2010	131	Yes	45-130	H	NCS
S298	314	JAIVAC-1	Dosage C	3	Potassium	mEq / L	Day 180	11/05/2011	5.4	Yes	3.5 - 5.1	H	CS
S298	314	JAIVAC-1	Dosage C	3	Potassium	mEq / L	Day 365	10/11/2011	5.5	Yes	3.5 - 5.1	H	CS
S298	314	JAIVAC-1	Dosage C	3	Serum creatinine	mg / dl	Day 365	10/11/2011	1.3	Yes	0.7 - 1.2	H	CS
S299	310	JAIVAC-1	Dosage C	3	Serum creatinine	mg / dl	Day 365	10/11/2011	1.3	Yes	0.7 - 1.2	H	CS

Note:
1) Flag: L- Below Range, N- Within Range, H- Above Range
2) Clinical Significance: CS-Clinically Significant, NCS-Not Clinically Significant

14.3.4.3: Listing of Abnormal Urine values by subject

Subject No.	Randomisation Number	Study Group	Dosage	Dose	Age (years)	Urine Parameters	Day	Date of sample Collection	Results
*	.	.	.	.	.	.	.	.	.

Note: **No subject with abnormal Urine Values

14.3.5 Abnormal Vital Signs

14.3.5.1: Listing of Abnormal Vital Signs by subject

Subject No.	Randomisation Number	Study Group	Dosage	Dose	Day	Not Done	Specify	Date of Assessment	*Was the assessment performed prior to vaccination?	Vital Signs Parameter	Units	Result	Out of Range	Clinical Significance
S108	115	Hepatitis B	Hepatitis B		Day -14 to Day -1	.		18/08/2010		Axillary temperature	°C	35.5	Yes	NCS
S108	115	Hepatitis B	Hepatitis B		Day -14 to Day -1	.		18/08/2010		Diastolic Blood pressure	mm of Hg	70	Yes	NCS
S108	115	Hepatitis B	Hepatitis B		Day -14 to Day -1	.		18/08/2010		Systolic Blood pressure	mm of Hg	104	Yes	NCS
S108	115	Hepatitis B	Hepatitis B	1	Day 0	.		27/08/2010	Yes	Axillary temperature	°C	35.6	Yes	NCS
S108	115	Hepatitis B	Hepatitis B	1	Day 14	.		09/09/2010		Axillary temperature	°C	35.7	Yes	NCS
S108	115	Hepatitis B	Hepatitis B	1	Day 14	.		09/09/2010		Diastolic Blood pressure	mm of Hg	70	Yes	NCS
S108	115	Hepatitis B	Hepatitis B	2	Day 35	.		01/10/2010		Axillary temperature	°C	35.9	Yes	NCS
S108	115	Hepatitis B	Hepatitis B	2	Day 35	.		01/10/2010		Respiratory rate	breaths/ minute	14	Yes	NCS
S108	115	Hepatitis B	Hepatitis B	2	Day 42	.		08/10/2010		Diastolic Blood pressure	mm of Hg	64	Yes	NCS
S108	115	Hepatitis B	Hepatitis B	2	Day 56	.		22/10/2010		Axillary temperature	°C	35.8	Yes	NCS

Subject No.	Randomisation Number	Study Group	Dosage	Dose	Day	Not Done	Specify	Date of Assessment	*Was the assessment performed prior to vaccination?	Vital Signs Parameter	Units	Result	Out of Range	Clinical Significance
S108	115	Hepatitis B	Hepatitis B	2	Day 56	.		22/10/2010		Diastolic Blood pressure	mm of Hg	70	Yes	NCS
S108	115	Hepatitis B	Hepatitis B	3	Day 180	.		23/02/2011	Yes	Axillary temperature	°C	35.5	Yes	NCS
S108	115	Hepatitis B	Hepatitis B	3	Day 180	.		23/02/2011	Yes	Diastolic Blood pressure	mm of Hg	74	Yes	NCS
S108	115	Hepatitis B	Hepatitis B	3	Day 180	.		23/02/2011	Yes	Respiratory rate	breaths/ minute	14	Yes	NCS
S108	115	Hepatitis B	Hepatitis B	3	Day 187	.		02/03/2011		Diastolic Blood pressure	mm of Hg	74	Yes	NCS
S108	115	Hepatitis B	Hepatitis B	3	Day 194	.		09/03/2011		Axillary temperature	°C	34.9	Yes	NCS
S108	115	Hepatitis B	Hepatitis B	3	Day 194	.		09/03/2011		Diastolic Blood pressure	mm of Hg	74	Yes	NCS
S118	114	JAIVAC-1	Dosage A		Day -14 to Day -1	.		17/08/2010		Axillary temperature	°C	35.8	Yes	NCS
S118	114	JAIVAC-1	Dosage A	1	Day 7	.		03/09/2010		Diastolic Blood pressure	mm of Hg	74	Yes	NCS
S118	114	JAIVAC-1	Dosage A	1	Day 14	.		09/09/2010		Axillary temperature	°C	37.6	Yes	NCS
S118	114	JAIVAC-1	Dosage A	1	Day 14	.		09/09/2010		Diastolic Blood pressure	mm of Hg	74	Yes	NCS
S118	114	JAIVAC-1	Dosage A	1	Day 14	.		09/09/2010		Pulse rate	beats/ minute	90	Yes	NCS

Subject No.	Randomisation Number	Study Group	Dosage	Dose	Day	Not Done	Specify	Date of Assessment	*Was the assessment performed prior to vaccination?	Vital Signs Parameter	Units	Result	Out of Range	Clinical Significance
S118	114	JAIVAC-1	Dosage A	2	Day 28	.		24/09/2010	Yes	Systolic Blood pressure	mm of Hg	102	Yes	NCS
S118	114	JAIVAC-1	Dosage A	2	Day 35	.		30/09/2010		Diastolic Blood pressure	mm of Hg	70	Yes	NCS
S118	114	JAIVAC-1	Dosage A	2	Day 42	.		08/10/2010		Diastolic Blood pressure	mm of Hg	74	Yes	NCS
S118	114	JAIVAC-1	Dosage A	2	Day 56	.		22/10/2010		Diastolic Blood pressure	mm of Hg	70	Yes	NCS
S118	114	JAIVAC-1	Dosage A	2	Day 56	.		22/10/2010		Systolic Blood pressure	mm of Hg	104	Yes	NCS
S118	114	JAIVAC-1	Dosage A	3	Day 194	.		09/03/2011		Diastolic Blood pressure	mm of Hg	68	Yes	NCS
S118	114	JAIVAC-1	Dosage A	3	Day 208	.		23/03/2011		Diastolic Blood pressure	mm of Hg	72	Yes	NCS
S118	114	JAIVAC-1	Dosage A	3	Day 365	.		26/08/2011		Axillary temperature	°C	35.4	Yes	NCS
S119	107	Hepatitis B	Hepatitis B		Day -14 to Day -1	.		18/08/2010		Axillary temperature	°C	35.8	Yes	NCS
S119	107	Hepatitis B	Hepatitis B	1	Day 0	.		26/08/2010	Yes	Axillary temperature	°C	35.7	Yes	NCS
S119	107	Hepatitis B	Hepatitis B	1	Day 7	.		02/09/2010		Axillary temperature	°C	35.2	Yes	NCS
S119	107	Hepatitis B	Hepatitis B	1	Day 7	.		02/09/2010		Diastolic Blood pressure	mm of Hg	74	Yes	NCS

Subject No.	Randomisation Number	Study Group	Dosage	Dose	Day	Not Done	Specify	Date of Assessment	*Was the assessment performed prior to vaccination?	Vital Signs Parameter	Units	Result	Out of Range	Clinical Significance
S119	107	Hepatitis B	Hepatitis B	1	Day 14	.		09/09/2010		Axillary temperature	°C	35	Yes	NCS
S119	107	Hepatitis B	Hepatitis B	2	Day 35	.		29/09/2010		Diastolic Blood pressure	mm of Hg	74	Yes	NCS
S119	107	Hepatitis B	Hepatitis B	2	Day 42	.		06/10/2010		Axillary temperature	°C	35.4	Yes	NCS
S119	107	Hepatitis B	Hepatitis B	2	Day 56	.		21/10/2010		Axillary temperature	°C	35.8	Yes	NCS
S119	107	Hepatitis B	Hepatitis B	3	Day 180	.		22/02/2011	Yes	Axillary temperature	°C	35.2	Yes	NCS
S119	107	Hepatitis B	Hepatitis B	3	Day 187	.		02/03/2011		Axillary temperature	°C	35.6	Yes	NCS
S119	107	Hepatitis B	Hepatitis B	3	Day 194	.		08/03/2011		Axillary temperature	°C	35.8	Yes	NCS
S119	107	Hepatitis B	Hepatitis B	3	Day 365	.		25/08/2011		Axillary temperature	°C	35.8	Yes	NCS
S119	107	Hepatitis B	Hepatitis B	3	Day 365	.		25/08/2011		Diastolic Blood pressure	mm of Hg	68	Yes	NCS
S119	107	Hepatitis B	Hepatitis B	3	Day 365	.		25/08/2011		Systolic Blood pressure	mm of Hg	134	Yes	NCS
S120	104	JAIVAC-1	Dosage A		Day -14 to Day -1	.		18/08/2010		Diastolic Blood pressure	mm of Hg	70	Yes	NCS
S120	104	JAIVAC-1	Dosage A	1	Day 7	.		02/09/2010		Diastolic Blood pressure	mm of Hg	70	Yes	NCS

Subject No.	Randomisation Number	Study Group	Dosage	Dose	Day	Not Done	Specify	Date of Assessment	*Was the assessment performed prior to vaccination?	Vital Signs Parameter	Units	Result	Out of Range	Clinical Significance
S120	104	JAIVAC-1	Dosage A	2	Day 28	.		23/09/2010	Yes	Axillary temperature	°C	35.4	Yes	NCS
S120	104	JAIVAC-1	Dosage A	2	Day 35	.		30/09/2010		Axillary temperature	°C	35.9	Yes	NCS
S120	104	JAIVAC-1	Dosage A	2	Day 35	.		30/09/2010		Diastolic Blood pressure	mm of Hg	70	Yes	NCS
S120	104	JAIVAC-1	Dosage A	2	Day 42	.		07/10/2010		Axillary temperature	°C	35.6	Yes	NCS
S120	104	JAIVAC-1	Dosage A	2	Day 42	.		07/10/2010		Diastolic Blood pressure	mm of Hg	70	Yes	NCS
S120	104	JAIVAC-1	Dosage A	2	Day 42	.		07/10/2010		Respiratory rate	breaths/ minute	14	Yes	NCS
S120	104	JAIVAC-1	Dosage A	2	Day 42	.		07/10/2010		Systolic Blood pressure	mm of Hg	106	Yes	NCS
S120	104	JAIVAC-1	Dosage A	3	Day 180	.		22/02/2011	Yes	Axillary temperature	°C	35.8	Yes	NCS
S120	104	JAIVAC-1	Dosage A	3	Day 180	.		22/02/2011	Yes	Diastolic Blood pressure	mm of Hg	74	Yes	NCS
S120	104	JAIVAC-1	Dosage A	3	Day 187	.		01/03/2011		Axillary temperature	°C	35.6	Yes	NCS
S120	104	JAIVAC-1	Dosage A	3	Day 194	.		08/03/2011		Axillary temperature	°C	35.9	Yes	NCS
S120	104	JAIVAC-1	Dosage A	3	Day 194	.		08/03/2011		Diastolic Blood pressure	mm of Hg	74	Yes	NCS

Subject No.	Randomisation Number	Study Group	Dosage	Dose	Day	Not Done	Specify	Date of Assessment	*Was the assessment performed prior to vaccination?	Vital Signs Parameter	Units	Result	Out of Range	Clinical Significance
S120	104	JAIVAC-1	Dosage A	3	Day 365	.		25/08/2011		Diastolic Blood pressure	mm of Hg	86	Yes	NCS
S124	106	Hepatitis B	Hepatitis B	1	Day 14	.		08/09/2010		Axillary temperature	°C	35.9	Yes	NCS
S124	106	Hepatitis B	Hepatitis B	1	Day 14	.		08/09/2010		Diastolic Blood pressure	mm of Hg	74	Yes	NCS
S124	106	Hepatitis B	Hepatitis B	2	Day 28	.		23/09/2010	Yes	Axillary temperature	°C	35.8	Yes	NCS
S124	106	Hepatitis B	Hepatitis B	2	Day 35	.		29/09/2010		Diastolic Blood pressure	mm of Hg	74	Yes	NCS
S124	106	Hepatitis B	Hepatitis B	2	Day 35	.		29/09/2010		Respiratory rate	breaths/ minute	14	Yes	NCS
S124	106	Hepatitis B	Hepatitis B	2	Day 42	.		07/10/2010		Axillary temperature	°C	35.9	Yes	NCS
S124	106	Hepatitis B	Hepatitis B	2	Day 42	.		07/10/2010		Diastolic Blood pressure	mm of Hg	70	Yes	NCS
S124	106	Hepatitis B	Hepatitis B	2	Day 56	.		21/10/2010		Diastolic Blood pressure	mm of Hg	74	Yes	NCS
S124	106	Hepatitis B	Hepatitis B	3	Day 180	.		22/02/2011	Yes	Diastolic Blood pressure	mm of Hg	64	Yes	NCS
S124	106	Hepatitis B	Hepatitis B	3	Day 187	.		01/03/2011		Respiratory rate	breaths/ minute	14	Yes	NCS
S124	106	Hepatitis B	Hepatitis B	3	Day 194	.		08/03/2011		Diastolic Blood pressure	mm of Hg	64	Yes	NCS

Subject No.	Randomisation Number	Study Group	Dosage	Dose	Day	Not Done	Specify	Date of Assessment	*Was the assessment performed prior to vaccination?	Vital Signs Parameter	Units	Result	Out of Range	Clinical Significance
S124	106	Hepatitis B	Hepatitis B	3	Day 208	.		22/03/2011		Axillary temperature	°C	35.2	Yes	NCS
S124	106	Hepatitis B	Hepatitis B	3	Day 208	.		22/03/2011		Diastolic Blood pressure	mm of Hg	68	Yes	NCS
S124	106	Hepatitis B	Hepatitis B	3	Day 365	.		25/08/2011		Axillary temperature	°C	35	Yes	NCS
S124	106	Hepatitis B	Hepatitis B	3	Day 365	.		25/08/2011		Respiratory rate	breaths/ minute	14	Yes	NCS
S125	102	JAIVAC-1	Dosage A		Day -14 to Day -1	.		18/08/2010		Axillary temperature	°C	35.4	Yes	NCS
S125	102	JAIVAC-1	Dosage A		Day -14 to Day -1	.		18/08/2010		Diastolic Blood pressure	mm of Hg	70	Yes	NCS
S125	102	JAIVAC-1	Dosage A		Day -14 to Day -1	.		18/08/2010		Systolic Blood pressure	mm of Hg	100	Yes	NCS
S125	102	JAIVAC-1	Dosage A	1	Day 0	.		24/08/2010	Yes	Diastolic Blood pressure	mm of Hg	70	Yes	NCS
S125	102	JAIVAC-1	Dosage A	1	Day 0	.		24/08/2010	Yes	Respiratory rate	breaths/ minute	14	Yes	NCS
S125	102	JAIVAC-1	Dosage A	1	Day 0	.		24/08/2010	Yes	Systolic Blood pressure	mm of Hg	108	Yes	NCS
S125	102	JAIVAC-1	Dosage A	1	Day 14	.		07/09/2010		Axillary temperature	°C	35.8	Yes	NCS
S125	102	JAIVAC-1	Dosage A	1	Day 14	.		07/09/2010		Diastolic Blood pressure	mm of Hg	74	Yes	NCS

Subject No.	Randomisation Number	Study Group	Dosage	Dose	Day	Not Done	Specify	Date of Assessment	*Was the assessment performed prior to vaccination?	Vital Signs Parameter	Units	Result	Out of Range	Clinical Significance
S125	102	JAIVAC-1	Dosage A	2	Day 35	.		28/09/2010		Axillary temperature	°C	35.6	Yes	NCS
S125	102	JAIVAC-1	Dosage A	2	Day 35	.		28/09/2010		Diastolic Blood pressure	mm of Hg	70	Yes	NCS
S125	102	JAIVAC-1	Dosage A	2	Day 35	.		28/09/2010		Systolic Blood pressure	mm of Hg	104	Yes	NCS
S125	102	JAIVAC-1	Dosage A	2	Day 42	.		05/10/2010		Axillary temperature	°C	35.6	Yes	NCS
S125	102	JAIVAC-1	Dosage A	2	Day 42	.		05/10/2010		Diastolic Blood pressure	mm of Hg	70	Yes	NCS
S125	102	JAIVAC-1	Dosage A	2	Day 42	.		05/10/2010		Systolic Blood pressure	mm of Hg	104	Yes	NCS
S125	102	JAIVAC-1	Dosage A	2	Day 56	.		19/10/2010		Axillary temperature	°C	35.9	Yes	NCS
S125	102	JAIVAC-1	Dosage A	3	Day 180	.		21/02/2011	Yes	Axillary temperature	°C	35.2	Yes	NCS
S125	102	JAIVAC-1	Dosage A	3	Day 180	.		21/02/2011	Yes	Diastolic Blood pressure	mm of Hg	74	Yes	NCS
S125	102	JAIVAC-1	Dosage A	3	Day 187	.		28/02/2011		Axillary temperature	°C	35.4	Yes	NCS
S125	102	JAIVAC-1	Dosage A	3	Day 187	.		28/02/2011		Diastolic Blood pressure	mm of Hg	64	Yes	NCS
S125	102	JAIVAC-1	Dosage A	3	Day 194	.		07/03/2011		Diastolic Blood pressure	mm of Hg	64	Yes	NCS

Subject No.	Randomisation Number	Study Group	Dosage	Dose	Day	Not Done	Specify	Date of Assessment	*Was the assessment performed prior to vaccination?	Vital Signs Parameter	Units	Result	Out of Range	Clinical Significance
S125	102	JAIVAC-1	Dosage A	3	Day 208	.		21/03/2011		Axillary temperature	°C	35.8	Yes	NCS
S125	102	JAIVAC-1	Dosage A	3	Day 208	.		21/03/2011		Diastolic Blood pressure	mm of Hg	62	Yes	NCS
S125	102	JAIVAC-1	Dosage A	3	Day 208	.		21/03/2011		Systolic Blood pressure	mm of Hg	108	Yes	NCS
S125	102	JAIVAC-1	Dosage A	3	Day 365	.		26/08/2011		Axillary temperature	°C	35.6	Yes	NCS
S125	102	JAIVAC-1	Dosage A	3	Day 365	.		26/08/2011		Diastolic Blood pressure	mm of Hg	74	Yes	NCS
S130	105	JAIVAC-1	Dosage A		Day -14 to Day -1	.		17/08/2010		Axillary temperature	°C	35.8	Yes	NCS
S130	105	JAIVAC-1	Dosage A	1	Day 7	.		02/09/2010		Axillary temperature	°C	35.5	Yes	NCS
S130	105	JAIVAC-1	Dosage A	1	Day 7	.		02/09/2010		Diastolic Blood pressure	mm of Hg	70	Yes	NCS
S130	105	JAIVAC-1	Dosage A	1	Day 14	.		08/09/2010		Axillary temperature	°C	35.8	Yes	NCS
S130	105	JAIVAC-1	Dosage A	1	Day 14	.		08/09/2010		Diastolic Blood pressure	mm of Hg	70	Yes	NCS
S130	105	JAIVAC-1	Dosage A	2	Day 28	.		23/09/2010	Yes	Diastolic Blood pressure	mm of Hg	70	Yes	NCS
S130	105	JAIVAC-1	Dosage A	2	Day 28	.		23/09/2010	Yes	Systolic Blood pressure	mm of Hg	106	Yes	NCS

Subject No.	Randomisation Number	Study Group	Dosage	Dose	Day	Not Done	Specify	Date of Assessment	*Was the assessment performed prior to vaccination?	Vital Signs Parameter	Units	Result	Out of Range	Clinical Significance
S130	105	JAIVAC-1	Dosage A	2	Day 35	.		30/09/2010		Diastolic Blood pressure	mm of Hg	64	Yes	NCS
S130	105	JAIVAC-1	Dosage A	2	Day 42	.		07/10/2010		Diastolic Blood pressure	mm of Hg	70	Yes	NCS
S130	105	JAIVAC-1	Dosage A	2	Day 56	.		21/10/2010		Axillary temperature	°C	35.8	Yes	NCS
S130	105	JAIVAC-1	Dosage A	3	Day 180	.		22/02/2011	Yes	Diastolic Blood pressure	mm of Hg	68	Yes	NCS
S130	105	JAIVAC-1	Dosage A	3	Day 187	.		01/03/2011		Axillary temperature	°C	35.5	Yes	NCS
S130	105	JAIVAC-1	Dosage A	3	Day 187	.		01/03/2011		Diastolic Blood pressure	mm of Hg	70	Yes	NCS
S130	105	JAIVAC-1	Dosage A	3	Day 194	.		08/03/2011		Axillary temperature	°C	35.8	Yes	NCS
S130	105	JAIVAC-1	Dosage A	3	Day 194	.		08/03/2011		Diastolic Blood pressure	mm of Hg	64	Yes	NCS
S130	105	JAIVAC-1	Dosage A	3	Day 208	.		22/03/2011		Diastolic Blood pressure	mm of Hg	68	Yes	NCS
S130	105	JAIVAC-1	Dosage A	3	Day 365	.		25/08/2011		Diastolic Blood pressure	mm of Hg	70	Yes	NCS
S132	108	Hepatitis B	Hepatitis B		Day -14 to Day -1	.		18/08/2010		Pulse rate	beats/ minute	90	Yes	NCS
S132	108	Hepatitis B	Hepatitis B	1	Day 7	.		02/09/2010		Diastolic Blood pressure	mm of Hg	70	Yes	NCS

Subject No.	Randomisation Number	Study Group	Dosage	Dose	Day	Not Done	Specify	Date of Assessment	*Was the assessment performed prior to vaccination?	Vital Signs Parameter	Units	Result	Out of Range	Clinical Significance
S132	108	Hepatitis B	Hepatitis B	2	Day 35	.		30/09/2010		Pulse rate	beats/ minute	86	Yes	NCS
S132	108	Hepatitis B	Hepatitis B	3	Day 187	.		01/03/2011		Axillary temperature	°C	35.5	Yes	NCS
S132	108	Hepatitis B	Hepatitis B	3	Day 208	.		22/03/2011		Diastolic Blood pressure	mm of Hg	86	Yes	NCS
S132	108	Hepatitis B	Hepatitis B	3	Day 365	.		25/08/2011		Axillary temperature	°C	35.8	Yes	NCS
S132	108	Hepatitis B	Hepatitis B	3	Day 365	.		25/08/2011		Respiratory rate	breaths/ minute	14	Yes	NCS
S136	109	JAIVAC-1	Dosage A		Day -14 to Day -1	.		19/08/2010		Pulse rate	beats/ minute	60	Yes	NCS
S136	109	JAIVAC-1	Dosage A	1	Day 0	.		26/08/2010	Yes	Axillary temperature	°C	35.2	Yes	NCS
S136	109	JAIVAC-1	Dosage A	1	Day 0	.		26/08/2010	Yes	Pulse rate	beats/ minute	60	Yes	NCS
S136	109	JAIVAC-1	Dosage A	2	Day 35	.		30/09/2010		Respiratory rate	breaths/ minute	14	Yes	NCS
S136	109	JAIVAC-1	Dosage A	2	Day 42	.		07/10/2010		Systolic Blood pressure	mm of Hg	140	Yes	NCS
S136	109	JAIVAC-1	Dosage A	3	Day 180	.		22/02/2011	Yes	Diastolic Blood pressure	mm of Hg	70	Yes	NCS
S136	109	JAIVAC-1	Dosage A	3	Day 187	.		01/03/2011		Diastolic Blood pressure	mm of Hg	70	Yes	NCS
S136	109	JAIVAC-1	Dosage A	3	Day 208	.		22/03/2011		Diastolic Blood pressure	mm of Hg	70	Yes	NCS

Subject No.	Randomisation Number	Study Group	Dosage	Dose	Day	Not Done	Specify	Date of Assessment	*Was the assessment performed prior to vaccination?	Vital Signs Parameter	Units	Result	Out of Range	Clinical Significance
S136	109	JAIVAC-1	Dosage A	3	Day 365	.		25/08/2011		Axillary temperature	°C	35.2	Yes	NCS
S136	109	JAIVAC-1	Dosage A	3	Day 365	.		25/08/2011		Diastolic Blood pressure	mm of Hg	86	Yes	NCS
S143	101	JAIVAC-1	Dosage A		Day -14 to Day -1	.		18/08/2010		Axillary temperature	°C	35.8	Yes	NCS
S143	101	JAIVAC-1	Dosage A		Day -14 to Day -1	.		18/08/2010		Pulse rate	beats/ minute	60	Yes	NCS
S143	101	JAIVAC-1	Dosage A		Day -14 to Day -1	.		18/08/2010		Systolic Blood pressure	mm of Hg	108	Yes	NCS
S143	101	JAIVAC-1	Dosage A	1	Day 0	.		24/08/2010	Yes	Diastolic Blood pressure	mm of Hg	70	Yes	NCS
S143	101	JAIVAC-1	Dosage A	1	Day 0	.		24/08/2010	Yes	Pulse rate	beats/ minute	60	Yes	NCS
S143	101	JAIVAC-1	Dosage A	1	Day 14	.		07/09/2010		Axillary temperature	°C	35.7	Yes	NCS
S143	101	JAIVAC-1	Dosage A	1	Day 14	.		07/09/2010		Diastolic Blood pressure	mm of Hg	70	Yes	NCS
S143	101	JAIVAC-1	Dosage A	2	Day 35	.		28/09/2010		Axillary temperature	°C	35.8	Yes	NCS
S143	101	JAIVAC-1	Dosage A	2	Day 35	.		28/09/2010		Diastolic Blood pressure	mm of Hg	74	Yes	NCS
S143	101	JAIVAC-1	Dosage A	2	Day 42	.		05/10/2010		Axillary temperature	°C	35.5	Yes	NCS

Subject No.	Randomisation Number	Study Group	Dosage	Dose	Day	Not Done	Specify	Date of Assessment	*Was the assessment performed prior to vaccination?	Vital Signs Parameter	Units	Result	Out of Range	Clinical Significance
S143	101	JAIVAC-1	Dosage A	3	Day 180	.		21/02/2011	Yes	Diastolic Blood pressure	mm of Hg	74	Yes	NCS
S143	101	JAIVAC-1	Dosage A	3	Day 208	.		21/03/2011		Diastolic Blood pressure	mm of Hg	68	Yes	NCS
S143	101	JAIVAC-1	Dosage A	3	Day 365	.		26/08/2011		Axillary temperature	°C	35.4	Yes	NCS
S143	101	JAIVAC-1	Dosage A	3	Day 365	.		26/08/2011		Diastolic Blood pressure	mm of Hg	86	Yes	NCS
S144	103	Hepatitis B	Hepatitis B	1	Day 0	.		24/08/2010	Yes	Diastolic Blood pressure	mm of Hg	88	Yes	NCS
S144	103	Hepatitis B	Hepatitis B	1	Day 0	.		24/08/2010	Yes	Pulse rate	beats/ minute	90	Yes	NCS
S144	103	Hepatitis B	Hepatitis B	1	Day 7	.		31/08/2010		Axillary temperature	°C	35.9	Yes	NCS
S144	103	Hepatitis B	Hepatitis B	1	Day 7	.		31/08/2010		Diastolic Blood pressure	mm of Hg	70	Yes	NCS
S144	103	Hepatitis B	Hepatitis B	1	Day 14	.		07/09/2010		Axillary temperature	°C	35.7	Yes	NCS
S144	103	Hepatitis B	Hepatitis B	1	Day 14	.		07/09/2010		Diastolic Blood pressure	mm of Hg	74	Yes	NCS
S144	103	Hepatitis B	Hepatitis B	2	Day 28	.		21/09/2010	Yes	Pulse rate	beats/ minute	90	Yes	NCS
S144	103	Hepatitis B	Hepatitis B	2	Day 35	.		28/09/2010		Axillary temperature	°C	35.7	Yes	NCS

Subject No.	Randomisation Number	Study Group	Dosage	Dose	Day	Not Done	Specify	Date of Assessment	*Was the assessment performed prior to vaccination?	Vital Signs Parameter	Units	Result	Out of Range	Clinical Significance
S144	103	Hepatitis B	Hepatitis B	2	Day 35	.		28/09/2010		Diastolic Blood pressure	mm of Hg	70	Yes	NCS
S144	103	Hepatitis B	Hepatitis B	2	Day 42	.		05/10/2010		Axillary temperature	°C	35.9	Yes	NCS
S144	103	Hepatitis B	Hepatitis B	2	Day 42	.		05/10/2010		Diastolic Blood pressure	mm of Hg	70	Yes	NCS
S144	103	Hepatitis B	Hepatitis B	2	Day 56	.		19/10/2010		Axillary temperature	°C	35.8	Yes	NCS
S144	103	Hepatitis B	Hepatitis B	3	Day 187	.		28/02/2011		Axillary temperature	°C	35.6	Yes	NCS
S144	103	Hepatitis B	Hepatitis B	3	Day 187	.		28/02/2011		Diastolic Blood pressure	mm of Hg	70	Yes	NCS
S144	103	Hepatitis B	Hepatitis B	3	Day 194	.		07/03/2011		Diastolic Blood pressure	mm of Hg	70	Yes	NCS
S144	103	Hepatitis B	Hepatitis B	3	Day 208	.		21/03/2011		Diastolic Blood pressure	mm of Hg	70	Yes	NCS
S144	103	Hepatitis B	Hepatitis B	3	Day 365	.		26/08/2011		Axillary temperature	°C	35.8	Yes	NCS
S144	103	Hepatitis B	Hepatitis B	3	Day 365	.		26/08/2011		Diastolic Blood pressure	mm of Hg	86	Yes	NCS
S145	112	JAIVAC-1	Dosage A		Day -14 to Day -1	.		19/08/2010		Diastolic Blood pressure	mm of Hg	70	Yes	NCS
S145	112	JAIVAC-1	Dosage A		Day -14 to Day -1	.		19/08/2010		Systolic Blood pressure	mm of Hg	100	Yes	NCS

Subject No.	Randomisation Number	Study Group	Dosage	Dose	Day	Not Done	Specify	Date of Assessment	*Was the assessment performed prior to vaccination?	Vital Signs Parameter	Units	Result	Out of Range	Clinical Significance
S145	112	JAIVAC-1	Dosage A	1	Day 0	.		27/08/2010	Yes	Pulse rate	beats/ minute	90	Yes	NCS
S145	112	JAIVAC-1	Dosage A	1	Day 7	.		04/09/2010		Axillary temperature	°C	35.9	Yes	NCS
S145	112	JAIVAC-1	Dosage A	1	Day 14	.		10/09/2010		Diastolic Blood pressure	mm of Hg	70	Yes	NCS
S145	112	JAIVAC-1	Dosage A	1	Day 14	.		10/09/2010		Systolic Blood pressure	mm of Hg	100	Yes	NCS
S145	112	JAIVAC-1	Dosage A	2	Day 28	.		24/09/2010	Yes	Diastolic Blood pressure	mm of Hg	70	Yes	NCS
S145	112	JAIVAC-1	Dosage A	2	Day 28	.		24/09/2010	Yes	Systolic Blood pressure	mm of Hg	106	Yes	NCS
S145	112	JAIVAC-1	Dosage A	2	Day 35	.		30/09/2010		Diastolic Blood pressure	mm of Hg	74	Yes	NCS
S145	112	JAIVAC-1	Dosage A	2	Day 42	.		08/10/2010		Diastolic Blood pressure	mm of Hg	70	Yes	NCS
S145	112	JAIVAC-1	Dosage A	2	Day 56	.		22/10/2010		Diastolic Blood pressure	mm of Hg	70	Yes	NCS
S145	112	JAIVAC-1	Dosage A	3	Day 180	.		23/02/2011	Yes	Diastolic Blood pressure	mm of Hg	70	Yes	NCS
S145	112	JAIVAC-1	Dosage A	3	Day 187	.		02/03/2011		Diastolic Blood pressure	mm of Hg	68	Yes	NCS
S145	112	JAIVAC-1	Dosage A	3	Day 194	.		09/03/2011		Diastolic Blood pressure	mm of Hg	72	Yes	NCS

Subject No.	Randomisation Number	Study Group	Dosage	Dose	Day	Not Done	Specify	Date of Assessment	*Was the assessment performed prior to vaccination?	Vital Signs Parameter	Units	Result	Out of Range	Clinical Significance
S145	112	JAIVAC-1	Dosage A	3	Day 208	.		23/03/2011		Diastolic Blood pressure	mm of Hg	70	Yes	NCS
S145	112	JAIVAC-1	Dosage A	3	Day 365	.		26/08/2011		Axillary temperature	°C	35.4	Yes	NCS
S145	112	JAIVAC-1	Dosage A	3	Day 365	.		26/08/2011		Diastolic Blood pressure	mm of Hg	86	Yes	NCS
S145	112	JAIVAC-1	Dosage A	3	Day 365	.		26/08/2011		Respiratory rate	breaths/ minute	14	Yes	NCS
S148	111	JAIVAC-1	Dosage A	1	Day 0	.		27/08/2010	Yes	Axillary temperature	°C	35.2	Yes	NCS
S148	111	JAIVAC-1	Dosage A	1	Day 7	.		03/09/2010		Diastolic Blood pressure	mm of Hg	70	Yes	NCS
S148	111	JAIVAC-1	Dosage A	1	Day 14	.		10/09/2010		Diastolic Blood pressure	mm of Hg	70	Yes	NCS
S148	111	JAIVAC-1	Dosage A	2	Day 28	.		24/09/2010	Yes	Axillary temperature	°C	35.8	Yes	NCS
S148	111	JAIVAC-1	Dosage A	2	Day 35	.		30/09/2010		Axillary temperature	°C	35.3	Yes	NCS
S148	111	JAIVAC-1	Dosage A	2	Day 35	.		30/09/2010		Diastolic Blood pressure	mm of Hg	70	Yes	NCS
S148	111	JAIVAC-1	Dosage A	2	Day 42	.		08/10/2010		Respiratory rate	breaths/ minute	14	Yes	NCS
S148	111	JAIVAC-1	Dosage A	2	Day 56	.		22/10/2010		Axillary temperature	°C	35.7	Yes	NCS

Subject No.	Randomisation Number	Study Group	Dosage	Dose	Day	Not Done	Specify	Date of Assessment	*Was the assessment performed prior to vaccination?	Vital Signs Parameter	Units	Result	Out of Range	Clinical Significance
S148	111	JAIVAC-1	Dosage A	3	Day 180	.		23/02/2011	Yes	Diastolic Blood pressure	mm of Hg	72	Yes	NCS
S148	111	JAIVAC-1	Dosage A	3	Day 194	.		09/03/2011		Axillary temperature	°C	35.8	Yes	NCS
S148	111	JAIVAC-1	Dosage A	3	Day 194	.		09/03/2011		Diastolic Blood pressure	mm of Hg	64	Yes	NCS
S148	111	JAIVAC-1	Dosage A	3	Day 208	.		23/03/2011		Axillary temperature	°C	35.1	Yes	NCS
S148	111	JAIVAC-1	Dosage A	3	Day 208	.		23/03/2011		Diastolic Blood pressure	mm of Hg	68	Yes	NCS
S148	111	JAIVAC-1	Dosage A	3	Day 365	.		26/08/2011		Diastolic Blood pressure	mm of Hg	88	Yes	NCS
S154	113	JAIVAC-1	Dosage A		Day -14 to Day -1	.		19/08/2010		Axillary temperature	°C	35.8	Yes	NCS
S154	113	JAIVAC-1	Dosage A		Day -14 to Day -1	.		19/08/2010		Diastolic Blood pressure	mm of Hg	74	Yes	NCS
S154	113	JAIVAC-1	Dosage A	1	Day 0	.		27/08/2010	Yes	Axillary temperature	°C	35.4	Yes	NCS
S154	113	JAIVAC-1	Dosage A	1	Day 14	.		09/09/2010		Diastolic Blood pressure	mm of Hg	70	Yes	NCS
S154	113	JAIVAC-1	Dosage A	2	Day 28	.		24/09/2010	Yes	Axillary temperature	°C	35.5	Yes	NCS
S154	113	JAIVAC-1	Dosage A	2	Day 42	.		08/10/2010		Axillary temperature	°C	35.7	Yes	NCS

Subject No.	Randomisation Number	Study Group	Dosage	Dose	Day	Not Done	Specify	Date of Assessment	*Was the assessment performed prior to vaccination?	Vital Signs Parameter	Units	Result	Out of Range	Clinical Significance
S154	113	JAIVAC-1	Dosage A	2	Day 42	.		08/10/2010		Respiratory rate	breaths/ minute	14	Yes	NCS
S154	113	JAIVAC-1	Dosage A	2	Day 56	.		22/10/2010		Respiratory rate	breaths/ minute	14	Yes	NCS
S154	113	JAIVAC-1	Dosage A	3	Day 180	.		23/02/2011	Yes	Diastolic Blood pressure	mm of Hg	74	Yes	NCS
S154	113	JAIVAC-1	Dosage A	3	Day 187	.		01/03/2011		Diastolic Blood pressure	mm of Hg	68	Yes	NCS
S154	113	JAIVAC-1	Dosage A	3	Day 194	.		09/03/2011		Diastolic Blood pressure	mm of Hg	68	Yes	NCS
S154	113	JAIVAC-1	Dosage A	3	Day 208	.		23/03/2011		Axillary temperature	°C	35.2	Yes	NCS
S154	113	JAIVAC-1	Dosage A	3	Day 208	.		23/03/2011		Diastolic Blood pressure	mm of Hg	74	Yes	NCS
S154	113	JAIVAC-1	Dosage A	3	Day 365	.		26/08/2011		Axillary temperature	°C	35.1	Yes	NCS
S156	110	JAIVAC-1	Dosage A	1	Day 0	.		27/08/2010	Yes	Axillary temperature	°C	35.8	Yes	NCS
S156	110	JAIVAC-1	Dosage A	1	Day 7	.		03/09/2010		Axillary temperature	°C	35.6	Yes	NCS
S156	110	JAIVAC-1	Dosage A	1	Day 7	.		03/09/2010		Diastolic Blood pressure	mm of Hg	70	Yes	NCS
S156	110	JAIVAC-1	Dosage A	1	Day 14	.		09/09/2010		Axillary temperature	°C	35.9	Yes	NCS

Subject No.	Randomisation Number	Study Group	Dosage	Dose	Day	Not Done	Specify	Date of Assessment	*Was the assessment performed prior to vaccination?	Vital Signs Parameter	Units	Result	Out of Range	Clinical Significance
S156	110	JAIVAC-1	Dosage A	1	Day 14	.		09/09/2010		Diastolic Blood pressure	mm of Hg	70	Yes	NCS
S156	110	JAIVAC-1	Dosage A	1	Day 14	.		09/09/2010		Systolic Blood pressure	mm of Hg	104	Yes	NCS
S156	110	JAIVAC-1	Dosage A	2	Day 42	.		08/10/2010		Axillary temperature	°C	35.9	Yes	NCS
S156	110	JAIVAC-1	Dosage A	2	Day 42	.		08/10/2010		Diastolic Blood pressure	mm of Hg	64	Yes	NCS
S156	110	JAIVAC-1	Dosage A	2	Day 42	.		08/10/2010		Respiratory rate	breaths/ minute	14	Yes	NCS
S156	110	JAIVAC-1	Dosage A	2	Day 42	.		08/10/2010		Systolic Blood pressure	mm of Hg	104	Yes	NCS
S156	110	JAIVAC-1	Dosage A	2	Day 56	.		22/10/2010		Axillary temperature	°C	35.5	Yes	NCS
S156	110	JAIVAC-1	Dosage A	2	Day 56	.		22/10/2010		Diastolic Blood pressure	mm of Hg	74	Yes	NCS
S156	110	JAIVAC-1	Dosage A	3	Day 180	.		23/02/2011	Yes	Diastolic Blood pressure	mm of Hg	68	Yes	NCS
S156	110	JAIVAC-1	Dosage A	3	Day 187	.		02/03/2011		Diastolic Blood pressure	mm of Hg	74	Yes	NCS
S156	110	JAIVAC-1	Dosage A	3	Day 194	.		09/03/2011		Diastolic Blood pressure	mm of Hg	66	Yes	NCS
S156	110	JAIVAC-1	Dosage A	3	Day 208	.		23/03/2011		Diastolic Blood pressure	mm of Hg	70	Yes	NCS

Subject No.	Randomisation Number	Study Group	Dosage	Dose	Day	Not Done	Specify	Date of Assessment	*Was the assessment performed prior to vaccination?	Vital Signs Parameter	Units	Result	Out of Range	Clinical Significance
S156	110	JAIVAC-1	Dosage A	3	Day 365	.		26/08/2011		Diastolic Blood pressure	mm of Hg	70	Yes	NCS
S170	204	Hepatitis B	Hepatitis B	1	Day 7	.		06/10/2010		Diastolic Blood pressure	mm of Hg	64	Yes	NCS
S170	204	Hepatitis B	Hepatitis B	1	Day 7	.		06/10/2010		Respiratory rate	breaths/ minute	14	Yes	NCS
S170	204	Hepatitis B	Hepatitis B	1	Day 7	.		06/10/2010		Systolic Blood pressure	mm of Hg	104	Yes	NCS
S170	204	Hepatitis B	Hepatitis B	2	Day 35	.		03/11/2010		Diastolic Blood pressure	mm of Hg	70	Yes	NCS
S170	204	Hepatitis B	Hepatitis B	2	Day 35	.		03/11/2010		Pulse rate	beats/ minute	60	Yes	NCS
S170	204	Hepatitis B	Hepatitis B	2	Day 42	.		10/11/2010		Diastolic Blood pressure	mm of Hg	70	Yes	NCS
S170	204	Hepatitis B	Hepatitis B	3	Day 180	.		28/03/2011	Yes	Diastolic Blood pressure	mm of Hg	74	Yes	NCS
S170	204	Hepatitis B	Hepatitis B	3	Day 180	.		28/03/2011	Yes	Systolic Blood pressure	mm of Hg	108	Yes	NCS
S170	204	Hepatitis B	Hepatitis B	3	Day 187	.		06/04/2011		Axillary temperature	°C	35.9	Yes	NCS
S170	204	Hepatitis B	Hepatitis B	3	Day 187	.		06/04/2011		Diastolic Blood pressure	mm of Hg	70	Yes	NCS
S170	204	Hepatitis B	Hepatitis B	3	Day 187	.		06/04/2011		Respiratory rate	breaths/ minute	14	Yes	NCS
S170	204	Hepatitis B	Hepatitis B	3	Day 194	.		11/04/2011		Diastolic Blood pressure	mm of Hg	70	Yes	NCS

Subject No.	Randomisation Number	Study Group	Dosage	Dose	Day	Not Done	Specify	Date of Assessment	*Was the assessment performed prior to vaccination?	Vital Signs Parameter	Units	Result	Out of Range	Clinical Significance
S170	204	Hepatitis B	Hepatitis B	3	Day 208	.		25/04/2011		Axillary temperature	°C	35.5	Yes	NCS
S170	204	Hepatitis B	Hepatitis B	3	Day 365	.		28/09/2011		Diastolic Blood pressure	mm of Hg	74	Yes	NCS
S175	209	JAIVAC-1	Dosage B		Day -14 to Day -1	.		21/09/2010		Axillary temperature	°C	35.5	Yes	NCS
S175	209	JAIVAC-1	Dosage B		Day -14 to Day -1	.		21/09/2010		Pulse rate	beats/ minute	60	Yes	NCS
S175	209	JAIVAC-1	Dosage B	2	Day 35	.		03/11/2010		Axillary temperature	°C	35.6	Yes	NCS
S175	209	JAIVAC-1	Dosage B	2	Day 35	.		03/11/2010		Diastolic Blood pressure	mm of Hg	70	Yes	NCS
S175	209	JAIVAC-1	Dosage B	2	Day 42	.		10/11/2010		Axillary temperature	°C	35.4	Yes	NCS
S175	209	JAIVAC-1	Dosage B	3	Day 180	.		28/03/2011	Yes	Diastolic Blood pressure	mm of Hg	70	Yes	NCS
S175	209	JAIVAC-1	Dosage B	3	Day 187	.		05/04/2011		Diastolic Blood pressure	mm of Hg	70	Yes	NCS
S175	209	JAIVAC-1	Dosage B	3	Day 194	.		11/04/2011		Axillary temperature	°C	35.9	Yes	NCS
S175	209	JAIVAC-1	Dosage B	3	Day 194	.		11/04/2011		Diastolic Blood pressure	mm of Hg	74	Yes	NCS
S175	209	JAIVAC-1	Dosage B	3	Day 208	.		25/04/2011		Axillary temperature	°C	35.4	Yes	NCS

Subject No.	Randomisation Number	Study Group	Dosage	Dose	Day	Not Done	Specify	Date of Assessment	*Was the assessment performed prior to vaccination?	Vital Signs Parameter	Units	Result	Out of Range	Clinical Significance
S175	209	JAIVAC-1	Dosage B	3	Day 365	.		28/09/2011		Axillary temperature	°C	35.7	Yes	NCS
S175	209	JAIVAC-1	Dosage B	3	Day 365	.		28/09/2011		Diastolic Blood pressure	mm of Hg	72	Yes	NCS
S176	205	Hepatitis B	Hepatitis B	1	Day 0	.		29/09/2010	Yes	Axillary temperature	°C	35.7	Yes	NCS
S176	205	Hepatitis B	Hepatitis B	1	Day 7	.		06/10/2010		Axillary temperature	°C	35.8	Yes	NCS
S176	205	Hepatitis B	Hepatitis B	1	Day 7	.		06/10/2010		Diastolic Blood pressure	mm of Hg	74	Yes	NCS
S176	205	Hepatitis B	Hepatitis B	1	Day 7	.		06/10/2010		Pulse rate	beats/ minute	86	Yes	NCS
S176	205	Hepatitis B	Hepatitis B	2	Day 28	.		27/10/2010	Yes	Axillary temperature	°C	35.6	Yes	NCS
S176	205	Hepatitis B	Hepatitis B	2	Day 56	.		24/11/2010		Axillary temperature	°C	35.9	Yes	NCS
S176	205	Hepatitis B	Hepatitis B	3	Day 180	.		28/03/2011	Yes	Axillary temperature	°C	35.4	Yes	NCS
S176	205	Hepatitis B	Hepatitis B	3	Day 180	.		28/03/2011	Yes	Diastolic Blood pressure	mm of Hg	70	Yes	NCS
S176	205	Hepatitis B	Hepatitis B	3	Day 187	.		05/04/2011		Axillary temperature	°C	35.7	Yes	NCS
S179	207	Hepatitis B	Hepatitis B	1	Day 0	.		29/09/2010	Yes	Axillary temperature	°C	35	Yes	NCS

Subject No.	Randomisation Number	Study Group	Dosage	Dose	Day	Not Done	Specify	Date of Assessment	*Was the assessment performed prior to vaccination?	Vital Signs Parameter	Units	Result	Out of Range	Clinical Significance
S179	207	Hepatitis B	Hepatitis B	1	Day 7	.		06/10/2010		Diastolic Blood pressure	mm of Hg	70	Yes	NCS
S179	207	Hepatitis B	Hepatitis B	2	Day 28	.		27/10/2010	Yes	Axillary temperature	°C	35.5	Yes	NCS
S179	207	Hepatitis B	Hepatitis B	2	Day 35	.		03/11/2010		Diastolic Blood pressure	mm of Hg	70	Yes	NCS
S179	207	Hepatitis B	Hepatitis B	3	Day 180	.		28/03/2011	Yes	Diastolic Blood pressure	mm of Hg	74	Yes	NCS
S179	207	Hepatitis B	Hepatitis B	3	Day 187	.		06/04/2011		Axillary temperature	°C	35.6	Yes	NCS
S179	207	Hepatitis B	Hepatitis B	3	Day 187	.		06/04/2011		Diastolic Blood pressure	mm of Hg	74	Yes	NCS
S179	207	Hepatitis B	Hepatitis B	3	Day 194	.		11/04/2011		Diastolic Blood pressure	mm of Hg	70	Yes	NCS
S179	207	Hepatitis B	Hepatitis B	3	Day 208	.		25/04/2011		Diastolic Blood pressure	mm of Hg	70	Yes	NCS
S179	207	Hepatitis B	Hepatitis B	3	Day 365	.		28/09/2011		Axillary temperature	°C	35.7	Yes	NCS
S179	207	Hepatitis B	Hepatitis B	3	Day 365	.		28/09/2011		Respiratory rate	breaths/ minute	14	Yes	NCS
S182	208	JAIVAC-1	Dosage B	1	Day 0	.		29/09/2010	Yes	Axillary temperature	°C	35.8	Yes	NCS
S182	208	JAIVAC-1	Dosage B	1	Day 7	.		06/10/2010		Diastolic Blood pressure	mm of Hg	70	Yes	NCS

Subject No.	Randomisation Number	Study Group	Dosage	Dose	Day	Not Done	Specify	Date of Assessment	*Was the assessment performed prior to vaccination?	Vital Signs Parameter	Units	Result	Out of Range	Clinical Significance
S182	208	JAIVAC-1	Dosage B	1	Day 14	.		13/10/2010		Diastolic Blood pressure	mm of Hg	70	Yes	NCS
S182	208	JAIVAC-1	Dosage B	2	Day 35	.		03/11/2010		Pulse rate	beats/ minute	86	Yes	NCS
S182	208	JAIVAC-1	Dosage B	2	Day 42	.		10/11/2010		Respiratory rate	breaths/ minute	14	Yes	NCS
S182	208	JAIVAC-1	Dosage B	2	Day 56	.		24/11/2010		Pulse rate	beats/ minute	86	Yes	NCS
S182	208	JAIVAC-1	Dosage B	3	Day 180	.		28/03/2011	Yes	Axillary temperature	°C	35.8	Yes	NCS
S182	208	JAIVAC-1	Dosage B	3	Day 180	.		28/03/2011	Yes	Diastolic Blood pressure	mm of Hg	64	Yes	NCS
S182	208	JAIVAC-1	Dosage B	3	Day 187	.		05/04/2011		Diastolic Blood pressure	mm of Hg	68	Yes	NCS
S182	208	JAIVAC-1	Dosage B	3	Day 187	.		05/04/2011		Systolic Blood pressure	mm of Hg	106	Yes	NCS
S182	208	JAIVAC-1	Dosage B	3	Day 194	.		11/04/2011		Diastolic Blood pressure	mm of Hg	70	Yes	NCS
S182	208	JAIVAC-1	Dosage B	3	Day 365	.		29/09/2011		Axillary temperature	°C	35.9	Yes	NCS
S182	208	JAIVAC-1	Dosage B	3	Day 365	.		29/09/2011		Respiratory rate	breaths/ minute	14	Yes	NCS
S185	202	JAIVAC-1	Dosage B		Day -14 to Day -1	.		21/09/2010		Axillary temperature	°C	35.4	Yes	NCS
S185	202	JAIVAC-1	Dosage B	1	Day 7	.		05/10/2010		Axillary temperature	°C	35.9	Yes	NCS

Subject No.	Randomisation Number	Study Group	Dosage	Dose	Day	Not Done	Specify	Date of Assessment	*Was the assessment performed prior to vaccination?	Vital Signs Parameter	Units	Result	Out of Range	Clinical Significance
S185	202	JAIVAC-1	Dosage B	1	Day 7	.		05/10/2010		Respiratory rate	breaths/ minute	14	Yes	NCS
S185	202	JAIVAC-1	Dosage B	2	Day 56	.		23/11/2010		Diastolic Blood pressure	mm of Hg	70	Yes	NCS
S185	202	JAIVAC-1	Dosage B	3	Day 180	.		28/03/2011	Yes	Diastolic Blood pressure	mm of Hg	70	Yes	NCS
S185	202	JAIVAC-1	Dosage B	3	Day 194	.		11/04/2011		Diastolic Blood pressure	mm of Hg	64	Yes	NCS
S185	202	JAIVAC-1	Dosage B	3	Day 208	.		25/04/2011		Diastolic Blood pressure	mm of Hg	70	Yes	NCS
S185	202	JAIVAC-1	Dosage B	3	Day 365	.		28/09/2011		Diastolic Blood pressure	mm of Hg	70	Yes	NCS
S185	202	JAIVAC-1	Dosage B	3	Day 365	.		28/09/2011		Respiratory rate	breaths/ minute	14	Yes	NCS
S186	201	JAIVAC-1	Dosage B	1	Day 0	.		28/09/2010	Yes	Diastolic Blood pressure	mm of Hg	88	Yes	NCS
S186	201	JAIVAC-1	Dosage B	1	Day 7	.		05/10/2010		Pulse rate	beats/ minute	90	Yes	NCS
S186	201	JAIVAC-1	Dosage B	2	Day 28	.		26/10/2010	Yes	Diastolic Blood pressure	mm of Hg	86	Yes	NCS
S186	201	JAIVAC-1	Dosage B	2	Day 28	.		26/10/2010	Yes	Systolic Blood pressure	mm of Hg	134	Yes	NCS
S186	201	JAIVAC-1	Dosage B	3	Day 365	.		28/09/2011		Axillary temperature	°C	35.9	Yes	NCS
S186	201	JAIVAC-1	Dosage B	3	Day 365	.		28/09/2011		Diastolic Blood pressure	mm of Hg	86	Yes	NCS

Subject No.	Randomisation Number	Study Group	Dosage	Dose	Day	Not Done	Specify	Date of Assessment	*Was the assessment performed prior to vaccination?	Vital Signs Parameter	Units	Result	Out of Range	Clinical Significance
S192	203	JAIVAC-1	Dosage B		Day -14 to Day -1	.		23/09/2010		Axillary temperature	°C	35.5	Yes	NCS
S192	203	JAIVAC-1	Dosage B		Day -14 to Day -1	.		23/09/2010		Diastolic Blood pressure	mm of Hg	70	Yes	NCS
S192	203	JAIVAC-1	Dosage B		Day -14 to Day -1	.		23/09/2010		Systolic Blood pressure	mm of Hg	100	Yes	NCS
S192	203	JAIVAC-1	Dosage B	1	Day 0	.		28/09/2010	Yes	Axillary temperature	°C	35.6	Yes	NCS
S192	203	JAIVAC-1	Dosage B	1	Day 0	.		28/09/2010	Yes	Systolic Blood pressure	mm of Hg	106	Yes	NCS
S192	203	JAIVAC-1	Dosage B	1	Day 7	.		05/10/2010		Respiratory rate	breaths/ minute	14	Yes	NCS
S192	203	JAIVAC-1	Dosage B	2	Day 28	.		26/10/2010	Yes	Axillary temperature	°C	35.5	Yes	NCS
S192	203	JAIVAC-1	Dosage B	2	Day 28	.		26/10/2010	Yes	Diastolic Blood pressure	mm of Hg	70	Yes	NCS
S192	203	JAIVAC-1	Dosage B	2	Day 28	.		26/10/2010	Yes	Systolic Blood pressure	mm of Hg	96	Yes	NCS
S192	203	JAIVAC-1	Dosage B	2	Day 35	.		02/11/2010		Diastolic Blood pressure	mm of Hg	70	Yes	NCS
S192	203	JAIVAC-1	Dosage B	2	Day 42	.		09/11/2010		Diastolic Blood pressure	mm of Hg	70	Yes	NCS
S192	203	JAIVAC-1	Dosage B	2	Day 42	.		09/11/2010		Respiratory rate	breaths/ minute	14	Yes	NCS

Subject No.	Randomisation Number	Study Group	Dosage	Dose	Day	Not Done	Specify	Date of Assessment	*Was the assessment performed prior to vaccination?	Vital Signs Parameter	Units	Result	Out of Range	Clinical Significance
S192	203	JAIVAC-1	Dosage B	2	Day 56	.		23/11/2010		Diastolic Blood pressure	mm of Hg	70	Yes	NCS
S192	203	JAIVAC-1	Dosage B	3	Day 180	.		28/03/2011	Yes	Diastolic Blood pressure	mm of Hg	70	Yes	NCS
S192	203	JAIVAC-1	Dosage B	3	Day 187	.		02/04/2011		Diastolic Blood pressure	mm of Hg	70	Yes	NCS
S192	203	JAIVAC-1	Dosage B	3	Day 187	.		02/04/2011		Respiratory rate	breaths/ minute	14	Yes	NCS
S192	203	JAIVAC-1	Dosage B	3	Day 194	.		11/04/2011		Axillary temperature	°C	35.9	Yes	NCS
S192	203	JAIVAC-1	Dosage B	3	Day 194	.		11/04/2011		Diastolic Blood pressure	mm of Hg	74	Yes	NCS
S192	203	JAIVAC-1	Dosage B	3	Day 208	.		25/04/2011		Axillary temperature	°C	35.9	Yes	NCS
S192	203	JAIVAC-1	Dosage B	3	Day 365	.		28/09/2011		Axillary temperature	°C	35.8	Yes	NCS
S193	215	JAIVAC-1	Dosage B		Day -14 to Day -1	.		24/09/2010		Axillary temperature	°C	35.2	Yes	NCS
S193	215	JAIVAC-1	Dosage B	1	Day 0	.		30/09/2010	Yes	Axillary temperature	°C	35.1	Yes	NCS
S193	215	JAIVAC-1	Dosage B	1	Day 7	.		07/10/2010		Diastolic Blood pressure	mm of Hg	90	Yes	NCS
S193	215	JAIVAC-1	Dosage B	1	Day 7	.		07/10/2010		Systolic Blood pressure	mm of Hg	140	Yes	NCS

Subject No.	Randomisation Number	Study Group	Dosage	Dose	Day	Not Done	Specify	Date of Assessment	*Was the assessment performed prior to vaccination?	Vital Signs Parameter	Units	Result	Out of Range	Clinical Significance
S193	215	JAIVAC-1	Dosage B	1	Day 14	.		14/10/2010		Respiratory rate	breaths/ minute	14	Yes	NCS
S193	215	JAIVAC-1	Dosage B	2	Day 35	.		04/11/2010		Axillary temperature	°C	35.7	Yes	NCS
S193	215	JAIVAC-1	Dosage B	2	Day 42	.		11/11/2010		Axillary temperature	°C	35.5	Yes	NCS
S193	215	JAIVAC-1	Dosage B	2	Day 42	.		11/11/2010		Systolic Blood pressure	mm of Hg	134	Yes	NCS
S193	215	JAIVAC-1	Dosage B	2	Day 56	.		25/11/2010		Axillary temperature	°C	35.5	Yes	NCS
S193	215	JAIVAC-1	Dosage B	2	Day 56	.		25/11/2010		Diastolic Blood pressure	mm of Hg	70	Yes	NCS
S193	215	JAIVAC-1	Dosage B	3	Day 180	.		29/03/2011	Yes	Axillary temperature	°C	35.3	Yes	NCS
S193	215	JAIVAC-1	Dosage B	3	Day 187	.		05/04/2011		Systolic Blood pressure	mm of Hg	134	Yes	NCS
S193	215	JAIVAC-1	Dosage B	3	Day 194	.		12/04/2011		Axillary temperature	°C	34.5	Yes	NCS
S193	215	JAIVAC-1	Dosage B	3	Day 208	.		26/04/2011		Axillary temperature	°C	35.3	Yes	NCS
S193	215	JAIVAC-1	Dosage B	3	Day 208	.		26/04/2011		Diastolic Blood pressure	mm of Hg	90	Yes	NCS
S193	215	JAIVAC-1	Dosage B	3	Day 208	.		26/04/2011		Systolic Blood pressure	mm of Hg	134	Yes	NCS

Subject No.	Randomisation Number	Study Group	Dosage	Dose	Day	Not Done	Specify	Date of Assessment	*Was the assessment performed prior to vaccination?	Vital Signs Parameter	Units	Result	Out of Range	Clinical Significance
S193	215	JAIVAC-1	Dosage B	3	Day 365	.		29/09/2011		Axillary temperature	°C	35.8	Yes	NCS
S193	215	JAIVAC-1	Dosage B	3	Day 365	.		29/09/2011		Diastolic Blood pressure	mm of Hg	86	Yes	NCS
S195	214	JAIVAC-1	Dosage B		Day -14 to Day -1	.		23/09/2010		Respiratory rate	breaths/ minute	14	Yes	NCS
S195	214	JAIVAC-1	Dosage B	1	Day 14	.		14/10/2010		Diastolic Blood pressure	mm of Hg	70	Yes	NCS
S195	214	JAIVAC-1	Dosage B	2	Day 28	.		28/10/2010	Yes	Pulse rate	beats/ minute	60	Yes	NCS
S195	214	JAIVAC-1	Dosage B	2	Day 35	.		04/11/2010		Diastolic Blood pressure	mm of Hg	74	Yes	NCS
S195	214	JAIVAC-1	Dosage B	2	Day 42	.		11/11/2010		Diastolic Blood pressure	mm of Hg	70	Yes	NCS
S195	214	JAIVAC-1	Dosage B	2	Day 56	.		24/11/2010		Axillary temperature	°C	35	Yes	NCS
S195	214	JAIVAC-1	Dosage B	3	Day 180	.		29/03/2011	Yes	Diastolic Blood pressure	mm of Hg	64	Yes	NCS
S195	214	JAIVAC-1	Dosage B	3	Day 187	.		05/04/2011		Diastolic Blood pressure	mm of Hg	70	Yes	NCS
S195	214	JAIVAC-1	Dosage B	3	Day 194	.		12/04/2011		Diastolic Blood pressure	mm of Hg	72	Yes	NCS
S195	214	JAIVAC-1	Dosage B	3	Day 208	.		26/04/2011		Axillary temperature	°C	35.9	Yes	NCS

Subject No.	Randomisation Number	Study Group	Dosage	Dose	Day	Not Done	Specify	Date of Assessment	*Was the assessment performed prior to vaccination?	Vital Signs Parameter	Units	Result	Out of Range	Clinical Significance
S195	214	JAIVAC-1	Dosage B	3	Day 365	.		29/09/2011		Diastolic Blood pressure	mm of Hg	74	Yes	NCS
S202	206	JAIVAC-1	Dosage B		Day -14 to Day -1	.		23/09/2010		Diastolic Blood pressure	mm of Hg	86	Yes	NCS
S202	206	JAIVAC-1	Dosage B		Day -14 to Day -1	.		23/09/2010		Systolic Blood pressure	mm of Hg	136	Yes	NCS
S202	206	JAIVAC-1	Dosage B	1	Day 0	.		29/09/2010	Yes	Axillary temperature	°C	35.7	Yes	NCS
S202	206	JAIVAC-1	Dosage B	1	Day 7	.		07/10/2010		Axillary temperature	°C	35.6	Yes	NCS
S202	206	JAIVAC-1	Dosage B	1	Day 7	.		07/10/2010		Diastolic Blood pressure	mm of Hg	90	Yes	NCS
S202	206	JAIVAC-1	Dosage B	2	Day 28	.		27/10/2010	Yes	Axillary temperature	°C	35.6	Yes	NCS
S202	206	JAIVAC-1	Dosage B	2	Day 35	.		03/11/2010		Axillary temperature	°C	35.8	Yes	NCS
S202	206	JAIVAC-1	Dosage B	2	Day 42	.		10/11/2010		Axillary temperature	°C	35.1	Yes	NCS
S202	206	JAIVAC-1	Dosage B	2	Day 42	.		10/11/2010		Diastolic Blood pressure	mm of Hg	90	Yes	NCS
S202	206	JAIVAC-1	Dosage B	2	Day 56	.		24/11/2010		Axillary temperature	°C	35.8	Yes	NCS
S202	206	JAIVAC-1	Dosage B	3	Day 180	.		28/03/2011	Yes	Axillary temperature	°C	35.4	Yes	NCS

Subject No.	Randomisation Number	Study Group	Dosage	Dose	Day	Not Done	Specify	Date of Assessment	*Was the assessment performed prior to vaccination?	Vital Signs Parameter	Units	Result	Out of Range	Clinical Significance
S202	206	JAIVAC-1	Dosage B	3	Day 180	.		28/03/2011	Yes	Diastolic Blood pressure	mm of Hg	68	Yes	NCS
S202	206	JAIVAC-1	Dosage B	3	Day 187	.		05/04/2011		Axillary temperature	°C	35.9	Yes	NCS
S202	206	JAIVAC-1	Dosage B	3	Day 187	.		05/04/2011		Diastolic Blood pressure	mm of Hg	70	Yes	NCS
S202	206	JAIVAC-1	Dosage B	3	Day 194	.		11/04/2011		Diastolic Blood pressure	mm of Hg	74	Yes	NCS
S202	206	JAIVAC-1	Dosage B	3	Day 208	.		25/04/2011		Axillary temperature	°C	35.9	Yes	NCS
S202	206	JAIVAC-1	Dosage B	3	Day 365	.		29/09/2011		Axillary temperature	°C	35.7	Yes	NCS
S202	206	JAIVAC-1	Dosage B	3	Day 365	.		29/09/2011		Diastolic Blood pressure	mm of Hg	86	Yes	NCS
S203	211	Hepatitis B	Hepatitis B	2	Day 35	.		04/11/2010		Diastolic Blood pressure	mm of Hg	70	Yes	NCS
S203	211	Hepatitis B	Hepatitis B	2	Day 56	.		25/11/2010		Diastolic Blood pressure	mm of Hg	74	Yes	NCS
S203	211	Hepatitis B	Hepatitis B	3	Day 180	.		29/03/2011	Yes	Diastolic Blood pressure	mm of Hg	68	Yes	NCS
S203	211	Hepatitis B	Hepatitis B	3	Day 187	.		05/04/2011		Diastolic Blood pressure	mm of Hg	72	Yes	NCS
S212	212	JAIVAC-1	Dosage B		Day -14 to Day -1	.		23/09/2010		Diastolic Blood pressure	mm of Hg	70	Yes	NCS

Subject No.	Randomisation Number	Study Group	Dosage	Dose	Day	Not Done	Specify	Date of Assessment	*Was the assessment performed prior to vaccination?	Vital Signs Parameter	Units	Result	Out of Range	Clinical Significance
S212	212	JAIVAC-1	Dosage B		Day -14 to Day -1	.		23/09/2010		Systolic Blood pressure	mm of Hg	100	Yes	NCS
S212	212	JAIVAC-1	Dosage B	1	Day 0	.		30/09/2010	Yes	Axillary temperature	°C	35.5	Yes	NCS
S212	212	JAIVAC-1	Dosage B	1	Day 0	.		30/09/2010	Yes	Diastolic Blood pressure	mm of Hg	86	Yes	NCS
S212	212	JAIVAC-1	Dosage B	1	Day 7	.		07/10/2010		Axillary temperature	°C	35.7	Yes	NCS
S212	212	JAIVAC-1	Dosage B	1	Day 7	.		07/10/2010		Diastolic Blood pressure	mm of Hg	70	Yes	NCS
S212	212	JAIVAC-1	Dosage B	1	Day 14	.		14/10/2010		Axillary temperature	°C	35.4	Yes	NCS
S212	212	JAIVAC-1	Dosage B	2	Day 28	.		28/10/2010	Yes	Axillary temperature	°C	35.5	Yes	NCS
S212	212	JAIVAC-1	Dosage B	2	Day 28	.		28/10/2010	Yes	Pulse rate	beats/ minute	90	Yes	NCS
S212	212	JAIVAC-1	Dosage B	2	Day 35	.		04/11/2010		Axillary temperature	°C	35.1	Yes	NCS
S212	212	JAIVAC-1	Dosage B	2	Day 42	.		11/11/2010		Axillary temperature	°C	35.3	Yes	NCS
S212	212	JAIVAC-1	Dosage B	2	Day 56	.		25/11/2010		Axillary temperature	°C	35.4	Yes	NCS
S212	212	JAIVAC-1	Dosage B	3	Day 187	.		05/04/2011		Axillary temperature	°C	35.3	Yes	NCS

Subject No.	Randomisation Number	Study Group	Dosage	Dose	Day	Not Done	Specify	Date of Assessment	*Was the assessment performed prior to vaccination?	Vital Signs Parameter	Units	Result	Out of Range	Clinical Significance
S212	212	JAIVAC-1	Dosage B	3	Day 194	.		12/04/2011		Axillary temperature	°C	35.4	Yes	NCS
S212	212	JAIVAC-1	Dosage B	3	Day 194	.		12/04/2011		Diastolic Blood pressure	mm of Hg	70	Yes	NCS
S212	212	JAIVAC-1	Dosage B	3	Day 208	.		19/04/2011		Axillary temperature	°C	35.8	Yes	NCS
S212	212	JAIVAC-1	Dosage B	3	Day 365	.		29/09/2011		Axillary temperature	°C	34.6	Yes	NCS
S212	212	JAIVAC-1	Dosage B	3	Day 365	.		29/09/2011		Respiratory rate	breaths/ minute	14	Yes	NCS
S215	213	Hepatitis B	Hepatitis B	1	Day 0	.		30/09/2010	Yes	Axillary temperature	°C	35.7	Yes	NCS
S215	213	Hepatitis B	Hepatitis B	1	Day 7	.		07/10/2010		Axillary temperature	°C	35.6	Yes	NCS
S215	213	Hepatitis B	Hepatitis B	1	Day 7	.		07/10/2010		Respiratory rate	breaths/ minute	14	Yes	NCS
S215	213	Hepatitis B	Hepatitis B	1	Day 14	.		14/10/2010		Axillary temperature	°C	35.5	Yes	NCS
S215	213	Hepatitis B	Hepatitis B	1	Day 14	.		14/10/2010		Diastolic Blood pressure	mm of Hg	70	Yes	NCS
S215	213	Hepatitis B	Hepatitis B	2	Day 28	.		28/10/2010	Yes	Axillary temperature	°C	35.4	Yes	NCS
S215	213	Hepatitis B	Hepatitis B	2	Day 35	.		04/11/2010		Axillary temperature	°C	35.6	Yes	NCS

Subject No.	Randomisation Number	Study Group	Dosage	Dose	Day	Not Done	Specify	Date of Assessment	*Was the assessment performed prior to vaccination?	Vital Signs Parameter	Units	Result	Out of Range	Clinical Significance
S215	213	Hepatitis B	Hepatitis B	2	Day 42	.		11/11/2010		Axillary temperature	°C	35.6	Yes	NCS
S215	213	Hepatitis B	Hepatitis B	3	Day 180	.		29/03/2011	Yes	Axillary temperature	°C	35.4	Yes	NCS
S215	213	Hepatitis B	Hepatitis B	3	Day 180	.		29/03/2011	Yes	Diastolic Blood pressure	mm of Hg	86	Yes	NCS
S215	213	Hepatitis B	Hepatitis B	3	Day 187	.		06/04/2011		Axillary temperature	°C	35.4	Yes	NCS
S215	213	Hepatitis B	Hepatitis B	3	Day 187	.		06/04/2011		Diastolic Blood pressure	mm of Hg	74	Yes	NCS
S215	213	Hepatitis B	Hepatitis B	3	Day 194	.		12/04/2011		Axillary temperature	°C	35.4	Yes	NCS
S215	213	Hepatitis B	Hepatitis B	3	Day 194	.		12/04/2011		Diastolic Blood pressure	mm of Hg	90	Yes	NCS
S215	213	Hepatitis B	Hepatitis B	3	Day 208	.		26/04/2011		Axillary temperature	°C	35.4	Yes	NCS
S215	213	Hepatitis B	Hepatitis B	3	Day 365	.		29/09/2011		Axillary temperature	°C	35.4	Yes	NCS
S215	213	Hepatitis B	Hepatitis B	3	Day 365	.		29/09/2011		Diastolic Blood pressure	mm of Hg	74	Yes	NCS
S215	213	Hepatitis B	Hepatitis B	3	Day 365	.		29/09/2011		Respiratory rate	breaths/ minute	14	Yes	NCS
S233	210	JAIVAC-1	Dosage B		Day -14 to Day -1	.		24/09/2010		Axillary temperature	°C	35.4	Yes	NCS

Subject No.	Randomisation Number	Study Group	Dosage	Dose	Day	Not Done	Specify	Date of Assessment	*Was the assessment performed prior to vaccination?	Vital Signs Parameter	Units	Result	Out of Range	Clinical Significance
S233	210	JAIVAC-1	Dosage B	1	Day 7	.		07/10/2010		Axillary temperature	°C	35.1	Yes	NCS
S233	210	JAIVAC-1	Dosage B	1	Day 7	.		07/10/2010		Diastolic Blood pressure	mm of Hg	70	Yes	NCS
S233	210	JAIVAC-1	Dosage B	1	Day 7	.		07/10/2010		Respiratory rate	breaths/ minute	14	Yes	NCS
S233	210	JAIVAC-1	Dosage B	1	Day 14	.		14/10/2010		Axillary temperature	°C	35.6	Yes	NCS
S233	210	JAIVAC-1	Dosage B	1	Day 14	.		14/10/2010		Diastolic Blood pressure	mm of Hg	90	Yes	NCS
S233	210	JAIVAC-1	Dosage B	2	Day 35	.		04/11/2010		Systolic Blood pressure	mm of Hg	140	Yes	NCS
S233	210	JAIVAC-1	Dosage B	2	Day 56	.		25/11/2010		Diastolic Blood pressure	mm of Hg	72	Yes	NCS
S233	210	JAIVAC-1	Dosage B	3	Day 180	.		29/03/2011	Yes	Diastolic Blood pressure	mm of Hg	72	Yes	NCS
S233	210	JAIVAC-1	Dosage B	3	Day 194	.		13/04/2011		Diastolic Blood pressure	mm of Hg	74	Yes	NCS
S233	210	JAIVAC-1	Dosage B	3	Day 208	.		27/04/2011		Diastolic Blood pressure	mm of Hg	74	Yes	NCS
S241	303	Hepatitis B	Hepatitis B		Day -14 to Day -1	.		03/11/2010		Axillary temperature	°C	35.7	Yes	NCS
S241	303	Hepatitis B	Hepatitis B		Day -14 to Day -1	.		03/11/2010		Pulse rate	beats/ minute	60	Yes	NCS

Subject No.	Randomisation Number	Study Group	Dosage	Dose	Day	Not Done	Specify	Date of Assessment	*Was the assessment performed prior to vaccination?	Vital Signs Parameter	Units	Result	Out of Range	Clinical Significance
S241	303	Hepatitis B	Hepatitis B	1	Day 7	.		17/11/2010		Axillary temperature	°C	35.3	Yes	NCS
S241	303	Hepatitis B	Hepatitis B	2	Day 28	.		08/12/2010	Yes	Axillary temperature	°C	35.4	Yes	NCS
S241	303	Hepatitis B	Hepatitis B	2	Day 42	.		22/12/2010		Diastolic Blood pressure	mm of Hg	74	Yes	NCS
S241	303	Hepatitis B	Hepatitis B	3	Day 180	.		09/05/2011	Yes	Diastolic Blood pressure	mm of Hg	74	Yes	NCS
S241	303	Hepatitis B	Hepatitis B	3	Day 187	.		16/05/2011		Axillary temperature	°C	35.9	Yes	NCS
S241	303	Hepatitis B	Hepatitis B	3	Day 187	.		16/05/2011		Diastolic Blood pressure	mm of Hg	74	Yes	NCS
S241	303	Hepatitis B	Hepatitis B	3	Day 194	.		23/05/2011		Diastolic Blood pressure	mm of Hg	70	Yes	NCS
S241	303	Hepatitis B	Hepatitis B	3	Day 208	.		06/06/2011		Axillary temperature	°C	35.9	Yes	NCS
S241	303	Hepatitis B	Hepatitis B	3	Day 365	.		09/11/2011		Axillary temperature	°C	35.9	Yes	NCS
S250	311	JAIVAC-1	Dosage C		Day -14 to Day -1	.		03/11/2010		Systolic Blood pressure	mm of Hg	106	Yes	NCS
S250	311	JAIVAC-1	Dosage C	2	Day 28	.		10/12/2010	Yes	Axillary temperature	°C	35.4	Yes	NCS
S250	311	JAIVAC-1	Dosage C	2	Day 35	.		17/12/2010		Axillary temperature	°C	35.9	Yes	NCS

Subject No.	Randomisation Number	Study Group	Dosage	Dose	Day	Not Done	Specify	Date of Assessment	*Was the assessment performed prior to vaccination?	Vital Signs Parameter	Units	Result	Out of Range	Clinical Significance
S250	311	JAIVAC-1	Dosage C	2	Day 42	.		24/12/2010		Axillary temperature	°C	35.5	Yes	NCS
S250	311	JAIVAC-1	Dosage C	2	Day 42	.		24/12/2010		Diastolic Blood pressure	mm of Hg	74	Yes	NCS
S250	311	JAIVAC-1	Dosage C	2	Day 56	.		07/01/2011		Axillary temperature	°C	35.8	Yes	NCS
S250	311	JAIVAC-1	Dosage C	2	Day 56	.		07/01/2011		Diastolic Blood pressure	mm of Hg	70	Yes	NCS
S250	311	JAIVAC-1	Dosage C	2	Day 56	.		07/01/2011		Respiratory rate	breaths/ minute	14	Yes	NCS
S250	311	JAIVAC-1	Dosage C	2	Day 56	.		07/01/2011		Systolic Blood pressure	mm of Hg	104	Yes	NCS
S250	311	JAIVAC-1	Dosage C	3	Day 180	.		11/05/2011	Yes	Axillary temperature	°C	35.6	Yes	NCS
S250	311	JAIVAC-1	Dosage C	3	Day 180	.		11/05/2011	Yes	Diastolic Blood pressure	mm of Hg	70	Yes	NCS
S250	311	JAIVAC-1	Dosage C	3	Day 187	.		18/05/2011		Axillary temperature	°C	35	Yes	NCS
S250	311	JAIVAC-1	Dosage C	3	Day 194	.		24/05/2011		Axillary temperature	°C	35.4	Yes	NCS
S250	311	JAIVAC-1	Dosage C	3	Day 194	.		24/05/2011		Diastolic Blood pressure	mm of Hg	70	Yes	NCS
S250	311	JAIVAC-1	Dosage C	3	Day 208	.		08/06/2011		Axillary temperature	°C	35.4	Yes	NCS

Subject No.	Randomisation Number	Study Group	Dosage	Dose	Day	Not Done	Specify	Date of Assessment	*Was the assessment performed prior to vaccination?	Vital Signs Parameter	Units	Result	Out of Range	Clinical Significance
S250	311	JAIVAC-1	Dosage C	3	Day 208	.		08/06/2011		Diastolic Blood pressure	mm of Hg	70	Yes	NCS
S250	311	JAIVAC-1	Dosage C	3	Day 208	.		08/06/2011		Respiratory rate	breaths/ minute	14	Yes	NCS
S250	311	JAIVAC-1	Dosage C	3	Day 208	.		08/06/2011		Systolic Blood pressure	mm of Hg	104	Yes	NCS
S250	311	JAIVAC-1	Dosage C	3	Day 365	.		10/11/2011		Axillary temperature	°C	35.9	Yes	NCS
S250	311	JAIVAC-1	Dosage C	3	Day 365	.		10/11/2011		Systolic Blood pressure	mm of Hg	106	Yes	NCS
S263	313	Hepatitis B	Hepatitis B	1	Day 0	.		12/11/2010	Yes	Diastolic Blood pressure	mm of Hg	70	Yes	NCS
S263	313	Hepatitis B	Hepatitis B	2	Day 42	.		24/12/2010		Respiratory rate	breaths/ minute	14	Yes	NCS
S263	313	Hepatitis B	Hepatitis B	2	Day 56	.		07/01/2011		Diastolic Blood pressure	mm of Hg	70	Yes	NCS
S263	313	Hepatitis B	Hepatitis B	3	Day 180	.		11/05/2011	Yes	Axillary temperature	°C	35.8	Yes	NCS
S263	313	Hepatitis B	Hepatitis B	3	Day 180	.		11/05/2011	Yes	Diastolic Blood pressure	mm of Hg	74	Yes	NCS
S263	313	Hepatitis B	Hepatitis B	3	Day 187	.		18/05/2011		Diastolic Blood pressure	mm of Hg	70	Yes	NCS
S263	313	Hepatitis B	Hepatitis B	3	Day 194	.		25/05/2011		Diastolic Blood pressure	mm of Hg	70	Yes	NCS

Subject No.	Randomisation Number	Study Group	Dosage	Dose	Day	Not Done	Specify	Date of Assessment	*Was the assessment performed prior to vaccination?	Vital Signs Parameter	Units	Result	Out of Range	Clinical Significance
S263	313	Hepatitis B	Hepatitis B	3	Day 208	.		08/06/2011		Diastolic Blood pressure	mm of Hg	70	Yes	NCS
S263	313	Hepatitis B	Hepatitis B	3	Day 365	.		10/11/2011		Diastolic Blood pressure	mm of Hg	74	Yes	NCS
S264	306	JAIVAC-1	Dosage C	1	Day 0	.		11/11/2010	Yes	Axillary temperature	°C	35.5	Yes	NCS
S264	306	JAIVAC-1	Dosage C	1	Day 0	.		11/11/2010	Yes	Systolic Blood pressure	mm of Hg	136	Yes	NCS
S264	306	JAIVAC-1	Dosage C	1	Day 7	.		18/11/2010		Axillary temperature	°C	35.8	Yes	NCS
S264	306	JAIVAC-1	Dosage C	1	Day 14	.		25/11/2010		Axillary temperature	°C	35.6	Yes	NCS
S264	306	JAIVAC-1	Dosage C	2	Day 28	.		09/12/2010	Yes	Axillary temperature	°C	35.7	Yes	NCS
S264	306	JAIVAC-1	Dosage C	2	Day 28	.		09/12/2010	Yes	Diastolic Blood pressure	mm of Hg	86	Yes	NCS
S264	306	JAIVAC-1	Dosage C	2	Day 35	.		16/12/2010		Axillary temperature	°C	35.6	Yes	NCS
S264	306	JAIVAC-1	Dosage C	2	Day 42	.		23/12/2010		Axillary temperature	°C	35.3	Yes	NCS
S264	306	JAIVAC-1	Dosage C	2	Day 56	.		06/01/2011		Axillary temperature	°C	35.7	Yes	NCS
S264	306	JAIVAC-1	Dosage C	3	Day 180	.		10/05/2011	Yes	Axillary temperature	°C	35.5	Yes	NCS

Subject No.	Randomisation Number	Study Group	Dosage	Dose	Day	Not Done	Specify	Date of Assessment	*Was the assessment performed prior to vaccination?	Vital Signs Parameter	Units	Result	Out of Range	Clinical Significance
S264	306	JAIVAC-1	Dosage C	3	Day 187	.		17/05/2011		Axillary temperature	°C	35.7	Yes	NCS
S264	306	JAIVAC-1	Dosage C	3	Day 194	.		24/05/2011		Axillary temperature	°C	35.9	Yes	NCS
S264	306	JAIVAC-1	Dosage C	3	Day 194	.		24/05/2011		Diastolic Blood pressure	mm of Hg	86	Yes	NCS
S264	306	JAIVAC-1	Dosage C	3	Day 208	.		07/06/2011		Axillary temperature	°C	35.3	Yes	NCS
S264	306	JAIVAC-1	Dosage C	3	Day 365	.		09/11/2011		Axillary temperature	°C	35.2	Yes	NCS
S266	308	JAIVAC-1	Dosage C		Day -14 to Day -1	.		03/11/2010		Pulse rate	beats/ minute	60	Yes	NCS
S266	308	JAIVAC-1	Dosage C	1	Day 7	.		18/11/2010		Diastolic Blood pressure	mm of Hg	70	Yes	NCS
S266	308	JAIVAC-1	Dosage C	1	Day 14	.		25/11/2010		Diastolic Blood pressure	mm of Hg	70	Yes	NCS
S266	308	JAIVAC-1	Dosage C	2	Day 28	.		09/12/2010	Yes	Axillary temperature	°C	35.5	Yes	NCS
S266	308	JAIVAC-1	Dosage C	2	Day 35	.		16/12/2010		Axillary temperature	°C	35.9	Yes	NCS
S266	308	JAIVAC-1	Dosage C	2	Day 42	.		23/12/2010		Axillary temperature	°C	35.5	Yes	NCS
S266	308	JAIVAC-1	Dosage C	2	Day 56	.		06/01/2011		Axillary temperature	°C	35.1	Yes	NCS

Subject No.	Randomisation Number	Study Group	Dosage	Dose	Day	Not Done	Specify	Date of Assessment	*Was the assessment performed prior to vaccination?	Vital Signs Parameter	Units	Result	Out of Range	Clinical Significance
S266	308	JAIVAC-1	Dosage C	3	Day 187	.		17/05/2011		Axillary temperature	°C	35.9	Yes	NCS
S266	308	JAIVAC-1	Dosage C	3	Day 187	.		17/05/2011		Diastolic Blood pressure	mm of Hg	74	Yes	NCS
S266	308	JAIVAC-1	Dosage C	3	Day 194	.		24/05/2011		Axillary temperature	°C	35.1	Yes	NCS
S266	308	JAIVAC-1	Dosage C	3	Day 365	.		09/11/2011		Axillary temperature	°C	35.4	Yes	NCS
S267	309	Hepatitis B	Hepatitis B		Day -14 to Day -1	.		02/11/2010		Diastolic Blood pressure	mm of Hg	70	Yes	NCS
S267	309	Hepatitis B	Hepatitis B		Day -14 to Day -1	.		02/11/2010		Systolic Blood pressure	mm of Hg	104	Yes	NCS
S267	309	Hepatitis B	Hepatitis B	1	Day 0	.		11/11/2010	Yes	Pulse rate	beats/ minute	60	Yes	NCS
S267	309	Hepatitis B	Hepatitis B	1	Day 7	.		19/11/2010		Axillary temperature	°C	35.8	Yes	NCS
S267	309	Hepatitis B	Hepatitis B	1	Day 7	.		19/11/2010		Diastolic Blood pressure	mm of Hg	70	Yes	NCS
S267	309	Hepatitis B	Hepatitis B	1	Day 7	.		19/11/2010		Respiratory rate	breaths/ minute	14	Yes	NCS
S267	309	Hepatitis B	Hepatitis B	1	Day 14	.		25/11/2010		Axillary temperature	°C	35.8	Yes	NCS
S267	309	Hepatitis B	Hepatitis B	2	Day 28	.		09/12/2010	Yes	Axillary temperature	°C	35.6	Yes	NCS

Subject No.	Randomisation Number	Study Group	Dosage	Dose	Day	Not Done	Specify	Date of Assessment	*Was the assessment performed prior to vaccination?	Vital Signs Parameter	Units	Result	Out of Range	Clinical Significance
S267	309	Hepatitis B	Hepatitis B	2	Day 35	.		16/12/2010		Axillary temperature	°C	35.2	Yes	NCS
S267	309	Hepatitis B	Hepatitis B	2	Day 42	.		23/12/2010		Axillary temperature	°C	35.6	Yes	NCS
S267	309	Hepatitis B	Hepatitis B	2	Day 42	.		23/12/2010		Diastolic Blood pressure	mm of Hg	70	Yes	NCS
S267	309	Hepatitis B	Hepatitis B	2	Day 56	.		06/01/2011		Axillary temperature	°C	35.5	Yes	NCS
S267	309	Hepatitis B	Hepatitis B	2	Day 56	.		06/01/2011		Diastolic Blood pressure	mm of Hg	74	Yes	NCS
S267	309	Hepatitis B	Hepatitis B	3	Day 180	.		10/05/2011	Yes	Axillary temperature	°C	35.4	Yes	NCS
S267	309	Hepatitis B	Hepatitis B	3	Day 187	.		17/05/2011		Diastolic Blood pressure	mm of Hg	70	Yes	NCS
S267	309	Hepatitis B	Hepatitis B	3	Day 194	.		24/05/2011		Axillary temperature	°C	35	Yes	NCS
S267	309	Hepatitis B	Hepatitis B	3	Day 194	.		24/05/2011		Diastolic Blood pressure	mm of Hg	66	Yes	NCS
S267	309	Hepatitis B	Hepatitis B	3	Day 194	.		24/05/2011		Systolic Blood pressure	mm of Hg	104	Yes	NCS
S267	309	Hepatitis B	Hepatitis B	3	Day 208	.		07/06/2011		Axillary temperature	°C	35.6	Yes	NCS
S267	309	Hepatitis B	Hepatitis B	3	Day 208	.		07/06/2011		Diastolic Blood pressure	mm of Hg	70	Yes	NCS

Subject No.	Randomisation Number	Study Group	Dosage	Dose	Day	Not Done	Specify	Date of Assessment	*Was the assessment performed prior to vaccination?	Vital Signs Parameter	Units	Result	Out of Range	Clinical Significance
S267	309	Hepatitis B	Hepatitis B	3	Day 365	.		09/11/2011		Axillary temperature	°C	35	Yes	NCS
S267	309	Hepatitis B	Hepatitis B	3	Day 365	.		09/11/2011		Diastolic Blood pressure	mm of Hg	70	Yes	NCS
S275	302	Hepatitis B	Hepatitis B		Day -14 to Day -1	.		02/11/2010		Axillary temperature	°C	35.8	Yes	NCS
S275	302	Hepatitis B	Hepatitis B	1	Day 14	.		24/11/2010		Axillary temperature	°C	35.3	Yes	NCS
S275	302	Hepatitis B	Hepatitis B	1	Day 14	.		24/11/2010		Diastolic Blood pressure	mm of Hg	70	Yes	NCS
S275	302	Hepatitis B	Hepatitis B	1	Day 14	.		24/11/2010		Systolic Blood pressure	mm of Hg	100	Yes	NCS
S275	302	Hepatitis B	Hepatitis B	2	Day 28	.		08/12/2010	Yes	Axillary temperature	°C	35	Yes	NCS
S275	302	Hepatitis B	Hepatitis B	2	Day 35	.		15/12/2010		Axillary temperature	°C	35.6	Yes	NCS
S275	302	Hepatitis B	Hepatitis B	2	Day 42	.		22/12/2010		Axillary temperature	°C	35.4	Yes	NCS
S275	302	Hepatitis B	Hepatitis B	2	Day 56	.		05/01/2011		Axillary temperature	°C	35.5	Yes	NCS
S275	302	Hepatitis B	Hepatitis B	2	Day 56	.		05/01/2011		Diastolic Blood pressure	mm of Hg	74	Yes	NCS
S275	302	Hepatitis B	Hepatitis B	3	Day 180	.		09/05/2011	Yes	Axillary temperature	°C	35.9	Yes	NCS

Subject No.	Randomisation Number	Study Group	Dosage	Dose	Day	Not Done	Specify	Date of Assessment	*Was the assessment performed prior to vaccination?	Vital Signs Parameter	Units	Result	Out of Range	Clinical Significance
S275	302	Hepatitis B	Hepatitis B	3	Day 180	.		09/05/2011	Yes	Diastolic Blood pressure	mm of Hg	70	Yes	NCS
S275	302	Hepatitis B	Hepatitis B	3	Day 187	.		16/05/2011		Axillary temperature	°C	35.4	Yes	NCS
S275	302	Hepatitis B	Hepatitis B	3	Day 187	.		16/05/2011		Diastolic Blood pressure	mm of Hg	70	Yes	NCS
S275	302	Hepatitis B	Hepatitis B	3	Day 194	.		23/05/2011		Axillary temperature	°C	35.8	Yes	NCS
S275	302	Hepatitis B	Hepatitis B	3	Day 194	.		23/05/2011		Diastolic Blood pressure	mm of Hg	74	Yes	NCS
S275	302	Hepatitis B	Hepatitis B	3	Day 208	.		06/06/2011		Axillary temperature	°C	35.4	Yes	NCS
S275	302	Hepatitis B	Hepatitis B	3	Day 208	.		06/06/2011		Diastolic Blood pressure	mm of Hg	70	Yes	NCS
S275	302	Hepatitis B	Hepatitis B	3	Day 365	.		09/11/2011		Axillary temperature	°C	35.8	Yes	NCS
S275	302	Hepatitis B	Hepatitis B	3	Day 365	.		09/11/2011		Diastolic Blood pressure	mm of Hg	74	Yes	NCS
S278	315	JAIVAC-1	Dosage C		Day -14 to Day -1	.		08/11/2010		Diastolic Blood pressure	mm of Hg	70	Yes	NCS
S278	315	JAIVAC-1	Dosage C		Day -14 to Day -1	.		08/11/2010		Pulse rate	beats/ minute	60	Yes	NCS
S278	315	JAIVAC-1	Dosage C		Day -14 to Day -1	.		08/11/2010		Systolic Blood pressure	mm of Hg	106	Yes	NCS

Subject No.	Randomisation Number	Study Group	Dosage	Dose	Day	Not Done	Specify	Date of Assessment	*Was the assessment performed prior to vaccination?	Vital Signs Parameter	Units	Result	Out of Range	Clinical Significance
S278	315	JAIVAC-1	Dosage C	1	Day 0	.		12/11/2010	Yes	Axillary temperature	°C	35.5	Yes	NCS
S278	315	JAIVAC-1	Dosage C	1	Day 7	.		19/11/2010		Axillary temperature	°C	35.7	Yes	NCS
S278	315	JAIVAC-1	Dosage C	1	Day 7	.		19/11/2010		Diastolic Blood pressure	mm of Hg	70	Yes	NCS
S278	315	JAIVAC-1	Dosage C	1	Day 14	.		26/11/2010		Axillary temperature	°C	35.9	Yes	NCS
S278	315	JAIVAC-1	Dosage C	1	Day 14	.		26/11/2010		Diastolic Blood pressure	mm of Hg	70	Yes	NCS
S278	315	JAIVAC-1	Dosage C	2	Day 35	.		17/12/2010		Axillary temperature	°C	35	Yes	NCS
S278	315	JAIVAC-1	Dosage C	2	Day 35	.		17/12/2010		Diastolic Blood pressure	mm of Hg	70	Yes	NCS
S278	315	JAIVAC-1	Dosage C	2	Day 42	.		24/12/2010		Axillary temperature	°C	35.8	Yes	NCS
S278	315	JAIVAC-1	Dosage C	2	Day 56	.		07/01/2011		Diastolic Blood pressure	mm of Hg	74	Yes	NCS
S278	315	JAIVAC-1	Dosage C	3	Day 180	.		11/05/2011	Yes	Axillary temperature	°C	35.2	Yes	NCS
S278	315	JAIVAC-1	Dosage C	3	Day 180	.		11/05/2011	Yes	Diastolic Blood pressure	mm of Hg	68	Yes	NCS
S278	315	JAIVAC-1	Dosage C	3	Day 187	.		18/05/2011		Diastolic Blood pressure	mm of Hg	74	Yes	NCS

Subject No.	Randomisation Number	Study Group	Dosage	Dose	Day	Not Done	Specify	Date of Assessment	*Was the assessment performed prior to vaccination?	Vital Signs Parameter	Units	Result	Out of Range	Clinical Significance
S278	315	JAIVAC-1	Dosage C	3	Day 194	.		24/05/2011		Axillary temperature	°C	35.8	Yes	NCS
S278	315	JAIVAC-1	Dosage C	3	Day 208	.		09/06/2011		Axillary temperature	°C	35.4	Yes	NCS
S278	315	JAIVAC-1	Dosage C	3	Day 365	.		10/11/2011		Axillary temperature	°C	35.8	Yes	NCS
S279	301	JAIVAC-1	Dosage C	1	Day 7	.		17/11/2010		Systolic Blood pressure	mm of Hg	136	Yes	NCS
S279	301	JAIVAC-1	Dosage C	2	Day 42	.		22/12/2010		Axillary temperature	°C	35.8	Yes	NCS
S279	301	JAIVAC-1	Dosage C	3	Day 187	.		16/05/2011		Diastolic Blood pressure	mm of Hg	74	Yes	NCS
S279	301	JAIVAC-1	Dosage C	3	Day 365	.		09/11/2011		Axillary temperature	°C	35.6	Yes	NCS
S281	304	JAIVAC-1	Dosage C		Day -14 to Day -1	.		03/11/2010		Axillary temperature	°C	35.8	Yes	NCS
S281	304	JAIVAC-1	Dosage C		Day -14 to Day -1	.		03/11/2010		Diastolic Blood pressure	mm of Hg	70	Yes	NCS
S281	304	JAIVAC-1	Dosage C		Day -14 to Day -1	.		03/11/2010		Systolic Blood pressure	mm of Hg	104	Yes	NCS
S281	304	JAIVAC-1	Dosage C	1	Day 7	.		18/11/2010		Diastolic Blood pressure	mm of Hg	74	Yes	NCS
S281	304	JAIVAC-1	Dosage C	1	Day 14	.		25/11/2010		Diastolic Blood pressure	mm of Hg	72	Yes	NCS

Subject No.	Randomisation Number	Study Group	Dosage	Dose	Day	Not Done	Specify	Date of Assessment	*Was the assessment performed prior to vaccination?	Vital Signs Parameter	Units	Result	Out of Range	Clinical Significance
S281	304	JAIVAC-1	Dosage C	2	Day 28	.		09/12/2010	Yes	Axillary temperature	°C	35.2	Yes	NCS
S281	304	JAIVAC-1	Dosage C	2	Day 35	.		16/12/2010		Axillary temperature	°C	35.6	Yes	NCS
S281	304	JAIVAC-1	Dosage C	2	Day 42	.		27/12/2010		Axillary temperature	°C	35.8	Yes	NCS
S281	304	JAIVAC-1	Dosage C		Early Termination	.		11/05/2011		Axillary temperature	°C	35.8	Yes	NCS
S284	305	JAIVAC-1	Dosage C		Day -14 to Day -1	.		03/11/2010		Diastolic Blood pressure	mm of Hg	70	Yes	NCS
S284	305	JAIVAC-1	Dosage C		Day -14 to Day -1	.		03/11/2010		Systolic Blood pressure	mm of Hg	106	Yes	NCS
S284	305	JAIVAC-1	Dosage C	1	Day 0	.		11/11/2010	Yes	Axillary temperature	°C	35.5	Yes	NCS
S284	305	JAIVAC-1	Dosage C	1	Day 14	.		25/11/2010		Diastolic Blood pressure	mm of Hg	74	Yes	NCS
S284	305	JAIVAC-1	Dosage C	2	Day 28	.		09/12/2010	Yes	Axillary temperature	°C	35.2	Yes	NCS
S284	305	JAIVAC-1	Dosage C	2	Day 42	.		23/12/2010		Diastolic Blood pressure	mm of Hg	72	Yes	NCS
S284	305	JAIVAC-1	Dosage C	3	Day 187	.		17/05/2011		Diastolic Blood pressure	mm of Hg	70	Yes	NCS
S284	305	JAIVAC-1	Dosage C	3	Day 194	.		24/05/2011		Axillary temperature	°C	35.4	Yes	NCS

Subject No.	Randomisation Number	Study Group	Dosage	Dose	Day	Not Done	Specify	Date of Assessment	*Was the assessment performed prior to vaccination?	Vital Signs Parameter	Units	Result	Out of Range	Clinical Significance
S284	305	JAIVAC-1	Dosage C	3	Day 208	.		07/06/2011		Axillary temperature	°C	35.2	Yes	NCS
S284	305	JAIVAC-1	Dosage C	3	Day 208	.		07/06/2011		Diastolic Blood pressure	mm of Hg	70	Yes	NCS
S284	305	JAIVAC-1	Dosage C	3	Day 365	.		09/11/2011		Axillary temperature	°C	35.2	Yes	NCS
S285	307	Hepatitis B	Hepatitis B		Day -14 to Day -1	.		02/11/2010		Diastolic Blood pressure	mm of Hg	70	Yes	NCS
S285	307	Hepatitis B	Hepatitis B		Day -14 to Day -1	.		02/11/2010		Pulse rate	beats/ minute	60	Yes	NCS
S285	307	Hepatitis B	Hepatitis B		Day -14 to Day -1	.		02/11/2010		Systolic Blood pressure	mm of Hg	100	Yes	NCS
S285	307	Hepatitis B	Hepatitis B	1	Day 0	.		11/11/2010	Yes	Systolic Blood pressure	mm of Hg	106	Yes	NCS
S285	307	Hepatitis B	Hepatitis B	1	Day 14	.		25/11/2010		Axillary temperature	°C	35.9	Yes	NCS
S285	307	Hepatitis B	Hepatitis B	1	Day 14	.		25/11/2010		Diastolic Blood pressure	mm of Hg	72	Yes	NCS
S285	307	Hepatitis B	Hepatitis B	2	Day 28	.		09/12/2010	Yes	Axillary temperature	°C	35.8	Yes	NCS
S285	307	Hepatitis B	Hepatitis B	2	Day 42	.		23/12/2010		Diastolic Blood pressure	mm of Hg	74	Yes	NCS
S285	307	Hepatitis B	Hepatitis B	2	Day 56	.		06/01/2011		Axillary temperature	°C	35.9	Yes	NCS

Subject No.	Randomisation Number	Study Group	Dosage	Dose	Day	Not Done	Specify	Date of Assessment	*Was the assessment performed prior to vaccination?	Vital Signs Parameter	Units	Result	Out of Range	Clinical Significance
S285	307	Hepatitis B	Hepatitis B	2	Day 56	.		06/01/2011		Diastolic Blood pressure	mm of Hg	64	Yes	NCS
S285	307	Hepatitis B	Hepatitis B	3	Day 180	.		10/05/2011	Yes	Axillary temperature	°C	35.8	Yes	NCS
S285	307	Hepatitis B	Hepatitis B	3	Day 187	.		17/05/2011		Diastolic Blood pressure	mm of Hg	68	Yes	NCS
S285	307	Hepatitis B	Hepatitis B	3	Day 194	.		24/05/2011		Diastolic Blood pressure	mm of Hg	70	Yes	NCS
S285	307	Hepatitis B	Hepatitis B	3	Day 208	.		07/06/2011		Diastolic Blood pressure	mm of Hg	74	Yes	NCS
S285	307	Hepatitis B	Hepatitis B	3	Day 365	.		09/11/2011		Axillary temperature	°C	35	Yes	NCS
S285	307	Hepatitis B	Hepatitis B	3	Day 365	.		09/11/2011		Diastolic Blood pressure	mm of Hg	70	Yes	NCS
S291	312	JAIVAC-1	Dosage C		Day -14 to Day -1	.		02/11/2010		Axillary temperature	°C	35.8	Yes	NCS
S291	312	JAIVAC-1	Dosage C	1	Day 0	.		12/11/2010	Yes	Axillary temperature	°C	35.8	Yes	NCS
S291	312	JAIVAC-1	Dosage C	1	Day 14	.		26/11/2010		Axillary temperature	°C	35.6	Yes	NCS
S291	312	JAIVAC-1	Dosage C	2	Day 35	.		17/12/2010		Axillary temperature	°C	35.2	Yes	NCS
S291	312	JAIVAC-1	Dosage C	2	Day 42	.		24/12/2010		Axillary temperature	°C	35.6	Yes	NCS

Subject No.	Randomisation Number	Study Group	Dosage	Dose	Day	Not Done	Specify	Date of Assessment	*Was the assessment performed prior to vaccination?	Vital Signs Parameter	Units	Result	Out of Range	Clinical Significance
S291	312	JAIVAC-1	Dosage C	2	Day 56	.		07/01/2011		Axillary temperature	°C	35	Yes	NCS
S291	312	JAIVAC-1	Dosage C	3	Day 180	.		11/05/2011	Yes	Axillary temperature	°C	35.8	Yes	NCS
S291	312	JAIVAC-1	Dosage C	3	Day 187	.		18/05/2011		Axillary temperature	°C	35.4	Yes	NCS
S291	312	JAIVAC-1	Dosage C	3	Day 187	.		18/05/2011		Diastolic Blood pressure	mm of Hg	64	Yes	NCS
S291	312	JAIVAC-1	Dosage C	3	Day 194	.		25/05/2011		Axillary temperature	°C	35.7	Yes	NCS
S291	312	JAIVAC-1	Dosage C	3	Day 208	.		08/06/2011		Axillary temperature	°C	35.6	Yes	NCS
S298	314	JAIVAC-1	Dosage C		Day -14 to Day -1	.		09/11/2010		Diastolic Blood pressure	mm of Hg	70	Yes	NCS
S298	314	JAIVAC-1	Dosage C	1	Day 0	.		12/11/2010	Yes	Axillary temperature	°C	35.7	Yes	NCS
S298	314	JAIVAC-1	Dosage C	1	Day 7	.		19/11/2010		Axillary temperature	°C	35.7	Yes	NCS
S298	314	JAIVAC-1	Dosage C	1	Day 14	.		26/11/2010		Axillary temperature	°C	35.8	Yes	NCS
S298	314	JAIVAC-1	Dosage C	1	Day 14	.		26/11/2010		Systolic Blood pressure	mm of Hg	140	Yes	NCS
S298	314	JAIVAC-1	Dosage C	2	Day 28	.		10/12/2010	Yes	Axillary temperature	°C	35.9	Yes	NCS

Subject No.	Randomisation Number	Study Group	Dosage	Dose	Day	Not Done	Specify	Date of Assessment	*Was the assessment performed prior to vaccination?	Vital Signs Parameter	Units	Result	Out of Range	Clinical Significance
S298	314	JAIVAC-1	Dosage C	2	Day 28	.		10/12/2010	Yes	Systolic Blood pressure	mm of Hg	136	Yes	NCS
S298	314	JAIVAC-1	Dosage C	2	Day 42	.		24/12/2010		Axillary temperature	°C	35.8	Yes	NCS
S298	314	JAIVAC-1	Dosage C	2	Day 42	.		24/12/2010		Respiratory rate	breaths/ minute	14	Yes	NCS
S298	314	JAIVAC-1	Dosage C	3	Day 180	.		11/05/2011	Yes	Diastolic Blood pressure	mm of Hg	74	Yes	NCS
S298	314	JAIVAC-1	Dosage C	3	Day 208	.		08/06/2011		Axillary temperature	°C	35.5	Yes	NCS
S298	314	JAIVAC-1	Dosage C	3	Day 365	.		10/11/2011		Axillary temperature	°C	35.9	Yes	NCS
S298	314	JAIVAC-1	Dosage C	3	Day 365	.		10/11/2011		Diastolic Blood pressure	mm of Hg	88	Yes	NCS
S299	310	JAIVAC-1	Dosage C	1	Day 0	.		12/11/2010	Yes	Axillary temperature	°C	35.5	Yes	NCS
S299	310	JAIVAC-1	Dosage C	1	Day 0	.		12/11/2010	Yes	Systolic Blood pressure	mm of Hg	106	Yes	NCS
S299	310	JAIVAC-1	Dosage C	1	Day 7	.		19/11/2010		Axillary temperature	°C	35.2	Yes	NCS
S299	310	JAIVAC-1	Dosage C	2	Day 35	.		17/12/2010		Axillary temperature	°C	35.5	Yes	NCS
S299	310	JAIVAC-1	Dosage C	2	Day 35	.		17/12/2010		Diastolic Blood pressure	mm of Hg	70	Yes	NCS

Subject No.	Randomisation Number	Study Group	Dosage	Dose	Day	Not Done	Specify	Date of Assessment	*Was the assessment performed prior to vaccination?	Vital Signs Parameter	Units	Result	Out of Range	Clinical Significance
S299	310	JAIVAC-1	Dosage C	2	Day 42	.		24/12/2010		Axillary temperature	°C	35.3	Yes	NCS
S299	310	JAIVAC-1	Dosage C	2	Day 56	.		07/01/2011		Axillary temperature	°C	35.6	Yes	NCS
S299	310	JAIVAC-1	Dosage C	3	Day 180	.		11/05/2011	Yes	Axillary temperature	°C	35.8	Yes	NCS
S299	310	JAIVAC-1	Dosage C	3	Day 180	.		11/05/2011	Yes	Diastolic Blood pressure	mm of Hg	70	Yes	NCS
S299	310	JAIVAC-1	Dosage C	3	Day 187	.		18/05/2011		Axillary temperature	°C	35.5	Yes	NCS
S299	310	JAIVAC-1	Dosage C	3	Day 194	.		25/05/2011		Axillary temperature	°C	35.2	Yes	NCS
S299	310	JAIVAC-1	Dosage C	3	Day 194	.		25/05/2011		Diastolic Blood pressure	mm of Hg	70	Yes	NCS
S299	310	JAIVAC-1	Dosage C	3	Day 208	.		08/06/2011		Axillary temperature	°C	35.3	Yes	NCS
S299	310	JAIVAC-1	Dosage C	3	Day 208	.		08/06/2011		Diastolic Blood pressure	mm of Hg	74	Yes	NCS

Note:
1) * Was the assessment performed prior to vaccination? Is applicable for Day 0, Day 28 and Day 180

14.3.6 Abnormal Physical Examination Results

14.3.6.1: Listing of Abnormal Physical Examination Results by subject

Subject No.	Randomisation Number	Study Group	Dosage	Dose	Day	Not Done	Specify	Date of Assessment	*Was the assessment performed prior to vaccination?	Physical Examination Parameter	Result	Abnormality	Clinical Significance
S120	104	JAIVAC-1	Dosage A		Day -14 to Day -1	.		18/08/2010		Dermatological	Abnormal	Pityriasis Versicolor	NCS
S120	104	JAIVAC-1	Dosage A	1	Day 0	.		26/08/2010	Yes	Dermatological	Abnormal	Ptyriasis Versicolor Over Abdomen And Upper Arms	NCS
S120	104	JAIVAC-1	Dosage A	1	Day 7	.		02/09/2010		Dermatological	Abnormal	Ptyriasis Versicolar Over Abdomen And Upper Arms	NCS
S120	104	JAIVAC-1	Dosage A	1	Day 14	.		08/09/2010		Dermatological	Abnormal	Ptyriasis Versicolar Over Abdomen And Upper Arms	NCS
S120	104	JAIVAC-1	Dosage A	2	Day 28	.		23/09/2010	Yes	Dermatological	Abnormal	Ptyriasis Versicolor Over Abdomen And Upper Arms	NCS
S120	104	JAIVAC-1	Dosage A	2	Day 35	.		30/09/2010		Dermatological	Abnormal	Ptyriasis Versicolor Over Abdomen And Upper Arms	NCS
S120	104	JAIVAC-1	Dosage A	2	Day 42	.		07/10/2010		Dermatological	Abnormal	Ptyriasis Versicolor Over Abdomen And Upper Arms	NCS

Subject No.	Randomisation Number	Study Group	Dosage	Dose	Day	Not Done	Specify	Date of Assessment	*Was the assessment performed prior to vaccination?	Physical Examination Parameter	Result	Abnormality	Clinical Significance
S120	104	JAIVAC-1	Dosage A	2	Day 56	.		21/10/2010		Dermatological	Abnormal	Ptyriasis Versicolor Over Abdomen And Upper Arms	NCS
S120	104	JAIVAC-1	Dosage A	3	Day 180	.		22/02/2011	Yes	Dermatological	Abnormal	Ptyriasis Versicolor Over Abdomen And Upper Arms	NCS
S120	104	JAIVAC-1	Dosage A	3	Day 187	.		01/03/2011		Dermatological	Abnormal	Ptyriasis Versicolor Over Abdomen And Upper Arms	NCS
S120	104	JAIVAC-1	Dosage A	3	Day 194	.		08/03/2011		Dermatological	Abnormal	Ptyriasis Versicolor Over Abdomen And Upper Arms	NCS
S120	104	JAIVAC-1	Dosage A	3	Day 208	.		22/03/2011		Dermatological	Abnormal	Ptyriasis Versicolor Over Abdomen And Upper Arms	NCS
S120	104	JAIVAC-1	Dosage A	3	Day 365	.		25/08/2011		Dermatological	Abnormal	Ptyriasis Versicolor Abdomen & Upper Arms	NCS
S132	108	Hepatitis B	Hepatitis B		Day -14 to Day -1	.		18/08/2010		Respiratory	Abnormal	Pectus Excavatum(Normal Variant)	NCS
S132	108	Hepatitis B	Hepatitis B	1	Day 0	.		26/08/2010	Yes	Respiratory	Abnormal	Pectus Excavatum	NCS
S132	108	Hepatitis B	Hepatitis B	1	Day 7	.		02/09/2010		Respiratory	Abnormal	Pectus Excavatum	NCS
S132	108	Hepatitis B	Hepatitis B	1	Day 14	.		09/09/2010		Respiratory	Abnormal	Pectus Excavatum	NCS

Subject No.	Randomisation Number	Study Group	Dosage	Dose	Day	Not Done	Specify	Date of Assessment	*Was the assessment performed prior to vaccination?	Physical Examination Parameter	Result	Abnormality	Clinical Significance
S132	108	Hepatitis B	Hepatitis B	2	Day 28	.		23/09/2010	Yes	Respiratory	Abnormal	Pectus Excavatum	NCS
S132	108	Hepatitis B	Hepatitis B	2	Day 35	.		30/09/2010		Respiratory	Abnormal	Pectus Excavatum	NCS
S132	108	Hepatitis B	Hepatitis B	2	Day 42	.		07/10/2010		Respiratory	Abnormal	Pectus Excavatum	NCS
S132	108	Hepatitis B	Hepatitis B	2	Day 56	.		21/10/2010		Respiratory	Abnormal	Pectus Excavatum	NCS
S132	108	Hepatitis B	Hepatitis B	3	Day 180	.		22/02/2011	Yes	Respiratory	Abnormal	Pectus Excavatum	NCS
S132	108	Hepatitis B	Hepatitis B	3	Day 187	.		01/03/2011		Respiratory	Abnormal	Pectus Excavatum	NCS
S132	108	Hepatitis B	Hepatitis B	3	Day 194	.		08/03/2011		Respiratory	Abnormal	Pectus Excavatum	NCS
S132	108	Hepatitis B	Hepatitis B	3	Day 208	.		22/03/2011		Respiratory	Abnormal	Pectus Excavatum	NCS
S132	108	Hepatitis B	Hepatitis B	3	Day 365	.		25/08/2011		Respiratory	Abnormal	Pectus Excavation	NCS
S148	111	JAIVAC-1	Dosage A	1	Day 14	.		10/09/2010		Musculoskeletal	Abnormal	Mild Tenderness Over Left Lower Forearm	CS
S182	208	JAIVAC-1	Dosage B	2	Day 35	.		03/11/2010		Eyes	Abnormal	Reddish Discoloration And Watering Of{l} Eye	CS
S279	301	JAIVAC-1	Dosage C	3	Day 194	.		31/05/2011		Dermatological	Abnormal	Sutured Wound Present Over Nose And Below Nostril(Soft Tissue Injury)	CS

Subject No.	Randomisation Number	Study Group	Dosage	Dose	Day	Not Done	Specify	Date of Assessment	*Was the assessment performed prior to vaccination?	Physical Examination Parameter	Result	Abnormality	Clinical Significance
S279	301	JAIVAC-1	Dosage C	3	Day 208	.		06/06/2011		Dermatological	Abnormal	Healed Wound Mark Present Over Nose And Below Left Nostril	NCS
S285	307	Hepatitis B	Hepatitis B	1	Day 7	.		18/11/2010		Eyes	Abnormal	Reddening Of Left Eye(+)	CS
S298	314	JAIVAC-1	Dosage C		Day -14 to Day -1	.		09/11/2010		Dermatological	Abnormal	Multiple Benign Lipomas On Both Arms	NCS
S298	314	JAIVAC-1	Dosage C	1	Day 0	.		12/11/2010	Yes	Dermatological	Abnormal	Multiple Benign Lipomas Seen	NCS
S298	314	JAIVAC-1	Dosage C	1	Day 7	.		19/11/2010		Dermatological	Abnormal	Multiple Benign Lipomas	NCS
S298	314	JAIVAC-1	Dosage C	1	Day 14	.		26/11/2010		Dermatological	Abnormal	Multiple Benign Lipomes Seen	NCS
S298	314	JAIVAC-1	Dosage C	2	Day 28	.		10/12/2010	Yes	Dermatological	Abnormal	Multiple Benign Lipomas	NCS
S298	314	JAIVAC-1	Dosage C	2	Day 35	.		17/12/2010		Dermatological	Abnormal	Multiple Benign Lipomas Seen On Left Arm	NCS
S298	314	JAIVAC-1	Dosage C	2	Day 42	.		24/12/2010		Dermatological	Abnormal	Multiple Benign Lipomas Seen On Left Arm	NCS
S298	314	JAIVAC-1	Dosage C	2	Day 56	.		07/01/2011		Dermatological	Abnormal	Benign Lipomas{l} Arm	NCS
S298	314	JAIVAC-1	Dosage C	3	Day 180	.		11/05/2011	Yes	Dermatological	Abnormal	Benign Lipomas{l} Arm	NCS
S298	314	JAIVAC-1	Dosage C	3	Day 187	.		18/05/2011		Dermatological	Abnormal	Benign Lipomas{l} Arm	NCS
S298	314	JAIVAC-1	Dosage C	3	Day 194	.		25/05/2011		Dermatological	Abnormal	Benign Lipomas{l} Arm	NCS
S298	314	JAIVAC-1	Dosage C	3	Day 208	.		08/06/2011		Dermatological	Abnormal	Benign Lipomas{l} Arm	NCS

Subject No.	Randomisation Number	Study Group	Dosage	Dose	Day	Not Done	Specify	Date of Assessment	*Was the assessment performed prior to vaccination?	Physical Examination Parameter	Result	Abnormality	Clinical Significance
S298	314	JAIVAC-1	Dosage C	3	Day 365	.		10/11/2011		Dermatological	Abnormal	Benign Lipomas(L) Arm	NCS
S125	102	JAIVAC-1	Dosage A		Day -14 to Day -1	.		18/08/2010		Other	Abnormal	Extra Rudimentory Nipple	NCS
S125	102	JAIVAC-1	Dosage A	1	Day 0	.		24/08/2010	Yes	Other	Abnormal	Extra Rudimentory Nipple	NCS
S125	102	JAIVAC-1	Dosage A	1	Day 7	.		31/08/2010		Other	Abnormal	Extra Rudimentory Nipple	NCS
S125	102	JAIVAC-1	Dosage A	1	Day 14	.		07/09/2010		Other	Abnormal	Extra Rudimentory Nipple	NCS
S125	102	JAIVAC-1	Dosage A	2	Day 28	.		21/09/2010	Yes	Other	Abnormal	Extra Rudimentory Nipple	NCS
S125	102	JAIVAC-1	Dosage A	2	Day 35	.		28/09/2010		Other	Abnormal	Extra Rudimentory Nipple	NCS
S125	102	JAIVAC-1	Dosage A	2	Day 42	.		05/10/2010		Other	Abnormal	Extra Rudimentory Nipple	NCS
S125	102	JAIVAC-1	Dosage A	2	Day 56	.		19/10/2010		Other	Abnormal	Extra Rudimentory Nipple	NCS

Note:
1) Clinical Significance: CS-Clinically Significant, NCS-Not Clinically Significant
2) * Was the assessment performed prior to vaccination? Is applicable for Day 0, Day 28 and Day 180

16.1 Study Information

Figure 16.1.1: Study Flowchart (Refer ICH E3 guidelines – ANNEX IV a)

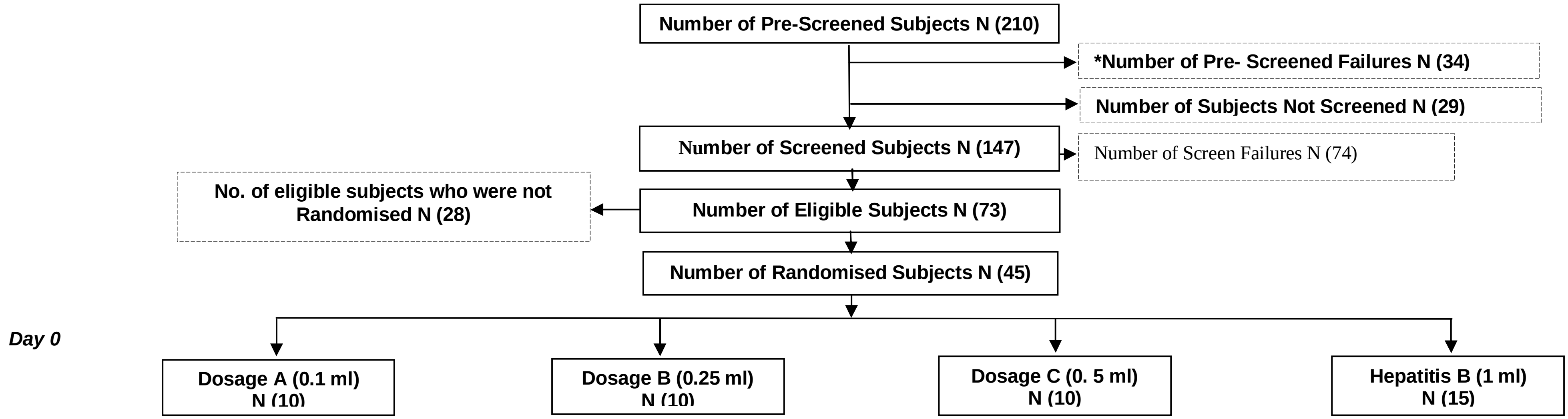

***Number of Pre-Screened Failures are subjects who do not qualify as per ELISA Assay for Anti-Malarial Antibodies**

Figure 16.1.2: Disposition of Subjects (Refer ICH E3 guidelines – ANNEX IV b)

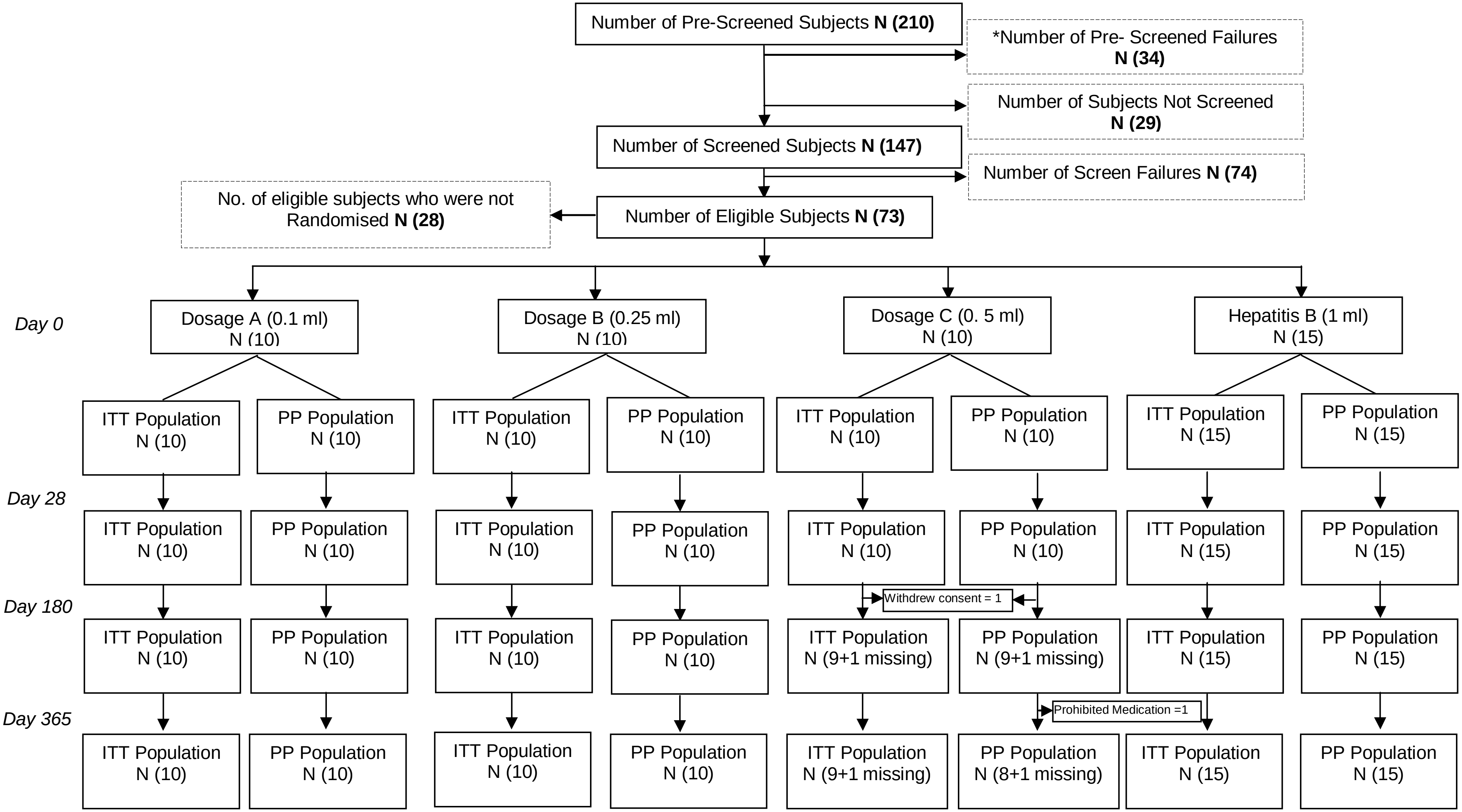

***Number of Pre-Screened Failures are subjects who does not qualifies ELISA for Anti-Malarial Antibodies**

16.1.6: Listing of Subjects receiving test vaccine/investigational product (s) from specific batches, where more than one batch was used

Note Applicable

16.1.7: Randomisation scheme and code (Subject identification and treatment assigned)

Original Final Randomisation List			Analysis Identification		
Cohort	Randomisation Number	Vaccine Assignment	Cohort	Randomisation Number	Actual Vaccine Received
1	101	JAIVAC-1	1	101	JAIVAC-1
1	102	JAIVAC-1	1	102	JAIVAC-1
1	103	Hepatitis B Vaccine	1	103	Hepatitis B Vaccine
1	104	JAIVAC-1	1	104	JAIVAC-1
1	105	JAIVAC-1	1	105	JAIVAC-1
1	106	Hepatitis B Vaccine	1	106	Hepatitis B Vaccine
1	107	Hepatitis B Vaccine	1	107	Hepatitis B Vaccine
1	108	Hepatitis B Vaccine	1	108	Hepatitis B Vaccine
1	109	JAIVAC-1	1	109	JAIVAC-1
1	110	JAIVAC-1	1	110	JAIVAC-1
1	111	JAIVAC-1	1	111	JAIVAC-1
1	112	JAIVAC-1	1	112	JAIVAC-1
1	113	JAIVAC-1	1	113	JAIVAC-1
1	114	JAIVAC-1	1	114	JAIVAC-1
1	115	Hepatitis B Vaccine	1	115	Hepatitis B Vaccine
2	201	JAIVAC-1	2	201	JAIVAC-1
2	202	JAIVAC-1	2	202	JAIVAC-1
2	203	JAIVAC-1	2	203	JAIVAC-1
2	204	Hepatitis B Vaccine	2	204	Hepatitis B Vaccine
2	205	Hepatitis B Vaccine	2	205	Hepatitis B Vaccine
2	206	JAIVAC-1	2	206	JAIVAC-1

Original Final Randomisation List			Analysis Identification		
**Cohort**	**Randomisation Number**	**Vaccine Assignment**	**Cohort**	**Randomisation Number**	**Actual Vaccine Received**
2	207	Hepatitis B Vaccine	2	207	Hepatitis B Vaccine
2	208	JAIVAC-1	2	208	JAIVAC-1
2	209	JAIVAC-1	2	209	JAIVAC-1
2	210	JAIVAC-1	2	210	JAIVAC-1
2	211	Hepatitis B Vaccine	2	211	Hepatitis B Vaccine
2	212	JAIVAC-1	2	212	JAIVAC-1
2	213	Hepatitis B Vaccine	2	213	Hepatitis B Vaccine
2	214	JAIVAC-1	2	214	JAIVAC-1
2	215	JAIVAC-1	2	215	JAIVAC-1
3	301	JAIVAC-1	3	301	JAIVAC-1
3	302	Hepatitis B Vaccine	3	302	Hepatitis B Vaccine
3	303	Hepatitis B Vaccine	3	303	Hepatitis B Vaccine
3	304	JAIVAC-1	3	304	JAIVAC-1
3	305	JAIVAC-1	3	305	JAIVAC-1
3	306	JAIVAC-1	3	306	JAIVAC-1
3	307	Hepatitis B Vaccine	3	307	Hepatitis B Vaccine
3	308	JAIVAC-1	3	308	JAIVAC-1
3	309	Hepatitis B Vaccine	3	309	Hepatitis B Vaccine
3	310	JAIVAC-1	3	310	JAIVAC-1
3	311	JAIVAC-1	3	311	JAIVAC-1
3	312	JAIVAC-1	3	312	JAIVAC-1
3	313	Hepatitis B Vaccine	3	313	Hepatitis B Vaccine

Original Final Randomisation List			Analysis Identification		
Cohort	Randomisation Number	Vaccine Assignment	Cohort	Randomisation Number	Actual Vaccine Received
3	314	JAIVAC-1	3	314	JAIVAC-1
3	315	JAIVAC-1	3	315	JAIVAC-1

16.1.9: Documentation of Statistical Methods

Categorical variable is summarised as follows:

1. Gender, Race, Physical Examination, Qualitative Laboratory Data: Frequency (n), Percentage (% - derived from Exact Binomial Proportion).

2. Subjects who experienced at least One Adverse Event of any category (LOcal AE / Systemic AE / Unsolicited AE / SAE): Frequency (n), Percentage (%- derived from Exact Binomial Proportion) and 95% CI.

Exact Binomial Proportion and 95% CIs are calculated using SAS by Clopper-Pearson Method.

3. Calculated Variable Geometric Mean Titers (GMTs) is summarised as Geometric Mean and 95% CI.

4. Incidences of Adverse Event of any category is summarised as Frequency (n).

Continuous variables except Titers is summarised using Mean, SEM, Median Minimum, Maximum, Range

1. Demographic Variables – Age, Height, weight,

2. Safety Data - Vital Signs, Quantitative Laboratory Data

3. Immunogenicity data: % Inhibition by GIA

Following Continuous variables is summarised using Mean, SEM, Median Minimum, Maximum, Inter-quartile Range (for log transformed data) / Range

1. Titers by ELISA – Log Transformed data.

2. ELISA for IgG subclass: Optical densities.

For comparing Continuous Immunogenicity Data among study groups:

1. Nonparametric Kruskal-Waliis test will be used for multiple group comparison is used.

2. Nonparametric Wilcoxon test will be used for paired comparison is used.

3. For GMT (log transformed data) - Two sample t-test is used.

4. For exploring Correlation between Assays - Spearman’s Rank correlation coefficient is used.

1. For comparing rest of the Continuous Data among study groups ANOVA is used.

For comparing Categorical Data among study groups Fisher’s Exact Test is used.

All comparisons mentioned above may not be powered as hypothesis testing is not planned as this is Phase I study and data is explored as much as possible for planning the further study.

16.1.10: Documentation of inter-laboratory standardization methods and quality assurance procedures if used

Note Applicable

16.2 Subject Data Listings

16.2.1 Discontinued Subjects

16.2.1.1: Listing of Subjects who are excluded from the study (Screen Failures)

Subject No.	Date pre-screening Informed Consent Signed	Is Subject Pre-screen Failure?	Date Main Informed Consent Signed	Date of Screening	Reason for Screen Failure	Date of Re-Screening	Reason for Re-Screen Failure
S097	09/08/2010	No	17/08/2010	17/08/2010	2	.	.
S098	09/08/2010	No	17/08/2010	17/08/2010	2	.	.
S100	09/08/2010	No	18/08/2010	18/08/2010	2	.	.
S101	09/08/2010	No	18/08/2010	18/08/2010	2	.	.
S102	09/08/2010	No	18/08/2010	18/08/2010	2	.	.
S104	09/08/2010	No	19/08/2010	19/08/2010	2	.	.
S105	09/08/2010	No	17/08/2010	17/08/2010	2	.	.
S107	09/08/2010	No	17/08/2010	17/08/2010	2	.	.
S109	09/08/2010	No	17/08/2010	17/08/2010	2	.	.
S110	09/08/2010	No	17/08/2010	17/08/2010	2	.	.
S111	09/08/2010	No	18/08/2010	18/08/2010	2	.	.
S113	09/08/2010	No	17/08/2010	17/08/2010	1	.	.
S115	09/08/2010	No	17/08/2010	17/08/2010	3	.	.
S126	10/08/2010	No	17/08/2010	17/08/2010	2	.	.
S128	11/08/2010	No	17/08/2010	17/08/2010	2	.	.
S129	11/08/2010	No	18/08/2010	18/08/2010	2	.	.
S131	11/08/2010	No	18/08/2010	18/08/2010	2	.	.
S134	11/08/2010	No	19/08/2010	19/08/2010	3	.	.
S135	12/08/2010	No	19/08/2010	19/08/2010	2	.	.
S137	12/08/2010	No	18/08/2010	18/08/2010	2	.	.
S139	12/08/2010	No	17/08/2010	17/08/2010	2	.	.
S146	13/08/2010	No	19/08/2010	19/08/2010	2	.	.
S151	13/08/2010	No	20/08/2010	20/08/2010	1	.	.

Subject No.	Date pre-screening Informed Consent Signed	Is Subject Pre-screen Failure?	Date Main Informed Consent Signed	Date of Screening	Reason for Screen Failure	Date of Re-Screening	Reason for Re-Screen Failure
S152	13/08/2010	No	19/08/2010	19/08/2010	2	.	.
S153	13/08/2010	No	19/08/2010	19/08/2010	2	.	.
S155	13/08/2010	No	19/08/2010	19/08/2010	2	.	.
S158	14/08/2010	No	20/08/2010	20/08/2010	2	.	.
S166	14/08/2010	No	19/08/2010	19/08/2010	2	.	.
S167	14/09/2010	No	21/09/2010	21/09/2010	2	.	.
S169	14/09/2010	No	21/09/2010	21/09/2010	1	.	.
S173	14/09/2010	No	23/09/2010	23/09/2010	2	.	.
S174	14/09/2010	No	23/09/2010	23/09/2010	2	.	.
S177	14/09/2010	No	21/09/2010	21/09/2010	1	.	.
S178	14/09/2010	No	23/09/2010	23/09/2010	2	.	.
S180	14/09/2010	No	22/09/2010	22/09/2010	2	.	.
S181	14/09/2010	No	23/09/2010	23/09/2010	2	.	.
S187	15/09/2010	No	22/09/2010	22/09/2010	1	.	.
S190	15/09/2010	No	22/09/2010	22/09/2010	2	.	.
S191	15/09/2010	No	23/09/2010	23/09/2010	1	.	.
S196	16/09/2010	No	23/09/2010	23/09/2010	2	.	.
S206	17/09/2010	No	23/09/2010	23/09/2010	1	.	.
S208	17/09/2010	No	23/09/2010	23/09/2010	2	.	.
S210	17/09/2010	No	23/09/2010	23/09/2010	2	.	.
S214	18/09/2010	No	24/09/2010	24/09/2010	2	.	.
S216	18/09/2010	No	24/09/2010	24/09/2010	2	.	.
S226	20/09/2010	No	24/09/2010	24/09/2010	2	.	.
S234	20/09/2010	No	24/09/2010	24/09/2010	2	.	.
S235	20/09/2010	No	24/09/2010	24/09/2010	2	.	.
S236	25/10/2010	No	03/11/2010	03/11/2010	2	.	.
S237	25/10/2010	No	03/11/2010	03/11/2010	2	.	.
S239	25/10/2010	No	02/11/2010	02/11/2010	2	.	.
S244	26/10/2010	No	02/11/2010	02/11/2010	2	.	.

Subject No.	Date pre-screening Informed Consent Signed	Is Subject Pre-screen Failure?	Date Main Informed Consent Signed	Date of Screening	Reason for Screen Failure	Date of Re-Screening	Reason for Re-Screen Failure
S246	26/10/2010	No	03/11/2010	03/11/2010	2	.	.
S247	26/10/2010	No	02/11/2010	02/11/2010	2	.	.
S249	26/10/2010	No	02/11/2010	02/11/2010	2	.	.
S251	26/10/2010	No	02/11/2010	02/11/2010	2	.	.
S252	26/10/2010	No	02/11/2010	02/11/2010	1	.	.
S254	26/10/2010	No	03/11/2010	03/11/2010	2	.	.
S268	27/10/2010	No	02/11/2010	02/11/2010	2	.	.
S273	27/10/2010	No	02/11/2010	02/11/2010	5	.	.
S276	28/10/2010	No	10/11/2010	10/11/2010	2	.	.
S280	28/10/2010	No	03/11/2010	03/11/2010	2	.	.
S282	28/10/2010	No	08/11/2010	08/11/2010	2	.	.
S286	29/10/2010	No	03/11/2010	03/11/2010	2	.	.
S287	29/10/2010	No	03/11/2010	03/11/2010	1	.	.
S289	29/10/2010	No	02/11/2010	02/11/2010	2	.	.
S290	29/10/2010	No	03/11/2010	03/11/2010	2	.	.
S292	29/10/2010	No	03/11/2010	03/11/2010	1	.	.
S294	29/10/2010	No	03/11/2010	03/11/2010	2	.	.
S295	29/10/2010	No	03/11/2010	03/11/2010	2	.	.
S296	29/10/2010	No	02/11/2010	02/11/2010	2	.	.
S300	30/10/2010	No	08/11/2010	08/11/2010	2	.	.
S303	30/10/2010	No	08/11/2010	08/11/2010	2	.	.
S305	30/10/2010	No	08/11/2010	08/11/2010	2	.	.

Note:
1) Reason for Screen Failure: 1 - Subject not fulfilling the Inclusion criteria, 2 - Subject not fulfilling the Exclusion criteria, 3 - Subject withdrew consent, 4 - Lost to follow up, 5 - Other

16.2.1.2: Listing of Subjects who Discontinued During the study

Subject No.	Randomisation Number	Study Group	Dosage	Date of Screening	Is the Subject Randomised?	If No, Specify	Date of Randomisation	Reason for Discontinuation	Detailed Reason for Discontinuation
S281	304	JAIVAC-1	Dosage C	03/11/2010	Yes		11/11/2010	Subject withdrew consent	Subject withdrew consent

16.2.1.3: Listing of Subjects who Discontinued During the study due to AEs

Subject No.	Randomisation Number	Study Group	Dosage	Dose	Date of Screening	Is the Subject Randomised?	If No, Specify	Date of Randomisation	Reason for Discontinuation	Reported AE Term	Preferred Term of MedDRA	Is AE Serious?	Seriousness Criteria
.	.	.	.	.	.	.	.	.	.	.	.	.	.

Note:
1) Seriousness Criteria: 1: Results In Death, 2-Is Life Threatening, 3-Requires Subject Hospitalization OR Prolongation of Existing Hospitalization, 4-Result in Persistent or Significant Disability/Incapacity, 5-Congenital Anomaly/ Birth Defect, 6-Other Important Medical Event
2) *No subject discontinued due to AE

16.2.2 Protocol Deviations

16.2.2.1: Listing of Protocol Deviations during the study

Subject No.	Randomisation Number	Study Group	Dosage	Day	Page Number	Module Name	Field Name	Reason for Protocol Deviation	Type of Deviation	Include in PP Analysis	Include in ITT Analysis
S108	115	Hepatitis B	Hepatitis B	Unscheduled Visit Dated 02/03/2011	4	Peripheral Blood Smear	Not Done: Specify	Protocol Violation: As Per The Resolution Provided For Qid 206, The Peripheral Blood Smear Test Was Not Preformed Even Though The Hemoglobin Level Was Less Than 13 G/Dl.	Minor	Yes	Yes
S108	115	Hepatitis B	Hepatitis B	Unscheduled Visit Dated 02/03/2011	4	Direct Coombs Test	Not Done: Specify	Protocol Violation: The Value Of Hemoglobin Is Less Than 13 G/Dl But The Direct Coomb's Test Was Not Performed As Per The Pis Discretion As Per The Resolution Provided On Qid 206.	Minor	Yes	Yes
S108	115	Hepatitis B	Hepatitis B	Unscheduled Visit Dated 13/04/2011	4	Direct Coombs Test	Not Done: Specify	Protocol Violation: The Value Of Hemoglobin Is Less Than 13 G/Dl But The Direct Coomb's Test Was Not Performed As Per The Pis Discretion As Per The Resolution Provided On Qid 205.	Minor	Yes	Yes
S170	204	Hepatitis B	Hepatitis B	Visit: 11 (Day 187)	1	Date Of Visit	Date Of Visit	Protocol Deviation: As Per Ntf Dated 20/May/2011 Visit 11 Was Scheduled On 04/April/2011, As The Subject Was Out Of Station And Came On 06/April/2011 Causing Deviation	Minor	Yes	Yes
S179	207	Hepatitis B	Hepatitis B	Visit: 11 (Day 187)	1	Date Of Visit	Date Of Visit	Protocol Deviation: As Per Ntf Dated 20/May/2011, Visit 11 Was Scheduled On 04 April 2011 But Subject Was Out Of Station. The Subject Came On 06th April, For The Visit Causing The Deviation	Minor	Yes	Yes
S182	208	JAIVAC-1	Dosage B	Visit: 3 (Day 0)	5	Immediate Reactogenicity	2 Hours: Actual Time	Protocol Deviation: As Per Resolution To Qid 60, Actual Time Of 2 Hours Post Vaccination To Be Considered As 13:48 And As Per The Note To File Dated 18/01/2011 Received From The Site, The First Dosing Was Done At 11:42 Hrs, The 2 Hr Time Point To Record Immediate Reactogenecity Should Have Ideally Done At 13:42 Hrs With +/- 5 Mins Window. However The Vitals Were Recorded At 13:48; Leading 1 Minute Deviation In The Time Point.	Minor	Yes	Yes

Subject No.	Randomisation Number	Study Group	Dosage	Day	Page Number	Module Name	Field Name	Reason for Protocol Deviation	Type of Deviation	Include in PP Analysis	Include in ITT Analysis
S195	214	JAIVAC-1	Dosage B	Visit: 4 (Day 7)	As Per Clinical Operations Tracker	As Per Clinical Operations Tracker	As Per Clinical Operations Tracker	Double Barrier Method Was Not Followed By Subject S195 When Assessed At Day 7 Visit. The Use Of Double Barrier Methods Of Contraception As Per Protocol Was Reiterated With The Subject And A Ntf Has Been Generated To Document This Deviation	Minor	Yes	Yes
S195	214	JAIVAC-1	Dosage B	Visit: 14 (Day 365)	5	Investigator Comments	None	Protocol Violation: The Value Of Hemoglobin Is Less Than 13 G/Dl But The Direct Coomb's Test Was Not Performed.	Minor	Yes	Yes
S202	206	JAIVAC-1	Dosage B	Visit: 4 (Day 7)	As Per Clinical Operations Tracker	As Per Clinical Operations Tracker	As Per Clinical Operations Tracker	Double Barrier Method Was Not Followed By Subject S202 When Assessed At Day 7 Visit. The Use Of Double Barrier Methods Of Contraception As Per Protocol Was Reiterated With The Subject And A Ntf Has Been Generated To Document This Deviation	Minor	Yes	Yes
S203	211	Hepatitis B	Hepatitis B	Unscheduled Visit Dated 03/01/2011	4	Peripheral Blood Smear	Not Done: Specify	Protocol Violation: As Per Updated Resolution For Qid 175 Received On 21/10/2011 Stated Direct Coombs Test And Peripheral Blood Smear Was Not Done And Only Hb Is Repeated At Local Lab As Per Investigator Discretion, Hence Columns For The Same Left Blank, Hb Value Being 12.7 Gm/Dl	Minor	Yes	Yes
S203	211	Hepatitis B	Hepatitis B	Visit: 13 (Day 208)	1	Date Of Visit	Date Of Visit	Protocol Deviation: As Per Ntf Dated 20/May/2011 Visit 13 Was Scheduled On 26/April/2011 And The Subject Had Scorpion Bite And Was Not Able To Come On This Day. The Subject Came On 28/April/2011 Causing The Deviation	Minor	Yes	Yes
S212	212	JAIVAC-1	Dosage B	Visit: 13 (Day 208)	1	Date Of Visit	Date Of Visit	Protocol Deviation: As Per Ntf Dated 20/May/2011 Visit 13 Was Scheduled On 26/April/2011, But The Subject Came On 19/April/2011 And Informed That He Would Be Out Of Country On The Scheduled Day, Hence Visit Was Conducted On 19/April/2011 Itself	Minor	Yes	Yes

Subject No.	Randomisation Number	Study Group	Dosage	Day	Page Number	Module Name	Field Name	Reason for Protocol Deviation	Type of Deviation	Include in PP Analysis	Include in ITT Analysis
S215	213	Hepatitis B	Hepatitis B	Visit: 3 (Day 0)	5	Immediate Reactogenicity	30 Minutes: Actual Time	Protocol Deviation: As Per Note To File Dated 06/10/2010 For Deviation In 30 Min Time Point: The Site Followed Their Sop Of Recording 30 Minutes After Completion Of Assessment Of Vital Signs Which Take 1 Minute. Hence, The Deviation Of 6 Minutes.	Minor	Yes	Yes
S279	301	JAIVAC-1	Dosage C	Concomitant Medications (Day 180 To Day 208)	1	Concomitant Medications (Day 180 To Day 208)	Drug Name	Protocol Violation: As Discussed(13/07/2011) With Sponsor And Ma Since The Said Subject Has Received The Prohibited Vaccine (Inj Tt) After The Third Dose Of The Study Vaccine, It Was Not Necessary To Discontinue The Subject At This Point In Time. It Would Be Prudent To Follow-Up The Subject For Safety Reasons And Conduct End-Of-Study Visit For This Subject In Line With The Protocol.	Major	No	Yes
S279	301	JAIVAC-1	Dosage C	Visit: 12 (Day 194)	1	Date Of Visit	Date Of Visit	Protocol Deviation: As Per The Ntf Dated 05/July/2011, Day 194 Visit Was Scheduled On 23/05/2011, However The Subject Had Informed That He Would Not Be Able To Come On The Scheduled Date And Visited Site On 31 May 2011 Causing Deviation Of 8 Days In Visit Window Period.	Minor	Yes	Yes
S281	304	JAIVAC-1	Dosage C	Visit: 8 (Day 42)	1	Date Of Visit	Date Of Visit	Protocol Deviation: As Per Note To File Dated 13/01/2011 For Visit Window Period: Subject Stated That His Friend Met With An Accident So He Was Pre Occupied And Could Not Visit The Site On 24/12/2010.	Minor	Yes	Yes
S291	312	JAIVAC-1	Dosage C	Visit: 4 (Day 7)	1	Date Of Visit	Date Of Visit	Protocol Deviation: As Per The Comments Mentioned On Visit 4 Page 4, There Was A Delay In The Date Of Visit 4. Ntf Has Been Generated For 2 Days Delay In Visit 4.	Minor	Yes	Yes

16.2.3 Subject excluded from the Immunogenicity analysis

16.2.3.1: Listing of Disposition of Subjects

Subject No.	Randomisation Number	Study Group	Dosage	Date of Pre-Screening	Date of Screening	Date of Re-Screening	Date of Randomisation	ITT Population*	Safety Population*	PP Population*
S097	.			09/08/2010	17/08/2010	.	.	No	No	No
S098	.			09/08/2010	17/08/2010	.	.	No	No	No
S099	.			09/08/2010	17/08/2010	.	.	No	No	No
S100	.			09/08/2010	18/08/2010	.	.	No	No	No
S101	.			09/08/2010	18/08/2010	.	.	No	No	No
S102	.			09/08/2010	18/08/2010	.	.	No	No	No
S103	.			09/08/2010	19/08/2010	.	.	No	No	No
S104	.			09/08/2010	19/08/2010	.	.	No	No	No
S105	.			09/08/2010	17/08/2010	.	.	No	No	No
S106	.			09/08/2010	17/08/2010	.	.	No	No	No
S107	.			09/08/2010	17/08/2010	.	.	No	No	No
S108	115	Hepatitis B	Hepatitis B	09/08/2010	18/08/2010	.	27/08/2010	Yes	Yes	Yes
S109	.			09/08/2010	17/08/2010	.	.	No	No	No
S110	.			09/08/2010	17/08/2010	.	.	No	No	No
S111	.			09/08/2010	18/08/2010	.	.	No	No	No
S112	.			09/08/2010	.	.	.	No	No	No
S113	.			09/08/2010	17/08/2010	.	.	No	No	No
S114	.			09/08/2010	.	.	.	No	No	No
S115	.			09/08/2010	17/08/2010	.	.	No	No	No
S116	.			09/08/2010	19/08/2010	.	.	No	No	No
S117	.			09/08/2010	19/08/2010	.	.	No	No	No
S118	114	JAIVAC-1	Dosage A	10/08/2010	17/08/2010	.	27/08/2010	Yes	Yes	Yes
S119	107	Hepatitis B	Hepatitis B	10/08/2010	18/08/2010	.	26/08/2010	Yes	Yes	Yes
S120	104	JAIVAC-1	Dosage A	10/08/2010	18/08/2010	.	26/08/2010	Yes	Yes	Yes
S121	.			10/08/2010	.	.	.	No	No	No

Subject No.	Randomisation Number	Study Group	Dosage	Date of Pre-Screening	Date of Screening	Date of Re-Screening	Date of Randomisation	ITT Population*	Safety Population*	PP Population*
S122	.			10/08/2010	.	.	.	No	No	No
S123	.			10/08/2010	19/08/2010	.	.	No	No	No
S124	106	Hepatitis B	Hepatitis B	10/08/2010	18/08/2010	.	26/08/2010	Yes	Yes	Yes
S125	102	JAIVAC-1	Dosage A	10/08/2010	18/08/2010	.	24/08/2010	Yes	Yes	Yes
S126	.			10/08/2010	17/08/2010	.	.	No	No	No
S127	.			10/08/2010	.	.	.	No	No	No
S128	.			11/08/2010	17/08/2010	.	.	No	No	No
S129	.			11/08/2010	18/08/2010	.	.	No	No	No
S130	105	JAIVAC-1	Dosage A	11/08/2010	17/08/2010	.	26/08/2010	Yes	Yes	Yes
S131	.			11/08/2010	18/08/2010	.	.	No	No	No
S132	108	Hepatitis B	Hepatitis B	11/08/2010	18/08/2010	.	26/08/2010	Yes	Yes	Yes
S133	.			11/08/2010	18/08/2010	.	.	No	No	No
S134	.			11/08/2010	19/08/2010	.	.	No	No	No
S135	.			12/08/2010	19/08/2010	.	.	No	No	No
S136	109	JAIVAC-1	Dosage A	12/08/2010	19/08/2010	.	26/08/2010	Yes	Yes	Yes
S137	.			12/08/2010	18/08/2010	.	.	No	No	No
S138	.			12/08/2010	18/08/2010	.	.	No	No	No
S139	.			12/08/2010	17/08/2010	.	.	No	No	No
S140	.			12/08/2010	18/08/2010	.	.	No	No	No
S141	.			12/08/2010	18/08/2010	.	.	No	No	No
S142	.			12/08/2010	.	.	.	No	No	No
S143	101	JAIVAC-1	Dosage A	12/08/2010	18/08/2010	.	24/08/2010	Yes	Yes	Yes
S144	103	Hepatitis B	Hepatitis B	13/08/2010	19/08/2010	.	24/08/2010	Yes	Yes	Yes
S145	112	JAIVAC-1	Dosage A	13/08/2010	19/08/2010	.	27/08/2010	Yes	Yes	Yes
S146	.			13/08/2010	19/08/2010	.	.	No	No	No
S147	.			13/08/2010	19/08/2010	.	.	No	No	No
S148	111	JAIVAC-1	Dosage A	13/08/2010	20/08/2010	.	27/08/2010	Yes	Yes	Yes
S149	.			13/08/2010	.	.	.	No	No	No
S150	.			13/08/2010	19/08/2010	.	.	No	No	No
S151	.			13/08/2010	20/08/2010	.	.	No	No	No

Subject No.	Randomisation Number	Study Group	Dosage	Date of Pre-Screening	Date of Screening	Date of Re-Screening	Date of Randomisation	ITT Population*	Safety Population*	PP Population*
S152	.			13/08/2010	19/08/2010	.	.	No	No	No
S153	.			13/08/2010	19/08/2010	.	.	No	No	No
S154	113	JAIVAC-1	Dosage A	13/08/2010	19/08/2010	.	27/08/2010	Yes	Yes	Yes
S155	.			13/08/2010	19/08/2010	.	.	No	No	No
S156	110	JAIVAC-1	Dosage A	13/08/2010	19/08/2010	.	27/08/2010	Yes	Yes	Yes
S157	.			13/08/2010	19/08/2010	.	.	No	No	No
S158	.			14/08/2010	20/08/2010	.	.	No	No	No
S159	.			14/08/2010	.	.	.	No	No	No
S160	.			14/08/2010	.	.	.	No	No	No
S161	.			14/08/2010	20/08/2010	.	.	No	No	No
S162	.			14/08/2010	.	.	.	No	No	No
S163	.			14/08/2010	.	.	.	No	No	No
S164	.			14/08/2010	.	.	.	No	No	No
S165	.			14/08/2010	.	.	.	No	No	No
S166	.			14/08/2010	19/08/2010	.	.	No	No	No
S167	.			14/09/2010	21/09/2010	.	.	No	No	No
S168	.			14/09/2010	.	.	.	No	No	No
S169	.			14/09/2010	21/09/2010	.	.	No	No	No
S170	204	Hepatitis B	Hepatitis B	14/09/2010	21/09/2010	.	29/09/2010	Yes	Yes	Yes
S171	.			14/09/2010	24/09/2010	.	.	No	No	No
S172	.			14/09/2010	.	.	.	No	No	No
S173	.			14/09/2010	23/09/2010	.	.	No	No	No
S174	.			14/09/2010	23/09/2010	.	.	No	No	No
S175	209	JAIVAC-1	Dosage B	14/09/2010	21/09/2010	.	29/09/2010	Yes	Yes	Yes
S176	205	Hepatitis B	Hepatitis B	14/09/2010	22/09/2010	.	29/09/2010	Yes	Yes	Yes
S177	.			14/09/2010	21/09/2010	.	.	No	No	No
S178	.			14/09/2010	23/09/2010	.	.	No	No	No
S179	207	Hepatitis B	Hepatitis B	14/09/2010	22/09/2010	.	29/09/2010	Yes	Yes	Yes
S180	.			14/09/2010	22/09/2010	.	.	No	No	No
S181	.			14/09/2010	23/09/2010	.	.	No	No	No

Subject No.	Randomisation Number	Study Group	Dosage	Date of Pre-Screening	Date of Screening	Date of Re-Screening	Date of Randomisation	ITT Population*	Safety Population*	PP Population*
S182	208	JAIVAC-1	Dosage B	15/09/2010	21/09/2010	.	29/09/2010	Yes	Yes	Yes
S183	.			15/09/2010	.	.	.	No	No	No
S184	.			15/09/2010	22/09/2010	.	.	No	No	No
S185	202	JAIVAC-1	Dosage B	15/09/2010	21/09/2010	.	28/09/2010	Yes	Yes	Yes
S186	201	JAIVAC-1	Dosage B	15/09/2010	21/09/2010	.	28/09/2010	Yes	Yes	Yes
S187	.			15/09/2010	22/09/2010	.	.	No	No	No
S188	.			15/09/2010	21/09/2010	.	.	No	No	No
S189	.			15/09/2010	.	.	.	No	No	No
S190	.			15/09/2010	22/09/2010	.	.	No	No	No
S191	.			15/09/2010	23/09/2010	.	.	No	No	No
S192	203	JAIVAC-1	Dosage B	16/09/2010	23/09/2010	.	28/09/2010	Yes	Yes	Yes
S193	215	JAIVAC-1	Dosage B	16/09/2010	24/09/2010	.	30/09/2010	Yes	Yes	Yes
S194	.			16/09/2010	.	.	.	No	No	No
S195	214	JAIVAC-1	Dosage B	16/09/2010	23/09/2010	.	30/09/2010	Yes	Yes	Yes
S196	.			16/09/2010	23/09/2010	.	.	No	No	No
S197	.			16/09/2010	.	.	.	No	No	No
S198	.			17/09/2010	.	.	.	No	No	No
S199	.			17/09/2010	.	.	.	No	No	No
S200	.			17/09/2010	23/09/2010	.	.	No	No	No
S201	.			17/09/2010	.	.	.	No	No	No
S202	206	JAIVAC-1	Dosage B	17/09/2010	23/09/2010	.	29/09/2010	Yes	Yes	Yes
S203	211	Hepatitis B	Hepatitis B	17/09/2010	24/09/2010	.	30/09/2010	Yes	Yes	Yes
S204	.			17/09/2010	.	.	.	No	No	No
S205	.			17/09/2010	.	.	.	No	No	No
S206	.			17/09/2010	23/09/2010	.	.	No	No	No
S207	.			17/09/2010	23/09/2010	.	.	No	No	No
S208	.			17/09/2010	23/09/2010	.	.	No	No	No
S209	.			17/09/2010	.	.	.	No	No	No
S210	.			17/09/2010	23/09/2010	.	.	No	No	No
S211	.			17/09/2010	.	.	.	No	No	No

Subject No.	Randomisation Number	Study Group	Dosage	Date of Pre-Screening	Date of Screening	Date of Re-Screening	Date of Randomisation	ITT Population*	Safety Population*	PP Population*
S212	212	JAIVAC-1	Dosage B	18/09/2010	23/09/2010	.	30/09/2010	Yes	Yes	Yes
S213	.			18/09/2010	.	.	.	No	No	No
S214	.			18/09/2010	24/09/2010	.	.	No	No	No
S215	213	Hepatitis B	Hepatitis B	18/09/2010	24/09/2010	.	30/09/2010	Yes	Yes	Yes
S216	.			18/09/2010	24/09/2010	.	.	No	No	No
S217	.			18/09/2010	.	.	.	No	No	No
S218	.			18/09/2010	24/09/2010	.	.	No	No	No
S219	.			18/09/2010	.	.	.	No	No	No
S220	.			18/09/2010	.	.	.	No	No	No
S221	.			18/09/2010	24/09/2010	.	.	No	No	No
S222	.			18/09/2010	.	.	.	No	No	No
S223	.			20/09/2010	.	.	.	No	No	No
S224	.			20/09/2010	.	.	.	No	No	No
S225	.			20/09/2010	.	.	.	No	No	No
S226	.			20/09/2010	24/09/2010	.	.	No	No	No
S227	.			20/09/2010	.	.	.	No	No	No
S228	.			20/09/2010	.	.	.	No	No	No
S229	.			20/09/2010	.	.	.	No	No	No
S230	.			20/09/2010	24/09/2010	.	.	No	No	No
S231	.			20/09/2010	.	.	.	No	No	No
S232	.			20/09/2010	.	.	.	No	No	No
S233	210	JAIVAC-1	Dosage B	20/09/2010	24/09/2010	.	30/09/2010	Yes	Yes	Yes
S234	.			20/09/2010	24/09/2010	.	.	No	No	No
S235	.			20/09/2010	24/09/2010	.	.	No	No	No
S236	.			25/10/2010	03/11/2010	.	.	No	No	No
S237	.			25/10/2010	03/11/2010	.	.	No	No	No
S238	.			25/10/2010	.	.	.	No	No	No
S239	.			25/10/2010	02/11/2010	.	.	No	No	No
S240	.			26/10/2010	.	.	.	No	No	No
S241	303	Hepatitis B	Hepatitis B	26/10/2010	03/11/2010	.	10/11/2010	Yes	Yes	Yes

Subject No.	Randomisation Number	Study Group	Dosage	Date of Pre-Screening	Date of Screening	Date of Re-Screening	Date of Randomisation	ITT Population*	Safety Population*	PP Population*
S242	.			26/10/2010	.	.	.	No	No	No
S243	.			26/10/2010	.	.	.	No	No	No
S244	.			26/10/2010	02/11/2010	.	.	No	No	No
S245	.			26/10/2010	.	.	.	No	No	No
S246	.			26/10/2010	03/11/2010	.	.	No	No	No
S247	.			26/10/2010	02/11/2010	.	.	No	No	No
S248	.			26/10/2010	.	.	.	No	No	No
S249	.			26/10/2010	02/11/2010	.	.	No	No	No
S250	311	JAIVAC-1	Dosage C	26/10/2010	03/11/2010	.	12/11/2010	Yes	Yes	Yes
S251	.			26/10/2010	02/11/2010	.	.	No	No	No
S252	.			26/10/2010	02/11/2010	.	.	No	No	No
S253	.			26/10/2010	.	.	.	No	No	No
S254	.			26/10/2010	03/11/2010	.	.	No	No	No
S255	.			26/10/2010	.	.	.	No	No	No
S256	.			27/10/2010	.	.	.	No	No	No
S257	.			27/10/2010	.	.	.	No	No	No
S258	.			27/10/2010	02/11/2010	.	.	No	No	No
S259	.			27/10/2010	.	.	.	No	No	No
S260	.			27/10/2010	02/11/2010	.	.	No	No	No
S261	.			27/10/2010	.	.	.	No	No	No
S262	.			27/10/2010	.	.	.	No	No	No
S263	313	Hepatitis B	Hepatitis B	27/10/2010	09/11/2010	.	12/11/2010	Yes	Yes	Yes
S264	306	JAIVAC-1	Dosage C	27/10/2010	03/11/2010	.	11/11/2010	Yes	Yes	Yes
S265	.			27/10/2010	.	.	.	No	No	No
S266	308	JAIVAC-1	Dosage C	27/10/2010	03/11/2010	.	11/11/2010	Yes	Yes	Yes
S267	309	Hepatitis B	Hepatitis B	27/10/2010	02/11/2010	.	11/11/2010	Yes	Yes	Yes
S268	.			27/10/2010	02/11/2010	.	.	No	No	No
S269	.			27/10/2010	.	.	.	No	No	No
S270	.			27/10/2010	.	.	.	No	No	No
S271	.			27/10/2010	.	.	.	No	No	No

Subject No.	Randomisation Number	Study Group	Dosage	Date of Pre-Screening	Date of Screening	Date of Re-Screening	Date of Randomisation	ITT Population*	Safety Population*	PP Population*
S272	.			27/10/2010	02/11/2010	.	.	No	No	No
S273	.			27/10/2010	02/11/2010	.	.	No	No	No
S274	.			27/10/2010	02/11/2010	.	.	No	No	No
S275	302	Hepatitis B	Hepatitis B	28/10/2010	02/11/2010	.	10/11/2010	Yes	Yes	Yes
S276	.			28/10/2010	10/11/2010	.	.	No	No	No
S277	.			28/10/2010	03/11/2010	.	.	No	No	No
S278	315	JAIVAC-1	Dosage C	28/10/2010	08/11/2010	.	12/11/2010	Yes	Yes	Yes
S279	301	JAIVAC-1	Dosage C	28/10/2010	03/11/2010	.	10/11/2010	Yes	Yes	No
S280	.			28/10/2010	03/11/2010	.	.	No	No	No
S281	304	JAIVAC-1	Dosage C	28/10/2010	03/11/2010	.	11/11/2010	Yes	Yes	Yes
S282	.			28/10/2010	08/11/2010	.	.	No	No	No
S283	.			29/10/2010	.	.	.	No	No	No
S284	305	JAIVAC-1	Dosage C	29/10/2010	03/11/2010	.	11/11/2010	Yes	Yes	Yes
S285	307	Hepatitis B	Hepatitis B	29/10/2010	02/11/2010	.	11/11/2010	Yes	Yes	Yes
S286	.			29/10/2010	03/11/2010	.	.	No	No	No
S287	.			29/10/2010	03/11/2010	.	.	No	No	No
S288	.			29/10/2010	.	.	.	No	No	No
S289	.			29/10/2010	02/11/2010	.	.	No	No	No
S290	.			29/10/2010	03/11/2010	.	.	No	No	No
S291	312	JAIVAC-1	Dosage C	29/10/2010	02/11/2010	.	12/11/2010	Yes	Yes	Yes
S292	.			29/10/2010	03/11/2010	.	.	No	No	No
S293	.			29/10/2010	.	.	.	No	No	No
S294	.			29/10/2010	03/11/2010	.	.	No	No	No
S295	.			29/10/2010	03/11/2010	.	.	No	No	No
S296	.			29/10/2010	02/11/2010	.	.	No	No	No
S297	.			29/10/2010	.	.	.	No	No	No
S298	314	JAIVAC-1	Dosage C	30/10/2010	09/11/2010	.	12/11/2010	Yes	Yes	Yes
S299	310	JAIVAC-1	Dosage C	30/10/2010	08/11/2010	.	12/11/2010	Yes	Yes	Yes
S300	.			30/10/2010	08/11/2010	.	.	No	No	No
S301	.			30/10/2010	.	.	.	No	No	No

Subject No.	Randomisation Number	Study Group	Dosage	Date of Pre-Screening	Date of Screening	Date of Re-Screening	Date of Randomisation	ITT Population*	Safety Population*	PP Population*
S302	.			30/10/2010	.	.	.	No	No	No
S303	.			30/10/2010	08/11/2010	.	.	No	No	No
S304	.			30/10/2010	08/11/2010	.	.	No	No	No
S305	.			30/10/2010	08/11/2010	.	.	No	No	No
S306	.			30/10/2010	.	.	.	No	No	No

Note:
1) ITT Population: Include the Randomised subjects who will receive at least one dose of either of the assigned vaccines.
2) PP Population: Include the number of subjects from the ITT set who are in compliance with the study protocol.
* Yes-Subject included in immunogenicity analysis, No-Subject not included in immunogenicity analysis.

16.2.4 Demographic data

16.2.4.1: Listing of Demographic data – Baseline Visit

Subject No.	Randomisation Number	Study Group	Dosage	Age (years)	Date of Birth	Height (cm)	Weight (Kg)	Race	Other, Specify
S108	115	Hepatitis B	Hepatitis B	24.88	29/09/1985	183.3	66.2	Indian	
S118	114	JAIVAC-1	Dosage A	21.55	25/01/1989	167.8	57.3	Indian	
S119	107	Hepatitis B	Hepatitis B	27.35	12/04/1983	168.8	58.4	Indian	
S120	104	JAIVAC-1	Dosage A	24.42	15/03/1986	160.6	55.5	Indian	
S124	106	Hepatitis B	Hepatitis B	22.59	13/01/1988	163.2	50.5	Indian	
S125	102	JAIVAC-1	Dosage A	28.63	31/12/1981	168.8	70	Indian	
S130	105	JAIVAC-1	Dosage A	31.48	25/02/1979	170.8	50.5	Indian	
S132	108	Hepatitis B	Hepatitis B	37.29	05/05/1973	174.5	63.1	Indian	
S136	109	JAIVAC-1	Dosage A	31.27	14/05/1979	180.1	82.4	Indian	
S143	101	JAIVAC-1	Dosage A	33.50	19/02/1977	167.6	71.4	Indian	
S144	103	Hepatitis B	Hepatitis B	25.50	17/02/1985	170	65.6	Indian	
S145	112	JAIVAC-1	Dosage A	25.96	03/09/1984	167.7	52.4	Indian	
S148	111	JAIVAC-1	Dosage A	27.37	06/04/1983	168.2	72.8	Indian	
S154	113	JAIVAC-1	Dosage A	24.21	03/06/1986	168	72.6	Indian	
S156	110	JAIVAC-1	Dosage A	19.58	17/01/1991	182.5	64.4	Indian	
S170	204	Hepatitis B	Hepatitis B	21.83	20/11/1988	160	56.3	Indian	
S175	209	JAIVAC-1	Dosage B	25.15	27/07/1985	159.4	50.3	Indian	
S176	205	Hepatitis B	Hepatitis B	36.17	22/07/1974	170	66	Indian	
S179	207	Hepatitis B	Hepatitis B	25.27	13/06/1985	169.9	57.6	Indian	
S182	208	JAIVAC-1	Dosage B	26.96	08/10/1983	177	66	Indian	
S185	202	JAIVAC-1	Dosage B	19.60	12/02/1991	164	54.3	Indian	
S186	201	JAIVAC-1	Dosage B	39.64	02/02/1971	169.1	72	Indian	
S192	203	JAIVAC-1	Dosage B	27.35	18/05/1983	171.4	58	Indian	
S193	215	JAIVAC-1	Dosage B	25.37	10/05/1985	177.3	82.7	Indian	
S195	214	JAIVAC-1	Dosage B	27.30	05/06/1983	157.4	55.8	Indian	
S202	206	JAIVAC-1	Dosage B	39.28	18/06/1971	170	71.8	Indian	

Subject No.	Randomisation Number	Study Group	Dosage	Age (years)	Date of Birth	Height (cm)	Weight (Kg)	Race	Other, Specify
S203	211	Hepatitis B	Hepatitis B	28.31	02/06/1982	166.7	70.2	Indian	
S212	212	JAIVAC-1	Dosage B	27.00	25/09/1983	170.8	55	Indian	
S215	213	Hepatitis B	Hepatitis B	26.98	01/10/1983	173.6	67.4	Indian	
S233	210	JAIVAC-1	Dosage B	31.72	10/01/1979	167.8	69.2	Indian	
S241	303	Hepatitis B	Hepatitis B	25.43	28/05/1985	170.8	57.5	Indian	
S250	311	JAIVAC-1	Dosage C	40.33	08/07/1970	164	57.5	Indian	
S263	313	Hepatitis B	Hepatitis B	23.56	13/04/1987	162.8	65.7	Indian	
S264	306	JAIVAC-1	Dosage C	31.07	10/10/1979	159.4	65	Indian	
S266	308	JAIVAC-1	Dosage C	38.24	09/08/1972	158.1	59.9	Indian	
S267	309	Hepatitis B	Hepatitis B	23.42	31/05/1987	170.4	67	Indian	
S275	302	Hepatitis B	Hepatitis B	25.37	21/06/1985	162.5	62.4	Indian	
S278	315	JAIVAC-1	Dosage C	25.24	09/08/1985	166.1	68.5	Indian	
S279	301	JAIVAC-1	Dosage C	26.31	13/07/1984	180.8	85	Indian	
S281	304	JAIVAC-1	Dosage C	26.59	03/04/1984	168	57	Indian	
S284	305	JAIVAC-1	Dosage C	21.62	23/03/1989	170.5	72.2	Indian	
S285	307	Hepatitis B	Hepatitis B	26.81	15/01/1984	161	63	Indian	
S291	312	JAIVAC-1	Dosage C	18.59	01/04/1992	170.1	64.7	Indian	
S298	314	JAIVAC-1	Dosage C	25.56	14/04/1985	168.1	72.4	Indian	
S299	310	JAIVAC-1	Dosage C	24.48	12/05/1986	168.1	52.9	Indian	

Note:
1) Age (years) is calculated as the difference between the date of first informed consent signed and date of birth

16.2.4.2: Demographic data – Other Evaluation Parameters

Subject No.	Randomisation Number	Study Group	Dosage	Age (years)	Date of sample Collection	Elisa (Anti-malarial antibody)
S108	115	Hepatitis B	Hepatitis B	24.88	09/08/2010	Subject qualifies
S118	114	JAIVAC-1	Dosage A	21.55	10/08/2010	Subject qualifies
S119	107	Hepatitis B	Hepatitis B	27.35	10/08/2010	Subject qualifies
S120	104	JAIVAC-1	Dosage A	24.42	10/08/2010	Subject qualifies
S124	106	Hepatitis B	Hepatitis B	22.59	10/08/2010	Subject qualifies
S125	102	JAIVAC-1	Dosage A	28.63	10/08/2010	Subject qualifies
S130	105	JAIVAC-1	Dosage A	31.48	11/08/2010	Subject qualifies
S132	108	Hepatitis B	Hepatitis B	37.29	11/08/2010	Subject qualifies
S136	109	JAIVAC-1	Dosage A	31.27	12/08/2010	Subject qualifies
S143	101	JAIVAC-1	Dosage A	33.50	12/08/2010	Subject qualifies
S144	103	Hepatitis B	Hepatitis B	25.50	13/08/2010	Subject qualifies
S145	112	JAIVAC-1	Dosage A	25.96	13/08/2010	Subject qualifies
S148	111	JAIVAC-1	Dosage A	27.37	13/08/2010	Subject qualifies
S154	113	JAIVAC-1	Dosage A	24.21	13/08/2010	Subject qualifies
S156	110	JAIVAC-1	Dosage A	19.58	13/08/2010	Subject qualifies
S170	204	Hepatitis B	Hepatitis B	21.83	14/09/2010	Subject qualifies
S175	209	JAIVAC-1	Dosage B	25.15	14/09/2010	Subject qualifies
S176	205	Hepatitis B	Hepatitis B	36.17	14/09/2010	Subject qualifies
S179	207	Hepatitis B	Hepatitis B	25.27	14/09/2010	Subject qualifies
S182	208	JAIVAC-1	Dosage B	26.96	15/09/2010	Subject qualifies
S185	202	JAIVAC-1	Dosage B	19.60	15/09/2010	Subject qualifies
S186	201	JAIVAC-1	Dosage B	39.64	15/09/2010	Subject qualifies
S192	203	JAIVAC-1	Dosage B	27.35	16/09/2010	Subject qualifies
S193	215	JAIVAC-1	Dosage B	25.37	16/09/2010	Subject qualifies
S195	214	JAIVAC-1	Dosage B	27.30	16/09/2010	Subject qualifies
S202	206	JAIVAC-1	Dosage B	39.28	17/09/2010	Subject qualifies
S203	211	Hepatitis B	Hepatitis B	28.31	17/09/2010	Subject qualifies
S212	212	JAIVAC-1	Dosage B	27.00	18/09/2010	Subject qualifies

Subject No.	Randomisation Number	Study Group	Dosage	Age (years)	Date of sample Collection	Elisa (Anti-malarial antibody)
S215	213	Hepatitis B	Hepatitis B	26.98	18/09/2010	Subject qualifies
S233	210	JAIVAC-1	Dosage B	31.72	20/09/2010	Subject qualifies
S241	303	Hepatitis B	Hepatitis B	25.43	26/10/2010	Subject qualifies
S250	311	JAIVAC-1	Dosage C	40.33	26/10/2010	Subject qualifies
S263	313	Hepatitis B	Hepatitis B	23.56	27/10/2010	Subject qualifies
S264	306	JAIVAC-1	Dosage C	31.07	27/10/2010	Subject qualifies
S266	308	JAIVAC-1	Dosage C	38.24	27/10/2010	Subject qualifies
S267	309	Hepatitis B	Hepatitis B	23.42	27/10/2010	Subject qualifies
S275	302	Hepatitis B	Hepatitis B	25.37	28/10/2010	Subject qualifies
S278	315	JAIVAC-1	Dosage C	25.24	28/10/2010	Subject qualifies
S279	301	JAIVAC-1	Dosage C	26.31	28/10/2010	Subject qualifies
S281	304	JAIVAC-1	Dosage C	26.59	28/10/2010	Subject qualifies
S284	305	JAIVAC-1	Dosage C	21.62	29/10/2010	Subject qualifies
S285	307	Hepatitis B	Hepatitis B	26.81	29/10/2010	Subject qualifies
S291	312	JAIVAC-1	Dosage C	18.59	29/10/2010	Subject qualifies
S298	314	JAIVAC-1	Dosage C	25.56	30/10/2010	Subject qualifies
S299	310	JAIVAC-1	Dosage C	24.48	30/10/2010	Subject qualifies

Note:
1) Age (years) is calculated as the difference between the date of first informed consent signed and date of birth

16.2.5 Compliance and/or drug concentration data

16.2.5.1: Listing of Vaccine Administration Record for Study Visits

Subject No.	Randomisation Number	Study Group	Dosage	Dose	Day	Date of Vaccination	Dose of Vaccination (ml)	Time of Vaccination (HH:MM)	Site of Vaccination
S108	115	Hepatitis B	Hepatitis B	1	Day 0	27/08/2010	1	15:02	Non-dominant deltoid
S108	115	Hepatitis B	Hepatitis B	2	Day 28	24/09/2010	1	11:33	Dominant deltoid
S108	115	Hepatitis B	Hepatitis B	3	Day 180	23/02/2011	1	12:49	Non-dominant deltoid
S118	114	JAIVAC-1	Dosage A	1	Day 0	27/08/2010	0.1	14:41	Non-dominant deltoid
S118	114	JAIVAC-1	Dosage A	2	Day 28	24/09/2010	0.1	11:20	Dominant deltoid
S118	114	JAIVAC-1	Dosage A	3	Day 180	23/02/2011	0.1	12:28	Non-dominant deltoid
S119	107	Hepatitis B	Hepatitis B	1	Day 0	26/08/2010	1	12:41	Non-dominant deltoid
S119	107	Hepatitis B	Hepatitis B	2	Day 28	23/09/2010	1	11:08	Dominant deltoid
S119	107	Hepatitis B	Hepatitis B	3	Day 180	22/02/2011	1	12:02	Non-dominant deltoid
S120	104	JAIVAC-1	Dosage A	1	Day 0	26/08/2010	0.1	11:23	Non-dominant deltoid
S120	104	JAIVAC-1	Dosage A	2	Day 28	23/09/2010	0.1	10:29	Dominant deltoid
S120	104	JAIVAC-1	Dosage A	3	Day 180	22/02/2011	0.1	10:58	Non-dominant deltoid
S124	106	Hepatitis B	Hepatitis B	1	Day 0	26/08/2010	1	12:28	Non-dominant deltoid
S124	106	Hepatitis B	Hepatitis B	2	Day 28	23/09/2010	1	10:47	Dominant deltoid
S124	106	Hepatitis B	Hepatitis B	3	Day 180	22/02/2011	1	11:53	Non-dominant deltoid
S125	102	JAIVAC-1	Dosage A	1	Day 0	24/08/2010	0.1	14:14	Non-dominant deltoid
S125	102	JAIVAC-1	Dosage A	2	Day 28	21/09/2010	0.1	11:38	Dominant deltoid
S125	102	JAIVAC-1	Dosage A	3	Day 180	21/02/2011	0.1	11:18	Non-dominant deltoid
S130	105	JAIVAC-1	Dosage A	1	Day 0	26/08/2010	0.1	12:05	Non-dominant deltoid
S130	105	JAIVAC-1	Dosage A	2	Day 28	23/09/2010	0.1	9:43	Dominant deltoid
S130	105	JAIVAC-1	Dosage A	3	Day 180	22/02/2011	0.1	11:15	Non-dominant deltoid
S132	108	Hepatitis B	Hepatitis B	1	Day 0	26/08/2010	1	12:56	Non-dominant deltoid
S132	108	Hepatitis B	Hepatitis B	2	Day 28	23/09/2010	1	11:16	Dominant deltoid
S132	108	Hepatitis B	Hepatitis B	3	Day 180	22/02/2011	1	12:12	Non-dominant deltoid
S136	109	JAIVAC-1	Dosage A	1	Day 0	26/08/2010	0.1	13:37	Non-dominant deltoid

Subject No.	Randomisation Number	Study Group	Dosage	Dose	Day	Date of Vaccination	Dose of Vaccination (ml)	Time of Vaccination (HH:MM)	Site of Vaccination
S136	109	JAIVAC-1	Dosage A	2	Day 28	23/09/2010	0.1	11:44	Dominant deltoid
S136	109	JAIVAC-1	Dosage A	3	Day 180	22/02/2011	0.1	11:43	Non-dominant deltoid
S143	101	JAIVAC-1	Dosage A	1	Day 0	24/08/2010	0.1	12:48	Non-dominant deltoid
S143	101	JAIVAC-1	Dosage A	2	Day 28	21/09/2010	0.1	10:43	Dominant deltoid
S143	101	JAIVAC-1	Dosage A	3	Day 180	21/02/2011	0.1	10:37	Non-dominant deltoid
S144	103	Hepatitis B	Hepatitis B	1	Day 0	24/08/2010	1	14:37	Non-dominant deltoid
S144	103	Hepatitis B	Hepatitis B	2	Day 28	21/09/2010	1	11:10	Dominant deltoid
S144	103	Hepatitis B	Hepatitis B	3	Day 180	21/02/2011	1	10:46	Non-dominant deltoid
S145	112	JAIVAC-1	Dosage A	1	Day 0	27/08/2010	0.1	12:52	Non-dominant deltoid
S145	112	JAIVAC-1	Dosage A	2	Day 28	24/09/2010	0.1	10:32	Dominant deltoid
S145	112	JAIVAC-1	Dosage A	3	Day 180	23/02/2011	0.1	11:41	Non-dominant deltoid
S148	111	JAIVAC-1	Dosage A	1	Day 0	27/08/2010	0.1	12:12	Non-dominant deltoid
S148	111	JAIVAC-1	Dosage A	2	Day 28	24/09/2010	0.1	10:08	Dominant deltoid
S148	111	JAIVAC-1	Dosage A	3	Day 180	23/02/2011	0.1	11:12	Non-dominant deltoid
S154	113	JAIVAC-1	Dosage A	1	Day 0	27/08/2010	0.1	13:30	Non-dominant deltoid
S154	113	JAIVAC-1	Dosage A	2	Day 28	24/09/2010	0.1	10:52	Dominant deltoid
S154	113	JAIVAC-1	Dosage A	3	Day 180	23/02/2011	0.1	12:04	Non-dominant deltoid
S156	110	JAIVAC-1	Dosage A	1	Day 0	27/08/2010	0.1	10:14	Non-dominant deltoid
S156	110	JAIVAC-1	Dosage A	2	Day 28	24/09/2010	0.1	9:47	Dominant deltoid
S156	110	JAIVAC-1	Dosage A	3	Day 180	23/02/2011	0.1	10:53	Non-dominant deltoid
S170	204	Hepatitis B	Hepatitis B	1	Day 0	29/09/2010	1	11:14	Non-dominant deltoid
S170	204	Hepatitis B	Hepatitis B	2	Day 28	27/10/2010	1	10:35	Dominant deltoid
S170	204	Hepatitis B	Hepatitis B	3	Day 180	28/03/2011	1	12:18	Non-dominant deltoid
S175	209	JAIVAC-1	Dosage B	1	Day 0	29/09/2010	0.25	11:50	Non-dominant deltoid
S175	209	JAIVAC-1	Dosage B	2	Day 28	27/10/2010	0.25	11:37	Dominant deltoid
S175	209	JAIVAC-1	Dosage B	3	Day 180	28/03/2011	0.25	12:09	Non-dominant deltoid
S176	205	Hepatitis B	Hepatitis B	1	Day 0	29/09/2010	1	11:21	Non-dominant deltoid
S176	205	Hepatitis B	Hepatitis B	2	Day 28	27/10/2010	1	12:09	Dominant deltoid
S176	205	Hepatitis B	Hepatitis B	3	Day 180	28/03/2011	1	12:26	Non-dominant deltoid

Subject No.	Randomisation Number	Study Group	Dosage	Dose	Day	Date of Vaccination	Dose of Vaccination (ml)	Time of Vaccination (HH:MM)	Site of Vaccination
S179	207	Hepatitis B	Hepatitis B	1	Day 0	29/09/2010	1	11:35	Non-dominant deltoid
S179	207	Hepatitis B	Hepatitis B	2	Day 28	27/10/2010	1	12:16	Dominant deltoid
S179	207	Hepatitis B	Hepatitis B	3	Day 180	28/03/2011	1	12:33	Non-dominant deltoid
S182	208	JAIVAC-1	Dosage B	1	Day 0	29/09/2010	0.25	11:42	Non-dominant deltoid
S182	208	JAIVAC-1	Dosage B	2	Day 28	27/10/2010	0.25	10:45	Dominant deltoid
S182	208	JAIVAC-1	Dosage B	3	Day 180	28/03/2011	0.25	11:41	Non-dominant deltoid
S185	202	JAIVAC-1	Dosage B	1	Day 0	28/09/2010	0.25	10:33	Non-dominant deltoid
S185	202	JAIVAC-1	Dosage B	2	Day 28	26/10/2010	0.25	11:22	Dominant deltoid
S185	202	JAIVAC-1	Dosage B	3	Day 180	28/03/2011	0.25	10:38	Non-dominant deltoid
S186	201	JAIVAC-1	Dosage B	1	Day 0	28/09/2010	0.25	10:22	Non-dominant deltoid
S186	201	JAIVAC-1	Dosage B	2	Day 28	26/10/2010	0.25	11:05	Dominant deltoid
S186	201	JAIVAC-1	Dosage B	3	Day 180	28/03/2011	0.25	10:16	Non-dominant deltoid
S192	203	JAIVAC-1	Dosage B	1	Day 0	28/09/2010	0.25	10:41	Non-dominant deltoid
S192	203	JAIVAC-1	Dosage B	2	Day 28	26/10/2010	0.25	11:50	Dominant deltoid
S192	203	JAIVAC-1	Dosage B	3	Day 180	28/03/2011	0.25	10:24	Non-dominant deltoid
S193	215	JAIVAC-1	Dosage B	1	Day 0	30/09/2010	0.25	10:55	Non-dominant deltoid
S193	215	JAIVAC-1	Dosage B	2	Day 28	28/10/2010	0.25	11:49	Dominant deltoid
S193	215	JAIVAC-1	Dosage B	3	Day 180	29/03/2011	0.25	11:10	Non-dominant deltoid
S195	214	JAIVAC-1	Dosage B	1	Day 0	30/09/2010	0.25	10:32	Non-dominant deltoid
S195	214	JAIVAC-1	Dosage B	2	Day 28	28/10/2010	0.25	11:36	Dominant deltoid
S195	214	JAIVAC-1	Dosage B	3	Day 180	29/03/2011	0.25	10:50	Non-dominant deltoid
S202	206	JAIVAC-1	Dosage B	1	Day 0	29/09/2010	0.25	11:28	Non-dominant deltoid
S202	206	JAIVAC-1	Dosage B	2	Day 28	27/10/2010	0.25	11:07	Dominant deltoid
S202	206	JAIVAC-1	Dosage B	3	Day 180	28/03/2011	0.25	10:43	Non-dominant deltoid
S203	211	Hepatitis B	Hepatitis B	1	Day 0	30/09/2010	1	10:04	Non-dominant deltoid
S203	211	Hepatitis B	Hepatitis B	2	Day 28	28/10/2010	1	11:23	Dominant deltoid
S203	211	Hepatitis B	Hepatitis B	3	Day 180	29/03/2011	1	11:27	Non-dominant deltoid
S212	212	JAIVAC-1	Dosage B	1	Day 0	30/09/2010	0.25	10:14	Non-dominant deltoid
S212	212	JAIVAC-1	Dosage B	2	Day 28	28/10/2010	0.25	11:15	Dominant deltoid

Subject No.	Randomisation Number	Study Group	Dosage	Dose	Day	Date of Vaccination	Dose of Vaccination (ml)	Time of Vaccination (HH:MM)	Site of Vaccination
S212	212	JAIVAC-1	Dosage B	3	Day 180	29/03/2011	0.25	10:23	Non-dominant deltoid
S215	213	Hepatitis B	Hepatitis B	1	Day 0	30/09/2010	1	10:22	Non-dominant deltoid
S215	213	Hepatitis B	Hepatitis B	2	Day 28	28/10/2010	1	11:30	Dominant deltoid
S215	213	Hepatitis B	Hepatitis B	3	Day 180	29/03/2011	1	11:32	Non-dominant deltoid
S233	210	JAIVAC-1	Dosage B	1	Day 0	30/09/2010	0.25	9:54	Non-dominant deltoid
S233	210	JAIVAC-1	Dosage B	2	Day 28	28/10/2010	0.25	11:43	Dominant deltoid
S233	210	JAIVAC-1	Dosage B	3	Day 180	29/03/2011	0.25	10:01	Non-dominant deltoid
S241	303	Hepatitis B	Hepatitis B	1	Day 0	10/11/2010	1	11:05	Non-dominant deltoid
S241	303	Hepatitis B	Hepatitis B	2	Day 28	08/12/2010	1	11:25	Dominant deltoid
S241	303	Hepatitis B	Hepatitis B	3	Day 180	09/05/2011	1	11:07	Non-dominant deltoid
S250	311	JAIVAC-1	Dosage C	1	Day 0	12/11/2010	0.5	10:42	Non-dominant deltoid
S250	311	JAIVAC-1	Dosage C	2	Day 28	10/12/2010	0.5	10:40	Dominant deltoid
S250	311	JAIVAC-1	Dosage C	3	Day 180	11/05/2011	0.5	10:56	Non-dominant deltoid
S263	313	Hepatitis B	Hepatitis B	1	Day 0	12/11/2010	1	11:22	Non-dominant deltoid
S263	313	Hepatitis B	Hepatitis B	2	Day 28	10/12/2010	1	11:13	Dominant deltoid
S263	313	Hepatitis B	Hepatitis B	3	Day 180	11/05/2011	1	13:31	Non-dominant deltoid
S264	306	JAIVAC-1	Dosage C	1	Day 0	11/11/2010	0.5	11:20	Non-dominant deltoid
S264	306	JAIVAC-1	Dosage C	2	Day 28	09/12/2010	0.5	10:57	Dominant deltoid
S264	306	JAIVAC-1	Dosage C	3	Day 180	10/05/2011	0.5	11:09	Non-dominant deltoid
S266	308	JAIVAC-1	Dosage C	1	Day 0	11/11/2010	0.5	12:02	Non-dominant deltoid
S266	308	JAIVAC-1	Dosage C	2	Day 28	09/12/2010	0.5	11:23	Dominant deltoid
S266	308	JAIVAC-1	Dosage C	3	Day 180	10/05/2011	0.5	11:59	Non-dominant deltoid
S267	309	Hepatitis B	Hepatitis B	1	Day 0	11/11/2010	1	12:09	Non-dominant deltoid
S267	309	Hepatitis B	Hepatitis B	2	Day 28	09/12/2010	1	11:47	Dominant deltoid
S267	309	Hepatitis B	Hepatitis B	3	Day 180	10/05/2011	1	12:46	Non-dominant deltoid
S275	302	Hepatitis B	Hepatitis B	1	Day 0	10/11/2010	1	10:49	Non-dominant deltoid
S275	302	Hepatitis B	Hepatitis B	2	Day 28	08/12/2010	1	11:20	Dominant deltoid
S275	302	Hepatitis B	Hepatitis B	3	Day 180	09/05/2011	1	10:57	Non-dominant deltoid
S278	315	JAIVAC-1	Dosage C	1	Day 0	12/11/2010	0.5	12:15	Non-dominant deltoid

Subject No.	Randomisation Number	Study Group	Dosage	Dose	Day	Date of Vaccination	Dose of Vaccination (ml)	Time of Vaccination (HH:MM)	Site of Vaccination
S278	315	JAIVAC-1	Dosage C	2	Day 28	10/12/2010	0.5	12:18	Dominant deltoid
S278	315	JAIVAC-1	Dosage C	3	Day 180	11/05/2011	0.5	12:23	Non-dominant deltoid
S279	301	JAIVAC-1	Dosage C	1	Day 0	10/11/2010	0.5	10:37	Non-dominant deltoid
S279	301	JAIVAC-1	Dosage C	2	Day 28	08/12/2010	0.5	11:13	Dominant deltoid
S279	301	JAIVAC-1	Dosage C	3	Day 180	09/05/2011	0.5	12:02	Non-dominant deltoid
S281	304	JAIVAC-1	Dosage C	1	Day 0	11/11/2010	0.5	10:32	Non-dominant deltoid
S281	304	JAIVAC-1	Dosage C	2	Day 28	09/12/2010	0.5	10:50	Dominant deltoid
S284	305	JAIVAC-1	Dosage C	1	Day 0	11/11/2010	0.5	10:40	Non-dominant deltoid
S284	305	JAIVAC-1	Dosage C	2	Day 28	09/12/2010	0.5	11:13	Dominant deltoid
S284	305	JAIVAC-1	Dosage C	3	Day 180	10/05/2011	0.5	10:23	Non-dominant deltoid
S285	307	Hepatitis B	Hepatitis B	1	Day 0	11/11/2010	1	11:51	Non-dominant deltoid
S285	307	Hepatitis B	Hepatitis B	2	Day 28	09/12/2010	1	11:35	Dominant deltoid
S285	307	Hepatitis B	Hepatitis B	3	Day 180	10/05/2011	1	12:25	Non-dominant deltoid
S291	312	JAIVAC-1	Dosage C	1	Day 0	12/11/2010	0.5	11:12	Non-dominant deltoid
S291	312	JAIVAC-1	Dosage C	2	Day 28	10/12/2010	0.5	11:04	Dominant deltoid
S291	312	JAIVAC-1	Dosage C	3	Day 180	11/05/2011	0.5	11:37	Non-dominant deltoid
S298	314	JAIVAC-1	Dosage C	1	Day 0	12/11/2010	0.5	11:47	Non-dominant deltoid
S298	314	JAIVAC-1	Dosage C	2	Day 28	10/12/2010	0.5	11:43	Dominant deltoid
S298	314	JAIVAC-1	Dosage C	3	Day 180	11/05/2011	0.5	13:09	Non-dominant deltoid
S299	310	JAIVAC-1	Dosage C	1	Day 0	12/11/2010	0.5	10:33	Non-dominant deltoid
S299	310	JAIVAC-1	Dosage C	2	Day 28	10/12/2010	0.5	11:35	Dominant deltoid
S299	310	JAIVAC-1	Dosage C	3	Day 180	11/05/2011	0.5	10:51	Non-dominant deltoid

16.2.6 Individual efficacy response data

16.2.6.1: Listing of Immunogenicity Parameter ELISA Titers for all visits

					ELISA Titers					
**Subject No.**	**Randomisation Number**	**Study Group**	**Dosage**	**Parameter**	**Day 0**	**Day 28**	**Day 56**	**Day 180**	**Day 208**	**Day 365**
S108	115	Hepatitis B	HepatitisB	$PfMSP\text{-}1_{19}$	10	10	10	10	10	51.27
S108	115	Hepatitis B	HepatitisB	PfF2	10	10	10	10	10	50.86
S118	114	JAIVAC-1	DosageA	$PfMSP\text{-}1_{19}$	10	10	10	10	10	27.21
S118	114	JAIVAC-1	DosageA	PfF2	10	13.26	164.32	24.3	365.94	88.64
S119	107	Hepatitis B	HepatitisB	$PfMSP\text{-}1_{19}$	10	10	10	10	10	48.19
S119	107	Hepatitis B	HepatitisB	PfF2	10	10	10.01	10	10.32	50.15
S120	104	JAIVAC-1	DosageA	$PfMSP\text{-}1_{19}$	10	10	10	10	143.91	77.59
S120	104	JAIVAC-1	DosageA	PfF2	10	10	162.78	11.69	268.17	76.56
S124	106	Hepatitis B	HepatitisB	$PfMSP\text{-}1_{19}$	10	10	10	10	10	21.89
S124	106	Hepatitis B	HepatitisB	PfF2	10	10	10	10	10	21.73
S125	102	JAIVAC-1	DosageA	$PfMSP\text{-}1_{19}$	10	10	10	10	10	38.48
S125	102	JAIVAC-1	DosageA	PfF2	10	10	109.13	28.5	251.08	78.05
S130	105	JAIVAC-1	DosageA	$PfMSP\text{-}1_{19}$	10	205.18	319.07	229.98	245.8	173.59
S130	105	JAIVAC-1	DosageA	PfF2	10	10	11.28	10.43	22.97	23.2
S132	108	Hepatitis B	HepatitisB	$PfMSP\text{-}1_{19}$	10	10	10	10	10	10.23
S132	108	Hepatitis B	HepatitisB	PfF2	10	10	10	10	10	12.66
S136	109	JAIVAC-1	DosageA	$PfMSP\text{-}1_{19}$	10	10	10	10	10	14.32
S136	109	JAIVAC-1	DosageA	PfF2	10	10	10	10	33.52	28.57
S143	101	JAIVAC-1	DosageA	$PfMSP\text{-}1_{19}$	10	10	10	10	10	21.34
S143	101	JAIVAC-1	DosageA	PfF2	10	26.91	325.93	28.07	10	66.25
S144	103	Hepatitis B	HepatitisB	$PfMSP\text{-}1_{19}$	10	10	10	10	98.48	10
S144	103	Hepatitis B	HepatitisB	PfF2	10	10	10	10	375.46	17.19
S145	112	JAIVAC-1	DosageA	$PfMSP\text{-}1_{19}$	10	10	10	10	10	10
S145	112	JAIVAC-1	DosageA	PfF2	10	10	39.8	10	289.67	87.03
S148	111	JAIVAC-1	DosageA	$PfMSP\text{-}1_{19}$	10	10	10	10	10	10.25

Subject No.	Randomisation Number	Study Group	Dosage	Parameter	ELISA Titers Day 0	Day 28	Day 56	Day 180	Day 208	Day 365
S148	111	JAIVAC-1	DosageA	PfF2	10	10	92.39	10	66.4	18.61
S154	113	JAIVAC-1	DosageA	PfMSP-1_{19}	10	10	10	10	10	27.98
S154	113	JAIVAC-1	DosageA	PfF2	10	10	55.72	16.69	74.39	40.39
S156	110	JAIVAC-1	DosageA	PfMSP-1_{19}	10	10	10	10	10	15.54
S156	110	JAIVAC-1	DosageA	PfF2	10	10	42.72	10	111.21	31.93
S170	204	Hepatitis B	HepatitisB	PfMSP-1_{19}	10	10	10	10	10	11.75
S170	204	Hepatitis B	HepatitisB	PfF2	10	10	10	10	10	10
S175	209	JAIVAC-1	DosageB	PfMSP-1_{19}	10	10	10	10	35.46	20.04
S175	209	JAIVAC-1	DosageB	PfF2	10	10	181.25	27.17	218.1	57.48
S176	205	Hepatitis B	HepatitisB	PfMSP-1_{19}	10	10	10	10	10	29.64
S176	205	Hepatitis B	HepatitisB	PfF2	10	10	10	10	10	12.03
S179	207	Hepatitis B	HepatitisB	PfMSP-1_{19}	10	10	10	10	10	10
S179	207	Hepatitis B	HepatitisB	PfF2	13.88	15.65	14.56	11.52	12.6	10
S182	208	JAIVAC-1	DosageB	PfMSP-1_{19}	10	10	10	10	10	23.2
S182	208	JAIVAC-1	DosageB	PfF2	10	10	22.85	15.03	40.53	46.58
S185	202	JAIVAC-1	DosageB	PfMSP-1_{19}	10	10	23.14	10	40.61	10.39
S185	202	JAIVAC-1	DosageB	PfF2	10	17.82	162.01	11.66	268.4	99.59
S186	201	JAIVAC-1	DosageB	PfMSP-1_{19}	10	10	10	10	10	32.39
S186	201	JAIVAC-1	DosageB	PfF2	10	10	31.5	11.99	186.17	111.22
S192	203	JAIVAC-1	DosageB	PfMSP-1_{19}	10	10	10	10	10	10
S192	203	JAIVAC-1	DosageB	PfF2	10	10	16.65	10	108.25	58.96
S193	215	JAIVAC-1	DosageB	PfMSP-1_{19}	10	10	10	10	67.62	23.73
S193	215	JAIVAC-1	DosageB	PfF2	10	10	83.49	10.05	302.5	56.69
S195	214	JAIVAC-1	DosageB	PfMSP-1_{19}	10	10	23.76	10	112.85	16.34
S195	214	JAIVAC-1	DosageB	PfF2	10	10	180.32	27.58	331.79	60.87
S202	206	JAIVAC-1	DosageB	PfMSP-1_{19}	10	10	10.87	10	41.84	15.01
S202	206	JAIVAC-1	DosageB	PfF2	11.44	21.64	425.21	45.02	720.61	187.35
S203	211	Hepatitis B	HepatitisB	PfMSP-1_{19}	10	10	10	10	10	24.85
S203	211	Hepatitis B	HepatitisB	PfF2	10	10	10	10	10	24.78

Subject No.	Randomisation Number	Study Group	Dosage	Parameter	ELISA Titers Day 0	Day 28	Day 56	Day 180	Day 208	Day 365
S212	212	JAIVAC-1	DosageB	PfMSP-1_{19}	10	10	10	10	10	10
S212	212	JAIVAC-1	DosageB	PfF2	13.86	21.79	206.38	23.78	445.09	72.45
S215	213	Hepatitis B	HepatitisB	PfMSP-1_{19}	10	10	10	10	10	10
S215	213	Hepatitis B	HepatitisB	PfF2	10	10	10	10	10	10
S233	210	JAIVAC-1	DosageB	PfMSP-1_{19}	10	10	10	10	16.85	12.62
S233	210	JAIVAC-1	DosageB	PfF2	10	10	409.95	37.99	728.59	155.42
S241	303	Hepatitis B	HepatitisB	PfMSP-1_{19}	10	10	10	10	10	10
S241	303	Hepatitis B	HepatitisB	PfF2	10	10	10	10	10	10
S250	311	JAIVAC-1	DosageC	PfMSP-1_{19}	10	44.45	50.72	11.23	39.86	15.25
S250	311	JAIVAC-1	DosageC	PfF2	10	45.43	51.84	11.48	40.74	53.46
S263	313	Hepatitis B	HepatitisB	PfMSP-1_{19}	10	10	10	10	10	10
S263	313	Hepatitis B	HepatitisB	PfF2	10	10	10	10	10	10
S264	306	JAIVAC-1	DosageC	PfMSP-1_{19}	10	10	10	10	157.92	13.25
S264	306	JAIVAC-1	DosageC	PfF2	11.78	129.18	2111.67	273.4	4654.97	594.7
S266	308	JAIVAC-1	DosageC	PfMSP-1_{19}	10	10	10	10	16.67	10
S266	308	JAIVAC-1	DosageC	PfF2	10	28.62	580.72	14.45	544.3	50.96
S267	309	Hepatitis B	HepatitisB	PfMSP-1_{19}	10	10	10	10	10	10
S267	309	Hepatitis B	HepatitisB	PfF2	10	10	10	10	10	17.82
S275	302	Hepatitis B	HepatitisB	PfMSP-1_{19}	10	10	10	10	10	10
S275	302	Hepatitis B	HepatitisB	PfF2	10	10	10	10	10.27	10
S278	315	JAIVAC-1	DosageC	PfMSP-1_{19}	10	10	55.61	10	45.42	10.19
S278	315	JAIVAC-1	DosageC	PfF2	13.13	72.06	2307.38	168.89	1989.58	419.74
S279	301	JAIVAC-1	DosageC	PfMSP-1_{19}	10	10	10	10	10	10
S279	301	JAIVAC-1	DosageC	PfF2	19.57	67.17	910.8	62.27	596.51	78.09
S281	304	JAIVAC-1	DosageC	PfMSP-1_{19}	10	10	10	.	.	.
S281	304	JAIVAC-1	DosageC	PfF2	11.06	38.74	524.66	.	.	.
S284	305	JAIVAC-1	DosageC	PfMSP-1_{19}	10	10	10	10	10	10
S284	305	JAIVAC-1	DosageC	PfF2	10	63.13	1052.11	107.56	1141.99	188.64
S285	307	Hepatitis B	HepatitisB	PfMSP-1_{19}	10	10	10	10	10	10

Subject No.	Randomisation Number	Study Group	Dosage	Parameter	ELISA Titers Day 0	Day 28	Day 56	Day 180	Day 208	Day 365
S285	307	Hepatitis B	HepatitisB	PfF2	10	10	10	10	10	10
S291	312	JAIVAC-1	DosageC	PfMSP-1_{19}	10	10	13.26	10	112.57	10
S291	312	JAIVAC-1	DosageC	PfF2	10	240.26	629.67	106.96	1044.49	111.96
S298	314	JAIVAC-1	DosageC	PfMSP-1_{19}	10	10	10	11.86	52.27	10
S298	314	JAIVAC-1	DosageC	PfF2	23.19	119.14	900.49	115.57	979.95	148.39
S299	310	JAIVAC-1	DosageC	PfMSP-1_{19}	10	10	10	10	16.34	10
S299	310	JAIVAC-1	DosageC	PfF2	10	31.06	255.01	42.45	317.73	73.16

Note:
An Early termination visit has been performed for Subject S281 and the ELISA Titers for PfMSP-1_{19} is 10 and for PfF2 is 86.

16.2.6.2: Listing of Immunogenicity Parameter by IFA for all Visits

Subject No.	Randomisation Number	Study Group	Dosage	Parameter	Day 0	Day 28	Day 56	Day 180	Day 208	Day 365
S108	115	Hepatitis B	HepatitisB	Native Proteins Detected	No	No	No	No	No	Yes
S108	115	Hepatitis B	HepatitisB	IFA Titers	0	0	0	0	0	100
S118	114	JAIVAC-1	DosageA	Native Proteins Detected	No	No	Yes	Yes	Yes	Yes
S118	114	JAIVAC-1	DosageA	IFA Titers	0	0	200	100	1000	200
S119	107	Hepatitis B	HepatitisB	Native Proteins Detected	No	No	No	No	No	Yes
S119	107	Hepatitis B	HepatitisB	IFA Titers	0	0	0	0	0	100
S120	104	JAIVAC-1	DosageA	Native Proteins Detected	No	No	Yes	No	Yes	Yes
S120	104	JAIVAC-1	DosageA	IFA Titers	0	0	200	0	800	100
S124	106	Hepatitis B	HepatitisB	Native Proteins Detected	No	No	No	No	No	No
S124	106	Hepatitis B	HepatitisB	IFA Titers	0	0	0	0	0	0
S125	102	JAIVAC-1	DosageA	Native Proteins Detected	No	No	Yes	Yes	Yes	Yes
S125	102	JAIVAC-1	DosageA	IFA Titers	0	0	400	50	400	100
S130	105	JAIVAC-1	DosageA	Native Proteins Detected	No	Yes	Yes	Yes	Yes	Yes
S130	105	JAIVAC-1	DosageA	IFA Titers	0	400	400	400	400	400
S132	108	Hepatitis B	HepatitisB	Native Proteins Detected	No	No	No	No	No	No
S132	108	Hepatitis B	HepatitisB	IFA Titers	0	0	0	0	0	0
S136	109	JAIVAC-1	DosageA	Native Proteins Detected	No	No	No	No	Yes	Yes
S136	109	JAIVAC-1	DosageA	IFA Titers	0	0	0	0	50	5
S143	101	JAIVAC-1	DosageA	Native Proteins Detected	No	Yes	Yes	Yes	No	Yes
S143	101	JAIVAC-1	DosageA	IFA Titers	0	50	400	50	0	100
S144	103	Hepatitis B	HepatitisB	Native Proteins Detected	No	No	No	No	Yes	No
S144	103	Hepatitis B	HepatitisB	IFA Titers	0	0	0	0	1000	0
S145	112	JAIVAC-1	DosageA	Native Proteins Detected	No	No	Yes	No	Yes	Yes
S145	112	JAIVAC-1	DosageA	IFA Titers	0	0	50	0	800	50
S148	111	JAIVAC-1	DosageA	Native Proteins Detected	No	No	Yes	No	Yes	No
S148	111	JAIVAC-1	DosageA	IFA Titers	0	0	100	0	100	0
S154	113	JAIVAC-1	DosageA	Native Proteins Detected	No	No	Yes	No	Yes	Yes
S154	113	JAIVAC-1	DosageA	IFA Titers	0	0	50	0	100	25

Subject No.	Randomisation Number	Study Group	Dosage	Parameter	Day 0	Day 28	Day 56	Day 180	Day 208	Day 365
S156	110	JAIVAC-1	DosageA	Native Proteins Detected	No	No	Yes	No	Yes	Yes
S156	110	JAIVAC-1	DosageA	IFA Titers	0	0	50	0	100	50
S170	204	Hepatitis B	HepatitisB	Native Proteins Detected	No	No	No	No	No	Yes
S170	204	Hepatitis B	HepatitisB	IFA Titers	0	0	0	0	0	0
S175	209	JAIVAC-1	DosageB	Native Proteins Detected	No	No	Yes	Yes	Yes	Yes
S175	209	JAIVAC-1	DosageB	IFA Titers	0	0	100	50	200	50
S176	205	Hepatitis B	HepatitisB	Native Proteins Detected	No	No	No	No	No	No
S176	205	Hepatitis B	HepatitisB	IFA Titers	0	0	0	0	0	0
S179	207	Hepatitis B	HepatitisB	Native Proteins Detected	No	No	No	No	No	No
S179	207	Hepatitis B	HepatitisB	IFA Titers	0	0	0	0	0	0
S182	208	JAIVAC-1	DosageB	Native Proteins Detected	No	No	Yes	No	Yes	Yes
S182	208	JAIVAC-1	DosageB	IFA Titers	0	0	25	0	25	50
S185	202	JAIVAC-1	DosageB	Native Proteins Detected	No	No	Yes	No	Yes	Yes
S185	202	JAIVAC-1	DosageB	IFA Titers	0	0	100	0	200	100
S186	201	JAIVAC-1	DosageB	Native Proteins Detected	No	No	Yes	No	Yes	Yes
S186	201	JAIVAC-1	DosageB	IFA Titers	0	0	50	0	100	200
S192	203	JAIVAC-1	DosageB	Native Proteins Detected	No	No	Yes	Yes	Yes	Yes
S192	203	JAIVAC-1	DosageB	IFA Titers	0	0	25	25	200	50
S193	215	JAIVAC-1	DosageB	Native Proteins Detected	No	No	Yes	Yes	Yes	Yes
S193	215	JAIVAC-1	DosageB	IFA Titers	0	0	200	50	400	50
S195	214	JAIVAC-1	DosageB	Native Proteins Detected	No	No	Yes	Yes	Yes	Yes
S195	214	JAIVAC-1	DosageB	IFA Titers	0	0	200	50	400	50
S202	206	JAIVAC-1	DosageB	Native Proteins Detected	No	Yes	Yes	Yes	Yes	Yes
S202	206	JAIVAC-1	DosageB	IFA Titers	0	50	400	50	400	200
S203	211	Hepatitis B	HepatitisB	Native Proteins Detected	No	No	No	No	No	No
S203	211	Hepatitis B	HepatitisB	IFA Titers	0	0	0	0	0	0
S212	212	JAIVAC-1	DosageB	Native Proteins Detected	No	Yes	Yes	Yes	Yes	Yes
S212	212	JAIVAC-1	DosageB	IFA Titers	0	50	400	50	400	50
S215	213	Hepatitis B	HepatitisB	Native Proteins Detected	No	No	No	No	No	No
S215	213	Hepatitis B	HepatitisB	IFA Titers	0	0	0	0	0	0

Subject No.	Randomisation Number	Study Group	Dosage	Parameter	Day 0	Day 28	Day 56	Day 180	Day 208	Day 365
S233	210	JAIVAC-1	DosageB	Native Proteins Detected	No	Yes	Yes	Yes	Yes	Yes
S233	210	JAIVAC-1	DosageB	IFA Titers	0	25	400	100	800	200
S241	303	Hepatitis B	HepatitisB	Native Proteins Detected	No	No	No	No	No	No
S241	303	Hepatitis B	HepatitisB	IFA Titers	0	0	0	0	0	0
S250	311	JAIVAC-1	DosageC	Native Proteins Detected	No	Yes	Yes	Yes	Yes	Yes
S250	311	JAIVAC-1	DosageC	IFA Titers	0	50	100	5	50	100
S263	313	Hepatitis B	HepatitisB	Native Proteins Detected	No	No	No	No	No	No
S263	313	Hepatitis B	HepatitisB	IFA Titers	0	0	0	0	0	0
S264	306	JAIVAC-1	DosageC	Native Proteins Detected	No	Yes	Yes	Yes	Yes	Yes
S264	306	JAIVAC-1	DosageC	IFA Titers	0	100	2000	400	8000	800
S266	308	JAIVAC-1	DosageC	Native Proteins Detected	No	Yes	Yes	No	Yes	Yes
S266	308	JAIVAC-1	DosageC	IFA Titers	0	25	800	0	800	50
S267	309	Hepatitis B	HepatitisB	Native Proteins Detected	No	No	No	No	No	No
S267	309	Hepatitis B	HepatitisB	IFA Titers	0	0	0	0	0	0
S275	302	Hepatitis B	HepatitisB	Native Proteins Detected	No	No	No	No	No	No
S275	302	Hepatitis B	HepatitisB	IFA Titers	0	0	0	0	0	0
S278	315	JAIVAC-1	DosageC	Native Proteins Detected	No	Yes	Yes	Yes	Yes	Yes
S278	315	JAIVAC-1	DosageC	IFA Titers	0	100	4000	200	4000	400
S279	301	JAIVAC-1	DosageC	Native Proteins Detected	No	Yes	Yes	Yes	Yes	Yes
S279	301	JAIVAC-1	DosageC	IFA Titers	0	100	800	100	400	50
S281	304	JAIVAC-1	DosageC	Native Proteins Detected	No	Yes	Yes	.	.	.
S281	304	JAIVAC-1	DosageC	IFA Titers	0	25	400	.	.	.
S284	305	JAIVAC-1	DosageC	Native Proteins Detected	No	Yes	Yes	Yes	Yes	Yes
S284	305	JAIVAC-1	DosageC	IFA Titers	0	50	1000	100	1000	100
S285	307	Hepatitis B	HepatitisB	Native Proteins Detected	No	No	No	No	No	No
S285	307	Hepatitis B	HepatitisB	IFA Titers	0	0	0	0	0	0
S291	312	JAIVAC-1	DosageC	Native Proteins Detected	No	Yes	Yes	Yes	Yes	Yes
S291	312	JAIVAC-1	DosageC	IFA Titers	0	400	2000	200	4000	50
S298	314	JAIVAC-1	DosageC	Native Proteins Detected	No	Yes	Yes	Yes	Yes	Yes
S298	314	JAIVAC-1	DosageC	IFA Titers	0	100	1000	100	1000	100

Subject No.	Randomisation Number	Study Group	Dosage	Parameter	Day 0	Day 28	Day 56	Day 180	Day 208	Day 365
S299	310	JAIVAC-1	DosageC	Native Proteins Detected	No	Yes	Yes	Yes	Yes	Yes
S299	310	JAIVAC-1	DosageC	IFA Titers	0	100	1000	100	1000	50

Note:
An Early termination visit has been performed for Subject S281 and the IFA Titer is 50.

16.2.6.3.a: Listing of Immunogenicity Parameter IgG Subclasses against PfMSP-1_{19} by ELISA for all Visits

		JAIVAC-1		Relative Abundance of IgG Subclass as measured by Absorbance at 492 nm (OD_{492})					
**Subject No.**	**Randomisation Number**	**Dosage**	**IgG Subclasses**	**Day 0**	**Day 28**	**Day 56**	**Day 180**	**Day 208**	**Day 365**
.	.	---	---	.	.	.	.	.	.

Note:
Data is not available for IgG Subclasses against PfMSP-119 by ELISA hence blank table is presented

16.2.6.3.b: Listing of Immunogenicity Parameter IgG Subclasses against PfF2 by ELISA for all Visits

		JAIVAC-1		Relative Abundance of IgG Subclass as measured by Absorbance at 492 nm (OD_{492})					
Subject No.	Randomisation Number	Dosage	IgG Subclasses	Day 0	Day 28	Day 56	Day 180	Day 208	Day 365
S175	209	Dosage B	IgG1	0.09	0.09	0.55	0.09	0.53	0.1
S175	209	Dosage B	IgG2	0.15	0.15	0.17	0.17	0.18	0.16
S175	209	Dosage B	IgG3	0.1	0.11	0.27	0.1	0.16	0.16
S175	209	Dosage B	IgG4	0.11	0.09	0.13	0.13	2.18	0.59
S182	208	Dosage B	IgG1	0.09	0.07	0.1	0.08	0.12	0.08
S182	208	Dosage B	IgG2	0.16	0.14	0.18	0.19	0.16	0.14
S182	208	Dosage B	IgG3	0.09	0.1	0.24	0.1	0.1	0.09
S182	208	Dosage B	IgG4	0.09	0.08	0.09	0.09	0.17	0.11
S185	202	Dosage B	IgG1	0.08	0.09	0.51	0.08	0.77	0.14
S185	202	Dosage B	IgG2	0.11	0.11	0.13	0.12	0.14	0.12
S185	202	Dosage B	IgG3	0.08	0.11	0.21	0.1	0.11	0.09
S185	202	Dosage B	IgG4	0.09	0.08	0.09	0.09	0.69	0.32
S186	201	Dosage B	IgG1	0.09	0.09	0.13	0.08	0.52	0.15
S186	201	Dosage B	IgG2	0.1	0.11	0.14	0.1	0.12	0.1
S186	201	Dosage B	IgG3	0.09	0.09	0.27	0.11	0.39	0.18
S186	201	Dosage B	IgG4	0.15	0.07	0.14	0.16	0.29	0.13
S192	203	Dosage B	IgG1	0.13	0.12	0.19	0.16	0.48	0.19
S192	203	Dosage B	IgG2	0.15	0.12	0.16	0.18	0.19	0.17
S192	203	Dosage B	IgG3	0.11	0.1	0.23	0.12	0.17	0.12
S192	203	Dosage B	IgG4	0.08	0.26	0.09	0.1	0.17	0.1
S193	215	Dosage B	IgG1	0.08	0.08	0.19	0.09	0.91	0.12
S193	215	Dosage B	IgG2	0.13	0.13	0.14	0.14	0.17	0.15
S193	215	Dosage B	IgG3	0.08	0.09	0.18	0.1	0.19	0.11
S193	215	Dosage B	IgG4	0.09	0.08	0.08	0.09	0.12	0.09
S195	214	Dosage B	IgG1	0.09	0.08	0.58	0.1	1.15	0.13

		JAIVAC-1		Relative Abundance of IgG Subclass as measured by Absorbance at 492 nm (OD_{492})					
**Subject No.**	**Randomisation Number**	**Dosage**	**IgG Subclasses**	**Day 0**	**Day 28**	**Day 56**	**Day 180**	**Day 208**	**Day 365**
S195	214	Dosage B	IgG2	0.12	0.12	0.15	0.14	0.17	0.14
S195	214	Dosage B	IgG3	0.13	0.13	0.33	0.15	0.19	0.14
S195	214	Dosage B	IgG4	0.09	0.08	0.1	0.11	0.48	0.13
S202	206	Dosage B	IgG1	0.16	0.19	1.04	0.17	2.26	0.38
S202	206	Dosage B	IgG2	0.11	0.1	0.1	0.12	0.12	0.1
S202	206	Dosage B	IgG3	0.11	0.18	2.79	0.4	0.95	0.35
S202	206	Dosage B	IgG4	0.09	0.07	0.07	0.08	0.23	0.1
S212	212	Dosage B	IgG1	0.06	0.06	0.75	0.06	1.98	0.11
S212	212	Dosage B	IgG2	0.13	0.1	0.14	0.11	0.12	0.11
S212	212	Dosage B	IgG3	0.09	0.08	0.27	0.09	0.25	0.09
S212	212	Dosage B	IgG4	0.08	0.06	0.07	0.07	0.08	0.08
S233	210	Dosage B	IgG1	0.06	0.06	2.43	0.14	2.89	0.27
S233	210	Dosage B	IgG2	0.11	0.08	0.09	0.09	0.11	0.09
S233	210	Dosage B	IgG3	0.08	0.08	0.6	0.1	0.54	0.1
S233	210	Dosage B	IgG4	0.07	0.06	0.07	0.06	0.12	0.07
S250	311	Dosage C	IgG1	0.06	0.18	0.62	0.12	1.02	0.14
S250	311	Dosage C	IgG2	0.11	0.08	0.11	0.1	0.12	0.1
S250	311	Dosage C	IgG3	0.11	0.21	1.02	0.15	0.26	0.13
S250	311	Dosage C	IgG4	0.07	0.08	0.11	0.08	0.21	0.11
S264	306	Dosage C	IgG1	0.11	0.56	3.91	1.55	3.96	3.58
S264	306	Dosage C	IgG2	0.09	0.09	0.3	0.1	1.42	0.14
S264	306	Dosage C	IgG3	0.1	0.6	3.31	0.43	2.52	0.42
S264	306	Dosage C	IgG4	0.18	0.2	0.47	0.31	3.78	2.11
S266	308	Dosage C	IgG1	0.15	0.18	3.26	0.14	3.31	0.18
S266	308	Dosage C	IgG2	0.09	0.08	0.1	0.08	0.11	0.09
S266	308	Dosage C	IgG3	0.08	0.12	0.54	0.09	0.26	0.09

		JAIVAC-1		Relative Abundance of IgG Subclass as measured by Absorbance at 492 nm (OD_{492})					
**Subject No.**	**Randomisation Number**	**Dosage**	**IgG Subclasses**	**Day 0**	**Day 28**	**Day 56**	**Day 180**	**Day 208**	**Day 365**
S266	308	Dosage C	IgG4	0.1	0.1	0.14	0.11	2.8	0.24
S278	315	Dosage C	IgG1	0.33	1.45	3.82	0.77	3.84	3.15
S278	315	Dosage C	IgG2	0.1	0.09	0.12	0.1	0.12	0.1
S278	315	Dosage C	IgG3	0.1	0.65	2.3	0.13	0.67	0.14
S278	315	Dosage C	IgG4	0.09	0.1	1.27	0.13	3.41	0.76
S279	301	Dosage C	IgG1	0.12	0.26	3.55	0.21	3.08	0.29
S279	301	Dosage C	IgG2	0.11	0.11	0.19	0.11	0.14	0.12
S279	301	Dosage C	IgG3	0.12	0.23	2.57	0.19	1.29	0.2
S279	301	Dosage C	IgG4	0.09	0.08	0.13	0.1	1.17	0.18
S281	304	Dosage C	IgG1	0.06	0.09	2.51	.	.	.
S281	304	Dosage C	IgG2	0.09	0.09	0.12	.	.	.
S281	304	Dosage C	IgG3	0.12	0.17	1.46	.	.	.
S281	304	Dosage C	IgG4	0.07	0.07	0.12	.	.	.
S284	305	Dosage C	IgG1	0.06	0.14	3.55	0.24	3.72	0.76
S284	305	Dosage C	IgG2	0.12	0.11	0.17	0.12	0.15	0.12
S284	305	Dosage C	IgG3	0.08	0.27	2.88	0.13	0.72	0.14
S284	305	Dosage C	IgG4	0.07	0.07	0.14	0.08	0.55	0.17
S291	312	Dosage C	IgG1	0.07	0.13	3.27	0.24	3.76	0.34
S291	312	Dosage C	IgG2	0.1	0.09	0.12	0.1	0.12	0.1
S291	312	Dosage C	IgG3	0.07	0.13	0.66	0.09	0.24	0.08
S291	312	Dosage C	IgG4	0.06	0.05	0.07	0.08	1.03	0.18
S298	314	Dosage C	IgG1	0.07	0.18	3.29	0.13	3.62	0.5
S298	314	Dosage C	IgG2	0.11	0.11	0.19	0.11	0.14	0.12
S298	314	Dosage C	IgG3	0.07	0.17	1.44	0.09	0.27	0.09
S298	314	Dosage C	IgG4	0.06	0.07	0.09	0.07	1.8	0.35
S299	310	Dosage C	IgG1	0.07	0.08	0.77	0.08	1.11	0.14

		JAIVAC-1		Relative Abundance of IgG Subclass as measured by Absorbance at 492 nm (OD_{492})					
**Subject No.**	**Randomisation Number**	**Dosage**	**IgG Subclasses**	**Day 0**	**Day 28**	**Day 56**	**Day 180**	**Day 208**	**Day 365**
S299	310	Dosage C	IgG2	0.1	0.09	0.12	0.09	0.12	0.11
S299	310	Dosage C	IgG3	0.07	0.11	0.67	0.09	0.18	0.08
S299	310	Dosage C	IgG4	0.06	0.06	0.07	0.07	0.62	0.26

Note:
An Early termination visit has been performed for Subject S281 and the result for IgG1, IgG2, IgG3 and IgG4 is 0.15, 0.1, 0.26, 0.09 respectively.

16.2.6.4: Listing of Immunogenicity Parameter Percent Inhibition by Growth Inhibition Assay (GIA) for all Visits

		JAIVAC-1		Percent Inhibition by Growth Inhibition Assay (GIA)			
Subject No.	Randomisation Number	Dosage	Parasite Strain	Day 0	Day 56	Day 208	Day 365
S241	303	Hepatitis B	P. falciparum CAMP	20.2	.	22.07	.
S241	303	Hepatitis B	P. falciparum 3D7	1.2	.	6.81	.
S241	303	Hepatitis B	P. falciparum FVO	.	.	.	.
S250	311	Dosage C	P. falciparum CAMP	11.78	.	34.89	.
S250	311	Dosage C	P. falciparum 3D7	2.07	.	22.53	.
S250	311	Dosage C	P. falciparum FVO	.	.	.	.
S264	306	Dosage C	P. falciparum CAMP	27.59	.	67.83	.
S264	306	Dosage C	P. falciparum 3D7	18.01	.	32.43	.
S264	306	Dosage C	P. falciparum FVO	.	.	.	.
S266	308	Dosage C	P. falciparum CAMP	24.83	.	67.09	.
S266	308	Dosage C	P. falciparum 3D7	20.16	.	13.47	.
S266	308	Dosage C	P. falciparum FVO	.	.	.	.
S275	302	Hepatitis B	P. falciparum CAMP	6.83	.	22.96	.
S275	302	Hepatitis B	P. falciparum 3D7	0.54	.	0.29	.
S275	302	Hepatitis B	P. falciparum FVO	.	.	.	.
S278	315	Dosage C	P. falciparum CAMP	22.68	.	61.13	.
S278	315	Dosage C	P. falciparum 3D7	4.76	.	26.08	.
S278	315	Dosage C	P. falciparum FVO	.	.	.	.
S279	301	Dosage C	P. falciparum CAMP	10.42	.	21.08	.
S279	301	Dosage C	P. falciparum 3D7	14.66	.	20.34	.
S279	301	Dosage C	P. falciparum FVO	.	.	.	.
S281	304	Dosage C	P. falciparum CAMP	21.5	.	.	.
S281	304	Dosage C	P. falciparum 3D7	-11.7	.	.	.
S281	304	Dosage C	P. falciparum FVO	.	.	.	.
S284	305	Dosage C	P. falciparum CAMP	20.54	.	52.95	.

		JAIVAC-1		Percent Inhibition by Growth Inhibition Assay (GIA)			
**Subject No.**	**Randomisation Number**	**Dosage**	**Parasite Strain**	**Day 0**	**Day 56**	**Day 208**	**Day 365**
S284	305	Dosage C	P. falciparum 3D7	17.49	.	18.53	.
S284	305	Dosage C	P. falciparum FVO	.	.	.	.
S291	312	Dosage C	P. falciparum CAMP	17.39	.	37.42	.
S291	312	Dosage C	P. falciparum 3D7	10.96	.	6.23	.
S291	312	Dosage C	P. falciparum FVO	.	.	.	.
S298	314	Dosage C	P. falciparum CAMP	12.74	.	37.41	.
S298	314	Dosage C	P. falciparum 3D7	13.85	.	31	.
S298	314	Dosage C	P. falciparum FVO	.	.	.	.
S299	310	Dosage C	P. falciparum CAMP	6.22	.	36.04	.
S299	310	Dosage C	P. falciparum 3D7	10.11	.	16.07	.
S299	310	Dosage C	P. falciparum FVO	.	.	.	.

Note:
An Early termination visit has been performed for Subject S281 and the Percentage inhibition for P. falciparum CAMP and P. falciparum 3D7 is 13.4 and -8.7 respectively.

16.2.7 Adverse Events Listings

16.2.7.1: Unsolicited Adverse Events Listings (each Subject) (I of II)

Subject No.	Randomisation Number	Study Group	Dosage	Dose	Reported Term	Lower Level Term	Preferred Term	Primary SOC	*Preferred Term Modified for Analysis	Serious	Seriousness Criteria	Start Date	Stop Date	Onset Day	Duration Days
S108	115	Hepatitis B	Hepatitis B	3	Iron Deficiency Anaemia	Iron Deficiency Anaemia	Iron Deficiency Anaemia	Blood And Lymphatic System Disorders	Iron Deficiency Anaemia	No	.	23/02/11	21/05/11	1	88
S120	104	JAIVAC-1	Dosage A	3	Raised Bilirubin Levels(Total & Direct)	Bilirubin Elevated	Blood Bilirubin Increased	Investigations	Blood Bilirubin Increased	No	.	22/03/11	11/04/11	29	21
S124	106	Hepatitis B	Hepatitis B	2	Hypochromic Microcytic Anaemia	Hypochromic Microcytic Anaemia	Hypochromic Anaemia	Blood And Lymphatic System Disorders	Hypochromic Anaemia	No	.	21/10/10	07/12/10	29	48
S130	105	JAIVAC-1	Dosage A	2	Cold	Cold	Nasopharyngitis	Infections And Infestations	Nasopharyngitis	No	.	27/09/10	29/09/10	5	3
S136	109	JAIVAC-1	Dosage A	3	Coryza	Coryza	Rhinitis	Infections And Infestations	Rhinitis	No	.	06/03/11	07/03/11	13	2
S144	103	Hepatitis B	Hepatitis B	1	Upper Respiratory Tract Infection	Upper Respiratory Tract Infection	Upper Respiratory Tract Infection	Infections And Infestations	Upper Respiratory Tract Infection	No	.	17/09/10	20/09/10	25	4
S148	111	JAIVAC-1	Dosage A	2	Cold	Cold	Nasopharyngitis	Infections And Infestations	Nasopharyngitis	No	.	09/10/10	10/10/10	16	2
S148	111	JAIVAC-1	Dosage A	2	Conjunctivitis	Conjunctivitis	Conjunctivitis	Eye Disorders	Conjunctivitis	No	.	08/10/10	15/10/10	15	8
S148	111	JAIVAC-1	Dosage A	2	Gastroenteritis	Gastroenteritis	Gastroenteritis	Infections And Infestations	Gastroenteritis	No	.	26/09/10	27/09/10	3	2

Subject No.	Randomisation Number	Study Group	Dosage	Dose	Reported Term	Lower Level Term	Preferred Term	Primary SOC	*Preferred Term Modified for Analysis	Serious	Seriousness Criteria	Start Date	Stop Date	Onset Day	Duration Days
S148	111	JAIVAC-1	Dosage A	1	Soft Tissue Injury To Left Lower Forearm	Soft Tissue Injury	Soft Tissue Injury	Injury, Poisoning And Procedural Complications	Soft Tissue Injury	No	.	07/09/10	14/09/10	12	8
S148	111	JAIVAC-1	Dosage A	1	Upper Respiratory Tract Infection	Upper Respiratory Tract Infection	Upper Respiratory Tract Infection	Infections And Infestations	Upper Respiratory Tract Infection	No	.	28/08/10	03/09/10	2	7
S175	209	JAIVAC-1	Dosage B	1	Upper Respiratory Tract Infection	Upper Respiratory Tract Infection	Upper Respiratory Tract Infection	Infections And Infestations	Upper Respiratory Tract Infection	No	.	21/10/10	25/10/10	23	5
S182	208	JAIVAC-1	Dosage B	1	Coryza	Coryza	Rhinitis	Infections And Infestations	Rhinitis	No	.	13/10/10	15/10/10	15	3
S182	208	JAIVAC-1	Dosage B	2	Left Eye Conjunctivitis	Conjunctivitis	Conjunctivitis	Eye Disorders	Conjunctivitis	No	.	03/11/10	08/11/10	8	6
S182	208	JAIVAC-1	Dosage B	1	Numbness Of Right Hand	Numbness In Hand	Hypoaesthesia	Nervous System Disorders	Hypoaesthesia	No	.	04/10/10	04/10/10	6	1
S186	201	JAIVAC-1	Dosage B	2	Back Pain	Back Pain	Back Pain	Musculoskeletal And Connective Tissue Disorders	Back Pain	No	.	30/10/10	09/11/10	5	11
S193	215	JAIVAC-1	Dosage B	3	Raised Eosinophil Count	Eosinophil Count Increased	Eosinophil Count Increased	Investigations	Eosinophil Count Increased	No	.	26/04/11	28/06/11	29	64
S203	211	Hepatitis B	Hepatitis B	2	Hypochromic Microcytic Anaemia	Hypochromic Microcytic Anaemia	Hypochromic Anaemia	Blood And Lymphatic System Disorders	Hypochromic Anaemia	No	.	25/11/10	23/03/11	29	119

Subject No.	Randomisation Number	Study Group	Dosage	Dose	Reported Term	Lower Level Term	Preferred Term	Primary SOC	*Preferred Term Modified for Analysis	Serious	Seriousness Criteria	Start Date	Stop Date	Onset Day	Duration Days
S203	211	Hepatitis B	Hepatitis B	3	Scorpion Sting	Scorpion Sting	Arthropod Sting	Injury, Poisoning And Procedural Complications	Arthropod Sting	No	.	25/04/11	26/04/11	28	2
S212	212	JAIVAC-1	Dosage B	1	Upper Respiratory Tract Infection	Upper Respiratory Tract Infection	Upper Respiratory Tract Infection	Infections And Infestations	Upper Respiratory Tract Infection	No	.	08/10/10	26/10/10	9	19
S263	313	Hepatitis B	Hepatitis B	2	Raised Potassium Level	Potassium Increased	Blood Potassium Increased	Investigations	Blood Potassium Increased	No	.	07/01/11	14/02/11	29	39
S264	306	JAIVAC-1	Dosage C	3	Raised Blood Sugar	Blood Sugar Increased	Blood Glucose Increased	Investigations	Blood Glucose Increased	No	.	07/06/11	15/07/11	29	39
S264	306	JAIVAC-1	Dosage C	3	Raised Liver Enzymes(Sgot, Sgpt & Ggt)	Raised Liver Enzymes	Hepatic Enzyme Increased	Investigations	Hepatic Enzyme Increased	No	.	07/06/11	.	29	.
S266	308	JAIVAC-1	Dosage C	2	Raised Potassium Level	Potassium Increased	Blood Potassium Increased	Investigations	Blood Potassium Increased	No	.	06/01/11	24/03/11	29	78
S267	309	Hepatitis B	Hepatitis B	3	Raised Serum Creatinine Level	Serum Creatinine Increased	Blood Creatinine Increased	Investigations	Blood Creatinine Increased	No	.	07/06/11	24/10/11	29	140
S267	309	Hepatitis B	Hepatitis B	2	Raised Serum Creatinine Levels	Serum Creatinine Increased	Blood Creatinine Increased	Investigations	Blood Creatinine Increased	No	.	06/01/11	27/01/11	29	22
S278	315	JAIVAC-1	Dosage C	2	Feeling Feverish	Fever	Pyrexia	General Disorders And Administration Site Conditions	Pyrexia	No	.	10/12/10	13/12/10	1	4

Subject No.	Randomisation Number	Study Group	Dosage	Dose	Reported Term	Lower Level Term	Preferred Term	Primary SOC	*Preferred Term Modified for Analysis	Serious	Seriousness Criteria	Start Date	Stop Date	Onset Day	Duration Days
S279	301	JAIVAC-1	Dosage C	3	Coryza	Coryza	Rhinitis	Infections And Infestations	Rhinitis	No	.	13/05/11	14/05/11	5	2
S279	301	JAIVAC-1	Dosage C	3	Soft Tissue Injury(Face)	Soft Tissue Injury	Soft Tissue Injury	Injury, Poisoning And Procedural Complications	Soft Tissue Injury	No	.	22/05/11	31/05/11	14	10
S279	301	JAIVAC-1	Dosage C	2	Upper Respiratory Tract Infection	Upper Respiratory Tract Infection	Upper Respiratory Tract Infection	Infections And Infestations	Upper Respiratory Tract Infection	No	.	13/12/10	16/12/10	6	4
S281	304	JAIVAC-1	Dosage C	2	Hypochromic Microcytic Anaemia	Hypochromic Microcytic Anaemia	Hypochromic Anaemia	Blood And Lymphatic System Disorders	Hypochromic Anaemia	No	.	06/01/11	.	29	.
S285	307	Hepatitis B	Hepatitis B	1	Left Eye Conjunctivitis	Conjunctivitis	Conjunctivitis	Eye Disorders	Conjunctivitis	No	.	18/11/10	20/11/10	8	3

Note:
1) The version of the MedDRA browser used is version 13.0
2) Seriousness Criteria: 1: Results In Death, 2-Is Life Threatening, 3-Requires Subject Hospitalization OR Prolongation of Existing Hospitalization, 4-Result in Persistent or Significant Disability/ Incapacity, 5-Congenital Anomaly/ Birth Defect, 6-Other Important Medical Event
3) Duration Days = Stop Date – Start Date + 1
4) Onset Day = Start Date – Vaccination Date + 1
5) *Adverse Events for which Preferred Term is not available were replaced by Low Level Term (if available), else by verbatim name as entered in the CRF.

16.2.7.1: Unsolicited Adverse Events Listings (each Subject) (II of II)

Subject No.	Randomisation Number	Study Group	Dosage	Dose	Reported Term	Intensity	Relationship	Outcome	Any Medication Given?
S108	115	Hepatitis B	Hepatitis B	3	Iron Deficiency Anaemia	1	2	1	Yes
S120	104	JAIVAC-1	Dosage A	3	Raised Bilirubin Levels(Total & Direct)	1	2	1	No
S124	106	Hepatitis B	Hepatitis B	2	Hypochromic Microcytic Anaemia	1	2	1	Yes
S130	105	JAIVAC-1	Dosage A	2	Cold	1	1	1	No
S136	109	JAIVAC-1	Dosage A	3	Coryza	1	1	1	Yes
S144	103	Hepatitis B	Hepatitis B	1	Upper Respiratory Tract Infection	1	2	1	Yes
S148	111	JAIVAC-1	Dosage A	2	Cold	1	2	1	Yes
S148	111	JAIVAC-1	Dosage A	2	Conjunctivitis	1	2	1	Yes
S148	111	JAIVAC-1	Dosage A	2	Gastroenteritis	1	1	1	Yes
S148	111	JAIVAC-1	Dosage A	1	Soft Tissue Injury To Left Lower Forearm	1	2	1	Yes
S148	111	JAIVAC-1	Dosage A	1	Upper Respiratory Tract Infection	1	1	1	Yes
S175	209	JAIVAC-1	Dosage B	1	Upper Respiratory Tract Infection	1	2	1	Yes
S182	208	JAIVAC-1	Dosage B	1	Coryza	1	2	1	No
S182	208	JAIVAC-1	Dosage B	2	Left Eye Conjunctivitis	1	2	1	Yes
S182	208	JAIVAC-1	Dosage B	1	Numbness Of Right Hand	1	2	1	No
S186	201	JAIVAC-1	Dosage B	2	Back Pain	1	1	1	Yes
S193	215	JAIVAC-1	Dosage B	3	Raised Eosinophil Count	1	2	1	Yes
S203	211	Hepatitis B	Hepatitis B	2	Hypochromic Microcytic Anaemia	1	2	1	Yes
S203	211	Hepatitis B	Hepatitis B	3	Scorpion Sting	2	2	1	Yes
S212	212	JAIVAC-1	Dosage B	1	Upper Respiratory Tract Infection	2	2	1	Yes
S263	313	Hepatitis B	Hepatitis B	2	Raised Potassium Level	1	2	1	No
S264	306	JAIVAC-1	Dosage C	3	Raised Blood Sugar	1	2	1	No
S264	306	JAIVAC-1	Dosage C	3	Raised Liver Enzymes(Sgot, Sgpt & Ggt)	1	2	3	No
S266	308	JAIVAC-1	Dosage C	2	Raised Potassium Level	1	2	1	No
S267	309	Hepatitis B	Hepatitis B	3	Raised Serum Creatinine Level	1	2	1	No
S267	309	Hepatitis B	Hepatitis B	2	Raised Serum Creatinine Levels	1	2	1	No

Subject No.	Randomisation Number	Study Group	Dosage	Dose	Reported Term	Intensity	Relationship	Outcome	Any Medication Given?
S278	315	JAIVAC-1	Dosage C	2	Feeling Feverish	1	1	1	Yes
S279	301	JAIVAC-1	Dosage C	3	Coryza	1	1	1	Yes
S279	301	JAIVAC-1	Dosage C	3	Soft Tissue Injury(Face)	2	2	1	Yes
S279	301	JAIVAC-1	Dosage C	2	Upper Respiratory Tract Infection	1	1	1	Yes
S281	304	JAIVAC-1	Dosage C	2	Hypochromic Microcytic Anaemia	1	2	5	Yes
S285	307	Hepatitis B	Hepatitis B	1	Left Eye Conjunctivitis	2	2	1	Yes

Note:
1) The version of the MedDRA browser used is version 13.0
2) Intensity: 1- Sign or symptom present, but easily tolerated, 2- Discomforting enough to interfere with normal activities, 3- Disabling, incapacitating with inability to perform usual activity
3) Relationship: 1-Related, 2-Not Related
4) Outcome: 1- Resolved, 2- Resolved with sequelae, 3- Ongoing, 4- Died, 5- Lost to follow up

16.2.7.2: Serious Adverse Events Listings (each Subject) (I of II)

Subject No.	Randomisation Number	Study Group	Dosage	Dose	Reported Term	Primary SOC	Preferred Term	Lower Level Term	*Preferred Term Modified for Analysis	Serious	Seriousness Criteria	Start Date	Stop Date	Duration Days	Onset Day
**	.	.	.	.	.	.	.	.	.	.	.	.	.	.	.

Note:
1) The version of the MedDRA browser used is version 13.0
2) Seriousness Criteria: 1: Death, 2-Life Threatening, 3-In-Subject Hospitalization OR Prolongation of Existing Hospitalization, 4-Perisistent or Significant Disability/ Incapacity, 5-Congenital Anomaly/ Birth Defect, 6-Other
3) Duration Days = Stop Date – Start Date + 1
4) Onset Day = Start Date – Vaccination Date + 1
5) *Adverse Events for which Preferred Term is not available were replaced by Low Level Term (if available), else by verbatim name as entered in the CRF.
6) **No subject experienced serious adverse event

16.2.7.2: Serious Adverse Events Listings (each Subject) (II of II)

Subject No.	Randomisation Number	Study Group	Dosage	Dose	Reported Term	Intensity	Relationship	Outcome	Any Medication Given?	Did SAE abate after study vaccine discontinuation ?
**	.	.	.	.	.	.	.	.	.	.

Note:
1) The version of the MedDRA browser used is version 13.0
2) Intensity: 1- Sign or symptom present, but easily tolerated, 2- Discomforting enough to interfere with normal activities, 3- Disabling, incapacitating with inability to perform usual activity
3) Relationship: 1-Related, 2-Not Related
4) Outcome: 1- Death, 2- SAE persisted/ongoing, 3- SAE resolved, 4- Unknown, 5- Other
5) **No subject experienced serious adverse event

16.2.8 Listings of Individual Laboratory Measurements by Subject

16.2.8.1: Listing of Clinical Laboratory Normal Reference Ranges

Laboratory Name	Type of Laboratory Test	Laboratory Test	Unit	Gender	Day	Normal Reference Range
DiagnoSearch Central Laboratory						
	Haematology	Haemoglobin	gm / dl	Male	All	13.5 - 18.0
		Haemoglobin	gm / dl	Male	Screening/Baseline	13.0 – 18.0
		Haematocrit	%		All	40.0 - 54.0
		MCV	fL		All	80.0 - 96.0
		MCH	pg		All	27.0 - 31.0
		MCHC	gm%		All	32.0 - 36.0
		RBC count	10E6/ul		All	4.60 - 6.20
		RBC count	10E6/ul		Screening/Baseline	4.0 - 7.0
		TLC (WBC count)	10E3/ul		All	4.50 - 11.00
		TLC (WBC count)	10E3/ul		Screening/Baseline	4.0 - 11.0
		% Neutrophils	%		All	40.0 - 75.0
		% Basophils	%		All	0.0 - 1.0

	% Lymphocytes	%	All	20.0 - 45.0
	% Monocytes	%	All	2.0 - 10.0
	% Eosinophils	%	All	1.0 - 6.0
	% Eosinophils	%	Screening/Baseline	0.0 - 10.0
	Platelet Count	10E3/ul	All	150 - 400
	Platelet Count	10E3/ul	Screening/Baseline	150 - 500
Serum Chemistry	Random blood glucose	mg /dl	All	45 - 130
	Sodium	mEq / L	All	3.5 - 5.1
	Potassium	mEq / L	All	136 - 145
	Serum creatinine	mg / dl	All	0.7 - 1.2
	Serum creatinine	mg / dl	Screening/Baseline	0.0 – 1.2
	AST	U / L	All	0 - 40
	ALT	U / L	All	0 - 41
	Direct Bilirubin	U / L	All	0.00 - 0.30
	Direct Bilirubin	U / L	Screening/Baseline	0.00 - 0.40
	Indirect Bilirubin	U / L	All	0.00 - 0.99

		Indirect Bilirubin	U / L	Screening/Baseline	0.00 – 1.2
		Total Bilirubin	U / L	All	0.00 - 0.99
		Total Bilirubin	U / L	Screening/Baseline	0.00 - 1.2
		Alkaline Phosphatase	U / L	All	40 - 129
		Gamma Glutaryl Transferase	U / L	All	10 - 71
		Gamma Glutaryl Transferase	U / L	Screening/Baseline	0 - 71
		Serum Ferritin	ng/ml	All	28 - 397
		Serum Iron	micrograms/dl	All	49 - 181
		TIBC	micrograms/dl	All	250 - 450
	Serology	Anti HCV	None	All	Negative
		HbsAg	None	All	Negative
		Anti-HIV	None	All	Negative

16.2.8.2: Listing of Clinical Laboratory Evaluations – Haematology

Subject No.	Randomisation Number	Study Group	Dosage	Dose	Haematology Parameters	Unit	Day	Date of sample Collection	Results	Out of Range	Reference Range	Flag	Clinical Significance **
S108	115	Hepatitis B	Hepatitis B		% Basophils	%	Day -14 to Day -1	18/08/2010	0.5	.	0.0 - 1.0		
S108	115	Hepatitis B	Hepatitis B		% Eosinophils	%	Day -14 to Day -1	18/08/2010	6.5	.	1.0 - 6.0	H	
S108	115	Hepatitis B	Hepatitis B		% Lymphocytes	%	Day -14 to Day -1	18/08/2010	29.5	.	20.0 - 45.0		
S108	115	Hepatitis B	Hepatitis B		% Monocytes	%	Day -14 to Day -1	18/08/2010	5.1	.	2.0 - 10.0		
S108	115	Hepatitis B	Hepatitis B		% Neutrophils	%	Day -14 to Day -1	18/08/2010	58.4	.	40.0 - 75.0		
S108	115	Hepatitis B	Hepatitis B		Haematocrit	%	Day -14 to Day -1	18/08/2010	46	.	40.0 - 54.0		
S108	115	Hepatitis B	Hepatitis B		Haemoglobin	gm / dl	Day -14 to Day -1	18/08/2010	14.6	.	13.5 - 18.0		
S108	115	Hepatitis B	Hepatitis B		MCH	pg	Day -14 to Day -1	18/08/2010	28.3	.	27.0 - 31.0		
S108	115	Hepatitis B	Hepatitis B		MCHC	gm %	Day -14 to Day -1	18/08/2010	31.6	Yes	32.0 - 36.0	L	NCS
S108	115	Hepatitis B	Hepatitis B		MCV	fL	Day -14 to Day -1	18/08/2010	89.5	.	80.0- 96.0		
S108	115	Hepatitis B	Hepatitis B		Platelet Count	10E3/ul	Day -14 to Day -1	18/08/2010	254	.	150 - 400		
S108	115	Hepatitis B	Hepatitis B		RBC count	10E6/ul	Day -14 to Day -1	18/08/2010	5.14	.	4.60 - 6.20		
S108	115	Hepatitis B	Hepatitis B		TLC	10E3/ul	Day -14 to Day -1	18/08/2010	6.7	.	4.50 - 11.00		
S108	115	Hepatitis B	Hepatitis B	2	% Basophils	%	Day 28	24/09/2010	1.2	Yes	0.0 - 1.0	H	NCS
S108	115	Hepatitis B	Hepatitis B	2	% Eosinophils	%	Day 28	24/09/2010	9.2	Yes	1.0 - 6.0	H	NCS
S108	115	Hepatitis B	Hepatitis B	2	% Lymphocytes	%	Day 28	24/09/2010	31.2	.	20.0 - 45.0		
S108	115	Hepatitis B	Hepatitis B	2	% Monocytes	%	Day 28	24/09/2010	4.5	.	2.0 - 10.0		
S108	115	Hepatitis B	Hepatitis B	2	% Neutrophils	%	Day 28	24/09/2010	53.9	.	40.0 - 75.0		
S108	115	Hepatitis B	Hepatitis B	2	Haematocrit	%	Day 28	24/09/2010	45.1	.	40.0 - 54.0		
S108	115	Hepatitis B	Hepatitis B	2	Haemoglobin	gm / dl	Day 28	24/09/2010	14.3	.	13.5 - 18.0		
S108	115	Hepatitis B	Hepatitis B	2	MCH	pg	Day 28	24/09/2010	28.4	.	27.0 - 31.0		
S108	115	Hepatitis B	Hepatitis B	2	MCHC	gm %	Day 28	24/09/2010	31.7	Yes	32.0 - 36.0	L	NCS
S108	115	Hepatitis B	Hepatitis B	2	MCV	fL	Day 28	24/09/2010	89.5	.	80.0- 96.0		
S108	115	Hepatitis B	Hepatitis B	2	Platelet Count	10E3/ul	Day 28	24/09/2010	259	.	150 - 400		
S108	115	Hepatitis B	Hepatitis B	2	RBC count	10E6/ul	Day 28	24/09/2010	5.04	.	4.60 - 6.20		
S108	115	Hepatitis B	Hepatitis B	2	TLC	10E3/ul	Day 28	24/09/2010	5.6	.	4.50 - 11.00		
S108	115	Hepatitis B	Hepatitis B	2	% Basophils	%	Day 56	22/10/2010	0.5	.	0.0 - 1.0		

Subject No.	Randomisation Number	Study Group	Dosage	Dose	Haematology Parameters	Unit	Day	Date of sample Collection	Results	Out of Range	Reference Range	Flag	Clinical Significance **
S108	115	Hepatitis B	Hepatitis B	2	% Eosinophils	%	Day 56	22/10/2010	7.8	Yes	1.0 - 6.0	H	NCS
S108	115	Hepatitis B	Hepatitis B	2	% Lymphocytes	%	Day 56	22/10/2010	27	.	20.0 - 45.0		
S108	115	Hepatitis B	Hepatitis B	2	% Monocytes	%	Day 56	22/10/2010	5.4	.	2.0 - 10.0		
S108	115	Hepatitis B	Hepatitis B	2	% Neutrophils	%	Day 56	22/10/2010	59.2	.	40.0 - 75.0		
S108	115	Hepatitis B	Hepatitis B	2	Haematocrit	%	Day 56	22/10/2010	44.1	.	40.0 - 54.0		
S108	115	Hepatitis B	Hepatitis B	2	Haemoglobin	gm / dl	Day 56	22/10/2010	14.6	.	13.5 - 18.0		
S108	115	Hepatitis B	Hepatitis B	2	MCH	pg	Day 56	22/10/2010	29.3	.	27.0 - 31.0		
S108	115	Hepatitis B	Hepatitis B	2	MCHC	gm %	Day 56	22/10/2010	33.2	.	32.0 - 36.0		
S108	115	Hepatitis B	Hepatitis B	2	MCV	fL	Day 56	22/10/2010	88.4	.	80.0- 96.0		
S108	115	Hepatitis B	Hepatitis B	2	Platelet Count	10E3/ul	Day 56	22/10/2010	335	.	150 - 400		
S108	115	Hepatitis B	Hepatitis B	2	RBC count	10E6/ul	Day 56	22/10/2010	4.99	.	4.60 - 6.20		
S108	115	Hepatitis B	Hepatitis B	2	TLC	10E3/ul	Day 56	22/10/2010	5.6	.	4.50 - 11.00		
S108	115	Hepatitis B	Hepatitis B	3	% Basophils	%	Day 180	23/02/2011	1	.	0.0 - 1.0		
S108	115	Hepatitis B	Hepatitis B	3	% Eosinophils	%	Day 180	23/02/2011	6.3	Yes	1.0 - 6.0	H	NCS
S108	115	Hepatitis B	Hepatitis B	3	% Lymphocytes	%	Day 180	23/02/2011	32.3	.	20.0 - 45.0		
S108	115	Hepatitis B	Hepatitis B	3	% Monocytes	%	Day 180	23/02/2011	4.9	.	2.0 - 10.0		
S108	115	Hepatitis B	Hepatitis B	3	% Neutrophils	%	Day 180	23/02/2011	55.5	.	40.0 - 75.0		
S108	115	Hepatitis B	Hepatitis B	3	Haematocrit	%	Day 180	23/02/2011	39.3	Yes	40.0 - 54.0	L	CS
S108	115	Hepatitis B	Hepatitis B	3	Haemoglobin*	gm / dl	Day 180	23/02/2011	12.5	Yes	13.5 - 18.0	L	CS
S108	115	Hepatitis B	Hepatitis B	3	MCH	pg	Day 180	23/02/2011	26.9	Yes	27.0 - 31.0	L	CS
S108	115	Hepatitis B	Hepatitis B	3	MCHC	gm %	Day 180	23/02/2011	31.8	Yes	32.0 - 36.0	L	CS
S108	115	Hepatitis B	Hepatitis B	3	MCV	fL	Day 180	23/02/2011	84.6	.	80.0- 96.0		
S108	115	Hepatitis B	Hepatitis B	3	Platelet Count	10E3/ul	Day 180	23/02/2011	324	.	150 - 400		
S108	115	Hepatitis B	Hepatitis B	3	RBC count	10E6/ul	Day 180	23/02/2011	4.64	.	4.60 - 6.20		
S108	115	Hepatitis B	Hepatitis B	3	TLC	10E3/ul	Day 180	23/02/2011	4.88	.	4.50 - 11.00		
S108	115	Hepatitis B	Hepatitis B	3	% Basophils	%	Day 208	23/03/2011	0.6	.	0.0 - 1.0		
S108	115	Hepatitis B	Hepatitis B	3	% Eosinophils	%	Day 208	23/03/2011	2.6	.	1.0 - 6.0		
S108	115	Hepatitis B	Hepatitis B	3	% Lymphocytes	%	Day 208	23/03/2011	16.7	Yes	20.0 - 45.0	L	NCS
S108	115	Hepatitis B	Hepatitis B	3	% Monocytes	%	Day 208	23/03/2011	6.9	.	2.0 - 10.0		

Subject No.	Randomisation Number	Study Group	Dosage	Dose	Haematology Parameters	Unit	Day	Date of sample Collection	Results	Out of Range	Reference Range	Flag	Clinical Significance **
S108	115	Hepatitis B	Hepatitis B	3	% Neutrophils	%	Day 208	23/03/2011	73.1	.	40.0 - 75.0		
S108	115	Hepatitis B	Hepatitis B	3	Haematocrit	%	Day 208	23/03/2011	38.8	Yes	40.0 - 54.0	L	CS
S108	115	Hepatitis B	Hepatitis B	3	Haemoglobin*	gm / dl	Day 208	23/03/2011	12.7	Yes	13.5 - 18.0	L	CS
S108	115	Hepatitis B	Hepatitis B	3	MCH	pg	Day 208	23/03/2011	27.1	.	27.0 - 31.0		
S108	115	Hepatitis B	Hepatitis B	3	MCHC	gm %	Day 208	23/03/2011	32.6	.	32.0 - 36.0		
S108	115	Hepatitis B	Hepatitis B	3	MCV	fL	Day 208	23/03/2011	82.9	.	80.0- 96.0		
S108	115	Hepatitis B	Hepatitis B	3	Platelet Count	10E3/ul	Day 208	23/03/2011	318	.	150 - 400		
S108	115	Hepatitis B	Hepatitis B	3	RBC count	10E6/ul	Day 208	23/03/2011	4.68	.	4.60 - 6.20		
S108	115	Hepatitis B	Hepatitis B	3	TLC	10E3/ul	Day 208	23/03/2011	7.69	.	4.50 - 11.00		
S108	115	Hepatitis B	Hepatitis B	3	% Basophils	%	Day 365	26/08/2011	0.8	.	0.0 - 1.0		
S108	115	Hepatitis B	Hepatitis B	3	% Eosinophils	%	Day 365	26/08/2011	7.1	Yes	1.0 - 6.0	H	NCS
S108	115	Hepatitis B	Hepatitis B	3	% Lymphocytes	%	Day 365	26/08/2011	26.6	.	20.0 - 45.0		
S108	115	Hepatitis B	Hepatitis B	3	% Monocytes	%	Day 365	26/08/2011	5	.	2.0 - 10.0		
S108	115	Hepatitis B	Hepatitis B	3	% Neutrophils	%	Day 365	26/08/2011	60.4	.	40.0 - 75.0		
S108	115	Hepatitis B	Hepatitis B	3	Haematocrit	%	Day 365	26/08/2011	42.3	.	40.0 - 54.0		
S108	115	Hepatitis B	Hepatitis B	3	Haemoglobin	gm / dl	Day 365	26/08/2011	14	.	13.5 - 18.0		
S108	115	Hepatitis B	Hepatitis B	3	MCH	pg	Day 365	26/08/2011	28.8	.	27.0 - 31.0		
S108	115	Hepatitis B	Hepatitis B	3	MCHC	gm %	Day 365	26/08/2011	33.2	.	32.0 - 36.0		
S108	115	Hepatitis B	Hepatitis B	3	MCV	fL	Day 365	26/08/2011	86.7	.	80.0- 96.0		
S108	115	Hepatitis B	Hepatitis B	3	Platelet Count	10E3/ul	Day 365	26/08/2011	289	.	150 - 400		
S108	115	Hepatitis B	Hepatitis B	3	RBC count	10E6/ul	Day 365	26/08/2011	4.88	.	4.60 - 6.20		
S108	115	Hepatitis B	Hepatitis B	3	TLC	10E3/ul	Day 365	26/08/2011	7.01	.	4.50 - 11.00		
S118	114	JAIVAC-1	Dosage A		% Basophils	%	Day -14 to Day -1	17/08/2010	0.5	.	0.0 - 1.0		
S118	114	JAIVAC-1	Dosage A		% Eosinophils	%	Day -14 to Day -1	17/08/2010	6.5	.	1.0 - 6.0	H	
S118	114	JAIVAC-1	Dosage A		% Lymphocytes	%	Day -14 to Day -1	17/08/2010	35.4	.	20.0 - 45.0		
S118	114	JAIVAC-1	Dosage A		% Monocytes	%	Day -14 to Day -1	17/08/2010	4.9	.	2.0 - 10.0		
S118	114	JAIVAC-1	Dosage A		% Neutrophils	%	Day -14 to Day -1	17/08/2010	52.6	.	40.0 - 75.0		
S118	114	JAIVAC-1	Dosage A		Haematocrit	%	Day -14 to Day -1	17/08/2010	47.6	.	40.0 - 54.0		
S118	114	JAIVAC-1	Dosage A		Haemoglobin	gm / dl	Day -14 to Day -1	17/08/2010	15.4	.	13.5 - 18.0		

Subject No.	Randomisation Number	Study Group	Dosage	Dose	Haematology Parameters	Unit	Day	Date of sample Collection	Results	Out of Range	Reference Range	Flag	Clinical Significance **
S118	114	JAIVAC-1	Dosage A		MCH	pg	Day -14 to Day -1	17/08/2010	28	.	27.0 - 31.0		
S118	114	JAIVAC-1	Dosage A		MCHC	gm %	Day -14 to Day -1	17/08/2010	32.5	.	32.0 - 36.0		
S118	114	JAIVAC-1	Dosage A		MCV	fL	Day -14 to Day -1	17/08/2010	86.3	.	80.0- 96.0		
S118	114	JAIVAC-1	Dosage A		Platelet Count	10E3/ul	Day -14 to Day -1	17/08/2010	201	.	150 - 400		
S118	114	JAIVAC-1	Dosage A		RBC count	10E6/ul	Day -14 to Day -1	17/08/2010	5.51	.	4.60 - 6.20		
S118	114	JAIVAC-1	Dosage A		TLC	10E3/ul	Day -14 to Day -1	17/08/2010	6.68	.	4.50 - 11.00		
S118	114	JAIVAC-1	Dosage A	2	% Basophils	%	Day 28	24/09/2010	1.3	Yes	0.0 - 1.0	H	NCS
S118	114	JAIVAC-1	Dosage A	2	% Eosinophils	%	Day 28	24/09/2010	4.4	.	1.0 - 6.0		
S118	114	JAIVAC-1	Dosage A	2	% Lymphocytes	%	Day 28	24/09/2010	32.2	.	20.0 - 45.0		
S118	114	JAIVAC-1	Dosage A	2	% Monocytes	%	Day 28	24/09/2010	6.7	.	2.0 - 10.0		
S118	114	JAIVAC-1	Dosage A	2	% Neutrophils	%	Day 28	24/09/2010	55.4	.	40.0 - 75.0		
S118	114	JAIVAC-1	Dosage A	2	Haematocrit	%	Day 28	24/09/2010	47.9	.	40.0 - 54.0		
S118	114	JAIVAC-1	Dosage A	2	Haemoglobin	gm / dl	Day 28	24/09/2010	15.3	.	13.5 - 18.0		
S118	114	JAIVAC-1	Dosage A	2	MCH	pg	Day 28	24/09/2010	27.6	.	27.0 - 31.0		
S118	114	JAIVAC-1	Dosage A	2	MCHC	gm %	Day 28	24/09/2010	31.9	Yes	32.0 - 36.0	L	NCS
S118	114	JAIVAC-1	Dosage A	2	MCV	fL	Day 28	24/09/2010	86.6	.	80.0- 96.0		
S118	114	JAIVAC-1	Dosage A	2	Platelet Count	10E3/ul	Day 28	24/09/2010	235	.	150 - 400		
S118	114	JAIVAC-1	Dosage A	2	RBC count	10E6/ul	Day 28	24/09/2010	5.53	.	4.60 - 6.20		
S118	114	JAIVAC-1	Dosage A	2	TLC	10E3/ul	Day 28	24/09/2010	6.99	.	4.50 - 11.00		
S118	114	JAIVAC-1	Dosage A	2	% Basophils	%	Day 56	22/10/2010	0.5	.	0.0 - 1.0		
S118	114	JAIVAC-1	Dosage A	2	% Eosinophils	%	Day 56	22/10/2010	6.5	Yes	1.0 - 6.0	H	NCS
S118	114	JAIVAC-1	Dosage A	2	% Lymphocytes	%	Day 56	22/10/2010	31.3	.	20.0 - 45.0		
S118	114	JAIVAC-1	Dosage A	2	% Monocytes	%	Day 56	22/10/2010	5.9	.	2.0 - 10.0		
S118	114	JAIVAC-1	Dosage A	2	% Neutrophils	%	Day 56	22/10/2010	55.9	.	40.0 - 75.0		
S118	114	JAIVAC-1	Dosage A	2	Haematocrit	%	Day 56	22/10/2010	44	.	40.0 - 54.0		
S118	114	JAIVAC-1	Dosage A	2	Haemoglobin	gm / dl	Day 56	22/10/2010	15	.	13.5 - 18.0		
S118	114	JAIVAC-1	Dosage A	2	MCH	pg	Day 56	22/10/2010	28.5	.	27.0 - 31.0		
S118	114	JAIVAC-1	Dosage A	2	MCHC	gm %	Day 56	22/10/2010	34	.	32.0 - 36.0		
S118	114	JAIVAC-1	Dosage A	2	MCV	fL	Day 56	22/10/2010	83.9	.	80.0- 96.0		

Subject No.	Randomisation Number	Study Group	Dosage	Dose	Haematology Parameters	Unit	Day	Date of sample Collection	Results	Out of Range	Reference Range	Flag	Clinical Significance **
S118	114	JAIVAC-1	Dosage A	2	Platelet Count	10E3/ul	Day 56	22/10/2010	224	.	150 - 400		
S118	114	JAIVAC-1	Dosage A	2	RBC count	10E6/ul	Day 56	22/10/2010	5.25	.	4.60 - 6.20		
S118	114	JAIVAC-1	Dosage A	2	TLC	10E3/ul	Day 56	22/10/2010	6.15	.	4.50 - 11.00		
S118	114	JAIVAC-1	Dosage A	3	% Basophils	%	Day 180	23/02/2011	0.6	.	0.0 - 1.0		
S118	114	JAIVAC-1	Dosage A	3	% Eosinophils	%	Day 180	23/02/2011	4.1	.	1.0 - 6.0		
S118	114	JAIVAC-1	Dosage A	3	% Lymphocytes	%	Day 180	23/02/2011	27.4	.	20.0 - 45.0		
S118	114	JAIVAC-1	Dosage A	3	% Monocytes	%	Day 180	23/02/2011	5	.	2.0 - 10.0		
S118	114	JAIVAC-1	Dosage A	3	% Neutrophils	%	Day 180	23/02/2011	63	.	40.0 - 75.0		
S118	114	JAIVAC-1	Dosage A	3	Haematocrit	%	Day 180	23/02/2011	46	.	40.0 - 54.0		
S118	114	JAIVAC-1	Dosage A	3	Haemoglobin	gm / dl	Day 180	23/02/2011	15.5	.	13.5 - 18.0		
S118	114	JAIVAC-1	Dosage A	3	MCH	pg	Day 180	23/02/2011	27.6	.	27.0 - 31.0		
S118	114	JAIVAC-1	Dosage A	3	MCHC	gm %	Day 180	23/02/2011	33.8	.	32.0 - 36.0		
S118	114	JAIVAC-1	Dosage A	3	MCV	fL	Day 180	23/02/2011	81.6	.	80.0- 96.0		
S118	114	JAIVAC-1	Dosage A	3	Platelet Count	10E3/ul	Day 180	23/02/2011	239	.	150 - 400		
S118	114	JAIVAC-1	Dosage A	3	RBC count	10E6/ul	Day 180	23/02/2011	5.63	.	4.60 - 6.20		
S118	114	JAIVAC-1	Dosage A	3	TLC	10E3/ul	Day 180	23/02/2011	8.93	.	4.50 - 11.00		
S118	114	JAIVAC-1	Dosage A	3	% Basophils	%	Day 208	23/03/2011	0.7	.	0.0 - 1.0		
S118	114	JAIVAC-1	Dosage A	3	% Eosinophils	%	Day 208	23/03/2011	4.3	.	1.0 - 6.0		
S118	114	JAIVAC-1	Dosage A	3	% Lymphocytes	%	Day 208	23/03/2011	28.2	.	20.0 - 45.0		
S118	114	JAIVAC-1	Dosage A	3	% Monocytes	%	Day 208	23/03/2011	5.5	.	2.0 - 10.0		
S118	114	JAIVAC-1	Dosage A	3	% Neutrophils	%	Day 208	23/03/2011	61.3	.	40.0 - 75.0		
S118	114	JAIVAC-1	Dosage A	3	Haematocrit	%	Day 208	23/03/2011	45.3	.	40.0 - 54.0		
S118	114	JAIVAC-1	Dosage A	3	Haemoglobin	gm / dl	Day 208	23/03/2011	15.3	.	13.5 - 18.0		
S118	114	JAIVAC-1	Dosage A	3	MCH	pg	Day 208	23/03/2011	27.7	.	27.0 - 31.0		
S118	114	JAIVAC-1	Dosage A	3	MCHC	gm %	Day 208	23/03/2011	33.8	.	32.0 - 36.0		
S118	114	JAIVAC-1	Dosage A	3	MCV	fL	Day 208	23/03/2011	81.9	.	80.0- 96.0		
S118	114	JAIVAC-1	Dosage A	3	Platelet Count	10E3/ul	Day 208	23/03/2011	223	.	150 - 400		
S118	114	JAIVAC-1	Dosage A	3	RBC count	10E6/ul	Day 208	23/03/2011	5.53	.	4.60 - 6.20		
S118	114	JAIVAC-1	Dosage A	3	TLC	10E3/ul	Day 208	23/03/2011	5.79	.	4.50 - 11.00		

Subject No.	Randomisation Number	Study Group	Dosage	Dose	Haematology Parameters	Unit	Day	Date of sample Collection	Results	Out of Range	Reference Range	Flag	Clinical Significance **
S118	114	JAIVAC-1	Dosage A	3	% Basophils	%	Day 365	26/08/2011	1.2	Yes	0.0 - 1.0	H	NCS
S118	114	JAIVAC-1	Dosage A	3	% Eosinophils	%	Day 365	26/08/2011	6.1	Yes	1.0 - 6.0	H	NCS
S118	114	JAIVAC-1	Dosage A	3	% Lymphocytes	%	Day 365	26/08/2011	32.2	.	20.0 - 45.0		
S118	114	JAIVAC-1	Dosage A	3	% Monocytes	%	Day 365	26/08/2011	7.9	.	2.0 - 10.0		
S118	114	JAIVAC-1	Dosage A	3	% Neutrophils	%	Day 365	26/08/2011	52.6	.	40.0 - 75.0		
S118	114	JAIVAC-1	Dosage A	3	Haematocrit	%	Day 365	26/08/2011	46.6	.	40.0 - 54.0		
S118	114	JAIVAC-1	Dosage A	3	Haemoglobin	gm / dl	Day 365	26/08/2011	15.6	.	13.5 - 18.0		
S118	114	JAIVAC-1	Dosage A	3	MCH	pg	Day 365	26/08/2011	27.7	.	27.0 - 31.0		
S118	114	JAIVAC-1	Dosage A	3	MCHC	gm %	Day 365	26/08/2011	33.4	.	32.0 - 36.0		
S118	114	JAIVAC-1	Dosage A	3	MCV	fL	Day 365	26/08/2011	83	.	80.0- 96.0		
S118	114	JAIVAC-1	Dosage A	3	Platelet Count	10E3/ul	Day 365	26/08/2011	206	.	150 - 400		
S118	114	JAIVAC-1	Dosage A	3	RBC count	10E6/ul	Day 365	26/08/2011	5.61	.	4.60 - 6.20		
S118	114	JAIVAC-1	Dosage A	3	TLC	10E3/ul	Day 365	26/08/2011	6.11	.	4.50 - 11.00		
S119	107	Hepatitis B	Hepatitis B		% Basophils	%	Day -14 to Day -1	18/08/2010	0.9	.	0.0 - 1.0		
S119	107	Hepatitis B	Hepatitis B		% Eosinophils	%	Day -14 to Day -1	18/08/2010	4.4	.	1.0 - 6.0		
S119	107	Hepatitis B	Hepatitis B		% Lymphocytes	%	Day -14 to Day -1	18/08/2010	25.8	.	20.0 - 45.0		
S119	107	Hepatitis B	Hepatitis B		% Monocytes	%	Day -14 to Day -1	18/08/2010	5.7	.	2.0 - 10.0		
S119	107	Hepatitis B	Hepatitis B		% Neutrophils	%	Day -14 to Day -1	18/08/2010	63.2	.	40.0 - 75.0		
S119	107	Hepatitis B	Hepatitis B		Haematocrit	%	Day -14 to Day -1	18/08/2010	50	.	40.0 - 54.0		
S119	107	Hepatitis B	Hepatitis B		Haemoglobin	gm / dl	Day -14 to Day -1	18/08/2010	16	.	13.5 - 18.0		
S119	107	Hepatitis B	Hepatitis B		MCH	pg	Day -14 to Day -1	18/08/2010	27.5	.	27.0 - 31.0		
S119	107	Hepatitis B	Hepatitis B		MCHC	gm %	Day -14 to Day -1	18/08/2010	32	.	32.0 - 36.0		
S119	107	Hepatitis B	Hepatitis B		MCV	fL	Day -14 to Day -1	18/08/2010	85.9	.	80.0- 96.0		
S119	107	Hepatitis B	Hepatitis B		Platelet Count	10E3/ul	Day -14 to Day -1	18/08/2010	221	.	150 - 400		
S119	107	Hepatitis B	Hepatitis B		RBC count	10E6/ul	Day -14 to Day -1	18/08/2010	5.83	.	4.60 - 6.20		
S119	107	Hepatitis B	Hepatitis B		TLC	10E3/ul	Day -14 to Day -1	18/08/2010	6.82	.	4.50 - 11.00		
S119	107	Hepatitis B	Hepatitis B	2	% Basophils	%	Day 28	23/09/2010	1.4	Yes	0.0 - 1.0	H	NCS
S119	107	Hepatitis B	Hepatitis B	2	% Eosinophils	%	Day 28	23/09/2010	5.4	.	1.0 - 6.0		
S119	107	Hepatitis B	Hepatitis B	2	% Lymphocytes	%	Day 28	23/09/2010	47.7	Yes	20.0 - 45.0	H	NCS

Subject No.	Randomisation Number	Study Group	Dosage	Dose	Haematology Parameters	Unit	Day	Date of sample Collection	Results	Out of Range	Reference Range	Flag	Clinical Significance **
S119	107	Hepatitis B	Hepatitis B	2	% Monocytes	%	Day 28	23/09/2010	5.8	.	2.0 - 10.0		
S119	107	Hepatitis B	Hepatitis B	2	% Neutrophils	%	Day 28	23/09/2010	39.6	Yes	40.0 - 75.0	L	NCS
S119	107	Hepatitis B	Hepatitis B	2	Haematocrit	%	Day 28	23/09/2010	45.2	.	40.0 - 54.0		
S119	107	Hepatitis B	Hepatitis B	2	Haemoglobin	gm / dl	Day 28	23/09/2010	15.3	.	13.5 - 18.0		
S119	107	Hepatitis B	Hepatitis B	2	MCH	pg	Day 28	23/09/2010	28.9	.	27.0 - 31.0		
S119	107	Hepatitis B	Hepatitis B	2	MCHC	gm %	Day 28	23/09/2010	33.9	.	32.0 - 36.0		
S119	107	Hepatitis B	Hepatitis B	2	MCV	fL	Day 28	23/09/2010	85.3	.	80.0- 96.0		
S119	107	Hepatitis B	Hepatitis B	2	Platelet Count	10E3/ul	Day 28	23/09/2010	179	.	150 - 400		
S119	107	Hepatitis B	Hepatitis B	2	RBC count	10E6/ul	Day 28	23/09/2010	5.3	.	4.60 - 6.20		
S119	107	Hepatitis B	Hepatitis B	2	TLC	10E3/ul	Day 28	23/09/2010	4.41	Yes	4.50 - 11.00	L	NCS
S119	107	Hepatitis B	Hepatitis B	2	% Basophils	%	Day 56	21/10/2010	2.3	Yes	0.0 - 1.0	H	NCS
S119	107	Hepatitis B	Hepatitis B	2	% Eosinophils	%	Day 56	21/10/2010	5	.	1.0 - 6.0		
S119	107	Hepatitis B	Hepatitis B	2	% Lymphocytes	%	Day 56	21/10/2010	38.5	.	20.0 - 45.0		
S119	107	Hepatitis B	Hepatitis B	2	% Monocytes	%	Day 56	21/10/2010	6.2	.	2.0 - 10.0		
S119	107	Hepatitis B	Hepatitis B	2	% Neutrophils	%	Day 56	21/10/2010	48.1	.	40.0 - 75.0		
S119	107	Hepatitis B	Hepatitis B	2	Haematocrit	%	Day 56	21/10/2010	48.4	.	40.0 - 54.0		
S119	107	Hepatitis B	Hepatitis B	2	Haemoglobin	gm / dl	Day 56	21/10/2010	16.6	.	13.5 - 18.0		
S119	107	Hepatitis B	Hepatitis B	2	MCH	pg	Day 56	21/10/2010	28.3	.	27.0 - 31.0		
S119	107	Hepatitis B	Hepatitis B	2	MCHC	gm %	Day 56	21/10/2010	34.4	.	32.0 - 36.0		
S119	107	Hepatitis B	Hepatitis B	2	MCV	fL	Day 56	21/10/2010	82.3	.	80.0- 96.0		
S119	107	Hepatitis B	Hepatitis B	2	Platelet Count	10E3/ul	Day 56	21/10/2010	242	.	150 - 400		
S119	107	Hepatitis B	Hepatitis B	2	RBC count	10E6/ul	Day 56	21/10/2010	5.89	.	4.60 - 6.20		
S119	107	Hepatitis B	Hepatitis B	2	TLC	10E3/ul	Day 56	21/10/2010	4.61	.	4.50 - 11.00		
S119	107	Hepatitis B	Hepatitis B	3	% Basophils	%	Day 180	22/02/2011	0.9	.	0.0 - 1.0		
S119	107	Hepatitis B	Hepatitis B	3	% Eosinophils	%	Day 180	22/02/2011	3.3	.	1.0 - 6.0		
S119	107	Hepatitis B	Hepatitis B	3	% Lymphocytes	%	Day 180	22/02/2011	37	.	20.0 - 45.0		
S119	107	Hepatitis B	Hepatitis B	3	% Monocytes	%	Day 180	22/02/2011	6.4	.	2.0 - 10.0		
S119	107	Hepatitis B	Hepatitis B	3	% Neutrophils	%	Day 180	22/02/2011	52.4	.	40.0 - 75.0		
S119	107	Hepatitis B	Hepatitis B	3	Haematocrit	%	Day 180	22/02/2011	45.8	.	40.0 - 54.0		

Subject No.	Randomisation Number	Study Group	Dosage	Dose	Haematology Parameters	Unit	Day	Date of sample Collection	Results	Out of Range	Reference Range	Flag	Clinical Significance **
S119	107	Hepatitis B	Hepatitis B	3	Haemoglobin	gm / dl	Day 180	22/02/2011	15.6	.	13.5 - 18.0		
S119	107	Hepatitis B	Hepatitis B	3	MCH	pg	Day 180	22/02/2011	29.7	.	27.0 - 31.0		
S119	107	Hepatitis B	Hepatitis B	3	MCHC	gm %	Day 180	22/02/2011	34.1	.	32.0 - 36.0		
S119	107	Hepatitis B	Hepatitis B	3	MCV	fL	Day 180	22/02/2011	87.1	.	80.0- 96.0		
S119	107	Hepatitis B	Hepatitis B	3	Platelet Count	10E3/ul	Day 180	22/02/2011	271	.	150 - 400		
S119	107	Hepatitis B	Hepatitis B	3	RBC count	10E6/ul	Day 180	22/02/2011	5.26	.	4.60 - 6.20		
S119	107	Hepatitis B	Hepatitis B	3	TLC	10E3/ul	Day 180	22/02/2011	5.81	.	4.50 - 11.00		
S119	107	Hepatitis B	Hepatitis B	3	% Basophils	%	Day 208	22/03/2011	0.7	.	0.0 - 1.0		
S119	107	Hepatitis B	Hepatitis B	3	% Eosinophils	%	Day 208	22/03/2011	4.6	.	1.0 - 6.0		
S119	107	Hepatitis B	Hepatitis B	3	% Lymphocytes	%	Day 208	22/03/2011	47.8	Yes	20.0 - 45.0	H	NCS
S119	107	Hepatitis B	Hepatitis B	3	% Monocytes	%	Day 208	22/03/2011	7.2	.	2.0 - 10.0		
S119	107	Hepatitis B	Hepatitis B	3	% Neutrophils	%	Day 208	22/03/2011	39.7	Yes	40.0 - 75.0	L	NCS
S119	107	Hepatitis B	Hepatitis B	3	Haematocrit	%	Day 208	22/03/2011	46.1	.	40.0 - 54.0		
S119	107	Hepatitis B	Hepatitis B	3	Haemoglobin	gm / dl	Day 208	22/03/2011	15.4	.	13.5 - 18.0		
S119	107	Hepatitis B	Hepatitis B	3	MCH	pg	Day 208	22/03/2011	29.2	.	27.0 - 31.0		
S119	107	Hepatitis B	Hepatitis B	3	MCHC	gm %	Day 208	22/03/2011	33.4	.	32.0 - 36.0		
S119	107	Hepatitis B	Hepatitis B	3	MCV	fL	Day 208	22/03/2011	87.4	.	80.0- 96.0		
S119	107	Hepatitis B	Hepatitis B	3	Platelet Count	10E3/ul	Day 208	22/03/2011	266	.	150 - 400		
S119	107	Hepatitis B	Hepatitis B	3	RBC count	10E6/ul	Day 208	22/03/2011	5.27	.	4.60 - 6.20		
S119	107	Hepatitis B	Hepatitis B	3	TLC	10E3/ul	Day 208	22/03/2011	4.73	.	4.50 - 11.00		
S119	107	Hepatitis B	Hepatitis B	3	% Basophils	%	Day 365	25/08/2011	0.8	.	0.0 - 1.0		
S119	107	Hepatitis B	Hepatitis B	3	% Eosinophils	%	Day 365	25/08/2011	3.8	.	1.0 - 6.0		
S119	107	Hepatitis B	Hepatitis B	3	% Lymphocytes	%	Day 365	25/08/2011	26.3	.	20.0 - 45.0		
S119	107	Hepatitis B	Hepatitis B	3	% Monocytes	%	Day 365	25/08/2011	6.5	.	2.0 - 10.0		
S119	107	Hepatitis B	Hepatitis B	3	% Neutrophils	%	Day 365	25/08/2011	62.5	.	40.0 - 75.0		
S119	107	Hepatitis B	Hepatitis B	3	Haematocrit	%	Day 365	25/08/2011	41.7	.	40.0 - 54.0		
S119	107	Hepatitis B	Hepatitis B	3	Haemoglobin	gm / dl	Day 365	25/08/2011	13.9	.	13.5 - 18.0		
S119	107	Hepatitis B	Hepatitis B	3	MCH	pg	Day 365	25/08/2011	28.1	.	27.0 - 31.0		
S119	107	Hepatitis B	Hepatitis B	3	MCHC	gm %	Day 365	25/08/2011	33.2	.	32.0 - 36.0		

Subject No.	Randomisation Number	Study Group	Dosage	Dose	Haematology Parameters	Unit	Day	Date of sample Collection	Results	Out of Range	Reference Range	Flag	Clinical Significance **
S119	107	Hepatitis B	Hepatitis B	3	MCV	fL	Day 365	25/08/2011	84.6	.	80.0- 96.0		
S119	107	Hepatitis B	Hepatitis B	3	Platelet Count	10E3/ul	Day 365	25/08/2011	343	.	150 - 400		
S119	107	Hepatitis B	Hepatitis B	3	RBC count	10E6/ul	Day 365	25/08/2011	4.93	.	4.60 - 6.20		
S119	107	Hepatitis B	Hepatitis B	3	TLC	10E3/ul	Day 365	25/08/2011	6.74	.	4.50 - 11.00		
S120	104	JAIVAC-1	Dosage A		% Basophils	%	Day -14 to Day -1	18/08/2010	1.4	Yes	0.0 - 1.0	H	NCS
S120	104	JAIVAC-1	Dosage A		% Eosinophils	%	Day -14 to Day -1	18/08/2010	2.9	.	1.0 - 6.0		
S120	104	JAIVAC-1	Dosage A		% Lymphocytes	%	Day -14 to Day -1	18/08/2010	19.5	Yes	20.0 - 45.0	L	NCS
S120	104	JAIVAC-1	Dosage A		% Monocytes	%	Day -14 to Day -1	18/08/2010	4.8	.	2.0 - 10.0		
S120	104	JAIVAC-1	Dosage A		% Neutrophils	%	Day -14 to Day -1	18/08/2010	71.4	.	40.0 - 75.0		
S120	104	JAIVAC-1	Dosage A		Haematocrit	%	Day -14 to Day -1	18/08/2010	46.3	.	40.0 - 54.0		
S120	104	JAIVAC-1	Dosage A		Haemoglobin	gm / dl	Day -14 to Day -1	18/08/2010	15.2	.	13.5 - 18.0		
S120	104	JAIVAC-1	Dosage A		MCH	pg	Day -14 to Day -1	18/08/2010	27.7	.	27.0 - 31.0		
S120	104	JAIVAC-1	Dosage A		MCHC	gm %	Day -14 to Day -1	18/08/2010	32.8	.	32.0 - 36.0		
S120	104	JAIVAC-1	Dosage A		MCV	fL	Day -14 to Day -1	18/08/2010	84.4	.	80.0- 96.0		
S120	104	JAIVAC-1	Dosage A		Platelet Count	10E3/ul	Day -14 to Day -1	18/08/2010	287	.	150 - 400		
S120	104	JAIVAC-1	Dosage A		RBC count	10E6/ul	Day -14 to Day -1	18/08/2010	5.49	.	4.60 - 6.20		
S120	104	JAIVAC-1	Dosage A		TLC	10E3/ul	Day -14 to Day -1	18/08/2010	9.57	.	4.50 - 11.00		
S120	104	JAIVAC-1	Dosage A	2	% Basophils	%	Day 28	23/09/2010	1.3	Yes	0.0 - 1.0	H	NCS
S120	104	JAIVAC-1	Dosage A	2	% Eosinophils	%	Day 28	23/09/2010	1.8	.	1.0 - 6.0		
S120	104	JAIVAC-1	Dosage A	2	% Lymphocytes	%	Day 28	23/09/2010	21.6	.	20.0 - 45.0		
S120	104	JAIVAC-1	Dosage A	2	% Monocytes	%	Day 28	23/09/2010	5.3	.	2.0 - 10.0		
S120	104	JAIVAC-1	Dosage A	2	% Neutrophils	%	Day 28	23/09/2010	70	.	40.0 - 75.0		
S120	104	JAIVAC-1	Dosage A	2	Haematocrit	%	Day 28	23/09/2010	44.7	.	40.0 - 54.0		
S120	104	JAIVAC-1	Dosage A	2	Haemoglobin	gm / dl	Day 28	23/09/2010	15.2	.	13.5 - 18.0		
S120	104	JAIVAC-1	Dosage A	2	MCH	pg	Day 28	23/09/2010	29	.	27.0 - 31.0		
S120	104	JAIVAC-1	Dosage A	2	MCHC	gm %	Day 28	23/09/2010	34.1	.	32.0 - 36.0		
S120	104	JAIVAC-1	Dosage A	2	MCV	fL	Day 28	23/09/2010	85	.	80.0- 96.0		
S120	104	JAIVAC-1	Dosage A	2	Platelet Count	10E3/ul	Day 28	23/09/2010	277	.	150 - 400		
S120	104	JAIVAC-1	Dosage A	2	RBC count	10E6/ul	Day 28	23/09/2010	5.26	.	4.60 - 6.20		

Subject No.	Randomisation Number	Study Group	Dosage	Dose	Haematology Parameters	Unit	Day	Date of sample Collection	Results	Out of Range	Reference Range	Flag	Clinical Significance **
S120	104	JAIVAC-1	Dosage A	2	TLC	10E3/ul	Day 28	23/09/2010	7.7	.	4.50 - 11.00		
S120	104	JAIVAC-1	Dosage A	2	% Basophils	%	Day 56	21/10/2010	0.6	.	0.0 - 1.0		
S120	104	JAIVAC-1	Dosage A	2	% Eosinophils	%	Day 56	21/10/2010	1.5	.	1.0 - 6.0		
S120	104	JAIVAC-1	Dosage A	2	% Lymphocytes	%	Day 56	21/10/2010	20.4	.	20.0 - 45.0		
S120	104	JAIVAC-1	Dosage A	2	% Monocytes	%	Day 56	21/10/2010	5.2	.	2.0 - 10.0		
S120	104	JAIVAC-1	Dosage A	2	% Neutrophils	%	Day 56	21/10/2010	72.4	.	40.0 - 75.0		
S120	104	JAIVAC-1	Dosage A	2	Haematocrit	%	Day 56	21/10/2010	45.4	.	40.0 - 54.0		
S120	104	JAIVAC-1	Dosage A	2	Haemoglobin	gm / dl	Day 56	21/10/2010	15.5	.	13.5 - 18.0		
S120	104	JAIVAC-1	Dosage A	2	MCH	pg	Day 56	21/10/2010	28.8	.	27.0 - 31.0		
S120	104	JAIVAC-1	Dosage A	2	MCHC	gm %	Day 56	21/10/2010	34.2	.	32.0 - 36.0		
S120	104	JAIVAC-1	Dosage A	2	MCV	fL	Day 56	21/10/2010	84.1	.	80.0- 96.0		
S120	104	JAIVAC-1	Dosage A	2	Platelet Count	10E3/ul	Day 56	21/10/2010	327	.	150 - 400		
S120	104	JAIVAC-1	Dosage A	2	RBC count	10E6/ul	Day 56	21/10/2010	5.4	.	4.60 - 6.20		
S120	104	JAIVAC-1	Dosage A	2	TLC	10E3/ul	Day 56	21/10/2010	10.16	.	4.50 - 11.00		
S120	104	JAIVAC-1	Dosage A	3	% Basophils	%	Day 180	22/02/2011	0.8	.	0.0 - 1.0		
S120	104	JAIVAC-1	Dosage A	3	% Eosinophils	%	Day 180	22/02/2011	1.4	.	1.0 - 6.0		
S120	104	JAIVAC-1	Dosage A	3	% Lymphocytes	%	Day 180	22/02/2011	19.5	Yes	20.0 - 45.0	L	NCS
S120	104	JAIVAC-1	Dosage A	3	% Monocytes	%	Day 180	22/02/2011	5.4	.	2.0 - 10.0		
S120	104	JAIVAC-1	Dosage A	3	% Neutrophils	%	Day 180	22/02/2011	73	.	40.0 - 75.0		
S120	104	JAIVAC-1	Dosage A	3	Haematocrit	%	Day 180	22/02/2011	45.4	.	40.0 - 54.0		
S120	104	JAIVAC-1	Dosage A	3	Haemoglobin	gm / dl	Day 180	22/02/2011	15.6	.	13.5 - 18.0		
S120	104	JAIVAC-1	Dosage A	3	MCH	pg	Day 180	22/02/2011	29.6	.	27.0 - 31.0		
S120	104	JAIVAC-1	Dosage A	3	MCHC	gm %	Day 180	22/02/2011	34.4	.	32.0 - 36.0		
S120	104	JAIVAC-1	Dosage A	3	MCV	fL	Day 180	22/02/2011	86.1	.	80.0- 96.0		
S120	104	JAIVAC-1	Dosage A	3	Platelet Count	10E3/ul	Day 180	22/02/2011	374	.	150 - 400		
S120	104	JAIVAC-1	Dosage A	3	RBC count	10E6/ul	Day 180	22/02/2011	5.27	.	4.60 - 6.20		
S120	104	JAIVAC-1	Dosage A	3	TLC	10E3/ul	Day 180	22/02/2011	10.33	.	4.50 - 11.00		
S120	104	JAIVAC-1	Dosage A	3	% Basophils	%	Day 208	22/03/2011	1.7	Yes	0.0 - 1.0	H	NCS
S120	104	JAIVAC-1	Dosage A	3	% Eosinophils	%	Day 208	22/03/2011	1.4	.	1.0 - 6.0		

Subject No.	Randomisation Number	Study Group	Dosage	Dose	Haematology Parameters	Unit	Day	Date of sample Collection	Results	Out of Range	Reference Range	Flag	Clinical Significance **
S120	104	JAIVAC-1	Dosage A	3	% Lymphocytes	%	Day 208	22/03/2011	22.6	.	20.0 - 45.0		
S120	104	JAIVAC-1	Dosage A	3	% Monocytes	%	Day 208	22/03/2011	4.9	.	2.0 - 10.0		
S120	104	JAIVAC-1	Dosage A	3	% Neutrophils	%	Day 208	22/03/2011	69.5	.	40.0 - 75.0		
S120	104	JAIVAC-1	Dosage A	3	Haematocrit	%	Day 208	22/03/2011	45.8	.	40.0 - 54.0		
S120	104	JAIVAC-1	Dosage A	3	Haemoglobin	gm / dl	Day 208	22/03/2011	15.8	.	13.5 - 18.0		
S120	104	JAIVAC-1	Dosage A	3	MCH	pg	Day 208	22/03/2011	29.7	.	27.0 - 31.0		
S120	104	JAIVAC-1	Dosage A	3	MCHC	gm %	Day 208	22/03/2011	34.5	.	32.0 - 36.0		
S120	104	JAIVAC-1	Dosage A	3	MCV	fL	Day 208	22/03/2011	86	.	80.0- 96.0		
S120	104	JAIVAC-1	Dosage A	3	Platelet Count	10E3/ul	Day 208	22/03/2011	296	.	150 - 400		
S120	104	JAIVAC-1	Dosage A	3	RBC count	10E6/ul	Day 208	22/03/2011	5.32	.	4.60 - 6.20		
S120	104	JAIVAC-1	Dosage A	3	TLC	10E3/ul	Day 208	22/03/2011	8.06	.	4.50 - 11.00		
S120	104	JAIVAC-1	Dosage A	3	% Basophils	%	Day 365	25/08/2011	0	.	0.0 - 1.0		
S120	104	JAIVAC-1	Dosage A	3	% Eosinophils	%	Day 365	25/08/2011	1	.	1.0 - 6.0		
S120	104	JAIVAC-1	Dosage A	3	% Lymphocytes	%	Day 365	25/08/2011	19	Yes	20.0 - 45.0	L	NCS
S120	104	JAIVAC-1	Dosage A	3	% Monocytes	%	Day 365	25/08/2011	4	.	2.0 - 10.0		
S120	104	JAIVAC-1	Dosage A	3	% Neutrophils	%	Day 365	25/08/2011	76	Yes	40.0 - 75.0	H	NCS
S120	104	JAIVAC-1	Dosage A	3	Haematocrit	%	Day 365	25/08/2011	46.8	.	40.0 - 54.0		
S120	104	JAIVAC-1	Dosage A	3	Haemoglobin	gm / dl	Day 365	25/08/2011	16.1	.	13.5 - 18.0		
S120	104	JAIVAC-1	Dosage A	3	MCH	pg	Day 365	25/08/2011	30.2	.	27.0 - 31.0		
S120	104	JAIVAC-1	Dosage A	3	MCHC	gm %	Day 365	25/08/2011	34.4	.	32.0 - 36.0		
S120	104	JAIVAC-1	Dosage A	3	MCV	fL	Day 365	25/08/2011	87.7	.	80.0- 96.0		
S120	104	JAIVAC-1	Dosage A	3	Platelet Count	10E3/ul	Day 365	25/08/2011	344	.	150 - 400		
S120	104	JAIVAC-1	Dosage A	3	RBC count	10E6/ul	Day 365	25/08/2011	5.34	.	4.60 - 6.20		
S120	104	JAIVAC-1	Dosage A	3	TLC	10E3/ul	Day 365	25/08/2011	10.91	.	4.50 - 11.00		
S124	106	Hepatitis B	Hepatitis B		% Basophils	%	Day -14 to Day -1	18/08/2010	1.8	Yes	0.0 - 1.0	H	NCS
S124	106	Hepatitis B	Hepatitis B		% Eosinophils	%	Day -14 to Day -1	18/08/2010	5.3	.	1.0 - 6.0		
S124	106	Hepatitis B	Hepatitis B		% Lymphocytes	%	Day -14 to Day -1	18/08/2010	31.3	.	20.0 - 45.0		
S124	106	Hepatitis B	Hepatitis B		% Monocytes	%	Day -14 to Day -1	18/08/2010	6	.	2.0 - 10.0		
S124	106	Hepatitis B	Hepatitis B		% Neutrophils	%	Day -14 to Day -1	18/08/2010	55.6	.	40.0 - 75.0		

Subject No.	Randomisation Number	Study Group	Dosage	Dose	Haematology Parameters	Unit	Day	Date of sample Collection	Results	Out of Range	Reference Range	Flag	Clinical Significance **
S124	106	Hepatitis B	Hepatitis B		Haematocrit	%	Day -14 to Day -1	18/08/2010	46.1	.	40.0 - 54.0		
S124	106	Hepatitis B	Hepatitis B		Haemoglobin	gm / dl	Day -14 to Day -1	18/08/2010	14	.	13.5 - 18.0		
S124	106	Hepatitis B	Hepatitis B		MCH	pg	Day -14 to Day -1	18/08/2010	25	Yes	27.0 - 31.0	L	NCS
S124	106	Hepatitis B	Hepatitis B		MCHC	gm %	Day -14 to Day -1	18/08/2010	30.4	Yes	32.0 - 36.0	L	NCS
S124	106	Hepatitis B	Hepatitis B		MCV	fL	Day -14 to Day -1	18/08/2010	82	.	80.0- 96.0		
S124	106	Hepatitis B	Hepatitis B		Platelet Count	10E3/ul	Day -14 to Day -1	18/08/2010	337	.	150 - 400		
S124	106	Hepatitis B	Hepatitis B		RBC count	10E6/ul	Day -14 to Day -1	18/08/2010	5.63	.	4.60 - 6.20		
S124	106	Hepatitis B	Hepatitis B		TLC	10E3/ul	Day -14 to Day -1	18/08/2010	7.28	.	4.50 - 11.00		
S124	106	Hepatitis B	Hepatitis B	2	% Basophils	%	Day 28	23/09/2010	1.4	Yes	0.0 - 1.0	H	NCS
S124	106	Hepatitis B	Hepatitis B	2	% Eosinophils	%	Day 28	23/09/2010	4.1	.	1.0 - 6.0		
S124	106	Hepatitis B	Hepatitis B	2	% Lymphocytes	%	Day 28	23/09/2010	24.3	.	20.0 - 45.0		
S124	106	Hepatitis B	Hepatitis B	2	% Monocytes	%	Day 28	23/09/2010	6.6	.	2.0 - 10.0		
S124	106	Hepatitis B	Hepatitis B	2	% Neutrophils	%	Day 28	23/09/2010	63.6	.	40.0 - 75.0		
S124	106	Hepatitis B	Hepatitis B	2	Haematocrit	%	Day 28	23/09/2010	41.9	.	40.0 - 54.0		
S124	106	Hepatitis B	Hepatitis B	2	Haemoglobin*	gm / dl	Day 28	23/09/2010	13.3	Yes	13.5 - 18.0	L	NCS
S124	106	Hepatitis B	Hepatitis B	2	MCH	pg	Day 28	23/09/2010	24.6	Yes	27.0 - 31.0	L	NCS
S124	106	Hepatitis B	Hepatitis B	2	MCHC	gm %	Day 28	23/09/2010	31.7	Yes	32.0 - 36.0	L	NCS
S124	106	Hepatitis B	Hepatitis B	2	MCV	fL	Day 28	23/09/2010	77.8	Yes	80.0- 96.0	L	NCS
S124	106	Hepatitis B	Hepatitis B	2	Platelet Count	10E3/ul	Day 28	23/09/2010	293	.	150 - 400		
S124	106	Hepatitis B	Hepatitis B	2	RBC count	10E6/ul	Day 28	23/09/2010	5.39	.	4.60 - 6.20		
S124	106	Hepatitis B	Hepatitis B	2	TLC	10E3/ul	Day 28	23/09/2010	7.81	.	4.50 - 11.00		
S124	106	Hepatitis B	Hepatitis B	2	% Basophils	%	Day 56	21/10/2010	2	Yes	0.0 - 1.0	H	NCS
S124	106	Hepatitis B	Hepatitis B	2	% Eosinophils	%	Day 56	21/10/2010	4.9	.	1.0 - 6.0		
S124	106	Hepatitis B	Hepatitis B	2	% Lymphocytes	%	Day 56	21/10/2010	26.3	.	20.0 - 45.0		
S124	106	Hepatitis B	Hepatitis B	2	% Monocytes	%	Day 56	21/10/2010	6.7	.	2.0 - 10.0		
S124	106	Hepatitis B	Hepatitis B	2	% Neutrophils	%	Day 56	21/10/2010	60	.	40.0 - 75.0		
S124	106	Hepatitis B	Hepatitis B	2	Haematocrit	%	Day 56	21/10/2010	41.7	.	40.0 - 54.0		
S124	106	Hepatitis B	Hepatitis B	2	Haemoglobin*	gm / dl	Day 56	21/10/2010	13.1	Yes	13.5 - 18.0	L	CS
S124	106	Hepatitis B	Hepatitis B	2	MCH	pg	Day 56	21/10/2010	23.6	Yes	27.0 - 31.0	L	CS

Subject No.	Randomisation Number	Study Group	Dosage	Dose	Haematology Parameters	Unit	Day	Date of sample Collection	Results	Out of Range	Reference Range	Flag	Clinical Significance **
S124	106	Hepatitis B	Hepatitis B	2	MCHC	gm %	Day 56	21/10/2010	31.4	Yes	32.0 - 36.0	L	CS
S124	106	Hepatitis B	Hepatitis B	2	MCV	fL	Day 56	21/10/2010	75.2	Yes	80.0- 96.0	L	CS
S124	106	Hepatitis B	Hepatitis B	2	Platelet Count	10E3/ul	Day 56	21/10/2010	377	.	150 - 400		
S124	106	Hepatitis B	Hepatitis B	2	RBC count	10E6/ul	Day 56	21/10/2010	5.54	.	4.60 - 6.20		
S124	106	Hepatitis B	Hepatitis B	2	TLC	10E3/ul	Day 56	21/10/2010	7.43	.	4.50 - 11.00		
S124	106	Hepatitis B	Hepatitis B	3	% Basophils	%	Day 180	22/02/2011	1	.	0.0 - 1.0		
S124	106	Hepatitis B	Hepatitis B	3	% Eosinophils	%	Day 180	22/02/2011	3.8	.	1.0 - 6.0		
S124	106	Hepatitis B	Hepatitis B	3	% Lymphocytes	%	Day 180	22/02/2011	27.6	.	20.0 - 45.0		
S124	106	Hepatitis B	Hepatitis B	3	% Monocytes	%	Day 180	22/02/2011	8.2	.	2.0 - 10.0		
S124	106	Hepatitis B	Hepatitis B	3	% Neutrophils	%	Day 180	22/02/2011	59.3	.	40.0 - 75.0		
S124	106	Hepatitis B	Hepatitis B	3	Haematocrit	%	Day 180	22/02/2011	45.3	.	40.0 - 54.0		
S124	106	Hepatitis B	Hepatitis B	3	Haemoglobin	gm / dl	Day 180	22/02/2011	14.4	.	13.5 - 18.0		
S124	106	Hepatitis B	Hepatitis B	3	MCH	pg	Day 180	22/02/2011	24.8	Yes	27.0 - 31.0	L	NCS
S124	106	Hepatitis B	Hepatitis B	3	MCHC	gm %	Day 180	22/02/2011	31.7	Yes	32.0 - 36.0	L	NCS
S124	106	Hepatitis B	Hepatitis B	3	MCV	fL	Day 180	22/02/2011	78.2	Yes	80.0- 96.0	L	NCS
S124	106	Hepatitis B	Hepatitis B	3	Platelet Count	10E3/ul	Day 180	22/02/2011	287	.	150 - 400		
S124	106	Hepatitis B	Hepatitis B	3	RBC count	10E6/ul	Day 180	22/02/2011	5.8	.	4.60 - 6.20		
S124	106	Hepatitis B	Hepatitis B	3	TLC	10E3/ul	Day 180	22/02/2011	6.14	.	4.50 - 11.00		
S124	106	Hepatitis B	Hepatitis B	3	% Basophils	%	Day 208	22/03/2011	2	Yes	0.0 - 1.0	H	NCS
S124	106	Hepatitis B	Hepatitis B	3	% Eosinophils	%	Day 208	22/03/2011	4	.	1.0 - 6.0		
S124	106	Hepatitis B	Hepatitis B	3	% Lymphocytes	%	Day 208	22/03/2011	28.1	.	20.0 - 45.0		
S124	106	Hepatitis B	Hepatitis B	3	% Monocytes	%	Day 208	22/03/2011	7.5	.	2.0 - 10.0		
S124	106	Hepatitis B	Hepatitis B	3	% Neutrophils	%	Day 208	22/03/2011	58.4	.	40.0 - 75.0		
S124	106	Hepatitis B	Hepatitis B	3	Haematocrit	%	Day 208	22/03/2011	45	.	40.0 - 54.0		
S124	106	Hepatitis B	Hepatitis B	3	Haemoglobin	gm / dl	Day 208	22/03/2011	14.1	.	13.5 - 18.0		
S124	106	Hepatitis B	Hepatitis B	3	MCH	pg	Day 208	22/03/2011	24.7	Yes	27.0 - 31.0	L	NCS
S124	106	Hepatitis B	Hepatitis B	3	MCHC	gm %	Day 208	22/03/2011	31.4	Yes	32.0 - 36.0	L	NCS
S124	106	Hepatitis B	Hepatitis B	3	MCV	fL	Day 208	22/03/2011	78.7	Yes	80.0- 96.0	L	NCS
S124	106	Hepatitis B	Hepatitis B	3	Platelet Count	10E3/ul	Day 208	22/03/2011	334	.	150 - 400		

Subject No.	Randomisation Number	Study Group	Dosage	Dose	Haematology Parameters	Unit	Day	Date of sample Collection	Results	Out of Range	Reference Range	Flag	Clinical Significance **
S124	106	Hepatitis B	Hepatitis B	3	RBC count	10E6/ul	Day 208	22/03/2011	5.72	.	4.60 - 6.20		
S124	106	Hepatitis B	Hepatitis B	3	TLC	10E3/ul	Day 208	22/03/2011	6.99	.	4.50 - 11.00		
S124	106	Hepatitis B	Hepatitis B	3	% Basophils	%	Day 365	25/08/2011	1.7	Yes	0.0 - 1.0	H	NCS
S124	106	Hepatitis B	Hepatitis B	3	% Eosinophils	%	Day 365	25/08/2011	4.9	.	1.0 - 6.0		
S124	106	Hepatitis B	Hepatitis B	3	% Lymphocytes	%	Day 365	25/08/2011	31.9	.	20.0 - 45.0		
S124	106	Hepatitis B	Hepatitis B	3	% Monocytes	%	Day 365	25/08/2011	8.4	.	2.0 - 10.0		
S124	106	Hepatitis B	Hepatitis B	3	% Neutrophils	%	Day 365	25/08/2011	53.1	.	40.0 - 75.0		
S124	106	Hepatitis B	Hepatitis B	3	Haematocrit	%	Day 365	25/08/2011	45	.	40.0 - 54.0		
S124	106	Hepatitis B	Hepatitis B	3	Haemoglobin	gm / dl	Day 365	25/08/2011	14	.	13.5 - 18.0		
S124	106	Hepatitis B	Hepatitis B	3	MCH	pg	Day 365	25/08/2011	23.7	Yes	27.0 - 31.0	L	NCS
S124	106	Hepatitis B	Hepatitis B	3	MCHC	gm %	Day 365	25/08/2011	31.1	Yes	32.0 - 36.0	L	NCS
S124	106	Hepatitis B	Hepatitis B	3	MCV	fL	Day 365	25/08/2011	76	Yes	80.0- 96.0	L	NCS
S124	106	Hepatitis B	Hepatitis B	3	Platelet Count	10E3/ul	Day 365	25/08/2011	297	.	150 - 400		
S124	106	Hepatitis B	Hepatitis B	3	RBC count	10E6/ul	Day 365	25/08/2011	5.92	.	4.60 - 6.20		
S124	106	Hepatitis B	Hepatitis B	3	TLC	10E3/ul	Day 365	25/08/2011	5.55	.	4.50 - 11.00		
S125	102	JAIVAC-1	Dosage A		% Basophils	%	Day -14 to Day -1	18/08/2010	0.9	.	0.0 - 1.0		
S125	102	JAIVAC-1	Dosage A		% Eosinophils	%	Day -14 to Day -1	18/08/2010	3.1	.	1.0 - 6.0		
S125	102	JAIVAC-1	Dosage A		% Lymphocytes	%	Day -14 to Day -1	18/08/2010	33.4	.	20.0 - 45.0		
S125	102	JAIVAC-1	Dosage A		% Monocytes	%	Day -14 to Day -1	18/08/2010	4.7	.	2.0 - 10.0		
S125	102	JAIVAC-1	Dosage A		% Neutrophils	%	Day -14 to Day -1	18/08/2010	57.9	.	40.0 - 75.0		
S125	102	JAIVAC-1	Dosage A		Haematocrit	%	Day -14 to Day -1	18/08/2010	50.7	.	40.0 - 54.0		
S125	102	JAIVAC-1	Dosage A		Haemoglobin	gm / dl	Day -14 to Day -1	18/08/2010	16.8	.	13.5 - 18.0		
S125	102	JAIVAC-1	Dosage A		MCH	pg	Day -14 to Day -1	18/08/2010	29.6	.	27.0 - 31.0		
S125	102	JAIVAC-1	Dosage A		MCHC	gm %	Day -14 to Day -1	18/08/2010	33.2	.	32.0 - 36.0		
S125	102	JAIVAC-1	Dosage A		MCV	fL	Day -14 to Day -1	18/08/2010	89	.	80.0- 96.0		
S125	102	JAIVAC-1	Dosage A		Platelet Count	10E3/ul	Day -14 to Day -1	18/08/2010	254	.	150 - 400		
S125	102	JAIVAC-1	Dosage A		RBC count	10E6/ul	Day -14 to Day -1	18/08/2010	5.7	.	4.60 - 6.20		
S125	102	JAIVAC-1	Dosage A		TLC	10E3/ul	Day -14 to Day -1	18/08/2010	6.19	.	4.50 - 11.00		
S125	102	JAIVAC-1	Dosage A	2	% Basophils	%	Day 28	21/09/2010	1.4	Yes	0.0 - 1.0	H	NCS

Subject No.	Randomisation Number	Study Group	Dosage	Dose	Haematology Parameters	Unit	Day	Date of sample Collection	Results	Out of Range	Reference Range	Flag	Clinical Significance **
S125	102	JAIVAC-1	Dosage A	2	% Eosinophils	%	Day 28	21/09/2010	2.9	.	1.0 - 6.0		
S125	102	JAIVAC-1	Dosage A	2	% Lymphocytes	%	Day 28	21/09/2010	30.2	.	20.0 - 45.0		
S125	102	JAIVAC-1	Dosage A	2	% Monocytes	%	Day 28	21/09/2010	5.1	.	2.0 - 10.0		
S125	102	JAIVAC-1	Dosage A	2	% Neutrophils	%	Day 28	21/09/2010	60.4	.	40.0 - 75.0		
S125	102	JAIVAC-1	Dosage A	2	Haematocrit	%	Day 28	21/09/2010	48	.	40.0 - 54.0		
S125	102	JAIVAC-1	Dosage A	2	Haemoglobin	gm / dl	Day 28	21/09/2010	16.5	.	13.5 - 18.0		
S125	102	JAIVAC-1	Dosage A	2	MCH	pg	Day 28	21/09/2010	30.5	.	27.0 - 31.0		
S125	102	JAIVAC-1	Dosage A	2	MCHC	gm %	Day 28	21/09/2010	34.3	.	32.0 - 36.0		
S125	102	JAIVAC-1	Dosage A	2	MCV	fL	Day 28	21/09/2010	88.8	.	80.0- 96.0		
S125	102	JAIVAC-1	Dosage A	2	Platelet Count	10E3/ul	Day 28	21/09/2010	241	.	150 - 400		
S125	102	JAIVAC-1	Dosage A	2	RBC count	10E6/ul	Day 28	21/09/2010	5.4	.	4.60 - 6.20		
S125	102	JAIVAC-1	Dosage A	2	TLC	10E3/ul	Day 28	21/09/2010	6.5	.	4.50 - 11.00		
S125	102	JAIVAC-1	Dosage A	2	% Basophils	%	Day 56	19/10/2010	2	Yes	0.0 - 1.0	H	NCS
S125	102	JAIVAC-1	Dosage A	2	% Eosinophils	%	Day 56	19/10/2010	4.5	.	1.0 - 6.0		
S125	102	JAIVAC-1	Dosage A	2	% Lymphocytes	%	Day 56	19/10/2010	31.3	.	20.0 - 45.0		
S125	102	JAIVAC-1	Dosage A	2	% Monocytes	%	Day 56	19/10/2010	5.3	.	2.0 - 10.0		
S125	102	JAIVAC-1	Dosage A	2	% Neutrophils	%	Day 56	19/10/2010	56.9	.	40.0 - 75.0		
S125	102	JAIVAC-1	Dosage A	2	Haematocrit	%	Day 56	19/10/2010	47	.	40.0 - 54.0		
S125	102	JAIVAC-1	Dosage A	2	Haemoglobin	gm / dl	Day 56	19/10/2010	15.9	.	13.5 - 18.0		
S125	102	JAIVAC-1	Dosage A	2	MCH	pg	Day 56	19/10/2010	30.1	.	27.0 - 31.0		
S125	102	JAIVAC-1	Dosage A	2	MCHC	gm %	Day 56	19/10/2010	33.9	.	32.0 - 36.0		
S125	102	JAIVAC-1	Dosage A	2	MCV	fL	Day 56	19/10/2010	88.8	.	80.0- 96.0		
S125	102	JAIVAC-1	Dosage A	2	Platelet Count	10E3/ul	Day 56	19/10/2010	299	.	150 - 400		
S125	102	JAIVAC-1	Dosage A	2	RBC count	10E6/ul	Day 56	19/10/2010	5.29	.	4.60 - 6.20		
S125	102	JAIVAC-1	Dosage A	2	TLC	10E3/ul	Day 56	19/10/2010	5.77	.	4.50 - 11.00		
S125	102	JAIVAC-1	Dosage A	3	% Basophils	%	Day 180	21/02/2011	1.1	Yes	0.0 - 1.0	H	NCS
S125	102	JAIVAC-1	Dosage A	3	% Eosinophils	%	Day 180	21/02/2011	2.1	.	1.0 - 6.0		
S125	102	JAIVAC-1	Dosage A	3	% Lymphocytes	%	Day 180	21/02/2011	26.1	.	20.0 - 45.0		
S125	102	JAIVAC-1	Dosage A	3	% Monocytes	%	Day 180	21/02/2011	3.3	.	2.0 - 10.0		

Subject No.	Randomisation Number	Study Group	Dosage	Dose	Haematology Parameters	Unit	Day	Date of sample Collection	Results	Out of Range	Reference Range	Flag	Clinical Significance **
S125	102	JAIVAC-1	Dosage A	3	% Neutrophils	%	Day 180	21/02/2011	67.3	.	40.0 - 75.0		
S125	102	JAIVAC-1	Dosage A	3	Haematocrit	%	Day 180	21/02/2011	47.1	.	40.0 - 54.0		
S125	102	JAIVAC-1	Dosage A	3	Haemoglobin	gm / dl	Day 180	21/02/2011	15.8	.	13.5 - 18.0		
S125	102	JAIVAC-1	Dosage A	3	MCH	pg	Day 180	21/02/2011	30	.	27.0 - 31.0		
S125	102	JAIVAC-1	Dosage A	3	MCHC	gm %	Day 180	21/02/2011	33.5	.	32.0 - 36.0		
S125	102	JAIVAC-1	Dosage A	3	MCV	fL	Day 180	21/02/2011	89.6	.	80.0- 96.0		
S125	102	JAIVAC-1	Dosage A	3	Platelet Count	10E3/ul	Day 180	21/02/2011	274	.	150 - 400		
S125	102	JAIVAC-1	Dosage A	3	RBC count	10E6/ul	Day 180	21/02/2011	5.26	.	4.60 - 6.20		
S125	102	JAIVAC-1	Dosage A	3	TLC	10E3/ul	Day 180	21/02/2011	5.04	.	4.50 - 11.00		
S125	102	JAIVAC-1	Dosage A	3	% Basophils	%	Day 208	21/03/2011	0.8	.	0.0 - 1.0		
S125	102	JAIVAC-1	Dosage A	3	% Eosinophils	%	Day 208	21/03/2011	1.8	.	1.0 - 6.0		
S125	102	JAIVAC-1	Dosage A	3	% Lymphocytes	%	Day 208	21/03/2011	19.3	Yes	20.0 - 45.0	L	NCS
S125	102	JAIVAC-1	Dosage A	3	% Monocytes	%	Day 208	21/03/2011	5.1	.	2.0 - 10.0		
S125	102	JAIVAC-1	Dosage A	3	% Neutrophils	%	Day 208	21/03/2011	73	.	40.0 - 75.0		
S125	102	JAIVAC-1	Dosage A	3	Haematocrit	%	Day 208	21/03/2011	46.5	.	40.0 - 54.0		
S125	102	JAIVAC-1	Dosage A	3	Haemoglobin	gm / dl	Day 208	21/03/2011	16.2	.	13.5 - 18.0		
S125	102	JAIVAC-1	Dosage A	3	MCH	pg	Day 208	21/03/2011	30.8	.	27.0 - 31.0		
S125	102	JAIVAC-1	Dosage A	3	MCHC	gm %	Day 208	21/03/2011	34.8	.	32.0 - 36.0		
S125	102	JAIVAC-1	Dosage A	3	MCV	fL	Day 208	21/03/2011	88.5	.	80.0- 96.0		
S125	102	JAIVAC-1	Dosage A	3	Platelet Count	10E3/ul	Day 208	21/03/2011	302	.	150 - 400		
S125	102	JAIVAC-1	Dosage A	3	RBC count	10E6/ul	Day 208	21/03/2011	5.26	.	4.60 - 6.20		
S125	102	JAIVAC-1	Dosage A	3	TLC	10E3/ul	Day 208	21/03/2011	8.02	.	4.50 - 11.00		
S125	102	JAIVAC-1	Dosage A	3	% Basophils	%	Day 365	26/08/2011	0.8	.	0.0 - 1.0		
S125	102	JAIVAC-1	Dosage A	3	% Eosinophils	%	Day 365	26/08/2011	2.6	.	1.0 - 6.0		
S125	102	JAIVAC-1	Dosage A	3	% Lymphocytes	%	Day 365	26/08/2011	23.8	.	20.0 - 45.0		
S125	102	JAIVAC-1	Dosage A	3	% Monocytes	%	Day 365	26/08/2011	5.2	.	2.0 - 10.0		
S125	102	JAIVAC-1	Dosage A	3	% Neutrophils	%	Day 365	26/08/2011	67.6	.	40.0 - 75.0		
S125	102	JAIVAC-1	Dosage A	3	Haematocrit	%	Day 365	26/08/2011	51.9	.	40.0 - 54.0		
S125	102	JAIVAC-1	Dosage A	3	Haemoglobin	gm / dl	Day 365	26/08/2011	17.7	.	13.5 - 18.0		

Subject No.	Randomisation Number	Study Group	Dosage	Dose	Haematology Parameters	Unit	Day	Date of sample Collection	Results	Out of Range	Reference Range	Flag	Clinical Significance **
S125	102	JAIVAC-1	Dosage A	3	MCH	pg	Day 365	26/08/2011	31.3	Yes	27.0 - 31.0	H	NCS
S125	102	JAIVAC-1	Dosage A	3	MCHC	gm %	Day 365	26/08/2011	34.2	.	32.0 - 36.0		
S125	102	JAIVAC-1	Dosage A	3	MCV	fL	Day 365	26/08/2011	91.7	.	80.0- 96.0		
S125	102	JAIVAC-1	Dosage A	3	Platelet Count	10E3/ul	Day 365	26/08/2011	333	.	150 - 400		
S125	102	JAIVAC-1	Dosage A	3	RBC count	10E6/ul	Day 365	26/08/2011	5.66	.	4.60 - 6.20		
S125	102	JAIVAC-1	Dosage A	3	TLC	10E3/ul	Day 365	26/08/2011	6.2	.	4.50 - 11.00		
S130	105	JAIVAC-1	Dosage A		% Basophils	%	Day -14 to Day -1	17/08/2010	0.5	.	0.0 - 1.0		
S130	105	JAIVAC-1	Dosage A		% Eosinophils	%	Day -14 to Day -1	17/08/2010	6.9	.	1.0 - 6.0	H	
S130	105	JAIVAC-1	Dosage A		% Lymphocytes	%	Day -14 to Day -1	17/08/2010	30.6	.	20.0 - 45.0		
S130	105	JAIVAC-1	Dosage A		% Monocytes	%	Day -14 to Day -1	17/08/2010	6.3	.	2.0 - 10.0		
S130	105	JAIVAC-1	Dosage A		% Neutrophils	%	Day -14 to Day -1	17/08/2010	55.7	.	40.0 - 75.0		
S130	105	JAIVAC-1	Dosage A		Haematocrit	%	Day -14 to Day -1	17/08/2010	45.6	.	40.0 - 54.0		
S130	105	JAIVAC-1	Dosage A		Haemoglobin	gm / dl	Day -14 to Day -1	17/08/2010	15.2	.	13.5 - 18.0		
S130	105	JAIVAC-1	Dosage A		MCH	pg	Day -14 to Day -1	17/08/2010	28.4	.	27.0 - 31.0		
S130	105	JAIVAC-1	Dosage A		MCHC	gm %	Day -14 to Day -1	17/08/2010	33.2	.	32.0 - 36.0		
S130	105	JAIVAC-1	Dosage A		MCV	fL	Day -14 to Day -1	17/08/2010	85.4	.	80.0- 96.0		
S130	105	JAIVAC-1	Dosage A		Platelet Count	10E3/ul	Day -14 to Day -1	17/08/2010	225	.	150 - 400		
S130	105	JAIVAC-1	Dosage A		RBC count	10E6/ul	Day -14 to Day -1	17/08/2010	5.34	.	4.60 - 6.20		
S130	105	JAIVAC-1	Dosage A		TLC	10E3/ul	Day -14 to Day -1	17/08/2010	5.68	.	4.50 - 11.00		
S130	105	JAIVAC-1	Dosage A	2	% Basophils	%	Day 28	23/09/2010	0.6	.	0.0 - 1.0		
S130	105	JAIVAC-1	Dosage A	2	% Eosinophils	%	Day 28	23/09/2010	5.5	.	1.0 - 6.0		
S130	105	JAIVAC-1	Dosage A	2	% Lymphocytes	%	Day 28	23/09/2010	33.9	.	20.0 - 45.0		
S130	105	JAIVAC-1	Dosage A	2	% Monocytes	%	Day 28	23/09/2010	7.5	.	2.0 - 10.0		
S130	105	JAIVAC-1	Dosage A	2	% Neutrophils	%	Day 28	23/09/2010	52.5	.	40.0 - 75.0		
S130	105	JAIVAC-1	Dosage A	2	Haematocrit	%	Day 28	23/09/2010	44.4	.	40.0 - 54.0		
S130	105	JAIVAC-1	Dosage A	2	Haemoglobin	gm / dl	Day 28	23/09/2010	14.8	.	13.5 - 18.0		
S130	105	JAIVAC-1	Dosage A	2	MCH	pg	Day 28	23/09/2010	29	.	27.0 - 31.0		
S130	105	JAIVAC-1	Dosage A	2	MCHC	gm %	Day 28	23/09/2010	33.4	.	32.0 - 36.0		
S130	105	JAIVAC-1	Dosage A	2	MCV	fL	Day 28	23/09/2010	86.8	.	80.0- 96.0		

Subject No.	Randomisation Number	Study Group	Dosage	Dose	Haematology Parameters	Unit	Day	Date of sample Collection	Results	Out of Range	Reference Range	Flag	Clinical Significance **
S130	105	JAIVAC-1	Dosage A	2	Platelet Count	10E3/ul	Day 28	23/09/2010	200	.	150 - 400		
S130	105	JAIVAC-1	Dosage A	2	RBC count	10E6/ul	Day 28	23/09/2010	5.12	.	4.60 - 6.20		
S130	105	JAIVAC-1	Dosage A	2	TLC	10E3/ul	Day 28	23/09/2010	4.93	.	4.50 - 11.00		
S130	105	JAIVAC-1	Dosage A	2	% Basophils	%	Day 56	21/10/2010	0.8	.	0.0 - 1.0		
S130	105	JAIVAC-1	Dosage A	2	% Eosinophils	%	Day 56	21/10/2010	1.7	.	1.0 - 6.0		
S130	105	JAIVAC-1	Dosage A	2	% Lymphocytes	%	Day 56	21/10/2010	23.4	.	20.0 - 45.0		
S130	105	JAIVAC-1	Dosage A	2	% Monocytes	%	Day 56	21/10/2010	6.2	.	2.0 - 10.0		
S130	105	JAIVAC-1	Dosage A	2	% Neutrophils	%	Day 56	21/10/2010	67.9	.	40.0 - 75.0		
S130	105	JAIVAC-1	Dosage A	2	Haematocrit	%	Day 56	21/10/2010	43.3	.	40.0 - 54.0		
S130	105	JAIVAC-1	Dosage A	2	Haemoglobin	gm / dl	Day 56	21/10/2010	15.1	.	13.5 - 18.0		
S130	105	JAIVAC-1	Dosage A	2	MCH	pg	Day 56	21/10/2010	30	.	27.0 - 31.0		
S130	105	JAIVAC-1	Dosage A	2	MCHC	gm %	Day 56	21/10/2010	34.9	.	32.0 - 36.0		
S130	105	JAIVAC-1	Dosage A	2	MCV	fL	Day 56	21/10/2010	85.8	.	80.0- 96.0		
S130	105	JAIVAC-1	Dosage A	2	Platelet Count	10E3/ul	Day 56	21/10/2010	239	.	150 - 400		
S130	105	JAIVAC-1	Dosage A	2	RBC count	10E6/ul	Day 56	21/10/2010	5.04	.	4.60 - 6.20		
S130	105	JAIVAC-1	Dosage A	2	TLC	10E3/ul	Day 56	21/10/2010	4.62	.	4.50 - 11.00		
S130	105	JAIVAC-1	Dosage A	3	% Basophils	%	Day 180	22/02/2011	0.7	.	0.0 - 1.0		
S130	105	JAIVAC-1	Dosage A	3	% Eosinophils	%	Day 180	22/02/2011	2.9	.	1.0 - 6.0		
S130	105	JAIVAC-1	Dosage A	3	% Lymphocytes	%	Day 180	22/02/2011	24.3	.	20.0 - 45.0		
S130	105	JAIVAC-1	Dosage A	3	% Monocytes	%	Day 180	22/02/2011	6.7	.	2.0 - 10.0		
S130	105	JAIVAC-1	Dosage A	3	% Neutrophils	%	Day 180	22/02/2011	65.4	.	40.0 - 75.0		
S130	105	JAIVAC-1	Dosage A	3	Haematocrit	%	Day 180	22/02/2011	44.8	.	40.0 - 54.0		
S130	105	JAIVAC-1	Dosage A	3	Haemoglobin	gm / dl	Day 180	22/02/2011	15.2	.	13.5 - 18.0		
S130	105	JAIVAC-1	Dosage A	3	MCH	pg	Day 180	22/02/2011	29.1	.	27.0 - 31.0		
S130	105	JAIVAC-1	Dosage A	3	MCHC	gm %	Day 180	22/02/2011	34	.	32.0 - 36.0		
S130	105	JAIVAC-1	Dosage A	3	MCV	fL	Day 180	22/02/2011	85.7	.	80.0- 96.0		
S130	105	JAIVAC-1	Dosage A	3	Platelet Count	10E3/ul	Day 180	22/02/2011	254	.	150 - 400		
S130	105	JAIVAC-1	Dosage A	3	RBC count	10E6/ul	Day 180	22/02/2011	5.23	.	4.60 - 6.20		
S130	105	JAIVAC-1	Dosage A	3	TLC	10E3/ul	Day 180	22/02/2011	5.86	.	4.50 - 11.00		

Subject No.	Randomisation Number	Study Group	Dosage	Dose	Haematology Parameters	Unit	Day	Date of sample Collection	Results	Out of Range	Reference Range	Flag	Clinical Significance **
S130	105	JAIVAC-1	Dosage A	3	% Basophils	%	Day 208	22/03/2011	0.7	.	0.0 - 1.0		
S130	105	JAIVAC-1	Dosage A	3	% Eosinophils	%	Day 208	22/03/2011	1.6	.	1.0 - 6.0		
S130	105	JAIVAC-1	Dosage A	3	% Lymphocytes	%	Day 208	22/03/2011	23.8	.	20.0 - 45.0		
S130	105	JAIVAC-1	Dosage A	3	% Monocytes	%	Day 208	22/03/2011	7.4	.	2.0 - 10.0		
S130	105	JAIVAC-1	Dosage A	3	% Neutrophils	%	Day 208	22/03/2011	66.5	.	40.0 - 75.0		
S130	105	JAIVAC-1	Dosage A	3	Haematocrit	%	Day 208	22/03/2011	43.2	.	40.0 - 54.0		
S130	105	JAIVAC-1	Dosage A	3	Haemoglobin	gm / dl	Day 208	22/03/2011	14.8	.	13.5 - 18.0		
S130	105	JAIVAC-1	Dosage A	3	MCH	pg	Day 208	22/03/2011	29	.	27.0 - 31.0		
S130	105	JAIVAC-1	Dosage A	3	MCHC	gm %	Day 208	22/03/2011	34.2	.	32.0 - 36.0		
S130	105	JAIVAC-1	Dosage A	3	MCV	fL	Day 208	22/03/2011	84.7	.	80.0- 96.0		
S130	105	JAIVAC-1	Dosage A	3	Platelet Count	10E3/ul	Day 208	22/03/2011	227	.	150 - 400		
S130	105	JAIVAC-1	Dosage A	3	RBC count	10E6/ul	Day 208	22/03/2011	5.1	.	4.60 - 6.20		
S130	105	JAIVAC-1	Dosage A	3	TLC	10E3/ul	Day 208	22/03/2011	5.41	.	4.50 - 11.00		
S130	105	JAIVAC-1	Dosage A	3	% Basophils	%	Day 365	25/08/2011	0.9	.	0.0 - 1.0		
S130	105	JAIVAC-1	Dosage A	3	% Eosinophils	%	Day 365	25/08/2011	10.1	Yes	1.0 - 6.0	H	CS
S130	105	JAIVAC-1	Dosage A	3	% Lymphocytes	%	Day 365	25/08/2011	28	.	20.0 - 45.0		
S130	105	JAIVAC-1	Dosage A	3	% Monocytes	%	Day 365	25/08/2011	7.6	.	2.0 - 10.0		
S130	105	JAIVAC-1	Dosage A	3	% Neutrophils	%	Day 365	25/08/2011	53.5	.	40.0 - 75.0		
S130	105	JAIVAC-1	Dosage A	3	Haematocrit	%	Day 365	25/08/2011	45.1	.	40.0 - 54.0		
S130	105	JAIVAC-1	Dosage A	3	Haemoglobin	gm / dl	Day 365	25/08/2011	15.2	.	13.5 - 18.0		
S130	105	JAIVAC-1	Dosage A	3	MCH	pg	Day 365	25/08/2011	29.1	.	27.0 - 31.0		
S130	105	JAIVAC-1	Dosage A	3	MCHC	gm %	Day 365	25/08/2011	33.8	.	32.0 - 36.0		
S130	105	JAIVAC-1	Dosage A	3	MCV	fL	Day 365	25/08/2011	86	.	80.0- 96.0		
S130	105	JAIVAC-1	Dosage A	3	Platelet Count	10E3/ul	Day 365	25/08/2011	267	.	150 - 400		
S130	105	JAIVAC-1	Dosage A	3	RBC count	10E6/ul	Day 365	25/08/2011	5.24	.	4.60 - 6.20		
S130	105	JAIVAC-1	Dosage A	3	TLC	10E3/ul	Day 365	25/08/2011	6.69	.	4.50 - 11.00		
S132	108	Hepatitis B	Hepatitis B		% Basophils	%	Day -14 to Day -1	18/08/2010	0.9	.	0.0 - 1.0		
S132	108	Hepatitis B	Hepatitis B		% Eosinophils	%	Day -14 to Day -1	18/08/2010	5.1	.	1.0 - 6.0		
S132	108	Hepatitis B	Hepatitis B		% Lymphocytes	%	Day -14 to Day -1	18/08/2010	16.1	Yes	20.0 - 45.0	L	NCS

Subject No.	Randomisation Number	Study Group	Dosage	Dose	Haematology Parameters	Unit	Day	Date of sample Collection	Results	Out of Range	Reference Range	Flag	Clinical Significance **
S132	108	Hepatitis B	Hepatitis B		% Monocytes	%	Day -14 to Day -1	18/08/2010	4.1	.	2.0 - 10.0		
S132	108	Hepatitis B	Hepatitis B		% Neutrophils	%	Day -14 to Day -1	18/08/2010	73.8	.	40.0 - 75.0		
S132	108	Hepatitis B	Hepatitis B		Haematocrit	%	Day -14 to Day -1	18/08/2010	44.7	.	40.0 - 54.0		
S132	108	Hepatitis B	Hepatitis B		Haemoglobin	gm / dl	Day -14 to Day -1	18/08/2010	14.9	.	13.5 - 18.0		
S132	108	Hepatitis B	Hepatitis B		MCH	pg	Day -14 to Day -1	18/08/2010	29.8	.	27.0 - 31.0		
S132	108	Hepatitis B	Hepatitis B		MCHC	gm %	Day -14 to Day -1	18/08/2010	33.2	.	32.0 - 36.0		
S132	108	Hepatitis B	Hepatitis B		MCV	fL	Day -14 to Day -1	18/08/2010	89.6	.	80.0- 96.0		
S132	108	Hepatitis B	Hepatitis B		Platelet Count	10E3/ul	Day -14 to Day -1	18/08/2010	284	.	150 - 400		
S132	108	Hepatitis B	Hepatitis B		RBC count	10E6/ul	Day -14 to Day -1	18/08/2010	4.99	.	4.60 - 6.20		
S132	108	Hepatitis B	Hepatitis B		TLC	10E3/ul	Day -14 to Day -1	18/08/2010	6.66	.	4.50 - 11.00		
S132	108	Hepatitis B	Hepatitis B	2	% Basophils	%	Day 28	23/09/2010	1.6	Yes	0.0 - 1.0	H	NCS
S132	108	Hepatitis B	Hepatitis B	2	% Eosinophils	%	Day 28	23/09/2010	3.8	.	1.0 - 6.0		
S132	108	Hepatitis B	Hepatitis B	2	% Lymphocytes	%	Day 28	23/09/2010	26.2	.	20.0 - 45.0		
S132	108	Hepatitis B	Hepatitis B	2	% Monocytes	%	Day 28	23/09/2010	5.2	.	2.0 - 10.0		
S132	108	Hepatitis B	Hepatitis B	2	% Neutrophils	%	Day 28	23/09/2010	63.3	.	40.0 - 75.0		
S132	108	Hepatitis B	Hepatitis B	2	Haematocrit	%	Day 28	23/09/2010	45.2	.	40.0 - 54.0		
S132	108	Hepatitis B	Hepatitis B	2	Haemoglobin	gm / dl	Day 28	23/09/2010	15	.	13.5 - 18.0		
S132	108	Hepatitis B	Hepatitis B	2	MCH	pg	Day 28	23/09/2010	29.9	.	27.0 - 31.0		
S132	108	Hepatitis B	Hepatitis B	2	MCHC	gm %	Day 28	23/09/2010	33.1	.	32.0 - 36.0		
S132	108	Hepatitis B	Hepatitis B	2	MCV	fL	Day 28	23/09/2010	90.3	.	80.0- 96.0		
S132	108	Hepatitis B	Hepatitis B	2	Platelet Count	10E3/ul	Day 28	23/09/2010	358	.	150 - 400		
S132	108	Hepatitis B	Hepatitis B	2	RBC count	10E6/ul	Day 28	23/09/2010	5	.	4.60 - 6.20		
S132	108	Hepatitis B	Hepatitis B	2	TLC	10E3/ul	Day 28	23/09/2010	8.32	.	4.50 - 11.00		
S132	108	Hepatitis B	Hepatitis B	2	% Basophils	%	Day 56	21/10/2010	1.7	Yes	0.0 - 1.0	H	NCS
S132	108	Hepatitis B	Hepatitis B	2	% Eosinophils	%	Day 56	21/10/2010	3.5	.	1.0 - 6.0		
S132	108	Hepatitis B	Hepatitis B	2	% Lymphocytes	%	Day 56	21/10/2010	32	.	20.0 - 45.0		
S132	108	Hepatitis B	Hepatitis B	2	% Monocytes	%	Day 56	21/10/2010	5.5	.	2.0 - 10.0		
S132	108	Hepatitis B	Hepatitis B	2	% Neutrophils	%	Day 56	21/10/2010	57.4	.	40.0 - 75.0		
S132	108	Hepatitis B	Hepatitis B	2	Haematocrit	%	Day 56	21/10/2010	44.1	.	40.0 - 54.0		

Subject No.	Randomisation Number	Study Group	Dosage	Dose	Haematology Parameters	Unit	Day	Date of sample Collection	Results	Out of Range	Reference Range	Flag	Clinical Significance **
S132	108	Hepatitis B	Hepatitis B	2	Haemoglobin	gm / dl	Day 56	21/10/2010	14.8	.	13.5 - 18.0		
S132	108	Hepatitis B	Hepatitis B	2	MCH	pg	Day 56	21/10/2010	29.1	.	27.0 - 31.0		
S132	108	Hepatitis B	Hepatitis B	2	MCHC	gm %	Day 56	21/10/2010	33.7	.	32.0 - 36.0		
S132	108	Hepatitis B	Hepatitis B	2	MCV	fL	Day 56	21/10/2010	86.3	.	80.0- 96.0		
S132	108	Hepatitis B	Hepatitis B	2	Platelet Count	10E3/ul	Day 56	21/10/2010	339	.	150 - 400		
S132	108	Hepatitis B	Hepatitis B	2	RBC count	10E6/ul	Day 56	21/10/2010	5.11	.	4.60 - 6.20		
S132	108	Hepatitis B	Hepatitis B	2	TLC	10E3/ul	Day 56	21/10/2010	7.5	.	4.50 - 11.00		
S132	108	Hepatitis B	Hepatitis B	3	% Basophils	%	Day 180	22/02/2011	0.5	.	0.0 - 1.0		
S132	108	Hepatitis B	Hepatitis B	3	% Eosinophils	%	Day 180	22/02/2011	5.1	.	1.0 - 6.0		
S132	108	Hepatitis B	Hepatitis B	3	% Lymphocytes	%	Day 180	22/02/2011	28.9	.	20.0 - 45.0		
S132	108	Hepatitis B	Hepatitis B	3	% Monocytes	%	Day 180	22/02/2011	3.8	.	2.0 - 10.0		
S132	108	Hepatitis B	Hepatitis B	3	% Neutrophils	%	Day 180	22/02/2011	61.6	.	40.0 - 75.0		
S132	108	Hepatitis B	Hepatitis B	3	Haematocrit	%	Day 180	22/02/2011	46.8	.	40.0 - 54.0		
S132	108	Hepatitis B	Hepatitis B	3	Haemoglobin	gm / dl	Day 180	22/02/2011	15.4	.	13.5 - 18.0		
S132	108	Hepatitis B	Hepatitis B	3	MCH	pg	Day 180	22/02/2011	28.4	.	27.0 - 31.0		
S132	108	Hepatitis B	Hepatitis B	3	MCHC	gm %	Day 180	22/02/2011	32.9	.	32.0 - 36.0		
S132	108	Hepatitis B	Hepatitis B	3	MCV	fL	Day 180	22/02/2011	86.4	.	80.0- 96.0		
S132	108	Hepatitis B	Hepatitis B	3	Platelet Count	10E3/ul	Day 180	22/02/2011	345	.	150 - 400		
S132	108	Hepatitis B	Hepatitis B	3	RBC count	10E6/ul	Day 180	22/02/2011	5.42	.	4.60 - 6.20		
S132	108	Hepatitis B	Hepatitis B	3	TLC	10E3/ul	Day 180	22/02/2011	9.51	.	4.50 - 11.00		
S132	108	Hepatitis B	Hepatitis B	3	% Basophils	%	Day 208	22/03/2011	0	.	0.0 - 1.0		
S132	108	Hepatitis B	Hepatitis B	3	% Eosinophils	%	Day 208	22/03/2011	8	Yes	1.0 - 6.0	H	NCS
S132	108	Hepatitis B	Hepatitis B	3	% Lymphocytes	%	Day 208	22/03/2011	29	.	20.0 - 45.0		
S132	108	Hepatitis B	Hepatitis B	3	% Monocytes	%	Day 208	22/03/2011	3	.	2.0 - 10.0		
S132	108	Hepatitis B	Hepatitis B	3	% Neutrophils	%	Day 208	22/03/2011	60	.	40.0 - 75.0		
S132	108	Hepatitis B	Hepatitis B	3	Haematocrit	%	Day 208	22/03/2011	45.5	.	40.0 - 54.0		
S132	108	Hepatitis B	Hepatitis B	3	Haemoglobin	gm / dl	Day 208	22/03/2011	15.1	.	13.5 - 18.0		
S132	108	Hepatitis B	Hepatitis B	3	MCH	pg	Day 208	22/03/2011	28.3	.	27.0 - 31.0		
S132	108	Hepatitis B	Hepatitis B	3	MCHC	gm %	Day 208	22/03/2011	33.2	.	32.0 - 36.0		

Subject No.	Randomisation Number	Study Group	Dosage	Dose	Haematology Parameters	Unit	Day	Date of sample Collection	Results	Out of Range	Reference Range	Flag	Clinical Significance **
S132	108	Hepatitis B	Hepatitis B	3	MCV	fL	Day 208	22/03/2011	85.2	.	80.0- 96.0		
S132	108	Hepatitis B	Hepatitis B	3	Platelet Count	10E3/ul	Day 208	22/03/2011	298	.	150 - 400		
S132	108	Hepatitis B	Hepatitis B	3	RBC count	10E6/ul	Day 208	22/03/2011	5.34	.	4.60 - 6.20		
S132	108	Hepatitis B	Hepatitis B	3	TLC	10E3/ul	Day 208	22/03/2011	8.65	.	4.50 - 11.00		
S132	108	Hepatitis B	Hepatitis B	3	% Basophils	%	Day 365	25/08/2011	0.9	.	0.0 - 1.0		
S132	108	Hepatitis B	Hepatitis B	3	% Eosinophils	%	Day 365	25/08/2011	6.6	Yes	1.0 - 6.0	H	NCS
S132	108	Hepatitis B	Hepatitis B	3	% Lymphocytes	%	Day 365	25/08/2011	27.5	.	20.0 - 45.0		
S132	108	Hepatitis B	Hepatitis B	3	% Monocytes	%	Day 365	25/08/2011	4.7	.	2.0 - 10.0		
S132	108	Hepatitis B	Hepatitis B	3	% Neutrophils	%	Day 365	25/08/2011	60.4	.	40.0 - 75.0		
S132	108	Hepatitis B	Hepatitis B	3	Haematocrit	%	Day 365	25/08/2011	44.9	.	40.0 - 54.0		
S132	108	Hepatitis B	Hepatitis B	3	Haemoglobin	gm / dl	Day 365	25/08/2011	15.2	.	13.5 - 18.0		
S132	108	Hepatitis B	Hepatitis B	3	MCH	pg	Day 365	25/08/2011	29.5	.	27.0 - 31.0		
S132	108	Hepatitis B	Hepatitis B	3	MCHC	gm %	Day 365	25/08/2011	33.9	.	32.0 - 36.0		
S132	108	Hepatitis B	Hepatitis B	3	MCV	fL	Day 365	25/08/2011	86.9	.	80.0- 96.0		
S132	108	Hepatitis B	Hepatitis B	3	Platelet Count	10E3/ul	Day 365	25/08/2011	307	.	150 - 400		
S132	108	Hepatitis B	Hepatitis B	3	RBC count	10E6/ul	Day 365	25/08/2011	5.17	.	4.60 - 6.20		
S132	108	Hepatitis B	Hepatitis B	3	TLC	10E3/ul	Day 365	25/08/2011	8.79	.	4.50 - 11.00		
S136	109	JAIVAC-1	Dosage A		% Basophils	%	Day -14 to Day -1	19/08/2010	0.9	.	0.0 - 1.0		
S136	109	JAIVAC-1	Dosage A		% Eosinophils	%	Day -14 to Day -1	19/08/2010	6.4	.	1.0 - 6.0	H	
S136	109	JAIVAC-1	Dosage A		% Lymphocytes	%	Day -14 to Day -1	19/08/2010	44.9	.	20.0 - 45.0		
S136	109	JAIVAC-1	Dosage A		% Monocytes	%	Day -14 to Day -1	19/08/2010	3.3	.	2.0 - 10.0		
S136	109	JAIVAC-1	Dosage A		% Neutrophils	%	Day -14 to Day -1	19/08/2010	44.4	.	40.0 - 75.0		
S136	109	JAIVAC-1	Dosage A		Haematocrit	%	Day -14 to Day -1	19/08/2010	41.7	.	40.0 - 54.0		
S136	109	JAIVAC-1	Dosage A		Haemoglobin*	gm / dl	Day -14 to Day -1	19/08/2010	13.2	.	13.5 - 18.0	L	
S136	109	JAIVAC-1	Dosage A		MCH	pg	Day -14 to Day -1	19/08/2010	27	.	27.0 - 31.0		
S136	109	JAIVAC-1	Dosage A		MCHC	gm %	Day -14 to Day -1	19/08/2010	31.7	Yes	32.0 - 36.0	L	NCS
S136	109	JAIVAC-1	Dosage A		MCV	fL	Day -14 to Day -1	19/08/2010	85.2	.	80.0- 96.0		
S136	109	JAIVAC-1	Dosage A		Platelet Count	10E3/ul	Day -14 to Day -1	19/08/2010	275	.	150 - 400		
S136	109	JAIVAC-1	Dosage A		RBC count	10E6/ul	Day -14 to Day -1	19/08/2010	4.89	.	4.60 - 6.20		

Subject No.	Randomisation Number	Study Group	Dosage	Dose	Haematology Parameters	Unit	Day	Date of sample Collection	Results	Out of Range	Reference Range	Flag	Clinical Significance **
S136	109	JAIVAC-1	Dosage A		TLC	10E3/ul	Day -14 to Day -1	19/08/2010	6.43	.	4.50 - 11.00		
S136	109	JAIVAC-1	Dosage A	2	% Basophils	%	Day 28	23/09/2010	1.4	Yes	0.0 - 1.0	H	NCS
S136	109	JAIVAC-1	Dosage A	2	% Eosinophils	%	Day 28	23/09/2010	6.3	Yes	1.0 - 6.0	H	NCS
S136	109	JAIVAC-1	Dosage A	2	% Lymphocytes	%	Day 28	23/09/2010	43.4	.	20.0 - 45.0		
S136	109	JAIVAC-1	Dosage A	2	% Monocytes	%	Day 28	23/09/2010	5.4	.	2.0 - 10.0		
S136	109	JAIVAC-1	Dosage A	2	% Neutrophils	%	Day 28	23/09/2010	43.4	.	40.0 - 75.0		
S136	109	JAIVAC-1	Dosage A	2	Haematocrit	%	Day 28	23/09/2010	43.1	.	40.0 - 54.0		
S136	109	JAIVAC-1	Dosage A	2	Haemoglobin	gm / dl	Day 28	23/09/2010	14	.	13.5 - 18.0		
S136	109	JAIVAC-1	Dosage A	2	MCH	pg	Day 28	23/09/2010	28.4	.	27.0 - 31.0		
S136	109	JAIVAC-1	Dosage A	2	MCHC	gm %	Day 28	23/09/2010	32.4	.	32.0 - 36.0		
S136	109	JAIVAC-1	Dosage A	2	MCV	fL	Day 28	23/09/2010	87.7	.	80.0- 96.0		
S136	109	JAIVAC-1	Dosage A	2	Platelet Count	10E3/ul	Day 28	23/09/2010	278	.	150 - 400		
S136	109	JAIVAC-1	Dosage A	2	RBC count	10E6/ul	Day 28	23/09/2010	4.92	.	4.60 - 6.20		
S136	109	JAIVAC-1	Dosage A	2	TLC	10E3/ul	Day 28	23/09/2010	4.98	.	4.50 - 11.00		
S136	109	JAIVAC-1	Dosage A	2	% Basophils	%	Day 56	21/10/2010	0.5	.	0.0 - 1.0		
S136	109	JAIVAC-1	Dosage A	2	% Eosinophils	%	Day 56	21/10/2010	6.9	Yes	1.0 - 6.0	H	NCS
S136	109	JAIVAC-1	Dosage A	2	% Lymphocytes	%	Day 56	21/10/2010	38.9	.	20.0 - 45.0		
S136	109	JAIVAC-1	Dosage A	2	% Monocytes	%	Day 56	21/10/2010	4.2	.	2.0 - 10.0		
S136	109	JAIVAC-1	Dosage A	2	% Neutrophils	%	Day 56	21/10/2010	49.6	.	40.0 - 75.0		
S136	109	JAIVAC-1	Dosage A	2	Haematocrit	%	Day 56	21/10/2010	41.3	.	40.0 - 54.0		
S136	109	JAIVAC-1	Dosage A	2	Haemoglobin*	gm / dl	Day 56	21/10/2010	13.4	Yes	13.5 - 18.0	L	NCS
S136	109	JAIVAC-1	Dosage A	2	MCH	pg	Day 56	21/10/2010	28.3	.	27.0 - 31.0		
S136	109	JAIVAC-1	Dosage A	2	MCHC	gm %	Day 56	21/10/2010	32.4	.	32.0 - 36.0		
S136	109	JAIVAC-1	Dosage A	2	MCV	fL	Day 56	21/10/2010	87.2	.	80.0- 96.0		
S136	109	JAIVAC-1	Dosage A	2	Platelet Count	10E3/ul	Day 56	21/10/2010	325	.	150 - 400		
S136	109	JAIVAC-1	Dosage A	2	RBC count	10E6/ul	Day 56	21/10/2010	4.73	.	4.60 - 6.20		
S136	109	JAIVAC-1	Dosage A	2	TLC	10E3/ul	Day 56	21/10/2010	5.25	.	4.50 - 11.00		
S136	109	JAIVAC-1	Dosage A	3	% Basophils	%	Day 180	22/02/2011	0.7	.	0.0 - 1.0		
S136	109	JAIVAC-1	Dosage A	3	% Eosinophils	%	Day 180	22/02/2011	3.7	.	1.0 - 6.0		

Subject No.	Randomisation Number	Study Group	Dosage	Dose	Haematology Parameters	Unit	Day	Date of sample Collection	Results	Out of Range	Reference Range	Flag	Clinical Significance **
S136	109	JAIVAC-1	Dosage A	3	% Lymphocytes	%	Day 180	22/02/2011	33.5	.	20.0 - 45.0		
S136	109	JAIVAC-1	Dosage A	3	% Monocytes	%	Day 180	22/02/2011	4.7	.	2.0 - 10.0		
S136	109	JAIVAC-1	Dosage A	3	% Neutrophils	%	Day 180	22/02/2011	57.3	.	40.0 - 75.0		
S136	109	JAIVAC-1	Dosage A	3	Haematocrit	%	Day 180	22/02/2011	42.5	.	40.0 - 54.0		
S136	109	JAIVAC-1	Dosage A	3	Haemoglobin	gm / dl	Day 180	22/02/2011	14	.	13.5 - 18.0		
S136	109	JAIVAC-1	Dosage A	3	MCH	pg	Day 180	22/02/2011	30.1	.	27.0 - 31.0		
S136	109	JAIVAC-1	Dosage A	3	MCHC	gm %	Day 180	22/02/2011	33	.	32.0 - 36.0		
S136	109	JAIVAC-1	Dosage A	3	MCV	fL	Day 180	22/02/2011	91.1	.	80.0- 96.0		
S136	109	JAIVAC-1	Dosage A	3	Platelet Count	10E3/ul	Day 180	22/02/2011	255	.	150 - 400		
S136	109	JAIVAC-1	Dosage A	3	RBC count	10E6/ul	Day 180	22/02/2011	4.66	.	4.60 - 6.20		
S136	109	JAIVAC-1	Dosage A	3	TLC	10E3/ul	Day 180	22/02/2011	7.18	.	4.50 - 11.00		
S136	109	JAIVAC-1	Dosage A	3	% Basophils	%	Day 208	22/03/2011	1.4	Yes	0.0 - 1.0	H	NCS
S136	109	JAIVAC-1	Dosage A	3	% Eosinophils	%	Day 208	22/03/2011	6.2	Yes	1.0 - 6.0	H	NCS
S136	109	JAIVAC-1	Dosage A	3	% Lymphocytes	%	Day 208	22/03/2011	35.9	.	20.0 - 45.0		
S136	109	JAIVAC-1	Dosage A	3	% Monocytes	%	Day 208	22/03/2011	5.2	.	2.0 - 10.0		
S136	109	JAIVAC-1	Dosage A	3	% Neutrophils	%	Day 208	22/03/2011	51.3	.	40.0 - 75.0		
S136	109	JAIVAC-1	Dosage A	3	Haematocrit	%	Day 208	22/03/2011	42.7	.	40.0 - 54.0		
S136	109	JAIVAC-1	Dosage A	3	Haemoglobin	gm / dl	Day 208	22/03/2011	14.5	.	13.5 - 18.0		
S136	109	JAIVAC-1	Dosage A	3	MCH	pg	Day 208	22/03/2011	31.4	Yes	27.0 - 31.0	H	NCS
S136	109	JAIVAC-1	Dosage A	3	MCHC	gm %	Day 208	22/03/2011	34	.	32.0 - 36.0		
S136	109	JAIVAC-1	Dosage A	3	MCV	fL	Day 208	22/03/2011	92.4	.	80.0- 96.0		
S136	109	JAIVAC-1	Dosage A	3	Platelet Count	10E3/ul	Day 208	22/03/2011	270	.	150 - 400		
S136	109	JAIVAC-1	Dosage A	3	RBC count	10E6/ul	Day 208	22/03/2011	4.62	.	4.60 - 6.20		
S136	109	JAIVAC-1	Dosage A	3	TLC	10E3/ul	Day 208	22/03/2011	6.11	.	4.50 - 11.00		
S136	109	JAIVAC-1	Dosage A	3	% Basophils	%	Day 365	25/08/2011	1.1	Yes	0.0 - 1.0	H	NCS
S136	109	JAIVAC-1	Dosage A	3	% Eosinophils	%	Day 365	25/08/2011	6.6	Yes	1.0 - 6.0	H	NCS
S136	109	JAIVAC-1	Dosage A	3	% Lymphocytes	%	Day 365	25/08/2011	39.8	.	20.0 - 45.0		
S136	109	JAIVAC-1	Dosage A	3	% Monocytes	%	Day 365	25/08/2011	4.5	.	2.0 - 10.0		
S136	109	JAIVAC-1	Dosage A	3	% Neutrophils	%	Day 365	25/08/2011	47.9	.	40.0 - 75.0		

Subject No.	Randomisation Number	Study Group	Dosage	Dose	Haematology Parameters	Unit	Day	Date of sample Collection	Results	Out of Range	Reference Range	Flag	Clinical Significance **
S136	109	JAIVAC-1	Dosage A	3	Haematocrit	%	Day 365	25/08/2011	44.1	.	40.0 - 54.0		
S136	109	JAIVAC-1	Dosage A	3	Haemoglobin	gm / dl	Day 365	25/08/2011	14.9	.	13.5 - 18.0		
S136	109	JAIVAC-1	Dosage A	3	MCH	pg	Day 365	25/08/2011	31.9	Yes	27.0 - 31.0	H	NCS
S136	109	JAIVAC-1	Dosage A	3	MCHC	gm %	Day 365	25/08/2011	33.8	.	32.0 - 36.0		
S136	109	JAIVAC-1	Dosage A	3	MCV	fL	Day 365	25/08/2011	94.4	.	80.0- 96.0		
S136	109	JAIVAC-1	Dosage A	3	Platelet Count	10E3/ul	Day 365	25/08/2011	266	.	150 - 400		
S136	109	JAIVAC-1	Dosage A	3	RBC count	10E6/ul	Day 365	25/08/2011	4.67	.	4.60 - 6.20		
S136	109	JAIVAC-1	Dosage A	3	TLC	10E3/ul	Day 365	25/08/2011	6.29	.	4.50 - 11.00		
S143	101	JAIVAC-1	Dosage A		% Basophils	%	Day -14 to Day -1	18/08/2010	0.7	.	0.0 - 1.0		
S143	101	JAIVAC-1	Dosage A		% Eosinophils	%	Day -14 to Day -1	18/08/2010	4.9	.	1.0 - 6.0		
S143	101	JAIVAC-1	Dosage A		% Lymphocytes	%	Day -14 to Day -1	18/08/2010	36.6	.	20.0 - 45.0		
S143	101	JAIVAC-1	Dosage A		% Monocytes	%	Day -14 to Day -1	18/08/2010	4.4	.	2.0 - 10.0		
S143	101	JAIVAC-1	Dosage A		% Neutrophils	%	Day -14 to Day -1	18/08/2010	53.4	.	40.0 - 75.0		
S143	101	JAIVAC-1	Dosage A		Haematocrit	%	Day -14 to Day -1	18/08/2010	47.4	.	40.0 - 54.0		
S143	101	JAIVAC-1	Dosage A		Haemoglobin	gm / dl	Day -14 to Day -1	18/08/2010	15.3	.	13.5 - 18.0		
S143	101	JAIVAC-1	Dosage A		MCH	pg	Day -14 to Day -1	18/08/2010	27.2	.	27.0 - 31.0		
S143	101	JAIVAC-1	Dosage A		MCHC	gm %	Day -14 to Day -1	18/08/2010	32.3	.	32.0 - 36.0		
S143	101	JAIVAC-1	Dosage A		MCV	fL	Day -14 to Day -1	18/08/2010	84.4	.	80.0- 96.0		
S143	101	JAIVAC-1	Dosage A		Platelet Count	10E3/ul	Day -14 to Day -1	18/08/2010	268	.	150 - 400		
S143	101	JAIVAC-1	Dosage A		RBC count	10E6/ul	Day -14 to Day -1	18/08/2010	5.61	.	4.60 - 6.20		
S143	101	JAIVAC-1	Dosage A		TLC	10E3/ul	Day -14 to Day -1	18/08/2010	5.38	.	4.50 - 11.00		
S143	101	JAIVAC-1	Dosage A	2	% Basophils	%	Day 28	21/09/2010	1.8	Yes	0.0 - 1.0	H	NCS
S143	101	JAIVAC-1	Dosage A	2	% Eosinophils	%	Day 28	21/09/2010	3.8	.	1.0 - 6.0		
S143	101	JAIVAC-1	Dosage A	2	% Lymphocytes	%	Day 28	21/09/2010	34.6	.	20.0 - 45.0		
S143	101	JAIVAC-1	Dosage A	2	% Monocytes	%	Day 28	21/09/2010	7.1	.	2.0 - 10.0		
S143	101	JAIVAC-1	Dosage A	2	% Neutrophils	%	Day 28	21/09/2010	52.7	.	40.0 - 75.0		
S143	101	JAIVAC-1	Dosage A	2	Haematocrit	%	Day 28	21/09/2010	46.1	.	40.0 - 54.0		
S143	101	JAIVAC-1	Dosage A	2	Haemoglobin	gm / dl	Day 28	21/09/2010	15.3	.	13.5 - 18.0		
S143	101	JAIVAC-1	Dosage A	2	MCH	pg	Day 28	21/09/2010	28.6	.	27.0 - 31.0		

Subject No.	Randomisation Number	Study Group	Dosage	Dose	Haematology Parameters	Unit	Day	Date of sample Collection	Results	Out of Range	Reference Range	Flag	Clinical Significance **
S143	101	JAIVAC-1	Dosage A	2	MCHC	gm %	Day 28	21/09/2010	33.1	.	32.0 - 36.0		
S143	101	JAIVAC-1	Dosage A	2	MCV	fL	Day 28	21/09/2010	86.3	.	80.0- 96.0		
S143	101	JAIVAC-1	Dosage A	2	Platelet Count	10E3/ul	Day 28	21/09/2010	259	.	150 - 400		
S143	101	JAIVAC-1	Dosage A	2	RBC count	10E6/ul	Day 28	21/09/2010	5.34	.	4.60 - 6.20		
S143	101	JAIVAC-1	Dosage A	2	TLC	10E3/ul	Day 28	21/09/2010	4	Yes	4.50 - 11.00	L	NCS
S143	101	JAIVAC-1	Dosage A	2	% Basophils	%	Day 56	19/10/2010	1.3	Yes	0.0 - 1.0	H	NCS
S143	101	JAIVAC-1	Dosage A	2	% Eosinophils	%	Day 56	19/10/2010	4.9	.	1.0 - 6.0		
S143	101	JAIVAC-1	Dosage A	2	% Lymphocytes	%	Day 56	19/10/2010	32.1	.	20.0 - 45.0		
S143	101	JAIVAC-1	Dosage A	2	% Monocytes	%	Day 56	19/10/2010	5.2	.	2.0 - 10.0		
S143	101	JAIVAC-1	Dosage A	2	% Neutrophils	%	Day 56	19/10/2010	56.5	.	40.0 - 75.0		
S143	101	JAIVAC-1	Dosage A	2	Haematocrit	%	Day 56	19/10/2010	47.3	.	40.0 - 54.0		
S143	101	JAIVAC-1	Dosage A	2	Haemoglobin	gm / dl	Day 56	19/10/2010	15.8	.	13.5 - 18.0		
S143	101	JAIVAC-1	Dosage A	2	MCH	pg	Day 56	19/10/2010	29	.	27.0 - 31.0		
S143	101	JAIVAC-1	Dosage A	2	MCHC	gm %	Day 56	19/10/2010	33.5	.	32.0 - 36.0		
S143	101	JAIVAC-1	Dosage A	2	MCV	fL	Day 56	19/10/2010	86.8	.	80.0- 96.0		
S143	101	JAIVAC-1	Dosage A	2	Platelet Count	10E3/ul	Day 56	19/10/2010	290	.	150 - 400		
S143	101	JAIVAC-1	Dosage A	2	RBC count	10E6/ul	Day 56	19/10/2010	5.45	.	4.60 - 6.20		
S143	101	JAIVAC-1	Dosage A	2	TLC	10E3/ul	Day 56	19/10/2010	4.74	.	4.50 - 11.00		
S143	101	JAIVAC-1	Dosage A	3	% Basophils	%	Day 180	21/02/2011	1.4	Yes	0.0 - 1.0	H	NCS
S143	101	JAIVAC-1	Dosage A	3	% Eosinophils	%	Day 180	21/02/2011	2.4	.	1.0 - 6.0		
S143	101	JAIVAC-1	Dosage A	3	% Lymphocytes	%	Day 180	21/02/2011	16.8	Yes	20.0 - 45.0	L	NCS
S143	101	JAIVAC-1	Dosage A	3	% Monocytes	%	Day 180	21/02/2011	6	.	2.0 - 10.0		
S143	101	JAIVAC-1	Dosage A	3	% Neutrophils	%	Day 180	21/02/2011	73.3	.	40.0 - 75.0		
S143	101	JAIVAC-1	Dosage A	3	Haematocrit	%	Day 180	21/02/2011	48.5	.	40.0 - 54.0		
S143	101	JAIVAC-1	Dosage A	3	Haemoglobin	gm / dl	Day 180	21/02/2011	16	.	13.5 - 18.0		
S143	101	JAIVAC-1	Dosage A	3	MCH	pg	Day 180	21/02/2011	28.3	.	27.0 - 31.0		
S143	101	JAIVAC-1	Dosage A	3	MCHC	gm %	Day 180	21/02/2011	32.9	.	32.0 - 36.0		
S143	101	JAIVAC-1	Dosage A	3	MCV	fL	Day 180	21/02/2011	86.1	.	80.0- 96.0		
S143	101	JAIVAC-1	Dosage A	3	Platelet Count	10E3/ul	Day 180	21/02/2011	302	.	150 - 400		

Subject No.	Randomisation Number	Study Group	Dosage	Dose	Haematology Parameters	Unit	Day	Date of sample Collection	Results	Out of Range	Reference Range	Flag	Clinical Significance **
S143	101	JAIVAC-1	Dosage A	3	RBC count	10E6/ul	Day 180	21/02/2011	5.63	.	4.60 - 6.20		
S143	101	JAIVAC-1	Dosage A	3	TLC	10E3/ul	Day 180	21/02/2011	7.79	.	4.50 - 11.00		
S143	101	JAIVAC-1	Dosage A	3	% Basophils	%	Day 208	21/03/2011	1.2	Yes	0.0 - 1.0	H	NCS
S143	101	JAIVAC-1	Dosage A	3	% Eosinophils	%	Day 208	21/03/2011	3.7	.	1.0 - 6.0		
S143	101	JAIVAC-1	Dosage A	3	% Lymphocytes	%	Day 208	21/03/2011	33.1	.	20.0 - 45.0		
S143	101	JAIVAC-1	Dosage A	3	% Monocytes	%	Day 208	21/03/2011	6.3	.	2.0 - 10.0		
S143	101	JAIVAC-1	Dosage A	3	% Neutrophils	%	Day 208	21/03/2011	55.7	.	40.0 - 75.0		
S143	101	JAIVAC-1	Dosage A	3	Haematocrit	%	Day 208	21/03/2011	48.8	.	40.0 - 54.0		
S143	101	JAIVAC-1	Dosage A	3	Haemoglobin	gm / dl	Day 208	21/03/2011	16.1	.	13.5 - 18.0		
S143	101	JAIVAC-1	Dosage A	3	MCH	pg	Day 208	21/03/2011	28.9	.	27.0 - 31.0		
S143	101	JAIVAC-1	Dosage A	3	MCHC	gm %	Day 208	21/03/2011	33	.	32.0 - 36.0		
S143	101	JAIVAC-1	Dosage A	3	MCV	fL	Day 208	21/03/2011	87.5	.	80.0- 96.0		
S143	101	JAIVAC-1	Dosage A	3	Platelet Count	10E3/ul	Day 208	21/03/2011	270	.	150 - 400		
S143	101	JAIVAC-1	Dosage A	3	RBC count	10E6/ul	Day 208	21/03/2011	5.58	.	4.60 - 6.20		
S143	101	JAIVAC-1	Dosage A	3	TLC	10E3/ul	Day 208	21/03/2011	4.34	Yes	4.50 - 11.00	L	NCS
S143	101	JAIVAC-1	Dosage A	3	% Basophils	%	Day 365	26/08/2011	1.5	Yes	0.0 - 1.0	H	NCS
S143	101	JAIVAC-1	Dosage A	3	% Eosinophils	%	Day 365	26/08/2011	2.6	.	1.0 - 6.0		
S143	101	JAIVAC-1	Dosage A	3	% Lymphocytes	%	Day 365	26/08/2011	35.1	.	20.0 - 45.0		
S143	101	JAIVAC-1	Dosage A	3	% Monocytes	%	Day 365	26/08/2011	7.4	.	2.0 - 10.0		
S143	101	JAIVAC-1	Dosage A	3	% Neutrophils	%	Day 365	26/08/2011	53.4	.	40.0 - 75.0		
S143	101	JAIVAC-1	Dosage A	3	Haematocrit	%	Day 365	26/08/2011	51.1	.	40.0 - 54.0		
S143	101	JAIVAC-1	Dosage A	3	Haemoglobin	gm / dl	Day 365	26/08/2011	17.4	.	13.5 - 18.0		
S143	101	JAIVAC-1	Dosage A	3	MCH	pg	Day 365	26/08/2011	30.1	.	27.0 - 31.0		
S143	101	JAIVAC-1	Dosage A	3	MCHC	gm %	Day 365	26/08/2011	34	.	32.0 - 36.0		
S143	101	JAIVAC-1	Dosage A	3	MCV	fL	Day 365	26/08/2011	88.6	.	80.0- 96.0		
S143	101	JAIVAC-1	Dosage A	3	Platelet Count	10E3/ul	Day 365	26/08/2011	305	.	150 - 400		
S143	101	JAIVAC-1	Dosage A	3	RBC count	10E6/ul	Day 365	26/08/2011	5.77	.	4.60 - 6.20		
S143	101	JAIVAC-1	Dosage A	3	TLC	10E3/ul	Day 365	26/08/2011	5.66	.	4.50 - 11.00		
S144	103	Hepatitis B	Hepatitis B		% Basophils	%	Day -14 to Day -1	19/08/2010	1.3	Yes	0.0 - 1.0	H	NCS

Subject No.	Randomisation Number	Study Group	Dosage	Dose	Haematology Parameters	Unit	Day	Date of sample Collection	Results	Out of Range	Reference Range	Flag	Clinical Significance **
S144	103	Hepatitis B	Hepatitis B		% Eosinophils	%	Day -14 to Day -1	19/08/2010	1.8	.	1.0 - 6.0		
S144	103	Hepatitis B	Hepatitis B		% Lymphocytes	%	Day -14 to Day -1	19/08/2010	31.6	.	20.0 - 45.0		
S144	103	Hepatitis B	Hepatitis B		% Monocytes	%	Day -14 to Day -1	19/08/2010	4.2	.	2.0 - 10.0		
S144	103	Hepatitis B	Hepatitis B		% Neutrophils	%	Day -14 to Day -1	19/08/2010	61.3	.	40.0 - 75.0		
S144	103	Hepatitis B	Hepatitis B		Haematocrit	%	Day -14 to Day -1	19/08/2010	49.5	.	40.0 - 54.0		
S144	103	Hepatitis B	Hepatitis B		Haemoglobin	gm / dl	Day -14 to Day -1	19/08/2010	16.4	.	13.5 - 18.0		
S144	103	Hepatitis B	Hepatitis B		MCH	pg	Day -14 to Day -1	19/08/2010	28.1	.	27.0 - 31.0		
S144	103	Hepatitis B	Hepatitis B		MCHC	gm %	Day -14 to Day -1	19/08/2010	33	.	32.0 - 36.0		
S144	103	Hepatitis B	Hepatitis B		MCV	fL	Day -14 to Day -1	19/08/2010	85.1	.	80.0- 96.0		
S144	103	Hepatitis B	Hepatitis B		Platelet Count	10E3/ul	Day -14 to Day -1	19/08/2010	322	.	150 - 400		
S144	103	Hepatitis B	Hepatitis B		RBC count	10E6/ul	Day -14 to Day -1	19/08/2010	5.82	.	4.60 - 6.20		
S144	103	Hepatitis B	Hepatitis B		TLC	10E3/ul	Day -14 to Day -1	19/08/2010	6.97	.	4.50 - 11.00		
S144	103	Hepatitis B	Hepatitis B	2	% Basophils	%	Day 28	21/09/2010	0.7	.	0.0 - 1.0		
S144	103	Hepatitis B	Hepatitis B	2	% Eosinophils	%	Day 28	21/09/2010	3.4	.	1.0 - 6.0		
S144	103	Hepatitis B	Hepatitis B	2	% Lymphocytes	%	Day 28	21/09/2010	20.8	.	20.0 - 45.0		
S144	103	Hepatitis B	Hepatitis B	2	% Monocytes	%	Day 28	21/09/2010	5.2	.	2.0 - 10.0		
S144	103	Hepatitis B	Hepatitis B	2	% Neutrophils	%	Day 28	21/09/2010	69.9	.	40.0 - 75.0		
S144	103	Hepatitis B	Hepatitis B	2	Haematocrit	%	Day 28	21/09/2010	44.4	.	40.0 - 54.0		
S144	103	Hepatitis B	Hepatitis B	2	Haemoglobin	gm / dl	Day 28	21/09/2010	14.8	.	13.5 - 18.0		
S144	103	Hepatitis B	Hepatitis B	2	MCH	pg	Day 28	21/09/2010	28.8	.	27.0 - 31.0		
S144	103	Hepatitis B	Hepatitis B	2	MCHC	gm %	Day 28	21/09/2010	33.3	.	32.0 - 36.0		
S144	103	Hepatitis B	Hepatitis B	2	MCV	fL	Day 28	21/09/2010	86.7	.	80.0- 96.0		
S144	103	Hepatitis B	Hepatitis B	2	Platelet Count	10E3/ul	Day 28	21/09/2010	225	.	150 - 400		
S144	103	Hepatitis B	Hepatitis B	2	RBC count	10E6/ul	Day 28	21/09/2010	5.12	.	4.60 - 6.20		
S144	103	Hepatitis B	Hepatitis B	2	TLC	10E3/ul	Day 28	21/09/2010	4.94	.	4.50 - 11.00		
S144	103	Hepatitis B	Hepatitis B	2	% Basophils	%	Day 56	19/10/2010	0.7	.	0.0 - 1.0		
S144	103	Hepatitis B	Hepatitis B	2	% Eosinophils	%	Day 56	19/10/2010	1	.	1.0 - 6.0		
S144	103	Hepatitis B	Hepatitis B	2	% Lymphocytes	%	Day 56	19/10/2010	27	.	20.0 - 45.0		
S144	103	Hepatitis B	Hepatitis B	2	% Monocytes	%	Day 56	19/10/2010	5.3	.	2.0 - 10.0		

Subject No.	Randomisation Number	Study Group	Dosage	Dose	Haematology Parameters	Unit	Day	Date of sample Collection	Results	Out of Range	Reference Range	Flag	Clinical Significance **
S144	103	Hepatitis B	Hepatitis B	2	% Neutrophils	%	Day 56	19/10/2010	66.1	.	40.0 - 75.0		
S144	103	Hepatitis B	Hepatitis B	2	Haematocrit	%	Day 56	19/10/2010	46.8	.	40.0 - 54.0		
S144	103	Hepatitis B	Hepatitis B	2	Haemoglobin	gm / dl	Day 56	19/10/2010	15.5	.	13.5 - 18.0		
S144	103	Hepatitis B	Hepatitis B	2	MCH	pg	Day 56	19/10/2010	28.9	.	27.0 - 31.0		
S144	103	Hepatitis B	Hepatitis B	2	MCHC	gm %	Day 56	19/10/2010	33	.	32.0 - 36.0		
S144	103	Hepatitis B	Hepatitis B	2	MCV	fL	Day 56	19/10/2010	87.4	.	80.0- 96.0		
S144	103	Hepatitis B	Hepatitis B	2	Platelet Count	10E3/ul	Day 56	19/10/2010	240	.	150 - 400		
S144	103	Hepatitis B	Hepatitis B	2	RBC count	10E6/ul	Day 56	19/10/2010	5.36	.	4.60 - 6.20		
S144	103	Hepatitis B	Hepatitis B	2	TLC	10E3/ul	Day 56	19/10/2010	5.4	.	4.50 - 11.00		
S144	103	Hepatitis B	Hepatitis B	3	% Basophils	%	Day 180	21/02/2011	0.9	.	0.0 - 1.0		
S144	103	Hepatitis B	Hepatitis B	3	% Eosinophils	%	Day 180	21/02/2011	1.2	.	1.0 - 6.0		
S144	103	Hepatitis B	Hepatitis B	3	% Lymphocytes	%	Day 180	21/02/2011	30.8	.	20.0 - 45.0		
S144	103	Hepatitis B	Hepatitis B	3	% Monocytes	%	Day 180	21/02/2011	5.6	.	2.0 - 10.0		
S144	103	Hepatitis B	Hepatitis B	3	% Neutrophils	%	Day 180	21/02/2011	61.5	.	40.0 - 75.0		
S144	103	Hepatitis B	Hepatitis B	3	Haematocrit	%	Day 180	21/02/2011	48	.	40.0 - 54.0		
S144	103	Hepatitis B	Hepatitis B	3	Haemoglobin	gm / dl	Day 180	21/02/2011	15.9	.	13.5 - 18.0		
S144	103	Hepatitis B	Hepatitis B	3	MCH	pg	Day 180	21/02/2011	27.9	.	27.0 - 31.0		
S144	103	Hepatitis B	Hepatitis B	3	MCHC	gm %	Day 180	21/02/2011	33.2	.	32.0 - 36.0		
S144	103	Hepatitis B	Hepatitis B	3	MCV	fL	Day 180	21/02/2011	84.1	.	80.0- 96.0		
S144	103	Hepatitis B	Hepatitis B	3	Platelet Count	10E3/ul	Day 180	21/02/2011	245	.	150 - 400		
S144	103	Hepatitis B	Hepatitis B	3	RBC count	10E6/ul	Day 180	21/02/2011	5.71	.	4.60 - 6.20		
S144	103	Hepatitis B	Hepatitis B	3	TLC	10E3/ul	Day 180	21/02/2011	4.78	.	4.50 - 11.00		
S144	103	Hepatitis B	Hepatitis B	3	% Basophils	%	Day 208	21/03/2011	0.7	.	0.0 - 1.0		
S144	103	Hepatitis B	Hepatitis B	3	% Eosinophils	%	Day 208	21/03/2011	1.9	.	1.0 - 6.0		
S144	103	Hepatitis B	Hepatitis B	3	% Lymphocytes	%	Day 208	21/03/2011	31.7	.	20.0 - 45.0		
S144	103	Hepatitis B	Hepatitis B	3	% Monocytes	%	Day 208	21/03/2011	6.1	.	2.0 - 10.0		
S144	103	Hepatitis B	Hepatitis B	3	% Neutrophils	%	Day 208	21/03/2011	59.6	.	40.0 - 75.0		
S144	103	Hepatitis B	Hepatitis B	3	Haematocrit	%	Day 208	21/03/2011	46.2	.	40.0 - 54.0		
S144	103	Hepatitis B	Hepatitis B	3	Haemoglobin	gm / dl	Day 208	21/03/2011	16	.	13.5 - 18.0		

Subject No.	Randomisation Number	Study Group	Dosage	Dose	Haematology Parameters	Unit	Day	Date of sample Collection	Results	Out of Range	Reference Range	Flag	Clinical Significance **
S144	103	Hepatitis B	Hepatitis B	3	MCH	pg	Day 208	21/03/2011	28.9	.	27.0 - 31.0		
S144	103	Hepatitis B	Hepatitis B	3	MCHC	gm %	Day 208	21/03/2011	34.6	.	32.0 - 36.0		
S144	103	Hepatitis B	Hepatitis B	3	MCV	fL	Day 208	21/03/2011	83.6	.	80.0- 96.0		
S144	103	Hepatitis B	Hepatitis B	3	Platelet Count	10E3/ul	Day 208	21/03/2011	232	.	150 - 400		
S144	103	Hepatitis B	Hepatitis B	3	RBC count	10E6/ul	Day 208	21/03/2011	5.53	.	4.60 - 6.20		
S144	103	Hepatitis B	Hepatitis B	3	TLC	10E3/ul	Day 208	21/03/2011	4.81	.	4.50 - 11.00		
S144	103	Hepatitis B	Hepatitis B	3	% Basophils	%	Day 365	26/08/2011	1.4	Yes	0.0 - 1.0	H	NCS
S144	103	Hepatitis B	Hepatitis B	3	% Eosinophils	%	Day 365	26/08/2011	1.4	.	1.0 - 6.0		
S144	103	Hepatitis B	Hepatitis B	3	% Lymphocytes	%	Day 365	26/08/2011	34.7	.	20.0 - 45.0		
S144	103	Hepatitis B	Hepatitis B	3	% Monocytes	%	Day 365	26/08/2011	5.7	.	2.0 - 10.0		
S144	103	Hepatitis B	Hepatitis B	3	% Neutrophils	%	Day 365	26/08/2011	56.9	.	40.0 - 75.0		
S144	103	Hepatitis B	Hepatitis B	3	Haematocrit	%	Day 365	26/08/2011	49.8	.	40.0 - 54.0		
S144	103	Hepatitis B	Hepatitis B	3	Haemoglobin	gm / dl	Day 365	26/08/2011	16.7	.	13.5 - 18.0		
S144	103	Hepatitis B	Hepatitis B	3	MCH	pg	Day 365	26/08/2011	28.6	.	27.0 - 31.0		
S144	103	Hepatitis B	Hepatitis B	3	MCHC	gm %	Day 365	26/08/2011	33.6	.	32.0 - 36.0		
S144	103	Hepatitis B	Hepatitis B	3	MCV	fL	Day 365	26/08/2011	85.1	.	80.0- 96.0		
S144	103	Hepatitis B	Hepatitis B	3	Platelet Count	10E3/ul	Day 365	26/08/2011	270	.	150 - 400		
S144	103	Hepatitis B	Hepatitis B	3	RBC count	10E6/ul	Day 365	26/08/2011	5.85	.	4.60 - 6.20		
S144	103	Hepatitis B	Hepatitis B	3	TLC	10E3/ul	Day 365	26/08/2011	5.12	.	4.50 - 11.00		
S145	112	JAIVAC-1	Dosage A		% Basophils	%	Day -14 to Day -1	19/08/2010	1	.	0.0 - 1.0		
S145	112	JAIVAC-1	Dosage A		% Eosinophils	%	Day -14 to Day -1	19/08/2010	2.1	.	1.0 - 6.0		
S145	112	JAIVAC-1	Dosage A		% Lymphocytes	%	Day -14 to Day -1	19/08/2010	31	.	20.0 - 45.0		
S145	112	JAIVAC-1	Dosage A		% Monocytes	%	Day -14 to Day -1	19/08/2010	4	.	2.0 - 10.0		
S145	112	JAIVAC-1	Dosage A		% Neutrophils	%	Day -14 to Day -1	19/08/2010	62	.	40.0 - 75.0		
S145	112	JAIVAC-1	Dosage A		Haematocrit	%	Day -14 to Day -1	19/08/2010	49.3	.	40.0 - 54.0		
S145	112	JAIVAC-1	Dosage A		Haemoglobin	gm / dl	Day -14 to Day -1	19/08/2010	16.1	.	13.5 - 18.0		
S145	112	JAIVAC-1	Dosage A		MCH	pg	Day -14 to Day -1	19/08/2010	29.9	.	27.0 - 31.0		
S145	112	JAIVAC-1	Dosage A		MCHC	gm %	Day -14 to Day -1	19/08/2010	32.7	.	32.0 - 36.0		
S145	112	JAIVAC-1	Dosage A		MCV	fL	Day -14 to Day -1	19/08/2010	91.4	.	80.0- 96.0		

Subject No.	Randomisation Number	Study Group	Dosage	Dose	Haematology Parameters	Unit	Day	Date of sample Collection	Results	Out of Range	Reference Range	Flag	Clinical Significance **
S145	112	JAIVAC-1	Dosage A		Platelet Count	10E3/ul	Day -14 to Day -1	19/08/2010	225	.	150 - 400		
S145	112	JAIVAC-1	Dosage A		RBC count	10E6/ul	Day -14 to Day -1	19/08/2010	5.39	.	4.60 - 6.20		
S145	112	JAIVAC-1	Dosage A		TLC	10E3/ul	Day -14 to Day -1	19/08/2010	7.25	.	4.50 - 11.00		
S145	112	JAIVAC-1	Dosage A	2	% Basophils	%	Day 28	24/09/2010	0.9	.	0.0 - 1.0		
S145	112	JAIVAC-1	Dosage A	2	% Eosinophils	%	Day 28	24/09/2010	1.1	.	1.0 - 6.0		
S145	112	JAIVAC-1	Dosage A	2	% Lymphocytes	%	Day 28	24/09/2010	26.6	.	20.0 - 45.0		
S145	112	JAIVAC-1	Dosage A	2	% Monocytes	%	Day 28	24/09/2010	5.9	.	2.0 - 10.0		
S145	112	JAIVAC-1	Dosage A	2	% Neutrophils	%	Day 28	24/09/2010	65.4	.	40.0 - 75.0		
S145	112	JAIVAC-1	Dosage A	2	Haematocrit	%	Day 28	24/09/2010	49.2	.	40.0 - 54.0		
S145	112	JAIVAC-1	Dosage A	2	Haemoglobin	gm / dl	Day 28	24/09/2010	16.4	.	13.5 - 18.0		
S145	112	JAIVAC-1	Dosage A	2	MCH	pg	Day 28	24/09/2010	30.8	.	27.0 - 31.0		
S145	112	JAIVAC-1	Dosage A	2	MCHC	gm %	Day 28	24/09/2010	33.3	.	32.0 - 36.0		
S145	112	JAIVAC-1	Dosage A	2	MCV	fL	Day 28	24/09/2010	92.6	.	80.0- 96.0		
S145	112	JAIVAC-1	Dosage A	2	Platelet Count	10E3/ul	Day 28	24/09/2010	224	.	150 - 400		
S145	112	JAIVAC-1	Dosage A	2	RBC count	10E6/ul	Day 28	24/09/2010	5.31	.	4.60 - 6.20		
S145	112	JAIVAC-1	Dosage A	2	TLC	10E3/ul	Day 28	24/09/2010	7.46	.	4.50 - 11.00		
S145	112	JAIVAC-1	Dosage A	2	% Basophils	%	Day 56	22/10/2010	1.1	Yes	0.0 - 1.0	H	NCS
S145	112	JAIVAC-1	Dosage A	2	% Eosinophils	%	Day 56	22/10/2010	0.9	Yes	1.0 - 6.0	L	NCS
S145	112	JAIVAC-1	Dosage A	2	% Lymphocytes	%	Day 56	22/10/2010	29.7	.	20.0 - 45.0		
S145	112	JAIVAC-1	Dosage A	2	% Monocytes	%	Day 56	22/10/2010	3.9	.	2.0 - 10.0		
S145	112	JAIVAC-1	Dosage A	2	% Neutrophils	%	Day 56	22/10/2010	64.5	.	40.0 - 75.0		
S145	112	JAIVAC-1	Dosage A	2	Haematocrit	%	Day 56	22/10/2010	46.7	.	40.0 - 54.0		
S145	112	JAIVAC-1	Dosage A	2	Haemoglobin	gm / dl	Day 56	22/10/2010	16.2	.	13.5 - 18.0		
S145	112	JAIVAC-1	Dosage A	2	MCH	pg	Day 56	22/10/2010	31.2	Yes	27.0 - 31.0	H	NCS
S145	112	JAIVAC-1	Dosage A	2	MCHC	gm %	Day 56	22/10/2010	34.6	.	32.0 - 36.0		
S145	112	JAIVAC-1	Dosage A	2	MCV	fL	Day 56	22/10/2010	90	.	80.0- 96.0		
S145	112	JAIVAC-1	Dosage A	2	Platelet Count	10E3/ul	Day 56	22/10/2010	277	.	150 - 400		
S145	112	JAIVAC-1	Dosage A	2	RBC count	10E6/ul	Day 56	22/10/2010	5.19	.	4.60 - 6.20		
S145	112	JAIVAC-1	Dosage A	2	TLC	10E3/ul	Day 56	22/10/2010	7.53	.	4.50 - 11.00		

Subject No.	Randomisation Number	Study Group	Dosage	Dose	Haematology Parameters	Unit	Day	Date of sample Collection	Results	Out of Range	Reference Range	Flag	Clinical Significance **
S145	112	JAIVAC-1	Dosage A	3	% Basophils	%	Day 180	23/02/2011	0.6	.	0.0 - 1.0		
S145	112	JAIVAC-1	Dosage A	3	% Eosinophils	%	Day 180	23/02/2011	1.5	.	1.0 - 6.0		
S145	112	JAIVAC-1	Dosage A	3	% Lymphocytes	%	Day 180	23/02/2011	14.1	Yes	20.0 - 45.0	L	NCS
S145	112	JAIVAC-1	Dosage A	3	% Monocytes	%	Day 180	23/02/2011	4.6	.	2.0 - 10.0		
S145	112	JAIVAC-1	Dosage A	3	% Neutrophils	%	Day 180	23/02/2011	79.3	Yes	40.0 - 75.0	H	NCS
S145	112	JAIVAC-1	Dosage A	3	Haematocrit	%	Day 180	23/02/2011	46.5	.	40.0 - 54.0		
S145	112	JAIVAC-1	Dosage A	3	Haemoglobin	gm / dl	Day 180	23/02/2011	16.2	.	13.5 - 18.0		
S145	112	JAIVAC-1	Dosage A	3	MCH	pg	Day 180	23/02/2011	30.6	.	27.0 - 31.0		
S145	112	JAIVAC-1	Dosage A	3	MCHC	gm %	Day 180	23/02/2011	34.8	.	32.0 - 36.0		
S145	112	JAIVAC-1	Dosage A	3	MCV	fL	Day 180	23/02/2011	87.7	.	80.0- 96.0		
S145	112	JAIVAC-1	Dosage A	3	Platelet Count	10E3/ul	Day 180	23/02/2011	272	.	150 - 400		
S145	112	JAIVAC-1	Dosage A	3	RBC count	10E6/ul	Day 180	23/02/2011	5.3	.	4.60 - 6.20		
S145	112	JAIVAC-1	Dosage A	3	TLC	10E3/ul	Day 180	23/02/2011	10.55	.	4.50 - 11.00		
S145	112	JAIVAC-1	Dosage A	3	% Basophils	%	Day 208	23/03/2011	0.7	.	0.0 - 1.0		
S145	112	JAIVAC-1	Dosage A	3	% Eosinophils	%	Day 208	23/03/2011	1.4	.	1.0 - 6.0		
S145	112	JAIVAC-1	Dosage A	3	% Lymphocytes	%	Day 208	23/03/2011	34.6	.	20.0 - 45.0		
S145	112	JAIVAC-1	Dosage A	3	% Monocytes	%	Day 208	23/03/2011	6	.	2.0 - 10.0		
S145	112	JAIVAC-1	Dosage A	3	% Neutrophils	%	Day 208	23/03/2011	57.4	.	40.0 - 75.0		
S145	112	JAIVAC-1	Dosage A	3	Haematocrit	%	Day 208	23/03/2011	45.5	.	40.0 - 54.0		
S145	112	JAIVAC-1	Dosage A	3	Haemoglobin	gm / dl	Day 208	23/03/2011	15.5	.	13.5 - 18.0		
S145	112	JAIVAC-1	Dosage A	3	MCH	pg	Day 208	23/03/2011	30.2	.	27.0 - 31.0		
S145	112	JAIVAC-1	Dosage A	3	MCHC	gm %	Day 208	23/03/2011	34.1	.	32.0 - 36.0		
S145	112	JAIVAC-1	Dosage A	3	MCV	fL	Day 208	23/03/2011	88.4	.	80.0- 96.0		
S145	112	JAIVAC-1	Dosage A	3	Platelet Count	10E3/ul	Day 208	23/03/2011	222	.	150 - 400		
S145	112	JAIVAC-1	Dosage A	3	RBC count	10E6/ul	Day 208	23/03/2011	5.15	.	4.60 - 6.20		
S145	112	JAIVAC-1	Dosage A	3	TLC	10E3/ul	Day 208	23/03/2011	5.22	.	4.50 - 11.00		
S145	112	JAIVAC-1	Dosage A	3	% Basophils	%	Day 365	26/08/2011	0.9	.	0.0 - 1.0		
S145	112	JAIVAC-1	Dosage A	3	% Eosinophils	%	Day 365	26/08/2011	1.1	.	1.0 - 6.0		
S145	112	JAIVAC-1	Dosage A	3	% Lymphocytes	%	Day 365	26/08/2011	33.6	.	20.0 - 45.0		

Subject No.	Randomisation Number	Study Group	Dosage	Dose	Haematology Parameters	Unit	Day	Date of sample Collection	Results	Out of Range	Reference Range	Flag	Clinical Significance **
S145	112	JAIVAC-1	Dosage A	3	% Monocytes	%	Day 365	26/08/2011	7.1	.	2.0 - 10.0		
S145	112	JAIVAC-1	Dosage A	3	% Neutrophils	%	Day 365	26/08/2011	57.2	.	40.0 - 75.0		
S145	112	JAIVAC-1	Dosage A	3	Haematocrit	%	Day 365	26/08/2011	47.2	.	40.0 - 54.0		
S145	112	JAIVAC-1	Dosage A	3	Haemoglobin	gm / dl	Day 365	26/08/2011	16.1	.	13.5 - 18.0		
S145	112	JAIVAC-1	Dosage A	3	MCH	pg	Day 365	26/08/2011	30.6	.	27.0 - 31.0		
S145	112	JAIVAC-1	Dosage A	3	MCHC	gm %	Day 365	26/08/2011	34	.	32.0 - 36.0		
S145	112	JAIVAC-1	Dosage A	3	MCV	fL	Day 365	26/08/2011	90	.	80.0- 96.0		
S145	112	JAIVAC-1	Dosage A	3	Platelet Count	10E3/ul	Day 365	26/08/2011	250	.	150 - 400		
S145	112	JAIVAC-1	Dosage A	3	RBC count	10E6/ul	Day 365	26/08/2011	5.25	.	4.60 - 6.20		
S145	112	JAIVAC-1	Dosage A	3	TLC	10E3/ul	Day 365	26/08/2011	6.44	.	4.50 - 11.00		
S148	111	JAIVAC-1	Dosage A		% Basophils	%	Day -14 to Day -1	20/08/2010	0.9	.	0.0 - 1.0		
S148	111	JAIVAC-1	Dosage A		% Eosinophils	%	Day -14 to Day -1	20/08/2010	5.6	.	1.0 - 6.0		
S148	111	JAIVAC-1	Dosage A		% Lymphocytes	%	Day -14 to Day -1	20/08/2010	30.5	.	20.0 - 45.0		
S148	111	JAIVAC-1	Dosage A		% Monocytes	%	Day -14 to Day -1	20/08/2010	4.5	.	2.0 - 10.0		
S148	111	JAIVAC-1	Dosage A		% Neutrophils	%	Day -14 to Day -1	20/08/2010	58.5	.	40.0 - 75.0		
S148	111	JAIVAC-1	Dosage A		Haematocrit	%	Day -14 to Day -1	20/08/2010	51.4	.	40.0 - 54.0		
S148	111	JAIVAC-1	Dosage A		Haemoglobin	gm / dl	Day -14 to Day -1	20/08/2010	16.1	.	13.5 - 18.0		
S148	111	JAIVAC-1	Dosage A		MCH	pg	Day -14 to Day -1	20/08/2010	26.8	Yes	27.0 - 31.0	L	NCS
S148	111	JAIVAC-1	Dosage A		MCHC	gm %	Day -14 to Day -1	20/08/2010	31.3	Yes	32.0 - 36.0	L	NCS
S148	111	JAIVAC-1	Dosage A		MCV	fL	Day -14 to Day -1	20/08/2010	85.7	.	80.0- 96.0		
S148	111	JAIVAC-1	Dosage A		Platelet Count	10E3/ul	Day -14 to Day -1	20/08/2010	418	.	150 - 400	H	
S148	111	JAIVAC-1	Dosage A		RBC count	10E6/ul	Day -14 to Day -1	20/08/2010	6	.	4.60 - 6.20		
S148	111	JAIVAC-1	Dosage A		TLC	10E3/ul	Day -14 to Day -1	20/08/2010	8.29	.	4.50 - 11.00		
S148	111	JAIVAC-1	Dosage A	2	% Basophils	%	Day 28	24/09/2010	0.7	.	0.0 - 1.0		
S148	111	JAIVAC-1	Dosage A	2	% Eosinophils	%	Day 28	24/09/2010	3.4	.	1.0 - 6.0		
S148	111	JAIVAC-1	Dosage A	2	% Lymphocytes	%	Day 28	24/09/2010	24.2	.	20.0 - 45.0		
S148	111	JAIVAC-1	Dosage A	2	% Monocytes	%	Day 28	24/09/2010	5	.	2.0 - 10.0		
S148	111	JAIVAC-1	Dosage A	2	% Neutrophils	%	Day 28	24/09/2010	66.6	.	40.0 - 75.0		
S148	111	JAIVAC-1	Dosage A	2	Haematocrit	%	Day 28	24/09/2010	49.4	.	40.0 - 54.0		

Subject No.	Randomisation Number	Study Group	Dosage	Dose	Haematology Parameters	Unit	Day	Date of sample Collection	Results	Out of Range	Reference Range	Flag	Clinical Significance **
S148	111	JAIVAC-1	Dosage A	2	Haemoglobin	gm / dl	Day 28	24/09/2010	15.4	.	13.5 - 18.0		
S148	111	JAIVAC-1	Dosage A	2	MCH	pg	Day 28	24/09/2010	27.2	.	27.0 - 31.0		
S148	111	JAIVAC-1	Dosage A	2	MCHC	gm %	Day 28	24/09/2010	31.1	Yes	32.0 - 36.0	L	NCS
S148	111	JAIVAC-1	Dosage A	2	MCV	fL	Day 28	24/09/2010	87.5	.	80.0- 96.0		
S148	111	JAIVAC-1	Dosage A	2	Platelet Count	10E3/ul	Day 28	24/09/2010	411	Yes	150 - 400	H	NCS
S148	111	JAIVAC-1	Dosage A	2	RBC count	10E6/ul	Day 28	24/09/2010	5.64	.	4.60 - 6.20		
S148	111	JAIVAC-1	Dosage A	2	TLC	10E3/ul	Day 28	24/09/2010	9.2	.	4.50 - 11.00		
S148	111	JAIVAC-1	Dosage A	2	% Basophils	%	Day 56	22/10/2010	1.5	Yes	0.0 - 1.0	H	NCS
S148	111	JAIVAC-1	Dosage A	2	% Eosinophils	%	Day 56	22/10/2010	5.7	.	1.0 - 6.0		
S148	111	JAIVAC-1	Dosage A	2	% Lymphocytes	%	Day 56	22/10/2010	34	.	20.0 - 45.0		
S148	111	JAIVAC-1	Dosage A	2	% Monocytes	%	Day 56	22/10/2010	4.8	.	2.0 - 10.0		
S148	111	JAIVAC-1	Dosage A	2	% Neutrophils	%	Day 56	22/10/2010	54	.	40.0 - 75.0		
S148	111	JAIVAC-1	Dosage A	2	Haematocrit	%	Day 56	22/10/2010	46.7	.	40.0 - 54.0		
S148	111	JAIVAC-1	Dosage A	2	Haemoglobin	gm / dl	Day 56	22/10/2010	15.5	.	13.5 - 18.0		
S148	111	JAIVAC-1	Dosage A	2	MCH	pg	Day 56	22/10/2010	28.4	.	27.0 - 31.0		
S148	111	JAIVAC-1	Dosage A	2	MCHC	gm %	Day 56	22/10/2010	33.1	.	32.0 - 36.0		
S148	111	JAIVAC-1	Dosage A	2	MCV	fL	Day 56	22/10/2010	85.7	.	80.0- 96.0		
S148	111	JAIVAC-1	Dosage A	2	Platelet Count	10E3/ul	Day 56	22/10/2010	484	Yes	150 - 400	H	NCS
S148	111	JAIVAC-1	Dosage A	2	RBC count	10E6/ul	Day 56	22/10/2010	5.45	.	4.60 - 6.20		
S148	111	JAIVAC-1	Dosage A	2	TLC	10E3/ul	Day 56	22/10/2010	8.28	.	4.50 - 11.00		
S148	111	JAIVAC-1	Dosage A	3	% Basophils	%	Day 180	23/02/2011	1.3	Yes	0.0 - 1.0	H	NCS
S148	111	JAIVAC-1	Dosage A	3	% Eosinophils	%	Day 180	23/02/2011	1.3	.	1.0 - 6.0		
S148	111	JAIVAC-1	Dosage A	3	% Lymphocytes	%	Day 180	23/02/2011	13.9	Yes	20.0 - 45.0	L	NCS
S148	111	JAIVAC-1	Dosage A	3	% Monocytes	%	Day 180	23/02/2011	5.3	.	2.0 - 10.0		
S148	111	JAIVAC-1	Dosage A	3	% Neutrophils	%	Day 180	23/02/2011	78.1	Yes	40.0 - 75.0	H	NCS
S148	111	JAIVAC-1	Dosage A	3	Haematocrit	%	Day 180	23/02/2011	49.3	.	40.0 - 54.0		
S148	111	JAIVAC-1	Dosage A	3	Haemoglobin	gm / dl	Day 180	23/02/2011	16.4	.	13.5 - 18.0		
S148	111	JAIVAC-1	Dosage A	3	MCH	pg	Day 180	23/02/2011	27.7	.	27.0 - 31.0		
S148	111	JAIVAC-1	Dosage A	3	MCHC	gm %	Day 180	23/02/2011	33.2	.	32.0 - 36.0		

Subject No.	Randomisation Number	Study Group	Dosage	Dose	Haematology Parameters	Unit	Day	Date of sample Collection	Results	Out of Range	Reference Range	Flag	Clinical Significance **
S148	111	JAIVAC-1	Dosage A	3	MCV	fL	Day 180	23/02/2011	83.6	.	80.0- 96.0		
S148	111	JAIVAC-1	Dosage A	3	Platelet Count	10E3/ul	Day 180	23/02/2011	507	Yes	150 - 400	H	NCS
S148	111	JAIVAC-1	Dosage A	3	RBC count	10E6/ul	Day 180	23/02/2011	5.9	.	4.60 - 6.20		
S148	111	JAIVAC-1	Dosage A	3	TLC	10E3/ul	Day 180	23/02/2011	11.82	Yes	4.50 - 11.00	H	NCS
S148	111	JAIVAC-1	Dosage A	3	% Basophils	%	Day 208	23/03/2011	1.4	Yes	0.0 - 1.0	H	NCS
S148	111	JAIVAC-1	Dosage A	3	% Eosinophils	%	Day 208	23/03/2011	3.5	.	1.0 - 6.0		
S148	111	JAIVAC-1	Dosage A	3	% Lymphocytes	%	Day 208	23/03/2011	35.9	.	20.0 - 45.0		
S148	111	JAIVAC-1	Dosage A	3	% Monocytes	%	Day 208	23/03/2011	5.6	.	2.0 - 10.0		
S148	111	JAIVAC-1	Dosage A	3	% Neutrophils	%	Day 208	23/03/2011	53.5	.	40.0 - 75.0		
S148	111	JAIVAC-1	Dosage A	3	Haematocrit	%	Day 208	23/03/2011	47.2	.	40.0 - 54.0		
S148	111	JAIVAC-1	Dosage A	3	Haemoglobin	gm / dl	Day 208	23/03/2011	15.9	.	13.5 - 18.0		
S148	111	JAIVAC-1	Dosage A	3	MCH	pg	Day 208	23/03/2011	27.8	.	27.0 - 31.0		
S148	111	JAIVAC-1	Dosage A	3	MCHC	gm %	Day 208	23/03/2011	33.7	.	32.0 - 36.0		
S148	111	JAIVAC-1	Dosage A	3	MCV	fL	Day 208	23/03/2011	82.3	.	80.0- 96.0		
S148	111	JAIVAC-1	Dosage A	3	Platelet Count	10E3/ul	Day 208	23/03/2011	415	Yes	150 - 400	H	NCS
S148	111	JAIVAC-1	Dosage A	3	RBC count	10E6/ul	Day 208	23/03/2011	5.73	.	4.60 - 6.20		
S148	111	JAIVAC-1	Dosage A	3	TLC	10E3/ul	Day 208	23/03/2011	8.4	.	4.50 - 11.00		
S148	111	JAIVAC-1	Dosage A	3	% Basophils	%	Day 365	26/08/2011	2.2	Yes	0.0 - 1.0	H	NCS
S148	111	JAIVAC-1	Dosage A	3	% Eosinophils	%	Day 365	26/08/2011	3.3	.	1.0 - 6.0		
S148	111	JAIVAC-1	Dosage A	3	% Lymphocytes	%	Day 365	26/08/2011	26.9	.	20.0 - 45.0		
S148	111	JAIVAC-1	Dosage A	3	% Monocytes	%	Day 365	26/08/2011	6	.	2.0 - 10.0		
S148	111	JAIVAC-1	Dosage A	3	% Neutrophils	%	Day 365	26/08/2011	61.5	.	40.0 - 75.0		
S148	111	JAIVAC-1	Dosage A	3	Haematocrit	%	Day 365	26/08/2011	49.1	.	40.0 - 54.0		
S148	111	JAIVAC-1	Dosage A	3	Haemoglobin	gm / dl	Day 365	26/08/2011	16.1	.	13.5 - 18.0		
S148	111	JAIVAC-1	Dosage A	3	MCH	pg	Day 365	26/08/2011	28	.	27.0 - 31.0		
S148	111	JAIVAC-1	Dosage A	3	MCHC	gm %	Day 365	26/08/2011	32.8	.	32.0 - 36.0		
S148	111	JAIVAC-1	Dosage A	3	MCV	fL	Day 365	26/08/2011	85.2	.	80.0- 96.0		
S148	111	JAIVAC-1	Dosage A	3	Platelet Count	10E3/ul	Day 365	26/08/2011	463	Yes	150 - 400	H	NCS
S148	111	JAIVAC-1	Dosage A	3	RBC count	10E6/ul	Day 365	26/08/2011	5.76	.	4.60 - 6.20		

Subject No.	Randomisation Number	Study Group	Dosage	Dose	Haematology Parameters	Unit	Day	Date of sample Collection	Results	Out of Range	Reference Range	Flag	Clinical Significance **
S148	111	JAIVAC-1	Dosage A	3	TLC	10E3/ul	Day 365	26/08/2011	8.64	.	4.50 - 11.00		
S154	113	JAIVAC-1	Dosage A		% Basophils	%	Day -14 to Day -1	19/08/2010	0.6	.	0.0 - 1.0		
S154	113	JAIVAC-1	Dosage A		% Eosinophils	%	Day -14 to Day -1	19/08/2010	3.9	.	1.0 - 6.0		
S154	113	JAIVAC-1	Dosage A		% Lymphocytes	%	Day -14 to Day -1	19/08/2010	48.8	Yes	20.0 - 45.0	H	NCS
S154	113	JAIVAC-1	Dosage A		% Monocytes	%	Day -14 to Day -1	19/08/2010	5.5	.	2.0 - 10.0		
S154	113	JAIVAC-1	Dosage A		% Neutrophils	%	Day -14 to Day -1	19/08/2010	41.2	.	40.0 - 75.0		
S154	113	JAIVAC-1	Dosage A		Haematocrit	%	Day -14 to Day -1	19/08/2010	44.5	.	40.0 - 54.0		
S154	113	JAIVAC-1	Dosage A		Haemoglobin	gm / dl	Day -14 to Day -1	19/08/2010	14.2	.	13.5 - 18.0		
S154	113	JAIVAC-1	Dosage A		MCH	pg	Day -14 to Day -1	19/08/2010	25.2	Yes	27.0 - 31.0	L	NCS
S154	113	JAIVAC-1	Dosage A		MCHC	gm %	Day -14 to Day -1	19/08/2010	31.8	Yes	32.0 - 36.0	L	NCS
S154	113	JAIVAC-1	Dosage A		MCV	fL	Day -14 to Day -1	19/08/2010	79.2	Yes	80.0- 96.0	L	NCS
S154	113	JAIVAC-1	Dosage A		Platelet Count	10E3/ul	Day -14 to Day -1	19/08/2010	285	.	150 - 400		
S154	113	JAIVAC-1	Dosage A		RBC count	10E6/ul	Day -14 to Day -1	19/08/2010	5.62	.	4.60 - 6.20		
S154	113	JAIVAC-1	Dosage A		TLC	10E3/ul	Day -14 to Day -1	19/08/2010	6.3	.	4.50 - 11.00		
S154	113	JAIVAC-1	Dosage A	2	% Basophils	%	Day 28	24/09/2010	1	.	0.0 - 1.0		
S154	113	JAIVAC-1	Dosage A	2	% Eosinophils	%	Day 28	24/09/2010	4	.	1.0 - 6.0		
S154	113	JAIVAC-1	Dosage A	2	% Lymphocytes	%	Day 28	24/09/2010	40.8	.	20.0 - 45.0		
S154	113	JAIVAC-1	Dosage A	2	% Monocytes	%	Day 28	24/09/2010	5	.	2.0 - 10.0		
S154	113	JAIVAC-1	Dosage A	2	% Neutrophils	%	Day 28	24/09/2010	49.1	.	40.0 - 75.0		
S154	113	JAIVAC-1	Dosage A	2	Haematocrit	%	Day 28	24/09/2010	46.9	.	40.0 - 54.0		
S154	113	JAIVAC-1	Dosage A	2	Haemoglobin	gm / dl	Day 28	24/09/2010	15	.	13.5 - 18.0		
S154	113	JAIVAC-1	Dosage A	2	MCH	pg	Day 28	24/09/2010	25.1	Yes	27.0 - 31.0	L	NCS
S154	113	JAIVAC-1	Dosage A	2	MCHC	gm %	Day 28	24/09/2010	31.9	Yes	32.0 - 36.0	L	NCS
S154	113	JAIVAC-1	Dosage A	2	MCV	fL	Day 28	24/09/2010	78.5	Yes	80.0- 96.0	L	NCS
S154	113	JAIVAC-1	Dosage A	2	Platelet Count	10E3/ul	Day 28	24/09/2010	258	.	150 - 400		
S154	113	JAIVAC-1	Dosage A	2	RBC count	10E6/ul	Day 28	24/09/2010	5.98	.	4.60 - 6.20		
S154	113	JAIVAC-1	Dosage A	2	TLC	10E3/ul	Day 28	24/09/2010	6.5	.	4.50 - 11.00		
S154	113	JAIVAC-1	Dosage A	2	% Basophils	%	Day 56	22/10/2010	0.6	.	0.0 - 1.0		
S154	113	JAIVAC-1	Dosage A	2	% Eosinophils	%	Day 56	22/10/2010	3.9	.	1.0 - 6.0		

Subject No.	Randomisation Number	Study Group	Dosage	Dose	Haematology Parameters	Unit	Day	Date of sample Collection	Results	Out of Range	Reference Range	Flag	Clinical Significance **
S154	113	JAIVAC-1	Dosage A	2	% Lymphocytes	%	Day 56	22/10/2010	42.9	.	20.0 - 45.0		
S154	113	JAIVAC-1	Dosage A	2	% Monocytes	%	Day 56	22/10/2010	7	.	2.0 - 10.0		
S154	113	JAIVAC-1	Dosage A	2	% Neutrophils	%	Day 56	22/10/2010	45.7	.	40.0 - 75.0		
S154	113	JAIVAC-1	Dosage A	2	Haematocrit	%	Day 56	22/10/2010	44.2	.	40.0 - 54.0		
S154	113	JAIVAC-1	Dosage A	2	Haemoglobin	gm / dl	Day 56	22/10/2010	14.8	.	13.5 - 18.0		
S154	113	JAIVAC-1	Dosage A	2	MCH	pg	Day 56	22/10/2010	25.6	Yes	27.0 - 31.0	L	NCS
S154	113	JAIVAC-1	Dosage A	2	MCHC	gm %	Day 56	22/10/2010	33.4	.	32.0 - 36.0		
S154	113	JAIVAC-1	Dosage A	2	MCV	fL	Day 56	22/10/2010	76.8	Yes	80.0- 96.0	L	NCS
S154	113	JAIVAC-1	Dosage A	2	Platelet Count	10E3/ul	Day 56	22/10/2010	340	.	150 - 400		
S154	113	JAIVAC-1	Dosage A	2	RBC count	10E6/ul	Day 56	22/10/2010	5.76	.	4.60 - 6.20		
S154	113	JAIVAC-1	Dosage A	2	TLC	10E3/ul	Day 56	22/10/2010	5.76	.	4.50 - 11.00		
S154	113	JAIVAC-1	Dosage A	3	% Basophils	%	Day 180	23/02/2011	0.6	.	0.0 - 1.0		
S154	113	JAIVAC-1	Dosage A	3	% Eosinophils	%	Day 180	23/02/2011	3.3	.	1.0 - 6.0		
S154	113	JAIVAC-1	Dosage A	3	% Lymphocytes	%	Day 180	23/02/2011	39.3	.	20.0 - 45.0		
S154	113	JAIVAC-1	Dosage A	3	% Monocytes	%	Day 180	23/02/2011	4.8	.	2.0 - 10.0		
S154	113	JAIVAC-1	Dosage A	3	% Neutrophils	%	Day 180	23/02/2011	52	.	40.0 - 75.0		
S154	113	JAIVAC-1	Dosage A	3	Haematocrit	%	Day 180	23/02/2011	42.1	.	40.0 - 54.0		
S154	113	JAIVAC-1	Dosage A	3	Haemoglobin	gm / dl	Day 180	23/02/2011	14	.	13.5 - 18.0		
S154	113	JAIVAC-1	Dosage A	3	MCH	pg	Day 180	23/02/2011	26	Yes	27.0 - 31.0	L	NCS
S154	113	JAIVAC-1	Dosage A	3	MCHC	gm %	Day 180	23/02/2011	33.3	.	32.0 - 36.0		
S154	113	JAIVAC-1	Dosage A	3	MCV	fL	Day 180	23/02/2011	78.1	Yes	80.0- 96.0	L	NCS
S154	113	JAIVAC-1	Dosage A	3	Platelet Count	10E3/ul	Day 180	23/02/2011	277	.	150 - 400		
S154	113	JAIVAC-1	Dosage A	3	RBC count	10E6/ul	Day 180	23/02/2011	5.39	.	4.60 - 6.20		
S154	113	JAIVAC-1	Dosage A	3	TLC	10E3/ul	Day 180	23/02/2011	7.13	.	4.50 - 11.00		
S154	113	JAIVAC-1	Dosage A	3	% Basophils	%	Day 208	23/03/2011	0.5	.	0.0 - 1.0		
S154	113	JAIVAC-1	Dosage A	3	% Eosinophils	%	Day 208	23/03/2011	2.4	.	1.0 - 6.0		
S154	113	JAIVAC-1	Dosage A	3	% Lymphocytes	%	Day 208	23/03/2011	43.2	.	20.0 - 45.0		
S154	113	JAIVAC-1	Dosage A	3	% Monocytes	%	Day 208	23/03/2011	7.4	.	2.0 - 10.0		
S154	113	JAIVAC-1	Dosage A	3	% Neutrophils	%	Day 208	23/03/2011	46.5	.	40.0 - 75.0		

Subject No.	Randomisation Number	Study Group	Dosage	Dose	Haematology Parameters	Unit	Day	Date of sample Collection	Results	Out of Range	Reference Range	Flag	Clinical Significance **
S154	113	JAIVAC-1	Dosage A	3	Haematocrit	%	Day 208	23/03/2011	43.1	.	40.0 - 54.0		
S154	113	JAIVAC-1	Dosage A	3	Haemoglobin	gm / dl	Day 208	23/03/2011	14.2	.	13.5 - 18.0		
S154	113	JAIVAC-1	Dosage A	3	MCH	pg	Day 208	23/03/2011	25.5	Yes	27.0 - 31.0	L	NCS
S154	113	JAIVAC-1	Dosage A	3	MCHC	gm %	Day 208	23/03/2011	32.8	.	32.0 - 36.0		
S154	113	JAIVAC-1	Dosage A	3	MCV	fL	Day 208	23/03/2011	77.8	Yes	80.0- 96.0	L	NCS
S154	113	JAIVAC-1	Dosage A	3	Platelet Count	10E3/ul	Day 208	23/03/2011	299	.	150 - 400		
S154	113	JAIVAC-1	Dosage A	3	RBC count	10E6/ul	Day 208	23/03/2011	5.54	.	4.60 - 6.20		
S154	113	JAIVAC-1	Dosage A	3	TLC	10E3/ul	Day 208	23/03/2011	5.41	.	4.50 - 11.00		
S154	113	JAIVAC-1	Dosage A	3	% Basophils	%	Day 365	26/08/2011	0.9	.	0.0 - 1.0		
S154	113	JAIVAC-1	Dosage A	3	% Eosinophils	%	Day 365	26/08/2011	2.5	.	1.0 - 6.0		
S154	113	JAIVAC-1	Dosage A	3	% Lymphocytes	%	Day 365	26/08/2011	38.3	.	20.0 - 45.0		
S154	113	JAIVAC-1	Dosage A	3	% Monocytes	%	Day 365	26/08/2011	7.8	.	2.0 - 10.0		
S154	113	JAIVAC-1	Dosage A	3	% Neutrophils	%	Day 365	26/08/2011	50.4	.	40.0 - 75.0		
S154	113	JAIVAC-1	Dosage A	3	Haematocrit	%	Day 365	26/08/2011	46.9	.	40.0 - 54.0		
S154	113	JAIVAC-1	Dosage A	3	Haemoglobin	gm / dl	Day 365	26/08/2011	15.6	.	13.5 - 18.0		
S154	113	JAIVAC-1	Dosage A	3	MCH	pg	Day 365	26/08/2011	26.1	Yes	27.0 - 31.0	L	NCS
S154	113	JAIVAC-1	Dosage A	3	MCHC	gm %	Day 365	26/08/2011	33.4	.	32.0 - 36.0		
S154	113	JAIVAC-1	Dosage A	3	MCV	fL	Day 365	26/08/2011	78.1	Yes	80.0- 96.0	L	NCS
S154	113	JAIVAC-1	Dosage A	3	Platelet Count	10E3/ul	Day 365	26/08/2011	306	.	150 - 400		
S154	113	JAIVAC-1	Dosage A	3	RBC count	10E6/ul	Day 365	26/08/2011	6.01	.	4.60 - 6.20		
S154	113	JAIVAC-1	Dosage A	3	TLC	10E3/ul	Day 365	26/08/2011	6.37	.	4.50 - 11.00		
S156	110	JAIVAC-1	Dosage A		% Basophils	%	Day -14 to Day -1	19/08/2010	2.1	Yes	0.0 - 1.0	H	NCS
S156	110	JAIVAC-1	Dosage A		% Eosinophils	%	Day -14 to Day -1	19/08/2010	8	.	1.0 - 6.0	H	
S156	110	JAIVAC-1	Dosage A		% Lymphocytes	%	Day -14 to Day -1	19/08/2010	36	.	20.0 - 45.0		
S156	110	JAIVAC-1	Dosage A		% Monocytes	%	Day -14 to Day -1	19/08/2010	4.8	.	2.0 - 10.0		
S156	110	JAIVAC-1	Dosage A		% Neutrophils	%	Day -14 to Day -1	19/08/2010	49.1	.	40.0 - 75.0		
S156	110	JAIVAC-1	Dosage A		Haematocrit	%	Day -14 to Day -1	19/08/2010	47.2	.	40.0 - 54.0		
S156	110	JAIVAC-1	Dosage A		Haemoglobin	gm / dl	Day -14 to Day -1	19/08/2010	15.6	.	13.5 - 18.0		
S156	110	JAIVAC-1	Dosage A		MCH	pg	Day -14 to Day -1	19/08/2010	30.5	.	27.0 - 31.0		

Subject No.	Randomisation Number	Study Group	Dosage	Dose	Haematology Parameters	Unit	Day	Date of sample Collection	Results	Out of Range	Reference Range	Flag	Clinical Significance **
S156	110	JAIVAC-1	Dosage A		MCHC	gm %	Day -14 to Day -1	19/08/2010	33	.	32.0 - 36.0		
S156	110	JAIVAC-1	Dosage A		MCV	fL	Day -14 to Day -1	19/08/2010	92.4	.	80.0- 96.0		
S156	110	JAIVAC-1	Dosage A		Platelet Count	10E3/ul	Day -14 to Day -1	19/08/2010	351	.	150 - 400		
S156	110	JAIVAC-1	Dosage A		RBC count	10E6/ul	Day -14 to Day -1	19/08/2010	5.1	.	4.60 - 6.20		
S156	110	JAIVAC-1	Dosage A		TLC	10E3/ul	Day -14 to Day -1	19/08/2010	7.43	.	4.50 - 11.00		
S156	110	JAIVAC-1	Dosage A	2	% Basophils	%	Day 28	24/09/2010	2.1	Yes	0.0 - 1.0	H	NCS
S156	110	JAIVAC-1	Dosage A	2	% Eosinophils	%	Day 28	24/09/2010	7.8	Yes	1.0 - 6.0	H	NCS
S156	110	JAIVAC-1	Dosage A	2	% Lymphocytes	%	Day 28	24/09/2010	39.4	.	20.0 - 45.0		
S156	110	JAIVAC-1	Dosage A	2	% Monocytes	%	Day 28	24/09/2010	5.8	.	2.0 - 10.0		
S156	110	JAIVAC-1	Dosage A	2	% Neutrophils	%	Day 28	24/09/2010	45	.	40.0 - 75.0		
S156	110	JAIVAC-1	Dosage A	2	Haematocrit	%	Day 28	24/09/2010	47.4	.	40.0 - 54.0		
S156	110	JAIVAC-1	Dosage A	2	Haemoglobin	gm / dl	Day 28	24/09/2010	15.6	.	13.5 - 18.0		
S156	110	JAIVAC-1	Dosage A	2	MCH	pg	Day 28	24/09/2010	30.8	.	27.0 - 31.0		
S156	110	JAIVAC-1	Dosage A	2	MCHC	gm %	Day 28	24/09/2010	33	.	32.0 - 36.0		
S156	110	JAIVAC-1	Dosage A	2	MCV	fL	Day 28	24/09/2010	93.5	.	80.0- 96.0		
S156	110	JAIVAC-1	Dosage A	2	Platelet Count	10E3/ul	Day 28	24/09/2010	325	.	150 - 400		
S156	110	JAIVAC-1	Dosage A	2	RBC count	10E6/ul	Day 28	24/09/2010	5.07	.	4.60 - 6.20		
S156	110	JAIVAC-1	Dosage A	2	TLC	10E3/ul	Day 28	24/09/2010	5.65	.	4.50 - 11.00		
S156	110	JAIVAC-1	Dosage A	2	% Basophils	%	Day 56	22/10/2010	1.9	Yes	0.0 - 1.0	H	NCS
S156	110	JAIVAC-1	Dosage A	2	% Eosinophils	%	Day 56	22/10/2010	6.6	Yes	1.0 - 6.0	H	NCS
S156	110	JAIVAC-1	Dosage A	2	% Lymphocytes	%	Day 56	22/10/2010	28.6	.	20.0 - 45.0		
S156	110	JAIVAC-1	Dosage A	2	% Monocytes	%	Day 56	22/10/2010	5.2	.	2.0 - 10.0		
S156	110	JAIVAC-1	Dosage A	2	% Neutrophils	%	Day 56	22/10/2010	57.6	.	40.0 - 75.0		
S156	110	JAIVAC-1	Dosage A	2	Haematocrit	%	Day 56	22/10/2010	44.7	.	40.0 - 54.0		
S156	110	JAIVAC-1	Dosage A	2	Haemoglobin	gm / dl	Day 56	22/10/2010	15.6	.	13.5 - 18.0		
S156	110	JAIVAC-1	Dosage A	2	MCH	pg	Day 56	22/10/2010	31.8	Yes	27.0 - 31.0	H	NCS
S156	110	JAIVAC-1	Dosage A	2	MCHC	gm %	Day 56	22/10/2010	34.8	.	32.0 - 36.0		
S156	110	JAIVAC-1	Dosage A	2	MCV	fL	Day 56	22/10/2010	91.5	.	80.0- 96.0		
S156	110	JAIVAC-1	Dosage A	2	Platelet Count	10E3/ul	Day 56	22/10/2010	374	.	150 - 400		

Subject No.	Randomisation Number	Study Group	Dosage	Dose	Haematology Parameters	Unit	Day	Date of sample Collection	Results	Out of Range	Reference Range	Flag	Clinical Significance **
S156	110	JAIVAC-1	Dosage A	2	RBC count	10E6/ul	Day 56	22/10/2010	4.89	.	4.60 - 6.20		
S156	110	JAIVAC-1	Dosage A	2	TLC	10E3/ul	Day 56	22/10/2010	8.16	.	4.50 - 11.00		
S156	110	JAIVAC-1	Dosage A	3	% Basophils	%	Day 180	23/02/2011	1.3	Yes	0.0 - 1.0	H	NCS
S156	110	JAIVAC-1	Dosage A	3	% Eosinophils	%	Day 180	23/02/2011	9.1	Yes	1.0 - 6.0	H	NCS
S156	110	JAIVAC-1	Dosage A	3	% Lymphocytes	%	Day 180	23/02/2011	43.6	.	20.0 - 45.0		
S156	110	JAIVAC-1	Dosage A	3	% Monocytes	%	Day 180	23/02/2011	5.4	.	2.0 - 10.0		
S156	110	JAIVAC-1	Dosage A	3	% Neutrophils	%	Day 180	23/02/2011	40.6	.	40.0 - 75.0		
S156	110	JAIVAC-1	Dosage A	3	Haematocrit	%	Day 180	23/02/2011	46.2	.	40.0 - 54.0		
S156	110	JAIVAC-1	Dosage A	3	Haemoglobin	gm / dl	Day 180	23/02/2011	15.6	.	13.5 - 18.0		
S156	110	JAIVAC-1	Dosage A	3	MCH	pg	Day 180	23/02/2011	30.8	.	27.0 - 31.0		
S156	110	JAIVAC-1	Dosage A	3	MCHC	gm %	Day 180	23/02/2011	33.8	.	32.0 - 36.0		
S156	110	JAIVAC-1	Dosage A	3	MCV	fL	Day 180	23/02/2011	91.1	.	80.0- 96.0		
S156	110	JAIVAC-1	Dosage A	3	Platelet Count	10E3/ul	Day 180	23/02/2011	320	.	150 - 400		
S156	110	JAIVAC-1	Dosage A	3	RBC count	10E6/ul	Day 180	23/02/2011	5.07	.	4.60 - 6.20		
S156	110	JAIVAC-1	Dosage A	3	TLC	10E3/ul	Day 180	23/02/2011	5.29	.	4.50 - 11.00		
S156	110	JAIVAC-1	Dosage A	3	% Basophils	%	Day 208	23/03/2011	1	.	0.0 - 1.0		
S156	110	JAIVAC-1	Dosage A	3	% Eosinophils	%	Day 208	23/03/2011	2.8	.	1.0 - 6.0		
S156	110	JAIVAC-1	Dosage A	3	% Lymphocytes	%	Day 208	23/03/2011	29.3	.	20.0 - 45.0		
S156	110	JAIVAC-1	Dosage A	3	% Monocytes	%	Day 208	23/03/2011	5.6	.	2.0 - 10.0		
S156	110	JAIVAC-1	Dosage A	3	% Neutrophils	%	Day 208	23/03/2011	61.3	.	40.0 - 75.0		
S156	110	JAIVAC-1	Dosage A	3	Haematocrit	%	Day 208	23/03/2011	46.7	.	40.0 - 54.0		
S156	110	JAIVAC-1	Dosage A	3	Haemoglobin	gm / dl	Day 208	23/03/2011	15.8	.	13.5 - 18.0		
S156	110	JAIVAC-1	Dosage A	3	MCH	pg	Day 208	23/03/2011	31.2	Yes	27.0 - 31.0	H	NCS
S156	110	JAIVAC-1	Dosage A	3	MCHC	gm %	Day 208	23/03/2011	33.9	.	32.0 - 36.0		
S156	110	JAIVAC-1	Dosage A	3	MCV	fL	Day 208	23/03/2011	92.2	.	80.0- 96.0		
S156	110	JAIVAC-1	Dosage A	3	Platelet Count	10E3/ul	Day 208	23/03/2011	326	.	150 - 400		
S156	110	JAIVAC-1	Dosage A	3	RBC count	10E6/ul	Day 208	23/03/2011	5.06	.	4.60 - 6.20		
S156	110	JAIVAC-1	Dosage A	3	TLC	10E3/ul	Day 208	23/03/2011	5.91	.	4.50 - 11.00		
S156	110	JAIVAC-1	Dosage A	3	% Basophils	%	Day 365	26/08/2011	2.2	Yes	0.0 - 1.0	H	NCS

Subject No.	Randomisation Number	Study Group	Dosage	Dose	Haematology Parameters	Unit	Day	Date of sample Collection	Results	Out of Range	Reference Range	Flag	Clinical Significance **
S156	110	JAIVAC-1	Dosage A	3	% Eosinophils	%	Day 365	26/08/2011	5.8	.	1.0 - 6.0		
S156	110	JAIVAC-1	Dosage A	3	% Lymphocytes	%	Day 365	26/08/2011	33.9	.	20.0 - 45.0		
S156	110	JAIVAC-1	Dosage A	3	% Monocytes	%	Day 365	26/08/2011	6	.	2.0 - 10.0		
S156	110	JAIVAC-1	Dosage A	3	% Neutrophils	%	Day 365	26/08/2011	52	.	40.0 - 75.0		
S156	110	JAIVAC-1	Dosage A	3	Haematocrit	%	Day 365	26/08/2011	47.1	.	40.0 - 54.0		
S156	110	JAIVAC-1	Dosage A	3	Haemoglobin	gm / dl	Day 365	26/08/2011	16	.	13.5 - 18.0		
S156	110	JAIVAC-1	Dosage A	3	MCH	pg	Day 365	26/08/2011	31.8	Yes	27.0 - 31.0	H	NCS
S156	110	JAIVAC-1	Dosage A	3	MCHC	gm %	Day 365	26/08/2011	33.9	.	32.0 - 36.0		
S156	110	JAIVAC-1	Dosage A	3	MCV	fL	Day 365	26/08/2011	93.6	.	80.0- 96.0		
S156	110	JAIVAC-1	Dosage A	3	Platelet Count	10E3/ul	Day 365	26/08/2011	371	.	150 - 400		
S156	110	JAIVAC-1	Dosage A	3	RBC count	10E6/ul	Day 365	26/08/2011	5.03	.	4.60 - 6.20		
S156	110	JAIVAC-1	Dosage A	3	TLC	10E3/ul	Day 365	26/08/2011	6.22	.	4.50 - 11.00		
S170	204	Hepatitis B	Hepatitis B		% Basophils	%	Day -14 to Day -1	21/09/2010	0.6	.	0.0 - 1.0		
S170	204	Hepatitis B	Hepatitis B		% Eosinophils	%	Day -14 to Day -1	21/09/2010	1.8	.	1.0 - 6.0		
S170	204	Hepatitis B	Hepatitis B		% Lymphocytes	%	Day -14 to Day -1	21/09/2010	36.6	.	20.0 - 45.0		
S170	204	Hepatitis B	Hepatitis B		% Monocytes	%	Day -14 to Day -1	21/09/2010	7.8	.	2.0 - 10.0		
S170	204	Hepatitis B	Hepatitis B		% Neutrophils	%	Day -14 to Day -1	21/09/2010	53.3	.	40.0 - 75.0		
S170	204	Hepatitis B	Hepatitis B		Haematocrit	%	Day -14 to Day -1	21/09/2010	45.7	.	40.0 - 54.0		
S170	204	Hepatitis B	Hepatitis B		Haemoglobin	gm / dl	Day -14 to Day -1	21/09/2010	15.6	.	13.5 - 18.0		
S170	204	Hepatitis B	Hepatitis B		MCH	pg	Day -14 to Day -1	21/09/2010	31.2	Yes	27.0 - 31.0	H	NCS
S170	204	Hepatitis B	Hepatitis B		MCHC	gm %	Day -14 to Day -1	21/09/2010	34	.	32.0 - 36.0		
S170	204	Hepatitis B	Hepatitis B		MCV	fL	Day -14 to Day -1	21/09/2010	91.6	.	80.0- 96.0		
S170	204	Hepatitis B	Hepatitis B		Platelet Count	10E3/ul	Day -14 to Day -1	21/09/2010	213	.	150 - 400		
S170	204	Hepatitis B	Hepatitis B		RBC count	10E6/ul	Day -14 to Day -1	21/09/2010	4.99	.	4.60 - 6.20		
S170	204	Hepatitis B	Hepatitis B		TLC	10E3/ul	Day -14 to Day -1	21/09/2010	5.66	.	4.50 - 11.00		
S170	204	Hepatitis B	Hepatitis B	2	% Basophils	%	Day 28	27/10/2010	1.5	Yes	0.0 - 1.0	H	NCS
S170	204	Hepatitis B	Hepatitis B	2	% Eosinophils	%	Day 28	27/10/2010	1.4	.	1.0 - 6.0		
S170	204	Hepatitis B	Hepatitis B	2	% Lymphocytes	%	Day 28	27/10/2010	37.5	.	20.0 - 45.0		
S170	204	Hepatitis B	Hepatitis B	2	% Monocytes	%	Day 28	27/10/2010	9.5	.	2.0 - 10.0		

Subject No.	Randomisation Number	Study Group	Dosage	Dose	Haematology Parameters	Unit	Day	Date of sample Collection	Results	Out of Range	Reference Range	Flag	Clinical Significance **
S170	204	Hepatitis B	Hepatitis B	2	% Neutrophils	%	Day 28	27/10/2010	50.1	.	40.0 - 75.0		
S170	204	Hepatitis B	Hepatitis B	2	Haematocrit	%	Day 28	27/10/2010	46.3	.	40.0 - 54.0		
S170	204	Hepatitis B	Hepatitis B	2	Haemoglobin	gm / dl	Day 28	27/10/2010	15.4	.	13.5 - 18.0		
S170	204	Hepatitis B	Hepatitis B	2	MCH	pg	Day 28	27/10/2010	30.2	.	27.0 - 31.0		
S170	204	Hepatitis B	Hepatitis B	2	MCHC	gm %	Day 28	27/10/2010	33.3	.	32.0 - 36.0		
S170	204	Hepatitis B	Hepatitis B	2	MCV	fL	Day 28	27/10/2010	90.8	.	80.0- 96.0		
S170	204	Hepatitis B	Hepatitis B	2	Platelet Count	10E3/ul	Day 28	27/10/2010	258	.	150 - 400		
S170	204	Hepatitis B	Hepatitis B	2	RBC count	10E6/ul	Day 28	27/10/2010	5.1	.	4.60 - 6.20		
S170	204	Hepatitis B	Hepatitis B	2	TLC	10E3/ul	Day 28	27/10/2010	4.43	Yes	4.50 - 11.00	L	NCS
S170	204	Hepatitis B	Hepatitis B	2	% Basophils	%	Day 56	25/11/2010	0.4	.	0.0 - 1.0		
S170	204	Hepatitis B	Hepatitis B	2	% Eosinophils	%	Day 56	25/11/2010	1.1	.	1.0 - 6.0		
S170	204	Hepatitis B	Hepatitis B	2	% Lymphocytes	%	Day 56	25/11/2010	24.9	.	20.0 - 45.0		
S170	204	Hepatitis B	Hepatitis B	2	% Monocytes	%	Day 56	25/11/2010	8.5	.	2.0 - 10.0		
S170	204	Hepatitis B	Hepatitis B	2	% Neutrophils	%	Day 56	25/11/2010	65.2	.	40.0 - 75.0		
S170	204	Hepatitis B	Hepatitis B	2	Haematocrit	%	Day 56	25/11/2010	48.6	.	40.0 - 54.0		
S170	204	Hepatitis B	Hepatitis B	2	Haemoglobin	gm / dl	Day 56	25/11/2010	16.5	.	13.5 - 18.0		
S170	204	Hepatitis B	Hepatitis B	2	MCH	pg	Day 56	25/11/2010	31	.	27.0 - 31.0		
S170	204	Hepatitis B	Hepatitis B	2	MCHC	gm %	Day 56	25/11/2010	33.8	.	32.0 - 36.0		
S170	204	Hepatitis B	Hepatitis B	2	MCV	fL	Day 56	25/11/2010	91.6	.	80.0- 96.0		
S170	204	Hepatitis B	Hepatitis B	2	Platelet Count	10E3/ul	Day 56	25/11/2010	228	.	150 - 400		
S170	204	Hepatitis B	Hepatitis B	2	RBC count	10E6/ul	Day 56	25/11/2010	5.31	.	4.60 - 6.20		
S170	204	Hepatitis B	Hepatitis B	2	TLC	10E3/ul	Day 56	25/11/2010	7	.	4.50 - 11.00		
S170	204	Hepatitis B	Hepatitis B	3	% Basophils	%	Day 180	28/03/2011	1.1	Yes	0.0 - 1.0	H	NCS
S170	204	Hepatitis B	Hepatitis B	3	% Eosinophils	%	Day 180	28/03/2011	2.3	.	1.0 - 6.0		
S170	204	Hepatitis B	Hepatitis B	3	% Lymphocytes	%	Day 180	28/03/2011	31.7	.	20.0 - 45.0		
S170	204	Hepatitis B	Hepatitis B	3	% Monocytes	%	Day 180	28/03/2011	8.5	.	2.0 - 10.0		
S170	204	Hepatitis B	Hepatitis B	3	% Neutrophils	%	Day 180	28/03/2011	56.4	.	40.0 - 75.0		
S170	204	Hepatitis B	Hepatitis B	3	Haematocrit	%	Day 180	28/03/2011	46.3	.	40.0 - 54.0		
S170	204	Hepatitis B	Hepatitis B	3	Haemoglobin	gm / dl	Day 180	28/03/2011	15.8	.	13.5 - 18.0		

Subject No.	Randomisation Number	Study Group	Dosage	Dose	Haematology Parameters	Unit	Day	Date of sample Collection	Results	Out of Range	Reference Range	Flag	Clinical Significance **
S170	204	Hepatitis B	Hepatitis B	3	MCH	pg	Day 180	28/03/2011	30.5	.	27.0 - 31.0		
S170	204	Hepatitis B	Hepatitis B	3	MCHC	gm %	Day 180	28/03/2011	34.2	.	32.0 - 36.0		
S170	204	Hepatitis B	Hepatitis B	3	MCV	fL	Day 180	28/03/2011	89.2	.	80.0- 96.0		
S170	204	Hepatitis B	Hepatitis B	3	Platelet Count	10E3/ul	Day 180	28/03/2011	224	.	150 - 400		
S170	204	Hepatitis B	Hepatitis B	3	RBC count	10E6/ul	Day 180	28/03/2011	5.19	.	4.60 - 6.20		
S170	204	Hepatitis B	Hepatitis B	3	TLC	10E3/ul	Day 180	28/03/2011	4.84	.	4.50 - 11.00		
S170	204	Hepatitis B	Hepatitis B	3	% Basophils	%	Day 208	25/04/2011	1.8	Yes	0.0 - 1.0	H	NCS
S170	204	Hepatitis B	Hepatitis B	3	% Eosinophils	%	Day 208	25/04/2011	2.9	.	1.0 - 6.0		
S170	204	Hepatitis B	Hepatitis B	3	% Lymphocytes	%	Day 208	25/04/2011	33.2	.	20.0 - 45.0		
S170	204	Hepatitis B	Hepatitis B	3	% Monocytes	%	Day 208	25/04/2011	9.9	.	2.0 - 10.0		
S170	204	Hepatitis B	Hepatitis B	3	% Neutrophils	%	Day 208	25/04/2011	52.2	.	40.0 - 75.0		
S170	204	Hepatitis B	Hepatitis B	3	Haematocrit	%	Day 208	25/04/2011	46.2	.	40.0 - 54.0		
S170	204	Hepatitis B	Hepatitis B	3	Haemoglobin	gm / dl	Day 208	25/04/2011	15.8	.	13.5 - 18.0		
S170	204	Hepatitis B	Hepatitis B	3	MCH	pg	Day 208	25/04/2011	31.2	Yes	27.0 - 31.0	H	NCS
S170	204	Hepatitis B	Hepatitis B	3	MCHC	gm %	Day 208	25/04/2011	34.2	.	32.0 - 36.0		
S170	204	Hepatitis B	Hepatitis B	3	MCV	fL	Day 208	25/04/2011	91.3	.	80.0- 96.0		
S170	204	Hepatitis B	Hepatitis B	3	Platelet Count	10E3/ul	Day 208	25/04/2011	245	.	150 - 400		
S170	204	Hepatitis B	Hepatitis B	3	RBC count	10E6/ul	Day 208	25/04/2011	5.06	.	4.60 - 6.20		
S170	204	Hepatitis B	Hepatitis B	3	TLC	10E3/ul	Day 208	25/04/2011	5.69	.	4.50 - 11.00		
S170	204	Hepatitis B	Hepatitis B	3	% Basophils	%	Day 365	28/09/2011	1.2	Yes	0.0 - 1.0	H	NCS
S170	204	Hepatitis B	Hepatitis B	3	% Eosinophils	%	Day 365	28/09/2011	2	.	1.0 - 6.0		
S170	204	Hepatitis B	Hepatitis B	3	% Lymphocytes	%	Day 365	28/09/2011	38.5	.	20.0 - 45.0		
S170	204	Hepatitis B	Hepatitis B	3	% Monocytes	%	Day 365	28/09/2011	8.6	.	2.0 - 10.0		
S170	204	Hepatitis B	Hepatitis B	3	% Neutrophils	%	Day 365	28/09/2011	49.7	.	40.0 - 75.0		
S170	204	Hepatitis B	Hepatitis B	3	Haematocrit	%	Day 365	28/09/2011	47.2	.	40.0 - 54.0		
S170	204	Hepatitis B	Hepatitis B	3	Haemoglobin	gm / dl	Day 365	28/09/2011	16	.	13.5 - 18.0		
S170	204	Hepatitis B	Hepatitis B	3	MCH	pg	Day 365	28/09/2011	30.1	.	27.0 - 31.0		
S170	204	Hepatitis B	Hepatitis B	3	MCHC	gm %	Day 365	28/09/2011	33.9	.	32.0 - 36.0		
S170	204	Hepatitis B	Hepatitis B	3	MCV	fL	Day 365	28/09/2011	88.9	.	80.0- 96.0		

Subject No.	Randomisation Number	Study Group	Dosage	Dose	Haematology Parameters	Unit	Day	Date of sample Collection	Results	Out of Range	Reference Range	Flag	Clinical Significance **
S170	204	Hepatitis B	Hepatitis B	3	Platelet Count	10E3/ul	Day 365	28/09/2011	205	.	150 - 400		
S170	204	Hepatitis B	Hepatitis B	3	RBC count	10E6/ul	Day 365	28/09/2011	5.31	.	4.60 - 6.20		
S170	204	Hepatitis B	Hepatitis B	3	TLC	10E3/ul	Day 365	28/09/2011	5.33	.	4.50 - 11.00		
S175	209	JAIVAC-1	Dosage B		% Basophils	%	Day -14 to Day -1	21/09/2010	1.6	Yes	0.0 - 1.0	H	NCS
S175	209	JAIVAC-1	Dosage B		% Eosinophils	%	Day -14 to Day -1	21/09/2010	4.4	.	1.0 - 6.0		
S175	209	JAIVAC-1	Dosage B		% Lymphocytes	%	Day -14 to Day -1	21/09/2010	32.1	.	20.0 - 45.0		
S175	209	JAIVAC-1	Dosage B		% Monocytes	%	Day -14 to Day -1	21/09/2010	4.3	.	2.0 - 10.0		
S175	209	JAIVAC-1	Dosage B		% Neutrophils	%	Day -14 to Day -1	21/09/2010	57.6	.	40.0 - 75.0		
S175	209	JAIVAC-1	Dosage B		Haematocrit	%	Day -14 to Day -1	21/09/2010	49.1	.	40.0 - 54.0		
S175	209	JAIVAC-1	Dosage B		Haemoglobin	gm / dl	Day -14 to Day -1	21/09/2010	16	.	13.5 - 18.0		
S175	209	JAIVAC-1	Dosage B		MCH	pg	Day -14 to Day -1	21/09/2010	26.4	Yes	27.0 - 31.0	L	NCS
S175	209	JAIVAC-1	Dosage B		MCHC	gm %	Day -14 to Day -1	21/09/2010	32.6	.	32.0 - 36.0		
S175	209	JAIVAC-1	Dosage B		MCV	fL	Day -14 to Day -1	21/09/2010	81.1	.	80.0- 96.0		
S175	209	JAIVAC-1	Dosage B		Platelet Count	10E3/ul	Day -14 to Day -1	21/09/2010	261	.	150 - 400		
S175	209	JAIVAC-1	Dosage B		RBC count	10E6/ul	Day -14 to Day -1	21/09/2010	6.05	.	4.60 - 6.20		
S175	209	JAIVAC-1	Dosage B		TLC	10E3/ul	Day -14 to Day -1	21/09/2010	5.49	.	4.50 - 11.00		
S175	209	JAIVAC-1	Dosage B	2	% Basophils	%	Day 28	27/10/2010	0.8	.	0.0 - 1.0		
S175	209	JAIVAC-1	Dosage B	2	% Eosinophils	%	Day 28	27/10/2010	1.4	.	1.0 - 6.0		
S175	209	JAIVAC-1	Dosage B	2	% Lymphocytes	%	Day 28	27/10/2010	40.4	.	20.0 - 45.0		
S175	209	JAIVAC-1	Dosage B	2	% Monocytes	%	Day 28	27/10/2010	7.6	.	2.0 - 10.0		
S175	209	JAIVAC-1	Dosage B	2	% Neutrophils	%	Day 28	27/10/2010	49.9	.	40.0 - 75.0		
S175	209	JAIVAC-1	Dosage B	2	Haematocrit	%	Day 28	27/10/2010	44.7	.	40.0 - 54.0		
S175	209	JAIVAC-1	Dosage B	2	Haemoglobin	gm / dl	Day 28	27/10/2010	14.2	.	13.5 - 18.0		
S175	209	JAIVAC-1	Dosage B	2	MCH	pg	Day 28	27/10/2010	25.6	Yes	27.0 - 31.0	L	NCS
S175	209	JAIVAC-1	Dosage B	2	MCHC	gm %	Day 28	27/10/2010	31.8	Yes	32.0 - 36.0	L	NCS
S175	209	JAIVAC-1	Dosage B	2	MCV	fL	Day 28	27/10/2010	80.5	.	80.0- 96.0		
S175	209	JAIVAC-1	Dosage B	2	Platelet Count	10E3/ul	Day 28	27/10/2010	317	.	150 - 400		
S175	209	JAIVAC-1	Dosage B	2	RBC count	10E6/ul	Day 28	27/10/2010	5.56	.	4.60 - 6.20		
S175	209	JAIVAC-1	Dosage B	2	TLC	10E3/ul	Day 28	27/10/2010	6.4	.	4.50 - 11.00		

Subject No.	Randomisation Number	Study Group	Dosage	Dose	Haematology Parameters	Unit	Day	Date of sample Collection	Results	Out of Range	Reference Range	Flag	Clinical Significance **
S175	209	JAIVAC-1	Dosage B	2	% Basophils	%	Day 56	24/11/2010	0.8	.	0.0 - 1.0		
S175	209	JAIVAC-1	Dosage B	2	% Eosinophils	%	Day 56	24/11/2010	5.2	.	1.0 - 6.0		
S175	209	JAIVAC-1	Dosage B	2	% Lymphocytes	%	Day 56	24/11/2010	42.7	.	20.0 - 45.0		
S175	209	JAIVAC-1	Dosage B	2	% Monocytes	%	Day 56	24/11/2010	4.9	.	2.0 - 10.0		
S175	209	JAIVAC-1	Dosage B	2	% Neutrophils	%	Day 56	24/11/2010	46.3	.	40.0 - 75.0		
S175	209	JAIVAC-1	Dosage B	2	Haematocrit	%	Day 56	24/11/2010	50.7	.	40.0 - 54.0		
S175	209	JAIVAC-1	Dosage B	2	Haemoglobin	gm / dl	Day 56	24/11/2010	16.1	.	13.5 - 18.0		
S175	209	JAIVAC-1	Dosage B	2	MCH	pg	Day 56	24/11/2010	25.8	Yes	27.0 - 31.0	L	NCS
S175	209	JAIVAC-1	Dosage B	2	MCHC	gm %	Day 56	24/11/2010	31.8	Yes	32.0 - 36.0	L	NCS
S175	209	JAIVAC-1	Dosage B	2	MCV	fL	Day 56	24/11/2010	81.1	.	80.0- 96.0		
S175	209	JAIVAC-1	Dosage B	2	Platelet Count	10E3/ul	Day 56	24/11/2010	254	.	150 - 400		
S175	209	JAIVAC-1	Dosage B	2	RBC count	10E6/ul	Day 56	24/11/2010	6.25	Yes	4.60 - 6.20	H	NCS
S175	209	JAIVAC-1	Dosage B	2	TLC	10E3/ul	Day 56	24/11/2010	4.91	.	4.50 - 11.00		
S175	209	JAIVAC-1	Dosage B	3	% Basophils	%	Day 180	28/03/2011	1.2	Yes	0.0 - 1.0	H	NCS
S175	209	JAIVAC-1	Dosage B	3	% Eosinophils	%	Day 180	28/03/2011	3.4	.	1.0 - 6.0		
S175	209	JAIVAC-1	Dosage B	3	% Lymphocytes	%	Day 180	28/03/2011	30.6	.	20.0 - 45.0		
S175	209	JAIVAC-1	Dosage B	3	% Monocytes	%	Day 180	28/03/2011	4.8	.	2.0 - 10.0		
S175	209	JAIVAC-1	Dosage B	3	% Neutrophils	%	Day 180	28/03/2011	60	.	40.0 - 75.0		
S175	209	JAIVAC-1	Dosage B	3	Haematocrit	%	Day 180	28/03/2011	46.7	.	40.0 - 54.0		
S175	209	JAIVAC-1	Dosage B	3	Haemoglobin	gm / dl	Day 180	28/03/2011	15.5	.	13.5 - 18.0		
S175	209	JAIVAC-1	Dosage B	3	MCH	pg	Day 180	28/03/2011	25.6	Yes	27.0 - 31.0	L	NCS
S175	209	JAIVAC-1	Dosage B	3	MCHC	gm %	Day 180	28/03/2011	33.2	.	32.0 - 36.0		
S175	209	JAIVAC-1	Dosage B	3	MCV	fL	Day 180	28/03/2011	77.2	Yes	80.0- 96.0	L	NCS
S175	209	JAIVAC-1	Dosage B	3	Platelet Count	10E3/ul	Day 180	28/03/2011	273	.	150 - 400		
S175	209	JAIVAC-1	Dosage B	3	RBC count	10E6/ul	Day 180	28/03/2011	6.05	.	4.60 - 6.20		
S175	209	JAIVAC-1	Dosage B	3	TLC	10E3/ul	Day 180	28/03/2011	5.01	.	4.50 - 11.00		
S175	209	JAIVAC-1	Dosage B	3	% Basophils	%	Day 208	25/04/2011	1	.	0.0 - 1.0		
S175	209	JAIVAC-1	Dosage B	3	% Eosinophils	%	Day 208	25/04/2011	4	.	1.0 - 6.0		
S175	209	JAIVAC-1	Dosage B	3	% Lymphocytes	%	Day 208	25/04/2011	36.2	.	20.0 - 45.0		

Subject No.	Randomisation Number	Study Group	Dosage	Dose	Haematology Parameters	Unit	Day	Date of sample Collection	Results	Out of Range	Reference Range	Flag	Clinical Significance **
S175	209	JAIVAC-1	Dosage B	3	% Monocytes	%	Day 208	25/04/2011	5.8	.	2.0 - 10.0		
S175	209	JAIVAC-1	Dosage B	3	% Neutrophils	%	Day 208	25/04/2011	53	.	40.0 - 75.0		
S175	209	JAIVAC-1	Dosage B	3	Haematocrit	%	Day 208	25/04/2011	49.3	.	40.0 - 54.0		
S175	209	JAIVAC-1	Dosage B	3	Haemoglobin	gm / dl	Day 208	25/04/2011	16.3	.	13.5 - 18.0		
S175	209	JAIVAC-1	Dosage B	3	MCH	pg	Day 208	25/04/2011	26.7	Yes	27.0 - 31.0	L	NCS
S175	209	JAIVAC-1	Dosage B	3	MCHC	gm %	Day 208	25/04/2011	33.1	.	32.0 - 36.0		
S175	209	JAIVAC-1	Dosage B	3	MCV	fL	Day 208	25/04/2011	80.4	.	80.0- 96.0		
S175	209	JAIVAC-1	Dosage B	3	Platelet Count	10E3/ul	Day 208	25/04/2011	292	.	150 - 400		
S175	209	JAIVAC-1	Dosage B	3	RBC count	10E6/ul	Day 208	25/04/2011	6.13	.	4.60 - 6.20		
S175	209	JAIVAC-1	Dosage B	3	TLC	10E3/ul	Day 208	25/04/2011	5.2	.	4.50 - 11.00		
S175	209	JAIVAC-1	Dosage B	3	% Basophils	%	Day 365	28/09/2011	1.8	Yes	0.0 - 1.0	H	NCS
S175	209	JAIVAC-1	Dosage B	3	% Eosinophils	%	Day 365	28/09/2011	4.8	.	1.0 - 6.0		
S175	209	JAIVAC-1	Dosage B	3	% Lymphocytes	%	Day 365	28/09/2011	31.5	.	20.0 - 45.0		
S175	209	JAIVAC-1	Dosage B	3	% Monocytes	%	Day 365	28/09/2011	5.1	.	2.0 - 10.0		
S175	209	JAIVAC-1	Dosage B	3	% Neutrophils	%	Day 365	28/09/2011	56.8	.	40.0 - 75.0		
S175	209	JAIVAC-1	Dosage B	3	Haematocrit	%	Day 365	28/09/2011	46	.	40.0 - 54.0		
S175	209	JAIVAC-1	Dosage B	3	Haemoglobin	gm / dl	Day 365	28/09/2011	15.1	.	13.5 - 18.0		
S175	209	JAIVAC-1	Dosage B	3	MCH	pg	Day 365	28/09/2011	26.4	Yes	27.0 - 31.0	L	NCS
S175	209	JAIVAC-1	Dosage B	3	MCHC	gm %	Day 365	28/09/2011	32.9	.	32.0 - 36.0		
S175	209	JAIVAC-1	Dosage B	3	MCV	fL	Day 365	28/09/2011	80.3	.	80.0- 96.0		
S175	209	JAIVAC-1	Dosage B	3	Platelet Count	10E3/ul	Day 365	28/09/2011	322	.	150 - 400		
S175	209	JAIVAC-1	Dosage B	3	RBC count	10E6/ul	Day 365	28/09/2011	5.73	.	4.60 - 6.20		
S175	209	JAIVAC-1	Dosage B	3	TLC	10E3/ul	Day 365	28/09/2011	5.32	.	4.50 - 11.00		
S176	205	Hepatitis B	Hepatitis B		% Basophils	%	Day -14 to Day -1	22/09/2010	1.1	Yes	0.0 - 1.0	H	NCS
S176	205	Hepatitis B	Hepatitis B		% Eosinophils	%	Day -14 to Day -1	22/09/2010	3	.	1.0 - 6.0		
S176	205	Hepatitis B	Hepatitis B		% Lymphocytes	%	Day -14 to Day -1	22/09/2010	35.3	.	20.0 - 45.0		
S176	205	Hepatitis B	Hepatitis B		% Monocytes	%	Day -14 to Day -1	22/09/2010	5.1	.	2.0 - 10.0		
S176	205	Hepatitis B	Hepatitis B		% Neutrophils	%	Day -14 to Day -1	22/09/2010	55.5	.	40.0 - 75.0		
S176	205	Hepatitis B	Hepatitis B		Haematocrit	%	Day -14 to Day -1	22/09/2010	44.4	.	40.0 - 54.0		

Subject No.	Randomisation Number	Study Group	Dosage	Dose	Haematology Parameters	Unit	Day	Date of sample Collection	Results	Out of Range	Reference Range	Flag	Clinical Significance **
S176	205	Hepatitis B	Hepatitis B		Haemoglobin	gm / dl	Day -14 to Day -1	22/09/2010	15.6	.	13.5 - 18.0		
S176	205	Hepatitis B	Hepatitis B		MCH	pg	Day -14 to Day -1	22/09/2010	30.4	.	27.0 - 31.0		
S176	205	Hepatitis B	Hepatitis B		MCHC	gm %	Day -14 to Day -1	22/09/2010	35.2	.	32.0 - 36.0		
S176	205	Hepatitis B	Hepatitis B		MCV	fL	Day -14 to Day -1	22/09/2010	86.5	.	80.0- 96.0		
S176	205	Hepatitis B	Hepatitis B		Platelet Count	10E3/ul	Day -14 to Day -1	22/09/2010	230	.	150 - 400		
S176	205	Hepatitis B	Hepatitis B		RBC count	10E6/ul	Day -14 to Day -1	22/09/2010	5.13	.	4.60 - 6.20		
S176	205	Hepatitis B	Hepatitis B		TLC	10E3/ul	Day -14 to Day -1	22/09/2010	5.28	.	4.50 - 11.00		
S176	205	Hepatitis B	Hepatitis B	2	% Basophils	%	Day 28	27/10/2010	1.7	Yes	0.0 - 1.0	H	NCS
S176	205	Hepatitis B	Hepatitis B	2	% Eosinophils	%	Day 28	27/10/2010	2.2	.	1.0 - 6.0		
S176	205	Hepatitis B	Hepatitis B	2	% Lymphocytes	%	Day 28	27/10/2010	32	.	20.0 - 45.0		
S176	205	Hepatitis B	Hepatitis B	2	% Monocytes	%	Day 28	27/10/2010	4.9	.	2.0 - 10.0		
S176	205	Hepatitis B	Hepatitis B	2	% Neutrophils	%	Day 28	27/10/2010	59.1	.	40.0 - 75.0		
S176	205	Hepatitis B	Hepatitis B	2	Haematocrit	%	Day 28	27/10/2010	45.3	.	40.0 - 54.0		
S176	205	Hepatitis B	Hepatitis B	2	Haemoglobin	gm / dl	Day 28	27/10/2010	15.7	.	13.5 - 18.0		
S176	205	Hepatitis B	Hepatitis B	2	MCH	pg	Day 28	27/10/2010	29.7	.	27.0 - 31.0		
S176	205	Hepatitis B	Hepatitis B	2	MCHC	gm %	Day 28	27/10/2010	34.6	.	32.0 - 36.0		
S176	205	Hepatitis B	Hepatitis B	2	MCV	fL	Day 28	27/10/2010	85.8	.	80.0- 96.0		
S176	205	Hepatitis B	Hepatitis B	2	Platelet Count	10E3/ul	Day 28	27/10/2010	261	.	150 - 400		
S176	205	Hepatitis B	Hepatitis B	2	RBC count	10E6/ul	Day 28	27/10/2010	5.28	.	4.60 - 6.20		
S176	205	Hepatitis B	Hepatitis B	2	TLC	10E3/ul	Day 28	27/10/2010	4.83	.	4.50 - 11.00		
S176	205	Hepatitis B	Hepatitis B	2	% Basophils	%	Day 56	24/11/2010	1.5	Yes	0.0 - 1.0	H	NCS
S176	205	Hepatitis B	Hepatitis B	2	% Eosinophils	%	Day 56	24/11/2010	3.1	.	1.0 - 6.0		
S176	205	Hepatitis B	Hepatitis B	2	% Lymphocytes	%	Day 56	24/11/2010	33.7	.	20.0 - 45.0		
S176	205	Hepatitis B	Hepatitis B	2	% Monocytes	%	Day 56	24/11/2010	6.1	.	2.0 - 10.0		
S176	205	Hepatitis B	Hepatitis B	2	% Neutrophils	%	Day 56	24/11/2010	55.5	.	40.0 - 75.0		
S176	205	Hepatitis B	Hepatitis B	2	Haematocrit	%	Day 56	24/11/2010	46.9	.	40.0 - 54.0		
S176	205	Hepatitis B	Hepatitis B	2	Haemoglobin	gm / dl	Day 56	24/11/2010	15.7	.	13.5 - 18.0		
S176	205	Hepatitis B	Hepatitis B	2	MCH	pg	Day 56	24/11/2010	29.3	.	27.0 - 31.0		
S176	205	Hepatitis B	Hepatitis B	2	MCHC	gm %	Day 56	24/11/2010	33.5	.	32.0 - 36.0		

Subject No.	Randomisation Number	Study Group	Dosage	Dose	Haematology Parameters	Unit	Day	Date of sample Collection	Results	Out of Range	Reference Range	Flag	Clinical Significance **
S176	205	Hepatitis B	Hepatitis B	2	MCV	fL	Day 56	24/11/2010	87.4	.	80.0- 96.0		
S176	205	Hepatitis B	Hepatitis B	2	Platelet Count	10E3/ul	Day 56	24/11/2010	282	.	150 - 400		
S176	205	Hepatitis B	Hepatitis B	2	RBC count	10E6/ul	Day 56	24/11/2010	5.36	.	4.60 - 6.20		
S176	205	Hepatitis B	Hepatitis B	2	TLC	10E3/ul	Day 56	24/11/2010	5.51	.	4.50 - 11.00		
S176	205	Hepatitis B	Hepatitis B	3	% Basophils	%	Day 180	28/03/2011	0	.	0.0 - 1.0		
S176	205	Hepatitis B	Hepatitis B	3	% Eosinophils	%	Day 180	28/03/2011	3	.	1.0 - 6.0		
S176	205	Hepatitis B	Hepatitis B	3	% Lymphocytes	%	Day 180	28/03/2011	30	.	20.0 - 45.0		
S176	205	Hepatitis B	Hepatitis B	3	% Monocytes	%	Day 180	28/03/2011	5	.	2.0 - 10.0		
S176	205	Hepatitis B	Hepatitis B	3	% Neutrophils	%	Day 180	28/03/2011	62	.	40.0 - 75.0		
S176	205	Hepatitis B	Hepatitis B	3	Haematocrit	%	Day 180	28/03/2011	42.2	.	40.0 - 54.0		
S176	205	Hepatitis B	Hepatitis B	3	Haemoglobin	gm / dl	Day 180	28/03/2011	14.8	.	13.5 - 18.0		
S176	205	Hepatitis B	Hepatitis B	3	MCH	pg	Day 180	28/03/2011	30.2	.	27.0 - 31.0		
S176	205	Hepatitis B	Hepatitis B	3	MCHC	gm %	Day 180	28/03/2011	35.2	.	32.0 - 36.0		
S176	205	Hepatitis B	Hepatitis B	3	MCV	fL	Day 180	28/03/2011	86	.	80.0- 96.0		
S176	205	Hepatitis B	Hepatitis B	3	Platelet Count	10E3/ul	Day 180	28/03/2011	252	.	150 - 400		
S176	205	Hepatitis B	Hepatitis B	3	RBC count	10E6/ul	Day 180	28/03/2011	4.91	.	4.60 - 6.20		
S176	205	Hepatitis B	Hepatitis B	3	TLC	10E3/ul	Day 180	28/03/2011	4.29	Yes	4.50 - 11.00	L	NCS
S176	205	Hepatitis B	Hepatitis B	3	% Basophils	%	Day 208	25/04/2011	1.5	Yes	0.0 - 1.0	H	NCS
S176	205	Hepatitis B	Hepatitis B	3	% Eosinophils	%	Day 208	25/04/2011	2.9	.	1.0 - 6.0		
S176	205	Hepatitis B	Hepatitis B	3	% Lymphocytes	%	Day 208	25/04/2011	29	.	20.0 - 45.0		
S176	205	Hepatitis B	Hepatitis B	3	% Monocytes	%	Day 208	25/04/2011	6.3	.	2.0 - 10.0		
S176	205	Hepatitis B	Hepatitis B	3	% Neutrophils	%	Day 208	25/04/2011	60.3	.	40.0 - 75.0		
S176	205	Hepatitis B	Hepatitis B	3	Haematocrit	%	Day 208	25/04/2011	44.6	.	40.0 - 54.0		
S176	205	Hepatitis B	Hepatitis B	3	Haemoglobin	gm / dl	Day 208	25/04/2011	15.7	.	13.5 - 18.0		
S176	205	Hepatitis B	Hepatitis B	3	MCH	pg	Day 208	25/04/2011	31	.	27.0 - 31.0		
S176	205	Hepatitis B	Hepatitis B	3	MCHC	gm %	Day 208	25/04/2011	35.3	.	32.0 - 36.0		
S176	205	Hepatitis B	Hepatitis B	3	MCV	fL	Day 208	25/04/2011	87.9	.	80.0- 96.0		
S176	205	Hepatitis B	Hepatitis B	3	Platelet Count	10E3/ul	Day 208	25/04/2011	257	.	150 - 400		
S176	205	Hepatitis B	Hepatitis B	3	RBC count	10E6/ul	Day 208	25/04/2011	5.07	.	4.60 - 6.20		

Subject No.	Randomisation Number	Study Group	Dosage	Dose	Haematology Parameters	Unit	Day	Date of sample Collection	Results	Out of Range	Reference Range	Flag	Clinical Significance **
S176	205	Hepatitis B	Hepatitis B	3	TLC	10E3/ul	Day 208	25/04/2011	5.01	.	4.50 - 11.00		
S176	205	Hepatitis B	Hepatitis B	3	% Basophils	%	Day 365	28/09/2011	0	.	0.0 - 1.0		
S176	205	Hepatitis B	Hepatitis B	3	% Eosinophils	%	Day 365	28/09/2011	2	.	1.0 - 6.0		
S176	205	Hepatitis B	Hepatitis B	3	% Lymphocytes	%	Day 365	28/09/2011	24	.	20.0 - 45.0		
S176	205	Hepatitis B	Hepatitis B	3	% Monocytes	%	Day 365	28/09/2011	6	.	2.0 - 10.0		
S176	205	Hepatitis B	Hepatitis B	3	% Neutrophils	%	Day 365	28/09/2011	68	.	40.0 - 75.0		
S176	205	Hepatitis B	Hepatitis B	3	Haematocrit	%	Day 365	28/09/2011	42.9	.	40.0 - 54.0		
S176	205	Hepatitis B	Hepatitis B	3	Haemoglobin	gm / dl	Day 365	28/09/2011	15.1	.	13.5 - 18.0		
S176	205	Hepatitis B	Hepatitis B	3	MCH	pg	Day 365	28/09/2011	30.5	.	27.0 - 31.0		
S176	205	Hepatitis B	Hepatitis B	3	MCHC	gm %	Day 365	28/09/2011	35.1	.	32.0 - 36.0		
S176	205	Hepatitis B	Hepatitis B	3	MCV	fL	Day 365	28/09/2011	87	.	80.0- 96.0		
S176	205	Hepatitis B	Hepatitis B	3	Platelet Count	10E3/ul	Day 365	28/09/2011	258	.	150 - 400		
S176	205	Hepatitis B	Hepatitis B	3	RBC count	10E6/ul	Day 365	28/09/2011	4.94	.	4.60 - 6.20		
S176	205	Hepatitis B	Hepatitis B	3	TLC	10E3/ul	Day 365	28/09/2011	5.2	.	4.50 - 11.00		
S179	207	Hepatitis B	Hepatitis B		% Basophils	%	Day -14 to Day -1	22/09/2010	1	.	0.0 - 1.0		
S179	207	Hepatitis B	Hepatitis B		% Eosinophils	%	Day -14 to Day -1	22/09/2010	1.3	.	1.0 - 6.0		
S179	207	Hepatitis B	Hepatitis B		% Lymphocytes	%	Day -14 to Day -1	22/09/2010	29.5	.	20.0 - 45.0		
S179	207	Hepatitis B	Hepatitis B		% Monocytes	%	Day -14 to Day -1	22/09/2010	3.9	.	2.0 - 10.0		
S179	207	Hepatitis B	Hepatitis B		% Neutrophils	%	Day -14 to Day -1	22/09/2010	64.3	.	40.0 - 75.0		
S179	207	Hepatitis B	Hepatitis B		Haematocrit	%	Day -14 to Day -1	22/09/2010	45.4	.	40.0 - 54.0		
S179	207	Hepatitis B	Hepatitis B		Haemoglobin	gm / dl	Day -14 to Day -1	22/09/2010	15.2	.	13.5 - 18.0		
S179	207	Hepatitis B	Hepatitis B		MCH	pg	Day -14 to Day -1	22/09/2010	28.9	.	27.0 - 31.0		
S179	207	Hepatitis B	Hepatitis B		MCHC	gm %	Day -14 to Day -1	22/09/2010	33.5	.	32.0 - 36.0		
S179	207	Hepatitis B	Hepatitis B		MCV	fL	Day -14 to Day -1	22/09/2010	86	.	80.0- 96.0		
S179	207	Hepatitis B	Hepatitis B		Platelet Count	10E3/ul	Day -14 to Day -1	22/09/2010	230	.	150 - 400		
S179	207	Hepatitis B	Hepatitis B		RBC count	10E6/ul	Day -14 to Day -1	22/09/2010	5.28	.	4.60 - 6.20		
S179	207	Hepatitis B	Hepatitis B		TLC	10E3/ul	Day -14 to Day -1	22/09/2010	6.67	.	4.50 - 11.00		
S179	207	Hepatitis B	Hepatitis B	2	% Basophils	%	Day 28	27/10/2010	0.7	.	0.0 - 1.0		
S179	207	Hepatitis B	Hepatitis B	2	% Eosinophils	%	Day 28	27/10/2010	1.9	.	1.0 - 6.0		

Subject No.	Randomisation Number	Study Group	Dosage	Dose	Haematology Parameters	Unit	Day	Date of sample Collection	Results	Out of Range	Reference Range	Flag	Clinical Significance **
S179	207	Hepatitis B	Hepatitis B	2	% Lymphocytes	%	Day 28	27/10/2010	33.5	.	20.0 - 45.0		
S179	207	Hepatitis B	Hepatitis B	2	% Monocytes	%	Day 28	27/10/2010	3.7	.	2.0 - 10.0		
S179	207	Hepatitis B	Hepatitis B	2	% Neutrophils	%	Day 28	27/10/2010	60.2	.	40.0 - 75.0		
S179	207	Hepatitis B	Hepatitis B	2	Haematocrit	%	Day 28	27/10/2010	48.8	.	40.0 - 54.0		
S179	207	Hepatitis B	Hepatitis B	2	Haemoglobin	gm / dl	Day 28	27/10/2010	15.9	.	13.5 - 18.0		
S179	207	Hepatitis B	Hepatitis B	2	MCH	pg	Day 28	27/10/2010	28.3	.	27.0 - 31.0		
S179	207	Hepatitis B	Hepatitis B	2	MCHC	gm %	Day 28	27/10/2010	32.5	.	32.0 - 36.0		
S179	207	Hepatitis B	Hepatitis B	2	MCV	fL	Day 28	27/10/2010	86.9	.	80.0- 96.0		
S179	207	Hepatitis B	Hepatitis B	2	Platelet Count	10E3/ul	Day 28	27/10/2010	290	.	150 - 400		
S179	207	Hepatitis B	Hepatitis B	2	RBC count	10E6/ul	Day 28	27/10/2010	5.61	.	4.60 - 6.20		
S179	207	Hepatitis B	Hepatitis B	2	TLC	10E3/ul	Day 28	27/10/2010	6.4	.	4.50 - 11.00		
S179	207	Hepatitis B	Hepatitis B	2	% Basophils	%	Day 56	24/11/2010	0.6	.	0.0 - 1.0		
S179	207	Hepatitis B	Hepatitis B	2	% Eosinophils	%	Day 56	24/11/2010	2.7	.	1.0 - 6.0		
S179	207	Hepatitis B	Hepatitis B	2	% Lymphocytes	%	Day 56	24/11/2010	35.5	.	20.0 - 45.0		
S179	207	Hepatitis B	Hepatitis B	2	% Monocytes	%	Day 56	24/11/2010	5	.	2.0 - 10.0		
S179	207	Hepatitis B	Hepatitis B	2	% Neutrophils	%	Day 56	24/11/2010	56.1	.	40.0 - 75.0		
S179	207	Hepatitis B	Hepatitis B	2	Haematocrit	%	Day 56	24/11/2010	48.1	.	40.0 - 54.0		
S179	207	Hepatitis B	Hepatitis B	2	Haemoglobin	gm / dl	Day 56	24/11/2010	15.6	.	13.5 - 18.0		
S179	207	Hepatitis B	Hepatitis B	2	MCH	pg	Day 56	24/11/2010	28	.	27.0 - 31.0		
S179	207	Hepatitis B	Hepatitis B	2	MCHC	gm %	Day 56	24/11/2010	32.4	.	32.0 - 36.0		
S179	207	Hepatitis B	Hepatitis B	2	MCV	fL	Day 56	24/11/2010	86.5	.	80.0- 96.0		
S179	207	Hepatitis B	Hepatitis B	2	Platelet Count	10E3/ul	Day 56	24/11/2010	247	.	150 - 400		
S179	207	Hepatitis B	Hepatitis B	2	RBC count	10E6/ul	Day 56	24/11/2010	5.56	.	4.60 - 6.20		
S179	207	Hepatitis B	Hepatitis B	2	TLC	10E3/ul	Day 56	24/11/2010	7.38	.	4.50 - 11.00		
S179	207	Hepatitis B	Hepatitis B	3	% Basophils	%	Day 180	28/03/2011	1.1	Yes	0.0 - 1.0	H	NCS
S179	207	Hepatitis B	Hepatitis B	3	% Eosinophils	%	Day 180	28/03/2011	2.4	.	1.0 - 6.0		
S179	207	Hepatitis B	Hepatitis B	3	% Lymphocytes	%	Day 180	28/03/2011	32.6	.	20.0 - 45.0		
S179	207	Hepatitis B	Hepatitis B	3	% Monocytes	%	Day 180	28/03/2011	5.4	.	2.0 - 10.0		
S179	207	Hepatitis B	Hepatitis B	3	% Neutrophils	%	Day 180	28/03/2011	58.4	.	40.0 - 75.0		

Subject No.	Randomisation Number	Study Group	Dosage	Dose	Haematology Parameters	Unit	Day	Date of sample Collection	Results	Out of Range	Reference Range	Flag	Clinical Significance **
S179	207	Hepatitis B	Hepatitis B	3	Haematocrit	%	Day 180	28/03/2011	46.9	.	40.0 - 54.0		
S179	207	Hepatitis B	Hepatitis B	3	Haemoglobin	gm / dl	Day 180	28/03/2011	15.8	.	13.5 - 18.0		
S179	207	Hepatitis B	Hepatitis B	3	MCH	pg	Day 180	28/03/2011	28.6	.	27.0 - 31.0		
S179	207	Hepatitis B	Hepatitis B	3	MCHC	gm %	Day 180	28/03/2011	33.6	.	32.0 - 36.0		
S179	207	Hepatitis B	Hepatitis B	3	MCV	fL	Day 180	28/03/2011	85	.	80.0- 96.0		
S179	207	Hepatitis B	Hepatitis B	3	Platelet Count	10E3/ul	Day 180	28/03/2011	257	.	150 - 400		
S179	207	Hepatitis B	Hepatitis B	3	RBC count	10E6/ul	Day 180	28/03/2011	5.52	.	4.60 - 6.20		
S179	207	Hepatitis B	Hepatitis B	3	TLC	10E3/ul	Day 180	28/03/2011	6.75	.	4.50 - 11.00		
S179	207	Hepatitis B	Hepatitis B	3	% Basophils	%	Day 208	25/04/2011	0.9	.	0.0 - 1.0		
S179	207	Hepatitis B	Hepatitis B	3	% Eosinophils	%	Day 208	25/04/2011	1.7	.	1.0 - 6.0		
S179	207	Hepatitis B	Hepatitis B	3	% Lymphocytes	%	Day 208	25/04/2011	31.4	.	20.0 - 45.0		
S179	207	Hepatitis B	Hepatitis B	3	% Monocytes	%	Day 208	25/04/2011	6.1	.	2.0 - 10.0		
S179	207	Hepatitis B	Hepatitis B	3	% Neutrophils	%	Day 208	25/04/2011	59.9	.	40.0 - 75.0		
S179	207	Hepatitis B	Hepatitis B	3	Haematocrit	%	Day 208	25/04/2011	47.6	.	40.0 - 54.0		
S179	207	Hepatitis B	Hepatitis B	3	Haemoglobin	gm / dl	Day 208	25/04/2011	15.9	.	13.5 - 18.0		
S179	207	Hepatitis B	Hepatitis B	3	MCH	pg	Day 208	25/04/2011	28.9	.	27.0 - 31.0		
S179	207	Hepatitis B	Hepatitis B	3	MCHC	gm %	Day 208	25/04/2011	33.5	.	32.0 - 36.0		
S179	207	Hepatitis B	Hepatitis B	3	MCV	fL	Day 208	25/04/2011	86.4	.	80.0- 96.0		
S179	207	Hepatitis B	Hepatitis B	3	Platelet Count	10E3/ul	Day 208	25/04/2011	279	.	150 - 400		
S179	207	Hepatitis B	Hepatitis B	3	RBC count	10E6/ul	Day 208	25/04/2011	5.5	.	4.60 - 6.20		
S179	207	Hepatitis B	Hepatitis B	3	TLC	10E3/ul	Day 208	25/04/2011	6.95	.	4.50 - 11.00		
S179	207	Hepatitis B	Hepatitis B	3	% Basophils	%	Day 365	28/09/2011	1	.	0.0 - 1.0		
S179	207	Hepatitis B	Hepatitis B	3	% Eosinophils	%	Day 365	28/09/2011	1.8	.	1.0 - 6.0		
S179	207	Hepatitis B	Hepatitis B	3	% Lymphocytes	%	Day 365	28/09/2011	26.9	.	20.0 - 45.0		
S179	207	Hepatitis B	Hepatitis B	3	% Monocytes	%	Day 365	28/09/2011	4.1	.	2.0 - 10.0		
S179	207	Hepatitis B	Hepatitis B	3	% Neutrophils	%	Day 365	28/09/2011	66.2	.	40.0 - 75.0		
S179	207	Hepatitis B	Hepatitis B	3	Haematocrit	%	Day 365	28/09/2011	46.2	.	40.0 - 54.0		
S179	207	Hepatitis B	Hepatitis B	3	Haemoglobin	gm / dl	Day 365	28/09/2011	15.7	.	13.5 - 18.0		
S179	207	Hepatitis B	Hepatitis B	3	MCH	pg	Day 365	28/09/2011	29.7	.	27.0 - 31.0		

Subject No.	Randomisation Number	Study Group	Dosage	Dose	Haematology Parameters	Unit	Day	Date of sample Collection	Results	Out of Range	Reference Range	Flag	Clinical Significance **
S179	207	Hepatitis B	Hepatitis B	3	MCHC	gm %	Day 365	28/09/2011	33.9	.	32.0 - 36.0		
S179	207	Hepatitis B	Hepatitis B	3	MCV	fL	Day 365	28/09/2011	87.7	.	80.0- 96.0		
S179	207	Hepatitis B	Hepatitis B	3	Platelet Count	10E3/ul	Day 365	28/09/2011	262	.	150 - 400		
S179	207	Hepatitis B	Hepatitis B	3	RBC count	10E6/ul	Day 365	28/09/2011	5.27	.	4.60 - 6.20		
S179	207	Hepatitis B	Hepatitis B	3	TLC	10E3/ul	Day 365	28/09/2011	7.25	.	4.50 - 11.00		
S182	208	JAIVAC-1	Dosage B		% Basophils	%	Day -14 to Day -1	21/09/2010	0.8	.	0.0 - 1.0		
S182	208	JAIVAC-1	Dosage B		% Eosinophils	%	Day -14 to Day -1	21/09/2010	2.1	.	1.0 - 6.0		
S182	208	JAIVAC-1	Dosage B		% Lymphocytes	%	Day -14 to Day -1	21/09/2010	30.9	.	20.0 - 45.0		
S182	208	JAIVAC-1	Dosage B		% Monocytes	%	Day -14 to Day -1	21/09/2010	5.3	.	2.0 - 10.0		
S182	208	JAIVAC-1	Dosage B		% Neutrophils	%	Day -14 to Day -1	21/09/2010	60.8	.	40.0 - 75.0		
S182	208	JAIVAC-1	Dosage B		Haematocrit	%	Day -14 to Day -1	21/09/2010	50.4	.	40.0 - 54.0		
S182	208	JAIVAC-1	Dosage B		Haemoglobin	gm / dl	Day -14 to Day -1	21/09/2010	17.1	.	13.5 - 18.0		
S182	208	JAIVAC-1	Dosage B		MCH	pg	Day -14 to Day -1	21/09/2010	28.8	.	27.0 - 31.0		
S182	208	JAIVAC-1	Dosage B		MCHC	gm %	Day -14 to Day -1	21/09/2010	33.9	.	32.0 - 36.0		
S182	208	JAIVAC-1	Dosage B		MCV	fL	Day -14 to Day -1	21/09/2010	84.8	.	80.0- 96.0		
S182	208	JAIVAC-1	Dosage B		Platelet Count	10E3/ul	Day -14 to Day -1	21/09/2010	288	.	150 - 400		
S182	208	JAIVAC-1	Dosage B		RBC count	10E6/ul	Day -14 to Day -1	21/09/2010	5.95	.	4.60 - 6.20		
S182	208	JAIVAC-1	Dosage B		TLC	10E3/ul	Day -14 to Day -1	21/09/2010	5.67	.	4.50 - 11.00		
S182	208	JAIVAC-1	Dosage B	2	% Basophils	%	Day 28	27/10/2010	0.7	.	0.0 - 1.0		
S182	208	JAIVAC-1	Dosage B	2	% Eosinophils	%	Day 28	27/10/2010	5.3	.	1.0 - 6.0		
S182	208	JAIVAC-1	Dosage B	2	% Lymphocytes	%	Day 28	27/10/2010	32.6	.	20.0 - 45.0		
S182	208	JAIVAC-1	Dosage B	2	% Monocytes	%	Day 28	27/10/2010	8.2	.	2.0 - 10.0		
S182	208	JAIVAC-1	Dosage B	2	% Neutrophils	%	Day 28	27/10/2010	53.2	.	40.0 - 75.0		
S182	208	JAIVAC-1	Dosage B	2	Haematocrit	%	Day 28	27/10/2010	50.9	.	40.0 - 54.0		
S182	208	JAIVAC-1	Dosage B	2	Haemoglobin	gm / dl	Day 28	27/10/2010	16.6	.	13.5 - 18.0		
S182	208	JAIVAC-1	Dosage B	2	MCH	pg	Day 28	27/10/2010	28	.	27.0 - 31.0		
S182	208	JAIVAC-1	Dosage B	2	MCHC	gm %	Day 28	27/10/2010	32.6	.	32.0 - 36.0		
S182	208	JAIVAC-1	Dosage B	2	MCV	fL	Day 28	27/10/2010	85.8	.	80.0- 96.0		
S182	208	JAIVAC-1	Dosage B	2	Platelet Count	10E3/ul	Day 28	27/10/2010	381	.	150 - 400		

Subject No.	Randomisation Number	Study Group	Dosage	Dose	Haematology Parameters	Unit	Day	Date of sample Collection	Results	Out of Range	Reference Range	Flag	Clinical Significance **
S182	208	JAIVAC-1	Dosage B	2	RBC count	10E6/ul	Day 28	27/10/2010	5.93	.	4.60 - 6.20		
S182	208	JAIVAC-1	Dosage B	2	TLC	10E3/ul	Day 28	27/10/2010	5.31	.	4.50 - 11.00		
S182	208	JAIVAC-1	Dosage B	2	% Basophils	%	Day 56	24/11/2010	0.5	.	0.0 - 1.0		
S182	208	JAIVAC-1	Dosage B	2	% Eosinophils	%	Day 56	24/11/2010	2.9	.	1.0 - 6.0		
S182	208	JAIVAC-1	Dosage B	2	% Lymphocytes	%	Day 56	24/11/2010	10.4	Yes	20.0 - 45.0	L	NCS
S182	208	JAIVAC-1	Dosage B	2	% Monocytes	%	Day 56	24/11/2010	6.8	.	2.0 - 10.0		
S182	208	JAIVAC-1	Dosage B	2	% Neutrophils	%	Day 56	24/11/2010	79.4	Yes	40.0 - 75.0	H	NCS
S182	208	JAIVAC-1	Dosage B	2	Haematocrit	%	Day 56	24/11/2010	53.8	.	40.0 - 54.0		
S182	208	JAIVAC-1	Dosage B	2	Haemoglobin	gm / dl	Day 56	24/11/2010	17.5	.	13.5 - 18.0		
S182	208	JAIVAC-1	Dosage B	2	MCH	pg	Day 56	24/11/2010	28.4	.	27.0 - 31.0		
S182	208	JAIVAC-1	Dosage B	2	MCHC	gm %	Day 56	24/11/2010	32.5	.	32.0 - 36.0		
S182	208	JAIVAC-1	Dosage B	2	MCV	fL	Day 56	24/11/2010	87.4	.	80.0- 96.0		
S182	208	JAIVAC-1	Dosage B	2	Platelet Count	10E3/ul	Day 56	24/11/2010	273	.	150 - 400		
S182	208	JAIVAC-1	Dosage B	2	RBC count	10E6/ul	Day 56	24/11/2010	6.15	.	4.60 - 6.20		
S182	208	JAIVAC-1	Dosage B	2	TLC	10E3/ul	Day 56	24/11/2010	7.91	.	4.50 - 11.00		
S182	208	JAIVAC-1	Dosage B	3	% Basophils	%	Day 180	28/03/2011	0.7	.	0.0 - 1.0		
S182	208	JAIVAC-1	Dosage B	3	% Eosinophils	%	Day 180	28/03/2011	2	.	1.0 - 6.0		
S182	208	JAIVAC-1	Dosage B	3	% Lymphocytes	%	Day 180	28/03/2011	25.9	.	20.0 - 45.0		
S182	208	JAIVAC-1	Dosage B	3	% Monocytes	%	Day 180	28/03/2011	6.8	.	2.0 - 10.0		
S182	208	JAIVAC-1	Dosage B	3	% Neutrophils	%	Day 180	28/03/2011	64.5	.	40.0 - 75.0		
S182	208	JAIVAC-1	Dosage B	3	Haematocrit	%	Day 180	28/03/2011	49.1	.	40.0 - 54.0		
S182	208	JAIVAC-1	Dosage B	3	Haemoglobin	gm / dl	Day 180	28/03/2011	17	.	13.5 - 18.0		
S182	208	JAIVAC-1	Dosage B	3	MCH	pg	Day 180	28/03/2011	28.9	.	27.0 - 31.0		
S182	208	JAIVAC-1	Dosage B	3	MCHC	gm %	Day 180	28/03/2011	34.6	.	32.0 - 36.0		
S182	208	JAIVAC-1	Dosage B	3	MCV	fL	Day 180	28/03/2011	83.7	.	80.0- 96.0		
S182	208	JAIVAC-1	Dosage B	3	Platelet Count	10E3/ul	Day 180	28/03/2011	296	.	150 - 400		
S182	208	JAIVAC-1	Dosage B	3	RBC count	10E6/ul	Day 180	28/03/2011	5.87	.	4.60 - 6.20		
S182	208	JAIVAC-1	Dosage B	3	TLC	10E3/ul	Day 180	28/03/2011	4.91	.	4.50 - 11.00		
S182	208	JAIVAC-1	Dosage B	3	% Basophils	%	Day 208	25/04/2011	1.1	Yes	0.0 - 1.0	H	NCS

Subject No.	Randomisation Number	Study Group	Dosage	Dose	Haematology Parameters	Unit	Day	Date of sample Collection	Results	Out of Range	Reference Range	Flag	Clinical Significance **
S182	208	JAIVAC-1	Dosage B	3	% Eosinophils	%	Day 208	25/04/2011	1.6	.	1.0 - 6.0		
S182	208	JAIVAC-1	Dosage B	3	% Lymphocytes	%	Day 208	25/04/2011	24.3	.	20.0 - 45.0		
S182	208	JAIVAC-1	Dosage B	3	% Monocytes	%	Day 208	25/04/2011	6.5	.	2.0 - 10.0		
S182	208	JAIVAC-1	Dosage B	3	% Neutrophils	%	Day 208	25/04/2011	66.4	.	40.0 - 75.0		
S182	208	JAIVAC-1	Dosage B	3	Haematocrit	%	Day 208	25/04/2011	52	.	40.0 - 54.0		
S182	208	JAIVAC-1	Dosage B	3	Haemoglobin	gm / dl	Day 208	25/04/2011	17.3	.	13.5 - 18.0		
S182	208	JAIVAC-1	Dosage B	3	MCH	pg	Day 208	25/04/2011	29.7	.	27.0 - 31.0		
S182	208	JAIVAC-1	Dosage B	3	MCHC	gm %	Day 208	25/04/2011	33.4	.	32.0 - 36.0		
S182	208	JAIVAC-1	Dosage B	3	MCV	fL	Day 208	25/04/2011	88.9	.	80.0- 96.0		
S182	208	JAIVAC-1	Dosage B	3	Platelet Count	10E3/ul	Day 208	25/04/2011	312	.	150 - 400		
S182	208	JAIVAC-1	Dosage B	3	RBC count	10E6/ul	Day 208	25/04/2011	5.84	.	4.60 - 6.20		
S182	208	JAIVAC-1	Dosage B	3	TLC	10E3/ul	Day 208	25/04/2011	5.76	.	4.50 - 11.00		
S182	208	JAIVAC-1	Dosage B	3	% Basophils	%	Day 365	29/09/2011	2.4	Yes	0.0 - 1.0	H	NCS
S182	208	JAIVAC-1	Dosage B	3	% Eosinophils	%	Day 365	29/09/2011	2.8	.	1.0 - 6.0		
S182	208	JAIVAC-1	Dosage B	3	% Lymphocytes	%	Day 365	29/09/2011	16.1	Yes	20.0 - 45.0	L	NCS
S182	208	JAIVAC-1	Dosage B	3	% Monocytes	%	Day 365	29/09/2011	5	.	2.0 - 10.0		
S182	208	JAIVAC-1	Dosage B	3	% Neutrophils	%	Day 365	29/09/2011	73.8	.	40.0 - 75.0		
S182	208	JAIVAC-1	Dosage B	3	Haematocrit	%	Day 365	29/09/2011	45.3	.	40.0 - 54.0		
S182	208	JAIVAC-1	Dosage B	3	Haemoglobin	gm / dl	Day 365	29/09/2011	16.1	.	13.5 - 18.0		
S182	208	JAIVAC-1	Dosage B	3	MCH	pg	Day 365	29/09/2011	29.8	.	27.0 - 31.0		
S182	208	JAIVAC-1	Dosage B	3	MCHC	gm %	Day 365	29/09/2011	35.6	.	32.0 - 36.0		
S182	208	JAIVAC-1	Dosage B	3	MCV	fL	Day 365	29/09/2011	83.7	.	80.0- 96.0		
S182	208	JAIVAC-1	Dosage B	3	Platelet Count	10E3/ul	Day 365	29/09/2011	279	.	150 - 400		
S182	208	JAIVAC-1	Dosage B	3	RBC count	10E6/ul	Day 365	29/09/2011	5.41	.	4.60 - 6.20		
S182	208	JAIVAC-1	Dosage B	3	TLC	10E3/ul	Day 365	29/09/2011	5.07	.	4.50 - 11.00		
S185	202	JAIVAC-1	Dosage B		% Basophils	%	Day -14 to Day -1	21/09/2010	1	.	0.0 - 1.0		
S185	202	JAIVAC-1	Dosage B		% Eosinophils	%	Day -14 to Day -1	21/09/2010	2.8	.	1.0 - 6.0		
S185	202	JAIVAC-1	Dosage B		% Lymphocytes	%	Day -14 to Day -1	21/09/2010	37.2	.	20.0 - 45.0		
S185	202	JAIVAC-1	Dosage B		% Monocytes	%	Day -14 to Day -1	21/09/2010	4.8	.	2.0 - 10.0		

Subject No.	Randomisation Number	Study Group	Dosage	Dose	Haematology Parameters	Unit	Day	Date of sample Collection	Results	Out of Range	Reference Range	Flag	Clinical Significance **
S185	202	JAIVAC-1	Dosage B		% Neutrophils	%	Day -14 to Day -1	21/09/2010	54.2	.	40.0 - 75.0		
S185	202	JAIVAC-1	Dosage B		Haematocrit	%	Day -14 to Day -1	21/09/2010	45.6	.	40.0 - 54.0		
S185	202	JAIVAC-1	Dosage B		Haemoglobin	gm / dl	Day -14 to Day -1	21/09/2010	15.7	.	13.5 - 18.0		
S185	202	JAIVAC-1	Dosage B		MCH	pg	Day -14 to Day -1	21/09/2010	29.6	.	27.0 - 31.0		
S185	202	JAIVAC-1	Dosage B		MCHC	gm %	Day -14 to Day -1	21/09/2010	34.4	.	32.0 - 36.0		
S185	202	JAIVAC-1	Dosage B		MCV	fL	Day -14 to Day -1	21/09/2010	86	.	80.0- 96.0		
S185	202	JAIVAC-1	Dosage B		Platelet Count	10E3/ul	Day -14 to Day -1	21/09/2010	188	.	150 - 400		
S185	202	JAIVAC-1	Dosage B		RBC count	10E6/ul	Day -14 to Day -1	21/09/2010	5.31	.	4.60 - 6.20		
S185	202	JAIVAC-1	Dosage B		TLC	10E3/ul	Day -14 to Day -1	21/09/2010	7.04	.	4.50 - 11.00		
S185	202	JAIVAC-1	Dosage B	2	% Basophils	%	Day 28	26/10/2010	1.5	Yes	0.0 - 1.0	H	NCS
S185	202	JAIVAC-1	Dosage B	2	% Eosinophils	%	Day 28	26/10/2010	3.7	.	1.0 - 6.0		
S185	202	JAIVAC-1	Dosage B	2	% Lymphocytes	%	Day 28	26/10/2010	40.8	.	20.0 - 45.0		
S185	202	JAIVAC-1	Dosage B	2	% Monocytes	%	Day 28	26/10/2010	5.7	.	2.0 - 10.0		
S185	202	JAIVAC-1	Dosage B	2	% Neutrophils	%	Day 28	26/10/2010	48.3	.	40.0 - 75.0		
S185	202	JAIVAC-1	Dosage B	2	Haematocrit	%	Day 28	26/10/2010	49.6	.	40.0 - 54.0		
S185	202	JAIVAC-1	Dosage B	2	Haemoglobin	gm / dl	Day 28	26/10/2010	17.1	.	13.5 - 18.0		
S185	202	JAIVAC-1	Dosage B	2	MCH	pg	Day 28	26/10/2010	29.6	.	27.0 - 31.0		
S185	202	JAIVAC-1	Dosage B	2	MCHC	gm %	Day 28	26/10/2010	34.5	.	32.0 - 36.0		
S185	202	JAIVAC-1	Dosage B	2	MCV	fL	Day 28	26/10/2010	85.8	.	80.0- 96.0		
S185	202	JAIVAC-1	Dosage B	2	Platelet Count	10E3/ul	Day 28	26/10/2010	223	.	150 - 400		
S185	202	JAIVAC-1	Dosage B	2	RBC count	10E6/ul	Day 28	26/10/2010	5.79	.	4.60 - 6.20		
S185	202	JAIVAC-1	Dosage B	2	TLC	10E3/ul	Day 28	26/10/2010	6.42	.	4.50 - 11.00		
S185	202	JAIVAC-1	Dosage B	2	% Basophils	%	Day 56	23/11/2010	1.1	Yes	0.0 - 1.0	H	NCS
S185	202	JAIVAC-1	Dosage B	2	% Eosinophils	%	Day 56	23/11/2010	5.6	.	1.0 - 6.0		
S185	202	JAIVAC-1	Dosage B	2	% Lymphocytes	%	Day 56	23/11/2010	40.7	.	20.0 - 45.0		
S185	202	JAIVAC-1	Dosage B	2	% Monocytes	%	Day 56	23/11/2010	5.1	.	2.0 - 10.0		
S185	202	JAIVAC-1	Dosage B	2	% Neutrophils	%	Day 56	23/11/2010	47.6	.	40.0 - 75.0		
S185	202	JAIVAC-1	Dosage B	2	Haematocrit	%	Day 56	23/11/2010	48.9	.	40.0 - 54.0		
S185	202	JAIVAC-1	Dosage B	2	Haemoglobin	gm / dl	Day 56	23/11/2010	16.5	.	13.5 - 18.0		

Subject No.	Randomisation Number	Study Group	Dosage	Dose	Haematology Parameters	Unit	Day	Date of sample Collection	Results	Out of Range	Reference Range	Flag	Clinical Significance **
S185	202	JAIVAC-1	Dosage B	2	MCH	pg	Day 56	23/11/2010	28.7	.	27.0 - 31.0		
S185	202	JAIVAC-1	Dosage B	2	MCHC	gm %	Day 56	23/11/2010	33.8	.	32.0 - 36.0		
S185	202	JAIVAC-1	Dosage B	2	MCV	fL	Day 56	23/11/2010	85.1	.	80.0- 96.0		
S185	202	JAIVAC-1	Dosage B	2	Platelet Count	10E3/ul	Day 56	23/11/2010	201	.	150 - 400		
S185	202	JAIVAC-1	Dosage B	2	RBC count	10E6/ul	Day 56	23/11/2010	5.74	.	4.60 - 6.20		
S185	202	JAIVAC-1	Dosage B	2	TLC	10E3/ul	Day 56	23/11/2010	5.73	.	4.50 - 11.00		
S185	202	JAIVAC-1	Dosage B	3	% Basophils	%	Day 180	28/03/2011	0.8	.	0.0 - 1.0		
S185	202	JAIVAC-1	Dosage B	3	% Eosinophils	%	Day 180	28/03/2011	3	.	1.0 - 6.0		
S185	202	JAIVAC-1	Dosage B	3	% Lymphocytes	%	Day 180	28/03/2011	40.4	.	20.0 - 45.0		
S185	202	JAIVAC-1	Dosage B	3	% Monocytes	%	Day 180	28/03/2011	6.3	.	2.0 - 10.0		
S185	202	JAIVAC-1	Dosage B	3	% Neutrophils	%	Day 180	28/03/2011	49.6	.	40.0 - 75.0		
S185	202	JAIVAC-1	Dosage B	3	Haematocrit	%	Day 180	28/03/2011	45.3	.	40.0 - 54.0		
S185	202	JAIVAC-1	Dosage B	3	Haemoglobin	gm / dl	Day 180	28/03/2011	15.5	.	13.5 - 18.0		
S185	202	JAIVAC-1	Dosage B	3	MCH	pg	Day 180	28/03/2011	28.4	.	27.0 - 31.0		
S185	202	JAIVAC-1	Dosage B	3	MCHC	gm %	Day 180	28/03/2011	34.3	.	32.0 - 36.0		
S185	202	JAIVAC-1	Dosage B	3	MCV	fL	Day 180	28/03/2011	82.7	.	80.0- 96.0		
S185	202	JAIVAC-1	Dosage B	3	Platelet Count	10E3/ul	Day 180	28/03/2011	221	.	150 - 400		
S185	202	JAIVAC-1	Dosage B	3	RBC count	10E6/ul	Day 180	28/03/2011	5.48	.	4.60 - 6.20		
S185	202	JAIVAC-1	Dosage B	3	TLC	10E3/ul	Day 180	28/03/2011	6.54	.	4.50 - 11.00		
S185	202	JAIVAC-1	Dosage B	3	% Basophils	%	Day 208	25/04/2011	0.7	.	0.0 - 1.0		
S185	202	JAIVAC-1	Dosage B	3	% Eosinophils	%	Day 208	25/04/2011	4.4	.	1.0 - 6.0		
S185	202	JAIVAC-1	Dosage B	3	% Lymphocytes	%	Day 208	25/04/2011	35.4	.	20.0 - 45.0		
S185	202	JAIVAC-1	Dosage B	3	% Monocytes	%	Day 208	25/04/2011	4.7	.	2.0 - 10.0		
S185	202	JAIVAC-1	Dosage B	3	% Neutrophils	%	Day 208	25/04/2011	54.8	.	40.0 - 75.0		
S185	202	JAIVAC-1	Dosage B	3	Haematocrit	%	Day 208	25/04/2011	51.5	.	40.0 - 54.0		
S185	202	JAIVAC-1	Dosage B	3	Haemoglobin	gm / dl	Day 208	25/04/2011	17.4	.	13.5 - 18.0		
S185	202	JAIVAC-1	Dosage B	3	MCH	pg	Day 208	25/04/2011	29.4	.	27.0 - 31.0		
S185	202	JAIVAC-1	Dosage B	3	MCHC	gm %	Day 208	25/04/2011	33.7	.	32.0 - 36.0		
S185	202	JAIVAC-1	Dosage B	3	MCV	fL	Day 208	25/04/2011	87.1	.	80.0- 96.0		

Subject No.	Randomisation Number	Study Group	Dosage	Dose	Haematology Parameters	Unit	Day	Date of sample Collection	Results	Out of Range	Reference Range	Flag	Clinical Significance **
S185	202	JAIVAC-1	Dosage B	3	Platelet Count	10E3/ul	Day 208	25/04/2011	231	.	150 - 400		
S185	202	JAIVAC-1	Dosage B	3	RBC count	10E6/ul	Day 208	25/04/2011	5.92	.	4.60 - 6.20		
S185	202	JAIVAC-1	Dosage B	3	TLC	10E3/ul	Day 208	25/04/2011	6.57	.	4.50 - 11.00		
S185	202	JAIVAC-1	Dosage B	3	% Basophils	%	Day 365	28/09/2011	1.2	Yes	0.0 - 1.0	H	NCS
S185	202	JAIVAC-1	Dosage B	3	% Eosinophils	%	Day 365	28/09/2011	4	.	1.0 - 6.0		
S185	202	JAIVAC-1	Dosage B	3	% Lymphocytes	%	Day 365	28/09/2011	41.3	.	20.0 - 45.0		
S185	202	JAIVAC-1	Dosage B	3	% Monocytes	%	Day 365	28/09/2011	5.5	.	2.0 - 10.0		
S185	202	JAIVAC-1	Dosage B	3	% Neutrophils	%	Day 365	28/09/2011	48	.	40.0 - 75.0		
S185	202	JAIVAC-1	Dosage B	3	Haematocrit	%	Day 365	28/09/2011	48.9	.	40.0 - 54.0		
S185	202	JAIVAC-1	Dosage B	3	Haemoglobin	gm / dl	Day 365	28/09/2011	17	.	13.5 - 18.0		
S185	202	JAIVAC-1	Dosage B	3	MCH	pg	Day 365	28/09/2011	29.9	.	27.0 - 31.0		
S185	202	JAIVAC-1	Dosage B	3	MCHC	gm %	Day 365	28/09/2011	34.7	.	32.0 - 36.0		
S185	202	JAIVAC-1	Dosage B	3	MCV	fL	Day 365	28/09/2011	86.1	.	80.0- 96.0		
S185	202	JAIVAC-1	Dosage B	3	Platelet Count	10E3/ul	Day 365	28/09/2011	224	.	150 - 400		
S185	202	JAIVAC-1	Dosage B	3	RBC count	10E6/ul	Day 365	28/09/2011	5.68	.	4.60 - 6.20		
S185	202	JAIVAC-1	Dosage B	3	TLC	10E3/ul	Day 365	28/09/2011	6.67	.	4.50 - 11.00		
S186	201	JAIVAC-1	Dosage B		% Basophils	%	Day -14 to Day -1	21/09/2010	1.5	Yes	0.0 - 1.0	H	NCS
S186	201	JAIVAC-1	Dosage B		% Eosinophils	%	Day -14 to Day -1	21/09/2010	7	.	1.0 - 6.0	H	
S186	201	JAIVAC-1	Dosage B		% Lymphocytes	%	Day -14 to Day -1	21/09/2010	34.9	.	20.0 - 45.0		
S186	201	JAIVAC-1	Dosage B		% Monocytes	%	Day -14 to Day -1	21/09/2010	4.7	.	2.0 - 10.0		
S186	201	JAIVAC-1	Dosage B		% Neutrophils	%	Day -14 to Day -1	21/09/2010	51.9	.	40.0 - 75.0		
S186	201	JAIVAC-1	Dosage B		Haematocrit	%	Day -14 to Day -1	21/09/2010	40.7	.	40.0 - 54.0		
S186	201	JAIVAC-1	Dosage B		Haemoglobin	gm / dl	Day -14 to Day -1	21/09/2010	13.9	.	13.5 - 18.0		
S186	201	JAIVAC-1	Dosage B		MCH	pg	Day -14 to Day -1	21/09/2010	28.2	.	27.0 - 31.0		
S186	201	JAIVAC-1	Dosage B		MCHC	gm %	Day -14 to Day -1	21/09/2010	34	.	32.0 - 36.0		
S186	201	JAIVAC-1	Dosage B		MCV	fL	Day -14 to Day -1	21/09/2010	82.8	.	80.0- 96.0		
S186	201	JAIVAC-1	Dosage B		Platelet Count	10E3/ul	Day -14 to Day -1	21/09/2010	235	.	150 - 400		
S186	201	JAIVAC-1	Dosage B		RBC count	10E6/ul	Day -14 to Day -1	21/09/2010	4.92	.	4.60 - 6.20		
S186	201	JAIVAC-1	Dosage B		TLC	10E3/ul	Day -14 to Day -1	21/09/2010	6.6	.	4.50 - 11.00		

Subject No.	Randomisation Number	Study Group	Dosage	Dose	Haematology Parameters	Unit	Day	Date of sample Collection	Results	Out of Range	Reference Range	Flag	Clinical Significance **
S186	201	JAIVAC-1	Dosage B	2	% Basophils	%	Day 28	26/10/2010	1.3	Yes	0.0 - 1.0	H	NCS
S186	201	JAIVAC-1	Dosage B	2	% Eosinophils	%	Day 28	26/10/2010	7.9	Yes	1.0 - 6.0	H	NCS
S186	201	JAIVAC-1	Dosage B	2	% Lymphocytes	%	Day 28	26/10/2010	36.8	.	20.0 - 45.0		
S186	201	JAIVAC-1	Dosage B	2	% Monocytes	%	Day 28	26/10/2010	6.7	.	2.0 - 10.0		
S186	201	JAIVAC-1	Dosage B	2	% Neutrophils	%	Day 28	26/10/2010	47.3	.	40.0 - 75.0		
S186	201	JAIVAC-1	Dosage B	2	Haematocrit	%	Day 28	26/10/2010	40.6	.	40.0 - 54.0		
S186	201	JAIVAC-1	Dosage B	2	Haemoglobin	gm / dl	Day 28	26/10/2010	14	.	13.5 - 18.0		
S186	201	JAIVAC-1	Dosage B	2	MCH	pg	Day 28	26/10/2010	28	.	27.0 - 31.0		
S186	201	JAIVAC-1	Dosage B	2	MCHC	gm %	Day 28	26/10/2010	34.5	.	32.0 - 36.0		
S186	201	JAIVAC-1	Dosage B	2	MCV	fL	Day 28	26/10/2010	81.1	.	80.0- 96.0		
S186	201	JAIVAC-1	Dosage B	2	Platelet Count	10E3/ul	Day 28	26/10/2010	292	.	150 - 400		
S186	201	JAIVAC-1	Dosage B	2	RBC count	10E6/ul	Day 28	26/10/2010	5.01	.	4.60 - 6.20		
S186	201	JAIVAC-1	Dosage B	2	TLC	10E3/ul	Day 28	26/10/2010	6.53	.	4.50 - 11.00		
S186	201	JAIVAC-1	Dosage B	2	% Basophils	%	Day 56	23/11/2010	1.1	Yes	0.0 - 1.0	H	NCS
S186	201	JAIVAC-1	Dosage B	2	% Eosinophils	%	Day 56	23/11/2010	7.8	Yes	1.0 - 6.0	H	NCS
S186	201	JAIVAC-1	Dosage B	2	% Lymphocytes	%	Day 56	23/11/2010	29.3	.	20.0 - 45.0		
S186	201	JAIVAC-1	Dosage B	2	% Monocytes	%	Day 56	23/11/2010	5	.	2.0 - 10.0		
S186	201	JAIVAC-1	Dosage B	2	% Neutrophils	%	Day 56	23/11/2010	56.8	.	40.0 - 75.0		
S186	201	JAIVAC-1	Dosage B	2	Haematocrit	%	Day 56	23/11/2010	42.4	.	40.0 - 54.0		
S186	201	JAIVAC-1	Dosage B	2	Haemoglobin	gm / dl	Day 56	23/11/2010	14.3	.	13.5 - 18.0		
S186	201	JAIVAC-1	Dosage B	2	MCH	pg	Day 56	23/11/2010	27.4	.	27.0 - 31.0		
S186	201	JAIVAC-1	Dosage B	2	MCHC	gm %	Day 56	23/11/2010	33.8	.	32.0 - 36.0		
S186	201	JAIVAC-1	Dosage B	2	MCV	fL	Day 56	23/11/2010	81	.	80.0- 96.0		
S186	201	JAIVAC-1	Dosage B	2	Platelet Count	10E3/ul	Day 56	23/11/2010	247	.	150 - 400		
S186	201	JAIVAC-1	Dosage B	2	RBC count	10E6/ul	Day 56	23/11/2010	5.24	.	4.60 - 6.20		
S186	201	JAIVAC-1	Dosage B	2	TLC	10E3/ul	Day 56	23/11/2010	7.13	.	4.50 - 11.00		
S186	201	JAIVAC-1	Dosage B	3	% Basophils	%	Day 180	28/03/2011	0.9	.	0.0 - 1.0		
S186	201	JAIVAC-1	Dosage B	3	% Eosinophils	%	Day 180	28/03/2011	7.9	Yes	1.0 - 6.0	H	NCS
S186	201	JAIVAC-1	Dosage B	3	% Lymphocytes	%	Day 180	28/03/2011	37.1	.	20.0 - 45.0		

Subject No.	Randomisation Number	Study Group	Dosage	Dose	Haematology Parameters	Unit	Day	Date of sample Collection	Results	Out of Range	Reference Range	Flag	Clinical Significance **
S186	201	JAIVAC-1	Dosage B	3	% Monocytes	%	Day 180	28/03/2011	5.8	.	2.0 - 10.0		
S186	201	JAIVAC-1	Dosage B	3	% Neutrophils	%	Day 180	28/03/2011	48.2	.	40.0 - 75.0		
S186	201	JAIVAC-1	Dosage B	3	Haematocrit	%	Day 180	28/03/2011	42.1	.	40.0 - 54.0		
S186	201	JAIVAC-1	Dosage B	3	Haemoglobin	gm / dl	Day 180	28/03/2011	14.2	.	13.5 - 18.0		
S186	201	JAIVAC-1	Dosage B	3	MCH	pg	Day 180	28/03/2011	27.2	.	27.0 - 31.0		
S186	201	JAIVAC-1	Dosage B	3	MCHC	gm %	Day 180	28/03/2011	33.7	.	32.0 - 36.0		
S186	201	JAIVAC-1	Dosage B	3	MCV	fL	Day 180	28/03/2011	80.8	.	80.0- 96.0		
S186	201	JAIVAC-1	Dosage B	3	Platelet Count	10E3/ul	Day 180	28/03/2011	252	.	150 - 400		
S186	201	JAIVAC-1	Dosage B	3	RBC count	10E6/ul	Day 180	28/03/2011	5.21	.	4.60 - 6.20		
S186	201	JAIVAC-1	Dosage B	3	TLC	10E3/ul	Day 180	28/03/2011	6.43	.	4.50 - 11.00		
S186	201	JAIVAC-1	Dosage B	3	% Basophils	%	Day 208	25/04/2011	1.2	Yes	0.0 - 1.0	H	NCS
S186	201	JAIVAC-1	Dosage B	3	% Eosinophils	%	Day 208	25/04/2011	7.1	Yes	1.0 - 6.0	H	NCS
S186	201	JAIVAC-1	Dosage B	3	% Lymphocytes	%	Day 208	25/04/2011	26.5	.	20.0 - 45.0		
S186	201	JAIVAC-1	Dosage B	3	% Monocytes	%	Day 208	25/04/2011	6	.	2.0 - 10.0		
S186	201	JAIVAC-1	Dosage B	3	% Neutrophils	%	Day 208	25/04/2011	59.2	.	40.0 - 75.0		
S186	201	JAIVAC-1	Dosage B	3	Haematocrit	%	Day 208	25/04/2011	41.5	.	40.0 - 54.0		
S186	201	JAIVAC-1	Dosage B	3	Haemoglobin	gm / dl	Day 208	25/04/2011	14.4	.	13.5 - 18.0		
S186	201	JAIVAC-1	Dosage B	3	MCH	pg	Day 208	25/04/2011	28.7	.	27.0 - 31.0		
S186	201	JAIVAC-1	Dosage B	3	MCHC	gm %	Day 208	25/04/2011	34.6	.	32.0 - 36.0		
S186	201	JAIVAC-1	Dosage B	3	MCV	fL	Day 208	25/04/2011	82.9	.	80.0- 96.0		
S186	201	JAIVAC-1	Dosage B	3	Platelet Count	10E3/ul	Day 208	25/04/2011	260	.	150 - 400		
S186	201	JAIVAC-1	Dosage B	3	RBC count	10E6/ul	Day 208	25/04/2011	5.01	.	4.60 - 6.20		
S186	201	JAIVAC-1	Dosage B	3	TLC	10E3/ul	Day 208	25/04/2011	7.5	.	4.50 - 11.00		
S186	201	JAIVAC-1	Dosage B	3	% Basophils	%	Day 365	28/09/2011	1.6	Yes	0.0 - 1.0	H	NCS
S186	201	JAIVAC-1	Dosage B	3	% Eosinophils	%	Day 365	28/09/2011	7.3	Yes	1.0 - 6.0	H	NCS
S186	201	JAIVAC-1	Dosage B	3	% Lymphocytes	%	Day 365	28/09/2011	30.7	.	20.0 - 45.0		
S186	201	JAIVAC-1	Dosage B	3	% Monocytes	%	Day 365	28/09/2011	6.5	.	2.0 - 10.0		
S186	201	JAIVAC-1	Dosage B	3	% Neutrophils	%	Day 365	28/09/2011	53.9	.	40.0 - 75.0		
S186	201	JAIVAC-1	Dosage B	3	Haematocrit	%	Day 365	28/09/2011	41.8	.	40.0 - 54.0		

Subject No.	Randomisation Number	Study Group	Dosage	Dose	Haematology Parameters	Unit	Day	Date of sample Collection	Results	Out of Range	Reference Range	Flag	Clinical Significance **
S186	201	JAIVAC-1	Dosage B	3	Haemoglobin	gm / dl	Day 365	28/09/2011	14.5	.	13.5 - 18.0		
S186	201	JAIVAC-1	Dosage B	3	MCH	pg	Day 365	28/09/2011	30.2	.	27.0 - 31.0		
S186	201	JAIVAC-1	Dosage B	3	MCHC	gm %	Day 365	28/09/2011	34.8	.	32.0 - 36.0		
S186	201	JAIVAC-1	Dosage B	3	MCV	fL	Day 365	28/09/2011	86.8	.	80.0- 96.0		
S186	201	JAIVAC-1	Dosage B	3	Platelet Count	10E3/ul	Day 365	28/09/2011	258	.	150 - 400		
S186	201	JAIVAC-1	Dosage B	3	RBC count	10E6/ul	Day 365	28/09/2011	4.81	.	4.60 - 6.20		
S186	201	JAIVAC-1	Dosage B	3	TLC	10E3/ul	Day 365	28/09/2011	7.41	.	4.50 - 11.00		
S192	203	JAIVAC-1	Dosage B		% Basophils	%	Day -14 to Day -1	23/09/2010	0.7	.	0.0 - 1.0		
S192	203	JAIVAC-1	Dosage B		% Eosinophils	%	Day -14 to Day -1	23/09/2010	3.4	.	1.0 - 6.0		
S192	203	JAIVAC-1	Dosage B		% Lymphocytes	%	Day -14 to Day -1	23/09/2010	27.4	.	20.0 - 45.0		
S192	203	JAIVAC-1	Dosage B		% Monocytes	%	Day -14 to Day -1	23/09/2010	5.5	.	2.0 - 10.0		
S192	203	JAIVAC-1	Dosage B		% Neutrophils	%	Day -14 to Day -1	23/09/2010	63.1	.	40.0 - 75.0		
S192	203	JAIVAC-1	Dosage B		Haematocrit	%	Day -14 to Day -1	23/09/2010	44.1	.	40.0 - 54.0		
S192	203	JAIVAC-1	Dosage B		Haemoglobin	gm / dl	Day -14 to Day -1	23/09/2010	14.9	.	13.5 - 18.0		
S192	203	JAIVAC-1	Dosage B		MCH	pg	Day -14 to Day -1	23/09/2010	31	.	27.0 - 31.0		
S192	203	JAIVAC-1	Dosage B		MCHC	gm %	Day -14 to Day -1	23/09/2010	33.8	.	32.0 - 36.0		
S192	203	JAIVAC-1	Dosage B		MCV	fL	Day -14 to Day -1	23/09/2010	91.7	.	80.0- 96.0		
S192	203	JAIVAC-1	Dosage B		Platelet Count	10E3/ul	Day -14 to Day -1	23/09/2010	227	.	150 - 400		
S192	203	JAIVAC-1	Dosage B		RBC count	10E6/ul	Day -14 to Day -1	23/09/2010	4.81	.	4.60 - 6.20		
S192	203	JAIVAC-1	Dosage B		TLC	10E3/ul	Day -14 to Day -1	23/09/2010	6.42	.	4.50 - 11.00		
S192	203	JAIVAC-1	Dosage B	2	% Basophils	%	Day 28	26/10/2010	1.1	Yes	0.0 - 1.0	H	NCS
S192	203	JAIVAC-1	Dosage B	2	% Eosinophils	%	Day 28	26/10/2010	3.9	.	1.0 - 6.0		
S192	203	JAIVAC-1	Dosage B	2	% Lymphocytes	%	Day 28	26/10/2010	31.1	.	20.0 - 45.0		
S192	203	JAIVAC-1	Dosage B	2	% Monocytes	%	Day 28	26/10/2010	6	.	2.0 - 10.0		
S192	203	JAIVAC-1	Dosage B	2	% Neutrophils	%	Day 28	26/10/2010	57.9	.	40.0 - 75.0		
S192	203	JAIVAC-1	Dosage B	2	Haematocrit	%	Day 28	26/10/2010	41.4	.	40.0 - 54.0		
S192	203	JAIVAC-1	Dosage B	2	Haemoglobin	gm / dl	Day 28	26/10/2010	13.9	.	13.5 - 18.0		
S192	203	JAIVAC-1	Dosage B	2	MCH	pg	Day 28	26/10/2010	30.1	.	27.0 - 31.0		
S192	203	JAIVAC-1	Dosage B	2	MCHC	gm %	Day 28	26/10/2010	33.6	.	32.0 - 36.0		

Subject No.	Randomisation Number	Study Group	Dosage	Dose	Haematology Parameters	Unit	Day	Date of sample Collection	Results	Out of Range	Reference Range	Flag	Clinical Significance **
S192	203	JAIVAC-1	Dosage B	2	MCV	fL	Day 28	26/10/2010	89.6	.	80.0- 96.0		
S192	203	JAIVAC-1	Dosage B	2	Platelet Count	10E3/ul	Day 28	26/10/2010	291	.	150 - 400		
S192	203	JAIVAC-1	Dosage B	2	RBC count	10E6/ul	Day 28	26/10/2010	4.62	.	4.60 - 6.20		
S192	203	JAIVAC-1	Dosage B	2	TLC	10E3/ul	Day 28	26/10/2010	4.75	.	4.50 - 11.00		
S192	203	JAIVAC-1	Dosage B	2	% Basophils	%	Day 56	23/11/2010	0.7	.	0.0 - 1.0		
S192	203	JAIVAC-1	Dosage B	2	% Eosinophils	%	Day 56	23/11/2010	13.6	Yes	1.0 - 6.0	H	NCS
S192	203	JAIVAC-1	Dosage B	2	% Lymphocytes	%	Day 56	23/11/2010	38.9	.	20.0 - 45.0		
S192	203	JAIVAC-1	Dosage B	2	% Monocytes	%	Day 56	23/11/2010	6.6	.	2.0 - 10.0		
S192	203	JAIVAC-1	Dosage B	2	% Neutrophils	%	Day 56	23/11/2010	40.3	.	40.0 - 75.0		
S192	203	JAIVAC-1	Dosage B	2	Haematocrit	%	Day 56	23/11/2010	41.9	.	40.0 - 54.0		
S192	203	JAIVAC-1	Dosage B	2	Haemoglobin	gm / dl	Day 56	23/11/2010	13.6	.	13.5 - 18.0		
S192	203	JAIVAC-1	Dosage B	2	MCH	pg	Day 56	23/11/2010	29.4	.	27.0 - 31.0		
S192	203	JAIVAC-1	Dosage B	2	MCHC	gm %	Day 56	23/11/2010	32.5	.	32.0 - 36.0		
S192	203	JAIVAC-1	Dosage B	2	MCV	fL	Day 56	23/11/2010	90.3	.	80.0- 96.0		
S192	203	JAIVAC-1	Dosage B	2	Platelet Count	10E3/ul	Day 56	23/11/2010	226	.	150 - 400		
S192	203	JAIVAC-1	Dosage B	2	RBC count	10E6/ul	Day 56	23/11/2010	4.64	.	4.60 - 6.20		
S192	203	JAIVAC-1	Dosage B	2	TLC	10E3/ul	Day 56	23/11/2010	4.32	Yes	4.50 - 11.00	L	NCS
S192	203	JAIVAC-1	Dosage B	3	% Basophils	%	Day 180	28/03/2011	1	.	0.0 - 1.0		
S192	203	JAIVAC-1	Dosage B	3	% Eosinophils	%	Day 180	28/03/2011	4.7	.	1.0 - 6.0		
S192	203	JAIVAC-1	Dosage B	3	% Lymphocytes	%	Day 180	28/03/2011	33.6	.	20.0 - 45.0		
S192	203	JAIVAC-1	Dosage B	3	% Monocytes	%	Day 180	28/03/2011	7.1	.	2.0 - 10.0		
S192	203	JAIVAC-1	Dosage B	3	% Neutrophils	%	Day 180	28/03/2011	53.7	.	40.0 - 75.0		
S192	203	JAIVAC-1	Dosage B	3	Haematocrit	%	Day 180	28/03/2011	43.6	.	40.0 - 54.0		
S192	203	JAIVAC-1	Dosage B	3	Haemoglobin	gm / dl	Day 180	28/03/2011	14.8	.	13.5 - 18.0		
S192	203	JAIVAC-1	Dosage B	3	MCH	pg	Day 180	28/03/2011	31	.	27.0 - 31.0		
S192	203	JAIVAC-1	Dosage B	3	MCHC	gm %	Day 180	28/03/2011	33.8	.	32.0 - 36.0		
S192	203	JAIVAC-1	Dosage B	3	MCV	fL	Day 180	28/03/2011	91.5	.	80.0- 96.0		
S192	203	JAIVAC-1	Dosage B	3	Platelet Count	10E3/ul	Day 180	28/03/2011	238	.	150 - 400		
S192	203	JAIVAC-1	Dosage B	3	RBC count	10E6/ul	Day 180	28/03/2011	4.76	.	4.60 - 6.20		

Subject No.	Randomisation Number	Study Group	Dosage	Dose	Haematology Parameters	Unit	Day	Date of sample Collection	Results	Out of Range	Reference Range	Flag	Clinical Significance **
S192	203	JAIVAC-1	Dosage B	3	TLC	10E3/ul	Day 180	28/03/2011	4.29	Yes	4.50 - 11.00	L	NCS
S192	203	JAIVAC-1	Dosage B	3	% Basophils	%	Day 208	25/04/2011	0.8	.	0.0 - 1.0		
S192	203	JAIVAC-1	Dosage B	3	% Eosinophils	%	Day 208	25/04/2011	6	.	1.0 - 6.0		
S192	203	JAIVAC-1	Dosage B	3	% Lymphocytes	%	Day 208	25/04/2011	34.4	.	20.0 - 45.0		
S192	203	JAIVAC-1	Dosage B	3	% Monocytes	%	Day 208	25/04/2011	5.1	.	2.0 - 10.0		
S192	203	JAIVAC-1	Dosage B	3	% Neutrophils	%	Day 208	25/04/2011	53.7	.	40.0 - 75.0		
S192	203	JAIVAC-1	Dosage B	3	Haematocrit	%	Day 208	25/04/2011	49.3	.	40.0 - 54.0		
S192	203	JAIVAC-1	Dosage B	3	Haemoglobin	gm / dl	Day 208	25/04/2011	16.4	.	13.5 - 18.0		
S192	203	JAIVAC-1	Dosage B	3	MCH	pg	Day 208	25/04/2011	31.6	Yes	27.0 - 31.0	H	NCS
S192	203	JAIVAC-1	Dosage B	3	MCHC	gm %	Day 208	25/04/2011	33.3	.	32.0 - 36.0		
S192	203	JAIVAC-1	Dosage B	3	MCV	fL	Day 208	25/04/2011	95	.	80.0- 96.0		
S192	203	JAIVAC-1	Dosage B	3	Platelet Count	10E3/ul	Day 208	25/04/2011	273	.	150 - 400		
S192	203	JAIVAC-1	Dosage B	3	RBC count	10E6/ul	Day 208	25/04/2011	5.19	.	4.60 - 6.20		
S192	203	JAIVAC-1	Dosage B	3	TLC	10E3/ul	Day 208	25/04/2011	4.89	.	4.50 - 11.00		
S192	203	JAIVAC-1	Dosage B	3	% Basophils	%	Day 365	28/09/2011	1.5	Yes	0.0 - 1.0	H	NCS
S192	203	JAIVAC-1	Dosage B	3	% Eosinophils	%	Day 365	28/09/2011	5.5	.	1.0 - 6.0		
S192	203	JAIVAC-1	Dosage B	3	% Lymphocytes	%	Day 365	28/09/2011	24	.	20.0 - 45.0		
S192	203	JAIVAC-1	Dosage B	3	% Monocytes	%	Day 365	28/09/2011	5.8	.	2.0 - 10.0		
S192	203	JAIVAC-1	Dosage B	3	% Neutrophils	%	Day 365	28/09/2011	63.1	.	40.0 - 75.0		
S192	203	JAIVAC-1	Dosage B	3	Haematocrit	%	Day 365	28/09/2011	40.8	.	40.0 - 54.0		
S192	203	JAIVAC-1	Dosage B	3	Haemoglobin	gm / dl	Day 365	28/09/2011	13.7	.	13.5 - 18.0		
S192	203	JAIVAC-1	Dosage B	3	MCH	pg	Day 365	28/09/2011	31.3	Yes	27.0 - 31.0	H	NCS
S192	203	JAIVAC-1	Dosage B	3	MCHC	gm %	Day 365	28/09/2011	33.5	.	32.0 - 36.0		
S192	203	JAIVAC-1	Dosage B	3	MCV	fL	Day 365	28/09/2011	93.4	.	80.0- 96.0		
S192	203	JAIVAC-1	Dosage B	3	Platelet Count	10E3/ul	Day 365	28/09/2011	347	.	150 - 400		
S192	203	JAIVAC-1	Dosage B	3	RBC count	10E6/ul	Day 365	28/09/2011	4.37	Yes	4.60 - 6.20	L	NCS
S192	203	JAIVAC-1	Dosage B	3	TLC	10E3/ul	Day 365	28/09/2011	5.31	.	4.50 - 11.00		
S193	215	JAIVAC-1	Dosage B		% Basophils	%	Day -14 to Day -1	24/09/2010	1.2	Yes	0.0 - 1.0	H	NCS
S193	215	JAIVAC-1	Dosage B		% Eosinophils	%	Day -14 to Day -1	24/09/2010	0.2	.	1.0 - 6.0	L	

Subject No.	Randomisation Number	Study Group	Dosage	Dose	Haematology Parameters	Unit	Day	Date of sample Collection	Results	Out of Range	Reference Range	Flag	Clinical Significance **
S193	215	JAIVAC-1	Dosage B		% Lymphocytes	%	Day -14 to Day -1	24/09/2010	29.4	.	20.0 - 45.0		
S193	215	JAIVAC-1	Dosage B		% Monocytes	%	Day -14 to Day -1	24/09/2010	17.4	Yes	2.0 - 10.0	H	NCS
S193	215	JAIVAC-1	Dosage B		% Neutrophils	%	Day -14 to Day -1	24/09/2010	51.9	.	40.0 - 75.0		
S193	215	JAIVAC-1	Dosage B		Haematocrit	%	Day -14 to Day -1	24/09/2010	49.2	.	40.0 - 54.0		
S193	215	JAIVAC-1	Dosage B		Haemoglobin	gm / dl	Day -14 to Day -1	24/09/2010	16.3	.	13.5 - 18.0		
S193	215	JAIVAC-1	Dosage B		MCH	pg	Day -14 to Day -1	24/09/2010	30.2	.	27.0 - 31.0		
S193	215	JAIVAC-1	Dosage B		MCHC	gm %	Day -14 to Day -1	24/09/2010	33.1	.	32.0 - 36.0		
S193	215	JAIVAC-1	Dosage B		MCV	fL	Day -14 to Day -1	24/09/2010	91.2	.	80.0- 96.0		
S193	215	JAIVAC-1	Dosage B		Platelet Count	10E3/ul	Day -14 to Day -1	24/09/2010	347	.	150 - 400		
S193	215	JAIVAC-1	Dosage B		RBC count	10E6/ul	Day -14 to Day -1	24/09/2010	5.4	.	4.60 - 6.20		
S193	215	JAIVAC-1	Dosage B		TLC	10E3/ul	Day -14 to Day -1	24/09/2010	10.54	.	4.50 - 11.00		
S193	215	JAIVAC-1	Dosage B	2	% Basophils	%	Day 28	28/10/2010	0	.	0.0 - 1.0		
S193	215	JAIVAC-1	Dosage B	2	% Eosinophils	%	Day 28	28/10/2010	10	Yes	1.0 - 6.0	H	NCS
S193	215	JAIVAC-1	Dosage B	2	% Lymphocytes	%	Day 28	28/10/2010	27	.	20.0 - 45.0		
S193	215	JAIVAC-1	Dosage B	2	% Monocytes	%	Day 28	28/10/2010	11	Yes	2.0 - 10.0	H	NCS
S193	215	JAIVAC-1	Dosage B	2	% Neutrophils	%	Day 28	28/10/2010	52	.	40.0 - 75.0		
S193	215	JAIVAC-1	Dosage B	2	Haematocrit	%	Day 28	28/10/2010	49.3	.	40.0 - 54.0		
S193	215	JAIVAC-1	Dosage B	2	Haemoglobin	gm / dl	Day 28	28/10/2010	15.9	.	13.5 - 18.0		
S193	215	JAIVAC-1	Dosage B	2	MCH	pg	Day 28	28/10/2010	29.2	.	27.0 - 31.0		
S193	215	JAIVAC-1	Dosage B	2	MCHC	gm %	Day 28	28/10/2010	32.3	.	32.0 - 36.0		
S193	215	JAIVAC-1	Dosage B	2	MCV	fL	Day 28	28/10/2010	90.6	.	80.0- 96.0		
S193	215	JAIVAC-1	Dosage B	2	Platelet Count	10E3/ul	Day 28	28/10/2010	402	Yes	150 - 400	H	NCS
S193	215	JAIVAC-1	Dosage B	2	RBC count	10E6/ul	Day 28	28/10/2010	5.45	.	4.60 - 6.20		
S193	215	JAIVAC-1	Dosage B	2	TLC	10E3/ul	Day 28	28/10/2010	10.59	.	4.50 - 11.00		
S193	215	JAIVAC-1	Dosage B	2	% Basophils	%	Day 56	25/11/2010	0	.	0.0 - 1.0		
S193	215	JAIVAC-1	Dosage B	2	% Eosinophils	%	Day 56	25/11/2010	12	Yes	1.0 - 6.0	H	NCS
S193	215	JAIVAC-1	Dosage B	2	% Lymphocytes	%	Day 56	25/11/2010	31	.	20.0 - 45.0		
S193	215	JAIVAC-1	Dosage B	2	% Monocytes	%	Day 56	25/11/2010	6	.	2.0 - 10.0		
S193	215	JAIVAC-1	Dosage B	2	% Neutrophils	%	Day 56	25/11/2010	51	.	40.0 - 75.0		

Subject No.	Randomisation Number	Study Group	Dosage	Dose	Haematology Parameters	Unit	Day	Date of sample Collection	Results	Out of Range	Reference Range	Flag	Clinical Significance **
S193	215	JAIVAC-1	Dosage B	2	Haematocrit	%	Day 56	25/11/2010	51.6	.	40.0 - 54.0		
S193	215	JAIVAC-1	Dosage B	2	Haemoglobin	gm / dl	Day 56	25/11/2010	17	.	13.5 - 18.0		
S193	215	JAIVAC-1	Dosage B	2	MCH	pg	Day 56	25/11/2010	29.9	.	27.0 - 31.0		
S193	215	JAIVAC-1	Dosage B	2	MCHC	gm %	Day 56	25/11/2010	32.9	.	32.0 - 36.0		
S193	215	JAIVAC-1	Dosage B	2	MCV	fL	Day 56	25/11/2010	91.1	.	80.0- 96.0		
S193	215	JAIVAC-1	Dosage B	2	Platelet Count	10E3/ul	Day 56	25/11/2010	438	Yes	150 - 400	H	NCS
S193	215	JAIVAC-1	Dosage B	2	RBC count	10E6/ul	Day 56	25/11/2010	5.66	.	4.60 - 6.20		
S193	215	JAIVAC-1	Dosage B	2	TLC	10E3/ul	Day 56	25/11/2010	10.26	.	4.50 - 11.00		
S193	215	JAIVAC-1	Dosage B	3	% Basophils	%	Day 180	29/03/2011	0	.	0.0 - 1.0		
S193	215	JAIVAC-1	Dosage B	3	% Eosinophils	%	Day 180	29/03/2011	17	Yes	1.0 - 6.0	H	NCS
S193	215	JAIVAC-1	Dosage B	3	% Lymphocytes	%	Day 180	29/03/2011	18	Yes	20.0 - 45.0	L	NCS
S193	215	JAIVAC-1	Dosage B	3	% Monocytes	%	Day 180	29/03/2011	4	.	2.0 - 10.0		
S193	215	JAIVAC-1	Dosage B	3	% Neutrophils	%	Day 180	29/03/2011	61	.	40.0 - 75.0		
S193	215	JAIVAC-1	Dosage B	3	Haematocrit	%	Day 180	29/03/2011	46.1	.	40.0 - 54.0		
S193	215	JAIVAC-1	Dosage B	3	Haemoglobin	gm / dl	Day 180	29/03/2011	15.7	.	13.5 - 18.0		
S193	215	JAIVAC-1	Dosage B	3	MCH	pg	Day 180	29/03/2011	29.4	.	27.0 - 31.0		
S193	215	JAIVAC-1	Dosage B	3	MCHC	gm %	Day 180	29/03/2011	34.1	.	32.0 - 36.0		
S193	215	JAIVAC-1	Dosage B	3	MCV	fL	Day 180	29/03/2011	86.1	.	80.0- 96.0		
S193	215	JAIVAC-1	Dosage B	3	Platelet Count	10E3/ul	Day 180	29/03/2011	366	.	150 - 400		
S193	215	JAIVAC-1	Dosage B	3	RBC count	10E6/ul	Day 180	29/03/2011	5.35	.	4.60 - 6.20		
S193	215	JAIVAC-1	Dosage B	3	TLC	10E3/ul	Day 180	29/03/2011	10.95	.	4.50 - 11.00		
S193	215	JAIVAC-1	Dosage B	3	% Basophils	%	Day 208	26/04/2011	0	.	0.0 - 1.0		
S193	215	JAIVAC-1	Dosage B	3	% Eosinophils	%	Day 208	26/04/2011	17	Yes	1.0 - 6.0	H	CS
S193	215	JAIVAC-1	Dosage B	3	% Lymphocytes	%	Day 208	26/04/2011	29	.	20.0 - 45.0		
S193	215	JAIVAC-1	Dosage B	3	% Monocytes	%	Day 208	26/04/2011	7	.	2.0 - 10.0		
S193	215	JAIVAC-1	Dosage B	3	% Neutrophils	%	Day 208	26/04/2011	47	.	40.0 - 75.0		
S193	215	JAIVAC-1	Dosage B	3	Haematocrit	%	Day 208	26/04/2011	50.8	.	40.0 - 54.0		
S193	215	JAIVAC-1	Dosage B	3	Haemoglobin	gm / dl	Day 208	26/04/2011	16.8	.	13.5 - 18.0		
S193	215	JAIVAC-1	Dosage B	3	MCH	pg	Day 208	26/04/2011	29.5	.	27.0 - 31.0		

Subject No.	Randomisation Number	Study Group	Dosage	Dose	Haematology Parameters	Unit	Day	Date of sample Collection	Results	Out of Range	Reference Range	Flag	Clinical Significance **
S193	215	JAIVAC-1	Dosage B	3	MCHC	gm %	Day 208	26/04/2011	33.1	.	32.0 - 36.0		
S193	215	JAIVAC-1	Dosage B	3	MCV	fL	Day 208	26/04/2011	89.2	.	80.0- 96.0		
S193	215	JAIVAC-1	Dosage B	3	Platelet Count	10E3/ul	Day 208	26/04/2011	461	Yes	150 - 400	H	NCS
S193	215	JAIVAC-1	Dosage B	3	RBC count	10E6/ul	Day 208	26/04/2011	5.7	.	4.60 - 6.20		
S193	215	JAIVAC-1	Dosage B	3	TLC	10E3/ul	Day 208	26/04/2011	10.64	.	4.50 - 11.00		
S193	215	JAIVAC-1	Dosage B	3	% Basophils	%	Day 365	29/09/2011	1.7	Yes	0.0 - 1.0	H	NCS
S193	215	JAIVAC-1	Dosage B	3	% Eosinophils	%	Day 365	29/09/2011	0.5	Yes	1.0 - 6.0	L	NCS
S193	215	JAIVAC-1	Dosage B	3	% Lymphocytes	%	Day 365	29/09/2011	22.3	.	20.0 - 45.0		
S193	215	JAIVAC-1	Dosage B	3	% Monocytes	%	Day 365	29/09/2011	11.6	Yes	2.0 - 10.0	H	NCS
S193	215	JAIVAC-1	Dosage B	3	% Neutrophils	%	Day 365	29/09/2011	63.9	.	40.0 - 75.0		
S193	215	JAIVAC-1	Dosage B	3	Haematocrit	%	Day 365	29/09/2011	49.5	.	40.0 - 54.0		
S193	215	JAIVAC-1	Dosage B	3	Haemoglobin	gm / dl	Day 365	29/09/2011	16.9	.	13.5 - 18.0		
S193	215	JAIVAC-1	Dosage B	3	MCH	pg	Day 365	29/09/2011	29.3	.	27.0 - 31.0		
S193	215	JAIVAC-1	Dosage B	3	MCHC	gm %	Day 365	29/09/2011	34.1	.	32.0 - 36.0		
S193	215	JAIVAC-1	Dosage B	3	MCV	fL	Day 365	29/09/2011	85.8	.	80.0- 96.0		
S193	215	JAIVAC-1	Dosage B	3	Platelet Count	10E3/ul	Day 365	29/09/2011	424	Yes	150 - 400	H	NCS
S193	215	JAIVAC-1	Dosage B	3	RBC count	10E6/ul	Day 365	29/09/2011	5.77	.	4.60 - 6.20		
S193	215	JAIVAC-1	Dosage B	3	TLC	10E3/ul	Day 365	29/09/2011	11.35	Yes	4.50 - 11.00	H	CS
S195	214	JAIVAC-1	Dosage B		% Basophils	%	Day -14 to Day -1	23/09/2010	1.6	Yes	0.0 - 1.0	H	NCS
S195	214	JAIVAC-1	Dosage B		% Eosinophils	%	Day -14 to Day -1	23/09/2010	3.5	.	1.0 - 6.0		
S195	214	JAIVAC-1	Dosage B		% Lymphocytes	%	Day -14 to Day -1	23/09/2010	37.9	.	20.0 - 45.0		
S195	214	JAIVAC-1	Dosage B		% Monocytes	%	Day -14 to Day -1	23/09/2010	5.8	.	2.0 - 10.0		
S195	214	JAIVAC-1	Dosage B		% Neutrophils	%	Day -14 to Day -1	23/09/2010	51.2	.	40.0 - 75.0		
S195	214	JAIVAC-1	Dosage B		Haematocrit	%	Day -14 to Day -1	23/09/2010	43.2	.	40.0 - 54.0		
S195	214	JAIVAC-1	Dosage B		Haemoglobin	gm / dl	Day -14 to Day -1	23/09/2010	14.6	.	13.5 - 18.0		
S195	214	JAIVAC-1	Dosage B		MCH	pg	Day -14 to Day -1	23/09/2010	29.3	.	27.0 - 31.0		
S195	214	JAIVAC-1	Dosage B		MCHC	gm %	Day -14 to Day -1	23/09/2010	33.9	.	32.0 - 36.0		
S195	214	JAIVAC-1	Dosage B		MCV	fL	Day -14 to Day -1	23/09/2010	86.3	.	80.0- 96.0		
S195	214	JAIVAC-1	Dosage B		Platelet Count	10E3/ul	Day -14 to Day -1	23/09/2010	203	.	150 - 400		

Subject No.	Randomisation Number	Study Group	Dosage	Dose	Haematology Parameters	Unit	Day	Date of sample Collection	Results	Out of Range	Reference Range	Flag	Clinical Significance **
S195	214	JAIVAC-1	Dosage B		RBC count	10E6/ul	Day -14 to Day -1	23/09/2010	5	.	4.60 - 6.20		
S195	214	JAIVAC-1	Dosage B		TLC	10E3/ul	Day -14 to Day -1	23/09/2010	6.77	.	4.50 - 11.00		
S195	214	JAIVAC-1	Dosage B	2	% Basophils	%	Day 28	28/10/2010	2.1	Yes	0.0 - 1.0	H	NCS
S195	214	JAIVAC-1	Dosage B	2	% Eosinophils	%	Day 28	28/10/2010	3.7	.	1.0 - 6.0		
S195	214	JAIVAC-1	Dosage B	2	% Lymphocytes	%	Day 28	28/10/2010	37.4	.	20.0 - 45.0		
S195	214	JAIVAC-1	Dosage B	2	% Monocytes	%	Day 28	28/10/2010	6.1	.	2.0 - 10.0		
S195	214	JAIVAC-1	Dosage B	2	% Neutrophils	%	Day 28	28/10/2010	50.7	.	40.0 - 75.0		
S195	214	JAIVAC-1	Dosage B	2	Haematocrit	%	Day 28	28/10/2010	42.4	.	40.0 - 54.0		
S195	214	JAIVAC-1	Dosage B	2	Haemoglobin	gm / dl	Day 28	28/10/2010	14.2	.	13.5 - 18.0		
S195	214	JAIVAC-1	Dosage B	2	MCH	pg	Day 28	28/10/2010	29.2	.	27.0 - 31.0		
S195	214	JAIVAC-1	Dosage B	2	MCHC	gm %	Day 28	28/10/2010	33.6	.	32.0 - 36.0		
S195	214	JAIVAC-1	Dosage B	2	MCV	fL	Day 28	28/10/2010	87.1	.	80.0- 96.0		
S195	214	JAIVAC-1	Dosage B	2	Platelet Count	10E3/ul	Day 28	28/10/2010	231	.	150 - 400		
S195	214	JAIVAC-1	Dosage B	2	RBC count	10E6/ul	Day 28	28/10/2010	4.87	.	4.60 - 6.20		
S195	214	JAIVAC-1	Dosage B	2	TLC	10E3/ul	Day 28	28/10/2010	5.58	.	4.50 - 11.00		
S195	214	JAIVAC-1	Dosage B	2	% Basophils	%	Day 56	24/11/2010	0	.	0.0 - 1.0		
S195	214	JAIVAC-1	Dosage B	2	% Eosinophils	%	Day 56	24/11/2010	3	.	1.0 - 6.0		
S195	214	JAIVAC-1	Dosage B	2	% Lymphocytes	%	Day 56	24/11/2010	37	.	20.0 - 45.0		
S195	214	JAIVAC-1	Dosage B	2	% Monocytes	%	Day 56	24/11/2010	5	.	2.0 - 10.0		
S195	214	JAIVAC-1	Dosage B	2	% Neutrophils	%	Day 56	24/11/2010	55	.	40.0 - 75.0		
S195	214	JAIVAC-1	Dosage B	2	Haematocrit	%	Day 56	24/11/2010	45.1	.	40.0 - 54.0		
S195	214	JAIVAC-1	Dosage B	2	Haemoglobin	gm / dl	Day 56	24/11/2010	14.6	.	13.5 - 18.0		
S195	214	JAIVAC-1	Dosage B	2	MCH	pg	Day 56	24/11/2010	28.6	.	27.0 - 31.0		
S195	214	JAIVAC-1	Dosage B	2	MCHC	gm %	Day 56	24/11/2010	32.3	.	32.0 - 36.0		
S195	214	JAIVAC-1	Dosage B	2	MCV	fL	Day 56	24/11/2010	88.4	.	80.0- 96.0		
S195	214	JAIVAC-1	Dosage B	2	Platelet Count	10E3/ul	Day 56	24/11/2010	229	.	150 - 400		
S195	214	JAIVAC-1	Dosage B	2	RBC count	10E6/ul	Day 56	24/11/2010	5.1	.	4.60 - 6.20		
S195	214	JAIVAC-1	Dosage B	2	TLC	10E3/ul	Day 56	24/11/2010	5.33	.	4.50 - 11.00		
S195	214	JAIVAC-1	Dosage B	3	% Basophils	%	Day 180	29/03/2011	1.1	Yes	0.0 - 1.0	H	NCS

Subject No.	Randomisation Number	Study Group	Dosage	Dose	Haematology Parameters	Unit	Day	Date of sample Collection	Results	Out of Range	Reference Range	Flag	Clinical Significance **
S195	214	JAIVAC-1	Dosage B	3	% Eosinophils	%	Day 180	29/03/2011	3.4	.	1.0 - 6.0		
S195	214	JAIVAC-1	Dosage B	3	% Lymphocytes	%	Day 180	29/03/2011	37.2	.	20.0 - 45.0		
S195	214	JAIVAC-1	Dosage B	3	% Monocytes	%	Day 180	29/03/2011	6.4	.	2.0 - 10.0		
S195	214	JAIVAC-1	Dosage B	3	% Neutrophils	%	Day 180	29/03/2011	52	.	40.0 - 75.0		
S195	214	JAIVAC-1	Dosage B	3	Haematocrit	%	Day 180	29/03/2011	41.7	.	40.0 - 54.0		
S195	214	JAIVAC-1	Dosage B	3	Haemoglobin	gm / dl	Day 180	29/03/2011	14.2	.	13.5 - 18.0		
S195	214	JAIVAC-1	Dosage B	3	MCH	pg	Day 180	29/03/2011	29.1	.	27.0 - 31.0		
S195	214	JAIVAC-1	Dosage B	3	MCHC	gm %	Day 180	29/03/2011	34	.	32.0 - 36.0		
S195	214	JAIVAC-1	Dosage B	3	MCV	fL	Day 180	29/03/2011	85.7	.	80.0- 96.0		
S195	214	JAIVAC-1	Dosage B	3	Platelet Count	10E3/ul	Day 180	29/03/2011	252	.	150 - 400		
S195	214	JAIVAC-1	Dosage B	3	RBC count	10E6/ul	Day 180	29/03/2011	4.86	.	4.60 - 6.20		
S195	214	JAIVAC-1	Dosage B	3	TLC	10E3/ul	Day 180	29/03/2011	6	.	4.50 - 11.00		
S195	214	JAIVAC-1	Dosage B	3	% Basophils	%	Day 208	26/04/2011	0	.	0.0 - 1.0		
S195	214	JAIVAC-1	Dosage B	3	% Eosinophils	%	Day 208	26/04/2011	5	.	1.0 - 6.0		
S195	214	JAIVAC-1	Dosage B	3	% Lymphocytes	%	Day 208	26/04/2011	42	.	20.0 - 45.0		
S195	214	JAIVAC-1	Dosage B	3	% Monocytes	%	Day 208	26/04/2011	9	.	2.0 - 10.0		
S195	214	JAIVAC-1	Dosage B	3	% Neutrophils	%	Day 208	26/04/2011	44	.	40.0 - 75.0		
S195	214	JAIVAC-1	Dosage B	3	Haematocrit	%	Day 208	26/04/2011	47.5	.	40.0 - 54.0		
S195	214	JAIVAC-1	Dosage B	3	Haemoglobin	gm / dl	Day 208	26/04/2011	15.8	.	13.5 - 18.0		
S195	214	JAIVAC-1	Dosage B	3	MCH	pg	Day 208	26/04/2011	29.9	.	27.0 - 31.0		
S195	214	JAIVAC-1	Dosage B	3	MCHC	gm %	Day 208	26/04/2011	33.2	.	32.0 - 36.0		
S195	214	JAIVAC-1	Dosage B	3	MCV	fL	Day 208	26/04/2011	90.2	.	80.0- 96.0		
S195	214	JAIVAC-1	Dosage B	3	Platelet Count	10E3/ul	Day 208	26/04/2011	253	.	150 - 400		
S195	214	JAIVAC-1	Dosage B	3	RBC count	10E6/ul	Day 208	26/04/2011	5.27	.	4.60 - 6.20		
S195	214	JAIVAC-1	Dosage B	3	TLC	10E3/ul	Day 208	26/04/2011	4.88	.	4.50 - 11.00		
S195	214	JAIVAC-1	Dosage B	3	% Basophils	%	Day 365	29/09/2011	0	.	0.0 - 1.0		
S195	214	JAIVAC-1	Dosage B	3	% Eosinophils	%	Day 365	29/09/2011	4	.	1.0 - 6.0		
S195	214	JAIVAC-1	Dosage B	3	% Lymphocytes	%	Day 365	29/09/2011	32	.	20.0 - 45.0		
S195	214	JAIVAC-1	Dosage B	3	% Monocytes	%	Day 365	29/09/2011	7	.	2.0 - 10.0		

Subject No.	Randomisation Number	Study Group	Dosage	Dose	Haematology Parameters	Unit	Day	Date of sample Collection	Results	Out of Range	Reference Range	Flag	Clinical Significance **
S195	214	JAIVAC-1	Dosage B	3	% Neutrophils	%	Day 365	29/09/2011	57	.	40.0 - 75.0		
S195	214	JAIVAC-1	Dosage B	3	Haematocrit	%	Day 365	29/09/2011	35.4	Yes	40.0 - 54.0	L	CS
S195	214	JAIVAC-1	Dosage B	3	Haemoglobin*	gm / dl	Day 365	29/09/2011	11.8	Yes	13.5 - 18.0	L	CS
S195	214	JAIVAC-1	Dosage B	3	MCH	pg	Day 365	29/09/2011	28.5	.	27.0 - 31.0		
S195	214	JAIVAC-1	Dosage B	3	MCHC	gm %	Day 365	29/09/2011	33.5	.	32.0 - 36.0		
S195	214	JAIVAC-1	Dosage B	3	MCV	fL	Day 365	29/09/2011	85.2	.	80.0- 96.0		
S195	214	JAIVAC-1	Dosage B	3	Platelet Count	10E3/ul	Day 365	29/09/2011	308	.	150 - 400		
S195	214	JAIVAC-1	Dosage B	3	RBC count	10E6/ul	Day 365	29/09/2011	4.16	Yes	4.60 - 6.20	L	NCS
S195	214	JAIVAC-1	Dosage B	3	TLC	10E3/ul	Day 365	29/09/2011	4.57	.	4.50 - 11.00		
S202	206	JAIVAC-1	Dosage B		% Basophils	%	Day -14 to Day -1	23/09/2010	0.7	.	0.0 - 1.0		
S202	206	JAIVAC-1	Dosage B		% Eosinophils	%	Day -14 to Day -1	23/09/2010	3.8	.	1.0 - 6.0		
S202	206	JAIVAC-1	Dosage B		% Lymphocytes	%	Day -14 to Day -1	23/09/2010	23.1	.	20.0 - 45.0		
S202	206	JAIVAC-1	Dosage B		% Monocytes	%	Day -14 to Day -1	23/09/2010	4.5	.	2.0 - 10.0		
S202	206	JAIVAC-1	Dosage B		% Neutrophils	%	Day -14 to Day -1	23/09/2010	67.8	.	40.0 - 75.0		
S202	206	JAIVAC-1	Dosage B		Haematocrit	%	Day -14 to Day -1	23/09/2010	46.9	.	40.0 - 54.0		
S202	206	JAIVAC-1	Dosage B		Haemoglobin	gm / dl	Day -14 to Day -1	23/09/2010	15.9	.	13.5 - 18.0		
S202	206	JAIVAC-1	Dosage B		MCH	pg	Day -14 to Day -1	23/09/2010	29	.	27.0 - 31.0		
S202	206	JAIVAC-1	Dosage B		MCHC	gm %	Day -14 to Day -1	23/09/2010	33.9	.	32.0 - 36.0		
S202	206	JAIVAC-1	Dosage B		MCV	fL	Day -14 to Day -1	23/09/2010	85.7	.	80.0- 96.0		
S202	206	JAIVAC-1	Dosage B		Platelet Count	10E3/ul	Day -14 to Day -1	23/09/2010	261	.	150 - 400		
S202	206	JAIVAC-1	Dosage B		RBC count	10E6/ul	Day -14 to Day -1	23/09/2010	5.47	.	4.60 - 6.20		
S202	206	JAIVAC-1	Dosage B		TLC	10E3/ul	Day -14 to Day -1	23/09/2010	7.65	.	4.50 - 11.00		
S202	206	JAIVAC-1	Dosage B	2	% Basophils	%	Day 28	27/10/2010	0.5	.	0.0 - 1.0		
S202	206	JAIVAC-1	Dosage B	2	% Eosinophils	%	Day 28	27/10/2010	3.3	.	1.0 - 6.0		
S202	206	JAIVAC-1	Dosage B	2	% Lymphocytes	%	Day 28	27/10/2010	42.2	.	20.0 - 45.0		
S202	206	JAIVAC-1	Dosage B	2	% Monocytes	%	Day 28	27/10/2010	5.3	.	2.0 - 10.0		
S202	206	JAIVAC-1	Dosage B	2	% Neutrophils	%	Day 28	27/10/2010	48.7	.	40.0 - 75.0		
S202	206	JAIVAC-1	Dosage B	2	Haematocrit	%	Day 28	27/10/2010	47	.	40.0 - 54.0		
S202	206	JAIVAC-1	Dosage B	2	Haemoglobin	gm / dl	Day 28	27/10/2010	15.7	.	13.5 - 18.0		

Subject No.	Randomisation Number	Study Group	Dosage	Dose	Haematology Parameters	Unit	Day	Date of sample Collection	Results	Out of Range	Reference Range	Flag	Clinical Significance **
S202	206	JAIVAC-1	Dosage B	2	MCH	pg	Day 28	27/10/2010	29.1	.	27.0 - 31.0		
S202	206	JAIVAC-1	Dosage B	2	MCHC	gm %	Day 28	27/10/2010	33.4	.	32.0 - 36.0		
S202	206	JAIVAC-1	Dosage B	2	MCV	fL	Day 28	27/10/2010	87.1	.	80.0- 96.0		
S202	206	JAIVAC-1	Dosage B	2	Platelet Count	10E3/ul	Day 28	27/10/2010	337	.	150 - 400		
S202	206	JAIVAC-1	Dosage B	2	RBC count	10E6/ul	Day 28	27/10/2010	5.39	.	4.60 - 6.20		
S202	206	JAIVAC-1	Dosage B	2	TLC	10E3/ul	Day 28	27/10/2010	4.87	.	4.50 - 11.00		
S202	206	JAIVAC-1	Dosage B	2	% Basophils	%	Day 56	24/11/2010	0.7	.	0.0 - 1.0		
S202	206	JAIVAC-1	Dosage B	2	% Eosinophils	%	Day 56	24/11/2010	3.9	.	1.0 - 6.0		
S202	206	JAIVAC-1	Dosage B	2	% Lymphocytes	%	Day 56	24/11/2010	26.7	.	20.0 - 45.0		
S202	206	JAIVAC-1	Dosage B	2	% Monocytes	%	Day 56	24/11/2010	5.4	.	2.0 - 10.0		
S202	206	JAIVAC-1	Dosage B	2	% Neutrophils	%	Day 56	24/11/2010	63.2	.	40.0 - 75.0		
S202	206	JAIVAC-1	Dosage B	2	Haematocrit	%	Day 56	24/11/2010	51.4	.	40.0 - 54.0		
S202	206	JAIVAC-1	Dosage B	2	Haemoglobin	gm / dl	Day 56	24/11/2010	16.7	.	13.5 - 18.0		
S202	206	JAIVAC-1	Dosage B	2	MCH	pg	Day 56	24/11/2010	28.5	.	27.0 - 31.0		
S202	206	JAIVAC-1	Dosage B	2	MCHC	gm %	Day 56	24/11/2010	32.4	.	32.0 - 36.0		
S202	206	JAIVAC-1	Dosage B	2	MCV	fL	Day 56	24/11/2010	88	.	80.0- 96.0		
S202	206	JAIVAC-1	Dosage B	2	Platelet Count	10E3/ul	Day 56	24/11/2010	362	.	150 - 400		
S202	206	JAIVAC-1	Dosage B	2	RBC count	10E6/ul	Day 56	24/11/2010	5.84	.	4.60 - 6.20		
S202	206	JAIVAC-1	Dosage B	2	TLC	10E3/ul	Day 56	24/11/2010	7.17	.	4.50 - 11.00		
S202	206	JAIVAC-1	Dosage B	3	% Basophils	%	Day 180	28/03/2011	0.5	.	0.0 - 1.0		
S202	206	JAIVAC-1	Dosage B	3	% Eosinophils	%	Day 180	28/03/2011	4.3	.	1.0 - 6.0		
S202	206	JAIVAC-1	Dosage B	3	% Lymphocytes	%	Day 180	28/03/2011	31.9	.	20.0 - 45.0		
S202	206	JAIVAC-1	Dosage B	3	% Monocytes	%	Day 180	28/03/2011	6.4	.	2.0 - 10.0		
S202	206	JAIVAC-1	Dosage B	3	% Neutrophils	%	Day 180	28/03/2011	57	.	40.0 - 75.0		
S202	206	JAIVAC-1	Dosage B	3	Haematocrit	%	Day 180	28/03/2011	47.2	.	40.0 - 54.0		
S202	206	JAIVAC-1	Dosage B	3	Haemoglobin	gm / dl	Day 180	28/03/2011	15.8	.	13.5 - 18.0		
S202	206	JAIVAC-1	Dosage B	3	MCH	pg	Day 180	28/03/2011	28.6	.	27.0 - 31.0		
S202	206	JAIVAC-1	Dosage B	3	MCHC	gm %	Day 180	28/03/2011	33.4	.	32.0 - 36.0		
S202	206	JAIVAC-1	Dosage B	3	MCV	fL	Day 180	28/03/2011	85.5	.	80.0- 96.0		

Subject No.	Randomisation Number	Study Group	Dosage	Dose	Haematology Parameters	Unit	Day	Date of sample Collection	Results	Out of Range	Reference Range	Flag	Clinical Significance **
S202	206	JAIVAC-1	Dosage B	3	Platelet Count	10E3/ul	Day 180	28/03/2011	311	.	150 - 400		
S202	206	JAIVAC-1	Dosage B	3	RBC count	10E6/ul	Day 180	28/03/2011	5.53	.	4.60 - 6.20		
S202	206	JAIVAC-1	Dosage B	3	TLC	10E3/ul	Day 180	28/03/2011	5.97	.	4.50 - 11.00		
S202	206	JAIVAC-1	Dosage B	3	% Basophils	%	Day 208	25/04/2011	0.5	.	0.0 - 1.0		
S202	206	JAIVAC-1	Dosage B	3	% Eosinophils	%	Day 208	25/04/2011	4.5	.	1.0 - 6.0		
S202	206	JAIVAC-1	Dosage B	3	% Lymphocytes	%	Day 208	25/04/2011	25.9	.	20.0 - 45.0		
S202	206	JAIVAC-1	Dosage B	3	% Monocytes	%	Day 208	25/04/2011	4.3	.	2.0 - 10.0		
S202	206	JAIVAC-1	Dosage B	3	% Neutrophils	%	Day 208	25/04/2011	64.8	.	40.0 - 75.0		
S202	206	JAIVAC-1	Dosage B	3	Haematocrit	%	Day 208	25/04/2011	46.6	.	40.0 - 54.0		
S202	206	JAIVAC-1	Dosage B	3	Haemoglobin	gm / dl	Day 208	25/04/2011	15.5	.	13.5 - 18.0		
S202	206	JAIVAC-1	Dosage B	3	MCH	pg	Day 208	25/04/2011	28.8	.	27.0 - 31.0		
S202	206	JAIVAC-1	Dosage B	3	MCHC	gm %	Day 208	25/04/2011	33.2	.	32.0 - 36.0		
S202	206	JAIVAC-1	Dosage B	3	MCV	fL	Day 208	25/04/2011	87	.	80.0- 96.0		
S202	206	JAIVAC-1	Dosage B	3	Platelet Count	10E3/ul	Day 208	25/04/2011	286	.	150 - 400		
S202	206	JAIVAC-1	Dosage B	3	RBC count	10E6/ul	Day 208	25/04/2011	5.36	.	4.60 - 6.20		
S202	206	JAIVAC-1	Dosage B	3	TLC	10E3/ul	Day 208	25/04/2011	6.82	.	4.50 - 11.00		
S202	206	JAIVAC-1	Dosage B	3	% Basophils	%	Day 365	29/09/2011	1.4	Yes	0.0 - 1.0	H	NCS
S202	206	JAIVAC-1	Dosage B	3	% Eosinophils	%	Day 365	29/09/2011	5.2	.	1.0 - 6.0		
S202	206	JAIVAC-1	Dosage B	3	% Lymphocytes	%	Day 365	29/09/2011	27.3	.	20.0 - 45.0		
S202	206	JAIVAC-1	Dosage B	3	% Monocytes	%	Day 365	29/09/2011	5.5	.	2.0 - 10.0		
S202	206	JAIVAC-1	Dosage B	3	% Neutrophils	%	Day 365	29/09/2011	60.5	.	40.0 - 75.0		
S202	206	JAIVAC-1	Dosage B	3	Haematocrit	%	Day 365	29/09/2011	45.2	.	40.0 - 54.0		
S202	206	JAIVAC-1	Dosage B	3	Haemoglobin	gm / dl	Day 365	29/09/2011	15.6	.	13.5 - 18.0		
S202	206	JAIVAC-1	Dosage B	3	MCH	pg	Day 365	29/09/2011	29.4	.	27.0 - 31.0		
S202	206	JAIVAC-1	Dosage B	3	MCHC	gm %	Day 365	29/09/2011	34.6	.	32.0 - 36.0		
S202	206	JAIVAC-1	Dosage B	3	MCV	fL	Day 365	29/09/2011	85.1	.	80.0- 96.0		
S202	206	JAIVAC-1	Dosage B	3	Platelet Count	10E3/ul	Day 365	29/09/2011	297	.	150 - 400		
S202	206	JAIVAC-1	Dosage B	3	RBC count	10E6/ul	Day 365	29/09/2011	5.31	.	4.60 - 6.20		
S202	_09 –AIVAC-1	JAIVAC-1	Dosage B	3	TLC	10E3/ul	Day 365	29/09/2011	7.08	.	4.50 - 11.00		

Subject No.	Randomisation Number	Study Group	Dosage	Dose	Haematology Parameters	Unit	Day	Date of sample Collection	Results	Out of Range	Reference Range	Flag	Clinical Significance **
S203	211	Hepatitis B	Hepatitis B		% Basophils	%	Day -14 to Day -1	24/09/2010	0.8	.	0.0 - 1.0		
S203	211	Hepatitis B	Hepatitis B		% Eosinophils	%	Day -14 to Day -1	24/09/2010	2	.	1.0 - 6.0		
S203	211	Hepatitis B	Hepatitis B		% Lymphocytes	%	Day -14 to Day -1	24/09/2010	33.1	.	20.0 - 45.0		
S203	211	Hepatitis B	Hepatitis B		% Monocytes	%	Day -14 to Day -1	24/09/2010	6.1	.	2.0 - 10.0		
S203	211	Hepatitis B	Hepatitis B		% Neutrophils	%	Day -14 to Day -1	24/09/2010	58	.	40.0 - 75.0		
S203	211	Hepatitis B	Hepatitis B		Haematocrit	%	Day -14 to Day -1	24/09/2010	39.9	Yes	40.0 - 54.0	L	NCS
S203	211	Hepatitis B	Hepatitis B		Haemoglobin	gm / dl	Day -14 to Day -1	24/09/2010	13.5	.	13.5 - 18.0		
S203	211	Hepatitis B	Hepatitis B		MCH	pg	Day -14 to Day -1	24/09/2010	24.7	Yes	27.0 - 31.0	L	NCS
S203	211	Hepatitis B	Hepatitis B		MCHC	gm %	Day -14 to Day -1	24/09/2010	33.7	.	32.0 - 36.0		
S203	211	Hepatitis B	Hepatitis B		MCV	fL	Day -14 to Day -1	24/09/2010	73.3	Yes	80.0- 96.0	L	NCS
S203	211	Hepatitis B	Hepatitis B		Platelet Count	10E3/ul	Day -14 to Day -1	24/09/2010	205	.	150 - 400		
S203	211	Hepatitis B	Hepatitis B		RBC count	10E6/ul	Day -14 to Day -1	24/09/2010	5.45	.	4.60 - 6.20		
S203	211	Hepatitis B	Hepatitis B		TLC	10E3/ul	Day -14 to Day -1	24/09/2010	5.47	.	4.50 - 11.00		
S203	211	Hepatitis B	Hepatitis B	2	% Basophils	%	Day 28	28/10/2010	2.5	Yes	0.0 - 1.0	H	NCS
S203	211	Hepatitis B	Hepatitis B	2	% Eosinophils	%	Day 28	28/10/2010	2.5	.	1.0 - 6.0		
S203	211	Hepatitis B	Hepatitis B	2	% Lymphocytes	%	Day 28	28/10/2010	28.8	.	20.0 - 45.0		
S203	211	Hepatitis B	Hepatitis B	2	% Monocytes	%	Day 28	28/10/2010	5.3	.	2.0 - 10.0		
S203	211	Hepatitis B	Hepatitis B	2	% Neutrophils	%	Day 28	28/10/2010	60.9	.	40.0 - 75.0		
S203	211	Hepatitis B	Hepatitis B	2	Haematocrit	%	Day 28	28/10/2010	41.7	.	40.0 - 54.0		
S203	211	Hepatitis B	Hepatitis B	2	Haemoglobin*	gm / dl	Day 28	28/10/2010	13	Yes	13.5 - 18.0	L	NCS
S203	211	Hepatitis B	Hepatitis B	2	MCH	pg	Day 28	28/10/2010	23	Yes	27.0 - 31.0	L	NCS
S203	211	Hepatitis B	Hepatitis B	2	MCHC	gm %	Day 28	28/10/2010	31.2	Yes	32.0 - 36.0	L	NCS
S203	211	Hepatitis B	Hepatitis B	2	MCV	fL	Day 28	28/10/2010	73.7	Yes	80.0- 96.0	L	NCS
S203	211	Hepatitis B	Hepatitis B	2	Platelet Count	10E3/ul	Day 28	28/10/2010	239	.	150 - 400		
S203	211	Hepatitis B	Hepatitis B	2	RBC count	10E6/ul	Day 28	28/10/2010	5.66	.	4.60 - 6.20		
S203	211	Hepatitis B	Hepatitis B	2	TLC	10E3/ul	Day 28	28/10/2010	6.46	.	4.50 - 11.00		
S203	211	Hepatitis B	Hepatitis B	2	% Basophils	%	Day 56	25/11/2010	0.7	.	0.0 - 1.0		
S203	211	Hepatitis B	Hepatitis B	2	% Eosinophils	%	Day 56	25/11/2010	1.7	.	1.0 - 6.0		
S203	211	Hepatitis B	Hepatitis B	2	% Lymphocytes	%	Day 56	25/11/2010	28.7	.	20.0 - 45.0		

Subject No.	Randomisation Number	Study Group	Dosage	Dose	Haematology Parameters	Unit	Day	Date of sample Collection	Results	Out of Range	Reference Range	Flag	Clinical Significance **
S203	211	Hepatitis B	Hepatitis B	2	% Monocytes	%	Day 56	25/11/2010	3.7	.	2.0 - 10.0		
S203	211	Hepatitis B	Hepatitis B	2	% Neutrophils	%	Day 56	25/11/2010	65.2	.	40.0 - 75.0		
S203	211	Hepatitis B	Hepatitis B	2	Haematocrit	%	Day 56	25/11/2010	40.4	.	40.0 - 54.0		
S203	211	Hepatitis B	Hepatitis B	2	Haemoglobin*	gm / dl	Day 56	25/11/2010	12.5	Yes	13.5 - 18.0	L	CS
S203	211	Hepatitis B	Hepatitis B	2	MCH	pg	Day 56	25/11/2010	23.1	Yes	27.0 - 31.0	L	CS
S203	211	Hepatitis B	Hepatitis B	2	MCHC	gm %	Day 56	25/11/2010	31	Yes	32.0 - 36.0	L	CS
S203	211	Hepatitis B	Hepatitis B	2	MCV	fL	Day 56	25/11/2010	74.6	Yes	80.0- 96.0	L	CS
S203	211	Hepatitis B	Hepatitis B	2	Platelet Count	10E3/ul	Day 56	25/11/2010	274	.	150 - 400		
S203	211	Hepatitis B	Hepatitis B	2	RBC count	10E6/ul	Day 56	25/11/2010	5.42	.	4.60 - 6.20		
S203	211	Hepatitis B	Hepatitis B	2	TLC	10E3/ul	Day 56	25/11/2010	8.66	.	4.50 - 11.00		
S203	211	Hepatitis B	Hepatitis B	3	% Basophils	%	Day 180	29/03/2011	1.7	Yes	0.0 - 1.0	H	NCS
S203	211	Hepatitis B	Hepatitis B	3	% Eosinophils	%	Day 180	29/03/2011	2.4	.	1.0 - 6.0		
S203	211	Hepatitis B	Hepatitis B	3	% Lymphocytes	%	Day 180	29/03/2011	26.6	.	20.0 - 45.0		
S203	211	Hepatitis B	Hepatitis B	3	% Monocytes	%	Day 180	29/03/2011	5.2	.	2.0 - 10.0		
S203	211	Hepatitis B	Hepatitis B	3	% Neutrophils	%	Day 180	29/03/2011	64	.	40.0 - 75.0		
S203	211	Hepatitis B	Hepatitis B	3	Haematocrit	%	Day 180	29/03/2011	43.3	.	40.0 - 54.0		
S203	211	Hepatitis B	Hepatitis B	3	Haemoglobin	gm / dl	Day 180	29/03/2011	14	.	13.5 - 18.0		
S203	211	Hepatitis B	Hepatitis B	3	MCH	pg	Day 180	29/03/2011	23.6	Yes	27.0 - 31.0	L	NCS
S203	211	Hepatitis B	Hepatitis B	3	MCHC	gm %	Day 180	29/03/2011	32.3	.	32.0 - 36.0		
S203	211	Hepatitis B	Hepatitis B	3	MCV	fL	Day 180	29/03/2011	73.2	Yes	80.0- 96.0	L	NCS
S203	211	Hepatitis B	Hepatitis B	3	Platelet Count	10E3/ul	Day 180	29/03/2011	231	.	150 - 400		
S203	211	Hepatitis B	Hepatitis B	3	RBC count	10E6/ul	Day 180	29/03/2011	5.92	.	4.60 - 6.20		
S203	211	Hepatitis B	Hepatitis B	3	TLC	10E3/ul	Day 180	29/03/2011	7.64	.	4.50 - 11.00		
S203	211	Hepatitis B	Hepatitis B	3	% Basophils	%	Day 208	28/04/2011	1.6	Yes	0.0 - 1.0	H	CS
S203	211	Hepatitis B	Hepatitis B	3	% Eosinophils	%	Day 208	28/04/2011	2.3	.	1.0 - 6.0		
S203	211	Hepatitis B	Hepatitis B	3	% Lymphocytes	%	Day 208	28/04/2011	20.4	.	20.0 - 45.0		
S203	211	Hepatitis B	Hepatitis B	3	% Monocytes	%	Day 208	28/04/2011	4.8	.	2.0 - 10.0		
S203	211	Hepatitis B	Hepatitis B	3	% Neutrophils	%	Day 208	28/04/2011	70.9	.	40.0 - 75.0		
S203	211	Hepatitis B	Hepatitis B	3	Haematocrit	%	Day 208	28/04/2011	46.3	.	40.0 - 54.0		

Subject No.	Randomisation Number	Study Group	Dosage	Dose	Haematology Parameters	Unit	Day	Date of sample Collection	Results	Out of Range	Reference Range	Flag	Clinical Significance **
S203	211	Hepatitis B	Hepatitis B	3	Haemoglobin	gm / dl	Day 208	28/04/2011	14.9	.	13.5 - 18.0		
S203	211	Hepatitis B	Hepatitis B	3	MCH	pg	Day 208	28/04/2011	24.4	Yes	27.0 - 31.0	L	NCS
S203	211	Hepatitis B	Hepatitis B	3	MCHC	gm %	Day 208	28/04/2011	32.3	.	32.0 - 36.0		
S203	211	Hepatitis B	Hepatitis B	3	MCV	fL	Day 208	28/04/2011	75.5	Yes	80.0- 96.0	L	NCS
S203	211	Hepatitis B	Hepatitis B	3	Platelet Count	10E3/ul	Day 208	28/04/2011	259	.	150 - 400		
S203	211	Hepatitis B	Hepatitis B	3	RBC count	10E6/ul	Day 208	28/04/2011	6.12	.	4.60 - 6.20		
S203	211	Hepatitis B	Hepatitis B	3	TLC	10E3/ul	Day 208	28/04/2011	12.56	Yes	4.50 - 11.00	H	CS
S203	211	Hepatitis B	Hepatitis B	3	% Basophils	%	Day 365	29/09/2011	0.6	.	0.0 - 1.0		
S203	211	Hepatitis B	Hepatitis B	3	% Eosinophils	%	Day 365	29/09/2011	1.6	.	1.0 - 6.0		
S203	211	Hepatitis B	Hepatitis B	3	% Lymphocytes	%	Day 365	29/09/2011	30.7	.	20.0 - 45.0		
S203	211	Hepatitis B	Hepatitis B	3	% Monocytes	%	Day 365	29/09/2011	5.3	.	2.0 - 10.0		
S203	211	Hepatitis B	Hepatitis B	3	% Neutrophils	%	Day 365	29/09/2011	61.8	.	40.0 - 75.0		
S203	211	Hepatitis B	Hepatitis B	3	Haematocrit	%	Day 365	29/09/2011	40.6	.	40.0 - 54.0		
S203	211	Hepatitis B	Hepatitis B	3	Haemoglobin*	gm / dl	Day 365	29/09/2011	13.3	Yes	13.5 - 18.0	L	NCS
S203	211	Hepatitis B	Hepatitis B	3	MCH	pg	Day 365	29/09/2011	24.6	Yes	27.0 - 31.0	L	NCS
S203	211	Hepatitis B	Hepatitis B	3	MCHC	gm %	Day 365	29/09/2011	32.9	.	32.0 - 36.0		
S203	211	Hepatitis B	Hepatitis B	3	MCV	fL	Day 365	29/09/2011	74.8	Yes	80.0- 96.0	L	NCS
S203	211	Hepatitis B	Hepatitis B	3	Platelet Count	10E3/ul	Day 365	29/09/2011	231	.	150 - 400		
S203	211	Hepatitis B	Hepatitis B	3	RBC count	10E6/ul	Day 365	29/09/2011	5.43	.	4.60 - 6.20		
S203	211	Hepatitis B	Hepatitis B	3	TLC	10E3/ul	Day 365	29/09/2011	6.77	.	4.50 - 11.00		
S212	212	JAIVAC-1	Dosage B		% Basophils	%	Day -14 to Day -1	23/09/2010	1.2	Yes	0.0 - 1.0	H	NCS
S212	212	JAIVAC-1	Dosage B		% Eosinophils	%	Day -14 to Day -1	23/09/2010	3.7	.	1.0 - 6.0		
S212	212	JAIVAC-1	Dosage B		% Lymphocytes	%	Day -14 to Day -1	23/09/2010	26.5	.	20.0 - 45.0		
S212	212	JAIVAC-1	Dosage B		% Monocytes	%	Day -14 to Day -1	23/09/2010	4.1	.	2.0 - 10.0		
S212	212	JAIVAC-1	Dosage B		% Neutrophils	%	Day -14 to Day -1	23/09/2010	64.6	.	40.0 - 75.0		
S212	212	JAIVAC-1	Dosage B		Haematocrit	%	Day -14 to Day -1	23/09/2010	45.9	.	40.0 - 54.0		
S212	212	JAIVAC-1	Dosage B		Haemoglobin	gm / dl	Day -14 to Day -1	23/09/2010	15.2	.	13.5 - 18.0		
S212	212	JAIVAC-1	Dosage B		MCH	pg	Day -14 to Day -1	23/09/2010	27.7	.	27.0 - 31.0		
S212	212	JAIVAC-1	Dosage B		MCHC	gm %	Day -14 to Day -1	23/09/2010	33.2	.	32.0 - 36.0		

Subject No.	Randomisation Number	Study Group	Dosage	Dose	Haematology Parameters	Unit	Day	Date of sample Collection	Results	Out of Range	Reference Range	Flag	Clinical Significance **
S212	212	JAIVAC-1	Dosage B		MCV	fL	Day -14 to Day -1	23/09/2010	83.5	.	80.0- 96.0		
S212	212	JAIVAC-1	Dosage B		Platelet Count	10E3/ul	Day -14 to Day -1	23/09/2010	287	.	150 - 400		
S212	212	JAIVAC-1	Dosage B		RBC count	10E6/ul	Day -14 to Day -1	23/09/2010	5.5	.	4.60 - 6.20		
S212	212	JAIVAC-1	Dosage B		TLC	10E3/ul	Day -14 to Day -1	23/09/2010	7.59	.	4.50 - 11.00		
S212	212	JAIVAC-1	Dosage B	2	% Basophils	%	Day 28	28/10/2010	1.2	Yes	0.0 - 1.0	H	NCS
S212	212	JAIVAC-1	Dosage B	2	% Eosinophils	%	Day 28	28/10/2010	1.9	.	1.0 - 6.0		
S212	212	JAIVAC-1	Dosage B	2	% Lymphocytes	%	Day 28	28/10/2010	17.7	Yes	20.0 - 45.0	L	NCS
S212	212	JAIVAC-1	Dosage B	2	% Monocytes	%	Day 28	28/10/2010	4.2	.	2.0 - 10.0		
S212	212	JAIVAC-1	Dosage B	2	% Neutrophils	%	Day 28	28/10/2010	75	.	40.0 - 75.0		
S212	212	JAIVAC-1	Dosage B	2	Haematocrit	%	Day 28	28/10/2010	45.7	.	40.0 - 54.0		
S212	212	JAIVAC-1	Dosage B	2	Haemoglobin	gm / dl	Day 28	28/10/2010	14.9	.	13.5 - 18.0		
S212	212	JAIVAC-1	Dosage B	2	MCH	pg	Day 28	28/10/2010	27.2	.	27.0 - 31.0		
S212	212	JAIVAC-1	Dosage B	2	MCHC	gm %	Day 28	28/10/2010	32.6	.	32.0 - 36.0		
S212	212	JAIVAC-1	Dosage B	2	MCV	fL	Day 28	28/10/2010	83.5	.	80.0- 96.0		
S212	212	JAIVAC-1	Dosage B	2	Platelet Count	10E3/ul	Day 28	28/10/2010	409	Yes	150 - 400	H	NCS
S212	212	JAIVAC-1	Dosage B	2	RBC count	10E6/ul	Day 28	28/10/2010	5.48	.	4.60 - 6.20		
S212	212	JAIVAC-1	Dosage B	2	TLC	10E3/ul	Day 28	28/10/2010	8.27	.	4.50 - 11.00		
S212	212	JAIVAC-1	Dosage B	2	% Basophils	%	Day 56	25/11/2010	0.6	.	0.0 - 1.0		
S212	212	JAIVAC-1	Dosage B	2	% Eosinophils	%	Day 56	25/11/2010	2.9	.	1.0 - 6.0		
S212	212	JAIVAC-1	Dosage B	2	% Lymphocytes	%	Day 56	25/11/2010	23.3	.	20.0 - 45.0		
S212	212	JAIVAC-1	Dosage B	2	% Monocytes	%	Day 56	25/11/2010	3.7	.	2.0 - 10.0		
S212	212	JAIVAC-1	Dosage B	2	% Neutrophils	%	Day 56	25/11/2010	69.5	.	40.0 - 75.0		
S212	212	JAIVAC-1	Dosage B	2	Haematocrit	%	Day 56	25/11/2010	48.7	.	40.0 - 54.0		
S212	212	JAIVAC-1	Dosage B	2	Haemoglobin	gm / dl	Day 56	25/11/2010	15.7	.	13.5 - 18.0		
S212	212	JAIVAC-1	Dosage B	2	MCH	pg	Day 56	25/11/2010	27.5	.	27.0 - 31.0		
S212	212	JAIVAC-1	Dosage B	2	MCHC	gm %	Day 56	25/11/2010	32.3	.	32.0 - 36.0		
S212	212	JAIVAC-1	Dosage B	2	MCV	fL	Day 56	25/11/2010	85.3	.	80.0- 96.0		
S212	212	JAIVAC-1	Dosage B	2	Platelet Count	10E3/ul	Day 56	25/11/2010	336	.	150 - 400		
S212	212	JAIVAC-1	Dosage B	2	RBC count	10E6/ul	Day 56	25/11/2010	5.71	.	4.60 - 6.20		

Subject No.	Randomisation Number	Study Group	Dosage	Dose	Haematology Parameters	Unit	Day	Date of sample Collection	Results	Out of Range	Reference Range	Flag	Clinical Significance **
S212	212	JAIVAC-1	Dosage B	2	TLC	10E3/ul	Day 56	25/11/2010	7.23	.	4.50 - 11.00		
S212	212	JAIVAC-1	Dosage B	3	% Basophils	%	Day 180	29/03/2011	1	.	0.0 - 1.0		
S212	212	JAIVAC-1	Dosage B	3	% Eosinophils	%	Day 180	29/03/2011	3.2	.	1.0 - 6.0		
S212	212	JAIVAC-1	Dosage B	3	% Lymphocytes	%	Day 180	29/03/2011	15.7	Yes	20.0 - 45.0	L	NCS
S212	212	JAIVAC-1	Dosage B	3	% Monocytes	%	Day 180	29/03/2011	4.9	.	2.0 - 10.0		
S212	212	JAIVAC-1	Dosage B	3	% Neutrophils	%	Day 180	29/03/2011	75.1	Yes	40.0 - 75.0	H	NCS
S212	212	JAIVAC-1	Dosage B	3	Haematocrit	%	Day 180	29/03/2011	40.6	.	40.0 - 54.0		
S212	212	JAIVAC-1	Dosage B	3	Haemoglobin*	gm / dl	Day 180	29/03/2011	13.3	Yes	13.5 - 18.0	L	NCS
S212	212	JAIVAC-1	Dosage B	3	MCH	pg	Day 180	29/03/2011	26.3	Yes	27.0 - 31.0	L	NCS
S212	212	JAIVAC-1	Dosage B	3	MCHC	gm %	Day 180	29/03/2011	32.6	.	32.0 - 36.0		
S212	212	JAIVAC-1	Dosage B	3	MCV	fL	Day 180	29/03/2011	80.6	.	80.0- 96.0		
S212	212	JAIVAC-1	Dosage B	3	Platelet Count	10E3/ul	Day 180	29/03/2011	329	.	150 - 400		
S212	212	JAIVAC-1	Dosage B	3	RBC count	10E6/ul	Day 180	29/03/2011	5.04	.	4.60 - 6.20		
S212	212	JAIVAC-1	Dosage B	3	TLC	10E3/ul	Day 180	29/03/2011	8.44	.	4.50 - 11.00		
S212	212	JAIVAC-1	Dosage B	3	% Basophils	%	Day 208	19/04/2011	1.7	Yes	0.0 - 1.0	H	NCS
S212	212	JAIVAC-1	Dosage B	3	% Eosinophils	%	Day 208	19/04/2011	4.9	.	1.0 - 6.0		
S212	212	JAIVAC-1	Dosage B	3	% Lymphocytes	%	Day 208	19/04/2011	29.5	.	20.0 - 45.0		
S212	212	JAIVAC-1	Dosage B	3	% Monocytes	%	Day 208	19/04/2011	6	.	2.0 - 10.0		
S212	212	JAIVAC-1	Dosage B	3	% Neutrophils	%	Day 208	19/04/2011	57.9	.	40.0 - 75.0		
S212	212	JAIVAC-1	Dosage B	3	Haematocrit	%	Day 208	19/04/2011	40	.	40.0 - 54.0		
S212	212	JAIVAC-1	Dosage B	3	Haemoglobin*	gm / dl	Day 208	19/04/2011	13.3	Yes	13.5 - 18.0	L	NCS
S212	212	JAIVAC-1	Dosage B	3	MCH	pg	Day 208	19/04/2011	26.9	Yes	27.0 - 31.0	L	NCS
S212	212	JAIVAC-1	Dosage B	3	MCHC	gm %	Day 208	19/04/2011	33.3	.	32.0 - 36.0		
S212	212	JAIVAC-1	Dosage B	3	MCV	fL	Day 208	19/04/2011	80.9	.	80.0- 96.0		
S212	212	JAIVAC-1	Dosage B	3	Platelet Count	10E3/ul	Day 208	19/04/2011	332	.	150 - 400		
S212	212	JAIVAC-1	Dosage B	3	RBC count	10E6/ul	Day 208	19/04/2011	4.94	.	4.60 - 6.20		
S212	212	JAIVAC-1	Dosage B	3	TLC	10E3/ul	Day 208	19/04/2011	7.34	.	4.50 - 11.00		
S212	212	JAIVAC-1	Dosage B	3	% Basophils	%	Day 365	29/09/2011	2.4	Yes	0.0 - 1.0	H	NCS
S212	212	JAIVAC-1	Dosage B	3	% Eosinophils	%	Day 365	29/09/2011	3.8	.	1.0 - 6.0		

Subject No.	Randomisation Number	Study Group	Dosage	Dose	Haematology Parameters	Unit	Day	Date of sample Collection	Results	Out of Range	Reference Range	Flag	Clinical Significance **
S212	212	JAIVAC-1	Dosage B	3	% Lymphocytes	%	Day 365	29/09/2011	19.7	Yes	20.0 - 45.0	L	NCS
S212	212	JAIVAC-1	Dosage B	3	% Monocytes	%	Day 365	29/09/2011	6.7	.	2.0 - 10.0		
S212	212	JAIVAC-1	Dosage B	3	% Neutrophils	%	Day 365	29/09/2011	67.5	.	40.0 - 75.0		
S212	212	JAIVAC-1	Dosage B	3	Haematocrit	%	Day 365	29/09/2011	43.1	.	40.0 - 54.0		
S212	212	JAIVAC-1	Dosage B	3	Haemoglobin	gm / dl	Day 365	29/09/2011	14.4	.	13.5 - 18.0		
S212	212	JAIVAC-1	Dosage B	3	MCH	pg	Day 365	29/09/2011	27	.	27.0 - 31.0		
S212	212	JAIVAC-1	Dosage B	3	MCHC	gm %	Day 365	29/09/2011	33.3	.	32.0 - 36.0		
S212	212	JAIVAC-1	Dosage B	3	MCV	fL	Day 365	29/09/2011	81.1	.	80.0- 96.0		
S212	212	JAIVAC-1	Dosage B	3	Platelet Count	10E3/ul	Day 365	29/09/2011	316	.	150 - 400		
S212	212	JAIVAC-1	Dosage B	3	RBC count	10E6/ul	Day 365	29/09/2011	5.32	.	4.60 - 6.20		
S212	212	JAIVAC-1	Dosage B	3	TLC	10E3/ul	Day 365	29/09/2011	6.35	.	4.50 - 11.00		
S215	213	Hepatitis B	Hepatitis B		% Basophils	%	Day -14 to Day -1	24/09/2010	0	.	0.0 - 1.0		
S215	213	Hepatitis B	Hepatitis B		% Eosinophils	%	Day -14 to Day -1	24/09/2010	3	.	1.0 - 6.0		
S215	213	Hepatitis B	Hepatitis B		% Lymphocytes	%	Day -14 to Day -1	24/09/2010	35	.	20.0 - 45.0		
S215	213	Hepatitis B	Hepatitis B		% Monocytes	%	Day -14 to Day -1	24/09/2010	6	.	2.0 - 10.0		
S215	213	Hepatitis B	Hepatitis B		% Neutrophils	%	Day -14 to Day -1	24/09/2010	56	.	40.0 - 75.0		
S215	213	Hepatitis B	Hepatitis B		Haematocrit	%	Day -14 to Day -1	24/09/2010	47.8	.	40.0 - 54.0		
S215	213	Hepatitis B	Hepatitis B		Haemoglobin	gm / dl	Day -14 to Day -1	24/09/2010	15.6	.	13.5 - 18.0		
S215	213	Hepatitis B	Hepatitis B		MCH	pg	Day -14 to Day -1	24/09/2010	27.3	.	27.0 - 31.0		
S215	213	Hepatitis B	Hepatitis B		MCHC	gm %	Day -14 to Day -1	24/09/2010	32.5	.	32.0 - 36.0		
S215	213	Hepatitis B	Hepatitis B		MCV	fL	Day -14 to Day -1	24/09/2010	84	.	80.0- 96.0		
S215	213	Hepatitis B	Hepatitis B		Platelet Count	10E3/ul	Day -14 to Day -1	24/09/2010	280	.	150 - 400		
S215	213	Hepatitis B	Hepatitis B		RBC count	10E6/ul	Day -14 to Day -1	24/09/2010	5.7	.	4.60 - 6.20		
S215	213	Hepatitis B	Hepatitis B		TLC	10E3/ul	Day -14 to Day -1	24/09/2010	6.27	.	4.50 - 11.00		
S215	213	Hepatitis B	Hepatitis B	2	% Basophils	%	Day 28	28/10/2010	0	.	0.0 - 1.0		
S215	213	Hepatitis B	Hepatitis B	2	% Eosinophils	%	Day 28	28/10/2010	3	.	1.0 - 6.0		
S215	213	Hepatitis B	Hepatitis B	2	% Lymphocytes	%	Day 28	28/10/2010	34	.	20.0 - 45.0		
S215	213	Hepatitis B	Hepatitis B	2	% Monocytes	%	Day 28	28/10/2010	8	.	2.0 - 10.0		
S215	213	Hepatitis B	Hepatitis B	2	% Neutrophils	%	Day 28	28/10/2010	55	.	40.0 - 75.0		

Subject No.	Randomisation Number	Study Group	Dosage	Dose	Haematology Parameters	Unit	Day	Date of sample Collection	Results	Out of Range	Reference Range	Flag	Clinical Significance **
S215	213	Hepatitis B	Hepatitis B	2	Haematocrit	%	Day 28	28/10/2010	44.5	.	40.0 - 54.0		
S215	213	Hepatitis B	Hepatitis B	2	Haemoglobin	gm / dl	Day 28	28/10/2010	15.1	.	13.5 - 18.0		
S215	213	Hepatitis B	Hepatitis B	2	MCH	pg	Day 28	28/10/2010	28.6	.	27.0 - 31.0		
S215	213	Hepatitis B	Hepatitis B	2	MCHC	gm %	Day 28	28/10/2010	33.9	.	32.0 - 36.0		
S215	213	Hepatitis B	Hepatitis B	2	MCV	fL	Day 28	28/10/2010	84.3	.	80.0- 96.0		
S215	213	Hepatitis B	Hepatitis B	2	Platelet Count	10E3/ul	Day 28	28/10/2010	297	.	150 - 400		
S215	213	Hepatitis B	Hepatitis B	2	RBC count	10E6/ul	Day 28	28/10/2010	5.27	.	4.60 - 6.20		
S215	213	Hepatitis B	Hepatitis B	2	TLC	10E3/ul	Day 28	28/10/2010	5.45	.	4.50 - 11.00		
S215	213	Hepatitis B	Hepatitis B	2	% Basophils	%	Day 56	25/11/2010	1	.	0.0 - 1.0		
S215	213	Hepatitis B	Hepatitis B	2	% Eosinophils	%	Day 56	25/11/2010	3.2	.	1.0 - 6.0		
S215	213	Hepatitis B	Hepatitis B	2	% Lymphocytes	%	Day 56	25/11/2010	37.8	.	20.0 - 45.0		
S215	213	Hepatitis B	Hepatitis B	2	% Monocytes	%	Day 56	25/11/2010	6.2	.	2.0 - 10.0		
S215	213	Hepatitis B	Hepatitis B	2	% Neutrophils	%	Day 56	25/11/2010	51.8	.	40.0 - 75.0		
S215	213	Hepatitis B	Hepatitis B	2	Haematocrit	%	Day 56	25/11/2010	50.9	.	40.0 - 54.0		
S215	213	Hepatitis B	Hepatitis B	2	Haemoglobin	gm / dl	Day 56	25/11/2010	16.3	.	13.5 - 18.0		
S215	213	Hepatitis B	Hepatitis B	2	MCH	pg	Day 56	25/11/2010	27.8	.	27.0 - 31.0		
S215	213	Hepatitis B	Hepatitis B	2	MCHC	gm %	Day 56	25/11/2010	32	.	32.0 - 36.0		
S215	213	Hepatitis B	Hepatitis B	2	MCV	fL	Day 56	25/11/2010	86.9	.	80.0- 96.0		
S215	213	Hepatitis B	Hepatitis B	2	Platelet Count	10E3/ul	Day 56	25/11/2010	288	.	150 - 400		
S215	213	Hepatitis B	Hepatitis B	2	RBC count	10E6/ul	Day 56	25/11/2010	5.86	.	4.60 - 6.20		
S215	213	Hepatitis B	Hepatitis B	2	TLC	10E3/ul	Day 56	25/11/2010	6.72	.	4.50 - 11.00		
S215	213	Hepatitis B	Hepatitis B	3	% Basophils	%	Day 180	29/03/2011	0	.	0.0 - 1.0		
S215	213	Hepatitis B	Hepatitis B	3	% Eosinophils	%	Day 180	29/03/2011	3	.	1.0 - 6.0		
S215	213	Hepatitis B	Hepatitis B	3	% Lymphocytes	%	Day 180	29/03/2011	27	.	20.0 - 45.0		
S215	213	Hepatitis B	Hepatitis B	3	% Monocytes	%	Day 180	29/03/2011	14	Yes	2.0 - 10.0	H	NCS
S215	213	Hepatitis B	Hepatitis B	3	% Neutrophils	%	Day 180	29/03/2011	56	.	40.0 - 75.0		
S215	213	Hepatitis B	Hepatitis B	3	Haematocrit	%	Day 180	29/03/2011	50.3	.	40.0 - 54.0		
S215	213	Hepatitis B	Hepatitis B	3	Haemoglobin	gm / dl	Day 180	29/03/2011	17.2	.	13.5 - 18.0		
S215	213	Hepatitis B	Hepatitis B	3	MCH	pg	Day 180	29/03/2011	30.5	.	27.0 - 31.0		

Subject No.	Randomisation Number	Study Group	Dosage	Dose	Haematology Parameters	Unit	Day	Date of sample Collection	Results	Out of Range	Reference Range	Flag	Clinical Significance **
S215	213	Hepatitis B	Hepatitis B	3	MCHC	gm %	Day 180	29/03/2011	34.2	.	32.0 - 36.0		
S215	213	Hepatitis B	Hepatitis B	3	MCV	fL	Day 180	29/03/2011	89.1	.	80.0- 96.0		
S215	213	Hepatitis B	Hepatitis B	3	Platelet Count	10E3/ul	Day 180	29/03/2011	277	.	150 - 400		
S215	213	Hepatitis B	Hepatitis B	3	RBC count	10E6/ul	Day 180	29/03/2011	5.64	.	4.60 - 6.20		
S215	213	Hepatitis B	Hepatitis B	3	TLC	10E3/ul	Day 180	29/03/2011	6.81	.	4.50 - 11.00		
S215	213	Hepatitis B	Hepatitis B	3	% Basophils	%	Day 208	26/04/2011	0	.	0.0 - 1.0		
S215	213	Hepatitis B	Hepatitis B	3	% Eosinophils	%	Day 208	26/04/2011	6	.	1.0 - 6.0		
S215	213	Hepatitis B	Hepatitis B	3	% Lymphocytes	%	Day 208	26/04/2011	21	.	20.0 - 45.0		
S215	213	Hepatitis B	Hepatitis B	3	% Monocytes	%	Day 208	26/04/2011	10	.	2.0 - 10.0		
S215	213	Hepatitis B	Hepatitis B	3	% Neutrophils	%	Day 208	26/04/2011	63	.	40.0 - 75.0		
S215	213	Hepatitis B	Hepatitis B	3	Haematocrit	%	Day 208	26/04/2011	50.9	.	40.0 - 54.0		
S215	213	Hepatitis B	Hepatitis B	3	Haemoglobin	gm / dl	Day 208	26/04/2011	17.1	.	13.5 - 18.0		
S215	213	Hepatitis B	Hepatitis B	3	MCH	pg	Day 208	26/04/2011	31.2	Yes	27.0 - 31.0	H	NCS
S215	213	Hepatitis B	Hepatitis B	3	MCHC	gm %	Day 208	26/04/2011	33.6	.	32.0 - 36.0		
S215	213	Hepatitis B	Hepatitis B	3	MCV	fL	Day 208	26/04/2011	93	.	80.0- 96.0		
S215	213	Hepatitis B	Hepatitis B	3	Platelet Count	10E3/ul	Day 208	26/04/2011	325	.	150 - 400		
S215	213	Hepatitis B	Hepatitis B	3	RBC count	10E6/ul	Day 208	26/04/2011	5.47	.	4.60 - 6.20		
S215	213	Hepatitis B	Hepatitis B	3	TLC	10E3/ul	Day 208	26/04/2011	6.26	.	4.50 - 11.00		
S215	213	Hepatitis B	Hepatitis B	3	% Basophils	%	Day 365	29/09/2011	0	.	0.0 - 1.0		
S215	213	Hepatitis B	Hepatitis B	3	% Eosinophils	%	Day 365	29/09/2011	5	.	1.0 - 6.0		
S215	213	Hepatitis B	Hepatitis B	3	% Lymphocytes	%	Day 365	29/09/2011	34.9	.	20.0 - 45.0		
S215	213	Hepatitis B	Hepatitis B	3	% Monocytes	%	Day 365	29/09/2011	10.2	Yes	2.0 - 10.0	H	NCS
S215	213	Hepatitis B	Hepatitis B	3	% Neutrophils	%	Day 365	29/09/2011	49.9	.	40.0 - 75.0		
S215	213	Hepatitis B	Hepatitis B	3	Haematocrit	%	Day 365	29/09/2011	46.8	.	40.0 - 54.0		
S215	213	Hepatitis B	Hepatitis B	3	Haemoglobin	gm / dl	Day 365	29/09/2011	16.5	.	13.5 - 18.0		
S215	213	Hepatitis B	Hepatitis B	3	MCH	pg	Day 365	29/09/2011	31.8	Yes	27.0 - 31.0	H	NCS
S215	213	Hepatitis B	Hepatitis B	3	MCHC	gm %	Day 365	29/09/2011	35.3	.	32.0 - 36.0		
S215	213	Hepatitis B	Hepatitis B	3	MCV	fL	Day 365	29/09/2011	89.9	.	80.0- 96.0		
S215	213	Hepatitis B	Hepatitis B	3	Platelet Count	10E3/ul	Day 365	29/09/2011	277	.	150 - 400		

Subject No.	Randomisation Number	Study Group	Dosage	Dose	Haematology Parameters	Unit	Day	Date of sample Collection	Results	Out of Range	Reference Range	Flag	Clinical Significance **
S215	213	Hepatitis B	Hepatitis B	3	RBC count	10E6/ul	Day 365	29/09/2011	5.2	.	4.60 - 6.20		
S215	213	Hepatitis B	Hepatitis B	3	TLC	10E3/ul	Day 365	29/09/2011	5.87	.	4.50 - 11.00		
S233	210	JAIVAC-1	Dosage B		% Basophils	%	Day -14 to Day -1	24/09/2010	1.2	Yes	0.0 - 1.0	H	NCS
S233	210	JAIVAC-1	Dosage B		% Eosinophils	%	Day -14 to Day -1	24/09/2010	2.7	.	1.0 - 6.0		
S233	210	JAIVAC-1	Dosage B		% Lymphocytes	%	Day -14 to Day -1	24/09/2010	23.5	.	20.0 - 45.0		
S233	210	JAIVAC-1	Dosage B		% Monocytes	%	Day -14 to Day -1	24/09/2010	4.4	.	2.0 - 10.0		
S233	210	JAIVAC-1	Dosage B		% Neutrophils	%	Day -14 to Day -1	24/09/2010	68.1	.	40.0 - 75.0		
S233	210	JAIVAC-1	Dosage B		Haematocrit	%	Day -14 to Day -1	24/09/2010	48.1	.	40.0 - 54.0		
S233	210	JAIVAC-1	Dosage B		Haemoglobin	gm / dl	Day -14 to Day -1	24/09/2010	15.1	.	13.5 - 18.0		
S233	210	JAIVAC-1	Dosage B		MCH	pg	Day -14 to Day -1	24/09/2010	23.6	Yes	27.0 - 31.0	L	NCS
S233	210	JAIVAC-1	Dosage B		MCHC	gm %	Day -14 to Day -1	24/09/2010	31.3	Yes	32.0 - 36.0	L	NCS
S233	210	JAIVAC-1	Dosage B		MCV	fL	Day -14 to Day -1	24/09/2010	75.5	Yes	80.0- 96.0	L	NCS
S233	210	JAIVAC-1	Dosage B		Platelet Count	10E3/ul	Day -14 to Day -1	24/09/2010	216	.	150 - 400		
S233	210	JAIVAC-1	Dosage B		RBC count	10E6/ul	Day -14 to Day -1	24/09/2010	6.37	.	4.60 - 6.20	H	
S233	210	JAIVAC-1	Dosage B		TLC	10E3/ul	Day -14 to Day -1	24/09/2010	7.26	.	4.50 - 11.00		
S233	210	JAIVAC-1	Dosage B	2	% Basophils	%	Day 28	28/10/2010	0.7	.	0.0 - 1.0		
S233	210	JAIVAC-1	Dosage B	2	% Eosinophils	%	Day 28	28/10/2010	3.1	.	1.0 - 6.0		
S233	210	JAIVAC-1	Dosage B	2	% Lymphocytes	%	Day 28	28/10/2010	24.2	.	20.0 - 45.0		
S233	210	JAIVAC-1	Dosage B	2	% Monocytes	%	Day 28	28/10/2010	4.5	.	2.0 - 10.0		
S233	210	JAIVAC-1	Dosage B	2	% Neutrophils	%	Day 28	28/10/2010	67.5	.	40.0 - 75.0		
S233	210	JAIVAC-1	Dosage B	2	Haematocrit	%	Day 28	28/10/2010	43.8	.	40.0 - 54.0		
S233	210	JAIVAC-1	Dosage B	2	Haemoglobin	gm / dl	Day 28	28/10/2010	14	.	13.5 - 18.0		
S233	210	JAIVAC-1	Dosage B	2	MCH	pg	Day 28	28/10/2010	23.3	Yes	27.0 - 31.0	L	NCS
S233	210	JAIVAC-1	Dosage B	2	MCHC	gm %	Day 28	28/10/2010	31.9	Yes	32.0 - 36.0	L	NCS
S233	210	JAIVAC-1	Dosage B	2	MCV	fL	Day 28	28/10/2010	73.1	Yes	80.0- 96.0	L	NCS
S233	210	JAIVAC-1	Dosage B	2	Platelet Count	10E3/ul	Day 28	28/10/2010	246	.	150 - 400		
S233	210	JAIVAC-1	Dosage B	2	RBC count	10E6/ul	Day 28	28/10/2010	5.99	.	4.60 - 6.20		
S233	210	JAIVAC-1	Dosage B	2	TLC	10E3/ul	Day 28	28/10/2010	6.48	.	4.50 - 11.00		
S233	210	JAIVAC-1	Dosage B	2	% Basophils	%	Day 56	25/11/2010	0.8	.	0.0 - 1.0		

Subject No.	Randomisation Number	Study Group	Dosage	Dose	Haematology Parameters	Unit	Day	Date of sample Collection	Results	Out of Range	Reference Range	Flag	Clinical Significance **
S233	210	JAIVAC-1	Dosage B	2	% Eosinophils	%	Day 56	25/11/2010	3.8	.	1.0 - 6.0		
S233	210	JAIVAC-1	Dosage B	2	% Lymphocytes	%	Day 56	25/11/2010	24.7	.	20.0 - 45.0		
S233	210	JAIVAC-1	Dosage B	2	% Monocytes	%	Day 56	25/11/2010	4.5	.	2.0 - 10.0		
S233	210	JAIVAC-1	Dosage B	2	% Neutrophils	%	Day 56	25/11/2010	66.2	.	40.0 - 75.0		
S233	210	JAIVAC-1	Dosage B	2	Haematocrit	%	Day 56	25/11/2010	48.9	.	40.0 - 54.0		
S233	210	JAIVAC-1	Dosage B	2	Haemoglobin	gm / dl	Day 56	25/11/2010	15.5	.	13.5 - 18.0		
S233	210	JAIVAC-1	Dosage B	2	MCH	pg	Day 56	25/11/2010	22.8	Yes	27.0 - 31.0	L	NCS
S233	210	JAIVAC-1	Dosage B	2	MCHC	gm %	Day 56	25/11/2010	31.7	Yes	32.0 - 36.0	L	NCS
S233	210	JAIVAC-1	Dosage B	2	MCV	fL	Day 56	25/11/2010	72	Yes	80.0- 96.0	L	NCS
S233	210	JAIVAC-1	Dosage B	2	Platelet Count	10E3/ul	Day 56	25/11/2010	254	.	150 - 400		
S233	210	JAIVAC-1	Dosage B	2	RBC count	10E6/ul	Day 56	25/11/2010	6.79	Yes	4.60 - 6.20	H	NCS
S233	210	JAIVAC-1	Dosage B	2	TLC	10E3/ul	Day 56	25/11/2010	6.92	.	4.50 - 11.00		
S233	210	JAIVAC-1	Dosage B	3	% Basophils	%	Day 180	29/03/2011	0.8	.	0.0 - 1.0		
S233	210	JAIVAC-1	Dosage B	3	% Eosinophils	%	Day 180	29/03/2011	2.8	.	1.0 - 6.0		
S233	210	JAIVAC-1	Dosage B	3	% Lymphocytes	%	Day 180	29/03/2011	36	.	20.0 - 45.0		
S233	210	JAIVAC-1	Dosage B	3	% Monocytes	%	Day 180	29/03/2011	7	.	2.0 - 10.0		
S233	210	JAIVAC-1	Dosage B	3	% Neutrophils	%	Day 180	29/03/2011	53.4	.	40.0 - 75.0		
S233	210	JAIVAC-1	Dosage B	3	Haematocrit	%	Day 180	29/03/2011	37.9	Yes	40.0 - 54.0	L	CS
S233	210	JAIVAC-1	Dosage B	3	Haemoglobin*	gm / dl	Day 180	29/03/2011	11.6	Yes	13.5 - 18.0	L	CS
S233	210	JAIVAC-1	Dosage B	3	MCH	pg	Day 180	29/03/2011	20.2	Yes	27.0 - 31.0	L	CS
S233	210	JAIVAC-1	Dosage B	3	MCHC	gm %	Day 180	29/03/2011	30.6	Yes	32.0 - 36.0	L	CS
S233	210	JAIVAC-1	Dosage B	3	MCV	fL	Day 180	29/03/2011	66	Yes	80.0- 96.0	L	CS
S233	210	JAIVAC-1	Dosage B	3	Platelet Count	10E3/ul	Day 180	29/03/2011	265	.	150 - 400		
S233	210	JAIVAC-1	Dosage B	3	RBC count	10E6/ul	Day 180	29/03/2011	5.74	.	4.60 - 6.20		
S233	210	JAIVAC-1	Dosage B	3	TLC	10E3/ul	Day 180	29/03/2011	7.24	.	4.50 - 11.00		
S233	210	JAIVAC-1	Dosage B	3	% Basophils	%	Day 208	27/04/2011	1.2	Yes	0.0 - 1.0	H	NCS
S233	210	JAIVAC-1	Dosage B	3	% Eosinophils	%	Day 208	27/04/2011	2.2	.	1.0 - 6.0		
S233	210	JAIVAC-1	Dosage B	3	% Lymphocytes	%	Day 208	27/04/2011	30.7	.	20.0 - 45.0		
S233	210	JAIVAC-1	Dosage B	3	% Monocytes	%	Day 208	27/04/2011	5.2	.	2.0 - 10.0		

Subject No.	Randomisation Number	Study Group	Dosage	Dose	Haematology Parameters	Unit	Day	Date of sample Collection	Results	Out of Range	Reference Range	Flag	Clinical Significance **
S233	210	JAIVAC-1	Dosage B	3	% Neutrophils	%	Day 208	27/04/2011	60.7	.	40.0 - 75.0		
S233	210	JAIVAC-1	Dosage B	3	Haematocrit	%	Day 208	27/04/2011	47.2	.	40.0 - 54.0		
S233	210	JAIVAC-1	Dosage B	3	Haemoglobin	gm / dl	Day 208	27/04/2011	14.2	.	13.5 - 18.0		
S233	210	JAIVAC-1	Dosage B	3	MCH	pg	Day 208	27/04/2011	22	Yes	27.0 - 31.0	L	NCS
S233	210	JAIVAC-1	Dosage B	3	MCHC	gm %	Day 208	27/04/2011	30	Yes	32.0 - 36.0	L	NCS
S233	210	JAIVAC-1	Dosage B	3	MCV	fL	Day 208	27/04/2011	73.2	Yes	80.0- 96.0	L	NCS
S233	210	JAIVAC-1	Dosage B	3	Platelet Count	10E3/ul	Day 208	27/04/2011	281	.	150 - 400		
S233	210	JAIVAC-1	Dosage B	3	RBC count	10E6/ul	Day 208	27/04/2011	6.45	Yes	4.60 - 6.20	H	NCS
S233	210	JAIVAC-1	Dosage B	3	TLC	10E3/ul	Day 208	27/04/2011	7.17	.	4.50 - 11.00		
S233	210	JAIVAC-1	Dosage B	3	% Basophils	%	Day 365	29/09/2011	0	.	0.0 - 1.0		
S233	210	JAIVAC-1	Dosage B	3	% Eosinophils	%	Day 365	29/09/2011	6	.	1.0 - 6.0		
S233	210	JAIVAC-1	Dosage B	3	% Lymphocytes	%	Day 365	29/09/2011	29	.	20.0 - 45.0		
S233	210	JAIVAC-1	Dosage B	3	% Monocytes	%	Day 365	29/09/2011	6	.	2.0 - 10.0		
S233	210	JAIVAC-1	Dosage B	3	% Neutrophils	%	Day 365	29/09/2011	59	.	40.0 - 75.0		
S233	210	JAIVAC-1	Dosage B	3	Haematocrit	%	Day 365	29/09/2011	44.4	.	40.0 - 54.0		
S233	210	JAIVAC-1	Dosage B	3	Haemoglobin	gm / dl	Day 365	29/09/2011	14.7	.	13.5 - 18.0		
S233	210	JAIVAC-1	Dosage B	3	MCH	pg	Day 365	29/09/2011	24.3	Yes	27.0 - 31.0	L	NCS
S233	210	JAIVAC-1	Dosage B	3	MCHC	gm %	Day 365	29/09/2011	33.2	.	32.0 - 36.0		
S233	210	JAIVAC-1	Dosage B	3	MCV	fL	Day 365	29/09/2011	73.2	Yes	80.0- 96.0	L	NCS
S233	210	JAIVAC-1	Dosage B	3	Platelet Count	10E3/ul	Day 365	29/09/2011	240	.	150 - 400		
S233	210	JAIVAC-1	Dosage B	3	RBC count	10E6/ul	Day 365	29/09/2011	6.06	.	4.60 - 6.20		
S233	210	JAIVAC-1	Dosage B	3	TLC	10E3/ul	Day 365	29/09/2011	6.64	.	4.50 - 11.00		
S241	303	Hepatitis B	Hepatitis B		% Basophils	%	Day -14 to Day -1	03/11/2010	0.7	.	0.0 - 1.0		
S241	303	Hepatitis B	Hepatitis B		% Eosinophils	%	Day -14 to Day -1	03/11/2010	4.1	.	1.0 - 6.0		
S241	303	Hepatitis B	Hepatitis B		% Lymphocytes	%	Day -14 to Day -1	03/11/2010	30.9	.	20.0 - 45.0		
S241	303	Hepatitis B	Hepatitis B		% Monocytes	%	Day -14 to Day -1	03/11/2010	5.2	.	2.0 - 10.0		
S241	303	Hepatitis B	Hepatitis B		% Neutrophils	%	Day -14 to Day -1	03/11/2010	59.1	.	40.0 - 75.0		
S241	303	Hepatitis B	Hepatitis B		Haematocrit	%	Day -14 to Day -1	03/11/2010	47.9	.	40.0 - 54.0		
S241	303	Hepatitis B	Hepatitis B		Haemoglobin	gm / dl	Day -14 to Day -1	03/11/2010	16.4	.	13.5 - 18.0		

Subject No.	Randomisation Number	Study Group	Dosage	Dose	Haematology Parameters	Unit	Day	Date of sample Collection	Results	Out of Range	Reference Range	Flag	Clinical Significance **
S241	303	Hepatitis B	Hepatitis B		MCH	pg	Day -14 to Day -1	03/11/2010	32.1	Yes	27.0 - 31.0	H	NCS
S241	303	Hepatitis B	Hepatitis B		MCHC	gm %	Day -14 to Day -1	03/11/2010	34.3	.	32.0 - 36.0		
S241	303	Hepatitis B	Hepatitis B		MCV	fL	Day -14 to Day -1	03/11/2010	93.6	.	80.0- 96.0		
S241	303	Hepatitis B	Hepatitis B		Platelet Count	10E3/ul	Day -14 to Day -1	03/11/2010	281	.	150 - 400		
S241	303	Hepatitis B	Hepatitis B		RBC count	10E6/ul	Day -14 to Day -1	03/11/2010	5.12	.	4.60 - 6.20		
S241	303	Hepatitis B	Hepatitis B		TLC	10E3/ul	Day -14 to Day -1	03/11/2010	8.25	.	4.50 - 11.00		
S241	303	Hepatitis B	Hepatitis B	2	% Basophils	%	Day 28	08/12/2010	2.5	Yes	0.0 - 1.0	H	NCS
S241	303	Hepatitis B	Hepatitis B	2	% Eosinophils	%	Day 28	08/12/2010	4.3	.	1.0 - 6.0		
S241	303	Hepatitis B	Hepatitis B	2	% Lymphocytes	%	Day 28	08/12/2010	27.6	.	20.0 - 45.0		
S241	303	Hepatitis B	Hepatitis B	2	% Monocytes	%	Day 28	08/12/2010	5.1	.	2.0 - 10.0		
S241	303	Hepatitis B	Hepatitis B	2	% Neutrophils	%	Day 28	08/12/2010	60.5	.	40.0 - 75.0		
S241	303	Hepatitis B	Hepatitis B	2	Haematocrit	%	Day 28	08/12/2010	50.1	.	40.0 - 54.0		
S241	303	Hepatitis B	Hepatitis B	2	Haemoglobin	gm / dl	Day 28	08/12/2010	17.3	.	13.5 - 18.0		
S241	303	Hepatitis B	Hepatitis B	2	MCH	pg	Day 28	08/12/2010	31.7	Yes	27.0 - 31.0	H	NCS
S241	303	Hepatitis B	Hepatitis B	2	MCHC	gm %	Day 28	08/12/2010	34.6	.	32.0 - 36.0		
S241	303	Hepatitis B	Hepatitis B	2	MCV	fL	Day 28	08/12/2010	91.6	.	80.0- 96.0		
S241	303	Hepatitis B	Hepatitis B	2	Platelet Count	10E3/ul	Day 28	08/12/2010	271	.	150 - 400		
S241	303	Hepatitis B	Hepatitis B	2	RBC count	10E6/ul	Day 28	08/12/2010	5.47	.	4.60 - 6.20		
S241	303	Hepatitis B	Hepatitis B	2	TLC	10E3/ul	Day 28	08/12/2010	7.76	.	4.50 - 11.00		
S241	303	Hepatitis B	Hepatitis B	2	% Basophils	%	Day 56	05/01/2011	1.8	Yes	0.0 - 1.0	H	NCS
S241	303	Hepatitis B	Hepatitis B	2	% Eosinophils	%	Day 56	05/01/2011	3.5	.	1.0 - 6.0		
S241	303	Hepatitis B	Hepatitis B	2	% Lymphocytes	%	Day 56	05/01/2011	33	.	20.0 - 45.0		
S241	303	Hepatitis B	Hepatitis B	2	% Monocytes	%	Day 56	05/01/2011	5	.	2.0 - 10.0		
S241	303	Hepatitis B	Hepatitis B	2	% Neutrophils	%	Day 56	05/01/2011	56.7	.	40.0 - 75.0		
S241	303	Hepatitis B	Hepatitis B	2	Haematocrit	%	Day 56	05/01/2011	49.4	.	40.0 - 54.0		
S241	303	Hepatitis B	Hepatitis B	2	Haemoglobin	gm / dl	Day 56	05/01/2011	16.8	.	13.5 - 18.0		
S241	303	Hepatitis B	Hepatitis B	2	MCH	pg	Day 56	05/01/2011	30.5	.	27.0 - 31.0		
S241	303	Hepatitis B	Hepatitis B	2	MCHC	gm %	Day 56	05/01/2011	34	.	32.0 - 36.0		
S241	303	Hepatitis B	Hepatitis B	2	MCV	fL	Day 56	05/01/2011	89.8	.	80.0- 96.0		

Subject No.	Randomisation Number	Study Group	Dosage	Dose	Haematology Parameters	Unit	Day	Date of sample Collection	Results	Out of Range	Reference Range	Flag	Clinical Significance **
S241	303	Hepatitis B	Hepatitis B	2	Platelet Count	10E3/ul	Day 56	05/01/2011	292	.	150 - 400		
S241	303	Hepatitis B	Hepatitis B	2	RBC count	10E6/ul	Day 56	05/01/2011	5.5	.	4.60 - 6.20		
S241	303	Hepatitis B	Hepatitis B	2	TLC	10E3/ul	Day 56	05/01/2011	7.69	.	4.50 - 11.00		
S241	303	Hepatitis B	Hepatitis B	3	% Basophils	%	Day 180	09/05/2011	1.1	Yes	0.0 - 1.0	H	NCS
S241	303	Hepatitis B	Hepatitis B	3	% Eosinophils	%	Day 180	09/05/2011	2	.	1.0 - 6.0		
S241	303	Hepatitis B	Hepatitis B	3	% Lymphocytes	%	Day 180	09/05/2011	28.3	.	20.0 - 45.0		
S241	303	Hepatitis B	Hepatitis B	3	% Monocytes	%	Day 180	09/05/2011	4.5	.	2.0 - 10.0		
S241	303	Hepatitis B	Hepatitis B	3	% Neutrophils	%	Day 180	09/05/2011	64.1	.	40.0 - 75.0		
S241	303	Hepatitis B	Hepatitis B	3	Haematocrit	%	Day 180	09/05/2011	52.1	.	40.0 - 54.0		
S241	303	Hepatitis B	Hepatitis B	3	Haemoglobin	gm / dl	Day 180	09/05/2011	17.8	.	13.5 - 18.0		
S241	303	Hepatitis B	Hepatitis B	3	MCH	pg	Day 180	09/05/2011	31	.	27.0 - 31.0		
S241	303	Hepatitis B	Hepatitis B	3	MCHC	gm %	Day 180	09/05/2011	34.2	.	32.0 - 36.0		
S241	303	Hepatitis B	Hepatitis B	3	MCV	fL	Day 180	09/05/2011	90.6	.	80.0- 96.0		
S241	303	Hepatitis B	Hepatitis B	3	Platelet Count	10E3/ul	Day 180	09/05/2011	295	.	150 - 400		
S241	303	Hepatitis B	Hepatitis B	3	RBC count	10E6/ul	Day 180	09/05/2011	5.75	.	4.60 - 6.20		
S241	303	Hepatitis B	Hepatitis B	3	TLC	10E3/ul	Day 180	09/05/2011	8.12	.	4.50 - 11.00		
S241	303	Hepatitis B	Hepatitis B	3	% Basophils	%	Day 208	06/06/2011	1.4	Yes	0.0 - 1.0	H	NCS
S241	303	Hepatitis B	Hepatitis B	3	% Eosinophils	%	Day 208	06/06/2011	1.8	.	1.0 - 6.0		
S241	303	Hepatitis B	Hepatitis B	3	% Lymphocytes	%	Day 208	06/06/2011	27.5	.	20.0 - 45.0		
S241	303	Hepatitis B	Hepatitis B	3	% Monocytes	%	Day 208	06/06/2011	4.3	.	2.0 - 10.0		
S241	303	Hepatitis B	Hepatitis B	3	% Neutrophils	%	Day 208	06/06/2011	64.9	.	40.0 - 75.0		
S241	303	Hepatitis B	Hepatitis B	3	Haematocrit	%	Day 208	06/06/2011	54.2	Yes	40.0 - 54.0	H	NCS
S241	303	Hepatitis B	Hepatitis B	3	Haemoglobin	gm / dl	Day 208	06/06/2011	18.4	Yes	13.5 - 18.0	H	NCS
S241	303	Hepatitis B	Hepatitis B	3	MCH	pg	Day 208	06/06/2011	30.7	.	27.0 - 31.0		
S241	303	Hepatitis B	Hepatitis B	3	MCHC	gm %	Day 208	06/06/2011	34.1	.	32.0 - 36.0		
S241	303	Hepatitis B	Hepatitis B	3	MCV	fL	Day 208	06/06/2011	90.2	.	80.0- 96.0		
S241	303	Hepatitis B	Hepatitis B	3	Platelet Count	10E3/ul	Day 208	06/06/2011	316	.	150 - 400		
S241	303	Hepatitis B	Hepatitis B	3	RBC count	10E6/ul	Day 208	06/06/2011	6.01	.	4.60 - 6.20		
S241	303	Hepatitis B	Hepatitis B	3	TLC	10E3/ul	Day 208	06/06/2011	8.31	.	4.50 - 11.00		

Subject No.	Randomisation Number	Study Group	Dosage	Dose	Haematology Parameters	Unit	Day	Date of sample Collection	Results	Out of Range	Reference Range	Flag	Clinical Significance **
S241	303	Hepatitis B	Hepatitis B	3	% Basophils	%	Day 365	09/11/2011	0	.	0.0 - 1.0		
S241	303	Hepatitis B	Hepatitis B	3	% Eosinophils	%	Day 365	09/11/2011	2	.	1.0 - 6.0		
S241	303	Hepatitis B	Hepatitis B	3	% Lymphocytes	%	Day 365	09/11/2011	30	.	20.0 - 45.0		
S241	303	Hepatitis B	Hepatitis B	3	% Monocytes	%	Day 365	09/11/2011	5	.	2.0 - 10.0		
S241	303	Hepatitis B	Hepatitis B	3	% Neutrophils	%	Day 365	09/11/2011	63	.	40.0 - 75.0		
S241	303	Hepatitis B	Hepatitis B	3	Haematocrit	%	Day 365	09/11/2011	48.9	.	40.0 - 54.0		
S241	303	Hepatitis B	Hepatitis B	3	Haemoglobin	gm / dl	Day 365	09/11/2011	16.5	.	13.5 - 18.0		
S241	303	Hepatitis B	Hepatitis B	3	MCH	pg	Day 365	09/11/2011	30	.	27.0 - 31.0		
S241	303	Hepatitis B	Hepatitis B	3	MCHC	gm %	Day 365	09/11/2011	33.7	.	32.0 - 36.0		
S241	303	Hepatitis B	Hepatitis B	3	MCV	fL	Day 365	09/11/2011	89.2	.	80.0- 96.0		
S241	303	Hepatitis B	Hepatitis B	3	Platelet Count	10E3/ul	Day 365	09/11/2011	288	.	150 - 400		
S241	303	Hepatitis B	Hepatitis B	3	RBC count	10E6/ul	Day 365	09/11/2011	5.49	.	4.60 - 6.20		
S241	303	Hepatitis B	Hepatitis B	3	TLC	10E3/ul	Day 365	09/11/2011	7.37	.	4.50 - 11.00		
S250	311	JAIVAC-1	Dosage C		% Basophils	%	Day -14 to Day -1	03/11/2010	1.4	Yes	0.0 - 1.0	H	NCS
S250	311	JAIVAC-1	Dosage C		% Eosinophils	%	Day -14 to Day -1	03/11/2010	6	.	1.0 - 6.0		
S250	311	JAIVAC-1	Dosage C		% Lymphocytes	%	Day -14 to Day -1	03/11/2010	40.3	.	20.0 - 45.0		
S250	311	JAIVAC-1	Dosage C		% Monocytes	%	Day -14 to Day -1	03/11/2010	4.4	.	2.0 - 10.0		
S250	311	JAIVAC-1	Dosage C		% Neutrophils	%	Day -14 to Day -1	03/11/2010	47.8	.	40.0 - 75.0		
S250	311	JAIVAC-1	Dosage C		Haematocrit	%	Day -14 to Day -1	03/11/2010	46.8	.	40.0 - 54.0		
S250	311	JAIVAC-1	Dosage C		Haemoglobin	gm / dl	Day -14 to Day -1	03/11/2010	15.7	.	13.5 - 18.0		
S250	311	JAIVAC-1	Dosage C		MCH	pg	Day -14 to Day -1	03/11/2010	30.3	.	27.0 - 31.0		
S250	311	JAIVAC-1	Dosage C		MCHC	gm %	Day -14 to Day -1	03/11/2010	33.6	.	32.0 - 36.0		
S250	311	JAIVAC-1	Dosage C		MCV	fL	Day -14 to Day -1	03/11/2010	90.1	.	80.0- 96.0		
S250	311	JAIVAC-1	Dosage C		Platelet Count	10E3/ul	Day -14 to Day -1	03/11/2010	210	.	150 - 400		
S250	311	JAIVAC-1	Dosage C		RBC count	10E6/ul	Day -14 to Day -1	03/11/2010	5.19	.	4.60 - 6.20		
S250	311	JAIVAC-1	Dosage C		TLC	10E3/ul	Day -14 to Day -1	03/11/2010	5.74	.	4.50 - 11.00		
S250	311	JAIVAC-1	Dosage C	2	% Basophils	%	Day 28	10/12/2010	0.8	.	0.0 - 1.0		
S250	311	JAIVAC-1	Dosage C	2	% Eosinophils	%	Day 28	10/12/2010	6.9	Yes	1.0 - 6.0	H	NCS
S250	311	JAIVAC-1	Dosage C	2	% Lymphocytes	%	Day 28	10/12/2010	33	.	20.0 - 45.0		

Subject No.	Randomisation Number	Study Group	Dosage	Dose	Haematology Parameters	Unit	Day	Date of sample Collection	Results	Out of Range	Reference Range	Flag	Clinical Significance **
S250	311	JAIVAC-1	Dosage C	2	% Monocytes	%	Day 28	10/12/2010	5.2	.	2.0 - 10.0		
S250	311	JAIVAC-1	Dosage C	2	% Neutrophils	%	Day 28	10/12/2010	54	.	40.0 - 75.0		
S250	311	JAIVAC-1	Dosage C	2	Haematocrit	%	Day 28	10/12/2010	50.3	.	40.0 - 54.0		
S250	311	JAIVAC-1	Dosage C	2	Haemoglobin	gm / dl	Day 28	10/12/2010	17	.	13.5 - 18.0		
S250	311	JAIVAC-1	Dosage C	2	MCH	pg	Day 28	10/12/2010	30.6	.	27.0 - 31.0		
S250	311	JAIVAC-1	Dosage C	2	MCHC	gm %	Day 28	10/12/2010	33.8	.	32.0 - 36.0		
S250	311	JAIVAC-1	Dosage C	2	MCV	fL	Day 28	10/12/2010	90.5	.	80.0- 96.0		
S250	311	JAIVAC-1	Dosage C	2	Platelet Count	10E3/ul	Day 28	10/12/2010	206	.	150 - 400		
S250	311	JAIVAC-1	Dosage C	2	RBC count	10E6/ul	Day 28	10/12/2010	5.56	.	4.60 - 6.20		
S250	311	JAIVAC-1	Dosage C	2	TLC	10E3/ul	Day 28	10/12/2010	5.61	.	4.50 - 11.00		
S250	311	JAIVAC-1	Dosage C	2	% Basophils	%	Day 56	07/01/2011	1.2	Yes	0.0 - 1.0	H	NCS
S250	311	JAIVAC-1	Dosage C	2	% Eosinophils	%	Day 56	07/01/2011	5.7	.	1.0 - 6.0		
S250	311	JAIVAC-1	Dosage C	2	% Lymphocytes	%	Day 56	07/01/2011	29.7	.	20.0 - 45.0		
S250	311	JAIVAC-1	Dosage C	2	% Monocytes	%	Day 56	07/01/2011	5.4	.	2.0 - 10.0		
S250	311	JAIVAC-1	Dosage C	2	% Neutrophils	%	Day 56	07/01/2011	58.1	.	40.0 - 75.0		
S250	311	JAIVAC-1	Dosage C	2	Haematocrit	%	Day 56	07/01/2011	48.4	.	40.0 - 54.0		
S250	311	JAIVAC-1	Dosage C	2	Haemoglobin	gm / dl	Day 56	07/01/2011	16.8	.	13.5 - 18.0		
S250	311	JAIVAC-1	Dosage C	2	MCH	pg	Day 56	07/01/2011	31.6	Yes	27.0 - 31.0	H	NCS
S250	311	JAIVAC-1	Dosage C	2	MCHC	gm %	Day 56	07/01/2011	34.7	.	32.0 - 36.0		
S250	311	JAIVAC-1	Dosage C	2	MCV	fL	Day 56	07/01/2011	91	.	80.0- 96.0		
S250	311	JAIVAC-1	Dosage C	2	Platelet Count	10E3/ul	Day 56	07/01/2011	230	.	150 - 400		
S250	311	JAIVAC-1	Dosage C	2	RBC count	10E6/ul	Day 56	07/01/2011	5.32	.	4.60 - 6.20		
S250	311	JAIVAC-1	Dosage C	2	TLC	10E3/ul	Day 56	07/01/2011	5	.	4.50 - 11.00		
S250	311	JAIVAC-1	Dosage C	3	% Basophils	%	Day 180	11/05/2011	1.2	Yes	0.0 - 1.0	H	NCS
S250	311	JAIVAC-1	Dosage C	3	% Eosinophils	%	Day 180	11/05/2011	6.3	Yes	1.0 - 6.0	H	NCS
S250	311	JAIVAC-1	Dosage C	3	% Lymphocytes	%	Day 180	11/05/2011	29.5	.	20.0 - 45.0		
S250	311	JAIVAC-1	Dosage C	3	% Monocytes	%	Day 180	11/05/2011	4.4	.	2.0 - 10.0		
S250	311	JAIVAC-1	Dosage C	3	% Neutrophils	%	Day 180	11/05/2011	58.6	.	40.0 - 75.0		
S250	311	JAIVAC-1	Dosage C	3	Haematocrit	%	Day 180	11/05/2011	46.6	.	40.0 - 54.0		

Subject No.	Randomisation Number	Study Group	Dosage	Dose	Haematology Parameters	Unit	Day	Date of sample Collection	Results	Out of Range	Reference Range	Flag	Clinical Significance **
S250	311	JAIVAC-1	Dosage C	3	Haemoglobin	gm / dl	Day 180	11/05/2011	16.1	.	13.5 - 18.0		
S250	311	JAIVAC-1	Dosage C	3	MCH	pg	Day 180	11/05/2011	32.9	Yes	27.0 - 31.0	H	NCS
S250	311	JAIVAC-1	Dosage C	3	MCHC	gm %	Day 180	11/05/2011	34.5	.	32.0 - 36.0		
S250	311	JAIVAC-1	Dosage C	3	MCV	fL	Day 180	11/05/2011	95.2	.	80.0- 96.0		
S250	311	JAIVAC-1	Dosage C	3	Platelet Count	10E3/ul	Day 180	11/05/2011	211	.	150 - 400		
S250	311	JAIVAC-1	Dosage C	3	RBC count	10E6/ul	Day 180	11/05/2011	4.89	.	4.60 - 6.20		
S250	311	JAIVAC-1	Dosage C	3	TLC	10E3/ul	Day 180	11/05/2011	5.24	.	4.50 - 11.00		
S250	311	JAIVAC-1	Dosage C	3	% Basophils	%	Day 208	08/06/2011	1.4	Yes	0.0 - 1.0	H	NCS
S250	311	JAIVAC-1	Dosage C	3	% Eosinophils	%	Day 208	08/06/2011	8.4	Yes	1.0 - 6.0	H	NCS
S250	311	JAIVAC-1	Dosage C	3	% Lymphocytes	%	Day 208	08/06/2011	28.8	.	20.0 - 45.0		
S250	311	JAIVAC-1	Dosage C	3	% Monocytes	%	Day 208	08/06/2011	6.3	.	2.0 - 10.0		
S250	311	JAIVAC-1	Dosage C	3	% Neutrophils	%	Day 208	08/06/2011	55.2	.	40.0 - 75.0		
S250	311	JAIVAC-1	Dosage C	3	Haematocrit	%	Day 208	08/06/2011	46.3	.	40.0 - 54.0		
S250	311	JAIVAC-1	Dosage C	3	Haemoglobin	gm / dl	Day 208	08/06/2011	15.9	.	13.5 - 18.0		
S250	311	JAIVAC-1	Dosage C	3	MCH	pg	Day 208	08/06/2011	32.6	Yes	27.0 - 31.0	H	NCS
S250	311	JAIVAC-1	Dosage C	3	MCHC	gm %	Day 208	08/06/2011	34.2	.	32.0 - 36.0		
S250	311	JAIVAC-1	Dosage C	3	MCV	fL	Day 208	08/06/2011	95.3	.	80.0- 96.0		
S250	311	JAIVAC-1	Dosage C	3	Platelet Count	10E3/ul	Day 208	08/06/2011	220	.	150 - 400		
S250	311	JAIVAC-1	Dosage C	3	RBC count	10E6/ul	Day 208	08/06/2011	4.86	.	4.60 - 6.20		
S250	311	JAIVAC-1	Dosage C	3	TLC	10E3/ul	Day 208	08/06/2011	4.47	Yes	4.50 - 11.00	L	NCS
S250	311	JAIVAC-1	Dosage C	3	% Basophils	%	Day 365	10/11/2011	1.7	Yes	0.0 - 1.0	H	NCS
S250	311	JAIVAC-1	Dosage C	3	% Eosinophils	%	Day 365	10/11/2011	6.2	Yes	1.0 - 6.0	H	NCS
S250	311	JAIVAC-1	Dosage C	3	% Lymphocytes	%	Day 365	10/11/2011	33.4	.	20.0 - 45.0		
S250	311	JAIVAC-1	Dosage C	3	% Monocytes	%	Day 365	10/11/2011	6.9	.	2.0 - 10.0		
S250	311	JAIVAC-1	Dosage C	3	% Neutrophils	%	Day 365	10/11/2011	51.9	.	40.0 - 75.0		
S250	311	JAIVAC-1	Dosage C	3	Haematocrit	%	Day 365	10/11/2011	46.4	.	40.0 - 54.0		
S250	311	JAIVAC-1	Dosage C	3	Haemoglobin	gm / dl	Day 365	10/11/2011	15.4	.	13.5 - 18.0		
S250	311	JAIVAC-1	Dosage C	3	MCH	pg	Day 365	10/11/2011	30.2	.	27.0 - 31.0		
S250	311	JAIVAC-1	Dosage C	3	MCHC	gm %	Day 365	10/11/2011	33.2	.	32.0 - 36.0		

Subject No.	Randomisation Number	Study Group	Dosage	Dose	Haematology Parameters	Unit	Day	Date of sample Collection	Results	Out of Range	Reference Range	Flag	Clinical Significance **
S250	311	JAIVAC-1	Dosage C	3	MCV	fL	Day 365	10/11/2011	90.9	.	80.0- 96.0		
S250	311	JAIVAC-1	Dosage C	3	Platelet Count	10E3/ul	Day 365	10/11/2011	165	.	150 - 400		
S250	311	JAIVAC-1	Dosage C	3	RBC count	10E6/ul	Day 365	10/11/2011	5.11	.	4.60 - 6.20		
S250	311	JAIVAC-1	Dosage C	3	TLC	10E3/ul	Day 365	10/11/2011	4.94	.	4.50 - 11.00		
S263	313	Hepatitis B	Hepatitis B		% Basophils	%	Day -14 to Day -1	09/11/2010	0.9	.	0.0 - 1.0		
S263	313	Hepatitis B	Hepatitis B		% Eosinophils	%	Day -14 to Day -1	09/11/2010	2.1	.	1.0 - 6.0		
S263	313	Hepatitis B	Hepatitis B		% Lymphocytes	%	Day -14 to Day -1	09/11/2010	23	.	20.0 - 45.0		
S263	313	Hepatitis B	Hepatitis B		% Monocytes	%	Day -14 to Day -1	09/11/2010	3.5	.	2.0 - 10.0		
S263	313	Hepatitis B	Hepatitis B		% Neutrophils	%	Day -14 to Day -1	09/11/2010	70.5	.	40.0 - 75.0		
S263	313	Hepatitis B	Hepatitis B		Haematocrit	%	Day -14 to Day -1	09/11/2010	49	.	40.0 - 54.0		
S263	313	Hepatitis B	Hepatitis B		Haemoglobin	gm / dl	Day -14 to Day -1	09/11/2010	16.3	.	13.5 - 18.0		
S263	313	Hepatitis B	Hepatitis B		MCH	pg	Day -14 to Day -1	09/11/2010	29.1	.	27.0 - 31.0		
S263	313	Hepatitis B	Hepatitis B		MCHC	gm %	Day -14 to Day -1	09/11/2010	33.3	.	32.0 - 36.0		
S263	313	Hepatitis B	Hepatitis B		MCV	fL	Day -14 to Day -1	09/11/2010	87.3	.	80.0- 96.0		
S263	313	Hepatitis B	Hepatitis B		Platelet Count	10E3/ul	Day -14 to Day -1	09/11/2010	345	.	150 - 400		
S263	313	Hepatitis B	Hepatitis B		RBC count	10E6/ul	Day -14 to Day -1	09/11/2010	5.61	.	4.60 - 6.20		
S263	313	Hepatitis B	Hepatitis B		TLC	10E3/ul	Day -14 to Day -1	09/11/2010	9.42	.	4.50 - 11.00		
S263	313	Hepatitis B	Hepatitis B	2	% Basophils	%	Day 28	10/12/2010	1.5	Yes	0.0 - 1.0	H	NCS
S263	313	Hepatitis B	Hepatitis B	2	% Eosinophils	%	Day 28	10/12/2010	3.6	.	1.0 - 6.0		
S263	313	Hepatitis B	Hepatitis B	2	% Lymphocytes	%	Day 28	10/12/2010	19.9	Yes	20.0 - 45.0	L	NCS
S263	313	Hepatitis B	Hepatitis B	2	% Monocytes	%	Day 28	10/12/2010	4.9	.	2.0 - 10.0		
S263	313	Hepatitis B	Hepatitis B	2	% Neutrophils	%	Day 28	10/12/2010	70.2	.	40.0 - 75.0		
S263	313	Hepatitis B	Hepatitis B	2	Haematocrit	%	Day 28	10/12/2010	51.5	.	40.0 - 54.0		
S263	313	Hepatitis B	Hepatitis B	2	Haemoglobin	gm / dl	Day 28	10/12/2010	16.6	.	13.5 - 18.0		
S263	313	Hepatitis B	Hepatitis B	2	MCH	pg	Day 28	10/12/2010	28.3	.	27.0 - 31.0		
S263	313	Hepatitis B	Hepatitis B	2	MCHC	gm %	Day 28	10/12/2010	32.2	.	32.0 - 36.0		
S263	313	Hepatitis B	Hepatitis B	2	MCV	fL	Day 28	10/12/2010	88	.	80.0- 96.0		
S263	313	Hepatitis B	Hepatitis B	2	Platelet Count	10E3/ul	Day 28	10/12/2010	327	.	150 - 400		
S263	313	Hepatitis B	Hepatitis B	2	RBC count	10E6/ul	Day 28	10/12/2010	5.86	.	4.60 - 6.20		

Subject No.	Randomisation Number	Study Group	Dosage	Dose	Haematology Parameters	Unit	Day	Date of sample Collection	Results	Out of Range	Reference Range	Flag	Clinical Significance **
S263	313	Hepatitis B	Hepatitis B	2	TLC	10E3/ul	Day 28	10/12/2010	7.94	.	4.50 - 11.00		
S263	313	Hepatitis B	Hepatitis B	2	% Basophils	%	Day 56	07/01/2011	1.6	Yes	0.0 - 1.0	H	NCS
S263	313	Hepatitis B	Hepatitis B	2	% Eosinophils	%	Day 56	07/01/2011	3.8	.	1.0 - 6.0		
S263	313	Hepatitis B	Hepatitis B	2	% Lymphocytes	%	Day 56	07/01/2011	24.1	.	20.0 - 45.0		
S263	313	Hepatitis B	Hepatitis B	2	% Monocytes	%	Day 56	07/01/2011	4.4	.	2.0 - 10.0		
S263	313	Hepatitis B	Hepatitis B	2	% Neutrophils	%	Day 56	07/01/2011	66.1	.	40.0 - 75.0		
S263	313	Hepatitis B	Hepatitis B	2	Haematocrit	%	Day 56	07/01/2011	50	.	40.0 - 54.0		
S263	313	Hepatitis B	Hepatitis B	2	Haemoglobin	gm / dl	Day 56	07/01/2011	16.6	.	13.5 - 18.0		
S263	313	Hepatitis B	Hepatitis B	2	MCH	pg	Day 56	07/01/2011	28.9	.	27.0 - 31.0		
S263	313	Hepatitis B	Hepatitis B	2	MCHC	gm %	Day 56	07/01/2011	33.2	.	32.0 - 36.0		
S263	313	Hepatitis B	Hepatitis B	2	MCV	fL	Day 56	07/01/2011	87.1	.	80.0- 96.0		
S263	313	Hepatitis B	Hepatitis B	2	Platelet Count	10E3/ul	Day 56	07/01/2011	300	.	150 - 400		
S263	313	Hepatitis B	Hepatitis B	2	RBC count	10E6/ul	Day 56	07/01/2011	5.74	.	4.60 - 6.20		
S263	313	Hepatitis B	Hepatitis B	2	TLC	10E3/ul	Day 56	07/01/2011	7.97	.	4.50 - 11.00		
S263	313	Hepatitis B	Hepatitis B	3	% Basophils	%	Day 180	11/05/2011	0.5	.	0.0 - 1.0		
S263	313	Hepatitis B	Hepatitis B	3	% Eosinophils	%	Day 180	11/05/2011	3.2	.	1.0 - 6.0		
S263	313	Hepatitis B	Hepatitis B	3	% Lymphocytes	%	Day 180	11/05/2011	12.6	Yes	20.0 - 45.0	L	NCS
S263	313	Hepatitis B	Hepatitis B	3	% Monocytes	%	Day 180	11/05/2011	3.6	.	2.0 - 10.0		
S263	313	Hepatitis B	Hepatitis B	3	% Neutrophils	%	Day 180	11/05/2011	80.1	Yes	40.0 - 75.0	H	NCS
S263	313	Hepatitis B	Hepatitis B	3	Haematocrit	%	Day 180	11/05/2011	53.5	.	40.0 - 54.0		
S263	313	Hepatitis B	Hepatitis B	3	Haemoglobin	gm / dl	Day 180	11/05/2011	17.2	.	13.5 - 18.0		
S263	313	Hepatitis B	Hepatitis B	3	MCH	pg	Day 180	11/05/2011	29.1	.	27.0 - 31.0		
S263	313	Hepatitis B	Hepatitis B	3	MCHC	gm %	Day 180	11/05/2011	32.2	.	32.0 - 36.0		
S263	313	Hepatitis B	Hepatitis B	3	MCV	fL	Day 180	11/05/2011	90.3	.	80.0- 96.0		
S263	313	Hepatitis B	Hepatitis B	3	Platelet Count	10E3/ul	Day 180	11/05/2011	305	.	150 - 400		
S263	313	Hepatitis B	Hepatitis B	3	RBC count	10E6/ul	Day 180	11/05/2011	5.92	.	4.60 - 6.20		
S263	313	Hepatitis B	Hepatitis B	3	TLC	10E3/ul	Day 180	11/05/2011	10.79	.	4.50 - 11.00		
S263	313	Hepatitis B	Hepatitis B	3	% Basophils	%	Day 208	08/06/2011	1.1	Yes	0.0 - 1.0	H	NCS
S263	313	Hepatitis B	Hepatitis B	3	% Eosinophils	%	Day 208	08/06/2011	5.7	.	1.0 - 6.0		

Subject No.	Randomisation Number	Study Group	Dosage	Dose	Haematology Parameters	Unit	Day	Date of sample Collection	Results	Out of Range	Reference Range	Flag	Clinical Significance **
S263	313	Hepatitis B	Hepatitis B	3	% Lymphocytes	%	Day 208	08/06/2011	27.3	.	20.0 - 45.0		
S263	313	Hepatitis B	Hepatitis B	3	% Monocytes	%	Day 208	08/06/2011	4.9	.	2.0 - 10.0		
S263	313	Hepatitis B	Hepatitis B	3	% Neutrophils	%	Day 208	08/06/2011	60.9	.	40.0 - 75.0		
S263	313	Hepatitis B	Hepatitis B	3	Haematocrit	%	Day 208	08/06/2011	49.4	.	40.0 - 54.0		
S263	313	Hepatitis B	Hepatitis B	3	Haemoglobin	gm / dl	Day 208	08/06/2011	16.1	.	13.5 - 18.0		
S263	313	Hepatitis B	Hepatitis B	3	MCH	pg	Day 208	08/06/2011	29.3	.	27.0 - 31.0		
S263	313	Hepatitis B	Hepatitis B	3	MCHC	gm %	Day 208	08/06/2011	32.6	.	32.0 - 36.0		
S263	313	Hepatitis B	Hepatitis B	3	MCV	fL	Day 208	08/06/2011	90	.	80.0- 96.0		
S263	313	Hepatitis B	Hepatitis B	3	Platelet Count	10E3/ul	Day 208	08/06/2011	326	.	150 - 400		
S263	313	Hepatitis B	Hepatitis B	3	RBC count	10E6/ul	Day 208	08/06/2011	5.5	.	4.60 - 6.20		
S263	313	Hepatitis B	Hepatitis B	3	TLC	10E3/ul	Day 208	08/06/2011	7.26	.	4.50 - 11.00		
S263	313	Hepatitis B	Hepatitis B	3	% Basophils	%	Day 365	10/11/2011	1.5	Yes	0.0 - 1.0	H	NCS
S263	313	Hepatitis B	Hepatitis B	3	% Eosinophils	%	Day 365	10/11/2011	3.8	.	1.0 - 6.0		
S263	313	Hepatitis B	Hepatitis B	3	% Lymphocytes	%	Day 365	10/11/2011	20.4	.	20.0 - 45.0		
S263	313	Hepatitis B	Hepatitis B	3	% Monocytes	%	Day 365	10/11/2011	3.4	.	2.0 - 10.0		
S263	313	Hepatitis B	Hepatitis B	3	% Neutrophils	%	Day 365	10/11/2011	70.9	.	40.0 - 75.0		
S263	313	Hepatitis B	Hepatitis B	3	Haematocrit	%	Day 365	10/11/2011	48.7	.	40.0 - 54.0		
S263	313	Hepatitis B	Hepatitis B	3	Haemoglobin	gm / dl	Day 365	10/11/2011	16.1	.	13.5 - 18.0		
S263	313	Hepatitis B	Hepatitis B	3	MCH	pg	Day 365	10/11/2011	30.1	.	27.0 - 31.0		
S263	313	Hepatitis B	Hepatitis B	3	MCHC	gm %	Day 365	10/11/2011	33.1	.	32.0 - 36.0		
S263	313	Hepatitis B	Hepatitis B	3	MCV	fL	Day 365	10/11/2011	90.7	.	80.0- 96.0		
S263	313	Hepatitis B	Hepatitis B	3	Platelet Count	10E3/ul	Day 365	10/11/2011	262	.	150 - 400		
S263	313	Hepatitis B	Hepatitis B	3	RBC count	10E6/ul	Day 365	10/11/2011	5.37	.	4.60 - 6.20		
S263	313	Hepatitis B	Hepatitis B	3	TLC	10E3/ul	Day 365	10/11/2011	7.72	.	4.50 - 11.00		
S264	306	JAIVAC-1	Dosage C		% Basophils	%	Day -14 to Day -1	03/11/2010	0.9	.	0.0 - 1.0		
S264	306	JAIVAC-1	Dosage C		% Eosinophils	%	Day -14 to Day -1	03/11/2010	6.2	.	1.0 - 6.0	H	
S264	306	JAIVAC-1	Dosage C		% Lymphocytes	%	Day -14 to Day -1	03/11/2010	42.2	.	20.0 - 45.0		
S264	306	JAIVAC-1	Dosage C		% Monocytes	%	Day -14 to Day -1	03/11/2010	5.3	.	2.0 - 10.0		
S264	306	JAIVAC-1	Dosage C		% Neutrophils	%	Day -14 to Day -1	03/11/2010	45.3	.	40.0 - 75.0		

Subject No.	Randomisation Number	Study Group	Dosage	Dose	Haematology Parameters	Unit	Day	Date of sample Collection	Results	Out of Range	Reference Range	Flag	Clinical Significance **
S264	306	JAIVAC-1	Dosage C		Haematocrit	%	Day -14 to Day -1	03/11/2010	44.5	.	40.0 - 54.0		
S264	306	JAIVAC-1	Dosage C		Haemoglobin	gm / dl	Day -14 to Day -1	03/11/2010	14.4	.	13.5 - 18.0		
S264	306	JAIVAC-1	Dosage C		MCH	pg	Day -14 to Day -1	03/11/2010	26.5	Yes	27.0 - 31.0	L	NCS
S264	306	JAIVAC-1	Dosage C		MCHC	gm %	Day -14 to Day -1	03/11/2010	32.4	.	32.0 - 36.0		
S264	306	JAIVAC-1	Dosage C		MCV	fL	Day -14 to Day -1	03/11/2010	81.8	.	80.0- 96.0		
S264	306	JAIVAC-1	Dosage C		Platelet Count	10E3/ul	Day -14 to Day -1	03/11/2010	353	.	150 - 400		
S264	306	JAIVAC-1	Dosage C		RBC count	10E6/ul	Day -14 to Day -1	03/11/2010	5.44	.	4.60 - 6.20		
S264	306	JAIVAC-1	Dosage C		TLC	10E3/ul	Day -14 to Day -1	03/11/2010	7.7	.	4.50 - 11.00		
S264	306	JAIVAC-1	Dosage C	2	% Basophils	%	Day 28	09/12/2010	0.5	.	0.0 - 1.0		
S264	306	JAIVAC-1	Dosage C	2	% Eosinophils	%	Day 28	09/12/2010	6	.	1.0 - 6.0		
S264	306	JAIVAC-1	Dosage C	2	% Lymphocytes	%	Day 28	09/12/2010	43.4	.	20.0 - 45.0		
S264	306	JAIVAC-1	Dosage C	2	% Monocytes	%	Day 28	09/12/2010	5.9	.	2.0 - 10.0		
S264	306	JAIVAC-1	Dosage C	2	% Neutrophils	%	Day 28	09/12/2010	44	.	40.0 - 75.0		
S264	306	JAIVAC-1	Dosage C	2	Haematocrit	%	Day 28	09/12/2010	45	.	40.0 - 54.0		
S264	306	JAIVAC-1	Dosage C	2	Haemoglobin	gm / dl	Day 28	09/12/2010	14.4	.	13.5 - 18.0		
S264	306	JAIVAC-1	Dosage C	2	MCH	pg	Day 28	09/12/2010	26.1	Yes	27.0 - 31.0	L	NCS
S264	306	JAIVAC-1	Dosage C	2	MCHC	gm %	Day 28	09/12/2010	31.9	Yes	32.0 - 36.0	L	NCS
S264	306	JAIVAC-1	Dosage C	2	MCV	fL	Day 28	09/12/2010	81.6	.	80.0- 96.0		
S264	306	JAIVAC-1	Dosage C	2	Platelet Count	10E3/ul	Day 28	09/12/2010	346	.	150 - 400		
S264	306	JAIVAC-1	Dosage C	2	RBC count	10E6/ul	Day 28	09/12/2010	5.52	.	4.60 - 6.20		
S264	306	JAIVAC-1	Dosage C	2	TLC	10E3/ul	Day 28	09/12/2010	6.58	.	4.50 - 11.00		
S264	306	JAIVAC-1	Dosage C	2	% Basophils	%	Day 56	06/01/2011	0.6	.	0.0 - 1.0		
S264	306	JAIVAC-1	Dosage C	2	% Eosinophils	%	Day 56	06/01/2011	4.2	.	1.0 - 6.0		
S264	306	JAIVAC-1	Dosage C	2	% Lymphocytes	%	Day 56	06/01/2011	35.5	.	20.0 - 45.0		
S264	306	JAIVAC-1	Dosage C	2	% Monocytes	%	Day 56	06/01/2011	8.5	.	2.0 - 10.0		
S264	306	JAIVAC-1	Dosage C	2	% Neutrophils	%	Day 56	06/01/2011	51.2	.	40.0 - 75.0		
S264	306	JAIVAC-1	Dosage C	2	Haematocrit	%	Day 56	06/01/2011	46.5	.	40.0 - 54.0		
S264	306	JAIVAC-1	Dosage C	2	Haemoglobin	gm / dl	Day 56	06/01/2011	15.2	.	13.5 - 18.0		
S264	306	JAIVAC-1	Dosage C	2	MCH	pg	Day 56	06/01/2011	26.6	Yes	27.0 - 31.0	L	NCS

Subject No.	Randomisation Number	Study Group	Dosage	Dose	Haematology Parameters	Unit	Day	Date of sample Collection	Results	Out of Range	Reference Range	Flag	Clinical Significance **
S264	306	JAIVAC-1	Dosage C	2	MCHC	gm %	Day 56	06/01/2011	32.7	.	32.0 - 36.0		
S264	306	JAIVAC-1	Dosage C	2	MCV	fL	Day 56	06/01/2011	81.4	.	80.0- 96.0		
S264	306	JAIVAC-1	Dosage C	2	Platelet Count	10E3/ul	Day 56	06/01/2011	341	.	150 - 400		
S264	306	JAIVAC-1	Dosage C	2	RBC count	10E6/ul	Day 56	06/01/2011	5.71	.	4.60 - 6.20		
S264	306	JAIVAC-1	Dosage C	2	TLC	10E3/ul	Day 56	06/01/2011	7.71	.	4.50 - 11.00		
S264	306	JAIVAC-1	Dosage C	3	% Basophils	%	Day 180	10/05/2011	1	.	0.0 - 1.0		
S264	306	JAIVAC-1	Dosage C	3	% Eosinophils	%	Day 180	10/05/2011	5.2	.	1.0 - 6.0		
S264	306	JAIVAC-1	Dosage C	3	% Lymphocytes	%	Day 180	10/05/2011	42.7	.	20.0 - 45.0		
S264	306	JAIVAC-1	Dosage C	3	% Monocytes	%	Day 180	10/05/2011	5.4	.	2.0 - 10.0		
S264	306	JAIVAC-1	Dosage C	3	% Neutrophils	%	Day 180	10/05/2011	45.7	.	40.0 - 75.0		
S264	306	JAIVAC-1	Dosage C	3	Haematocrit	%	Day 180	10/05/2011	43.8	.	40.0 - 54.0		
S264	306	JAIVAC-1	Dosage C	3	Haemoglobin	gm / dl	Day 180	10/05/2011	14	.	13.5 - 18.0		
S264	306	JAIVAC-1	Dosage C	3	MCH	pg	Day 180	10/05/2011	25.7	Yes	27.0 - 31.0	L	NCS
S264	306	JAIVAC-1	Dosage C	3	MCHC	gm %	Day 180	10/05/2011	32	.	32.0 - 36.0		
S264	306	JAIVAC-1	Dosage C	3	MCV	fL	Day 180	10/05/2011	80.3	.	80.0- 96.0		
S264	306	JAIVAC-1	Dosage C	3	Platelet Count	10E3/ul	Day 180	10/05/2011	336	.	150 - 400		
S264	306	JAIVAC-1	Dosage C	3	RBC count	10E6/ul	Day 180	10/05/2011	5.45	.	4.60 - 6.20		
S264	306	JAIVAC-1	Dosage C	3	TLC	10E3/ul	Day 180	10/05/2011	6.47	.	4.50 - 11.00		
S264	306	JAIVAC-1	Dosage C	3	% Basophils	%	Day 208	07/06/2011	0	.	0.0 - 1.0		
S264	306	JAIVAC-1	Dosage C	3	% Eosinophils	%	Day 208	07/06/2011	8	Yes	1.0 - 6.0	H	NCS
S264	306	JAIVAC-1	Dosage C	3	% Lymphocytes	%	Day 208	07/06/2011	50	Yes	20.0 - 45.0	H	NCS
S264	306	JAIVAC-1	Dosage C	3	% Monocytes	%	Day 208	07/06/2011	4	.	2.0 - 10.0		
S264	306	JAIVAC-1	Dosage C	3	% Neutrophils	%	Day 208	07/06/2011	38	Yes	40.0 - 75.0	L	NCS
S264	306	JAIVAC-1	Dosage C	3	Haematocrit	%	Day 208	07/06/2011	45.7	.	40.0 - 54.0		
S264	306	JAIVAC-1	Dosage C	3	Haemoglobin	gm / dl	Day 208	07/06/2011	14.4	.	13.5 - 18.0		
S264	306	JAIVAC-1	Dosage C	3	MCH	pg	Day 208	07/06/2011	25.1	Yes	27.0 - 31.0	L	NCS
S264	306	JAIVAC-1	Dosage C	3	MCHC	gm %	Day 208	07/06/2011	31.5	Yes	32.0 - 36.0	L	NCS
S264	306	JAIVAC-1	Dosage C	3	MCV	fL	Day 208	07/06/2011	79.8	Yes	80.0- 96.0	L	NCS
S264	306	JAIVAC-1	Dosage C	3	Platelet Count	10E3/ul	Day 208	07/06/2011	353	.	150 - 400		

Subject No.	Randomisation Number	Study Group	Dosage	Dose	Haematology Parameters	Unit	Day	Date of sample Collection	Results	Out of Range	Reference Range	Flag	Clinical Significance **
S264	306	JAIVAC-1	Dosage C	3	RBC count	10E6/ul	Day 208	07/06/2011	5.72	.	4.60 - 6.20		
S264	306	JAIVAC-1	Dosage C	3	TLC	10E3/ul	Day 208	07/06/2011	5.8	.	4.50 - 11.00		
S264	306	JAIVAC-1	Dosage C	3	% Basophils	%	Day 365	09/11/2011	1.1	Yes	0.0 - 1.0	H	NCS
S264	306	JAIVAC-1	Dosage C	3	% Eosinophils	%	Day 365	09/11/2011	6.6	Yes	1.0 - 6.0	H	NCS
S264	306	JAIVAC-1	Dosage C	3	% Lymphocytes	%	Day 365	09/11/2011	43.7	.	20.0 - 45.0		
S264	306	JAIVAC-1	Dosage C	3	% Monocytes	%	Day 365	09/11/2011	6.3	.	2.0 - 10.0		
S264	306	JAIVAC-1	Dosage C	3	% Neutrophils	%	Day 365	09/11/2011	42.3	.	40.0 - 75.0		
S264	306	JAIVAC-1	Dosage C	3	Haematocrit	%	Day 365	09/11/2011	43.3	.	40.0 - 54.0		
S264	306	JAIVAC-1	Dosage C	3	Haemoglobin	gm / dl	Day 365	09/11/2011	13.6	.	13.5 - 18.0		
S264	306	JAIVAC-1	Dosage C	3	MCH	pg	Day 365	09/11/2011	24.9	Yes	27.0 - 31.0	L	NCS
S264	306	JAIVAC-1	Dosage C	3	MCHC	gm %	Day 365	09/11/2011	31.5	Yes	32.0 - 36.0	L	NCS
S264	306	JAIVAC-1	Dosage C	3	MCV	fL	Day 365	09/11/2011	79.2	Yes	80.0- 96.0	L	NCS
S264	306	JAIVAC-1	Dosage C	3	Platelet Count	10E3/ul	Day 365	09/11/2011	337	.	150 - 400		
S264	306	JAIVAC-1	Dosage C	3	RBC count	10E6/ul	Day 365	09/11/2011	5.46	.	4.60 - 6.20		
S264	306	JAIVAC-1	Dosage C	3	TLC	10E3/ul	Day 365	09/11/2011	6.53	.	4.50 - 11.00		
S266	308	JAIVAC-1	Dosage C		% Basophils	%	Day -14 to Day -1	03/11/2010	0	.	0.0 - 1.0		
S266	308	JAIVAC-1	Dosage C		% Eosinophils	%	Day -14 to Day -1	03/11/2010	3	.	1.0 - 6.0		
S266	308	JAIVAC-1	Dosage C		% Lymphocytes	%	Day -14 to Day -1	03/11/2010	40	.	20.0 - 45.0		
S266	308	JAIVAC-1	Dosage C		% Monocytes	%	Day -14 to Day -1	03/11/2010	8	.	2.0 - 10.0		
S266	308	JAIVAC-1	Dosage C		% Neutrophils	%	Day -14 to Day -1	03/11/2010	49	.	40.0 - 75.0		
S266	308	JAIVAC-1	Dosage C		Haematocrit	%	Day -14 to Day -1	03/11/2010	50.1	.	40.0 - 54.0		
S266	308	JAIVAC-1	Dosage C		Haemoglobin	gm / dl	Day -14 to Day -1	03/11/2010	16.5	.	13.5 - 18.0		
S266	308	JAIVAC-1	Dosage C		MCH	pg	Day -14 to Day -1	03/11/2010	29.8	.	27.0 - 31.0		
S266	308	JAIVAC-1	Dosage C		MCHC	gm %	Day -14 to Day -1	03/11/2010	32.9	.	32.0 - 36.0		
S266	308	JAIVAC-1	Dosage C		MCV	fL	Day -14 to Day -1	03/11/2010	90.4	.	80.0- 96.0		
S266	308	JAIVAC-1	Dosage C		Platelet Count	10E3/ul	Day -14 to Day -1	03/11/2010	408	.	150 - 400	H	
S266	308	JAIVAC-1	Dosage C		RBC count	10E6/ul	Day -14 to Day -1	03/11/2010	5.54	.	4.60 - 6.20		
S266	308	JAIVAC-1	Dosage C		TLC	10E3/ul	Day -14 to Day -1	03/11/2010	6.78	.	4.50 - 11.00		
S266	308	JAIVAC-1	Dosage C	2	% Basophils	%	Day 28	09/12/2010	0	.	0.0 - 1.0		

Subject No.	Randomisation Number	Study Group	Dosage	Dose	Haematology Parameters	Unit	Day	Date of sample Collection	Results	Out of Range	Reference Range	Flag	Clinical Significance **
S266	308	JAIVAC-1	Dosage C	2	% Eosinophils	%	Day 28	09/12/2010	2	.	1.0 - 6.0		
S266	308	JAIVAC-1	Dosage C	2	% Lymphocytes	%	Day 28	09/12/2010	33	.	20.0 - 45.0		
S266	308	JAIVAC-1	Dosage C	2	% Monocytes	%	Day 28	09/12/2010	8	.	2.0 - 10.0		
S266	308	JAIVAC-1	Dosage C	2	% Neutrophils	%	Day 28	09/12/2010	57	.	40.0 - 75.0		
S266	308	JAIVAC-1	Dosage C	2	Haematocrit	%	Day 28	09/12/2010	47.4	.	40.0 - 54.0		
S266	308	JAIVAC-1	Dosage C	2	Haemoglobin	gm / dl	Day 28	09/12/2010	15.7	.	13.5 - 18.0		
S266	308	JAIVAC-1	Dosage C	2	MCH	pg	Day 28	09/12/2010	29.7	.	27.0 - 31.0		
S266	308	JAIVAC-1	Dosage C	2	MCHC	gm %	Day 28	09/12/2010	33.2	.	32.0 - 36.0		
S266	308	JAIVAC-1	Dosage C	2	MCV	fL	Day 28	09/12/2010	89.5	.	80.0- 96.0		
S266	308	JAIVAC-1	Dosage C	2	Platelet Count	10E3/ul	Day 28	09/12/2010	429	Yes	150 - 400	H	NCS
S266	308	JAIVAC-1	Dosage C	2	RBC count	10E6/ul	Day 28	09/12/2010	5.3	.	4.60 - 6.20		
S266	308	JAIVAC-1	Dosage C	2	TLC	10E3/ul	Day 28	09/12/2010	6.55	.	4.50 - 11.00		
S266	308	JAIVAC-1	Dosage C	2	% Basophils	%	Day 56	06/01/2011	0.8	.	0.0 - 1.0		
S266	308	JAIVAC-1	Dosage C	2	% Eosinophils	%	Day 56	06/01/2011	0.5	Yes	1.0 - 6.0	L	NCS
S266	308	JAIVAC-1	Dosage C	2	% Lymphocytes	%	Day 56	06/01/2011	35.9	.	20.0 - 45.0		
S266	308	JAIVAC-1	Dosage C	2	% Monocytes	%	Day 56	06/01/2011	8.4	.	2.0 - 10.0		
S266	308	JAIVAC-1	Dosage C	2	% Neutrophils	%	Day 56	06/01/2011	54.6	.	40.0 - 75.0		
S266	308	JAIVAC-1	Dosage C	2	Haematocrit	%	Day 56	06/01/2011	49.5	.	40.0 - 54.0		
S266	308	JAIVAC-1	Dosage C	2	Haemoglobin	gm / dl	Day 56	06/01/2011	16.4	.	13.5 - 18.0		
S266	308	JAIVAC-1	Dosage C	2	MCH	pg	Day 56	06/01/2011	29.8	.	27.0 - 31.0		
S266	308	JAIVAC-1	Dosage C	2	MCHC	gm %	Day 56	06/01/2011	33.1	.	32.0 - 36.0		
S266	308	JAIVAC-1	Dosage C	2	MCV	fL	Day 56	06/01/2011	89.8	.	80.0- 96.0		
S266	308	JAIVAC-1	Dosage C	2	Platelet Count	10E3/ul	Day 56	06/01/2011	374	.	150 - 400		
S266	308	JAIVAC-1	Dosage C	2	RBC count	10E6/ul	Day 56	06/01/2011	5.51	.	4.60 - 6.20		
S266	308	JAIVAC-1	Dosage C	2	TLC	10E3/ul	Day 56	06/01/2011	6.45	.	4.50 - 11.00		
S266	308	JAIVAC-1	Dosage C	3	% Basophils	%	Day 180	10/05/2011	0.9	.	0.0 - 1.0		
S266	308	JAIVAC-1	Dosage C	3	% Eosinophils	%	Day 180	10/05/2011	1	.	1.0 - 6.0		
S266	308	JAIVAC-1	Dosage C	3	% Lymphocytes	%	Day 180	10/05/2011	32.5	.	20.0 - 45.0		
S266	308	JAIVAC-1	Dosage C	3	% Monocytes	%	Day 180	10/05/2011	7.2	.	2.0 - 10.0		

Subject No.	Randomisation Number	Study Group	Dosage	Dose	Haematology Parameters	Unit	Day	Date of sample Collection	Results	Out of Range	Reference Range	Flag	Clinical Significance **
S266	308	JAIVAC-1	Dosage C	3	% Neutrophils	%	Day 180	10/05/2011	58.4	.	40.0 - 75.0		
S266	308	JAIVAC-1	Dosage C	3	Haematocrit	%	Day 180	10/05/2011	45.7	.	40.0 - 54.0		
S266	308	JAIVAC-1	Dosage C	3	Haemoglobin	gm / dl	Day 180	10/05/2011	15.1	.	13.5 - 18.0		
S266	308	JAIVAC-1	Dosage C	3	MCH	pg	Day 180	10/05/2011	30.3	.	27.0 - 31.0		
S266	308	JAIVAC-1	Dosage C	3	MCHC	gm %	Day 180	10/05/2011	33	.	32.0 - 36.0		
S266	308	JAIVAC-1	Dosage C	3	MCV	fL	Day 180	10/05/2011	91.7	.	80.0- 96.0		
S266	308	JAIVAC-1	Dosage C	3	Platelet Count	10E3/ul	Day 180	10/05/2011	412	Yes	150 - 400	H	NCS
S266	308	JAIVAC-1	Dosage C	3	RBC count	10E6/ul	Day 180	10/05/2011	4.99	.	4.60 - 6.20		
S266	308	JAIVAC-1	Dosage C	3	TLC	10E3/ul	Day 180	10/05/2011	6.4	.	4.50 - 11.00		
S266	308	JAIVAC-1	Dosage C	3	% Basophils	%	Day 208	07/06/2011	1.7	Yes	0.0 - 1.0	H	NCS
S266	308	JAIVAC-1	Dosage C	3	% Eosinophils	%	Day 208	07/06/2011	1.2	.	1.0 - 6.0		
S266	308	JAIVAC-1	Dosage C	3	% Lymphocytes	%	Day 208	07/06/2011	21.7	.	20.0 - 45.0		
S266	308	JAIVAC-1	Dosage C	3	% Monocytes	%	Day 208	07/06/2011	8	.	2.0 - 10.0		
S266	308	JAIVAC-1	Dosage C	3	% Neutrophils	%	Day 208	07/06/2011	67.5	.	40.0 - 75.0		
S266	308	JAIVAC-1	Dosage C	3	Haematocrit	%	Day 208	07/06/2011	49.4	.	40.0 - 54.0		
S266	308	JAIVAC-1	Dosage C	3	Haemoglobin	gm / dl	Day 208	07/06/2011	16.4	.	13.5 - 18.0		
S266	308	JAIVAC-1	Dosage C	3	MCH	pg	Day 208	07/06/2011	30.6	.	27.0 - 31.0		
S266	308	JAIVAC-1	Dosage C	3	MCHC	gm %	Day 208	07/06/2011	33.3	.	32.0 - 36.0		
S266	308	JAIVAC-1	Dosage C	3	MCV	fL	Day 208	07/06/2011	91.9	.	80.0- 96.0		
S266	308	JAIVAC-1	Dosage C	3	Platelet Count	10E3/ul	Day 208	07/06/2011	409	Yes	150 - 400	H	NCS
S266	308	JAIVAC-1	Dosage C	3	RBC count	10E6/ul	Day 208	07/06/2011	5.38	.	4.60 - 6.20		
S266	308	JAIVAC-1	Dosage C	3	TLC	10E3/ul	Day 208	07/06/2011	10.1	.	4.50 - 11.00		
S266	308	JAIVAC-1	Dosage C	3	% Basophils	%	Day 365	09/11/2011	2.3	Yes	0.0 - 1.0	H	NCS
S266	308	JAIVAC-1	Dosage C	3	% Eosinophils	%	Day 365	09/11/2011	1.3	.	1.0 - 6.0		
S266	308	JAIVAC-1	Dosage C	3	% Lymphocytes	%	Day 365	09/11/2011	33.9	.	20.0 - 45.0		
S266	308	JAIVAC-1	Dosage C	3	% Monocytes	%	Day 365	09/11/2011	8	.	2.0 - 10.0		
S266	308	JAIVAC-1	Dosage C	3	% Neutrophils	%	Day 365	09/11/2011	54.5	.	40.0 - 75.0		
S266	308	JAIVAC-1	Dosage C	3	Haematocrit	%	Day 365	09/11/2011	49.5	.	40.0 - 54.0		
S266	308	JAIVAC-1	Dosage C	3	Haemoglobin	gm / dl	Day 365	09/11/2011	16	.	13.5 - 18.0		

Subject No.	Randomisation Number	Study Group	Dosage	Dose	Haematology Parameters	Unit	Day	Date of sample Collection	Results	Out of Range	Reference Range	Flag	Clinical Significance **
S266	308	JAIVAC-1	Dosage C	3	MCH	pg	Day 365	09/11/2011	29.7	.	27.0 - 31.0		
S266	308	JAIVAC-1	Dosage C	3	MCHC	gm %	Day 365	09/11/2011	32.4	.	32.0 - 36.0		
S266	308	JAIVAC-1	Dosage C	3	MCV	fL	Day 365	09/11/2011	91.9	.	80.0- 96.0		
S266	308	JAIVAC-1	Dosage C	3	Platelet Count	10E3/ul	Day 365	09/11/2011	399	.	150 - 400		
S266	308	JAIVAC-1	Dosage C	3	RBC count	10E6/ul	Day 365	09/11/2011	5.38	.	4.60 - 6.20		
S266	308	JAIVAC-1	Dosage C	3	TLC	10E3/ul	Day 365	09/11/2011	7.16	.	4.50 - 11.00		
S267	309	Hepatitis B	Hepatitis B		% Basophils	%	Day -14 to Day -1	02/11/2010	1.5	Yes	0.0 - 1.0	H	NCS
S267	309	Hepatitis B	Hepatitis B		% Eosinophils	%	Day -14 to Day -1	02/11/2010	8.1	.	1.0 - 6.0	H	
S267	309	Hepatitis B	Hepatitis B		% Lymphocytes	%	Day -14 to Day -1	02/11/2010	26.9	.	20.0 - 45.0		
S267	309	Hepatitis B	Hepatitis B		% Monocytes	%	Day -14 to Day -1	02/11/2010	4.6	.	2.0 - 10.0		
S267	309	Hepatitis B	Hepatitis B		% Neutrophils	%	Day -14 to Day -1	02/11/2010	58.8	.	40.0 - 75.0		
S267	309	Hepatitis B	Hepatitis B		Haematocrit	%	Day -14 to Day -1	02/11/2010	48.6	.	40.0 - 54.0		
S267	309	Hepatitis B	Hepatitis B		Haemoglobin	gm / dl	Day -14 to Day -1	02/11/2010	16.7	.	13.5 - 18.0		
S267	309	Hepatitis B	Hepatitis B		MCH	pg	Day -14 to Day -1	02/11/2010	31.1	Yes	27.0 - 31.0	H	NCS
S267	309	Hepatitis B	Hepatitis B		MCHC	gm %	Day -14 to Day -1	02/11/2010	34.3	.	32.0 - 36.0		
S267	309	Hepatitis B	Hepatitis B		MCV	fL	Day -14 to Day -1	02/11/2010	90.6	.	80.0- 96.0		
S267	309	Hepatitis B	Hepatitis B		Platelet Count	10E3/ul	Day -14 to Day -1	02/11/2010	263	.	150 - 400		
S267	309	Hepatitis B	Hepatitis B		RBC count	10E6/ul	Day -14 to Day -1	02/11/2010	5.36	.	4.60 - 6.20		
S267	309	Hepatitis B	Hepatitis B		TLC	10E3/ul	Day -14 to Day -1	02/11/2010	7.98	.	4.50 - 11.00		
S267	309	Hepatitis B	Hepatitis B	2	% Basophils	%	Day 28	09/12/2010	0	.	0.0 - 1.0		
S267	309	Hepatitis B	Hepatitis B	2	% Eosinophils	%	Day 28	09/12/2010	8	Yes	1.0 - 6.0	H	NCS
S267	309	Hepatitis B	Hepatitis B	2	% Lymphocytes	%	Day 28	09/12/2010	21	.	20.0 - 45.0		
S267	309	Hepatitis B	Hepatitis B	2	% Monocytes	%	Day 28	09/12/2010	4	.	2.0 - 10.0		
S267	309	Hepatitis B	Hepatitis B	2	% Neutrophils	%	Day 28	09/12/2010	67	.	40.0 - 75.0		
S267	309	Hepatitis B	Hepatitis B	2	Haematocrit	%	Day 28	09/12/2010	46.1	.	40.0 - 54.0		
S267	309	Hepatitis B	Hepatitis B	2	Haemoglobin	gm / dl	Day 28	09/12/2010	15.6	.	13.5 - 18.0		
S267	309	Hepatitis B	Hepatitis B	2	MCH	pg	Day 28	09/12/2010	30.2	.	27.0 - 31.0		
S267	309	Hepatitis B	Hepatitis B	2	MCHC	gm %	Day 28	09/12/2010	33.9	.	32.0 - 36.0		
S267	309	Hepatitis B	Hepatitis B	2	MCV	fL	Day 28	09/12/2010	89.3	.	80.0- 96.0		

Subject No.	Randomisation Number	Study Group	Dosage	Dose	Haematology Parameters	Unit	Day	Date of sample Collection	Results	Out of Range	Reference Range	Flag	Clinical Significance **
S267	309	Hepatitis B	Hepatitis B	2	Platelet Count	10E3/ul	Day 28	09/12/2010	261	.	150 - 400		
S267	309	Hepatitis B	Hepatitis B	2	RBC count	10E6/ul	Day 28	09/12/2010	5.17	.	4.60 - 6.20		
S267	309	Hepatitis B	Hepatitis B	2	TLC	10E3/ul	Day 28	09/12/2010	6.47	.	4.50 - 11.00		
S267	309	Hepatitis B	Hepatitis B	2	% Basophils	%	Day 56	06/01/2011	0.8	.	0.0 - 1.0		
S267	309	Hepatitis B	Hepatitis B	2	% Eosinophils	%	Day 56	06/01/2011	3.2	.	1.0 - 6.0		
S267	309	Hepatitis B	Hepatitis B	2	% Lymphocytes	%	Day 56	06/01/2011	26	.	20.0 - 45.0		
S267	309	Hepatitis B	Hepatitis B	2	% Monocytes	%	Day 56	06/01/2011	3.7	.	2.0 - 10.0		
S267	309	Hepatitis B	Hepatitis B	2	% Neutrophils	%	Day 56	06/01/2011	66.4	.	40.0 - 75.0		
S267	309	Hepatitis B	Hepatitis B	2	Haematocrit	%	Day 56	06/01/2011	44.3	.	40.0 - 54.0		
S267	309	Hepatitis B	Hepatitis B	2	Haemoglobin	gm / dl	Day 56	06/01/2011	15	.	13.5 - 18.0		
S267	309	Hepatitis B	Hepatitis B	2	MCH	pg	Day 56	06/01/2011	30	.	27.0 - 31.0		
S267	309	Hepatitis B	Hepatitis B	2	MCHC	gm %	Day 56	06/01/2011	34	.	32.0 - 36.0		
S267	309	Hepatitis B	Hepatitis B	2	MCV	fL	Day 56	06/01/2011	88.3	.	80.0- 96.0		
S267	309	Hepatitis B	Hepatitis B	2	Platelet Count	10E3/ul	Day 56	06/01/2011	278	.	150 - 400		
S267	309	Hepatitis B	Hepatitis B	2	RBC count	10E6/ul	Day 56	06/01/2011	5.01	.	4.60 - 6.20		
S267	309	Hepatitis B	Hepatitis B	2	TLC	10E3/ul	Day 56	06/01/2011	9.08	.	4.50 - 11.00		
S267	309	Hepatitis B	Hepatitis B	3	% Basophils	%	Day 180	10/05/2011	1.3	Yes	0.0 - 1.0	H	NCS
S267	309	Hepatitis B	Hepatitis B	3	% Eosinophils	%	Day 180	10/05/2011	7.3	Yes	1.0 - 6.0	H	NCS
S267	309	Hepatitis B	Hepatitis B	3	% Lymphocytes	%	Day 180	10/05/2011	32.2	.	20.0 - 45.0		
S267	309	Hepatitis B	Hepatitis B	3	% Monocytes	%	Day 180	10/05/2011	4.5	.	2.0 - 10.0		
S267	309	Hepatitis B	Hepatitis B	3	% Neutrophils	%	Day 180	10/05/2011	54.7	.	40.0 - 75.0		
S267	309	Hepatitis B	Hepatitis B	3	Haematocrit	%	Day 180	10/05/2011	41.4	.	40.0 - 54.0		
S267	309	Hepatitis B	Hepatitis B	3	Haemoglobin	gm / dl	Day 180	10/05/2011	14.2	.	13.5 - 18.0		
S267	309	Hepatitis B	Hepatitis B	3	MCH	pg	Day 180	10/05/2011	31.8	Yes	27.0 - 31.0	H	NCS
S267	309	Hepatitis B	Hepatitis B	3	MCHC	gm %	Day 180	10/05/2011	34.3	.	32.0 - 36.0		
S267	309	Hepatitis B	Hepatitis B	3	MCV	fL	Day 180	10/05/2011	92.5	.	80.0- 96.0		
S267	309	Hepatitis B	Hepatitis B	3	Platelet Count	10E3/ul	Day 180	10/05/2011	231	.	150 - 400		
S267	309	Hepatitis B	Hepatitis B	3	RBC count	10E6/ul	Day 180	10/05/2011	4.48	Yes	4.60 - 6.20	L	NCS
S267	309	Hepatitis B	Hepatitis B	3	TLC	10E3/ul	Day 180	10/05/2011	6.54	.	4.50 - 11.00		

Subject No.	Randomisation Number	Study Group	Dosage	Dose	Haematology Parameters	Unit	Day	Date of sample Collection	Results	Out of Range	Reference Range	Flag	Clinical Significance **
S267	309	Hepatitis B	Hepatitis B	3	% Basophils	%	Day 208	07/06/2011	0	.	0.0 - 1.0		
S267	309	Hepatitis B	Hepatitis B	3	% Eosinophils	%	Day 208	07/06/2011	6	.	1.0 - 6.0		
S267	309	Hepatitis B	Hepatitis B	3	% Lymphocytes	%	Day 208	07/06/2011	24	.	20.0 - 45.0		
S267	309	Hepatitis B	Hepatitis B	3	% Monocytes	%	Day 208	07/06/2011	3	.	2.0 - 10.0		
S267	309	Hepatitis B	Hepatitis B	3	% Neutrophils	%	Day 208	07/06/2011	67	.	40.0 - 75.0		
S267	309	Hepatitis B	Hepatitis B	3	Haematocrit	%	Day 208	07/06/2011	43.9	.	40.0 - 54.0		
S267	309	Hepatitis B	Hepatitis B	3	Haemoglobin	gm / dl	Day 208	07/06/2011	15	.	13.5 - 18.0		
S267	309	Hepatitis B	Hepatitis B	3	MCH	pg	Day 208	07/06/2011	31.9	Yes	27.0 - 31.0	H	NCS
S267	309	Hepatitis B	Hepatitis B	3	MCHC	gm %	Day 208	07/06/2011	34.1	.	32.0 - 36.0		
S267	309	Hepatitis B	Hepatitis B	3	MCV	fL	Day 208	07/06/2011	93.6	.	80.0- 96.0		
S267	309	Hepatitis B	Hepatitis B	3	Platelet Count	10E3/ul	Day 208	07/06/2011	259	.	150 - 400		
S267	309	Hepatitis B	Hepatitis B	3	RBC count	10E6/ul	Day 208	07/06/2011	4.69	.	4.60 - 6.20		
S267	309	Hepatitis B	Hepatitis B	3	TLC	10E3/ul	Day 208	07/06/2011	7.52	.	4.50 - 11.00		
S267	309	Hepatitis B	Hepatitis B	3	% Basophils	%	Day 365	09/11/2011	0	.	0.0 - 1.0		
S267	309	Hepatitis B	Hepatitis B	3	% Eosinophils	%	Day 365	09/11/2011	6	.	1.0 - 6.0		
S267	309	Hepatitis B	Hepatitis B	3	% Lymphocytes	%	Day 365	09/11/2011	30	.	20.0 - 45.0		
S267	309	Hepatitis B	Hepatitis B	3	% Monocytes	%	Day 365	09/11/2011	5	.	2.0 - 10.0		
S267	309	Hepatitis B	Hepatitis B	3	% Neutrophils	%	Day 365	09/11/2011	59	.	40.0 - 75.0		
S267	309	Hepatitis B	Hepatitis B	3	Haematocrit	%	Day 365	09/11/2011	40.8	.	40.0 - 54.0		
S267	309	Hepatitis B	Hepatitis B	3	Haemoglobin	gm / dl	Day 365	09/11/2011	13.8	.	13.5 - 18.0		
S267	309	Hepatitis B	Hepatitis B	3	MCH	pg	Day 365	09/11/2011	30.5	.	27.0 - 31.0		
S267	309	Hepatitis B	Hepatitis B	3	MCHC	gm %	Day 365	09/11/2011	33.7	.	32.0 - 36.0		
S267	309	Hepatitis B	Hepatitis B	3	MCV	fL	Day 365	09/11/2011	90.5	.	80.0- 96.0		
S267	309	Hepatitis B	Hepatitis B	3	Platelet Count	10E3/ul	Day 365	09/11/2011	217	.	150 - 400		
S267	309	Hepatitis B	Hepatitis B	3	RBC count	10E6/ul	Day 365	09/11/2011	4.51	Yes	4.60 - 6.20	L	NCS
S267	309	Hepatitis B	Hepatitis B	3	TLC	10E3/ul	Day 365	09/11/2011	3.44	Yes	4.50 - 11.00	L	CS
S275	302	Hepatitis B	Hepatitis B		% Basophils	%	Day -14 to Day -1	02/11/2010	0.8	.	0.0 - 1.0		
S275	302	Hepatitis B	Hepatitis B		% Eosinophils	%	Day -14 to Day -1	02/11/2010	6.8	.	1.0 - 6.0	H	
S275	302	Hepatitis B	Hepatitis B		% Lymphocytes	%	Day -14 to Day -1	02/11/2010	35.2	.	20.0 - 45.0		

Subject No.	Randomisation Number	Study Group	Dosage	Dose	Haematology Parameters	Unit	Day	Date of sample Collection	Results	Out of Range	Reference Range	Flag	Clinical Significance **
S275	302	Hepatitis B	Hepatitis B		% Monocytes	%	Day -14 to Day -1	02/11/2010	4.8	.	2.0 - 10.0		
S275	302	Hepatitis B	Hepatitis B		% Neutrophils	%	Day -14 to Day -1	02/11/2010	52.4	.	40.0 - 75.0		
S275	302	Hepatitis B	Hepatitis B		Haematocrit	%	Day -14 to Day -1	02/11/2010	47.2	.	40.0 - 54.0		
S275	302	Hepatitis B	Hepatitis B		Haemoglobin	gm / dl	Day -14 to Day -1	02/11/2010	15.5	.	13.5 - 18.0		
S275	302	Hepatitis B	Hepatitis B		MCH	pg	Day -14 to Day -1	02/11/2010	28.3	.	27.0 - 31.0		
S275	302	Hepatitis B	Hepatitis B		MCHC	gm %	Day -14 to Day -1	02/11/2010	32.8	.	32.0 - 36.0		
S275	302	Hepatitis B	Hepatitis B		MCV	fL	Day -14 to Day -1	02/11/2010	86.4	.	80.0- 96.0		
S275	302	Hepatitis B	Hepatitis B		Platelet Count	10E3/ul	Day -14 to Day -1	02/11/2010	203	.	150 - 400		
S275	302	Hepatitis B	Hepatitis B		RBC count	10E6/ul	Day -14 to Day -1	02/11/2010	5.46	.	4.60 - 6.20		
S275	302	Hepatitis B	Hepatitis B		TLC	10E3/ul	Day -14 to Day -1	02/11/2010	6.41	.	4.50 - 11.00		
S275	302	Hepatitis B	Hepatitis B	2	% Basophils	%	Day 28	08/12/2010	1	.	0.0 - 1.0		
S275	302	Hepatitis B	Hepatitis B	2	% Eosinophils	%	Day 28	08/12/2010	5	.	1.0 - 6.0		
S275	302	Hepatitis B	Hepatitis B	2	% Lymphocytes	%	Day 28	08/12/2010	21.2	.	20.0 - 45.0		
S275	302	Hepatitis B	Hepatitis B	2	% Monocytes	%	Day 28	08/12/2010	3.5	.	2.0 - 10.0		
S275	302	Hepatitis B	Hepatitis B	2	% Neutrophils	%	Day 28	08/12/2010	69.3	.	40.0 - 75.0		
S275	302	Hepatitis B	Hepatitis B	2	Haematocrit	%	Day 28	08/12/2010	49	.	40.0 - 54.0		
S275	302	Hepatitis B	Hepatitis B	2	Haemoglobin	gm / dl	Day 28	08/12/2010	16.5	.	13.5 - 18.0		
S275	302	Hepatitis B	Hepatitis B	2	MCH	pg	Day 28	08/12/2010	29.3	.	27.0 - 31.0		
S275	302	Hepatitis B	Hepatitis B	2	MCHC	gm %	Day 28	08/12/2010	33.7	.	32.0 - 36.0		
S275	302	Hepatitis B	Hepatitis B	2	MCV	fL	Day 28	08/12/2010	86.8	.	80.0- 96.0		
S275	302	Hepatitis B	Hepatitis B	2	Platelet Count	10E3/ul	Day 28	08/12/2010	198	.	150 - 400		
S275	302	Hepatitis B	Hepatitis B	2	RBC count	10E6/ul	Day 28	08/12/2010	5.65	.	4.60 - 6.20		
S275	302	Hepatitis B	Hepatitis B	2	TLC	10E3/ul	Day 28	08/12/2010	6.77	.	4.50 - 11.00		
S275	302	Hepatitis B	Hepatitis B	2	% Basophils	%	Day 56	05/01/2011	0.5	.	0.0 - 1.0		
S275	302	Hepatitis B	Hepatitis B	2	% Eosinophils	%	Day 56	05/01/2011	8.7	Yes	1.0 - 6.0	H	NCS
S275	302	Hepatitis B	Hepatitis B	2	% Lymphocytes	%	Day 56	05/01/2011	32.2	.	20.0 - 45.0		
S275	302	Hepatitis B	Hepatitis B	2	% Monocytes	%	Day 56	05/01/2011	4.8	.	2.0 - 10.0		
S275	302	Hepatitis B	Hepatitis B	2	% Neutrophils	%	Day 56	05/01/2011	53.7	.	40.0 - 75.0		
S275	302	Hepatitis B	Hepatitis B	2	Haematocrit	%	Day 56	05/01/2011	42.9	.	40.0 - 54.0		

Subject No.	Randomisation Number	Study Group	Dosage	Dose	Haematology Parameters	Unit	Day	Date of sample Collection	Results	Out of Range	Reference Range	Flag	Clinical Significance **
S275	302	Hepatitis B	Hepatitis B	2	Haemoglobin	gm / dl	Day 56	05/01/2011	14.8	.	13.5 - 18.0		
S275	302	Hepatitis B	Hepatitis B	2	MCH	pg	Day 56	05/01/2011	29.5	.	27.0 - 31.0		
S275	302	Hepatitis B	Hepatitis B	2	MCHC	gm %	Day 56	05/01/2011	34.4	.	32.0 - 36.0		
S275	302	Hepatitis B	Hepatitis B	2	MCV	fL	Day 56	05/01/2011	85.9	.	80.0- 96.0		
S275	302	Hepatitis B	Hepatitis B	2	Platelet Count	10E3/ul	Day 56	05/01/2011	204	.	150 - 400		
S275	302	Hepatitis B	Hepatitis B	2	RBC count	10E6/ul	Day 56	05/01/2011	5	.	4.60 - 6.20		
S275	302	Hepatitis B	Hepatitis B	2	TLC	10E3/ul	Day 56	05/01/2011	4.82	.	4.50 - 11.00		
S275	302	Hepatitis B	Hepatitis B	3	% Basophils	%	Day 180	09/05/2011	1.2	Yes	0.0 - 1.0	H	NCS
S275	302	Hepatitis B	Hepatitis B	3	% Eosinophils	%	Day 180	09/05/2011	5.5	.	1.0 - 6.0		
S275	302	Hepatitis B	Hepatitis B	3	% Lymphocytes	%	Day 180	09/05/2011	20.2	.	20.0 - 45.0		
S275	302	Hepatitis B	Hepatitis B	3	% Monocytes	%	Day 180	09/05/2011	5.4	.	2.0 - 10.0		
S275	302	Hepatitis B	Hepatitis B	3	% Neutrophils	%	Day 180	09/05/2011	67.7	.	40.0 - 75.0		
S275	302	Hepatitis B	Hepatitis B	3	Haematocrit	%	Day 180	09/05/2011	46.1	.	40.0 - 54.0		
S275	302	Hepatitis B	Hepatitis B	3	Haemoglobin	gm / dl	Day 180	09/05/2011	15.9	.	13.5 - 18.0		
S275	302	Hepatitis B	Hepatitis B	3	MCH	pg	Day 180	09/05/2011	30.6	.	27.0 - 31.0		
S275	302	Hepatitis B	Hepatitis B	3	MCHC	gm %	Day 180	09/05/2011	34.6	.	32.0 - 36.0		
S275	302	Hepatitis B	Hepatitis B	3	MCV	fL	Day 180	09/05/2011	88.5	.	80.0- 96.0		
S275	302	Hepatitis B	Hepatitis B	3	Platelet Count	10E3/ul	Day 180	09/05/2011	198	.	150 - 400		
S275	302	Hepatitis B	Hepatitis B	3	RBC count	10E6/ul	Day 180	09/05/2011	5.21	.	4.60 - 6.20		
S275	302	Hepatitis B	Hepatitis B	3	TLC	10E3/ul	Day 180	09/05/2011	5.84	.	4.50 - 11.00		
S275	302	Hepatitis B	Hepatitis B	3	% Basophils	%	Day 208	06/06/2011	0.9	.	0.0 - 1.0		
S275	302	Hepatitis B	Hepatitis B	3	% Eosinophils	%	Day 208	06/06/2011	3.3	.	1.0 - 6.0		
S275	302	Hepatitis B	Hepatitis B	3	% Lymphocytes	%	Day 208	06/06/2011	15.5	Yes	20.0 - 45.0	L	NCS
S275	302	Hepatitis B	Hepatitis B	3	% Monocytes	%	Day 208	06/06/2011	3.1	.	2.0 - 10.0		
S275	302	Hepatitis B	Hepatitis B	3	% Neutrophils	%	Day 208	06/06/2011	77.2	Yes	40.0 - 75.0	H	NCS
S275	302	Hepatitis B	Hepatitis B	3	Haematocrit	%	Day 208	06/06/2011	45.5	.	40.0 - 54.0		
S275	302	Hepatitis B	Hepatitis B	3	Haemoglobin	gm / dl	Day 208	06/06/2011	15.9	.	13.5 - 18.0		
S275	302	Hepatitis B	Hepatitis B	3	MCH	pg	Day 208	06/06/2011	31.1	Yes	27.0 - 31.0	H	NCS
S275	302	Hepatitis B	Hepatitis B	3	MCHC	gm %	Day 208	06/06/2011	35	.	32.0 - 36.0		

Subject No.	Randomisation Number	Study Group	Dosage	Dose	Haematology Parameters	Unit	Day	Date of sample Collection	Results	Out of Range	Reference Range	Flag	Clinical Significance **
S275	302	Hepatitis B	Hepatitis B	3	MCV	fL	Day 208	06/06/2011	88.8	.	80.0- 96.0		
S275	302	Hepatitis B	Hepatitis B	3	Platelet Count	10E3/ul	Day 208	06/06/2011	237	.	150 - 400		
S275	302	Hepatitis B	Hepatitis B	3	RBC count	10E6/ul	Day 208	06/06/2011	5.12	.	4.60 - 6.20		
S275	302	Hepatitis B	Hepatitis B	3	TLC	10E3/ul	Day 208	06/06/2011	9.23	.	4.50 - 11.00		
S275	302	Hepatitis B	Hepatitis B	3	% Basophils	%	Day 365	09/11/2011	1.3	Yes	0.0 - 1.0	H	NCS
S275	302	Hepatitis B	Hepatitis B	3	% Eosinophils	%	Day 365	09/11/2011	5.5	.	1.0 - 6.0		
S275	302	Hepatitis B	Hepatitis B	3	% Lymphocytes	%	Day 365	09/11/2011	25.3	.	20.0 - 45.0		
S275	302	Hepatitis B	Hepatitis B	3	% Monocytes	%	Day 365	09/11/2011	4.1	.	2.0 - 10.0		
S275	302	Hepatitis B	Hepatitis B	3	% Neutrophils	%	Day 365	09/11/2011	64	.	40.0 - 75.0		
S275	302	Hepatitis B	Hepatitis B	3	Haematocrit	%	Day 365	09/11/2011	45.5	.	40.0 - 54.0		
S275	302	Hepatitis B	Hepatitis B	3	Haemoglobin	gm / dl	Day 365	09/11/2011	15.5	.	13.5 - 18.0		
S275	302	Hepatitis B	Hepatitis B	3	MCH	pg	Day 365	09/11/2011	29.8	.	27.0 - 31.0		
S275	302	Hepatitis B	Hepatitis B	3	MCHC	gm %	Day 365	09/11/2011	34	.	32.0 - 36.0		
S275	302	Hepatitis B	Hepatitis B	3	MCV	fL	Day 365	09/11/2011	87.6	.	80.0- 96.0		
S275	302	Hepatitis B	Hepatitis B	3	Platelet Count	10E3/ul	Day 365	09/11/2011	219	.	150 - 400		
S275	302	Hepatitis B	Hepatitis B	3	RBC count	10E6/ul	Day 365	09/11/2011	5.19	.	4.60 - 6.20		
S275	302	Hepatitis B	Hepatitis B	3	TLC	10E3/ul	Day 365	09/11/2011	6.72	.	4.50 - 11.00		
S278	315	JAIVAC-1	Dosage C		% Basophils	%	Day -14 to Day -1	08/11/2010	1.3	Yes	0.0 - 1.0	H	NCS
S278	315	JAIVAC-1	Dosage C		% Eosinophils	%	Day -14 to Day -1	08/11/2010	8.5	.	1.0 - 6.0	H	
S278	315	JAIVAC-1	Dosage C		% Lymphocytes	%	Day -14 to Day -1	08/11/2010	32	.	20.0 - 45.0		
S278	315	JAIVAC-1	Dosage C		% Monocytes	%	Day -14 to Day -1	08/11/2010	3.6	.	2.0 - 10.0		
S278	315	JAIVAC-1	Dosage C		% Neutrophils	%	Day -14 to Day -1	08/11/2010	54.6	.	40.0 - 75.0		
S278	315	JAIVAC-1	Dosage C		Haematocrit	%	Day -14 to Day -1	08/11/2010	47	.	40.0 - 54.0		
S278	315	JAIVAC-1	Dosage C		Haemoglobin	gm / dl	Day -14 to Day -1	08/11/2010	16.6	.	13.5 - 18.0		
S278	315	JAIVAC-1	Dosage C		MCH	pg	Day -14 to Day -1	08/11/2010	32.1	Yes	27.0 - 31.0	H	NCS
S278	315	JAIVAC-1	Dosage C		MCHC	gm %	Day -14 to Day -1	08/11/2010	35.3	.	32.0 - 36.0		
S278	315	JAIVAC-1	Dosage C		MCV	fL	Day -14 to Day -1	08/11/2010	90.8	.	80.0- 96.0		
S278	315	JAIVAC-1	Dosage C		Platelet Count	10E3/ul	Day -14 to Day -1	08/11/2010	272	.	150 - 400		
S278	315	JAIVAC-1	Dosage C		RBC count	10E6/ul	Day -14 to Day -1	08/11/2010	5.17	.	4.60 - 6.20		

Subject No.	Randomisation Number	Study Group	Dosage	Dose	Haematology Parameters	Unit	Day	Date of sample Collection	Results	Out of Range	Reference Range	Flag	Clinical Significance **
S278	315	JAIVAC-1	Dosage C		TLC	10E3/ul	Day -14 to Day -1	08/11/2010	8.04	.	4.50 - 11.00		
S278	315	JAIVAC-1	Dosage C	2	% Basophils	%	Day 28	10/12/2010	0.7	.	0.0 - 1.0		
S278	315	JAIVAC-1	Dosage C	2	% Eosinophils	%	Day 28	10/12/2010	5.6	.	1.0 - 6.0		
S278	315	JAIVAC-1	Dosage C	2	% Lymphocytes	%	Day 28	10/12/2010	22.2	.	20.0 - 45.0		
S278	315	JAIVAC-1	Dosage C	2	% Monocytes	%	Day 28	10/12/2010	2.9	.	2.0 - 10.0		
S278	315	JAIVAC-1	Dosage C	2	% Neutrophils	%	Day 28	10/12/2010	68.6	.	40.0 - 75.0		
S278	315	JAIVAC-1	Dosage C	2	Haematocrit	%	Day 28	10/12/2010	47	.	40.0 - 54.0		
S278	315	JAIVAC-1	Dosage C	2	Haemoglobin	gm / dl	Day 28	10/12/2010	16.1	.	13.5 - 18.0		
S278	315	JAIVAC-1	Dosage C	2	MCH	pg	Day 28	10/12/2010	31.2	Yes	27.0 - 31.0	H	NCS
S278	315	JAIVAC-1	Dosage C	2	MCHC	gm %	Day 28	10/12/2010	34.3	.	32.0 - 36.0		
S278	315	JAIVAC-1	Dosage C	2	MCV	fL	Day 28	10/12/2010	91	.	80.0- 96.0		
S278	315	JAIVAC-1	Dosage C	2	Platelet Count	10E3/ul	Day 28	10/12/2010	277	.	150 - 400		
S278	315	JAIVAC-1	Dosage C	2	RBC count	10E6/ul	Day 28	10/12/2010	5.17	.	4.60 - 6.20		
S278	315	JAIVAC-1	Dosage C	2	TLC	10E3/ul	Day 28	10/12/2010	10.51	.	4.50 - 11.00		
S278	315	JAIVAC-1	Dosage C	2	% Basophils	%	Day 56	07/01/2011	0.7	.	0.0 - 1.0		
S278	315	JAIVAC-1	Dosage C	2	% Eosinophils	%	Day 56	07/01/2011	6.4	Yes	1.0 - 6.0	H	NCS
S278	315	JAIVAC-1	Dosage C	2	% Lymphocytes	%	Day 56	07/01/2011	31.3	.	20.0 - 45.0		
S278	315	JAIVAC-1	Dosage C	2	% Monocytes	%	Day 56	07/01/2011	3.9	.	2.0 - 10.0		
S278	315	JAIVAC-1	Dosage C	2	% Neutrophils	%	Day 56	07/01/2011	57.7	.	40.0 - 75.0		
S278	315	JAIVAC-1	Dosage C	2	Haematocrit	%	Day 56	07/01/2011	44.8	.	40.0 - 54.0		
S278	315	JAIVAC-1	Dosage C	2	Haemoglobin	gm / dl	Day 56	07/01/2011	15.4	.	13.5 - 18.0		
S278	315	JAIVAC-1	Dosage C	2	MCH	pg	Day 56	07/01/2011	31	.	27.0 - 31.0		
S278	315	JAIVAC-1	Dosage C	2	MCHC	gm %	Day 56	07/01/2011	34.3	.	32.0 - 36.0		
S278	315	JAIVAC-1	Dosage C	2	MCV	fL	Day 56	07/01/2011	90.2	.	80.0- 96.0		
S278	315	JAIVAC-1	Dosage C	2	Platelet Count	10E3/ul	Day 56	07/01/2011	289	.	150 - 400		
S278	315	JAIVAC-1	Dosage C	2	RBC count	10E6/ul	Day 56	07/01/2011	4.96	.	4.60 - 6.20		
S278	315	JAIVAC-1	Dosage C	2	TLC	10E3/ul	Day 56	07/01/2011	8.45	.	4.50 - 11.00		
S278	315	JAIVAC-1	Dosage C	3	% Basophils	%	Day 180	11/05/2011	0.8	.	0.0 - 1.0		
S278	315	JAIVAC-1	Dosage C	3	% Eosinophils	%	Day 180	11/05/2011	6.4	Yes	1.0 - 6.0	H	NCS

Subject No.	Randomisation Number	Study Group	Dosage	Dose	Haematology Parameters	Unit	Day	Date of sample Collection	Results	Out of Range	Reference Range	Flag	Clinical Significance **
S278	315	JAIVAC-1	Dosage C	3	% Lymphocytes	%	Day 180	11/05/2011	28.3	.	20.0 - 45.0		
S278	315	JAIVAC-1	Dosage C	3	% Monocytes	%	Day 180	11/05/2011	4	.	2.0 - 10.0		
S278	315	JAIVAC-1	Dosage C	3	% Neutrophils	%	Day 180	11/05/2011	60.5	.	40.0 - 75.0		
S278	315	JAIVAC-1	Dosage C	3	Haematocrit	%	Day 180	11/05/2011	47.3	.	40.0 - 54.0		
S278	315	JAIVAC-1	Dosage C	3	Haemoglobin	gm / dl	Day 180	11/05/2011	16.4	.	13.5 - 18.0		
S278	315	JAIVAC-1	Dosage C	3	MCH	pg	Day 180	11/05/2011	32	Yes	27.0 - 31.0	H	NCS
S278	315	JAIVAC-1	Dosage C	3	MCHC	gm %	Day 180	11/05/2011	34.7	.	32.0 - 36.0		
S278	315	JAIVAC-1	Dosage C	3	MCV	fL	Day 180	11/05/2011	92.4	.	80.0- 96.0		
S278	315	JAIVAC-1	Dosage C	3	Platelet Count	10E3/ul	Day 180	11/05/2011	301	.	150 - 400		
S278	315	JAIVAC-1	Dosage C	3	RBC count	10E6/ul	Day 180	11/05/2011	5.11	.	4.60 - 6.20		
S278	315	JAIVAC-1	Dosage C	3	TLC	10E3/ul	Day 180	11/05/2011	9.25	.	4.50 - 11.00		
S278	315	JAIVAC-1	Dosage C	3	% Basophils	%	Day 208	09/06/2011	1.9	Yes	0.0 - 1.0	H	NCS
S278	315	JAIVAC-1	Dosage C	3	% Eosinophils	%	Day 208	09/06/2011	8	Yes	1.0 - 6.0	H	NCS
S278	315	JAIVAC-1	Dosage C	3	% Lymphocytes	%	Day 208	09/06/2011	21.1	.	20.0 - 45.0		
S278	315	JAIVAC-1	Dosage C	3	% Monocytes	%	Day 208	09/06/2011	4.2	.	2.0 - 10.0		
S278	315	JAIVAC-1	Dosage C	3	% Neutrophils	%	Day 208	09/06/2011	64.9	.	40.0 - 75.0		
S278	315	JAIVAC-1	Dosage C	3	Haematocrit	%	Day 208	09/06/2011	48.5	.	40.0 - 54.0		
S278	315	JAIVAC-1	Dosage C	3	Haemoglobin	gm / dl	Day 208	09/06/2011	16.5	.	13.5 - 18.0		
S278	315	JAIVAC-1	Dosage C	3	MCH	pg	Day 208	09/06/2011	31.5	Yes	27.0 - 31.0	H	NCS
S278	315	JAIVAC-1	Dosage C	3	MCHC	gm %	Day 208	09/06/2011	34	.	32.0 - 36.0		
S278	315	JAIVAC-1	Dosage C	3	MCV	fL	Day 208	09/06/2011	92.7	.	80.0- 96.0		
S278	315	JAIVAC-1	Dosage C	3	Platelet Count	10E3/ul	Day 208	09/06/2011	247	.	150 - 400		
S278	315	JAIVAC-1	Dosage C	3	RBC count	10E6/ul	Day 208	09/06/2011	5.23	.	4.60 - 6.20		
S278	315	JAIVAC-1	Dosage C	3	TLC	10E3/ul	Day 208	09/06/2011	7.23	.	4.50 - 11.00		
S278	315	JAIVAC-1	Dosage C	3	% Basophils	%	Day 365	10/11/2011	1.8	Yes	0.0 - 1.0	H	NCS
S278	315	JAIVAC-1	Dosage C	3	% Eosinophils	%	Day 365	10/11/2011	4.9	.	1.0 - 6.0		
S278	315	JAIVAC-1	Dosage C	3	% Lymphocytes	%	Day 365	10/11/2011	24.2	.	20.0 - 45.0		
S278	315	JAIVAC-1	Dosage C	3	% Monocytes	%	Day 365	10/11/2011	4.2	.	2.0 - 10.0		
S278	315	JAIVAC-1	Dosage C	3	% Neutrophils	%	Day 365	10/11/2011	64.8	.	40.0 - 75.0		

Subject No.	Randomisation Number	Study Group	Dosage	Dose	Haematology Parameters	Unit	Day	Date of sample Collection	Results	Out of Range	Reference Range	Flag	Clinical Significance **
S278	315	JAIVAC-1	Dosage C	3	Haematocrit	%	Day 365	10/11/2011	48.4	.	40.0 - 54.0		
S278	315	JAIVAC-1	Dosage C	3	Haemoglobin	gm / dl	Day 365	10/11/2011	16.7	.	13.5 - 18.0		
S278	315	JAIVAC-1	Dosage C	3	MCH	pg	Day 365	10/11/2011	32.1	Yes	27.0 - 31.0	H	NCS
S278	315	JAIVAC-1	Dosage C	3	MCHC	gm %	Day 365	10/11/2011	34.4	.	32.0 - 36.0		
S278	315	JAIVAC-1	Dosage C	3	MCV	fL	Day 365	10/11/2011	93.1	.	80.0- 96.0		
S278	315	JAIVAC-1	Dosage C	3	Platelet Count	10E3/ul	Day 365	10/11/2011	288	.	150 - 400		
S278	315	JAIVAC-1	Dosage C	3	RBC count	10E6/ul	Day 365	10/11/2011	5.21	.	4.60 - 6.20		
S278	315	JAIVAC-1	Dosage C	3	TLC	10E3/ul	Day 365	10/11/2011	9.82	.	4.50 - 11.00		
S279	301	JAIVAC-1	Dosage C		% Basophils	%	Day -14 to Day -1	03/11/2010	0.5	.	0.0 - 1.0		
S279	301	JAIVAC-1	Dosage C		% Eosinophils	%	Day -14 to Day -1	03/11/2010	3.2	.	1.0 - 6.0		
S279	301	JAIVAC-1	Dosage C		% Lymphocytes	%	Day -14 to Day -1	03/11/2010	38	.	20.0 - 45.0		
S279	301	JAIVAC-1	Dosage C		% Monocytes	%	Day -14 to Day -1	03/11/2010	4.9	.	2.0 - 10.0		
S279	301	JAIVAC-1	Dosage C		% Neutrophils	%	Day -14 to Day -1	03/11/2010	53.4	.	40.0 - 75.0		
S279	301	JAIVAC-1	Dosage C		Haematocrit	%	Day -14 to Day -1	03/11/2010	44.4	.	40.0 - 54.0		
S279	301	JAIVAC-1	Dosage C		Haemoglobin	gm / dl	Day -14 to Day -1	03/11/2010	14.1	.	13.5 - 18.0		
S279	301	JAIVAC-1	Dosage C		MCH	pg	Day -14 to Day -1	03/11/2010	28	.	27.0 - 31.0		
S279	301	JAIVAC-1	Dosage C		MCHC	gm %	Day -14 to Day -1	03/11/2010	31.8	Yes	32.0 - 36.0	L	NCS
S279	301	JAIVAC-1	Dosage C		MCV	fL	Day -14 to Day -1	03/11/2010	88.2	.	80.0- 96.0		
S279	301	JAIVAC-1	Dosage C		Platelet Count	10E3/ul	Day -14 to Day -1	03/11/2010	281	.	150 - 400		
S279	301	JAIVAC-1	Dosage C		RBC count	10E6/ul	Day -14 to Day -1	03/11/2010	5.03	.	4.60 - 6.20		
S279	301	JAIVAC-1	Dosage C		TLC	10E3/ul	Day -14 to Day -1	03/11/2010	8.63	.	4.50 - 11.00		
S279	301	JAIVAC-1	Dosage C	2	% Basophils	%	Day 28	08/12/2010	1.1	Yes	0.0 - 1.0	H	NCS
S279	301	JAIVAC-1	Dosage C	2	% Eosinophils	%	Day 28	08/12/2010	4.5	.	1.0 - 6.0		
S279	301	JAIVAC-1	Dosage C	2	% Lymphocytes	%	Day 28	08/12/2010	38.4	.	20.0 - 45.0		
S279	301	JAIVAC-1	Dosage C	2	% Monocytes	%	Day 28	08/12/2010	6.1	.	2.0 - 10.0		
S279	301	JAIVAC-1	Dosage C	2	% Neutrophils	%	Day 28	08/12/2010	50	.	40.0 - 75.0		
S279	301	JAIVAC-1	Dosage C	2	Haematocrit	%	Day 28	08/12/2010	46.6	.	40.0 - 54.0		
S279	301	JAIVAC-1	Dosage C	2	Haemoglobin	gm / dl	Day 28	08/12/2010	15	.	13.5 - 18.0		
S279	301	JAIVAC-1	Dosage C	2	MCH	pg	Day 28	08/12/2010	28	.	27.0 - 31.0		

Subject No.	Randomisation Number	Study Group	Dosage	Dose	Haematology Parameters	Unit	Day	Date of sample Collection	Results	Out of Range	Reference Range	Flag	Clinical Significance **
S279	301	JAIVAC-1	Dosage C	2	MCHC	gm %	Day 28	08/12/2010	32.2	.	32.0 - 36.0		
S279	301	JAIVAC-1	Dosage C	2	MCV	fL	Day 28	08/12/2010	86.8	.	80.0- 96.0		
S279	301	JAIVAC-1	Dosage C	2	Platelet Count	10E3/ul	Day 28	08/12/2010	270	.	150 - 400		
S279	301	JAIVAC-1	Dosage C	2	RBC count	10E6/ul	Day 28	08/12/2010	5.37	.	4.60 - 6.20		
S279	301	JAIVAC-1	Dosage C	2	TLC	10E3/ul	Day 28	08/12/2010	7.54	.	4.50 - 11.00		
S279	301	JAIVAC-1	Dosage C	2	% Basophils	%	Day 56	05/01/2011	0.8	.	0.0 - 1.0		
S279	301	JAIVAC-1	Dosage C	2	% Eosinophils	%	Day 56	05/01/2011	3.2	.	1.0 - 6.0		
S279	301	JAIVAC-1	Dosage C	2	% Lymphocytes	%	Day 56	05/01/2011	37.4	.	20.0 - 45.0		
S279	301	JAIVAC-1	Dosage C	2	% Monocytes	%	Day 56	05/01/2011	5	.	2.0 - 10.0		
S279	301	JAIVAC-1	Dosage C	2	% Neutrophils	%	Day 56	05/01/2011	53.7	.	40.0 - 75.0		
S279	301	JAIVAC-1	Dosage C	2	Haematocrit	%	Day 56	05/01/2011	42.7	.	40.0 - 54.0		
S279	301	JAIVAC-1	Dosage C	2	Haemoglobin	gm / dl	Day 56	05/01/2011	13.6	.	13.5 - 18.0		
S279	301	JAIVAC-1	Dosage C	2	MCH	pg	Day 56	05/01/2011	27.6	.	27.0 - 31.0		
S279	301	JAIVAC-1	Dosage C	2	MCHC	gm %	Day 56	05/01/2011	31.9	Yes	32.0 - 36.0	L	NCS
S279	301	JAIVAC-1	Dosage C	2	MCV	fL	Day 56	05/01/2011	86.6	.	80.0- 96.0		
S279	301	JAIVAC-1	Dosage C	2	Platelet Count	10E3/ul	Day 56	05/01/2011	344	.	150 - 400		
S279	301	JAIVAC-1	Dosage C	2	RBC count	10E6/ul	Day 56	05/01/2011	4.93	.	4.60 - 6.20		
S279	301	JAIVAC-1	Dosage C	2	TLC	10E3/ul	Day 56	05/01/2011	7.35	.	4.50 - 11.00		
S279	301	JAIVAC-1	Dosage C	3	% Basophils	%	Day 180	09/05/2011	1.4	Yes	0.0 - 1.0	H	NCS
S279	301	JAIVAC-1	Dosage C	3	% Eosinophils	%	Day 180	09/05/2011	6.8	Yes	1.0 - 6.0	H	NCS
S279	301	JAIVAC-1	Dosage C	3	% Lymphocytes	%	Day 180	09/05/2011	28.6	.	20.0 - 45.0		
S279	301	JAIVAC-1	Dosage C	3	% Monocytes	%	Day 180	09/05/2011	4.2	.	2.0 - 10.0		
S279	301	JAIVAC-1	Dosage C	3	% Neutrophils	%	Day 180	09/05/2011	59	.	40.0 - 75.0		
S279	301	JAIVAC-1	Dosage C	3	Haematocrit	%	Day 180	09/05/2011	43.5	.	40.0 - 54.0		
S279	301	JAIVAC-1	Dosage C	3	Haemoglobin	gm / dl	Day 180	09/05/2011	14.5	.	13.5 - 18.0		
S279	301	JAIVAC-1	Dosage C	3	MCH	pg	Day 180	09/05/2011	29.6	.	27.0 - 31.0		
S279	301	JAIVAC-1	Dosage C	3	MCHC	gm %	Day 180	09/05/2011	33.3	.	32.0 - 36.0		
S279	301	JAIVAC-1	Dosage C	3	MCV	fL	Day 180	09/05/2011	89.1	.	80.0- 96.0		
S279	301	JAIVAC-1	Dosage C	3	Platelet Count	10E3/ul	Day 180	09/05/2011	287	.	150 - 400		

Subject No.	Randomisation Number	Study Group	Dosage	Dose	Haematology Parameters	Unit	Day	Date of sample Collection	Results	Out of Range	Reference Range	Flag	Clinical Significance **
S279	301	JAIVAC-1	Dosage C	3	RBC count	10E6/ul	Day 180	09/05/2011	4.88	.	4.60 - 6.20		
S279	301	JAIVAC-1	Dosage C	3	TLC	10E3/ul	Day 180	09/05/2011	6.94	.	4.50 - 11.00		
S279	301	JAIVAC-1	Dosage C	3	% Basophils	%	Day 208	06/06/2011	1.2	Yes	0.0 - 1.0	H	NCS
S279	301	JAIVAC-1	Dosage C	3	% Eosinophils	%	Day 208	06/06/2011	2.8	.	1.0 - 6.0		
S279	301	JAIVAC-1	Dosage C	3	% Lymphocytes	%	Day 208	06/06/2011	33.8	.	20.0 - 45.0		
S279	301	JAIVAC-1	Dosage C	3	% Monocytes	%	Day 208	06/06/2011	4.6	.	2.0 - 10.0		
S279	301	JAIVAC-1	Dosage C	3	% Neutrophils	%	Day 208	06/06/2011	57.6	.	40.0 - 75.0		
S279	301	JAIVAC-1	Dosage C	3	Haematocrit	%	Day 208	06/06/2011	44.9	.	40.0 - 54.0		
S279	301	JAIVAC-1	Dosage C	3	Haemoglobin	gm / dl	Day 208	06/06/2011	14.9	.	13.5 - 18.0		
S279	301	JAIVAC-1	Dosage C	3	MCH	pg	Day 208	06/06/2011	29.7	.	27.0 - 31.0		
S279	301	JAIVAC-1	Dosage C	3	MCHC	gm %	Day 208	06/06/2011	33.1	.	32.0 - 36.0		
S279	301	JAIVAC-1	Dosage C	3	MCV	fL	Day 208	06/06/2011	89.7	.	80.0- 96.0		
S279	301	JAIVAC-1	Dosage C	3	Platelet Count	10E3/ul	Day 208	06/06/2011	344	.	150 - 400		
S279	301	JAIVAC-1	Dosage C	3	RBC count	10E6/ul	Day 208	06/06/2011	5.01	.	4.60 - 6.20		
S279	301	JAIVAC-1	Dosage C	3	TLC	10E3/ul	Day 208	06/06/2011	8.37	.	4.50 - 11.00		
S279	301	JAIVAC-1	Dosage C	3	% Basophils	%	Day 365	09/11/2011	1.1	Yes	0.0 - 1.0	H	NCS
S279	301	JAIVAC-1	Dosage C	3	% Eosinophils	%	Day 365	09/11/2011	3	.	1.0 - 6.0		
S279	301	JAIVAC-1	Dosage C	3	% Lymphocytes	%	Day 365	09/11/2011	32.1	.	20.0 - 45.0		
S279	301	JAIVAC-1	Dosage C	3	% Monocytes	%	Day 365	09/11/2011	5.8	.	2.0 - 10.0		
S279	301	JAIVAC-1	Dosage C	3	% Neutrophils	%	Day 365	09/11/2011	58.1	.	40.0 - 75.0		
S279	301	JAIVAC-1	Dosage C	3	Haematocrit	%	Day 365	09/11/2011	48.2	.	40.0 - 54.0		
S279	301	JAIVAC-1	Dosage C	3	Haemoglobin	gm / dl	Day 365	09/11/2011	15.3	.	13.5 - 18.0		
S279	301	JAIVAC-1	Dosage C	3	MCH	pg	Day 365	09/11/2011	28.9	.	27.0 - 31.0		
S279	301	JAIVAC-1	Dosage C	3	MCHC	gm %	Day 365	09/11/2011	31.7	Yes	32.0 - 36.0	L	NCS
S279	301	JAIVAC-1	Dosage C	3	MCV	fL	Day 365	09/11/2011	91.1	.	80.0- 96.0		
S279	301	JAIVAC-1	Dosage C	3	Platelet Count	10E3/ul	Day 365	09/11/2011	343	.	150 - 400		
S279	301	JAIVAC-1	Dosage C	3	RBC count	10E6/ul	Day 365	09/11/2011	5.29	.	4.60 - 6.20		
S279	301	JAIVAC-1	Dosage C	3	TLC	10E3/ul	Day 365	09/11/2011	7.58	.	4.50 - 11.00		
S281	304	JAIVAC-1	Dosage C		% Basophils	%	Day -14 to Day -1	03/11/2010	0	.	0.0 - 1.0		

Subject No.	Randomisation Number	Study Group	Dosage	Dose	Haematology Parameters	Unit	Day	Date of sample Collection	Results	Out of Range	Reference Range	Flag	Clinical Significance **
S281	304	JAIVAC-1	Dosage C		% Eosinophils	%	Day -14 to Day -1	03/11/2010	6	.	1.0 - 6.0		
S281	304	JAIVAC-1	Dosage C		% Lymphocytes	%	Day -14 to Day -1	03/11/2010	32	.	20.0 - 45.0		
S281	304	JAIVAC-1	Dosage C		% Monocytes	%	Day -14 to Day -1	03/11/2010	9	.	2.0 - 10.0		
S281	304	JAIVAC-1	Dosage C		% Neutrophils	%	Day -14 to Day -1	03/11/2010	53	.	40.0 - 75.0		
S281	304	JAIVAC-1	Dosage C		Haematocrit	%	Day -14 to Day -1	03/11/2010	41.1	.	40.0 - 54.0		
S281	304	JAIVAC-1	Dosage C		Haemoglobin*	gm / dl	Day -14 to Day -1	03/11/2010	13.4	.	13.5 - 18.0	L	
S281	304	JAIVAC-1	Dosage C		MCH	pg	Day -14 to Day -1	03/11/2010	26.4	Yes	27.0 - 31.0	L	NCS
S281	304	JAIVAC-1	Dosage C		MCHC	gm %	Day -14 to Day -1	03/11/2010	32.5	.	32.0 - 36.0		
S281	304	JAIVAC-1	Dosage C		MCV	fL	Day -14 to Day -1	03/11/2010	81.1	.	80.0- 96.0		
S281	304	JAIVAC-1	Dosage C		Platelet Count	10E3/ul	Day -14 to Day -1	03/11/2010	216	.	150 - 400		
S281	304	JAIVAC-1	Dosage C		RBC count	10E6/ul	Day -14 to Day -1	03/11/2010	5.07	.	4.60 - 6.20		
S281	304	JAIVAC-1	Dosage C		TLC	10E3/ul	Day -14 to Day -1	03/11/2010	4.39	.	4.50 - 11.00	L	
S281	304	JAIVAC-1	Dosage C	2	% Basophils	%	Day 28	09/12/2010	4.1	Yes	0.0 - 1.0	H	NCS
S281	304	JAIVAC-1	Dosage C	2	% Eosinophils	%	Day 28	09/12/2010	2.7	.	1.0 - 6.0		
S281	304	JAIVAC-1	Dosage C	2	% Lymphocytes	%	Day 28	09/12/2010	24.2	.	20.0 - 45.0		
S281	304	JAIVAC-1	Dosage C	2	% Monocytes	%	Day 28	09/12/2010	8.7	.	2.0 - 10.0		
S281	304	JAIVAC-1	Dosage C	2	% Neutrophils	%	Day 28	09/12/2010	60.4	.	40.0 - 75.0		
S281	304	JAIVAC-1	Dosage C	2	Haematocrit	%	Day 28	09/12/2010	41	.	40.0 - 54.0		
S281	304	JAIVAC-1	Dosage C	2	Haemoglobin*	gm / dl	Day 28	09/12/2010	13.1	Yes	13.5 - 18.0	L	NCS
S281	304	JAIVAC-1	Dosage C	2	MCH	pg	Day 28	09/12/2010	25	Yes	27.0 - 31.0	L	NCS
S281	304	JAIVAC-1	Dosage C	2	MCHC	gm %	Day 28	09/12/2010	31.9	Yes	32.0 - 36.0	L	NCS
S281	304	JAIVAC-1	Dosage C	2	MCV	fL	Day 28	09/12/2010	78.2	Yes	80.0- 96.0	L	NCS
S281	304	JAIVAC-1	Dosage C	2	Platelet Count	10E3/ul	Day 28	09/12/2010	181	.	150 - 400		
S281	304	JAIVAC-1	Dosage C	2	RBC count	10E6/ul	Day 28	09/12/2010	5.25	.	4.60 - 6.20		
S281	304	JAIVAC-1	Dosage C	2	TLC	10E3/ul	Day 28	09/12/2010	3.32	Yes	4.50 - 11.00	L	NCS
S281	304	JAIVAC-1	Dosage C	2	% Basophils	%	Day 56	06/01/2011	0.7	.	0.0 - 1.0		
S281	304	JAIVAC-1	Dosage C	2	% Eosinophils	%	Day 56	06/01/2011	2	.	1.0 - 6.0		
S281	304	JAIVAC-1	Dosage C	2	% Lymphocytes	%	Day 56	06/01/2011	22.8	.	20.0 - 45.0		
S281	304	JAIVAC-1	Dosage C	2	% Monocytes	%	Day 56	06/01/2011	5.3	.	2.0 - 10.0		

Subject No.	Randomisation Number	Study Group	Dosage	Dose	Haematology Parameters	Unit	Day	Date of sample Collection	Results	Out of Range	Reference Range	Flag	Clinical Significance **
S281	304	JAIVAC-1	Dosage C	2	% Neutrophils	%	Day 56	06/01/2011	69.2	.	40.0 - 75.0		
S281	304	JAIVAC-1	Dosage C	2	Haematocrit	%	Day 56	06/01/2011	39.2	Yes	40.0 - 54.0	L	NCS
S281	304	JAIVAC-1	Dosage C	2	Haemoglobin*	gm / dl	Day 56	06/01/2011	12.7	Yes	13.5 - 18.0	L	CS
S281	304	JAIVAC-1	Dosage C	2	MCH	pg	Day 56	06/01/2011	24.8	Yes	27.0 - 31.0	L	CS
S281	304	JAIVAC-1	Dosage C	2	MCHC	gm %	Day 56	06/01/2011	32.5	.	32.0 - 36.0		
S281	304	JAIVAC-1	Dosage C	2	MCV	fL	Day 56	06/01/2011	76.2	Yes	80.0- 96.0	L	CS
S281	304	JAIVAC-1	Dosage C	2	Platelet Count	10E3/ul	Day 56	06/01/2011	193	.	150 - 400		
S281	304	JAIVAC-1	Dosage C	2	RBC count	10E6/ul	Day 56	06/01/2011	5.14	.	4.60 - 6.20		
S281	304	JAIVAC-1	Dosage C	2	TLC	10E3/ul	Day 56	06/01/2011	4.85	.	4.50 - 11.00		
S281	304	JAIVAC-1	Dosage C		% Basophils	%	Early Termination	11/05/2011	0.9	.	0.0 - 1.0		
S281	304	JAIVAC-1	Dosage C		% Eosinophils	%	Early Termination	11/05/2011	2	.	1.0 - 6.0		
S281	304	JAIVAC-1	Dosage C		% Lymphocytes	%	Early Termination	11/05/2011	28.3	.	20.0 - 45.0		
S281	304	JAIVAC-1	Dosage C		% Monocytes	%	Early Termination	11/05/2011	8.1	.	2.0 - 10.0		
S281	304	JAIVAC-1	Dosage C		% Neutrophils	%	Early Termination	11/05/2011	60.7	.	40.0 - 75.0		
S281	304	JAIVAC-1	Dosage C		Haematocrit	%	Early Termination	11/05/2011	39.7	Yes	40.0 - 54.0	L	CS
S281	304	JAIVAC-1	Dosage C		Haemoglobin*	gm / dl	Early Termination	11/05/2011	12.4	Yes	13.5 - 18.0	L	CS
S281	304	JAIVAC-1	Dosage C		MCH	pg	Early Termination	11/05/2011	22.9	Yes	27.0 - 31.0	L	CS
S281	304	JAIVAC-1	Dosage C		MCHC	gm %	Early Termination	11/05/2011	31.2	Yes	32.0 - 36.0	L	CS
S281	304	JAIVAC-1	Dosage C		MCV	fL	Early Termination	11/05/2011	73.2	Yes	80.0- 96.0	L	CS
S281	304	JAIVAC-1	Dosage C		Platelet Count	10E3/ul	Early Termination	11/05/2011	187	.	150 - 400		
S281	304	JAIVAC-1	Dosage C		RBC count	10E6/ul	Early Termination	11/05/2011	5.43	.	4.60 - 6.20		
S281	304	JAIVAC-1	Dosage C		TLC	10E3/ul	Early Termination	11/05/2011	4	Yes	4.50 - 11.00	L	NCS
S284	305	JAIVAC-1	Dosage C		% Basophils	%	Day -14 to Day -1	03/11/2010	1.9	Yes	0.0 - 1.0	H	NCS
S284	305	JAIVAC-1	Dosage C		% Eosinophils	%	Day -14 to Day -1	03/11/2010	1.8	.	1.0 - 6.0		
S284	305	JAIVAC-1	Dosage C		% Lymphocytes	%	Day -14 to Day -1	03/11/2010	28	.	20.0 - 45.0		
S284	305	JAIVAC-1	Dosage C		% Monocytes	%	Day -14 to Day -1	03/11/2010	4.9	.	2.0 - 10.0		
S284	305	JAIVAC-1	Dosage C		% Neutrophils	%	Day -14 to Day -1	03/11/2010	63.4	.	40.0 - 75.0		
S284	305	JAIVAC-1	Dosage C		Haematocrit	%	Day -14 to Day -1	03/11/2010	45.6	.	40.0 - 54.0		
S284	305	JAIVAC-1	Dosage C		Haemoglobin	gm / dl	Day -14 to Day -1	03/11/2010	15.2	.	13.5 - 18.0		

Subject No.	Randomisation Number	Study Group	Dosage	Dose	Haematology Parameters	Unit	Day	Date of sample Collection	Results	Out of Range	Reference Range	Flag	Clinical Significance **
S284	305	JAIVAC-1	Dosage C		MCH	pg	Day -14 to Day -1	03/11/2010	27.8	.	27.0 - 31.0		
S284	305	JAIVAC-1	Dosage C		MCHC	gm %	Day -14 to Day -1	03/11/2010	33.4	.	32.0 - 36.0		
S284	305	JAIVAC-1	Dosage C		MCV	fL	Day -14 to Day -1	03/11/2010	83.2	.	80.0- 96.0		
S284	305	JAIVAC-1	Dosage C		Platelet Count	10E3/ul	Day -14 to Day -1	03/11/2010	228	.	150 - 400		
S284	305	JAIVAC-1	Dosage C		RBC count	10E6/ul	Day -14 to Day -1	03/11/2010	5.48	.	4.60 - 6.20		
S284	305	JAIVAC-1	Dosage C		TLC	10E3/ul	Day -14 to Day -1	03/11/2010	8.85	.	4.50 - 11.00		
S284	305	JAIVAC-1	Dosage C	2	% Basophils	%	Day 28	09/12/2010	2.1	Yes	0.0 - 1.0	H	NCS
S284	305	JAIVAC-1	Dosage C	2	% Eosinophils	%	Day 28	09/12/2010	1.4	.	1.0 - 6.0		
S284	305	JAIVAC-1	Dosage C	2	% Lymphocytes	%	Day 28	09/12/2010	20.7	.	20.0 - 45.0		
S284	305	JAIVAC-1	Dosage C	2	% Monocytes	%	Day 28	09/12/2010	6.4	.	2.0 - 10.0		
S284	305	JAIVAC-1	Dosage C	2	% Neutrophils	%	Day 28	09/12/2010	69.2	.	40.0 - 75.0		
S284	305	JAIVAC-1	Dosage C	2	Haematocrit	%	Day 28	09/12/2010	48	.	40.0 - 54.0		
S284	305	JAIVAC-1	Dosage C	2	Haemoglobin	gm / dl	Day 28	09/12/2010	16.1	.	13.5 - 18.0		
S284	305	JAIVAC-1	Dosage C	2	MCH	pg	Day 28	09/12/2010	27.3	.	27.0 - 31.0		
S284	305	JAIVAC-1	Dosage C	2	MCHC	gm %	Day 28	09/12/2010	33.5	.	32.0 - 36.0		
S284	305	JAIVAC-1	Dosage C	2	MCV	fL	Day 28	09/12/2010	81.6	.	80.0- 96.0		
S284	305	JAIVAC-1	Dosage C	2	Platelet Count	10E3/ul	Day 28	09/12/2010	225	.	150 - 400		
S284	305	JAIVAC-1	Dosage C	2	RBC count	10E6/ul	Day 28	09/12/2010	5.88	.	4.60 - 6.20		
S284	305	JAIVAC-1	Dosage C	2	TLC	10E3/ul	Day 28	09/12/2010	7.53	.	4.50 - 11.00		
S284	305	JAIVAC-1	Dosage C	2	% Basophils	%	Day 56	06/01/2011	1.8	Yes	0.0 - 1.0	H	NCS
S284	305	JAIVAC-1	Dosage C	2	% Eosinophils	%	Day 56	06/01/2011	1.9	.	1.0 - 6.0		
S284	305	JAIVAC-1	Dosage C	2	% Lymphocytes	%	Day 56	06/01/2011	20.7	.	20.0 - 45.0		
S284	305	JAIVAC-1	Dosage C	2	% Monocytes	%	Day 56	06/01/2011	7.3	.	2.0 - 10.0		
S284	305	JAIVAC-1	Dosage C	2	% Neutrophils	%	Day 56	06/01/2011	68.2	.	40.0 - 75.0		
S284	305	JAIVAC-1	Dosage C	2	Haematocrit	%	Day 56	06/01/2011	49.3	.	40.0 - 54.0		
S284	305	JAIVAC-1	Dosage C	2	Haemoglobin	gm / dl	Day 56	06/01/2011	16.6	.	13.5 - 18.0		
S284	305	JAIVAC-1	Dosage C	2	MCH	pg	Day 56	06/01/2011	27.2	.	27.0 - 31.0		
S284	305	JAIVAC-1	Dosage C	2	MCHC	gm %	Day 56	06/01/2011	33.6	.	32.0 - 36.0		
S284	305	JAIVAC-1	Dosage C	2	MCV	fL	Day 56	06/01/2011	80.8	.	80.0- 96.0		

Subject No.	Randomisation Number	Study Group	Dosage	Dose	Haematology Parameters	Unit	Day	Date of sample Collection	Results	Out of Range	Reference Range	Flag	Clinical Significance **
S284	305	JAIVAC-1	Dosage C	2	Platelet Count	10E3/ul	Day 56	06/01/2011	239	.	150 - 400		
S284	305	JAIVAC-1	Dosage C	2	RBC count	10E6/ul	Day 56	06/01/2011	6.1	.	4.60 - 6.20		
S284	305	JAIVAC-1	Dosage C	2	TLC	10E3/ul	Day 56	06/01/2011	9.02	.	4.50 - 11.00		
S284	305	JAIVAC-1	Dosage C	3	% Basophils	%	Day 180	10/05/2011	1	.	0.0 - 1.0		
S284	305	JAIVAC-1	Dosage C	3	% Eosinophils	%	Day 180	10/05/2011	1.3	.	1.0 - 6.0		
S284	305	JAIVAC-1	Dosage C	3	% Lymphocytes	%	Day 180	10/05/2011	23.4	.	20.0 - 45.0		
S284	305	JAIVAC-1	Dosage C	3	% Monocytes	%	Day 180	10/05/2011	3.8	.	2.0 - 10.0		
S284	305	JAIVAC-1	Dosage C	3	% Neutrophils	%	Day 180	10/05/2011	70.4	.	40.0 - 75.0		
S284	305	JAIVAC-1	Dosage C	3	Haematocrit	%	Day 180	10/05/2011	47.6	.	40.0 - 54.0		
S284	305	JAIVAC-1	Dosage C	3	Haemoglobin	gm / dl	Day 180	10/05/2011	16	.	13.5 - 18.0		
S284	305	JAIVAC-1	Dosage C	3	MCH	pg	Day 180	10/05/2011	27.9	.	27.0 - 31.0		
S284	305	JAIVAC-1	Dosage C	3	MCHC	gm %	Day 180	10/05/2011	33.7	.	32.0 - 36.0		
S284	305	JAIVAC-1	Dosage C	3	MCV	fL	Day 180	10/05/2011	82.9	.	80.0- 96.0		
S284	305	JAIVAC-1	Dosage C	3	Platelet Count	10E3/ul	Day 180	10/05/2011	238	.	150 - 400		
S284	305	JAIVAC-1	Dosage C	3	RBC count	10E6/ul	Day 180	10/05/2011	5.74	.	4.60 - 6.20		
S284	305	JAIVAC-1	Dosage C	3	TLC	10E3/ul	Day 180	10/05/2011	8.87	.	4.50 - 11.00		
S284	305	JAIVAC-1	Dosage C	3	% Basophils	%	Day 208	07/06/2011	0	.	0.0 - 1.0		
S284	305	JAIVAC-1	Dosage C	3	% Eosinophils	%	Day 208	07/06/2011	3	.	1.0 - 6.0		
S284	305	JAIVAC-1	Dosage C	3	% Lymphocytes	%	Day 208	07/06/2011	25	.	20.0 - 45.0		
S284	305	JAIVAC-1	Dosage C	3	% Monocytes	%	Day 208	07/06/2011	6	.	2.0 - 10.0		
S284	305	JAIVAC-1	Dosage C	3	% Neutrophils	%	Day 208	07/06/2011	66	.	40.0 - 75.0		
S284	305	JAIVAC-1	Dosage C	3	Haematocrit	%	Day 208	07/06/2011	45.5	.	40.0 - 54.0		
S284	305	JAIVAC-1	Dosage C	3	Haemoglobin	gm / dl	Day 208	07/06/2011	15.2	.	13.5 - 18.0		
S284	305	JAIVAC-1	Dosage C	3	MCH	pg	Day 208	07/06/2011	27.9	.	27.0 - 31.0		
S284	305	JAIVAC-1	Dosage C	3	MCHC	gm %	Day 208	07/06/2011	33.5	.	32.0 - 36.0		
S284	305	JAIVAC-1	Dosage C	3	MCV	fL	Day 208	07/06/2011	83.2	.	80.0- 96.0		
S284	305	JAIVAC-1	Dosage C	3	Platelet Count	10E3/ul	Day 208	07/06/2011	208	.	150 - 400		
S284	305	JAIVAC-1	Dosage C	3	RBC count	10E6/ul	Day 208	07/06/2011	5.46	.	4.60 - 6.20		
S284	305	JAIVAC-1	Dosage C	3	TLC	10E3/ul	Day 208	07/06/2011	5.11	.	4.50 - 11.00		

Subject No.	Randomisation Number	Study Group	Dosage	Dose	Haematology Parameters	Unit	Day	Date of sample Collection	Results	Out of Range	Reference Range	Flag	Clinical Significance **
S284	305	JAIVAC-1	Dosage C	3	% Basophils	%	Day 365	09/11/2011	0	.	0.0 - 1.0		
S284	305	JAIVAC-1	Dosage C	3	% Eosinophils	%	Day 365	09/11/2011	2	.	1.0 - 6.0		
S284	305	JAIVAC-1	Dosage C	3	% Lymphocytes	%	Day 365	09/11/2011	27	.	20.0 - 45.0		
S284	305	JAIVAC-1	Dosage C	3	% Monocytes	%	Day 365	09/11/2011	6	.	2.0 - 10.0		
S284	305	JAIVAC-1	Dosage C	3	% Neutrophils	%	Day 365	09/11/2011	65	.	40.0 - 75.0		
S284	305	JAIVAC-1	Dosage C	3	Haematocrit	%	Day 365	09/11/2011	43.4	.	40.0 - 54.0		
S284	305	JAIVAC-1	Dosage C	3	Haemoglobin	gm / dl	Day 365	09/11/2011	14.3	.	13.5 - 18.0		
S284	305	JAIVAC-1	Dosage C	3	MCH	pg	Day 365	09/11/2011	26.9	Yes	27.0 - 31.0	L	NCS
S284	305	JAIVAC-1	Dosage C	3	MCHC	gm %	Day 365	09/11/2011	32.9	.	32.0 - 36.0		
S284	305	JAIVAC-1	Dosage C	3	MCV	fL	Day 365	09/11/2011	81.7	.	80.0- 96.0		
S284	305	JAIVAC-1	Dosage C	3	Platelet Count	10E3/ul	Day 365	09/11/2011	217	.	150 - 400		
S284	305	JAIVAC-1	Dosage C	3	RBC count	10E6/ul	Day 365	09/11/2011	5.31	.	4.60 - 6.20		
S284	305	JAIVAC-1	Dosage C	3	TLC	10E3/ul	Day 365	09/11/2011	7.87	.	4.50 - 11.00		
S285	307	Hepatitis B	Hepatitis B		% Basophils	%	Day -14 to Day -1	02/11/2010	1.3	Yes	0.0 - 1.0	H	NCS
S285	307	Hepatitis B	Hepatitis B		% Eosinophils	%	Day -14 to Day -1	02/11/2010	3.3	.	1.0 - 6.0		
S285	307	Hepatitis B	Hepatitis B		% Lymphocytes	%	Day -14 to Day -1	02/11/2010	42.6	.	20.0 - 45.0		
S285	307	Hepatitis B	Hepatitis B		% Monocytes	%	Day -14 to Day -1	02/11/2010	5.6	.	2.0 - 10.0		
S285	307	Hepatitis B	Hepatitis B		% Neutrophils	%	Day -14 to Day -1	02/11/2010	47.3	.	40.0 - 75.0		
S285	307	Hepatitis B	Hepatitis B		Haematocrit	%	Day -14 to Day -1	02/11/2010	45.4	.	40.0 - 54.0		
S285	307	Hepatitis B	Hepatitis B		Haemoglobin	gm / dl	Day -14 to Day -1	02/11/2010	15.5	.	13.5 - 18.0		
S285	307	Hepatitis B	Hepatitis B		MCH	pg	Day -14 to Day -1	02/11/2010	29.3	.	27.0 - 31.0		
S285	307	Hepatitis B	Hepatitis B		MCHC	gm %	Day -14 to Day -1	02/11/2010	34.1	.	32.0 - 36.0		
S285	307	Hepatitis B	Hepatitis B		MCV	fL	Day -14 to Day -1	02/11/2010	85.7	.	80.0- 96.0		
S285	307	Hepatitis B	Hepatitis B		Platelet Count	10E3/ul	Day -14 to Day -1	02/11/2010	287	.	150 - 400		
S285	307	Hepatitis B	Hepatitis B		RBC count	10E6/ul	Day -14 to Day -1	02/11/2010	5.29	.	4.60 - 6.20		
S285	307	Hepatitis B	Hepatitis B		TLC	10E3/ul	Day -14 to Day -1	02/11/2010	6.35	.	4.50 - 11.00		
S285	307	Hepatitis B	Hepatitis B	2	% Basophils	%	Day 28	09/12/2010	1.7	Yes	0.0 - 1.0	H	NCS
S285	307	Hepatitis B	Hepatitis B	2	% Eosinophils	%	Day 28	09/12/2010	2.9	.	1.0 - 6.0		
S285	307	Hepatitis B	Hepatitis B	2	% Lymphocytes	%	Day 28	09/12/2010	31.4	.	20.0 - 45.0		

Subject No.	Randomisation Number	Study Group	Dosage	Dose	Haematology Parameters	Unit	Day	Date of sample Collection	Results	Out of Range	Reference Range	Flag	Clinical Significance **
S285	307	Hepatitis B	Hepatitis B	2	% Monocytes	%	Day 28	09/12/2010	4.3	.	2.0 - 10.0		
S285	307	Hepatitis B	Hepatitis B	2	% Neutrophils	%	Day 28	09/12/2010	59.7	.	40.0 - 75.0		
S285	307	Hepatitis B	Hepatitis B	2	Haematocrit	%	Day 28	09/12/2010	44.6	.	40.0 - 54.0		
S285	307	Hepatitis B	Hepatitis B	2	Haemoglobin	gm / dl	Day 28	09/12/2010	15	.	13.5 - 18.0		
S285	307	Hepatitis B	Hepatitis B	2	MCH	pg	Day 28	09/12/2010	28.8	.	27.0 - 31.0		
S285	307	Hepatitis B	Hepatitis B	2	MCHC	gm %	Day 28	09/12/2010	33.6	.	32.0 - 36.0		
S285	307	Hepatitis B	Hepatitis B	2	MCV	fL	Day 28	09/12/2010	85.6	.	80.0- 96.0		
S285	307	Hepatitis B	Hepatitis B	2	Platelet Count	10E3/ul	Day 28	09/12/2010	302	.	150 - 400		
S285	307	Hepatitis B	Hepatitis B	2	RBC count	10E6/ul	Day 28	09/12/2010	5.21	.	4.60 - 6.20		
S285	307	Hepatitis B	Hepatitis B	2	TLC	10E3/ul	Day 28	09/12/2010	5.84	.	4.50 - 11.00		
S285	307	Hepatitis B	Hepatitis B	2	% Basophils	%	Day 56	06/01/2011	0	.	0.0 - 1.0		
S285	307	Hepatitis B	Hepatitis B	2	% Eosinophils	%	Day 56	06/01/2011	6	.	1.0 - 6.0		
S285	307	Hepatitis B	Hepatitis B	2	% Lymphocytes	%	Day 56	06/01/2011	26	.	20.0 - 45.0		
S285	307	Hepatitis B	Hepatitis B	2	% Monocytes	%	Day 56	06/01/2011	7	.	2.0 - 10.0		
S285	307	Hepatitis B	Hepatitis B	2	% Neutrophils	%	Day 56	06/01/2011	61	.	40.0 - 75.0		
S285	307	Hepatitis B	Hepatitis B	2	Haematocrit	%	Day 56	06/01/2011	46.9	.	40.0 - 54.0		
S285	307	Hepatitis B	Hepatitis B	2	Haemoglobin	gm / dl	Day 56	06/01/2011	16	.	13.5 - 18.0		
S285	307	Hepatitis B	Hepatitis B	2	MCH	pg	Day 56	06/01/2011	29.1	.	27.0 - 31.0		
S285	307	Hepatitis B	Hepatitis B	2	MCHC	gm %	Day 56	06/01/2011	34.1	.	32.0 - 36.0		
S285	307	Hepatitis B	Hepatitis B	2	MCV	fL	Day 56	06/01/2011	85.1	.	80.0- 96.0		
S285	307	Hepatitis B	Hepatitis B	2	Platelet Count	10E3/ul	Day 56	06/01/2011	314	.	150 - 400		
S285	307	Hepatitis B	Hepatitis B	2	RBC count	10E6/ul	Day 56	06/01/2011	5.51	.	4.60 - 6.20		
S285	307	Hepatitis B	Hepatitis B	2	TLC	10E3/ul	Day 56	06/01/2011	5.88	.	4.50 - 11.00		
S285	307	Hepatitis B	Hepatitis B	3	% Basophils	%	Day 180	10/05/2011	1.2	Yes	0.0 - 1.0	H	NCS
S285	307	Hepatitis B	Hepatitis B	3	% Eosinophils	%	Day 180	10/05/2011	3.4	.	1.0 - 6.0		
S285	307	Hepatitis B	Hepatitis B	3	% Lymphocytes	%	Day 180	10/05/2011	33.8	.	20.0 - 45.0		
S285	307	Hepatitis B	Hepatitis B	3	% Monocytes	%	Day 180	10/05/2011	5.6	.	2.0 - 10.0		
S285	307	Hepatitis B	Hepatitis B	3	% Neutrophils	%	Day 180	10/05/2011	56	.	40.0 - 75.0		
S285	307	Hepatitis B	Hepatitis B	3	Haematocrit	%	Day 180	10/05/2011	47.1	.	40.0 - 54.0		

Subject No.	Randomisation Number	Study Group	Dosage	Dose	Haematology Parameters	Unit	Day	Date of sample Collection	Results	Out of Range	Reference Range	Flag	Clinical Significance **
S285	307	Hepatitis B	Hepatitis B	3	Haemoglobin	gm / dl	Day 180	10/05/2011	16	.	13.5 - 18.0		
S285	307	Hepatitis B	Hepatitis B	3	MCH	pg	Day 180	10/05/2011	29.6	.	27.0 - 31.0		
S285	307	Hepatitis B	Hepatitis B	3	MCHC	gm %	Day 180	10/05/2011	34	.	32.0 - 36.0		
S285	307	Hepatitis B	Hepatitis B	3	MCV	fL	Day 180	10/05/2011	87.2	.	80.0- 96.0		
S285	307	Hepatitis B	Hepatitis B	3	Platelet Count	10E3/ul	Day 180	10/05/2011	321	.	150 - 400		
S285	307	Hepatitis B	Hepatitis B	3	RBC count	10E6/ul	Day 180	10/05/2011	5.4	.	4.60 - 6.20		
S285	307	Hepatitis B	Hepatitis B	3	TLC	10E3/ul	Day 180	10/05/2011	6.93	.	4.50 - 11.00		
S285	307	Hepatitis B	Hepatitis B	3	% Basophils	%	Day 208	07/06/2011	2.4	Yes	0.0 - 1.0	H	NCS
S285	307	Hepatitis B	Hepatitis B	3	% Eosinophils	%	Day 208	07/06/2011	3.9	.	1.0 - 6.0		
S285	307	Hepatitis B	Hepatitis B	3	% Lymphocytes	%	Day 208	07/06/2011	32.4	.	20.0 - 45.0		
S285	307	Hepatitis B	Hepatitis B	3	% Monocytes	%	Day 208	07/06/2011	5.6	.	2.0 - 10.0		
S285	307	Hepatitis B	Hepatitis B	3	% Neutrophils	%	Day 208	07/06/2011	55.7	.	40.0 - 75.0		
S285	307	Hepatitis B	Hepatitis B	3	Haematocrit	%	Day 208	07/06/2011	47.7	.	40.0 - 54.0		
S285	307	Hepatitis B	Hepatitis B	3	Haemoglobin	gm / dl	Day 208	07/06/2011	15.9	.	13.5 - 18.0		
S285	307	Hepatitis B	Hepatitis B	3	MCH	pg	Day 208	07/06/2011	29.4	.	27.0 - 31.0		
S285	307	Hepatitis B	Hepatitis B	3	MCHC	gm %	Day 208	07/06/2011	33.4	.	32.0 - 36.0		
S285	307	Hepatitis B	Hepatitis B	3	MCV	fL	Day 208	07/06/2011	88	.	80.0- 96.0		
S285	307	Hepatitis B	Hepatitis B	3	Platelet Count	10E3/ul	Day 208	07/06/2011	298	.	150 - 400		
S285	307	Hepatitis B	Hepatitis B	3	RBC count	10E6/ul	Day 208	07/06/2011	5.43	.	4.60 - 6.20		
S285	307	Hepatitis B	Hepatitis B	3	TLC	10E3/ul	Day 208	07/06/2011	6.39	.	4.50 - 11.00		
S285	307	Hepatitis B	Hepatitis B	3	% Basophils	%	Day 365	09/11/2011	0	.	0.0 - 1.0		
S285	307	Hepatitis B	Hepatitis B	3	% Eosinophils	%	Day 365	09/11/2011	4	.	1.0 - 6.0		
S285	307	Hepatitis B	Hepatitis B	3	% Lymphocytes	%	Day 365	09/11/2011	32	.	20.0 - 45.0		
S285	307	Hepatitis B	Hepatitis B	3	% Monocytes	%	Day 365	09/11/2011	6	.	2.0 - 10.0		
S285	307	Hepatitis B	Hepatitis B	3	% Neutrophils	%	Day 365	09/11/2011	58	.	40.0 - 75.0		
S285	307	Hepatitis B	Hepatitis B	3	Haematocrit	%	Day 365	09/11/2011	49	.	40.0 - 54.0		
S285	307	Hepatitis B	Hepatitis B	3	Haemoglobin	gm / dl	Day 365	09/11/2011	16.5	.	13.5 - 18.0		
S285	307	Hepatitis B	Hepatitis B	3	MCH	pg	Day 365	09/11/2011	28.9	.	27.0 - 31.0		
S285	307	Hepatitis B	Hepatitis B	3	MCHC	gm %	Day 365	09/11/2011	33.6	.	32.0 - 36.0		

Subject No.	Randomisation Number	Study Group	Dosage	Dose	Haematology Parameters	Unit	Day	Date of sample Collection	Results	Out of Range	Reference Range	Flag	Clinical Significance **
S285	307	Hepatitis B	Hepatitis B	3	MCV	fL	Day 365	09/11/2011	86.1	.	80.0- 96.0		
S285	307	Hepatitis B	Hepatitis B	3	Platelet Count	10E3/ul	Day 365	09/11/2011	302	.	150 - 400		
S285	307	Hepatitis B	Hepatitis B	3	RBC count	10E6/ul	Day 365	09/11/2011	5.7	.	4.60 - 6.20		
S285	307	Hepatitis B	Hepatitis B	3	TLC	10E3/ul	Day 365	09/11/2011	4.56	.	4.50 - 11.00		
S291	312	JAIVAC-1	Dosage C		% Basophils	%	Day -14 to Day -1	02/11/2010	0.6	.	0.0 - 1.0		
S291	312	JAIVAC-1	Dosage C		% Eosinophils	%	Day -14 to Day -1	02/11/2010	7.1	.	1.0 - 6.0	H	
S291	312	JAIVAC-1	Dosage C		% Lymphocytes	%	Day -14 to Day -1	02/11/2010	23.9	.	20.0 - 45.0		
S291	312	JAIVAC-1	Dosage C		% Monocytes	%	Day -14 to Day -1	02/11/2010	3.3	.	2.0 - 10.0		
S291	312	JAIVAC-1	Dosage C		% Neutrophils	%	Day -14 to Day -1	02/11/2010	65.1	.	40.0 - 75.0		
S291	312	JAIVAC-1	Dosage C		Haematocrit	%	Day -14 to Day -1	02/11/2010	46.4	.	40.0 - 54.0		
S291	312	JAIVAC-1	Dosage C		Haemoglobin	gm / dl	Day -14 to Day -1	02/11/2010	16.1	.	13.5 - 18.0		
S291	312	JAIVAC-1	Dosage C		MCH	pg	Day -14 to Day -1	02/11/2010	29.5	.	27.0 - 31.0		
S291	312	JAIVAC-1	Dosage C		MCHC	gm %	Day -14 to Day -1	02/11/2010	34.7	.	32.0 - 36.0		
S291	312	JAIVAC-1	Dosage C		MCV	fL	Day -14 to Day -1	02/11/2010	85	.	80.0- 96.0		
S291	312	JAIVAC-1	Dosage C		Platelet Count	10E3/ul	Day -14 to Day -1	02/11/2010	265	.	150 - 400		
S291	312	JAIVAC-1	Dosage C		RBC count	10E6/ul	Day -14 to Day -1	02/11/2010	5.45	.	4.60 - 6.20		
S291	312	JAIVAC-1	Dosage C		TLC	10E3/ul	Day -14 to Day -1	02/11/2010	8.26	.	4.50 - 11.00		
S291	312	JAIVAC-1	Dosage C	2	% Basophils	%	Day 28	10/12/2010	0.3	.	0.0 - 1.0		
S291	312	JAIVAC-1	Dosage C	2	% Eosinophils	%	Day 28	10/12/2010	5.8	.	1.0 - 6.0		
S291	312	JAIVAC-1	Dosage C	2	% Lymphocytes	%	Day 28	10/12/2010	33	.	20.0 - 45.0		
S291	312	JAIVAC-1	Dosage C	2	% Monocytes	%	Day 28	10/12/2010	3.1	.	2.0 - 10.0		
S291	312	JAIVAC-1	Dosage C	2	% Neutrophils	%	Day 28	10/12/2010	57.8	.	40.0 - 75.0		
S291	312	JAIVAC-1	Dosage C	2	Haematocrit	%	Day 28	10/12/2010	45.6	.	40.0 - 54.0		
S291	312	JAIVAC-1	Dosage C	2	Haemoglobin	gm / dl	Day 28	10/12/2010	15.4	.	13.5 - 18.0		
S291	312	JAIVAC-1	Dosage C	2	MCH	pg	Day 28	10/12/2010	28.9	.	27.0 - 31.0		
S291	312	JAIVAC-1	Dosage C	2	MCHC	gm %	Day 28	10/12/2010	33.8	.	32.0 - 36.0		
S291	312	JAIVAC-1	Dosage C	2	MCV	fL	Day 28	10/12/2010	85.5	.	80.0- 96.0		
S291	312	JAIVAC-1	Dosage C	2	Platelet Count	10E3/ul	Day 28	10/12/2010	283	.	150 - 400		
S291	312	JAIVAC-1	Dosage C	2	RBC count	10E6/ul	Day 28	10/12/2010	5.33	.	4.60 - 6.20		

Subject No.	Randomisation Number	Study Group	Dosage	Dose	Haematology Parameters	Unit	Day	Date of sample Collection	Results	Out of Range	Reference Range	Flag	Clinical Significance **
S291	312	JAIVAC-1	Dosage C	2	TLC	10E3/ul	Day 28	10/12/2010	5.71	.	4.50 - 11.00		
S291	312	JAIVAC-1	Dosage C	2	% Basophils	%	Day 56	07/01/2011	0.8	.	0.0 - 1.0		
S291	312	JAIVAC-1	Dosage C	2	% Eosinophils	%	Day 56	07/01/2011	10.7	Yes	1.0 - 6.0	H	NCS
S291	312	JAIVAC-1	Dosage C	2	% Lymphocytes	%	Day 56	07/01/2011	31.9	.	20.0 - 45.0		
S291	312	JAIVAC-1	Dosage C	2	% Monocytes	%	Day 56	07/01/2011	3.9	.	2.0 - 10.0		
S291	312	JAIVAC-1	Dosage C	2	% Neutrophils	%	Day 56	07/01/2011	52.7	.	40.0 - 75.0		
S291	312	JAIVAC-1	Dosage C	2	Haematocrit	%	Day 56	07/01/2011	44.3	.	40.0 - 54.0		
S291	312	JAIVAC-1	Dosage C	2	Haemoglobin	gm / dl	Day 56	07/01/2011	15.6	.	13.5 - 18.0		
S291	312	JAIVAC-1	Dosage C	2	MCH	pg	Day 56	07/01/2011	29.8	.	27.0 - 31.0		
S291	312	JAIVAC-1	Dosage C	2	MCHC	gm %	Day 56	07/01/2011	35.3	.	32.0 - 36.0		
S291	312	JAIVAC-1	Dosage C	2	MCV	fL	Day 56	07/01/2011	84.4	.	80.0- 96.0		
S291	312	JAIVAC-1	Dosage C	2	Platelet Count	10E3/ul	Day 56	07/01/2011	272	.	150 - 400		
S291	312	JAIVAC-1	Dosage C	2	RBC count	10E6/ul	Day 56	07/01/2011	5.25	.	4.60 - 6.20		
S291	312	JAIVAC-1	Dosage C	2	TLC	10E3/ul	Day 56	07/01/2011	6.82	.	4.50 - 11.00		
S291	312	JAIVAC-1	Dosage C	3	% Basophils	%	Day 180	11/05/2011	1.2	Yes	0.0 - 1.0	H	NCS
S291	312	JAIVAC-1	Dosage C	3	% Eosinophils	%	Day 180	11/05/2011	8.9	Yes	1.0 - 6.0	H	NCS
S291	312	JAIVAC-1	Dosage C	3	% Lymphocytes	%	Day 180	11/05/2011	24.2	.	20.0 - 45.0		
S291	312	JAIVAC-1	Dosage C	3	% Monocytes	%	Day 180	11/05/2011	6.4	.	2.0 - 10.0		
S291	312	JAIVAC-1	Dosage C	3	% Neutrophils	%	Day 180	11/05/2011	59.4	.	40.0 - 75.0		
S291	312	JAIVAC-1	Dosage C	3	Haematocrit	%	Day 180	11/05/2011	48.5	.	40.0 - 54.0		
S291	312	JAIVAC-1	Dosage C	3	Haemoglobin	gm / dl	Day 180	11/05/2011	16.6	.	13.5 - 18.0		
S291	312	JAIVAC-1	Dosage C	3	MCH	pg	Day 180	11/05/2011	31	.	27.0 - 31.0		
S291	312	JAIVAC-1	Dosage C	3	MCHC	gm %	Day 180	11/05/2011	34.3	.	32.0 - 36.0		
S291	312	JAIVAC-1	Dosage C	3	MCV	fL	Day 180	11/05/2011	90.4	.	80.0- 96.0		
S291	312	JAIVAC-1	Dosage C	3	Platelet Count	10E3/ul	Day 180	11/05/2011	245	.	150 - 400		
S291	312	JAIVAC-1	Dosage C	3	RBC count	10E6/ul	Day 180	11/05/2011	5.37	.	4.60 - 6.20		
S291	312	JAIVAC-1	Dosage C	3	TLC	10E3/ul	Day 180	11/05/2011	5.88	.	4.50 - 11.00		
S291	312	JAIVAC-1	Dosage C	3	% Basophils	%	Day 208	08/06/2011	1.3	Yes	0.0 - 1.0	H	NCS
S291	312	JAIVAC-1	Dosage C	3	% Eosinophils	%	Day 208	08/06/2011	6.2	Yes	1.0 - 6.0	H	NCS

Subject No.	Randomisation Number	Study Group	Dosage	Dose	Haematology Parameters	Unit	Day	Date of sample Collection	Results	Out of Range	Reference Range	Flag	Clinical Significance **
S291	312	JAIVAC-1	Dosage C	3	% Lymphocytes	%	Day 208	08/06/2011	35	.	20.0 - 45.0		
S291	312	JAIVAC-1	Dosage C	3	% Monocytes	%	Day 208	08/06/2011	5.5	.	2.0 - 10.0		
S291	312	JAIVAC-1	Dosage C	3	% Neutrophils	%	Day 208	08/06/2011	52	.	40.0 - 75.0		
S291	312	JAIVAC-1	Dosage C	3	Haematocrit	%	Day 208	08/06/2011	49	.	40.0 - 54.0		
S291	312	JAIVAC-1	Dosage C	3	Haemoglobin	gm / dl	Day 208	08/06/2011	16.6	.	13.5 - 18.0		
S291	312	JAIVAC-1	Dosage C	3	MCH	pg	Day 208	08/06/2011	30.1	.	27.0 - 31.0		
S291	312	JAIVAC-1	Dosage C	3	MCHC	gm %	Day 208	08/06/2011	33.8	.	32.0 - 36.0		
S291	312	JAIVAC-1	Dosage C	3	MCV	fL	Day 208	08/06/2011	89	.	80.0- 96.0		
S291	312	JAIVAC-1	Dosage C	3	Platelet Count	10E3/ul	Day 208	08/06/2011	261	.	150 - 400		
S291	312	JAIVAC-1	Dosage C	3	RBC count	10E6/ul	Day 208	08/06/2011	5.51	.	4.60 - 6.20		
S291	312	JAIVAC-1	Dosage C	3	TLC	10E3/ul	Day 208	08/06/2011	5.88	.	4.50 - 11.00		
S291	312	JAIVAC-1	Dosage C	3	% Basophils	%	Day 365	10/11/2011	1.2	Yes	0.0 - 1.0	H	NCS
S291	312	JAIVAC-1	Dosage C	3	% Eosinophils	%	Day 365	10/11/2011	5.5	.	1.0 - 6.0		
S291	312	JAIVAC-1	Dosage C	3	% Lymphocytes	%	Day 365	10/11/2011	32.2	.	20.0 - 45.0		
S291	312	JAIVAC-1	Dosage C	3	% Monocytes	%	Day 365	10/11/2011	3.6	.	2.0 - 10.0		
S291	312	JAIVAC-1	Dosage C	3	% Neutrophils	%	Day 365	10/11/2011	57.5	.	40.0 - 75.0		
S291	312	JAIVAC-1	Dosage C	3	Haematocrit	%	Day 365	10/11/2011	43.2	.	40.0 - 54.0		
S291	312	JAIVAC-1	Dosage C	3	Haemoglobin	gm / dl	Day 365	10/11/2011	14.6	.	13.5 - 18.0		
S291	312	JAIVAC-1	Dosage C	3	MCH	pg	Day 365	10/11/2011	28.8	.	27.0 - 31.0		
S291	312	JAIVAC-1	Dosage C	3	MCHC	gm %	Day 365	10/11/2011	33.7	.	32.0 - 36.0		
S291	312	JAIVAC-1	Dosage C	3	MCV	fL	Day 365	10/11/2011	85.4	.	80.0- 96.0		
S291	312	JAIVAC-1	Dosage C	3	Platelet Count	10E3/ul	Day 365	10/11/2011	264	.	150 - 400		
S291	312	JAIVAC-1	Dosage C	3	RBC count	10E6/ul	Day 365	10/11/2011	5.06	.	4.60 - 6.20		
S291	312	JAIVAC-1	Dosage C	3	TLC	10E3/ul	Day 365	10/11/2011	6.54	.	4.50 - 11.00		
S298	314	JAIVAC-1	Dosage C		% Basophils	%	Day -14 to Day -1	09/11/2010	1.4	Yes	0.0 - 1.0	H	NCS
S298	314	JAIVAC-1	Dosage C		% Eosinophils	%	Day -14 to Day -1	09/11/2010	1.3	.	1.0 - 6.0		
S298	314	JAIVAC-1	Dosage C		% Lymphocytes	%	Day -14 to Day -1	09/11/2010	29.6	.	20.0 - 45.0		
S298	314	JAIVAC-1	Dosage C		% Monocytes	%	Day -14 to Day -1	09/11/2010	4.9	.	2.0 - 10.0		
S298	314	JAIVAC-1	Dosage C		% Neutrophils	%	Day -14 to Day -1	09/11/2010	62.8	.	40.0 - 75.0		

Subject No.	Randomisation Number	Study Group	Dosage	Dose	Haematology Parameters	Unit	Day	Date of sample Collection	Results	Out of Range	Reference Range	Flag	Clinical Significance **
S298	314	JAIVAC-1	Dosage C		Haematocrit	%	Day -14 to Day -1	09/11/2010	47	.	40.0 - 54.0		
S298	314	JAIVAC-1	Dosage C		Haemoglobin	gm / dl	Day -14 to Day -1	09/11/2010	16	.	13.5 - 18.0		
S298	314	JAIVAC-1	Dosage C		MCH	pg	Day -14 to Day -1	09/11/2010	27.6	.	27.0 - 31.0		
S298	314	JAIVAC-1	Dosage C		MCHC	gm %	Day -14 to Day -1	09/11/2010	33.9	.	32.0 - 36.0		
S298	314	JAIVAC-1	Dosage C		MCV	fL	Day -14 to Day -1	09/11/2010	81.4	.	80.0- 96.0		
S298	314	JAIVAC-1	Dosage C		Platelet Count	10E3/ul	Day -14 to Day -1	09/11/2010	297	.	150 - 400		
S298	314	JAIVAC-1	Dosage C		RBC count	10E6/ul	Day -14 to Day -1	09/11/2010	5.78	.	4.60 - 6.20		
S298	314	JAIVAC-1	Dosage C		TLC	10E3/ul	Day -14 to Day -1	09/11/2010	6.54	.	4.50 - 11.00		
S298	314	JAIVAC-1	Dosage C	2	% Basophils	%	Day 28	10/12/2010	1.3	Yes	0.0 - 1.0	H	NCS
S298	314	JAIVAC-1	Dosage C	2	% Eosinophils	%	Day 28	10/12/2010	2.2	.	1.0 - 6.0		
S298	314	JAIVAC-1	Dosage C	2	% Lymphocytes	%	Day 28	10/12/2010	37	.	20.0 - 45.0		
S298	314	JAIVAC-1	Dosage C	2	% Monocytes	%	Day 28	10/12/2010	5.3	.	2.0 - 10.0		
S298	314	JAIVAC-1	Dosage C	2	% Neutrophils	%	Day 28	10/12/2010	54.1	.	40.0 - 75.0		
S298	314	JAIVAC-1	Dosage C	2	Haematocrit	%	Day 28	10/12/2010	48.8	.	40.0 - 54.0		
S298	314	JAIVAC-1	Dosage C	2	Haemoglobin	gm / dl	Day 28	10/12/2010	16.3	.	13.5 - 18.0		
S298	314	JAIVAC-1	Dosage C	2	MCH	pg	Day 28	10/12/2010	27.2	.	27.0 - 31.0		
S298	314	JAIVAC-1	Dosage C	2	MCHC	gm %	Day 28	10/12/2010	33.4	.	32.0 - 36.0		
S298	314	JAIVAC-1	Dosage C	2	MCV	fL	Day 28	10/12/2010	81.3	.	80.0- 96.0		
S298	314	JAIVAC-1	Dosage C	2	Platelet Count	10E3/ul	Day 28	10/12/2010	320	.	150 - 400		
S298	314	JAIVAC-1	Dosage C	2	RBC count	10E6/ul	Day 28	10/12/2010	6	.	4.60 - 6.20		
S298	314	JAIVAC-1	Dosage C	2	TLC	10E3/ul	Day 28	10/12/2010	5.57	.	4.50 - 11.00		
S298	314	JAIVAC-1	Dosage C	2	% Basophils	%	Day 56	07/01/2011	0.6	.	0.0 - 1.0		
S298	314	JAIVAC-1	Dosage C	2	% Eosinophils	%	Day 56	07/01/2011	0.7	Yes	1.0 - 6.0	L	NCS
S298	314	JAIVAC-1	Dosage C	2	% Lymphocytes	%	Day 56	07/01/2011	27.1	.	20.0 - 45.0		
S298	314	JAIVAC-1	Dosage C	2	% Monocytes	%	Day 56	07/01/2011	4.1	.	2.0 - 10.0		
S298	314	JAIVAC-1	Dosage C	2	% Neutrophils	%	Day 56	07/01/2011	67.5	.	40.0 - 75.0		
S298	314	JAIVAC-1	Dosage C	2	Haematocrit	%	Day 56	07/01/2011	47.1	.	40.0 - 54.0		
S298	314	JAIVAC-1	Dosage C	2	Haemoglobin	gm / dl	Day 56	07/01/2011	15.8	.	13.5 - 18.0		
S298	314	JAIVAC-1	Dosage C	2	MCH	pg	Day 56	07/01/2011	27	.	27.0 - 31.0		

Subject No.	Randomisation Number	Study Group	Dosage	Dose	Haematology Parameters	Unit	Day	Date of sample Collection	Results	Out of Range	Reference Range	Flag	Clinical Significance **
S298	314	JAIVAC-1	Dosage C	2	MCHC	gm %	Day 56	07/01/2011	33.6	.	32.0 - 36.0		
S298	314	JAIVAC-1	Dosage C	2	MCV	fL	Day 56	07/01/2011	80.5	.	80.0- 96.0		
S298	314	JAIVAC-1	Dosage C	2	Platelet Count	10E3/ul	Day 56	07/01/2011	381	.	150 - 400		
S298	314	JAIVAC-1	Dosage C	2	RBC count	10E6/ul	Day 56	07/01/2011	5.86	.	4.60 - 6.20		
S298	314	JAIVAC-1	Dosage C	2	TLC	10E3/ul	Day 56	07/01/2011	7.52	.	4.50 - 11.00		
S298	314	JAIVAC-1	Dosage C	3	% Basophils	%	Day 180	11/05/2011	1.2	Yes	0.0 - 1.0	H	NCS
S298	314	JAIVAC-1	Dosage C	3	% Eosinophils	%	Day 180	11/05/2011	1.5	.	1.0 - 6.0		
S298	314	JAIVAC-1	Dosage C	3	% Lymphocytes	%	Day 180	11/05/2011	28.2	.	20.0 - 45.0		
S298	314	JAIVAC-1	Dosage C	3	% Monocytes	%	Day 180	11/05/2011	5.3	.	2.0 - 10.0		
S298	314	JAIVAC-1	Dosage C	3	% Neutrophils	%	Day 180	11/05/2011	63.8	.	40.0 - 75.0		
S298	314	JAIVAC-1	Dosage C	3	Haematocrit	%	Day 180	11/05/2011	50.6	.	40.0 - 54.0		
S298	314	JAIVAC-1	Dosage C	3	Haemoglobin	gm / dl	Day 180	11/05/2011	16.6	.	13.5 - 18.0		
S298	314	JAIVAC-1	Dosage C	3	MCH	pg	Day 180	11/05/2011	27.3	.	27.0 - 31.0		
S298	314	JAIVAC-1	Dosage C	3	MCHC	gm %	Day 180	11/05/2011	32.7	.	32.0 - 36.0		
S298	314	JAIVAC-1	Dosage C	3	MCV	fL	Day 180	11/05/2011	83.4	.	80.0- 96.0		
S298	314	JAIVAC-1	Dosage C	3	Platelet Count	10E3/ul	Day 180	11/05/2011	325	.	150 - 400		
S298	314	JAIVAC-1	Dosage C	3	RBC count	10E6/ul	Day 180	11/05/2011	6.07	.	4.60 - 6.20		
S298	314	JAIVAC-1	Dosage C	3	TLC	10E3/ul	Day 180	11/05/2011	7.65	.	4.50 - 11.00		
S298	314	JAIVAC-1	Dosage C	3	% Basophils	%	Day 208	08/06/2011	1.1	Yes	0.0 - 1.0	H	NCS
S298	314	JAIVAC-1	Dosage C	3	% Eosinophils	%	Day 208	08/06/2011	1.4	.	1.0 - 6.0		
S298	314	JAIVAC-1	Dosage C	3	% Lymphocytes	%	Day 208	08/06/2011	31.3	.	20.0 - 45.0		
S298	314	JAIVAC-1	Dosage C	3	% Monocytes	%	Day 208	08/06/2011	5.5	.	2.0 - 10.0		
S298	314	JAIVAC-1	Dosage C	3	% Neutrophils	%	Day 208	08/06/2011	60.7	.	40.0 - 75.0		
S298	314	JAIVAC-1	Dosage C	3	Haematocrit	%	Day 208	08/06/2011	46.5	.	40.0 - 54.0		
S298	314	JAIVAC-1	Dosage C	3	Haemoglobin	gm / dl	Day 208	08/06/2011	15.5	.	13.5 - 18.0		
S298	314	JAIVAC-1	Dosage C	3	MCH	pg	Day 208	08/06/2011	28.1	.	27.0 - 31.0		
S298	314	JAIVAC-1	Dosage C	3	MCHC	gm %	Day 208	08/06/2011	33.3	.	32.0 - 36.0		
S298	314	JAIVAC-1	Dosage C	3	MCV	fL	Day 208	08/06/2011	84.4	.	80.0- 96.0		
S298	314	JAIVAC-1	Dosage C	3	Platelet Count	10E3/ul	Day 208	08/06/2011	334	.	150 - 400		

Subject No.	Randomisation Number	Study Group	Dosage	Dose	Haematology Parameters	Unit	Day	Date of sample Collection	Results	Out of Range	Reference Range	Flag	Clinical Significance **
S298	314	JAIVAC-1	Dosage C	3	RBC count	10E6/ul	Day 208	08/06/2011	5.51	.	4.60 - 6.20		
S298	314	JAIVAC-1	Dosage C	3	TLC	10E3/ul	Day 208	08/06/2011	6.51	.	4.50 - 11.00		
S298	314	JAIVAC-1	Dosage C	3	% Basophils	%	Day 365	10/11/2011	2.4	Yes	0.0 - 1.0	H	NCS
S298	314	JAIVAC-1	Dosage C	3	% Eosinophils	%	Day 365	10/11/2011	3	.	1.0 - 6.0		
S298	314	JAIVAC-1	Dosage C	3	% Lymphocytes	%	Day 365	10/11/2011	23.5	.	20.0 - 45.0		
S298	314	JAIVAC-1	Dosage C	3	% Monocytes	%	Day 365	10/11/2011	4.8	.	2.0 - 10.0		
S298	314	JAIVAC-1	Dosage C	3	% Neutrophils	%	Day 365	10/11/2011	66.4	.	40.0 - 75.0		
S298	314	JAIVAC-1	Dosage C	3	Haematocrit	%	Day 365	10/11/2011	46.2	.	40.0 - 54.0		
S298	314	JAIVAC-1	Dosage C	3	Haemoglobin	gm / dl	Day 365	10/11/2011	15	.	13.5 - 18.0		
S298	314	JAIVAC-1	Dosage C	3	MCH	pg	Day 365	10/11/2011	27.2	.	27.0 - 31.0		
S298	314	JAIVAC-1	Dosage C	3	MCHC	gm %	Day 365	10/11/2011	32.5	.	32.0 - 36.0		
S298	314	JAIVAC-1	Dosage C	3	MCV	fL	Day 365	10/11/2011	83.7	.	80.0- 96.0		
S298	314	JAIVAC-1	Dosage C	3	Platelet Count	10E3/ul	Day 365	10/11/2011	306	.	150 - 400		
S298	314	JAIVAC-1	Dosage C	3	RBC count	10E6/ul	Day 365	10/11/2011	5.52	.	4.60 - 6.20		
S298	314	JAIVAC-1	Dosage C	3	TLC	10E3/ul	Day 365	10/11/2011	7.31	.	4.50 - 11.00		
S299	310	JAIVAC-1	Dosage C		% Basophils	%	Day -14 to Day -1	08/11/2010	0.7	.	0.0 - 1.0		
S299	310	JAIVAC-1	Dosage C		% Eosinophils	%	Day -14 to Day -1	08/11/2010	3.1	.	1.0 - 6.0		
S299	310	JAIVAC-1	Dosage C		% Lymphocytes	%	Day -14 to Day -1	08/11/2010	30.2	.	20.0 - 45.0		
S299	310	JAIVAC-1	Dosage C		% Monocytes	%	Day -14 to Day -1	08/11/2010	4.4	.	2.0 - 10.0		
S299	310	JAIVAC-1	Dosage C		% Neutrophils	%	Day -14 to Day -1	08/11/2010	61.5	.	40.0 - 75.0		
S299	310	JAIVAC-1	Dosage C		Haematocrit	%	Day -14 to Day -1	08/11/2010	43.9	.	40.0 - 54.0		
S299	310	JAIVAC-1	Dosage C		Haemoglobin	gm / dl	Day -14 to Day -1	08/11/2010	14.6	.	13.5 - 18.0		
S299	310	JAIVAC-1	Dosage C		MCH	pg	Day -14 to Day -1	08/11/2010	27.4	.	27.0 - 31.0		
S299	310	JAIVAC-1	Dosage C		MCHC	gm %	Day -14 to Day -1	08/11/2010	33.3	.	32.0 - 36.0		
S299	310	JAIVAC-1	Dosage C		MCV	fL	Day -14 to Day -1	08/11/2010	82.2	.	80.0- 96.0		
S299	310	JAIVAC-1	Dosage C		Platelet Count	10E3/ul	Day -14 to Day -1	08/11/2010	215	.	150 - 400		
S299	310	JAIVAC-1	Dosage C		RBC count	10E6/ul	Day -14 to Day -1	08/11/2010	5.34	.	4.60 - 6.20		
S299	310	JAIVAC-1	Dosage C		TLC	10E3/ul	Day -14 to Day -1	08/11/2010	6.3	.	4.50 - 11.00		
S299	310	JAIVAC-1	Dosage C	2	% Basophils	%	Day 28	10/12/2010	0.3	.	0.0 - 1.0		

Subject No.	Randomisation Number	Study Group	Dosage	Dose	Haematology Parameters	Unit	Day	Date of sample Collection	Results	Out of Range	Reference Range	Flag	Clinical Significance **
S299	310	JAIVAC-1	Dosage C	2	% Eosinophils	%	Day 28	10/12/2010	2.5	.	1.0 - 6.0		
S299	310	JAIVAC-1	Dosage C	2	% Lymphocytes	%	Day 28	10/12/2010	26.3	.	20.0 - 45.0		
S299	310	JAIVAC-1	Dosage C	2	% Monocytes	%	Day 28	10/12/2010	5.3	.	2.0 - 10.0		
S299	310	JAIVAC-1	Dosage C	2	% Neutrophils	%	Day 28	10/12/2010	65.6	.	40.0 - 75.0		
S299	310	JAIVAC-1	Dosage C	2	Haematocrit	%	Day 28	10/12/2010	46.2	.	40.0 - 54.0		
S299	310	JAIVAC-1	Dosage C	2	Haemoglobin	gm / dl	Day 28	10/12/2010	15.3	.	13.5 - 18.0		
S299	310	JAIVAC-1	Dosage C	2	MCH	pg	Day 28	10/12/2010	27.2	.	27.0 - 31.0		
S299	310	JAIVAC-1	Dosage C	2	MCHC	gm %	Day 28	10/12/2010	33.1	.	32.0 - 36.0		
S299	310	JAIVAC-1	Dosage C	2	MCV	fL	Day 28	10/12/2010	82	.	80.0- 96.0		
S299	310	JAIVAC-1	Dosage C	2	Platelet Count	10E3/ul	Day 28	10/12/2010	254	.	150 - 400		
S299	310	JAIVAC-1	Dosage C	2	RBC count	10E6/ul	Day 28	10/12/2010	5.63	.	4.60 - 6.20		
S299	310	JAIVAC-1	Dosage C	2	TLC	10E3/ul	Day 28	10/12/2010	6.09	.	4.50 - 11.00		
S299	310	JAIVAC-1	Dosage C	2	% Basophils	%	Day 56	07/01/2011	0.7	.	0.0 - 1.0		
S299	310	JAIVAC-1	Dosage C	2	% Eosinophils	%	Day 56	07/01/2011	3.8	.	1.0 - 6.0		
S299	310	JAIVAC-1	Dosage C	2	% Lymphocytes	%	Day 56	07/01/2011	30.1	.	20.0 - 45.0		
S299	310	JAIVAC-1	Dosage C	2	% Monocytes	%	Day 56	07/01/2011	4.3	.	2.0 - 10.0		
S299	310	JAIVAC-1	Dosage C	2	% Neutrophils	%	Day 56	07/01/2011	61.1	.	40.0 - 75.0		
S299	310	JAIVAC-1	Dosage C	2	Haematocrit	%	Day 56	07/01/2011	43	.	40.0 - 54.0		
S299	310	JAIVAC-1	Dosage C	2	Haemoglobin	gm / dl	Day 56	07/01/2011	14.4	.	13.5 - 18.0		
S299	310	JAIVAC-1	Dosage C	2	MCH	pg	Day 56	07/01/2011	27.9	.	27.0 - 31.0		
S299	310	JAIVAC-1	Dosage C	2	MCHC	gm %	Day 56	07/01/2011	33.5	.	32.0 - 36.0		
S299	310	JAIVAC-1	Dosage C	2	MCV	fL	Day 56	07/01/2011	83.1	.	80.0- 96.0		
S299	310	JAIVAC-1	Dosage C	2	Platelet Count	10E3/ul	Day 56	07/01/2011	310	.	150 - 400		
S299	310	JAIVAC-1	Dosage C	2	RBC count	10E6/ul	Day 56	07/01/2011	5.17	.	4.60 - 6.20		
S299	310	JAIVAC-1	Dosage C	2	TLC	10E3/ul	Day 56	07/01/2011	6.61	.	4.50 - 11.00		
S299	310	JAIVAC-1	Dosage C	3	% Basophils	%	Day 180	11/05/2011	0.9	.	0.0 - 1.0		
S299	310	JAIVAC-1	Dosage C	3	% Eosinophils	%	Day 180	11/05/2011	2.8	.	1.0 - 6.0		
S299	310	JAIVAC-1	Dosage C	3	% Lymphocytes	%	Day 180	11/05/2011	28.2	.	20.0 - 45.0		
S299	310	JAIVAC-1	Dosage C	3	% Monocytes	%	Day 180	11/05/2011	5.8	.	2.0 - 10.0		

Subject No.	Randomisation Number	Study Group	Dosage	Dose	Haematology Parameters	Unit	Day	Date of sample Collection	Results	Out of Range	Reference Range	Flag	Clinical Significance **
S299	310	JAIVAC-1	Dosage C	3	% Neutrophils	%	Day 180	11/05/2011	62.4	.	40.0 - 75.0		
S299	310	JAIVAC-1	Dosage C	3	Haematocrit	%	Day 180	11/05/2011	46.5	.	40.0 - 54.0		
S299	310	JAIVAC-1	Dosage C	3	Haemoglobin	gm / dl	Day 180	11/05/2011	15	.	13.5 - 18.0		
S299	310	JAIVAC-1	Dosage C	3	MCH	pg	Day 180	11/05/2011	27.5	.	27.0 - 31.0		
S299	310	JAIVAC-1	Dosage C	3	MCHC	gm %	Day 180	11/05/2011	32.2	.	32.0 - 36.0		
S299	310	JAIVAC-1	Dosage C	3	MCV	fL	Day 180	11/05/2011	85.3	.	80.0- 96.0		
S299	310	JAIVAC-1	Dosage C	3	Platelet Count	10E3/ul	Day 180	11/05/2011	278	.	150 - 400		
S299	310	JAIVAC-1	Dosage C	3	RBC count	10E6/ul	Day 180	11/05/2011	5.45	.	4.60 - 6.20		
S299	310	JAIVAC-1	Dosage C	3	TLC	10E3/ul	Day 180	11/05/2011	5.62	.	4.50 - 11.00		
S299	310	JAIVAC-1	Dosage C	3	% Basophils	%	Day 208	08/06/2011	1.4	Yes	0.0 - 1.0	H	NCS
S299	310	JAIVAC-1	Dosage C	3	% Eosinophils	%	Day 208	08/06/2011	3	.	1.0 - 6.0		
S299	310	JAIVAC-1	Dosage C	3	% Lymphocytes	%	Day 208	08/06/2011	25.7	.	20.0 - 45.0		
S299	310	JAIVAC-1	Dosage C	3	% Monocytes	%	Day 208	08/06/2011	5.8	.	2.0 - 10.0		
S299	310	JAIVAC-1	Dosage C	3	% Neutrophils	%	Day 208	08/06/2011	64.2	.	40.0 - 75.0		
S299	310	JAIVAC-1	Dosage C	3	Haematocrit	%	Day 208	08/06/2011	48.3	.	40.0 - 54.0		
S299	310	JAIVAC-1	Dosage C	3	Haemoglobin	gm / dl	Day 208	08/06/2011	15.8	.	13.5 - 18.0		
S299	310	JAIVAC-1	Dosage C	3	MCH	pg	Day 208	08/06/2011	27.4	.	27.0 - 31.0		
S299	310	JAIVAC-1	Dosage C	3	MCHC	gm %	Day 208	08/06/2011	32.6	.	32.0 - 36.0		
S299	310	JAIVAC-1	Dosage C	3	MCV	fL	Day 208	08/06/2011	83.9	.	80.0- 96.0		
S299	310	JAIVAC-1	Dosage C	3	Platelet Count	10E3/ul	Day 208	08/06/2011	315	.	150 - 400		
S299	310	JAIVAC-1	Dosage C	3	RBC count	10E6/ul	Day 208	08/06/2011	5.76	.	4.60 - 6.20		
S299	310	JAIVAC-1	Dosage C	3	TLC	10E3/ul	Day 208	08/06/2011	6.48	.	4.50 - 11.00		
S299	310	JAIVAC-1	Dosage C	3	% Basophils	%	Day 365	10/11/2011	1.9	Yes	0.0 - 1.0	H	NCS
S299	310	JAIVAC-1	Dosage C	3	% Eosinophils	%	Day 365	10/11/2011	4.4	.	1.0 - 6.0		
S299	310	JAIVAC-1	Dosage C	3	% Lymphocytes	%	Day 365	10/11/2011	25.4	.	20.0 - 45.0		
S299	310	JAIVAC-1	Dosage C	3	% Monocytes	%	Day 365	10/11/2011	3.9	.	2.0 - 10.0		
S299	310	JAIVAC-1	Dosage C	3	% Neutrophils	%	Day 365	10/11/2011	64.4	.	40.0 - 75.0		
S299	310	JAIVAC-1	Dosage C	3	Haematocrit	%	Day 365	10/11/2011	49.6	.	40.0 - 54.0		
S299	310	JAIVAC-1	Dosage C	3	Haemoglobin	gm / dl	Day 365	10/11/2011	16.4	.	13.5 - 18.0		

Subject No.	Randomisation Number	Study Group	Dosage	Dose	Haematology Parameters	Unit	Day	Date of sample Collection	Results	Out of Range	Reference Range	Flag	Clinical Significance **
S299	310	JAIVAC-1	Dosage C	3	MCH	pg	Day 365	10/11/2011	28.2	.	27.0 - 31.0		
S299	310	JAIVAC-1	Dosage C	3	MCHC	gm %	Day 365	10/11/2011	33	.	32.0 - 36.0		
S299	310	JAIVAC-1	Dosage C	3	MCV	fL	Day 365	10/11/2011	85.2	.	80.0- 96.0		
S299	310	JAIVAC-1	Dosage C	3	Platelet Count	10E3/ul	Day 365	10/11/2011	274	.	150 - 400		
S299	310	JAIVAC-1	Dosage C	3	RBC count	10E6/ul	Day 365	10/11/2011	5.82	.	4.60 - 6.20		
S299	310	JAIVAC-1	Dosage C	3	TLC	10E3/ul	Day 365	10/11/2011	5.99	.	4.50 - 11.00		

Note:
1) *If the haemoglobin drops below the lower limit of the normal range, then a direct Coomb's test and a peripheral blood smear/film will be prepared and examined for evidence of possible haemolysis
2) Flag: L- Below Range, N- Within Range, H- Above Range
3) Clinical Significance: CS-Clinically Significant, NCS-Not Clinically Significant
4) At Day -14 to Day -1, as per central Laboratory reference range even if the the value is out of range (H/L) but as per protocol specified reference range related to exclusion criteria the subject is eligible and Randomised and hence CS /NCS not marked for Haemoglobin, % Eosinophils, RBC count, Platelet Count and TLC at Screening.

16.2.8.3: Listing of Clinical Laboratory Evaluations – Serum Chemistry

Subject No.	Randomisation Number	Study Group	Dosage	Dose	Serum Chemistry Parameters	Unit	Day	Date of sample Collection	Results	Out of Range	Reference Range	Flag	Clinical Significance*
S108	115	Hepatitis B	Hepatitis B		ALT	U / L	Day -14 to Day -1	18/08/2010	22	.	0 - 41		
S108	115	Hepatitis B	Hepatitis B		AST	U / L	Day -14 to Day -1	18/08/2010	24	.	0 - 40		
S108	115	Hepatitis B	Hepatitis B		Alkaline Phosphatase	U / L	Day -14 to Day -1	18/08/2010	68	.	40 - 129		
S108	115	Hepatitis B	Hepatitis B		Direct Bilirubin	mg / dl	Day -14 to Day -1	18/08/2010	0.2	.	0.00 - 0.30		
S108	115	Hepatitis B	Hepatitis B		Gamma Glutamyl Transpeptidase	U / L	Day -14 to Day -1	18/08/2010	15	.	10 - 71		
S108	115	Hepatitis B	Hepatitis B		Indirect Bilirubin	mg / dl	Day -14 to Day -1	18/08/2010	0.38	.	0.00 - 0.99		
S108	115	Hepatitis B	Hepatitis B		Total Bilirubin	mg / dl	Day -14 to Day -1	18/08/2010	0.58	.	0.00 - 0.99		
S108	115	Hepatitis B	Hepatitis B		Serum creatinine	mg / dl	Day -14 to Day -1	18/08/2010	0.8	.	0.7 - 1.2		
S108	115	Hepatitis B	Hepatitis B		Potassium	mEq / L	Day -14 to Day -1	18/08/2010	4.2	.	3.5 - 5.1		
S108	115	Hepatitis B	Hepatitis B		Random blood glucose	mg / dl	Day -14 to Day -1	18/08/2010	85	.	45-130		
S108	115	Hepatitis B	Hepatitis B		Sodium	mEq / L	Day -14 to Day -1	18/08/2010	139	.	136 - 145		
S108	115	Hepatitis B	Hepatitis B	2	ALT	U / L	Day 28	24/09/2010	38	.	0 - 41		
S108	115	Hepatitis B	Hepatitis B	2	AST	U / L	Day 28	24/09/2010	35	.	0 - 40		
S108	115	Hepatitis B	Hepatitis B	2	Alkaline Phosphatase	U / L	Day 28	24/09/2010	71	.	40 - 129		
S108	115	Hepatitis B	Hepatitis B	2	Direct Bilirubin	mg / dl	Day 28	24/09/2010	0.21	.	0.00 - 0.30		
S108	115	Hepatitis B	Hepatitis B	2	Gamma Glutamyl Transpeptidase	U / L	Day 28	24/09/2010	15	.	10 - 71		
S108	115	Hepatitis B	Hepatitis B	2	Indirect Bilirubin	mg / dl	Day 28	24/09/2010	0.6	.	0.00 - 0.99		
S108	115	Hepatitis B	Hepatitis B	2	Total Bilirubin	mg / dl	Day 28	24/09/2010	0.81	.	0.00 - 0.99		
S108	115	Hepatitis B	Hepatitis B	2	Serum creatinine	mg / dl	Day 28	24/09/2010	0.8	.	0.7 - 1.2		
S108	115	Hepatitis B	Hepatitis B	2	Potassium	mEq / L	Day 28	24/09/2010	4.4	.	3.5 - 5.1		
S108	115	Hepatitis B	Hepatitis B	2	Random blood glucose	mg / dl	Day 28	24/09/2010	78	.	45-130		
S108	115	Hepatitis B	Hepatitis B	2	Sodium	mEq / L	Day 28	24/09/2010	137	.	136 - 145		
S108	115	Hepatitis B	Hepatitis B	2	ALT	U / L	Day 56	22/10/2010	43	Yes	0 - 41	H	NCS
S108	115	Hepatitis B	Hepatitis B	2	AST	U / L	Day 56	22/10/2010	41	Yes	0 - 40	H	NCS
S108	115	Hepatitis B	Hepatitis B	2	Alkaline Phosphatase	U / L	Day 56	22/10/2010	79	.	40 - 129		
S108	115	Hepatitis B	Hepatitis B	2	Direct Bilirubin	mg / dl	Day 56	22/10/2010	0.2	.	0.00 - 0.30		
S108	115	Hepatitis B	Hepatitis B	2	Gamma Glutamyl Transpeptidase	U / L	Day 56	22/10/2010	15	.	10 - 71		

Subject No.	Randomisation Number	Study Group	Dosage	Dose	Serum Chemistry Parameters	Unit	Day	Date of sample Collection	Results	Out of Range	Reference Range	Flag	Clinical Significance*
S108	115	Hepatitis B	Hepatitis B	2	Indirect Bilirubin	mg / dl	Day 56	22/10/2010	0.37	.	0.00 - 0.99		
S108	115	Hepatitis B	Hepatitis B	2	Total Bilirubin	mg / dl	Day 56	22/10/2010	0.57	.	0.00 - 0.99		
S108	115	Hepatitis B	Hepatitis B	2	Serum creatinine	mg / dl	Day 56	22/10/2010	0.8	.	0.7 - 1.2		
S108	115	Hepatitis B	Hepatitis B	2	Potassium	mEq / L	Day 56	22/10/2010	4.2	.	3.5 - 5.1		
S108	115	Hepatitis B	Hepatitis B	2	Random blood glucose	mg / dl	Day 56	22/10/2010	73	.	45-130		
S108	115	Hepatitis B	Hepatitis B	2	Sodium	mEq / L	Day 56	22/10/2010	140	.	136 - 145		
S108	115	Hepatitis B	Hepatitis B	3	ALT	U / L	Day 180	23/02/2011	22	.	0 - 41		
S108	115	Hepatitis B	Hepatitis B	3	AST	U / L	Day 180	23/02/2011	34	.	0 - 40		
S108	115	Hepatitis B	Hepatitis B	3	Alkaline Phosphatase	U / L	Day 180	23/02/2011	71	.	40 - 129		
S108	115	Hepatitis B	Hepatitis B	3	Direct Bilirubin	mg / dl	Day 180	23/02/2011	0.21	.	0.00 - 0.30		
S108	115	Hepatitis B	Hepatitis B	3	Gamma Glutamyl Transpeptidase	U / L	Day 180	23/02/2011	11	.	10 - 71		
S108	115	Hepatitis B	Hepatitis B	3	Indirect Bilirubin	mg / dl	Day 180	23/02/2011	0.36	.	0.00 - 0.99		
S108	115	Hepatitis B	Hepatitis B	3	Total Bilirubin	mg / dl	Day 180	23/02/2011	0.57	.	0.00 - 0.99		
S108	115	Hepatitis B	Hepatitis B	3	Serum creatinine	mg / dl	Day 180	23/02/2011	1.1	.	0.7 - 1.2		
S108	115	Hepatitis B	Hepatitis B	3	Potassium	mEq / L	Day 180	23/02/2011	4.3	.	3.5 - 5.1		
S108	115	Hepatitis B	Hepatitis B	3	Random blood glucose	mg / dl	Day 180	23/02/2011	72	.	45-130		
S108	115	Hepatitis B	Hepatitis B	3	Sodium	mEq / L	Day 180	23/02/2011	138	.	136 - 145		
S108	115	Hepatitis B	Hepatitis B	3	ALT	U / L	Day 208	23/03/2011	21	.	0 - 41		
S108	115	Hepatitis B	Hepatitis B	3	AST	U / L	Day 208	23/03/2011	24	.	0 - 40		
S108	115	Hepatitis B	Hepatitis B	3	Alkaline Phosphatase	U / L	Day 208	23/03/2011	72	.	40 - 129		
S108	115	Hepatitis B	Hepatitis B	3	Direct Bilirubin	mg / dl	Day 208	23/03/2011	0.3	.	0.00 - 0.30		
S108	115	Hepatitis B	Hepatitis B	3	Gamma Glutamyl Transpeptidase	U / L	Day 208	23/03/2011	17	.	10 - 71		
S108	115	Hepatitis B	Hepatitis B	3	Indirect Bilirubin	mg / dl	Day 208	23/03/2011	0.55	.	0.00 - 0.99		
S108	115	Hepatitis B	Hepatitis B	3	Total Bilirubin	mg / dl	Day 208	23/03/2011	0.85	.	0.00 - 0.99		
S108	115	Hepatitis B	Hepatitis B	3	Serum creatinine	mg / dl	Day 208	23/03/2011	1	.	0.7 - 1.2		
S108	115	Hepatitis B	Hepatitis B	3	Potassium	mEq / L	Day 208	23/03/2011	4.1	.	3.5 - 5.1		
S108	115	Hepatitis B	Hepatitis B	3	Random blood glucose	mg / dl	Day 208	23/03/2011	83	.	45-130		
S108	115	Hepatitis B	Hepatitis B	3	Sodium	mEq / L	Day 208	23/03/2011	137	.	136 - 145		
S108	115	Hepatitis B	Hepatitis B	3	ALT	U / L	Day 365	26/08/2011	19	.	0 - 41		

Subject No.	Randomisation Number	Study Group	Dosage	Dose	Serum Chemistry Parameters	Unit	Day	Date of sample Collection	Results	Out of Range	Reference Range	Flag	Clinical Significance*
S108	115	Hepatitis B	Hepatitis B	3	AST	U / L	Day 365	26/08/2011	25	.	0 - 40		
S108	115	Hepatitis B	Hepatitis B	3	Alkaline Phosphatase	U / L	Day 365	26/08/2011	82	.	40 - 129		
S108	115	Hepatitis B	Hepatitis B	3	Direct Bilirubin	mg / dl	Day 365	26/08/2011	0.14	.	0.00 - 0.30		
S108	115	Hepatitis B	Hepatitis B	3	Gamma Glutamyl Transpeptidase	U / L	Day 365	26/08/2011	12	.	10 - 71		
S108	115	Hepatitis B	Hepatitis B	3	Indirect Bilirubin	mg / dl	Day 365	26/08/2011	0.21	.	0.00 - 0.99		
S108	115	Hepatitis B	Hepatitis B	3	Total Bilirubin	mg / dl	Day 365	26/08/2011	0.35	.	0.00 - 0.99		
S108	115	Hepatitis B	Hepatitis B	3	Serum creatinine	mg / dl	Day 365	26/08/2011	1	.	0.7 - 1.2		
S108	115	Hepatitis B	Hepatitis B	3	Potassium	mEq / L	Day 365	26/08/2011	4.6	.	3.5 - 5.1		
S108	115	Hepatitis B	Hepatitis B	3	Random blood glucose	mg / dl	Day 365	26/08/2011	81	.	45-130		
S108	115	Hepatitis B	Hepatitis B	3	Sodium	mEq / L	Day 365	26/08/2011	139	.	136 - 145		
S118	114	JAIVAC-1	Dosage A		ALT	U / L	Day -14 to Day -1	17/08/2010	35	.	0 - 41		
S118	114	JAIVAC-1	Dosage A		AST	U / L	Day -14 to Day -1	17/08/2010	23	.	0 - 40		
S118	114	JAIVAC-1	Dosage A		Alkaline Phosphatase	U / L	Day -14 to Day -1	17/08/2010	46	.	40 - 129		
S118	114	JAIVAC-1	Dosage A		Direct Bilirubin	mg / dl	Day -14 to Day -1	17/08/2010	0.21	.	0.00 - 0.30		
S118	114	JAIVAC-1	Dosage A		Gamma Glutamyl Transpeptidase	U / L	Day -14 to Day -1	17/08/2010	16	.	10 - 71		
S118	114	JAIVAC-1	Dosage A		Indirect Bilirubin	mg / dl	Day -14 to Day -1	17/08/2010	0.33	.	0.00 - 0.99		
S118	114	JAIVAC-1	Dosage A		Total Bilirubin	mg / dl	Day -14 to Day -1	17/08/2010	0.54	.	0.00 - 0.99		
S118	114	JAIVAC-1	Dosage A		Serum creatinine	mg / dl	Day -14 to Day -1	17/08/2010	0.8	.	0.7 - 1.2		
S118	114	JAIVAC-1	Dosage A		Potassium	mEq / L	Day -14 to Day -1	17/08/2010	4.5	.	3.5 - 5.1		
S118	114	JAIVAC-1	Dosage A		Random blood glucose	mg / dl	Day -14 to Day -1	17/08/2010	77	.	45-130		
S118	114	JAIVAC-1	Dosage A		Sodium	mEq / L	Day -14 to Day -1	17/08/2010	139	.	136 - 145		
S118	114	JAIVAC-1	Dosage A	2	ALT	U / L	Day 28	24/09/2010	16	.	0 - 41		
S118	114	JAIVAC-1	Dosage A	2	AST	U / L	Day 28	24/09/2010	19	.	0 - 40		
S118	114	JAIVAC-1	Dosage A	2	Alkaline Phosphatase	U / L	Day 28	24/09/2010	44	.	40 - 129		
S118	114	JAIVAC-1	Dosage A	2	Direct Bilirubin	mg / dl	Day 28	24/09/2010	0.13	.	0.00 - 0.30		
S118	114	JAIVAC-1	Dosage A	2	Gamma Glutamyl Transpeptidase	U / L	Day 28	24/09/2010	10	.	10 - 71		
S118	114	JAIVAC-1	Dosage A	2	Indirect Bilirubin	mg / dl	Day 28	24/09/2010	0.38	.	0.00 - 0.99		
S118	114	JAIVAC-1	Dosage A	2	Total Bilirubin	mg / dl	Day 28	24/09/2010	0.51	.	0.00 - 0.99		
S118	114	JAIVAC-1	Dosage A	2	Serum creatinine	mg / dl	Day 28	24/09/2010	0.9	.	0.7 - 1.2		

Subject No.	Randomisation Number	Study Group	Dosage	Dose	Serum Chemistry Parameters	Unit	Day	Date of sample Collection	Results	Out of Range	Reference Range	Flag	Clinical Significance*
S118	114	JAIVAC-1	Dosage A	2	Potassium	mEq / L	Day 28	24/09/2010	4.6	.	3.5 - 5.1		
S118	114	JAIVAC-1	Dosage A	2	Random blood glucose	mg / dl	Day 28	24/09/2010	83	.	45-130		
S118	114	JAIVAC-1	Dosage A	2	Sodium	mEq / L	Day 28	24/09/2010	139	.	136 - 145		
S118	114	JAIVAC-1	Dosage A	2	ALT	U / L	Day 56	22/10/2010	21	.	0 - 41		
S118	114	JAIVAC-1	Dosage A	2	AST	U / L	Day 56	22/10/2010	23	.	0 - 40		
S118	114	JAIVAC-1	Dosage A	2	Alkaline Phosphatase	U / L	Day 56	22/10/2010	47	.	40 - 129		
S118	114	JAIVAC-1	Dosage A	2	Direct Bilirubin	mg / dl	Day 56	22/10/2010	0.25	.	0.00 - 0.30		
S118	114	JAIVAC-1	Dosage A	2	Gamma Glutamyl Transpeptidase	U / L	Day 56	22/10/2010	13	.	10 - 71		
S118	114	JAIVAC-1	Dosage A	2	Indirect Bilirubin	mg / dl	Day 56	22/10/2010	0.43	.	0.00 - 0.99		
S118	114	JAIVAC-1	Dosage A	2	Total Bilirubin	mg / dl	Day 56	22/10/2010	0.68	.	0.00 - 0.99		
S118	114	JAIVAC-1	Dosage A	2	Serum creatinine	mg / dl	Day 56	22/10/2010	0.9	.	0.7 - 1.2		
S118	114	JAIVAC-1	Dosage A	2	Potassium	mEq / L	Day 56	22/10/2010	4.1	.	3.5 - 5.1		
S118	114	JAIVAC-1	Dosage A	2	Random blood glucose	mg / dl	Day 56	22/10/2010	80	.	45-130		
S118	114	JAIVAC-1	Dosage A	2	Sodium	mEq / L	Day 56	22/10/2010	138	.	136 - 145		
S118	114	JAIVAC-1	Dosage A	3	ALT	U / L	Day 180	23/02/2011	24	.	0 - 41		
S118	114	JAIVAC-1	Dosage A	3	AST	U / L	Day 180	23/02/2011	20	.	0 - 40		
S118	114	JAIVAC-1	Dosage A	3	Alkaline Phosphatase	U / L	Day 180	23/02/2011	51	.	40 - 129		
S118	114	JAIVAC-1	Dosage A	3	Direct Bilirubin	mg / dl	Day 180	23/02/2011	0.16	.	0.00 - 0.30		
S118	114	JAIVAC-1	Dosage A	3	Gamma Glutamyl Transpeptidase	U / L	Day 180	23/02/2011	11	.	10 - 71		
S118	114	JAIVAC-1	Dosage A	3	Indirect Bilirubin	mg / dl	Day 180	23/02/2011	0.28	.	0.00 - 0.99		
S118	114	JAIVAC-1	Dosage A	3	Total Bilirubin	mg / dl	Day 180	23/02/2011	0.44	.	0.00 - 0.99		
S118	114	JAIVAC-1	Dosage A	3	Serum creatinine	mg / dl	Day 180	23/02/2011	1	.	0.7 - 1.2		
S118	114	JAIVAC-1	Dosage A	3	Potassium	mEq / L	Day 180	23/02/2011	4.7	.	3.5 - 5.1		
S118	114	JAIVAC-1	Dosage A	3	Random blood glucose	mg / dl	Day 180	23/02/2011	71	.	45-130		
S118	114	JAIVAC-1	Dosage A	3	Sodium	mEq / L	Day 180	23/02/2011	138	.	136 - 145		
S118	114	JAIVAC-1	Dosage A	3	ALT	U / L	Day 208	23/03/2011	41	.	0 - 41		
S118	114	JAIVAC-1	Dosage A	3	AST	U / L	Day 208	23/03/2011	25	.	0 - 40		
S118	114	JAIVAC-1	Dosage A	3	Alkaline Phosphatase	U / L	Day 208	23/03/2011	51	.	40 - 129		
S118	114	JAIVAC-1	Dosage A	3	Direct Bilirubin	mg / dl	Day 208	23/03/2011	0.22	.	0.00 - 0.30		

Subject No.	Randomisation Number	Study Group	Dosage	Dose	Serum Chemistry Parameters	Unit	Day	Date of sample Collection	Results	Out of Range	Reference Range	Flag	Clinical Significance*
S118	114	JAIVAC-1	Dosage A	3	Gamma Glutamyl Transpeptidase	U / L	Day 208	23/03/2011	16	.	10 - 71		
S118	114	JAIVAC-1	Dosage A	3	Indirect Bilirubin	mg / dl	Day 208	23/03/2011	0.33	.	0.00 - 0.99		
S118	114	JAIVAC-1	Dosage A	3	Total Bilirubin	mg / dl	Day 208	23/03/2011	0.55	.	0.00 - 0.99		
S118	114	JAIVAC-1	Dosage A	3	Serum creatinine	mg / dl	Day 208	23/03/2011	1.1	.	0.7 - 1.2		
S118	114	JAIVAC-1	Dosage A	3	Potassium	mEq / L	Day 208	23/03/2011	4.2	.	3.5 - 5.1		
S118	114	JAIVAC-1	Dosage A	3	Random blood glucose	mg / dl	Day 208	23/03/2011	96	.	45-130		
S118	114	JAIVAC-1	Dosage A	3	Sodium	mEq / L	Day 208	23/03/2011	138	.	136 - 145		
S118	114	JAIVAC-1	Dosage A	3	ALT	U / L	Day 365	26/08/2011	21	.	0 - 41		
S118	114	JAIVAC-1	Dosage A	3	AST	U / L	Day 365	26/08/2011	21	.	0 - 40		
S118	114	JAIVAC-1	Dosage A	3	Alkaline Phosphatase	U / L	Day 365	26/08/2011	47	.	40 - 129		
S118	114	JAIVAC-1	Dosage A	3	Direct Bilirubin	mg / dl	Day 365	26/08/2011	0.2	.	0.00 - 0.30		
S118	114	JAIVAC-1	Dosage A	3	Gamma Glutamyl Transpeptidase	U / L	Day 365	26/08/2011	15	.	10 - 71		
S118	114	JAIVAC-1	Dosage A	3	Indirect Bilirubin	mg / dl	Day 365	26/08/2011	0.28	.	0.00 - 0.99		
S118	114	JAIVAC-1	Dosage A	3	Total Bilirubin	mg / dl	Day 365	26/08/2011	0.48	.	0.00 - 0.99		
S118	114	JAIVAC-1	Dosage A	3	Serum creatinine	mg / dl	Day 365	26/08/2011	1	.	0.7 - 1.2		
S118	114	JAIVAC-1	Dosage A	3	Potassium	mEq / L	Day 365	26/08/2011	5.1	.	3.5 - 5.1		
S118	114	JAIVAC-1	Dosage A	3	Random blood glucose	mg / dl	Day 365	26/08/2011	76	.	45-130		
S118	114	JAIVAC-1	Dosage A	3	Sodium	mEq / L	Day 365	26/08/2011	139	.	136 - 145		
S119	107	Hepatitis B	Hepatitis B		ALT	U / L	Day -14 to Day -1	18/08/2010	17	.	0 - 41		
S119	107	Hepatitis B	Hepatitis B		AST	U / L	Day -14 to Day -1	18/08/2010	19	.	0 - 40		
S119	107	Hepatitis B	Hepatitis B		Alkaline Phosphatase	U / L	Day -14 to Day -1	18/08/2010	102	.	40 - 129		
S119	107	Hepatitis B	Hepatitis B		Direct Bilirubin	mg / dl	Day -14 to Day -1	18/08/2010	0.19	.	0.00 - 0.30		
S119	107	Hepatitis B	Hepatitis B		Gamma Glutamyl Transpeptidase	U / L	Day -14 to Day -1	18/08/2010	34	.	10 - 71		
S119	107	Hepatitis B	Hepatitis B		Indirect Bilirubin	mg / dl	Day -14 to Day -1	18/08/2010	0.58	.	0.00 - 0.99		
S119	107	Hepatitis B	Hepatitis B		Total Bilirubin	mg / dl	Day -14 to Day -1	18/08/2010	0.77	.	0.00 - 0.99		
S119	107	Hepatitis B	Hepatitis B		Serum creatinine	mg / dl	Day -14 to Day -1	18/08/2010	0.8	.	0.7 - 1.2		
S119	107	Hepatitis B	Hepatitis B		Potassium	mEq / L	Day -14 to Day -1	18/08/2010	4.2	.	3.5 - 5.1		
S119	107	Hepatitis B	Hepatitis B		Random blood glucose	mg / dl	Day -14 to Day -1	18/08/2010	130	.	45-130		
S119	107	Hepatitis B	Hepatitis B		Sodium	mEq / L	Day -14 to Day -1	18/08/2010	138	.	136 - 145		

Subject No.	Randomisation Number	Study Group	Dosage	Dose	Serum Chemistry Parameters	Unit	Day	Date of sample Collection	Results	Out of Range	Reference Range	Flag	Clinical Significance*
S119	107	Hepatitis B	Hepatitis B	2	ALT	U / L	Day 28	23/09/2010	14	.	0 - 41		
S119	107	Hepatitis B	Hepatitis B	2	AST	U / L	Day 28	23/09/2010	16	.	0 - 40		
S119	107	Hepatitis B	Hepatitis B	2	Alkaline Phosphatase	U / L	Day 28	23/09/2010	84	.	40 - 129		
S119	107	Hepatitis B	Hepatitis B	2	Direct Bilirubin	mg / dl	Day 28	23/09/2010	0.14	.	0.00 - 0.30		
S119	107	Hepatitis B	Hepatitis B	2	Gamma Glutamyl Transpeptidase	U / L	Day 28	23/09/2010	37	.	10 - 71		
S119	107	Hepatitis B	Hepatitis B	2	Indirect Bilirubin	mg / dl	Day 28	23/09/2010	0.54	.	0.00 - 0.99		
S119	107	Hepatitis B	Hepatitis B	2	Total Bilirubin	mg / dl	Day 28	23/09/2010	0.68	.	0.00 - 0.99		
S119	107	Hepatitis B	Hepatitis B	2	Serum creatinine	mg / dl	Day 28	23/09/2010	0.9	.	0.7 - 1.2		
S119	107	Hepatitis B	Hepatitis B	2	Potassium	mEq / L	Day 28	23/09/2010	3.7	.	3.5 - 5.1		
S119	107	Hepatitis B	Hepatitis B	2	Random blood glucose	mg / dl	Day 28	23/09/2010	130	.	45-130		
S119	107	Hepatitis B	Hepatitis B	2	Sodium	mEq / L	Day 28	23/09/2010	141	.	136 - 145		
S119	107	Hepatitis B	Hepatitis B	2	ALT	U / L	Day 56	21/10/2010	18	.	0 - 41		
S119	107	Hepatitis B	Hepatitis B	2	AST	U / L	Day 56	21/10/2010	20	.	0 - 40		
S119	107	Hepatitis B	Hepatitis B	2	Alkaline Phosphatase	U / L	Day 56	21/10/2010	103	.	40 - 129		
S119	107	Hepatitis B	Hepatitis B	2	Direct Bilirubin	mg / dl	Day 56	21/10/2010	0.14	.	0.00 - 0.30		
S119	107	Hepatitis B	Hepatitis B	2	Gamma Glutamyl Transpeptidase	U / L	Day 56	21/10/2010	48	.	10 - 71		
S119	107	Hepatitis B	Hepatitis B	2	Indirect Bilirubin	mg / dl	Day 56	21/10/2010	0.56	.	0.00 - 0.99		
S119	107	Hepatitis B	Hepatitis B	2	Total Bilirubin	mg / dl	Day 56	21/10/2010	0.7	.	0.00 - 0.99		
S119	107	Hepatitis B	Hepatitis B	2	Serum creatinine	mg / dl	Day 56	21/10/2010	0.9	.	0.7 - 1.2		
S119	107	Hepatitis B	Hepatitis B	2	Potassium	mEq / L	Day 56	21/10/2010	4.7	.	3.5 - 5.1		
S119	107	Hepatitis B	Hepatitis B	2	Random blood glucose	mg / dl	Day 56	21/10/2010	90	.	45-130		
S119	107	Hepatitis B	Hepatitis B	2	Sodium	mEq / L	Day 56	21/10/2010	140	.	136 - 145		
S119	107	Hepatitis B	Hepatitis B	3	ALT	U / L	Day 180	22/02/2011	17	.	0 - 41		
S119	107	Hepatitis B	Hepatitis B	3	AST	U / L	Day 180	22/02/2011	17	.	0 - 40		
S119	107	Hepatitis B	Hepatitis B	3	Alkaline Phosphatase	U / L	Day 180	22/02/2011	99	.	40 - 129		
S119	107	Hepatitis B	Hepatitis B	3	Direct Bilirubin	mg / dl	Day 180	22/02/2011	0.15	.	0.00 - 0.30		
S119	107	Hepatitis B	Hepatitis B	3	Gamma Glutamyl Transpeptidase	U / L	Day 180	22/02/2011	40	.	10 - 71		
S119	107	Hepatitis B	Hepatitis B	3	Indirect Bilirubin	mg / dl	Day 180	22/02/2011	0.61	.	0.00 - 0.99		
S119	107	Hepatitis B	Hepatitis B	3	Total Bilirubin	mg / dl	Day 180	22/02/2011	0.76	.	0.00 - 0.99		

Subject No.	Randomisation Number	Study Group	Dosage	Dose	Serum Chemistry Parameters	Unit	Day	Date of sample Collection	Results	Out of Range	Reference Range	Flag	Clinical Significance*
S119	107	Hepatitis B	Hepatitis B	3	Serum creatinine	mg / dl	Day 180	22/02/2011	0.9	.	0.7 - 1.2		
S119	107	Hepatitis B	Hepatitis B	3	Potassium	mEq / L	Day 180	22/02/2011	5.2	Yes	3.5 - 5.1	H	NCS
S119	107	Hepatitis B	Hepatitis B	3	Random blood glucose	mg / dl	Day 180	22/02/2011	81	.	45-130		
S119	107	Hepatitis B	Hepatitis B	3	Sodium	mEq / L	Day 180	22/02/2011	141	.	136 - 145		
S119	107	Hepatitis B	Hepatitis B	3	ALT	U / L	Day 208	22/03/2011	17	.	0 - 41		
S119	107	Hepatitis B	Hepatitis B	3	AST	U / L	Day 208	22/03/2011	15	.	0 - 40		
S119	107	Hepatitis B	Hepatitis B	3	Alkaline Phosphatase	U / L	Day 208	22/03/2011	112	.	40 - 129		
S119	107	Hepatitis B	Hepatitis B	3	Direct Bilirubin	mg / dl	Day 208	22/03/2011	0.26	.	0.00 - 0.30		
S119	107	Hepatitis B	Hepatitis B	3	Gamma Glutamyl Transpeptidase	U / L	Day 208	22/03/2011	46	.	10 - 71		
S119	107	Hepatitis B	Hepatitis B	3	Indirect Bilirubin	mg / dl	Day 208	22/03/2011	0.66	.	0.00 - 0.99		
S119	107	Hepatitis B	Hepatitis B	3	Total Bilirubin	mg / dl	Day 208	22/03/2011	0.92	.	0.00 - 0.99		
S119	107	Hepatitis B	Hepatitis B	3	Serum creatinine	mg / dl	Day 208	22/03/2011	1	.	0.7 - 1.2		
S119	107	Hepatitis B	Hepatitis B	3	Potassium	mEq / L	Day 208	22/03/2011	4	.	3.5 - 5.1		
S119	107	Hepatitis B	Hepatitis B	3	Random blood glucose	mg / dl	Day 208	22/03/2011	125	.	45-130		
S119	107	Hepatitis B	Hepatitis B	3	Sodium	mEq / L	Day 208	22/03/2011	141	.	136 - 145		
S119	107	Hepatitis B	Hepatitis B	3	ALT	U / L	Day 365	25/08/2011	19	.	0 - 41		
S119	107	Hepatitis B	Hepatitis B	3	AST	U / L	Day 365	25/08/2011	16	.	0 - 40		
S119	107	Hepatitis B	Hepatitis B	3	Alkaline Phosphatase	U / L	Day 365	25/08/2011	106	.	40 - 129		
S119	107	Hepatitis B	Hepatitis B	3	Direct Bilirubin	mg / dl	Day 365	25/08/2011	0.13	.	0.00 - 0.30		
S119	107	Hepatitis B	Hepatitis B	3	Gamma Glutamyl Transpeptidase	U / L	Day 365	25/08/2011	39	.	10 - 71		
S119	107	Hepatitis B	Hepatitis B	3	Indirect Bilirubin	mg / dl	Day 365	25/08/2011	0.33	.	0.00 - 0.99		
S119	107	Hepatitis B	Hepatitis B	3	Total Bilirubin	mg / dl	Day 365	25/08/2011	0.46	.	0.00 - 0.99		
S119	107	Hepatitis B	Hepatitis B	3	Serum creatinine	mg / dl	Day 365	25/08/2011	1	.	0.7 - 1.2		
S119	107	Hepatitis B	Hepatitis B	3	Potassium	mEq / L	Day 365	25/08/2011	4.6	.	3.5 - 5.1		
S119	107	Hepatitis B	Hepatitis B	3	Random blood glucose	mg / dl	Day 365	25/08/2011	84	.	45-130		
S119	107	Hepatitis B	Hepatitis B	3	Sodium	mEq / L	Day 365	25/08/2011	137	.	136 - 145		
S120	104	JAIVAC-1	Dosage A		ALT	U / L	Day -14 to Day -1	18/08/2010	12	.	0 - 41		
S120	104	JAIVAC-1	Dosage A		AST	U / L	Day -14 to Day -1	18/08/2010	16	.	0 - 40		
S120	104	JAIVAC-1	Dosage A		Alkaline Phosphatase	U / L	Day -14 to Day -1	18/08/2010	85	.	40 - 129		

Subject No.	Randomisation Number	Study Group	Dosage	Dose	Serum Chemistry Parameters	Unit	Day	Date of sample Collection	Results	Out of Range	Reference Range	Flag	Clinical Significance*
S120	104	JAIVAC-1	Dosage A		Direct Bilirubin	mg / dl	Day -14 to Day -1	18/08/2010	0.29	.	0.00 - 0.30		
S120	104	JAIVAC-1	Dosage A		Gamma Glutamyl Transpeptidase	U / L	Day -14 to Day -1	18/08/2010	12	.	10 - 71		
S120	104	JAIVAC-1	Dosage A		Indirect Bilirubin	mg / dl	Day -14 to Day -1	18/08/2010	0.64	.	0.00 - 0.99		
S120	104	JAIVAC-1	Dosage A		Total Bilirubin	mg / dl	Day -14 to Day -1	18/08/2010	0.93	.	0.00 - 0.99		
S120	104	JAIVAC-1	Dosage A		Serum creatinine	mg / dl	Day -14 to Day -1	18/08/2010	1	.	0.7 - 1.2		
S120	104	JAIVAC-1	Dosage A		Potassium	mEq / L	Day -14 to Day -1	18/08/2010	4.2	.	3.5 - 5.1		
S120	104	JAIVAC-1	Dosage A		Random blood glucose	mg / dl	Day -14 to Day -1	18/08/2010	75	.	45-130		
S120	104	JAIVAC-1	Dosage A		Sodium	mEq / L	Day -14 to Day -1	18/08/2010	142	.	136 - 145		
S120	104	JAIVAC-1	Dosage A	2	ALT	U / L	Day 28	23/09/2010	12	.	0 - 41		
S120	104	JAIVAC-1	Dosage A	2	AST	U / L	Day 28	23/09/2010	17	.	0 - 40		
S120	104	JAIVAC-1	Dosage A	2	Alkaline Phosphatase	U / L	Day 28	23/09/2010	88	.	40 - 129		
S120	104	JAIVAC-1	Dosage A	2	Direct Bilirubin	mg / dl	Day 28	23/09/2010	0.27	.	0.00 - 0.30		
S120	104	JAIVAC-1	Dosage A	2	Gamma Glutamyl Transpeptidase	U / L	Day 28	23/09/2010	14	.	10 - 71		
S120	104	JAIVAC-1	Dosage A	2	Indirect Bilirubin	mg / dl	Day 28	23/09/2010	0.72	.	0.00 - 0.99		
S120	104	JAIVAC-1	Dosage A	2	Total Bilirubin	mg / dl	Day 28	23/09/2010	0.99	.	0.00 - 0.99		
S120	104	JAIVAC-1	Dosage A	2	Serum creatinine	mg / dl	Day 28	23/09/2010	1	.	0.7 - 1.2		
S120	104	JAIVAC-1	Dosage A	2	Potassium	mEq / L	Day 28	23/09/2010	4.2	.	3.5 - 5.1		
S120	104	JAIVAC-1	Dosage A	2	Random blood glucose	mg / dl	Day 28	23/09/2010	92	.	45-130		
S120	104	JAIVAC-1	Dosage A	2	Sodium	mEq / L	Day 28	23/09/2010	141	.	136 - 145		
S120	104	JAIVAC-1	Dosage A	2	ALT	U / L	Day 56	21/10/2010	13	.	0 - 41		
S120	104	JAIVAC-1	Dosage A	2	AST	U / L	Day 56	21/10/2010	19	.	0 - 40		
S120	104	JAIVAC-1	Dosage A	2	Alkaline Phosphatase	U / L	Day 56	21/10/2010	91	.	40 - 129		
S120	104	JAIVAC-1	Dosage A	2	Direct Bilirubin	mg / dl	Day 56	21/10/2010	0.28	.	0.00 - 0.30		
S120	104	JAIVAC-1	Dosage A	2	Gamma Glutamyl Transpeptidase	U / L	Day 56	21/10/2010	12	.	10 - 71		
S120	104	JAIVAC-1	Dosage A	2	Indirect Bilirubin	mg / dl	Day 56	21/10/2010	0.68	.	0.00 - 0.99		
S120	104	JAIVAC-1	Dosage A	2	Total Bilirubin	mg / dl	Day 56	21/10/2010	0.96	.	0.00 - 0.99		
S120	104	JAIVAC-1	Dosage A	2	Serum creatinine	mg / dl	Day 56	21/10/2010	1	.	0.7 - 1.2		
S120	104	JAIVAC-1	Dosage A	2	Potassium	mEq / L	Day 56	21/10/2010	3.8	.	3.5 - 5.1		
S120	104	JAIVAC-1	Dosage A	2	Random blood glucose	mg / dl	Day 56	21/10/2010	84	.	45-130		

Subject No.	Randomisation Number	Study Group	Dosage	Dose	Serum Chemistry Parameters	Unit	Day	Date of sample Collection	Results	Out of Range	Reference Range	Flag	Clinical Significance*
S120	104	JAIVAC-1	Dosage A	2	Sodium	mEq / L	Day 56	21/10/2010	142	.	136 - 145		
S120	104	JAIVAC-1	Dosage A	3	ALT	U / L	Day 180	22/02/2011	13	.	0 - 41		
S120	104	JAIVAC-1	Dosage A	3	AST	U / L	Day 180	22/02/2011	17	.	0 - 40		
S120	104	JAIVAC-1	Dosage A	3	Alkaline Phosphatase	U / L	Day 180	22/02/2011	97	.	40 - 129		
S120	104	JAIVAC-1	Dosage A	3	Direct Bilirubin	mg / dl	Day 180	22/02/2011	0.23	.	0.00 - 0.30		
S120	104	JAIVAC-1	Dosage A	3	Gamma Glutamyl Transpeptidase	U / L	Day 180	22/02/2011	15	.	10 - 71		
S120	104	JAIVAC-1	Dosage A	3	Indirect Bilirubin	mg / dl	Day 180	22/02/2011	0.62	.	0.00 - 0.99		
S120	104	JAIVAC-1	Dosage A	3	Total Bilirubin	mg / dl	Day 180	22/02/2011	0.85	.	0.00 - 0.99		
S120	104	JAIVAC-1	Dosage A	3	Serum creatinine	mg / dl	Day 180	22/02/2011	1.1	.	0.7 - 1.2		
S120	104	JAIVAC-1	Dosage A	3	Potassium	mEq / L	Day 180	22/02/2011	4.3	.	3.5 - 5.1		
S120	104	JAIVAC-1	Dosage A	3	Random blood glucose	mg / dl	Day 180	22/02/2011	73	.	45-130		
S120	104	JAIVAC-1	Dosage A	3	Sodium	mEq / L	Day 180	22/02/2011	142	.	136 - 145		
S120	104	JAIVAC-1	Dosage A	3	ALT	U / L	Day 208	22/03/2011	18	.	0 - 41		
S120	104	JAIVAC-1	Dosage A	3	AST	U / L	Day 208	22/03/2011	18	.	0 - 40		
S120	104	JAIVAC-1	Dosage A	3	Alkaline Phosphatase	U / L	Day 208	22/03/2011	91	.	40 - 129		
S120	104	JAIVAC-1	Dosage A	3	Direct Bilirubin	mg / dl	Day 208	22/03/2011	0.41	Yes	0.00 - 0.30	H	CS
S120	104	JAIVAC-1	Dosage A	3	Gamma Glutamyl Transpeptidase	U / L	Day 208	22/03/2011	19	.	10 - 71		
S120	104	JAIVAC-1	Dosage A	3	Indirect Bilirubin	mg / dl	Day 208	22/03/2011	0.97	.	0.00 - 0.99		
S120	104	JAIVAC-1	Dosage A	3	Total Bilirubin	mg / dl	Day 208	22/03/2011	1.38	Yes	0.00 - 0.99	H	CS
S120	104	JAIVAC-1	Dosage A	3	Serum creatinine	mg / dl	Day 208	22/03/2011	1.2	.	0.7 - 1.2		
S120	104	JAIVAC-1	Dosage A	3	Potassium	mEq / L	Day 208	22/03/2011	4.3	.	3.5 - 5.1		
S120	104	JAIVAC-1	Dosage A	3	Random blood glucose	mg / dl	Day 208	22/03/2011	122	.	45-130		
S120	104	JAIVAC-1	Dosage A	3	Sodium	mEq / L	Day 208	22/03/2011	139	.	136 - 145		
S120	104	JAIVAC-1	Dosage A	3	ALT	U / L	Day 365	25/08/2011	16	.	0 - 41		
S120	104	JAIVAC-1	Dosage A	3	AST	U / L	Day 365	25/08/2011	18	.	0 - 40		
S120	104	JAIVAC-1	Dosage A	3	Alkaline Phosphatase	U / L	Day 365	25/08/2011	91	.	40 - 129		
S120	104	JAIVAC-1	Dosage A	3	Direct Bilirubin	mg / dl	Day 365	25/08/2011	0.24	.	0.00 - 0.30		
S120	104	JAIVAC-1	Dosage A	3	Gamma Glutamyl Transpeptidase	U / L	Day 365	25/08/2011	15	.	10 - 71		
S120	104	JAIVAC-1	Dosage A	3	Indirect Bilirubin	mg / dl	Day 365	25/08/2011	0.47	.	0.00 - 0.99		

Subject No.	Randomisation Number	Study Group	Dosage	Dose	Serum Chemistry Parameters	Unit	Day	Date of sample Collection	Results	Out of Range	Reference Range	Flag	Clinical Significance*
S120	104	JAIVAC-1	Dosage A	3	Total Bilirubin	mg / dl	Day 365	25/08/2011	0.71	.	0.00 - 0.99		
S120	104	JAIVAC-1	Dosage A	3	Serum creatinine	mg / dl	Day 365	25/08/2011	1.2	.	0.7 - 1.2		
S120	104	JAIVAC-1	Dosage A	3	Potassium	mEq / L	Day 365	25/08/2011	4.1	.	3.5 - 5.1		
S120	104	JAIVAC-1	Dosage A	3	Random blood glucose	mg / dl	Day 365	25/08/2011	73	.	45-130		
S120	104	JAIVAC-1	Dosage A	3	Sodium	mEq / L	Day 365	25/08/2011	140	.	136 - 145		
S124	106	Hepatitis B	Hepatitis B		ALT	U / L	Day -14 to Day -1	18/08/2010	10	.	0 - 41		
S124	106	Hepatitis B	Hepatitis B		AST	U / L	Day -14 to Day -1	18/08/2010	9	.	0 - 40		
S124	106	Hepatitis B	Hepatitis B		Alkaline Phosphatase	U / L	Day -14 to Day -1	18/08/2010	87	.	40 - 129		
S124	106	Hepatitis B	Hepatitis B		Direct Bilirubin	mg / dl	Day -14 to Day -1	18/08/2010	0.35	.	0.00 - 0.30	H	
S124	106	Hepatitis B	Hepatitis B		Gamma Glutamyl Transpeptidase	U / L	Day -14 to Day -1	18/08/2010	19	.	10 - 71		
S124	106	Hepatitis B	Hepatitis B		Indirect Bilirubin	mg / dl	Day -14 to Day -1	18/08/2010	0.63	.	0.00 - 0.99		
S124	106	Hepatitis B	Hepatitis B		Total Bilirubin	mg / dl	Day -14 to Day -1	18/08/2010	0.98	.	0.00 - 0.99		
S124	106	Hepatitis B	Hepatitis B		Serum creatinine	mg / dl	Day -14 to Day -1	18/08/2010	1	.	0.7 - 1.2		
S124	106	Hepatitis B	Hepatitis B		Potassium	mEq / L	Day -14 to Day -1	18/08/2010	4.6	.	3.5 - 5.1		
S124	106	Hepatitis B	Hepatitis B		Random blood glucose	mg / dl	Day -14 to Day -1	18/08/2010	73	.	45-130		
S124	106	Hepatitis B	Hepatitis B		Sodium	mEq / L	Day -14 to Day -1	18/08/2010	141	.	136 - 145		
S124	106	Hepatitis B	Hepatitis B	2	ALT	U / L	Day 28	23/09/2010	8	.	0 - 41		
S124	106	Hepatitis B	Hepatitis B	2	AST	U / L	Day 28	23/09/2010	12	.	0 - 40		
S124	106	Hepatitis B	Hepatitis B	2	Alkaline Phosphatase	U / L	Day 28	23/09/2010	93	.	40 - 129		
S124	106	Hepatitis B	Hepatitis B	2	Direct Bilirubin	mg / dl	Day 28	23/09/2010	0.32	Yes	0.00 - 0.30	H	NCS
S124	106	Hepatitis B	Hepatitis B	2	Gamma Glutamyl Transpeptidase	U / L	Day 28	23/09/2010	18	.	10 - 71		
S124	106	Hepatitis B	Hepatitis B	2	Indirect Bilirubin	mg / dl	Day 28	23/09/2010	0.63	.	0.00 - 0.99		
S124	106	Hepatitis B	Hepatitis B	2	Total Bilirubin	mg / dl	Day 28	23/09/2010	0.95	.	0.00 - 0.99		
S124	106	Hepatitis B	Hepatitis B	2	Serum creatinine	mg / dl	Day 28	23/09/2010	1.1	.	0.7 - 1.2		
S124	106	Hepatitis B	Hepatitis B	2	Potassium	mEq / L	Day 28	23/09/2010	4.4	.	3.5 - 5.1		
S124	106	Hepatitis B	Hepatitis B	2	Random blood glucose	mg / dl	Day 28	23/09/2010	77	.	45-130		
S124	106	Hepatitis B	Hepatitis B	2	Sodium	mEq / L	Day 28	23/09/2010	139	.	136 - 145		
S124	106	Hepatitis B	Hepatitis B	2	ALT	U / L	Day 56	21/10/2010	11	.	0 - 41		
S124	106	Hepatitis B	Hepatitis B	2	AST	U / L	Day 56	21/10/2010	13	.	0 - 40		

Subject No.	Randomisation Number	Study Group	Dosage	Dose	Serum Chemistry Parameters	Unit	Day	Date of sample Collection	Results	Out of Range	Reference Range	Flag	Clinical Significance*
S124	106	Hepatitis B	Hepatitis B	2	Alkaline Phosphatase	U / L	Day 56	21/10/2010	83	.	40 - 129		
S124	106	Hepatitis B	Hepatitis B	2	Direct Bilirubin	mg / dl	Day 56	21/10/2010	0.38	Yes	0.00 - 0.30	H	NCS
S124	106	Hepatitis B	Hepatitis B	2	Gamma Glutamyl Transpeptidase	U / L	Day 56	21/10/2010	19	.	10 - 71		
S124	106	Hepatitis B	Hepatitis B	2	Indirect Bilirubin	mg / dl	Day 56	21/10/2010	0.8	.	0.00 - 0.99		
S124	106	Hepatitis B	Hepatitis B	2	Total Bilirubin	mg / dl	Day 56	21/10/2010	1.18	Yes	0.00 - 0.99	H	NCS
S124	106	Hepatitis B	Hepatitis B	2	Serum creatinine	mg / dl	Day 56	21/10/2010	1	.	0.7 - 1.2		
S124	106	Hepatitis B	Hepatitis B	2	Potassium	mEq / L	Day 56	21/10/2010	4.5	.	3.5 - 5.1		
S124	106	Hepatitis B	Hepatitis B	2	Random blood glucose	mg / dl	Day 56	21/10/2010	100	.	45-130		
S124	106	Hepatitis B	Hepatitis B	2	Sodium	mEq / L	Day 56	21/10/2010	139	.	136 - 145		
S124	106	Hepatitis B	Hepatitis B	3	ALT	U / L	Day 180	22/02/2011	16	.	0 - 41		
S124	106	Hepatitis B	Hepatitis B	3	AST	U / L	Day 180	22/02/2011	14	.	0 - 40		
S124	106	Hepatitis B	Hepatitis B	3	Alkaline Phosphatase	U / L	Day 180	22/02/2011	93	.	40 - 129		
S124	106	Hepatitis B	Hepatitis B	3	Direct Bilirubin	mg / dl	Day 180	22/02/2011	0.31	Yes	0.00 - 0.30	H	NCS
S124	106	Hepatitis B	Hepatitis B	3	Gamma Glutamyl Transpeptidase	U / L	Day 180	22/02/2011	22	.	10 - 71		
S124	106	Hepatitis B	Hepatitis B	3	Indirect Bilirubin	mg / dl	Day 180	22/02/2011	0.74	.	0.00 - 0.99		
S124	106	Hepatitis B	Hepatitis B	3	Total Bilirubin	mg / dl	Day 180	22/02/2011	1.05	Yes	0.00 - 0.99	H	NCS
S124	106	Hepatitis B	Hepatitis B	3	Serum creatinine	mg / dl	Day 180	22/02/2011	1.1	.	0.7 - 1.2		
S124	106	Hepatitis B	Hepatitis B	3	Potassium	mEq / L	Day 180	22/02/2011	4.3	.	3.5 - 5.1		
S124	106	Hepatitis B	Hepatitis B	3	Random blood glucose	mg / dl	Day 180	22/02/2011	84	.	45-130		
S124	106	Hepatitis B	Hepatitis B	3	Sodium	mEq / L	Day 180	22/02/2011	140	.	136 - 145		
S124	106	Hepatitis B	Hepatitis B	3	ALT	U / L	Day 208	22/03/2011	13	.	0 - 41		
S124	106	Hepatitis B	Hepatitis B	3	AST	U / L	Day 208	22/03/2011	11	.	0 - 40		
S124	106	Hepatitis B	Hepatitis B	3	Alkaline Phosphatase	U / L	Day 208	22/03/2011	102	.	40 - 129		
S124	106	Hepatitis B	Hepatitis B	3	Direct Bilirubin	mg / dl	Day 208	22/03/2011	0.26	.	0.00 - 0.30		
S124	106	Hepatitis B	Hepatitis B	3	Gamma Glutamyl Transpeptidase	U / L	Day 208	22/03/2011	28	.	10 - 71		
S124	106	Hepatitis B	Hepatitis B	3	Indirect Bilirubin	mg / dl	Day 208	22/03/2011	0.44	.	0.00 - 0.99		
S124	106	Hepatitis B	Hepatitis B	3	Total Bilirubin	mg / dl	Day 208	22/03/2011	0.7	.	0.00 - 0.99		
S124	106	Hepatitis B	Hepatitis B	3	Serum creatinine	mg / dl	Day 208	22/03/2011	1.1	.	0.7 - 1.2		
S124	106	Hepatitis B	Hepatitis B	3	Potassium	mEq / L	Day 208	22/03/2011	4.4	.	3.5 - 5.1		

Subject No.	Randomisation Number	Study Group	Dosage	Dose	Serum Chemistry Parameters	Unit	Day	Date of sample Collection	Results	Out of Range	Reference Range	Flag	Clinical Significance*
S124	106	Hepatitis B	Hepatitis B	3	Random blood glucose	mg / dl	Day 208	22/03/2011	81	.	45-130		
S124	106	Hepatitis B	Hepatitis B	3	Sodium	mEq / L	Day 208	22/03/2011	138	.	136 - 145		
S124	106	Hepatitis B	Hepatitis B	3	ALT	U / L	Day 365	25/08/2011	19	.	0 - 41		
S124	106	Hepatitis B	Hepatitis B	3	AST	U / L	Day 365	25/08/2011	13	.	0 - 40		
S124	106	Hepatitis B	Hepatitis B	3	Alkaline Phosphatase	U / L	Day 365	25/08/2011	93	.	40 - 129		
S124	106	Hepatitis B	Hepatitis B	3	Direct Bilirubin	mg / dl	Day 365	25/08/2011	0.23	.	0.00 - 0.30		
S124	106	Hepatitis B	Hepatitis B	3	Gamma Glutamyl Transpeptidase	U / L	Day 365	25/08/2011	21	.	10 - 71		
S124	106	Hepatitis B	Hepatitis B	3	Indirect Bilirubin	mg / dl	Day 365	25/08/2011	0.35	.	0.00 - 0.99		
S124	106	Hepatitis B	Hepatitis B	3	Total Bilirubin	mg / dl	Day 365	25/08/2011	0.58	.	0.00 - 0.99		
S124	106	Hepatitis B	Hepatitis B	3	Serum creatinine	mg / dl	Day 365	25/08/2011	1.1	.	0.7 - 1.2		
S124	106	Hepatitis B	Hepatitis B	3	Potassium	mEq / L	Day 365	25/08/2011	4.4	.	3.5 - 5.1		
S124	106	Hepatitis B	Hepatitis B	3	Random blood glucose	mg / dl	Day 365	25/08/2011	85	.	45-130		
S124	106	Hepatitis B	Hepatitis B	3	Sodium	mEq / L	Day 365	25/08/2011	138	.	136 - 145		
S125	102	JAIVAC-1	Dosage A		ALT	U / L	Day -14 to Day -1	18/08/2010	28	.	0 - 41		
S125	102	JAIVAC-1	Dosage A		AST	U / L	Day -14 to Day -1	18/08/2010	24	.	0 - 40		
S125	102	JAIVAC-1	Dosage A		Alkaline Phosphatase	U / L	Day -14 to Day -1	18/08/2010	52	.	40 - 129		
S125	102	JAIVAC-1	Dosage A		Direct Bilirubin	mg / dl	Day -14 to Day -1	18/08/2010	0.12	.	0.00 - 0.30		
S125	102	JAIVAC-1	Dosage A		Gamma Glutamyl Transpeptidase	U / L	Day -14 to Day -1	18/08/2010	18	.	10 - 71		
S125	102	JAIVAC-1	Dosage A		Indirect Bilirubin	mg / dl	Day -14 to Day -1	18/08/2010	0.34	.	0.00 - 0.99		
S125	102	JAIVAC-1	Dosage A		Total Bilirubin	mg / dl	Day -14 to Day -1	18/08/2010	0.46	.	0.00 - 0.99		
S125	102	JAIVAC-1	Dosage A		Serum creatinine	mg / dl	Day -14 to Day -1	18/08/2010	0.9	.	0.7 - 1.2		
S125	102	JAIVAC-1	Dosage A		Potassium	mEq / L	Day -14 to Day -1	18/08/2010	4	.	3.5 - 5.1		
S125	102	JAIVAC-1	Dosage A		Random blood glucose	mg / dl	Day -14 to Day -1	18/08/2010	90	.	45-130		
S125	102	JAIVAC-1	Dosage A		Sodium	mEq / L	Day -14 to Day -1	18/08/2010	140	.	136 - 145		
S125	102	JAIVAC-1	Dosage A	2	ALT	U / L	Day 28	21/09/2010	27	.	0 - 41		
S125	102	JAIVAC-1	Dosage A	2	AST	U / L	Day 28	21/09/2010	23	.	0 - 40		
S125	102	JAIVAC-1	Dosage A	2	Alkaline Phosphatase	U / L	Day 28	21/09/2010	60	.	40 - 129		
S125	102	JAIVAC-1	Dosage A	2	Direct Bilirubin	mg / dl	Day 28	21/09/2010	0.24	.	0.00 - 0.30		
S125	102	JAIVAC-1	Dosage A	2	Gamma Glutamyl Transpeptidase	U / L	Day 28	21/09/2010	19	.	10 - 71		

Subject No.	Randomisation Number	Study Group	Dosage	Dose	Serum Chemistry Parameters	Unit	Day	Date of sample Collection	Results	Out of Range	Reference Range	Flag	Clinical Significance*
S125	102	JAIVAC-1	Dosage A	2	Indirect Bilirubin	mg / dl	Day 28	21/09/2010	0.55	.	0.00 - 0.99		
S125	102	JAIVAC-1	Dosage A	2	Total Bilirubin	mg / dl	Day 28	21/09/2010	0.79	.	0.00 - 0.99		
S125	102	JAIVAC-1	Dosage A	2	Serum creatinine	mg / dl	Day 28	21/09/2010	1.1	.	0.7 - 1.2		
S125	102	JAIVAC-1	Dosage A	2	Potassium	mEq / L	Day 28	21/09/2010	4	.	3.5 - 5.1		
S125	102	JAIVAC-1	Dosage A	2	Random blood glucose	mg / dl	Day 28	21/09/2010	118	.	45-130		
S125	102	JAIVAC-1	Dosage A	2	Sodium	mEq / L	Day 28	21/09/2010	138	.	136 - 145		
S125	102	JAIVAC-1	Dosage A	2	ALT	U / L	Day 56	19/10/2010	24	.	0 - 41		
S125	102	JAIVAC-1	Dosage A	2	AST	U / L	Day 56	19/10/2010	21	.	0 - 40		
S125	102	JAIVAC-1	Dosage A	2	Alkaline Phosphatase	U / L	Day 56	19/10/2010	54	.	40 - 129		
S125	102	JAIVAC-1	Dosage A	2	Direct Bilirubin	mg / dl	Day 56	19/10/2010	0.16	.	0.00 - 0.30		
S125	102	JAIVAC-1	Dosage A	2	Gamma Glutamyl Transpeptidase	U / L	Day 56	19/10/2010	18	.	10 - 71		
S125	102	JAIVAC-1	Dosage A	2	Indirect Bilirubin	mg / dl	Day 56	19/10/2010	0.42	.	0.00 - 0.99		
S125	102	JAIVAC-1	Dosage A	2	Total Bilirubin	mg / dl	Day 56	19/10/2010	0.58	.	0.00 - 0.99		
S125	102	JAIVAC-1	Dosage A	2	Serum creatinine	mg / dl	Day 56	19/10/2010	1	.	0.7 - 1.2		
S125	102	JAIVAC-1	Dosage A	2	Potassium	mEq / L	Day 56	19/10/2010	4	.	3.5 - 5.1		
S125	102	JAIVAC-1	Dosage A	2	Random blood glucose	mg / dl	Day 56	19/10/2010	93	.	45-130		
S125	102	JAIVAC-1	Dosage A	2	Sodium	mEq / L	Day 56	19/10/2010	138	.	136 - 145		
S125	102	JAIVAC-1	Dosage A	3	ALT	U / L	Day 180	21/02/2011	28	.	0 - 41		
S125	102	JAIVAC-1	Dosage A	3	AST	U / L	Day 180	21/02/2011	25	.	0 - 40		
S125	102	JAIVAC-1	Dosage A	3	Alkaline Phosphatase	U / L	Day 180	21/02/2011	55	.	40 - 129		
S125	102	JAIVAC-1	Dosage A	3	Direct Bilirubin	mg / dl	Day 180	21/02/2011	0.14	.	0.00 - 0.30		
S125	102	JAIVAC-1	Dosage A	3	Gamma Glutamyl Transpeptidase	U / L	Day 180	21/02/2011	17	.	10 - 71		
S125	102	JAIVAC-1	Dosage A	3	Indirect Bilirubin	mg / dl	Day 180	21/02/2011	0.52	.	0.00 - 0.99		
S125	102	JAIVAC-1	Dosage A	3	Total Bilirubin	mg / dl	Day 180	21/02/2011	0.66	.	0.00 - 0.99		
S125	102	JAIVAC-1	Dosage A	3	Serum creatinine	mg / dl	Day 180	21/02/2011	1.1	.	0.7 - 1.2		
S125	102	JAIVAC-1	Dosage A	3	Potassium	mEq / L	Day 180	21/02/2011	4.2	.	3.5 - 5.1		
S125	102	JAIVAC-1	Dosage A	3	Random blood glucose	mg / dl	Day 180	21/02/2011	149	Yes	45-130	H	CS
S125	102	JAIVAC-1	Dosage A	3	Sodium	mEq / L	Day 180	21/02/2011	139	.	136 - 145		
S125	102	JAIVAC-1	Dosage A	3	ALT	U / L	Day 208	21/03/2011	32	.	0 - 41		

Subject No.	Randomisation Number	Study Group	Dosage	Dose	Serum Chemistry Parameters	Unit	Day	Date of sample Collection	Results	Out of Range	Reference Range	Flag	Clinical Significance*
S125	102	JAIVAC-1	Dosage A	3	AST	U / L	Day 208	21/03/2011	28	.	0 - 40		
S125	102	JAIVAC-1	Dosage A	3	Alkaline Phosphatase	U / L	Day 208	21/03/2011	65	.	40 - 129		
S125	102	JAIVAC-1	Dosage A	3	Direct Bilirubin	mg / dl	Day 208	21/03/2011	0.22	.	0.00 - 0.30		
S125	102	JAIVAC-1	Dosage A	3	Gamma Glutamyl Transpeptidase	U / L	Day 208	21/03/2011	21	.	10 - 71		
S125	102	JAIVAC-1	Dosage A	3	Indirect Bilirubin	mg / dl	Day 208	21/03/2011	0.52	.	0.00 - 0.99		
S125	102	JAIVAC-1	Dosage A	3	Total Bilirubin	mg / dl	Day 208	21/03/2011	0.74	.	0.00 - 0.99		
S125	102	JAIVAC-1	Dosage A	3	Serum creatinine	mg / dl	Day 208	21/03/2011	1.2	.	0.7 - 1.2		
S125	102	JAIVAC-1	Dosage A	3	Potassium	mEq / L	Day 208	21/03/2011	4.4	.	3.5 - 5.1		
S125	102	JAIVAC-1	Dosage A	3	Random blood glucose	mg / dl	Day 208	21/03/2011	78	.	45-130		
S125	102	JAIVAC-1	Dosage A	3	Sodium	mEq / L	Day 208	21/03/2011	138	.	136 - 145		
S125	102	JAIVAC-1	Dosage A	3	ALT	U / L	Day 365	26/08/2011	37	.	0 - 41		
S125	102	JAIVAC-1	Dosage A	3	AST	U / L	Day 365	26/08/2011	28	.	0 - 40		
S125	102	JAIVAC-1	Dosage A	3	Alkaline Phosphatase	U / L	Day 365	26/08/2011	55	.	40 - 129		
S125	102	JAIVAC-1	Dosage A	3	Direct Bilirubin	mg / dl	Day 365	26/08/2011	0.31	Yes	0.00 - 0.30	H	CS
S125	102	JAIVAC-1	Dosage A	3	Gamma Glutamyl Transpeptidase	U / L	Day 365	26/08/2011	27	.	10 - 71		
S125	102	JAIVAC-1	Dosage A	3	Indirect Bilirubin	mg / dl	Day 365	26/08/2011	0.69	.	0.00 - 0.99		
S125	102	JAIVAC-1	Dosage A	3	Total Bilirubin	mg / dl	Day 365	26/08/2011	1	Yes	0.00 - 0.99	H	CS
S125	102	JAIVAC-1	Dosage A	3	Serum creatinine	mg / dl	Day 365	26/08/2011	1.1	.	0.7 - 1.2		
S125	102	JAIVAC-1	Dosage A	3	Potassium	mEq / L	Day 365	26/08/2011	4.8	.	3.5 - 5.1		
S125	102	JAIVAC-1	Dosage A	3	Random blood glucose	mg / dl	Day 365	26/08/2011	160	Yes	45-130	H	CS
S125	102	JAIVAC-1	Dosage A	3	Sodium	mEq / L	Day 365	26/08/2011	138	.	136 - 145		
S130	105	JAIVAC-1	Dosage A		ALT	U / L	Day -14 to Day -1	17/08/2010	16	.	0 - 41		
S130	105	JAIVAC-1	Dosage A		AST	U / L	Day -14 to Day -1	17/08/2010	19	.	0 - 40		
S130	105	JAIVAC-1	Dosage A		Alkaline Phosphatase	U / L	Day -14 to Day -1	17/08/2010	61	.	40 - 129		
S130	105	JAIVAC-1	Dosage A		Direct Bilirubin	mg / dl	Day -14 to Day -1	17/08/2010	0.25	.	0.00 - 0.30		
S130	105	JAIVAC-1	Dosage A		Gamma Glutamyl Transpeptidase	U / L	Day -14 to Day -1	17/08/2010	8	.	10 - 71	L	
S130	105	JAIVAC-1	Dosage A		Indirect Bilirubin	mg / dl	Day -14 to Day -1	17/08/2010	0.4	.	0.00 - 0.99		
S130	105	JAIVAC-1	Dosage A		Total Bilirubin	mg / dl	Day -14 to Day -1	17/08/2010	0.65	.	0.00 - 0.99		
S130	105	JAIVAC-1	Dosage A		Serum creatinine	mg / dl	Day -14 to Day -1	17/08/2010	0.8	.	0.7 - 1.2		

Subject No.	Randomisation Number	Study Group	Dosage	Dose	Serum Chemistry Parameters	Unit	Day	Date of sample Collection	Results	Out of Range	Reference Range	Flag	Clinical Significance*
S130	105	JAIVAC-1	Dosage A		Potassium	mEq / L	Day -14 to Day -1	17/08/2010	4.3	.	3.5 - 5.1		
S130	105	JAIVAC-1	Dosage A		Random blood glucose	mg / dl	Day -14 to Day -1	17/08/2010	81	.	45-130		
S130	105	JAIVAC-1	Dosage A		Sodium	mEq / L	Day -14 to Day -1	17/08/2010	141	.	136 - 145		
S130	105	JAIVAC-1	Dosage A	2	ALT	U / L	Day 28	23/09/2010	40	.	0 - 41		
S130	105	JAIVAC-1	Dosage A	2	AST	U / L	Day 28	23/09/2010	27	.	0 - 40		
S130	105	JAIVAC-1	Dosage A	2	Alkaline Phosphatase	U / L	Day 28	23/09/2010	50	.	40 - 129		
S130	105	JAIVAC-1	Dosage A	2	Direct Bilirubin	mg / dl	Day 28	23/09/2010	0.16	.	0.00 - 0.30		
S130	105	JAIVAC-1	Dosage A	2	Gamma Glutamyl Transpeptidase	U / L	Day 28	23/09/2010	10	.	10 - 71		
S130	105	JAIVAC-1	Dosage A	2	Indirect Bilirubin	mg / dl	Day 28	23/09/2010	0.35	.	0.00 - 0.99		
S130	105	JAIVAC-1	Dosage A	2	Total Bilirubin	mg / dl	Day 28	23/09/2010	0.51	.	0.00 - 0.99		
S130	105	JAIVAC-1	Dosage A	2	Serum creatinine	mg / dl	Day 28	23/09/2010	0.8	.	0.7 - 1.2		
S130	105	JAIVAC-1	Dosage A	2	Potassium	mEq / L	Day 28	23/09/2010	4.6	.	3.5 - 5.1		
S130	105	JAIVAC-1	Dosage A	2	Random blood glucose	mg / dl	Day 28	23/09/2010	71	.	45-130		
S130	105	JAIVAC-1	Dosage A	2	Sodium	mEq / L	Day 28	23/09/2010	141	.	136 - 145		
S130	105	JAIVAC-1	Dosage A	2	ALT	U / L	Day 56	21/10/2010	17	.	0 - 41		
S130	105	JAIVAC-1	Dosage A	2	AST	U / L	Day 56	21/10/2010	22	.	0 - 40		
S130	105	JAIVAC-1	Dosage A	2	Alkaline Phosphatase	U / L	Day 56	21/10/2010	56	.	40 - 129		
S130	105	JAIVAC-1	Dosage A	2	Direct Bilirubin	mg / dl	Day 56	21/10/2010	0.16	.	0.00 - 0.30		
S130	105	JAIVAC-1	Dosage A	2	Gamma Glutamyl Transpeptidase	U / L	Day 56	21/10/2010	9	Yes	10 - 71	L	NCS
S130	105	JAIVAC-1	Dosage A	2	Indirect Bilirubin	mg / dl	Day 56	21/10/2010	0.43	.	0.00 - 0.99		
S130	105	JAIVAC-1	Dosage A	2	Total Bilirubin	mg / dl	Day 56	21/10/2010	0.59	.	0.00 - 0.99		
S130	105	JAIVAC-1	Dosage A	2	Serum creatinine	mg / dl	Day 56	21/10/2010	0.8	.	0.7 - 1.2		
S130	105	JAIVAC-1	Dosage A	2	Potassium	mEq / L	Day 56	21/10/2010	4.7	.	3.5 - 5.1		
S130	105	JAIVAC-1	Dosage A	2	Random blood glucose	mg / dl	Day 56	21/10/2010	82	.	45-130		
S130	105	JAIVAC-1	Dosage A	2	Sodium	mEq / L	Day 56	21/10/2010	141	.	136 - 145		
S130	105	JAIVAC-1	Dosage A	3	ALT	U / L	Day 180	22/02/2011	18	.	0 - 41		
S130	105	JAIVAC-1	Dosage A	3	AST	U / L	Day 180	22/02/2011	20	.	0 - 40		
S130	105	JAIVAC-1	Dosage A	3	Alkaline Phosphatase	U / L	Day 180	22/02/2011	59	.	40 - 129		
S130	105	JAIVAC-1	Dosage A	3	Direct Bilirubin	mg / dl	Day 180	22/02/2011	0.19	.	0.00 - 0.30		

Subject No.	Randomisation Number	Study Group	Dosage	Dose	Serum Chemistry Parameters	Unit	Day	Date of sample Collection	Results	Out of Range	Reference Range	Flag	Clinical Significance*
S130	105	JAIVAC-1	Dosage A	3	Gamma Glutamyl Transpeptidase	U / L	Day 180	22/02/2011	11	.	10 - 71		
S130	105	JAIVAC-1	Dosage A	3	Indirect Bilirubin	mg / dl	Day 180	22/02/2011	0.4	.	0.00 - 0.99		
S130	105	JAIVAC-1	Dosage A	3	Total Bilirubin	mg / dl	Day 180	22/02/2011	0.59	.	0.00 - 0.99		
S130	105	JAIVAC-1	Dosage A	3	Serum creatinine	mg / dl	Day 180	22/02/2011	1	.	0.7 - 1.2		
S130	105	JAIVAC-1	Dosage A	3	Potassium	mEq / L	Day 180	22/02/2011	4	.	3.5 - 5.1		
S130	105	JAIVAC-1	Dosage A	3	Random blood glucose	mg / dl	Day 180	22/02/2011	83	.	45-130		
S130	105	JAIVAC-1	Dosage A	3	Sodium	mEq / L	Day 180	22/02/2011	142	.	136 - 145		
S130	105	JAIVAC-1	Dosage A	3	ALT	U / L	Day 208	22/03/2011	14	.	0 - 41		
S130	105	JAIVAC-1	Dosage A	3	AST	U / L	Day 208	22/03/2011	19	.	0 - 40		
S130	105	JAIVAC-1	Dosage A	3	Alkaline Phosphatase	U / L	Day 208	22/03/2011	69	.	40 - 129		
S130	105	JAIVAC-1	Dosage A	3	Direct Bilirubin	mg / dl	Day 208	22/03/2011	0.18	.	0.00 - 0.30		
S130	105	JAIVAC-1	Dosage A	3	Gamma Glutamyl Transpeptidase	U / L	Day 208	22/03/2011	10	.	10 - 71		
S130	105	JAIVAC-1	Dosage A	3	Indirect Bilirubin	mg / dl	Day 208	22/03/2011	0.34	.	0.00 - 0.99		
S130	105	JAIVAC-1	Dosage A	3	Total Bilirubin	mg / dl	Day 208	22/03/2011	0.52	.	0.00 - 0.99		
S130	105	JAIVAC-1	Dosage A	3	Serum creatinine	mg / dl	Day 208	22/03/2011	0.9	.	0.7 - 1.2		
S130	105	JAIVAC-1	Dosage A	3	Potassium	mEq / L	Day 208	22/03/2011	3.8	.	3.5 - 5.1		
S130	105	JAIVAC-1	Dosage A	3	Random blood glucose	mg / dl	Day 208	22/03/2011	92	.	45-130		
S130	105	JAIVAC-1	Dosage A	3	Sodium	mEq / L	Day 208	22/03/2011	143	.	136 - 145		
S130	105	JAIVAC-1	Dosage A	3	ALT	U / L	Day 365	25/08/2011	18	.	0 - 41		
S130	105	JAIVAC-1	Dosage A	3	AST	U / L	Day 365	25/08/2011	21	.	0 - 40		
S130	105	JAIVAC-1	Dosage A	3	Alkaline Phosphatase	U / L	Day 365	25/08/2011	86	.	40 - 129		
S130	105	JAIVAC-1	Dosage A	3	Direct Bilirubin	mg / dl	Day 365	25/08/2011	0.19	.	0.00 - 0.30		
S130	105	JAIVAC-1	Dosage A	3	Gamma Glutamyl Transpeptidase	U / L	Day 365	25/08/2011	10	.	10 - 71		
S130	105	JAIVAC-1	Dosage A	3	Indirect Bilirubin	mg / dl	Day 365	25/08/2011	0.35	.	0.00 - 0.99		
S130	105	JAIVAC-1	Dosage A	3	Total Bilirubin	mg / dl	Day 365	25/08/2011	0.54	.	0.00 - 0.99		
S130	105	JAIVAC-1	Dosage A	3	Serum creatinine	mg / dl	Day 365	25/08/2011	0.8	.	0.7 - 1.2		
S130	105	JAIVAC-1	Dosage A	3	Potassium	mEq / L	Day 365	25/08/2011	4.5	.	3.5 - 5.1		
S130	105	JAIVAC-1	Dosage A	3	Random blood glucose	mg / dl	Day 365	25/08/2011	89	.	45-130		
S130	105	JAIVAC-1	Dosage A	3	Sodium	mEq / L	Day 365	25/08/2011	141	.	136 - 145		

Subject No.	Randomisation Number	Study Group	Dosage	Dose	Serum Chemistry Parameters	Unit	Day	Date of sample Collection	Results	Out of Range	Reference Range	Flag	Clinical Significance*
S132	108	Hepatitis B	Hepatitis B		ALT	U / L	Day -14 to Day -1	18/08/2010	30	.	0 - 41		
S132	108	Hepatitis B	Hepatitis B		AST	U / L	Day -14 to Day -1	18/08/2010	23	.	0 - 40		
S132	108	Hepatitis B	Hepatitis B		Alkaline Phosphatase	U / L	Day -14 to Day -1	18/08/2010	94	.	40 - 129		
S132	108	Hepatitis B	Hepatitis B		Direct Bilirubin	mg / dl	Day -14 to Day -1	18/08/2010	0.14	.	0.00 - 0.30		
S132	108	Hepatitis B	Hepatitis B		Gamma Glutamyl Transpeptidase	U / L	Day -14 to Day -1	18/08/2010	23	.	10 - 71		
S132	108	Hepatitis B	Hepatitis B		Indirect Bilirubin	mg / dl	Day -14 to Day -1	18/08/2010	0.26	.	0.00 - 0.99		
S132	108	Hepatitis B	Hepatitis B		Total Bilirubin	mg / dl	Day -14 to Day -1	18/08/2010	0.4	.	0.00 - 0.99		
S132	108	Hepatitis B	Hepatitis B		Serum creatinine	mg / dl	Day -14 to Day -1	18/08/2010	0.8	.	0.7 - 1.2		
S132	108	Hepatitis B	Hepatitis B		Potassium	mEq / L	Day -14 to Day -1	18/08/2010	4	.	3.5 - 5.1		
S132	108	Hepatitis B	Hepatitis B		Random blood glucose	mg / dl	Day -14 to Day -1	18/08/2010	97	.	45-130		
S132	108	Hepatitis B	Hepatitis B		Sodium	mEq / L	Day -14 to Day -1	18/08/2010	141	.	136 - 145		
S132	108	Hepatitis B	Hepatitis B	2	ALT	U / L	Day 28	23/09/2010	40	.	0 - 41		
S132	108	Hepatitis B	Hepatitis B	2	AST	U / L	Day 28	23/09/2010	28	.	0 - 40		
S132	108	Hepatitis B	Hepatitis B	2	Alkaline Phosphatase	U / L	Day 28	23/09/2010	86	.	40 - 129		
S132	108	Hepatitis B	Hepatitis B	2	Direct Bilirubin	mg / dl	Day 28	23/09/2010	<0.10	.	0.00 - 0.30		
S132	108	Hepatitis B	Hepatitis B	2	Gamma Glutamyl Transpeptidase	U / L	Day 28	23/09/2010	24	.	10 - 71		
S132	108	Hepatitis B	Hepatitis B	2	Indirect Bilirubin	mg / dl	Day 28	23/09/2010	NA	.	0.00 - 0.99		
S132	108	Hepatitis B	Hepatitis B	2	Total Bilirubin	mg / dl	Day 28	23/09/2010	0.34	.	0.00 - 0.99		
S132	108	Hepatitis B	Hepatitis B	2	Serum creatinine	mg / dl	Day 28	23/09/2010	0.8	.	0.7 - 1.2		
S132	108	Hepatitis B	Hepatitis B	2	Potassium	mEq / L	Day 28	23/09/2010	4.3	.	3.5 - 5.1		
S132	108	Hepatitis B	Hepatitis B	2	Random blood glucose	mg / dl	Day 28	23/09/2010	126	.	45-130		
S132	108	Hepatitis B	Hepatitis B	2	Sodium	mEq / L	Day 28	23/09/2010	139	.	136 - 145		
S132	108	Hepatitis B	Hepatitis B	2	ALT	U / L	Day 56	21/10/2010	41	.	0 - 41		
S132	108	Hepatitis B	Hepatitis B	2	AST	U / L	Day 56	21/10/2010	28	.	0 - 40		
S132	108	Hepatitis B	Hepatitis B	2	Alkaline Phosphatase	U / L	Day 56	21/10/2010	99	.	40 - 129		
S132	108	Hepatitis B	Hepatitis B	2	Direct Bilirubin	mg / dl	Day 56	21/10/2010	<0.10	.	0.00 - 0.30		
S132	108	Hepatitis B	Hepatitis B	2	Gamma Glutamyl Transpeptidase	U / L	Day 56	21/10/2010	36	.	10 - 71		
S132	108	Hepatitis B	Hepatitis B	2	Indirect Bilirubin	mg / dl	Day 56	21/10/2010	NA	.	0.00 - 0.99		
S132	108	Hepatitis B	Hepatitis B	2	Total Bilirubin	mg / dl	Day 56	21/10/2010	0.27	.	0.00 - 0.99		

Subject No.	Randomisation Number	Study Group	Dosage	Dose	Serum Chemistry Parameters	Unit	Day	Date of sample Collection	Results	Out of Range	Reference Range	Flag	Clinical Significance*
S132	108	Hepatitis B	Hepatitis B	2	Serum creatinine	mg / dl	Day 56	21/10/2010	0.8	.	0.7 - 1.2		
S132	108	Hepatitis B	Hepatitis B	2	Potassium	mEq / L	Day 56	21/10/2010	4.5	.	3.5 - 5.1		
S132	108	Hepatitis B	Hepatitis B	2	Random blood glucose	mg / dl	Day 56	21/10/2010	94	.	45-130		
S132	108	Hepatitis B	Hepatitis B	2	Sodium	mEq / L	Day 56	21/10/2010	139	.	136 - 145		
S132	108	Hepatitis B	Hepatitis B	3	ALT	U / L	Day 180	22/02/2011	29	.	0 - 41		
S132	108	Hepatitis B	Hepatitis B	3	AST	U / L	Day 180	22/02/2011	24	.	0 - 40		
S132	108	Hepatitis B	Hepatitis B	3	Alkaline Phosphatase	U / L	Day 180	22/02/2011	78	.	40 - 129		
S132	108	Hepatitis B	Hepatitis B	3	Direct Bilirubin	mg / dl	Day 180	22/02/2011	<0.10	.	0.00 - 0.30		
S132	108	Hepatitis B	Hepatitis B	3	Gamma Glutamyl Transpeptidase	U / L	Day 180	22/02/2011	24	.	10 - 71		
S132	108	Hepatitis B	Hepatitis B	3	Indirect Bilirubin	mg / dl	Day 180	22/02/2011	NA	.	0.00 - 0.99		
S132	108	Hepatitis B	Hepatitis B	3	Total Bilirubin	mg / dl	Day 180	22/02/2011	0.3	.	0.00 - 0.99		
S132	108	Hepatitis B	Hepatitis B	3	Serum creatinine	mg / dl	Day 180	22/02/2011	0.9	.	0.7 - 1.2		
S132	108	Hepatitis B	Hepatitis B	3	Potassium	mEq / L	Day 180	22/02/2011	4.8	.	3.5 - 5.1		
S132	108	Hepatitis B	Hepatitis B	3	Random blood glucose	mg / dl	Day 180	22/02/2011	75	.	45-130		
S132	108	Hepatitis B	Hepatitis B	3	Sodium	mEq / L	Day 180	22/02/2011	142	.	136 - 145		
S132	108	Hepatitis B	Hepatitis B	3	ALT	U / L	Day 208	22/03/2011	27	.	0 - 41		
S132	108	Hepatitis B	Hepatitis B	3	AST	U / L	Day 208	22/03/2011	21	.	0 - 40		
S132	108	Hepatitis B	Hepatitis B	3	Alkaline Phosphatase	U / L	Day 208	22/03/2011	85	.	40 - 129		
S132	108	Hepatitis B	Hepatitis B	3	Direct Bilirubin	mg / dl	Day 208	22/03/2011	<0.10	.	0.00 - 0.30		
S132	108	Hepatitis B	Hepatitis B	3	Gamma Glutamyl Transpeptidase	U / L	Day 208	22/03/2011	24	.	10 - 71		
S132	108	Hepatitis B	Hepatitis B	3	Indirect Bilirubin	mg / dl	Day 208	22/03/2011	NA	.	0.00 - 0.99		
S132	108	Hepatitis B	Hepatitis B	3	Total Bilirubin	mg / dl	Day 208	22/03/2011	0.17	.	0.00 - 0.99		
S132	108	Hepatitis B	Hepatitis B	3	Serum creatinine	mg / dl	Day 208	22/03/2011	1	.	0.7 - 1.2		
S132	108	Hepatitis B	Hepatitis B	3	Potassium	mEq / L	Day 208	22/03/2011	4.2	.	3.5 - 5.1		
S132	108	Hepatitis B	Hepatitis B	3	Random blood glucose	mg / dl	Day 208	22/03/2011	131	Yes	45-130	H	NCS
S132	108	Hepatitis B	Hepatitis B	3	Sodium	mEq / L	Day 208	22/03/2011	144	.	136 - 145		
S132	108	Hepatitis B	Hepatitis B	3	ALT	U / L	Day 365	25/08/2011	40	.	0 - 41		
S132	108	Hepatitis B	Hepatitis B	3	AST	U / L	Day 365	25/08/2011	29	.	0 - 40		
S132	108	Hepatitis B	Hepatitis B	3	Alkaline Phosphatase	U / L	Day 365	25/08/2011	81	.	40 - 129		

Subject No.	Randomisation Number	Study Group	Dosage	Dose	Serum Chemistry Parameters	Unit	Day	Date of sample Collection	Results	Out of Range	Reference Range	Flag	Clinical Significance*
S132	108	Hepatitis B	Hepatitis B	3	Direct Bilirubin	mg / dl	Day 365	25/08/2011	<0.10	.	0.00 - 0.30		
S132	108	Hepatitis B	Hepatitis B	3	Gamma Glutamyl Transpeptidase	U / L	Day 365	25/08/2011	17	.	10 - 71		
S132	108	Hepatitis B	Hepatitis B	3	Indirect Bilirubin	mg / dl	Day 365	25/08/2011	NA	.	0.00 - 0.99		
S132	108	Hepatitis B	Hepatitis B	3	Total Bilirubin	mg / dl	Day 365	25/08/2011	0.24	.	0.00 - 0.99		
S132	108	Hepatitis B	Hepatitis B	3	Serum creatinine	mg / dl	Day 365	25/08/2011	1	.	0.7 - 1.2		
S132	108	Hepatitis B	Hepatitis B	3	Potassium	mEq / L	Day 365	25/08/2011	4.5	.	3.5 - 5.1		
S132	108	Hepatitis B	Hepatitis B	3	Random blood glucose	mg / dl	Day 365	25/08/2011	75	.	45-130		
S132	108	Hepatitis B	Hepatitis B	3	Sodium	mEq / L	Day 365	25/08/2011	140	.	136 - 145		
S136	109	JAIVAC-1	Dosage A		ALT	U / L	Day -14 to Day -1	19/08/2010	37	.	0 - 41		
S136	109	JAIVAC-1	Dosage A		AST	U / L	Day -14 to Day -1	19/08/2010	27	.	0 - 40		
S136	109	JAIVAC-1	Dosage A		Alkaline Phosphatase	U / L	Day -14 to Day -1	19/08/2010	69	.	40 - 129		
S136	109	JAIVAC-1	Dosage A		Direct Bilirubin	mg / dl	Day -14 to Day -1	19/08/2010	0.25	.	0.00 - 0.30		
S136	109	JAIVAC-1	Dosage A		Gamma Glutamyl Transpeptidase	U / L	Day -14 to Day -1	19/08/2010	28	.	10 - 71		
S136	109	JAIVAC-1	Dosage A		Indirect Bilirubin	mg / dl	Day -14 to Day -1	19/08/2010	0.4	.	0.00 - 0.99		
S136	109	JAIVAC-1	Dosage A		Total Bilirubin	mg / dl	Day -14 to Day -1	19/08/2010	0.65	.	0.00 - 0.99		
S136	109	JAIVAC-1	Dosage A		Serum creatinine	mg / dl	Day -14 to Day -1	19/08/2010	1	.	0.7 - 1.2		
S136	109	JAIVAC-1	Dosage A		Potassium	mEq / L	Day -14 to Day -1	19/08/2010	3.9	.	3.5 - 5.1		
S136	109	JAIVAC-1	Dosage A		Random blood glucose	mg / dl	Day -14 to Day -1	19/08/2010	68	.	45-130		
S136	109	JAIVAC-1	Dosage A		Sodium	mEq / L	Day -14 to Day -1	19/08/2010	140	.	136 - 145		
S136	109	JAIVAC-1	Dosage A	2	ALT	U / L	Day 28	23/09/2010	31	.	0 - 41		
S136	109	JAIVAC-1	Dosage A	2	AST	U / L	Day 28	23/09/2010	26	.	0 - 40		
S136	109	JAIVAC-1	Dosage A	2	Alkaline Phosphatase	U / L	Day 28	23/09/2010	74	.	40 - 129		
S136	109	JAIVAC-1	Dosage A	2	Direct Bilirubin	mg / dl	Day 28	23/09/2010	0.14	.	0.00 - 0.30		
S136	109	JAIVAC-1	Dosage A	2	Gamma Glutamyl Transpeptidase	U / L	Day 28	23/09/2010	29	.	10 - 71		
S136	109	JAIVAC-1	Dosage A	2	Indirect Bilirubin	mg / dl	Day 28	23/09/2010	0.34	.	0.00 - 0.99		
S136	109	JAIVAC-1	Dosage A	2	Total Bilirubin	mg / dl	Day 28	23/09/2010	0.48	.	0.00 - 0.99		
S136	109	JAIVAC-1	Dosage A	2	Serum creatinine	mg / dl	Day 28	23/09/2010	1.1	.	0.7 - 1.2		
S136	109	JAIVAC-1	Dosage A	2	Potassium	mEq / L	Day 28	23/09/2010	4.6	.	3.5 - 5.1		
S136	109	JAIVAC-1	Dosage A	2	Random blood glucose	mg / dl	Day 28	23/09/2010	83	.	45-130		

Subject No.	Randomisation Number	Study Group	Dosage	Dose	Serum Chemistry Parameters	Unit	Day	Date of sample Collection	Results	Out of Range	Reference Range	Flag	Clinical Significance*
S136	109	JAIVAC-1	Dosage A	2	Sodium	mEq / L	Day 28	23/09/2010	138	.	136 - 145		
S136	109	JAIVAC-1	Dosage A	2	ALT	U / L	Day 56	21/10/2010	35	.	0 - 41		
S136	109	JAIVAC-1	Dosage A	2	AST	U / L	Day 56	21/10/2010	31	.	0 - 40		
S136	109	JAIVAC-1	Dosage A	2	Alkaline Phosphatase	U / L	Day 56	21/10/2010	76	.	40 - 129		
S136	109	JAIVAC-1	Dosage A	2	Direct Bilirubin	mg / dl	Day 56	21/10/2010	<0.10	.	0.00 - 0.30		
S136	109	JAIVAC-1	Dosage A	2	Gamma Glutamyl Transpeptidase	U / L	Day 56	21/10/2010	38	.	10 - 71		
S136	109	JAIVAC-1	Dosage A	2	Indirect Bilirubin	mg / dl	Day 56	21/10/2010	NA	.	0.00 - 0.99		
S136	109	JAIVAC-1	Dosage A	2	Total Bilirubin	mg / dl	Day 56	21/10/2010	0.27	.	0.00 - 0.99		
S136	109	JAIVAC-1	Dosage A	2	Serum creatinine	mg / dl	Day 56	21/10/2010	0.9	.	0.7 - 1.2		
S136	109	JAIVAC-1	Dosage A	2	Potassium	mEq / L	Day 56	21/10/2010	4.7	.	3.5 - 5.1		
S136	109	JAIVAC-1	Dosage A	2	Random blood glucose	mg / dl	Day 56	21/10/2010	93	.	45-130		
S136	109	JAIVAC-1	Dosage A	2	Sodium	mEq / L	Day 56	21/10/2010	140	.	136 - 145		
S136	109	JAIVAC-1	Dosage A	3	ALT	U / L	Day 180	22/02/2011	23	.	0 - 41		
S136	109	JAIVAC-1	Dosage A	3	AST	U / L	Day 180	22/02/2011	18	.	0 - 40		
S136	109	JAIVAC-1	Dosage A	3	Alkaline Phosphatase	U / L	Day 180	22/02/2011	67	.	40 - 129		
S136	109	JAIVAC-1	Dosage A	3	Direct Bilirubin	mg / dl	Day 180	22/02/2011	0.15	.	0.00 - 0.30		
S136	109	JAIVAC-1	Dosage A	3	Gamma Glutamyl Transpeptidase	U / L	Day 180	22/02/2011	32	.	10 - 71		
S136	109	JAIVAC-1	Dosage A	3	Indirect Bilirubin	mg / dl	Day 180	22/02/2011	0.38	.	0.00 - 0.99		
S136	109	JAIVAC-1	Dosage A	3	Total Bilirubin	mg / dl	Day 180	22/02/2011	0.53	.	0.00 - 0.99		
S136	109	JAIVAC-1	Dosage A	3	Serum creatinine	mg / dl	Day 180	22/02/2011	1	.	0.7 - 1.2		
S136	109	JAIVAC-1	Dosage A	3	Potassium	mEq / L	Day 180	22/02/2011	3.8	.	3.5 - 5.1		
S136	109	JAIVAC-1	Dosage A	3	Random blood glucose	mg / dl	Day 180	22/02/2011	74	.	45-130		
S136	109	JAIVAC-1	Dosage A	3	Sodium	mEq / L	Day 180	22/02/2011	141	.	136 - 145		
S136	109	JAIVAC-1	Dosage A	3	ALT	U / L	Day 208	22/03/2011	29	.	0 - 41		
S136	109	JAIVAC-1	Dosage A	3	AST	U / L	Day 208	22/03/2011	19	.	0 - 40		
S136	109	JAIVAC-1	Dosage A	3	Alkaline Phosphatase	U / L	Day 208	22/03/2011	69	.	40 - 129		
S136	109	JAIVAC-1	Dosage A	3	Direct Bilirubin	mg / dl	Day 208	22/03/2011	0.22	.	0.00 - 0.30		
S136	109	JAIVAC-1	Dosage A	3	Gamma Glutamyl Transpeptidase	U / L	Day 208	22/03/2011	36	.	10 - 71		
S136	109	JAIVAC-1	Dosage A	3	Indirect Bilirubin	mg / dl	Day 208	22/03/2011	0.47	.	0.00 - 0.99		

Subject No.	Randomisation Number	Study Group	Dosage	Dose	Serum Chemistry Parameters	Unit	Day	Date of sample Collection	Results	Out of Range	Reference Range	Flag	Clinical Significance*
S136	109	JAIVAC-1	Dosage A	3	Total Bilirubin	mg / dl	Day 208	22/03/2011	0.69	.	0.00 - 0.99		
S136	109	JAIVAC-1	Dosage A	3	Serum creatinine	mg / dl	Day 208	22/03/2011	1.1	.	0.7 - 1.2		
S136	109	JAIVAC-1	Dosage A	3	Potassium	mEq / L	Day 208	22/03/2011	4.6	.	3.5 - 5.1		
S136	109	JAIVAC-1	Dosage A	3	Random blood glucose	mg / dl	Day 208	22/03/2011	86	.	45-130		
S136	109	JAIVAC-1	Dosage A	3	Sodium	mEq / L	Day 208	22/03/2011	139	.	136 - 145		
S136	109	JAIVAC-1	Dosage A	3	ALT	U / L	Day 365	25/08/2011	37	.	0 - 41		
S136	109	JAIVAC-1	Dosage A	3	AST	U / L	Day 365	25/08/2011	27	.	0 - 40		
S136	109	JAIVAC-1	Dosage A	3	Alkaline Phosphatase	U / L	Day 365	25/08/2011	72	.	40 - 129		
S136	109	JAIVAC-1	Dosage A	3	Direct Bilirubin	mg / dl	Day 365	25/08/2011	0.15	.	0.00 - 0.30		
S136	109	JAIVAC-1	Dosage A	3	Gamma Glutamyl Transpeptidase	U / L	Day 365	25/08/2011	25	.	10 - 71		
S136	109	JAIVAC-1	Dosage A	3	Indirect Bilirubin	mg / dl	Day 365	25/08/2011	0.42	.	0.00 - 0.99		
S136	109	JAIVAC-1	Dosage A	3	Total Bilirubin	mg / dl	Day 365	25/08/2011	0.57	.	0.00 - 0.99		
S136	109	JAIVAC-1	Dosage A	3	Serum creatinine	mg / dl	Day 365	25/08/2011	1.2	.	0.7 - 1.2		
S136	109	JAIVAC-1	Dosage A	3	Potassium	mEq / L	Day 365	25/08/2011	4.5	.	3.5 - 5.1		
S136	109	JAIVAC-1	Dosage A	3	Random blood glucose	mg / dl	Day 365	25/08/2011	86	.	45-130		
S136	109	JAIVAC-1	Dosage A	3	Sodium	mEq / L	Day 365	25/08/2011	141	.	136 - 145		
S143	101	JAIVAC-1	Dosage A		ALT	U / L	Day -14 to Day -1	18/08/2010	33	.	0 - 41		
S143	101	JAIVAC-1	Dosage A		AST	U / L	Day -14 to Day -1	18/08/2010	28	.	0 - 40		
S143	101	JAIVAC-1	Dosage A		Alkaline Phosphatase	U / L	Day -14 to Day -1	18/08/2010	73	.	40 - 129		
S143	101	JAIVAC-1	Dosage A		Direct Bilirubin	mg / dl	Day -14 to Day -1	18/08/2010	0.21	.	0.00 - 0.30		
S143	101	JAIVAC-1	Dosage A		Gamma Glutamyl Transpeptidase	U / L	Day -14 to Day -1	18/08/2010	12	.	10 - 71		
S143	101	JAIVAC-1	Dosage A		Indirect Bilirubin	mg / dl	Day -14 to Day -1	18/08/2010	0.45	.	0.00 - 0.99		
S143	101	JAIVAC-1	Dosage A		Total Bilirubin	mg / dl	Day -14 to Day -1	18/08/2010	0.66	.	0.00 - 0.99		
S143	101	JAIVAC-1	Dosage A		Serum creatinine	mg / dl	Day -14 to Day -1	18/08/2010	1	.	0.7 - 1.2		
S143	101	JAIVAC-1	Dosage A		Potassium	mEq / L	Day -14 to Day -1	18/08/2010	3.8	.	3.5 - 5.1		
S143	101	JAIVAC-1	Dosage A		Random blood glucose	mg / dl	Day -14 to Day -1	18/08/2010	88	.	45-130		
S143	101	JAIVAC-1	Dosage A		Sodium	mEq / L	Day -14 to Day -1	18/08/2010	141	.	136 - 145		
S143	101	JAIVAC-1	Dosage A	2	ALT	U / L	Day 28	21/09/2010	30	.	0 - 41		
S143	101	JAIVAC-1	Dosage A	2	AST	U / L	Day 28	21/09/2010	26	.	0 - 40		

Subject No.	Randomisation Number	Study Group	Dosage	Dose	Serum Chemistry Parameters	Unit	Day	Date of sample Collection	Results	Out of Range	Reference Range	Flag	Clinical Significance*
S143	101	JAIVAC-1	Dosage A	2	Alkaline Phosphatase	U / L	Day 28	21/09/2010	73	.	40 - 129		
S143	101	JAIVAC-1	Dosage A	2	Direct Bilirubin	mg / dl	Day 28	21/09/2010	0.19	.	0.00 - 0.30		
S143	101	JAIVAC-1	Dosage A	2	Gamma Glutamyl Transpeptidase	U / L	Day 28	21/09/2010	10	.	10 - 71		
S143	101	JAIVAC-1	Dosage A	2	Indirect Bilirubin	mg / dl	Day 28	21/09/2010	0.49	.	0.00 - 0.99		
S143	101	JAIVAC-1	Dosage A	2	Total Bilirubin	mg / dl	Day 28	21/09/2010	0.68	.	0.00 - 0.99		
S143	101	JAIVAC-1	Dosage A	2	Serum creatinine	mg / dl	Day 28	21/09/2010	1.1	.	0.7 - 1.2		
S143	101	JAIVAC-1	Dosage A	2	Potassium	mEq / L	Day 28	21/09/2010	3.9	.	3.5 - 5.1		
S143	101	JAIVAC-1	Dosage A	2	Random blood glucose	mg / dl	Day 28	21/09/2010	91	.	45-130		
S143	101	JAIVAC-1	Dosage A	2	Sodium	mEq / L	Day 28	21/09/2010	139	.	136 - 145		
S143	101	JAIVAC-1	Dosage A	2	ALT	U / L	Day 56	19/10/2010	29	.	0 - 41		
S143	101	JAIVAC-1	Dosage A	2	AST	U / L	Day 56	19/10/2010	25	.	0 - 40		
S143	101	JAIVAC-1	Dosage A	2	Alkaline Phosphatase	U / L	Day 56	19/10/2010	78	.	40 - 129		
S143	101	JAIVAC-1	Dosage A	2	Direct Bilirubin	mg / dl	Day 56	19/10/2010	0.16	.	0.00 - 0.30		
S143	101	JAIVAC-1	Dosage A	2	Gamma Glutamyl Transpeptidase	U / L	Day 56	19/10/2010	15	.	10 - 71		
S143	101	JAIVAC-1	Dosage A	2	Indirect Bilirubin	mg / dl	Day 56	19/10/2010	0.41	.	0.00 - 0.99		
S143	101	JAIVAC-1	Dosage A	2	Total Bilirubin	mg / dl	Day 56	19/10/2010	0.57	.	0.00 - 0.99		
S143	101	JAIVAC-1	Dosage A	2	Serum creatinine	mg / dl	Day 56	19/10/2010	1	.	0.7 - 1.2		
S143	101	JAIVAC-1	Dosage A	2	Potassium	mEq / L	Day 56	19/10/2010	4	.	3.5 - 5.1		
S143	101	JAIVAC-1	Dosage A	2	Random blood glucose	mg / dl	Day 56	19/10/2010	130	.	45-130		
S143	101	JAIVAC-1	Dosage A	2	Sodium	mEq / L	Day 56	19/10/2010	141	.	136 - 145		
S143	101	JAIVAC-1	Dosage A	3	ALT	U / L	Day 180	21/02/2011	32	.	0 - 41		
S143	101	JAIVAC-1	Dosage A	3	AST	U / L	Day 180	21/02/2011	29	.	0 - 40		
S143	101	JAIVAC-1	Dosage A	3	Alkaline Phosphatase	U / L	Day 180	21/02/2011	78	.	40 - 129		
S143	101	JAIVAC-1	Dosage A	3	Direct Bilirubin	mg / dl	Day 180	21/02/2011	0.2	.	0.00 - 0.30		
S143	101	JAIVAC-1	Dosage A	3	Gamma Glutamyl Transpeptidase	U / L	Day 180	21/02/2011	15	.	10 - 71		
S143	101	JAIVAC-1	Dosage A	3	Indirect Bilirubin	mg / dl	Day 180	21/02/2011	0.45	.	0.00 - 0.99		
S143	101	JAIVAC-1	Dosage A	3	Total Bilirubin	mg / dl	Day 180	21/02/2011	0.65	.	0.00 - 0.99		
S143	101	JAIVAC-1	Dosage A	3	Serum creatinine	mg / dl	Day 180	21/02/2011	1.2	.	0.7 - 1.2		
S143	101	JAIVAC-1	Dosage A	3	Potassium	mEq / L	Day 180	21/02/2011	4	.	3.5 - 5.1		

Subject No.	Randomisation Number	Study Group	Dosage	Dose	Serum Chemistry Parameters	Unit	Day	Date of sample Collection	Results	Out of Range	Reference Range	Flag	Clinical Significance*
S143	101	JAIVAC-1	Dosage A	3	Random blood glucose	mg / dl	Day 180	21/02/2011	85	.	45-130		
S143	101	JAIVAC-1	Dosage A	3	Sodium	mEq / L	Day 180	21/02/2011	143	.	136 - 145		
S143	101	JAIVAC-1	Dosage A	3	ALT	U / L	Day 208	21/03/2011	34	.	0 - 41		
S143	101	JAIVAC-1	Dosage A	3	AST	U / L	Day 208	21/03/2011	26	.	0 - 40		
S143	101	JAIVAC-1	Dosage A	3	Alkaline Phosphatase	U / L	Day 208	21/03/2011	73	.	40 - 129		
S143	101	JAIVAC-1	Dosage A	3	Direct Bilirubin	mg / dl	Day 208	21/03/2011	0.21	.	0.00 - 0.30		
S143	101	JAIVAC-1	Dosage A	3	Gamma Glutamyl Transpeptidase	U / L	Day 208	21/03/2011	14	.	10 - 71		
S143	101	JAIVAC-1	Dosage A	3	Indirect Bilirubin	mg / dl	Day 208	21/03/2011	0.58	.	0.00 - 0.99		
S143	101	JAIVAC-1	Dosage A	3	Total Bilirubin	mg / dl	Day 208	21/03/2011	0.79	.	0.00 - 0.99		
S143	101	JAIVAC-1	Dosage A	3	Serum creatinine	mg / dl	Day 208	21/03/2011	1.2	.	0.7 - 1.2		
S143	101	JAIVAC-1	Dosage A	3	Potassium	mEq / L	Day 208	21/03/2011	4.3	.	3.5 - 5.1		
S143	101	JAIVAC-1	Dosage A	3	Random blood glucose	mg / dl	Day 208	21/03/2011	85	.	45-130		
S143	101	JAIVAC-1	Dosage A	3	Sodium	mEq / L	Day 208	21/03/2011	140	.	136 - 145		
S143	101	JAIVAC-1	Dosage A	3	ALT	U / L	Day 365	26/08/2011	33	.	0 - 41		
S143	101	JAIVAC-1	Dosage A	3	AST	U / L	Day 365	26/08/2011	26	.	0 - 40		
S143	101	JAIVAC-1	Dosage A	3	Alkaline Phosphatase	U / L	Day 365	26/08/2011	69	.	40 - 129		
S143	101	JAIVAC-1	Dosage A	3	Direct Bilirubin	mg / dl	Day 365	26/08/2011	0.27	.	0.00 - 0.30		
S143	101	JAIVAC-1	Dosage A	3	Gamma Glutamyl Transpeptidase	U / L	Day 365	26/08/2011	13	.	10 - 71		
S143	101	JAIVAC-1	Dosage A	3	Indirect Bilirubin	mg / dl	Day 365	26/08/2011	0.48	.	0.00 - 0.99		
S143	101	JAIVAC-1	Dosage A	3	Total Bilirubin	mg / dl	Day 365	26/08/2011	0.75	.	0.00 - 0.99		
S143	101	JAIVAC-1	Dosage A	3	Serum creatinine	mg / dl	Day 365	26/08/2011	1.1	.	0.7 - 1.2		
S143	101	JAIVAC-1	Dosage A	3	Potassium	mEq / L	Day 365	26/08/2011	4.2	.	3.5 - 5.1		
S143	101	JAIVAC-1	Dosage A	3	Random blood glucose	mg / dl	Day 365	26/08/2011	90	.	45-130		
S143	101	JAIVAC-1	Dosage A	3	Sodium	mEq / L	Day 365	26/08/2011	142	.	136 - 145		
S144	103	Hepatitis B	Hepatitis B		ALT	U / L	Day -14 to Day -1	19/08/2010	17	.	0 - 41		
S144	103	Hepatitis B	Hepatitis B		AST	U / L	Day -14 to Day -1	19/08/2010	22	.	0 - 40		
S144	103	Hepatitis B	Hepatitis B		Alkaline Phosphatase	U / L	Day -14 to Day -1	19/08/2010	100	.	40 - 129		
S144	103	Hepatitis B	Hepatitis B		Direct Bilirubin	mg / dl	Day -14 to Day -1	19/08/2010	0.11	.	0.00 - 0.30		
S144	103	Hepatitis B	Hepatitis B		Gamma Glutamyl Transpeptidase	U / L	Day -14 to Day -1	19/08/2010	7	.	10 - 71	L	

Subject No.	Randomisation Number	Study Group	Dosage	Dose	Serum Chemistry Parameters	Unit	Day	Date of sample Collection	Results	Out of Range	Reference Range	Flag	Clinical Significance*
S144	103	Hepatitis B	Hepatitis B		Indirect Bilirubin	mg / dl	Day -14 to Day -1	19/08/2010	0.3	.	0.00 - 0.99		
S144	103	Hepatitis B	Hepatitis B		Total Bilirubin	mg / dl	Day -14 to Day -1	19/08/2010	0.41	.	0.00 - 0.99		
S144	103	Hepatitis B	Hepatitis B		Serum creatinine	mg / dl	Day -14 to Day -1	19/08/2010	1	.	0.7 - 1.2		
S144	103	Hepatitis B	Hepatitis B		Potassium	mEq / L	Day -14 to Day -1	19/08/2010	4	.	3.5 - 5.1		
S144	103	Hepatitis B	Hepatitis B		Random blood glucose	mg / dl	Day -14 to Day -1	19/08/2010	83	.	45-130		
S144	103	Hepatitis B	Hepatitis B		Sodium	mEq / L	Day -14 to Day -1	19/08/2010	141	.	136 - 145		
S144	103	Hepatitis B	Hepatitis B	2	ALT	U / L	Day 28	21/09/2010	15	.	0 - 41		
S144	103	Hepatitis B	Hepatitis B	2	AST	U / L	Day 28	21/09/2010	17	.	0 - 40		
S144	103	Hepatitis B	Hepatitis B	2	Alkaline Phosphatase	U / L	Day 28	21/09/2010	81	.	40 - 129		
S144	103	Hepatitis B	Hepatitis B	2	Direct Bilirubin	mg / dl	Day 28	21/09/2010	0.18	.	0.00 - 0.30		
S144	103	Hepatitis B	Hepatitis B	2	Gamma Glutamyl Transpeptidase	U / L	Day 28	21/09/2010	6	Yes	10 - 71	L	NCS
S144	103	Hepatitis B	Hepatitis B	2	Indirect Bilirubin	mg / dl	Day 28	21/09/2010	0.43	.	0.00 - 0.99		
S144	103	Hepatitis B	Hepatitis B	2	Total Bilirubin	mg / dl	Day 28	21/09/2010	0.61	.	0.00 - 0.99		
S144	103	Hepatitis B	Hepatitis B	2	Serum creatinine	mg / dl	Day 28	21/09/2010	0.9	.	0.7 - 1.2		
S144	103	Hepatitis B	Hepatitis B	2	Potassium	mEq / L	Day 28	21/09/2010	4.1	.	3.5 - 5.1		
S144	103	Hepatitis B	Hepatitis B	2	Random blood glucose	mg / dl	Day 28	21/09/2010	92	.	45-130		
S144	103	Hepatitis B	Hepatitis B	2	Sodium	mEq / L	Day 28	21/09/2010	145	.	136 - 145		
S144	103	Hepatitis B	Hepatitis B	2	ALT	U / L	Day 56	19/10/2010	19	.	0 - 41		
S144	103	Hepatitis B	Hepatitis B	2	AST	U / L	Day 56	19/10/2010	23	.	0 - 40		
S144	103	Hepatitis B	Hepatitis B	2	Alkaline Phosphatase	U / L	Day 56	19/10/2010	99	.	40 - 129		
S144	103	Hepatitis B	Hepatitis B	2	Direct Bilirubin	mg / dl	Day 56	19/10/2010	0.1	.	0.00 - 0.30		
S144	103	Hepatitis B	Hepatitis B	2	Gamma Glutamyl Transpeptidase	U / L	Day 56	19/10/2010	8	Yes	10 - 71	L	NCS
S144	103	Hepatitis B	Hepatitis B	2	Indirect Bilirubin	mg / dl	Day 56	19/10/2010	0.41	.	0.00 - 0.99		
S144	103	Hepatitis B	Hepatitis B	2	Total Bilirubin	mg / dl	Day 56	19/10/2010	0.51	.	0.00 - 0.99		
S144	103	Hepatitis B	Hepatitis B	2	Serum creatinine	mg / dl	Day 56	19/10/2010	0.8	.	0.7 - 1.2		
S144	103	Hepatitis B	Hepatitis B	2	Potassium	mEq / L	Day 56	19/10/2010	4	.	3.5 - 5.1		
S144	103	Hepatitis B	Hepatitis B	2	Random blood glucose	mg / dl	Day 56	19/10/2010	85	.	45-130		
S144	103	Hepatitis B	Hepatitis B	2	Sodium	mEq / L	Day 56	19/10/2010	143	.	136 - 145		
S144	103	Hepatitis B	Hepatitis B	3	ALT	U / L	Day 180	21/02/2011	15	.	0 - 41		

Subject No.	Randomisation Number	Study Group	Dosage	Dose	Serum Chemistry Parameters	Unit	Day	Date of sample Collection	Results	Out of Range	Reference Range	Flag	Clinical Significance*
S144	103	Hepatitis B	Hepatitis B	3	AST	U / L	Day 180	21/02/2011	21	.	0 - 40		
S144	103	Hepatitis B	Hepatitis B	3	Alkaline Phosphatase	U / L	Day 180	21/02/2011	88	.	40 - 129		
S144	103	Hepatitis B	Hepatitis B	3	Direct Bilirubin	mg / dl	Day 180	21/02/2011	0.1	.	0.00 - 0.30		
S144	103	Hepatitis B	Hepatitis B	3	Gamma Glutamyl Transpeptidase	U / L	Day 180	21/02/2011	6	Yes	10 - 71	L	NCS
S144	103	Hepatitis B	Hepatitis B	3	Indirect Bilirubin	mg / dl	Day 180	21/02/2011	0.42	.	0.00 - 0.99		
S144	103	Hepatitis B	Hepatitis B	3	Total Bilirubin	mg / dl	Day 180	21/02/2011	0.52	.	0.00 - 0.99		
S144	103	Hepatitis B	Hepatitis B	3	Serum creatinine	mg / dl	Day 180	21/02/2011	1	.	0.7 - 1.2		
S144	103	Hepatitis B	Hepatitis B	3	Potassium	mEq / L	Day 180	21/02/2011	4.1	.	3.5 - 5.1		
S144	103	Hepatitis B	Hepatitis B	3	Random blood glucose	mg / dl	Day 180	21/02/2011	92	.	45-130		
S144	103	Hepatitis B	Hepatitis B	3	Sodium	mEq / L	Day 180	21/02/2011	142	.	136 - 145		
S144	103	Hepatitis B	Hepatitis B	3	ALT	U / L	Day 208	21/03/2011	22	.	0 - 41		
S144	103	Hepatitis B	Hepatitis B	3	AST	U / L	Day 208	21/03/2011	20	.	0 - 40		
S144	103	Hepatitis B	Hepatitis B	3	Alkaline Phosphatase	U / L	Day 208	21/03/2011	93	.	40 - 129		
S144	103	Hepatitis B	Hepatitis B	3	Direct Bilirubin	mg / dl	Day 208	21/03/2011	0.17	.	0.00 - 0.30		
S144	103	Hepatitis B	Hepatitis B	3	Gamma Glutamyl Transpeptidase	U / L	Day 208	21/03/2011	8	Yes	10 - 71	L	NCS
S144	103	Hepatitis B	Hepatitis B	3	Indirect Bilirubin	mg / dl	Day 208	21/03/2011	0.48	.	0.00 - 0.99		
S144	103	Hepatitis B	Hepatitis B	3	Total Bilirubin	mg / dl	Day 208	21/03/2011	0.65	.	0.00 - 0.99		
S144	103	Hepatitis B	Hepatitis B	3	Serum creatinine	mg / dl	Day 208	21/03/2011	1	.	0.7 - 1.2		
S144	103	Hepatitis B	Hepatitis B	3	Potassium	mEq / L	Day 208	21/03/2011	4	.	3.5 - 5.1		
S144	103	Hepatitis B	Hepatitis B	3	Random blood glucose	mg / dl	Day 208	21/03/2011	88	.	45-130		
S144	103	Hepatitis B	Hepatitis B	3	Sodium	mEq / L	Day 208	21/03/2011	142	.	136 - 145		
S144	103	Hepatitis B	Hepatitis B	3	ALT	U / L	Day 365	26/08/2011	27	.	0 - 41		
S144	103	Hepatitis B	Hepatitis B	3	AST	U / L	Day 365	26/08/2011	25	.	0 - 40		
S144	103	Hepatitis B	Hepatitis B	3	Alkaline Phosphatase	U / L	Day 365	26/08/2011	85	.	40 - 129		
S144	103	Hepatitis B	Hepatitis B	3	Direct Bilirubin	mg / dl	Day 365	26/08/2011	0.11	.	0.00 - 0.30		
S144	103	Hepatitis B	Hepatitis B	3	Gamma Glutamyl Transpeptidase	U / L	Day 365	26/08/2011	8	Yes	10 - 71	L	NCS
S144	103	Hepatitis B	Hepatitis B	3	Indirect Bilirubin	mg / dl	Day 365	26/08/2011	0.26	.	0.00 - 0.99		
S144	103	Hepatitis B	Hepatitis B	3	Total Bilirubin	mg / dl	Day 365	26/08/2011	0.37	.	0.00 - 0.99		
S144	103	Hepatitis B	Hepatitis B	3	Serum creatinine	mg / dl	Day 365	26/08/2011	1.1	.	0.7 - 1.2		

Subject No.	Randomisation Number	Study Group	Dosage	Dose	Serum Chemistry Parameters	Unit	Day	Date of sample Collection	Results	Out of Range	Reference Range	Flag	Clinical Significance*
S144	103	Hepatitis B	Hepatitis B	3	Potassium	mEq / L	Day 365	26/08/2011	4.9	.	3.5 - 5.1		
S144	103	Hepatitis B	Hepatitis B	3	Random blood glucose	mg / dl	Day 365	26/08/2011	88	.	45-130		
S144	103	Hepatitis B	Hepatitis B	3	Sodium	mEq / L	Day 365	26/08/2011	142	.	136 - 145		
S145	112	JAIVAC-1	Dosage A		ALT	U / L	Day -14 to Day -1	19/08/2010	19	.	0 - 41		
S145	112	JAIVAC-1	Dosage A		AST	U / L	Day -14 to Day -1	19/08/2010	17	.	0 - 40		
S145	112	JAIVAC-1	Dosage A		Alkaline Phosphatase	U / L	Day -14 to Day -1	19/08/2010	51	.	40 - 129		
S145	112	JAIVAC-1	Dosage A		Direct Bilirubin	mg / dl	Day -14 to Day -1	19/08/2010	0.21	.	0.00 - 0.30		
S145	112	JAIVAC-1	Dosage A		Gamma Glutamyl Transpeptidase	U / L	Day -14 to Day -1	19/08/2010	14	.	10 - 71		
S145	112	JAIVAC-1	Dosage A		Indirect Bilirubin	mg / dl	Day -14 to Day -1	19/08/2010	0.43	.	0.00 - 0.99		
S145	112	JAIVAC-1	Dosage A		Total Bilirubin	mg / dl	Day -14 to Day -1	19/08/2010	0.64	.	0.00 - 0.99		
S145	112	JAIVAC-1	Dosage A		Serum creatinine	mg / dl	Day -14 to Day -1	19/08/2010	1	.	0.7 - 1.2		
S145	112	JAIVAC-1	Dosage A		Potassium	mEq / L	Day -14 to Day -1	19/08/2010	3.9	.	3.5 - 5.1		
S145	112	JAIVAC-1	Dosage A		Random blood glucose	mg / dl	Day -14 to Day -1	19/08/2010	81	.	45-130		
S145	112	JAIVAC-1	Dosage A		Sodium	mEq / L	Day -14 to Day -1	19/08/2010	142	.	136 - 145		
S145	112	JAIVAC-1	Dosage A	2	ALT	U / L	Day 28	24/09/2010	14	.	0 - 41		
S145	112	JAIVAC-1	Dosage A	2	AST	U / L	Day 28	24/09/2010	16	.	0 - 40		
S145	112	JAIVAC-1	Dosage A	2	Alkaline Phosphatase	U / L	Day 28	24/09/2010	55	.	40 - 129		
S145	112	JAIVAC-1	Dosage A	2	Direct Bilirubin	mg / dl	Day 28	24/09/2010	0.15	.	0.00 - 0.30		
S145	112	JAIVAC-1	Dosage A	2	Gamma Glutamyl Transpeptidase	U / L	Day 28	24/09/2010	15	.	10 - 71		
S145	112	JAIVAC-1	Dosage A	2	Indirect Bilirubin	mg / dl	Day 28	24/09/2010	0.5	.	0.00 - 0.99		
S145	112	JAIVAC-1	Dosage A	2	Total Bilirubin	mg / dl	Day 28	24/09/2010	0.65	.	0.00 - 0.99		
S145	112	JAIVAC-1	Dosage A	2	Serum creatinine	mg / dl	Day 28	24/09/2010	0.9	.	0.7 - 1.2		
S145	112	JAIVAC-1	Dosage A	2	Potassium	mEq / L	Day 28	24/09/2010	4.3	.	3.5 - 5.1		
S145	112	JAIVAC-1	Dosage A	2	Random blood glucose	mg / dl	Day 28	24/09/2010	114	.	45-130		
S145	112	JAIVAC-1	Dosage A	2	Sodium	mEq / L	Day 28	24/09/2010	139	.	136 - 145		
S145	112	JAIVAC-1	Dosage A	2	ALT	U / L	Day 56	22/10/2010	21	.	0 - 41		
S145	112	JAIVAC-1	Dosage A	2	AST	U / L	Day 56	22/10/2010	16	.	0 - 40		
S145	112	JAIVAC-1	Dosage A	2	Alkaline Phosphatase	U / L	Day 56	22/10/2010	53	.	40 - 129		
S145	112	JAIVAC-1	Dosage A	2	Direct Bilirubin	mg / dl	Day 56	22/10/2010	0.12	.	0.00 - 0.30		

Subject No.	Randomisation Number	Study Group	Dosage	Dose	Serum Chemistry Parameters	Unit	Day	Date of sample Collection	Results	Out of Range	Reference Range	Flag	Clinical Significance*
S145	112	JAIVAC-1	Dosage A	2	Gamma Glutamyl Transpeptidase	U / L	Day 56	22/10/2010	18	.	10 - 71		
S145	112	JAIVAC-1	Dosage A	2	Indirect Bilirubin	mg / dl	Day 56	22/10/2010	0.36	.	0.00 - 0.99		
S145	112	JAIVAC-1	Dosage A	2	Total Bilirubin	mg / dl	Day 56	22/10/2010	0.48	.	0.00 - 0.99		
S145	112	JAIVAC-1	Dosage A	2	Serum creatinine	mg / dl	Day 56	22/10/2010	1	.	0.7 - 1.2		
S145	112	JAIVAC-1	Dosage A	2	Potassium	mEq / L	Day 56	22/10/2010	4.1	.	3.5 - 5.1		
S145	112	JAIVAC-1	Dosage A	2	Random blood glucose	mg / dl	Day 56	22/10/2010	95	.	45-130		
S145	112	JAIVAC-1	Dosage A	2	Sodium	mEq / L	Day 56	22/10/2010	140	.	136 - 145		
S145	112	JAIVAC-1	Dosage A	3	ALT	U / L	Day 180	23/02/2011	22	.	0 - 41		
S145	112	JAIVAC-1	Dosage A	3	AST	U / L	Day 180	23/02/2011	18	.	0 - 40		
S145	112	JAIVAC-1	Dosage A	3	Alkaline Phosphatase	U / L	Day 180	23/02/2011	62	.	40 - 129		
S145	112	JAIVAC-1	Dosage A	3	Direct Bilirubin	mg / dl	Day 180	23/02/2011	0.2	.	0.00 - 0.30		
S145	112	JAIVAC-1	Dosage A	3	Gamma Glutamyl Transpeptidase	U / L	Day 180	23/02/2011	21	.	10 - 71		
S145	112	JAIVAC-1	Dosage A	3	Indirect Bilirubin	mg / dl	Day 180	23/02/2011	0.34	.	0.00 - 0.99		
S145	112	JAIVAC-1	Dosage A	3	Total Bilirubin	mg / dl	Day 180	23/02/2011	0.54	.	0.00 - 0.99		
S145	112	JAIVAC-1	Dosage A	3	Serum creatinine	mg / dl	Day 180	23/02/2011	1	.	0.7 - 1.2		
S145	112	JAIVAC-1	Dosage A	3	Potassium	mEq / L	Day 180	23/02/2011	4.4	.	3.5 - 5.1		
S145	112	JAIVAC-1	Dosage A	3	Random blood glucose	mg / dl	Day 180	23/02/2011	98	.	45-130		
S145	112	JAIVAC-1	Dosage A	3	Sodium	mEq / L	Day 180	23/02/2011	141	.	136 - 145		
S145	112	JAIVAC-1	Dosage A	3	ALT	U / L	Day 208	23/03/2011	24	.	0 - 41		
S145	112	JAIVAC-1	Dosage A	3	AST	U / L	Day 208	23/03/2011	16	.	0 - 40		
S145	112	JAIVAC-1	Dosage A	3	Alkaline Phosphatase	U / L	Day 208	23/03/2011	60	.	40 - 129		
S145	112	JAIVAC-1	Dosage A	3	Direct Bilirubin	mg / dl	Day 208	23/03/2011	0.24	.	0.00 - 0.30		
S145	112	JAIVAC-1	Dosage A	3	Gamma Glutamyl Transpeptidase	U / L	Day 208	23/03/2011	19	.	10 - 71		
S145	112	JAIVAC-1	Dosage A	3	Indirect Bilirubin	mg / dl	Day 208	23/03/2011	0.42	.	0.00 - 0.99		
S145	112	JAIVAC-1	Dosage A	3	Total Bilirubin	mg / dl	Day 208	23/03/2011	0.66	.	0.00 - 0.99		
S145	112	JAIVAC-1	Dosage A	3	Serum creatinine	mg / dl	Day 208	23/03/2011	1	.	0.7 - 1.2		
S145	112	JAIVAC-1	Dosage A	3	Potassium	mEq / L	Day 208	23/03/2011	4.2	.	3.5 - 5.1		
S145	112	JAIVAC-1	Dosage A	3	Random blood glucose	mg / dl	Day 208	23/03/2011	98	.	45-130		
S145	112	JAIVAC-1	Dosage A	3	Sodium	mEq / L	Day 208	23/03/2011	140	.	136 - 145		

Subject No.	Randomisation Number	Study Group	Dosage	Dose	Serum Chemistry Parameters	Unit	Day	Date of sample Collection	Results	Out of Range	Reference Range	Flag	Clinical Significance*
S145	112	JAIVAC-1	Dosage A	3	ALT	U / L	Day 365	26/08/2011	20	.	0 - 41		
S145	112	JAIVAC-1	Dosage A	3	AST	U / L	Day 365	26/08/2011	17	.	0 - 40		
S145	112	JAIVAC-1	Dosage A	3	Alkaline Phosphatase	U / L	Day 365	26/08/2011	49	.	40 - 129		
S145	112	JAIVAC-1	Dosage A	3	Direct Bilirubin	mg / dl	Day 365	26/08/2011	0.33	Yes	0.00 - 0.30	H	NCS
S145	112	JAIVAC-1	Dosage A	3	Gamma Glutamyl Transpeptidase	U / L	Day 365	26/08/2011	17	.	10 - 71		
S145	112	JAIVAC-1	Dosage A	3	Indirect Bilirubin	mg / dl	Day 365	26/08/2011	0.6	.	0.00 - 0.99		
S145	112	JAIVAC-1	Dosage A	3	Total Bilirubin	mg / dl	Day 365	26/08/2011	0.93	.	0.00 - 0.99		
S145	112	JAIVAC-1	Dosage A	3	Serum creatinine	mg / dl	Day 365	26/08/2011	1.2	.	0.7 - 1.2		
S145	112	JAIVAC-1	Dosage A	3	Potassium	mEq / L	Day 365	26/08/2011	4.5	.	3.5 - 5.1		
S145	112	JAIVAC-1	Dosage A	3	Random blood glucose	mg / dl	Day 365	26/08/2011	105	.	45-130		
S145	112	JAIVAC-1	Dosage A	3	Sodium	mEq / L	Day 365	26/08/2011	141	.	136 - 145		
S148	111	JAIVAC-1	Dosage A		ALT	U / L	Day -14 to Day -1	20/08/2010	26	.	0 - 41		
S148	111	JAIVAC-1	Dosage A		AST	U / L	Day -14 to Day -1	20/08/2010	25	.	0 - 40		
S148	111	JAIVAC-1	Dosage A		Alkaline Phosphatase	U / L	Day -14 to Day -1	20/08/2010	90	.	40 - 129		
S148	111	JAIVAC-1	Dosage A		Direct Bilirubin	mg / dl	Day -14 to Day -1	20/08/2010	0.19	.	0.00 - 0.30		
S148	111	JAIVAC-1	Dosage A		Gamma Glutamyl Transpeptidase	U / L	Day -14 to Day -1	20/08/2010	18	.	10 - 71		
S148	111	JAIVAC-1	Dosage A		Indirect Bilirubin	mg / dl	Day -14 to Day -1	20/08/2010	0.31	.	0.00 - 0.99		
S148	111	JAIVAC-1	Dosage A		Total Bilirubin	mg / dl	Day -14 to Day -1	20/08/2010	0.5	.	0.00 - 0.99		
S148	111	JAIVAC-1	Dosage A		Serum creatinine	mg / dl	Day -14 to Day -1	20/08/2010	0.9	.	0.7 - 1.2		
S148	111	JAIVAC-1	Dosage A		Potassium	mEq / L	Day -14 to Day -1	20/08/2010	4.3	.	3.5 - 5.1		
S148	111	JAIVAC-1	Dosage A		Random blood glucose	mg / dl	Day -14 to Day -1	20/08/2010	80	.	45-130		
S148	111	JAIVAC-1	Dosage A		Sodium	mEq / L	Day -14 to Day -1	20/08/2010	142	.	136 - 145		
S148	111	JAIVAC-1	Dosage A	2	ALT	U / L	Day 28	24/09/2010	19	.	0 - 41		
S148	111	JAIVAC-1	Dosage A	2	AST	U / L	Day 28	24/09/2010	24	.	0 - 40		
S148	111	JAIVAC-1	Dosage A	2	Alkaline Phosphatase	U / L	Day 28	24/09/2010	102	.	40 - 129		
S148	111	JAIVAC-1	Dosage A	2	Direct Bilirubin	mg / dl	Day 28	24/09/2010	0.1	.	0.00 - 0.30		
S148	111	JAIVAC-1	Dosage A	2	Gamma Glutamyl Transpeptidase	U / L	Day 28	24/09/2010	18	.	10 - 71		
S148	111	JAIVAC-1	Dosage A	2	Indirect Bilirubin	mg / dl	Day 28	24/09/2010	0.31	.	0.00 - 0.99		
S148	111	JAIVAC-1	Dosage A	2	Total Bilirubin	mg / dl	Day 28	24/09/2010	0.41	.	0.00 - 0.99		

Subject No.	Randomisation Number	Study Group	Dosage	Dose	Serum Chemistry Parameters	Unit	Day	Date of sample Collection	Results	Out of Range	Reference Range	Flag	Clinical Significance*
S148	111	JAIVAC-1	Dosage A	2	Serum creatinine	mg / dl	Day 28	24/09/2010	0.9	.	0.7 - 1.2		
S148	111	JAIVAC-1	Dosage A	2	Potassium	mEq / L	Day 28	24/09/2010	4.1	.	3.5 - 5.1		
S148	111	JAIVAC-1	Dosage A	2	Random blood glucose	mg / dl	Day 28	24/09/2010	95	.	45-130		
S148	111	JAIVAC-1	Dosage A	2	Sodium	mEq / L	Day 28	24/09/2010	142	.	136 - 145		
S148	111	JAIVAC-1	Dosage A	2	ALT	U / L	Day 56	22/10/2010	25	.	0 - 41		
S148	111	JAIVAC-1	Dosage A	2	AST	U / L	Day 56	22/10/2010	26	.	0 - 40		
S148	111	JAIVAC-1	Dosage A	2	Alkaline Phosphatase	U / L	Day 56	22/10/2010	100	.	40 - 129		
S148	111	JAIVAC-1	Dosage A	2	Direct Bilirubin	mg / dl	Day 56	22/10/2010	<0.10	.	0.00 - 0.30		
S148	111	JAIVAC-1	Dosage A	2	Gamma Glutamyl Transpeptidase	U / L	Day 56	22/10/2010	18	.	10 - 71		
S148	111	JAIVAC-1	Dosage A	2	Indirect Bilirubin	mg / dl	Day 56	22/10/2010	NA	.	0.00 - 0.99		
S148	111	JAIVAC-1	Dosage A	2	Total Bilirubin	mg / dl	Day 56	22/10/2010	0.22	.	0.00 - 0.99		
S148	111	JAIVAC-1	Dosage A	2	Serum creatinine	mg / dl	Day 56	22/10/2010	0.9	.	0.7 - 1.2		
S148	111	JAIVAC-1	Dosage A	2	Potassium	mEq / L	Day 56	22/10/2010	4.2	.	3.5 - 5.1		
S148	111	JAIVAC-1	Dosage A	2	Random blood glucose	mg / dl	Day 56	22/10/2010	81	.	45-130		
S148	111	JAIVAC-1	Dosage A	2	Sodium	mEq / L	Day 56	22/10/2010	142	.	136 - 145		
S148	111	JAIVAC-1	Dosage A	3	ALT	U / L	Day 180	23/02/2011	27	.	0 - 41		
S148	111	JAIVAC-1	Dosage A	3	AST	U / L	Day 180	23/02/2011	24	.	0 - 40		
S148	111	JAIVAC-1	Dosage A	3	Alkaline Phosphatase	U / L	Day 180	23/02/2011	121	.	40 - 129		
S148	111	JAIVAC-1	Dosage A	3	Direct Bilirubin	mg / dl	Day 180	23/02/2011	0.12	.	0.00 - 0.30		
S148	111	JAIVAC-1	Dosage A	3	Gamma Glutamyl Transpeptidase	U / L	Day 180	23/02/2011	29	.	10 - 71		
S148	111	JAIVAC-1	Dosage A	3	Indirect Bilirubin	mg / dl	Day 180	23/02/2011	0.17	.	0.00 - 0.99		
S148	111	JAIVAC-1	Dosage A	3	Total Bilirubin	mg / dl	Day 180	23/02/2011	0.29	.	0.00 - 0.99		
S148	111	JAIVAC-1	Dosage A	3	Serum creatinine	mg / dl	Day 180	23/02/2011	1	.	0.7 - 1.2		
S148	111	JAIVAC-1	Dosage A	3	Potassium	mEq / L	Day 180	23/02/2011	4.7	.	3.5 - 5.1		
S148	111	JAIVAC-1	Dosage A	3	Random blood glucose	mg / dl	Day 180	23/02/2011	94	.	45-130		
S148	111	JAIVAC-1	Dosage A	3	Sodium	mEq / L	Day 180	23/02/2011	141	.	136 - 145		
S148	111	JAIVAC-1	Dosage A	3	ALT	U / L	Day 208	23/03/2011	23	.	0 - 41		
S148	111	JAIVAC-1	Dosage A	3	AST	U / L	Day 208	23/03/2011	24	.	0 - 40		
S148	111	JAIVAC-1	Dosage A	3	Alkaline Phosphatase	U / L	Day 208	23/03/2011	116	.	40 - 129		

Subject No.	Randomisation Number	Study Group	Dosage	Dose	Serum Chemistry Parameters	Unit	Day	Date of sample Collection	Results	Out of Range	Reference Range	Flag	Clinical Significance*
S148	111	JAIVAC-1	Dosage A	3	Direct Bilirubin	mg / dl	Day 208	23/03/2011	0.15	.	0.00 - 0.30		
S148	111	JAIVAC-1	Dosage A	3	Gamma Glutamyl Transpeptidase	U / L	Day 208	23/03/2011	25	.	10 - 71		
S148	111	JAIVAC-1	Dosage A	3	Indirect Bilirubin	mg / dl	Day 208	23/03/2011	0.24	.	0.00 - 0.99		
S148	111	JAIVAC-1	Dosage A	3	Total Bilirubin	mg / dl	Day 208	23/03/2011	0.39	.	0.00 - 0.99		
S148	111	JAIVAC-1	Dosage A	3	Serum creatinine	mg / dl	Day 208	23/03/2011	1	.	0.7 - 1.2		
S148	111	JAIVAC-1	Dosage A	3	Potassium	mEq / L	Day 208	23/03/2011	4.3	.	3.5 - 5.1		
S148	111	JAIVAC-1	Dosage A	3	Random blood glucose	mg / dl	Day 208	23/03/2011	85	.	45-130		
S148	111	JAIVAC-1	Dosage A	3	Sodium	mEq / L	Day 208	23/03/2011	141	.	136 - 145		
S148	111	JAIVAC-1	Dosage A	3	ALT	U / L	Day 365	26/08/2011	26	.	0 - 41		
S148	111	JAIVAC-1	Dosage A	3	AST	U / L	Day 365	26/08/2011	24	.	0 - 40		
S148	111	JAIVAC-1	Dosage A	3	Alkaline Phosphatase	U / L	Day 365	26/08/2011	101	.	40 - 129		
S148	111	JAIVAC-1	Dosage A	3	Direct Bilirubin	mg / dl	Day 365	26/08/2011	0.12	.	0.00 - 0.30		
S148	111	JAIVAC-1	Dosage A	3	Gamma Glutamyl Transpeptidase	U / L	Day 365	26/08/2011	25	.	10 - 71		
S148	111	JAIVAC-1	Dosage A	3	Indirect Bilirubin	mg / dl	Day 365	26/08/2011	0.18	.	0.00 - 0.99		
S148	111	JAIVAC-1	Dosage A	3	Total Bilirubin	mg / dl	Day 365	26/08/2011	0.3	.	0.00 - 0.99		
S148	111	JAIVAC-1	Dosage A	3	Serum creatinine	mg / dl	Day 365	26/08/2011	1.1	.	0.7 - 1.2		
S148	111	JAIVAC-1	Dosage A	3	Potassium	mEq / L	Day 365	26/08/2011	4.4	.	3.5 - 5.1		
S148	111	JAIVAC-1	Dosage A	3	Random blood glucose	mg / dl	Day 365	26/08/2011	94	.	45-130		
S148	111	JAIVAC-1	Dosage A	3	Sodium	mEq / L	Day 365	26/08/2011	143	.	136 - 145		
S154	113	JAIVAC-1	Dosage A		ALT	U / L	Day -14 to Day -1	19/08/2010	16	.	0 - 41		
S154	113	JAIVAC-1	Dosage A		AST	U / L	Day -14 to Day -1	19/08/2010	20	.	0 - 40		
S154	113	JAIVAC-1	Dosage A		Alkaline Phosphatase	U / L	Day -14 to Day -1	19/08/2010	56	.	40 - 129		
S154	113	JAIVAC-1	Dosage A		Direct Bilirubin	mg / dl	Day -14 to Day -1	19/08/2010	0.17	.	0.00 - 0.30		
S154	113	JAIVAC-1	Dosage A		Gamma Glutamyl Transpeptidase	U / L	Day -14 to Day -1	19/08/2010	15	.	10 - 71		
S154	113	JAIVAC-1	Dosage A		Indirect Bilirubin	mg / dl	Day -14 to Day -1	19/08/2010	0.32	.	0.00 - 0.99		
S154	113	JAIVAC-1	Dosage A		Total Bilirubin	mg / dl	Day -14 to Day -1	19/08/2010	0.49	.	0.00 - 0.99		
S154	113	JAIVAC-1	Dosage A		Serum creatinine	mg / dl	Day -14 to Day -1	19/08/2010	1	.	0.7 - 1.2		
S154	113	JAIVAC-1	Dosage A		Potassium	mEq / L	Day -14 to Day -1	19/08/2010	4.3	.	3.5 - 5.1		
S154	113	JAIVAC-1	Dosage A		Random blood glucose	mg / dl	Day -14 to Day -1	19/08/2010	76	.	45-130		

Subject No.	Randomisation Number	Study Group	Dosage	Dose	Serum Chemistry Parameters	Unit	Day	Date of sample Collection	Results	Out of Range	Reference Range	Flag	Clinical Significance*
S154	113	JAIVAC-1	Dosage A		Sodium	mEq / L	Day -14 to Day -1	19/08/2010	139	.	136 - 145		
S154	113	JAIVAC-1	Dosage A	2	ALT	U / L	Day 28	24/09/2010	21	.	0 - 41		
S154	113	JAIVAC-1	Dosage A	2	AST	U / L	Day 28	24/09/2010	18	.	0 - 40		
S154	113	JAIVAC-1	Dosage A	2	Alkaline Phosphatase	U / L	Day 28	24/09/2010	57	.	40 - 129		
S154	113	JAIVAC-1	Dosage A	2	Direct Bilirubin	mg / dl	Day 28	24/09/2010	0.1	.	0.00 - 0.30		
S154	113	JAIVAC-1	Dosage A	2	Gamma Glutamyl Transpeptidase	U / L	Day 28	24/09/2010	18	.	10 - 71		
S154	113	JAIVAC-1	Dosage A	2	Indirect Bilirubin	mg / dl	Day 28	24/09/2010	0.29	.	0.00 - 0.99		
S154	113	JAIVAC-1	Dosage A	2	Total Bilirubin	mg / dl	Day 28	24/09/2010	0.39	.	0.00 - 0.99		
S154	113	JAIVAC-1	Dosage A	2	Serum creatinine	mg / dl	Day 28	24/09/2010	1.1	.	0.7 - 1.2		
S154	113	JAIVAC-1	Dosage A	2	Potassium	mEq / L	Day 28	24/09/2010	4.5	.	3.5 - 5.1		
S154	113	JAIVAC-1	Dosage A	2	Random blood glucose	mg / dl	Day 28	24/09/2010	103	.	45-130		
S154	113	JAIVAC-1	Dosage A	2	Sodium	mEq / L	Day 28	24/09/2010	140	.	136 - 145		
S154	113	JAIVAC-1	Dosage A	2	ALT	U / L	Day 56	22/10/2010	16	.	0 - 41		
S154	113	JAIVAC-1	Dosage A	2	AST	U / L	Day 56	22/10/2010	21	.	0 - 40		
S154	113	JAIVAC-1	Dosage A	2	Alkaline Phosphatase	U / L	Day 56	22/10/2010	54	.	40 - 129		
S154	113	JAIVAC-1	Dosage A	2	Direct Bilirubin	mg / dl	Day 56	22/10/2010	0.12	.	0.00 - 0.30		
S154	113	JAIVAC-1	Dosage A	2	Gamma Glutamyl Transpeptidase	U / L	Day 56	22/10/2010	18	.	10 - 71		
S154	113	JAIVAC-1	Dosage A	2	Indirect Bilirubin	mg / dl	Day 56	22/10/2010	0.25	.	0.00 - 0.99		
S154	113	JAIVAC-1	Dosage A	2	Total Bilirubin	mg / dl	Day 56	22/10/2010	0.37	.	0.00 - 0.99		
S154	113	JAIVAC-1	Dosage A	2	Serum creatinine	mg / dl	Day 56	22/10/2010	1.1	.	0.7 - 1.2		
S154	113	JAIVAC-1	Dosage A	2	Potassium	mEq / L	Day 56	22/10/2010	4.6	.	3.5 - 5.1		
S154	113	JAIVAC-1	Dosage A	2	Random blood glucose	mg / dl	Day 56	22/10/2010	84	.	45-130		
S154	113	JAIVAC-1	Dosage A	2	Sodium	mEq / L	Day 56	22/10/2010	139	.	136 - 145		
S154	113	JAIVAC-1	Dosage A	3	ALT	U / L	Day 180	23/02/2011	21	.	0 - 41		
S154	113	JAIVAC-1	Dosage A	3	AST	U / L	Day 180	23/02/2011	22	.	0 - 40		
S154	113	JAIVAC-1	Dosage A	3	Alkaline Phosphatase	U / L	Day 180	23/02/2011	57	.	40 - 129		
S154	113	JAIVAC-1	Dosage A	3	Direct Bilirubin	mg / dl	Day 180	23/02/2011	0.14	.	0.00 - 0.30		
S154	113	JAIVAC-1	Dosage A	3	Gamma Glutamyl Transpeptidase	U / L	Day 180	23/02/2011	18	.	10 - 71		
S154	113	JAIVAC-1	Dosage A	3	Indirect Bilirubin	mg / dl	Day 180	23/02/2011	0.23	.	0.00 - 0.99		

Subject No.	Randomisation Number	Study Group	Dosage	Dose	Serum Chemistry Parameters	Unit	Day	Date of sample Collection	Results	Out of Range	Reference Range	Flag	Clinical Significance*
S154	113	JAIVAC-1	Dosage A	3	Total Bilirubin	mg / dl	Day 180	23/02/2011	0.37	.	0.00 - 0.99		
S154	113	JAIVAC-1	Dosage A	3	Serum creatinine	mg / dl	Day 180	23/02/2011	1.1	.	0.7 - 1.2		
S154	113	JAIVAC-1	Dosage A	3	Potassium	mEq / L	Day 180	23/02/2011	4.1	.	3.5 - 5.1		
S154	113	JAIVAC-1	Dosage A	3	Random blood glucose	mg / dl	Day 180	23/02/2011	80	.	45-130		
S154	113	JAIVAC-1	Dosage A	3	Sodium	mEq / L	Day 180	23/02/2011	138	.	136 - 145		
S154	113	JAIVAC-1	Dosage A	3	ALT	U / L	Day 208	23/03/2011	21	.	0 - 41		
S154	113	JAIVAC-1	Dosage A	3	AST	U / L	Day 208	23/03/2011	21	.	0 - 40		
S154	113	JAIVAC-1	Dosage A	3	Alkaline Phosphatase	U / L	Day 208	23/03/2011	53	.	40 - 129		
S154	113	JAIVAC-1	Dosage A	3	Direct Bilirubin	mg / dl	Day 208	23/03/2011	0.15	.	0.00 - 0.30		
S154	113	JAIVAC-1	Dosage A	3	Gamma Glutamyl Transpeptidase	U / L	Day 208	23/03/2011	19	.	10 - 71		
S154	113	JAIVAC-1	Dosage A	3	Indirect Bilirubin	mg / dl	Day 208	23/03/2011	0.34	.	0.00 - 0.99		
S154	113	JAIVAC-1	Dosage A	3	Total Bilirubin	mg / dl	Day 208	23/03/2011	0.49	.	0.00 - 0.99		
S154	113	JAIVAC-1	Dosage A	3	Serum creatinine	mg / dl	Day 208	23/03/2011	1.1	.	0.7 - 1.2		
S154	113	JAIVAC-1	Dosage A	3	Potassium	mEq / L	Day 208	23/03/2011	4.4	.	3.5 - 5.1		
S154	113	JAIVAC-1	Dosage A	3	Random blood glucose	mg / dl	Day 208	23/03/2011	67	.	45-130		
S154	113	JAIVAC-1	Dosage A	3	Sodium	mEq / L	Day 208	23/03/2011	139	.	136 - 145		
S154	113	JAIVAC-1	Dosage A	3	ALT	U / L	Day 365	26/08/2011	20	.	0 - 41		
S154	113	JAIVAC-1	Dosage A	3	AST	U / L	Day 365	26/08/2011	23	.	0 - 40		
S154	113	JAIVAC-1	Dosage A	3	Alkaline Phosphatase	U / L	Day 365	26/08/2011	55	.	40 - 129		
S154	113	JAIVAC-1	Dosage A	3	Direct Bilirubin	mg / dl	Day 365	26/08/2011	0.13	.	0.00 - 0.30		
S154	113	JAIVAC-1	Dosage A	3	Gamma Glutamyl Transpeptidase	U / L	Day 365	26/08/2011	21	.	10 - 71		
S154	113	JAIVAC-1	Dosage A	3	Indirect Bilirubin	mg / dl	Day 365	26/08/2011	0.33	.	0.00 - 0.99		
S154	113	JAIVAC-1	Dosage A	3	Total Bilirubin	mg / dl	Day 365	26/08/2011	0.46	.	0.00 - 0.99		
S154	113	JAIVAC-1	Dosage A	3	Serum creatinine	mg / dl	Day 365	26/08/2011	1.2	.	0.7 - 1.2		
S154	113	JAIVAC-1	Dosage A	3	Potassium	mEq / L	Day 365	26/08/2011	4.8	.	3.5 - 5.1		
S154	113	JAIVAC-1	Dosage A	3	Random blood glucose	mg / dl	Day 365	26/08/2011	84	.	45-130		
S154	113	JAIVAC-1	Dosage A	3	Sodium	mEq / L	Day 365	26/08/2011	140	.	136 - 145		
S156	110	JAIVAC-1	Dosage A		ALT	U / L	Day -14 to Day -1	19/08/2010	14	.	0 - 41		
S156	110	JAIVAC-1	Dosage A		AST	U / L	Day -14 to Day -1	19/08/2010	22	.	0 - 40		

Subject No.	Randomisation Number	Study Group	Dosage	Dose	Serum Chemistry Parameters	Unit	Day	Date of sample Collection	Results	Out of Range	Reference Range	Flag	Clinical Significance*
S156	110	JAIVAC-1	Dosage A		Alkaline Phosphatase	U / L	Day -14 to Day -1	19/08/2010	101	.	40 - 129		
S156	110	JAIVAC-1	Dosage A		Direct Bilirubin	mg / dl	Day -14 to Day -1	19/08/2010	0.19	.	0.00 - 0.30		
S156	110	JAIVAC-1	Dosage A		Gamma Glutamyl Transpeptidase	U / L	Day -14 to Day -1	19/08/2010	13	.	10 - 71		
S156	110	JAIVAC-1	Dosage A		Indirect Bilirubin	mg / dl	Day -14 to Day -1	19/08/2010	0.34	.	0.00 - 0.99		
S156	110	JAIVAC-1	Dosage A		Total Bilirubin	mg / dl	Day -14 to Day -1	19/08/2010	0.53	.	0.00 - 0.99		
S156	110	JAIVAC-1	Dosage A		Serum creatinine	mg / dl	Day -14 to Day -1	19/08/2010	0.8	.	0.7 - 1.2		
S156	110	JAIVAC-1	Dosage A		Potassium	mEq / L	Day -14 to Day -1	19/08/2010	4.9	.	3.5 - 5.1		
S156	110	JAIVAC-1	Dosage A		Random blood glucose	mg / dl	Day -14 to Day -1	19/08/2010	81	.	45-130		
S156	110	JAIVAC-1	Dosage A		Sodium	mEq / L	Day -14 to Day -1	19/08/2010	141	.	136 - 145		
S156	110	JAIVAC-1	Dosage A	2	ALT	U / L	Day 28	24/09/2010	18	.	0 - 41		
S156	110	JAIVAC-1	Dosage A	2	AST	U / L	Day 28	24/09/2010	24	.	0 - 40		
S156	110	JAIVAC-1	Dosage A	2	Alkaline Phosphatase	U / L	Day 28	24/09/2010	106	.	40 - 129		
S156	110	JAIVAC-1	Dosage A	2	Direct Bilirubin	mg / dl	Day 28	24/09/2010	0.19	.	0.00 - 0.30		
S156	110	JAIVAC-1	Dosage A	2	Gamma Glutamyl Transpeptidase	U / L	Day 28	24/09/2010	11	.	10 - 71		
S156	110	JAIVAC-1	Dosage A	2	Indirect Bilirubin	mg / dl	Day 28	24/09/2010	0.5	.	0.00 - 0.99		
S156	110	JAIVAC-1	Dosage A	2	Total Bilirubin	mg / dl	Day 28	24/09/2010	0.69	.	0.00 - 0.99		
S156	110	JAIVAC-1	Dosage A	2	Serum creatinine	mg / dl	Day 28	24/09/2010	0.7	.	0.7 - 1.2		
S156	110	JAIVAC-1	Dosage A	2	Potassium	mEq / L	Day 28	24/09/2010	4.2	.	3.5 - 5.1		
S156	110	JAIVAC-1	Dosage A	2	Random blood glucose	mg / dl	Day 28	24/09/2010	71	.	45-130		
S156	110	JAIVAC-1	Dosage A	2	Sodium	mEq / L	Day 28	24/09/2010	140	.	136 - 145		
S156	110	JAIVAC-1	Dosage A	2	ALT	U / L	Day 56	22/10/2010	15	.	0 - 41		
S156	110	JAIVAC-1	Dosage A	2	AST	U / L	Day 56	22/10/2010	21	.	0 - 40		
S156	110	JAIVAC-1	Dosage A	2	Alkaline Phosphatase	U / L	Day 56	22/10/2010	99	.	40 - 129		
S156	110	JAIVAC-1	Dosage A	2	Direct Bilirubin	mg / dl	Day 56	22/10/2010	0.23	.	0.00 - 0.30		
S156	110	JAIVAC-1	Dosage A	2	Gamma Glutamyl Transpeptidase	U / L	Day 56	22/10/2010	10	.	10 - 71		
S156	110	JAIVAC-1	Dosage A	2	Indirect Bilirubin	mg / dl	Day 56	22/10/2010	0.51	.	0.00 - 0.99		
S156	110	JAIVAC-1	Dosage A	2	Total Bilirubin	mg / dl	Day 56	22/10/2010	0.74	.	0.00 - 0.99		
S156	110	JAIVAC-1	Dosage A	2	Serum creatinine	mg / dl	Day 56	22/10/2010	0.8	.	0.7 - 1.2		
S156	110	JAIVAC-1	Dosage A	2	Potassium	mEq / L	Day 56	22/10/2010	4.8	.	3.5 - 5.1		

Subject No.	Randomisation Number	Study Group	Dosage	Dose	Serum Chemistry Parameters	Unit	Day	Date of sample Collection	Results	Out of Range	Reference Range	Flag	Clinical Significance*
S156	110	JAIVAC-1	Dosage A	2	Random blood glucose	mg / dl	Day 56	22/10/2010	56	.	45-130		
S156	110	JAIVAC-1	Dosage A	2	Sodium	mEq / L	Day 56	22/10/2010	141	.	136 - 145		
S156	110	JAIVAC-1	Dosage A	3	ALT	U / L	Day 180	23/02/2011	15	.	0 - 41		
S156	110	JAIVAC-1	Dosage A	3	AST	U / L	Day 180	23/02/2011	21	.	0 - 40		
S156	110	JAIVAC-1	Dosage A	3	Alkaline Phosphatase	U / L	Day 180	23/02/2011	90	.	40 - 129		
S156	110	JAIVAC-1	Dosage A	3	Direct Bilirubin	mg / dl	Day 180	23/02/2011	0.27	.	0.00 - 0.30		
S156	110	JAIVAC-1	Dosage A	3	Gamma Glutamyl Transpeptidase	U / L	Day 180	23/02/2011	12	.	10 - 71		
S156	110	JAIVAC-1	Dosage A	3	Indirect Bilirubin	mg / dl	Day 180	23/02/2011	0.6	.	0.00 - 0.99		
S156	110	JAIVAC-1	Dosage A	3	Total Bilirubin	mg / dl	Day 180	23/02/2011	0.87	.	0.00 - 0.99		
S156	110	JAIVAC-1	Dosage A	3	Serum creatinine	mg / dl	Day 180	23/02/2011	0.9	.	0.7 - 1.2		
S156	110	JAIVAC-1	Dosage A	3	Potassium	mEq / L	Day 180	23/02/2011	4.8	.	3.5 - 5.1		
S156	110	JAIVAC-1	Dosage A	3	Random blood glucose	mg / dl	Day 180	23/02/2011	62	.	45-130		
S156	110	JAIVAC-1	Dosage A	3	Sodium	mEq / L	Day 180	23/02/2011	141	.	136 - 145		
S156	110	JAIVAC-1	Dosage A	3	ALT	U / L	Day 208	23/03/2011	13	.	0 - 41		
S156	110	JAIVAC-1	Dosage A	3	AST	U / L	Day 208	23/03/2011	19	.	0 - 40		
S156	110	JAIVAC-1	Dosage A	3	Alkaline Phosphatase	U / L	Day 208	23/03/2011	79	.	40 - 129		
S156	110	JAIVAC-1	Dosage A	3	Direct Bilirubin	mg / dl	Day 208	23/03/2011	0.34	Yes	0.00 - 0.30	H	NCS
S156	110	JAIVAC-1	Dosage A	3	Gamma Glutamyl Transpeptidase	U / L	Day 208	23/03/2011	15	.	10 - 71		
S156	110	JAIVAC-1	Dosage A	3	Indirect Bilirubin	mg / dl	Day 208	23/03/2011	0.75	.	0.00 - 0.99		
S156	110	JAIVAC-1	Dosage A	3	Total Bilirubin	mg / dl	Day 208	23/03/2011	1.09	Yes	0.00 - 0.99	H	NCS
S156	110	JAIVAC-1	Dosage A	3	Serum creatinine	mg / dl	Day 208	23/03/2011	0.9	.	0.7 - 1.2		
S156	110	JAIVAC-1	Dosage A	3	Potassium	mEq / L	Day 208	23/03/2011	4.5	.	3.5 - 5.1		
S156	110	JAIVAC-1	Dosage A	3	Random blood glucose	mg / dl	Day 208	23/03/2011	83	.	45-130		
S156	110	JAIVAC-1	Dosage A	3	Sodium	mEq / L	Day 208	23/03/2011	140	.	136 - 145		
S156	110	JAIVAC-1	Dosage A	3	ALT	U / L	Day 365	26/08/2011	13	.	0 - 41		
S156	110	JAIVAC-1	Dosage A	3	AST	U / L	Day 365	26/08/2011	20	.	0 - 40		
S156	110	JAIVAC-1	Dosage A	3	Alkaline Phosphatase	U / L	Day 365	26/08/2011	74	.	40 - 129		
S156	110	JAIVAC-1	Dosage A	3	Direct Bilirubin	mg / dl	Day 365	26/08/2011	0.25	.	0.00 - 0.30		
S156	110	JAIVAC-1	Dosage A	3	Gamma Glutamyl Transpeptidase	U / L	Day 365	26/08/2011	12	.	10 - 71		

Subject No.	Randomisation Number	Study Group	Dosage	Dose	Serum Chemistry Parameters	Unit	Day	Date of sample Collection	Results	Out of Range	Reference Range	Flag	Clinical Significance*
S156	110	JAIVAC-1	Dosage A	3	Indirect Bilirubin	mg / dl	Day 365	26/08/2011	0.49	.	0.00 - 0.99		
S156	110	JAIVAC-1	Dosage A	3	Total Bilirubin	mg / dl	Day 365	26/08/2011	0.74	.	0.00 - 0.99		
S156	110	JAIVAC-1	Dosage A	3	Serum creatinine	mg / dl	Day 365	26/08/2011	1	.	0.7 - 1.2		
S156	110	JAIVAC-1	Dosage A	3	Potassium	mEq / L	Day 365	26/08/2011	4.6	.	3.5 - 5.1		
S156	110	JAIVAC-1	Dosage A	3	Random blood glucose	mg / dl	Day 365	26/08/2011	84	.	45-130		
S156	110	JAIVAC-1	Dosage A	3	Sodium	mEq / L	Day 365	26/08/2011	140	.	136 - 145		
S170	204	Hepatitis B	Hepatitis B		ALT	U / L	Day -14 to Day -1	21/09/2010	13	.	0 - 41		
S170	204	Hepatitis B	Hepatitis B		AST	U / L	Day -14 to Day -1	21/09/2010	20	.	0 - 40		
S170	204	Hepatitis B	Hepatitis B		Alkaline Phosphatase	U / L	Day -14 to Day -1	21/09/2010	62	.	40 - 129		
S170	204	Hepatitis B	Hepatitis B		Direct Bilirubin	mg / dl	Day -14 to Day -1	21/09/2010	0.29	.	0.00 - 0.30		
S170	204	Hepatitis B	Hepatitis B		Gamma Glutamyl Transpeptidase	U / L	Day -14 to Day -1	21/09/2010	12	.	10 - 71		
S170	204	Hepatitis B	Hepatitis B		Indirect Bilirubin	mg / dl	Day -14 to Day -1	21/09/2010	0.54	.	0.00 - 0.99		
S170	204	Hepatitis B	Hepatitis B		Total Bilirubin	mg / dl	Day -14 to Day -1	21/09/2010	0.83	.	0.00 - 0.99		
S170	204	Hepatitis B	Hepatitis B		Serum creatinine	mg / dl	Day -14 to Day -1	21/09/2010	1	.	0.7 - 1.2		
S170	204	Hepatitis B	Hepatitis B		Potassium	mEq / L	Day -14 to Day -1	21/09/2010	4.3	.	3.5 - 5.1		
S170	204	Hepatitis B	Hepatitis B		Random blood glucose	mg / dl	Day -14 to Day -1	21/09/2010	86	.	45-130		
S170	204	Hepatitis B	Hepatitis B		Sodium	mEq / L	Day -14 to Day -1	21/09/2010	139	.	136 - 145		
S170	204	Hepatitis B	Hepatitis B	2	ALT	U / L	Day 28	27/10/2010	15	.	0 - 41		
S170	204	Hepatitis B	Hepatitis B	2	AST	U / L	Day 28	27/10/2010	23	.	0 - 40		
S170	204	Hepatitis B	Hepatitis B	2	Alkaline Phosphatase	U / L	Day 28	27/10/2010	70	.	40 - 129		
S170	204	Hepatitis B	Hepatitis B	2	Direct Bilirubin	mg / dl	Day 28	27/10/2010	0.12	.	0.00 - 0.30		
S170	204	Hepatitis B	Hepatitis B	2	Gamma Glutamyl Transpeptidase	U / L	Day 28	27/10/2010	12	.	10 - 71		
S170	204	Hepatitis B	Hepatitis B	2	Indirect Bilirubin	mg / dl	Day 28	27/10/2010	0.2	.	0.00 - 0.99		
S170	204	Hepatitis B	Hepatitis B	2	Total Bilirubin	mg / dl	Day 28	27/10/2010	0.32	.	0.00 - 0.99		
S170	204	Hepatitis B	Hepatitis B	2	Serum creatinine	mg / dl	Day 28	27/10/2010	0.9	.	0.7 - 1.2		
S170	204	Hepatitis B	Hepatitis B	2	Potassium	mEq / L	Day 28	27/10/2010	4.1	.	3.5 - 5.1		
S170	204	Hepatitis B	Hepatitis B	2	Random blood glucose	mg / dl	Day 28	27/10/2010	55	.	45-130		
S170	204	Hepatitis B	Hepatitis B	2	Sodium	mEq / L	Day 28	27/10/2010	143	.	136 - 145		
S170	204	Hepatitis B	Hepatitis B	2	ALT	U / L	Day 56	25/11/2010	18	.	0 - 41		

Subject No.	Randomisation Number	Study Group	Dosage	Dose	Serum Chemistry Parameters	Unit	Day	Date of sample Collection	Results	Out of Range	Reference Range	Flag	Clinical Significance*
S170	204	Hepatitis B	Hepatitis B	2	AST	U / L	Day 56	25/11/2010	26	.	0 - 40		
S170	204	Hepatitis B	Hepatitis B	2	Alkaline Phosphatase	U / L	Day 56	25/11/2010	68	.	40 - 129		
S170	204	Hepatitis B	Hepatitis B	2	Direct Bilirubin	mg / dl	Day 56	25/11/2010	0.27	.	0.00 - 0.30		
S170	204	Hepatitis B	Hepatitis B	2	Gamma Glutamyl Transpeptidase	U / L	Day 56	25/11/2010	12	.	10 - 71		
S170	204	Hepatitis B	Hepatitis B	2	Indirect Bilirubin	mg / dl	Day 56	25/11/2010	0.57	.	0.00 - 0.99		
S170	204	Hepatitis B	Hepatitis B	2	Total Bilirubin	mg / dl	Day 56	25/11/2010	0.84	.	0.00 - 0.99		
S170	204	Hepatitis B	Hepatitis B	2	Serum creatinine	mg / dl	Day 56	25/11/2010	0.9	.	0.7 - 1.2		
S170	204	Hepatitis B	Hepatitis B	2	Potassium	mEq / L	Day 56	25/11/2010	4.5	.	3.5 - 5.1		
S170	204	Hepatitis B	Hepatitis B	2	Random blood glucose	mg / dl	Day 56	25/11/2010	90	.	45-130		
S170	204	Hepatitis B	Hepatitis B	2	Sodium	mEq / L	Day 56	25/11/2010	139	.	136 - 145		
S170	204	Hepatitis B	Hepatitis B	3	ALT	U / L	Day 180	28/03/2011	17	.	0 - 41		
S170	204	Hepatitis B	Hepatitis B	3	AST	U / L	Day 180	28/03/2011	20	.	0 - 40		
S170	204	Hepatitis B	Hepatitis B	3	Alkaline Phosphatase	U / L	Day 180	28/03/2011	62	.	40 - 129		
S170	204	Hepatitis B	Hepatitis B	3	Direct Bilirubin	mg / dl	Day 180	28/03/2011	0.17	.	0.00 - 0.30		
S170	204	Hepatitis B	Hepatitis B	3	Gamma Glutamyl Transpeptidase	U / L	Day 180	28/03/2011	16	.	10 - 71		
S170	204	Hepatitis B	Hepatitis B	3	Indirect Bilirubin	mg / dl	Day 180	28/03/2011	0.28	.	0.00 - 0.99		
S170	204	Hepatitis B	Hepatitis B	3	Total Bilirubin	mg / dl	Day 180	28/03/2011	0.45	.	0.00 - 0.99		
S170	204	Hepatitis B	Hepatitis B	3	Serum creatinine	mg / dl	Day 180	28/03/2011	1.1	.	0.7 - 1.2		
S170	204	Hepatitis B	Hepatitis B	3	Potassium	mEq / L	Day 180	28/03/2011	4.1	.	3.5 - 5.1		
S170	204	Hepatitis B	Hepatitis B	3	Random blood glucose	mg / dl	Day 180	28/03/2011	72	.	45-130		
S170	204	Hepatitis B	Hepatitis B	3	Sodium	mEq / L	Day 180	28/03/2011	142	.	136 - 145		
S170	204	Hepatitis B	Hepatitis B	3	ALT	U / L	Day 208	25/04/2011	16	.	0 - 41		
S170	204	Hepatitis B	Hepatitis B	3	AST	U / L	Day 208	25/04/2011	20	.	0 - 40		
S170	204	Hepatitis B	Hepatitis B	3	Alkaline Phosphatase	U / L	Day 208	25/04/2011	58	.	40 - 129		
S170	204	Hepatitis B	Hepatitis B	3	Direct Bilirubin	mg / dl	Day 208	25/04/2011	0.17	.	0.00 - 0.30		
S170	204	Hepatitis B	Hepatitis B	3	Gamma Glutamyl Transpeptidase	U / L	Day 208	25/04/2011	15	.	10 - 71		
S170	204	Hepatitis B	Hepatitis B	3	Indirect Bilirubin	mg / dl	Day 208	25/04/2011	0.29	.	0.00 - 0.99		
S170	204	Hepatitis B	Hepatitis B	3	Total Bilirubin	mg / dl	Day 208	25/04/2011	0.46	.	0.00 - 0.99		
S170	204	Hepatitis B	Hepatitis B	3	Serum creatinine	mg / dl	Day 208	25/04/2011	1	.	0.7 - 1.2		

Subject No.	Randomisation Number	Study Group	Dosage	Dose	Serum Chemistry Parameters	Unit	Day	Date of sample Collection	Results	Out of Range	Reference Range	Flag	Clinical Significance*
S170	204	Hepatitis B	Hepatitis B	3	Potassium	mEq / L	Day 208	25/04/2011	4.6	.	3.5 - 5.1		
S170	204	Hepatitis B	Hepatitis B	3	Random blood glucose	mg / dl	Day 208	25/04/2011	87	.	45-130		
S170	204	Hepatitis B	Hepatitis B	3	Sodium	mEq / L	Day 208	25/04/2011	140	.	136 - 145		
S170	204	Hepatitis B	Hepatitis B	3	ALT	U / L	Day 365	28/09/2011	23	.	0 - 41		
S170	204	Hepatitis B	Hepatitis B	3	AST	U / L	Day 365	28/09/2011	25	.	0 - 40		
S170	204	Hepatitis B	Hepatitis B	3	Alkaline Phosphatase	U / L	Day 365	28/09/2011	49	.	40 - 129		
S170	204	Hepatitis B	Hepatitis B	3	Direct Bilirubin	mg / dl	Day 365	28/09/2011	0.13	.	0.00 - 0.30		
S170	204	Hepatitis B	Hepatitis B	3	Gamma Glutamyl Transpeptidase	U / L	Day 365	28/09/2011	15	.	10 - 71		
S170	204	Hepatitis B	Hepatitis B	3	Indirect Bilirubin	mg / dl	Day 365	28/09/2011	0.32	.	0.00 - 0.99		
S170	204	Hepatitis B	Hepatitis B	3	Total Bilirubin	mg / dl	Day 365	28/09/2011	0.45	.	0.00 - 0.99		
S170	204	Hepatitis B	Hepatitis B	3	Serum creatinine	mg / dl	Day 365	28/09/2011	1.2	.	0.7 - 1.2		
S170	204	Hepatitis B	Hepatitis B	3	Potassium	mEq / L	Day 365	28/09/2011	4.6	.	3.5 - 5.1		
S170	204	Hepatitis B	Hepatitis B	3	Random blood glucose	mg / dl	Day 365	28/09/2011	88	.	45-130		
S170	204	Hepatitis B	Hepatitis B	3	Sodium	mEq / L	Day 365	28/09/2011	141	.	136 - 145		
S175	209	JAIVAC-1	Dosage B		ALT	U / L	Day -14 to Day -1	21/09/2010	10	.	0 - 41		
S175	209	JAIVAC-1	Dosage B		AST	U / L	Day -14 to Day -1	21/09/2010	14	.	0 - 40		
S175	209	JAIVAC-1	Dosage B		Alkaline Phosphatase	U / L	Day -14 to Day -1	21/09/2010	53	.	40 - 129		
S175	209	JAIVAC-1	Dosage B		Direct Bilirubin	mg / dl	Day -14 to Day -1	21/09/2010	0.17	.	0.00 - 0.30		
S175	209	JAIVAC-1	Dosage B		Gamma Glutamyl Transpeptidase	U / L	Day -14 to Day -1	21/09/2010	10	.	10 - 71		
S175	209	JAIVAC-1	Dosage B		Indirect Bilirubin	mg / dl	Day -14 to Day -1	21/09/2010	0.37	.	0.00 - 0.99		
S175	209	JAIVAC-1	Dosage B		Total Bilirubin	mg / dl	Day -14 to Day -1	21/09/2010	0.54	.	0.00 - 0.99		
S175	209	JAIVAC-1	Dosage B		Serum creatinine	mg / dl	Day -14 to Day -1	21/09/2010	0.8	.	0.7 - 1.2		
S175	209	JAIVAC-1	Dosage B		Potassium	mEq / L	Day -14 to Day -1	21/09/2010	4	.	3.5 - 5.1		
S175	209	JAIVAC-1	Dosage B		Random blood glucose	mg / dl	Day -14 to Day -1	21/09/2010	87	.	45-130		
S175	209	JAIVAC-1	Dosage B		Sodium	mEq / L	Day -14 to Day -1	21/09/2010	139	.	136 - 145		
S175	209	JAIVAC-1	Dosage B	2	ALT	U / L	Day 28	27/10/2010	18	.	0 - 41		
S175	209	JAIVAC-1	Dosage B	2	AST	U / L	Day 28	27/10/2010	17	.	0 - 40		
S175	209	JAIVAC-1	Dosage B	2	Alkaline Phosphatase	U / L	Day 28	27/10/2010	58	.	40 - 129		
S175	209	JAIVAC-1	Dosage B	2	Direct Bilirubin	mg / dl	Day 28	27/10/2010	0.19	.	0.00 - 0.30		

Subject No.	Randomisation Number	Study Group	Dosage	Dose	Serum Chemistry Parameters	Unit	Day	Date of sample Collection	Results	Out of Range	Reference Range	Flag	Clinical Significance*
S175	209	JAIVAC-1	Dosage B	2	Gamma Glutamyl Transpeptidase	U / L	Day 28	27/10/2010	17	.	10 - 71		
S175	209	JAIVAC-1	Dosage B	2	Indirect Bilirubin	mg / dl	Day 28	27/10/2010	0.56	.	0.00 - 0.99		
S175	209	JAIVAC-1	Dosage B	2	Total Bilirubin	mg / dl	Day 28	27/10/2010	0.75	.	0.00 - 0.99		
S175	209	JAIVAC-1	Dosage B	2	Serum creatinine	mg / dl	Day 28	27/10/2010	0.8	.	0.7 - 1.2		
S175	209	JAIVAC-1	Dosage B	2	Potassium	mEq / L	Day 28	27/10/2010	4.4	.	3.5 - 5.1		
S175	209	JAIVAC-1	Dosage B	2	Random blood glucose	mg / dl	Day 28	27/10/2010	90	.	45-130		
S175	209	JAIVAC-1	Dosage B	2	Sodium	mEq / L	Day 28	27/10/2010	141	.	136 - 145		
S175	209	JAIVAC-1	Dosage B	2	ALT	U / L	Day 56	24/11/2010	16	.	0 - 41		
S175	209	JAIVAC-1	Dosage B	2	AST	U / L	Day 56	24/11/2010	21	.	0 - 40		
S175	209	JAIVAC-1	Dosage B	2	Alkaline Phosphatase	U / L	Day 56	24/11/2010	51	.	40 - 129		
S175	209	JAIVAC-1	Dosage B	2	Direct Bilirubin	mg / dl	Day 56	24/11/2010	0.27	.	0.00 - 0.30		
S175	209	JAIVAC-1	Dosage B	2	Gamma Glutamyl Transpeptidase	U / L	Day 56	24/11/2010	12	.	10 - 71		
S175	209	JAIVAC-1	Dosage B	2	Indirect Bilirubin	mg / dl	Day 56	24/11/2010	0.53	.	0.00 - 0.99		
S175	209	JAIVAC-1	Dosage B	2	Total Bilirubin	mg / dl	Day 56	24/11/2010	0.8	.	0.00 - 0.99		
S175	209	JAIVAC-1	Dosage B	2	Serum creatinine	mg / dl	Day 56	24/11/2010	0.8	.	0.7 - 1.2		
S175	209	JAIVAC-1	Dosage B	2	Potassium	mEq / L	Day 56	24/11/2010	4.5	.	3.5 - 5.1		
S175	209	JAIVAC-1	Dosage B	2	Random blood glucose	mg / dl	Day 56	24/11/2010	97	.	45-130		
S175	209	JAIVAC-1	Dosage B	2	Sodium	mEq / L	Day 56	24/11/2010	139	.	136 - 145		
S175	209	JAIVAC-1	Dosage B	3	ALT	U / L	Day 180	28/03/2011	12	.	0 - 41		
S175	209	JAIVAC-1	Dosage B	3	AST	U / L	Day 180	28/03/2011	15	.	0 - 40		
S175	209	JAIVAC-1	Dosage B	3	Alkaline Phosphatase	U / L	Day 180	28/03/2011	46	.	40 - 129		
S175	209	JAIVAC-1	Dosage B	3	Direct Bilirubin	mg / dl	Day 180	28/03/2011	0.26	.	0.00 - 0.30		
S175	209	JAIVAC-1	Dosage B	3	Gamma Glutamyl Transpeptidase	U / L	Day 180	28/03/2011	10	.	10 - 71		
S175	209	JAIVAC-1	Dosage B	3	Indirect Bilirubin	mg / dl	Day 180	28/03/2011	0.53	.	0.00 - 0.99		
S175	209	JAIVAC-1	Dosage B	3	Total Bilirubin	mg / dl	Day 180	28/03/2011	0.79	.	0.00 - 0.99		
S175	209	JAIVAC-1	Dosage B	3	Serum creatinine	mg / dl	Day 180	28/03/2011	1	.	0.7 - 1.2		
S175	209	JAIVAC-1	Dosage B	3	Potassium	mEq / L	Day 180	28/03/2011	4.7	.	3.5 - 5.1		
S175	209	JAIVAC-1	Dosage B	3	Random blood glucose	mg / dl	Day 180	28/03/2011	83	.	45-130		
S175	209	JAIVAC-1	Dosage B	3	Sodium	mEq / L	Day 180	28/03/2011	141	.	136 - 145		

Subject No.	Randomisation Number	Study Group	Dosage	Dose	Serum Chemistry Parameters	Unit	Day	Date of sample Collection	Results	Out of Range	Reference Range	Flag	Clinical Significance*
S175	209	JAIVAC-1	Dosage B	3	ALT	U / L	Day 208	25/04/2011	12	.	0 - 41		
S175	209	JAIVAC-1	Dosage B	3	AST	U / L	Day 208	25/04/2011	14	.	0 - 40		
S175	209	JAIVAC-1	Dosage B	3	Alkaline Phosphatase	U / L	Day 208	25/04/2011	47	.	40 - 129		
S175	209	JAIVAC-1	Dosage B	3	Direct Bilirubin	mg / dl	Day 208	25/04/2011	0.16	.	0.00 - 0.30		
S175	209	JAIVAC-1	Dosage B	3	Gamma Glutamyl Transpeptidase	U / L	Day 208	25/04/2011	9	Yes	10 - 71	L	NCS
S175	209	JAIVAC-1	Dosage B	3	Indirect Bilirubin	mg / dl	Day 208	25/04/2011	0.31	.	0.00 - 0.99		
S175	209	JAIVAC-1	Dosage B	3	Total Bilirubin	mg / dl	Day 208	25/04/2011	0.47	.	0.00 - 0.99		
S175	209	JAIVAC-1	Dosage B	3	Serum creatinine	mg / dl	Day 208	25/04/2011	1	.	0.7 - 1.2		
S175	209	JAIVAC-1	Dosage B	3	Potassium	mEq / L	Day 208	25/04/2011	4.5	.	3.5 - 5.1		
S175	209	JAIVAC-1	Dosage B	3	Random blood glucose	mg / dl	Day 208	25/04/2011	80	.	45-130		
S175	209	JAIVAC-1	Dosage B	3	Sodium	mEq / L	Day 208	25/04/2011	140	.	136 - 145		
S175	209	JAIVAC-1	Dosage B	3	ALT	U / L	Day 365	28/09/2011	9	.	0 - 41		
S175	209	JAIVAC-1	Dosage B	3	AST	U / L	Day 365	28/09/2011	13	.	0 - 40		
S175	209	JAIVAC-1	Dosage B	3	Alkaline Phosphatase	U / L	Day 365	28/09/2011	49	.	40 - 129		
S175	209	JAIVAC-1	Dosage B	3	Direct Bilirubin	mg / dl	Day 365	28/09/2011	0.16	.	0.00 - 0.30		
S175	209	JAIVAC-1	Dosage B	3	Gamma Glutamyl Transpeptidase	U / L	Day 365	28/09/2011	9	Yes	10 - 71	L	NCS
S175	209	JAIVAC-1	Dosage B	3	Indirect Bilirubin	mg / dl	Day 365	28/09/2011	0.24	.	0.00 - 0.99		
S175	209	JAIVAC-1	Dosage B	3	Total Bilirubin	mg / dl	Day 365	28/09/2011	0.4	.	0.00 - 0.99		
S175	209	JAIVAC-1	Dosage B	3	Serum creatinine	mg / dl	Day 365	28/09/2011	0.9	.	0.7 - 1.2		
S175	209	JAIVAC-1	Dosage B	3	Potassium	mEq / L	Day 365	28/09/2011	4.6	.	3.5 - 5.1		
S175	209	JAIVAC-1	Dosage B	3	Random blood glucose	mg / dl	Day 365	28/09/2011	81	.	45-130		
S175	209	JAIVAC-1	Dosage B	3	Sodium	mEq / L	Day 365	28/09/2011	143	.	136 - 145		
S176	205	Hepatitis B	Hepatitis B		ALT	U / L	Day -14 to Day -1	22/09/2010	33	.	0 - 41		
S176	205	Hepatitis B	Hepatitis B		AST	U / L	Day -14 to Day -1	22/09/2010	24	.	0 - 40		
S176	205	Hepatitis B	Hepatitis B		Alkaline Phosphatase	U / L	Day -14 to Day -1	22/09/2010	73	.	40 - 129		
S176	205	Hepatitis B	Hepatitis B		Direct Bilirubin	mg / dl	Day -14 to Day -1	22/09/2010	0.2	.	0.00 - 0.30		
S176	205	Hepatitis B	Hepatitis B		Gamma Glutamyl Transpeptidase	U / L	Day -14 to Day -1	22/09/2010	38	.	10 - 71		
S176	205	Hepatitis B	Hepatitis B		Indirect Bilirubin	mg / dl	Day -14 to Day -1	22/09/2010	0.49	.	0.00 - 0.99		
S176	205	Hepatitis B	Hepatitis B		Total Bilirubin	mg / dl	Day -14 to Day -1	22/09/2010	0.69	.	0.00 - 0.99		

Subject No.	Randomisation Number	Study Group	Dosage	Dose	Serum Chemistry Parameters	Unit	Day	Date of sample Collection	Results	Out of Range	Reference Range	Flag	Clinical Significance*
S176	205	Hepatitis B	Hepatitis B		Serum creatinine	mg / dl	Day -14 to Day -1	22/09/2010	0.9	.	0.7 - 1.2		
S176	205	Hepatitis B	Hepatitis B		Potassium	mEq / L	Day -14 to Day -1	22/09/2010	3.9	.	3.5 - 5.1		
S176	205	Hepatitis B	Hepatitis B		Random blood glucose	mg / dl	Day -14 to Day -1	22/09/2010	83	.	45-130		
S176	205	Hepatitis B	Hepatitis B		Sodium	mEq / L	Day -14 to Day -1	22/09/2010	139	.	136 - 145		
S176	205	Hepatitis B	Hepatitis B	2	ALT	U / L	Day 28	27/10/2010	41	.	0 - 41		
S176	205	Hepatitis B	Hepatitis B	2	AST	U / L	Day 28	27/10/2010	30	.	0 - 40		
S176	205	Hepatitis B	Hepatitis B	2	Alkaline Phosphatase	U / L	Day 28	27/10/2010	71	.	40 - 129		
S176	205	Hepatitis B	Hepatitis B	2	Direct Bilirubin	mg / dl	Day 28	27/10/2010	0.23	.	0.00 - 0.30		
S176	205	Hepatitis B	Hepatitis B	2	Gamma Glutamyl Transpeptidase	U / L	Day 28	27/10/2010	43	.	10 - 71		
S176	205	Hepatitis B	Hepatitis B	2	Indirect Bilirubin	mg / dl	Day 28	27/10/2010	0.57	.	0.00 - 0.99		
S176	205	Hepatitis B	Hepatitis B	2	Total Bilirubin	mg / dl	Day 28	27/10/2010	0.8	.	0.00 - 0.99		
S176	205	Hepatitis B	Hepatitis B	2	Serum creatinine	mg / dl	Day 28	27/10/2010	0.8	.	0.7 - 1.2		
S176	205	Hepatitis B	Hepatitis B	2	Potassium	mEq / L	Day 28	27/10/2010	4.1	.	3.5 - 5.1		
S176	205	Hepatitis B	Hepatitis B	2	Random blood glucose	mg / dl	Day 28	27/10/2010	78	.	45-130		
S176	205	Hepatitis B	Hepatitis B	2	Sodium	mEq / L	Day 28	27/10/2010	141	.	136 - 145		
S176	205	Hepatitis B	Hepatitis B	2	ALT	U / L	Day 56	24/11/2010	44	Yes	0 - 41	H	NCS
S176	205	Hepatitis B	Hepatitis B	2	AST	U / L	Day 56	24/11/2010	34	.	0 - 40		
S176	205	Hepatitis B	Hepatitis B	2	Alkaline Phosphatase	U / L	Day 56	24/11/2010	79	.	40 - 129		
S176	205	Hepatitis B	Hepatitis B	2	Direct Bilirubin	mg / dl	Day 56	24/11/2010	0.23	.	0.00 - 0.30		
S176	205	Hepatitis B	Hepatitis B	2	Gamma Glutamyl Transpeptidase	U / L	Day 56	24/11/2010	44	.	10 - 71		
S176	205	Hepatitis B	Hepatitis B	2	Indirect Bilirubin	mg / dl	Day 56	24/11/2010	0.44	.	0.00 - 0.99		
S176	205	Hepatitis B	Hepatitis B	2	Total Bilirubin	mg / dl	Day 56	24/11/2010	0.67	.	0.00 - 0.99		
S176	205	Hepatitis B	Hepatitis B	2	Serum creatinine	mg / dl	Day 56	24/11/2010	0.8	.	0.7 - 1.2		
S176	205	Hepatitis B	Hepatitis B	2	Potassium	mEq / L	Day 56	24/11/2010	4.3	.	3.5 - 5.1		
S176	205	Hepatitis B	Hepatitis B	2	Random blood glucose	mg / dl	Day 56	24/11/2010	98	.	45-130		
S176	205	Hepatitis B	Hepatitis B	2	Sodium	mEq / L	Day 56	24/11/2010	140	.	136 - 145		
S176	205	Hepatitis B	Hepatitis B	3	ALT	U / L	Day 180	28/03/2011	32	.	0 - 41		
S176	205	Hepatitis B	Hepatitis B	3	AST	U / L	Day 180	28/03/2011	24	.	0 - 40		
S176	205	Hepatitis B	Hepatitis B	3	Alkaline Phosphatase	U / L	Day 180	28/03/2011	90	.	40 - 129		

Subject No.	Randomisation Number	Study Group	Dosage	Dose	Serum Chemistry Parameters	Unit	Day	Date of sample Collection	Results	Out of Range	Reference Range	Flag	Clinical Significance*
S176	205	Hepatitis B	Hepatitis B	3	Direct Bilirubin	mg / dl	Day 180	28/03/2011	0.17	.	0.00 - 0.30		
S176	205	Hepatitis B	Hepatitis B	3	Gamma Glutamyl Transpeptidase	U / L	Day 180	28/03/2011	37	.	10 - 71		
S176	205	Hepatitis B	Hepatitis B	3	Indirect Bilirubin	mg / dl	Day 180	28/03/2011	0.3	.	0.00 - 0.99		
S176	205	Hepatitis B	Hepatitis B	3	Total Bilirubin	mg / dl	Day 180	28/03/2011	0.47	.	0.00 - 0.99		
S176	205	Hepatitis B	Hepatitis B	3	Serum creatinine	mg / dl	Day 180	28/03/2011	1	.	0.7 - 1.2		
S176	205	Hepatitis B	Hepatitis B	3	Potassium	mEq / L	Day 180	28/03/2011	4.1	.	3.5 - 5.1		
S176	205	Hepatitis B	Hepatitis B	3	Random blood glucose	mg / dl	Day 180	28/03/2011	88	.	45-130		
S176	205	Hepatitis B	Hepatitis B	3	Sodium	mEq / L	Day 180	28/03/2011	142	.	136 - 145		
S176	205	Hepatitis B	Hepatitis B	3	ALT	U / L	Day 208	25/04/2011	42	Yes	0 - 41	H	NCS
S176	205	Hepatitis B	Hepatitis B	3	AST	U / L	Day 208	25/04/2011	31	.	0 - 40		
S176	205	Hepatitis B	Hepatitis B	3	Alkaline Phosphatase	U / L	Day 208	25/04/2011	93	.	40 - 129		
S176	205	Hepatitis B	Hepatitis B	3	Direct Bilirubin	mg / dl	Day 208	25/04/2011	0.18	.	0.00 - 0.30		
S176	205	Hepatitis B	Hepatitis B	3	Gamma Glutamyl Transpeptidase	U / L	Day 208	25/04/2011	47	.	10 - 71		
S176	205	Hepatitis B	Hepatitis B	3	Indirect Bilirubin	mg / dl	Day 208	25/04/2011	0.37	.	0.00 - 0.99		
S176	205	Hepatitis B	Hepatitis B	3	Total Bilirubin	mg / dl	Day 208	25/04/2011	0.55	.	0.00 - 0.99		
S176	205	Hepatitis B	Hepatitis B	3	Serum creatinine	mg / dl	Day 208	25/04/2011	1	.	0.7 - 1.2		
S176	205	Hepatitis B	Hepatitis B	3	Potassium	mEq / L	Day 208	25/04/2011	4.3	.	3.5 - 5.1		
S176	205	Hepatitis B	Hepatitis B	3	Random blood glucose	mg / dl	Day 208	25/04/2011	69	.	45-130		
S176	205	Hepatitis B	Hepatitis B	3	Sodium	mEq / L	Day 208	25/04/2011	143	.	136 - 145		
S176	205	Hepatitis B	Hepatitis B	3	ALT	U / L	Day 365	28/09/2011	44	Yes	0 - 41	H	CS
S176	205	Hepatitis B	Hepatitis B	3	AST	U / L	Day 365	28/09/2011	31	.	0 - 40		
S176	205	Hepatitis B	Hepatitis B	3	Alkaline Phosphatase	U / L	Day 365	28/09/2011	73	.	40 - 129		
S176	205	Hepatitis B	Hepatitis B	3	Direct Bilirubin	mg / dl	Day 365	28/09/2011	0.19	.	0.00 - 0.30		
S176	205	Hepatitis B	Hepatitis B	3	Gamma Glutamyl Transpeptidase	U / L	Day 365	28/09/2011	41	.	10 - 71		
S176	205	Hepatitis B	Hepatitis B	3	Indirect Bilirubin	mg / dl	Day 365	28/09/2011	0.54	.	0.00 - 0.99		
S176	205	Hepatitis B	Hepatitis B	3	Total Bilirubin	mg / dl	Day 365	28/09/2011	0.73	.	0.00 - 0.99		
S176	205	Hepatitis B	Hepatitis B	3	Serum creatinine	mg / dl	Day 365	28/09/2011	1	.	0.7 - 1.2		
S176	205	Hepatitis B	Hepatitis B	3	Potassium	mEq / L	Day 365	28/09/2011	4.2	.	3.5 - 5.1		
S176	205	Hepatitis B	Hepatitis B	3	Random blood glucose	mg / dl	Day 365	28/09/2011	86	.	45-130		

Subject No.	Randomisation Number	Study Group	Dosage	Dose	Serum Chemistry Parameters	Unit	Day	Date of sample Collection	Results	Out of Range	Reference Range	Flag	Clinical Significance*
S176	205	Hepatitis B	Hepatitis B	3	Sodium	mEq / L	Day 365	28/09/2011	142	.	136 - 145		
S179	207	Hepatitis B	Hepatitis B		ALT	U / L	Day -14 to Day -1	22/09/2010	28	.	0 - 41		
S179	207	Hepatitis B	Hepatitis B		AST	U / L	Day -14 to Day -1	22/09/2010	28	.	0 - 40		
S179	207	Hepatitis B	Hepatitis B		Alkaline Phosphatase	U / L	Day -14 to Day -1	22/09/2010	54	.	40 - 129		
S179	207	Hepatitis B	Hepatitis B		Direct Bilirubin	mg / dl	Day -14 to Day -1	22/09/2010	0.17	.	0.00 - 0.30		
S179	207	Hepatitis B	Hepatitis B		Gamma Glutamyl Transpeptidase	U / L	Day -14 to Day -1	22/09/2010	5	.	10 - 71	L	
S179	207	Hepatitis B	Hepatitis B		Indirect Bilirubin	mg / dl	Day -14 to Day -1	22/09/2010	0.24	.	0.00 - 0.99		
S179	207	Hepatitis B	Hepatitis B		Total Bilirubin	mg / dl	Day -14 to Day -1	22/09/2010	0.41	.	0.00 - 0.99		
S179	207	Hepatitis B	Hepatitis B		Serum creatinine	mg / dl	Day -14 to Day -1	22/09/2010	1	.	0.7 - 1.2		
S179	207	Hepatitis B	Hepatitis B		Potassium	mEq / L	Day -14 to Day -1	22/09/2010	4.2	.	3.5 - 5.1		
S179	207	Hepatitis B	Hepatitis B		Random blood glucose	mg / dl	Day -14 to Day -1	22/09/2010	82	.	45-130		
S179	207	Hepatitis B	Hepatitis B		Sodium	mEq / L	Day -14 to Day -1	22/09/2010	140	.	136 - 145		
S179	207	Hepatitis B	Hepatitis B	2	ALT	U / L	Day 28	27/10/2010	29	.	0 - 41		
S179	207	Hepatitis B	Hepatitis B	2	AST	U / L	Day 28	27/10/2010	28	.	0 - 40		
S179	207	Hepatitis B	Hepatitis B	2	Alkaline Phosphatase	U / L	Day 28	27/10/2010	54	.	40 - 129		
S179	207	Hepatitis B	Hepatitis B	2	Direct Bilirubin	mg / dl	Day 28	27/10/2010	<0.10	.	0.00 - 0.30		
S179	207	Hepatitis B	Hepatitis B	2	Gamma Glutamyl Transpeptidase	U / L	Day 28	27/10/2010	5	Yes	10 - 71	L	NCS
S179	207	Hepatitis B	Hepatitis B	2	Indirect Bilirubin	mg / dl	Day 28	27/10/2010	NA	.	0.00 - 0.99		
S179	207	Hepatitis B	Hepatitis B	2	Total Bilirubin	mg / dl	Day 28	27/10/2010	0.38	.	0.00 - 0.99		
S179	207	Hepatitis B	Hepatitis B	2	Serum creatinine	mg / dl	Day 28	27/10/2010	1	.	0.7 - 1.2		
S179	207	Hepatitis B	Hepatitis B	2	Potassium	mEq / L	Day 28	27/10/2010	4.3	.	3.5 - 5.1		
S179	207	Hepatitis B	Hepatitis B	2	Random blood glucose	mg / dl	Day 28	27/10/2010	86	.	45-130		
S179	207	Hepatitis B	Hepatitis B	2	Sodium	mEq / L	Day 28	27/10/2010	143	.	136 - 145		
S179	207	Hepatitis B	Hepatitis B	2	ALT	U / L	Day 56	24/11/2010	24	.	0 - 41		
S179	207	Hepatitis B	Hepatitis B	2	AST	U / L	Day 56	24/11/2010	25	.	0 - 40		
S179	207	Hepatitis B	Hepatitis B	2	Alkaline Phosphatase	U / L	Day 56	24/11/2010	55	.	40 - 129		
S179	207	Hepatitis B	Hepatitis B	2	Direct Bilirubin	mg / dl	Day 56	24/11/2010	0.14	.	0.00 - 0.30		
S179	207	Hepatitis B	Hepatitis B	2	Gamma Glutamyl Transpeptidase	U / L	Day 56	24/11/2010	7	Yes	10 - 71	L	NCS
S179	207	Hepatitis B	Hepatitis B	2	Indirect Bilirubin	mg / dl	Day 56	24/11/2010	0.27	.	0.00 - 0.99		

Subject No.	Randomisation Number	Study Group	Dosage	Dose	Serum Chemistry Parameters	Unit	Day	Date of sample Collection	Results	Out of Range	Reference Range	Flag	Clinical Significance*
S179	207	Hepatitis B	Hepatitis B	2	Total Bilirubin	mg / dl	Day 56	24/11/2010	0.41	.	0.00 - 0.99		
S179	207	Hepatitis B	Hepatitis B	2	Serum creatinine	mg / dl	Day 56	24/11/2010	0.9	.	0.7 - 1.2		
S179	207	Hepatitis B	Hepatitis B	2	Potassium	mEq / L	Day 56	24/11/2010	4	.	3.5 - 5.1		
S179	207	Hepatitis B	Hepatitis B	2	Random blood glucose	mg / dl	Day 56	24/11/2010	87	.	45-130		
S179	207	Hepatitis B	Hepatitis B	2	Sodium	mEq / L	Day 56	24/11/2010	141	.	136 - 145		
S179	207	Hepatitis B	Hepatitis B	3	ALT	U / L	Day 180	28/03/2011	23	.	0 - 41		
S179	207	Hepatitis B	Hepatitis B	3	AST	U / L	Day 180	28/03/2011	21	.	0 - 40		
S179	207	Hepatitis B	Hepatitis B	3	Alkaline Phosphatase	U / L	Day 180	28/03/2011	62	.	40 - 129		
S179	207	Hepatitis B	Hepatitis B	3	Direct Bilirubin	mg / dl	Day 180	28/03/2011	0.11	.	0.00 - 0.30		
S179	207	Hepatitis B	Hepatitis B	3	Gamma Glutamyl Transpeptidase	U / L	Day 180	28/03/2011	8	Yes	10 - 71	L	NCS
S179	207	Hepatitis B	Hepatitis B	3	Indirect Bilirubin	mg / dl	Day 180	28/03/2011	0.17	.	0.00 - 0.99		
S179	207	Hepatitis B	Hepatitis B	3	Total Bilirubin	mg / dl	Day 180	28/03/2011	0.28	.	0.00 - 0.99		
S179	207	Hepatitis B	Hepatitis B	3	Serum creatinine	mg / dl	Day 180	28/03/2011	1.1	.	0.7 - 1.2		
S179	207	Hepatitis B	Hepatitis B	3	Potassium	mEq / L	Day 180	28/03/2011	4.1	.	3.5 - 5.1		
S179	207	Hepatitis B	Hepatitis B	3	Random blood glucose	mg / dl	Day 180	28/03/2011	80	.	45-130		
S179	207	Hepatitis B	Hepatitis B	3	Sodium	mEq / L	Day 180	28/03/2011	142	.	136 - 145		
S179	207	Hepatitis B	Hepatitis B	3	ALT	U / L	Day 208	25/04/2011	21	.	0 - 41		
S179	207	Hepatitis B	Hepatitis B	3	AST	U / L	Day 208	25/04/2011	24	.	0 - 40		
S179	207	Hepatitis B	Hepatitis B	3	Alkaline Phosphatase	U / L	Day 208	25/04/2011	59	.	40 - 129		
S179	207	Hepatitis B	Hepatitis B	3	Direct Bilirubin	mg / dl	Day 208	25/04/2011	0.13	.	0.00 - 0.30		
S179	207	Hepatitis B	Hepatitis B	3	Gamma Glutamyl Transpeptidase	U / L	Day 208	25/04/2011	7	Yes	10 - 71	L	NCS
S179	207	Hepatitis B	Hepatitis B	3	Indirect Bilirubin	mg / dl	Day 208	25/04/2011	0.3	.	0.00 - 0.99		
S179	207	Hepatitis B	Hepatitis B	3	Total Bilirubin	mg / dl	Day 208	25/04/2011	0.43	.	0.00 - 0.99		
S179	207	Hepatitis B	Hepatitis B	3	Serum creatinine	mg / dl	Day 208	25/04/2011	1.2	.	0.7 - 1.2		
S179	207	Hepatitis B	Hepatitis B	3	Potassium	mEq / L	Day 208	25/04/2011	4.1	.	3.5 - 5.1		
S179	207	Hepatitis B	Hepatitis B	3	Random blood glucose	mg / dl	Day 208	25/04/2011	67	.	45-130		
S179	207	Hepatitis B	Hepatitis B	3	Sodium	mEq / L	Day 208	25/04/2011	141	.	136 - 145		
S179	207	Hepatitis B	Hepatitis B	3	ALT	U / L	Day 365	28/09/2011	20	.	0 - 41		
S179	207	Hepatitis B	Hepatitis B	3	AST	U / L	Day 365	28/09/2011	17	.	0 - 40		

Subject No.	Randomisation Number	Study Group	Dosage	Dose	Serum Chemistry Parameters	Unit	Day	Date of sample Collection	Results	Out of Range	Reference Range	Flag	Clinical Significance*
S179	207	Hepatitis B	Hepatitis B	3	Alkaline Phosphatase	U / L	Day 365	28/09/2011	60	.	40 - 129		
S179	207	Hepatitis B	Hepatitis B	3	Direct Bilirubin	mg / dl	Day 365	28/09/2011	<0.10	.	0.00 - 0.30		
S179	207	Hepatitis B	Hepatitis B	3	Gamma Glutamyl Transpeptidase	U / L	Day 365	28/09/2011	6	Yes	10 - 71	L	NCS
S179	207	Hepatitis B	Hepatitis B	3	Indirect Bilirubin	mg / dl	Day 365	28/09/2011	NA	.	0.00 - 0.99		
S179	207	Hepatitis B	Hepatitis B	3	Total Bilirubin	mg / dl	Day 365	28/09/2011	0.41	.	0.00 - 0.99		
S179	207	Hepatitis B	Hepatitis B	3	Serum creatinine	mg / dl	Day 365	28/09/2011	1.2	.	0.7 - 1.2		
S179	207	Hepatitis B	Hepatitis B	3	Potassium	mEq / L	Day 365	28/09/2011	3.9	.	3.5 - 5.1		
S179	207	Hepatitis B	Hepatitis B	3	Random blood glucose	mg / dl	Day 365	28/09/2011	85	.	45-130		
S179	207	Hepatitis B	Hepatitis B	3	Sodium	mEq / L	Day 365	28/09/2011	143	.	136 - 145		
S182	208	JAIVAC-1	Dosage B		ALT	U / L	Day -14 to Day -1	21/09/2010	17	.	0 - 41		
S182	208	JAIVAC-1	Dosage B		AST	U / L	Day -14 to Day -1	21/09/2010	21	.	0 - 40		
S182	208	JAIVAC-1	Dosage B		Alkaline Phosphatase	U / L	Day -14 to Day -1	21/09/2010	104	.	40 - 129		
S182	208	JAIVAC-1	Dosage B		Direct Bilirubin	mg / dl	Day -14 to Day -1	21/09/2010	0.23	.	0.00 - 0.30		
S182	208	JAIVAC-1	Dosage B		Gamma Glutamyl Transpeptidase	U / L	Day -14 to Day -1	21/09/2010	21	.	10 - 71		
S182	208	JAIVAC-1	Dosage B		Indirect Bilirubin	mg / dl	Day -14 to Day -1	21/09/2010	0.51	.	0.00 - 0.99		
S182	208	JAIVAC-1	Dosage B		Total Bilirubin	mg / dl	Day -14 to Day -1	21/09/2010	0.74	.	0.00 - 0.99		
S182	208	JAIVAC-1	Dosage B		Serum creatinine	mg / dl	Day -14 to Day -1	21/09/2010	1.1	.	0.7 - 1.2		
S182	208	JAIVAC-1	Dosage B		Potassium	mEq / L	Day -14 to Day -1	21/09/2010	4.1	.	3.5 - 5.1		
S182	208	JAIVAC-1	Dosage B		Random blood glucose	mg / dl	Day -14 to Day -1	21/09/2010	86	.	45-130		
S182	208	JAIVAC-1	Dosage B		Sodium	mEq / L	Day -14 to Day -1	21/09/2010	140	.	136 - 145		
S182	208	JAIVAC-1	Dosage B	2	ALT	U / L	Day 28	27/10/2010	17	.	0 - 41		
S182	208	JAIVAC-1	Dosage B	2	AST	U / L	Day 28	27/10/2010	24	.	0 - 40		
S182	208	JAIVAC-1	Dosage B	2	Alkaline Phosphatase	U / L	Day 28	27/10/2010	98	.	40 - 129		
S182	208	JAIVAC-1	Dosage B	2	Direct Bilirubin	mg / dl	Day 28	27/10/2010	0.22	.	0.00 - 0.30		
S182	208	JAIVAC-1	Dosage B	2	Gamma Glutamyl Transpeptidase	U / L	Day 28	27/10/2010	17	.	10 - 71		
S182	208	JAIVAC-1	Dosage B	2	Indirect Bilirubin	mg / dl	Day 28	27/10/2010	0.5	.	0.00 - 0.99		
S182	208	JAIVAC-1	Dosage B	2	Total Bilirubin	mg / dl	Day 28	27/10/2010	0.72	.	0.00 - 0.99		
S182	208	JAIVAC-1	Dosage B	2	Serum creatinine	mg / dl	Day 28	27/10/2010	0.9	.	0.7 - 1.2		
S182	208	JAIVAC-1	Dosage B	2	Potassium	mEq / L	Day 28	27/10/2010	4.2	.	3.5 - 5.1		

Subject No.	Randomisation Number	Study Group	Dosage	Dose	Serum Chemistry Parameters	Unit	Day	Date of sample Collection	Results	Out of Range	Reference Range	Flag	Clinical Significance*
S182	208	JAIVAC-1	Dosage B	2	Random blood glucose	mg / dl	Day 28	27/10/2010	94	.	45-130		
S182	208	JAIVAC-1	Dosage B	2	Sodium	mEq / L	Day 28	27/10/2010	141	.	136 - 145		
S182	208	JAIVAC-1	Dosage B	2	ALT	U / L	Day 56	24/11/2010	20	.	0 - 41		
S182	208	JAIVAC-1	Dosage B	2	AST	U / L	Day 56	24/11/2010	26	.	0 - 40		
S182	208	JAIVAC-1	Dosage B	2	Alkaline Phosphatase	U / L	Day 56	24/11/2010	114	.	40 - 129		
S182	208	JAIVAC-1	Dosage B	2	Direct Bilirubin	mg / dl	Day 56	24/11/2010	0.13	.	0.00 - 0.30		
S182	208	JAIVAC-1	Dosage B	2	Gamma Glutamyl Transpeptidase	U / L	Day 56	24/11/2010	18	.	10 - 71		
S182	208	JAIVAC-1	Dosage B	2	Indirect Bilirubin	mg / dl	Day 56	24/11/2010	0.3	.	0.00 - 0.99		
S182	208	JAIVAC-1	Dosage B	2	Total Bilirubin	mg / dl	Day 56	24/11/2010	0.43	.	0.00 - 0.99		
S182	208	JAIVAC-1	Dosage B	2	Serum creatinine	mg / dl	Day 56	24/11/2010	1.1	.	0.7 - 1.2		
S182	208	JAIVAC-1	Dosage B	2	Potassium	mEq / L	Day 56	24/11/2010	4.4	.	3.5 - 5.1		
S182	208	JAIVAC-1	Dosage B	2	Random blood glucose	mg / dl	Day 56	24/11/2010	93	.	45-130		
S182	208	JAIVAC-1	Dosage B	2	Sodium	mEq / L	Day 56	24/11/2010	142	.	136 - 145		
S182	208	JAIVAC-1	Dosage B	3	ALT	U / L	Day 180	28/03/2011	17	.	0 - 41		
S182	208	JAIVAC-1	Dosage B	3	AST	U / L	Day 180	28/03/2011	24	.	0 - 40		
S182	208	JAIVAC-1	Dosage B	3	Alkaline Phosphatase	U / L	Day 180	28/03/2011	98	.	40 - 129		
S182	208	JAIVAC-1	Dosage B	3	Direct Bilirubin	mg / dl	Day 180	28/03/2011	0.16	.	0.00 - 0.30		
S182	208	JAIVAC-1	Dosage B	3	Gamma Glutamyl Transpeptidase	U / L	Day 180	28/03/2011	13	.	10 - 71		
S182	208	JAIVAC-1	Dosage B	3	Indirect Bilirubin	mg / dl	Day 180	28/03/2011	0.29	.	0.00 - 0.99		
S182	208	JAIVAC-1	Dosage B	3	Total Bilirubin	mg / dl	Day 180	28/03/2011	0.45	.	0.00 - 0.99		
S182	208	JAIVAC-1	Dosage B	3	Serum creatinine	mg / dl	Day 180	28/03/2011	1.2	.	0.7 - 1.2		
S182	208	JAIVAC-1	Dosage B	3	Potassium	mEq / L	Day 180	28/03/2011	4.4	.	3.5 - 5.1		
S182	208	JAIVAC-1	Dosage B	3	Random blood glucose	mg / dl	Day 180	28/03/2011	89	.	45-130		
S182	208	JAIVAC-1	Dosage B	3	Sodium	mEq / L	Day 180	28/03/2011	142	.	136 - 145		
S182	208	JAIVAC-1	Dosage B	3	ALT	U / L	Day 208	25/04/2011	27	.	0 - 41		
S182	208	JAIVAC-1	Dosage B	3	AST	U / L	Day 208	25/04/2011	32	.	0 - 40		
S182	208	JAIVAC-1	Dosage B	3	Alkaline Phosphatase	U / L	Day 208	25/04/2011	97	.	40 - 129		
S182	208	JAIVAC-1	Dosage B	3	Direct Bilirubin	mg / dl	Day 208	25/04/2011	0.18	.	0.00 - 0.30		
S182	208	JAIVAC-1	Dosage B	3	Gamma Glutamyl Transpeptidase	U / L	Day 208	25/04/2011	19	.	10 - 71		

Subject No.	Randomisation Number	Study Group	Dosage	Dose	Serum Chemistry Parameters	Unit	Day	Date of sample Collection	Results	Out of Range	Reference Range	Flag	Clinical Significance*
S182	208	JAIVAC-1	Dosage B	3	Indirect Bilirubin	mg / dl	Day 208	25/04/2011	0.47	.	0.00 - 0.99		
S182	208	JAIVAC-1	Dosage B	3	Total Bilirubin	mg / dl	Day 208	25/04/2011	0.65	.	0.00 - 0.99		
S182	208	JAIVAC-1	Dosage B	3	Serum creatinine	mg / dl	Day 208	25/04/2011	1.1	.	0.7 - 1.2		
S182	208	JAIVAC-1	Dosage B	3	Potassium	mEq / L	Day 208	25/04/2011	4.9	.	3.5 - 5.1		
S182	208	JAIVAC-1	Dosage B	3	Random blood glucose	mg / dl	Day 208	25/04/2011	82	.	45-130		
S182	208	JAIVAC-1	Dosage B	3	Sodium	mEq / L	Day 208	25/04/2011	143	.	136 - 145		
S182	208	JAIVAC-1	Dosage B	3	ALT	U / L	Day 365	29/09/2011	18	.	0 - 41		
S182	208	JAIVAC-1	Dosage B	3	AST	U / L	Day 365	29/09/2011	20	.	0 - 40		
S182	208	JAIVAC-1	Dosage B	3	Alkaline Phosphatase	U / L	Day 365	29/09/2011	87	.	40 - 129		
S182	208	JAIVAC-1	Dosage B	3	Direct Bilirubin	mg / dl	Day 365	29/09/2011	0.23	.	0.00 - 0.30		
S182	208	JAIVAC-1	Dosage B	3	Gamma Glutamyl Transpeptidase	U / L	Day 365	29/09/2011	12	.	10 - 71		
S182	208	JAIVAC-1	Dosage B	3	Indirect Bilirubin	mg / dl	Day 365	29/09/2011	0.42	.	0.00 - 0.99		
S182	208	JAIVAC-1	Dosage B	3	Total Bilirubin	mg / dl	Day 365	29/09/2011	0.65	.	0.00 - 0.99		
S182	208	JAIVAC-1	Dosage B	3	Serum creatinine	mg / dl	Day 365	29/09/2011	1.3	Yes	0.7 - 1.2	H	CS
S182	208	JAIVAC-1	Dosage B	3	Potassium	mEq / L	Day 365	29/09/2011	4.3	.	3.5 - 5.1		
S182	208	JAIVAC-1	Dosage B	3	Random blood glucose	mg / dl	Day 365	29/09/2011	83	.	45-130		
S182	208	JAIVAC-1	Dosage B	3	Sodium	mEq / L	Day 365	29/09/2011	142	.	136 - 145		
S185	202	JAIVAC-1	Dosage B		ALT	U / L	Day -14 to Day -1	21/09/2010	12	.	0 - 41		
S185	202	JAIVAC-1	Dosage B		AST	U / L	Day -14 to Day -1	21/09/2010	22	.	0 - 40		
S185	202	JAIVAC-1	Dosage B		Alkaline Phosphatase	U / L	Day -14 to Day -1	21/09/2010	80	.	40 - 129		
S185	202	JAIVAC-1	Dosage B		Direct Bilirubin	mg / dl	Day -14 to Day -1	21/09/2010	0.28	.	0.00 - 0.30		
S185	202	JAIVAC-1	Dosage B		Gamma Glutamyl Transpeptidase	U / L	Day -14 to Day -1	21/09/2010	13	.	10 - 71		
S185	202	JAIVAC-1	Dosage B		Indirect Bilirubin	mg / dl	Day -14 to Day -1	21/09/2010	0.48	.	0.00 - 0.99		
S185	202	JAIVAC-1	Dosage B		Total Bilirubin	mg / dl	Day -14 to Day -1	21/09/2010	0.76	.	0.00 - 0.99		
S185	202	JAIVAC-1	Dosage B		Serum creatinine	mg / dl	Day -14 to Day -1	21/09/2010	0.8	.	0.7 - 1.2		
S185	202	JAIVAC-1	Dosage B		Potassium	mEq / L	Day -14 to Day -1	21/09/2010	3.9	.	3.5 - 5.1		
S185	202	JAIVAC-1	Dosage B		Random blood glucose	mg / dl	Day -14 to Day -1	21/09/2010	80	.	45-130		
S185	202	JAIVAC-1	Dosage B		Sodium	mEq / L	Day -14 to Day -1	21/09/2010	137	.	136 - 145		
S185	202	JAIVAC-1	Dosage B	2	ALT	U / L	Day 28	26/10/2010	14	.	0 - 41		

Subject No.	Randomisation Number	Study Group	Dosage	Dose	Serum Chemistry Parameters	Unit	Day	Date of sample Collection	Results	Out of Range	Reference Range	Flag	Clinical Significance*
S185	202	JAIVAC-1	Dosage B	2	AST	U / L	Day 28	26/10/2010	22	.	0 - 40		
S185	202	JAIVAC-1	Dosage B	2	Alkaline Phosphatase	U / L	Day 28	26/10/2010	83	.	40 - 129		
S185	202	JAIVAC-1	Dosage B	2	Direct Bilirubin	mg / dl	Day 28	26/10/2010	0.25	.	0.00 - 0.30		
S185	202	JAIVAC-1	Dosage B	2	Gamma Glutamyl Transpeptidase	U / L	Day 28	26/10/2010	15	.	10 - 71		
S185	202	JAIVAC-1	Dosage B	2	Indirect Bilirubin	mg / dl	Day 28	26/10/2010	0.67	.	0.00 - 0.99		
S185	202	JAIVAC-1	Dosage B	2	Total Bilirubin	mg / dl	Day 28	26/10/2010	0.92	.	0.00 - 0.99		
S185	202	JAIVAC-1	Dosage B	2	Serum creatinine	mg / dl	Day 28	26/10/2010	0.8	.	0.7 - 1.2		
S185	202	JAIVAC-1	Dosage B	2	Potassium	mEq / L	Day 28	26/10/2010	4	.	3.5 - 5.1		
S185	202	JAIVAC-1	Dosage B	2	Random blood glucose	mg / dl	Day 28	26/10/2010	110	.	45-130		
S185	202	JAIVAC-1	Dosage B	2	Sodium	mEq / L	Day 28	26/10/2010	138	.	136 - 145		
S185	202	JAIVAC-1	Dosage B	2	ALT	U / L	Day 56	23/11/2010	14	.	0 - 41		
S185	202	JAIVAC-1	Dosage B	2	AST	U / L	Day 56	23/11/2010	25	.	0 - 40		
S185	202	JAIVAC-1	Dosage B	2	Alkaline Phosphatase	U / L	Day 56	23/11/2010	84	.	40 - 129		
S185	202	JAIVAC-1	Dosage B	2	Direct Bilirubin	mg / dl	Day 56	23/11/2010	0.31	Yes	0.00 - 0.30	H	NCS
S185	202	JAIVAC-1	Dosage B	2	Gamma Glutamyl Transpeptidase	U / L	Day 56	23/11/2010	14	.	10 - 71		
S185	202	JAIVAC-1	Dosage B	2	Indirect Bilirubin	mg / dl	Day 56	23/11/2010	0.49	.	0.00 - 0.99		
S185	202	JAIVAC-1	Dosage B	2	Total Bilirubin	mg / dl	Day 56	23/11/2010	0.8	.	0.00 - 0.99		
S185	202	JAIVAC-1	Dosage B	2	Serum creatinine	mg / dl	Day 56	23/11/2010	0.8	.	0.7 - 1.2		
S185	202	JAIVAC-1	Dosage B	2	Potassium	mEq / L	Day 56	23/11/2010	4.7	.	3.5 - 5.1		
S185	202	JAIVAC-1	Dosage B	2	Random blood glucose	mg / dl	Day 56	23/11/2010	110	.	45-130		
S185	202	JAIVAC-1	Dosage B	2	Sodium	mEq / L	Day 56	23/11/2010	140	.	136 - 145		
S185	202	JAIVAC-1	Dosage B	3	ALT	U / L	Day 180	28/03/2011	12	.	0 - 41		
S185	202	JAIVAC-1	Dosage B	3	AST	U / L	Day 180	28/03/2011	19	.	0 - 40		
S185	202	JAIVAC-1	Dosage B	3	Alkaline Phosphatase	U / L	Day 180	28/03/2011	84	.	40 - 129		
S185	202	JAIVAC-1	Dosage B	3	Direct Bilirubin	mg / dl	Day 180	28/03/2011	0.25	.	0.00 - 0.30		
S185	202	JAIVAC-1	Dosage B	3	Gamma Glutamyl Transpeptidase	U / L	Day 180	28/03/2011	15	.	10 - 71		
S185	202	JAIVAC-1	Dosage B	3	Indirect Bilirubin	mg / dl	Day 180	28/03/2011	0.34	.	0.00 - 0.99		
S185	202	JAIVAC-1	Dosage B	3	Total Bilirubin	mg / dl	Day 180	28/03/2011	0.59	.	0.00 - 0.99		
S185	202	JAIVAC-1	Dosage B	3	Serum creatinine	mg / dl	Day 180	28/03/2011	0.9	.	0.7 - 1.2		

Subject No.	Randomisation Number	Study Group	Dosage	Dose	Serum Chemistry Parameters	Unit	Day	Date of sample Collection	Results	Out of Range	Reference Range	Flag	Clinical Significance*
S185	202	JAIVAC-1	Dosage B	3	Potassium	mEq / L	Day 180	28/03/2011	3.7	.	3.5 - 5.1		
S185	202	JAIVAC-1	Dosage B	3	Random blood glucose	mg / dl	Day 180	28/03/2011	87	.	45-130		
S185	202	JAIVAC-1	Dosage B	3	Sodium	mEq / L	Day 180	28/03/2011	139	.	136 - 145		
S185	202	JAIVAC-1	Dosage B	3	ALT	U / L	Day 208	25/04/2011	17	.	0 - 41		
S185	202	JAIVAC-1	Dosage B	3	AST	U / L	Day 208	25/04/2011	21	.	0 - 40		
S185	202	JAIVAC-1	Dosage B	3	Alkaline Phosphatase	U / L	Day 208	25/04/2011	89	.	40 - 129		
S185	202	JAIVAC-1	Dosage B	3	Direct Bilirubin	mg / dl	Day 208	25/04/2011	0.22	.	0.00 - 0.30		
S185	202	JAIVAC-1	Dosage B	3	Gamma Glutamyl Transpeptidase	U / L	Day 208	25/04/2011	14	.	10 - 71		
S185	202	JAIVAC-1	Dosage B	3	Indirect Bilirubin	mg / dl	Day 208	25/04/2011	0.47	.	0.00 - 0.99		
S185	202	JAIVAC-1	Dosage B	3	Total Bilirubin	mg / dl	Day 208	25/04/2011	0.69	.	0.00 - 0.99		
S185	202	JAIVAC-1	Dosage B	3	Serum creatinine	mg / dl	Day 208	25/04/2011	0.9	.	0.7 - 1.2		
S185	202	JAIVAC-1	Dosage B	3	Potassium	mEq / L	Day 208	25/04/2011	4.1	.	3.5 - 5.1		
S185	202	JAIVAC-1	Dosage B	3	Random blood glucose	mg / dl	Day 208	25/04/2011	101	.	45-130		
S185	202	JAIVAC-1	Dosage B	3	Sodium	mEq / L	Day 208	25/04/2011	142	.	136 - 145		
S185	202	JAIVAC-1	Dosage B	3	ALT	U / L	Day 365	28/09/2011	17	.	0 - 41		
S185	202	JAIVAC-1	Dosage B	3	AST	U / L	Day 365	28/09/2011	22	.	0 - 40		
S185	202	JAIVAC-1	Dosage B	3	Alkaline Phosphatase	U / L	Day 365	28/09/2011	80	.	40 - 129		
S185	202	JAIVAC-1	Dosage B	3	Direct Bilirubin	mg / dl	Day 365	28/09/2011	0.29	.	0.00 - 0.30		
S185	202	JAIVAC-1	Dosage B	3	Gamma Glutamyl Transpeptidase	U / L	Day 365	28/09/2011	14	.	10 - 71		
S185	202	JAIVAC-1	Dosage B	3	Indirect Bilirubin	mg / dl	Day 365	28/09/2011	0.66	.	0.00 - 0.99		
S185	202	JAIVAC-1	Dosage B	3	Total Bilirubin	mg / dl	Day 365	28/09/2011	0.95	.	0.00 - 0.99		
S185	202	JAIVAC-1	Dosage B	3	Serum creatinine	mg / dl	Day 365	28/09/2011	1	.	0.7 - 1.2		
S185	202	JAIVAC-1	Dosage B	3	Potassium	mEq / L	Day 365	28/09/2011	4.1	.	3.5 - 5.1		
S185	202	JAIVAC-1	Dosage B	3	Random blood glucose	mg / dl	Day 365	28/09/2011	69	.	45-130		
S185	202	JAIVAC-1	Dosage B	3	Sodium	mEq / L	Day 365	28/09/2011	141	.	136 - 145		
S186	201	JAIVAC-1	Dosage B		ALT	U / L	Day -14 to Day -1	21/09/2010	13	.	0 - 41		
S186	201	JAIVAC-1	Dosage B		AST	U / L	Day -14 to Day -1	21/09/2010	16	.	0 - 40		
S186	201	JAIVAC-1	Dosage B		Alkaline Phosphatase	U / L	Day -14 to Day -1	21/09/2010	113	.	40 - 129		
S186	201	JAIVAC-1	Dosage B		Direct Bilirubin	mg / dl	Day -14 to Day -1	21/09/2010	0.33	.	0.00 - 0.30	H	

Subject No.	Randomisation Number	Study Group	Dosage	Dose	Serum Chemistry Parameters	Unit	Day	Date of sample Collection	Results	Out of Range	Reference Range	Flag	Clinical Significance*
S186	201	JAIVAC-1	Dosage B		Gamma Glutamyl Transpeptidase	U / L	Day -14 to Day -1	21/09/2010	4	.	10 - 71	L	
S186	201	JAIVAC-1	Dosage B		Indirect Bilirubin	mg / dl	Day -14 to Day -1	21/09/2010	0.76	.	0.00 - 0.99		
S186	201	JAIVAC-1	Dosage B		Total Bilirubin	mg / dl	Day -14 to Day -1	21/09/2010	1.09	.	0.00 - 0.99	H	
S186	201	JAIVAC-1	Dosage B		Serum creatinine	mg / dl	Day -14 to Day -1	21/09/2010	0.9	.	0.7 - 1.2		
S186	201	JAIVAC-1	Dosage B		Potassium	mEq / L	Day -14 to Day -1	21/09/2010	4	.	3.5 - 5.1		
S186	201	JAIVAC-1	Dosage B		Random blood glucose	mg / dl	Day -14 to Day -1	21/09/2010	89	.	45-130		
S186	201	JAIVAC-1	Dosage B		Sodium	mEq / L	Day -14 to Day -1	21/09/2010	139	.	136 - 145		
S186	201	JAIVAC-1	Dosage B	2	ALT	U / L	Day 28	26/10/2010	16	.	0 - 41		
S186	201	JAIVAC-1	Dosage B	2	AST	U / L	Day 28	26/10/2010	17	.	0 - 40		
S186	201	JAIVAC-1	Dosage B	2	Alkaline Phosphatase	U / L	Day 28	26/10/2010	126	.	40 - 129		
S186	201	JAIVAC-1	Dosage B	2	Direct Bilirubin	mg / dl	Day 28	26/10/2010	0.23	.	0.00 - 0.30		
S186	201	JAIVAC-1	Dosage B	2	Gamma Glutamyl Transpeptidase	U / L	Day 28	26/10/2010	7	Yes	10 - 71	L	NCS
S186	201	JAIVAC-1	Dosage B	2	Indirect Bilirubin	mg / dl	Day 28	26/10/2010	0.69	.	0.00 - 0.99		
S186	201	JAIVAC-1	Dosage B	2	Total Bilirubin	mg / dl	Day 28	26/10/2010	0.92	.	0.00 - 0.99		
S186	201	JAIVAC-1	Dosage B	2	Serum creatinine	mg / dl	Day 28	26/10/2010	0.8	.	0.7 - 1.2		
S186	201	JAIVAC-1	Dosage B	2	Potassium	mEq / L	Day 28	26/10/2010	4.1	.	3.5 - 5.1		
S186	201	JAIVAC-1	Dosage B	2	Random blood glucose	mg / dl	Day 28	26/10/2010	87	.	45-130		
S186	201	JAIVAC-1	Dosage B	2	Sodium	mEq / L	Day 28	26/10/2010	140	.	136 - 145		
S186	201	JAIVAC-1	Dosage B	2	ALT	U / L	Day 56	23/11/2010	10	.	0 - 41		
S186	201	JAIVAC-1	Dosage B	2	AST	U / L	Day 56	23/11/2010	16	.	0 - 40		
S186	201	JAIVAC-1	Dosage B	2	Alkaline Phosphatase	U / L	Day 56	23/11/2010	120	.	40 - 129		
S186	201	JAIVAC-1	Dosage B	2	Direct Bilirubin	mg / dl	Day 56	23/11/2010	0.24	.	0.00 - 0.30		
S186	201	JAIVAC-1	Dosage B	2	Gamma Glutamyl Transpeptidase	U / L	Day 56	23/11/2010	6	Yes	10 - 71	L	NCS
S186	201	JAIVAC-1	Dosage B	2	Indirect Bilirubin	mg / dl	Day 56	23/11/2010	0.44	.	0.00 - 0.99		
S186	201	JAIVAC-1	Dosage B	2	Total Bilirubin	mg / dl	Day 56	23/11/2010	0.68	.	0.00 - 0.99		
S186	201	JAIVAC-1	Dosage B	2	Serum creatinine	mg / dl	Day 56	23/11/2010	0.8	.	0.7 - 1.2		
S186	201	JAIVAC-1	Dosage B	2	Potassium	mEq / L	Day 56	23/11/2010	4.3	.	3.5 - 5.1		
S186	201	JAIVAC-1	Dosage B	2	Random blood glucose	mg / dl	Day 56	23/11/2010	122	.	45-130		
S186	201	JAIVAC-1	Dosage B	2	Sodium	mEq / L	Day 56	23/11/2010	141	.	136 - 145		

Subject No.	Randomisation Number	Study Group	Dosage	Dose	Serum Chemistry Parameters	Unit	Day	Date of sample Collection	Results	Out of Range	Reference Range	Flag	Clinical Significance*
S186	201	JAIVAC-1	Dosage B	3	ALT	U / L	Day 180	28/03/2011	12	.	0 - 41		
S186	201	JAIVAC-1	Dosage B	3	AST	U / L	Day 180	28/03/2011	15	.	0 - 40		
S186	201	JAIVAC-1	Dosage B	3	Alkaline Phosphatase	U / L	Day 180	28/03/2011	111	.	40 - 129		
S186	201	JAIVAC-1	Dosage B	3	Direct Bilirubin	mg / dl	Day 180	28/03/2011	0.24	.	0.00 - 0.30		
S186	201	JAIVAC-1	Dosage B	3	Gamma Glutamyl Transpeptidase	U / L	Day 180	28/03/2011	7	Yes	10 - 71	L	NCS
S186	201	JAIVAC-1	Dosage B	3	Indirect Bilirubin	mg / dl	Day 180	28/03/2011	0.53	.	0.00 - 0.99		
S186	201	JAIVAC-1	Dosage B	3	Total Bilirubin	mg / dl	Day 180	28/03/2011	0.77	.	0.00 - 0.99		
S186	201	JAIVAC-1	Dosage B	3	Serum creatinine	mg / dl	Day 180	28/03/2011	1	.	0.7 - 1.2		
S186	201	JAIVAC-1	Dosage B	3	Potassium	mEq / L	Day 180	28/03/2011	4.2	.	3.5 - 5.1		
S186	201	JAIVAC-1	Dosage B	3	Random blood glucose	mg / dl	Day 180	28/03/2011	86	.	45-130		
S186	201	JAIVAC-1	Dosage B	3	Sodium	mEq / L	Day 180	28/03/2011	141	.	136 - 145		
S186	201	JAIVAC-1	Dosage B	3	ALT	U / L	Day 208	25/04/2011	11	.	0 - 41		
S186	201	JAIVAC-1	Dosage B	3	AST	U / L	Day 208	25/04/2011	14	.	0 - 40		
S186	201	JAIVAC-1	Dosage B	3	Alkaline Phosphatase	U / L	Day 208	25/04/2011	106	.	40 - 129		
S186	201	JAIVAC-1	Dosage B	3	Direct Bilirubin	mg / dl	Day 208	25/04/2011	0.32	Yes	0.00 - 0.30	H	NCS
S186	201	JAIVAC-1	Dosage B	3	Gamma Glutamyl Transpeptidase	U / L	Day 208	25/04/2011	6	Yes	10 - 71	L	NCS
S186	201	JAIVAC-1	Dosage B	3	Indirect Bilirubin	mg / dl	Day 208	25/04/2011	0.7	.	0.00 - 0.99		
S186	201	JAIVAC-1	Dosage B	3	Total Bilirubin	mg / dl	Day 208	25/04/2011	1.02	Yes	0.00 - 0.99	H	NCS
S186	201	JAIVAC-1	Dosage B	3	Serum creatinine	mg / dl	Day 208	25/04/2011	0.9	.	0.7 - 1.2		
S186	201	JAIVAC-1	Dosage B	3	Potassium	mEq / L	Day 208	25/04/2011	4.3	.	3.5 - 5.1		
S186	201	JAIVAC-1	Dosage B	3	Random blood glucose	mg / dl	Day 208	25/04/2011	75	.	45-130		
S186	201	JAIVAC-1	Dosage B	3	Sodium	mEq / L	Day 208	25/04/2011	142	.	136 - 145		
S186	201	JAIVAC-1	Dosage B	3	ALT	U / L	Day 365	28/09/2011	15	.	0 - 41		
S186	201	JAIVAC-1	Dosage B	3	AST	U / L	Day 365	28/09/2011	16	.	0 - 40		
S186	201	JAIVAC-1	Dosage B	3	Alkaline Phosphatase	U / L	Day 365	28/09/2011	107	.	40 - 129		
S186	201	JAIVAC-1	Dosage B	3	Direct Bilirubin	mg / dl	Day 365	28/09/2011	0.28	.	0.00 - 0.30		
S186	201	JAIVAC-1	Dosage B	3	Gamma Glutamyl Transpeptidase	U / L	Day 365	28/09/2011	8	Yes	10 - 71	L	NCS
S186	201	JAIVAC-1	Dosage B	3	Indirect Bilirubin	mg / dl	Day 365	28/09/2011	0.78	.	0.00 - 0.99		
S186	201	JAIVAC-1	Dosage B	3	Total Bilirubin	mg / dl	Day 365	28/09/2011	1.06	Yes	0.00 - 0.99	H	NCS

Subject No.	Randomisation Number	Study Group	Dosage	Dose	Serum Chemistry Parameters	Unit	Day	Date of sample Collection	Results	Out of Range	Reference Range	Flag	Clinical Significance*
S186	201	JAIVAC-1	Dosage B	3	Serum creatinine	mg / dl	Day 365	28/09/2011	0.9	.	0.7 - 1.2		
S186	201	JAIVAC-1	Dosage B	3	Potassium	mEq / L	Day 365	28/09/2011	4.2	.	3.5 - 5.1		
S186	201	JAIVAC-1	Dosage B	3	Random blood glucose	mg / dl	Day 365	28/09/2011	80	.	45-130		
S186	201	JAIVAC-1	Dosage B	3	Sodium	mEq / L	Day 365	28/09/2011	143	.	136 - 145		
S192	203	JAIVAC-1	Dosage B		ALT	U / L	Day -14 to Day -1	23/09/2010	10	.	0 - 41		
S192	203	JAIVAC-1	Dosage B		AST	U / L	Day -14 to Day -1	23/09/2010	19	.	0 - 40		
S192	203	JAIVAC-1	Dosage B		Alkaline Phosphatase	U / L	Day -14 to Day -1	23/09/2010	51	.	40 - 129		
S192	203	JAIVAC-1	Dosage B		Direct Bilirubin	mg / dl	Day -14 to Day -1	23/09/2010	0.14	.	0.00 - 0.30		
S192	203	JAIVAC-1	Dosage B		Gamma Glutamyl Transpeptidase	U / L	Day -14 to Day -1	23/09/2010	11	.	10 - 71		
S192	203	JAIVAC-1	Dosage B		Indirect Bilirubin	mg / dl	Day -14 to Day -1	23/09/2010	0.45	.	0.00 - 0.99		
S192	203	JAIVAC-1	Dosage B		Total Bilirubin	mg / dl	Day -14 to Day -1	23/09/2010	0.59	.	0.00 - 0.99		
S192	203	JAIVAC-1	Dosage B		Serum creatinine	mg / dl	Day -14 to Day -1	23/09/2010	0.9	.	0.7 - 1.2		
S192	203	JAIVAC-1	Dosage B		Potassium	mEq / L	Day -14 to Day -1	23/09/2010	4	.	3.5 - 5.1		
S192	203	JAIVAC-1	Dosage B		Random blood glucose	mg / dl	Day -14 to Day -1	23/09/2010	104	.	45-130		
S192	203	JAIVAC-1	Dosage B		Sodium	mEq / L	Day -14 to Day -1	23/09/2010	140	.	136 - 145		
S192	203	JAIVAC-1	Dosage B	2	ALT	U / L	Day 28	26/10/2010	14	.	0 - 41		
S192	203	JAIVAC-1	Dosage B	2	AST	U / L	Day 28	26/10/2010	19	.	0 - 40		
S192	203	JAIVAC-1	Dosage B	2	Alkaline Phosphatase	U / L	Day 28	26/10/2010	57	.	40 - 129		
S192	203	JAIVAC-1	Dosage B	2	Direct Bilirubin	mg / dl	Day 28	26/10/2010	0.14	.	0.00 - 0.30		
S192	203	JAIVAC-1	Dosage B	2	Gamma Glutamyl Transpeptidase	U / L	Day 28	26/10/2010	10	.	10 - 71		
S192	203	JAIVAC-1	Dosage B	2	Indirect Bilirubin	mg / dl	Day 28	26/10/2010	0.42	.	0.00 - 0.99		
S192	203	JAIVAC-1	Dosage B	2	Total Bilirubin	mg / dl	Day 28	26/10/2010	0.56	.	0.00 - 0.99		
S192	203	JAIVAC-1	Dosage B	2	Serum creatinine	mg / dl	Day 28	26/10/2010	0.9	.	0.7 - 1.2		
S192	203	JAIVAC-1	Dosage B	2	Potassium	mEq / L	Day 28	26/10/2010	4.2	.	3.5 - 5.1		
S192	203	JAIVAC-1	Dosage B	2	Random blood glucose	mg / dl	Day 28	26/10/2010	85	.	45-130		
S192	203	JAIVAC-1	Dosage B	2	Sodium	mEq / L	Day 28	26/10/2010	137	.	136 - 145		
S192	203	JAIVAC-1	Dosage B	2	ALT	U / L	Day 56	23/11/2010	10	.	0 - 41		
S192	203	JAIVAC-1	Dosage B	2	AST	U / L	Day 56	23/11/2010	19	.	0 - 40		
S192	203	JAIVAC-1	Dosage B	2	Alkaline Phosphatase	U / L	Day 56	23/11/2010	51	.	40 - 129		

Subject No.	Randomisation Number	Study Group	Dosage	Dose	Serum Chemistry Parameters	Unit	Day	Date of sample Collection	Results	Out of Range	Reference Range	Flag	Clinical Significance*
S192	203	JAIVAC-1	Dosage B	2	Direct Bilirubin	mg / dl	Day 56	23/11/2010	0.12	.	0.00 - 0.30		
S192	203	JAIVAC-1	Dosage B	2	Gamma Glutamyl Transpeptidase	U / L	Day 56	23/11/2010	10	.	10 - 71		
S192	203	JAIVAC-1	Dosage B	2	Indirect Bilirubin	mg / dl	Day 56	23/11/2010	0.24	.	0.00 - 0.99		
S192	203	JAIVAC-1	Dosage B	2	Total Bilirubin	mg / dl	Day 56	23/11/2010	0.36	.	0.00 - 0.99		
S192	203	JAIVAC-1	Dosage B	2	Serum creatinine	mg / dl	Day 56	23/11/2010	0.9	.	0.7 - 1.2		
S192	203	JAIVAC-1	Dosage B	2	Potassium	mEq / L	Day 56	23/11/2010	4.3	.	3.5 - 5.1		
S192	203	JAIVAC-1	Dosage B	2	Random blood glucose	mg / dl	Day 56	23/11/2010	87	.	45-130		
S192	203	JAIVAC-1	Dosage B	2	Sodium	mEq / L	Day 56	23/11/2010	140	.	136 - 145		
S192	203	JAIVAC-1	Dosage B	3	ALT	U / L	Day 180	28/03/2011	17	.	0 - 41		
S192	203	JAIVAC-1	Dosage B	3	AST	U / L	Day 180	28/03/2011	30	.	0 - 40		
S192	203	JAIVAC-1	Dosage B	3	Alkaline Phosphatase	U / L	Day 180	28/03/2011	46	.	40 - 129		
S192	203	JAIVAC-1	Dosage B	3	Direct Bilirubin	mg / dl	Day 180	28/03/2011	0.21	.	0.00 - 0.30		
S192	203	JAIVAC-1	Dosage B	3	Gamma Glutamyl Transpeptidase	U / L	Day 180	28/03/2011	11	.	10 - 71		
S192	203	JAIVAC-1	Dosage B	3	Indirect Bilirubin	mg / dl	Day 180	28/03/2011	0.73	.	0.00 - 0.99		
S192	203	JAIVAC-1	Dosage B	3	Total Bilirubin	mg / dl	Day 180	28/03/2011	0.94	.	0.00 - 0.99		
S192	203	JAIVAC-1	Dosage B	3	Serum creatinine	mg / dl	Day 180	28/03/2011	1	.	0.7 - 1.2		
S192	203	JAIVAC-1	Dosage B	3	Potassium	mEq / L	Day 180	28/03/2011	3.7	.	3.5 - 5.1		
S192	203	JAIVAC-1	Dosage B	3	Random blood glucose	mg / dl	Day 180	28/03/2011	69	.	45-130		
S192	203	JAIVAC-1	Dosage B	3	Sodium	mEq / L	Day 180	28/03/2011	141	.	136 - 145		
S192	203	JAIVAC-1	Dosage B	3	ALT	U / L	Day 208	25/04/2011	18	.	0 - 41		
S192	203	JAIVAC-1	Dosage B	3	AST	U / L	Day 208	25/04/2011	21	.	0 - 40		
S192	203	JAIVAC-1	Dosage B	3	Alkaline Phosphatase	U / L	Day 208	25/04/2011	53	.	40 - 129		
S192	203	JAIVAC-1	Dosage B	3	Direct Bilirubin	mg / dl	Day 208	25/04/2011	0.15	.	0.00 - 0.30		
S192	203	JAIVAC-1	Dosage B	3	Gamma Glutamyl Transpeptidase	U / L	Day 208	25/04/2011	13	.	10 - 71		
S192	203	JAIVAC-1	Dosage B	3	Indirect Bilirubin	mg / dl	Day 208	25/04/2011	0.38	.	0.00 - 0.99		
S192	203	JAIVAC-1	Dosage B	3	Total Bilirubin	mg / dl	Day 208	25/04/2011	0.53	.	0.00 - 0.99		
S192	203	JAIVAC-1	Dosage B	3	Serum creatinine	mg / dl	Day 208	25/04/2011	0.9	.	0.7 - 1.2		
S192	203	JAIVAC-1	Dosage B	3	Potassium	mEq / L	Day 208	25/04/2011	4.8	.	3.5 - 5.1		
S192	203	JAIVAC-1	Dosage B	3	Random blood glucose	mg / dl	Day 208	25/04/2011	87	.	45-130		

Subject No.	Randomisation Number	Study Group	Dosage	Dose	Serum Chemistry Parameters	Unit	Day	Date of sample Collection	Results	Out of Range	Reference Range	Flag	Clinical Significance*
S192	203	JAIVAC-1	Dosage B	3	Sodium	mEq / L	Day 208	25/04/2011	141	.	136 - 145		
S192	203	JAIVAC-1	Dosage B	3	ALT	U / L	Day 365	28/09/2011	12	.	0 - 41		
S192	203	JAIVAC-1	Dosage B	3	AST	U / L	Day 365	28/09/2011	19	.	0 - 40		
S192	203	JAIVAC-1	Dosage B	3	Alkaline Phosphatase	U / L	Day 365	28/09/2011	53	.	40 - 129		
S192	203	JAIVAC-1	Dosage B	3	Direct Bilirubin	mg / dl	Day 365	28/09/2011	0.12	.	0.00 - 0.30		
S192	203	JAIVAC-1	Dosage B	3	Gamma Glutamyl Transpeptidase	U / L	Day 365	28/09/2011	9	Yes	10 - 71	L	NCS
S192	203	JAIVAC-1	Dosage B	3	Indirect Bilirubin	mg / dl	Day 365	28/09/2011	0.38	.	0.00 - 0.99		
S192	203	JAIVAC-1	Dosage B	3	Total Bilirubin	mg / dl	Day 365	28/09/2011	0.5	.	0.00 - 0.99		
S192	203	JAIVAC-1	Dosage B	3	Serum creatinine	mg / dl	Day 365	28/09/2011	0.9	.	0.7 - 1.2		
S192	203	JAIVAC-1	Dosage B	3	Potassium	mEq / L	Day 365	28/09/2011	4.2	.	3.5 - 5.1		
S192	203	JAIVAC-1	Dosage B	3	Random blood glucose	mg / dl	Day 365	28/09/2011	82	.	45-130		
S192	203	JAIVAC-1	Dosage B	3	Sodium	mEq / L	Day 365	28/09/2011	138	.	136 - 145		
S193	215	JAIVAC-1	Dosage B		ALT	U / L	Day -14 to Day -1	24/09/2010	24	.	0 - 41		
S193	215	JAIVAC-1	Dosage B		AST	U / L	Day -14 to Day -1	24/09/2010	22	.	0 - 40		
S193	215	JAIVAC-1	Dosage B		Alkaline Phosphatase	U / L	Day -14 to Day -1	24/09/2010	108	.	40 - 129		
S193	215	JAIVAC-1	Dosage B		Direct Bilirubin	mg / dl	Day -14 to Day -1	24/09/2010	<0.10	.	0.00 - 0.30		
S193	215	JAIVAC-1	Dosage B		Gamma Glutamyl Transpeptidase	U / L	Day -14 to Day -1	24/09/2010	14	.	10 - 71		
S193	215	JAIVAC-1	Dosage B		Indirect Bilirubin	mg / dl	Day -14 to Day -1	24/09/2010	NA	.	0.00 - 0.99		
S193	215	JAIVAC-1	Dosage B		Total Bilirubin	mg / dl	Day -14 to Day -1	24/09/2010	0.47	.	0.00 - 0.99		
S193	215	JAIVAC-1	Dosage B		Serum creatinine	mg / dl	Day -14 to Day -1	24/09/2010	0.7	.	0.7 - 1.2		
S193	215	JAIVAC-1	Dosage B		Potassium	mEq / L	Day -14 to Day -1	24/09/2010	4.6	.	3.5 - 5.1		
S193	215	JAIVAC-1	Dosage B		Random blood glucose	mg / dl	Day -14 to Day -1	24/09/2010	76	.	45-130		
S193	215	JAIVAC-1	Dosage B		Sodium	mEq / L	Day -14 to Day -1	24/09/2010	140	.	136 - 145		
S193	215	JAIVAC-1	Dosage B	2	ALT	U / L	Day 28	28/10/2010	34	.	0 - 41		
S193	215	JAIVAC-1	Dosage B	2	AST	U / L	Day 28	28/10/2010	23	.	0 - 40		
S193	215	JAIVAC-1	Dosage B	2	Alkaline Phosphatase	U / L	Day 28	28/10/2010	103	.	40 - 129		
S193	215	JAIVAC-1	Dosage B	2	Direct Bilirubin	mg / dl	Day 28	28/10/2010	<0.10	.	0.00 - 0.30		
S193	215	JAIVAC-1	Dosage B	2	Gamma Glutamyl Transpeptidase	U / L	Day 28	28/10/2010	18	.	10 - 71		
S193	215	JAIVAC-1	Dosage B	2	Indirect Bilirubin	mg / dl	Day 28	28/10/2010	NA	.	0.00 - 0.99		

Subject No.	Randomisation Number	Study Group	Dosage	Dose	Serum Chemistry Parameters	Unit	Day	Date of sample Collection	Results	Out of Range	Reference Range	Flag	Clinical Significance*
S193	215	JAIVAC-1	Dosage B	2	Total Bilirubin	mg / dl	Day 28	28/10/2010	0.39	.	0.00 - 0.99		
S193	215	JAIVAC-1	Dosage B	2	Serum creatinine	mg / dl	Day 28	28/10/2010	0.6	Yes	0.7 - 1.2	L	NCS
S193	215	JAIVAC-1	Dosage B	2	Potassium	mEq / L	Day 28	28/10/2010	4.3	.	3.5 - 5.1		
S193	215	JAIVAC-1	Dosage B	2	Random blood glucose	mg / dl	Day 28	28/10/2010	112	.	45-130		
S193	215	JAIVAC-1	Dosage B	2	Sodium	mEq / L	Day 28	28/10/2010	140	.	136 - 145		
S193	215	JAIVAC-1	Dosage B	2	ALT	U / L	Day 56	25/11/2010	35	.	0 - 41		
S193	215	JAIVAC-1	Dosage B	2	AST	U / L	Day 56	25/11/2010	26	.	0 - 40		
S193	215	JAIVAC-1	Dosage B	2	Alkaline Phosphatase	U / L	Day 56	25/11/2010	105	.	40 - 129		
S193	215	JAIVAC-1	Dosage B	2	Direct Bilirubin	mg / dl	Day 56	25/11/2010	0.14	.	0.00 - 0.30		
S193	215	JAIVAC-1	Dosage B	2	Gamma Glutamyl Transpeptidase	U / L	Day 56	25/11/2010	16	.	10 - 71		
S193	215	JAIVAC-1	Dosage B	2	Indirect Bilirubin	mg / dl	Day 56	25/11/2010	0.27	.	0.00 - 0.99		
S193	215	JAIVAC-1	Dosage B	2	Total Bilirubin	mg / dl	Day 56	25/11/2010	0.41	.	0.00 - 0.99		
S193	215	JAIVAC-1	Dosage B	2	Serum creatinine	mg / dl	Day 56	25/11/2010	0.8	.	0.7 - 1.2		
S193	215	JAIVAC-1	Dosage B	2	Potassium	mEq / L	Day 56	25/11/2010	4.5	.	3.5 - 5.1		
S193	215	JAIVAC-1	Dosage B	2	Random blood glucose	mg / dl	Day 56	25/11/2010	104	.	45-130		
S193	215	JAIVAC-1	Dosage B	2	Sodium	mEq / L	Day 56	25/11/2010	140	.	136 - 145		
S193	215	JAIVAC-1	Dosage B	3	ALT	U / L	Day 180	29/03/2011	35	.	0 - 41		
S193	215	JAIVAC-1	Dosage B	3	AST	U / L	Day 180	29/03/2011	27	.	0 - 40		
S193	215	JAIVAC-1	Dosage B	3	Alkaline Phosphatase	U / L	Day 180	29/03/2011	120	.	40 - 129		
S193	215	JAIVAC-1	Dosage B	3	Direct Bilirubin	mg / dl	Day 180	29/03/2011	0.1	.	0.00 - 0.30		
S193	215	JAIVAC-1	Dosage B	3	Gamma Glutamyl Transpeptidase	U / L	Day 180	29/03/2011	19	.	10 - 71		
S193	215	JAIVAC-1	Dosage B	3	Indirect Bilirubin	mg / dl	Day 180	29/03/2011	0.23	.	0.00 - 0.99		
S193	215	JAIVAC-1	Dosage B	3	Total Bilirubin	mg / dl	Day 180	29/03/2011	0.33	.	0.00 - 0.99		
S193	215	JAIVAC-1	Dosage B	3	Serum creatinine	mg / dl	Day 180	29/03/2011	0.7	.	0.7 - 1.2		
S193	215	JAIVAC-1	Dosage B	3	Potassium	mEq / L	Day 180	29/03/2011	4.6	.	3.5 - 5.1		
S193	215	JAIVAC-1	Dosage B	3	Random blood glucose	mg / dl	Day 180	29/03/2011	83	.	45-130		
S193	215	JAIVAC-1	Dosage B	3	Sodium	mEq / L	Day 180	29/03/2011	139	.	136 - 145		
S193	215	JAIVAC-1	Dosage B	3	ALT	U / L	Day 208	26/04/2011	32	.	0 - 41		
S193	215	JAIVAC-1	Dosage B	3	AST	U / L	Day 208	26/04/2011	21	.	0 - 40		

Subject No.	Randomisation Number	Study Group	Dosage	Dose	Serum Chemistry Parameters	Unit	Day	Date of sample Collection	Results	Out of Range	Reference Range	Flag	Clinical Significance*
S193	215	JAIVAC-1	Dosage B	3	Alkaline Phosphatase	U / L	Day 208	26/04/2011	115	.	40 - 129		
S193	215	JAIVAC-1	Dosage B	3	Direct Bilirubin	mg / dl	Day 208	26/04/2011	0.14	.	0.00 - 0.30		
S193	215	JAIVAC-1	Dosage B	3	Gamma Glutamyl Transpeptidase	U / L	Day 208	26/04/2011	18	.	10 - 71		
S193	215	JAIVAC-1	Dosage B	3	Indirect Bilirubin	mg / dl	Day 208	26/04/2011	0.24	.	0.00 - 0.99		
S193	215	JAIVAC-1	Dosage B	3	Total Bilirubin	mg / dl	Day 208	26/04/2011	0.38	.	0.00 - 0.99		
S193	215	JAIVAC-1	Dosage B	3	Serum creatinine	mg / dl	Day 208	26/04/2011	0.7	.	0.7 - 1.2		
S193	215	JAIVAC-1	Dosage B	3	Potassium	mEq / L	Day 208	26/04/2011	4.8	.	3.5 - 5.1		
S193	215	JAIVAC-1	Dosage B	3	Random blood glucose	mg / dl	Day 208	26/04/2011	76	.	45-130		
S193	215	JAIVAC-1	Dosage B	3	Sodium	mEq / L	Day 208	26/04/2011	139	.	136 - 145		
S193	215	JAIVAC-1	Dosage B	3	ALT	U / L	Day 365	29/09/2011	31	.	0 - 41		
S193	215	JAIVAC-1	Dosage B	3	AST	U / L	Day 365	29/09/2011	23	.	0 - 40		
S193	215	JAIVAC-1	Dosage B	3	Alkaline Phosphatase	U / L	Day 365	29/09/2011	119	.	40 - 129		
S193	215	JAIVAC-1	Dosage B	3	Direct Bilirubin	mg / dl	Day 365	29/09/2011	0.19	.	0.00 - 0.30		
S193	215	JAIVAC-1	Dosage B	3	Gamma Glutamyl Transpeptidase	U / L	Day 365	29/09/2011	18	.	10 - 71		
S193	215	JAIVAC-1	Dosage B	3	Indirect Bilirubin	mg / dl	Day 365	29/09/2011	0.37	.	0.00 - 0.99		
S193	215	JAIVAC-1	Dosage B	3	Total Bilirubin	mg / dl	Day 365	29/09/2011	0.56	.	0.00 - 0.99		
S193	215	JAIVAC-1	Dosage B	3	Serum creatinine	mg / dl	Day 365	29/09/2011	0.9	.	0.7 - 1.2		
S193	215	JAIVAC-1	Dosage B	3	Potassium	mEq / L	Day 365	29/09/2011	5.2	Yes	3.5 - 5.1	H	CS
S193	215	JAIVAC-1	Dosage B	3	Random blood glucose	mg / dl	Day 365	29/09/2011	75	.	45-130		
S193	215	JAIVAC-1	Dosage B	3	Sodium	mEq / L	Day 365	29/09/2011	138	.	136 - 145		
S195	214	JAIVAC-1	Dosage B		ALT	U / L	Day -14 to Day -1	23/09/2010	13	.	0 - 41		
S195	214	JAIVAC-1	Dosage B		AST	U / L	Day -14 to Day -1	23/09/2010	21	.	0 - 40		
S195	214	JAIVAC-1	Dosage B		Alkaline Phosphatase	U / L	Day -14 to Day -1	23/09/2010	83	.	40 - 129		
S195	214	JAIVAC-1	Dosage B		Direct Bilirubin	mg / dl	Day -14 to Day -1	23/09/2010	0.11	.	0.00 - 0.30		
S195	214	JAIVAC-1	Dosage B		Gamma Glutamyl Transpeptidase	U / L	Day -14 to Day -1	23/09/2010	14	.	10 - 71		
S195	214	JAIVAC-1	Dosage B		Indirect Bilirubin	mg / dl	Day -14 to Day -1	23/09/2010	0.25	.	0.00 - 0.99		
S195	214	JAIVAC-1	Dosage B		Total Bilirubin	mg / dl	Day -14 to Day -1	23/09/2010	0.36	.	0.00 - 0.99		
S195	214	JAIVAC-1	Dosage B		Serum creatinine	mg / dl	Day -14 to Day -1	23/09/2010	0.8	.	0.7 - 1.2		
S195	214	JAIVAC-1	Dosage B		Potassium	mEq / L	Day -14 to Day -1	23/09/2010	3.9	.	3.5 - 5.1		

Subject No.	Randomisation Number	Study Group	Dosage	Dose	Serum Chemistry Parameters	Unit	Day	Date of sample Collection	Results	Out of Range	Reference Range	Flag	Clinical Significance*
S195	214	JAIVAC-1	Dosage B		Random blood glucose	mg / dl	Day -14 to Day -1	23/09/2010	95	.	45-130		
S195	214	JAIVAC-1	Dosage B		Sodium	mEq / L	Day -14 to Day -1	23/09/2010	140	.	136 - 145		
S195	214	JAIVAC-1	Dosage B	2	ALT	U / L	Day 28	28/10/2010	14	.	0 - 41		
S195	214	JAIVAC-1	Dosage B	2	AST	U / L	Day 28	28/10/2010	21	.	0 - 40		
S195	214	JAIVAC-1	Dosage B	2	Alkaline Phosphatase	U / L	Day 28	28/10/2010	81	.	40 - 129		
S195	214	JAIVAC-1	Dosage B	2	Direct Bilirubin	mg / dl	Day 28	28/10/2010	0.12	.	0.00 - 0.30		
S195	214	JAIVAC-1	Dosage B	2	Gamma Glutamyl Transpeptidase	U / L	Day 28	28/10/2010	13	.	10 - 71		
S195	214	JAIVAC-1	Dosage B	2	Indirect Bilirubin	mg / dl	Day 28	28/10/2010	0.45	.	0.00 - 0.99		
S195	214	JAIVAC-1	Dosage B	2	Total Bilirubin	mg / dl	Day 28	28/10/2010	0.57	.	0.00 - 0.99		
S195	214	JAIVAC-1	Dosage B	2	Serum creatinine	mg / dl	Day 28	28/10/2010	0.8	.	0.7 - 1.2		
S195	214	JAIVAC-1	Dosage B	2	Potassium	mEq / L	Day 28	28/10/2010	4	.	3.5 - 5.1		
S195	214	JAIVAC-1	Dosage B	2	Random blood glucose	mg / dl	Day 28	28/10/2010	82	.	45-130		
S195	214	JAIVAC-1	Dosage B	2	Sodium	mEq / L	Day 28	28/10/2010	140	.	136 - 145		
S195	214	JAIVAC-1	Dosage B	2	ALT	U / L	Day 56	24/11/2010	13	.	0 - 41		
S195	214	JAIVAC-1	Dosage B	2	AST	U / L	Day 56	24/11/2010	20	.	0 - 40		
S195	214	JAIVAC-1	Dosage B	2	Alkaline Phosphatase	U / L	Day 56	24/11/2010	91	.	40 - 129		
S195	214	JAIVAC-1	Dosage B	2	Direct Bilirubin	mg / dl	Day 56	24/11/2010	0.12	.	0.00 - 0.30		
S195	214	JAIVAC-1	Dosage B	2	Gamma Glutamyl Transpeptidase	U / L	Day 56	24/11/2010	15	.	10 - 71		
S195	214	JAIVAC-1	Dosage B	2	Indirect Bilirubin	mg / dl	Day 56	24/11/2010	0.31	.	0.00 - 0.99		
S195	214	JAIVAC-1	Dosage B	2	Total Bilirubin	mg / dl	Day 56	24/11/2010	0.43	.	0.00 - 0.99		
S195	214	JAIVAC-1	Dosage B	2	Serum creatinine	mg / dl	Day 56	24/11/2010	0.9	.	0.7 - 1.2		
S195	214	JAIVAC-1	Dosage B	2	Potassium	mEq / L	Day 56	24/11/2010	4	.	3.5 - 5.1		
S195	214	JAIVAC-1	Dosage B	2	Random blood glucose	mg / dl	Day 56	24/11/2010	105	.	45-130		
S195	214	JAIVAC-1	Dosage B	2	Sodium	mEq / L	Day 56	24/11/2010	142	.	136 - 145		
S195	214	JAIVAC-1	Dosage B	3	ALT	U / L	Day 180	29/03/2011	12	.	0 - 41		
S195	214	JAIVAC-1	Dosage B	3	AST	U / L	Day 180	29/03/2011	18	.	0 - 40		
S195	214	JAIVAC-1	Dosage B	3	Alkaline Phosphatase	U / L	Day 180	29/03/2011	83	.	40 - 129		
S195	214	JAIVAC-1	Dosage B	3	Direct Bilirubin	mg / dl	Day 180	29/03/2011	<0.10	.	0.00 - 0.30		
S195	214	JAIVAC-1	Dosage B	3	Gamma Glutamyl Transpeptidase	U / L	Day 180	29/03/2011	14	.	10 - 71		

Subject No.	Randomisation Number	Study Group	Dosage	Dose	Serum Chemistry Parameters	Unit	Day	Date of sample Collection	Results	Out of Range	Reference Range	Flag	Clinical Significance*
S195	214	JAIVAC-1	Dosage B	3	Indirect Bilirubin	mg / dl	Day 180	29/03/2011	NA	.	0.00 - 0.99		
S195	214	JAIVAC-1	Dosage B	3	Total Bilirubin	mg / dl	Day 180	29/03/2011	0.38	.	0.00 - 0.99		
S195	214	JAIVAC-1	Dosage B	3	Serum creatinine	mg / dl	Day 180	29/03/2011	1	.	0.7 - 1.2		
S195	214	JAIVAC-1	Dosage B	3	Potassium	mEq / L	Day 180	29/03/2011	4	.	3.5 - 5.1		
S195	214	JAIVAC-1	Dosage B	3	Random blood glucose	mg / dl	Day 180	29/03/2011	68	.	45-130		
S195	214	JAIVAC-1	Dosage B	3	Sodium	mEq / L	Day 180	29/03/2011	140	.	136 - 145		
S195	214	JAIVAC-1	Dosage B	3	ALT	U / L	Day 208	26/04/2011	18	.	0 - 41		
S195	214	JAIVAC-1	Dosage B	3	AST	U / L	Day 208	26/04/2011	17	.	0 - 40		
S195	214	JAIVAC-1	Dosage B	3	Alkaline Phosphatase	U / L	Day 208	26/04/2011	102	.	40 - 129		
S195	214	JAIVAC-1	Dosage B	3	Direct Bilirubin	mg / dl	Day 208	26/04/2011	0.17	.	0.00 - 0.30		
S195	214	JAIVAC-1	Dosage B	3	Gamma Glutamyl Transpeptidase	U / L	Day 208	26/04/2011	14	.	10 - 71		
S195	214	JAIVAC-1	Dosage B	3	Indirect Bilirubin	mg / dl	Day 208	26/04/2011	0.34	.	0.00 - 0.99		
S195	214	JAIVAC-1	Dosage B	3	Total Bilirubin	mg / dl	Day 208	26/04/2011	0.51	.	0.00 - 0.99		
S195	214	JAIVAC-1	Dosage B	3	Serum creatinine	mg / dl	Day 208	26/04/2011	1.1	.	0.7 - 1.2		
S195	214	JAIVAC-1	Dosage B	3	Potassium	mEq / L	Day 208	26/04/2011	5	.	3.5 - 5.1		
S195	214	JAIVAC-1	Dosage B	3	Random blood glucose	mg / dl	Day 208	26/04/2011	82	.	45-130		
S195	214	JAIVAC-1	Dosage B	3	Sodium	mEq / L	Day 208	26/04/2011	141	.	136 - 145		
S195	214	JAIVAC-1	Dosage B	3	ALT	U / L	Day 365	29/09/2011	15	.	0 - 41		
S195	214	JAIVAC-1	Dosage B	3	AST	U / L	Day 365	29/09/2011	16	.	0 - 40		
S195	214	JAIVAC-1	Dosage B	3	Alkaline Phosphatase	U / L	Day 365	29/09/2011	106	.	40 - 129		
S195	214	JAIVAC-1	Dosage B	3	Direct Bilirubin	mg / dl	Day 365	29/09/2011	<0.10	.	0.00 - 0.30		
S195	214	JAIVAC-1	Dosage B	3	Gamma Glutamyl Transpeptidase	U / L	Day 365	29/09/2011	16	.	10 - 71		
S195	214	JAIVAC-1	Dosage B	3	Indirect Bilirubin	mg / dl	Day 365	29/09/2011	NA	.	0.00 - 0.99		
S195	214	JAIVAC-1	Dosage B	3	Total Bilirubin	mg / dl	Day 365	29/09/2011	0.33	.	0.00 - 0.99		
S195	214	JAIVAC-1	Dosage B	3	Serum creatinine	mg / dl	Day 365	29/09/2011	1	.	0.7 - 1.2		
S195	214	JAIVAC-1	Dosage B	3	Potassium	mEq / L	Day 365	29/09/2011	4.6	.	3.5 - 5.1		
S195	214	JAIVAC-1	Dosage B	3	Random blood glucose	mg / dl	Day 365	29/09/2011	82	.	45-130		
S195	214	JAIVAC-1	Dosage B	3	Sodium	mEq / L	Day 365	29/09/2011	140	.	136 - 145		
S202	206	JAIVAC-1	Dosage B		ALT	U / L	Day -14 to Day -1	23/09/2010	29	.	0 - 41		

Subject No.	Randomisation Number	Study Group	Dosage	Dose	Serum Chemistry Parameters	Unit	Day	Date of sample Collection	Results	Out of Range	Reference Range	Flag	Clinical Significance*
S202	206	JAIVAC-1	Dosage B		AST	U / L	Day -14 to Day -1	23/09/2010	17	.	0 - 40		
S202	206	JAIVAC-1	Dosage B		Alkaline Phosphatase	U / L	Day -14 to Day -1	23/09/2010	101	.	40 - 129		
S202	206	JAIVAC-1	Dosage B		Direct Bilirubin	mg / dl	Day -14 to Day -1	23/09/2010	0.15	.	0.00 - 0.30		
S202	206	JAIVAC-1	Dosage B		Gamma Glutamyl Transpeptidase	U / L	Day -14 to Day -1	23/09/2010	31	.	10 - 71		
S202	206	JAIVAC-1	Dosage B		Indirect Bilirubin	mg / dl	Day -14 to Day -1	23/09/2010	0.34	.	0.00 - 0.99		
S202	206	JAIVAC-1	Dosage B		Total Bilirubin	mg / dl	Day -14 to Day -1	23/09/2010	0.49	.	0.00 - 0.99		
S202	206	JAIVAC-1	Dosage B		Serum creatinine	mg / dl	Day -14 to Day -1	23/09/2010	0.9	.	0.7 - 1.2		
S202	206	JAIVAC-1	Dosage B		Potassium	mEq / L	Day -14 to Day -1	23/09/2010	4	.	3.5 - 5.1		
S202	206	JAIVAC-1	Dosage B		Random blood glucose	mg / dl	Day -14 to Day -1	23/09/2010	85	.	45-130		
S202	206	JAIVAC-1	Dosage B		Sodium	mEq / L	Day -14 to Day -1	23/09/2010	139	.	136 - 145		
S202	206	JAIVAC-1	Dosage B	2	ALT	U / L	Day 28	27/10/2010	26	.	0 - 41		
S202	206	JAIVAC-1	Dosage B	2	AST	U / L	Day 28	27/10/2010	16	.	0 - 40		
S202	206	JAIVAC-1	Dosage B	2	Alkaline Phosphatase	U / L	Day 28	27/10/2010	79	.	40 - 129		
S202	206	JAIVAC-1	Dosage B	2	Direct Bilirubin	mg / dl	Day 28	27/10/2010	0.13	.	0.00 - 0.30		
S202	206	JAIVAC-1	Dosage B	2	Gamma Glutamyl Transpeptidase	U / L	Day 28	27/10/2010	32	.	10 - 71		
S202	206	JAIVAC-1	Dosage B	2	Indirect Bilirubin	mg / dl	Day 28	27/10/2010	0.38	.	0.00 - 0.99		
S202	206	JAIVAC-1	Dosage B	2	Total Bilirubin	mg / dl	Day 28	27/10/2010	0.51	.	0.00 - 0.99		
S202	206	JAIVAC-1	Dosage B	2	Serum creatinine	mg / dl	Day 28	27/10/2010	0.9	.	0.7 - 1.2		
S202	206	JAIVAC-1	Dosage B	2	Potassium	mEq / L	Day 28	27/10/2010	4.4	.	3.5 - 5.1		
S202	206	JAIVAC-1	Dosage B	2	Random blood glucose	mg / dl	Day 28	27/10/2010	72	.	45-130		
S202	206	JAIVAC-1	Dosage B	2	Sodium	mEq / L	Day 28	27/10/2010	142	.	136 - 145		
S202	206	JAIVAC-1	Dosage B	2	ALT	U / L	Day 56	24/11/2010	28	.	0 - 41		
S202	206	JAIVAC-1	Dosage B	2	AST	U / L	Day 56	24/11/2010	17	.	0 - 40		
S202	206	JAIVAC-1	Dosage B	2	Alkaline Phosphatase	U / L	Day 56	24/11/2010	92	.	40 - 129		
S202	206	JAIVAC-1	Dosage B	2	Direct Bilirubin	mg / dl	Day 56	24/11/2010	0.17	.	0.00 - 0.30		
S202	206	JAIVAC-1	Dosage B	2	Gamma Glutamyl Transpeptidase	U / L	Day 56	24/11/2010	41	.	10 - 71		
S202	206	JAIVAC-1	Dosage B	2	Indirect Bilirubin	mg / dl	Day 56	24/11/2010	0.37	.	0.00 - 0.99		
S202	206	JAIVAC-1	Dosage B	2	Total Bilirubin	mg / dl	Day 56	24/11/2010	0.54	.	0.00 - 0.99		
S202	206	JAIVAC-1	Dosage B	2	Serum creatinine	mg / dl	Day 56	24/11/2010	1	.	0.7 - 1.2		

Subject No.	Randomisation Number	Study Group	Dosage	Dose	Serum Chemistry Parameters	Unit	Day	Date of sample Collection	Results	Out of Range	Reference Range	Flag	Clinical Significance*
S202	206	JAIVAC-1	Dosage B	2	Potassium	mEq / L	Day 56	24/11/2010	4.7	.	3.5 - 5.1		
S202	206	JAIVAC-1	Dosage B	2	Random blood glucose	mg / dl	Day 56	24/11/2010	91	.	45-130		
S202	206	JAIVAC-1	Dosage B	2	Sodium	mEq / L	Day 56	24/11/2010	141	.	136 - 145		
S202	206	JAIVAC-1	Dosage B	3	ALT	U / L	Day 180	28/03/2011	17	.	0 - 41		
S202	206	JAIVAC-1	Dosage B	3	AST	U / L	Day 180	28/03/2011	11	.	0 - 40		
S202	206	JAIVAC-1	Dosage B	3	Alkaline Phosphatase	U / L	Day 180	28/03/2011	82	.	40 - 129		
S202	206	JAIVAC-1	Dosage B	3	Direct Bilirubin	mg / dl	Day 180	28/03/2011	0.13	.	0.00 - 0.30		
S202	206	JAIVAC-1	Dosage B	3	Gamma Glutamyl Transpeptidase	U / L	Day 180	28/03/2011	24	.	10 - 71		
S202	206	JAIVAC-1	Dosage B	3	Indirect Bilirubin	mg / dl	Day 180	28/03/2011	0.3	.	0.00 - 0.99		
S202	206	JAIVAC-1	Dosage B	3	Total Bilirubin	mg / dl	Day 180	28/03/2011	0.43	.	0.00 - 0.99		
S202	206	JAIVAC-1	Dosage B	3	Serum creatinine	mg / dl	Day 180	28/03/2011	1	.	0.7 - 1.2		
S202	206	JAIVAC-1	Dosage B	3	Potassium	mEq / L	Day 180	28/03/2011	4.4	.	3.5 - 5.1		
S202	206	JAIVAC-1	Dosage B	3	Random blood glucose	mg / dl	Day 180	28/03/2011	65	.	45-130		
S202	206	JAIVAC-1	Dosage B	3	Sodium	mEq / L	Day 180	28/03/2011	141	.	136 - 145		
S202	206	JAIVAC-1	Dosage B	3	ALT	U / L	Day 208	25/04/2011	29	.	0 - 41		
S202	206	JAIVAC-1	Dosage B	3	AST	U / L	Day 208	25/04/2011	16	.	0 - 40		
S202	206	JAIVAC-1	Dosage B	3	Alkaline Phosphatase	U / L	Day 208	25/04/2011	93	.	40 - 129		
S202	206	JAIVAC-1	Dosage B	3	Direct Bilirubin	mg / dl	Day 208	25/04/2011	0.17	.	0.00 - 0.30		
S202	206	JAIVAC-1	Dosage B	3	Gamma Glutamyl Transpeptidase	U / L	Day 208	25/04/2011	27	.	10 - 71		
S202	206	JAIVAC-1	Dosage B	3	Indirect Bilirubin	mg / dl	Day 208	25/04/2011	0.29	.	0.00 - 0.99		
S202	206	JAIVAC-1	Dosage B	3	Total Bilirubin	mg / dl	Day 208	25/04/2011	0.46	.	0.00 - 0.99		
S202	206	JAIVAC-1	Dosage B	3	Serum creatinine	mg / dl	Day 208	25/04/2011	1	.	0.7 - 1.2		
S202	206	JAIVAC-1	Dosage B	3	Potassium	mEq / L	Day 208	25/04/2011	4.4	.	3.5 - 5.1		
S202	206	JAIVAC-1	Dosage B	3	Random blood glucose	mg / dl	Day 208	25/04/2011	88	.	45-130		
S202	206	JAIVAC-1	Dosage B	3	Sodium	mEq / L	Day 208	25/04/2011	143	.	136 - 145		
S202	206	JAIVAC-1	Dosage B	3	ALT	U / L	Day 365	29/09/2011	26	.	0 - 41		
S202	206	JAIVAC-1	Dosage B	3	AST	U / L	Day 365	29/09/2011	16	.	0 - 40		
S202	206	JAIVAC-1	Dosage B	3	Alkaline Phosphatase	U / L	Day 365	29/09/2011	83	.	40 - 129		
S202	206	JAIVAC-1	Dosage B	3	Direct Bilirubin	mg / dl	Day 365	29/09/2011	0.16	.	0.00 - 0.30		

Subject No.	Randomisation Number	Study Group	Dosage	Dose	Serum Chemistry Parameters	Unit	Day	Date of sample Collection	Results	Out of Range	Reference Range	Flag	Clinical Significance*
S202	206	JAIVAC-1	Dosage B	3	Gamma Glutamyl Transpeptidase	U / L	Day 365	29/09/2011	26	.	10 - 71		
S202	206	JAIVAC-1	Dosage B	3	Indirect Bilirubin	mg / dl	Day 365	29/09/2011	0.34	.	0.00 - 0.99		
S202	206	JAIVAC-1	Dosage B	3	Total Bilirubin	mg / dl	Day 365	29/09/2011	0.5	.	0.00 - 0.99		
S202	206	JAIVAC-1	Dosage B	3	Serum creatinine	mg / dl	Day 365	29/09/2011	1	.	0.7 - 1.2		
S202	206	JAIVAC-1	Dosage B	3	Potassium	mEq / L	Day 365	29/09/2011	4.5	.	3.5 - 5.1		
S202	206	JAIVAC-1	Dosage B	3	Random blood glucose	mg / dl	Day 365	29/09/2011	73	.	45-130		
S202	206	JAIVAC-1	Dosage B	3	Sodium	mEq / L	Day 365	29/09/2011	140	.	136 - 145		
S203	211	Hepatitis B	Hepatitis B		ALT	U / L	Day -14 to Day -1	24/09/2010	19	.	0 - 41		
S203	211	Hepatitis B	Hepatitis B		AST	U / L	Day -14 to Day -1	24/09/2010	20	.	0 - 40		
S203	211	Hepatitis B	Hepatitis B		Alkaline Phosphatase	U / L	Day -14 to Day -1	24/09/2010	82	.	40 - 129		
S203	211	Hepatitis B	Hepatitis B		Direct Bilirubin	mg / dl	Day -14 to Day -1	24/09/2010	0.18	.	0.00 - 0.30		
S203	211	Hepatitis B	Hepatitis B		Gamma Glutamyl Transpeptidase	U / L	Day -14 to Day -1	24/09/2010	17	.	10 - 71		
S203	211	Hepatitis B	Hepatitis B		Indirect Bilirubin	mg / dl	Day -14 to Day -1	24/09/2010	0.45	.	0.00 - 0.99		
S203	211	Hepatitis B	Hepatitis B		Total Bilirubin	mg / dl	Day -14 to Day -1	24/09/2010	0.63	.	0.00 - 0.99		
S203	211	Hepatitis B	Hepatitis B		Serum creatinine	mg / dl	Day -14 to Day -1	24/09/2010	0.8	.	0.7 - 1.2		
S203	211	Hepatitis B	Hepatitis B		Potassium	mEq / L	Day -14 to Day -1	24/09/2010	4.1	.	3.5 - 5.1		
S203	211	Hepatitis B	Hepatitis B		Random blood glucose	mg / dl	Day -14 to Day -1	24/09/2010	88	.	45-130		
S203	211	Hepatitis B	Hepatitis B		Sodium	mEq / L	Day -14 to Day -1	24/09/2010	139	.	136 - 145		
S203	211	Hepatitis B	Hepatitis B	2	ALT	U / L	Day 28	28/10/2010	13	.	0 - 41		
S203	211	Hepatitis B	Hepatitis B	2	AST	U / L	Day 28	28/10/2010	20	.	0 - 40		
S203	211	Hepatitis B	Hepatitis B	2	Alkaline Phosphatase	U / L	Day 28	28/10/2010	93	.	40 - 129		
S203	211	Hepatitis B	Hepatitis B	2	Direct Bilirubin	mg / dl	Day 28	28/10/2010	0.11	.	0.00 - 0.30		
S203	211	Hepatitis B	Hepatitis B	2	Gamma Glutamyl Transpeptidase	U / L	Day 28	28/10/2010	17	.	10 - 71		
S203	211	Hepatitis B	Hepatitis B	2	Indirect Bilirubin	mg / dl	Day 28	28/10/2010	0.28	.	0.00 - 0.99		
S203	211	Hepatitis B	Hepatitis B	2	Total Bilirubin	mg / dl	Day 28	28/10/2010	0.39	.	0.00 - 0.99		
S203	211	Hepatitis B	Hepatitis B	2	Serum creatinine	mg / dl	Day 28	28/10/2010	0.9	.	0.7 - 1.2		
S203	211	Hepatitis B	Hepatitis B	2	Potassium	mEq / L	Day 28	28/10/2010	4.1	.	3.5 - 5.1		
S203	211	Hepatitis B	Hepatitis B	2	Random blood glucose	mg / dl	Day 28	28/10/2010	107	.	45-130		
S203	211	Hepatitis B	Hepatitis B	2	Sodium	mEq / L	Day 28	28/10/2010	139	.	136 - 145		

Subject No.	Randomisation Number	Study Group	Dosage	Dose	Serum Chemistry Parameters	Unit	Day	Date of sample Collection	Results	Out of Range	Reference Range	Flag	Clinical Significance*
S203	211	Hepatitis B	Hepatitis B	2	ALT	U / L	Day 56	25/11/2010	20	.	0 - 41		
S203	211	Hepatitis B	Hepatitis B	2	AST	U / L	Day 56	25/11/2010	25	.	0 - 40		
S203	211	Hepatitis B	Hepatitis B	2	Alkaline Phosphatase	U / L	Day 56	25/11/2010	99	.	40 - 129		
S203	211	Hepatitis B	Hepatitis B	2	Direct Bilirubin	mg / dl	Day 56	25/11/2010	0.13	.	0.00 - 0.30		
S203	211	Hepatitis B	Hepatitis B	2	Gamma Glutamyl Transpeptidase	U / L	Day 56	25/11/2010	17	.	10 - 71		
S203	211	Hepatitis B	Hepatitis B	2	Indirect Bilirubin	mg / dl	Day 56	25/11/2010	0.28	.	0.00 - 0.99		
S203	211	Hepatitis B	Hepatitis B	2	Total Bilirubin	mg / dl	Day 56	25/11/2010	0.41	.	0.00 - 0.99		
S203	211	Hepatitis B	Hepatitis B	2	Serum creatinine	mg / dl	Day 56	25/11/2010	1	.	0.7 - 1.2		
S203	211	Hepatitis B	Hepatitis B	2	Potassium	mEq / L	Day 56	25/11/2010	4.4	.	3.5 - 5.1		
S203	211	Hepatitis B	Hepatitis B	2	Random blood glucose	mg / dl	Day 56	25/11/2010	88	.	45-130		
S203	211	Hepatitis B	Hepatitis B	2	Sodium	mEq / L	Day 56	25/11/2010	140	.	136 - 145		
S203	211	Hepatitis B	Hepatitis B	3	ALT	U / L	Day 180	29/03/2011	21	.	0 - 41		
S203	211	Hepatitis B	Hepatitis B	3	AST	U / L	Day 180	29/03/2011	21	.	0 - 40		
S203	211	Hepatitis B	Hepatitis B	3	Alkaline Phosphatase	U / L	Day 180	29/03/2011	93	.	40 - 129		
S203	211	Hepatitis B	Hepatitis B	3	Direct Bilirubin	mg / dl	Day 180	29/03/2011	<0.10	.	0.00 - 0.30		
S203	211	Hepatitis B	Hepatitis B	3	Gamma Glutamyl Transpeptidase	U / L	Day 180	29/03/2011	21	.	10 - 71		
S203	211	Hepatitis B	Hepatitis B	3	Indirect Bilirubin	mg / dl	Day 180	29/03/2011	NA	.	0.00 - 0.99		
S203	211	Hepatitis B	Hepatitis B	3	Total Bilirubin	mg / dl	Day 180	29/03/2011	0.36	.	0.00 - 0.99		
S203	211	Hepatitis B	Hepatitis B	3	Serum creatinine	mg / dl	Day 180	29/03/2011	0.9	.	0.7 - 1.2		
S203	211	Hepatitis B	Hepatitis B	3	Potassium	mEq / L	Day 180	29/03/2011	4.1	.	3.5 - 5.1		
S203	211	Hepatitis B	Hepatitis B	3	Random blood glucose	mg / dl	Day 180	29/03/2011	77	.	45-130		
S203	211	Hepatitis B	Hepatitis B	3	Sodium	mEq / L	Day 180	29/03/2011	138	.	136 - 145		
S203	211	Hepatitis B	Hepatitis B	3	ALT	U / L	Day 208	28/04/2011	23	.	0 - 41		
S203	211	Hepatitis B	Hepatitis B	3	AST	U / L	Day 208	28/04/2011	21	.	0 - 40		
S203	211	Hepatitis B	Hepatitis B	3	Alkaline Phosphatase	U / L	Day 208	28/04/2011	101	.	40 - 129		
S203	211	Hepatitis B	Hepatitis B	3	Direct Bilirubin	mg / dl	Day 208	28/04/2011	0.18	.	0.00 - 0.30		
S203	211	Hepatitis B	Hepatitis B	3	Gamma Glutamyl Transpeptidase	U / L	Day 208	28/04/2011	24	.	10 - 71		
S203	211	Hepatitis B	Hepatitis B	3	Indirect Bilirubin	mg / dl	Day 208	28/04/2011	0.33	.	0.00 - 0.99		
S203	211	Hepatitis B	Hepatitis B	3	Total Bilirubin	mg / dl	Day 208	28/04/2011	0.51	.	0.00 - 0.99		

Subject No.	Randomisation Number	Study Group	Dosage	Dose	Serum Chemistry Parameters	Unit	Day	Date of sample Collection	Results	Out of Range	Reference Range	Flag	Clinical Significance*
S203	211	Hepatitis B	Hepatitis B	3	Serum creatinine	mg / dl	Day 208	28/04/2011	1	.	0.7 - 1.2		
S203	211	Hepatitis B	Hepatitis B	3	Potassium	mEq / L	Day 208	28/04/2011	4	.	3.5 - 5.1		
S203	211	Hepatitis B	Hepatitis B	3	Random blood glucose	mg / dl	Day 208	28/04/2011	107	.	45-130		
S203	211	Hepatitis B	Hepatitis B	3	Sodium	mEq / L	Day 208	28/04/2011	141	.	136 - 145		
S203	211	Hepatitis B	Hepatitis B	3	ALT	U / L	Day 365	29/09/2011	21	.	0 - 41		
S203	211	Hepatitis B	Hepatitis B	3	AST	U / L	Day 365	29/09/2011	23	.	0 - 40		
S203	211	Hepatitis B	Hepatitis B	3	Alkaline Phosphatase	U / L	Day 365	29/09/2011	91	.	40 - 129		
S203	211	Hepatitis B	Hepatitis B	3	Direct Bilirubin	mg / dl	Day 365	29/09/2011	0.18	.	0.00 - 0.30		
S203	211	Hepatitis B	Hepatitis B	3	Gamma Glutamyl Transpeptidase	U / L	Day 365	29/09/2011	16	.	10 - 71		
S203	211	Hepatitis B	Hepatitis B	3	Indirect Bilirubin	mg / dl	Day 365	29/09/2011	0.41	.	0.00 - 0.99		
S203	211	Hepatitis B	Hepatitis B	3	Total Bilirubin	mg / dl	Day 365	29/09/2011	0.59	.	0.00 - 0.99		
S203	211	Hepatitis B	Hepatitis B	3	Serum creatinine	mg / dl	Day 365	29/09/2011	1.1	.	0.7 - 1.2		
S203	211	Hepatitis B	Hepatitis B	3	Potassium	mEq / L	Day 365	29/09/2011	4.3	.	3.5 - 5.1		
S203	211	Hepatitis B	Hepatitis B	3	Random blood glucose	mg / dl	Day 365	29/09/2011	83	.	45-130		
S203	211	Hepatitis B	Hepatitis B	3	Sodium	mEq / L	Day 365	29/09/2011	138	.	136 - 145		
S212	212	JAIVAC-1	Dosage B		ALT	U / L	Day -14 to Day -1	23/09/2010	21	.	0 - 41		
S212	212	JAIVAC-1	Dosage B		AST	U / L	Day -14 to Day -1	23/09/2010	26	.	0 - 40		
S212	212	JAIVAC-1	Dosage B		Alkaline Phosphatase	U / L	Day -14 to Day -1	23/09/2010	88	.	40 - 129		
S212	212	JAIVAC-1	Dosage B		Direct Bilirubin	mg / dl	Day -14 to Day -1	23/09/2010	0.13	.	0.00 - 0.30		
S212	212	JAIVAC-1	Dosage B		Gamma Glutamyl Transpeptidase	U / L	Day -14 to Day -1	23/09/2010	22	.	10 - 71		
S212	212	JAIVAC-1	Dosage B		Indirect Bilirubin	mg / dl	Day -14 to Day -1	23/09/2010	0.29	.	0.00 - 0.99		
S212	212	JAIVAC-1	Dosage B		Total Bilirubin	mg / dl	Day -14 to Day -1	23/09/2010	0.42	.	0.00 - 0.99		
S212	212	JAIVAC-1	Dosage B		Serum creatinine	mg / dl	Day -14 to Day -1	23/09/2010	1	.	0.7 - 1.2		
S212	212	JAIVAC-1	Dosage B		Potassium	mEq / L	Day -14 to Day -1	23/09/2010	4.1	.	3.5 - 5.1		
S212	212	JAIVAC-1	Dosage B		Random blood glucose	mg / dl	Day -14 to Day -1	23/09/2010	90	.	45-130		
S212	212	JAIVAC-1	Dosage B		Sodium	mEq / L	Day -14 to Day -1	23/09/2010	141	.	136 - 145		
S212	212	JAIVAC-1	Dosage B	2	ALT	U / L	Day 28	28/10/2010	18	.	0 - 41		
S212	212	JAIVAC-1	Dosage B	2	AST	U / L	Day 28	28/10/2010	22	.	0 - 40		
S212	212	JAIVAC-1	Dosage B	2	Alkaline Phosphatase	U / L	Day 28	28/10/2010	93	.	40 - 129		

Subject No.	Randomisation Number	Study Group	Dosage	Dose	Serum Chemistry Parameters	Unit	Day	Date of sample Collection	Results	Out of Range	Reference Range	Flag	Clinical Significance*
S212	212	JAIVAC-1	Dosage B	2	Direct Bilirubin	mg / dl	Day 28	28/10/2010	0.11	.	0.00 - 0.30		
S212	212	JAIVAC-1	Dosage B	2	Gamma Glutamyl Transpeptidase	U / L	Day 28	28/10/2010	57	.	10 - 71		
S212	212	JAIVAC-1	Dosage B	2	Indirect Bilirubin	mg / dl	Day 28	28/10/2010	0.42	.	0.00 - 0.99		
S212	212	JAIVAC-1	Dosage B	2	Total Bilirubin	mg / dl	Day 28	28/10/2010	0.53	.	0.00 - 0.99		
S212	212	JAIVAC-1	Dosage B	2	Serum creatinine	mg / dl	Day 28	28/10/2010	1	.	0.7 - 1.2		
S212	212	JAIVAC-1	Dosage B	2	Potassium	mEq / L	Day 28	28/10/2010	4.7	.	3.5 - 5.1		
S212	212	JAIVAC-1	Dosage B	2	Random blood glucose	mg / dl	Day 28	28/10/2010	86	.	45-130		
S212	212	JAIVAC-1	Dosage B	2	Sodium	mEq / L	Day 28	28/10/2010	139	.	136 - 145		
S212	212	JAIVAC-1	Dosage B	2	ALT	U / L	Day 56	25/11/2010	18	.	0 - 41		
S212	212	JAIVAC-1	Dosage B	2	AST	U / L	Day 56	25/11/2010	24	.	0 - 40		
S212	212	JAIVAC-1	Dosage B	2	Alkaline Phosphatase	U / L	Day 56	25/11/2010	80	.	40 - 129		
S212	212	JAIVAC-1	Dosage B	2	Direct Bilirubin	mg / dl	Day 56	25/11/2010	0.21	.	0.00 - 0.30		
S212	212	JAIVAC-1	Dosage B	2	Gamma Glutamyl Transpeptidase	U / L	Day 56	25/11/2010	28	.	10 - 71		
S212	212	JAIVAC-1	Dosage B	2	Indirect Bilirubin	mg / dl	Day 56	25/11/2010	0.44	.	0.00 - 0.99		
S212	212	JAIVAC-1	Dosage B	2	Total Bilirubin	mg / dl	Day 56	25/11/2010	0.65	.	0.00 - 0.99		
S212	212	JAIVAC-1	Dosage B	2	Serum creatinine	mg / dl	Day 56	25/11/2010	1.1	.	0.7 - 1.2		
S212	212	JAIVAC-1	Dosage B	2	Potassium	mEq / L	Day 56	25/11/2010	4.2	.	3.5 - 5.1		
S212	212	JAIVAC-1	Dosage B	2	Random blood glucose	mg / dl	Day 56	25/11/2010	95	.	45-130		
S212	212	JAIVAC-1	Dosage B	2	Sodium	mEq / L	Day 56	25/11/2010	140	.	136 - 145		
S212	212	JAIVAC-1	Dosage B	3	ALT	U / L	Day 180	29/03/2011	31	.	0 - 41		
S212	212	JAIVAC-1	Dosage B	3	AST	U / L	Day 180	29/03/2011	28	.	0 - 40		
S212	212	JAIVAC-1	Dosage B	3	Alkaline Phosphatase	U / L	Day 180	29/03/2011	97	.	40 - 129		
S212	212	JAIVAC-1	Dosage B	3	Direct Bilirubin	mg / dl	Day 180	29/03/2011	0.14	.	0.00 - 0.30		
S212	212	JAIVAC-1	Dosage B	3	Gamma Glutamyl Transpeptidase	U / L	Day 180	29/03/2011	40	.	10 - 71		
S212	212	JAIVAC-1	Dosage B	3	Indirect Bilirubin	mg / dl	Day 180	29/03/2011	0.27	.	0.00 - 0.99		
S212	212	JAIVAC-1	Dosage B	3	Total Bilirubin	mg / dl	Day 180	29/03/2011	0.41	.	0.00 - 0.99		
S212	212	JAIVAC-1	Dosage B	3	Serum creatinine	mg / dl	Day 180	29/03/2011	1	.	0.7 - 1.2		
S212	212	JAIVAC-1	Dosage B	3	Potassium	mEq / L	Day 180	29/03/2011	3.8	.	3.5 - 5.1		
S212	212	JAIVAC-1	Dosage B	3	Random blood glucose	mg / dl	Day 180	29/03/2011	80	.	45-130		

Subject No.	Randomisation Number	Study Group	Dosage	Dose	Serum Chemistry Parameters	Unit	Day	Date of sample Collection	Results	Out of Range	Reference Range	Flag	Clinical Significance*
S212	212	JAIVAC-1	Dosage B	3	Sodium	mEq / L	Day 180	29/03/2011	141	.	136 - 145		
S212	212	JAIVAC-1	Dosage B	3	ALT	U / L	Day 208	19/04/2011	19	.	0 - 41		
S212	212	JAIVAC-1	Dosage B	3	AST	U / L	Day 208	19/04/2011	17	.	0 - 40		
S212	212	JAIVAC-1	Dosage B	3	Alkaline Phosphatase	U / L	Day 208	19/04/2011	87	.	40 - 129		
S212	212	JAIVAC-1	Dosage B	3	Direct Bilirubin	mg / dl	Day 208	19/04/2011	0.12	.	0.00 - 0.30		
S212	212	JAIVAC-1	Dosage B	3	Gamma Glutamyl Transpeptidase	U / L	Day 208	19/04/2011	23	.	10 - 71		
S212	212	JAIVAC-1	Dosage B	3	Indirect Bilirubin	mg / dl	Day 208	19/04/2011	0.26	.	0.00 - 0.99		
S212	212	JAIVAC-1	Dosage B	3	Total Bilirubin	mg / dl	Day 208	19/04/2011	0.38	.	0.00 - 0.99		
S212	212	JAIVAC-1	Dosage B	3	Serum creatinine	mg / dl	Day 208	19/04/2011	1	.	0.7 - 1.2		
S212	212	JAIVAC-1	Dosage B	3	Potassium	mEq / L	Day 208	19/04/2011	4.4	.	3.5 - 5.1		
S212	212	JAIVAC-1	Dosage B	3	Random blood glucose	mg / dl	Day 208	19/04/2011	89	.	45-130		
S212	212	JAIVAC-1	Dosage B	3	Sodium	mEq / L	Day 208	19/04/2011	141	.	136 - 145		
S212	212	JAIVAC-1	Dosage B	3	ALT	U / L	Day 365	29/09/2011	22	.	0 - 41		
S212	212	JAIVAC-1	Dosage B	3	AST	U / L	Day 365	29/09/2011	26	.	0 - 40		
S212	212	JAIVAC-1	Dosage B	3	Alkaline Phosphatase	U / L	Day 365	29/09/2011	80	.	40 - 129		
S212	212	JAIVAC-1	Dosage B	3	Direct Bilirubin	mg / dl	Day 365	29/09/2011	0.17	.	0.00 - 0.30		
S212	212	JAIVAC-1	Dosage B	3	Gamma Glutamyl Transpeptidase	U / L	Day 365	29/09/2011	20	.	10 - 71		
S212	212	JAIVAC-1	Dosage B	3	Indirect Bilirubin	mg / dl	Day 365	29/09/2011	0.29	.	0.00 - 0.99		
S212	212	JAIVAC-1	Dosage B	3	Total Bilirubin	mg / dl	Day 365	29/09/2011	0.46	.	0.00 - 0.99		
S212	212	JAIVAC-1	Dosage B	3	Serum creatinine	mg / dl	Day 365	29/09/2011	1.2	.	0.7 - 1.2		
S212	212	JAIVAC-1	Dosage B	3	Potassium	mEq / L	Day 365	29/09/2011	4.2	.	3.5 - 5.1		
S212	212	JAIVAC-1	Dosage B	3	Random blood glucose	mg / dl	Day 365	29/09/2011	85	.	45-130		
S212	212	JAIVAC-1	Dosage B	3	Sodium	mEq / L	Day 365	29/09/2011	139	.	136 - 145		
S215	213	Hepatitis B	Hepatitis B		ALT	U / L	Day -14 to Day -1	24/09/2010	16	.	0 - 41		
S215	213	Hepatitis B	Hepatitis B		AST	U / L	Day -14 to Day -1	24/09/2010	19	.	0 - 40		
S215	213	Hepatitis B	Hepatitis B		Alkaline Phosphatase	U / L	Day -14 to Day -1	24/09/2010	71	.	40 - 129		
S215	213	Hepatitis B	Hepatitis B		Direct Bilirubin	mg / dl	Day -14 to Day -1	24/09/2010	0.21	.	0.00 - 0.30		
S215	213	Hepatitis B	Hepatitis B		Gamma Glutamyl Transpeptidase	U / L	Day -14 to Day -1	24/09/2010	9	.	10 - 71	L	
S215	213	Hepatitis B	Hepatitis B		Indirect Bilirubin	mg / dl	Day -14 to Day -1	24/09/2010	0.49	.	0.00 - 0.99		

Subject No.	Randomisation Number	Study Group	Dosage	Dose	Serum Chemistry Parameters	Unit	Day	Date of sample Collection	Results	Out of Range	Reference Range	Flag	Clinical Significance*
S215	213	Hepatitis B	Hepatitis B		Total Bilirubin	mg / dl	Day -14 to Day -1	24/09/2010	0.7	.	0.00 - 0.99		
S215	213	Hepatitis B	Hepatitis B		Serum creatinine	mg / dl	Day -14 to Day -1	24/09/2010	0.8	.	0.7 - 1.2		
S215	213	Hepatitis B	Hepatitis B		Potassium	mEq / L	Day -14 to Day -1	24/09/2010	4.3	.	3.5 - 5.1		
S215	213	Hepatitis B	Hepatitis B		Random blood glucose	mg / dl	Day -14 to Day -1	24/09/2010	90	.	45-130		
S215	213	Hepatitis B	Hepatitis B		Sodium	mEq / L	Day -14 to Day -1	24/09/2010	139	.	136 - 145		
S215	213	Hepatitis B	Hepatitis B	2	ALT	U / L	Day 28	28/10/2010	18	.	0 - 41		
S215	213	Hepatitis B	Hepatitis B	2	AST	U / L	Day 28	28/10/2010	22	.	0 - 40		
S215	213	Hepatitis B	Hepatitis B	2	Alkaline Phosphatase	U / L	Day 28	28/10/2010	64	.	40 - 129		
S215	213	Hepatitis B	Hepatitis B	2	Direct Bilirubin	mg / dl	Day 28	28/10/2010	0.31	Yes	0.00 - 0.30	H	NCS
S215	213	Hepatitis B	Hepatitis B	2	Gamma Glutamyl Transpeptidase	U / L	Day 28	28/10/2010	8	Yes	10 - 71	L	NCS
S215	213	Hepatitis B	Hepatitis B	2	Indirect Bilirubin	mg / dl	Day 28	28/10/2010	0.82	.	0.00 - 0.99		
S215	213	Hepatitis B	Hepatitis B	2	Total Bilirubin	mg / dl	Day 28	28/10/2010	1.13	Yes	0.00 - 0.99	H	NCS
S215	213	Hepatitis B	Hepatitis B	2	Serum creatinine	mg / dl	Day 28	28/10/2010	0.8	.	0.7 - 1.2		
S215	213	Hepatitis B	Hepatitis B	2	Potassium	mEq / L	Day 28	28/10/2010	4.1	.	3.5 - 5.1		
S215	213	Hepatitis B	Hepatitis B	2	Random blood glucose	mg / dl	Day 28	28/10/2010	90	.	45-130		
S215	213	Hepatitis B	Hepatitis B	2	Sodium	mEq / L	Day 28	28/10/2010	141	.	136 - 145		
S215	213	Hepatitis B	Hepatitis B	2	ALT	U / L	Day 56	25/11/2010	16	.	0 - 41		
S215	213	Hepatitis B	Hepatitis B	2	AST	U / L	Day 56	25/11/2010	22	.	0 - 40		
S215	213	Hepatitis B	Hepatitis B	2	Alkaline Phosphatase	U / L	Day 56	25/11/2010	67	.	40 - 129		
S215	213	Hepatitis B	Hepatitis B	2	Direct Bilirubin	mg / dl	Day 56	25/11/2010	0.37	Yes	0.00 - 0.30	H	NCS
S215	213	Hepatitis B	Hepatitis B	2	Gamma Glutamyl Transpeptidase	U / L	Day 56	25/11/2010	8	Yes	10 - 71	L	NCS
S215	213	Hepatitis B	Hepatitis B	2	Indirect Bilirubin	mg / dl	Day 56	25/11/2010	0.69	.	0.00 - 0.99		
S215	213	Hepatitis B	Hepatitis B	2	Total Bilirubin	mg / dl	Day 56	25/11/2010	1.06	Yes	0.00 - 0.99	H	NCS
S215	213	Hepatitis B	Hepatitis B	2	Serum creatinine	mg / dl	Day 56	25/11/2010	0.9	.	0.7 - 1.2		
S215	213	Hepatitis B	Hepatitis B	2	Potassium	mEq / L	Day 56	25/11/2010	4.7	.	3.5 - 5.1		
S215	213	Hepatitis B	Hepatitis B	2	Random blood glucose	mg / dl	Day 56	25/11/2010	86	.	45-130		
S215	213	Hepatitis B	Hepatitis B	2	Sodium	mEq / L	Day 56	25/11/2010	140	.	136 - 145		
S215	213	Hepatitis B	Hepatitis B	3	ALT	U / L	Day 180	29/03/2011	32	.	0 - 41		
S215	213	Hepatitis B	Hepatitis B	3	AST	U / L	Day 180	29/03/2011	27	.	0 - 40		

Subject No.	Randomisation Number	Study Group	Dosage	Dose	Serum Chemistry Parameters	Unit	Day	Date of sample Collection	Results	Out of Range	Reference Range	Flag	Clinical Significance*
S215	213	Hepatitis B	Hepatitis B	3	Alkaline Phosphatase	U / L	Day 180	29/03/2011	63	.	40 - 129		
S215	213	Hepatitis B	Hepatitis B	3	Direct Bilirubin	mg / dl	Day 180	29/03/2011	0.44	Yes	0.00 - 0.30	H	CS
S215	213	Hepatitis B	Hepatitis B	3	Gamma Glutamyl Transpeptidase	U / L	Day 180	29/03/2011	10	.	10 - 71		
S215	213	Hepatitis B	Hepatitis B	3	Indirect Bilirubin	mg / dl	Day 180	29/03/2011	1.08	Yes	0.00 - 0.99	H	CS
S215	213	Hepatitis B	Hepatitis B	3	Total Bilirubin	mg / dl	Day 180	29/03/2011	1.52	Yes	0.00 - 0.99	H	CS
S215	213	Hepatitis B	Hepatitis B	3	Serum creatinine	mg / dl	Day 180	29/03/2011	0.9	.	0.7 - 1.2		
S215	213	Hepatitis B	Hepatitis B	3	Potassium	mEq / L	Day 180	29/03/2011	4	.	3.5 - 5.1		
S215	213	Hepatitis B	Hepatitis B	3	Random blood glucose	mg / dl	Day 180	29/03/2011	90	.	45-130		
S215	213	Hepatitis B	Hepatitis B	3	Sodium	mEq / L	Day 180	29/03/2011	138	.	136 - 145		
S215	213	Hepatitis B	Hepatitis B	3	ALT	U / L	Day 208	26/04/2011	21	.	0 - 41		
S215	213	Hepatitis B	Hepatitis B	3	AST	U / L	Day 208	26/04/2011	23	.	0 - 40		
S215	213	Hepatitis B	Hepatitis B	3	Alkaline Phosphatase	U / L	Day 208	26/04/2011	65	.	40 - 129		
S215	213	Hepatitis B	Hepatitis B	3	Direct Bilirubin	mg / dl	Day 208	26/04/2011	0.39	Yes	0.00 - 0.30	H	CS
S215	213	Hepatitis B	Hepatitis B	3	Gamma Glutamyl Transpeptidase	U / L	Day 208	26/04/2011	8	Yes	10 - 71	L	NCS
S215	213	Hepatitis B	Hepatitis B	3	Indirect Bilirubin	mg / dl	Day 208	26/04/2011	0.84	.	0.00 - 0.99		
S215	213	Hepatitis B	Hepatitis B	3	Total Bilirubin	mg / dl	Day 208	26/04/2011	1.23	Yes	0.00 - 0.99	H	CS
S215	213	Hepatitis B	Hepatitis B	3	Serum creatinine	mg / dl	Day 208	26/04/2011	1	.	0.7 - 1.2		
S215	213	Hepatitis B	Hepatitis B	3	Potassium	mEq / L	Day 208	26/04/2011	4.2	.	3.5 - 5.1		
S215	213	Hepatitis B	Hepatitis B	3	Random blood glucose	mg / dl	Day 208	26/04/2011	81	.	45-130		
S215	213	Hepatitis B	Hepatitis B	3	Sodium	mEq / L	Day 208	26/04/2011	141	.	136 - 145		
S215	213	Hepatitis B	Hepatitis B	3	ALT	U / L	Day 365	29/09/2011	60	Yes	0 - 41	H	CS
S215	213	Hepatitis B	Hepatitis B	3	AST	U / L	Day 365	29/09/2011	37	.	0 - 40		
S215	213	Hepatitis B	Hepatitis B	3	Alkaline Phosphatase	U / L	Day 365	29/09/2011	55	.	40 - 129		
S215	213	Hepatitis B	Hepatitis B	3	Direct Bilirubin	mg / dl	Day 365	29/09/2011	0.34	Yes	0.00 - 0.30	H	NCS
S215	213	Hepatitis B	Hepatitis B	3	Gamma Glutamyl Transpeptidase	U / L	Day 365	29/09/2011	9	Yes	10 - 71	L	NCS
S215	213	Hepatitis B	Hepatitis B	3	Indirect Bilirubin	mg / dl	Day 365	29/09/2011	0.83	.	0.00 - 0.99		
S215	213	Hepatitis B	Hepatitis B	3	Total Bilirubin	mg / dl	Day 365	29/09/2011	1.17	Yes	0.00 - 0.99	H	NCS
S215	213	Hepatitis B	Hepatitis B	3	Serum creatinine	mg / dl	Day 365	29/09/2011	1	.	0.7 - 1.2		
S215	213	Hepatitis B	Hepatitis B	3	Potassium	mEq / L	Day 365	29/09/2011	4.2	.	3.5 - 5.1		

Subject No.	Randomisation Number	Study Group	Dosage	Dose	Serum Chemistry Parameters	Unit	Day	Date of sample Collection	Results	Out of Range	Reference Range	Flag	Clinical Significance*
S215	213	Hepatitis B	Hepatitis B	3	Random blood glucose	mg / dl	Day 365	29/09/2011	61	.	45-130		
S215	213	Hepatitis B	Hepatitis B	3	Sodium	mEq / L	Day 365	29/09/2011	141	.	136 - 145		
S233	210	JAIVAC-1	Dosage B		ALT	U / L	Day -14 to Day -1	24/09/2010	37	.	0 - 41		
S233	210	JAIVAC-1	Dosage B		AST	U / L	Day -14 to Day -1	24/09/2010	24	.	0 - 40		
S233	210	JAIVAC-1	Dosage B		Alkaline Phosphatase	U / L	Day -14 to Day -1	24/09/2010	72	.	40 - 129		
S233	210	JAIVAC-1	Dosage B		Direct Bilirubin	mg / dl	Day -14 to Day -1	24/09/2010	0.25	.	0.00 - 0.30		
S233	210	JAIVAC-1	Dosage B		Gamma Glutamyl Transpeptidase	U / L	Day -14 to Day -1	24/09/2010	23	.	10 - 71		
S233	210	JAIVAC-1	Dosage B		Indirect Bilirubin	mg / dl	Day -14 to Day -1	24/09/2010	0.52	.	0.00 - 0.99		
S233	210	JAIVAC-1	Dosage B		Total Bilirubin	mg / dl	Day -14 to Day -1	24/09/2010	0.77	.	0.00 - 0.99		
S233	210	JAIVAC-1	Dosage B		Serum creatinine	mg / dl	Day -14 to Day -1	24/09/2010	0.7	.	0.7 - 1.2		
S233	210	JAIVAC-1	Dosage B		Potassium	mEq / L	Day -14 to Day -1	24/09/2010	3.6	.	3.5 - 5.1		
S233	210	JAIVAC-1	Dosage B		Random blood glucose	mg / dl	Day -14 to Day -1	24/09/2010	93	.	45-130		
S233	210	JAIVAC-1	Dosage B		Sodium	mEq / L	Day -14 to Day -1	24/09/2010	140	.	136 - 145		
S233	210	JAIVAC-1	Dosage B	2	ALT	U / L	Day 28	28/10/2010	32	.	0 - 41		
S233	210	JAIVAC-1	Dosage B	2	AST	U / L	Day 28	28/10/2010	19	.	0 - 40		
S233	210	JAIVAC-1	Dosage B	2	Alkaline Phosphatase	U / L	Day 28	28/10/2010	60	.	40 - 129		
S233	210	JAIVAC-1	Dosage B	2	Direct Bilirubin	mg / dl	Day 28	28/10/2010	0.21	.	0.00 - 0.30		
S233	210	JAIVAC-1	Dosage B	2	Gamma Glutamyl Transpeptidase	U / L	Day 28	28/10/2010	18	.	10 - 71		
S233	210	JAIVAC-1	Dosage B	2	Indirect Bilirubin	mg / dl	Day 28	28/10/2010	0.57	.	0.00 - 0.99		
S233	210	JAIVAC-1	Dosage B	2	Total Bilirubin	mg / dl	Day 28	28/10/2010	0.78	.	0.00 - 0.99		
S233	210	JAIVAC-1	Dosage B	2	Serum creatinine	mg / dl	Day 28	28/10/2010	0.7	.	0.7 - 1.2		
S233	210	JAIVAC-1	Dosage B	2	Potassium	mEq / L	Day 28	28/10/2010	3.6	.	3.5 - 5.1		
S233	210	JAIVAC-1	Dosage B	2	Random blood glucose	mg / dl	Day 28	28/10/2010	112	.	45-130		
S233	210	JAIVAC-1	Dosage B	2	Sodium	mEq / L	Day 28	28/10/2010	142	.	136 - 145		
S233	210	JAIVAC-1	Dosage B	2	ALT	U / L	Day 56	25/11/2010	47	Yes	0 - 41	H	NCS
S233	210	JAIVAC-1	Dosage B	2	AST	U / L	Day 56	25/11/2010	30	.	0 - 40		
S233	210	JAIVAC-1	Dosage B	2	Alkaline Phosphatase	U / L	Day 56	25/11/2010	75	.	40 - 129		
S233	210	JAIVAC-1	Dosage B	2	Direct Bilirubin	mg / dl	Day 56	25/11/2010	0.2	.	0.00 - 0.30		
S233	210	JAIVAC-1	Dosage B	2	Gamma Glutamyl Transpeptidase	U / L	Day 56	25/11/2010	26	.	10 - 71		

Subject No.	Randomisation Number	Study Group	Dosage	Dose	Serum Chemistry Parameters	Unit	Day	Date of sample Collection	Results	Out of Range	Reference Range	Flag	Clinical Significance*
S233	210	JAIVAC-1	Dosage B	2	Indirect Bilirubin	mg / dl	Day 56	25/11/2010	0.38	.	0.00 - 0.99		
S233	210	JAIVAC-1	Dosage B	2	Total Bilirubin	mg / dl	Day 56	25/11/2010	0.58	.	0.00 - 0.99		
S233	210	JAIVAC-1	Dosage B	2	Serum creatinine	mg / dl	Day 56	25/11/2010	1	.	0.7 - 1.2		
S233	210	JAIVAC-1	Dosage B	2	Potassium	mEq / L	Day 56	25/11/2010	4.3	.	3.5 - 5.1		
S233	210	JAIVAC-1	Dosage B	2	Random blood glucose	mg / dl	Day 56	25/11/2010	89	.	45-130		
S233	210	JAIVAC-1	Dosage B	2	Sodium	mEq / L	Day 56	25/11/2010	141	.	136 - 145		
S233	210	JAIVAC-1	Dosage B	3	ALT	U / L	Day 180	29/03/2011	36	.	0 - 41		
S233	210	JAIVAC-1	Dosage B	3	AST	U / L	Day 180	29/03/2011	22	.	0 - 40		
S233	210	JAIVAC-1	Dosage B	3	Alkaline Phosphatase	U / L	Day 180	29/03/2011	72	.	40 - 129		
S233	210	JAIVAC-1	Dosage B	3	Direct Bilirubin	mg / dl	Day 180	29/03/2011	0.2	.	0.00 - 0.30		
S233	210	JAIVAC-1	Dosage B	3	Gamma Glutamyl Transpeptidase	U / L	Day 180	29/03/2011	24	.	10 - 71		
S233	210	JAIVAC-1	Dosage B	3	Indirect Bilirubin	mg / dl	Day 180	29/03/2011	0.38	.	0.00 - 0.99		
S233	210	JAIVAC-1	Dosage B	3	Total Bilirubin	mg / dl	Day 180	29/03/2011	0.58	.	0.00 - 0.99		
S233	210	JAIVAC-1	Dosage B	3	Serum creatinine	mg / dl	Day 180	29/03/2011	0.9	.	0.7 - 1.2		
S233	210	JAIVAC-1	Dosage B	3	Potassium	mEq / L	Day 180	29/03/2011	3.7	.	3.5 - 5.1		
S233	210	JAIVAC-1	Dosage B	3	Random blood glucose	mg / dl	Day 180	29/03/2011	84	.	45-130		
S233	210	JAIVAC-1	Dosage B	3	Sodium	mEq / L	Day 180	29/03/2011	139	.	136 - 145		
S233	210	JAIVAC-1	Dosage B	3	ALT	U / L	Day 208	27/04/2011	45	Yes	0 - 41	H	NCS
S233	210	JAIVAC-1	Dosage B	3	AST	U / L	Day 208	27/04/2011	25	.	0 - 40		
S233	210	JAIVAC-1	Dosage B	3	Alkaline Phosphatase	U / L	Day 208	27/04/2011	75	.	40 - 129		
S233	210	JAIVAC-1	Dosage B	3	Direct Bilirubin	mg / dl	Day 208	27/04/2011	0.16	.	0.00 - 0.30		
S233	210	JAIVAC-1	Dosage B	3	Gamma Glutamyl Transpeptidase	U / L	Day 208	27/04/2011	24	.	10 - 71		
S233	210	JAIVAC-1	Dosage B	3	Indirect Bilirubin	mg / dl	Day 208	27/04/2011	0.38	.	0.00 - 0.99		
S233	210	JAIVAC-1	Dosage B	3	Total Bilirubin	mg / dl	Day 208	27/04/2011	0.54	.	0.00 - 0.99		
S233	210	JAIVAC-1	Dosage B	3	Serum creatinine	mg / dl	Day 208	27/04/2011	0.8	.	0.7 - 1.2		
S233	210	JAIVAC-1	Dosage B	3	Potassium	mEq / L	Day 208	27/04/2011	5	.	3.5 - 5.1		
S233	210	JAIVAC-1	Dosage B	3	Random blood glucose	mg / dl	Day 208	27/04/2011	133	Yes	45-130	H	NCS
S233	210	JAIVAC-1	Dosage B	3	Sodium	mEq / L	Day 208	27/04/2011	142	.	136 - 145		
S233	210	JAIVAC-1	Dosage B	3	ALT	U / L	Day 365	29/09/2011	44	Yes	0 - 41	H	CS

Subject No.	Randomisation Number	Study Group	Dosage	Dose	Serum Chemistry Parameters	Unit	Day	Date of sample Collection	Results	Out of Range	Reference Range	Flag	Clinical Significance*
S233	210	JAIVAC-1	Dosage B	3	AST	U / L	Day 365	29/09/2011	25	.	0 - 40		
S233	210	JAIVAC-1	Dosage B	3	Alkaline Phosphatase	U / L	Day 365	29/09/2011	76	.	40 - 129		
S233	210	JAIVAC-1	Dosage B	3	Direct Bilirubin	mg / dl	Day 365	29/09/2011	0.23	.	0.00 - 0.30		
S233	210	JAIVAC-1	Dosage B	3	Gamma Glutamyl Transpeptidase	U / L	Day 365	29/09/2011	24	.	10 - 71		
S233	210	JAIVAC-1	Dosage B	3	Indirect Bilirubin	mg / dl	Day 365	29/09/2011	0.44	.	0.00 - 0.99		
S233	210	JAIVAC-1	Dosage B	3	Total Bilirubin	mg / dl	Day 365	29/09/2011	0.67	.	0.00 - 0.99		
S233	210	JAIVAC-1	Dosage B	3	Serum creatinine	mg / dl	Day 365	29/09/2011	1	.	0.7 - 1.2		
S233	210	JAIVAC-1	Dosage B	3	Potassium	mEq / L	Day 365	29/09/2011	4.2	.	3.5 - 5.1		
S233	210	JAIVAC-1	Dosage B	3	Random blood glucose	mg / dl	Day 365	29/09/2011	72	.	45-130		
S233	210	JAIVAC-1	Dosage B	3	Sodium	mEq / L	Day 365	29/09/2011	140	.	136 - 145		
S241	303	Hepatitis B	Hepatitis B		ALT	U / L	Day -14 to Day -1	03/11/2010	38	.	0 - 41		
S241	303	Hepatitis B	Hepatitis B		AST	U / L	Day -14 to Day -1	03/11/2010	22	.	0 - 40		
S241	303	Hepatitis B	Hepatitis B		Alkaline Phosphatase	U / L	Day -14 to Day -1	03/11/2010	56	.	40 - 129		
S241	303	Hepatitis B	Hepatitis B		Direct Bilirubin	mg / dl	Day -14 to Day -1	03/11/2010	0.18	.	0.00 - 0.30		
S241	303	Hepatitis B	Hepatitis B		Gamma Glutamyl Transpeptidase	U / L	Day -14 to Day -1	03/11/2010	20	.	10 - 71		
S241	303	Hepatitis B	Hepatitis B		Indirect Bilirubin	mg / dl	Day -14 to Day -1	03/11/2010	0.64	.	0.00 - 0.99		
S241	303	Hepatitis B	Hepatitis B		Total Bilirubin	mg / dl	Day -14 to Day -1	03/11/2010	0.82	.	0.00 - 0.99		
S241	303	Hepatitis B	Hepatitis B		Serum creatinine	mg / dl	Day -14 to Day -1	03/11/2010	0.9	.	0.7 - 1.2		
S241	303	Hepatitis B	Hepatitis B		Potassium	mEq / L	Day -14 to Day -1	03/11/2010	4.1	.	3.5 - 5.1		
S241	303	Hepatitis B	Hepatitis B		Random blood glucose	mg / dl	Day -14 to Day -1	03/11/2010	82	.	45-130		
S241	303	Hepatitis B	Hepatitis B		Sodium	mEq / L	Day -14 to Day -1	03/11/2010	138	.	136 - 145		
S241	303	Hepatitis B	Hepatitis B	2	ALT	U / L	Day 28	08/12/2010	22	.	0 - 41		
S241	303	Hepatitis B	Hepatitis B	2	AST	U / L	Day 28	08/12/2010	18	.	0 - 40		
S241	303	Hepatitis B	Hepatitis B	2	Alkaline Phosphatase	U / L	Day 28	08/12/2010	65	.	40 - 129		
S241	303	Hepatitis B	Hepatitis B	2	Direct Bilirubin	mg / dl	Day 28	08/12/2010	0.12	.	0.00 - 0.30		
S241	303	Hepatitis B	Hepatitis B	2	Gamma Glutamyl Transpeptidase	U / L	Day 28	08/12/2010	16	.	10 - 71		
S241	303	Hepatitis B	Hepatitis B	2	Indirect Bilirubin	mg / dl	Day 28	08/12/2010	0.35	.	0.00 - 0.99		
S241	303	Hepatitis B	Hepatitis B	2	Total Bilirubin	mg / dl	Day 28	08/12/2010	0.47	.	0.00 - 0.99		
S241	303	Hepatitis B	Hepatitis B	2	Serum creatinine	mg / dl	Day 28	08/12/2010	1	.	0.7 - 1.2		

Subject No.	Randomisation Number	Study Group	Dosage	Dose	Serum Chemistry Parameters	Unit	Day	Date of sample Collection	Results	Out of Range	Reference Range	Flag	Clinical Significance*
S241	303	Hepatitis B	Hepatitis B	2	Potassium	mEq / L	Day 28	08/12/2010	3.8	.	3.5 - 5.1		
S241	303	Hepatitis B	Hepatitis B	2	Random blood glucose	mg / dl	Day 28	08/12/2010	83	.	45-130		
S241	303	Hepatitis B	Hepatitis B	2	Sodium	mEq / L	Day 28	08/12/2010	139	.	136 - 145		
S241	303	Hepatitis B	Hepatitis B	2	ALT	U / L	Day 56	05/01/2011	49	Yes	0 - 41	H	NCS
S241	303	Hepatitis B	Hepatitis B	2	AST	U / L	Day 56	05/01/2011	28	.	0 - 40		
S241	303	Hepatitis B	Hepatitis B	2	Alkaline Phosphatase	U / L	Day 56	05/01/2011	57	.	40 - 129		
S241	303	Hepatitis B	Hepatitis B	2	Direct Bilirubin	mg / dl	Day 56	05/01/2011	0.12	.	0.00 - 0.30		
S241	303	Hepatitis B	Hepatitis B	2	Gamma Glutamyl Transpeptidase	U / L	Day 56	05/01/2011	31	.	10 - 71		
S241	303	Hepatitis B	Hepatitis B	2	Indirect Bilirubin	mg / dl	Day 56	05/01/2011	0.4	.	0.00 - 0.99		
S241	303	Hepatitis B	Hepatitis B	2	Total Bilirubin	mg / dl	Day 56	05/01/2011	0.52	.	0.00 - 0.99		
S241	303	Hepatitis B	Hepatitis B	2	Serum creatinine	mg / dl	Day 56	05/01/2011	1.1	.	0.7 - 1.2		
S241	303	Hepatitis B	Hepatitis B	2	Potassium	mEq / L	Day 56	05/01/2011	3.9	.	3.5 - 5.1		
S241	303	Hepatitis B	Hepatitis B	2	Random blood glucose	mg / dl	Day 56	05/01/2011	99	.	45-130		
S241	303	Hepatitis B	Hepatitis B	2	Sodium	mEq / L	Day 56	05/01/2011	138	.	136 - 145		
S241	303	Hepatitis B	Hepatitis B	3	ALT	U / L	Day 180	09/05/2011	29	.	0 - 41		
S241	303	Hepatitis B	Hepatitis B	3	AST	U / L	Day 180	09/05/2011	19	.	0 - 40		
S241	303	Hepatitis B	Hepatitis B	3	Alkaline Phosphatase	U / L	Day 180	09/05/2011	59	.	40 - 129		
S241	303	Hepatitis B	Hepatitis B	3	Direct Bilirubin	mg / dl	Day 180	09/05/2011	0.22	.	0.00 - 0.30		
S241	303	Hepatitis B	Hepatitis B	3	Gamma Glutamyl Transpeptidase	U / L	Day 180	09/05/2011	17	.	10 - 71		
S241	303	Hepatitis B	Hepatitis B	3	Indirect Bilirubin	mg / dl	Day 180	09/05/2011	0.4	.	0.00 - 0.99		
S241	303	Hepatitis B	Hepatitis B	3	Total Bilirubin	mg / dl	Day 180	09/05/2011	0.62	.	0.00 - 0.99		
S241	303	Hepatitis B	Hepatitis B	3	Serum creatinine	mg / dl	Day 180	09/05/2011	1	.	0.7 - 1.2		
S241	303	Hepatitis B	Hepatitis B	3	Potassium	mEq / L	Day 180	09/05/2011	3.8	.	3.5 - 5.1		
S241	303	Hepatitis B	Hepatitis B	3	Random blood glucose	mg / dl	Day 180	09/05/2011	82	.	45-130		
S241	303	Hepatitis B	Hepatitis B	3	Sodium	mEq / L	Day 180	09/05/2011	142	.	136 - 145		
S241	303	Hepatitis B	Hepatitis B	3	ALT	U / L	Day 208	06/06/2011	19	.	0 - 41		
S241	303	Hepatitis B	Hepatitis B	3	AST	U / L	Day 208	06/06/2011	16	.	0 - 40		
S241	303	Hepatitis B	Hepatitis B	3	Alkaline Phosphatase	U / L	Day 208	06/06/2011	60	.	40 - 129		
S241	303	Hepatitis B	Hepatitis B	3	Direct Bilirubin	mg / dl	Day 208	06/06/2011	0.18	.	0.00 - 0.30		

Subject No.	Randomisation Number	Study Group	Dosage	Dose	Serum Chemistry Parameters	Unit	Day	Date of sample Collection	Results	Out of Range	Reference Range	Flag	Clinical Significance*
S241	303	Hepatitis B	Hepatitis B	3	Gamma Glutamyl Transpeptidase	U / L	Day 208	06/06/2011	18	.	10 - 71		
S241	303	Hepatitis B	Hepatitis B	3	Indirect Bilirubin	mg / dl	Day 208	06/06/2011	0.51	.	0.00 - 0.99		
S241	303	Hepatitis B	Hepatitis B	3	Total Bilirubin	mg / dl	Day 208	06/06/2011	0.69	.	0.00 - 0.99		
S241	303	Hepatitis B	Hepatitis B	3	Serum creatinine	mg / dl	Day 208	06/06/2011	1.2	.	0.7 - 1.2		
S241	303	Hepatitis B	Hepatitis B	3	Potassium	mEq / L	Day 208	06/06/2011	3.8	.	3.5 - 5.1		
S241	303	Hepatitis B	Hepatitis B	3	Random blood glucose	mg / dl	Day 208	06/06/2011	109	.	45-130		
S241	303	Hepatitis B	Hepatitis B	3	Sodium	mEq / L	Day 208	06/06/2011	138	.	136 - 145		
S241	303	Hepatitis B	Hepatitis B	3	ALT	U / L	Day 365	09/11/2011	31	.	0 - 41		
S241	303	Hepatitis B	Hepatitis B	3	AST	U / L	Day 365	09/11/2011	19	.	0 - 40		
S241	303	Hepatitis B	Hepatitis B	3	Alkaline Phosphatase	U / L	Day 365	09/11/2011	61	.	40 - 129		
S241	303	Hepatitis B	Hepatitis B	3	Direct Bilirubin	mg / dl	Day 365	09/11/2011	0.11	.	0.00 - 0.30		
S241	303	Hepatitis B	Hepatitis B	3	Gamma Glutamyl Transpeptidase	U / L	Day 365	09/11/2011	21	.	10 - 71		
S241	303	Hepatitis B	Hepatitis B	3	Indirect Bilirubin	mg / dl	Day 365	09/11/2011	0.36	.	0.00 - 0.99		
S241	303	Hepatitis B	Hepatitis B	3	Total Bilirubin	mg / dl	Day 365	09/11/2011	0.47	.	0.00 - 0.99		
S241	303	Hepatitis B	Hepatitis B	3	Serum creatinine	mg / dl	Day 365	09/11/2011	1.1	.	0.7 - 1.2		
S241	303	Hepatitis B	Hepatitis B	3	Potassium	mEq / L	Day 365	09/11/2011	4	.	3.5 - 5.1		
S241	303	Hepatitis B	Hepatitis B	3	Random blood glucose	mg / dl	Day 365	09/11/2011	72	.	45-130		
S241	303	Hepatitis B	Hepatitis B	3	Sodium	mEq / L	Day 365	09/11/2011	141	.	136 - 145		
S250	311	JAIVAC-1	Dosage C		ALT	U / L	Day -14 to Day -1	03/11/2010	27	.	0 - 41		
S250	311	JAIVAC-1	Dosage C		AST	U / L	Day -14 to Day -1	03/11/2010	29	.	0 - 40		
S250	311	JAIVAC-1	Dosage C		Alkaline Phosphatase	U / L	Day -14 to Day -1	03/11/2010	92	.	40 - 129		
S250	311	JAIVAC-1	Dosage C		Direct Bilirubin	mg / dl	Day -14 to Day -1	03/11/2010	0.1	.	0.00 - 0.30		
S250	311	JAIVAC-1	Dosage C		Gamma Glutamyl Transpeptidase	U / L	Day -14 to Day -1	03/11/2010	30	.	10 - 71		
S250	311	JAIVAC-1	Dosage C		Indirect Bilirubin	mg / dl	Day -14 to Day -1	03/11/2010	0.38	.	0.00 - 0.99		
S250	311	JAIVAC-1	Dosage C		Total Bilirubin	mg / dl	Day -14 to Day -1	03/11/2010	0.48	.	0.00 - 0.99		
S250	311	JAIVAC-1	Dosage C		Serum creatinine	mg / dl	Day -14 to Day -1	03/11/2010	0.9	.	0.7 - 1.2		
S250	311	JAIVAC-1	Dosage C		Potassium	mEq / L	Day -14 to Day -1	03/11/2010	4.5	.	3.5 - 5.1		
S250	311	JAIVAC-1	Dosage C		Random blood glucose	mg / dl	Day -14 to Day -1	03/11/2010	83	.	45-130		
S250	311	JAIVAC-1	Dosage C		Sodium	mEq / L	Day -14 to Day -1	03/11/2010	139	.	136 - 145		

Subject No.	Randomisation Number	Study Group	Dosage	Dose	Serum Chemistry Parameters	Unit	Day	Date of sample Collection	Results	Out of Range	Reference Range	Flag	Clinical Significance*
S250	311	JAIVAC-1	Dosage C	2	ALT	U / L	Day 28	10/12/2010	27	.	0 - 41		
S250	311	JAIVAC-1	Dosage C	2	AST	U / L	Day 28	10/12/2010	29	.	0 - 40		
S250	311	JAIVAC-1	Dosage C	2	Alkaline Phosphatase	U / L	Day 28	10/12/2010	109	.	40 - 129		
S250	311	JAIVAC-1	Dosage C	2	Direct Bilirubin	mg / dl	Day 28	10/12/2010	0.13	.	0.00 - 0.30		
S250	311	JAIVAC-1	Dosage C	2	Gamma Glutamyl Transpeptidase	U / L	Day 28	10/12/2010	28	.	10 - 71		
S250	311	JAIVAC-1	Dosage C	2	Indirect Bilirubin	mg / dl	Day 28	10/12/2010	0.37	.	0.00 - 0.99		
S250	311	JAIVAC-1	Dosage C	2	Total Bilirubin	mg / dl	Day 28	10/12/2010	0.5	.	0.00 - 0.99		
S250	311	JAIVAC-1	Dosage C	2	Serum creatinine	mg / dl	Day 28	10/12/2010	1.1	.	0.7 - 1.2		
S250	311	JAIVAC-1	Dosage C	2	Potassium	mEq / L	Day 28	10/12/2010	3.9	.	3.5 - 5.1		
S250	311	JAIVAC-1	Dosage C	2	Random blood glucose	mg / dl	Day 28	10/12/2010	128	.	45-130		
S250	311	JAIVAC-1	Dosage C	2	Sodium	mEq / L	Day 28	10/12/2010	141	.	136 - 145		
S250	311	JAIVAC-1	Dosage C	2	ALT	U / L	Day 56	07/01/2011	35	.	0 - 41		
S250	311	JAIVAC-1	Dosage C	2	AST	U / L	Day 56	07/01/2011	35	.	0 - 40		
S250	311	JAIVAC-1	Dosage C	2	Alkaline Phosphatase	U / L	Day 56	07/01/2011	103	.	40 - 129		
S250	311	JAIVAC-1	Dosage C	2	Direct Bilirubin	mg / dl	Day 56	07/01/2011	0.15	.	0.00 - 0.30		
S250	311	JAIVAC-1	Dosage C	2	Gamma Glutamyl Transpeptidase	U / L	Day 56	07/01/2011	55	.	10 - 71		
S250	311	JAIVAC-1	Dosage C	2	Indirect Bilirubin	mg / dl	Day 56	07/01/2011	0.34	.	0.00 - 0.99		
S250	311	JAIVAC-1	Dosage C	2	Total Bilirubin	mg / dl	Day 56	07/01/2011	0.49	.	0.00 - 0.99		
S250	311	JAIVAC-1	Dosage C	2	Serum creatinine	mg / dl	Day 56	07/01/2011	1	.	0.7 - 1.2		
S250	311	JAIVAC-1	Dosage C	2	Potassium	mEq / L	Day 56	07/01/2011	5	.	3.5 - 5.1		
S250	311	JAIVAC-1	Dosage C	2	Random blood glucose	mg / dl	Day 56	07/01/2011	105	.	45-130		
S250	311	JAIVAC-1	Dosage C	2	Sodium	mEq / L	Day 56	07/01/2011	139	.	136 - 145		
S250	311	JAIVAC-1	Dosage C	3	ALT	U / L	Day 180	11/05/2011	36	.	0 - 41		
S250	311	JAIVAC-1	Dosage C	3	AST	U / L	Day 180	11/05/2011	35	.	0 - 40		
S250	311	JAIVAC-1	Dosage C	3	Alkaline Phosphatase	U / L	Day 180	11/05/2011	97	.	40 - 129		
S250	311	JAIVAC-1	Dosage C	3	Direct Bilirubin	mg / dl	Day 180	11/05/2011	0.1	.	0.00 - 0.30		
S250	311	JAIVAC-1	Dosage C	3	Gamma Glutamyl Transpeptidase	U / L	Day 180	11/05/2011	74	Yes	10 - 71	H	NCS
S250	311	JAIVAC-1	Dosage C	3	Indirect Bilirubin	mg / dl	Day 180	11/05/2011	0.19	.	0.00 - 0.99		
S250	311	JAIVAC-1	Dosage C	3	Total Bilirubin	mg / dl	Day 180	11/05/2011	0.29	.	0.00 - 0.99		

Subject No.	Randomisation Number	Study Group	Dosage	Dose	Serum Chemistry Parameters	Unit	Day	Date of sample Collection	Results	Out of Range	Reference Range	Flag	Clinical Significance*
S250	311	JAIVAC-1	Dosage C	3	Serum creatinine	mg / dl	Day 180	11/05/2011	1.1	.	0.7 - 1.2		
S250	311	JAIVAC-1	Dosage C	3	Potassium	mEq / L	Day 180	11/05/2011	4.6	.	3.5 - 5.1		
S250	311	JAIVAC-1	Dosage C	3	Random blood glucose	mg / dl	Day 180	11/05/2011	96	.	45-130		
S250	311	JAIVAC-1	Dosage C	3	Sodium	mEq / L	Day 180	11/05/2011	142	.	136 - 145		
S250	311	JAIVAC-1	Dosage C	3	ALT	U / L	Day 208	08/06/2011	30	.	0 - 41		
S250	311	JAIVAC-1	Dosage C	3	AST	U / L	Day 208	08/06/2011	27	.	0 - 40		
S250	311	JAIVAC-1	Dosage C	3	Alkaline Phosphatase	U / L	Day 208	08/06/2011	85	.	40 - 129		
S250	311	JAIVAC-1	Dosage C	3	Direct Bilirubin	mg / dl	Day 208	08/06/2011	<0.10	.	0.00 - 0.30		
S250	311	JAIVAC-1	Dosage C	3	Gamma Glutamyl Transpeptidase	U / L	Day 208	08/06/2011	33	.	10 - 71		
S250	311	JAIVAC-1	Dosage C	3	Indirect Bilirubin	mg / dl	Day 208	08/06/2011	NA	.	0.00 - 0.99		
S250	311	JAIVAC-1	Dosage C	3	Total Bilirubin	mg / dl	Day 208	08/06/2011	0.44	.	0.00 - 0.99		
S250	311	JAIVAC-1	Dosage C	3	Serum creatinine	mg / dl	Day 208	08/06/2011	0.9	.	0.7 - 1.2		
S250	311	JAIVAC-1	Dosage C	3	Potassium	mEq / L	Day 208	08/06/2011	4.2	.	3.5 - 5.1		
S250	311	JAIVAC-1	Dosage C	3	Random blood glucose	mg / dl	Day 208	08/06/2011	85	.	45-130		
S250	311	JAIVAC-1	Dosage C	3	Sodium	mEq / L	Day 208	08/06/2011	142	.	136 - 145		
S250	311	JAIVAC-1	Dosage C	3	ALT	U / L	Day 365	10/11/2011	31	.	0 - 41		
S250	311	JAIVAC-1	Dosage C	3	AST	U / L	Day 365	10/11/2011	28	.	0 - 40		
S250	311	JAIVAC-1	Dosage C	3	Alkaline Phosphatase	U / L	Day 365	10/11/2011	87	.	40 - 129		
S250	311	JAIVAC-1	Dosage C	3	Direct Bilirubin	mg / dl	Day 365	10/11/2011	<0.10	.	0.00 - 0.30		
S250	311	JAIVAC-1	Dosage C	3	Gamma Glutamyl Transpeptidase	U / L	Day 365	10/11/2011	26	.	10 - 71		
S250	311	JAIVAC-1	Dosage C	3	Indirect Bilirubin	mg / dl	Day 365	10/11/2011	NA	.	0.00 - 0.99		
S250	311	JAIVAC-1	Dosage C	3	Total Bilirubin	mg / dl	Day 365	10/11/2011	0.29	.	0.00 - 0.99		
S250	311	JAIVAC-1	Dosage C	3	Serum creatinine	mg / dl	Day 365	10/11/2011	1.1	.	0.7 - 1.2		
S250	311	JAIVAC-1	Dosage C	3	Potassium	mEq / L	Day 365	10/11/2011	4.5	.	3.5 - 5.1		
S250	311	JAIVAC-1	Dosage C	3	Random blood glucose	mg / dl	Day 365	10/11/2011	84	.	45-130		
S250	311	JAIVAC-1	Dosage C	3	Sodium	mEq / L	Day 365	10/11/2011	143	.	136 - 145		
S263	313	Hepatitis B	Hepatitis B		ALT	U / L	Day -14 to Day -1	09/11/2010	25	.	0 - 41		
S263	313	Hepatitis B	Hepatitis B		AST	U / L	Day -14 to Day -1	09/11/2010	20	.	0 - 40		
S263	313	Hepatitis B	Hepatitis B		Alkaline Phosphatase	U / L	Day -14 to Day -1	09/11/2010	129	.	40 - 129		

Subject No.	Randomisation Number	Study Group	Dosage	Dose	Serum Chemistry Parameters	Unit	Day	Date of sample Collection	Results	Out of Range	Reference Range	Flag	Clinical Significance*
S263	313	Hepatitis B	Hepatitis B		Direct Bilirubin	mg / dl	Day -14 to Day -1	09/11/2010	0.14	.	0.00 - 0.30		
S263	313	Hepatitis B	Hepatitis B		Gamma Glutamyl Transpeptidase	U / L	Day -14 to Day -1	09/11/2010	35	.	10 - 71		
S263	313	Hepatitis B	Hepatitis B		Indirect Bilirubin	mg / dl	Day -14 to Day -1	09/11/2010	0.31	.	0.00 - 0.99		
S263	313	Hepatitis B	Hepatitis B		Total Bilirubin	mg / dl	Day -14 to Day -1	09/11/2010	0.45	.	0.00 - 0.99		
S263	313	Hepatitis B	Hepatitis B		Serum creatinine	mg / dl	Day -14 to Day -1	09/11/2010	0.9	.	0.7 - 1.2		
S263	313	Hepatitis B	Hepatitis B		Potassium	mEq / L	Day -14 to Day -1	09/11/2010	4.4	.	3.5 - 5.1		
S263	313	Hepatitis B	Hepatitis B		Random blood glucose	mg / dl	Day -14 to Day -1	09/11/2010	79	.	45-130		
S263	313	Hepatitis B	Hepatitis B		Sodium	mEq / L	Day -14 to Day -1	09/11/2010	139	.	136 - 145		
S263	313	Hepatitis B	Hepatitis B	2	ALT	U / L	Day 28	10/12/2010	33	.	0 - 41		
S263	313	Hepatitis B	Hepatitis B	2	AST	U / L	Day 28	10/12/2010	NA	.	0 - 40		
S263	313	Hepatitis B	Hepatitis B	2	Alkaline Phosphatase	U / L	Day 28	10/12/2010	107	.	40 - 129		
S263	313	Hepatitis B	Hepatitis B	2	Direct Bilirubin	mg / dl	Day 28	10/12/2010	NA	.	0.00 - 0.30		
S263	313	Hepatitis B	Hepatitis B	2	Gamma Glutamyl Transpeptidase	U / L	Day 28	10/12/2010	21	.	10 - 71		
S263	313	Hepatitis B	Hepatitis B	2	Indirect Bilirubin	mg / dl	Day 28	10/12/2010	NA	.	0.00 - 0.99		
S263	313	Hepatitis B	Hepatitis B	2	Total Bilirubin	mg / dl	Day 28	10/12/2010	0.54	.	0.00 - 0.99		
S263	313	Hepatitis B	Hepatitis B	2	Serum creatinine	mg / dl	Day 28	10/12/2010	1	.	0.7 - 1.2		
S263	313	Hepatitis B	Hepatitis B	2	Potassium	mEq / L	Day 28	10/12/2010	4.5	.	3.5 - 5.1		
S263	313	Hepatitis B	Hepatitis B	2	Random blood glucose	mg / dl	Day 28	10/12/2010	86	.	45-130		
S263	313	Hepatitis B	Hepatitis B	2	Sodium	mEq / L	Day 28	10/12/2010	138	.	136 - 145		
S263	313	Hepatitis B	Hepatitis B	2	ALT	U / L	Day 56	07/01/2011	28	.	0 - 41		
S263	313	Hepatitis B	Hepatitis B	2	AST	U / L	Day 56	07/01/2011	26	.	0 - 40		
S263	313	Hepatitis B	Hepatitis B	2	Alkaline Phosphatase	U / L	Day 56	07/01/2011	113	.	40 - 129		
S263	313	Hepatitis B	Hepatitis B	2	Direct Bilirubin	mg / dl	Day 56	07/01/2011	0.18	.	0.00 - 0.30		
S263	313	Hepatitis B	Hepatitis B	2	Gamma Glutamyl Transpeptidase	U / L	Day 56	07/01/2011	30	.	10 - 71		
S263	313	Hepatitis B	Hepatitis B	2	Indirect Bilirubin	mg / dl	Day 56	07/01/2011	0.38	.	0.00 - 0.99		
S263	313	Hepatitis B	Hepatitis B	2	Total Bilirubin	mg / dl	Day 56	07/01/2011	0.56	.	0.00 - 0.99		
S263	313	Hepatitis B	Hepatitis B	2	Serum creatinine	mg / dl	Day 56	07/01/2011	1	.	0.7 - 1.2		
S263	313	Hepatitis B	Hepatitis B	2	Potassium	mEq / L	Day 56	07/01/2011	5.2	Yes	3.5 - 5.1	H	NCS
S263	313	Hepatitis B	Hepatitis B	2	Random blood glucose	mg / dl	Day 56	07/01/2011	99	.	45-130		

Subject No.	Randomisation Number	Study Group	Dosage	Dose	Serum Chemistry Parameters	Unit	Day	Date of sample Collection	Results	Out of Range	Reference Range	Flag	Clinical Significance*
S263	313	Hepatitis B	Hepatitis B	2	Sodium	mEq / L	Day 56	07/01/2011	139	.	136 - 145		
S263	313	Hepatitis B	Hepatitis B	3	ALT	U / L	Day 180	11/05/2011	21	.	0 - 41		
S263	313	Hepatitis B	Hepatitis B	3	AST	U / L	Day 180	11/05/2011	20	.	0 - 40		
S263	313	Hepatitis B	Hepatitis B	3	Alkaline Phosphatase	U / L	Day 180	11/05/2011	117	.	40 - 129		
S263	313	Hepatitis B	Hepatitis B	3	Direct Bilirubin	mg / dl	Day 180	11/05/2011	0.26	.	0.00 - 0.30		
S263	313	Hepatitis B	Hepatitis B	3	Gamma Glutamyl Transpeptidase	U / L	Day 180	11/05/2011	38	.	10 - 71		
S263	313	Hepatitis B	Hepatitis B	3	Indirect Bilirubin	mg / dl	Day 180	11/05/2011	0.48	.	0.00 - 0.99		
S263	313	Hepatitis B	Hepatitis B	3	Total Bilirubin	mg / dl	Day 180	11/05/2011	0.74	.	0.00 - 0.99		
S263	313	Hepatitis B	Hepatitis B	3	Serum creatinine	mg / dl	Day 180	11/05/2011	1.2	.	0.7 - 1.2		
S263	313	Hepatitis B	Hepatitis B	3	Potassium	mEq / L	Day 180	11/05/2011	4.8	.	3.5 - 5.1		
S263	313	Hepatitis B	Hepatitis B	3	Random blood glucose	mg / dl	Day 180	11/05/2011	81	.	45-130		
S263	313	Hepatitis B	Hepatitis B	3	Sodium	mEq / L	Day 180	11/05/2011	142	.	136 - 145		
S263	313	Hepatitis B	Hepatitis B	3	ALT	U / L	Day 208	08/06/2011	25	.	0 - 41		
S263	313	Hepatitis B	Hepatitis B	3	AST	U / L	Day 208	08/06/2011	23	.	0 - 40		
S263	313	Hepatitis B	Hepatitis B	3	Alkaline Phosphatase	U / L	Day 208	08/06/2011	102	.	40 - 129		
S263	313	Hepatitis B	Hepatitis B	3	Direct Bilirubin	mg / dl	Day 208	08/06/2011	0.12	.	0.00 - 0.30		
S263	313	Hepatitis B	Hepatitis B	3	Gamma Glutamyl Transpeptidase	U / L	Day 208	08/06/2011	28	.	10 - 71		
S263	313	Hepatitis B	Hepatitis B	3	Indirect Bilirubin	mg / dl	Day 208	08/06/2011	0.41	.	0.00 - 0.99		
S263	313	Hepatitis B	Hepatitis B	3	Total Bilirubin	mg / dl	Day 208	08/06/2011	0.53	.	0.00 - 0.99		
S263	313	Hepatitis B	Hepatitis B	3	Serum creatinine	mg / dl	Day 208	08/06/2011	1	.	0.7 - 1.2		
S263	313	Hepatitis B	Hepatitis B	3	Potassium	mEq / L	Day 208	08/06/2011	4.7	.	3.5 - 5.1		
S263	313	Hepatitis B	Hepatitis B	3	Random blood glucose	mg / dl	Day 208	08/06/2011	93	.	45-130		
S263	313	Hepatitis B	Hepatitis B	3	Sodium	mEq / L	Day 208	08/06/2011	140	.	136 - 145		
S263	313	Hepatitis B	Hepatitis B	3	ALT	U / L	Day 365	10/11/2011	32	.	0 - 41		
S263	313	Hepatitis B	Hepatitis B	3	AST	U / L	Day 365	10/11/2011	25	.	0 - 40		
S263	313	Hepatitis B	Hepatitis B	3	Alkaline Phosphatase	U / L	Day 365	10/11/2011	95	.	40 - 129		
S263	313	Hepatitis B	Hepatitis B	3	Direct Bilirubin	mg / dl	Day 365	10/11/2011	0.18	.	0.00 - 0.30		
S263	313	Hepatitis B	Hepatitis B	3	Gamma Glutamyl Transpeptidase	U / L	Day 365	10/11/2011	31	.	10 - 71		
S263	313	Hepatitis B	Hepatitis B	3	Indirect Bilirubin	mg / dl	Day 365	10/11/2011	0.27	.	0.00 - 0.99		

Subject No.	Randomisation Number	Study Group	Dosage	Dose	Serum Chemistry Parameters	Unit	Day	Date of sample Collection	Results	Out of Range	Reference Range	Flag	Clinical Significance*
S263	313	Hepatitis B	Hepatitis B	3	Total Bilirubin	mg / dl	Day 365	10/11/2011	0.45	.	0.00 - 0.99		
S263	313	Hepatitis B	Hepatitis B	3	Serum creatinine	mg / dl	Day 365	10/11/2011	1.1	.	0.7 - 1.2		
S263	313	Hepatitis B	Hepatitis B	3	Potassium	mEq / L	Day 365	10/11/2011	4.4	.	3.5 - 5.1		
S263	313	Hepatitis B	Hepatitis B	3	Random blood glucose	mg / dl	Day 365	10/11/2011	112	.	45-130		
S263	313	Hepatitis B	Hepatitis B	3	Sodium	mEq / L	Day 365	10/11/2011	140	.	136 - 145		
S264	306	JAIVAC-1	Dosage C		ALT	U / L	Day -14 to Day -1	03/11/2010	29	.	0 - 41		
S264	306	JAIVAC-1	Dosage C		AST	U / L	Day -14 to Day -1	03/11/2010	30	.	0 - 40		
S264	306	JAIVAC-1	Dosage C		Alkaline Phosphatase	U / L	Day -14 to Day -1	03/11/2010	94	.	40 - 129		
S264	306	JAIVAC-1	Dosage C		Direct Bilirubin	mg / dl	Day -14 to Day -1	03/11/2010	0.16	.	0.00 - 0.30		
S264	306	JAIVAC-1	Dosage C		Gamma Glutamyl Transpeptidase	U / L	Day -14 to Day -1	03/11/2010	39	.	10 - 71		
S264	306	JAIVAC-1	Dosage C		Indirect Bilirubin	mg / dl	Day -14 to Day -1	03/11/2010	0.53	.	0.00 - 0.99		
S264	306	JAIVAC-1	Dosage C		Total Bilirubin	mg / dl	Day -14 to Day -1	03/11/2010	0.69	.	0.00 - 0.99		
S264	306	JAIVAC-1	Dosage C		Serum creatinine	mg / dl	Day -14 to Day -1	03/11/2010	0.9	.	0.7 - 1.2		
S264	306	JAIVAC-1	Dosage C		Potassium	mEq / L	Day -14 to Day -1	03/11/2010	4	.	3.5 - 5.1		
S264	306	JAIVAC-1	Dosage C		Random blood glucose	mg / dl	Day -14 to Day -1	03/11/2010	112	.	45-130		
S264	306	JAIVAC-1	Dosage C		Sodium	mEq / L	Day -14 to Day -1	03/11/2010	137	.	136 - 145		
S264	306	JAIVAC-1	Dosage C	2	ALT	U / L	Day 28	09/12/2010	36	.	0 - 41		
S264	306	JAIVAC-1	Dosage C	2	AST	U / L	Day 28	09/12/2010	36	.	0 - 40		
S264	306	JAIVAC-1	Dosage C	2	Alkaline Phosphatase	U / L	Day 28	09/12/2010	86	.	40 - 129		
S264	306	JAIVAC-1	Dosage C	2	Direct Bilirubin	mg / dl	Day 28	09/12/2010	0.19	.	0.00 - 0.30		
S264	306	JAIVAC-1	Dosage C	2	Gamma Glutamyl Transpeptidase	U / L	Day 28	09/12/2010	51	.	10 - 71		
S264	306	JAIVAC-1	Dosage C	2	Indirect Bilirubin	mg / dl	Day 28	09/12/2010	0.48	.	0.00 - 0.99		
S264	306	JAIVAC-1	Dosage C	2	Total Bilirubin	mg / dl	Day 28	09/12/2010	0.67	.	0.00 - 0.99		
S264	306	JAIVAC-1	Dosage C	2	Serum creatinine	mg / dl	Day 28	09/12/2010	1.1	.	0.7 - 1.2		
S264	306	JAIVAC-1	Dosage C	2	Potassium	mEq / L	Day 28	09/12/2010	4.5	.	3.5 - 5.1		
S264	306	JAIVAC-1	Dosage C	2	Random blood glucose	mg / dl	Day 28	09/12/2010	121	.	45-130		
S264	306	JAIVAC-1	Dosage C	2	Sodium	mEq / L	Day 28	09/12/2010	138	.	136 - 145		
S264	306	JAIVAC-1	Dosage C	2	ALT	U / L	Day 56	06/01/2011	33	.	0 - 41		
S264	306	JAIVAC-1	Dosage C	2	AST	U / L	Day 56	06/01/2011	32	.	0 - 40		

Subject No.	Randomisation Number	Study Group	Dosage	Dose	Serum Chemistry Parameters	Unit	Day	Date of sample Collection	Results	Out of Range	Reference Range	Flag	Clinical Significance*
S264	306	JAIVAC-1	Dosage C	2	Alkaline Phosphatase	U / L	Day 56	06/01/2011	94	.	40 - 129		
S264	306	JAIVAC-1	Dosage C	2	Direct Bilirubin	mg / dl	Day 56	06/01/2011	0.23	.	0.00 - 0.30		
S264	306	JAIVAC-1	Dosage C	2	Gamma Glutamyl Transpeptidase	U / L	Day 56	06/01/2011	55	.	10 - 71		
S264	306	JAIVAC-1	Dosage C	2	Indirect Bilirubin	mg / dl	Day 56	06/01/2011	0.53	.	0.00 - 0.99		
S264	306	JAIVAC-1	Dosage C	2	Total Bilirubin	mg / dl	Day 56	06/01/2011	0.76	.	0.00 - 0.99		
S264	306	JAIVAC-1	Dosage C	2	Serum creatinine	mg / dl	Day 56	06/01/2011	1.1	.	0.7 - 1.2		
S264	306	JAIVAC-1	Dosage C	2	Potassium	mEq / L	Day 56	06/01/2011	4.7	.	3.5 - 5.1		
S264	306	JAIVAC-1	Dosage C	2	Random blood glucose	mg / dl	Day 56	06/01/2011	95	.	45-130		
S264	306	JAIVAC-1	Dosage C	2	Sodium	mEq / L	Day 56	06/01/2011	138	.	136 - 145		
S264	306	JAIVAC-1	Dosage C	3	ALT	U / L	Day 180	10/05/2011	30	.	0 - 41		
S264	306	JAIVAC-1	Dosage C	3	AST	U / L	Day 180	10/05/2011	26	.	0 - 40		
S264	306	JAIVAC-1	Dosage C	3	Alkaline Phosphatase	U / L	Day 180	10/05/2011	78	.	40 - 129		
S264	306	JAIVAC-1	Dosage C	3	Direct Bilirubin	mg / dl	Day 180	10/05/2011	0.16	.	0.00 - 0.30		
S264	306	JAIVAC-1	Dosage C	3	Gamma Glutamyl Transpeptidase	U / L	Day 180	10/05/2011	35	.	10 - 71		
S264	306	JAIVAC-1	Dosage C	3	Indirect Bilirubin	mg / dl	Day 180	10/05/2011	0.4	.	0.00 - 0.99		
S264	306	JAIVAC-1	Dosage C	3	Total Bilirubin	mg / dl	Day 180	10/05/2011	0.56	.	0.00 - 0.99		
S264	306	JAIVAC-1	Dosage C	3	Serum creatinine	mg / dl	Day 180	10/05/2011	1	.	0.7 - 1.2		
S264	306	JAIVAC-1	Dosage C	3	Potassium	mEq / L	Day 180	10/05/2011	4.6	.	3.5 - 5.1		
S264	306	JAIVAC-1	Dosage C	3	Random blood glucose	mg / dl	Day 180	10/05/2011	108	.	45-130		
S264	306	JAIVAC-1	Dosage C	3	Sodium	mEq / L	Day 180	10/05/2011	137	.	136 - 145		
S264	306	JAIVAC-1	Dosage C	3	ALT	U / L	Day 208	07/06/2011	66	Yes	0 - 41	H	CS
S264	306	JAIVAC-1	Dosage C	3	AST	U / L	Day 208	07/06/2011	59	Yes	0 - 40	H	CS
S264	306	JAIVAC-1	Dosage C	3	Alkaline Phosphatase	U / L	Day 208	07/06/2011	93	.	40 - 129		
S264	306	JAIVAC-1	Dosage C	3	Direct Bilirubin	mg / dl	Day 208	07/06/2011	0.21	.	0.00 - 0.30		
S264	306	JAIVAC-1	Dosage C	3	Gamma Glutamyl Transpeptidase	U / L	Day 208	07/06/2011	76	Yes	10 - 71	H	CS
S264	306	JAIVAC-1	Dosage C	3	Indirect Bilirubin	mg / dl	Day 208	07/06/2011	0.4	.	0.00 - 0.99		
S264	306	JAIVAC-1	Dosage C	3	Total Bilirubin	mg / dl	Day 208	07/06/2011	0.61	.	0.00 - 0.99		
S264	306	JAIVAC-1	Dosage C	3	Serum creatinine	mg / dl	Day 208	07/06/2011	1.1	.	0.7 - 1.2		
S264	306	JAIVAC-1	Dosage C	3	Potassium	mEq / L	Day 208	07/06/2011	4.1	.	3.5 - 5.1		

Subject No.	Randomisation Number	Study Group	Dosage	Dose	Serum Chemistry Parameters	Unit	Day	Date of sample Collection	Results	Out of Range	Reference Range	Flag	Clinical Significance*
S264	306	JAIVAC-1	Dosage C	3	Random blood glucose	mg / dl	Day 208	07/06/2011	145	Yes	45-130	H	CS
S264	306	JAIVAC-1	Dosage C	3	Sodium	mEq / L	Day 208	07/06/2011	140	.	136 - 145		
S264	306	JAIVAC-1	Dosage C	3	ALT	U / L	Day 365	09/11/2011	68	Yes	0 - 41	H	CS
S264	306	JAIVAC-1	Dosage C	3	AST	U / L	Day 365	09/11/2011	46	Yes	0 - 40	H	CS
S264	306	JAIVAC-1	Dosage C	3	Alkaline Phosphatase	U / L	Day 365	09/11/2011	84	.	40 - 129		
S264	306	JAIVAC-1	Dosage C	3	Direct Bilirubin	mg / dl	Day 365	09/11/2011	0.19	.	0.00 - 0.30		
S264	306	JAIVAC-1	Dosage C	3	Gamma Glutamyl Transpeptidase	U / L	Day 365	09/11/2011	64	.	10 - 71		
S264	306	JAIVAC-1	Dosage C	3	Indirect Bilirubin	mg / dl	Day 365	09/11/2011	0.53	.	0.00 - 0.99		
S264	306	JAIVAC-1	Dosage C	3	Total Bilirubin	mg / dl	Day 365	09/11/2011	0.72	.	0.00 - 0.99		
S264	306	JAIVAC-1	Dosage C	3	Serum creatinine	mg / dl	Day 365	09/11/2011	1.2	.	0.7 - 1.2		
S264	306	JAIVAC-1	Dosage C	3	Potassium	mEq / L	Day 365	09/11/2011	4.4	.	3.5 - 5.1		
S264	306	JAIVAC-1	Dosage C	3	Random blood glucose	mg / dl	Day 365	09/11/2011	156	Yes	45-130	H	CS
S264	306	JAIVAC-1	Dosage C	3	Sodium	mEq / L	Day 365	09/11/2011	138	.	136 - 145		
S266	308	JAIVAC-1	Dosage C		ALT	U / L	Day -14 to Day -1	03/11/2010	32	.	0 - 41		
S266	308	JAIVAC-1	Dosage C		AST	U / L	Day -14 to Day -1	03/11/2010	24	.	0 - 40		
S266	308	JAIVAC-1	Dosage C		Alkaline Phosphatase	U / L	Day -14 to Day -1	03/11/2010	57	.	40 - 129		
S266	308	JAIVAC-1	Dosage C		Direct Bilirubin	mg / dl	Day -14 to Day -1	03/11/2010	0.17	.	0.00 - 0.30		
S266	308	JAIVAC-1	Dosage C		Gamma Glutamyl Transpeptidase	U / L	Day -14 to Day -1	03/11/2010	56	.	10 - 71		
S266	308	JAIVAC-1	Dosage C		Indirect Bilirubin	mg / dl	Day -14 to Day -1	03/11/2010	0.54	.	0.00 - 0.99		
S266	308	JAIVAC-1	Dosage C		Total Bilirubin	mg / dl	Day -14 to Day -1	03/11/2010	0.71	.	0.00 - 0.99		
S266	308	JAIVAC-1	Dosage C		Serum creatinine	mg / dl	Day -14 to Day -1	03/11/2010	0.8	.	0.7 - 1.2		
S266	308	JAIVAC-1	Dosage C		Potassium	mEq / L	Day -14 to Day -1	03/11/2010	4.5	.	3.5 - 5.1		
S266	308	JAIVAC-1	Dosage C		Random blood glucose	mg / dl	Day -14 to Day -1	03/11/2010	99	.	45-130		
S266	308	JAIVAC-1	Dosage C		Sodium	mEq / L	Day -14 to Day -1	03/11/2010	138	.	136 - 145		
S266	308	JAIVAC-1	Dosage C	2	ALT	U / L	Day 28	09/12/2010	38	.	0 - 41		
S266	308	JAIVAC-1	Dosage C	2	AST	U / L	Day 28	09/12/2010	27	.	0 - 40		
S266	308	JAIVAC-1	Dosage C	2	Alkaline Phosphatase	U / L	Day 28	09/12/2010	62	.	40 - 129		
S266	308	JAIVAC-1	Dosage C	2	Direct Bilirubin	mg / dl	Day 28	09/12/2010	0.17	.	0.00 - 0.30		
S266	308	JAIVAC-1	Dosage C	2	Gamma Glutamyl Transpeptidase	U / L	Day 28	09/12/2010	64	.	10 - 71		

Subject No.	Randomisation Number	Study Group	Dosage	Dose	Serum Chemistry Parameters	Unit	Day	Date of sample Collection	Results	Out of Range	Reference Range	Flag	Clinical Significance*
S266	308	JAIVAC-1	Dosage C	2	Indirect Bilirubin	mg / dl	Day 28	09/12/2010	0.42	.	0.00 - 0.99		
S266	308	JAIVAC-1	Dosage C	2	Total Bilirubin	mg / dl	Day 28	09/12/2010	0.59	.	0.00 - 0.99		
S266	308	JAIVAC-1	Dosage C	2	Serum creatinine	mg / dl	Day 28	09/12/2010	1	.	0.7 - 1.2		
S266	308	JAIVAC-1	Dosage C	2	Potassium	mEq / L	Day 28	09/12/2010	5.1	.	3.5 - 5.1		
S266	308	JAIVAC-1	Dosage C	2	Random blood glucose	mg / dl	Day 28	09/12/2010	123	.	45-130		
S266	308	JAIVAC-1	Dosage C	2	Sodium	mEq / L	Day 28	09/12/2010	137	.	136 - 145		
S266	308	JAIVAC-1	Dosage C	2	ALT	U / L	Day 56	06/01/2011	33	.	0 - 41		
S266	308	JAIVAC-1	Dosage C	2	AST	U / L	Day 56	06/01/2011	26	.	0 - 40		
S266	308	JAIVAC-1	Dosage C	2	Alkaline Phosphatase	U / L	Day 56	06/01/2011	59	.	40 - 129		
S266	308	JAIVAC-1	Dosage C	2	Direct Bilirubin	mg / dl	Day 56	06/01/2011	0.17	.	0.00 - 0.30		
S266	308	JAIVAC-1	Dosage C	2	Gamma Glutamyl Transpeptidase	U / L	Day 56	06/01/2011	62	.	10 - 71		
S266	308	JAIVAC-1	Dosage C	2	Indirect Bilirubin	mg / dl	Day 56	06/01/2011	0.55	.	0.00 - 0.99		
S266	308	JAIVAC-1	Dosage C	2	Total Bilirubin	mg / dl	Day 56	06/01/2011	0.72	.	0.00 - 0.99		
S266	308	JAIVAC-1	Dosage C	2	Serum creatinine	mg / dl	Day 56	06/01/2011	0.9	.	0.7 - 1.2		
S266	308	JAIVAC-1	Dosage C	2	Potassium	mEq / L	Day 56	06/01/2011	5.6	Yes	3.5 - 5.1	H	NCS
S266	308	JAIVAC-1	Dosage C	2	Random blood glucose	mg / dl	Day 56	06/01/2011	110	.	45-130		
S266	308	JAIVAC-1	Dosage C	2	Sodium	mEq / L	Day 56	06/01/2011	139	.	136 - 145		
S266	308	JAIVAC-1	Dosage C	3	ALT	U / L	Day 180	10/05/2011	26	.	0 - 41		
S266	308	JAIVAC-1	Dosage C	3	AST	U / L	Day 180	10/05/2011	20	.	0 - 40		
S266	308	JAIVAC-1	Dosage C	3	Alkaline Phosphatase	U / L	Day 180	10/05/2011	52	.	40 - 129		
S266	308	JAIVAC-1	Dosage C	3	Direct Bilirubin	mg / dl	Day 180	10/05/2011	0.13	.	0.00 - 0.30		
S266	308	JAIVAC-1	Dosage C	3	Gamma Glutamyl Transpeptidase	U / L	Day 180	10/05/2011	49	.	10 - 71		
S266	308	JAIVAC-1	Dosage C	3	Indirect Bilirubin	mg / dl	Day 180	10/05/2011	0.36	.	0.00 - 0.99		
S266	308	JAIVAC-1	Dosage C	3	Total Bilirubin	mg / dl	Day 180	10/05/2011	0.49	.	0.00 - 0.99		
S266	308	JAIVAC-1	Dosage C	3	Serum creatinine	mg / dl	Day 180	10/05/2011	0.9	.	0.7 - 1.2		
S266	308	JAIVAC-1	Dosage C	3	Potassium	mEq / L	Day 180	10/05/2011	4.6	.	3.5 - 5.1		
S266	308	JAIVAC-1	Dosage C	3	Random blood glucose	mg / dl	Day 180	10/05/2011	117	.	45-130		
S266	308	JAIVAC-1	Dosage C	3	Sodium	mEq / L	Day 180	10/05/2011	140	.	136 - 145		
S266	308	JAIVAC-1	Dosage C	3	ALT	U / L	Day 208	07/06/2011	23	.	0 - 41		

Subject No.	Randomisation Number	Study Group	Dosage	Dose	Serum Chemistry Parameters	Unit	Day	Date of sample Collection	Results	Out of Range	Reference Range	Flag	Clinical Significance*
S266	308	JAIVAC-1	Dosage C	3	AST	U / L	Day 208	07/06/2011	21	.	0 - 40		
S266	308	JAIVAC-1	Dosage C	3	Alkaline Phosphatase	U / L	Day 208	07/06/2011	56	.	40 - 129		
S266	308	JAIVAC-1	Dosage C	3	Direct Bilirubin	mg / dl	Day 208	07/06/2011	0.24	.	0.00 - 0.30		
S266	308	JAIVAC-1	Dosage C	3	Gamma Glutamyl Transpeptidase	U / L	Day 208	07/06/2011	67	.	10 - 71		
S266	308	JAIVAC-1	Dosage C	3	Indirect Bilirubin	mg / dl	Day 208	07/06/2011	0.53	.	0.00 - 0.99		
S266	308	JAIVAC-1	Dosage C	3	Total Bilirubin	mg / dl	Day 208	07/06/2011	0.77	.	0.00 - 0.99		
S266	308	JAIVAC-1	Dosage C	3	Serum creatinine	mg / dl	Day 208	07/06/2011	0.9	.	0.7 - 1.2		
S266	308	JAIVAC-1	Dosage C	3	Potassium	mEq / L	Day 208	07/06/2011	5.1	.	3.5 - 5.1		
S266	308	JAIVAC-1	Dosage C	3	Random blood glucose	mg / dl	Day 208	07/06/2011	101	.	45-130		
S266	308	JAIVAC-1	Dosage C	3	Sodium	mEq / L	Day 208	07/06/2011	136	.	136 - 145		
S266	308	JAIVAC-1	Dosage C	3	ALT	U / L	Day 365	09/11/2011	26	.	0 - 41		
S266	308	JAIVAC-1	Dosage C	3	AST	U / L	Day 365	09/11/2011	25	.	0 - 40		
S266	308	JAIVAC-1	Dosage C	3	Alkaline Phosphatase	U / L	Day 365	09/11/2011	53	.	40 - 129		
S266	308	JAIVAC-1	Dosage C	3	Direct Bilirubin	mg / dl	Day 365	09/11/2011	0.18	.	0.00 - 0.30		
S266	308	JAIVAC-1	Dosage C	3	Gamma Glutamyl Transpeptidase	U / L	Day 365	09/11/2011	62	.	10 - 71		
S266	308	JAIVAC-1	Dosage C	3	Indirect Bilirubin	mg / dl	Day 365	09/11/2011	0.43	.	0.00 - 0.99		
S266	308	JAIVAC-1	Dosage C	3	Total Bilirubin	mg / dl	Day 365	09/11/2011	0.61	.	0.00 - 0.99		
S266	308	JAIVAC-1	Dosage C	3	Serum creatinine	mg / dl	Day 365	09/11/2011	1	.	0.7 - 1.2		
S266	308	JAIVAC-1	Dosage C	3	Potassium	mEq / L	Day 365	09/11/2011	4.8	.	3.5 - 5.1		
S266	308	JAIVAC-1	Dosage C	3	Random blood glucose	mg / dl	Day 365	09/11/2011	100	.	45-130		
S266	308	JAIVAC-1	Dosage C	3	Sodium	mEq / L	Day 365	09/11/2011	139	.	136 - 145		
S267	309	Hepatitis B	Hepatitis B		ALT	U / L	Day -14 to Day -1	02/11/2010	23	.	0 - 41		
S267	309	Hepatitis B	Hepatitis B		AST	U / L	Day -14 to Day -1	02/11/2010	32	.	0 - 40		
S267	309	Hepatitis B	Hepatitis B		Alkaline Phosphatase	U / L	Day -14 to Day -1	02/11/2010	61	.	40 - 129		
S267	309	Hepatitis B	Hepatitis B		Direct Bilirubin	mg / dl	Day -14 to Day -1	02/11/2010	0.21	.	0.00 - 0.30		
S267	309	Hepatitis B	Hepatitis B		Gamma Glutamyl Transpeptidase	U / L	Day -14 to Day -1	02/11/2010	15	.	10 - 71		
S267	309	Hepatitis B	Hepatitis B		Indirect Bilirubin	mg / dl	Day -14 to Day -1	02/11/2010	0.61	.	0.00 - 0.99		
S267	309	Hepatitis B	Hepatitis B		Total Bilirubin	mg / dl	Day -14 to Day -1	02/11/2010	0.82	.	0.00 - 0.99		
S267	309	Hepatitis B	Hepatitis B		Serum creatinine	mg / dl	Day -14 to Day -1	02/11/2010	1.2	.	0.7 - 1.2		

Subject No.	Randomisation Number	Study Group	Dosage	Dose	Serum Chemistry Parameters	Unit	Day	Date of sample Collection	Results	Out of Range	Reference Range	Flag	Clinical Significance*
S267	309	Hepatitis B	Hepatitis B		Potassium	mEq / L	Day -14 to Day -1	02/11/2010	4.8	.	3.5 - 5.1		
S267	309	Hepatitis B	Hepatitis B		Random blood glucose	mg / dl	Day -14 to Day -1	02/11/2010	73	.	45-130		
S267	309	Hepatitis B	Hepatitis B		Sodium	mEq / L	Day -14 to Day -1	02/11/2010	140	.	136 - 145		
S267	309	Hepatitis B	Hepatitis B	2	ALT	U / L	Day 28	09/12/2010	24	.	0 - 41		
S267	309	Hepatitis B	Hepatitis B	2	AST	U / L	Day 28	09/12/2010	31	.	0 - 40		
S267	309	Hepatitis B	Hepatitis B	2	Alkaline Phosphatase	U / L	Day 28	09/12/2010	58	.	40 - 129		
S267	309	Hepatitis B	Hepatitis B	2	Direct Bilirubin	mg / dl	Day 28	09/12/2010	0.19	.	0.00 - 0.30		
S267	309	Hepatitis B	Hepatitis B	2	Gamma Glutamyl Transpeptidase	U / L	Day 28	09/12/2010	19	.	10 - 71		
S267	309	Hepatitis B	Hepatitis B	2	Indirect Bilirubin	mg / dl	Day 28	09/12/2010	0.64	.	0.00 - 0.99		
S267	309	Hepatitis B	Hepatitis B	2	Total Bilirubin	mg / dl	Day 28	09/12/2010	0.83	.	0.00 - 0.99		
S267	309	Hepatitis B	Hepatitis B	2	Serum creatinine	mg / dl	Day 28	09/12/2010	1.3	Yes	0.7 - 1.2	H	NCS
S267	309	Hepatitis B	Hepatitis B	2	Potassium	mEq / L	Day 28	09/12/2010	4.4	.	3.5 - 5.1		
S267	309	Hepatitis B	Hepatitis B	2	Random blood glucose	mg / dl	Day 28	09/12/2010	85	.	45-130		
S267	309	Hepatitis B	Hepatitis B	2	Sodium	mEq / L	Day 28	09/12/2010	136	.	136 - 145		
S267	309	Hepatitis B	Hepatitis B	2	ALT	U / L	Day 56	06/01/2011	21	.	0 - 41		
S267	309	Hepatitis B	Hepatitis B	2	AST	U / L	Day 56	06/01/2011	32	.	0 - 40		
S267	309	Hepatitis B	Hepatitis B	2	Alkaline Phosphatase	U / L	Day 56	06/01/2011	67	.	40 - 129		
S267	309	Hepatitis B	Hepatitis B	2	Direct Bilirubin	mg / dl	Day 56	06/01/2011	0.19	.	0.00 - 0.30		
S267	309	Hepatitis B	Hepatitis B	2	Gamma Glutamyl Transpeptidase	U / L	Day 56	06/01/2011	20	.	10 - 71		
S267	309	Hepatitis B	Hepatitis B	2	Indirect Bilirubin	mg / dl	Day 56	06/01/2011	0.49	.	0.00 - 0.99		
S267	309	Hepatitis B	Hepatitis B	2	Total Bilirubin	mg / dl	Day 56	06/01/2011	0.68	.	0.00 - 0.99		
S267	309	Hepatitis B	Hepatitis B	2	Serum creatinine	mg / dl	Day 56	06/01/2011	1.4	Yes	0.7 - 1.2	H	NCS
S267	309	Hepatitis B	Hepatitis B	2	Potassium	mEq / L	Day 56	06/01/2011	4.2	.	3.5 - 5.1		
S267	309	Hepatitis B	Hepatitis B	2	Random blood glucose	mg / dl	Day 56	06/01/2011	87	.	45-130		
S267	309	Hepatitis B	Hepatitis B	2	Sodium	mEq / L	Day 56	06/01/2011	138	.	136 - 145		
S267	309	Hepatitis B	Hepatitis B	3	ALT	U / L	Day 180	10/05/2011	17	.	0 - 41		
S267	309	Hepatitis B	Hepatitis B	3	AST	U / L	Day 180	10/05/2011	28	.	0 - 40		
S267	309	Hepatitis B	Hepatitis B	3	Alkaline Phosphatase	U / L	Day 180	10/05/2011	43	.	40 - 129		
S267	309	Hepatitis B	Hepatitis B	3	Direct Bilirubin	mg / dl	Day 180	10/05/2011	0.17	.	0.00 - 0.30		

Subject No.	Randomisation Number	Study Group	Dosage	Dose	Serum Chemistry Parameters	Unit	Day	Date of sample Collection	Results	Out of Range	Reference Range	Flag	Clinical Significance*
S267	309	Hepatitis B	Hepatitis B	3	Gamma Glutamyl Transpeptidase	U / L	Day 180	10/05/2011	16	.	10 - 71		
S267	309	Hepatitis B	Hepatitis B	3	Indirect Bilirubin	mg / dl	Day 180	10/05/2011	0.53	.	0.00 - 0.99		
S267	309	Hepatitis B	Hepatitis B	3	Total Bilirubin	mg / dl	Day 180	10/05/2011	0.7	.	0.00 - 0.99		
S267	309	Hepatitis B	Hepatitis B	3	Serum creatinine	mg / dl	Day 180	10/05/2011	1.4	Yes	0.7 - 1.2	H	NCS
S267	309	Hepatitis B	Hepatitis B	3	Potassium	mEq / L	Day 180	10/05/2011	4.3	.	3.5 - 5.1		
S267	309	Hepatitis B	Hepatitis B	3	Random blood glucose	mg / dl	Day 180	10/05/2011	53	.	45-130		
S267	309	Hepatitis B	Hepatitis B	3	Sodium	mEq / L	Day 180	10/05/2011	141	.	136 - 145		
S267	309	Hepatitis B	Hepatitis B	3	ALT	U / L	Day 208	07/06/2011	26	.	0 - 41		
S267	309	Hepatitis B	Hepatitis B	3	AST	U / L	Day 208	07/06/2011	37	.	0 - 40		
S267	309	Hepatitis B	Hepatitis B	3	Alkaline Phosphatase	U / L	Day 208	07/06/2011	51	.	40 - 129		
S267	309	Hepatitis B	Hepatitis B	3	Direct Bilirubin	mg / dl	Day 208	07/06/2011	0.17	.	0.00 - 0.30		
S267	309	Hepatitis B	Hepatitis B	3	Gamma Glutamyl Transpeptidase	U / L	Day 208	07/06/2011	19	.	10 - 71		
S267	309	Hepatitis B	Hepatitis B	3	Indirect Bilirubin	mg / dl	Day 208	07/06/2011	0.4	.	0.00 - 0.99		
S267	309	Hepatitis B	Hepatitis B	3	Total Bilirubin	mg / dl	Day 208	07/06/2011	0.57	.	0.00 - 0.99		
S267	309	Hepatitis B	Hepatitis B	3	Serum creatinine	mg / dl	Day 208	07/06/2011	1.5	Yes	0.7 - 1.2	H	CS
S267	309	Hepatitis B	Hepatitis B	3	Potassium	mEq / L	Day 208	07/06/2011	4	.	3.5 - 5.1		
S267	309	Hepatitis B	Hepatitis B	3	Random blood glucose	mg / dl	Day 208	07/06/2011	78	.	45-130		
S267	309	Hepatitis B	Hepatitis B	3	Sodium	mEq / L	Day 208	07/06/2011	139	.	136 - 145		
S267	309	Hepatitis B	Hepatitis B	3	ALT	U / L	Day 365	09/11/2011	27	.	0 - 41		
S267	309	Hepatitis B	Hepatitis B	3	AST	U / L	Day 365	09/11/2011	38	.	0 - 40		
S267	309	Hepatitis B	Hepatitis B	3	Alkaline Phosphatase	U / L	Day 365	09/11/2011	56	.	40 - 129		
S267	309	Hepatitis B	Hepatitis B	3	Direct Bilirubin	mg / dl	Day 365	09/11/2011	0.17	.	0.00 - 0.30		
S267	309	Hepatitis B	Hepatitis B	3	Gamma Glutamyl Transpeptidase	U / L	Day 365	09/11/2011	26	.	10 - 71		
S267	309	Hepatitis B	Hepatitis B	3	Indirect Bilirubin	mg / dl	Day 365	09/11/2011	0.51	.	0.00 - 0.99		
S267	309	Hepatitis B	Hepatitis B	3	Total Bilirubin	mg / dl	Day 365	09/11/2011	0.68	.	0.00 - 0.99		
S267	309	Hepatitis B	Hepatitis B	3	Serum creatinine	mg / dl	Day 365	09/11/2011	1.4	Yes	0.7 - 1.2	H	CS
S267	309	Hepatitis B	Hepatitis B	3	Potassium	mEq / L	Day 365	09/11/2011	4.2	.	3.5 - 5.1		
S267	309	Hepatitis B	Hepatitis B	3	Random blood glucose	mg / dl	Day 365	09/11/2011	78	.	45-130		
S267	309	Hepatitis B	Hepatitis B	3	Sodium	mEq / L	Day 365	09/11/2011	140	.	136 - 145		

Subject No.	Randomisation Number	Study Group	Dosage	Dose	Serum Chemistry Parameters	Unit	Day	Date of sample Collection	Results	Out of Range	Reference Range	Flag	Clinical Significance*
S275	302	Hepatitis B	Hepatitis B		ALT	U / L	Day -14 to Day -1	02/11/2010	33	.	0 - 41		
S275	302	Hepatitis B	Hepatitis B		AST	U / L	Day -14 to Day -1	02/11/2010	28	.	0 - 40		
S275	302	Hepatitis B	Hepatitis B		Alkaline Phosphatase	U / L	Day -14 to Day -1	02/11/2010	63	.	40 - 129		
S275	302	Hepatitis B	Hepatitis B		Direct Bilirubin	mg / dl	Day -14 to Day -1	02/11/2010	0.14	.	0.00 - 0.30		
S275	302	Hepatitis B	Hepatitis B		Gamma Glutamyl Transpeptidase	U / L	Day -14 to Day -1	02/11/2010	48	.	10 - 71		
S275	302	Hepatitis B	Hepatitis B		Indirect Bilirubin	mg / dl	Day -14 to Day -1	02/11/2010	0.43	.	0.00 - 0.99		
S275	302	Hepatitis B	Hepatitis B		Total Bilirubin	mg / dl	Day -14 to Day -1	02/11/2010	0.57	.	0.00 - 0.99		
S275	302	Hepatitis B	Hepatitis B		Serum creatinine	mg / dl	Day -14 to Day -1	02/11/2010	1.1	.	0.7 - 1.2		
S275	302	Hepatitis B	Hepatitis B		Potassium	mEq / L	Day -14 to Day -1	02/11/2010	4.1	.	3.5 - 5.1		
S275	302	Hepatitis B	Hepatitis B		Random blood glucose	mg / dl	Day -14 to Day -1	02/11/2010	78	.	45-130		
S275	302	Hepatitis B	Hepatitis B		Sodium	mEq / L	Day -14 to Day -1	02/11/2010	140	.	136 - 145		
S275	302	Hepatitis B	Hepatitis B	2	ALT	U / L	Day 28	08/12/2010	24	.	0 - 41		
S275	302	Hepatitis B	Hepatitis B	2	AST	U / L	Day 28	08/12/2010	23	.	0 - 40		
S275	302	Hepatitis B	Hepatitis B	2	Alkaline Phosphatase	U / L	Day 28	08/12/2010	73	.	40 - 129		
S275	302	Hepatitis B	Hepatitis B	2	Direct Bilirubin	mg / dl	Day 28	08/12/2010	0.15	.	0.00 - 0.30		
S275	302	Hepatitis B	Hepatitis B	2	Gamma Glutamyl Transpeptidase	U / L	Day 28	08/12/2010	31	.	10 - 71		
S275	302	Hepatitis B	Hepatitis B	2	Indirect Bilirubin	mg / dl	Day 28	08/12/2010	0.4	.	0.00 - 0.99		
S275	302	Hepatitis B	Hepatitis B	2	Total Bilirubin	mg / dl	Day 28	08/12/2010	0.55	.	0.00 - 0.99		
S275	302	Hepatitis B	Hepatitis B	2	Serum creatinine	mg / dl	Day 28	08/12/2010	1.3	Yes	0.7 - 1.2	H	NCS
S275	302	Hepatitis B	Hepatitis B	2	Potassium	mEq / L	Day 28	08/12/2010	4.1	.	3.5 - 5.1		
S275	302	Hepatitis B	Hepatitis B	2	Random blood glucose	mg / dl	Day 28	08/12/2010	123	.	45-130		
S275	302	Hepatitis B	Hepatitis B	2	Sodium	mEq / L	Day 28	08/12/2010	139	.	136 - 145		
S275	302	Hepatitis B	Hepatitis B	2	ALT	U / L	Day 56	05/01/2011	35	.	0 - 41		
S275	302	Hepatitis B	Hepatitis B	2	AST	U / L	Day 56	05/01/2011	29	.	0 - 40		
S275	302	Hepatitis B	Hepatitis B	2	Alkaline Phosphatase	U / L	Day 56	05/01/2011	61	.	40 - 129		
S275	302	Hepatitis B	Hepatitis B	2	Direct Bilirubin	mg / dl	Day 56	05/01/2011	0.15	.	0.00 - 0.30		
S275	302	Hepatitis B	Hepatitis B	2	Gamma Glutamyl Transpeptidase	U / L	Day 56	05/01/2011	32	.	10 - 71		
S275	302	Hepatitis B	Hepatitis B	2	Indirect Bilirubin	mg / dl	Day 56	05/01/2011	0.28	.	0.00 - 0.99		
S275	302	Hepatitis B	Hepatitis B	2	Total Bilirubin	mg / dl	Day 56	05/01/2011	0.43	.	0.00 - 0.99		

Subject No.	Randomisation Number	Study Group	Dosage	Dose	Serum Chemistry Parameters	Unit	Day	Date of sample Collection	Results	Out of Range	Reference Range	Flag	Clinical Significance*
S275	302	Hepatitis B	Hepatitis B	2	Serum creatinine	mg / dl	Day 56	05/01/2011	1.1	.	0.7 - 1.2		
S275	302	Hepatitis B	Hepatitis B	2	Potassium	mEq / L	Day 56	05/01/2011	3.5	.	3.5 - 5.1		
S275	302	Hepatitis B	Hepatitis B	2	Random blood glucose	mg / dl	Day 56	05/01/2011	97	.	45-130		
S275	302	Hepatitis B	Hepatitis B	2	Sodium	mEq / L	Day 56	05/01/2011	140	.	136 - 145		
S275	302	Hepatitis B	Hepatitis B	3	ALT	U / L	Day 180	09/05/2011	38	.	0 - 41		
S275	302	Hepatitis B	Hepatitis B	3	AST	U / L	Day 180	09/05/2011	26	.	0 - 40		
S275	302	Hepatitis B	Hepatitis B	3	Alkaline Phosphatase	U / L	Day 180	09/05/2011	65	.	40 - 129		
S275	302	Hepatitis B	Hepatitis B	3	Direct Bilirubin	mg / dl	Day 180	09/05/2011	0.19	.	0.00 - 0.30		
S275	302	Hepatitis B	Hepatitis B	3	Gamma Glutamyl Transpeptidase	U / L	Day 180	09/05/2011	44	.	10 - 71		
S275	302	Hepatitis B	Hepatitis B	3	Indirect Bilirubin	mg / dl	Day 180	09/05/2011	0.32	.	0.00 - 0.99		
S275	302	Hepatitis B	Hepatitis B	3	Total Bilirubin	mg / dl	Day 180	09/05/2011	0.51	.	0.00 - 0.99		
S275	302	Hepatitis B	Hepatitis B	3	Serum creatinine	mg / dl	Day 180	09/05/2011	1.1	.	0.7 - 1.2		
S275	302	Hepatitis B	Hepatitis B	3	Potassium	mEq / L	Day 180	09/05/2011	3.6	.	3.5 - 5.1		
S275	302	Hepatitis B	Hepatitis B	3	Random blood glucose	mg / dl	Day 180	09/05/2011	93	.	45-130		
S275	302	Hepatitis B	Hepatitis B	3	Sodium	mEq / L	Day 180	09/05/2011	144	.	136 - 145		
S275	302	Hepatitis B	Hepatitis B	3	ALT	U / L	Day 208	06/06/2011	29	.	0 - 41		
S275	302	Hepatitis B	Hepatitis B	3	AST	U / L	Day 208	06/06/2011	25	.	0 - 40		
S275	302	Hepatitis B	Hepatitis B	3	Alkaline Phosphatase	U / L	Day 208	06/06/2011	73	.	40 - 129		
S275	302	Hepatitis B	Hepatitis B	3	Direct Bilirubin	mg / dl	Day 208	06/06/2011	0.17	.	0.00 - 0.30		
S275	302	Hepatitis B	Hepatitis B	3	Gamma Glutamyl Transpeptidase	U / L	Day 208	06/06/2011	48	.	10 - 71		
S275	302	Hepatitis B	Hepatitis B	3	Indirect Bilirubin	mg / dl	Day 208	06/06/2011	0.37	.	0.00 - 0.99		
S275	302	Hepatitis B	Hepatitis B	3	Total Bilirubin	mg / dl	Day 208	06/06/2011	0.54	.	0.00 - 0.99		
S275	302	Hepatitis B	Hepatitis B	3	Serum creatinine	mg / dl	Day 208	06/06/2011	1.1	.	0.7 - 1.2		
S275	302	Hepatitis B	Hepatitis B	3	Potassium	mEq / L	Day 208	06/06/2011	3.4	Yes	3.5 - 5.1	L	NCS
S275	302	Hepatitis B	Hepatitis B	3	Random blood glucose	mg / dl	Day 208	06/06/2011	95	.	45-130		
S275	302	Hepatitis B	Hepatitis B	3	Sodium	mEq / L	Day 208	06/06/2011	141	.	136 - 145		
S275	302	Hepatitis B	Hepatitis B	3	ALT	U / L	Day 365	09/11/2011	27	.	0 - 41		
S275	302	Hepatitis B	Hepatitis B	3	AST	U / L	Day 365	09/11/2011	27	.	0 - 40		
S275	302	Hepatitis B	Hepatitis B	3	Alkaline Phosphatase	U / L	Day 365	09/11/2011	57	.	40 - 129		

Subject No.	Randomisation Number	Study Group	Dosage	Dose	Serum Chemistry Parameters	Unit	Day	Date of sample Collection	Results	Out of Range	Reference Range	Flag	Clinical Significance*
S275	302	Hepatitis B	Hepatitis B	3	Direct Bilirubin	mg / dl	Day 365	09/11/2011	0.13	.	0.00 - 0.30		
S275	302	Hepatitis B	Hepatitis B	3	Gamma Glutamyl Transpeptidase	U / L	Day 365	09/11/2011	38	.	10 - 71		
S275	302	Hepatitis B	Hepatitis B	3	Indirect Bilirubin	mg / dl	Day 365	09/11/2011	0.31	.	0.00 - 0.99		
S275	302	Hepatitis B	Hepatitis B	3	Total Bilirubin	mg / dl	Day 365	09/11/2011	0.44	.	0.00 - 0.99		
S275	302	Hepatitis B	Hepatitis B	3	Serum creatinine	mg / dl	Day 365	09/11/2011	1.3	Yes	0.7 - 1.2	H	CS
S275	302	Hepatitis B	Hepatitis B	3	Potassium	mEq / L	Day 365	09/11/2011	4	.	3.5 - 5.1		
S275	302	Hepatitis B	Hepatitis B	3	Random blood glucose	mg / dl	Day 365	09/11/2011	100	.	45-130		
S275	302	Hepatitis B	Hepatitis B	3	Sodium	mEq / L	Day 365	09/11/2011	142	.	136 - 145		
S278	315	JAIVAC-1	Dosage C		ALT	U / L	Day -14 to Day -1	08/11/2010	19	.	0 - 41		
S278	315	JAIVAC-1	Dosage C		AST	U / L	Day -14 to Day -1	08/11/2010	19	.	0 - 40		
S278	315	JAIVAC-1	Dosage C		Alkaline Phosphatase	U / L	Day -14 to Day -1	08/11/2010	71	.	40 - 129		
S278	315	JAIVAC-1	Dosage C		Direct Bilirubin	mg / dl	Day -14 to Day -1	08/11/2010	0.22	.	0.00 - 0.30		
S278	315	JAIVAC-1	Dosage C		Gamma Glutamyl Transpeptidase	U / L	Day -14 to Day -1	08/11/2010	32	.	10 - 71		
S278	315	JAIVAC-1	Dosage C		Indirect Bilirubin	mg / dl	Day -14 to Day -1	08/11/2010	0.45	.	0.00 - 0.99		
S278	315	JAIVAC-1	Dosage C		Total Bilirubin	mg / dl	Day -14 to Day -1	08/11/2010	0.67	.	0.00 - 0.99		
S278	315	JAIVAC-1	Dosage C		Serum creatinine	mg / dl	Day -14 to Day -1	08/11/2010	0.8	.	0.7 - 1.2		
S278	315	JAIVAC-1	Dosage C		Potassium	mEq / L	Day -14 to Day -1	08/11/2010	4.2	.	3.5 - 5.1		
S278	315	JAIVAC-1	Dosage C		Random blood glucose	mg / dl	Day -14 to Day -1	08/11/2010	92	.	45-130		
S278	315	JAIVAC-1	Dosage C		Sodium	mEq / L	Day -14 to Day -1	08/11/2010	140	.	136 - 145		
S278	315	JAIVAC-1	Dosage C	2	ALT	U / L	Day 28	10/12/2010	28	.	0 - 41		
S278	315	JAIVAC-1	Dosage C	2	AST	U / L	Day 28	10/12/2010	33	.	0 - 40		
S278	315	JAIVAC-1	Dosage C	2	Alkaline Phosphatase	U / L	Day 28	10/12/2010	58	.	40 - 129		
S278	315	JAIVAC-1	Dosage C	2	Direct Bilirubin	mg / dl	Day 28	10/12/2010	0.14	.	0.00 - 0.30		
S278	315	JAIVAC-1	Dosage C	2	Gamma Glutamyl Transpeptidase	U / L	Day 28	10/12/2010	34	.	10 - 71		
S278	315	JAIVAC-1	Dosage C	2	Indirect Bilirubin	mg / dl	Day 28	10/12/2010	0.39	.	0.00 - 0.99		
S278	315	JAIVAC-1	Dosage C	2	Total Bilirubin	mg / dl	Day 28	10/12/2010	0.53	.	0.00 - 0.99		
S278	315	JAIVAC-1	Dosage C	2	Serum creatinine	mg / dl	Day 28	10/12/2010	1.1	.	0.7 - 1.2		
S278	315	JAIVAC-1	Dosage C	2	Potassium	mEq / L	Day 28	10/12/2010	3.8	.	3.5 - 5.1		
S278	315	JAIVAC-1	Dosage C	2	Random blood glucose	mg / dl	Day 28	10/12/2010	126	.	45-130		

Subject No.	Randomisation Number	Study Group	Dosage	Dose	Serum Chemistry Parameters	Unit	Day	Date of sample Collection	Results	Out of Range	Reference Range	Flag	Clinical Significance*
S278	315	JAIVAC-1	Dosage C	2	Sodium	mEq / L	Day 28	10/12/2010	139	.	136 - 145		
S278	315	JAIVAC-1	Dosage C	2	ALT	U / L	Day 56	07/01/2011	20	.	0 - 41		
S278	315	JAIVAC-1	Dosage C	2	AST	U / L	Day 56	07/01/2011	22	.	0 - 40		
S278	315	JAIVAC-1	Dosage C	2	Alkaline Phosphatase	U / L	Day 56	07/01/2011	76	.	40 - 129		
S278	315	JAIVAC-1	Dosage C	2	Direct Bilirubin	mg / dl	Day 56	07/01/2011	0.15	.	0.00 - 0.30		
S278	315	JAIVAC-1	Dosage C	2	Gamma Glutamyl Transpeptidase	U / L	Day 56	07/01/2011	29	.	10 - 71		
S278	315	JAIVAC-1	Dosage C	2	Indirect Bilirubin	mg / dl	Day 56	07/01/2011	0.18	.	0.00 - 0.99		
S278	315	JAIVAC-1	Dosage C	2	Total Bilirubin	mg / dl	Day 56	07/01/2011	0.33	.	0.00 - 0.99		
S278	315	JAIVAC-1	Dosage C	2	Serum creatinine	mg / dl	Day 56	07/01/2011	0.9	.	0.7 - 1.2		
S278	315	JAIVAC-1	Dosage C	2	Potassium	mEq / L	Day 56	07/01/2011	3.8	.	3.5 - 5.1		
S278	315	JAIVAC-1	Dosage C	2	Random blood glucose	mg / dl	Day 56	07/01/2011	74	.	45-130		
S278	315	JAIVAC-1	Dosage C	2	Sodium	mEq / L	Day 56	07/01/2011	143	.	136 - 145		
S278	315	JAIVAC-1	Dosage C	3	ALT	U / L	Day 180	11/05/2011	23	.	0 - 41		
S278	315	JAIVAC-1	Dosage C	3	AST	U / L	Day 180	11/05/2011	20	.	0 - 40		
S278	315	JAIVAC-1	Dosage C	3	Alkaline Phosphatase	U / L	Day 180	11/05/2011	68	.	40 - 129		
S278	315	JAIVAC-1	Dosage C	3	Direct Bilirubin	mg / dl	Day 180	11/05/2011	0.14	.	0.00 - 0.30		
S278	315	JAIVAC-1	Dosage C	3	Gamma Glutamyl Transpeptidase	U / L	Day 180	11/05/2011	26	.	10 - 71		
S278	315	JAIVAC-1	Dosage C	3	Indirect Bilirubin	mg / dl	Day 180	11/05/2011	0.23	.	0.00 - 0.99		
S278	315	JAIVAC-1	Dosage C	3	Total Bilirubin	mg / dl	Day 180	11/05/2011	0.37	.	0.00 - 0.99		
S278	315	JAIVAC-1	Dosage C	3	Serum creatinine	mg / dl	Day 180	11/05/2011	1.1	.	0.7 - 1.2		
S278	315	JAIVAC-1	Dosage C	3	Potassium	mEq / L	Day 180	11/05/2011	4.2	.	3.5 - 5.1		
S278	315	JAIVAC-1	Dosage C	3	Random blood glucose	mg / dl	Day 180	11/05/2011	74	.	45-130		
S278	315	JAIVAC-1	Dosage C	3	Sodium	mEq / L	Day 180	11/05/2011	141	.	136 - 145		
S278	315	JAIVAC-1	Dosage C	3	ALT	U / L	Day 208	09/06/2011	34	.	0 - 41		
S278	315	JAIVAC-1	Dosage C	3	AST	U / L	Day 208	09/06/2011	40	.	0 - 40		
S278	315	JAIVAC-1	Dosage C	3	Alkaline Phosphatase	U / L	Day 208	09/06/2011	52	.	40 - 129		
S278	315	JAIVAC-1	Dosage C	3	Direct Bilirubin	mg / dl	Day 208	09/06/2011	0.15	.	0.00 - 0.30		
S278	315	JAIVAC-1	Dosage C	3	Gamma Glutamyl Transpeptidase	U / L	Day 208	09/06/2011	33	.	10 - 71		
S278	315	JAIVAC-1	Dosage C	3	Indirect Bilirubin	mg / dl	Day 208	09/06/2011	0.45	.	0.00 - 0.99		

Subject No.	Randomisation Number	Study Group	Dosage	Dose	Serum Chemistry Parameters	Unit	Day	Date of sample Collection	Results	Out of Range	Reference Range	Flag	Clinical Significance*
S278	315	JAIVAC-1	Dosage C	3	Total Bilirubin	mg / dl	Day 208	09/06/2011	0.6	.	0.00 - 0.99		
S278	315	JAIVAC-1	Dosage C	3	Serum creatinine	mg / dl	Day 208	09/06/2011	0.8	.	0.7 - 1.2		
S278	315	JAIVAC-1	Dosage C	3	Potassium	mEq / L	Day 208	09/06/2011	3.8	.	3.5 - 5.1		
S278	315	JAIVAC-1	Dosage C	3	Random blood glucose	mg / dl	Day 208	09/06/2011	99	.	45-130		
S278	315	JAIVAC-1	Dosage C	3	Sodium	mEq / L	Day 208	09/06/2011	139	.	136 - 145		
S278	315	JAIVAC-1	Dosage C	3	ALT	U / L	Day 365	10/11/2011	30	.	0 - 41		
S278	315	JAIVAC-1	Dosage C	3	AST	U / L	Day 365	10/11/2011	29	.	0 - 40		
S278	315	JAIVAC-1	Dosage C	3	Alkaline Phosphatase	U / L	Day 365	10/11/2011	62	.	40 - 129		
S278	315	JAIVAC-1	Dosage C	3	Direct Bilirubin	mg / dl	Day 365	10/11/2011	0.17	.	0.00 - 0.30		
S278	315	JAIVAC-1	Dosage C	3	Gamma Glutamyl Transpeptidase	U / L	Day 365	10/11/2011	52	.	10 - 71		
S278	315	JAIVAC-1	Dosage C	3	Indirect Bilirubin	mg / dl	Day 365	10/11/2011	0.31	.	0.00 - 0.99		
S278	315	JAIVAC-1	Dosage C	3	Total Bilirubin	mg / dl	Day 365	10/11/2011	0.48	.	0.00 - 0.99		
S278	315	JAIVAC-1	Dosage C	3	Serum creatinine	mg / dl	Day 365	10/11/2011	1.2	.	0.7 - 1.2		
S278	315	JAIVAC-1	Dosage C	3	Potassium	mEq / L	Day 365	10/11/2011	4.2	.	3.5 - 5.1		
S278	315	JAIVAC-1	Dosage C	3	Random blood glucose	mg / dl	Day 365	10/11/2011	86	.	45-130		
S278	315	JAIVAC-1	Dosage C	3	Sodium	mEq / L	Day 365	10/11/2011	139	.	136 - 145		
S279	301	JAIVAC-1	Dosage C		ALT	U / L	Day -14 to Day -1	03/11/2010	39	.	0 - 41		
S279	301	JAIVAC-1	Dosage C		AST	U / L	Day -14 to Day -1	03/11/2010	27	.	0 - 40		
S279	301	JAIVAC-1	Dosage C		Alkaline Phosphatase	U / L	Day -14 to Day -1	03/11/2010	58	.	40 - 129		
S279	301	JAIVAC-1	Dosage C		Direct Bilirubin	mg / dl	Day -14 to Day -1	03/11/2010	<0.10	.	0.00 - 0.30		
S279	301	JAIVAC-1	Dosage C		Gamma Glutamyl Transpeptidase	U / L	Day -14 to Day -1	03/11/2010	29	.	10 - 71		
S279	301	JAIVAC-1	Dosage C		Indirect Bilirubin	mg / dl	Day -14 to Day -1	03/11/2010	NA	.	0.00 - 0.99		
S279	301	JAIVAC-1	Dosage C		Total Bilirubin	mg / dl	Day -14 to Day -1	03/11/2010	0.36	.	0.00 - 0.99		
S279	301	JAIVAC-1	Dosage C		Serum creatinine	mg / dl	Day -14 to Day -1	03/11/2010	1	.	0.7 - 1.2		
S279	301	JAIVAC-1	Dosage C		Potassium	mEq / L	Day -14 to Day -1	03/11/2010	4.5	.	3.5 - 5.1		
S279	301	JAIVAC-1	Dosage C		Random blood glucose	mg / dl	Day -14 to Day -1	03/11/2010	83	.	45-130		
S279	301	JAIVAC-1	Dosage C		Sodium	mEq / L	Day -14 to Day -1	03/11/2010	138	.	136 - 145		
S279	301	JAIVAC-1	Dosage C	2	ALT	U / L	Day 28	08/12/2010	40	.	0 - 41		
S279	301	JAIVAC-1	Dosage C	2	AST	U / L	Day 28	08/12/2010	25	.	0 - 40		

Subject No.	Randomisation Number	Study Group	Dosage	Dose	Serum Chemistry Parameters	Unit	Day	Date of sample Collection	Results	Out of Range	Reference Range	Flag	Clinical Significance*
S279	301	JAIVAC-1	Dosage C	2	Alkaline Phosphatase	U / L	Day 28	08/12/2010	60	.	40 - 129		
S279	301	JAIVAC-1	Dosage C	2	Direct Bilirubin	mg / dl	Day 28	08/12/2010	<0.10	.	0.00 - 0.30		
S279	301	JAIVAC-1	Dosage C	2	Gamma Glutamyl Transpeptidase	U / L	Day 28	08/12/2010	29	.	10 - 71		
S279	301	JAIVAC-1	Dosage C	2	Indirect Bilirubin	mg / dl	Day 28	08/12/2010	NA	.	0.00 - 0.99		
S279	301	JAIVAC-1	Dosage C	2	Total Bilirubin	mg / dl	Day 28	08/12/2010	0.4	.	0.00 - 0.99		
S279	301	JAIVAC-1	Dosage C	2	Serum creatinine	mg / dl	Day 28	08/12/2010	1.4	Yes	0.7 - 1.2	H	NCS
S279	301	JAIVAC-1	Dosage C	2	Potassium	mEq / L	Day 28	08/12/2010	4.1	.	3.5 - 5.1		
S279	301	JAIVAC-1	Dosage C	2	Random blood glucose	mg / dl	Day 28	08/12/2010	108	.	45-130		
S279	301	JAIVAC-1	Dosage C	2	Sodium	mEq / L	Day 28	08/12/2010	135	Yes	136 - 145	L	NCS
S279	301	JAIVAC-1	Dosage C	2	ALT	U / L	Day 56	05/01/2011	30	.	0 - 41		
S279	301	JAIVAC-1	Dosage C	2	AST	U / L	Day 56	05/01/2011	22	.	0 - 40		
S279	301	JAIVAC-1	Dosage C	2	Alkaline Phosphatase	U / L	Day 56	05/01/2011	66	.	40 - 129		
S279	301	JAIVAC-1	Dosage C	2	Direct Bilirubin	mg / dl	Day 56	05/01/2011	0.1	.	0.00 - 0.30		
S279	301	JAIVAC-1	Dosage C	2	Gamma Glutamyl Transpeptidase	U / L	Day 56	05/01/2011	33	.	10 - 71		
S279	301	JAIVAC-1	Dosage C	2	Indirect Bilirubin	mg / dl	Day 56	05/01/2011	0.25	.	0.00 - 0.99		
S279	301	JAIVAC-1	Dosage C	2	Total Bilirubin	mg / dl	Day 56	05/01/2011	0.35	.	0.00 - 0.99		
S279	301	JAIVAC-1	Dosage C	2	Serum creatinine	mg / dl	Day 56	05/01/2011	1	.	0.7 - 1.2		
S279	301	JAIVAC-1	Dosage C	2	Potassium	mEq / L	Day 56	05/01/2011	4.3	.	3.5 - 5.1		
S279	301	JAIVAC-1	Dosage C	2	Random blood glucose	mg / dl	Day 56	05/01/2011	123	.	45-130		
S279	301	JAIVAC-1	Dosage C	2	Sodium	mEq / L	Day 56	05/01/2011	138	.	136 - 145		
S279	301	JAIVAC-1	Dosage C	3	ALT	U / L	Day 180	09/05/2011	31	.	0 - 41		
S279	301	JAIVAC-1	Dosage C	3	AST	U / L	Day 180	09/05/2011	19	.	0 - 40		
S279	301	JAIVAC-1	Dosage C	3	Alkaline Phosphatase	U / L	Day 180	09/05/2011	63	.	40 - 129		
S279	301	JAIVAC-1	Dosage C	3	Direct Bilirubin	mg / dl	Day 180	09/05/2011	0.11	.	0.00 - 0.30		
S279	301	JAIVAC-1	Dosage C	3	Gamma Glutamyl Transpeptidase	U / L	Day 180	09/05/2011	35	.	10 - 71		
S279	301	JAIVAC-1	Dosage C	3	Indirect Bilirubin	mg / dl	Day 180	09/05/2011	0.18	.	0.00 - 0.99		
S279	301	JAIVAC-1	Dosage C	3	Total Bilirubin	mg / dl	Day 180	09/05/2011	0.29	.	0.00 - 0.99		
S279	301	JAIVAC-1	Dosage C	3	Serum creatinine	mg / dl	Day 180	09/05/2011	1.2	.	0.7 - 1.2		
S279	301	JAIVAC-1	Dosage C	3	Potassium	mEq / L	Day 180	09/05/2011	5.1	.	3.5 - 5.1		

Subject No.	Randomisation Number	Study Group	Dosage	Dose	Serum Chemistry Parameters	Unit	Day	Date of sample Collection	Results	Out of Range	Reference Range	Flag	Clinical Significance*
S279	301	JAIVAC-1	Dosage C	3	Random blood glucose	mg / dl	Day 180	09/05/2011	189	Yes	45-130	H	CS
S279	301	JAIVAC-1	Dosage C	3	Sodium	mEq / L	Day 180	09/05/2011	140	.	136 - 145		
S279	301	JAIVAC-1	Dosage C	3	ALT	U / L	Day 208	06/06/2011	59	Yes	0 - 41	H	NCS
S279	301	JAIVAC-1	Dosage C	3	AST	U / L	Day 208	06/06/2011	31	.	0 - 40		
S279	301	JAIVAC-1	Dosage C	3	Alkaline Phosphatase	U / L	Day 208	06/06/2011	67	.	40 - 129		
S279	301	JAIVAC-1	Dosage C	3	Direct Bilirubin	mg / dl	Day 208	06/06/2011	<0.10	.	0.00 - 0.30		
S279	301	JAIVAC-1	Dosage C	3	Gamma Glutamyl Transpeptidase	U / L	Day 208	06/06/2011	39	.	10 - 71		
S279	301	JAIVAC-1	Dosage C	3	Indirect Bilirubin	mg / dl	Day 208	06/06/2011	NA	.	0.00 - 0.99		
S279	301	JAIVAC-1	Dosage C	3	Total Bilirubin	mg / dl	Day 208	06/06/2011	0.24	.	0.00 - 0.99		
S279	301	JAIVAC-1	Dosage C	3	Serum creatinine	mg / dl	Day 208	06/06/2011	1	.	0.7 - 1.2		
S279	301	JAIVAC-1	Dosage C	3	Potassium	mEq / L	Day 208	06/06/2011	4.6	.	3.5 - 5.1		
S279	301	JAIVAC-1	Dosage C	3	Random blood glucose	mg / dl	Day 208	06/06/2011	83	.	45-130		
S279	301	JAIVAC-1	Dosage C	3	Sodium	mEq / L	Day 208	06/06/2011	138	.	136 - 145		
S279	301	JAIVAC-1	Dosage C	3	ALT	U / L	Day 365	09/11/2011	61	Yes	0 - 41	H	CS
S279	301	JAIVAC-1	Dosage C	3	AST	U / L	Day 365	09/11/2011	33	.	0 - 40		
S279	301	JAIVAC-1	Dosage C	3	Alkaline Phosphatase	U / L	Day 365	09/11/2011	74	.	40 - 129		
S279	301	JAIVAC-1	Dosage C	3	Direct Bilirubin	mg / dl	Day 365	09/11/2011	<0.10	.	0.00 - 0.30		
S279	301	JAIVAC-1	Dosage C	3	Gamma Glutamyl Transpeptidase	U / L	Day 365	09/11/2011	52	.	10 - 71		
S279	301	JAIVAC-1	Dosage C	3	Indirect Bilirubin	mg / dl	Day 365	09/11/2011	NA	.	0.00 - 0.99		
S279	301	JAIVAC-1	Dosage C	3	Total Bilirubin	mg / dl	Day 365	09/11/2011	0.33	.	0.00 - 0.99		
S279	301	JAIVAC-1	Dosage C	3	Serum creatinine	mg / dl	Day 365	09/11/2011	1.3	Yes	0.7 - 1.2	H	CS
S279	301	JAIVAC-1	Dosage C	3	Potassium	mEq / L	Day 365	09/11/2011	4.5	.	3.5 - 5.1		
S279	301	JAIVAC-1	Dosage C	3	Random blood glucose	mg / dl	Day 365	09/11/2011	87	.	45-130		
S279	301	JAIVAC-1	Dosage C	3	Sodium	mEq / L	Day 365	09/11/2011	140	.	136 - 145		
S281	304	JAIVAC-1	Dosage C		ALT	U / L	Day -14 to Day -1	03/11/2010	15	.	0 - 41		
S281	304	JAIVAC-1	Dosage C		AST	U / L	Day -14 to Day -1	03/11/2010	22	.	0 - 40		
S281	304	JAIVAC-1	Dosage C		Alkaline Phosphatase	U / L	Day -14 to Day -1	03/11/2010	47	.	40 - 129		
S281	304	JAIVAC-1	Dosage C		Direct Bilirubin	mg / dl	Day -14 to Day -1	03/11/2010	0.16	.	0.00 - 0.30		
S281	304	JAIVAC-1	Dosage C		Gamma Glutamyl Transpeptidase	U / L	Day -14 to Day -1	03/11/2010	14	.	10 - 71		

Subject No.	Randomisation Number	Study Group	Dosage	Dose	Serum Chemistry Parameters	Unit	Day	Date of sample Collection	Results	Out of Range	Reference Range	Flag	Clinical Significance*
S281	304	JAIVAC-1	Dosage C		Indirect Bilirubin	mg / dl	Day -14 to Day -1	03/11/2010	0.36	.	0.00 - 0.99		
S281	304	JAIVAC-1	Dosage C		Total Bilirubin	mg / dl	Day -14 to Day -1	03/11/2010	0.52	.	0.00 - 0.99		
S281	304	JAIVAC-1	Dosage C		Serum creatinine	mg / dl	Day -14 to Day -1	03/11/2010	1.1	.	0.7 - 1.2		
S281	304	JAIVAC-1	Dosage C		Potassium	mEq / L	Day -14 to Day -1	03/11/2010	4	.	3.5 - 5.1		
S281	304	JAIVAC-1	Dosage C		Random blood glucose	mg / dl	Day -14 to Day -1	03/11/2010	88	.	45-130		
S281	304	JAIVAC-1	Dosage C		Sodium	mEq / L	Day -14 to Day -1	03/11/2010	139	.	136 - 145		
S281	304	JAIVAC-1	Dosage C	2	ALT	U / L	Day 28	09/12/2010	15	.	0 - 41		
S281	304	JAIVAC-1	Dosage C	2	AST	U / L	Day 28	09/12/2010	21	.	0 - 40		
S281	304	JAIVAC-1	Dosage C	2	Alkaline Phosphatase	U / L	Day 28	09/12/2010	45	.	40 - 129		
S281	304	JAIVAC-1	Dosage C	2	Direct Bilirubin	mg / dl	Day 28	09/12/2010	0.16	.	0.00 - 0.30		
S281	304	JAIVAC-1	Dosage C	2	Gamma Glutamyl Transpeptidase	U / L	Day 28	09/12/2010	15	.	10 - 71		
S281	304	JAIVAC-1	Dosage C	2	Indirect Bilirubin	mg / dl	Day 28	09/12/2010	0.41	.	0.00 - 0.99		
S281	304	JAIVAC-1	Dosage C	2	Total Bilirubin	mg / dl	Day 28	09/12/2010	0.57	.	0.00 - 0.99		
S281	304	JAIVAC-1	Dosage C	2	Serum creatinine	mg / dl	Day 28	09/12/2010	1.1	.	0.7 - 1.2		
S281	304	JAIVAC-1	Dosage C	2	Potassium	mEq / L	Day 28	09/12/2010	3.8	.	3.5 - 5.1		
S281	304	JAIVAC-1	Dosage C	2	Random blood glucose	mg / dl	Day 28	09/12/2010	109	.	45-130		
S281	304	JAIVAC-1	Dosage C	2	Sodium	mEq / L	Day 28	09/12/2010	137	.	136 - 145		
S281	304	JAIVAC-1	Dosage C	2	ALT	U / L	Day 56	06/01/2011	20	.	0 - 41		
S281	304	JAIVAC-1	Dosage C	2	AST	U / L	Day 56	06/01/2011	21	.	0 - 40		
S281	304	JAIVAC-1	Dosage C	2	Alkaline Phosphatase	U / L	Day 56	06/01/2011	45	.	40 - 129		
S281	304	JAIVAC-1	Dosage C	2	Direct Bilirubin	mg / dl	Day 56	06/01/2011	0.13	.	0.00 - 0.30		
S281	304	JAIVAC-1	Dosage C	2	Gamma Glutamyl Transpeptidase	U / L	Day 56	06/01/2011	20	.	10 - 71		
S281	304	JAIVAC-1	Dosage C	2	Indirect Bilirubin	mg / dl	Day 56	06/01/2011	0.31	.	0.00 - 0.99		
S281	304	JAIVAC-1	Dosage C	2	Total Bilirubin	mg / dl	Day 56	06/01/2011	0.44	.	0.00 - 0.99		
S281	304	JAIVAC-1	Dosage C	2	Serum creatinine	mg / dl	Day 56	06/01/2011	1.1	.	0.7 - 1.2		
S281	304	JAIVAC-1	Dosage C	2	Potassium	mEq / L	Day 56	06/01/2011	3.9	.	3.5 - 5.1		
S281	304	JAIVAC-1	Dosage C	2	Random blood glucose	mg / dl	Day 56	06/01/2011	114	.	45-130		
S281	304	JAIVAC-1	Dosage C	2	Sodium	mEq / L	Day 56	06/01/2011	140	.	136 - 145		
S281	304	JAIVAC-1	Dosage C		ALT	U / L	Early Termination	11/05/2011	18	.	0 - 41		

Subject No.	Randomisation Number	Study Group	Dosage	Dose	Serum Chemistry Parameters	Unit	Day	Date of sample Collection	Results	Out of Range	Reference Range	Flag	Clinical Significance*
S281	304	JAIVAC-1	Dosage C		AST	U / L	Early Termination	11/05/2011	18	.	0 - 40		
S281	304	JAIVAC-1	Dosage C		Alkaline Phosphatase	U / L	Early Termination	11/05/2011	57	.	40 - 129		
S281	304	JAIVAC-1	Dosage C		Direct Bilirubin	mg / dl	Early Termination	11/05/2011	0.14	.	0.00 - 0.30		
S281	304	JAIVAC-1	Dosage C		Gamma Glutamyl Transpeptidase	U / L	Early Termination	11/05/2011	15	.	10 - 71		
S281	304	JAIVAC-1	Dosage C		Indirect Bilirubin	mg / dl	Early Termination	11/05/2011	0.29	.	0.00 - 0.99		
S281	304	JAIVAC-1	Dosage C		Total Bilirubin	mg / dl	Early Termination	11/05/2011	0.43	.	0.00 - 0.99		
S281	304	JAIVAC-1	Dosage C		Serum creatinine	mg / dl	Early Termination	11/05/2011	1.3	Yes	0.7 - 1.2	H	NCS
S281	304	JAIVAC-1	Dosage C		Potassium	mEq / L	Early Termination	11/05/2011	4.2	.	3.5 - 5.1		
S281	304	JAIVAC-1	Dosage C		Random blood glucose	mg / dl	Early Termination	11/05/2011	92	.	45-130		
S281	304	JAIVAC-1	Dosage C		Sodium	mEq / L	Early Termination	11/05/2011	141	.	136 - 145		
S284	305	JAIVAC-1	Dosage C		ALT	U / L	Day -14 to Day -1	03/11/2010	29	.	0 - 41		
S284	305	JAIVAC-1	Dosage C		AST	U / L	Day -14 to Day -1	03/11/2010	25	.	0 - 40		
S284	305	JAIVAC-1	Dosage C		Alkaline Phosphatase	U / L	Day -14 to Day -1	03/11/2010	91	.	40 - 129		
S284	305	JAIVAC-1	Dosage C		Direct Bilirubin	mg / dl	Day -14 to Day -1	03/11/2010	0.14	.	0.00 - 0.30		
S284	305	JAIVAC-1	Dosage C		Gamma Glutamyl Transpeptidase	U / L	Day -14 to Day -1	03/11/2010	12	.	10 - 71		
S284	305	JAIVAC-1	Dosage C		Indirect Bilirubin	mg / dl	Day -14 to Day -1	03/11/2010	0.42	.	0.00 - 0.99		
S284	305	JAIVAC-1	Dosage C		Total Bilirubin	mg / dl	Day -14 to Day -1	03/11/2010	0.56	.	0.00 - 0.99		
S284	305	JAIVAC-1	Dosage C		Serum creatinine	mg / dl	Day -14 to Day -1	03/11/2010	1	.	0.7 - 1.2		
S284	305	JAIVAC-1	Dosage C		Potassium	mEq / L	Day -14 to Day -1	03/11/2010	4.2	.	3.5 - 5.1		
S284	305	JAIVAC-1	Dosage C		Random blood glucose	mg / dl	Day -14 to Day -1	03/11/2010	113	.	45-130		
S284	305	JAIVAC-1	Dosage C		Sodium	mEq / L	Day -14 to Day -1	03/11/2010	140	.	136 - 145		
S284	305	JAIVAC-1	Dosage C	2	ALT	U / L	Day 28	09/12/2010	36	.	0 - 41		
S284	305	JAIVAC-1	Dosage C	2	AST	U / L	Day 28	09/12/2010	29	.	0 - 40		
S284	305	JAIVAC-1	Dosage C	2	Alkaline Phosphatase	U / L	Day 28	09/12/2010	92	.	40 - 129		
S284	305	JAIVAC-1	Dosage C	2	Direct Bilirubin	mg / dl	Day 28	09/12/2010	0.17	.	0.00 - 0.30		
S284	305	JAIVAC-1	Dosage C	2	Gamma Glutamyl Transpeptidase	U / L	Day 28	09/12/2010	13	.	10 - 71		
S284	305	JAIVAC-1	Dosage C	2	Indirect Bilirubin	mg / dl	Day 28	09/12/2010	0.45	.	0.00 - 0.99		
S284	305	JAIVAC-1	Dosage C	2	Total Bilirubin	mg / dl	Day 28	09/12/2010	0.62	.	0.00 - 0.99		
S284	305	JAIVAC-1	Dosage C	2	Serum creatinine	mg / dl	Day 28	09/12/2010	1.2	.	0.7 - 1.2		

Subject No.	Randomisation Number	Study Group	Dosage	Dose	Serum Chemistry Parameters	Unit	Day	Date of sample Collection	Results	Out of Range	Reference Range	Flag	Clinical Significance*
S284	305	JAIVAC-1	Dosage C	2	Potassium	mEq / L	Day 28	09/12/2010	4.6	.	3.5 - 5.1		
S284	305	JAIVAC-1	Dosage C	2	Random blood glucose	mg / dl	Day 28	09/12/2010	115	.	45-130		
S284	305	JAIVAC-1	Dosage C	2	Sodium	mEq / L	Day 28	09/12/2010	139	.	136 - 145		
S284	305	JAIVAC-1	Dosage C	2	ALT	U / L	Day 56	06/01/2011	30	.	0 - 41		
S284	305	JAIVAC-1	Dosage C	2	AST	U / L	Day 56	06/01/2011	28	.	0 - 40		
S284	305	JAIVAC-1	Dosage C	2	Alkaline Phosphatase	U / L	Day 56	06/01/2011	107	.	40 - 129		
S284	305	JAIVAC-1	Dosage C	2	Direct Bilirubin	mg / dl	Day 56	06/01/2011	<0.10	.	0.00 - 0.30		
S284	305	JAIVAC-1	Dosage C	2	Gamma Glutamyl Transpeptidase	U / L	Day 56	06/01/2011	13	.	10 - 71		
S284	305	JAIVAC-1	Dosage C	2	Indirect Bilirubin	mg / dl	Day 56	06/01/2011	NA	.	0.00 - 0.99		
S284	305	JAIVAC-1	Dosage C	2	Total Bilirubin	mg / dl	Day 56	06/01/2011	0.34	.	0.00 - 0.99		
S284	305	JAIVAC-1	Dosage C	2	Serum creatinine	mg / dl	Day 56	06/01/2011	1.1	.	0.7 - 1.2		
S284	305	JAIVAC-1	Dosage C	2	Potassium	mEq / L	Day 56	06/01/2011	4.8	.	3.5 - 5.1		
S284	305	JAIVAC-1	Dosage C	2	Random blood glucose	mg / dl	Day 56	06/01/2011	96	.	45-130		
S284	305	JAIVAC-1	Dosage C	2	Sodium	mEq / L	Day 56	06/01/2011	142	.	136 - 145		
S284	305	JAIVAC-1	Dosage C	3	ALT	U / L	Day 180	10/05/2011	21	.	0 - 41		
S284	305	JAIVAC-1	Dosage C	3	AST	U / L	Day 180	10/05/2011	21	.	0 - 40		
S284	305	JAIVAC-1	Dosage C	3	Alkaline Phosphatase	U / L	Day 180	10/05/2011	101	.	40 - 129		
S284	305	JAIVAC-1	Dosage C	3	Direct Bilirubin	mg / dl	Day 180	10/05/2011	0.18	.	0.00 - 0.30		
S284	305	JAIVAC-1	Dosage C	3	Gamma Glutamyl Transpeptidase	U / L	Day 180	10/05/2011	12	.	10 - 71		
S284	305	JAIVAC-1	Dosage C	3	Indirect Bilirubin	mg / dl	Day 180	10/05/2011	0.54	.	0.00 - 0.99		
S284	305	JAIVAC-1	Dosage C	3	Total Bilirubin	mg / dl	Day 180	10/05/2011	0.72	.	0.00 - 0.99		
S284	305	JAIVAC-1	Dosage C	3	Serum creatinine	mg / dl	Day 180	10/05/2011	1.1	.	0.7 - 1.2		
S284	305	JAIVAC-1	Dosage C	3	Potassium	mEq / L	Day 180	10/05/2011	4.6	.	3.5 - 5.1		
S284	305	JAIVAC-1	Dosage C	3	Random blood glucose	mg / dl	Day 180	10/05/2011	82	.	45-130		
S284	305	JAIVAC-1	Dosage C	3	Sodium	mEq / L	Day 180	10/05/2011	139	.	136 - 145		
S284	305	JAIVAC-1	Dosage C	3	ALT	U / L	Day 208	07/06/2011	26	.	0 - 41		
S284	305	JAIVAC-1	Dosage C	3	AST	U / L	Day 208	07/06/2011	29	.	0 - 40		
S284	305	JAIVAC-1	Dosage C	3	Alkaline Phosphatase	U / L	Day 208	07/06/2011	95	.	40 - 129		
S284	305	JAIVAC-1	Dosage C	3	Direct Bilirubin	mg / dl	Day 208	07/06/2011	0.25	.	0.00 - 0.30		

Subject No.	Randomisation Number	Study Group	Dosage	Dose	Serum Chemistry Parameters	Unit	Day	Date of sample Collection	Results	Out of Range	Reference Range	Flag	Clinical Significance*
S284	305	JAIVAC-1	Dosage C	3	Gamma Glutamyl Transpeptidase	U / L	Day 208	07/06/2011	11	.	10 - 71		
S284	305	JAIVAC-1	Dosage C	3	Indirect Bilirubin	mg / dl	Day 208	07/06/2011	0.39	.	0.00 - 0.99		
S284	305	JAIVAC-1	Dosage C	3	Total Bilirubin	mg / dl	Day 208	07/06/2011	0.64	.	0.00 - 0.99		
S284	305	JAIVAC-1	Dosage C	3	Serum creatinine	mg / dl	Day 208	07/06/2011	1	.	0.7 - 1.2		
S284	305	JAIVAC-1	Dosage C	3	Potassium	mEq / L	Day 208	07/06/2011	4.2	.	3.5 - 5.1		
S284	305	JAIVAC-1	Dosage C	3	Random blood glucose	mg / dl	Day 208	07/06/2011	83	.	45-130		
S284	305	JAIVAC-1	Dosage C	3	Sodium	mEq / L	Day 208	07/06/2011	141	.	136 - 145		
S284	305	JAIVAC-1	Dosage C	3	ALT	U / L	Day 365	09/11/2011	21	.	0 - 41		
S284	305	JAIVAC-1	Dosage C	3	AST	U / L	Day 365	09/11/2011	22	.	0 - 40		
S284	305	JAIVAC-1	Dosage C	3	Alkaline Phosphatase	U / L	Day 365	09/11/2011	103	.	40 - 129		
S284	305	JAIVAC-1	Dosage C	3	Direct Bilirubin	mg / dl	Day 365	09/11/2011	0.12	.	0.00 - 0.30		
S284	305	JAIVAC-1	Dosage C	3	Gamma Glutamyl Transpeptidase	U / L	Day 365	09/11/2011	10	.	10 - 71		
S284	305	JAIVAC-1	Dosage C	3	Indirect Bilirubin	mg / dl	Day 365	09/11/2011	0.36	.	0.00 - 0.99		
S284	305	JAIVAC-1	Dosage C	3	Total Bilirubin	mg / dl	Day 365	09/11/2011	0.48	.	0.00 - 0.99		
S284	305	JAIVAC-1	Dosage C	3	Serum creatinine	mg / dl	Day 365	09/11/2011	1.3	Yes	0.7 - 1.2	H	CS
S284	305	JAIVAC-1	Dosage C	3	Potassium	mEq / L	Day 365	09/11/2011	4.8	.	3.5 - 5.1		
S284	305	JAIVAC-1	Dosage C	3	Random blood glucose	mg / dl	Day 365	09/11/2011	91	.	45-130		
S284	305	JAIVAC-1	Dosage C	3	Sodium	mEq / L	Day 365	09/11/2011	141	.	136 - 145		
S285	307	Hepatitis B	Hepatitis B		ALT	U / L	Day -14 to Day -1	02/11/2010	27	.	0 - 41		
S285	307	Hepatitis B	Hepatitis B		AST	U / L	Day -14 to Day -1	02/11/2010	23	.	0 - 40		
S285	307	Hepatitis B	Hepatitis B		Alkaline Phosphatase	U / L	Day -14 to Day -1	02/11/2010	115	.	40 - 129		
S285	307	Hepatitis B	Hepatitis B		Direct Bilirubin	mg / dl	Day -14 to Day -1	02/11/2010	0.14	.	0.00 - 0.30		
S285	307	Hepatitis B	Hepatitis B		Gamma Glutamyl Transpeptidase	U / L	Day -14 to Day -1	02/11/2010	13	.	10 - 71		
S285	307	Hepatitis B	Hepatitis B		Indirect Bilirubin	mg / dl	Day -14 to Day -1	02/11/2010	0.35	.	0.00 - 0.99		
S285	307	Hepatitis B	Hepatitis B		Total Bilirubin	mg / dl	Day -14 to Day -1	02/11/2010	0.49	.	0.00 - 0.99		
S285	307	Hepatitis B	Hepatitis B		Serum creatinine	mg / dl	Day -14 to Day -1	02/11/2010	0.8	.	0.7 - 1.2		
S285	307	Hepatitis B	Hepatitis B		Potassium	mEq / L	Day -14 to Day -1	02/11/2010	4.4	.	3.5 - 5.1		
S285	307	Hepatitis B	Hepatitis B		Random blood glucose	mg / dl	Day -14 to Day -1	02/11/2010	67	.	45-130		
S285	307	Hepatitis B	Hepatitis B		Sodium	mEq / L	Day -14 to Day -1	02/11/2010	139	.	136 - 145		

Subject No.	Randomisation Number	Study Group	Dosage	Dose	Serum Chemistry Parameters	Unit	Day	Date of sample Collection	Results	Out of Range	Reference Range	Flag	Clinical Significance*
S285	307	Hepatitis B	Hepatitis B	2	ALT	U / L	Day 28	09/12/2010	16	.	0 - 41		
S285	307	Hepatitis B	Hepatitis B	2	AST	U / L	Day 28	09/12/2010	20	.	0 - 40		
S285	307	Hepatitis B	Hepatitis B	2	Alkaline Phosphatase	U / L	Day 28	09/12/2010	108	.	40 - 129		
S285	307	Hepatitis B	Hepatitis B	2	Direct Bilirubin	mg / dl	Day 28	09/12/2010	0.24	.	0.00 - 0.30		
S285	307	Hepatitis B	Hepatitis B	2	Gamma Glutamyl Transpeptidase	U / L	Day 28	09/12/2010	12	.	10 - 71		
S285	307	Hepatitis B	Hepatitis B	2	Indirect Bilirubin	mg / dl	Day 28	09/12/2010	0.5	.	0.00 - 0.99		
S285	307	Hepatitis B	Hepatitis B	2	Total Bilirubin	mg / dl	Day 28	09/12/2010	0.74	.	0.00 - 0.99		
S285	307	Hepatitis B	Hepatitis B	2	Serum creatinine	mg / dl	Day 28	09/12/2010	1.1	.	0.7 - 1.2		
S285	307	Hepatitis B	Hepatitis B	2	Potassium	mEq / L	Day 28	09/12/2010	4.3	.	3.5 - 5.1		
S285	307	Hepatitis B	Hepatitis B	2	Random blood glucose	mg / dl	Day 28	09/12/2010	100	.	45-130		
S285	307	Hepatitis B	Hepatitis B	2	Sodium	mEq / L	Day 28	09/12/2010	138	.	136 - 145		
S285	307	Hepatitis B	Hepatitis B	2	ALT	U / L	Day 56	06/01/2011	25	.	0 - 41		
S285	307	Hepatitis B	Hepatitis B	2	AST	U / L	Day 56	06/01/2011	20	.	0 - 40		
S285	307	Hepatitis B	Hepatitis B	2	Alkaline Phosphatase	U / L	Day 56	06/01/2011	93	.	40 - 129		
S285	307	Hepatitis B	Hepatitis B	2	Direct Bilirubin	mg / dl	Day 56	06/01/2011	0.12	.	0.00 - 0.30		
S285	307	Hepatitis B	Hepatitis B	2	Gamma Glutamyl Transpeptidase	U / L	Day 56	06/01/2011	14	.	10 - 71		
S285	307	Hepatitis B	Hepatitis B	2	Indirect Bilirubin	mg / dl	Day 56	06/01/2011	0.29	.	0.00 - 0.99		
S285	307	Hepatitis B	Hepatitis B	2	Total Bilirubin	mg / dl	Day 56	06/01/2011	0.41	.	0.00 - 0.99		
S285	307	Hepatitis B	Hepatitis B	2	Serum creatinine	mg / dl	Day 56	06/01/2011	0.9	.	0.7 - 1.2		
S285	307	Hepatitis B	Hepatitis B	2	Potassium	mEq / L	Day 56	06/01/2011	4.3	.	3.5 - 5.1		
S285	307	Hepatitis B	Hepatitis B	2	Random blood glucose	mg / dl	Day 56	06/01/2011	104	.	45-130		
S285	307	Hepatitis B	Hepatitis B	2	Sodium	mEq / L	Day 56	06/01/2011	142	.	136 - 145		
S285	307	Hepatitis B	Hepatitis B	3	ALT	U / L	Day 180	10/05/2011	32	.	0 - 41		
S285	307	Hepatitis B	Hepatitis B	3	AST	U / L	Day 180	10/05/2011	25	.	0 - 40		
S285	307	Hepatitis B	Hepatitis B	3	Alkaline Phosphatase	U / L	Day 180	10/05/2011	71	.	40 - 129		
S285	307	Hepatitis B	Hepatitis B	3	Direct Bilirubin	mg / dl	Day 180	10/05/2011	0.12	.	0.00 - 0.30		
S285	307	Hepatitis B	Hepatitis B	3	Gamma Glutamyl Transpeptidase	U / L	Day 180	10/05/2011	14	.	10 - 71		
S285	307	Hepatitis B	Hepatitis B	3	Indirect Bilirubin	mg / dl	Day 180	10/05/2011	0.4	.	0.00 - 0.99		
S285	307	Hepatitis B	Hepatitis B	3	Total Bilirubin	mg / dl	Day 180	10/05/2011	0.52	.	0.00 - 0.99		

Subject No.	Randomisation Number	Study Group	Dosage	Dose	Serum Chemistry Parameters	Unit	Day	Date of sample Collection	Results	Out of Range	Reference Range	Flag	Clinical Significance*
S285	307	Hepatitis B	Hepatitis B	3	Serum creatinine	mg / dl	Day 180	10/05/2011	1	.	0.7 - 1.2		
S285	307	Hepatitis B	Hepatitis B	3	Potassium	mEq / L	Day 180	10/05/2011	4.3	.	3.5 - 5.1		
S285	307	Hepatitis B	Hepatitis B	3	Random blood glucose	mg / dl	Day 180	10/05/2011	71	.	45-130		
S285	307	Hepatitis B	Hepatitis B	3	Sodium	mEq / L	Day 180	10/05/2011	141	.	136 - 145		
S285	307	Hepatitis B	Hepatitis B	3	ALT	U / L	Day 208	07/06/2011	27	.	0 - 41		
S285	307	Hepatitis B	Hepatitis B	3	AST	U / L	Day 208	07/06/2011	34	.	0 - 40		
S285	307	Hepatitis B	Hepatitis B	3	Alkaline Phosphatase	U / L	Day 208	07/06/2011	77	.	40 - 129		
S285	307	Hepatitis B	Hepatitis B	3	Direct Bilirubin	mg / dl	Day 208	07/06/2011	0.16	.	0.00 - 0.30		
S285	307	Hepatitis B	Hepatitis B	3	Gamma Glutamyl Transpeptidase	U / L	Day 208	07/06/2011	15	.	10 - 71		
S285	307	Hepatitis B	Hepatitis B	3	Indirect Bilirubin	mg / dl	Day 208	07/06/2011	0.2	.	0.00 - 0.99		
S285	307	Hepatitis B	Hepatitis B	3	Total Bilirubin	mg / dl	Day 208	07/06/2011	0.36	.	0.00 - 0.99		
S285	307	Hepatitis B	Hepatitis B	3	Serum creatinine	mg / dl	Day 208	07/06/2011	0.9	.	0.7 - 1.2		
S285	307	Hepatitis B	Hepatitis B	3	Potassium	mEq / L	Day 208	07/06/2011	4.2	.	3.5 - 5.1		
S285	307	Hepatitis B	Hepatitis B	3	Random blood glucose	mg / dl	Day 208	07/06/2011	76	.	45-130		
S285	307	Hepatitis B	Hepatitis B	3	Sodium	mEq / L	Day 208	07/06/2011	141	.	136 - 145		
S285	307	Hepatitis B	Hepatitis B	3	ALT	U / L	Day 365	09/11/2011	49	Yes	0 - 41	H	CS
S285	307	Hepatitis B	Hepatitis B	3	AST	U / L	Day 365	09/11/2011	31	.	0 - 40		
S285	307	Hepatitis B	Hepatitis B	3	Alkaline Phosphatase	U / L	Day 365	09/11/2011	67	.	40 - 129		
S285	307	Hepatitis B	Hepatitis B	3	Direct Bilirubin	mg / dl	Day 365	09/11/2011	<0.10	.	0.00 - 0.30		
S285	307	Hepatitis B	Hepatitis B	3	Gamma Glutamyl Transpeptidase	U / L	Day 365	09/11/2011	17	.	10 - 71		
S285	307	Hepatitis B	Hepatitis B	3	Indirect Bilirubin	mg / dl	Day 365	09/11/2011	NA	.	0.00 - 0.99		
S285	307	Hepatitis B	Hepatitis B	3	Total Bilirubin	mg / dl	Day 365	09/11/2011	0.26	.	0.00 - 0.99		
S285	307	Hepatitis B	Hepatitis B	3	Serum creatinine	mg / dl	Day 365	09/11/2011	1.2	.	0.7 - 1.2		
S285	307	Hepatitis B	Hepatitis B	3	Potassium	mEq / L	Day 365	09/11/2011	4.6	.	3.5 - 5.1		
S285	307	Hepatitis B	Hepatitis B	3	Random blood glucose	mg / dl	Day 365	09/11/2011	74	.	45-130		
S285	307	Hepatitis B	Hepatitis B	3	Sodium	mEq / L	Day 365	09/11/2011	140	.	136 - 145		
S291	312	JAIVAC-1	Dosage C		ALT	U / L	Day -14 to Day -1	02/11/2010	30	.	0 - 41		
S291	312	JAIVAC-1	Dosage C		AST	U / L	Day -14 to Day -1	02/11/2010	28	.	0 - 40		
S291	312	JAIVAC-1	Dosage C		Alkaline Phosphatase	U / L	Day -14 to Day -1	02/11/2010	76	.	40 - 129		

Subject No.	Randomisation Number	Study Group	Dosage	Dose	Serum Chemistry Parameters	Unit	Day	Date of sample Collection	Results	Out of Range	Reference Range	Flag	Clinical Significance*
S291	312	JAIVAC-1	Dosage C		Direct Bilirubin	mg / dl	Day -14 to Day -1	02/11/2010	<0.10	.	0.00 - 0.30		
S291	312	JAIVAC-1	Dosage C		Gamma Glutamyl Transpeptidase	U / L	Day -14 to Day -1	02/11/2010	15	.	10 - 71		
S291	312	JAIVAC-1	Dosage C		Indirect Bilirubin	mg / dl	Day -14 to Day -1	02/11/2010	NA	.	0.00 - 0.99		
S291	312	JAIVAC-1	Dosage C		Total Bilirubin	mg / dl	Day -14 to Day -1	02/11/2010	0.41	.	0.00 - 0.99		
S291	312	JAIVAC-1	Dosage C		Serum creatinine	mg / dl	Day -14 to Day -1	02/11/2010	0.8	.	0.7 - 1.2		
S291	312	JAIVAC-1	Dosage C		Potassium	mEq / L	Day -14 to Day -1	02/11/2010	3.9	.	3.5 - 5.1		
S291	312	JAIVAC-1	Dosage C		Random blood glucose	mg / dl	Day -14 to Day -1	02/11/2010	75	.	45-130		
S291	312	JAIVAC-1	Dosage C		Sodium	mEq / L	Day -14 to Day -1	02/11/2010	141	.	136 - 145		
S291	312	JAIVAC-1	Dosage C	2	ALT	U / L	Day 28	10/12/2010	20	.	0 - 41		
S291	312	JAIVAC-1	Dosage C	2	AST	U / L	Day 28	10/12/2010	22	.	0 - 40		
S291	312	JAIVAC-1	Dosage C	2	Alkaline Phosphatase	U / L	Day 28	10/12/2010	67	.	40 - 129		
S291	312	JAIVAC-1	Dosage C	2	Direct Bilirubin	mg / dl	Day 28	10/12/2010	0.13	.	0.00 - 0.30		
S291	312	JAIVAC-1	Dosage C	2	Gamma Glutamyl Transpeptidase	U / L	Day 28	10/12/2010	10	.	10 - 71		
S291	312	JAIVAC-1	Dosage C	2	Indirect Bilirubin	mg / dl	Day 28	10/12/2010	0.4	.	0.00 - 0.99		
S291	312	JAIVAC-1	Dosage C	2	Total Bilirubin	mg / dl	Day 28	10/12/2010	0.53	.	0.00 - 0.99		
S291	312	JAIVAC-1	Dosage C	2	Serum creatinine	mg / dl	Day 28	10/12/2010	0.8	.	0.7 - 1.2		
S291	312	JAIVAC-1	Dosage C	2	Potassium	mEq / L	Day 28	10/12/2010	4.2	.	3.5 - 5.1		
S291	312	JAIVAC-1	Dosage C	2	Random blood glucose	mg / dl	Day 28	10/12/2010	109	.	45-130		
S291	312	JAIVAC-1	Dosage C	2	Sodium	mEq / L	Day 28	10/12/2010	143	.	136 - 145		
S291	312	JAIVAC-1	Dosage C	2	ALT	U / L	Day 56	07/01/2011	21	.	0 - 41		
S291	312	JAIVAC-1	Dosage C	2	AST	U / L	Day 56	07/01/2011	23	.	0 - 40		
S291	312	JAIVAC-1	Dosage C	2	Alkaline Phosphatase	U / L	Day 56	07/01/2011	78	.	40 - 129		
S291	312	JAIVAC-1	Dosage C	2	Direct Bilirubin	mg / dl	Day 56	07/01/2011	0.16	.	0.00 - 0.30		
S291	312	JAIVAC-1	Dosage C	2	Gamma Glutamyl Transpeptidase	U / L	Day 56	07/01/2011	13	.	10 - 71		
S291	312	JAIVAC-1	Dosage C	2	Indirect Bilirubin	mg / dl	Day 56	07/01/2011	0.41	.	0.00 - 0.99		
S291	312	JAIVAC-1	Dosage C	2	Total Bilirubin	mg / dl	Day 56	07/01/2011	0.57	.	0.00 - 0.99		
S291	312	JAIVAC-1	Dosage C	2	Serum creatinine	mg / dl	Day 56	07/01/2011	0.9	.	0.7 - 1.2		
S291	312	JAIVAC-1	Dosage C	2	Potassium	mEq / L	Day 56	07/01/2011	4	.	3.5 - 5.1		
S291	312	JAIVAC-1	Dosage C	2	Random blood glucose	mg / dl	Day 56	07/01/2011	86	.	45-130		

Subject No.	Randomisation Number	Study Group	Dosage	Dose	Serum Chemistry Parameters	Unit	Day	Date of sample Collection	Results	Out of Range	Reference Range	Flag	Clinical Significance*
S291	312	JAIVAC-1	Dosage C	2	Sodium	mEq / L	Day 56	07/01/2011	140	.	136 - 145		
S291	312	JAIVAC-1	Dosage C	3	ALT	U / L	Day 180	11/05/2011	18	.	0 - 41		
S291	312	JAIVAC-1	Dosage C	3	AST	U / L	Day 180	11/05/2011	20	.	0 - 40		
S291	312	JAIVAC-1	Dosage C	3	Alkaline Phosphatase	U / L	Day 180	11/05/2011	73	.	40 - 129		
S291	312	JAIVAC-1	Dosage C	3	Direct Bilirubin	mg / dl	Day 180	11/05/2011	0.16	.	0.00 - 0.30		
S291	312	JAIVAC-1	Dosage C	3	Gamma Glutamyl Transpeptidase	U / L	Day 180	11/05/2011	12	.	10 - 71		
S291	312	JAIVAC-1	Dosage C	3	Indirect Bilirubin	mg / dl	Day 180	11/05/2011	0.44	.	0.00 - 0.99		
S291	312	JAIVAC-1	Dosage C	3	Total Bilirubin	mg / dl	Day 180	11/05/2011	0.6	.	0.00 - 0.99		
S291	312	JAIVAC-1	Dosage C	3	Serum creatinine	mg / dl	Day 180	11/05/2011	1	.	0.7 - 1.2		
S291	312	JAIVAC-1	Dosage C	3	Potassium	mEq / L	Day 180	11/05/2011	4.4	.	3.5 - 5.1		
S291	312	JAIVAC-1	Dosage C	3	Random blood glucose	mg / dl	Day 180	11/05/2011	75	.	45-130		
S291	312	JAIVAC-1	Dosage C	3	Sodium	mEq / L	Day 180	11/05/2011	141	.	136 - 145		
S291	312	JAIVAC-1	Dosage C	3	ALT	U / L	Day 208	08/06/2011	32	.	0 - 41		
S291	312	JAIVAC-1	Dosage C	3	AST	U / L	Day 208	08/06/2011	26	.	0 - 40		
S291	312	JAIVAC-1	Dosage C	3	Alkaline Phosphatase	U / L	Day 208	08/06/2011	65	.	40 - 129		
S291	312	JAIVAC-1	Dosage C	3	Direct Bilirubin	mg / dl	Day 208	08/06/2011	<0.10	.	0.00 - 0.30		
S291	312	JAIVAC-1	Dosage C	3	Gamma Glutamyl Transpeptidase	U / L	Day 208	08/06/2011	15	.	10 - 71		
S291	312	JAIVAC-1	Dosage C	3	Indirect Bilirubin	mg / dl	Day 208	08/06/2011	NA	.	0.00 - 0.99		
S291	312	JAIVAC-1	Dosage C	3	Total Bilirubin	mg / dl	Day 208	08/06/2011	0.44	.	0.00 - 0.99		
S291	312	JAIVAC-1	Dosage C	3	Serum creatinine	mg / dl	Day 208	08/06/2011	0.9	.	0.7 - 1.2		
S291	312	JAIVAC-1	Dosage C	3	Potassium	mEq / L	Day 208	08/06/2011	4	.	3.5 - 5.1		
S291	312	JAIVAC-1	Dosage C	3	Random blood glucose	mg / dl	Day 208	08/06/2011	76	.	45-130		
S291	312	JAIVAC-1	Dosage C	3	Sodium	mEq / L	Day 208	08/06/2011	140	.	136 - 145		
S291	312	JAIVAC-1	Dosage C	3	ALT	U / L	Day 365	10/11/2011	37	.	0 - 41		
S291	312	JAIVAC-1	Dosage C	3	AST	U / L	Day 365	10/11/2011	28	.	0 - 40		
S291	312	JAIVAC-1	Dosage C	3	Alkaline Phosphatase	U / L	Day 365	10/11/2011	66	.	40 - 129		
S291	312	JAIVAC-1	Dosage C	3	Direct Bilirubin	mg / dl	Day 365	10/11/2011	0.15	.	0.00 - 0.30		
S291	312	JAIVAC-1	Dosage C	3	Gamma Glutamyl Transpeptidase	U / L	Day 365	10/11/2011	11	.	10 - 71		
S291	312	JAIVAC-1	Dosage C	3	Indirect Bilirubin	mg / dl	Day 365	10/11/2011	0.38	.	0.00 - 0.99		

Subject No.	Randomisation Number	Study Group	Dosage	Dose	Serum Chemistry Parameters	Unit	Day	Date of sample Collection	Results	Out of Range	Reference Range	Flag	Clinical Significance*
S291	312	JAIVAC-1	Dosage C	3	Total Bilirubin	mg / dl	Day 365	10/11/2011	0.53	.	0.00 - 0.99		
S291	312	JAIVAC-1	Dosage C	3	Serum creatinine	mg / dl	Day 365	10/11/2011	1	.	0.7 - 1.2		
S291	312	JAIVAC-1	Dosage C	3	Potassium	mEq / L	Day 365	10/11/2011	3.9	.	3.5 - 5.1		
S291	312	JAIVAC-1	Dosage C	3	Random blood glucose	mg / dl	Day 365	10/11/2011	88	.	45-130		
S291	312	JAIVAC-1	Dosage C	3	Sodium	mEq / L	Day 365	10/11/2011	140	.	136 - 145		
S298	314	JAIVAC-1	Dosage C		ALT	U / L	Day -14 to Day -1	09/11/2010	22	.	0 - 41		
S298	314	JAIVAC-1	Dosage C		AST	U / L	Day -14 to Day -1	09/11/2010	26	.	0 - 40		
S298	314	JAIVAC-1	Dosage C		Alkaline Phosphatase	U / L	Day -14 to Day -1	09/11/2010	69	.	40 - 129		
S298	314	JAIVAC-1	Dosage C		Direct Bilirubin	mg / dl	Day -14 to Day -1	09/11/2010	0.21	.	0.00 - 0.30		
S298	314	JAIVAC-1	Dosage C		Gamma Glutamyl Transpeptidase	U / L	Day -14 to Day -1	09/11/2010	28	.	10 - 71		
S298	314	JAIVAC-1	Dosage C		Indirect Bilirubin	mg / dl	Day -14 to Day -1	09/11/2010	0.51	.	0.00 - 0.99		
S298	314	JAIVAC-1	Dosage C		Total Bilirubin	mg / dl	Day -14 to Day -1	09/11/2010	0.72	.	0.00 - 0.99		
S298	314	JAIVAC-1	Dosage C		Serum creatinine	mg / dl	Day -14 to Day -1	09/11/2010	1	.	0.7 - 1.2		
S298	314	JAIVAC-1	Dosage C		Potassium	mEq / L	Day -14 to Day -1	09/11/2010	4.6	.	3.5 - 5.1		
S298	314	JAIVAC-1	Dosage C		Random blood glucose	mg / dl	Day -14 to Day -1	09/11/2010	97	.	45-130		
S298	314	JAIVAC-1	Dosage C		Sodium	mEq / L	Day -14 to Day -1	09/11/2010	139	.	136 - 145		
S298	314	JAIVAC-1	Dosage C	2	ALT	U / L	Day 28	10/12/2010	24	.	0 - 41		
S298	314	JAIVAC-1	Dosage C	2	AST	U / L	Day 28	10/12/2010	26	.	0 - 40		
S298	314	JAIVAC-1	Dosage C	2	Alkaline Phosphatase	U / L	Day 28	10/12/2010	83	.	40 - 129		
S298	314	JAIVAC-1	Dosage C	2	Direct Bilirubin	mg / dl	Day 28	10/12/2010	0.23	.	0.00 - 0.30		
S298	314	JAIVAC-1	Dosage C	2	Gamma Glutamyl Transpeptidase	U / L	Day 28	10/12/2010	23	.	10 - 71		
S298	314	JAIVAC-1	Dosage C	2	Indirect Bilirubin	mg / dl	Day 28	10/12/2010	0.4	.	0.00 - 0.99		
S298	314	JAIVAC-1	Dosage C	2	Total Bilirubin	mg / dl	Day 28	10/12/2010	0.63	.	0.00 - 0.99		
S298	314	JAIVAC-1	Dosage C	2	Serum creatinine	mg / dl	Day 28	10/12/2010	1	.	0.7 - 1.2		
S298	314	JAIVAC-1	Dosage C	2	Potassium	mEq / L	Day 28	10/12/2010	4.5	.	3.5 - 5.1		
S298	314	JAIVAC-1	Dosage C	2	Random blood glucose	mg / dl	Day 28	10/12/2010	131	Yes	45-130	H	NCS
S298	314	JAIVAC-1	Dosage C	2	Sodium	mEq / L	Day 28	10/12/2010	140	.	136 - 145		
S298	314	JAIVAC-1	Dosage C	2	ALT	U / L	Day 56	07/01/2011	24	.	0 - 41		
S298	314	JAIVAC-1	Dosage C	2	AST	U / L	Day 56	07/01/2011	28	.	0 - 40		

Subject No.	Randomisation Number	Study Group	Dosage	Dose	Serum Chemistry Parameters	Unit	Day	Date of sample Collection	Results	Out of Range	Reference Range	Flag	Clinical Significance*
S298	314	JAIVAC-1	Dosage C	2	Alkaline Phosphatase	U / L	Day 56	07/01/2011	78	.	40 - 129		
S298	314	JAIVAC-1	Dosage C	2	Direct Bilirubin	mg / dl	Day 56	07/01/2011	0.29	.	0.00 - 0.30		
S298	314	JAIVAC-1	Dosage C	2	Gamma Glutamyl Transpeptidase	U / L	Day 56	07/01/2011	29	.	10 - 71		
S298	314	JAIVAC-1	Dosage C	2	Indirect Bilirubin	mg / dl	Day 56	07/01/2011	0.65	.	0.00 - 0.99		
S298	314	JAIVAC-1	Dosage C	2	Total Bilirubin	mg / dl	Day 56	07/01/2011	0.94	.	0.00 - 0.99		
S298	314	JAIVAC-1	Dosage C	2	Serum creatinine	mg / dl	Day 56	07/01/2011	1.2	.	0.7 - 1.2		
S298	314	JAIVAC-1	Dosage C	2	Potassium	mEq / L	Day 56	07/01/2011	5	.	3.5 - 5.1		
S298	314	JAIVAC-1	Dosage C	2	Random blood glucose	mg / dl	Day 56	07/01/2011	110	.	45-130		
S298	314	JAIVAC-1	Dosage C	2	Sodium	mEq / L	Day 56	07/01/2011	139	.	136 - 145		
S298	314	JAIVAC-1	Dosage C	3	ALT	U / L	Day 180	11/05/2011	21	.	0 - 41		
S298	314	JAIVAC-1	Dosage C	3	AST	U / L	Day 180	11/05/2011	23	.	0 - 40		
S298	314	JAIVAC-1	Dosage C	3	Alkaline Phosphatase	U / L	Day 180	11/05/2011	64	.	40 - 129		
S298	314	JAIVAC-1	Dosage C	3	Direct Bilirubin	mg / dl	Day 180	11/05/2011	0.22	.	0.00 - 0.30		
S298	314	JAIVAC-1	Dosage C	3	Gamma Glutamyl Transpeptidase	U / L	Day 180	11/05/2011	30	.	10 - 71		
S298	314	JAIVAC-1	Dosage C	3	Indirect Bilirubin	mg / dl	Day 180	11/05/2011	0.44	.	0.00 - 0.99		
S298	314	JAIVAC-1	Dosage C	3	Total Bilirubin	mg / dl	Day 180	11/05/2011	0.66	.	0.00 - 0.99		
S298	314	JAIVAC-1	Dosage C	3	Serum creatinine	mg / dl	Day 180	11/05/2011	1.2	.	0.7 - 1.2		
S298	314	JAIVAC-1	Dosage C	3	Potassium	mEq / L	Day 180	11/05/2011	5.4	Yes	3.5 - 5.1	H	CS
S298	314	JAIVAC-1	Dosage C	3	Random blood glucose	mg / dl	Day 180	11/05/2011	87	.	45-130		
S298	314	JAIVAC-1	Dosage C	3	Sodium	mEq / L	Day 180	11/05/2011	141	.	136 - 145		
S298	314	JAIVAC-1	Dosage C	3	ALT	U / L	Day 208	08/06/2011	20	.	0 - 41		
S298	314	JAIVAC-1	Dosage C	3	AST	U / L	Day 208	08/06/2011	26	.	0 - 40		
S298	314	JAIVAC-1	Dosage C	3	Alkaline Phosphatase	U / L	Day 208	08/06/2011	77	.	40 - 129		
S298	314	JAIVAC-1	Dosage C	3	Direct Bilirubin	mg / dl	Day 208	08/06/2011	0.23	.	0.00 - 0.30		
S298	314	JAIVAC-1	Dosage C	3	Gamma Glutamyl Transpeptidase	U / L	Day 208	08/06/2011	27	.	10 - 71		
S298	314	JAIVAC-1	Dosage C	3	Indirect Bilirubin	mg / dl	Day 208	08/06/2011	0.69	.	0.00 - 0.99		
S298	314	JAIVAC-1	Dosage C	3	Total Bilirubin	mg / dl	Day 208	08/06/2011	0.92	.	0.00 - 0.99		
S298	314	JAIVAC-1	Dosage C	3	Serum creatinine	mg / dl	Day 208	08/06/2011	1.2	.	0.7 - 1.2		
S298	314	JAIVAC-1	Dosage C	3	Potassium	mEq / L	Day 208	08/06/2011	4.4	.	3.5 - 5.1		

Subject No.	Randomisation Number	Study Group	Dosage	Dose	Serum Chemistry Parameters	Unit	Day	Date of sample Collection	Results	Out of Range	Reference Range	Flag	Clinical Significance*
S298	314	JAIVAC-1	Dosage C	3	Random blood glucose	mg / dl	Day 208	08/06/2011	116	.	45-130		
S298	314	JAIVAC-1	Dosage C	3	Sodium	mEq / L	Day 208	08/06/2011	139	.	136 - 145		
S298	314	JAIVAC-1	Dosage C	3	ALT	U / L	Day 365	10/11/2011	25	.	0 - 41		
S298	314	JAIVAC-1	Dosage C	3	AST	U / L	Day 365	10/11/2011	27	.	0 - 40		
S298	314	JAIVAC-1	Dosage C	3	Alkaline Phosphatase	U / L	Day 365	10/11/2011	54	.	40 - 129		
S298	314	JAIVAC-1	Dosage C	3	Direct Bilirubin	mg / dl	Day 365	10/11/2011	0.25	.	0.00 - 0.30		
S298	314	JAIVAC-1	Dosage C	3	Gamma Glutamyl Transpeptidase	U / L	Day 365	10/11/2011	38	.	10 - 71		
S298	314	JAIVAC-1	Dosage C	3	Indirect Bilirubin	mg / dl	Day 365	10/11/2011	0.47	.	0.00 - 0.99		
S298	314	JAIVAC-1	Dosage C	3	Total Bilirubin	mg / dl	Day 365	10/11/2011	0.72	.	0.00 - 0.99		
S298	314	JAIVAC-1	Dosage C	3	Serum creatinine	mg / dl	Day 365	10/11/2011	1.3	Yes	0.7 - 1.2	H	CS
S298	314	JAIVAC-1	Dosage C	3	Potassium	mEq / L	Day 365	10/11/2011	5.5	Yes	3.5 - 5.1	H	CS
S298	314	JAIVAC-1	Dosage C	3	Random blood glucose	mg / dl	Day 365	10/11/2011	100	.	45-130		
S298	314	JAIVAC-1	Dosage C	3	Sodium	mEq / L	Day 365	10/11/2011	143	.	136 - 145		
S299	310	JAIVAC-1	Dosage C		ALT	U / L	Day -14 to Day -1	08/11/2010	12	.	0 - 41		
S299	310	JAIVAC-1	Dosage C		AST	U / L	Day -14 to Day -1	08/11/2010	20	.	0 - 40		
S299	310	JAIVAC-1	Dosage C		Alkaline Phosphatase	U / L	Day -14 to Day -1	08/11/2010	98	.	40 - 129		
S299	310	JAIVAC-1	Dosage C		Direct Bilirubin	mg / dl	Day -14 to Day -1	08/11/2010	0.18	.	0.00 - 0.30		
S299	310	JAIVAC-1	Dosage C		Gamma Glutamyl Transpeptidase	U / L	Day -14 to Day -1	08/11/2010	10	.	10 - 71		
S299	310	JAIVAC-1	Dosage C		Indirect Bilirubin	mg / dl	Day -14 to Day -1	08/11/2010	0.36	.	0.00 - 0.99		
S299	310	JAIVAC-1	Dosage C		Total Bilirubin	mg / dl	Day -14 to Day -1	08/11/2010	0.54	.	0.00 - 0.99		
S299	310	JAIVAC-1	Dosage C		Serum creatinine	mg / dl	Day -14 to Day -1	08/11/2010	0.9	.	0.7 - 1.2		
S299	310	JAIVAC-1	Dosage C		Potassium	mEq / L	Day -14 to Day -1	08/11/2010	4.6	.	3.5 - 5.1		
S299	310	JAIVAC-1	Dosage C		Random blood glucose	mg / dl	Day -14 to Day -1	08/11/2010	99	.	45-130		
S299	310	JAIVAC-1	Dosage C		Sodium	mEq / L	Day -14 to Day -1	08/11/2010	141	.	136 - 145		
S299	310	JAIVAC-1	Dosage C	2	ALT	U / L	Day 28	10/12/2010	14	.	0 - 41		
S299	310	JAIVAC-1	Dosage C	2	AST	U / L	Day 28	10/12/2010	21	.	0 - 40		
S299	310	JAIVAC-1	Dosage C	2	Alkaline Phosphatase	U / L	Day 28	10/12/2010	84	.	40 - 129		
S299	310	JAIVAC-1	Dosage C	2	Direct Bilirubin	mg / dl	Day 28	10/12/2010	0.19	.	0.00 - 0.30		
S299	310	JAIVAC-1	Dosage C	2	Gamma Glutamyl Transpeptidase	U / L	Day 28	10/12/2010	10	.	10 - 71		

Subject No.	Randomisation Number	Study Group	Dosage	Dose	Serum Chemistry Parameters	Unit	Day	Date of sample Collection	Results	Out of Range	Reference Range	Flag	Clinical Significance*
S299	310	JAIVAC-1	Dosage C	2	Indirect Bilirubin	mg / dl	Day 28	10/12/2010	0.57	.	0.00 - 0.99		
S299	310	JAIVAC-1	Dosage C	2	Total Bilirubin	mg / dl	Day 28	10/12/2010	0.76	.	0.00 - 0.99		
S299	310	JAIVAC-1	Dosage C	2	Serum creatinine	mg / dl	Day 28	10/12/2010	0.9	.	0.7 - 1.2		
S299	310	JAIVAC-1	Dosage C	2	Potassium	mEq / L	Day 28	10/12/2010	4.2	.	3.5 - 5.1		
S299	310	JAIVAC-1	Dosage C	2	Random blood glucose	mg / dl	Day 28	10/12/2010	118	.	45-130		
S299	310	JAIVAC-1	Dosage C	2	Sodium	mEq / L	Day 28	10/12/2010	137	.	136 - 145		
S299	310	JAIVAC-1	Dosage C	2	ALT	U / L	Day 56	07/01/2011	15	.	0 - 41		
S299	310	JAIVAC-1	Dosage C	2	AST	U / L	Day 56	07/01/2011	22	.	0 - 40		
S299	310	JAIVAC-1	Dosage C	2	Alkaline Phosphatase	U / L	Day 56	07/01/2011	99	.	40 - 129		
S299	310	JAIVAC-1	Dosage C	2	Direct Bilirubin	mg / dl	Day 56	07/01/2011	0.19	.	0.00 - 0.30		
S299	310	JAIVAC-1	Dosage C	2	Gamma Glutamyl Transpeptidase	U / L	Day 56	07/01/2011	11	.	10 - 71		
S299	310	JAIVAC-1	Dosage C	2	Indirect Bilirubin	mg / dl	Day 56	07/01/2011	0.43	.	0.00 - 0.99		
S299	310	JAIVAC-1	Dosage C	2	Total Bilirubin	mg / dl	Day 56	07/01/2011	0.62	.	0.00 - 0.99		
S299	310	JAIVAC-1	Dosage C	2	Serum creatinine	mg / dl	Day 56	07/01/2011	1	.	0.7 - 1.2		
S299	310	JAIVAC-1	Dosage C	2	Potassium	mEq / L	Day 56	07/01/2011	4.3	.	3.5 - 5.1		
S299	310	JAIVAC-1	Dosage C	2	Random blood glucose	mg / dl	Day 56	07/01/2011	91	.	45-130		
S299	310	JAIVAC-1	Dosage C	2	Sodium	mEq / L	Day 56	07/01/2011	137	.	136 - 145		
S299	310	JAIVAC-1	Dosage C	3	ALT	U / L	Day 180	11/05/2011	13	.	0 - 41		
S299	310	JAIVAC-1	Dosage C	3	AST	U / L	Day 180	11/05/2011	19	.	0 - 40		
S299	310	JAIVAC-1	Dosage C	3	Alkaline Phosphatase	U / L	Day 180	11/05/2011	91	.	40 - 129		
S299	310	JAIVAC-1	Dosage C	3	Direct Bilirubin	mg / dl	Day 180	11/05/2011	0.19	.	0.00 - 0.30		
S299	310	JAIVAC-1	Dosage C	3	Gamma Glutamyl Transpeptidase	U / L	Day 180	11/05/2011	10	.	10 - 71		
S299	310	JAIVAC-1	Dosage C	3	Indirect Bilirubin	mg / dl	Day 180	11/05/2011	0.32	.	0.00 - 0.99		
S299	310	JAIVAC-1	Dosage C	3	Total Bilirubin	mg / dl	Day 180	11/05/2011	0.51	.	0.00 - 0.99		
S299	310	JAIVAC-1	Dosage C	3	Serum creatinine	mg / dl	Day 180	11/05/2011	1.2	.	0.7 - 1.2		
S299	310	JAIVAC-1	Dosage C	3	Potassium	mEq / L	Day 180	11/05/2011	5	.	3.5 - 5.1		
S299	310	JAIVAC-1	Dosage C	3	Random blood glucose	mg / dl	Day 180	11/05/2011	72	.	45-130		
S299	310	JAIVAC-1	Dosage C	3	Sodium	mEq / L	Day 180	11/05/2011	140	.	136 - 145		
S299	310	JAIVAC-1	Dosage C	3	ALT	U / L	Day 208	08/06/2011	15	.	0 - 41		

Subject No.	Randomisation Number	Study Group	Dosage	Dose	Serum Chemistry Parameters	Unit	Day	Date of sample Collection	Results	Out of Range	Reference Range	Flag	Clinical Significance*
S299	310	JAIVAC-1	Dosage C	3	AST	U / L	Day 208	08/06/2011	21	.	0 - 40		
S299	310	JAIVAC-1	Dosage C	3	Alkaline Phosphatase	U / L	Day 208	08/06/2011	89	.	40 - 129		
S299	310	JAIVAC-1	Dosage C	3	Direct Bilirubin	mg / dl	Day 208	08/06/2011	0.15	.	0.00 - 0.30		
S299	310	JAIVAC-1	Dosage C	3	Gamma Glutamyl Transpeptidase	U / L	Day 208	08/06/2011	11	.	10 - 71		
S299	310	JAIVAC-1	Dosage C	3	Indirect Bilirubin	mg / dl	Day 208	08/06/2011	0.47	.	0.00 - 0.99		
S299	310	JAIVAC-1	Dosage C	3	Total Bilirubin	mg / dl	Day 208	08/06/2011	0.62	.	0.00 - 0.99		
S299	310	JAIVAC-1	Dosage C	3	Serum creatinine	mg / dl	Day 208	08/06/2011	1	.	0.7 - 1.2		
S299	310	JAIVAC-1	Dosage C	3	Potassium	mEq / L	Day 208	08/06/2011	5	.	3.5 - 5.1		
S299	310	JAIVAC-1	Dosage C	3	Random blood glucose	mg / dl	Day 208	08/06/2011	85	.	45-130		
S299	310	JAIVAC-1	Dosage C	3	Sodium	mEq / L	Day 208	08/06/2011	139	.	136 - 145		
S299	310	JAIVAC-1	Dosage C	3	ALT	U / L	Day 365	10/11/2011	17	.	0 - 41		
S299	310	JAIVAC-1	Dosage C	3	AST	U / L	Day 365	10/11/2011	19	.	0 - 40		
S299	310	JAIVAC-1	Dosage C	3	Alkaline Phosphatase	U / L	Day 365	10/11/2011	88	.	40 - 129		
S299	310	JAIVAC-1	Dosage C	3	Direct Bilirubin	mg / dl	Day 365	10/11/2011	0.19	.	0.00 - 0.30		
S299	310	JAIVAC-1	Dosage C	3	Gamma Glutamyl Transpeptidase	U / L	Day 365	10/11/2011	11	.	10 - 71		
S299	310	JAIVAC-1	Dosage C	3	Indirect Bilirubin	mg / dl	Day 365	10/11/2011	0.43	.	0.00 - 0.99		
S299	310	JAIVAC-1	Dosage C	3	Total Bilirubin	mg / dl	Day 365	10/11/2011	0.62	.	0.00 - 0.99		
S299	310	JAIVAC-1	Dosage C	3	Serum creatinine	mg / dl	Day 365	10/11/2011	1.3	Yes	0.7 - 1.2	H	CS
S299	310	JAIVAC-1	Dosage C	3	Potassium	mEq / L	Day 365	10/11/2011	4.8	.	3.5 - 5.1		
S299	310	JAIVAC-1	Dosage C	3	Random blood glucose	mg / dl	Day 365	10/11/2011	103	.	45-130		
S299	310	JAIVAC-1	Dosage C	3	Sodium	mEq / L	Day 365	10/11/2011	142	.	136 - 145		
S124	106	Hepatitis B	Hepatitis B		Serum Ferritin	ng/ml	Unscheduled	21/10/2010	4		28 – 397	L	CS
S124	106	Hepatitis B	Hepatitis B		Serum Iron	microgr am/dl	Unscheduled	21/10/2010	35		49 – 181	L	CS
S124	106	Hepatitis B	Hepatitis B		TIBC	microgr am/dl	Unscheduled	21/10/2010	346		250 - 450		
S263	313	Hepatitis B	Hepatitis B		AST	U / L	Unscheduled	17/12/2010	23	.	0 - 40		
S263	313	Hepatitis B	Hepatitis B		Direct Bilirubin	mg / dl	Unscheduled	17/12/2010	0.2	.	0.00 - 0.30		
S263	313	Hepatitis B	Hepatitis B		Indirect Bilirubin	mg / dl	Unscheduled	17/12/2010	0.33	.	0.00 - 0.99		
S263	313	Hepatitis B	Hepatitis B		BUN	mg/dl	Unscheduled	14/02/2011	7	.	6 - 20		

Subject No.	Randomisation Number	Study Group	Dosage	Dose	Serum Chemistry Parameters	Unit	Day	Date of sample Collection	Results	Out of Range	Reference Range	Flag	Clinical Significance*
S263	313	Hepatitis B	Hepatitis B		Serum creatinine	mg / dl	Unscheduled	14/02/2011	0.9	.	0.7 - 1.2		
S263	313	Hepatitis B	Hepatitis B		Potassium	mEq / L	Unscheduled	14/02/2011	4.3	.	3.5 - 5.1		
S266	308	JAIVAC-1	Dosage C		BUN	mg/dl	Unscheduled	27/01/2011	11		6 - 20		
S266	308	JAIVAC-1	Dosage C		Serum creatinine	mg / dl	Unscheduled	27/01/2011	0.8	.	0.7 - 1.2		
S266	308	JAIVAC-1	Dosage C		Potassium	mEq / L	Unscheduled	27/01/2011	5	.	3.5 - 5.1		
S267	309	Hepatitis B	Hepatitis B		BUN	mg/dl	Unscheduled	27/01/2011	13	.	6 - 20		
S267	309	Hepatitis B	Hepatitis B		Serum creatinine	mg / dl	Unscheduled	27/01/2011	1.3	.	0.7 - 1.2	H	NCS

Note:
1) Flag: L- Below Range, N- Within Range, H- Above Range
2) Clinical Significance: CS-Clinically Significant, NCS-Not Clinically Significant
3) At Day -14 to Day -1, as per central Laboratory reference range even if the the value is out of range (H/L) but as per protocol specified reference range related to exclusion criteria the subject is eligible and Randomised and hence CS /NCS not marked for Total Bilirubin, Direct Bilirubin and Gamma Glutamyl Transpeptidase at Screening.

16.2.8.4: Listing of Clinical Laboratory Evaluations – Urinalysis

Subject No.	Randomisation Number	Study Group	Dosage	Dose	Urine Parameters	Day	Date of sample Collection	Results
S108	115	Hepatitis B	Hepatitis B		Benzodiazepines	Day -14 to Day -1	18/08/2010	Negative
S108	115	Hepatitis B	Hepatitis B		Opioids	Day -14 to Day -1	18/08/2010	Negative
S108	115	Hepatitis B	Hepatitis B		Amphetamine	Day -14 to Day -1	18/08/2010	Negative
S108	115	Hepatitis B	Hepatitis B		Cocaine	Day -14 to Day -1	18/08/2010	Negative
S108	115	Hepatitis B	Hepatitis B		THC	Day -14 to Day -1	18/08/2010	Negative
S108	115	Hepatitis B	Hepatitis B	2	Benzodiazepines	Day 28	24/09/2010	Negative
S108	115	Hepatitis B	Hepatitis B	2	Opioids	Day 28	24/09/2010	Negative
S108	115	Hepatitis B	Hepatitis B	2	Amphetamine	Day 28	24/09/2010	Negative
S108	115	Hepatitis B	Hepatitis B	2	Cocaine	Day 28	24/09/2010	Negative
S108	115	Hepatitis B	Hepatitis B	2	THC	Day 28	24/09/2010	Negative
S108	115	Hepatitis B	Hepatitis B	3	Benzodiazepines	Day 180	23/02/2011	Negative
S108	115	Hepatitis B	Hepatitis B	3	Opioids	Day 180	23/02/2011	Negative
S108	115	Hepatitis B	Hepatitis B	3	Amphetamine	Day 180	23/02/2011	Negative
S108	115	Hepatitis B	Hepatitis B	3	Cocaine	Day 180	23/02/2011	Negative
S108	115	Hepatitis B	Hepatitis B	3	THC	Day 180	23/02/2011	Negative
S108	115	Hepatitis B	Hepatitis B	3	Benzodiazepines	Day 365	26/08/2011	Negative
S108	115	Hepatitis B	Hepatitis B	3	Opioids	Day 365	26/08/2011	Negative
S108	115	Hepatitis B	Hepatitis B	3	Amphetamine	Day 365	26/08/2011	Negative
S108	115	Hepatitis B	Hepatitis B	3	Cocaine	Day 365	26/08/2011	Negative
S108	115	Hepatitis B	Hepatitis B	3	THC	Day 365	26/08/2011	Negative
S108	115	Hepatitis B	Hepatitis B		Benzodiazepines	Unscheduled Visit	.	
S108	115	Hepatitis B	Hepatitis B		Opioids	Unscheduled Visit	.	
S108	115	Hepatitis B	Hepatitis B		Amphetamine	Unscheduled Visit	.	
S108	115	Hepatitis B	Hepatitis B		Cocaine	Unscheduled Visit	.	
S108	115	Hepatitis B	Hepatitis B		THC	Unscheduled Visit	.	
S108	115	Hepatitis B	Hepatitis B		Benzodiazepines	Unscheduled Visit	.	
S108	115	Hepatitis B	Hepatitis B		Opioids	Unscheduled Visit	.	
S108	115	Hepatitis B	Hepatitis B		Amphetamine	Unscheduled Visit	.	

Subject No.	Randomisation Number	Study Group	Dosage	Dose	Urine Parameters	Day	Date of sample Collection	Results
S108	115	Hepatitis B	Hepatitis B		Cocaine	Unscheduled Visit	.	
S108	115	Hepatitis B	Hepatitis B		THC	Unscheduled Visit	.	
S108	115	Hepatitis B	Hepatitis B		Benzodiazepines	Unscheduled Visit	.	
S108	115	Hepatitis B	Hepatitis B		Opioids	Unscheduled Visit	.	
S108	115	Hepatitis B	Hepatitis B		Amphetamine	Unscheduled Visit	.	
S108	115	Hepatitis B	Hepatitis B		Cocaine	Unscheduled Visit	.	
S108	115	Hepatitis B	Hepatitis B		THC	Unscheduled Visit	.	
S118	114	JAIVAC-1	Dosage A		Benzodiazepines	Day -14 to Day -1	17/08/2010	Negative
S118	114	JAIVAC-1	Dosage A		Opioids	Day -14 to Day -1	17/08/2010	Negative
S118	114	JAIVAC-1	Dosage A		Amphetamine	Day -14 to Day -1	17/08/2010	Negative
S118	114	JAIVAC-1	Dosage A		Cocaine	Day -14 to Day -1	17/08/2010	Negative
S118	114	JAIVAC-1	Dosage A		THC	Day -14 to Day -1	17/08/2010	Negative
S118	114	JAIVAC-1	Dosage A	2	Benzodiazepines	Day 28	24/09/2010	Negative
S118	114	JAIVAC-1	Dosage A	2	Opioids	Day 28	24/09/2010	Negative
S118	114	JAIVAC-1	Dosage A	2	Amphetamine	Day 28	24/09/2010	Negative
S118	114	JAIVAC-1	Dosage A	2	Cocaine	Day 28	24/09/2010	Negative
S118	114	JAIVAC-1	Dosage A	2	THC	Day 28	24/09/2010	Negative
S118	114	JAIVAC-1	Dosage A	3	Benzodiazepines	Day 180	23/02/2011	Negative
S118	114	JAIVAC-1	Dosage A	3	Opioids	Day 180	23/02/2011	Negative
S118	114	JAIVAC-1	Dosage A	3	Amphetamine	Day 180	23/02/2011	Negative
S118	114	JAIVAC-1	Dosage A	3	Cocaine	Day 180	23/02/2011	Negative
S118	114	JAIVAC-1	Dosage A	3	THC	Day 180	23/02/2011	Negative
S118	114	JAIVAC-1	Dosage A	3	Benzodiazepines	Day 365	26/08/2011	Negative
S118	114	JAIVAC-1	Dosage A	3	Opioids	Day 365	26/08/2011	Negative
S118	114	JAIVAC-1	Dosage A	3	Amphetamine	Day 365	26/08/2011	Negative
S118	114	JAIVAC-1	Dosage A	3	Cocaine	Day 365	26/08/2011	Negative
S118	114	JAIVAC-1	Dosage A	3	THC	Day 365	26/08/2011	Negative
S119	107	Hepatitis B	Hepatitis B		Benzodiazepines	Day -14 to Day -1	18/08/2010	Negative
S119	107	Hepatitis B	Hepatitis B		Opioids	Day -14 to Day -1	18/08/2010	Negative
S119	107	Hepatitis B	Hepatitis B		Amphetamine	Day -14 to Day -1	18/08/2010	Negative

Subject No.	Randomisation Number	Study Group	Dosage	Dose	Urine Parameters	Day	Date of sample Collection	Results
S119	107	Hepatitis B	Hepatitis B		Cocaine	Day -14 to Day -1	18/08/2010	Negative
S119	107	Hepatitis B	Hepatitis B		THC	Day -14 to Day -1	18/08/2010	Negative
S119	107	Hepatitis B	Hepatitis B	2	Benzodiazepines	Day 28	23/09/2010	Negative
S119	107	Hepatitis B	Hepatitis B	2	Opioids	Day 28	23/09/2010	Negative
S119	107	Hepatitis B	Hepatitis B	2	Amphetamine	Day 28	23/09/2010	Negative
S119	107	Hepatitis B	Hepatitis B	2	Cocaine	Day 28	23/09/2010	Negative
S119	107	Hepatitis B	Hepatitis B	2	THC	Day 28	23/09/2010	Negative
S119	107	Hepatitis B	Hepatitis B	3	Benzodiazepines	Day 180	22/02/2011	Negative
S119	107	Hepatitis B	Hepatitis B	3	Opioids	Day 180	22/02/2011	Negative
S119	107	Hepatitis B	Hepatitis B	3	Amphetamine	Day 180	22/02/2011	Negative
S119	107	Hepatitis B	Hepatitis B	3	Cocaine	Day 180	22/02/2011	Negative
S119	107	Hepatitis B	Hepatitis B	3	THC	Day 180	22/02/2011	Negative
S119	107	Hepatitis B	Hepatitis B	3	Benzodiazepines	Day 365	25/08/2011	Negative
S119	107	Hepatitis B	Hepatitis B	3	Opioids	Day 365	25/08/2011	Negative
S119	107	Hepatitis B	Hepatitis B	3	Amphetamine	Day 365	25/08/2011	Negative
S119	107	Hepatitis B	Hepatitis B	3	Cocaine	Day 365	25/08/2011	Negative
S119	107	Hepatitis B	Hepatitis B	3	THC	Day 365	25/08/2011	Negative
S120	104	JAIVAC-1	Dosage A		Benzodiazepines	Day -14 to Day -1	18/08/2010	Negative
S120	104	JAIVAC-1	Dosage A		Opioids	Day -14 to Day -1	18/08/2010	Negative
S120	104	JAIVAC-1	Dosage A		Amphetamine	Day -14 to Day -1	18/08/2010	Negative
S120	104	JAIVAC-1	Dosage A		Cocaine	Day -14 to Day -1	18/08/2010	Negative
S120	104	JAIVAC-1	Dosage A		THC	Day -14 to Day -1	18/08/2010	Negative
S120	104	JAIVAC-1	Dosage A	2	Benzodiazepines	Day 28	23/09/2010	Negative
S120	104	JAIVAC-1	Dosage A	2	Opioids	Day 28	23/09/2010	Negative
S120	104	JAIVAC-1	Dosage A	2	Amphetamine	Day 28	23/09/2010	Negative
S120	104	JAIVAC-1	Dosage A	2	Cocaine	Day 28	23/09/2010	Negative
S120	104	JAIVAC-1	Dosage A	2	THC	Day 28	23/09/2010	Negative
S120	104	JAIVAC-1	Dosage A	3	Benzodiazepines	Day 180	22/02/2011	Negative
S120	104	JAIVAC-1	Dosage A	3	Opioids	Day 180	22/02/2011	Negative
S120	104	JAIVAC-1	Dosage A	3	Amphetamine	Day 180	22/02/2011	Negative

Subject No.	Randomisation Number	Study Group	Dosage	Dose	Urine Parameters	Day	Date of sample Collection	Results
S120	104	JAIVAC-1	Dosage A	3	Cocaine	Day 180	22/02/2011	Negative
S120	104	JAIVAC-1	Dosage A	3	THC	Day 180	22/02/2011	Negative
S120	104	JAIVAC-1	Dosage A	3	Benzodiazepines	Day 365	25/08/2011	Negative
S120	104	JAIVAC-1	Dosage A	3	Opioids	Day 365	25/08/2011	Negative
S120	104	JAIVAC-1	Dosage A	3	Amphetamine	Day 365	25/08/2011	Negative
S120	104	JAIVAC-1	Dosage A	3	Cocaine	Day 365	25/08/2011	Negative
S120	104	JAIVAC-1	Dosage A	3	THC	Day 365	25/08/2011	Negative
S120	104	JAIVAC-1	Dosage A		Benzodiazepines	Unscheduled Visit	.	
S120	104	JAIVAC-1	Dosage A		Opioids	Unscheduled Visit	.	
S120	104	JAIVAC-1	Dosage A		Amphetamine	Unscheduled Visit	.	
S120	104	JAIVAC-1	Dosage A		Cocaine	Unscheduled Visit	.	
S120	104	JAIVAC-1	Dosage A		THC	Unscheduled Visit	.	
S124	106	Hepatitis B	Hepatitis B		Benzodiazepines	Day -14 to Day -1	18/08/2010	Negative
S124	106	Hepatitis B	Hepatitis B		Opioids	Day -14 to Day -1	18/08/2010	Negative
S124	106	Hepatitis B	Hepatitis B		Amphetamine	Day -14 to Day -1	18/08/2010	Negative
S124	106	Hepatitis B	Hepatitis B		Cocaine	Day -14 to Day -1	18/08/2010	Negative
S124	106	Hepatitis B	Hepatitis B		THC	Day -14 to Day -1	18/08/2010	Negative
S124	106	Hepatitis B	Hepatitis B	2	Benzodiazepines	Day 28	23/09/2010	Negative
S124	106	Hepatitis B	Hepatitis B	2	Opioids	Day 28	23/09/2010	Negative
S124	106	Hepatitis B	Hepatitis B	2	Amphetamine	Day 28	23/09/2010	Negative
S124	106	Hepatitis B	Hepatitis B	2	Cocaine	Day 28	23/09/2010	Negative
S124	106	Hepatitis B	Hepatitis B	2	THC	Day 28	23/09/2010	Negative
S124	106	Hepatitis B	Hepatitis B	3	Benzodiazepines	Day 180	22/02/2011	Negative
S124	106	Hepatitis B	Hepatitis B	3	Opioids	Day 180	22/02/2011	Negative
S124	106	Hepatitis B	Hepatitis B	3	Amphetamine	Day 180	22/02/2011	Negative
S124	106	Hepatitis B	Hepatitis B	3	Cocaine	Day 180	22/02/2011	Negative
S124	106	Hepatitis B	Hepatitis B	3	THC	Day 180	22/02/2011	Negative
S124	106	Hepatitis B	Hepatitis B	3	Benzodiazepines	Day 365	25/08/2011	Negative
S124	106	Hepatitis B	Hepatitis B	3	Opioids	Day 365	25/08/2011	Negative
S124	106	Hepatitis B	Hepatitis B	3	Amphetamine	Day 365	25/08/2011	Negative

Subject No.	Randomisation Number	Study Group	Dosage	Dose	Urine Parameters	Day	Date of sample Collection	Results
S124	106	Hepatitis B	Hepatitis B	3	Cocaine	Day 365	25/08/2011	Negative
S124	106	Hepatitis B	Hepatitis B	3	THC	Day 365	25/08/2011	Negative
S124	106	Hepatitis B	Hepatitis B		Benzodiazepines	Unscheduled Visit	.	
S124	106	Hepatitis B	Hepatitis B		Opioids	Unscheduled Visit	.	
S124	106	Hepatitis B	Hepatitis B		Amphetamine	Unscheduled Visit	.	
S124	106	Hepatitis B	Hepatitis B		Cocaine	Unscheduled Visit	.	
S124	106	Hepatitis B	Hepatitis B		THC	Unscheduled Visit	.	
S124	106	Hepatitis B	Hepatitis B		Benzodiazepines	Unscheduled Visit	.	
S124	106	Hepatitis B	Hepatitis B		Opioids	Unscheduled Visit	.	
S124	106	Hepatitis B	Hepatitis B		Amphetamine	Unscheduled Visit	.	
S124	106	Hepatitis B	Hepatitis B		Cocaine	Unscheduled Visit	.	
S124	106	Hepatitis B	Hepatitis B		THC	Unscheduled Visit	.	
S124	106	Hepatitis B	Hepatitis B		Benzodiazepines	Unscheduled Visit	.	
S124	106	Hepatitis B	Hepatitis B		Opioids	Unscheduled Visit	.	
S124	106	Hepatitis B	Hepatitis B		Amphetamine	Unscheduled Visit	.	
S124	106	Hepatitis B	Hepatitis B		Cocaine	Unscheduled Visit	.	
S124	106	Hepatitis B	Hepatitis B		THC	Unscheduled Visit	.	
S125	102	JAIVAC-1	Dosage A		Benzodiazepines	Day -14 to Day -1	18/08/2010	Negative
S125	102	JAIVAC-1	Dosage A		Opioids	Day -14 to Day -1	18/08/2010	Negative
S125	102	JAIVAC-1	Dosage A		Amphetamine	Day -14 to Day -1	18/08/2010	Negative
S125	102	JAIVAC-1	Dosage A		Cocaine	Day -14 to Day -1	18/08/2010	Negative
S125	102	JAIVAC-1	Dosage A		THC	Day -14 to Day -1	18/08/2010	Negative
S125	102	JAIVAC-1	Dosage A	2	Benzodiazepines	Day 28	21/09/2010	Negative
S125	102	JAIVAC-1	Dosage A	2	Opioids	Day 28	21/09/2010	Negative
S125	102	JAIVAC-1	Dosage A	2	Amphetamine	Day 28	21/09/2010	Negative
S125	102	JAIVAC-1	Dosage A	2	Cocaine	Day 28	21/09/2010	Negative
S125	102	JAIVAC-1	Dosage A	2	THC	Day 28	21/09/2010	Negative
S125	102	JAIVAC-1	Dosage A	3	Benzodiazepines	Day 180	21/02/2011	Negative
S125	102	JAIVAC-1	Dosage A	3	Opioids	Day 180	21/02/2011	Negative
S125	102	JAIVAC-1	Dosage A	3	Amphetamine	Day 180	21/02/2011	Negative

Subject No.	Randomisation Number	Study Group	Dosage	Dose	Urine Parameters	Day	Date of sample Collection	Results
S125	102	JAIVAC-1	Dosage A	3	Cocaine	Day 180	21/02/2011	Negative
S125	102	JAIVAC-1	Dosage A	3	THC	Day 180	21/02/2011	Negative
S125	102	JAIVAC-1	Dosage A	3	Benzodiazepines	Day 365	26/08/2011	Negative
S125	102	JAIVAC-1	Dosage A	3	Opioids	Day 365	26/08/2011	Negative
S125	102	JAIVAC-1	Dosage A	3	Amphetamine	Day 365	26/08/2011	Negative
S125	102	JAIVAC-1	Dosage A	3	Cocaine	Day 365	26/08/2011	Negative
S125	102	JAIVAC-1	Dosage A	3	THC	Day 365	26/08/2011	Negative
S125	102	JAIVAC-1	Dosage A		Benzodiazepines	Unscheduled Visit	.	
S125	102	JAIVAC-1	Dosage A		Opioids	Unscheduled Visit	.	
S125	102	JAIVAC-1	Dosage A		Amphetamine	Unscheduled Visit	.	
S125	102	JAIVAC-1	Dosage A		Cocaine	Unscheduled Visit	.	
S125	102	JAIVAC-1	Dosage A		THC	Unscheduled Visit	.	
S125	102	JAIVAC-1	Dosage A		Benzodiazepines	Unscheduled Visit	.	
S125	102	JAIVAC-1	Dosage A		Opioids	Unscheduled Visit	.	
S125	102	JAIVAC-1	Dosage A		Amphetamine	Unscheduled Visit	.	
S125	102	JAIVAC-1	Dosage A		Cocaine	Unscheduled Visit	.	
S125	102	JAIVAC-1	Dosage A		THC	Unscheduled Visit	.	
S130	105	JAIVAC-1	Dosage A		Benzodiazepines	Day -14 to Day -1	17/08/2010	Negative
S130	105	JAIVAC-1	Dosage A		Opioids	Day -14 to Day -1	17/08/2010	Negative
S130	105	JAIVAC-1	Dosage A		Amphetamine	Day -14 to Day -1	17/08/2010	Negative
S130	105	JAIVAC-1	Dosage A		Cocaine	Day -14 to Day -1	17/08/2010	Negative
S130	105	JAIVAC-1	Dosage A		THC	Day -14 to Day -1	17/08/2010	Negative
S130	105	JAIVAC-1	Dosage A	2	Benzodiazepines	Day 28	23/09/2010	Negative
S130	105	JAIVAC-1	Dosage A	2	Opioids	Day 28	23/09/2010	Negative
S130	105	JAIVAC-1	Dosage A	2	Amphetamine	Day 28	23/09/2010	Negative
S130	105	JAIVAC-1	Dosage A	2	Cocaine	Day 28	23/09/2010	Negative
S130	105	JAIVAC-1	Dosage A	2	THC	Day 28	23/09/2010	Negative
S130	105	JAIVAC-1	Dosage A	3	Benzodiazepines	Day 180	22/02/2011	Negative
S130	105	JAIVAC-1	Dosage A	3	Opioids	Day 180	22/02/2011	Negative
S130	105	JAIVAC-1	Dosage A	3	Amphetamine	Day 180	22/02/2011	Negative

Subject No.	Randomisation Number	Study Group	Dosage	Dose	Urine Parameters	Day	Date of sample Collection	Results
S130	105	JAIVAC-1	Dosage A	3	Cocaine	Day 180	22/02/2011	Negative
S130	105	JAIVAC-1	Dosage A	3	THC	Day 180	22/02/2011	Negative
S130	105	JAIVAC-1	Dosage A	3	Benzodiazepines	Day 365	25/08/2011	Negative
S130	105	JAIVAC-1	Dosage A	3	Opioids	Day 365	25/08/2011	Negative
S130	105	JAIVAC-1	Dosage A	3	Amphetamine	Day 365	25/08/2011	Negative
S130	105	JAIVAC-1	Dosage A	3	Cocaine	Day 365	25/08/2011	Negative
S130	105	JAIVAC-1	Dosage A	3	THC	Day 365	25/08/2011	Negative
S130	105	JAIVAC-1	Dosage A		Benzodiazepines	Unscheduled Visit	.	
S130	105	JAIVAC-1	Dosage A		Opioids	Unscheduled Visit	.	
S130	105	JAIVAC-1	Dosage A		Amphetamine	Unscheduled Visit	.	
S130	105	JAIVAC-1	Dosage A		Cocaine	Unscheduled Visit	.	
S130	105	JAIVAC-1	Dosage A		THC	Unscheduled Visit	.	
S132	108	Hepatitis B	Hepatitis B		Benzodiazepines	Day -14 to Day -1	18/08/2010	Negative
S132	108	Hepatitis B	Hepatitis B		Opioids	Day -14 to Day -1	18/08/2010	Negative
S132	108	Hepatitis B	Hepatitis B		Amphetamine	Day -14 to Day -1	18/08/2010	Negative
S132	108	Hepatitis B	Hepatitis B		Cocaine	Day -14 to Day -1	18/08/2010	Negative
S132	108	Hepatitis B	Hepatitis B		THC	Day -14 to Day -1	18/08/2010	Negative
S132	108	Hepatitis B	Hepatitis B	2	Benzodiazepines	Day 28	23/09/2010	Negative
S132	108	Hepatitis B	Hepatitis B	2	Opioids	Day 28	23/09/2010	Negative
S132	108	Hepatitis B	Hepatitis B	2	Amphetamine	Day 28	23/09/2010	Negative
S132	108	Hepatitis B	Hepatitis B	2	Cocaine	Day 28	23/09/2010	Negative
S132	108	Hepatitis B	Hepatitis B	2	THC	Day 28	23/09/2010	Negative
S132	108	Hepatitis B	Hepatitis B	3	Benzodiazepines	Day 180	22/02/2011	Negative
S132	108	Hepatitis B	Hepatitis B	3	Opioids	Day 180	22/02/2011	Negative
S132	108	Hepatitis B	Hepatitis B	3	Amphetamine	Day 180	22/02/2011	Negative
S132	108	Hepatitis B	Hepatitis B	3	Cocaine	Day 180	22/02/2011	Negative
S132	108	Hepatitis B	Hepatitis B	3	THC	Day 180	22/02/2011	Negative
S132	108	Hepatitis B	Hepatitis B	3	Benzodiazepines	Day 365	25/08/2011	Negative
S132	108	Hepatitis B	Hepatitis B	3	Opioids	Day 365	25/08/2011	Negative
S132	108	Hepatitis B	Hepatitis B	3	Amphetamine	Day 365	25/08/2011	Negative

Subject No.	Randomisation Number	Study Group	Dosage	Dose	Urine Parameters	Day	Date of sample Collection	Results
S132	108	Hepatitis B	Hepatitis B	3	Cocaine	Day 365	25/08/2011	Negative
S132	108	Hepatitis B	Hepatitis B	3	THC	Day 365	25/08/2011	Negative
S136	109	JAIVAC-1	Dosage A		Benzodiazepines	Day -14 to Day -1	19/08/2010	Negative
S136	109	JAIVAC-1	Dosage A		Opioids	Day -14 to Day -1	19/08/2010	Negative
S136	109	JAIVAC-1	Dosage A		Amphetamine	Day -14 to Day -1	19/08/2010	Negative
S136	109	JAIVAC-1	Dosage A		Cocaine	Day -14 to Day -1	19/08/2010	Negative
S136	109	JAIVAC-1	Dosage A		THC	Day -14 to Day -1	19/08/2010	Negative
S136	109	JAIVAC-1	Dosage A	2	Benzodiazepines	Day 28	23/09/2010	Negative
S136	109	JAIVAC-1	Dosage A	2	Opioids	Day 28	23/09/2010	Negative
S136	109	JAIVAC-1	Dosage A	2	Amphetamine	Day 28	23/09/2010	Negative
S136	109	JAIVAC-1	Dosage A	2	Cocaine	Day 28	23/09/2010	Negative
S136	109	JAIVAC-1	Dosage A	2	THC	Day 28	23/09/2010	Negative
S136	109	JAIVAC-1	Dosage A	3	Benzodiazepines	Day 180	22/02/2011	Negative
S136	109	JAIVAC-1	Dosage A	3	Opioids	Day 180	22/02/2011	Negative
S136	109	JAIVAC-1	Dosage A	3	Amphetamine	Day 180	22/02/2011	Negative
S136	109	JAIVAC-1	Dosage A	3	Cocaine	Day 180	22/02/2011	Negative
S136	109	JAIVAC-1	Dosage A	3	THC	Day 180	22/02/2011	Negative
S136	109	JAIVAC-1	Dosage A	3	Benzodiazepines	Day 365	25/08/2011	Negative
S136	109	JAIVAC-1	Dosage A	3	Opioids	Day 365	25/08/2011	Negative
S136	109	JAIVAC-1	Dosage A	3	Amphetamine	Day 365	25/08/2011	Negative
S136	109	JAIVAC-1	Dosage A	3	Cocaine	Day 365	25/08/2011	Negative
S136	109	JAIVAC-1	Dosage A	3	THC	Day 365	25/08/2011	Negative
S143	101	JAIVAC-1	Dosage A		Benzodiazepines	Day -14 to Day -1	18/08/2010	Negative
S143	101	JAIVAC-1	Dosage A		Opioids	Day -14 to Day -1	18/08/2010	Negative
S143	101	JAIVAC-1	Dosage A		Amphetamine	Day -14 to Day -1	18/08/2010	Negative
S143	101	JAIVAC-1	Dosage A		Cocaine	Day -14 to Day -1	18/08/2010	Negative
S143	101	JAIVAC-1	Dosage A		THC	Day -14 to Day -1	18/08/2010	Negative
S143	101	JAIVAC-1	Dosage A	2	Benzodiazepines	Day 28	21/09/2010	Negative
S143	101	JAIVAC-1	Dosage A	2	Opioids	Day 28	21/09/2010	Negative
S143	101	JAIVAC-1	Dosage A	2	Amphetamine	Day 28	21/09/2010	Negative

Subject No.	Randomisation Number	Study Group	Dosage	Dose	Urine Parameters	Day	Date of sample Collection	Results
S143	101	JAIVAC-1	Dosage A	2	Cocaine	Day 28	21/09/2010	Negative
S143	101	JAIVAC-1	Dosage A	2	THC	Day 28	21/09/2010	Negative
S143	101	JAIVAC-1	Dosage A	3	Benzodiazepines	Day 180	21/02/2011	Negative
S143	101	JAIVAC-1	Dosage A	3	Opioids	Day 180	21/02/2011	Negative
S143	101	JAIVAC-1	Dosage A	3	Amphetamine	Day 180	21/02/2011	Negative
S143	101	JAIVAC-1	Dosage A	3	Cocaine	Day 180	21/02/2011	Negative
S143	101	JAIVAC-1	Dosage A	3	THC	Day 180	21/02/2011	Negative
S143	101	JAIVAC-1	Dosage A	3	Benzodiazepines	Day 365	26/08/2011	Negative
S143	101	JAIVAC-1	Dosage A	3	Opioids	Day 365	26/08/2011	Negative
S143	101	JAIVAC-1	Dosage A	3	Amphetamine	Day 365	26/08/2011	Negative
S143	101	JAIVAC-1	Dosage A	3	Cocaine	Day 365	26/08/2011	Negative
S143	101	JAIVAC-1	Dosage A	3	THC	Day 365	26/08/2011	Negative
S144	103	Hepatitis B	Hepatitis B		Benzodiazepines	Day -14 to Day -1	19/08/2010	Negative
S144	103	Hepatitis B	Hepatitis B		Opioids	Day -14 to Day -1	19/08/2010	Negative
S144	103	Hepatitis B	Hepatitis B		Amphetamine	Day -14 to Day -1	19/08/2010	Negative
S144	103	Hepatitis B	Hepatitis B		Cocaine	Day -14 to Day -1	19/08/2010	Negative
S144	103	Hepatitis B	Hepatitis B		THC	Day -14 to Day -1	19/08/2010	Negative
S144	103	Hepatitis B	Hepatitis B	2	Benzodiazepines	Day 28	21/09/2010	Negative
S144	103	Hepatitis B	Hepatitis B	2	Opioids	Day 28	21/09/2010	Negative
S144	103	Hepatitis B	Hepatitis B	2	Amphetamine	Day 28	21/09/2010	Negative
S144	103	Hepatitis B	Hepatitis B	2	Cocaine	Day 28	21/09/2010	Negative
S144	103	Hepatitis B	Hepatitis B	2	THC	Day 28	21/09/2010	Negative
S144	103	Hepatitis B	Hepatitis B	3	Benzodiazepines	Day 180	21/02/2011	Negative
S144	103	Hepatitis B	Hepatitis B	3	Opioids	Day 180	21/02/2011	Negative
S144	103	Hepatitis B	Hepatitis B	3	Amphetamine	Day 180	21/02/2011	Negative
S144	103	Hepatitis B	Hepatitis B	3	Cocaine	Day 180	21/02/2011	Negative
S144	103	Hepatitis B	Hepatitis B	3	THC	Day 180	21/02/2011	Negative
S144	103	Hepatitis B	Hepatitis B	3	Benzodiazepines	Day 365	26/08/2011	Negative
S144	103	Hepatitis B	Hepatitis B	3	Opioids	Day 365	26/08/2011	Negative
S144	103	Hepatitis B	Hepatitis B	3	Amphetamine	Day 365	26/08/2011	Negative

Subject No.	Randomisation Number	Study Group	Dosage	Dose	Urine Parameters	Day	Date of sample Collection	Results
S144	103	Hepatitis B	Hepatitis B	3	Cocaine	Day 365	26/08/2011	Negative
S144	103	Hepatitis B	Hepatitis B	3	THC	Day 365	26/08/2011	Negative
S145	112	JAIVAC-1	Dosage A		Benzodiazepines	Day -14 to Day -1	19/08/2010	Negative
S145	112	JAIVAC-1	Dosage A		Opioids	Day -14 to Day -1	19/08/2010	Negative
S145	112	JAIVAC-1	Dosage A		Amphetamine	Day -14 to Day -1	19/08/2010	Negative
S145	112	JAIVAC-1	Dosage A		Cocaine	Day -14 to Day -1	19/08/2010	Negative
S145	112	JAIVAC-1	Dosage A		THC	Day -14 to Day -1	19/08/2010	Negative
S145	112	JAIVAC-1	Dosage A	2	Benzodiazepines	Day 28	24/09/2010	Negative
S145	112	JAIVAC-1	Dosage A	2	Opioids	Day 28	24/09/2010	Negative
S145	112	JAIVAC-1	Dosage A	2	Amphetamine	Day 28	24/09/2010	Negative
S145	112	JAIVAC-1	Dosage A	2	Cocaine	Day 28	24/09/2010	Negative
S145	112	JAIVAC-1	Dosage A	2	THC	Day 28	24/09/2010	Negative
S145	112	JAIVAC-1	Dosage A	3	Benzodiazepines	Day 180	23/02/2011	Negative
S145	112	JAIVAC-1	Dosage A	3	Opioids	Day 180	23/02/2011	Negative
S145	112	JAIVAC-1	Dosage A	3	Amphetamine	Day 180	23/02/2011	Negative
S145	112	JAIVAC-1	Dosage A	3	Cocaine	Day 180	23/02/2011	Negative
S145	112	JAIVAC-1	Dosage A	3	THC	Day 180	23/02/2011	Negative
S145	112	JAIVAC-1	Dosage A	3	Benzodiazepines	Day 365	26/08/2011	Negative
S145	112	JAIVAC-1	Dosage A	3	Opioids	Day 365	26/08/2011	Negative
S145	112	JAIVAC-1	Dosage A	3	Amphetamine	Day 365	26/08/2011	Negative
S145	112	JAIVAC-1	Dosage A	3	Cocaine	Day 365	26/08/2011	Negative
S145	112	JAIVAC-1	Dosage A	3	THC	Day 365	26/08/2011	Negative
S148	111	JAIVAC-1	Dosage A		Benzodiazepines	Day -14 to Day -1	20/08/2010	Negative
S148	111	JAIVAC-1	Dosage A		Opioids	Day -14 to Day -1	20/08/2010	Negative
S148	111	JAIVAC-1	Dosage A		Amphetamine	Day -14 to Day -1	20/08/2010	Negative
S148	111	JAIVAC-1	Dosage A		Cocaine	Day -14 to Day -1	20/08/2010	Negative
S148	111	JAIVAC-1	Dosage A		THC	Day -14 to Day -1	20/08/2010	Negative
S148	111	JAIVAC-1	Dosage A	2	Benzodiazepines	Day 28	24/09/2010	Negative
S148	111	JAIVAC-1	Dosage A	2	Opioids	Day 28	24/09/2010	Negative
S148	111	JAIVAC-1	Dosage A	2	Amphetamine	Day 28	24/09/2010	Negative

Subject No.	Randomisation Number	Study Group	Dosage	Dose	Urine Parameters	Day	Date of sample Collection	Results
S148	111	JAIVAC-1	Dosage A	2	Cocaine	Day 28	24/09/2010	Negative
S148	111	JAIVAC-1	Dosage A	2	THC	Day 28	24/09/2010	Negative
S148	111	JAIVAC-1	Dosage A	3	Benzodiazepines	Day 180	23/02/2011	Negative
S148	111	JAIVAC-1	Dosage A	3	Opioids	Day 180	23/02/2011	Negative
S148	111	JAIVAC-1	Dosage A	3	Amphetamine	Day 180	23/02/2011	Negative
S148	111	JAIVAC-1	Dosage A	3	Cocaine	Day 180	23/02/2011	Negative
S148	111	JAIVAC-1	Dosage A	3	THC	Day 180	23/02/2011	Negative
S148	111	JAIVAC-1	Dosage A	3	Benzodiazepines	Day 365	26/08/2011	Negative
S148	111	JAIVAC-1	Dosage A	3	Opioids	Day 365	26/08/2011	Negative
S148	111	JAIVAC-1	Dosage A	3	Amphetamine	Day 365	26/08/2011	Negative
S148	111	JAIVAC-1	Dosage A	3	Cocaine	Day 365	26/08/2011	Negative
S148	111	JAIVAC-1	Dosage A	3	THC	Day 365	26/08/2011	Negative
S154	113	JAIVAC-1	Dosage A		Benzodiazepines	Day -14 to Day -1	19/08/2010	Negative
S154	113	JAIVAC-1	Dosage A		Opioids	Day -14 to Day -1	19/08/2010	Negative
S154	113	JAIVAC-1	Dosage A		Amphetamine	Day -14 to Day -1	19/08/2010	Negative
S154	113	JAIVAC-1	Dosage A		Cocaine	Day -14 to Day -1	19/08/2010	Negative
S154	113	JAIVAC-1	Dosage A		THC	Day -14 to Day -1	19/08/2010	Negative
S154	113	JAIVAC-1	Dosage A	2	Benzodiazepines	Day 28	24/09/2010	Negative
S154	113	JAIVAC-1	Dosage A	2	Opioids	Day 28	24/09/2010	Negative
S154	113	JAIVAC-1	Dosage A	2	Amphetamine	Day 28	24/09/2010	Negative
S154	113	JAIVAC-1	Dosage A	2	Cocaine	Day 28	24/09/2010	Negative
S154	113	JAIVAC-1	Dosage A	2	THC	Day 28	24/09/2010	Negative
S154	113	JAIVAC-1	Dosage A	3	Benzodiazepines	Day 180	23/02/2011	Negative
S154	113	JAIVAC-1	Dosage A	3	Opioids	Day 180	23/02/2011	Negative
S154	113	JAIVAC-1	Dosage A	3	Amphetamine	Day 180	23/02/2011	Negative
S154	113	JAIVAC-1	Dosage A	3	Cocaine	Day 180	23/02/2011	Negative
S154	113	JAIVAC-1	Dosage A	3	THC	Day 180	23/02/2011	Negative
S154	113	JAIVAC-1	Dosage A	3	Benzodiazepines	Day 365	26/08/2011	Negative
S154	113	JAIVAC-1	Dosage A	3	Opioids	Day 365	26/08/2011	Negative
S154	113	JAIVAC-1	Dosage A	3	Amphetamine	Day 365	26/08/2011	Negative

Subject No.	Randomisation Number	Study Group	Dosage	Dose	Urine Parameters	Day	Date of sample Collection	Results
S154	113	JAIVAC-1	Dosage A	3	Cocaine	Day 365	26/08/2011	Negative
S154	113	JAIVAC-1	Dosage A	3	THC	Day 365	26/08/2011	Negative
S156	110	JAIVAC-1	Dosage A		Benzodiazepines	Day -14 to Day -1	19/08/2010	Negative
S156	110	JAIVAC-1	Dosage A		Opioids	Day -14 to Day -1	19/08/2010	Negative
S156	110	JAIVAC-1	Dosage A		Amphetamine	Day -14 to Day -1	19/08/2010	Negative
S156	110	JAIVAC-1	Dosage A		Cocaine	Day -14 to Day -1	19/08/2010	Negative
S156	110	JAIVAC-1	Dosage A		THC	Day -14 to Day -1	19/08/2010	Negative
S156	110	JAIVAC-1	Dosage A	2	Benzodiazepines	Day 28	24/09/2010	Negative
S156	110	JAIVAC-1	Dosage A	2	Opioids	Day 28	24/09/2010	Negative
S156	110	JAIVAC-1	Dosage A	2	Amphetamine	Day 28	24/09/2010	Negative
S156	110	JAIVAC-1	Dosage A	2	Cocaine	Day 28	24/09/2010	Negative
S156	110	JAIVAC-1	Dosage A	2	THC	Day 28	24/09/2010	Negative
S156	110	JAIVAC-1	Dosage A	3	Benzodiazepines	Day 180	23/02/2011	Negative
S156	110	JAIVAC-1	Dosage A	3	Opioids	Day 180	23/02/2011	Negative
S156	110	JAIVAC-1	Dosage A	3	Amphetamine	Day 180	23/02/2011	Negative
S156	110	JAIVAC-1	Dosage A	3	Cocaine	Day 180	23/02/2011	Negative
S156	110	JAIVAC-1	Dosage A	3	THC	Day 180	23/02/2011	Negative
S156	110	JAIVAC-1	Dosage A	3	Benzodiazepines	Day 365	26/08/2011	Negative
S156	110	JAIVAC-1	Dosage A	3	Opioids	Day 365	26/08/2011	Negative
S156	110	JAIVAC-1	Dosage A	3	Amphetamine	Day 365	26/08/2011	Negative
S156	110	JAIVAC-1	Dosage A	3	Cocaine	Day 365	26/08/2011	Negative
S156	110	JAIVAC-1	Dosage A	3	THC	Day 365	26/08/2011	Negative
S170	204	Hepatitis B	Hepatitis B		Benzodiazepines	Day -14 to Day -1	21/09/2010	Negative
S170	204	Hepatitis B	Hepatitis B		Opioids	Day -14 to Day -1	21/09/2010	Negative
S170	204	Hepatitis B	Hepatitis B		Amphetamine	Day -14 to Day -1	21/09/2010	Negative
S170	204	Hepatitis B	Hepatitis B		Cocaine	Day -14 to Day -1	21/09/2010	Negative
S170	204	Hepatitis B	Hepatitis B		THC	Day -14 to Day -1	21/09/2010	Negative
S170	204	Hepatitis B	Hepatitis B	2	Benzodiazepines	Day 28	27/10/2010	Negative
S170	204	Hepatitis B	Hepatitis B	2	Opioids	Day 28	27/10/2010	Negative
S170	204	Hepatitis B	Hepatitis B	2	Amphetamine	Day 28	27/10/2010	Negative

Subject No.	Randomisation Number	Study Group	Dosage	Dose	Urine Parameters	Day	Date of sample Collection	Results
S170	204	Hepatitis B	Hepatitis B	2	Cocaine	Day 28	27/10/2010	Negative
S170	204	Hepatitis B	Hepatitis B	2	THC	Day 28	27/10/2010	Negative
S170	204	Hepatitis B	Hepatitis B	3	Benzodiazepines	Day 180	28/03/2011	Negative
S170	204	Hepatitis B	Hepatitis B	3	Opioids	Day 180	28/03/2011	Negative
S170	204	Hepatitis B	Hepatitis B	3	Amphetamine	Day 180	28/03/2011	Negative
S170	204	Hepatitis B	Hepatitis B	3	Cocaine	Day 180	28/03/2011	Negative
S170	204	Hepatitis B	Hepatitis B	3	THC	Day 180	28/03/2011	Negative
S170	204	Hepatitis B	Hepatitis B	3	Benzodiazepines	Day 365	28/09/2011	Negative
S170	204	Hepatitis B	Hepatitis B	3	Opioids	Day 365	28/09/2011	Negative
S170	204	Hepatitis B	Hepatitis B	3	Amphetamine	Day 365	28/09/2011	Negative
S170	204	Hepatitis B	Hepatitis B	3	Cocaine	Day 365	28/09/2011	Negative
S170	204	Hepatitis B	Hepatitis B	3	THC	Day 365	28/09/2011	Negative
S175	209	JAIVAC-1	Dosage B		Benzodiazepines	Day -14 to Day -1	21/09/2010	Negative
S175	209	JAIVAC-1	Dosage B		Opioids	Day -14 to Day -1	21/09/2010	Negative
S175	209	JAIVAC-1	Dosage B		Amphetamine	Day -14 to Day -1	21/09/2010	Negative
S175	209	JAIVAC-1	Dosage B		Cocaine	Day -14 to Day -1	21/09/2010	Negative
S175	209	JAIVAC-1	Dosage B		THC	Day -14 to Day -1	21/09/2010	Negative
S175	209	JAIVAC-1	Dosage B	2	Benzodiazepines	Day 28	27/10/2010	Negative
S175	209	JAIVAC-1	Dosage B	2	Opioids	Day 28	27/10/2010	Negative
S175	209	JAIVAC-1	Dosage B	2	Amphetamine	Day 28	27/10/2010	Negative
S175	209	JAIVAC-1	Dosage B	2	Cocaine	Day 28	27/10/2010	Negative
S175	209	JAIVAC-1	Dosage B	2	THC	Day 28	27/10/2010	Negative
S175	209	JAIVAC-1	Dosage B	3	Benzodiazepines	Day 180	28/03/2011	Negative
S175	209	JAIVAC-1	Dosage B	3	Opioids	Day 180	28/03/2011	Negative
S175	209	JAIVAC-1	Dosage B	3	Amphetamine	Day 180	28/03/2011	Negative
S175	209	JAIVAC-1	Dosage B	3	Cocaine	Day 180	28/03/2011	Negative
S175	209	JAIVAC-1	Dosage B	3	THC	Day 180	28/03/2011	Negative
S175	209	JAIVAC-1	Dosage B	3	Benzodiazepines	Day 365	28/09/2011	Negative
S175	209	JAIVAC-1	Dosage B	3	Opioids	Day 365	28/09/2011	Negative
S175	209	JAIVAC-1	Dosage B	3	Amphetamine	Day 365	28/09/2011	Negative

Subject No.	Randomisation Number	Study Group	Dosage	Dose	Urine Parameters	Day	Date of sample Collection	Results
S175	209	JAIVAC-1	Dosage B	3	Cocaine	Day 365	28/09/2011	Negative
S175	209	JAIVAC-1	Dosage B	3	THC	Day 365	28/09/2011	Negative
S176	205	Hepatitis B	Hepatitis B		Benzodiazepines	Day -14 to Day -1	22/09/2010	Negative
S176	205	Hepatitis B	Hepatitis B		Opioids	Day -14 to Day -1	22/09/2010	Negative
S176	205	Hepatitis B	Hepatitis B		Amphetamine	Day -14 to Day -1	22/09/2010	Negative
S176	205	Hepatitis B	Hepatitis B		Cocaine	Day -14 to Day -1	22/09/2010	Negative
S176	205	Hepatitis B	Hepatitis B		THC	Day -14 to Day -1	22/09/2010	Negative
S176	205	Hepatitis B	Hepatitis B	2	Benzodiazepines	Day 28	27/10/2010	Negative
S176	205	Hepatitis B	Hepatitis B	2	Opioids	Day 28	27/10/2010	Negative
S176	205	Hepatitis B	Hepatitis B	2	Amphetamine	Day 28	27/10/2010	Negative
S176	205	Hepatitis B	Hepatitis B	2	Cocaine	Day 28	27/10/2010	Negative
S176	205	Hepatitis B	Hepatitis B	2	THC	Day 28	27/10/2010	Negative
S176	205	Hepatitis B	Hepatitis B	3	Benzodiazepines	Day 180	28/03/2011	Negative
S176	205	Hepatitis B	Hepatitis B	3	Opioids	Day 180	28/03/2011	Negative
S176	205	Hepatitis B	Hepatitis B	3	Amphetamine	Day 180	28/03/2011	Negative
S176	205	Hepatitis B	Hepatitis B	3	Cocaine	Day 180	28/03/2011	Negative
S176	205	Hepatitis B	Hepatitis B	3	THC	Day 180	28/03/2011	Negative
S176	205	Hepatitis B	Hepatitis B	3	Benzodiazepines	Day 365	28/09/2011	Negative
S176	205	Hepatitis B	Hepatitis B	3	Opioids	Day 365	28/09/2011	Negative
S176	205	Hepatitis B	Hepatitis B	3	Amphetamine	Day 365	28/09/2011	Negative
S176	205	Hepatitis B	Hepatitis B	3	Cocaine	Day 365	28/09/2011	Negative
S176	205	Hepatitis B	Hepatitis B	3	THC	Day 365	28/09/2011	Negative
S176	205	Hepatitis B	Hepatitis B		Benzodiazepines	Unscheduled Visit	.	
S176	205	Hepatitis B	Hepatitis B		Opioids	Unscheduled Visit	.	
S176	205	Hepatitis B	Hepatitis B		Amphetamine	Unscheduled Visit	.	
S176	205	Hepatitis B	Hepatitis B		Cocaine	Unscheduled Visit	.	
S176	205	Hepatitis B	Hepatitis B		THC	Unscheduled Visit	.	
S179	207	Hepatitis B	Hepatitis B		Benzodiazepines	Day -14 to Day -1	22/09/2010	Negative
S179	207	Hepatitis B	Hepatitis B		Opioids	Day -14 to Day -1	22/09/2010	Negative
S179	207	Hepatitis B	Hepatitis B		Amphetamine	Day -14 to Day -1	22/09/2010	Negative

Subject No.	Randomisation Number	Study Group	Dosage	Dose	Urine Parameters	Day	Date of sample Collection	Results
S179	207	Hepatitis B	Hepatitis B		Cocaine	Day -14 to Day -1	22/09/2010	Negative
S179	207	Hepatitis B	Hepatitis B		THC	Day -14 to Day -1	22/09/2010	Negative
S179	207	Hepatitis B	Hepatitis B	2	Benzodiazepines	Day 28	27/10/2010	Negative
S179	207	Hepatitis B	Hepatitis B	2	Opioids	Day 28	27/10/2010	Negative
S179	207	Hepatitis B	Hepatitis B	2	Amphetamine	Day 28	27/10/2010	Negative
S179	207	Hepatitis B	Hepatitis B	2	Cocaine	Day 28	27/10/2010	Negative
S179	207	Hepatitis B	Hepatitis B	2	THC	Day 28	27/10/2010	Negative
S179	207	Hepatitis B	Hepatitis B	3	Benzodiazepines	Day 180	28/03/2011	Negative
S179	207	Hepatitis B	Hepatitis B	3	Opioids	Day 180	28/03/2011	Negative
S179	207	Hepatitis B	Hepatitis B	3	Amphetamine	Day 180	28/03/2011	Negative
S179	207	Hepatitis B	Hepatitis B	3	Cocaine	Day 180	28/03/2011	Negative
S179	207	Hepatitis B	Hepatitis B	3	THC	Day 180	28/03/2011	Negative
S179	207	Hepatitis B	Hepatitis B	3	Benzodiazepines	Day 365	28/09/2011	Negative
S179	207	Hepatitis B	Hepatitis B	3	Opioids	Day 365	28/09/2011	Negative
S179	207	Hepatitis B	Hepatitis B	3	Amphetamine	Day 365	28/09/2011	Negative
S179	207	Hepatitis B	Hepatitis B	3	Cocaine	Day 365	28/09/2011	Negative
S179	207	Hepatitis B	Hepatitis B	3	THC	Day 365	28/09/2011	Negative
S182	208	JAIVAC-1	Dosage B		Benzodiazepines	Day -14 to Day -1	21/09/2010	Negative
S182	208	JAIVAC-1	Dosage B		Opioids	Day -14 to Day -1	21/09/2010	Negative
S182	208	JAIVAC-1	Dosage B		Amphetamine	Day -14 to Day -1	21/09/2010	Negative
S182	208	JAIVAC-1	Dosage B		Cocaine	Day -14 to Day -1	21/09/2010	Negative
S182	208	JAIVAC-1	Dosage B		THC	Day -14 to Day -1	21/09/2010	Negative
S182	208	JAIVAC-1	Dosage B	2	Benzodiazepines	Day 28	27/10/2010	Negative
S182	208	JAIVAC-1	Dosage B	2	Opioids	Day 28	27/10/2010	Negative
S182	208	JAIVAC-1	Dosage B	2	Amphetamine	Day 28	27/10/2010	Negative
S182	208	JAIVAC-1	Dosage B	2	Cocaine	Day 28	27/10/2010	Negative
S182	208	JAIVAC-1	Dosage B	2	THC	Day 28	27/10/2010	Negative
S182	208	JAIVAC-1	Dosage B	3	Benzodiazepines	Day 180	28/03/2011	Negative
S182	208	JAIVAC-1	Dosage B	3	Opioids	Day 180	28/03/2011	Negative
S182	208	JAIVAC-1	Dosage B	3	Amphetamine	Day 180	28/03/2011	Negative

Subject No.	Randomisation Number	Study Group	Dosage	Dose	Urine Parameters	Day	Date of sample Collection	Results
S182	208	JAIVAC-1	Dosage B	3	Cocaine	Day 180	28/03/2011	Negative
S182	208	JAIVAC-1	Dosage B	3	THC	Day 180	28/03/2011	Negative
S182	208	JAIVAC-1	Dosage B	3	Benzodiazepines	Day 365	29/09/2011	Negative
S182	208	JAIVAC-1	Dosage B	3	Opioids	Day 365	29/09/2011	Negative
S182	208	JAIVAC-1	Dosage B	3	Amphetamine	Day 365	29/09/2011	Negative
S182	208	JAIVAC-1	Dosage B	3	Cocaine	Day 365	29/09/2011	Negative
S182	208	JAIVAC-1	Dosage B	3	THC	Day 365	29/09/2011	Negative
S182	208	JAIVAC-1	Dosage B		Benzodiazepines	Unscheduled Visit	.	
S182	208	JAIVAC-1	Dosage B		Opioids	Unscheduled Visit	.	
S182	208	JAIVAC-1	Dosage B		Amphetamine	Unscheduled Visit	.	
S182	208	JAIVAC-1	Dosage B		Cocaine	Unscheduled Visit	.	
S182	208	JAIVAC-1	Dosage B		THC	Unscheduled Visit	.	
S185	202	JAIVAC-1	Dosage B		Benzodiazepines	Day -14 to Day -1	21/09/2010	Negative
S185	202	JAIVAC-1	Dosage B		Opioids	Day -14 to Day -1	21/09/2010	Negative
S185	202	JAIVAC-1	Dosage B		Amphetamine	Day -14 to Day -1	21/09/2010	Negative
S185	202	JAIVAC-1	Dosage B		Cocaine	Day -14 to Day -1	21/09/2010	Negative
S185	202	JAIVAC-1	Dosage B		THC	Day -14 to Day -1	21/09/2010	Negative
S185	202	JAIVAC-1	Dosage B	2	Benzodiazepines	Day 28	26/10/2010	Negative
S185	202	JAIVAC-1	Dosage B	2	Opioids	Day 28	26/10/2010	Negative
S185	202	JAIVAC-1	Dosage B	2	Amphetamine	Day 28	26/10/2010	Negative
S185	202	JAIVAC-1	Dosage B	2	Cocaine	Day 28	26/10/2010	Negative
S185	202	JAIVAC-1	Dosage B	2	THC	Day 28	26/10/2010	Negative
S185	202	JAIVAC-1	Dosage B	3	Benzodiazepines	Day 180	28/03/2011	Negative
S185	202	JAIVAC-1	Dosage B	3	Opioids	Day 180	28/03/2011	Negative
S185	202	JAIVAC-1	Dosage B	3	Amphetamine	Day 180	28/03/2011	Negative
S185	202	JAIVAC-1	Dosage B	3	Cocaine	Day 180	28/03/2011	Negative
S185	202	JAIVAC-1	Dosage B	3	THC	Day 180	28/03/2011	Negative
S185	202	JAIVAC-1	Dosage B	3	Benzodiazepines	Day 365	28/09/2011	Negative
S185	202	JAIVAC-1	Dosage B	3	Opioids	Day 365	28/09/2011	Negative
S185	202	JAIVAC-1	Dosage B	3	Amphetamine	Day 365	28/09/2011	Negative

Subject No.	Randomisation Number	Study Group	Dosage	Dose	Urine Parameters	Day	Date of sample Collection	Results
S185	202	JAIVAC-1	Dosage B	3	Cocaine	Day 365	28/09/2011	Negative
S185	202	JAIVAC-1	Dosage B	3	THC	Day 365	28/09/2011	Negative
S186	201	JAIVAC-1	Dosage B		Benzodiazepines	Day -14 to Day -1	21/09/2010	Negative
S186	201	JAIVAC-1	Dosage B		Opioids	Day -14 to Day -1	21/09/2010	Negative
S186	201	JAIVAC-1	Dosage B		Amphetamine	Day -14 to Day -1	21/09/2010	Negative
S186	201	JAIVAC-1	Dosage B		Cocaine	Day -14 to Day -1	21/09/2010	Negative
S186	201	JAIVAC-1	Dosage B		THC	Day -14 to Day -1	21/09/2010	Negative
S186	201	JAIVAC-1	Dosage B	2	Benzodiazepines	Day 28	26/10/2010	Negative
S186	201	JAIVAC-1	Dosage B	2	Opioids	Day 28	26/10/2010	Negative
S186	201	JAIVAC-1	Dosage B	2	Amphetamine	Day 28	26/10/2010	Negative
S186	201	JAIVAC-1	Dosage B	2	Cocaine	Day 28	26/10/2010	Negative
S186	201	JAIVAC-1	Dosage B	2	THC	Day 28	26/10/2010	Negative
S186	201	JAIVAC-1	Dosage B	3	Benzodiazepines	Day 180	28/03/2011	Negative
S186	201	JAIVAC-1	Dosage B	3	Opioids	Day 180	28/03/2011	Negative
S186	201	JAIVAC-1	Dosage B	3	Amphetamine	Day 180	28/03/2011	Negative
S186	201	JAIVAC-1	Dosage B	3	Cocaine	Day 180	28/03/2011	Negative
S186	201	JAIVAC-1	Dosage B	3	THC	Day 180	28/03/2011	Negative
S186	201	JAIVAC-1	Dosage B	3	Benzodiazepines	Day 365	28/09/2011	Negative
S186	201	JAIVAC-1	Dosage B	3	Opioids	Day 365	28/09/2011	Negative
S186	201	JAIVAC-1	Dosage B	3	Amphetamine	Day 365	28/09/2011	Negative
S186	201	JAIVAC-1	Dosage B	3	Cocaine	Day 365	28/09/2011	Negative
S186	201	JAIVAC-1	Dosage B	3	THC	Day 365	28/09/2011	Negative
S192	203	JAIVAC-1	Dosage B		Benzodiazepines	Day -14 to Day -1	23/09/2010	Negative
S192	203	JAIVAC-1	Dosage B		Opioids	Day -14 to Day -1	23/09/2010	Negative
S192	203	JAIVAC-1	Dosage B		Amphetamine	Day -14 to Day -1	23/09/2010	Negative
S192	203	JAIVAC-1	Dosage B		Cocaine	Day -14 to Day -1	23/09/2010	Negative
S192	203	JAIVAC-1	Dosage B		THC	Day -14 to Day -1	23/09/2010	Negative
S192	203	JAIVAC-1	Dosage B	2	Benzodiazepines	Day 28	26/10/2010	Negative
S192	203	JAIVAC-1	Dosage B	2	Opioids	Day 28	26/10/2010	Negative
S192	203	JAIVAC-1	Dosage B	2	Amphetamine	Day 28	26/10/2010	Negative

Subject No.	Randomisation Number	Study Group	Dosage	Dose	Urine Parameters	Day	Date of sample Collection	Results
S192	203	JAIVAC-1	Dosage B	2	Cocaine	Day 28	26/10/2010	Negative
S192	203	JAIVAC-1	Dosage B	2	THC	Day 28	26/10/2010	Negative
S192	203	JAIVAC-1	Dosage B	3	Benzodiazepines	Day 180	28/03/2011	Negative
S192	203	JAIVAC-1	Dosage B	3	Opioids	Day 180	28/03/2011	Negative
S192	203	JAIVAC-1	Dosage B	3	Amphetamine	Day 180	28/03/2011	Negative
S192	203	JAIVAC-1	Dosage B	3	Cocaine	Day 180	28/03/2011	Negative
S192	203	JAIVAC-1	Dosage B	3	THC	Day 180	28/03/2011	Negative
S192	203	JAIVAC-1	Dosage B	3	Benzodiazepines	Day 365	28/09/2011	Negative
S192	203	JAIVAC-1	Dosage B	3	Opioids	Day 365	28/09/2011	Negative
S192	203	JAIVAC-1	Dosage B	3	Amphetamine	Day 365	28/09/2011	Negative
S192	203	JAIVAC-1	Dosage B	3	Cocaine	Day 365	28/09/2011	Negative
S192	203	JAIVAC-1	Dosage B	3	THC	Day 365	28/09/2011	Negative
S193	215	JAIVAC-1	Dosage B		Benzodiazepines	Day -14 to Day -1	24/09/2010	Negative
S193	215	JAIVAC-1	Dosage B		Opioids	Day -14 to Day -1	24/09/2010	Negative
S193	215	JAIVAC-1	Dosage B		Amphetamine	Day -14 to Day -1	24/09/2010	Negative
S193	215	JAIVAC-1	Dosage B		Cocaine	Day -14 to Day -1	24/09/2010	Negative
S193	215	JAIVAC-1	Dosage B		THC	Day -14 to Day -1	24/09/2010	Negative
S193	215	JAIVAC-1	Dosage B	2	Benzodiazepines	Day 28	28/10/2010	Negative
S193	215	JAIVAC-1	Dosage B	2	Opioids	Day 28	28/10/2010	Negative
S193	215	JAIVAC-1	Dosage B	2	Amphetamine	Day 28	28/10/2010	Negative
S193	215	JAIVAC-1	Dosage B	2	Cocaine	Day 28	28/10/2010	Negative
S193	215	JAIVAC-1	Dosage B	2	THC	Day 28	28/10/2010	Negative
S193	215	JAIVAC-1	Dosage B	3	Benzodiazepines	Day 180	29/03/2011	Negative
S193	215	JAIVAC-1	Dosage B	3	Opioids	Day 180	29/03/2011	Negative
S193	215	JAIVAC-1	Dosage B	3	Amphetamine	Day 180	29/03/2011	Negative
S193	215	JAIVAC-1	Dosage B	3	Cocaine	Day 180	29/03/2011	Negative
S193	215	JAIVAC-1	Dosage B	3	THC	Day 180	29/03/2011	Negative
S193	215	JAIVAC-1	Dosage B	3	Benzodiazepines	Day 365	29/09/2011	Negative
S193	215	JAIVAC-1	Dosage B	3	Opioids	Day 365	29/09/2011	Negative
S193	215	JAIVAC-1	Dosage B	3	Amphetamine	Day 365	29/09/2011	Negative

Subject No.	Randomisation Number	Study Group	Dosage	Dose	Urine Parameters	Day	Date of sample Collection	Results
S193	215	JAIVAC-1	Dosage B	3	Cocaine	Day 365	29/09/2011	Negative
S193	215	JAIVAC-1	Dosage B	3	THC	Day 365	29/09/2011	Negative
S195	214	JAIVAC-1	Dosage B		Benzodiazepines	Day -14 to Day -1	23/09/2010	Negative
S195	214	JAIVAC-1	Dosage B		Opioids	Day -14 to Day -1	23/09/2010	Negative
S195	214	JAIVAC-1	Dosage B		Amphetamine	Day -14 to Day -1	23/09/2010	Negative
S195	214	JAIVAC-1	Dosage B		Cocaine	Day -14 to Day -1	23/09/2010	Negative
S195	214	JAIVAC-1	Dosage B		THC	Day -14 to Day -1	23/09/2010	Negative
S195	214	JAIVAC-1	Dosage B	2	Benzodiazepines	Day 28	28/10/2010	Negative
S195	214	JAIVAC-1	Dosage B	2	Opioids	Day 28	28/10/2010	Negative
S195	214	JAIVAC-1	Dosage B	2	Amphetamine	Day 28	28/10/2010	Negative
S195	214	JAIVAC-1	Dosage B	2	Cocaine	Day 28	28/10/2010	Negative
S195	214	JAIVAC-1	Dosage B	2	THC	Day 28	28/10/2010	Negative
S195	214	JAIVAC-1	Dosage B	3	Benzodiazepines	Day 180	29/03/2011	Negative
S195	214	JAIVAC-1	Dosage B	3	Opioids	Day 180	29/03/2011	Negative
S195	214	JAIVAC-1	Dosage B	3	Amphetamine	Day 180	29/03/2011	Negative
S195	214	JAIVAC-1	Dosage B	3	Cocaine	Day 180	29/03/2011	Negative
S195	214	JAIVAC-1	Dosage B	3	THC	Day 180	29/03/2011	Negative
S195	214	JAIVAC-1	Dosage B	3	Benzodiazepines	Day 365	29/09/2011	Negative
S195	214	JAIVAC-1	Dosage B	3	Opioids	Day 365	29/09/2011	Negative
S195	214	JAIVAC-1	Dosage B	3	Amphetamine	Day 365	29/09/2011	Negative
S195	214	JAIVAC-1	Dosage B	3	Cocaine	Day 365	29/09/2011	Negative
S195	214	JAIVAC-1	Dosage B	3	THC	Day 365	29/09/2011	Negative
S202	206	JAIVAC-1	Dosage B		Benzodiazepines	Day -14 to Day -1	23/09/2010	Negative
S202	206	JAIVAC-1	Dosage B		Opioids	Day -14 to Day -1	23/09/2010	Negative
S202	206	JAIVAC-1	Dosage B		Amphetamine	Day -14 to Day -1	23/09/2010	Negative
S202	206	JAIVAC-1	Dosage B		Cocaine	Day -14 to Day -1	23/09/2010	Negative
S202	206	JAIVAC-1	Dosage B		THC	Day -14 to Day -1	23/09/2010	Negative
S202	206	JAIVAC-1	Dosage B	2	Benzodiazepines	Day 28	27/10/2010	Negative
S202	206	JAIVAC-1	Dosage B	2	Opioids	Day 28	27/10/2010	Negative
S202	206	JAIVAC-1	Dosage B	2	Amphetamine	Day 28	27/10/2010	Negative

Subject No.	Randomisation Number	Study Group	Dosage	Dose	Urine Parameters	Day	Date of sample Collection	Results
S202	206	JAIVAC-1	Dosage B	2	Cocaine	Day 28	27/10/2010	Negative
S202	206	JAIVAC-1	Dosage B	2	THC	Day 28	27/10/2010	Negative
S202	206	JAIVAC-1	Dosage B	3	Benzodiazepines	Day 180	28/03/2011	Negative
S202	206	JAIVAC-1	Dosage B	3	Opioids	Day 180	28/03/2011	Negative
S202	206	JAIVAC-1	Dosage B	3	Amphetamine	Day 180	28/03/2011	Negative
S202	206	JAIVAC-1	Dosage B	3	Cocaine	Day 180	28/03/2011	Negative
S202	206	JAIVAC-1	Dosage B	3	THC	Day 180	28/03/2011	Negative
S202	206	JAIVAC-1	Dosage B	3	Benzodiazepines	Day 365	29/09/2011	Negative
S202	206	JAIVAC-1	Dosage B	3	Opioids	Day 365	29/09/2011	Negative
S202	206	JAIVAC-1	Dosage B	3	Amphetamine	Day 365	29/09/2011	Negative
S202	206	JAIVAC-1	Dosage B	3	Cocaine	Day 365	29/09/2011	Negative
S202	206	JAIVAC-1	Dosage B	3	THC	Day 365	29/09/2011	Negative
S203	211	Hepatitis B	Hepatitis B		Benzodiazepines	Day -14 to Day -1	24/09/2010	Negative
S203	211	Hepatitis B	Hepatitis B		Opioids	Day -14 to Day -1	24/09/2010	Negative
S203	211	Hepatitis B	Hepatitis B		Amphetamine	Day -14 to Day -1	24/09/2010	Negative
S203	211	Hepatitis B	Hepatitis B		Cocaine	Day -14 to Day -1	24/09/2010	Negative
S203	211	Hepatitis B	Hepatitis B		THC	Day -14 to Day -1	24/09/2010	Negative
S203	211	Hepatitis B	Hepatitis B	2	Benzodiazepines	Day 28	28/10/2010	Negative
S203	211	Hepatitis B	Hepatitis B	2	Opioids	Day 28	28/10/2010	Negative
S203	211	Hepatitis B	Hepatitis B	2	Amphetamine	Day 28	28/10/2010	Negative
S203	211	Hepatitis B	Hepatitis B	2	Cocaine	Day 28	28/10/2010	Negative
S203	211	Hepatitis B	Hepatitis B	2	THC	Day 28	28/10/2010	Negative
S203	211	Hepatitis B	Hepatitis B	3	Benzodiazepines	Day 180	29/03/2011	Negative
S203	211	Hepatitis B	Hepatitis B	3	Opioids	Day 180	29/03/2011	Negative
S203	211	Hepatitis B	Hepatitis B	3	Amphetamine	Day 180	29/03/2011	Negative
S203	211	Hepatitis B	Hepatitis B	3	Cocaine	Day 180	29/03/2011	Negative
S203	211	Hepatitis B	Hepatitis B	3	THC	Day 180	29/03/2011	Negative
S203	211	Hepatitis B	Hepatitis B	3	Benzodiazepines	Day 365	29/09/2011	Negative
S203	211	Hepatitis B	Hepatitis B	3	Opioids	Day 365	29/09/2011	Negative
S203	211	Hepatitis B	Hepatitis B	3	Amphetamine	Day 365	29/09/2011	Negative

Subject No.	Randomisation Number	Study Group	Dosage	Dose	Urine Parameters	Day	Date of sample Collection	Results
S203	211	Hepatitis B	Hepatitis B	3	Cocaine	Day 365	29/09/2011	Negative
S203	211	Hepatitis B	Hepatitis B	3	THC	Day 365	29/09/2011	Negative
S203	211	Hepatitis B	Hepatitis B		Benzodiazepines	Unscheduled Visit	.	
S203	211	Hepatitis B	Hepatitis B		Opioids	Unscheduled Visit	.	
S203	211	Hepatitis B	Hepatitis B		Amphetamine	Unscheduled Visit	.	
S203	211	Hepatitis B	Hepatitis B		Cocaine	Unscheduled Visit	.	
S203	211	Hepatitis B	Hepatitis B		THC	Unscheduled Visit	.	
S203	211	Hepatitis B	Hepatitis B		Benzodiazepines	Unscheduled Visit	.	
S203	211	Hepatitis B	Hepatitis B		Opioids	Unscheduled Visit	.	
S203	211	Hepatitis B	Hepatitis B		Amphetamine	Unscheduled Visit	.	
S203	211	Hepatitis B	Hepatitis B		Cocaine	Unscheduled Visit	.	
S203	211	Hepatitis B	Hepatitis B		THC	Unscheduled Visit	.	
S203	211	Hepatitis B	Hepatitis B		Benzodiazepines	Unscheduled Visit	.	
S203	211	Hepatitis B	Hepatitis B		Opioids	Unscheduled Visit	.	
S203	211	Hepatitis B	Hepatitis B		Amphetamine	Unscheduled Visit	.	
S203	211	Hepatitis B	Hepatitis B		Cocaine	Unscheduled Visit	.	
S203	211	Hepatitis B	Hepatitis B		THC	Unscheduled Visit	.	
S212	212	JAIVAC-1	Dosage B		Benzodiazepines	Day -14 to Day -1	23/09/2010	Negative
S212	212	JAIVAC-1	Dosage B		Opioids	Day -14 to Day -1	23/09/2010	Negative
S212	212	JAIVAC-1	Dosage B		Amphetamine	Day -14 to Day -1	23/09/2010	Negative
S212	212	JAIVAC-1	Dosage B		Cocaine	Day -14 to Day -1	23/09/2010	Negative
S212	212	JAIVAC-1	Dosage B		THC	Day -14 to Day -1	23/09/2010	Negative
S212	212	JAIVAC-1	Dosage B	2	Benzodiazepines	Day 28	28/10/2010	Negative
S212	212	JAIVAC-1	Dosage B	2	Opioids	Day 28	28/10/2010	Negative
S212	212	JAIVAC-1	Dosage B	2	Amphetamine	Day 28	28/10/2010	Negative
S212	212	JAIVAC-1	Dosage B	2	Cocaine	Day 28	28/10/2010	Negative
S212	212	JAIVAC-1	Dosage B	2	THC	Day 28	28/10/2010	Negative
S212	212	JAIVAC-1	Dosage B	3	Benzodiazepines	Day 180	29/03/2011	Negative
S212	212	JAIVAC-1	Dosage B	3	Opioids	Day 180	29/03/2011	Negative
S212	212	JAIVAC-1	Dosage B	3	Amphetamine	Day 180	29/03/2011	Negative

Subject No.	Randomisation Number	Study Group	Dosage	Dose	Urine Parameters	Day	Date of sample Collection	Results
S212	212	JAIVAC-1	Dosage B	3	Cocaine	Day 180	29/03/2011	Negative
S212	212	JAIVAC-1	Dosage B	3	THC	Day 180	29/03/2011	Negative
S212	212	JAIVAC-1	Dosage B	3	Benzodiazepines	Day 365	29/09/2011	Negative
S212	212	JAIVAC-1	Dosage B	3	Opioids	Day 365	29/09/2011	Negative
S212	212	JAIVAC-1	Dosage B	3	Amphetamine	Day 365	29/09/2011	Negative
S212	212	JAIVAC-1	Dosage B	3	Cocaine	Day 365	29/09/2011	Negative
S212	212	JAIVAC-1	Dosage B	3	THC	Day 365	29/09/2011	Negative
S215	213	Hepatitis B	Hepatitis B		Benzodiazepines	Day -14 to Day -1	24/09/2010	Negative
S215	213	Hepatitis B	Hepatitis B		Opioids	Day -14 to Day -1	24/09/2010	Negative
S215	213	Hepatitis B	Hepatitis B		Amphetamine	Day -14 to Day -1	24/09/2010	Negative
S215	213	Hepatitis B	Hepatitis B		Cocaine	Day -14 to Day -1	24/09/2010	Negative
S215	213	Hepatitis B	Hepatitis B		THC	Day -14 to Day -1	24/09/2010	Negative
S215	213	Hepatitis B	Hepatitis B	2	Benzodiazepines	Day 28	28/10/2010	Negative
S215	213	Hepatitis B	Hepatitis B	2	Opioids	Day 28	28/10/2010	Negative
S215	213	Hepatitis B	Hepatitis B	2	Amphetamine	Day 28	28/10/2010	Negative
S215	213	Hepatitis B	Hepatitis B	2	Cocaine	Day 28	28/10/2010	Negative
S215	213	Hepatitis B	Hepatitis B	2	THC	Day 28	28/10/2010	Negative
S215	213	Hepatitis B	Hepatitis B	3	Benzodiazepines	Day 180	29/03/2011	Negative
S215	213	Hepatitis B	Hepatitis B	3	Opioids	Day 180	29/03/2011	Negative
S215	213	Hepatitis B	Hepatitis B	3	Amphetamine	Day 180	29/03/2011	Negative
S215	213	Hepatitis B	Hepatitis B	3	Cocaine	Day 180	29/03/2011	Negative
S215	213	Hepatitis B	Hepatitis B	3	THC	Day 180	29/03/2011	Negative
S215	213	Hepatitis B	Hepatitis B	3	Benzodiazepines	Day 365	29/09/2011	Negative
S215	213	Hepatitis B	Hepatitis B	3	Opioids	Day 365	29/09/2011	Negative
S215	213	Hepatitis B	Hepatitis B	3	Amphetamine	Day 365	29/09/2011	Negative
S215	213	Hepatitis B	Hepatitis B	3	Cocaine	Day 365	29/09/2011	Negative
S215	213	Hepatitis B	Hepatitis B	3	THC	Day 365	29/09/2011	Negative
S233	210	JAIVAC-1	Dosage B		Benzodiazepines	Day -14 to Day -1	24/09/2010	Negative
S233	210	JAIVAC-1	Dosage B		Opioids	Day -14 to Day -1	24/09/2010	Negative
S233	210	JAIVAC-1	Dosage B		Amphetamine	Day -14 to Day -1	24/09/2010	Negative

Subject No.	Randomisation Number	Study Group	Dosage	Dose	Urine Parameters	Day	Date of sample Collection	Results
S233	210	JAIVAC-1	Dosage B		Cocaine	Day -14 to Day -1	24/09/2010	Negative
S233	210	JAIVAC-1	Dosage B		THC	Day -14 to Day -1	24/09/2010	Negative
S233	210	JAIVAC-1	Dosage B	2	Benzodiazepines	Day 28	28/10/2010	Negative
S233	210	JAIVAC-1	Dosage B	2	Opioids	Day 28	28/10/2010	Negative
S233	210	JAIVAC-1	Dosage B	2	Amphetamine	Day 28	28/10/2010	Negative
S233	210	JAIVAC-1	Dosage B	2	Cocaine	Day 28	28/10/2010	Negative
S233	210	JAIVAC-1	Dosage B	2	THC	Day 28	28/10/2010	Negative
S233	210	JAIVAC-1	Dosage B	3	Benzodiazepines	Day 180	29/03/2011	Negative
S233	210	JAIVAC-1	Dosage B	3	Opioids	Day 180	29/03/2011	Negative
S233	210	JAIVAC-1	Dosage B	3	Amphetamine	Day 180	29/03/2011	Negative
S233	210	JAIVAC-1	Dosage B	3	Cocaine	Day 180	29/03/2011	Negative
S233	210	JAIVAC-1	Dosage B	3	THC	Day 180	29/03/2011	Negative
S233	210	JAIVAC-1	Dosage B	3	Benzodiazepines	Day 365	29/09/2011	Negative
S233	210	JAIVAC-1	Dosage B	3	Opioids	Day 365	29/09/2011	Negative
S233	210	JAIVAC-1	Dosage B	3	Amphetamine	Day 365	29/09/2011	Negative
S233	210	JAIVAC-1	Dosage B	3	Cocaine	Day 365	29/09/2011	Negative
S233	210	JAIVAC-1	Dosage B	3	THC	Day 365	29/09/2011	Negative
S241	303	Hepatitis B	Hepatitis B		Benzodiazepines	Day -14 to Day -1	03/11/2010	Negative
S241	303	Hepatitis B	Hepatitis B		Opioids	Day -14 to Day -1	03/11/2010	Negative
S241	303	Hepatitis B	Hepatitis B		Amphetamine	Day -14 to Day -1	03/11/2010	Negative
S241	303	Hepatitis B	Hepatitis B		Cocaine	Day -14 to Day -1	03/11/2010	Negative
S241	303	Hepatitis B	Hepatitis B		THC	Day -14 to Day -1	03/11/2010	Negative
S241	303	Hepatitis B	Hepatitis B	2	Benzodiazepines	Day 28	08/12/2010	Negative
S241	303	Hepatitis B	Hepatitis B	2	Opioids	Day 28	08/12/2010	Negative
S241	303	Hepatitis B	Hepatitis B	2	Amphetamine	Day 28	08/12/2010	Negative
S241	303	Hepatitis B	Hepatitis B	2	Cocaine	Day 28	08/12/2010	Negative
S241	303	Hepatitis B	Hepatitis B	2	THC	Day 28	08/12/2010	Negative
S241	303	Hepatitis B	Hepatitis B	3	Benzodiazepines	Day 180	09/05/2011	Negative
S241	303	Hepatitis B	Hepatitis B	3	Opioids	Day 180	09/05/2011	Negative
S241	303	Hepatitis B	Hepatitis B	3	Amphetamine	Day 180	09/05/2011	Negative

Subject No.	Randomisation Number	Study Group	Dosage	Dose	Urine Parameters	Day	Date of sample Collection	Results
S241	303	Hepatitis B	Hepatitis B	3	Cocaine	Day 180	09/05/2011	Negative
S241	303	Hepatitis B	Hepatitis B	3	THC	Day 180	09/05/2011	Negative
S241	303	Hepatitis B	Hepatitis B	3	Benzodiazepines	Day 365	09/11/2011	Negative
S241	303	Hepatitis B	Hepatitis B	3	Opioids	Day 365	09/11/2011	Negative
S241	303	Hepatitis B	Hepatitis B	3	Amphetamine	Day 365	09/11/2011	Negative
S241	303	Hepatitis B	Hepatitis B	3	Cocaine	Day 365	09/11/2011	Negative
S241	303	Hepatitis B	Hepatitis B	3	THC	Day 365	09/11/2011	Negative
S250	311	JAIVAC-1	Dosage C		Benzodiazepines	Day -14 to Day -1	03/11/2010	Negative
S250	311	JAIVAC-1	Dosage C		Opioids	Day -14 to Day -1	03/11/2010	Negative
S250	311	JAIVAC-1	Dosage C		Amphetamine	Day -14 to Day -1	03/11/2010	Negative
S250	311	JAIVAC-1	Dosage C		Cocaine	Day -14 to Day -1	03/11/2010	Negative
S250	311	JAIVAC-1	Dosage C		THC	Day -14 to Day -1	03/11/2010	Negative
S250	311	JAIVAC-1	Dosage C	2	Benzodiazepines	Day 28	10/12/2010	Negative
S250	311	JAIVAC-1	Dosage C	2	Opioids	Day 28	10/12/2010	Negative
S250	311	JAIVAC-1	Dosage C	2	Amphetamine	Day 28	10/12/2010	Negative
S250	311	JAIVAC-1	Dosage C	2	Cocaine	Day 28	10/12/2010	Negative
S250	311	JAIVAC-1	Dosage C	2	THC	Day 28	10/12/2010	Negative
S250	311	JAIVAC-1	Dosage C	3	Benzodiazepines	Day 180	11/05/2011	Negative
S250	311	JAIVAC-1	Dosage C	3	Opioids	Day 180	11/05/2011	Negative
S250	311	JAIVAC-1	Dosage C	3	Amphetamine	Day 180	11/05/2011	Negative
S250	311	JAIVAC-1	Dosage C	3	Cocaine	Day 180	11/05/2011	Negative
S250	311	JAIVAC-1	Dosage C	3	THC	Day 180	11/05/2011	Negative
S250	311	JAIVAC-1	Dosage C	3	Benzodiazepines	Day 365	10/11/2011	Negative
S250	311	JAIVAC-1	Dosage C	3	Opioids	Day 365	10/11/2011	Negative
S250	311	JAIVAC-1	Dosage C	3	Amphetamine	Day 365	10/11/2011	Negative
S250	311	JAIVAC-1	Dosage C	3	Cocaine	Day 365	10/11/2011	Negative
S250	311	JAIVAC-1	Dosage C	3	THC	Day 365	10/11/2011	Negative
S263	313	Hepatitis B	Hepatitis B		Benzodiazepines	Day -14 to Day -1	09/11/2010	Negative
S263	313	Hepatitis B	Hepatitis B		Opioids	Day -14 to Day -1	09/11/2010	Negative
S263	313	Hepatitis B	Hepatitis B		Amphetamine	Day -14 to Day -1	09/11/2010	Negative

Subject No.	Randomisation Number	Study Group	Dosage	Dose	Urine Parameters	Day	Date of sample Collection	Results
S263	313	Hepatitis B	Hepatitis B		Cocaine	Day -14 to Day -1	09/11/2010	Negative
S263	313	Hepatitis B	Hepatitis B		THC	Day -14 to Day -1	09/11/2010	Negative
S263	313	Hepatitis B	Hepatitis B	2	Benzodiazepines	Day 28	10/12/2010	Negative
S263	313	Hepatitis B	Hepatitis B	2	Opioids	Day 28	10/12/2010	Negative
S263	313	Hepatitis B	Hepatitis B	2	Amphetamine	Day 28	10/12/2010	Negative
S263	313	Hepatitis B	Hepatitis B	2	Cocaine	Day 28	10/12/2010	Negative
S263	313	Hepatitis B	Hepatitis B	2	THC	Day 28	10/12/2010	Negative
S263	313	Hepatitis B	Hepatitis B	3	Benzodiazepines	Day 180	11/05/2011	Negative
S263	313	Hepatitis B	Hepatitis B	3	Opioids	Day 180	11/05/2011	Negative
S263	313	Hepatitis B	Hepatitis B	3	Amphetamine	Day 180	11/05/2011	Negative
S263	313	Hepatitis B	Hepatitis B	3	Cocaine	Day 180	11/05/2011	Negative
S263	313	Hepatitis B	Hepatitis B	3	THC	Day 180	11/05/2011	Negative
S263	313	Hepatitis B	Hepatitis B	3	Benzodiazepines	Day 365	10/11/2011	Negative
S263	313	Hepatitis B	Hepatitis B	3	Opioids	Day 365	10/11/2011	Negative
S263	313	Hepatitis B	Hepatitis B	3	Amphetamine	Day 365	10/11/2011	Negative
S263	313	Hepatitis B	Hepatitis B	3	Cocaine	Day 365	10/11/2011	Negative
S263	313	Hepatitis B	Hepatitis B	3	THC	Day 365	10/11/2011	Negative
S263	313	Hepatitis B	Hepatitis B		Benzodiazepines	Unscheduled Visit	.	
S263	313	Hepatitis B	Hepatitis B		Opioids	Unscheduled Visit	.	
S263	313	Hepatitis B	Hepatitis B		Amphetamine	Unscheduled Visit	.	
S263	313	Hepatitis B	Hepatitis B		Cocaine	Unscheduled Visit	.	
S263	313	Hepatitis B	Hepatitis B		THC	Unscheduled Visit	.	
S264	306	JAIVAC-1	Dosage C		Benzodiazepines	Day -14 to Day -1	03/11/2010	Negative
S264	306	JAIVAC-1	Dosage C		Opioids	Day -14 to Day -1	03/11/2010	Negative
S264	306	JAIVAC-1	Dosage C		Amphetamine	Day -14 to Day -1	03/11/2010	Negative
S264	306	JAIVAC-1	Dosage C		Cocaine	Day -14 to Day -1	03/11/2010	Negative
S264	306	JAIVAC-1	Dosage C		THC	Day -14 to Day -1	03/11/2010	Negative
S264	306	JAIVAC-1	Dosage C	2	Benzodiazepines	Day 28	09/12/2010	Negative
S264	306	JAIVAC-1	Dosage C	2	Opioids	Day 28	09/12/2010	Negative
S264	306	JAIVAC-1	Dosage C	2	Amphetamine	Day 28	09/12/2010	Negative

Subject No.	Randomisation Number	Study Group	Dosage	Dose	Urine Parameters	Day	Date of sample Collection	Results
S264	306	JAIVAC-1	Dosage C	2	Cocaine	Day 28	09/12/2010	Negative
S264	306	JAIVAC-1	Dosage C	2	THC	Day 28	09/12/2010	Negative
S264	306	JAIVAC-1	Dosage C	3	Benzodiazepines	Day 180	10/05/2011	Negative
S264	306	JAIVAC-1	Dosage C	3	Opioids	Day 180	10/05/2011	Negative
S264	306	JAIVAC-1	Dosage C	3	Amphetamine	Day 180	10/05/2011	Negative
S264	306	JAIVAC-1	Dosage C	3	Cocaine	Day 180	10/05/2011	Negative
S264	306	JAIVAC-1	Dosage C	3	THC	Day 180	10/05/2011	Negative
S264	306	JAIVAC-1	Dosage C	3	Benzodiazepines	Day 365	09/11/2011	Negative
S264	306	JAIVAC-1	Dosage C	3	Opioids	Day 365	09/11/2011	Negative
S264	306	JAIVAC-1	Dosage C	3	Amphetamine	Day 365	09/11/2011	Negative
S264	306	JAIVAC-1	Dosage C	3	Cocaine	Day 365	09/11/2011	Negative
S264	306	JAIVAC-1	Dosage C	3	THC	Day 365	09/11/2011	Negative
S266	308	JAIVAC-1	Dosage C		Benzodiazepines	Day -14 to Day -1	03/11/2010	Negative
S266	308	JAIVAC-1	Dosage C		Opioids	Day -14 to Day -1	03/11/2010	Negative
S266	308	JAIVAC-1	Dosage C		Amphetamine	Day -14 to Day -1	03/11/2010	Negative
S266	308	JAIVAC-1	Dosage C		Cocaine	Day -14 to Day -1	03/11/2010	Negative
S266	308	JAIVAC-1	Dosage C		THC	Day -14 to Day -1	03/11/2010	Negative
S266	308	JAIVAC-1	Dosage C	2	Benzodiazepines	Day 28	09/12/2010	Negative
S266	308	JAIVAC-1	Dosage C	2	Opioids	Day 28	09/12/2010	Negative
S266	308	JAIVAC-1	Dosage C	2	Amphetamine	Day 28	09/12/2010	Negative
S266	308	JAIVAC-1	Dosage C	2	Cocaine	Day 28	09/12/2010	Negative
S266	308	JAIVAC-1	Dosage C	2	THC	Day 28	09/12/2010	Negative
S266	308	JAIVAC-1	Dosage C	3	Benzodiazepines	Day 180	10/05/2011	Negative
S266	308	JAIVAC-1	Dosage C	3	Opioids	Day 180	10/05/2011	Negative
S266	308	JAIVAC-1	Dosage C	3	Amphetamine	Day 180	10/05/2011	Negative
S266	308	JAIVAC-1	Dosage C	3	Cocaine	Day 180	10/05/2011	Negative
S266	308	JAIVAC-1	Dosage C	3	THC	Day 180	10/05/2011	Negative
S266	308	JAIVAC-1	Dosage C	3	Benzodiazepines	Day 365	09/11/2011	Negative
S266	308	JAIVAC-1	Dosage C	3	Opioids	Day 365	09/11/2011	Negative
S266	308	JAIVAC-1	Dosage C	3	Amphetamine	Day 365	09/11/2011	Negative

Subject No.	Randomisation Number	Study Group	Dosage	Dose	Urine Parameters	Day	Date of sample Collection	Results
S266	308	JAIVAC-1	Dosage C	3	Cocaine	Day 365	09/11/2011	Negative
S266	308	JAIVAC-1	Dosage C	3	THC	Day 365	09/11/2011	Negative
S266	308	JAIVAC-1	Dosage C		Benzodiazepines	Unscheduled Visit	.	
S266	308	JAIVAC-1	Dosage C		Opioids	Unscheduled Visit	.	
S266	308	JAIVAC-1	Dosage C		Amphetamine	Unscheduled Visit	.	
S266	308	JAIVAC-1	Dosage C		Cocaine	Unscheduled Visit	.	
S266	308	JAIVAC-1	Dosage C		THC	Unscheduled Visit	.	
S266	308	JAIVAC-1	Dosage C		Benzodiazepines	Unscheduled Visit	.	
S266	308	JAIVAC-1	Dosage C		Opioids	Unscheduled Visit	.	
S266	308	JAIVAC-1	Dosage C		Amphetamine	Unscheduled Visit	.	
S266	308	JAIVAC-1	Dosage C		Cocaine	Unscheduled Visit	.	
S266	308	JAIVAC-1	Dosage C		THC	Unscheduled Visit	.	
S266	308	JAIVAC-1	Dosage C		Benzodiazepines	Unscheduled Visit	.	
S266	308	JAIVAC-1	Dosage C		Opioids	Unscheduled Visit	.	
S266	308	JAIVAC-1	Dosage C		Amphetamine	Unscheduled Visit	.	
S266	308	JAIVAC-1	Dosage C		Cocaine	Unscheduled Visit	.	
S266	308	JAIVAC-1	Dosage C		THC	Unscheduled Visit	.	
S267	309	Hepatitis B	Hepatitis B		Benzodiazepines	Day -14 to Day -1	02/11/2010	Negative
S267	309	Hepatitis B	Hepatitis B		Opioids	Day -14 to Day -1	02/11/2010	Negative
S267	309	Hepatitis B	Hepatitis B		Amphetamine	Day -14 to Day -1	02/11/2010	Negative
S267	309	Hepatitis B	Hepatitis B		Cocaine	Day -14 to Day -1	02/11/2010	Negative
S267	309	Hepatitis B	Hepatitis B		THC	Day -14 to Day -1	02/11/2010	Negative
S267	309	Hepatitis B	Hepatitis B	2	Benzodiazepines	Day 28	09/12/2010	Negative
S267	309	Hepatitis B	Hepatitis B	2	Opioids	Day 28	09/12/2010	Negative
S267	309	Hepatitis B	Hepatitis B	2	Amphetamine	Day 28	09/12/2010	Negative
S267	309	Hepatitis B	Hepatitis B	2	Cocaine	Day 28	09/12/2010	Negative
S267	309	Hepatitis B	Hepatitis B	2	THC	Day 28	09/12/2010	Negative
S267	309	Hepatitis B	Hepatitis B	3	Benzodiazepines	Day 180	10/05/2011	Negative
S267	309	Hepatitis B	Hepatitis B	3	Opioids	Day 180	10/05/2011	Negative
S267	309	Hepatitis B	Hepatitis B	3	Amphetamine	Day 180	10/05/2011	Negative

Subject No.	Randomisation Number	Study Group	Dosage	Dose	Urine Parameters	Day	Date of sample Collection	Results
S267	309	Hepatitis B	Hepatitis B	3	Cocaine	Day 180	10/05/2011	Negative
S267	309	Hepatitis B	Hepatitis B	3	THC	Day 180	10/05/2011	Negative
S267	309	Hepatitis B	Hepatitis B	3	Benzodiazepines	Day 365	09/11/2011	Negative
S267	309	Hepatitis B	Hepatitis B	3	Opioids	Day 365	09/11/2011	Negative
S267	309	Hepatitis B	Hepatitis B	3	Amphetamine	Day 365	09/11/2011	Negative
S267	309	Hepatitis B	Hepatitis B	3	Cocaine	Day 365	09/11/2011	Negative
S267	309	Hepatitis B	Hepatitis B	3	THC	Day 365	09/11/2011	Negative
S267	309	Hepatitis B	Hepatitis B		Benzodiazepines	Unscheduled Visit	.	
S267	309	Hepatitis B	Hepatitis B		Opioids	Unscheduled Visit	.	
S267	309	Hepatitis B	Hepatitis B		Amphetamine	Unscheduled Visit	.	
S267	309	Hepatitis B	Hepatitis B		Cocaine	Unscheduled Visit	.	
S267	309	Hepatitis B	Hepatitis B		THC	Unscheduled Visit	.	
S275	302	Hepatitis B	Hepatitis B		Benzodiazepines	Day -14 to Day -1	02/11/2010	Negative
S275	302	Hepatitis B	Hepatitis B		Opioids	Day -14 to Day -1	02/11/2010	Negative
S275	302	Hepatitis B	Hepatitis B		Amphetamine	Day -14 to Day -1	02/11/2010	Negative
S275	302	Hepatitis B	Hepatitis B		Cocaine	Day -14 to Day -1	02/11/2010	Negative
S275	302	Hepatitis B	Hepatitis B		THC	Day -14 to Day -1	02/11/2010	Negative
S275	302	Hepatitis B	Hepatitis B	2	Benzodiazepines	Day 28	08/12/2010	Negative
S275	302	Hepatitis B	Hepatitis B	2	Opioids	Day 28	08/12/2010	Negative
S275	302	Hepatitis B	Hepatitis B	2	Amphetamine	Day 28	08/12/2010	Negative
S275	302	Hepatitis B	Hepatitis B	2	Cocaine	Day 28	08/12/2010	Negative
S275	302	Hepatitis B	Hepatitis B	2	THC	Day 28	08/12/2010	Negative
S275	302	Hepatitis B	Hepatitis B	3	Benzodiazepines	Day 180	09/05/2011	Negative
S275	302	Hepatitis B	Hepatitis B	3	Opioids	Day 180	09/05/2011	Negative
S275	302	Hepatitis B	Hepatitis B	3	Amphetamine	Day 180	09/05/2011	Negative
S275	302	Hepatitis B	Hepatitis B	3	Cocaine	Day 180	09/05/2011	Negative
S275	302	Hepatitis B	Hepatitis B	3	THC	Day 180	09/05/2011	Negative
S275	302	Hepatitis B	Hepatitis B	3	Benzodiazepines	Day 365	09/11/2011	Negative
S275	302	Hepatitis B	Hepatitis B	3	Opioids	Day 365	09/11/2011	Negative
S275	302	Hepatitis B	Hepatitis B	3	Amphetamine	Day 365	09/11/2011	Negative

Subject No.	Randomisation Number	Study Group	Dosage	Dose	Urine Parameters	Day	Date of sample Collection	Results
S275	302	Hepatitis B	Hepatitis B	3	Cocaine	Day 365	09/11/2011	Negative
S275	302	Hepatitis B	Hepatitis B	3	THC	Day 365	09/11/2011	Negative
S278	315	JAIVAC-1	Dosage C		Benzodiazepines	Day -14 to Day -1	08/11/2010	Negative
S278	315	JAIVAC-1	Dosage C		Opioids	Day -14 to Day -1	08/11/2010	Negative
S278	315	JAIVAC-1	Dosage C		Amphetamine	Day -14 to Day -1	08/11/2010	Negative
S278	315	JAIVAC-1	Dosage C		Cocaine	Day -14 to Day -1	08/11/2010	Negative
S278	315	JAIVAC-1	Dosage C		THC	Day -14 to Day -1	08/11/2010	Negative
S278	315	JAIVAC-1	Dosage C	2	Benzodiazepines	Day 28	10/12/2010	Negative
S278	315	JAIVAC-1	Dosage C	2	Opioids	Day 28	10/12/2010	Negative
S278	315	JAIVAC-1	Dosage C	2	Amphetamine	Day 28	10/12/2010	Negative
S278	315	JAIVAC-1	Dosage C	2	Cocaine	Day 28	10/12/2010	Negative
S278	315	JAIVAC-1	Dosage C	2	THC	Day 28	10/12/2010	Negative
S278	315	JAIVAC-1	Dosage C	3	Benzodiazepines	Day 180	11/05/2011	Negative
S278	315	JAIVAC-1	Dosage C	3	Opioids	Day 180	11/05/2011	Negative
S278	315	JAIVAC-1	Dosage C	3	Amphetamine	Day 180	11/05/2011	Negative
S278	315	JAIVAC-1	Dosage C	3	Cocaine	Day 180	11/05/2011	Negative
S278	315	JAIVAC-1	Dosage C	3	THC	Day 180	11/05/2011	Negative
S278	315	JAIVAC-1	Dosage C	3	Benzodiazepines	Day 365	10/11/2011	Negative
S278	315	JAIVAC-1	Dosage C	3	Opioids	Day 365	10/11/2011	Negative
S278	315	JAIVAC-1	Dosage C	3	Amphetamine	Day 365	10/11/2011	Negative
S278	315	JAIVAC-1	Dosage C	3	Cocaine	Day 365	10/11/2011	Negative
S278	315	JAIVAC-1	Dosage C	3	THC	Day 365	10/11/2011	Negative
S279	301	JAIVAC-1	Dosage C		Benzodiazepines	Day -14 to Day -1	03/11/2010	Negative
S279	301	JAIVAC-1	Dosage C		Opioids	Day -14 to Day -1	03/11/2010	Negative
S279	301	JAIVAC-1	Dosage C		Amphetamine	Day -14 to Day -1	03/11/2010	Negative
S279	301	JAIVAC-1	Dosage C		Cocaine	Day -14 to Day -1	03/11/2010	Negative
S279	301	JAIVAC-1	Dosage C		THC	Day -14 to Day -1	03/11/2010	Negative
S279	301	JAIVAC-1	Dosage C	2	Benzodiazepines	Day 28	08/12/2010	Negative
S279	301	JAIVAC-1	Dosage C	2	Opioids	Day 28	08/12/2010	Negative
S279	301	JAIVAC-1	Dosage C	2	Amphetamine	Day 28	08/12/2010	Negative

Subject No.	Randomisation Number	Study Group	Dosage	Dose	Urine Parameters	Day	Date of sample Collection	Results
S279	301	JAIVAC-1	Dosage C	2	Cocaine	Day 28	08/12/2010	Negative
S279	301	JAIVAC-1	Dosage C	2	THC	Day 28	08/12/2010	Negative
S279	301	JAIVAC-1	Dosage C	3	Benzodiazepines	Day 180	09/05/2011	Negative
S279	301	JAIVAC-1	Dosage C	3	Opioids	Day 180	09/05/2011	Negative
S279	301	JAIVAC-1	Dosage C	3	Amphetamine	Day 180	09/05/2011	Negative
S279	301	JAIVAC-1	Dosage C	3	Cocaine	Day 180	09/05/2011	Negative
S279	301	JAIVAC-1	Dosage C	3	THC	Day 180	09/05/2011	Negative
S279	301	JAIVAC-1	Dosage C	3	Benzodiazepines	Day 365	09/11/2011	Negative
S279	301	JAIVAC-1	Dosage C	3	Opioids	Day 365	09/11/2011	Negative
S279	301	JAIVAC-1	Dosage C	3	Amphetamine	Day 365	09/11/2011	Negative
S279	301	JAIVAC-1	Dosage C	3	Cocaine	Day 365	09/11/2011	Negative
S279	301	JAIVAC-1	Dosage C	3	THC	Day 365	09/11/2011	Negative
S281	304	JAIVAC-1	Dosage C		Benzodiazepines	Day -14 to Day -1	03/11/2010	Negative
S281	304	JAIVAC-1	Dosage C		Opioids	Day -14 to Day -1	03/11/2010	Negative
S281	304	JAIVAC-1	Dosage C		Amphetamine	Day -14 to Day -1	03/11/2010	Negative
S281	304	JAIVAC-1	Dosage C		Cocaine	Day -14 to Day -1	03/11/2010	Negative
S281	304	JAIVAC-1	Dosage C		THC	Day -14 to Day -1	03/11/2010	Negative
S281	304	JAIVAC-1	Dosage C	2	Benzodiazepines	Day 28	09/12/2010	Negative
S281	304	JAIVAC-1	Dosage C	2	Opioids	Day 28	09/12/2010	Negative
S281	304	JAIVAC-1	Dosage C	2	Amphetamine	Day 28	09/12/2010	Negative
S281	304	JAIVAC-1	Dosage C	2	Cocaine	Day 28	09/12/2010	Negative
S281	304	JAIVAC-1	Dosage C	2	THC	Day 28	09/12/2010	Negative
S281	304	JAIVAC-1	Dosage C		Benzodiazepines	Early Termination	11/05/2011	Negative
S281	304	JAIVAC-1	Dosage C		Opioids	Early Termination	11/05/2011	Negative
S281	304	JAIVAC-1	Dosage C		Amphetamine	Early Termination	11/05/2011	Negative
S281	304	JAIVAC-1	Dosage C		Cocaine	Early Termination	11/05/2011	Negative
S281	304	JAIVAC-1	Dosage C		THC	Early Termination	11/05/2011	Negative
S284	305	JAIVAC-1	Dosage C		Benzodiazepines	Day -14 to Day -1	03/11/2010	Negative
S284	305	JAIVAC-1	Dosage C		Opioids	Day -14 to Day -1	03/11/2010	Negative
S284	305	JAIVAC-1	Dosage C		Amphetamine	Day -14 to Day -1	03/11/2010	Negative

Subject No.	Randomisation Number	Study Group	Dosage	Dose	Urine Parameters	Day	Date of sample Collection	Results
S284	305	JAIVAC-1	Dosage C		Cocaine	Day -14 to Day -1	03/11/2010	Negative
S284	305	JAIVAC-1	Dosage C		THC	Day -14 to Day -1	03/11/2010	Negative
S284	305	JAIVAC-1	Dosage C	2	Benzodiazepines	Day 28	09/12/2010	Negative
S284	305	JAIVAC-1	Dosage C	2	Opioids	Day 28	09/12/2010	Negative
S284	305	JAIVAC-1	Dosage C	2	Amphetamine	Day 28	09/12/2010	Negative
S284	305	JAIVAC-1	Dosage C	2	Cocaine	Day 28	09/12/2010	Negative
S284	305	JAIVAC-1	Dosage C	2	THC	Day 28	09/12/2010	Negative
S284	305	JAIVAC-1	Dosage C	3	Benzodiazepines	Day 180	10/05/2011	Negative
S284	305	JAIVAC-1	Dosage C	3	Opioids	Day 180	10/05/2011	Negative
S284	305	JAIVAC-1	Dosage C	3	Amphetamine	Day 180	10/05/2011	Negative
S284	305	JAIVAC-1	Dosage C	3	Cocaine	Day 180	10/05/2011	Negative
S284	305	JAIVAC-1	Dosage C	3	THC	Day 180	10/05/2011	Negative
S284	305	JAIVAC-1	Dosage C	3	Benzodiazepines	Day 365	09/11/2011	Negative
S284	305	JAIVAC-1	Dosage C	3	Opioids	Day 365	09/11/2011	Negative
S284	305	JAIVAC-1	Dosage C	3	Amphetamine	Day 365	09/11/2011	Negative
S284	305	JAIVAC-1	Dosage C	3	Cocaine	Day 365	09/11/2011	Negative
S284	305	JAIVAC-1	Dosage C	3	THC	Day 365	09/11/2011	Negative
S285	307	Hepatitis B	Hepatitis B		Benzodiazepines	Day -14 to Day -1	02/11/2010	Negative
S285	307	Hepatitis B	Hepatitis B		Opioids	Day -14 to Day -1	02/11/2010	Negative
S285	307	Hepatitis B	Hepatitis B		Amphetamine	Day -14 to Day -1	02/11/2010	Negative
S285	307	Hepatitis B	Hepatitis B		Cocaine	Day -14 to Day -1	02/11/2010	Negative
S285	307	Hepatitis B	Hepatitis B		THC	Day -14 to Day -1	02/11/2010	Negative
S285	307	Hepatitis B	Hepatitis B	2	Benzodiazepines	Day 28	09/12/2010	Negative
S285	307	Hepatitis B	Hepatitis B	2	Opioids	Day 28	09/12/2010	Negative
S285	307	Hepatitis B	Hepatitis B	2	Amphetamine	Day 28	09/12/2010	Negative
S285	307	Hepatitis B	Hepatitis B	2	Cocaine	Day 28	09/12/2010	Negative
S285	307	Hepatitis B	Hepatitis B	2	THC	Day 28	09/12/2010	Negative
S285	307	Hepatitis B	Hepatitis B	3	Benzodiazepines	Day 180	10/05/2011	Negative
S285	307	Hepatitis B	Hepatitis B	3	Opioids	Day 180	10/05/2011	Negative
S285	307	Hepatitis B	Hepatitis B	3	Amphetamine	Day 180	10/05/2011	Negative

Subject No.	Randomisation Number	Study Group	Dosage	Dose	Urine Parameters	Day	Date of sample Collection	Results
S285	307	Hepatitis B	Hepatitis B	3	Cocaine	Day 180	10/05/2011	Negative
S285	307	Hepatitis B	Hepatitis B	3	THC	Day 180	10/05/2011	Negative
S285	307	Hepatitis B	Hepatitis B	3	Benzodiazepines	Day 365	09/11/2011	Negative
S285	307	Hepatitis B	Hepatitis B	3	Opioids	Day 365	09/11/2011	Negative
S285	307	Hepatitis B	Hepatitis B	3	Amphetamine	Day 365	09/11/2011	Negative
S285	307	Hepatitis B	Hepatitis B	3	Cocaine	Day 365	09/11/2011	Negative
S285	307	Hepatitis B	Hepatitis B	3	THC	Day 365	09/11/2011	Negative
S291	312	JAIVAC-1	Dosage C		Benzodiazepines	Day -14 to Day -1	02/11/2010	Negative
S291	312	JAIVAC-1	Dosage C		Opioids	Day -14 to Day -1	02/11/2010	Negative
S291	312	JAIVAC-1	Dosage C		Amphetamine	Day -14 to Day -1	02/11/2010	Negative
S291	312	JAIVAC-1	Dosage C		Cocaine	Day -14 to Day -1	02/11/2010	Negative
S291	312	JAIVAC-1	Dosage C		THC	Day -14 to Day -1	02/11/2010	Negative
S291	312	JAIVAC-1	Dosage C	2	Benzodiazepines	Day 28	10/12/2010	Negative
S291	312	JAIVAC-1	Dosage C	2	Opioids	Day 28	10/12/2010	Negative
S291	312	JAIVAC-1	Dosage C	2	Amphetamine	Day 28	10/12/2010	Negative
S291	312	JAIVAC-1	Dosage C	2	Cocaine	Day 28	10/12/2010	Negative
S291	312	JAIVAC-1	Dosage C	2	THC	Day 28	10/12/2010	Negative
S291	312	JAIVAC-1	Dosage C	3	Benzodiazepines	Day 180	11/05/2011	Negative
S291	312	JAIVAC-1	Dosage C	3	Opioids	Day 180	11/05/2011	Negative
S291	312	JAIVAC-1	Dosage C	3	Amphetamine	Day 180	11/05/2011	Negative
S291	312	JAIVAC-1	Dosage C	3	Cocaine	Day 180	11/05/2011	Negative
S291	312	JAIVAC-1	Dosage C	3	THC	Day 180	11/05/2011	Negative
S291	312	JAIVAC-1	Dosage C	3	Benzodiazepines	Day 365	10/11/2011	Negative
S291	312	JAIVAC-1	Dosage C	3	Opioids	Day 365	10/11/2011	Negative
S291	312	JAIVAC-1	Dosage C	3	Amphetamine	Day 365	10/11/2011	Negative
S291	312	JAIVAC-1	Dosage C	3	Cocaine	Day 365	10/11/2011	Negative
S291	312	JAIVAC-1	Dosage C	3	THC	Day 365	10/11/2011	Negative
S298	314	JAIVAC-1	Dosage C		Benzodiazepines	Day -14 to Day -1	09/11/2010	Negative
S298	314	JAIVAC-1	Dosage C		Opioids	Day -14 to Day -1	09/11/2010	Negative
S298	314	JAIVAC-1	Dosage C		Amphetamine	Day -14 to Day -1	09/11/2010	Negative

Subject No.	Randomisation Number	Study Group	Dosage	Dose	Urine Parameters	Day	Date of sample Collection	Results
S298	314	JAIVAC-1	Dosage C		Cocaine	Day -14 to Day -1	09/11/2010	Negative
S298	314	JAIVAC-1	Dosage C		THC	Day -14 to Day -1	09/11/2010	Negative
S298	314	JAIVAC-1	Dosage C	2	Benzodiazepines	Day 28	10/12/2010	Negative
S298	314	JAIVAC-1	Dosage C	2	Opioids	Day 28	10/12/2010	Negative
S298	314	JAIVAC-1	Dosage C	2	Amphetamine	Day 28	10/12/2010	Negative
S298	314	JAIVAC-1	Dosage C	2	Cocaine	Day 28	10/12/2010	Negative
S298	314	JAIVAC-1	Dosage C	2	THC	Day 28	10/12/2010	Negative
S298	314	JAIVAC-1	Dosage C	3	Benzodiazepines	Day 180	11/05/2011	Negative
S298	314	JAIVAC-1	Dosage C	3	Opioids	Day 180	11/05/2011	Negative
S298	314	JAIVAC-1	Dosage C	3	Amphetamine	Day 180	11/05/2011	Negative
S298	314	JAIVAC-1	Dosage C	3	Cocaine	Day 180	11/05/2011	Negative
S298	314	JAIVAC-1	Dosage C	3	THC	Day 180	11/05/2011	Negative
S298	314	JAIVAC-1	Dosage C	3	Benzodiazepines	Day 365	10/11/2011	Negative
S298	314	JAIVAC-1	Dosage C	3	Opioids	Day 365	10/11/2011	Negative
S298	314	JAIVAC-1	Dosage C	3	Amphetamine	Day 365	10/11/2011	Negative
S298	314	JAIVAC-1	Dosage C	3	Cocaine	Day 365	10/11/2011	Negative
S298	314	JAIVAC-1	Dosage C	3	THC	Day 365	10/11/2011	Negative
S299	310	JAIVAC-1	Dosage C		Benzodiazepines	Day -14 to Day -1	08/11/2010	Negative
S299	310	JAIVAC-1	Dosage C		Opioids	Day -14 to Day -1	08/11/2010	Negative
S299	310	JAIVAC-1	Dosage C		Amphetamine	Day -14 to Day -1	08/11/2010	Negative
S299	310	JAIVAC-1	Dosage C		Cocaine	Day -14 to Day -1	08/11/2010	Negative
S299	310	JAIVAC-1	Dosage C		THC	Day -14 to Day -1	08/11/2010	Negative
S299	310	JAIVAC-1	Dosage C	2	Benzodiazepines	Day 28	10/12/2010	Negative
S299	310	JAIVAC-1	Dosage C	2	Opioids	Day 28	10/12/2010	Negative
S299	310	JAIVAC-1	Dosage C	2	Amphetamine	Day 28	10/12/2010	Negative
S299	310	JAIVAC-1	Dosage C	2	Cocaine	Day 28	10/12/2010	Negative
S299	310	JAIVAC-1	Dosage C	2	THC	Day 28	10/12/2010	Negative
S299	310	JAIVAC-1	Dosage C	3	Benzodiazepines	Day 180	11/05/2011	Negative
S299	310	JAIVAC-1	Dosage C	3	Opioids	Day 180	11/05/2011	Negative
S299	310	JAIVAC-1	Dosage C	3	Amphetamine	Day 180	11/05/2011	Negative

Subject No.	Randomisation Number	Study Group	Dosage	Dose	Urine Parameters	Day	Date of sample Collection	Results
S299	310	JAIVAC-1	Dosage C	3	Cocaine	Day 180	11/05/2011	Negative
S299	310	JAIVAC-1	Dosage C	3	THC	Day 180	11/05/2011	Negative
S299	310	JAIVAC-1	Dosage C	3	Benzodiazepines	Day 365	10/11/2011	Negative
S299	310	JAIVAC-1	Dosage C	3	Opioids	Day 365	10/11/2011	Negative
S299	310	JAIVAC-1	Dosage C	3	Amphetamine	Day 365	10/11/2011	Negative
S299	310	JAIVAC-1	Dosage C	3	Cocaine	Day 365	10/11/2011	Negative
S299	310	JAIVAC-1	Dosage C	3	THC	Day 365	10/11/2011	Negative

16.2.8.5: Listing of Direct Coomb's Test

Subject No.	Randomisation Number	Study Group	Dosage	Dose	Day	Not Done	Specify	Result
S108	115	Hepatitis B	Hepatitis B		Day -14 to Day -1	.		Negative
S108	115	Hepatitis B	Hepatitis B	2	Day 28	.		
S108	115	Hepatitis B	Hepatitis B	2	Day 56	.		
S108	115	Hepatitis B	Hepatitis B	3	Day 180	.		Negative
S108	115	Hepatitis B	Hepatitis B	3	Day 208	.		Negative
S108	115	Hepatitis B	Hepatitis B	3	Day 365	.		
S108	115	Hepatitis B	Hepatitis B		Unscheduled Visit	Yes	As Per PI Discretion	
S108	115	Hepatitis B	Hepatitis B		Unscheduled Visit	Yes	As Per PI Discretion	
S108	115	Hepatitis B	Hepatitis B		Unscheduled Visit	.		
S118	114	JAIVAC-1	Dosage A		Day -14 to Day -1	.		Negative
S118	114	JAIVAC-1	Dosage A	2	Day 28	.		
S118	114	JAIVAC-1	Dosage A	2	Day 56	.		
S118	114	JAIVAC-1	Dosage A	3	Day 180	.		
S118	114	JAIVAC-1	Dosage A	3	Day 208	.		
S118	114	JAIVAC-1	Dosage A	3	Day 365	.		
S119	107	Hepatitis B	Hepatitis B		Day -14 to Day -1	.		Negative
S119	107	Hepatitis B	Hepatitis B	2	Day 28	.		
S119	107	Hepatitis B	Hepatitis B	2	Day 56	.		
S119	107	Hepatitis B	Hepatitis B	3	Day 180	.		
S119	107	Hepatitis B	Hepatitis B	3	Day 208	.		
S119	107	Hepatitis B	Hepatitis B	3	Day 365	.		
S120	104	JAIVAC-1	Dosage A		Day -14 to Day -1	.		Negative
S120	104	JAIVAC-1	Dosage A	2	Day 28	.		
S120	104	JAIVAC-1	Dosage A	2	Day 56	.		
S120	104	JAIVAC-1	Dosage A	3	Day 180	.		
S120	104	JAIVAC-1	Dosage A	3	Day 208	.		
S120	104	JAIVAC-1	Dosage A	3	Day 365	.		
S120	104	JAIVAC-1	Dosage A		Unscheduled Visit	.		

Subject No.	Randomisation Number	Study Group	Dosage	Dose	Day	Not Done	Specify	Result
S124	106	Hepatitis B	Hepatitis B		Day -14 to Day -1	.		Negative
S124	106	Hepatitis B	Hepatitis B	2	Day 28	.		
S124	106	Hepatitis B	Hepatitis B	2	Day 56	.		
S124	106	Hepatitis B	Hepatitis B	3	Day 180	.		
S124	106	Hepatitis B	Hepatitis B	3	Day 208	.		
S124	106	Hepatitis B	Hepatitis B	3	Day 365	.		
S124	106	Hepatitis B	Hepatitis B		Unscheduled Visit	.		
S124	106	Hepatitis B	Hepatitis B		Unscheduled Visit	.		Negative
S124	106	Hepatitis B	Hepatitis B		Unscheduled Visit	.		
S125	102	JAIVAC-1	Dosage A		Day -14 to Day -1	.		Negative
S125	102	JAIVAC-1	Dosage A	2	Day 28	.		
S125	102	JAIVAC-1	Dosage A	2	Day 56	.		
S125	102	JAIVAC-1	Dosage A	3	Day 180	.		
S125	102	JAIVAC-1	Dosage A	3	Day 208	.		
S125	102	JAIVAC-1	Dosage A	3	Day 365	.		
S125	102	JAIVAC-1	Dosage A		Unscheduled Visit	.		
S125	102	JAIVAC-1	Dosage A		Unscheduled Visit	.		
S130	105	JAIVAC-1	Dosage A		Day -14 to Day -1	.		Negative
S130	105	JAIVAC-1	Dosage A	2	Day 28	.		
S130	105	JAIVAC-1	Dosage A	2	Day 56	.		
S130	105	JAIVAC-1	Dosage A	3	Day 180	.		
S130	105	JAIVAC-1	Dosage A	3	Day 208	.		
S130	105	JAIVAC-1	Dosage A	3	Day 365	.		
S130	105	JAIVAC-1	Dosage A		Unscheduled Visit	.		
S132	108	Hepatitis B	Hepatitis B		Day -14 to Day -1	.		Negative
S132	108	Hepatitis B	Hepatitis B	2	Day 28	.		
S132	108	Hepatitis B	Hepatitis B	2	Day 56	.		
S132	108	Hepatitis B	Hepatitis B	3	Day 180	.		
S132	108	Hepatitis B	Hepatitis B	3	Day 208	.		
S132	108	Hepatitis B	Hepatitis B	3	Day 365	.		

Subject No.	Randomisation Number	Study Group	Dosage	Dose	Day	Not Done	Specify	Result
S136	109	JAIVAC-1	Dosage A		Day -14 to Day -1	.		Negative
S136	109	JAIVAC-1	Dosage A	2	Day 28	.		
S136	109	JAIVAC-1	Dosage A	2	Day 56	.		
S136	109	JAIVAC-1	Dosage A	3	Day 180	.		
S136	109	JAIVAC-1	Dosage A	3	Day 208	.		
S136	109	JAIVAC-1	Dosage A	3	Day 365	.		
S143	101	JAIVAC-1	Dosage A		Day -14 to Day -1	.		Negative
S143	101	JAIVAC-1	Dosage A	2	Day 28	.		
S143	101	JAIVAC-1	Dosage A	2	Day 56	.		
S143	101	JAIVAC-1	Dosage A	3	Day 180	.		
S143	101	JAIVAC-1	Dosage A	3	Day 208	.		
S143	101	JAIVAC-1	Dosage A	3	Day 365	.		
S144	103	Hepatitis B	Hepatitis B		Day -14 to Day -1	.		Negative
S144	103	Hepatitis B	Hepatitis B	2	Day 28	.		
S144	103	Hepatitis B	Hepatitis B	2	Day 56	.		
S144	103	Hepatitis B	Hepatitis B	3	Day 180	.		
S144	103	Hepatitis B	Hepatitis B	3	Day 208	.		
S144	103	Hepatitis B	Hepatitis B	3	Day 365	.		
S145	112	JAIVAC-1	Dosage A		Day -14 to Day -1	.		Negative
S145	112	JAIVAC-1	Dosage A	2	Day 28	.		
S145	112	JAIVAC-1	Dosage A	2	Day 56	.		
S145	112	JAIVAC-1	Dosage A	3	Day 180	.		
S145	112	JAIVAC-1	Dosage A	3	Day 208	.		
S145	112	JAIVAC-1	Dosage A	3	Day 365	.		
S148	111	JAIVAC-1	Dosage A		Day -14 to Day -1	.		Negative
S148	111	JAIVAC-1	Dosage A	2	Day 28	.		
S148	111	JAIVAC-1	Dosage A	2	Day 56	.		
S148	111	JAIVAC-1	Dosage A	3	Day 180	.		
S148	111	JAIVAC-1	Dosage A	3	Day 208	.		
S148	111	JAIVAC-1	Dosage A	3	Day 365	.		

Subject No.	Randomisation Number	Study Group	Dosage	Dose	Day	Not Done	Specify	Result
S154	113	JAIVAC-1	Dosage A		Day -14 to Day -1	.		Negative
S154	113	JAIVAC-1	Dosage A	2	Day 28	.		
S154	113	JAIVAC-1	Dosage A	2	Day 56	.		
S154	113	JAIVAC-1	Dosage A	3	Day 180	.		
S154	113	JAIVAC-1	Dosage A	3	Day 208	.		
S154	113	JAIVAC-1	Dosage A	3	Day 365	.		
S156	110	JAIVAC-1	Dosage A		Day -14 to Day -1	.		Negative
S156	110	JAIVAC-1	Dosage A	2	Day 28	.		
S156	110	JAIVAC-1	Dosage A	2	Day 56	.		
S156	110	JAIVAC-1	Dosage A	3	Day 180	.		
S156	110	JAIVAC-1	Dosage A	3	Day 208	.		
S156	110	JAIVAC-1	Dosage A	3	Day 365	.		
S170	204	Hepatitis B	Hepatitis B		Day -14 to Day -1	.		Negative
S170	204	Hepatitis B	Hepatitis B	2	Day 28	.		
S170	204	Hepatitis B	Hepatitis B	2	Day 56	.		
S170	204	Hepatitis B	Hepatitis B	3	Day 180	.		
S170	204	Hepatitis B	Hepatitis B	3	Day 208	.		
S170	204	Hepatitis B	Hepatitis B	3	Day 365	.		
S175	209	JAIVAC-1	Dosage B		Day -14 to Day -1	.		Negative
S175	209	JAIVAC-1	Dosage B	2	Day 28	.		
S175	209	JAIVAC-1	Dosage B	2	Day 56	.		
S175	209	JAIVAC-1	Dosage B	3	Day 180	.		
S175	209	JAIVAC-1	Dosage B	3	Day 208	.		
S175	209	JAIVAC-1	Dosage B	3	Day 365	.		
S176	205	Hepatitis B	Hepatitis B		Day -14 to Day -1	.		Negative
S176	205	Hepatitis B	Hepatitis B	2	Day 28	.		
S176	205	Hepatitis B	Hepatitis B	2	Day 56	.		
S176	205	Hepatitis B	Hepatitis B	3	Day 180	.		
S176	205	Hepatitis B	Hepatitis B	3	Day 208	.		
S176	205	Hepatitis B	Hepatitis B	3	Day 365	.		

Subject No.	Randomisation Number	Study Group	Dosage	Dose	Day	Not Done	Specify	Result
S176	205	Hepatitis B	Hepatitis B		Unscheduled Visit	.		
S179	207	Hepatitis B	Hepatitis B		Day -14 to Day -1	.		Negative
S179	207	Hepatitis B	Hepatitis B	2	Day 28	.		
S179	207	Hepatitis B	Hepatitis B	2	Day 56	.		
S179	207	Hepatitis B	Hepatitis B	3	Day 180	.		
S179	207	Hepatitis B	Hepatitis B	3	Day 208	.		
S179	207	Hepatitis B	Hepatitis B	3	Day 365	.		
S182	208	JAIVAC-1	Dosage B		Day -14 to Day -1	.		Negative
S182	208	JAIVAC-1	Dosage B	2	Day 28	.		
S182	208	JAIVAC-1	Dosage B	2	Day 56	.		
S182	208	JAIVAC-1	Dosage B	3	Day 180	.		
S182	208	JAIVAC-1	Dosage B	3	Day 208	.		
S182	208	JAIVAC-1	Dosage B	3	Day 365	.		
S182	208	JAIVAC-1	Dosage B		Unscheduled Visit	.		
S185	202	JAIVAC-1	Dosage B		Day -14 to Day -1	.		Negative
S185	202	JAIVAC-1	Dosage B	2	Day 28	.		
S185	202	JAIVAC-1	Dosage B	2	Day 56	.		
S185	202	JAIVAC-1	Dosage B	3	Day 180	.		
S185	202	JAIVAC-1	Dosage B	3	Day 208	.		
S185	202	JAIVAC-1	Dosage B	3	Day 365	.		
S186	201	JAIVAC-1	Dosage B		Day -14 to Day -1	.		Negative
S186	201	JAIVAC-1	Dosage B	2	Day 28	.		
S186	201	JAIVAC-1	Dosage B	2	Day 56	.		
S186	201	JAIVAC-1	Dosage B	3	Day 180	.		
S186	201	JAIVAC-1	Dosage B	3	Day 208	.		
S186	201	JAIVAC-1	Dosage B	3	Day 365	.		
S192	203	JAIVAC-1	Dosage B		Day -14 to Day -1	.		Negative
S192	203	JAIVAC-1	Dosage B	2	Day 28	.		
S192	203	JAIVAC-1	Dosage B	2	Day 56	.		
S192	203	JAIVAC-1	Dosage B	3	Day 180	.		

Subject No.	Randomisation Number	Study Group	Dosage	Dose	Day	Not Done	Specify	Result
S192	203	JAIVAC-1	Dosage B	3	Day 208	.		
S192	203	JAIVAC-1	Dosage B	3	Day 365	.		
S193	215	JAIVAC-1	Dosage B		Day -14 to Day -1	.		Negative
S193	215	JAIVAC-1	Dosage B	2	Day 28	.		
S193	215	JAIVAC-1	Dosage B	2	Day 56	.		
S193	215	JAIVAC-1	Dosage B	3	Day 180	.		
S193	215	JAIVAC-1	Dosage B	3	Day 208	.		
S193	215	JAIVAC-1	Dosage B	3	Day 365	.		
S195	214	JAIVAC-1	Dosage B		Day -14 to Day -1	.		Negative
S195	214	JAIVAC-1	Dosage B	2	Day 28	.		
S195	214	JAIVAC-1	Dosage B	2	Day 56	.		
S195	214	JAIVAC-1	Dosage B	3	Day 180	.		
S195	214	JAIVAC-1	Dosage B	3	Day 208	.		
S195	214	JAIVAC-1	Dosage B	3	Day 365	.		Negative
S202	206	JAIVAC-1	Dosage B		Day -14 to Day -1	.		Negative
S202	206	JAIVAC-1	Dosage B	2	Day 28	.		
S202	206	JAIVAC-1	Dosage B	2	Day 56	.		
S202	206	JAIVAC-1	Dosage B	3	Day 180	.		
S202	206	JAIVAC-1	Dosage B	3	Day 208	.		
S202	206	JAIVAC-1	Dosage B	3	Day 365	.		
S203	211	Hepatitis B	Hepatitis B		Day -14 to Day -1	.		Negative
S203	211	Hepatitis B	Hepatitis B	2	Day 28	.		
S203	211	Hepatitis B	Hepatitis B	2	Day 56	.		Negative
S203	211	Hepatitis B	Hepatitis B	3	Day 180	.		
S203	211	Hepatitis B	Hepatitis B	3	Day 208	.		
S203	211	Hepatitis B	Hepatitis B	3	Day 365	.		
S203	211	Hepatitis B	Hepatitis B		Unscheduled Visit	Yes	As Per Investigator Discretion	
S203	211	Hepatitis B	Hepatitis B		Unscheduled Visit	.		
S203	211	Hepatitis B	Hepatitis B		Unscheduled Visit	.		
S212	212	JAIVAC-1	Dosage B		Day -14 to Day -1	.		Negative

Subject No.	Randomisation Number	Study Group	Dosage	Dose	Day	Not Done	Specify	Result
S212	212	JAIVAC-1	Dosage B	2	Day 28	.		
S212	212	JAIVAC-1	Dosage B	2	Day 56	.		
S212	212	JAIVAC-1	Dosage B	3	Day 180	.		
S212	212	JAIVAC-1	Dosage B	3	Day 208	.		
S212	212	JAIVAC-1	Dosage B	3	Day 365	.		
S215	213	Hepatitis B	Hepatitis B		Day -14 to Day -1	.		Negative
S215	213	Hepatitis B	Hepatitis B	2	Day 28	.		
S215	213	Hepatitis B	Hepatitis B	2	Day 56	.		
S215	213	Hepatitis B	Hepatitis B	3	Day 180	.		
S215	213	Hepatitis B	Hepatitis B	3	Day 208	.		
S215	213	Hepatitis B	Hepatitis B	3	Day 365	.		
S233	210	JAIVAC-1	Dosage B		Day -14 to Day -1	.		Negative
S233	210	JAIVAC-1	Dosage B	2	Day 28	.		
S233	210	JAIVAC-1	Dosage B	2	Day 56	.		
S233	210	JAIVAC-1	Dosage B	3	Day 180	.		Negative
S233	210	JAIVAC-1	Dosage B	3	Day 208	.		
S233	210	JAIVAC-1	Dosage B	3	Day 365	.		
S241	303	Hepatitis B	Hepatitis B		Day -14 to Day -1	.		Negative
S241	303	Hepatitis B	Hepatitis B	2	Day 28	.		
S241	303	Hepatitis B	Hepatitis B	2	Day 56	.		
S241	303	Hepatitis B	Hepatitis B	3	Day 180	.		
S241	303	Hepatitis B	Hepatitis B	3	Day 208	.		
S241	303	Hepatitis B	Hepatitis B	3	Day 365	.		
S250	311	JAIVAC-1	Dosage C		Day -14 to Day -1	.		Negative
S250	311	JAIVAC-1	Dosage C	2	Day 28	.		
S250	311	JAIVAC-1	Dosage C	2	Day 56	.		
S250	311	JAIVAC-1	Dosage C	3	Day 180	.		
S250	311	JAIVAC-1	Dosage C	3	Day 208	.		
S250	311	JAIVAC-1	Dosage C	3	Day 365	.		
S263	313	Hepatitis B	Hepatitis B		Day -14 to Day -1	.		Negative

Subject No.	Randomisation Number	Study Group	Dosage	Dose	Day	Not Done	Specify	Result
S263	313	Hepatitis B	Hepatitis B	2	Day 28	.		
S263	313	Hepatitis B	Hepatitis B	2	Day 56	.		
S263	313	Hepatitis B	Hepatitis B	3	Day 180	.		
S263	313	Hepatitis B	Hepatitis B	3	Day 208	.		
S263	313	Hepatitis B	Hepatitis B	3	Day 365	.		
S263	313	Hepatitis B	Hepatitis B		Unscheduled Visit	.		
S264	306	JAIVAC-1	Dosage C		Day -14 to Day -1	.		Negative
S264	306	JAIVAC-1	Dosage C	2	Day 28	.		
S264	306	JAIVAC-1	Dosage C	2	Day 56	.		
S264	306	JAIVAC-1	Dosage C	3	Day 180	.		
S264	306	JAIVAC-1	Dosage C	3	Day 208	.		
S264	306	JAIVAC-1	Dosage C	3	Day 365	.		
S266	308	JAIVAC-1	Dosage C		Day -14 to Day -1	.		Negative
S266	308	JAIVAC-1	Dosage C	2	Day 28	.		
S266	308	JAIVAC-1	Dosage C	2	Day 56	.		
S266	308	JAIVAC-1	Dosage C	3	Day 180	.		
S266	308	JAIVAC-1	Dosage C	3	Day 208	.		
S266	308	JAIVAC-1	Dosage C	3	Day 365	.		
S266	308	JAIVAC-1	Dosage C		Unscheduled Visit	.		
S266	308	JAIVAC-1	Dosage C		Unscheduled Visit	.		
S266	308	JAIVAC-1	Dosage C		Unscheduled Visit	.		
S267	309	Hepatitis B	Hepatitis B		Day -14 to Day -1	.		Negative
S267	309	Hepatitis B	Hepatitis B	2	Day 28	.		
S267	309	Hepatitis B	Hepatitis B	2	Day 56	.		
S267	309	Hepatitis B	Hepatitis B	3	Day 180	.		
S267	309	Hepatitis B	Hepatitis B	3	Day 208	.		
S267	309	Hepatitis B	Hepatitis B	3	Day 365	.		
S267	309	Hepatitis B	Hepatitis B		Unscheduled Visit	.		
S275	302	Hepatitis B	Hepatitis B		Day -14 to Day -1	.		Negative
S275	302	Hepatitis B	Hepatitis B	2	Day 28	.		

Subject No.	Randomisation Number	Study Group	Dosage	Dose	Day	Not Done	Specify	Result
S275	302	Hepatitis B	Hepatitis B	2	Day 56	.		
S275	302	Hepatitis B	Hepatitis B	3	Day 180	.		
S275	302	Hepatitis B	Hepatitis B	3	Day 208	.		
S275	302	Hepatitis B	Hepatitis B	3	Day 365	.		
S278	315	JAIVAC-1	Dosage C		Day -14 to Day -1	.		Negative
S278	315	JAIVAC-1	Dosage C	2	Day 28	.		
S278	315	JAIVAC-1	Dosage C	2	Day 56	.		
S278	315	JAIVAC-1	Dosage C	3	Day 180	.		
S278	315	JAIVAC-1	Dosage C	3	Day 208	.		
S278	315	JAIVAC-1	Dosage C	3	Day 365	.		
S279	301	JAIVAC-1	Dosage C		Day -14 to Day -1	.		Negative
S279	301	JAIVAC-1	Dosage C	2	Day 28	.		
S279	301	JAIVAC-1	Dosage C	2	Day 56	.		
S279	301	JAIVAC-1	Dosage C	3	Day 180	.		
S279	301	JAIVAC-1	Dosage C	3	Day 208	.		
S279	301	JAIVAC-1	Dosage C	3	Day 365	.		
S281	304	JAIVAC-1	Dosage C		Day -14 to Day -1	.		Negative
S281	304	JAIVAC-1	Dosage C	2	Day 28	.		
S281	304	JAIVAC-1	Dosage C	2	Day 56	.		Negative
S281	304	JAIVAC-1	Dosage C		Early Termination	.		Negative
S284	305	JAIVAC-1	Dosage C		Day -14 to Day -1	.		Negative
S284	305	JAIVAC-1	Dosage C	2	Day 28	.		
S284	305	JAIVAC-1	Dosage C	2	Day 56	.		
S284	305	JAIVAC-1	Dosage C	3	Day 180	.		
S284	305	JAIVAC-1	Dosage C	3	Day 208	.		
S284	305	JAIVAC-1	Dosage C	3	Day 365	.		
S285	307	Hepatitis B	Hepatitis B		Day -14 to Day -1	.		Negative
S285	307	Hepatitis B	Hepatitis B	2	Day 28	.		
S285	307	Hepatitis B	Hepatitis B	2	Day 56	.		

Subject No.	Randomisation Number	Study Group	Dosage	Dose	Day	Not Done	Specify	Result
S285	307	Hepatitis B	Hepatitis B	3	Day 180	.		
S285	307	Hepatitis B	Hepatitis B	3	Day 208	.		
S285	307	Hepatitis B	Hepatitis B	3	Day 365	.		
S291	312	JAIVAC-1	Dosage C		Day -14 to Day -1	.		Negative
S291	312	JAIVAC-1	Dosage C	2	Day 28	.		
S291	312	JAIVAC-1	Dosage C	2	Day 56	.		
S291	312	JAIVAC-1	Dosage C	3	Day 180	.		
S291	312	JAIVAC-1	Dosage C	3	Day 208	.		
S291	312	JAIVAC-1	Dosage C	3	Day 365	.		
S298	314	JAIVAC-1	Dosage C		Day -14 to Day -1	.		Negative
S298	314	JAIVAC-1	Dosage C	2	Day 28	.		
S298	314	JAIVAC-1	Dosage C	2	Day 56	.		
S298	314	JAIVAC-1	Dosage C	3	Day 180	.		
S298	314	JAIVAC-1	Dosage C	3	Day 208	.		
S298	314	JAIVAC-1	Dosage C	3	Day 365	.		
S299	310	JAIVAC-1	Dosage C		Day -14 to Day -1	.		Negative
S299	310	JAIVAC-1	Dosage C	2	Day 28	.		
S299	310	JAIVAC-1	Dosage C	2	Day 56	.		
S299	310	JAIVAC-1	Dosage C	3	Day 180	.		
S299	310	JAIVAC-1	Dosage C	3	Day 208	.		
S299	310	JAIVAC-1	Dosage C	3	Day 365	.		

16.2.8.6: Listing of Peripheral Blood Smear (PBS)

Subject No.	Randomisation Number	Study Group	Dosage	Dose	Day	Not Done	Specify	Result / Comment (CRF data-As per Investigator's discretion)	Result / Comment (As per Central laboratory)
S108	115	Hepatitis B	Hepatitis B		Day -14 to Day -1	.		Negative	Normocytic, Normochromic RBCs seen on peripheral blood smear, not suggestive of hemolysis.
S108	115	Hepatitis B	Hepatitis B	2	Day 28	.			
S108	115	Hepatitis B	Hepatitis B	2	Day 56	.			
S108	115	Hepatitis B	Hepatitis B	3	Day 180	.		Negative	Normocytic, Normochromic RBCs seen on peripheral blood smear, not suggestive of hemolysis.
S108	115	Hepatitis B	Hepatitis B	3	Day 208	.		Negative	Normocytic, Normochromic RBCs seen on peripheral blood smear, not suggestive of hemolysis.
S108	115	Hepatitis B	Hepatitis B	3	Day 365	.			
S108	115	Hepatitis B	Hepatitis B		Unscheduled Visit	Yes	AS PER PI DISCRETION		
S108	115	Hepatitis B	Hepatitis B		Unscheduled Visit	.			
S108	115	Hepatitis B	Hepatitis B		Unscheduled Visit	.			
S118	114	JAIVAC-1	Dosage A		Day -14 to Day -1	.		Negative	Normocytic, Normochromic RBCs seen on peripheral blood smear, not suggestive of hemolysis.
S118	114	JAIVAC-1	Dosage A	2	Day 28	.			
S118	114	JAIVAC-1	Dosage A	2	Day 56	.			
S118	114	JAIVAC-1	Dosage A	3	Day 180	.			
S118	114	JAIVAC-1	Dosage A	3	Day 208	.			
S118	114	JAIVAC-1	Dosage A	3	Day 365	.			
S119	107	Hepatitis B	Hepatitis B		Day -14 to Day -1	.		Negative	Normocytic, Normochromic RBCs seen on peripheral blood smear, not suggestive of hemolysis.
S119	107	Hepatitis B	Hepatitis B	2	Day 28	.			
S119	107	Hepatitis B	Hepatitis B	2	Day 56	.			
S119	107	Hepatitis B	Hepatitis B	3	Day 180	.			
S119	107	Hepatitis B	Hepatitis B	3	Day 208	.			
S119	107	Hepatitis B	Hepatitis B	3	Day 365	.			

Subject No.	Randomisation Number	Study Group	Dosage	Dose	Day	Not Done	Specify	Result / Comment (CRF data-As per Investigator's discretion)	Result / Comment (As per Central laboratory)
S120	104	JAIVAC-1	Dosage A		Day -14 to Day -1	.		Negative	Normocytic, Normochromic RBCs seen on peripheral blood smear, not suggestive of hemolysis.
S120	104	JAIVAC-1	Dosage A	2	Day 28	.			
S120	104	JAIVAC-1	Dosage A	2	Day 56	.			
S120	104	JAIVAC-1	Dosage A	3	Day 180	.			
S120	104	JAIVAC-1	Dosage A	3	Day 208	.			
S120	104	JAIVAC-1	Dosage A	3	Day 365	.			
S120	104	JAIVAC-1	Dosage A		Unscheduled Visit	.			
S124	106	Hepatitis B	Hepatitis B		Day -14 to Day -1	.		Negative	Normocytic, Normochromic RBCs seen on peripheral blood smear, not suggestive of hemolysis.
S124	106	Hepatitis B	Hepatitis B	2	Day 28	.			
S124	106	Hepatitis B	Hepatitis B	2	Day 56	.			
S124	106	Hepatitis B	Hepatitis B	3	Day 180	.			
S124	106	Hepatitis B	Hepatitis B	3	Day 208	.			
S124	106	Hepatitis B	Hepatitis B	3	Day 365	.			
S124	106	Hepatitis B	Hepatitis B		Unscheduled Visit	.		Negative	
S124	106	Hepatitis B	Hepatitis B		Unscheduled Visit	.		Negative	Hypochromic (++), Microcytic (+) RBCs seen on peripheral blood smear, not suggestive of hemolysis
S124	106	Hepatitis B	Hepatitis B		Unscheduled Visit	.			
S125	102	JAIVAC-1	Dosage A		Day -14 to Day -1	.		Negative	Normocytic, Normochromic RBCs seen on peripheral blood smear, not suggestive of hemolysis.
S125	102	JAIVAC-1	Dosage A	2	Day 28	.			
S125	102	JAIVAC-1	Dosage A	2	Day 56	.			
S125	102	JAIVAC-1	Dosage A	3	Day 180	.			
S125	102	JAIVAC-1	Dosage A	3	Day 208	.			
S125	102	JAIVAC-1	Dosage A	3	Day 365	.			
S125	102	JAIVAC-1	Dosage A		Unscheduled Visit	.			

Subject No.	Randomisation Number	Study Group	Dosage	Dose	Day	Not Done	Specify	Result / Comment (CRF data-As per Investigator's discretion)	Result / Comment (As per Central laboratory)
S125	102	JAIVAC-1	Dosage A		Unscheduled Visit	.			
S130	105	JAIVAC-1	Dosage A		Day -14 to Day -1	.		Negative	Normocytic, Normochromic RBCs seen on peripheral blood smear, not suggestive of hemolysis.
S130	105	JAIVAC-1	Dosage A	2	Day 28	.			
S130	105	JAIVAC-1	Dosage A	2	Day 56	.			
S130	105	JAIVAC-1	Dosage A	3	Day 180	.			
S130	105	JAIVAC-1	Dosage A	3	Day 208	.			
S130	105	JAIVAC-1	Dosage A	3	Day 365	.			
S130	105	JAIVAC-1	Dosage A		Unscheduled Visit	.			
S132	108	Hepatitis B	Hepatitis B		Day -14 to Day -1	.		Negative	Normocytic, Normochromic RBCs seen on peripheral blood smear, not suggestive of hemolysis.
S132	108	Hepatitis B	Hepatitis B	2	Day 28	.			
S132	108	Hepatitis B	Hepatitis B	2	Day 56	.			
S132	108	Hepatitis B	Hepatitis B	3	Day 180	.			
S132	108	Hepatitis B	Hepatitis B	3	Day 208	.			
S132	108	Hepatitis B	Hepatitis B	3	Day 365	.			
S136	109	JAIVAC-1	Dosage A		Day -14 to Day -1	.		Negative	Hypochromic (+) RBCs seen on peripheral blood smear, not suggestive of hemolysis.
S136	109	JAIVAC-1	Dosage A	2	Day 28	.			
S136	109	JAIVAC-1	Dosage A	2	Day 56	.			
S136	109	JAIVAC-1	Dosage A	3	Day 180	.			
S136	109	JAIVAC-1	Dosage A	3	Day 208	.			
S136	109	JAIVAC-1	Dosage A	3	Day 365	.			
S143	101	JAIVAC-1	Dosage A		Day -14 to Day -1	.		Negative	Normocytic, Normochromic RBCs seen on peripheral blood smear, not suggestive of hemolysis.
S143	101	JAIVAC-1	Dosage A	2	Day 28	.			
S143	101	JAIVAC-1	Dosage A	2	Day 56	.			
S143	101	JAIVAC-1	Dosage A	3	Day 180	.			
S143	101	JAIVAC-1	Dosage A	3	Day 208	.			

Subject No.	Randomisation Number	Study Group	Dosage	Dose	Day	Not Done	Specify	Result / Comment (CRF data-As per Investigator's discretion)	Result / Comment (As per Central laboratory)
S143	101	JAIVAC-1	Dosage A	3	Day 365	.			
S144	103	Hepatitis B	Hepatitis B		Day -14 to Day -1	.		Negative	Normocytic, Normochromic RBCs seen on peripheral blood smear, not suggestive of hemolysis.
S144	103	Hepatitis B	Hepatitis B	2	Day 28	.			
S144	103	Hepatitis B	Hepatitis B	2	Day 56	.			
S144	103	Hepatitis B	Hepatitis B	3	Day 180	.			
S144	103	Hepatitis B	Hepatitis B	3	Day 208	.			
S144	103	Hepatitis B	Hepatitis B	3	Day 365	.			
S145	112	JAIVAC-1	Dosage A		Day -14 to Day -1	.		Negative	Normocytic, Normochromic RBCs seen on peripheral blood smear, not suggestive of hemolysis.
S145	112	JAIVAC-1	Dosage A	2	Day 28	.			
S145	112	JAIVAC-1	Dosage A	2	Day 56	.			
S145	112	JAIVAC-1	Dosage A	3	Day 180	.			
S145	112	JAIVAC-1	Dosage A	3	Day 208	.			
S145	112	JAIVAC-1	Dosage A	3	Day 365	.			
S148	111	JAIVAC-1	Dosage A		Day -14 to Day -1	.		Negative	Normocytic, Normochromic RBCs seen on peripheral blood smear, not suggestive of hemolysis.
S148	111	JAIVAC-1	Dosage A	2	Day 28	.			
S148	111	JAIVAC-1	Dosage A	2	Day 56	.			
S148	111	JAIVAC-1	Dosage A	3	Day 180	.			
S148	111	JAIVAC-1	Dosage A	3	Day 208	.			
S148	111	JAIVAC-1	Dosage A	3	Day 365	.			
S154	113	JAIVAC-1	Dosage A		Day -14 to Day -1	.		Negative	Normocytic, Normochromic RBCs seen on peripheral blood smear, not suggestive of hemolysis.
S154	113	JAIVAC-1	Dosage A	2	Day 28	.			
S154	113	JAIVAC-1	Dosage A	2	Day 56	.			
S154	113	JAIVAC-1	Dosage A	3	Day 180	.			
S154	113	JAIVAC-1	Dosage A	3	Day 208	.			
S154	113	JAIVAC-1	Dosage A	3	Day 365	.			

Subject No.	Randomisation Number	Study Group	Dosage	Dose	Day	Not Done	Specify	Result / Comment (CRF data-As per Investigator's discretion)	Result / Comment (As per Central laboratory)
S156	110	JAIVAC-1	Dosage A		Day -14 to Day -1	.		Negative	Normocytic, Normochromic RBCs seen on peripheral blood smear, not suggestive of hemolysis.
S156	110	JAIVAC-1	Dosage A	2	Day 28	.			
S156	110	JAIVAC-1	Dosage A	2	Day 56	.			
S156	110	JAIVAC-1	Dosage A	3	Day 180	.			
S156	110	JAIVAC-1	Dosage A	3	Day 208	.			
S156	110	JAIVAC-1	Dosage A	3	Day 365	.			
S170	204	Hepatitis B	Hepatitis B		Day -14 to Day -1	.		Negative	Normocytic, Normochromic RBCs seen on peripheral blood smear, not suggestive of hemolysis.
S170	204	Hepatitis B	Hepatitis B	2	Day 28	.			
S170	204	Hepatitis B	Hepatitis B	2	Day 56	.			
S170	204	Hepatitis B	Hepatitis B	3	Day 180	.			
S170	204	Hepatitis B	Hepatitis B	3	Day 208	.			
S170	204	Hepatitis B	Hepatitis B	3	Day 365	.			
S175	209	JAIVAC-1	Dosage B		Day -14 to Day -1	.		Negative	Normocytic, Normochromic RBCs seen on peripheral blood smear, not suggestive of hemolysis.
S175	209	JAIVAC-1	Dosage B	2	Day 28	.			
S175	209	JAIVAC-1	Dosage B	2	Day 56	.			
S175	209	JAIVAC-1	Dosage B	3	Day 180	.			
S175	209	JAIVAC-1	Dosage B	3	Day 208	.			
S175	209	JAIVAC-1	Dosage B	3	Day 365	.			
S176	205	Hepatitis B	Hepatitis B		Day -14 to Day -1	.		Negative	Normocytic, Normochromic RBCs seen on peripheral blood smear, not suggestive of hemolysis.
S176	205	Hepatitis B	Hepatitis B	2	Day 28	.			
S176	205	Hepatitis B	Hepatitis B	2	Day 56	.			
S176	205	Hepatitis B	Hepatitis B	3	Day 180	.			
S176	205	Hepatitis B	Hepatitis B	3	Day 208	.			
S176	205	Hepatitis B	Hepatitis B	3	Day 365	.			
S176	205	Hepatitis B	Hepatitis B		Unscheduled Visit	.			

Subject No.	Randomisation Number	Study Group	Dosage	Dose	Day	Not Done	Specify	Result / Comment (CRF data-As per Investigator's discretion)	Result / Comment (As per Central laboratory)
S179	207	Hepatitis B	Hepatitis B		Day -14 to Day -1	.		Negative	Normocytic, Normochromic RBCs seen on peripheral blood smear, not suggestive of hemolysis.
S179	207	Hepatitis B	Hepatitis B	2	Day 28	.			
S179	207	Hepatitis B	Hepatitis B	2	Day 56	.			
S179	207	Hepatitis B	Hepatitis B	3	Day 180	.			
S179	207	Hepatitis B	Hepatitis B	3	Day 208	.			
S179	207	Hepatitis B	Hepatitis B	3	Day 365	.			
S182	208	JAIVAC-1	Dosage B		Day -14 to Day -1	.		Negative	Normocytic, Normochromic RBCs seen on peripheral blood smear, not suggestive of hemolysis.
S182	208	JAIVAC-1	Dosage B	2	Day 28	.			
S182	208	JAIVAC-1	Dosage B	2	Day 56	.			
S182	208	JAIVAC-1	Dosage B	3	Day 180	.			
S182	208	JAIVAC-1	Dosage B	3	Day 208	.			
S182	208	JAIVAC-1	Dosage B	3	Day 365	.			
S182	208	JAIVAC-1	Dosage B		Unscheduled Visit	.			
S185	202	JAIVAC-1	Dosage B		Day -14 to Day -1	.		Negative	Normocytic, Normochromic RBCs seen on peripheral blood smear, not suggestive of hemolysis.
S185	202	JAIVAC-1	Dosage B	2	Day 28	.			
S185	202	JAIVAC-1	Dosage B	2	Day 56	.			
S185	202	JAIVAC-1	Dosage B	3	Day 180	.			
S185	202	JAIVAC-1	Dosage B	3	Day 208	.			
S185	202	JAIVAC-1	Dosage B	3	Day 365	.			
S186	201	JAIVAC-1	Dosage B		Day -14 to Day -1	.		Negative	Normocytic, Normochromic RBCs seen on peripheral blood smear, not suggestive of hemolysis.
S186	201	JAIVAC-1	Dosage B	2	Day 28	.			
S186	201	JAIVAC-1	Dosage B	2	Day 56	.			
S186	201	JAIVAC-1	Dosage B	3	Day 180	.			
S186	201	JAIVAC-1	Dosage B	3	Day 208	.			
S186	201	JAIVAC-1	Dosage B	3	Day 365	.			

Subject No.	Randomisation Number	Study Group	Dosage	Dose	Day	Not Done	Specify	Result / Comment (CRF data-As per Investigator's discretion)	Result / Comment (As per Central laboratory)
S192	203	JAIVAC-1	Dosage B		Day -14 to Day -1	.		Negative	Normocytic, Normochromic RBCs seen on peripheral blood smear, not suggestive of hemolysis.
S192	203	JAIVAC-1	Dosage B	2	Day 28	.			
S192	203	JAIVAC-1	Dosage B	2	Day 56	.			
S192	203	JAIVAC-1	Dosage B	3	Day 180	.			
S192	203	JAIVAC-1	Dosage B	3	Day 208	.			
S192	203	JAIVAC-1	Dosage B	3	Day 365	.			
S193	215	JAIVAC-1	Dosage B		Day -14 to Day -1	.		Negative	Normocytic, Normochromic RBCs seen on peripheral blood smear, not suggestive of hemolysis.
S193	215	JAIVAC-1	Dosage B	2	Day 28	.			
S193	215	JAIVAC-1	Dosage B	2	Day 56	.			
S193	215	JAIVAC-1	Dosage B	3	Day 180	.			
S193	215	JAIVAC-1	Dosage B	3	Day 208	.			
S193	215	JAIVAC-1	Dosage B	3	Day 365	.			
S195	214	JAIVAC-1	Dosage B		Day -14 to Day -1	.		Negative	Normocytic, Normochromic RBCs seen on peripheral blood smear, not suggestive of hemolysis.
S195	214	JAIVAC-1	Dosage B	2	Day 28	.			
S195	214	JAIVAC-1	Dosage B	2	Day 56	.			
S195	214	JAIVAC-1	Dosage B	3	Day 180	.			
S195	214	JAIVAC-1	Dosage B	3	Day 208	.			
S195	214	JAIVAC-1	Dosage B	3	Day 365	.		Negative	Normocytic, Normochromic RBCs seen on peripheral blood smear, not suggestive of hemolysis.
S202	206	JAIVAC-1	Dosage B		Day -14 to Day -1	.		Negative	Normocytic, Normochromic RBCs seen on peripheral blood smear, not suggestive of hemolysis.
S202	206	JAIVAC-1	Dosage B	2	Day 28	.			
S202	206	JAIVAC-1	Dosage B	2	Day 56	.			
S202	206	JAIVAC-1	Dosage B	3	Day 180	.			
S202	206	JAIVAC-1	Dosage B	3	Day 208	.			
S202	206	JAIVAC-1	Dosage B	3	Day 365	.			
S203	211	Hepatitis B	Hepatitis B		Day -14 to Day -1	.		Negative	Hypochromic (+) RBCs seen on peripheral blood smear, not suggestive of hemolysis.

Subject No.	Randomisation Number	Study Group	Dosage	Dose	Day	Not Done	Specify	Result / Comment (CRF data-As per Investigator's discretion)	Result / Comment (As per Central laboratory)
S203	211	Hepatitis B	Hepatitis B	2	Day 28	.			
S203	211	Hepatitis B	Hepatitis B	2	Day 56	.		Negative	Hypochromic (++), Microcytic (+) RBCs seen on peripheral blood smear, not suggestive of hemolysis.
S203	211	Hepatitis B	Hepatitis B	3	Day 180	.			
S203	211	Hepatitis B	Hepatitis B	3	Day 208	.			
S203	211	Hepatitis B	Hepatitis B	3	Day 365	.			
S203	211	Hepatitis B	Hepatitis B		Unscheduled Visit	Yes	AS PER INVESTIGATOR DISCRETION		
S203	211	Hepatitis B	Hepatitis B		Unscheduled Visit	.			
S203	211	Hepatitis B	Hepatitis B		Unscheduled Visit	.			
S212	212	JAIVAC-1	Dosage B		Day -14 to Day -1	.		Negative	Microcytic (+) RBCs seen on peripheral blood smear, not suggestive of hemolysis.
S212	212	JAIVAC-1	Dosage B	2	Day 28	.			
S212	212	JAIVAC-1	Dosage B	2	Day 56	.			
S212	212	JAIVAC-1	Dosage B	3	Day 180	.			
S212	212	JAIVAC-1	Dosage B	3	Day 208	.			
S212	212	JAIVAC-1	Dosage B	3	Day 365	.			
S215	213	Hepatitis B	Hepatitis B		Day -14 to Day -1	.		Negative	Normocytic, Normochromic RBCs seen on peripheral blood smear, not suggestive of hemolysis.
S215	213	Hepatitis B	Hepatitis B	2	Day 28	.			
S215	213	Hepatitis B	Hepatitis B	2	Day 56	.			
S215	213	Hepatitis B	Hepatitis B	3	Day 180	.			
S215	213	Hepatitis B	Hepatitis B	3	Day 208	.			
S215	213	Hepatitis B	Hepatitis B	3	Day 365	.			
S233	210	JAIVAC-1	Dosage B		Day -14 to Day -1	.		Negative	Normocytic, Normochromic RBCs seen on peripheral blood smear, not suggestive of hemolysis.
S233	210	JAIVAC-1	Dosage B	2	Day 28	.			
S233	210	JAIVAC-1	Dosage B	2	Day 56	.			
S233	210	JAIVAC-1	Dosage B	3	Day 180	.		Negative	Hypochromic (++), Microcytic (++) RBCs seen on peripheral blood smear, not suggestive of hemolysis.

Subject No.	Randomisation Number	Study Group	Dosage	Dose	Day	Not Done	Specify	Result / Comment (CRF data-As per Investigator's discretion)	Result / Comment (As per Central laboratory)
S233	210	JAIVAC-1	Dosage B	3	Day 208	.			
S233	210	JAIVAC-1	Dosage B	3	Day 365	.			
S241	303	Hepatitis B	Hepatitis B		Day -14 to Day -1	.		Negative	Normocytic, Normochromic RBCs seen on peripheral blood smear, not suggestive of hemolysis.
S241	303	Hepatitis B	Hepatitis B	2	Day 28	.			
S241	303	Hepatitis B	Hepatitis B	2	Day 56	.			
S241	303	Hepatitis B	Hepatitis B	3	Day 180	.			
S241	303	Hepatitis B	Hepatitis B	3	Day 208	.			
S241	303	Hepatitis B	Hepatitis B	3	Day 365	.			
S250	311	JAIVAC-1	Dosage C		Day -14 to Day -1	.		Negative	Normocytic, Normochromic RBCs seen on peripheral blood smear, not suggestive of hemolysis.
S250	311	JAIVAC-1	Dosage C	2	Day 28	.			
S250	311	JAIVAC-1	Dosage C	2	Day 56	.			
S250	311	JAIVAC-1	Dosage C	3	Day 180	.			
S250	311	JAIVAC-1	Dosage C	3	Day 208	.			
S250	311	JAIVAC-1	Dosage C	3	Day 365	.			
S263	313	Hepatitis B	Hepatitis B		Day -14 to Day -1	.		Negative	Normocytic, Normochromic RBCs seen on peripheral blood smear, not suggestive of hemolysis.
S263	313	Hepatitis B	Hepatitis B	2	Day 28	.			
S263	313	Hepatitis B	Hepatitis B	2	Day 56	.			
S263	313	Hepatitis B	Hepatitis B	3	Day 180	.			
S263	313	Hepatitis B	Hepatitis B	3	Day 208	.			
S263	313	Hepatitis B	Hepatitis B	3	Day 365	.			
S263	313	Hepatitis B	Hepatitis B		Unscheduled Visit	.			
S264	306	JAIVAC-1	Dosage C		Day -14 to Day -1	.		Negative	Normocytic, Normochromic RBCs seen on peripheral blood smear, not suggestive of hemolysis.
S264	306	JAIVAC-1	Dosage C	2	Day 28	.			
S264	306	JAIVAC-1	Dosage C	2	Day 56	.			
S264	306	JAIVAC-1	Dosage C	3	Day 180	.			

Subject No.	Randomisation Number	Study Group	Dosage	Dose	Day	Not Done	Specify	Result / Comment (CRF data-As per Investigator's discretion)	Result / Comment (As per Central laboratory)
S264	306	JAIVAC-1	Dosage C	3	Day 208	.			
S264	306	JAIVAC-1	Dosage C	3	Day 365	.			
S266	308	JAIVAC-1	Dosage C		Day -14 to Day -1	.		Negative	Normocytic, Normochromic RBCs seen on peripheral blood smear, not suggestive of hemolysis.
S266	308	JAIVAC-1	Dosage C	2	Day 28	.			
S266	308	JAIVAC-1	Dosage C	2	Day 56	.			
S266	308	JAIVAC-1	Dosage C	3	Day 180	.			
S266	308	JAIVAC-1	Dosage C	3	Day 208	.			
S266	308	JAIVAC-1	Dosage C	3	Day 365	.			
S266	308	JAIVAC-1	Dosage C		Unscheduled Visit	.			
S266	308	JAIVAC-1	Dosage C		Unscheduled Visit	.			
S266	308	JAIVAC-1	Dosage C		Unscheduled Visit	.			
S267	309	Hepatitis B	Hepatitis B		Day -14 to Day -1	.		Negative	Normocytic, Normochromic RBCs seen on peripheral blood smear, not suggestive of hemolysis.
S267	309	Hepatitis B	Hepatitis B	2	Day 28	.			
S267	309	Hepatitis B	Hepatitis B	2	Day 56	.			
S267	309	Hepatitis B	Hepatitis B	3	Day 180	.			
S267	309	Hepatitis B	Hepatitis B	3	Day 208	.			
S267	309	Hepatitis B	Hepatitis B	3	Day 365	.			
S267	309	Hepatitis B	Hepatitis B		Unscheduled Visit	.			
S275	302	Hepatitis B	Hepatitis B		Day -14 to Day -1	.		Negative	Normocytic, Normochromic RBCs seen on peripheral blood smear, not suggestive of hemolysis.
S275	302	Hepatitis B	Hepatitis B	2	Day 28	.			
S275	302	Hepatitis B	Hepatitis B	2	Day 56	.			
S275	302	Hepatitis B	Hepatitis B	3	Day 180	.			
S275	302	Hepatitis B	Hepatitis B	3	Day 208	.			
S275	302	Hepatitis B	Hepatitis B	3	Day 365	.			

Subject No.	Randomisation Number	Study Group	Dosage	Dose	Day	Not Done	Specify	Result / Comment (CRF data-As per Investigator's discretion)	Result / Comment (As per Central laboratory)
S278	315	JAIVAC-1	Dosage C		Day -14 to Day -1	.		Negative	Normocytic, Normochromic RBCs seen on peripheral blood smear, not suggestive of hemolysis.
S278	315	JAIVAC-1	Dosage C	2	Day 28	.			
S278	315	JAIVAC-1	Dosage C	2	Day 56	.			
S278	315	JAIVAC-1	Dosage C	3	Day 180	.			
S278	315	JAIVAC-1	Dosage C	3	Day 208	.			
S278	315	JAIVAC-1	Dosage C	3	Day 365	.			
S279	301	JAIVAC-1	Dosage C		Day -14 to Day -1	.		Negative	Anisocytic (+) RBCs seen on peripheral blood smear, not suggestive of hemolysis.
S279	301	JAIVAC-1	Dosage C	2	Day 28	.			
S279	301	JAIVAC-1	Dosage C	2	Day 56	.			
S279	301	JAIVAC-1	Dosage C	3	Day 180	.			
S279	301	JAIVAC-1	Dosage C	3	Day 208	.			
S279	301	JAIVAC-1	Dosage C	3	Day 365	.			
S281	304	JAIVAC-1	Dosage C		Day -14 to Day -1	.		Negative	Normocytic, Normochromic RBCs seen on peripheral blood smear, not suggestive of hemolysis.
S281	304	JAIVAC-1	Dosage C	2	Day 28	.			
S281	304	JAIVAC-1	Dosage C	2	Day 56	.		Negative	Hypochromic (+), Microcytic (+) RBCs seen on peripheral blood smear, not suggestive of hemolysis.
S281	304	JAIVAC-1	Dosage C		Early Termination	.		Negative	Microcytic (+) RBCs seen on peripheral blood smear, not suggestive of hemolysis.
S284	305	JAIVAC-1	Dosage C		Day -14 to Day -1	.		Negative	Normocytic, Normochromic RBCs seen on peripheral blood smear, not suggestive of hemolysis.
S284	305	JAIVAC-1	Dosage C	2	Day 28	.			
S284	305	JAIVAC-1	Dosage C	2	Day 56	.			
S284	305	JAIVAC-1	Dosage C	3	Day 180	.			
S284	305	JAIVAC-1	Dosage C	3	Day 208	.			
S284	305	JAIVAC-1	Dosage C	3	Day 365	.			
S285	307	Hepatitis B	Hepatitis B		Day -14 to Day -1	.		Negative	Normocytic, Normochromic RBCs seen on peripheral blood smear, not suggestive of hemolysis.
S285	307	Hepatitis B	Hepatitis B	2	Day 28	.			

Subject No.	Randomisation Number	Study Group	Dosage	Dose	Day	Not Done	Specify	Result / Comment (CRF data-As per Investigator's discretion)	Result / Comment (As per Central laboratory)
S285	307	Hepatitis B	Hepatitis B	2	Day 56	.			
S285	307	Hepatitis B	Hepatitis B	3	Day 180	.			
S285	307	Hepatitis B	Hepatitis B	3	Day 208	.			
S285	307	Hepatitis B	Hepatitis B	3	Day 365	.			
S291	312	JAIVAC-1	Dosage C		Day -14 to Day -1	.		Negative	Normocytic, Normochromic RBCs seen on peripheral blood smear, not suggestive of hemolysis.
S291	312	JAIVAC-1	Dosage C	2	Day 28	.			
S291	312	JAIVAC-1	Dosage C	2	Day 56	.			
S291	312	JAIVAC-1	Dosage C	3	Day 180	.			
S291	312	JAIVAC-1	Dosage C	3	Day 208	.			
S291	312	JAIVAC-1	Dosage C	3	Day 365	.			
S298	314	JAIVAC-1	Dosage C		Day -14 to Day -1	.		Negative	Normocytic, Normochromic RBCs seen on peripheral blood smear, not suggestive of hemolysis.
S298	314	JAIVAC-1	Dosage C	2	Day 28	.			
S298	314	JAIVAC-1	Dosage C	2	Day 56	.			
S298	314	JAIVAC-1	Dosage C	3	Day 180	.			
S298	314	JAIVAC-1	Dosage C	3	Day 208	.			
S298	314	JAIVAC-1	Dosage C	3	Day 365	.			
S299	310	JAIVAC-1	Dosage C		Day -14 to Day -1	.		Negative	Normocytic, Normochromic RBCs seen on peripheral blood smear, not suggestive of hemolysis.
S299	310	JAIVAC-1	Dosage C	2	Day 28	.			
S299	310	JAIVAC-1	Dosage C	2	Day 56	.			
S299	310	JAIVAC-1	Dosage C	3	Day 180	.			
S299	310	JAIVAC-1	Dosage C	3	Day 208	.			
S299	310	JAIVAC-1	Dosage C	3	Day 365	.			

16.4 Individual Subject Data Listings

16.4.1: Listing of Individual Accrual Status (I of II)

Subject No.	Randomisation Number	Study Group	Dosage	Pre-Screening Day	Screening Day	Re-Screening Day	Randomisation Day	Day 7	Day 14	Day 28	Day 35
S097	.			09/08/2010	17/08/2010	.	.	.	.	.	.
S098	.			09/08/2010	17/08/2010	.	.	.	.	.	.
S099	.			09/08/2010	17/08/2010	.	.	.	.	.	.
S100	.			09/08/2010	18/08/2010	.	.	.	.	.	.
S101	.			09/08/2010	18/08/2010	.	.	.	.	.	.
S102	.			09/08/2010	18/08/2010	.	.	.	.	.	.
S103	.			09/08/2010	19/08/2010	.	.	.	.	.	.
S104	.			09/08/2010	19/08/2010	.	.	.	.	.	.
S105	.			09/08/2010	17/08/2010	.	.	.	.	.	.
S106	.			09/08/2010	17/08/2010	.	.	.	.	.	.
S107	.			09/08/2010	17/08/2010	.	.	.	.	.	.
S108	115	Hepatitis B	Hepatitis B	09/08/2010	18/08/2010	.	27/08/2010	03/09/2010	09/09/2010	24/09/2010	01/10/2010
S109	.			09/08/2010	17/08/2010	.	.	.	.	.	.
S110	.			09/08/2010	17/08/2010	.	.	.	.	.	.
S111	.			09/08/2010	18/08/2010	.	.	.	.	.	.
S112	.			09/08/2010	.	.	.	.	.	.	.
S113	.			09/08/2010	17/08/2010	.	.	.	.	.	.
S114	.			09/08/2010	.	.	.	.	.	.	.
S115	.			09/08/2010	17/08/2010	.	.	.	.	.	.
S116	.			09/08/2010	19/08/2010	.	.	.	.	.	.
S117	.			09/08/2010	19/08/2010	.	.	.	.	.	.
S118	114	JAIVAC-1	Dosage A	10/08/2010	17/08/2010	.	27/08/2010	03/09/2010	09/09/2010	24/09/2010	30/09/2010
S119	107	Hepatitis B	Hepatitis B	10/08/2010	18/08/2010	.	26/08/2010	02/09/2010	09/09/2010	23/09/2010	29/09/2010
S120	104	JAIVAC-1	Dosage A	10/08/2010	18/08/2010	.	26/08/2010	02/09/2010	08/09/2010	23/09/2010	30/09/2010
S121	.			10/08/2010	.	.	.	.	.	.	.

Subject No.	Randomisation Number	Study Group	Dosage	Pre-Screening Day	Screening Day	Re-Screening Day	Randomisation Day	Day 7	Day 14	Day 28	Day 35
S122	.			10/08/2010	.	.	.	.	.	.	.
S123	.			10/08/2010	19/08/2010	.	.	.	.	.	.
S124	106	Hepatitis B	Hepatitis B	10/08/2010	18/08/2010	.	26/08/2010	02/09/2010	08/09/2010	23/09/2010	29/09/2010
S125	102	JAIVAC-1	Dosage A	10/08/2010	18/08/2010	.	24/08/2010	31/08/2010	07/09/2010	21/09/2010	28/09/2010
S126	.			10/08/2010	17/08/2010	.	.	.	.	.	.
S127	.			10/08/2010	.	.	.	.	.	.	.
S128	.			11/08/2010	17/08/2010	.	.	.	.	.	.
S129	.			11/08/2010	18/08/2010	.	.	.	.	.	.
S130	105	JAIVAC-1	Dosage A	11/08/2010	17/08/2010	.	26/08/2010	02/09/2010	08/09/2010	23/09/2010	30/09/2010
S131	.			11/08/2010	18/08/2010	.	.	.	.	.	.
S132	108	Hepatitis B	Hepatitis B	11/08/2010	18/08/2010	.	26/08/2010	02/09/2010	09/09/2010	23/09/2010	30/09/2010
S133	.			11/08/2010	18/08/2010	.	.	.	.	.	.
S134	.			11/08/2010	19/08/2010	.	.	.	.	.	.
S135	.			12/08/2010	19/08/2010	.	.	.	.	.	.
S136	109	JAIVAC-1	Dosage A	12/08/2010	19/08/2010	.	26/08/2010	02/09/2010	09/09/2010	23/09/2010	30/09/2010
S137	.			12/08/2010	18/08/2010	.	.	.	.	.	.
S138	.			12/08/2010	18/08/2010	.	.	.	.	.	.
S139	.			12/08/2010	17/08/2010	.	.	.	.	.	.
S140	.			12/08/2010	18/08/2010	.	.	.	.	.	.
S141	.			12/08/2010	18/08/2010	.	.	.	.	.	.
S142	.			12/08/2010	.	.	.	.	.	.	.
S143	101	JAIVAC-1	Dosage A	12/08/2010	18/08/2010	.	24/08/2010	31/08/2010	07/09/2010	21/09/2010	28/09/2010
S144	103	Hepatitis B	Hepatitis B	13/08/2010	19/08/2010	.	24/08/2010	31/08/2010	07/09/2010	21/09/2010	28/09/2010
S145	112	JAIVAC-1	Dosage A	13/08/2010	19/08/2010	.	27/08/2010	04/09/2010	10/09/2010	24/09/2010	30/09/2010
S146	.			13/08/2010	19/08/2010	.	.	.	.	.	.
S147	.			13/08/2010	19/08/2010	.	.	.	.	.	.
S148	111	JAIVAC-1	Dosage A	13/08/2010	20/08/2010	.	27/08/2010	03/09/2010	10/09/2010	24/09/2010	30/09/2010
S149	.			13/08/2010	.	.	.	.	.	.	.
S150	.			13/08/2010	19/08/2010	.	.	.	.	.	.
S151	.			13/08/2010	20/08/2010	.	.	.	.	.	.

Subject No.	Randomisation Number	Study Group	Dosage	Pre-Screening Day	Screening Day	Re-Screening Day	Randomisation Day	Day 7	Day 14	Day 28	Day 35
S152	.			13/08/2010	19/08/2010	.	.	.	.	.	.
S153	.			13/08/2010	19/08/2010	.	.	.	.	.	.
S154	113	JAIVAC-1	Dosage A	13/08/2010	19/08/2010	.	27/08/2010	03/09/2010	09/09/2010	24/09/2010	30/09/2010
S155	.			13/08/2010	19/08/2010	.	.	.	.	.	.
S156	110	JAIVAC-1	Dosage A	13/08/2010	19/08/2010	.	27/08/2010	03/09/2010	09/09/2010	24/09/2010	01/10/2010
S157	.			13/08/2010	19/08/2010	.	.	.	.	.	.
S158	.			14/08/2010	20/08/2010	.	.	.	.	.	.
S159	.			14/08/2010	.	.	.	.	.	.	.
S160	.			14/08/2010	.	.	.	.	.	.	.
S161	.			14/08/2010	20/08/2010	.	.	.	.	.	.
S162	.			14/08/2010	.	.	.	.	.	.	.
S163	.			14/08/2010	.	.	.	.	.	.	.
S164	.			14/08/2010	.	.	.	.	.	.	.
S165	.			14/08/2010	.	.	.	.	.	.	.
S166	.			14/08/2010	19/08/2010	.	.	.	.	.	.
S167	.			14/09/2010	21/09/2010	.	.	.	.	.	.
S168	.			14/09/2010	.	.	.	.	.	.	.
S169	.			14/09/2010	21/09/2010	.	.	.	.	.	.
S170	204	Hepatitis B	Hepatitis B	14/09/2010	21/09/2010	.	29/09/2010	06/10/2010	13/10/2010	27/10/2010	03/11/2010
S171	.			14/09/2010	24/09/2010	.	.	.	.	.	.
S172	.			14/09/2010	.	.	.	.	.	.	.
S173	.			14/09/2010	23/09/2010	.	.	.	.	.	.
S174	.			14/09/2010	23/09/2010	.	.	.	.	.	.
S175	209	JAIVAC-1	Dosage B	14/09/2010	21/09/2010	.	29/09/2010	06/10/2010	13/10/2010	27/10/2010	03/11/2010
S176	205	Hepatitis B	Hepatitis B	14/09/2010	22/09/2010	.	29/09/2010	06/10/2010	13/10/2010	27/10/2010	03/11/2010
S177	.			14/09/2010	21/09/2010	.	.	.	.	.	.
S178	.			14/09/2010	23/09/2010	.	.	.	.	.	.
S179	207	Hepatitis B	Hepatitis B	14/09/2010	22/09/2010	.	29/09/2010	06/10/2010	13/10/2010	27/10/2010	03/11/2010
S180	.			14/09/2010	22/09/2010	.	.	.	.	.	.
S181	.			14/09/2010	23/09/2010	.	.	.	.	.	.

Subject No.	Randomisation Number	Study Group	Dosage	Pre-Screening Day	Screening Day	Re-Screening Day	Randomisation Day	Day 7	Day 14	Day 28	Day 35
S182	208	JAIVAC-1	Dosage B	15/09/2010	21/09/2010	.	29/09/2010	06/10/2010	13/10/2010	27/10/2010	03/11/2010
S183	.			15/09/2010	.	.	.	.	.	.	.
S184	.			15/09/2010	22/09/2010	.	.	.	.	.	.
S185	202	JAIVAC-1	Dosage B	15/09/2010	21/09/2010	.	28/09/2010	05/10/2010	13/10/2010	26/10/2010	02/11/2010
S186	201	JAIVAC-1	Dosage B	15/09/2010	21/09/2010	.	28/09/2010	05/10/2010	12/10/2010	26/10/2010	02/11/2010
S187	.			15/09/2010	22/09/2010	.	.	.	.	.	.
S188	.			15/09/2010	21/09/2010	.	.	.	.	.	.
S189	.			15/09/2010	.	.	.	.	.	.	.
S190	.			15/09/2010	22/09/2010	.	.	.	.	.	.
S191	.			15/09/2010	23/09/2010	.	.	.	.	.	.
S192	203	JAIVAC-1	Dosage B	16/09/2010	23/09/2010	.	28/09/2010	05/10/2010	12/10/2010	26/10/2010	02/11/2010
S193	215	JAIVAC-1	Dosage B	16/09/2010	24/09/2010	.	30/09/2010	07/10/2010	14/10/2010	28/10/2010	04/11/2010
S194	.			16/09/2010	.	.	.	.	.	.	.
S195	214	JAIVAC-1	Dosage B	16/09/2010	23/09/2010	.	30/09/2010	07/10/2010	14/10/2010	28/10/2010	04/11/2010
S196	.			16/09/2010	23/09/2010	.	.	.	.	.	.
S197	.			16/09/2010	.	.	.	.	.	.	.
S198	.			17/09/2010	.	.	.	.	.	.	.
S199	.			17/09/2010	.	.	.	.	.	.	.
S200	.			17/09/2010	23/09/2010	.	.	.	.	.	.
S201	.			17/09/2010	.	.	.	.	.	.	.
S202	206	JAIVAC-1	Dosage B	17/09/2010	23/09/2010	.	29/09/2010	07/10/2010	13/10/2010	27/10/2010	03/11/2010
S203	211	Hepatitis B	Hepatitis B	17/09/2010	24/09/2010	.	30/09/2010	07/10/2010	14/10/2010	28/10/2010	04/11/2010
S204	.			17/09/2010	.	.	.	.	.	.	.
S205	.			17/09/2010	.	.	.	.	.	.	.
S206	.			17/09/2010	23/09/2010	.	.	.	.	.	.
S207	.			17/09/2010	23/09/2010	.	.	.	.	.	.
S208	.			17/09/2010	23/09/2010	.	.	.	.	.	.
S209	.			17/09/2010	.	.	.	.	.	.	.
S210	.			17/09/2010	23/09/2010	.	.	.	.	.	.
S211	.			17/09/2010	.	.	.	.	.	.	.

Subject No.	Randomisation Number	Study Group	Dosage	Pre-Screening Day	Screening Day	Re-Screening Day	Randomisation Day	Day 7	Day 14	Day 28	Day 35
S212	212	JAIVAC-1	Dosage B	18/09/2010	23/09/2010	.	30/09/2010	07/10/2010	14/10/2010	28/10/2010	04/11/2010
S213	.			18/09/2010	.	.	.	.	.	.	.
S214	.			18/09/2010	24/09/2010	.	.	.	.	.	.
S215	213	Hepatitis B	Hepatitis B	18/09/2010	24/09/2010	.	30/09/2010	07/10/2010	14/10/2010	28/10/2010	04/11/2010
S216	.			18/09/2010	24/09/2010	.	.	.	.	.	.
S217	.			18/09/2010	.	.	.	.	.	.	.
S218	.			18/09/2010	24/09/2010	.	.	.	.	.	.
S219	.			18/09/2010	.	.	.	.	.	.	.
S220	.			18/09/2010	.	.	.	.	.	.	.
S221	.			18/09/2010	24/09/2010	.	.	.	.	.	.
S222	.			18/09/2010	.	.	.	.	.	.	.
S223	.			20/09/2010	.	.	.	.	.	.	.
S224	.			20/09/2010	.	.	.	.	.	.	.
S225	.			20/09/2010	.	.	.	.	.	.	.
S226	.			20/09/2010	24/09/2010	.	.	.	.	.	.
S227	.			20/09/2010	.	.	.	.	.	.	.
S228	.			20/09/2010	.	.	.	.	.	.	.
S229	.			20/09/2010	.	.	.	.	.	.	.
S230	.			20/09/2010	24/09/2010	.	.	.	.	.	.
S231	.			20/09/2010	.	.	.	.	.	.	.
S232	.			20/09/2010	.	.	.	.	.	.	.
S233	210	JAIVAC-1	Dosage B	20/09/2010	24/09/2010	.	30/09/2010	07/10/2010	14/10/2010	28/10/2010	04/11/2010
S234	.			20/09/2010	24/09/2010	.	.	.	.	.	.
S235	.			20/09/2010	24/09/2010	.	.	.	.	.	.
S236	.			25/10/2010	03/11/2010	.	.	.	.	.	.
S237	.			25/10/2010	03/11/2010	.	.	.	.	.	.
S238	.			25/10/2010	.	.	.	.	.	.	.
S239	.			25/10/2010	02/11/2010	.	.	.	.	.	.
S240	.			26/10/2010	.	.	.	.	.	.	.
S241	303	Hepatitis B	Hepatitis B	26/10/2010	03/11/2010	.	10/11/2010	17/11/2010	24/11/2010	08/12/2010	15/12/2010

Subject No.	Randomisation Number	Study Group	Dosage	Pre-Screening Day	Screening Day	Re-Screening Day	Randomisation Day	Day 7	Day 14	Day 28	Day 35
S242	.			26/10/2010	.	.	.	.	.	.	.
S243	.			26/10/2010	.	.	.	.	.	.	.
S244	.			26/10/2010	02/11/2010	.	.	.	.	.	.
S245	.			26/10/2010	.	.	.	.	.	.	.
S246	.			26/10/2010	03/11/2010	.	.	.	.	.	.
S247	.			26/10/2010	02/11/2010	.	.	.	.	.	.
S248	.			26/10/2010	.	.	.	.	.	.	.
S249	.			26/10/2010	02/11/2010	.	.	.	.	.	.
S250	311	JAIVAC-1	Dosage C	26/10/2010	03/11/2010	.	12/11/2010	19/11/2010	26/11/2010	10/12/2010	17/12/2010
S251	.			26/10/2010	02/11/2010	.	.	.	.	.	.
S252	.			26/10/2010	02/11/2010	.	.	.	.	.	.
S253	.			26/10/2010	.	.	.	.	.	.	.
S254	.			26/10/2010	03/11/2010	.	.	.	.	.	.
S255	.			26/10/2010	.	.	.	.	.	.	.
S256	.			27/10/2010	.	.	.	.	.	.	.
S257	.			27/10/2010	.	.	.	.	.	.	.
S258	.			27/10/2010	02/11/2010	.	.	.	.	.	.
S259	.			27/10/2010	.	.	.	.	.	.	.
S260	.			27/10/2010	02/11/2010	.	.	.	.	.	.
S261	.			27/10/2010	.	.	.	.	.	.	.
S262	.			27/10/2010	.	.	.	.	.	.	.
S263	313	Hepatitis B	Hepatitis B	27/10/2010	09/11/2010	.	12/11/2010	19/11/2010	26/11/2010	10/12/2010	17/12/2010
S264	306	JAIVAC-1	Dosage C	27/10/2010	03/11/2010	.	11/11/2010	18/11/2010	25/11/2010	09/12/2010	16/12/2010
S265	.			27/10/2010	.	.	.	.	.	.	.
S266	308	JAIVAC-1	Dosage C	27/10/2010	03/11/2010	.	11/11/2010	18/11/2010	25/11/2010	09/12/2010	16/12/2010
S267	309	Hepatitis B	Hepatitis B	27/10/2010	02/11/2010	.	11/11/2010	19/11/2010	25/11/2010	09/12/2010	16/12/2010
S268	.			27/10/2010	02/11/2010	.	.	.	.	.	.
S269	.			27/10/2010	.	.	.	.	.	.	.
S270	.			27/10/2010	.	.	.	.	.	.	.
S271	.			27/10/2010	.	.	.	.	.	.	.

Subject No.	Randomisation Number	Study Group	Dosage	Pre-Screening Day	Screening Day	Re-Screening Day	Randomisation Day	Day 7	Day 14	Day 28	Day 35
S272	.			27/10/2010	02/11/2010	.	.	.	.	.	.
S273	.			27/10/2010	02/11/2010	.	.	.	.	.	.
S274	.			27/10/2010	02/11/2010	.	.	.	.	.	.
S275	302	Hepatitis B	Hepatitis B	28/10/2010	02/11/2010	.	10/11/2010	17/11/2010	24/11/2010	08/12/2010	15/12/2010
S276	.			28/10/2010	10/11/2010	.	.	.	.	.	.
S277	.			28/10/2010	03/11/2010	.	.	.	.	.	.
S278	315	JAIVAC-1	Dosage C	28/10/2010	08/11/2010	.	12/11/2010	19/11/2010	26/11/2010	10/12/2010	17/12/2010
S279	301	JAIVAC-1	Dosage C	28/10/2010	03/11/2010	.	10/11/2010	17/11/2010	24/11/2010	08/12/2010	15/12/2010
S280	.			28/10/2010	03/11/2010	.	.	.	.	.	.
S281	304	JAIVAC-1	Dosage C	28/10/2010	03/11/2010	.	11/11/2010	18/11/2010	25/11/2010	09/12/2010	16/12/2010
S282	.			28/10/2010	08/11/2010	.	.	.	.	.	.
S283	.			29/10/2010	.	.	.	.	.	.	.
S284	305	JAIVAC-1	Dosage C	29/10/2010	03/11/2010	.	11/11/2010	18/11/2010	25/11/2010	09/12/2010	16/12/2010
S285	307	Hepatitis B	Hepatitis B	29/10/2010	02/11/2010	.	11/11/2010	18/11/2010	25/11/2010	09/12/2010	16/12/2010
S286	.			29/10/2010	03/11/2010	.	.	.	.	.	.
S287	.			29/10/2010	03/11/2010	.	.	.	.	.	.
S288	.			29/10/2010	.	.	.	.	.	.	.
S289	.			29/10/2010	02/11/2010	.	.	.	.	.	.
S290	.			29/10/2010	03/11/2010	.	.	.	.	.	.
S291	312	JAIVAC-1	Dosage C	29/10/2010	02/11/2010	.	12/11/2010	22/11/2010	26/11/2010	10/12/2010	17/12/2010
S292	.			29/10/2010	03/11/2010	.	.	.	.	.	.
S293	.			29/10/2010	.	.	.	.	.	.	.
S294	.			29/10/2010	03/11/2010	.	.	.	.	.	.
S295	.			29/10/2010	03/11/2010	.	.	.	.	.	.
S296	.			29/10/2010	02/11/2010	.	.	.	.	.	.
S297	.			29/10/2010	.	.	.	.	.	.	.
S298	314	JAIVAC-1	Dosage C	30/10/2010	09/11/2010	.	12/11/2010	19/11/2010	26/11/2010	10/12/2010	17/12/2010
S299	310	JAIVAC-1	Dosage C	30/10/2010	08/11/2010	.	12/11/2010	19/11/2010	26/11/2010	10/12/2010	17/12/2010
S300	.			30/10/2010	08/11/2010	.	.	.	.	.	.
S301	.			30/10/2010	.	.	.	.	.	.	.

Subject No.	Randomisation Number	Study Group	Dosage	Pre-Screening Day	Screening Day	Re-Screening Day	Randomisation Day	Day 7	Day 14	Day 28	Day 35
S302	.			30/10/2010	.	.	.	.	.	.	.
S303	.			30/10/2010	08/11/2010	.	.	.	.	.	.
S304	.			30/10/2010	08/11/2010	.	.	.	.	.	.
S305	.			30/10/2010	08/11/2010	.	.	.	.	.	.
S306	.			30/10/2010	.	.	.	.	.	.	.

16.4.1: Listing of Individual Accrual Status (II of II)

Subject No.	Randomisation Number	Study Group	Dosage	Day 42	Day 56	Day 180	Day 187	Day 194	Day 208	Day 365	Early Termination
S097	.			.	.	.	.	.	.	.	.
S098	.			.	.	.	.	.	.	.	.
S099	.			.	.	.	.	.	.	.	.
S100	.			.	.	.	.	.	.	.	.
S101	.			.	.	.	.	.	.	.	.
S102	.			.	.	.	.	.	.	.	.
S103	.			.	.	.	.	.	.	.	.
S104	.			.	.	.	.	.	.	.	.
S105	.			.	.	.	.	.	.	.	.
S106	.			.	.	.	.	.	.	.	.
S107	.			.	.	.	.	.	.	.	.
S108	115	Hepatitis B	Hepatitis B	08/10/2010	22/10/2010	23/02/2011	02/03/2011	09/03/2011	23/03/2011	26/08/2011	.
S109	.			.	.	.	.	.	.	.	.
S110	.			.	.	.	.	.	.	.	.
S111	.			.	.	.	.	.	.	.	.
S112	.			.	.	.	.	.	.	.	.
S113	.			.	.	.	.	.	.	.	.
S114	.			.	.	.	.	.	.	.	.
S115	.			.	.	.	.	.	.	.	.
S116	.			.	.	.	.	.	.	.	.
S117	.			.	.	.	.	.	.	.	.
S118	114	JAIVAC-1	Dosage A	08/10/2010	22/10/2010	23/02/2011	02/03/2011	09/03/2011	23/03/2011	26/08/2011	.
S119	107	Hepatitis B	Hepatitis B	06/10/2010	21/10/2010	22/02/2011	02/03/2011	08/03/2011	22/03/2011	25/08/2011	.
S120	104	JAIVAC-1	Dosage A	07/10/2010	21/10/2010	22/02/2011	01/03/2011	08/03/2011	22/03/2011	25/08/2011	.
S121	.			.	.	.	.	.	.	.	.
S122	.			.	.	.	.	.	.	.	.
S123	.			.	.	.	.	.	.	.	.
S124	106	Hepatitis B	Hepatitis B	07/10/2010	21/10/2010	22/02/2011	01/03/2011	08/03/2011	22/03/2011	25/08/2011	.

Subject No.	Randomisation Number	Study Group	Dosage	Day 42	Day 56	Day 180	Day 187	Day 194	Day 208	Day 365	Early Termination
S125	102	JAIVAC-1	Dosage A	05/10/2010	19/10/2010	21/02/2011	28/02/2011	07/03/2011	21/03/2011	26/08/2011	.
S126	.			.	.	.	.	.	.	.	.
S127	.			.	.	.	.	.	.	.	.
S128	.			.	.	.	.	.	.	.	.
S129	.			.	.	.	.	.	.	.	.
S130	105	JAIVAC-1	Dosage A	07/10/2010	21/10/2010	22/02/2011	01/03/2011	08/03/2011	22/03/2011	25/08/2011	.
S131	.			.	.	.	.	.	.	.	.
S132	108	Hepatitis B	Hepatitis B	07/10/2010	21/10/2010	22/02/2011	01/03/2011	08/03/2011	22/03/2011	25/08/2011	.
S133	.			.	.	.	.	.	.	.	.
S134	.			.	.	.	.	.	.	.	.
S135	.			.	.	.	.	.	.	.	.
S136	109	JAIVAC-1	Dosage A	07/10/2010	21/10/2010	22/02/2011	01/03/2011	08/03/2011	22/03/2011	25/08/2011	.
S137	.			.	.	.	.	.	.	.	.
S138	.			.	.	.	.	.	.	.	.
S139	.			.	.	.	.	.	.	.	.
S140	.			.	.	.	.	.	.	.	.
S141	.			.	.	.	.	.	.	.	.
S142	.			.	.	.	.	.	.	.	.
S143	101	JAIVAC-1	Dosage A	05/10/2010	19/10/2010	21/02/2011	28/02/2011	07/03/2011	21/03/2011	26/08/2011	.
S144	103	Hepatitis B	Hepatitis B	05/10/2010	19/10/2010	21/02/2011	28/02/2011	07/03/2011	21/03/2011	26/08/2011	.
S145	112	JAIVAC-1	Dosage A	08/10/2010	22/10/2010	23/02/2011	02/03/2011	09/03/2011	23/03/2011	26/08/2011	.
S146	.			.	.	.	.	.	.	.	.
S147	.			.	.	.	.	.	.	.	.
S148	111	JAIVAC-1	Dosage A	08/10/2010	22/10/2010	23/02/2011	03/03/2011	09/03/2011	23/03/2011	26/08/2011	.
S149	.			.	.	.	.	.	.	.	.
S150	.			.	.	.	.	.	.	.	.
S151	.			.	.	.	.	.	.	.	.
S152	.			.	.	.	.	.	.	.	.
S153	.			.	.	.	.	.	.	.	.
S154	113	JAIVAC-1	Dosage A	08/10/2010	22/10/2010	23/02/2011	01/03/2011	09/03/2011	23/03/2011	26/08/2011	.

Subject No.	Randomisation Number	Study Group	Dosage	Day 42	Day 56	Day 180	Day 187	Day 194	Day 208	Day 365	Early Termination
S155	.			.	.	.	.	.	.	.	.
S156	110	JAIVAC-1	Dosage A	08/10/2010	22/10/2010	23/02/2011	02/03/2011	09/03/2011	23/03/2011	26/08/2011	.
S157	.			.	.	.	.	.	.	.	.
S158	.			.	.	.	.	.	.	.	.
S159	.			.	.	.	.	.	.	.	.
S160	.			.	.	.	.	.	.	.	.
S161	.			.	.	.	.	.	.	.	.
S162	.			.	.	.	.	.	.	.	.
S163	.			.	.	.	.	.	.	.	.
S164	.			.	.	.	.	.	.	.	.
S165	.			.	.	.	.	.	.	.	.
S166	.			.	.	.	.	.	.	.	.
S167	.			.	.	.	.	.	.	.	.
S168	.			.	.	.	.	.	.	.	.
S169	.			.	.	.	.	.	.	.	.
S170	204	Hepatitis B	Hepatitis B	10/11/2010	25/11/2010	28/03/2011	06/04/2011	11/04/2011	25/04/2011	28/09/2011	.
S171	.			.	.	.	.	.	.	.	.
S172	.			.	.	.	.	.	.	.	.
S173	.			.	.	.	.	.	.	.	.
S174	.			.	.	.	.	.	.	.	.
S175	209	JAIVAC-1	Dosage B	10/11/2010	24/11/2010	28/03/2011	05/04/2011	11/04/2011	25/04/2011	28/09/2011	.
S176	205	Hepatitis B	Hepatitis B	10/11/2010	24/11/2010	28/03/2011	05/04/2011	11/04/2011	25/04/2011	28/09/2011	.
S177	.			.	.	.	.	.	.	.	.
S178	.			.	.	.	.	.	.	.	.
S179	207	Hepatitis B	Hepatitis B	10/11/2010	24/11/2010	28/03/2011	06/04/2011	11/04/2011	25/04/2011	28/09/2011	.
S180	.			.	.	.	.	.	.	.	.
S181	.			.	.	.	.	.	.	.	.
S182	208	JAIVAC-1	Dosage B	10/11/2010	24/11/2010	28/03/2011	05/04/2011	11/04/2011	25/04/2011	29/09/2011	.
S183	.			.	.	.	.	.	.	.	.
S184	.			.	.	.	.	.	.	.	.

Subject No.	Randomisation Number	Study Group	Dosage	Day 42	Day 56	Day 180	Day 187	Day 194	Day 208	Day 365	Early Termination
S185	202	JAIVAC-1	Dosage B	10/11/2010	23/11/2010	28/03/2011	02/04/2011	11/04/2011	25/04/2011	28/09/2011	.
S186	201	JAIVAC-1	Dosage B	09/11/2010	23/11/2010	28/03/2011	02/04/2011	11/04/2011	25/04/2011	28/09/2011	.
S187	.			.	.	.	.	.	.	.	.
S188	.			.	.	.	.	.	.	.	.
S189	.			.	.	.	.	.	.	.	.
S190	.			.	.	.	.	.	.	.	.
S191	.			.	.	.	.	.	.	.	.
S192	203	JAIVAC-1	Dosage B	09/11/2010	23/11/2010	28/03/2011	02/04/2011	11/04/2011	25/04/2011	28/09/2011	.
S193	215	JAIVAC-1	Dosage B	11/11/2010	25/11/2010	29/03/2011	05/04/2011	12/04/2011	26/04/2011	29/09/2011	.
S194	.			.	.	.	.	.	.	.	.
S195	214	JAIVAC-1	Dosage B	11/11/2010	24/11/2010	29/03/2011	05/04/2011	12/04/2011	26/04/2011	29/09/2011	.
S196	.			.	.	.	.	.	.	.	.
S197	.			.	.	.	.	.	.	.	.
S198	.			.	.	.	.	.	.	.	.
S199	.			.	.	.	.	.	.	.	.
S200	.			.	.	.	.	.	.	.	.
S201	.			.	.	.	.	.	.	.	.
S202	206	JAIVAC-1	Dosage B	10/11/2010	24/11/2010	28/03/2011	05/04/2011	11/04/2011	25/04/2011	29/09/2011	.
S203	211	Hepatitis B	Hepatitis B	11/11/2010	25/11/2010	29/03/2011	05/04/2011	12/04/2011	28/04/2011	29/09/2011	.
S204	.			.	.	.	.	.	.	.	.
S205	.			.	.	.	.	.	.	.	.
S206	.			.	.	.	.	.	.	.	.
S207	.			.	.	.	.	.	.	.	.
S208	.			.	.	.	.	.	.	.	.
S209	.			.	.	.	.	.	.	.	.
S210	.			.	.	.	.	.	.	.	.
S211	.			.	.	.	.	.	.	.	.
S212	212	JAIVAC-1	Dosage B	11/11/2010	25/11/2010	29/03/2011	05/04/2011	12/04/2011	19/04/2011	29/09/2011	.
S213	.			.	.	.	.	.	.	.	.
S214	.			.	.	.	.	.	.	.	.

Subject No.	Randomisation Number	Study Group	Dosage	Day 42	Day 56	Day 180	Day 187	Day 194	Day 208	Day 365	Early Termination
S215	213	Hepatitis B	Hepatitis B	11/11/2010	25/11/2010	29/03/2011	06/04/2011	12/04/2011	26/04/2011	29/09/2011	.
S216	.			.	.	.	.	.	.	.	.
S217	.			.	.	.	.	.	.	.	.
S218	.			.	.	.	.	.	.	.	.
S219	.			.	.	.	.	.	.	.	.
S220	.			.	.	.	.	.	.	.	.
S221	.			.	.	.	.	.	.	.	.
S222	.			.	.	.	.	.	.	.	.
S223	.			.	.	.	.	.	.	.	.
S224	.			.	.	.	.	.	.	.	.
S225	.			.	.	.	.	.	.	.	.
S226	.			.	.	.	.	.	.	.	.
S227	.			.	.	.	.	.	.	.	.
S228	.			.	.	.	.	.	.	.	.
S229	.			.	.	.	.	.	.	.	.
S230	.			.	.	.	.	.	.	.	.
S231	.			.	.	.	.	.	.	.	.
S232	.			.	.	.	.	.	.	.	.
S233	210	JAIVAC-1	Dosage B	11/11/2010	25/11/2010	29/03/2011	05/04/2011	13/04/2011	27/04/2011	29/09/2011	.
S234	.			.	.	.	.	.	.	.	.
S235	.			.	.	.	.	.	.	.	.
S236	.			.	.	.	.	.	.	.	.
S237	.			.	.	.	.	.	.	.	.
S238	.			.	.	.	.	.	.	.	.
S239	.			.	.	.	.	.	.	.	.
S240	.			.	.	.	.	.	.	.	.
S241	303	Hepatitis B	Hepatitis B	22/12/2010	05/01/2011	09/05/2011	16/05/2011	23/05/2011	06/06/2011	09/11/2011	.
S242	.			.	.	.	.	.	.	.	.
S243	.			.	.	.	.	.	.	.	.
S244	.			.	.	.	.	.	.	.	.

Subject No.	Randomisation Number	Study Group	Dosage	Day 42	Day 56	Day 180	Day 187	Day 194	Day 208	Day 365	Early Termination
S245	.			.	.	.	.	.	.	.	.
S246	.			.	.	.	.	.	.	.	.
S247	.			.	.	.	.	.	.	.	.
S248	.			.	.	.	.	.	.	.	.
S249	.			.	.	.	.	.	.	.	.
S250	311	JAIVAC-1	Dosage C	24/12/2010	07/01/2011	11/05/2011	18/05/2011	24/05/2011	08/06/2011	10/11/2011	.
S251	.			.	.	.	.	.	.	.	.
S252	.			.	.	.	.	.	.	.	.
S253	.			.	.	.	.	.	.	.	.
S254	.			.	.	.	.	.	.	.	.
S255	.			.	.	.	.	.	.	.	.
S256	.			.	.	.	.	.	.	.	.
S257	.			.	.	.	.	.	.	.	.
S258	.			.	.	.	.	.	.	.	.
S259	.			.	.	.	.	.	.	.	.
S260	.			.	.	.	.	.	.	.	.
S261	.			.	.	.	.	.	.	.	.
S262	.			.	.	.	.	.	.	.	.
S263	313	Hepatitis B	Hepatitis B	24/12/2010	07/01/2011	11/05/2011	18/05/2011	25/05/2011	08/06/2011	10/11/2011	.
S264	306	JAIVAC-1	Dosage C	23/12/2010	06/01/2011	10/05/2011	17/05/2011	24/05/2011	07/06/2011	09/11/2011	.
S265	.			.	.	.	.	.	.	.	.
S266	308	JAIVAC-1	Dosage C	23/12/2010	06/01/2011	10/05/2011	17/05/2011	24/05/2011	07/06/2011	09/11/2011	.
S267	309	Hepatitis B	Hepatitis B	23/12/2010	06/01/2011	10/05/2011	17/05/2011	24/05/2011	07/06/2011	09/11/2011	.
S268	.			.	.	.	.	.	.	.	.
S269	.			.	.	.	.	.	.	.	.
S270	.			.	.	.	.	.	.	.	.
S271	.			.	.	.	.	.	.	.	.
S272	.			.	.	.	.	.	.	.	.
S273	.			.	.	.	.	.	.	.	.
S274	.			.	.	.	.	.	.	.	.

Subject No.	Randomisation Number	Study Group	Dosage	Day 42	Day 56	Day 180	Day 187	Day 194	Day 208	Day 365	Early Termination
S275	302	Hepatitis B	Hepatitis B	22/12/2010	05/01/2011	09/05/2011	16/05/2011	23/05/2011	06/06/2011	09/11/2011	.
S276	.			.	.	.	.	.	.	.	.
S277	.			.	.	.	.	.	.	.	.
S278	315	JAIVAC-1	Dosage C	24/12/2010	07/01/2011	11/05/2011	18/05/2011	24/05/2011	09/06/2011	10/11/2011	.
S279	301	JAIVAC-1	Dosage C	22/12/2010	05/01/2011	09/05/2011	16/05/2011	31/05/2011	06/06/2011	09/11/2011	.
S280	.			.	.	.	.	.	.	.	.
S281	304	JAIVAC-1	Dosage C	27/12/2010	06/01/2011	.	.	.	.	.	11/05/2011
S282	.			.	.	.	.	.	.	.	.
S283	.			.	.	.	.	.	.	.	.
S284	305	JAIVAC-1	Dosage C	23/12/2010	06/01/2011	10/05/2011	17/05/2011	24/05/2011	07/06/2011	09/11/2011	.
S285	307	Hepatitis B	Hepatitis B	23/12/2010	06/01/2011	10/05/2011	17/05/2011	24/05/2011	07/06/2011	09/11/2011	.
S286	.			.	.	.	.	.	.	.	.
S287	.			.	.	.	.	.	.	.	.
S288	.			.	.	.	.	.	.	.	.
S289	.			.	.	.	.	.	.	.	.
S290	.			.	.	.	.	.	.	.	.
S291	312	JAIVAC-1	Dosage C	24/12/2010	07/01/2011	11/05/2011	18/05/2011	25/05/2011	08/06/2011	10/11/2011	.
S292	.			.	.	.	.	.	.	.	.
S293	.			.	.	.	.	.	.	.	.
S294	.			.	.	.	.	.	.	.	.
S295	.			.	.	.	.	.	.	.	.
S296	.			.	.	.	.	.	.	.	.
S297	.			.	.	.	.	.	.	.	.
S298	314	JAIVAC-1	Dosage C	24/12/2010	07/01/2011	11/05/2011	18/05/2011	25/05/2011	08/06/2011	10/11/2011	.
S299	310	JAIVAC-1	Dosage C	24/12/2010	07/01/2011	11/05/2011	18/05/2011	25/05/2011	08/06/2011	10/11/2011	.
S300	.			.	.	.	.	.	.	.	.
S301	.			.	.	.	.	.	.	.	.
S302	.			.	.	.	.	.	.	.	.
S303	.			.	.	.	.	.	.	.	.
S304	.			.	.	.	.	.	.	.	.

Subject No.	Randomisation Number	Study Group	Dosage	Day 42	Day 56	Day 180	Day 187	Day 194	Day 208	Day 365	Early Termination
S305	.			.	.	.	.	.	.	.	.
S306	.			.	.	.	.	.	.	.	.

16.4.2: Listing of Subject Eligibility Criteria

Subject No.	Randomisation Number	Study Group	Dosage	Is the Subject Pre-Screen Failure?	Is the Subject Screen Failure?	Reason for Screen Failure	Is the Subject Re-Screen Failure?	Reason for Re-Screen Failure	Is the Subject Randomised?	Is the Subject Eligible?
S097	.			No	Yes	Subject not fulfilling the Exclusion criteria		.		No
S098	.			No	Yes	Subject not fulfilling the Exclusion criteria		.		No
S099	.			No	No	.		.		Yes
S100	.			No	Yes	Subject not fulfilling the Exclusion criteria		.		No
S101	.			No	Yes	Subject not fulfilling the Exclusion criteria		.		No
S102	.			No	Yes	Subject not fulfilling the Exclusion criteria		.		No
S103	.			No	No	.		.		Yes
S104	.			No	Yes	Subject not fulfilling the Exclusion criteria		.		No
S105	.			No	Yes	Subject not fulfilling the Exclusion criteria		.		No
S106	.			No	No	.		.		Yes
S107	.			No	Yes	Subject not fulfilling the Exclusion criteria		.		No
S108	115	Hepatitis B	Hepatitis B	No	No	.		.	Yes	Yes
S109	.			No	Yes	Subject not fulfilling the Exclusion criteria		.		No
S110	.			No	Yes	Subject not fulfilling the Exclusion criteria		.		No
S111	.			No	Yes	Subject not fulfilling the Exclusion criteria		.		No
S113	.			No	Yes	Subject not fulfilling the Inclusion criteria		.		No
S115	.			No	Yes	Subject withdrew consent		.		No
S116	.			No	No	.		.		Yes
S117	.			No	No	.		.		Yes
S118	114	JAIVAC-1	Dosage A	No	No	.		.	Yes	Yes
S119	107	Hepatitis B	Hepatitis B	No	No	.		.	Yes	Yes
S120	104	JAIVAC-1	Dosage A	No	No	.		.	Yes	Yes
S123	.			No	No	.		.		Yes
S124	106	Hepatitis B	Hepatitis B	No	No	.		.	Yes	Yes
S125	102	JAIVAC-1	Dosage A	No	No	.		.	Yes	Yes
S126	.			No	Yes	Subject not fulfilling the Exclusion criteria		.		No
S128	.			No	Yes	Subject not fulfilling the Exclusion criteria		.		No

Subject No.	Randomisation Number	Study Group	Dosage	Is the Subject Pre-Screen Failure?	Is the Subject Screen Failure?	Reason for Screen Failure	Is the Subject Re-Screen Failure?	Reason for Re-Screen Failure	Is the Subject Randomised?	Is the Subject Eligible?
S129	.			No	Yes	Subject not fulfilling the Exclusion criteria		.		No
S130	105	JAIVAC-1	Dosage A	No	No	.		.	Yes	Yes
S131	.			No	Yes	Subject not fulfilling the Exclusion criteria		.		No
S132	108	Hepatitis B	Hepatitis B	No	No	.		.	Yes	Yes
S133	.			No	No	.		.		Yes
S134	.			No	Yes	Subject withdrew consent		.		No
S135	.			No	Yes	Subject not fulfilling the Exclusion criteria		.		No
S136	109	JAIVAC-1	Dosage A	No	No	.		.	Yes	Yes
S137	.			No	Yes	Subject not fulfilling the Exclusion criteria		.		No
S138	.			No	No	.		.		Yes
S139	.			No	Yes	Subject not fulfilling the Exclusion criteria		.		No
S140	.			No	No	.		.		Yes
S141	.			No	No	.		.		Yes
S143	101	JAIVAC-1	Dosage A	No	No	.		.	Yes	Yes
S144	103	Hepatitis B	Hepatitis B	No	No	.		.	Yes	Yes
S145	112	JAIVAC-1	Dosage A	No	No	.		.	Yes	Yes
S146	.			No	Yes	Subject not fulfilling the Exclusion criteria		.		No
S147	.			No	No	.		.		Yes
S148	111	JAIVAC-1	Dosage A	No	No	.		.	Yes	Yes
S150	.			No	No	.		.		Yes
S151	.			No	Yes	Subject not fulfilling the Inclusion criteria		.		No
S152	.			No	Yes	Subject not fulfilling the Exclusion criteria		.		No
S153	.			No	Yes	Subject not fulfilling the Exclusion criteria		.		No
S154	113	JAIVAC-1	Dosage A	No	No	.		.	Yes	Yes
S155	.			No	Yes	Subject not fulfilling the Exclusion criteria		.		No
S156	110	JAIVAC-1	Dosage A	No	No	.		.	Yes	Yes
S157	.			No	No	.		.		Yes
S158	.			No	Yes	Subject not fulfilling the Exclusion criteria		.		No

Subject No.	Randomisation Number	Study Group	Dosage	Is the Subject Pre-Screen Failure?	Is the Subject Screen Failure?	Reason for Screen Failure	Is the Subject Re-Screen Failure?	Reason for Re-Screen Failure	Is the Subject Randomised?	Is the Subject Eligible?
S161	.			No	No	.		.		Yes
S166	.			No	Yes	Subject not fulfilling the Exclusion criteria		.		No
S167	.			No	Yes	Subject not fulfilling the Exclusion criteria		.		No
S169	.			No	Yes	Subject not fulfilling the Inclusion criteria		.		No
S170	204	Hepatitis B	Hepatitis B	No	No	.		.	Yes	Yes
S171	.			No	No	.		.		Yes
S173	.			No	Yes	Subject not fulfilling the Exclusion criteria		.		No
S174	.			No	Yes	Subject not fulfilling the Exclusion criteria		.		No
S175	209	JAIVAC-1	Dosage B	No	No	.		.	Yes	Yes
S176	205	Hepatitis B	Hepatitis B	No	No	.		.	Yes	Yes
S177	.			No	Yes	Subject not fulfilling the Inclusion criteria		.		No
S178	.			No	Yes	Subject not fulfilling the Exclusion criteria		.		No
S179	207	Hepatitis B	Hepatitis B	No	No	.		.	Yes	Yes
S180	.			No	Yes	Subject not fulfilling the Exclusion criteria		.		No
S181	.			No	Yes	Subject not fulfilling the Exclusion criteria		.		No
S182	208	JAIVAC-1	Dosage B	No	No	.		.	Yes	Yes
S184	.			No	No	.		.		Yes
S185	202	JAIVAC-1	Dosage B	No	No	.		.	Yes	Yes
S186	201	JAIVAC-1	Dosage B	No	No	.		.	Yes	Yes
S187	.			No	Yes	Subject not fulfilling the Inclusion criteria		.		No
S188	.			No	No	.		.		Yes
S190	.			No	Yes	Subject not fulfilling the Exclusion criteria		.		No
S191	.			No	Yes	Subject not fulfilling the Inclusion criteria		.		No
S192	203	JAIVAC-1	Dosage B	No	No	.		.	Yes	Yes
S193	215	JAIVAC-1	Dosage B	No	No	.		.	Yes	Yes
S195	214	JAIVAC-1	Dosage B	No	No	.		.	Yes	Yes
S196	.			No	Yes	Subject not fulfilling the Exclusion criteria		.		No
S200	.			No	No	.		.		Yes

Subject No.	Randomisation Number	Study Group	Dosage	Is the Subject Pre-Screen Failure?	Is the Subject Screen Failure?	Reason for Screen Failure	Is the Subject Re-Screen Failure?	Reason for Re-Screen Failure	Is the Subject Randomised?	Is the Subject Eligible?
S202	206	JAIVAC-1	Dosage B	No	No	.		.	Yes	Yes
S203	211	Hepatitis B	Hepatitis B	No	No	.		.	Yes	Yes
S206	.			No	Yes	Subject not fulfilling the Inclusion criteria		.		No
S207	.			No	No	.		.		Yes
S208	.			No	Yes	Subject not fulfilling the Exclusion criteria		.		No
S210	.			No	Yes	Subject not fulfilling the Exclusion criteria		.		No
S212	212	JAIVAC-1	Dosage B	No	No	.		.	Yes	Yes
S214	.			No	Yes	Subject not fulfilling the Exclusion criteria		.		No
S215	213	Hepatitis B	Hepatitis B	No	No	.		.	Yes	Yes
S216	.			No	Yes	Subject not fulfilling the Exclusion criteria		.		No
S218	.			No	No	.		.		Yes
S221	.			No	No	.		.		Yes
S226	.			No	Yes	Subject not fulfilling the Exclusion criteria		.		No
S230	.			No	No	.		.		Yes
S233	210	JAIVAC-1	Dosage B	No	No	.		.	Yes	Yes
S234	.			No	Yes	Subject not fulfilling the Exclusion criteria		.		No
S235	.			No	Yes	Subject not fulfilling the Exclusion criteria		.		No
S236	.			No	Yes	Subject not fulfilling the Exclusion criteria		.		No
S237	.			No	Yes	Subject not fulfilling the Exclusion criteria		.		No
S239	.			No	Yes	Subject not fulfilling the Exclusion criteria		.		No
S241	303	Hepatitis B	Hepatitis B	No	No	.		.	Yes	Yes
S244	.			No	Yes	Subject not fulfilling the Exclusion criteria		.		No
S246	.			No	Yes	Subject not fulfilling the Exclusion criteria		.		No
S247	.			No	Yes	Subject not fulfilling the Exclusion criteria		.		No
S249	.			No	Yes	Subject not fulfilling the Exclusion criteria		.		No
S250	311	JAIVAC-1	Dosage C	No	No	.		.	Yes	Yes
S251	.			No	Yes	Subject not fulfilling the Exclusion criteria		.		No
S252	.			No	Yes	Subject not fulfilling the Inclusion criteria		.		No

Subject No.	Randomisation Number	Study Group	Dosage	Is the Subject Pre-Screen Failure?	Is the Subject Screen Failure?	Reason for Screen Failure	Is the Subject Re-Screen Failure?	Reason for Re-Screen Failure	Is the Subject Randomised?	Is the Subject Eligible?
S254	.			No	Yes	Subject not fulfilling the Exclusion criteria		.		No
S258	.			No	No	.		.		Yes
S260	.			No	No	.		.		Yes
S263	313	Hepatitis B	Hepatitis B	No	No	.		.	Yes	Yes
S264	306	JAIVAC-1	Dosage C	No	No	.		.	Yes	Yes
S266	308	JAIVAC-1	Dosage C	No	No	.		.	Yes	Yes
S267	309	Hepatitis B	Hepatitis B	No	No	.		.	Yes	Yes
S268	.			No	Yes	Subject not fulfilling the Exclusion criteria		.		No
S272	.			No	No	.		.		Yes
S273	.			No	Yes	Other		.		No
S274	.			No	No	.		.		Yes
S275	302	Hepatitis B	Hepatitis B	No	No	.		.	Yes	Yes
S276	.			No	Yes	Subject not fulfilling the Exclusion criteria		.		No
S277	.			No	No	.		.		Yes
S278	315	JAIVAC-1	Dosage C	No	No	.		.	Yes	Yes
S279	301	JAIVAC-1	Dosage C	No	No	.		.	Yes	Yes
S280	.			No	Yes	Subject not fulfilling the Exclusion criteria		.		No
S281	304	JAIVAC-1	Dosage C	No	No	.		.	Yes	Yes
S282	.			No	Yes	Subject not fulfilling the Exclusion criteria		.		No
S284	305	JAIVAC-1	Dosage C	No	No	.		.	Yes	Yes
S285	307	Hepatitis B	Hepatitis B	No	No	.		.	Yes	Yes
S286	.			No	Yes	Subject not fulfilling the Exclusion criteria		.		No
S287	.			No	Yes	Subject not fulfilling the Inclusion criteria		.		No
S289	.			No	Yes	Subject not fulfilling the Exclusion criteria		.		No
S290	.			No	Yes	Subject not fulfilling the Exclusion criteria		.		No
S291	312	JAIVAC-1	Dosage C	No	No	.		.	Yes	Yes
S292	.			No	Yes	Subject not fulfilling the Inclusion criteria		.		No
S294	.			No	Yes	Subject not fulfilling the Exclusion criteria		.		No

Subject No.	Randomisation Number	Study Group	Dosage	Is the Subject Pre-Screen Failure?	Is the Subject Screen Failure?	Reason for Screen Failure	Is the Subject Re-Screen Failure?	Reason for Re-Screen Failure	Is the Subject Randomised?	Is the Subject Eligible?
S295	.			No	Yes	Subject not fulfilling the Exclusion criteria		.		No
S296	.			No	Yes	Subject not fulfilling the Exclusion criteria		.		No
S298	314	JAIVAC-1	Dosage C	No	No	.		.	Yes	Yes
S299	310	JAIVAC-1	Dosage C	No	No	.		.	Yes	Yes
S300	.			No	Yes	Subject not fulfilling the Exclusion criteria		.		No
S303	.			No	Yes	Subject not fulfilling the Exclusion criteria		.		No
S304	.			No	No	.		.		Yes
S305	.			No	Yes	Subject not fulfilling the Exclusion criteria		.		No

Note:
1) Reason for Screen/Re-Screen Failure: 1-Subject not fulfilling the Inclusion criteria, 2-Subject not fulfilling the Exclusion criteria, 3-Subject withdrew consent, 4-Lost to follow up, 5-Other

16.4.3: Listing of Subject Inclusion and Exclusion Criteria Details

Criteria Type	Criteria Number	Criteria Description
Inclusion	1	Male subject aged 18 to 45 years (both inclusive)
	2	Subject with general good health based on the medical history and clinical examination
	3	Subject must be willing to sign the Informed Consent Form
	4	Subject must be reachable by phone during the entire study period (12 months)
	5	Subject must be capable and willing to complete and return diary cards and to attend all follow-up visits
	6	Male subject must agree to use one of the following medicallyacceptable birth control measures throughout the duration of the study (birth control counselling and measures will be provided by sites as required)
	6a	Double barrier method (e.g. condom with spermicidal jelly) used for the entire study period
	6b	Should be Surgically sterile (vasectomy)
Exclusion	1	Subject with evidence of IgG antibodies against PfMSP-119 and PfF2 as measured by ELISA
	2	Subject with prior history of immunisation with Hepatitis B vaccine
	3	Subject with known history of malaria
	4	Subject with history of allergic reactions, hypersensitivity or anaphylaxis to any of the components of the study vaccines (JAIVAC-1 – Montanide ISA 720 malaria vaccine or Hepatitis B vaccine) (including adjuvant or peptide) or with history of serious allergic reactions to any substance, requiring hospitalisation or emergency medical care
	5	Subject with previous vaccination with any other malaria candidate vaccines
	6	Subject with use of an investigational or non-registered drug or vaccine other than the study vaccines within three (3) months preceding the first study vaccination, or planned use during the entire clinical trial period
	7	Subject, who receives any vaccination or gamma globulin during the three-month period prior to the first vaccination
	8	Subject with chronic administration (defined as more than 14 days) of immuno-suppressants or other immune-modifying drugs within six months prior to the first vaccination. This includes any dose level of oral steroids or inhaled steroids, but not topical steroids
	9	Subjects will be excluded if AST > 40 IU/L, ALT > 41 IU/L, γ GT > 71 IU/L, Total Bilirubin > 1.2 mg/ dL, Indirect Bilirubin > 1.2 mg/ dL, Direct Bilirubin > 0.4 mg/dL, Serum Creatinine > 1.2 mg/ dL(Appendix B and B-1)
	10	Subjects will be excluded in case of out of range values for the following parameters: Hemoglobin 13 to 18 g/ dL, RBC count 4.0 to 7.0 × 10E6/µL TLC 4.0 to 11.0 × 10E3/µL, platelet count 150 to 500 × 10E3/µL, Neutrophils 40 to 75 % or Eosinophils > 10 %, Sodium 136 to 145 mEq/L, Potassium 3.5 to 5.1 mEq/L, Random Blood Glucose 45 to 130 mg/dl and Alkaline Phosphatase 40 to 129 U/L (Appendix B and B-1)
	11	Subjects with other clinically significant abnormal laboratory values based on the normal reference range (Refer Appendix B and B1) apart from the laboratory parameters listed above.
	12	Subject with positive HIV, HBV or HCV test

Criteria Type	Criteria Number	Criteria Description
	13	Subject with positive direct Coomb's test
	14	Subject with a peripheral blood film/smear with features of haemolysis or any other haematological disorder
	15	Subject unable to give written informed consent
	16	Subject unable to be closely followed for social, geographic or psychological reasons
	17	Subject with previous history of drug or alcohol abuse interfering with normal social function during a period of one year prior to enrolment in the study or positive urine drug test
	18	Subject with an abnormal 12-lead ECG at screening associated with relevant clinical symptoms/signs suggestive of cardiac pathology (including conduction disturbances)
	19	Subject with an abnormal Chest X-Ray associated with relevant clinical symptoms/signs of respiratory pathology at screening/ anytime in the past 6 months
	20	Subject with known history of congenital/hereditary immunodeficiency disorders
	21	Subject with acute or chronic pulmonary, reticulo-endothelial, cardiovascular, hepatic, renal or neurological condition, or any other clinical findings that in the opinion of the clinical investigator, may increase the risk of participating in the study
	22	Subject with any other condition that in the opinion of the clinical investigator would jeopardise the safety or rights of a participant in the trial or would render the participant unable to comply with the protocol

16.4.4: Listing of Subject Inclusion Criteria

Subject No.	Randomisation Number	Study Group	Dosage	No.1	No.2	No.3	No.4	No.5	No.6	* No.6a,b	All Inclusion Criteria Met?
S097	.			Yes	Yes	Yes	Yes	Yes	Yes	1	Yes
S098	.			Yes	Yes	Yes	Yes	Yes	Yes	1	Yes
S099	.			Yes	Yes	Yes	Yes	Yes	Yes	1	Yes
S100	.			Yes	Yes	Yes	Yes	Yes	Yes	1	Yes
S101	.			Yes	Yes	Yes	Yes	Yes	Yes	1	Yes
S102	.			Yes	Yes	Yes	Yes	Yes	Yes	1	Yes
S103	.			Yes	Yes	Yes	Yes	Yes	Yes	1	Yes
S104	.			Yes	Yes	Yes	Yes	Yes	Yes	1	Yes
S105	.			Yes	Yes	Yes	Yes	Yes	Yes	1	Yes
S106	.			Yes	Yes	Yes	Yes	Yes	Yes	1	Yes
S107	.			Yes	Yes	Yes	Yes	Yes	Yes	1	Yes
S108	115	Hepatitis B	Hepatitis B	Yes	Yes	Yes	Yes	Yes	Yes	1	Yes
S109	.			Yes	Yes	Yes	Yes	Yes	Yes	1	Yes
S110	.			Yes	Yes	Yes	Yes	Yes	Yes	1	Yes
S111	.			Yes	Yes	Yes	Yes	Yes	Yes	1	Yes
S113	.			Yes	No	Yes	Yes	Yes	Yes	1	No
S115	.			Yes		Yes	Yes	Yes	Yes	1	No
S116	.			Yes	Yes	Yes	Yes	Yes	Yes	1	Yes
S117	.			Yes	Yes	Yes	Yes	Yes	Yes	1	Yes
S118	114	JAIVAC-1	Dosage A	Yes	Yes	Yes	Yes	Yes	Yes	1	Yes
S119	107	Hepatitis B	Hepatitis B	Yes	Yes	Yes	Yes	Yes	Yes	1	Yes
S120	104	JAIVAC-1	Dosage A	Yes	Yes	Yes	Yes	Yes	Yes	1	Yes
S123	.			Yes	Yes	Yes	Yes	Yes	Yes	1	Yes
S124	106	Hepatitis B	Hepatitis B	Yes	Yes	Yes	Yes	Yes	Yes	1	Yes
S125	102	JAIVAC-1	Dosage A	Yes	Yes	Yes	Yes	Yes	Yes	1	Yes
S126	.			Yes	Yes	Yes	Yes	Yes	Yes	1	Yes
S128	.			Yes	Yes	Yes	Yes	Yes	Yes	1	Yes
S129	.			Yes	Yes	Yes	Yes	Yes	Yes	1	Yes

Subject No.	Randomisation Number	Study Group	Dosage	No.1	No.2	No.3	No.4	No.5	No.6	* No.6a,b	All Inclusion Criteria Met?
S130	105	JAIVAC-1	Dosage A	Yes	Yes	Yes	Yes	Yes	Yes	1	Yes
S131	.			Yes	Yes	Yes	Yes	Yes	Yes	1	Yes
S132	108	Hepatitis B	Hepatitis B	Yes	Yes	Yes	Yes	Yes	Yes	1	Yes
S133	.			Yes	Yes	Yes	Yes	Yes	Yes	1	Yes
S134	.										No
S135	.			Yes	Yes	Yes	Yes	Yes	Yes	1	Yes
S136	109	JAIVAC-1	Dosage A	Yes	Yes	Yes	Yes	Yes	Yes	1	Yes
S137	.			Yes	Yes	Yes	Yes	Yes	Yes	1	Yes
S138	.			Yes	Yes	Yes	Yes	Yes	Yes	1	Yes
S139	.			Yes	Yes	Yes	Yes	Yes	Yes	1	Yes
S140	.			Yes	Yes	Yes	Yes	Yes	Yes	1	Yes
S141	.			Yes	Yes	Yes	Yes	Yes	Yes	1	Yes
S143	101	JAIVAC-1	Dosage A	Yes	Yes	Yes	Yes	Yes	Yes	1	Yes
S144	103	Hepatitis B	Hepatitis B	Yes	Yes	Yes	Yes	Yes	Yes	1	Yes
S145	112	JAIVAC-1	Dosage A	Yes	Yes	Yes	Yes	Yes	Yes	1	Yes
S146	.			Yes	Yes	Yes	Yes	Yes	Yes	2	Yes
S147	.			Yes	Yes	Yes	Yes	Yes	Yes	1	Yes
S148	111	JAIVAC-1	Dosage A	Yes	Yes	Yes	Yes	Yes	Yes	1	Yes
S150	.			Yes	Yes	Yes	Yes	Yes	Yes	1	Yes
S151	.			Yes	No	Yes	Yes				No
S152	.			Yes	Yes	Yes	Yes	Yes	Yes	1	Yes
S153	.			Yes	Yes	Yes	Yes	Yes	Yes	1	Yes
S154	113	JAIVAC-1	Dosage A	Yes	Yes	Yes	Yes	Yes	Yes	1	Yes
S155	.			Yes	Yes	Yes	Yes	Yes	Yes	1	Yes
S156	110	JAIVAC-1	Dosage A	Yes	Yes	Yes	Yes	Yes	Yes	1	Yes
S157	.			Yes	Yes	Yes	Yes	Yes	Yes	1	Yes
S158	.			Yes	Yes	Yes	Yes	Yes	Yes	1	Yes
S161	.			Yes	Yes	Yes	Yes	Yes	Yes	1	Yes
S166	.			Yes	Yes	Yes	Yes	Yes	Yes	1	Yes
S167	.			Yes	Yes	Yes	Yes	Yes	Yes	1	Yes

Subject No.	Randomisation Number	Study Group	Dosage	No.1	No.2	No.3	No.4	No.5	No.6	* No.6a,b	All Inclusion Criteria Met?
S169	.			Yes	No	Yes	Yes	Yes	Yes	1	No
S170	204	Hepatitis B	Hepatitis B	Yes	Yes	Yes	Yes	Yes	Yes	1	Yes
S171	.			Yes	Yes	Yes	Yes	Yes	Yes	1	Yes
S173	.			Yes	Yes	Yes	Yes	Yes	Yes	1	Yes
S174	.			Yes	Yes	Yes	Yes	Yes	Yes	1	Yes
S175	209	JAIVAC-1	Dosage B	Yes	Yes	Yes	Yes	Yes	Yes	1	Yes
S176	205	Hepatitis B	Hepatitis B	Yes	Yes	Yes	Yes	Yes	Yes	1	Yes
S177	.			Yes	No	Yes	Yes	Yes	Yes	1	No
S178	.			Yes	Yes	Yes	Yes	Yes	Yes	1	Yes
S179	207	Hepatitis B	Hepatitis B	Yes	Yes	Yes	Yes	Yes	Yes	1	Yes
S180	.			Yes	Yes	Yes	Yes	Yes	Yes	1	Yes
S181	.			Yes	Yes	Yes	Yes	Yes	Yes	1	Yes
S182	208	JAIVAC-1	Dosage B	Yes	Yes	Yes	Yes	Yes	Yes	1	Yes
S184	.			Yes	Yes	Yes	Yes	Yes	Yes	1	Yes
S185	202	JAIVAC-1	Dosage B	Yes	Yes	Yes	Yes	Yes	Yes	1	Yes
S186	201	JAIVAC-1	Dosage B	Yes	Yes	Yes	Yes	Yes	Yes	1	Yes
S187	.			Yes	No	Yes	Yes	Yes	Yes	1	No
S188	.			Yes	Yes	Yes	Yes	Yes	Yes	1	Yes
S190	.			Yes	Yes	Yes	Yes	Yes	Yes	2	Yes
S191	.			Yes	No	Yes	Yes	Yes	Yes	1	No
S192	203	JAIVAC-1	Dosage B	Yes	Yes	Yes	Yes	Yes	Yes	1	Yes
S193	215	JAIVAC-1	Dosage B	Yes	Yes	Yes	Yes	Yes	Yes	1	Yes
S195	214	JAIVAC-1	Dosage B	Yes	Yes	Yes	Yes	Yes	Yes	1	Yes
S196	.			Yes	Yes	Yes	Yes	Yes	Yes	1	Yes
S200	.			Yes	Yes	Yes	Yes	Yes	Yes	1	Yes
S202	206	JAIVAC-1	Dosage B	Yes	Yes	Yes	Yes	Yes	Yes	1	Yes
S203	211	Hepatitis B	Hepatitis B	Yes	Yes	Yes	Yes	Yes	Yes	1	Yes
S206	.			Yes	No	Yes	Yes	Yes	Yes	1	No
S207	.			Yes	Yes	Yes	Yes	Yes	Yes	1	Yes
S208	.			Yes	Yes	Yes	Yes	Yes	Yes	1	Yes

Subject No.	Randomisation Number	Study Group	Dosage	No.1	No.2	No.3	No.4	No.5	No.6	* No.6a,b	All Inclusion Criteria Met?
S210	.			Yes	Yes	Yes	Yes	Yes	Yes	1	Yes
S212	212	JAIVAC-1	Dosage B	Yes	Yes	Yes	Yes	Yes	Yes	1	Yes
S214	.			Yes	Yes	Yes	Yes	Yes	Yes	2	Yes
S215	213	Hepatitis B	Hepatitis B	Yes	Yes	Yes	Yes	Yes	Yes	1	Yes
S216	.			Yes	Yes	Yes	Yes	Yes	Yes	1	Yes
S218	.			Yes	Yes	Yes	Yes	Yes	Yes	1	Yes
S221	.			Yes	Yes	Yes	Yes	Yes	Yes	2	Yes
S226	.			Yes	Yes	Yes	Yes	Yes	Yes	1	Yes
S230	.			Yes	Yes	Yes	Yes	Yes	Yes	1	Yes
S233	210	JAIVAC-1	Dosage B	Yes	Yes	Yes	Yes	Yes	Yes	1	Yes
S234	.			Yes	Yes	Yes	Yes	Yes	Yes	1	Yes
S235	.			Yes	Yes	Yes	Yes	Yes	Yes	1	Yes
S236	.			Yes	Yes	Yes	Yes	Yes	Yes	1	Yes
S237	.			Yes	Yes	Yes	Yes	Yes	Yes	1	Yes
S239	.			Yes	Yes	Yes	Yes	Yes	Yes	1	Yes
S241	303	Hepatitis B	Hepatitis B	Yes	Yes	Yes	Yes	Yes	Yes	1	Yes
S244	.			Yes	Yes	Yes	Yes	Yes	Yes	1	Yes
S246	.			Yes	Yes	Yes	Yes	Yes	Yes	1	Yes
S247	.			Yes	Yes	Yes	Yes	Yes	Yes	1	Yes
S249	.			Yes	Yes	Yes	Yes	Yes	Yes	1	Yes
S250	311	JAIVAC-1	Dosage C	Yes	Yes	Yes	Yes	Yes	Yes	1	Yes
S251	.			Yes	Yes	Yes	Yes	Yes	Yes	1	Yes
S252	.			Yes	No	Yes	Yes	Yes	Yes	1	No
S254	.			Yes	Yes	Yes	Yes	Yes	Yes	1	Yes
S258	.			Yes	Yes	Yes	Yes	Yes	Yes	1	Yes
S260	.			Yes	Yes	Yes	Yes	Yes	Yes	1	Yes
S263	313	Hepatitis B	Hepatitis B	Yes	Yes	Yes	Yes	Yes	Yes	1	Yes
S264	306	JAIVAC-1	Dosage C	Yes	Yes	Yes	Yes	Yes	Yes	1	Yes
S266	308	JAIVAC-1	Dosage C	Yes	Yes	Yes	Yes	Yes	Yes	1	Yes
S267	309	Hepatitis B	Hepatitis B	Yes	Yes	Yes	Yes	Yes	Yes	1	Yes

Subject No.	Randomisation Number	Study Group	Dosage	No.1	No.2	No.3	No.4	No.5	No.6	* No.6a,b	All Inclusion Criteria Met?
S268	.			Yes	Yes	Yes	Yes	Yes	Yes	1	Yes
S272	.			Yes	Yes	Yes	Yes	Yes	Yes	1	Yes
S273	.										No
S274	.			Yes	Yes	Yes	Yes	Yes	Yes	1	Yes
S275	302	Hepatitis B	Hepatitis B	Yes	Yes	Yes	Yes	Yes	Yes	1	Yes
S276	.			Yes	Yes	Yes	Yes	Yes	Yes	1	Yes
S277	.			Yes	Yes	Yes	Yes	Yes	Yes	1	Yes
S278	315	JAIVAC-1	Dosage C	Yes	Yes	Yes	Yes	Yes	Yes	1	Yes
S279	301	JAIVAC-1	Dosage C	Yes	Yes	Yes	Yes	Yes	Yes	1	Yes
S280	.			Yes	Yes	Yes	Yes	Yes	Yes	1	Yes
S281	304	JAIVAC-1	Dosage C	Yes	Yes	Yes	Yes	Yes	Yes	1	Yes
S282	.			Yes	Yes	Yes	Yes	Yes	Yes	1	Yes
S284	305	JAIVAC-1	Dosage C	Yes	Yes	Yes	Yes	Yes	Yes	1	Yes
S285	307	Hepatitis B	Hepatitis B	Yes	Yes	Yes	Yes	Yes	Yes	1	Yes
S286	.			Yes	Yes	Yes	Yes	Yes	Yes	1	Yes
S287	.			Yes	No	Yes	Yes	Yes	Yes	1	No
S289	.			Yes	Yes	Yes	Yes	Yes	Yes	1	Yes
S290	.			Yes	Yes	Yes	Yes	Yes	Yes	1	Yes
S291	312	JAIVAC-1	Dosage C	Yes	Yes	Yes	Yes	Yes	Yes	1	Yes
S292	.			Yes	No	Yes	Yes	Yes	Yes	1	No
S294	.			Yes	Yes	Yes	Yes	Yes	Yes	1	Yes
S295	.			Yes	Yes	Yes	Yes	Yes	Yes	1	Yes
S296	.			Yes	Yes	Yes	Yes	Yes	Yes	1	Yes
S298	314	JAIVAC-1	Dosage C	Yes	Yes	Yes	Yes	Yes	Yes	1	Yes
S299	310	JAIVAC-1	Dosage C	Yes	Yes	Yes	Yes	Yes	Yes	1	Yes
S300	.			Yes	Yes	Yes	Yes	Yes	Yes	1	Yes
S303	.										No
S304	.			Yes	Yes	Yes	Yes	Yes	Yes	1	Yes
S305	.										No

Note:
1) If any of the Inclusion Criteria has a response No then the Subject is not eligible for the study
2) *1 - Double barrier method (e.g. condom with spermicidal jelly) used for the entire study period, 2 - Should be Surgically sterile (vasectomy)

16.4.5: Listing of Subject Exclusion Criteria (I of II)

Subject No.	Randomisation Number	Study Group	Dosage	No.1	No.2	No.3	No.4	No.5	No.6	No.7	No.8	No.9	No.10	No.11
S097	.			No	No	No	No	No	No	No	No	Yes	No	No
S098	.			No	No	No	No	No	No	No	No	Yes	No	No
S099	.			No	No	No	No	No	No	No	No	No	No	No
S100	.			No	No	No	No	No	No	No	No	Yes	No	No
S101	.			No	No	No	No	No	No	No	No	Yes	No	No
S102	.			No	No	No	No	No	No	No	No	Yes	No	No
S103	.			No	No	No	No	No	No	No	No	No	No	No
S104	.			No	No	No	No	No	No	No	No	No	Yes	Yes
S105	.			No	No	No	No	No	No	No	No	Yes	No	No
S106	.			No	No	No	No	No	No	No	No	No	No	No
S107	.			No	No	No	No	No	No	No	No	Yes	No	No
S108	115	Hepatitis B	Hepatitis B	No	No	No	No	No	No	No	No	No	No	No
S109	.			No	No	No	No	No	No	No	No			
S110	.			No	No	No	No	No	No	No	No	Yes	No	No
S111	.			No	No	No	No	No	No	No	No	Yes	No	No
S113	.			No	No	No	No	No	No	No	No			
S115	.			No	No	No	No	No	No	No	No			
S116	.			No	No	No	No	No	No	No	No	No	No	No
S117	.			No	No	No	No	No	No	No	No	No	No	No
S118	114	JAIVAC-1	Dosage A	No	No	No	No	No	No	No	No	No	No	No
S119	107	Hepatitis B	Hepatitis B	No	No	No	No	No	No	No	No	No	No	No
S120	104	JAIVAC-1	Dosage A	No	No	No	No	No	No	No	No	No	No	No
S123	.			No	No	No	No	No	No	No	No	No	No	No
S124	106	Hepatitis B	Hepatitis B	No	No	No	No	No	No	No	No	No	No	No
S125	102	JAIVAC-1	Dosage A	No	No	No	No	No	No	No	No	No	No	No
S126	.			No	No	No	No	No	No	No	No	No	Yes	Yes
S128	.			No	No	No	No	No	No	No	No	Yes	No	Yes
S129	.			No	No	No	No	No	No	No	No	No	Yes	No

Subject No.	Randomisation Number	Study Group	Dosage	No.1	No.2	No.3	No.4	No.5	No.6	No.7	No.8	No.9	No.10	No.11
S130	105	JAIVAC-1	Dosage A	No	No	No	No	No	No	No	No	No	No	No
S131	.			No	No	No	No	No	No	No	No			
S132	108	Hepatitis B	Hepatitis B	No	No	No	No	No	No	No	No	No	No	No
S133	.			No	No	No	No	No	No	No	No	No	No	No
S134	.													
S135	.			No	No	No	No	No	No	No	No	Yes	No	Yes
S136	109	JAIVAC-1	Dosage A	No	No	No	No	No	No	No	No	No	No	No
S137	.			No	No	No	No	No	No	No	No	No	Yes	No
S138	.			No	No	No	No	No	No	No	No	No	No	No
S139	.			No	No	No	No	No	No	No	No	No	Yes	Yes
S140	.			No	No	No	No	No	No	No	No	No	No	No
S141	.			No	No	No	No	No	No	No	No	No	No	No
S143	101	JAIVAC-1	Dosage A	No	No	No	No	No	No	No	No	No	No	No
S144	103	Hepatitis B	Hepatitis B	No	No	No	No	No	No	No	No	No	No	No
S145	112	JAIVAC-1	Dosage A	No	No	No	No	No	No	No	No	No	No	No
S146	.			No	No	No	No	No	No	No	No	Yes	Yes	Yes
S147	.			No	No	No	No	No	No	No	No	No	No	No
S148	111	JAIVAC-1	Dosage A	No	No	No	No	No	No	No	No	No	No	No
S150	.			No	No	No	No	No	No	No	No	No	No	No
S151	.			No	No	No		No						
S152	.			No	No	No	No	No	No	No	No	Yes	No	No
S153	.			No	No	No	No	No	No	No	No	No	Yes	No
S154	113	JAIVAC-1	Dosage A	No	No	No	No	No	No	No	No	No	No	No
S155	.			No	No	No	No	No	No	No	No	No	No	Yes
S156	110	JAIVAC-1	Dosage A	No	No	No	No	No	No	No	No	No	No	No
S157	.			No	No	No	No	No	No	No	No	No	No	No
S158	.			No	No	No	No	No	No	No	No	No	Yes	No
S161	.			No	No	No	No	No	No	No	No	No	No	No
S166	.			No	No	No	No	No	No	No	No	No	Yes	No
S167	.			No	No	No	No	No	No	No	No	No	Yes	No

Subject No.	Randomisation Number	Study Group	Dosage	No.1	No.2	No.3	No.4	No.5	No.6	No.7	No.8	No.9	No.10	No.11
S169	.			No	No	No	No	No	No	No	No			
S170	204	Hepatitis B	Hepatitis B	No	No	No	No	No	No	No	No	No	No	No
S171	.			No	No	No	No	No	No	No	No	No	No	No
S173	.			No	No	No	No	No	No	No	No	No	No	No
S174	.			No	No	No	No	No	No	No	No	Yes	No	No
S175	209	JAIVAC-1	Dosage B	No	No	No	No	No	No	No	No	No	No	No
S176	□ ▪ ▪ ▪ ▪ epatitis B	Hepatitis B	Hepatitis B	No	No	No	No	No	No	No	No	No	No	No
S177	.			No	No	No	No	No	No	No	No	No	No	No
S178	.			No	No	No	No	No	No	No	No	No	Yes	Yes
S179	207	Hepatitis B	Hepatitis B	No	No	No	No	No	No	No	No	No	No	No
S180	.			No	No	No	No	No	No	No	No	No	Yes	Yes
S181	.			No	No	No	No	No	No	No	No	Yes	No	No
S182	208	JAIVAC-1	Dosage B	No	No	No	No	No	No	No	No	No	No	No
S184	.			No	No	No	No	No	No	No	No			
S185	202	JAIVAC-1	Dosage B	No	No	No	No	No	No	No	No	No	No	No
S186	201	JAIVAC-1	Dosage B	No	No	No	No	No	No	No	No	No	No	No
S187	.			No	No	No	No	No	No	No	No			
S188	.			No	No	No	No	No	No	No	No	No	No	No
S190	.			No	No	No	No	No	No	No	No	No	Yes	No
S191	.			No	No	No	No	No	No	No	No	No	No	No
S192	203	JAIVAC-1	Dosage B	No	No	No	No	No	No	No	No	No	No	No
S193	215	JAIVAC-1	Dosage B	No	No	No	No	No	No	No	No	No	No	No
S195	214	JAIVAC-1	Dosage B	No	No	No	No	No	No	No	No	No	No	No
S196	.			No	No	No	No	No	No	No	No	No	Yes	No
S200	.			No	No	No	No	No	No	No	No	No	No	No
S202	206	JAIVAC-1	Dosage B	No	No	No	No	No	No	No	No	No	No	No
S203	211	Hepatitis B	Hepatitis B	No	No	No	No	No	No	No	No	No	No	No
S206	.			No	No	No	No	No	No	No	No			
S207	.			No	No	No	No	No	No	No	No	No	No	No
S208	.			No	No	No	No	No	No	No	No	No	Yes	No

Subject No.	Randomisation Number	Study Group	Dosage	No.1	No.2	No.3	No.4	No.5	No.6	No.7	No.8	No.9	No.10	No.11
S210	.			No	No	No	No	No	No	No	No	Yes	No	No
S212	212	JAIVAC-1	Dosage B	No	No	No	No	No	No	No	No	No	No	No
S214	.			No	No	No	No	No	No	No	No	Yes	No	No
S215	213	Hepatitis B	Hepatitis B	No	No	No	No	No	No	No	No	No	No	No
S216	.			No	No	No	No	No	No	No	No	No	Yes	No
S218	.			No	No	No	No	No	No	No	No	No	No	No
S221	.			No	No	No	No	No	No	No	No	No	No	No
S226	.			No	No	No	No	No	No	No	No	No	Yes	No
S230	.			No	No	No	No	No	No	No	No	No	No	No
S233	210	JAIVAC-1	Dosage B	No	No	No	No	No	No	No	No	No	No	No
S234	.			No	No	No	No	No	No	No	No			
S235	.			No	No	No	No	No	No	No	No	No	Yes	No
S236	.			No	No	No	No	No	No	No	No	Yes	No	No
S237	.			No	No	No	No	No	No	No	No	Yes	No	No
S239	.			No	No	No	No	No	No	No	No	Yes	No	No
S241	303	Hepatitis B	Hepatitis B	No	No	No	No	No	No	No	No	No	No	No
S244	.			No	No	No	No	No	No	No	No	No	Yes	No
S246	.			No	No	No	No	No	No	No	No	No	No	Yes
S247	.			No	No	No	No	No	No	No	No	No	Yes	No
S249	.			No	No	No	No	No	No	No	No	Yes	No	No
S250	311	JAIVAC-1	Dosage C	No	No	No	No	No	No	No	No	No	No	No
S251	.			No	No	No	No	No	No	No	No	Yes	Yes	No
S252	.			No	No	No	No	No	No	No	No			
S254	.			No	No	No	No	No	No	No	No	No	Yes	Yes
S258	.			No	No	No	No	No	No	No	No	No	No	No
S260	.			No	No	No	No	No	No	No	No	No	No	No
S263	313	Hepatitis B	Hepatitis B	No	No	No	No	No	No	No	No	No	No	No
S264	306	JAIVAC-1	Dosage C	No	No	No	No	No	No	No	No	No	No	No
S266	308	JAIVAC-1	Dosage C	No	No	No	No	No	No	No	No	No	No	No
S267	309	Hepatitis B	Hepatitis B	No	No	No	No	No	No	No	No	No	No	No

Subject No.	Randomisation Number	Study Group	Dosage	No.1	No.2	No.3	No.4	No.5	No.6	No.7	No.8	No.9	No.10	No.11
S268	.			No	No	No	No	No	No	No	No	Yes	No	No
S272	.			No	No	No	No	No	No	No	No	No	No	No
S273	.													
S274	.			No	No	No	No	No	No	No	No	No	No	No
S275	302	Hepatitis B	Hepatitis B	No	No	No	No	No	No	No	No	No	No	No
S276	.			No	No	No	No	No	No	No	No	No	Yes	No
S277	.			No	No	No	No	No	No	No	No	No	No	No
S278	315	JAIVAC-1	Dosage C	No	No	No	No	No	No	No	No	No	No	No
S279	301	JAIVAC-1	Dosage C	No	No	No	No	No	No	No	No	No	No	No
S280	.			No	No	No	No	No	No	No	No	No	Yes	Yes
S281	304	JAIVAC-1	Dosage C	No	No	No	No	No	No	No	No	No	No	No
S282	.			No	No	No	No	No	No	No	No	Yes	No	No
S284	305	JAIVAC-1	Dosage C	No	No	No	No	No	No	No	No	No	No	No
S285	307	Hepatitis B	Hepatitis B	No	No	No	No	No	No	No	No	No	No	No
S286	.			No	No	No	No	No	No	No	No	No	Yes	Yes
S287	.			No	No	No	No	No	No	No	No			
S289	.			No	No	No	No	No	No	No	No	Yes	Yes	No
S290	.			No	No	No	No	No	No	No	No	Yes	No	No
S291	312	JAIVAC-1	Dosage C	No	No	No	No	No	No	No	No	No	No	No
S292	.			No	No	No	No	No	No	No	No			
S294	.			No	No	No	No	No	No	No	No	Yes	No	Yes
S295	.			No	No	No	No	No	No	No	No	No	Yes	No
S296	.			No	No	No	No	No	No	No	No	No	Yes	No
S298	314	JAIVAC-1	Dosage C	No	No	No	No	No	No	No	No	No	No	No
S299	310	JAIVAC-1	Dosage C	No	No	No	No	No	No	No	No	No	No	No
S300	.			No	No	No	No	No	No	No	No	No	Yes	No
S303	.													
S304	.			No	No	No	No	No	No	No	No	No	No	No
S305	.													

Note:
1) If any of the Exclusion Criteria has a response Yes then the Subject is not eligible for the study

16.4.5: Listing of Subject Exclusion Criteria (II of II)

Subject No.	Randomisation Number	Study Group	Dosage	No.12	No.13	No.14	No.15	No.16	No.17	No.18	No.19	No.20	No.21	No.22	All Exclusion Criteria Not Met?
S097	.			No	No	No	No	No	No	No	No	No	No	No	No
S098	.			No	No	No	No	No	No	No	No	No	No	No	No
S099	.			No	No	No	No	No	No	No	No	No	No	No	Yes
S100	.			No	No	No	No	No	No	No	No	No	No	No	No
S101	.			No	No	No	No	No	No	No	No	No	No	No	No
S102	.			No	No	No	No	No	No	No	No	No	No	No	No
S103	.			No	No	No	No	No	No	No	No	No	No	No	Yes
S104	.			No	No	No	No	No	No	No	No	No	No	No	No
S105	.			No	No	No	No	No	No	No	No	No	No	No	No
S106	.			No	No	No	No	No	No	No	No	No	No	No	Yes
S107	.			No	No	No	No	No	No	No	No	No	No	No	No
S108	115	Hepatitis B	Hepatitis B	No	No	No	No	No	No	No	No	No	No	No	Yes
S109	.						No	No		Yes		No	No	No	No
S110	.			No	No	No	No	No	No	No	No	No	No	No	No
S111	.			No	No	No	No	No	No	No	No	No	No	No	No
S113	.						No	No				No	No	No	No
S115	.						No	No				No	No	No	No
S116	.			No	No	No	No	No	No	No	No	No	No	No	Yes
S117	.			No	No	No	No	No	No	No	No	No	No	No	Yes
S118	114	JAIVAC-1	Dosage A	No	No	No	No	No	No	No	No	No	No	No	Yes
S119	107	Hepatitis B	Hepatitis B	No	No	No	No	No	No	No	No	No	No	No	Yes
S120	104	JAIVAC-1	Dosage A	No	No	No	No	No	No	No	No	No	No	No	Yes
S123	.			No	No	No	No	No	No	No	No	No	No	No	Yes
S124	106	Hepatitis B	Hepatitis B	No	No	No	No	No	No	No	No	No	No	No	Yes
S125	102	JAIVAC-1	Dosage A	No	No	No	No	No	No	No	No	No	No	No	Yes
S126	.			No	No	No	No	No	No	No	No	No	No	No	No
S128	.			No	No	No	No	No	No	No	No	No	No	No	No

Subject No.	Randomisation Number	Study Group	Dosage	No.12	No.13	No.14	No.15	No.16	No.17	No.18	No.19	No.20	No.21	No.22	All Exclusion Criteria Not Met?
S129	.			No	No	No	No	No	No	No	No	No	No	No	No
S130	105	JAIVAC-1	Dosage A	No	No	No	No	No	No	No	No	No	No	No	Yes
S131	.						No	No		Yes		No	No	No	No
S132	108	Hepatitis B	Hepatitis B	No	No	No	No	No	No	No	No	No	No	No	Yes
S133	.			No	No	No	No	No	No	No	No	No	No	No	Yes
S134	.														No
S135	.			No	No	No	No	No	No	No	No	No	No	No	No
S136	109	JAIVAC-1	Dosage A	No	No	No	No	No	No	No	No	No	No	No	Yes
S137	.			No	No	No	No	No	No	No	No	No	No	No	No
S138	.			No	No	No	No	No	No	No	No	No	No	No	Yes
S139	.			No	No	No	No	No	No	No	No	No	No	No	No
S140	.			No	No	No	No	No	No	No	No	No	No	No	Yes
S141	.			No	No	No	No	No	No	No	No	No	No	No	Yes
S143	101	JAIVAC-1	Dosage A	No	No	No	No	No	No	No	No	No	No	No	Yes
S144	103	Hepatitis B	Hepatitis B	No	No	No	No	No	No	No	No	No	No	No	Yes
S145	112	JAIVAC-1	Dosage A	No	No	No	No	No	No	No	No	No	No	No	Yes
S146	.			Yes	No	No	No	No	No	No	No	No	No	No	No
S147	.			No	No	No	No	No	No	No	No	No	No	No	Yes
S148	111	JAIVAC-1	Dosage A	No	No	No	No	No	No	No	No	No	No	No	Yes
S150	.			No	No	No	No	No	No	No	No	No	No	No	Yes
S151	.						No	No							No
S152	.			No	No	No	No	No	No	No	No	No	No	No	No
S153	.			No	No	No	No	No	No	No	No	No	No	No	No
S154	113	JAIVAC-1	Dosage A	No	No	No	No	No	No	No	No	No	No	No	Yes
S155	.			No	No	No	No	No	No	No	No	No	No	No	No
S156	110	JAIVAC-1	Dosage A	No	No	No	No	No	No	No	No	No	No	No	Yes
S157	.			No	No	No	No	No	No	No	No	No	No	No	Yes
S158	.			No	No	No	No	No	No	No	No	No	No	No	No
S161	.			No	No	No	No	No	No	No	No	No	No	No	Yes

Subject No.	Randomisation Number	Study Group	Dosage	No.12	No.13	No.14	No.15	No.16	No.17	No.18	No.19	No.20	No.21	No.22	All Exclusion Criteria Not Met?
S166	.			No	No	No	No	No	No	No	No	No	No	No	No
S167	.			No	No	No	No	No	No	No	No	No	No	No	No
S169	.						No	No				No	No	No	No
S170	204	Hepatitis B	Hepatitis B	No	No	No	No	No	No	No	No	No	No	No	Yes
S171	.			No	No	No	No	No	No	No	No	No	No	No	Yes
S173	.			Yes	No	No	No	No	No	No	No	No	No	No	No
S174	.			No	No	No	No	No	No	No	No	No	No	No	No
S175	209	JAIVAC-1	Dosage B	No	No	No	No	No	No	No	No	No	No	No	Yes
S176	205	Hepatitis B	Hepatitis B	No	No	No	No	No	No	No	No	No	No	No	Yes
S177	.			No	No	No	No	No	No	No	No	No	No	No	Yes
S178	.			No	No	No	No	No	No	No	No	No	No	No	No
S179	207	Hepatitis B	Hepatitis B	No	No	No	No	No	No	No	No	No	No	No	Yes
S180	.			No	No	No	No	No	No	No	No	No	No	No	No
S181	.			No	No	No	No	No	No	No	No	No	No	No	No
S182	208	JAIVAC-1	Dosage B	No	No	No	No	No	No	No	No	No	No	No	Yes
S184	.			No	No	No	No	No	No	No	No	No	No	No	No
S185	202	JAIVAC-1	Dosage B	No	No	No	No	No	No	No	No	No	No	No	Yes
S186	201	JAIVAC-1	Dosage B	No	No	No	No	No	No	No	No	No	No	No	Yes
S187	.						No	No				No	No	No	No
S188	.			No	No	No	No	No	No	No	No	No	No	No	Yes
S190	.			No	No	No	No	No	No	No	No	No	No	No	No
S191	.			No	No	No	No	No	No	No	No	No	No	No	Yes
S192	203	JAIVAC-1	Dosage B	No	No	No	No	No	No	No	No	No	No	No	Yes
S193	215	JAIVAC-1	Dosage B	No	No	No	No	No	No	No	No	No	No	No	Yes
S195	214	JAIVAC-1	Dosage B	No	No	No	No	No	No	No	No	No	No	No	Yes
S196	.			No	No	No	No	No	No	No	No	No	No	No	No
S200	.			No	No	No	No	No	No	No	No	No	No	No	Yes
S202	206	JAIVAC-1	Dosage B	No	No	No	No	No	No	No	No	No	No	No	Yes
S203	211	Hepatitis B	Hepatitis B	No	No	No	No	No	No	No	No	No	No	No	Yes

Subject No.	Randomisation Number	Study Group	Dosage	No.12	No.13	No.14	No.15	No.16	No.17	No.18	No.19	No.20	No.21	No.22	All Exclusion Criteria Not Met?
S206	.						No	No				No	No	No	No
S207	.			No	No	No	No	No	No	No	No	No	No	No	Yes
S208	.			No	No	No	No	No	No	No	No	No	No	No	No
S210	.			No	No	No	No	No	No	No	No	No	No	No	No
S212	212	JAIVAC-1	Dosage B	No	No	No	No	No	No	No	No	No	No	No	Yes
S214	.			No	No	No	No	No	No	No	No	No	No	No	No
S215	213	Hepatitis B	Hepatitis B	No	No	No	No	No	No	No	No	No	No	No	Yes
S216	.			No	No	No	No	No	No	No	No	No	No	No	No
S218	.			No	No	No	No	No	No	No	No	No	No	No	Yes
S221	.			No	No	No	No	No	No	No	No	No	No	No	Yes
S226	.			No	No	No	No	No	No	No	No	No	No	No	No
S230	.			No	No	No	No	No	No	No	No	No	No	No	Yes
S233	210	JAIVAC-1	Dosage B	No	No	No	No	No	No	No	No	No	No	No	Yes
S234	.						No	No		Yes		No	No	No	No
S235	.			No	No	No	No	No	No	No	No	No	No	No	No
S236	.			No	No	No	No	No	No	No	No	No	No	No	No
S237	.			No	No	No	No	No	No	No	No	No	No	No	No
S239	.			No	No	No	No	No	No	No	No	No	No	No	No
S241	303	Hepatitis B	Hepatitis B	No	No	No	No	No	No	No	No	No	No	No	Yes
S244	.			No	No	No	No	No	No	No	No	No	No	No	No
S246	.			No	No	No	No	No	No	No	No	No	No	No	No
S247	.			No	No	No	No	No	No	No	No	No	No	No	No
S249	.			No	No	No	No	No	No	No	No	No	No	No	No
S250	311	JAIVAC-1	Dosage C	No	No	No	No	No	No	No	No	No	No	No	Yes
S251	.			No	No	No	No	No	No	No	No	No	No	No	No
S252	.						No	No				No	No	No	No
S254	.			No	No	No	No	No	No	No	Yes	No	No	No	No
S258	.			No	No	No	No	No	No	No	No	No	No	No	Yes
S260	.			No	No	No	No	No	No	No	No	No	No	No	Yes

Subject No.	Randomisation Number	Study Group	Dosage	No.12	No.13	No.14	No.15	No.16	No.17	No.18	No.19	No.20	No.21	No.22	All Exclusion Criteria Not Met?
S263	313	Hepatitis B	Hepatitis B	No	No	No	No	No	No	No	No	No	No	No	Yes
S264	306	JAIVAC-1	Dosage C	No	No	No	No	No	No	No	No	No	No	No	Yes
S266	308	JAIVAC-1	Dosage C	No	No	No	No	No	No	No	No	No	No	No	Yes
S267	309	Hepatitis B	Hepatitis B	No	No	No	No	No	No	No	No	No	No	No	Yes
S268	.			No	No	No	No	No	No	No	No	No	No	No	No
S272	.			No	No	No	No	No	No	No	No	No	No	No	Yes
S273	.														No
S274	.			No	No	No	No	No	No	No	No	No	No	No	Yes
S275	302	Hepatitis B	Hepatitis B	No	No	No	No	No	No	No	No	No	No	No	Yes
S276	.			No	No	No	No	No	No	No	No	No	No	No	No
S277	.			No	No	No	No	No	No	No	No	No	No	No	Yes
S278	315	JAIVAC-1	Dosage C	No	No	No	No	No	No	No	No	No	No	No	Yes
S279	301	JAIVAC-1	Dosage C	No	No	No	No	No	No	No	No	No	No	No	Yes
S280	.			No	No	No	No	No	No	No	No	No	No	No	No
S281	304	JAIVAC-1	Dosage C	No	No	No	No	No	No	No	No	No	No	No	Yes
S282	.			No	No	No	No	No	No	No	No	No	No	No	No
S284	305	JAIVAC-1	Dosage C	No	No	No	No	No	No	No	No	No	No	No	Yes
S285	307	Hepatitis B	Hepatitis B	No	No	No	No	No	No	No	No	No	No	No	Yes
S286	.			No	No	No	No	No	No	No	No	No	No	No	No
S287	.						No	No				No	No	No	No
S289	.			No	No	No	No	No	No	No	No	No	No	No	No
S290	.			No	No	No	No	No	No	No	No	No	No	No	No
S291	312	JAIVAC-1	Dosage C	No	No	No	No	No	No	No	No	No	No	No	Yes
S292	.						No	No				No	No	No	No
S294	.			No	No	No	No	No	No	No	No	No	No	No	No
S295	.			No	No	No	No	No	No	No	No	No	No	No	No
S296	.			No	No	No	No	No	No	No	No	No	No	No	No
S298	314	JAIVAC-1	Dosage C	No	No	No	No	No	No	No	No	No	No	No	Yes
S299	310	JAIVAC-1	Dosage C	No	No	No	No	No	No	No	No	No	No	No	Yes

Subject No.	Randomisation Number	Study Group	Dosage	No.12	No.13	No.14	No.15	No.16	No.17	No.18	No.19	No.20	No.21	No.22	All Exclusion Criteria Not Met?
S300	.			No	No	No	No	No	No	No	No	No	No	No	No
S303	.								Yes						No
S304	.			No	No	No	No	No	No	No	No	No	No	No	Yes
S305	.								Yes						No

Note:
1) If any of the Exclusion Criteria has a response Yes then the Subject is not eligible for the study

16.4.6: Listing of Past Medical and Surgical History

Subject No.	Randomisation Number	Study Group	Dosage	Diagnosis	Primary System Organ Class	Preferred Term	Low Level Term	Start Date	Stop Date	Any Medication?
**	.	.	.	.	.	.	.	.	.	.

Note:
1) Medical History Conditions are those events which occurred during past but are absent at the time of first dose of vaccine administration Day 0
2) ** No event is recorded

16.4.7: Listing of Pre-existing (Current Medical) Conditions

Subject No.	Randomisation Number	Study Group	Dosage	Diagnosis	Primary System Organ Class	Preferred Term	Low Level Term	Start Date	Start Date Unknown	Stop Date	Ongoing	Any Medication?
S120	104	JAIVAC-1	Dosage A	Pityriasis Versicolor	Infections And Infestations	Tinea Versicolour	Pityriasis Versicolor	.	UK/UK/UKUK	.	Yes	No
S125	102	JAIVAC-1	Dosage A	Raised Blood Sugar	Investigations	Blood Glucose Increased	Blood Sugar Increased	21/02/2011		07/03/2011	No	No
S215	213	Hepatitis B	Hepatitis B	Raised Total, Direct And Indirect Bilirubin	Investigations	Blood Bilirubin Increased	Raised Bilirubin	29/03/2011		16/06/2011	No	No
S233	210	JAIVAC-1	Dosage B	Mycrocytic Hypochromic Anaemia	Blood And Lymphatic System Disorders	Hypochromic Anaemia	Hypochromic Microcytic Anemia	29/03/2011		27/04/2011	No	Yes
S279	301	JAIVAC-1	Dosage C	Raised Blood Sugar	Investigations	Blood Glucose Increased	Blood Sugar Increased	09/05/2011		16/05/2011	No	No
S298	314	JAIVAC-1	Dosage C	Multiple Benign Lipomas On Both Arms	Neoplasms Benign, Malignant And Unspecified (Incl Cysts And Polyps)	Lipoma	Lipoma	.	UK/UK/2003	.	Yes	No
S298	314	JAIVAC-1	Dosage C	Raised Potassium Levels	Investigations	Blood Potassium Increased	Potassium High	11/05/2011		30/05/2011	No	No

Note:
1) Pre-Existing Conditions are those events which are ongoing at the time of first dose of vaccine administration Day 0.

16.4.8: Listing of Pregnancy Reporting Conditions (I of III)

						Female Partner Details			Relevant History And SOC ial Habits Of Female Partner			
Subject No.	Randomisation Number	Study Group	Dosage	Type of Report	Date of Report	Date of Birth	Height (cm)	Weight (Kg)	Birth Control Method (s)	Appropriate usage of method	Occupational History	Medical History
**	.	.	.	.	.	.	.	.	.	.	.	.

Note:
1) ** No pregnancy is reported.

16.4.8: Listing of Pregnancy Reporting Conditions (II of III)

				Relevant History And Social Habits Of Female Partner				
Subject No.	Randomisation Number	Study Group	Dosage	Family History	Social Habits	Pregnancy History	Did a Birth defect in any pervious pregnancy?	Did a stillbirth or miscarriage occur in any previous pregnancy?
**	.	.	.	.	.	.	.	.

Note:

1) ** No pregnancy is reported

16.4.8: Listing of Pregnancy Reporting Conditions (III of III)

				Pregnancy Information				
Subject No.	Randomisation Number	Study Group	Dosage	Date of Last Menstrual Period	Date of Pregnancy Test	Expected Date of Delivery	Status of pregnancy	Medical problems during current pregnancy
**	.	.	.	.	.	.	.	.

Note:
1) ** No pregnancy is reported

16.4.9: Listing of ECG Results

Subject No.	Randomisation Number	Study Group	Dosage	Not Done	Specify	Date of Assessment	Result	Abnormal Specify	Clinical Significance
S108	115	Hepatitis B	Hepatitis B			18/08/2010	Normal		
S118	114	JAIVAC-1	Dosage A			17/08/2010	Normal		
S119	107	Hepatitis B	Hepatitis B			18/08/2010	Normal		
S120	104	JAIVAC-1	Dosage A			18/08/2010	Normal		
S124	106	Hepatitis B	Hepatitis B			18/08/2010	Normal		
S125	102	JAIVAC-1	Dosage A			18/08/2010	Normal		
S130	105	JAIVAC-1	Dosage A			17/08/2010	Normal		
S132	108	Hepatitis B	Hepatitis B			18/08/2010	Normal		
S136	109	JAIVAC-1	Dosage A			19/08/2010	Normal		
S143	101	JAIVAC-1	Dosage A			18/08/2010	Normal		
S144	103	Hepatitis B	Hepatitis B			19/08/2010	Normal		
S145	112	JAIVAC-1	Dosage A			19/08/2010	Abnormal	Minimum Right Axis	Not Clinically Significant (NCS)
S148	111	JAIVAC-1	Dosage A			20/08/2010	Normal		
S154	113	JAIVAC-1	Dosage A			19/08/2010	Normal		
S156	110	JAIVAC-1	Dosage A			19/08/2010	Normal		
S170	204	Hepatitis B	Hepatitis B			21/09/2010	Normal		
S175	209	JAIVAC-1	Dosage B			21/09/2010	Normal		
S176	205	Hepatitis B	Hepatitis B			22/09/2010	Normal		
S179	207	Hepatitis B	Hepatitis B			22/09/2010	Normal		
S182	208	JAIVAC-1	Dosage B			21/09/2010	Normal		
S185	202	JAIVAC-1	Dosage B			21/09/2010	Normal		
S186	201	JAIVAC-1	Dosage B			21/09/2010	Normal		
S192	203	JAIVAC-1	Dosage B			23/09/2010	Normal		
S193	215	JAIVAC-1	Dosage B			24/09/2010	Normal		
S195	214	JAIVAC-1	Dosage B			23/09/2010	Normal		
S202	206	JAIVAC-1	Dosage B			23/09/2010	Normal		
S203	211	Hepatitis B	Hepatitis B			24/09/2010	Normal		
S212	212	JAIVAC-1	Dosage B			23/09/2010	Normal		

Subject No.	Randomisation Number	Study Group	Dosage	Not Done	Specify	Date of Assessment	Result	Abnormal Specify	Clinical Significance
S215	213	Hepatitis B	Hepatitis B			24/09/2010	Normal		
S233	210	JAIVAC-1	Dosage B			24/09/2010	Normal		
S241	303	Hepatitis B	Hepatitis B			03/11/2010	Normal		
S250	311	JAIVAC-1	Dosage C			03/11/2010	Normal		
S263	313	Hepatitis B	Hepatitis B			09/11/2010	Normal		
S264	306	JAIVAC-1	Dosage C			03/11/2010	Normal		
S266	308	JAIVAC-1	Dosage C			03/11/2010	Normal		
S267	309	Hepatitis B	Hepatitis B			02/11/2010	Normal		
S275	302	Hepatitis B	Hepatitis B			02/11/2010	Normal		
S278	315	JAIVAC-1	Dosage C			08/11/2010	Normal		
S279	301	JAIVAC-1	Dosage C			03/11/2010	Normal		
S281	304	JAIVAC-1	Dosage C			03/11/2010	Normal		
S284	305	JAIVAC-1	Dosage C			03/11/2010	Normal		
S285	307	Hepatitis B	Hepatitis B			02/11/2010	Normal		
S291	312	JAIVAC-1	Dosage C			02/11/2010	Normal		
S298	314	JAIVAC-1	Dosage C			09/11/2010	Normal		
S299	310	JAIVAC-1	Dosage C			08/11/2010	Normal		

Note:
1) Clinical Significance: CS-Clinically Significant, NCS-Not Clinically Significant

16.4.10: Listing of Chest X-Ray – Post Anterior View

Subject No.	Study	Study Group	Dosage	Not Done	Specify	Date of Assessment	Result	Abnormal Specify	Clinical Significance
S108	115	Hepatitis B	Hepatitis B			09/04/2010	Normal		
S118	114	JAIVAC-1	Dosage A			07/06/2010	Normal		
S119	107	Hepatitis B	Hepatitis B			18/08/2010	Normal		
S120	104	JAIVAC-1	Dosage A			18/08/2010	Normal		
S124	106	Hepatitis B	Hepatitis B			18/08/2010	Normal		
S125	102	JAIVAC-1	Dosage A			18/08/2010	Normal		
S130	105	JAIVAC-1	Dosage A			17/08/2010	Normal		
S132	108	Hepatitis B	Hepatitis B			18/08/2010	Normal		
S136	109	JAIVAC-1	Dosage A			19/08/2010	Normal		
S143	101	JAIVAC-1	Dosage A			18/08/2010	Normal		
S144	103	Hepatitis B	Hepatitis B			19/08/2010	Normal		
S145	112	JAIVAC-1	Dosage A			19/08/2010	Normal		
S148	111	JAIVAC-1	Dosage A			20/08/2010	Normal		
S154	113	JAIVAC-1	Dosage A			08/04/2010	Normal		
S156	110	JAIVAC-1	Dosage A			05/04/2010	Normal		
S170	204	Hepatitis B	Hepatitis B			19/08/2010	Normal		
S175	209	JAIVAC-1	Dosage B			24/07/2010	Normal		
S176	205	Hepatitis B	Hepatitis B			19/08/2010	Normal		
S179	207	Hepatitis B	Hepatitis B			19/08/2010	Normal		
S182	208	JAIVAC-1	Dosage B			22/07/2010	Normal		
S185	202	JAIVAC-1	Dosage B			19/08/2010	Normal		
S186	201	JAIVAC-1	Dosage B			24/07/2010	Normal		
S192	203	JAIVAC-1	Dosage B			23/09/2010	Normal		
S193	215	JAIVAC-1	Dosage B			14/05/2010	Normal		
S195	214	JAIVAC-1	Dosage B			23/09/2010	Normal		
S202	206	JAIVAC-1	Dosage B			03/06/2010	Normal		
S203	211	Hepatitis B	Hepatitis B			24/09/2010	Normal		
S212	212	JAIVAC-1	Dosage B			23/09/2010	Normal		

Subject No.	Randomisation Number	Study Group	Dosage	Not Done	Specify	Date of Assessment	Result	Abnormal Specify	Clinical Significance
S215	213	Hepatitis B	Hepatitis B			24/09/2010	Normal		
S233	210	JAIVAC-1	Dosage B			24/09/2010	Normal		
S241	303	Hepatitis B	Hepatitis B			21/10/2010	Normal		
S250	311	JAIVAC-1	Dosage C			03/06/2010	Normal		
S263	313	Hepatitis B	Hepatitis B			22/05/2010	Normal		
S264	306	JAIVAC-1	Dosage C			14/10/2010	Normal		
S266	308	JAIVAC-1	Dosage C			21/10/2010	Normal		
S267	309	Hepatitis B	Hepatitis B			30/06/2010	Normal		
S275	302	Hepatitis B	Hepatitis B			02/11/2010	Normal		
S278	315	JAIVAC-1	Dosage C			22/09/2010	Normal		
S279	301	JAIVAC-1	Dosage C			03/11/2010	Normal		
S281	304	JAIVAC-1	Dosage C			07/06/2010	Normal		
S284	305	JAIVAC-1	Dosage C			03/11/2010	Normal		
S285	307	Hepatitis B	Hepatitis B			02/11/2010	Normal		
S291	312	JAIVAC-1	Dosage C			02/11/2010	Normal		
S298	314	JAIVAC-1	Dosage C			09/11/2010	Normal		
S299	310	JAIVAC-1	Dosage C			04/06/2010	Normal		

Note:
1) Clinical Significance: CS-Clinically Significant, NCS-Not Clinically Significant

16.4.11: Listing of Physical Examination Results

Subject No.	Randomisation Number	Study Group	Dosage	Dose	Day	Not Done	Specify	Date of Assessment	*Was the assessment performed prior to vaccination?	Physical Examination Parameter	Result	Abnormality	Clinical Significance
S108	115	Hepatitis B	Hepatitis B		Day -14 to Day -1			18/08/2010		Head and neck	Normal		
S108	115	Hepatitis B	Hepatitis B		Day -14 to Day -1			18/08/2010		Eyes	Normal		
S108	115	Hepatitis B	Hepatitis B		Day -14 to Day -1			18/08/2010		Ears, nose and throat	Normal		
S108	115	Hepatitis B	Hepatitis B		Day -14 to Day -1			18/08/2010		Cardiovascular	Normal		
S108	115	Hepatitis B	Hepatitis B		Day -14 to Day -1			18/08/2010		Respiratory	Normal		
S108	115	Hepatitis B	Hepatitis B		Day -14 to Day -1			18/08/2010		Musculoskeletal	Normal		
S108	115	Hepatitis B	Hepatitis B		Day -14 to Day -1			18/08/2010		Venous system	Normal		
S108	115	Hepatitis B	Hepatitis B		Day -14 to Day -1			18/08/2010		Gastrointestinal	Normal		
S108	115	Hepatitis B	Hepatitis B		Day -14 to Day -1			18/08/2010		Lymphatic	Normal		
S108	115	Hepatitis B	Hepatitis B		Day -14 to Day -1			18/08/2010		Neurological	Normal		
S108	115	Hepatitis B	Hepatitis B		Day -14 to Day -1			18/08/2010		Genitourinary	Normal		
S108	115	Hepatitis B	Hepatitis B		Day -14 to Day -1			18/08/2010		Dermatological	Normal		
S108	115	Hepatitis B	Hepatitis B	1	Day 0			27/08/2010	Yes	Head and neck	Normal		
S108	115	Hepatitis B	Hepatitis B	1	Day 0			27/08/2010	Yes	Eyes	Normal		
S108	115	Hepatitis B	Hepatitis B	1	Day 0			27/08/2010	Yes	Ears, nose and throat	Normal		
S108	115	Hepatitis B	Hepatitis B	1	Day 0			27/08/2010	Yes	Cardiovascular	Normal		

Subject No.	Randomisation Number	Study Group	Dosage	Dose	Day	Not Done	Specify	Date of Assessment	*Was the assessment performed prior to vaccination?	Physical Examination Parameter	Result	Abnormality	Clinical Significance
S108	115	Hepatitis B	Hepatitis B	1	Day 0			27/08/2010	Yes	Respiratory	Normal		
S108	115	Hepatitis B	Hepatitis B	1	Day 0			27/08/2010	Yes	Musculoskeletal	Normal		
S108	115	Hepatitis B	Hepatitis B	1	Day 0			27/08/2010	Yes	Venous system	Normal		
S108	115	Hepatitis B	Hepatitis B	1	Day 0			27/08/2010	Yes	Gastrointestinal	Normal		
S108	115	Hepatitis B	Hepatitis B	1	Day 0			27/08/2010	Yes	Lymphatic	Normal		
S108	115	Hepatitis B	Hepatitis B	1	Day 0			27/08/2010	Yes	Neurological	Normal		
S108	115	Hepatitis B	Hepatitis B	1	Day 0			27/08/2010	Yes	Genitourinary	Normal		
S108	115	Hepatitis B	Hepatitis B	1	Day 0			27/08/2010	Yes	Dermatological	Normal		
S108	115	Hepatitis B	Hepatitis B	1	Day 7			03/09/2010		Head and Neck	Normal		
S108	115	Hepatitis B	Hepatitis B	1	Day 7			03/09/2010		Eyes	Normal		
S108	115	Hepatitis B	Hepatitis B	1	Day 7			03/09/2010		Ears, nose and throat	Normal		
S108	115	Hepatitis B	Hepatitis B	1	Day 7			03/09/2010		Cardiovascular	Normal		
S108	115	Hepatitis B	Hepatitis B	1	Day 7			03/09/2010		Respiratory	Normal		
S108	115	Hepatitis B	Hepatitis B	1	Day 7			03/09/2010		Musculoskeletal	Normal		
S108	115	Hepatitis B	Hepatitis B	1	Day 7			03/09/2010		Venous system	Normal		
S108	115	Hepatitis B	Hepatitis B	1	Day 7			03/09/2010		Gastrointestinal	Normal		
S108	115	Hepatitis B	Hepatitis B	1	Day 7			03/09/2010		Lymphatic	Normal		
S108	115	Hepatitis B	Hepatitis B	1	Day 7			03/09/2010		Neurological	Normal		
S108	115	Hepatitis B	Hepatitis B	1	Day 7			03/09/2010		Genitourinary	Normal		

Subject No.	Randomisation Number	Study Group	Dosage	Dose	Day	Not Done	Specify	Date of Assessment	*Was the assessment performed prior to vaccination ?	Physical Examination Parameter	Result	Abnormality	Clinical Significance
S108	115	Hepatitis B	Hepatitis B	1	Day 7			03/09/2010		Dermatological	Normal		
S108	115	Hepatitis B	Hepatitis B	1	Day 14			09/09/2010		Head and Neck	Normal		
S108	115	Hepatitis B	Hepatitis B	1	Day 14			09/09/2010		Eyes	Normal		
S108	115	Hepatitis B	Hepatitis B	1	Day 14			09/09/2010		Ears, nose and throat	Normal		
S108	115	Hepatitis B	Hepatitis B	1	Day 14			09/09/2010		Cardiovascular	Normal		
S108	115	Hepatitis B	Hepatitis B	1	Day 14			09/09/2010		Respiratory	Normal		
S108	115	Hepatitis B	Hepatitis B	1	Day 14			09/09/2010		Musculoskeletal	Normal		
S108	115	Hepatitis B	Hepatitis B	1	Day 14			09/09/2010		Venous system	Normal		
S108	115	Hepatitis B	Hepatitis B	1	Day 14			09/09/2010		Gastrointestinal	Normal		
S108	115	Hepatitis B	Hepatitis B	1	Day 14			09/09/2010		Lymphatic	Normal		
S108	115	Hepatitis B	Hepatitis B	1	Day 14			09/09/2010		Neurological	Normal		
S108	115	Hepatitis B	Hepatitis B	1	Day 14			09/09/2010		Genitourinary	Normal		
S108	115	Hepatitis B	Hepatitis B	1	Day 14			09/09/2010		Dermatological	Normal		
S108	115	Hepatitis B	Hepatitis B	2	Day 28			24/09/2010	Yes	Head and Neck	Normal		
S108	115	Hepatitis B	Hepatitis B	2	Day 28			24/09/2010	Yes	Eyes	Normal		
S108	115	Hepatitis B	Hepatitis B	2	Day 28			24/09/2010	Yes	Ears, nose and throat	Normal		
S108	115	Hepatitis B	Hepatitis B	2	Day 28			24/09/2010	Yes	Cardiovascular	Normal		
S108	115	Hepatitis B	Hepatitis B	2	Day 28			24/09/2010	Yes	Respiratory	Normal		
S108	115	Hepatitis B	Hepatitis B	2	Day 28			24/09/2010	Yes	Musculoskeletal	Normal		

Subject No.	Randomisation Number	Study Group	Dosage	Dose	Day	Not Done	Specify	Date of Assessment	*Was the assessment performed prior to vaccination?	Physical Examination Parameter	Result	Abnormality	Clinical Significance
S108	115	Hepatitis B	Hepatitis B	2	Day 28			24/09/2010	Yes	Venous system	Normal		
S108	115	Hepatitis B	Hepatitis B	2	Day 28			24/09/2010	Yes	Gastrointestinal	Normal		
S108	115	Hepatitis B	Hepatitis B	2	Day 28			24/09/2010	Yes	Lymphatic	Normal		
S108	115	Hepatitis B	Hepatitis B	2	Day 28			24/09/2010	Yes	Neurological	Normal		
S108	115	Hepatitis B	Hepatitis B	2	Day 28			24/09/2010	Yes	Genitourinary	Normal		
S108	115	Hepatitis B	Hepatitis B	2	Day 28			24/09/2010	Yes	Dermatological	Normal		
S108	115	Hepatitis B	Hepatitis B	2	Day 35			01/10/2010		Head and Neck	Normal		
S108	115	Hepatitis B	Hepatitis B	2	Day 35			01/10/2010		Eyes	Normal		
S108	115	Hepatitis B	Hepatitis B	2	Day 35			01/10/2010		Ears, nose and throat	Normal		
S108	115	Hepatitis B	Hepatitis B	2	Day 35			01/10/2010		Cardiovascular	Normal		
S108	115	Hepatitis B	Hepatitis B	2	Day 35			01/10/2010		Respiratory	Normal		
S108	115	Hepatitis B	Hepatitis B	2	Day 35			01/10/2010		Musculoskeletal	Normal		
S108	115	Hepatitis B	Hepatitis B	2	Day 35			01/10/2010		Venous system	Normal		
S108	115	Hepatitis B	Hepatitis B	2	Day 35			01/10/2010		Gastrointestinal	Normal		
S108	115	Hepatitis B	Hepatitis B	2	Day 35			01/10/2010		Lymphatic	Normal		
S108	115	Hepatitis B	Hepatitis B	2	Day 35			01/10/2010		Neurological	Normal		
S108	115	Hepatitis B	Hepatitis B	2	Day 35			01/10/2010		Genitourinary	Normal		
S108	115	Hepatitis B	Hepatitis B	2	Day 35			01/10/2010		Dermatological	Normal		
S108	115	Hepatitis B	Hepatitis B	2	Day 42			08/10/2010		Head and Neck	Normal		

Subject No.	Randomisation Number	Study Group	Dosage	Dose	Day	Not Done	Specify	Date of Assessment	*Was the assessment performed prior to vaccination?	Physical Examination Parameter	Result	Abnormality	Clinical Significance
S108	115	Hepatitis B	Hepatitis B	2	Day 42			08/10/2010		Eyes	Normal		
S108	115	Hepatitis B	Hepatitis B	2	Day 42			08/10/2010		Ears, nose and throat	Normal		
S108	115	Hepatitis B	Hepatitis B	2	Day 42			08/10/2010		Cardiovascular	Normal		
S108	115	Hepatitis B	Hepatitis B	2	Day 42			08/10/2010		Respiratory	Normal		
S108	115	Hepatitis B	Hepatitis B	2	Day 42			08/10/2010		Musculoskeletal	Normal		
S108	115	Hepatitis B	Hepatitis B	2	Day 42			08/10/2010		Venous system	Normal		
S108	115	Hepatitis B	Hepatitis B	2	Day 42			08/10/2010		Gastrointestinal	Normal		
S108	115	Hepatitis B	Hepatitis B	2	Day 42			08/10/2010		Lymphatic	Normal		
S108	115	Hepatitis B	Hepatitis B	2	Day 42			08/10/2010		Neurological	Normal		
S108	115	Hepatitis B	Hepatitis B	2	Day 42			08/10/2010		Genitourinary	Normal		
S108	115	Hepatitis B	Hepatitis B	2	Day 42			08/10/2010		Dermatological	Normal		
S108	115	Hepatitis B	Hepatitis B	2	Day 56			22/10/2010		Head and Neck	Normal		
S108	115	Hepatitis B	Hepatitis B	2	Day 56			22/10/2010		Eyes	Normal		
S108	115	Hepatitis B	Hepatitis B	2	Day 56			22/10/2010		Ears, nose and throat	Normal		
S108	115	Hepatitis B	Hepatitis B	2	Day 56			22/10/2010		Cardiovascular	Normal		
S108	115	Hepatitis B	Hepatitis B	2	Day 56			22/10/2010		Respiratory	Normal		
S108	115	Hepatitis B	Hepatitis B	2	Day 56			22/10/2010		Musculoskeletal	Normal		
S108	115	Hepatitis B	Hepatitis B	2	Day 56			22/10/2010		Venous system	Normal		
S108	115	Hepatitis B	Hepatitis B	2	Day 56			22/10/2010		Gastrointestinal	Normal		

Subject No.	Randomisation Number	Study Group	Dosage	Dose	Day	Not Done	Specify	Date of Assessment	*Was the assessment performed prior to vaccination?	Physical Examination Parameter	Result	Abnormality	Clinical Significance
S108	115	Hepatitis B	Hepatitis B	2	Day 56			22/10/2010		Lymphatic	Normal		
S108	115	Hepatitis B	Hepatitis B	2	Day 56			22/10/2010		Neurological	Normal		
S108	115	Hepatitis B	Hepatitis B	2	Day 56			22/10/2010		Genitourinary	Normal		
S108	115	Hepatitis B	Hepatitis B	2	Day 56			22/10/2010		Dermatological	Normal		
S108	115	Hepatitis B	Hepatitis B	3	Day 180			23/02/2011	Yes	Head and Neck	Normal		
S108	115	Hepatitis B	Hepatitis B	3	Day 180			23/02/2011	Yes	Eyes	Normal		
S108	115	Hepatitis B	Hepatitis B	3	Day 180			23/02/2011	Yes	Ears, nose and throat	Normal		
S108	115	Hepatitis B	Hepatitis B	3	Day 180			23/02/2011	Yes	Cardiovascular	Normal		
S108	115	Hepatitis B	Hepatitis B	3	Day 180			23/02/2011	Yes	Respiratory	Normal		
S108	115	Hepatitis B	Hepatitis B	3	Day 180			23/02/2011	Yes	Musculoskeletal	Normal		
S108	115	Hepatitis B	Hepatitis B	3	Day 180			23/02/2011	Yes	Venous system	Normal		
S108	115	Hepatitis B	Hepatitis B	3	Day 180			23/02/2011	Yes	Gastrointestinal	Normal		
S108	115	Hepatitis B	Hepatitis B	3	Day 180			23/02/2011	Yes	Lymphatic	Normal		
S108	115	Hepatitis B	Hepatitis B	3	Day 180			23/02/2011	Yes	Neurological	Normal		
S108	115	Hepatitis B	Hepatitis B	3	Day 180			23/02/2011	Yes	Genitourinary	Normal		
S108	115	Hepatitis B	Hepatitis B	3	Day 180			23/02/2011	Yes	Dermatological	Normal		
S108	115	Hepatitis B	Hepatitis B	3	Day 187			02/03/2011		Head and Neck	Normal		
S108	115	Hepatitis B	Hepatitis B	3	Day 187			02/03/2011		Eyes	Normal		
S108	115	Hepatitis B	Hepatitis B	3	Day 187			02/03/2011		Ears, nose and throat	Normal		

Subject No.	Randomisation Number	Study Group	Dosage	Dose	Day	Not Done	Specify	Date of Assessment	*Was the assessment performed prior to vaccination?	Physical Examination Parameter	Result	Abnormality	Clinical Significance
S108	115	Hepatitis B	Hepatitis B	3	Day 187			02/03/2011		Cardiovascular	Normal		
S108	115	Hepatitis B	Hepatitis B	3	Day 187			02/03/2011		Respiratory	Normal		
S108	115	Hepatitis B	Hepatitis B	3	Day 187			02/03/2011		Musculoskeletal	Normal		
S108	115	Hepatitis B	Hepatitis B	3	Day 187			02/03/2011		Venous system	Normal		
S108	115	Hepatitis B	Hepatitis B	3	Day 187			02/03/2011		Gastrointestinal	Normal		
S108	115	Hepatitis B	Hepatitis B	3	Day 187			02/03/2011		Lymphatic	Normal		
S108	115	Hepatitis B	Hepatitis B	3	Day 187			02/03/2011		Neurological	Normal		
S108	115	Hepatitis B	Hepatitis B	3	Day 187			02/03/2011		Genitourinary	Normal		
S108	115	Hepatitis B	Hepatitis B	3	Day 187			02/03/2011		Dermatological	Normal		
S108	115	Hepatitis B	Hepatitis B	3	Day 194			09/03/2011		Head and Neck	Normal		
S108	115	Hepatitis B	Hepatitis B	3	Day 194			09/03/2011		Eyes	Normal		
S108	115	Hepatitis B	Hepatitis B	3	Day 194			09/03/2011		Ears, nose and throat	Normal		
S108	115	Hepatitis B	Hepatitis B	3	Day 194			09/03/2011		Cardiovascular	Normal		
S108	115	Hepatitis B	Hepatitis B	3	Day 194			09/03/2011		Respiratory	Normal		
S108	115	Hepatitis B	Hepatitis B	3	Day 194			09/03/2011		Musculoskeletal	Normal		
S108	115	Hepatitis B	Hepatitis B	3	Day 194			09/03/2011		Venous system	Normal		
S108	115	Hepatitis B	Hepatitis B	3	Day 194			09/03/2011		Gastrointestinal	Normal		
S108	115	Hepatitis B	Hepatitis B	3	Day 194			09/03/2011		Lymphatic	Normal		
S108	115	Hepatitis B	Hepatitis B	3	Day 194			09/03/2011		Neurological	Normal		

Subject No.	Randomisation Number	Study Group	Dosage	Dose	Day	Not Done	Specify	Date of Assessment	*Was the assessment performed prior to vaccination?	Physical Examination Parameter	Result	Abnormality	Clinical Significance
S108	115	Hepatitis B	Hepatitis B	3	Day 194			09/03/2011		Genitourinary	Normal		
S108	115	Hepatitis B	Hepatitis B	3	Day 194			09/03/2011		Dermatological	Normal		
S108	115	Hepatitis B	Hepatitis B	3	Day 208			23/03/2011		Head and Neck	Normal		
S108	115	Hepatitis B	Hepatitis B	3	Day 208			23/03/2011		Eyes	Normal		
S108	115	Hepatitis B	Hepatitis B	3	Day 208			23/03/2011		Ears, nose and throat	Normal		
S108	115	Hepatitis B	Hepatitis B	3	Day 208			23/03/2011		Cardiovascular	Normal		
S108	115	Hepatitis B	Hepatitis B	3	Day 208			23/03/2011		Respiratory	Normal		
S108	115	Hepatitis B	Hepatitis B	3	Day 208			23/03/2011		Musculoskeletal	Normal		
S108	115	Hepatitis B	Hepatitis B	3	Day 208			23/03/2011		Venous system	Normal		
S108	115	Hepatitis B	Hepatitis B	3	Day 208			23/03/2011		Gastrointestinal	Normal		
S108	115	Hepatitis B	Hepatitis B	3	Day 208			23/03/2011		Lymphatic	Normal		
S108	115	Hepatitis B	Hepatitis B	3	Day 208			23/03/2011		Neurological	Normal		
S108	115	Hepatitis B	Hepatitis B	3	Day 208			23/03/2011		Genitourinary	Normal		
S108	115	Hepatitis B	Hepatitis B	3	Day 208			23/03/2011		Dermatological	Normal		
S108	115	Hepatitis B	Hepatitis B	3	Day 365			26/08/2011		Head and Neck	Normal		
S108	115	Hepatitis B	Hepatitis B	3	Day 365			26/08/2011		Eyes	Normal		
S108	115	Hepatitis B	Hepatitis B	3	Day 365			26/08/2011		Ears, nose and throat	Normal		
S108	115	Hepatitis B	Hepatitis B	3	Day 365			26/08/2011		Cardiovascular	Normal		
S108	115	Hepatitis B	Hepatitis B	3	Day 365			26/08/2011		Respiratory	Normal		

Subject No.	Randomisation Number	Study Group	Dosage	Dose	Day	Not Done	Specify	Date of Assessment	*Was the assessment performed prior to vaccination?	Physical Examination Parameter	Result	Abnormality	Clinical Significance
S108	115	Hepatitis B	Hepatitis B	3	Day 365			26/08/2011		Musculoskeletal	Normal		
S108	115	Hepatitis B	Hepatitis B	3	Day 365			26/08/2011		Venous system	Normal		
S108	115	Hepatitis B	Hepatitis B	3	Day 365			26/08/2011		Gastrointestinal	Normal		
S108	115	Hepatitis B	Hepatitis B	3	Day 365			26/08/2011		Lymphatic	Normal		
S108	115	Hepatitis B	Hepatitis B	3	Day 365			26/08/2011		Neurological	Normal		
S108	115	Hepatitis B	Hepatitis B	3	Day 365			26/08/2011		Genitourinary	Normal		
S108	115	Hepatitis B	Hepatitis B	3	Day 365			26/08/2011		Dermatological	Normal		
S108	115	Hepatitis B	Hepatitis B		Unscheduled Visit	Yes	Laboratory Repeat At Local Lab	.		Head and Neck			
S108	115	Hepatitis B	Hepatitis B		Unscheduled Visit	Yes	Laboratory Repeat At Local Lab	.		Eyes			
S108	115	Hepatitis B	Hepatitis B		Unscheduled Visit	Yes	Laboratory Repeat At Local Lab	.		Ears, nose and throat			
S108	115	Hepatitis B	Hepatitis B		Unscheduled Visit	Yes	Laboratory Repeat At Local Lab	.		Cardiovascular			
S108	115	Hepatitis B	Hepatitis B		Unscheduled Visit	Yes	Laboratory Repeat At Local Lab	.		Respiratory			
S108	115	Hepatitis B	Hepatitis B		Unscheduled Visit	Yes	Laboratory Repeat At Local Lab	.		Musculoskeletal			
S108	115	Hepatitis B	Hepatitis B		Unscheduled Visit	Yes	Laboratory Repeat At Local Lab	.		Venous system			

Subject No.	Randomisation Number	Study Group	Dosage	Dose	Day	Not Done	Specify	Date of Assessment	*Was the assessment performed prior to vaccination?	Physical Examination Parameter	Result	Abnormality	Clinical Significance
S108	115	Hepatitis B	Hepatitis B		Unscheduled Visit	Yes	Laboratory Repeat At Local Lab	.		Gastrointestinal			
S108	115	Hepatitis B	Hepatitis B		Unscheduled Visit	Yes	Laboratory Repeat At Local Lab	.		Lymphatic			
S108	115	Hepatitis B	Hepatitis B		Unscheduled Visit	Yes	Laboratory Repeat At Local Lab	.		Neurological			
S108	115	Hepatitis B	Hepatitis B		Unscheduled Visit	Yes	Laboratory Repeat At Local Lab	.		Genitourinary			
S108	115	Hepatitis B	Hepatitis B		Unscheduled Visit	Yes	Laboratory Repeat At Local Lab	.		Dermatological			
S108	115	Hepatitis B	Hepatitis B		Unscheduled Visit	Yes	Laboratory Repeat At Local Lab	.		Head and Neck			
S108	115	Hepatitis B	Hepatitis B		Unscheduled Visit	Yes	Laboratory Repeat At Local Lab	.		Eyes			
S108	115	Hepatitis B	Hepatitis B		Unscheduled Visit	Yes	Laboratory Repeat At Local Lab	.		Ears, nose and throat			
S108	115	Hepatitis B	Hepatitis B		Unscheduled Visit	Yes	Laboratory Repeat At Local Lab	.		Cardiovascular			
S108	115	Hepatitis B	Hepatitis B		Unscheduled Visit	Yes	Laboratory Repeat At Local Lab	.		Respiratory			
S108	115	Hepatitis B	Hepatitis B		Unscheduled Visit	Yes	Laboratory Repeat At Local Lab	.		Musculoskeletal			
S108	115	Hepatitis B	Hepatitis B		Unscheduled Visit	Yes	Laboratory Repeat At Local Lab	.		Venous system			

Subject No.	Randomisation Number	Study Group	Dosage	Dose	Day	Not Done	Specify	Date of Assessment	*Was the assessment performed prior to vaccination?	Physical Examination Parameter	Result	Abnormality	Clinical Significance
S108	115	Hepatitis B	Hepatitis B		Unscheduled Visit	Yes	Laboratory Repeat At Local Lab	.		Gastrointestinal			
S108	115	Hepatitis B	Hepatitis B		Unscheduled Visit	Yes	Laboratory Repeat At Local Lab	.		Lymphatic			
S108	115	Hepatitis B	Hepatitis B		Unscheduled Visit	Yes	Laboratory Repeat At Local Lab	.		Neurological			
S108	115	Hepatitis B	Hepatitis B		Unscheduled Visit	Yes	Laboratory Repeat At Local Lab	.		Genitourinary			
S108	115	Hepatitis B	Hepatitis B		Unscheduled Visit	Yes	Laboratory Repeat At Local Lab	.		Dermatological			
S108	115	Hepatitis B	Hepatitis B		Unscheduled Visit	Yes	Laboratory Repeat At Local Lab	.		Head and Neck			
S108	115	Hepatitis B	Hepatitis B		Unscheduled Visit	Yes	Laboratory Repeat At Local Lab	.		Eyes			
S108	115	Hepatitis B	Hepatitis B		Unscheduled Visit	Yes	Laboratory Repeat At Local Lab	.		Ears, nose and throat			
S108	115	Hepatitis B	Hepatitis B		Unscheduled Visit	Yes	Laboratory Repeat At Local Lab	.		Cardiovascular			
S108	115	Hepatitis B	Hepatitis B		Unscheduled Visit	Yes	Laboratory Repeat At Local Lab	.		Respiratory			
S108	115	Hepatitis B	Hepatitis B		Unscheduled Visit	Yes	Laboratory Repeat At Local Lab	.		Musculoskeletal			
S108	115	Hepatitis B	Hepatitis B		Unscheduled Visit	Yes	Laboratory Repeat At Local Lab	.		Venous system			

Subject No.	Randomisation Number	Study Group	Dosage	Dose	Day	Not Done	Specify	Date of Assessment	*Was the assessment performed prior to vaccination?	Physical Examination Parameter	Result	Abnormality	Clinical Significance
S108	115	Hepatitis B	Hepatitis B		Unscheduled Visit	Yes	Laboratory Repeat At Local Lab	.		Gastrointestinal			
S108	115	Hepatitis B	Hepatitis B		Unscheduled Visit	Yes	Laboratory Repeat At Local Lab	.		Lymphatic			
S108	115	Hepatitis B	Hepatitis B		Unscheduled Visit	Yes	Laboratory Repeat At Local Lab	.		Neurological			
S108	115	Hepatitis B	Hepatitis B		Unscheduled Visit	Yes	Laboratory Repeat At Local Lab	.		Genitourinary			
S108	115	Hepatitis B	Hepatitis B		Unscheduled Visit	Yes	Laboratory Repeat At Local Lab	.		Dermatological			
S118	114	JAIVAC-1	Dosage A		Day -14 to Day -1			17/08/2010		Head and neck	Normal		
S118	114	JAIVAC-1	Dosage A		Day -14 to Day -1			17/08/2010		Eyes	Normal		
S118	114	JAIVAC-1	Dosage A		Day -14 to Day -1			17/08/2010		Ears, nose and throat	Normal		
S118	114	JAIVAC-1	Dosage A		Day -14 to Day -1			17/08/2010		Cardiovascular	Normal		
S118	114	JAIVAC-1	Dosage A		Day -14 to Day -1			17/08/2010		Respiratory	Normal		
S118	114	JAIVAC-1	Dosage A		Day -14 to Day -1			17/08/2010		Musculoskeletal	Normal		
S118	114	JAIVAC-1	Dosage A		Day -14 to Day -1			17/08/2010		Venous system	Normal		
S118	114	JAIVAC-1	Dosage A		Day -14 to Day -1			17/08/2010		Gastrointestinal	Normal		
S118	114	JAIVAC-1	Dosage A		Day -14 to Day -1			17/08/2010		Lymphatic	Normal		

Subject No.	Randomisation Number	Study Group	Dosage	Dose	Day	Not Done	Specify	Date of Assessment	*Was the assessment performed prior to vaccination?	Physical Examination Parameter	Result	Abnormality	Clinical Significance
S118	114	JAIVAC-1	Dosage A		Day -14 to Day -1			17/08/2010		Neurological	Normal		
S118	114	JAIVAC-1	Dosage A		Day -14 to Day -1			17/08/2010		Genitourinary	Normal		
S118	114	JAIVAC-1	Dosage A		Day -14 to Day -1			17/08/2010		Dermatological	Normal		
S118	114	JAIVAC-1	Dosage A	1	Day 0			27/08/2010	Yes	Head and neck	Normal		
S118	114	JAIVAC-1	Dosage A	1	Day 0			27/08/2010	Yes	Eyes	Normal		
S118	114	JAIVAC-1	Dosage A	1	Day 0			27/08/2010	Yes	Ears, nose and throat	Normal		
S118	114	JAIVAC-1	Dosage A	1	Day 0			27/08/2010	Yes	Cardiovascular	Normal		
S118	114	JAIVAC-1	Dosage A	1	Day 0			27/08/2010	Yes	Respiratory	Normal		
S118	114	JAIVAC-1	Dosage A	1	Day 0			27/08/2010	Yes	Musculoskeletal	Normal		
S118	114	JAIVAC-1	Dosage A	1	Day 0			27/08/2010	Yes	Venous system	Normal		
S118	114	JAIVAC-1	Dosage A	1	Day 0			27/08/2010	Yes	Gastrointestinal	Normal		
S118	114	JAIVAC-1	Dosage A	1	Day 0			27/08/2010	Yes	Lymphatic	Normal		
S118	114	JAIVAC-1	Dosage A	1	Day 0			27/08/2010	Yes	Neurological	Normal		
S118	114	JAIVAC-1	Dosage A	1	Day 0			27/08/2010	Yes	Genitourinary	Normal		
S118	114	JAIVAC-1	Dosage A	1	Day 0			27/08/2010	Yes	Dermatological	Normal		
S118	114	JAIVAC-1	Dosage A	1	Day 7			03/09/2010		Head and Neck	Normal		
S118	114	JAIVAC-1	Dosage A	1	Day 7			03/09/2010		Eyes	Normal		
S118	114	JAIVAC-1	Dosage A	1	Day 7			03/09/2010		Ears, nose and throat	Normal		

Subject No.	Randomisation Number	Study Group	Dosage	Dose	Day	Not Done	Specify	Date of Assessment	*Was the assessment performed prior to vaccination?	Physical Examination Parameter	Result	Abnormality	Clinical Significance
S118	114	JAIVAC-1	Dosage A	1	Day 7			03/09/2010		Cardiovascular	Normal		
S118	114	JAIVAC-1	Dosage A	1	Day 7			03/09/2010		Respiratory	Normal		
S118	114	JAIVAC-1	Dosage A	1	Day 7			03/09/2010		Musculoskeletal	Normal		
S118	114	JAIVAC-1	Dosage A	1	Day 7			03/09/2010		Venous system	Normal		
S118	114	JAIVAC-1	Dosage A	1	Day 7			03/09/2010		Gastrointestinal	Normal		
S118	114	JAIVAC-1	Dosage A	1	Day 7			03/09/2010		Lymphatic	Normal		
S118	114	JAIVAC-1	Dosage A	1	Day 7			03/09/2010		Neurological	Normal		
S118	114	JAIVAC-1	Dosage A	1	Day 7			03/09/2010		Genitourinary	Normal		
S118	114	JAIVAC-1	Dosage A	1	Day 7			03/09/2010		Dermatological	Normal		
S118	114	JAIVAC-1	Dosage A	1	Day 14			09/09/2010		Head and Neck	Normal		
S118	114	JAIVAC-1	Dosage A	1	Day 14			09/09/2010		Eyes	Normal		
S118	114	JAIVAC-1	Dosage A	1	Day 14			09/09/2010		Ears, nose and throat	Normal		
S118	114	JAIVAC-1	Dosage A	1	Day 14			09/09/2010		Cardiovascular	Normal		
S118	114	JAIVAC-1	Dosage A	1	Day 14			09/09/2010		Respiratory	Normal		
S118	114	JAIVAC-1	Dosage A	1	Day 14			09/09/2010		Musculoskeletal	Normal		
S118	114	JAIVAC-1	Dosage A	1	Day 14			09/09/2010		Venous system	Normal		
S118	114	JAIVAC-1	Dosage A	1	Day 14			09/09/2010		Gastrointestinal	Normal		
S118	114	JAIVAC-1	Dosage A	1	Day 14			09/09/2010		Lymphatic	Normal		
S118	114	JAIVAC-1	Dosage A	1	Day 14			09/09/2010		Neurological	Normal		

Subject No.	Randomisation Number	Study Group	Dosage	Dose	Day	Not Done	Specify	Date of Assessment	*Was the assessment performed prior to vaccination?	Physical Examination Parameter	Result	Abnormality	Clinical Significance
S118	114	JAIVAC-1	Dosage A	1	Day 14			09/09/2010		Genitourinary	Normal		
S118	114	JAIVAC-1	Dosage A	1	Day 14			09/09/2010		Dermatological	Normal		
S118	114	JAIVAC-1	Dosage A	2	Day 28			24/09/2010	Yes	Head and Neck	Normal		
S118	114	JAIVAC-1	Dosage A	2	Day 28			24/09/2010	Yes	Eyes	Normal		
S118	114	JAIVAC-1	Dosage A	2	Day 28			24/09/2010	Yes	Ears, nose and throat	Normal		
S118	114	JAIVAC-1	Dosage A	2	Day 28			24/09/2010	Yes	Cardiovascular	Normal		
S118	114	JAIVAC-1	Dosage A	2	Day 28			24/09/2010	Yes	Respiratory	Normal		
S118	114	JAIVAC-1	Dosage A	2	Day 28			24/09/2010	Yes	Musculoskeletal	Normal		
S118	114	JAIVAC-1	Dosage A	2	Day 28			24/09/2010	Yes	Venous system	Normal		
S118	114	JAIVAC-1	Dosage A	2	Day 28			24/09/2010	Yes	Gastrointestinal	Normal		
S118	114	JAIVAC-1	Dosage A	2	Day 28			24/09/2010	Yes	Lymphatic	Normal		
S118	114	JAIVAC-1	Dosage A	2	Day 28			24/09/2010	Yes	Neurological	Normal		
S118	114	JAIVAC-1	Dosage A	2	Day 28			24/09/2010	Yes	Genitourinary	Normal		
S118	114	JAIVAC-1	Dosage A	2	Day 28			24/09/2010	Yes	Dermatological	Normal		
S118	114	JAIVAC-1	Dosage A	2	Day 35			30/09/2010		Head and Neck	Normal		
S118	114	JAIVAC-1	Dosage A	2	Day 35			30/09/2010		Eyes	Normal		
S118	114	JAIVAC-1	Dosage A	2	Day 35			30/09/2010		Ears, nose and throat	Normal		
S118	114	JAIVAC-1	Dosage A	2	Day 35			30/09/2010		Cardiovascular	Normal		
S118	114	JAIVAC-1	Dosage A	2	Day 35			30/09/2010		Respiratory	Normal		

Subject No.	Randomisation Number	Study Group	Dosage	Dose	Day	Not Done	Specify	Date of Assessment	*Was the assessment performed prior to vaccination?	Physical Examination Parameter	Result	Abnormality	Clinical Significance
S118	114	JAIVAC-1	Dosage A	2	Day 35			30/09/2010		Musculoskeletal	Normal		
S118	114	JAIVAC-1	Dosage A	2	Day 35			30/09/2010		Venous system	Normal		
S118	114	JAIVAC-1	Dosage A	2	Day 35			30/09/2010		Gastrointestinal	Normal		
S118	114	JAIVAC-1	Dosage A	2	Day 35			30/09/2010		Lymphatic	Normal		
S118	114	JAIVAC-1	Dosage A	2	Day 35			30/09/2010		Neurological	Normal		
S118	114	JAIVAC-1	Dosage A	2	Day 35			30/09/2010		Genitourinary	Normal		
S118	114	JAIVAC-1	Dosage A	2	Day 35			30/09/2010		Dermatological	Normal		
S118	114	JAIVAC-1	Dosage A	2	Day 42			08/10/2010		Head and Neck	Normal		
S118	114	JAIVAC-1	Dosage A	2	Day 42			08/10/2010		Eyes	Normal		
S118	114	JAIVAC-1	Dosage A	2	Day 42			08/10/2010		Ears, nose and throat	Normal		
S118	114	JAIVAC-1	Dosage A	2	Day 42			08/10/2010		Cardiovascular	Normal		
S118	114	JAIVAC-1	Dosage A	2	Day 42			08/10/2010		Respiratory	Normal		
S118	114	JAIVAC-1	Dosage A	2	Day 42			08/10/2010		Musculoskeletal	Normal		
S118	114	JAIVAC-1	Dosage A	2	Day 42			08/10/2010		Venous system	Normal		
S118	114	JAIVAC-1	Dosage A	2	Day 42			08/10/2010		Gastrointestinal	Normal		
S118	114	JAIVAC-1	Dosage A	2	Day 42			08/10/2010		Lymphatic	Normal		
S118	114	JAIVAC-1	Dosage A	2	Day 42			08/10/2010		Neurological	Normal		
S118	114	JAIVAC-1	Dosage A	2	Day 42			08/10/2010		Genitourinary	Normal		
S118	114	JAIVAC-1	Dosage A	2	Day 42			08/10/2010		Dermatological	Normal		

Subject No.	Randomisation Number	Study Group	Dosage	Dose	Day	Not Done	Specify	Date of Assessment	*Was the assessment performed prior to vaccination?	Physical Examination Parameter	Result	Abnormality	Clinical Significance
S118	114	JAIVAC-1	Dosage A	2	Day 56			22/10/2010		Head and Neck	Normal		
S118	114	JAIVAC-1	Dosage A	2	Day 56			22/10/2010		Eyes	Normal		
S118	114	JAIVAC-1	Dosage A	2	Day 56			22/10/2010		Ears, nose and throat	Normal		
S118	114	JAIVAC-1	Dosage A	2	Day 56			22/10/2010		Cardiovascular	Normal		
S118	114	JAIVAC-1	Dosage A	2	Day 56			22/10/2010		Respiratory	Normal		
S118	114	JAIVAC-1	Dosage A	2	Day 56			22/10/2010		Musculoskeletal	Normal		
S118	114	JAIVAC-1	Dosage A	2	Day 56			22/10/2010		Venous system	Normal		
S118	114	JAIVAC-1	Dosage A	2	Day 56			22/10/2010		Gastrointestinal	Normal		
S118	114	JAIVAC-1	Dosage A	2	Day 56			22/10/2010		Lymphatic	Normal		
S118	114	JAIVAC-1	Dosage A	2	Day 56			22/10/2010		Neurological	Normal		
S118	114	JAIVAC-1	Dosage A	2	Day 56			22/10/2010		Genitourinary	Normal		
S118	114	JAIVAC-1	Dosage A	2	Day 56			22/10/2010		Dermatological	Normal		
S118	114	JAIVAC-1	Dosage A	3	Day 180			23/02/2011	Yes	Head and Neck	Normal		
S118	114	JAIVAC-1	Dosage A	3	Day 180			23/02/2011	Yes	Eyes	Normal		
S118	114	JAIVAC-1	Dosage A	3	Day 180			23/02/2011	Yes	Ears, nose and throat	Normal		
S118	114	JAIVAC-1	Dosage A	3	Day 180			23/02/2011	Yes	Cardiovascular	Normal		
S118	114	JAIVAC-1	Dosage A	3	Day 180			23/02/2011	Yes	Respiratory	Normal		
S118	114	JAIVAC-1	Dosage A	3	Day 180			23/02/2011	Yes	Musculoskeletal	Normal		
S118	114	JAIVAC-1	Dosage A	3	Day 180			23/02/2011	Yes	Venous system	Normal		

Subject No.	Randomisation Number	Study Group	Dosage	Dose	Day	Not Done	Specify	Date of Assessment	*Was the assessment performed prior to vaccination?	Physical Examination Parameter	Result	Abnormality	Clinical Significance
S118	114	JAIVAC-1	Dosage A	3	Day 180			23/02/2011	Yes	Gastrointestinal	Normal		
S118	114	JAIVAC-1	Dosage A	3	Day 180			23/02/2011	Yes	Lymphatic	Normal		
S118	114	JAIVAC-1	Dosage A	3	Day 180			23/02/2011	Yes	Neurological	Normal		
S118	114	JAIVAC-1	Dosage A	3	Day 180			23/02/2011	Yes	Genitourinary	Normal		
S118	114	JAIVAC-1	Dosage A	3	Day 180			23/02/2011	Yes	Dermatological	Normal		
S118	114	JAIVAC-1	Dosage A	3	Day 187			02/03/2011		Head and Neck	Normal		
S118	114	JAIVAC-1	Dosage A	3	Day 187			02/03/2011		Eyes	Normal		
S118	114	JAIVAC-1	Dosage A	3	Day 187			02/03/2011		Ears, nose and throat	Normal		
S118	114	JAIVAC-1	Dosage A	3	Day 187			02/03/2011		Cardiovascular	Normal		
S118	114	JAIVAC-1	Dosage A	3	Day 187			02/03/2011		Respiratory	Normal		
S118	114	JAIVAC-1	Dosage A	3	Day 187			02/03/2011		Musculoskeletal	Normal		
S118	114	JAIVAC-1	Dosage A	3	Day 187			02/03/2011		Venous system	Normal		
S118	114	JAIVAC-1	Dosage A	3	Day 187			02/03/2011		Gastrointestinal	Normal		
S118	114	JAIVAC-1	Dosage A	3	Day 187			02/03/2011		Lymphatic	Normal		
S118	114	JAIVAC-1	Dosage A	3	Day 187			02/03/2011		Neurological	Normal		
S118	114	JAIVAC-1	Dosage A	3	Day 187			02/03/2011		Genitourinary	Normal		
S118	114	JAIVAC-1	Dosage A	3	Day 187			02/03/2011		Dermatological	Normal		
S118	114	JAIVAC-1	Dosage A	3	Day 194			09/03/2011		Head and Neck	Normal		
S118	114	JAIVAC-1	Dosage A	3	Day 194			09/03/2011		Eyes	Normal		

Subject No.	Randomisation Number	Study Group	Dosage	Dose	Day	Not Done	Specify	Date of Assessment	*Was the assessment performed prior to vaccination?	Physical Examination Parameter	Result	Abnormality	Clinical Significance
S118	114	JAIVAC-1	Dosage A	3	Day 194			09/03/2011		Ears, nose and throat	Normal		
S118	114	JAIVAC-1	Dosage A	3	Day 194			09/03/2011		Cardiovascular	Normal		
S118	114	JAIVAC-1	Dosage A	3	Day 194			09/03/2011		Respiratory	Normal		
S118	114	JAIVAC-1	Dosage A	3	Day 194			09/03/2011		Musculoskeletal	Normal		
S118	114	JAIVAC-1	Dosage A	3	Day 194			09/03/2011		Venous system	Normal		
S118	114	JAIVAC-1	Dosage A	3	Day 194			09/03/2011		Gastrointestinal	Normal		
S118	114	JAIVAC-1	Dosage A	3	Day 194			09/03/2011		Lymphatic	Normal		
S118	114	JAIVAC-1	Dosage A	3	Day 194			09/03/2011		Neurological	Normal		
S118	114	JAIVAC-1	Dosage A	3	Day 194			09/03/2011		Genitourinary	Normal		
S118	114	JAIVAC-1	Dosage A	3	Day 194			09/03/2011		Dermatological	Normal		
S118	114	JAIVAC-1	Dosage A	3	Day 208			23/03/2011		Head and Neck	Normal		
S118	114	JAIVAC-1	Dosage A	3	Day 208			23/03/2011		Eyes	Normal		
S118	114	JAIVAC-1	Dosage A	3	Day 208			23/03/2011		Ears, nose and throat	Normal		
S118	114	JAIVAC-1	Dosage A	3	Day 208			23/03/2011		Cardiovascular	Normal		
S118	114	JAIVAC-1	Dosage A	3	Day 208			23/03/2011		Respiratory	Normal		
S118	114	JAIVAC-1	Dosage A	3	Day 208			23/03/2011		Musculoskeletal	Normal		
S118	114	JAIVAC-1	Dosage A	3	Day 208			23/03/2011		Venous system	Normal		
S118	114	JAIVAC-1	Dosage A	3	Day 208			23/03/2011		Gastrointestinal	Normal		
S118	114	JAIVAC-1	Dosage A	3	Day 208			23/03/2011		Lymphatic	Normal		

Subject No.	Randomisation Number	Study Group	Dosage	Dose	Day	Not Done	Specify	Date of Assessment	*Was the assessment performed prior to vaccination?	Physical Examination Parameter	Result	Abnormality	Clinical Significance
S118	114	JAIVAC-1	Dosage A	3	Day 208			23/03/2011		Neurological	Normal		
S118	114	JAIVAC-1	Dosage A	3	Day 208			23/03/2011		Genitourinary	Normal		
S118	114	JAIVAC-1	Dosage A	3	Day 208			23/03/2011		Dermatological	Normal		
S118	114	JAIVAC-1	Dosage A	3	Day 365			26/08/2011		Head and Neck	Normal		
S118	114	JAIVAC-1	Dosage A	3	Day 365			26/08/2011		Eyes	Normal		
S118	114	JAIVAC-1	Dosage A	3	Day 365			26/08/2011		Ears, nose and throat	Normal		
S118	114	JAIVAC-1	Dosage A	3	Day 365			26/08/2011		Cardiovascular	Normal		
S118	114	JAIVAC-1	Dosage A	3	Day 365			26/08/2011		Respiratory	Normal		
S118	114	JAIVAC-1	Dosage A	3	Day 365			26/08/2011		Musculoskeletal	Normal		
S118	114	JAIVAC-1	Dosage A	3	Day 365			26/08/2011		Venous system	Normal		
S118	114	JAIVAC-1	Dosage A	3	Day 365			26/08/2011		Gastrointestinal	Normal		
S118	114	JAIVAC-1	Dosage A	3	Day 365			26/08/2011		Lymphatic	Normal		
S118	114	JAIVAC-1	Dosage A	3	Day 365			26/08/2011		Neurological	Normal		
S118	114	JAIVAC-1	Dosage A	3	Day 365			26/08/2011		Genitourinary	Normal		
S118	114	JAIVAC-1	Dosage A	3	Day 365			26/08/2011		Dermatological	Normal		
S119	107	Hepatitis B	Hepatitis B		Day -14 to Day -1			18/08/2010		Head and neck	Normal		
S119	107	Hepatitis B	Hepatitis B		Day -14 to Day -1			18/08/2010		Eyes	Normal		
S119	107	Hepatitis B	Hepatitis B		Day -14 to Day -1			18/08/2010		Ears, nose and throat	Normal		

Subject No.	Randomisation Number	Study Group	Dosage	Dose	Day	Not Done	Specify	Date of Assessment	*Was the assessment performed prior to vaccination?	Physical Examination Parameter	Result	Abnormality	Clinical Significance
S119	107	Hepatitis B	Hepatitis B		Day -14 to Day -1			18/08/2010		Cardiovascular	Normal		
S119	107	Hepatitis B	Hepatitis B		Day -14 to Day -1			18/08/2010		Respiratory	Normal		
S119	107	Hepatitis B	Hepatitis B		Day -14 to Day -1			18/08/2010		Musculoskeletal	Normal		
S119	107	Hepatitis B	Hepatitis B		Day -14 to Day -1			18/08/2010		Venous system	Normal		
S119	107	Hepatitis B	Hepatitis B		Day -14 to Day -1			18/08/2010		Gastrointestinal	Normal		
S119	107	Hepatitis B	Hepatitis B		Day -14 to Day -1			18/08/2010		Lymphatic	Normal		
S119	107	Hepatitis B	Hepatitis B		Day -14 to Day -1			18/08/2010		Neurological	Normal		
S119	107	Hepatitis B	Hepatitis B		Day -14 to Day -1			18/08/2010		Genitourinary	Normal		
S119	107	Hepatitis B	Hepatitis B		Day -14 to Day -1			18/08/2010		Dermatological	Normal		
S119	107	Hepatitis B	Hepatitis B	1	Day 0			26/08/2010	Yes	Head and neck	Normal		
S119	107	Hepatitis B	Hepatitis B	1	Day 0			26/08/2010	Yes	Eyes	Normal		
S119	107	Hepatitis B	Hepatitis B	1	Day 0			26/08/2010	Yes	Ears, nose and throat	Normal		
S119	107	Hepatitis B	Hepatitis B	1	Day 0			26/08/2010	Yes	Cardiovascular	Normal		
S119	107	Hepatitis B	Hepatitis B	1	Day 0			26/08/2010	Yes	Respiratory	Normal		
S119	107	Hepatitis B	Hepatitis B	1	Day 0			26/08/2010	Yes	Musculoskeletal	Normal		
S119	107	Hepatitis B	Hepatitis B	1	Day 0			26/08/2010	Yes	Venous system	Normal		
S119	107	Hepatitis B	Hepatitis B	1	Day 0			26/08/2010	Yes	Gastrointestinal	Normal		
S119	107	Hepatitis B	Hepatitis B	1	Day 0			26/08/2010	Yes	Lymphatic	Normal		

Subject No.	Randomisation Number	Study Group	Dosage	Dose	Day	Not Done	Specify	Date of Assessment	*Was the assessment performed prior to vaccination?	Physical Examination Parameter	Result	Abnormality	Clinical Significance
S119	107	Hepatitis B	Hepatitis B	1	Day 0			26/08/2010	Yes	Neurological	Normal		
S119	107	Hepatitis B	Hepatitis B	1	Day 0			26/08/2010	Yes	Genitourinary	Normal		
S119	107	Hepatitis B	Hepatitis B	1	Day 0			26/08/2010	Yes	Dermatological	Normal		
S119	107	Hepatitis B	Hepatitis B	1	Day 7			02/09/2010		Head and Neck	Normal		
S119	107	Hepatitis B	Hepatitis B	1	Day 7			02/09/2010		Eyes	Normal		
S119	107	Hepatitis B	Hepatitis B	1	Day 7			02/09/2010		Ears, nose and throat	Normal		
S119	107	Hepatitis B	Hepatitis B	1	Day 7			02/09/2010		Cardiovascular	Normal		
S119	107	Hepatitis B	Hepatitis B	1	Day 7			02/09/2010		Respiratory	Normal		
S119	107	Hepatitis B	Hepatitis B	1	Day 7			02/09/2010		Musculoskeletal	Normal		
S119	107	Hepatitis B	Hepatitis B	1	Day 7			02/09/2010		Venous system	Normal		
S119	107	Hepatitis B	Hepatitis B	1	Day 7			02/09/2010		Gastrointestinal	Normal		
S119	107	Hepatitis B	Hepatitis B	1	Day 7			02/09/2010		Lymphatic	Normal		
S119	107	Hepatitis B	Hepatitis B	1	Day 7			02/09/2010		Neurological	Normal		
S119	107	Hepatitis B	Hepatitis B	1	Day 7			02/09/2010		Genitourinary	Normal		
S119	107	Hepatitis B	Hepatitis B	1	Day 7			02/09/2010		Dermatological	Normal		
S119	107	Hepatitis B	Hepatitis B	1	Day 14			09/09/2010		Head and Neck	Normal		
S119	107	Hepatitis B	Hepatitis B	1	Day 14			09/09/2010		Eyes	Normal		
S119	107	Hepatitis B	Hepatitis B	1	Day 14			09/09/2010		Ears, nose and throat	Normal		
S119	107	Hepatitis B	Hepatitis B	1	Day 14			09/09/2010		Cardiovascular	Normal		

Subject No.	Randomisation Number	Study Group	Dosage	Dose	Day	Not Done	Specify	Date of Assessment	*Was the assessment performed prior to vaccination?	Physical Examination Parameter	Result	Abnormality	Clinical Significance
S119	107	Hepatitis B	Hepatitis B	1	Day 14			09/09/2010		Respiratory	Normal		
S119	107	Hepatitis B	Hepatitis B	1	Day 14			09/09/2010		Musculoskeletal	Normal		
S119	107	Hepatitis B	Hepatitis B	1	Day 14			09/09/2010		Venous system	Normal		
S119	107	Hepatitis B	Hepatitis B	1	Day 14			09/09/2010		Gastrointestinal	Normal		
S119	107	Hepatitis B	Hepatitis B	1	Day 14			09/09/2010		Lymphatic	Normal		
S119	107	Hepatitis B	Hepatitis B	1	Day 14			09/09/2010		Neurological	Normal		
S119	107	Hepatitis B	Hepatitis B	1	Day 14			09/09/2010		Genitourinary	Normal		
S119	107	Hepatitis B	Hepatitis B	1	Day 14			09/09/2010		Dermatological	Normal		
S119	107	Hepatitis B	Hepatitis B	2	Day 28			23/09/2010	Yes	Head and Neck	Normal		
S119	107	Hepatitis B	Hepatitis B	2	Day 28			23/09/2010	Yes	Eyes	Normal		
S119	107	Hepatitis B	Hepatitis B	2	Day 28			23/09/2010	Yes	Ears, nose and throat	Normal		
S119	107	Hepatitis B	Hepatitis B	2	Day 28			23/09/2010	Yes	Cardiovascular	Normal		
S119	107	Hepatitis B	Hepatitis B	2	Day 28			23/09/2010	Yes	Respiratory	Normal		
S119	107	Hepatitis B	Hepatitis B	2	Day 28			23/09/2010	Yes	Musculoskeletal	Normal		
S119	107	Hepatitis B	Hepatitis B	2	Day 28			23/09/2010	Yes	Venous system	Normal		
S119	107	Hepatitis B	Hepatitis B	2	Day 28			23/09/2010	Yes	Gastrointestinal	Normal		
S119	107	Hepatitis B	Hepatitis B	2	Day 28			23/09/2010	Yes	Lymphatic	Normal		
S119	107	Hepatitis B	Hepatitis B	2	Day 28			23/09/2010	Yes	Neurological	Normal		
S119	107	Hepatitis B	Hepatitis B	2	Day 28			23/09/2010	Yes	Genitourinary	Normal		

Subject No.	Randomisation Number	Study Group	Dosage	Dose	Day	Not Done	Specify	Date of Assessment	*Was the assessment performed prior to vaccination?	Physical Examination Parameter	Result	Abnormality	Clinical Significance
S119	107	Hepatitis B	Hepatitis B	2	Day 28			23/09/2010	Yes	Dermatological	Normal		
S119	107	Hepatitis B	Hepatitis B	2	Day 35			29/09/2010		Head and Neck	Normal		
S119	107	Hepatitis B	Hepatitis B	2	Day 35			29/09/2010		Eyes	Normal		
S119	107	Hepatitis B	Hepatitis B	2	Day 35			29/09/2010		Ears, nose and throat	Normal		
S119	107	Hepatitis B	Hepatitis B	2	Day 35			29/09/2010		Cardiovascular	Normal		
S119	107	Hepatitis B	Hepatitis B	2	Day 35			29/09/2010		Respiratory	Normal		
S119	107	Hepatitis B	Hepatitis B	2	Day 35			29/09/2010		Musculoskeletal	Normal		
S119	107	Hepatitis B	Hepatitis B	2	Day 35			29/09/2010		Venous system	Normal		
S119	107	Hepatitis B	Hepatitis B	2	Day 35			29/09/2010		Gastrointestinal	Normal		
S119	107	Hepatitis B	Hepatitis B	2	Day 35			29/09/2010		Lymphatic	Normal		
S119	107	Hepatitis B	Hepatitis B	2	Day 35			29/09/2010		Neurological	Normal		
S119	107	Hepatitis B	Hepatitis B	2	Day 35			29/09/2010		Genitourinary	Normal		
S119	107	Hepatitis B	Hepatitis B	2	Day 35			29/09/2010		Dermatological	Normal		
S119	107	Hepatitis B	Hepatitis B	2	Day 42			06/10/2010		Head and Neck	Normal		
S119	107	Hepatitis B	Hepatitis B	2	Day 42			06/10/2010		Eyes	Normal		
S119	107	Hepatitis B	Hepatitis B	2	Day 42			06/10/2010		Ears, nose and throat	Normal		
S119	107	Hepatitis B	Hepatitis B	2	Day 42			06/10/2010		Cardiovascular	Normal		
S119	107	Hepatitis B	Hepatitis B	2	Day 42			06/10/2010		Respiratory	Normal		
S119	107	Hepatitis B	Hepatitis B	2	Day 42			06/10/2010		Musculoskeletal	Normal		

Subject No.	Randomisation Number	Study Group	Dosage	Dose	Day	Not Done	Specify	Date of Assessment	*Was the assessment performed prior to vaccination?	Physical Examination Parameter	Result	Abnormality	Clinical Significance
S119	107	Hepatitis B	Hepatitis B	2	Day 42			06/10/2010		Venous system	Normal		
S119	107	Hepatitis B	Hepatitis B	2	Day 42			06/10/2010		Gastrointestinal	Normal		
S119	107	Hepatitis B	Hepatitis B	2	Day 42			06/10/2010		Lymphatic	Normal		
S119	107	Hepatitis B	Hepatitis B	2	Day 42			06/10/2010		Neurological	Normal		
S119	107	Hepatitis B	Hepatitis B	2	Day 42			06/10/2010		Genitourinary	Normal		
S119	107	Hepatitis B	Hepatitis B	2	Day 42			06/10/2010		Dermatological	Normal		
S119	107	Hepatitis B	Hepatitis B	2	Day 56			21/10/2010		Head and Neck	Normal		
S119	107	Hepatitis B	Hepatitis B	2	Day 56			21/10/2010		Eyes	Normal		
S119	107	Hepatitis B	Hepatitis B	2	Day 56			21/10/2010		Ears, nose and throat	Normal		
S119	107	Hepatitis B	Hepatitis B	2	Day 56			21/10/2010		Cardiovascular	Normal		
S119	107	Hepatitis B	Hepatitis B	2	Day 56			21/10/2010		Respiratory	Normal		
S119	107	Hepatitis B	Hepatitis B	2	Day 56			21/10/2010		Musculoskeletal	Normal		
S119	107	Hepatitis B	Hepatitis B	2	Day 56			21/10/2010		Venous system	Normal		
S119	107	Hepatitis B	Hepatitis B	2	Day 56			21/10/2010		Gastrointestinal	Normal		
S119	107	Hepatitis B	Hepatitis B	2	Day 56			21/10/2010		Lymphatic	Normal		
S119	107	Hepatitis B	Hepatitis B	2	Day 56			21/10/2010		Neurological	Normal		
S119	107	Hepatitis B	Hepatitis B	2	Day 56			21/10/2010		Genitourinary	Normal		
S119	107	Hepatitis B	Hepatitis B	2	Day 56			21/10/2010		Dermatological	Normal		
S119	107	Hepatitis B	Hepatitis B	3	Day 180			22/02/2011	Yes	Head and Neck	Normal		

Subject No.	Randomisation Number	Study Group	Dosage	Dose	Day	Not Done	Specify	Date of Assessment	*Was the assessment performed prior to vaccination?	Physical Examination Parameter	Result	Abnormality	Clinical Significance
S119	107	Hepatitis B	Hepatitis B	3	Day 180			22/02/2011	Yes	Eyes	Normal		
S119	107	Hepatitis B	Hepatitis B	3	Day 180			22/02/2011	Yes	Ears, nose and throat	Normal		
S119	107	Hepatitis B	Hepatitis B	3	Day 180			22/02/2011	Yes	Cardiovascular	Normal		
S119	107	Hepatitis B	Hepatitis B	3	Day 180			22/02/2011	Yes	Respiratory	Normal		
S119	107	Hepatitis B	Hepatitis B	3	Day 180			22/02/2011	Yes	Musculoskeletal	Normal		
S119	107	Hepatitis B	Hepatitis B	3	Day 180			22/02/2011	Yes	Venous system	Normal		
S119	107	Hepatitis B	Hepatitis B	3	Day 180			22/02/2011	Yes	Gastrointestinal	Normal		
S119	107	Hepatitis B	Hepatitis B	3	Day 180			22/02/2011	Yes	Lymphatic	Normal		
S119	107	Hepatitis B	Hepatitis B	3	Day 180			22/02/2011	Yes	Neurological	Normal		
S119	107	Hepatitis B	Hepatitis B	3	Day 180			22/02/2011	Yes	Genitourinary	Normal		
S119	107	Hepatitis B	Hepatitis B	3	Day 180			22/02/2011	Yes	Dermatological	Normal		
S119	107	Hepatitis B	Hepatitis B	3	Day 187			02/03/2011		Head and Neck	Normal		
S119	107	Hepatitis B	Hepatitis B	3	Day 187			02/03/2011		Eyes	Normal		
S119	107	Hepatitis B	Hepatitis B	3	Day 187			02/03/2011		Ears, nose and throat	Normal		
S119	107	Hepatitis B	Hepatitis B	3	Day 187			02/03/2011		Cardiovascular	Normal		
S119	107	Hepatitis B	Hepatitis B	3	Day 187			02/03/2011		Respiratory	Normal		
S119	107	Hepatitis B	Hepatitis B	3	Day 187			02/03/2011		Musculoskeletal	Normal		
S119	107	Hepatitis B	Hepatitis B	3	Day 187			02/03/2011		Venous system	Normal		
S119	107	Hepatitis B	Hepatitis B	3	Day 187			02/03/2011		Gastrointestinal	Normal		

Subject No.	Randomisation Number	Study Group	Dosage	Dose	Day	Not Done	Specify	Date of Assessment	*Was the assessment performed prior to vaccination?	Physical Examination Parameter	Result	Abnormality	Clinical Significance
S119	107	Hepatitis B	Hepatitis B	3	Day 187			02/03/2011		Lymphatic	Normal		
S119	107	Hepatitis B	Hepatitis B	3	Day 187			02/03/2011		Neurological	Normal		
S119	107	Hepatitis B	Hepatitis B	3	Day 187			02/03/2011		Genitourinary	Normal		
S119	107	Hepatitis B	Hepatitis B	3	Day 187			02/03/2011		Dermatological	Normal		
S119	107	Hepatitis B	Hepatitis B	3	Day 194			08/03/2011		Head and Neck	Normal		
S119	107	Hepatitis B	Hepatitis B	3	Day 194			08/03/2011		Eyes	Normal		
S119	107	Hepatitis B	Hepatitis B	3	Day 194			08/03/2011		Ears, nose and throat	Normal		
S119	107	Hepatitis B	Hepatitis B	3	Day 194			08/03/2011		Cardiovascular	Normal		
S119	107	Hepatitis B	Hepatitis B	3	Day 194			08/03/2011		Respiratory	Normal		
S119	107	Hepatitis B	Hepatitis B	3	Day 194			08/03/2011		Musculoskeletal	Normal		
S119	107	Hepatitis B	Hepatitis B	3	Day 194			08/03/2011		Venous system	Normal		
S119	107	Hepatitis B	Hepatitis B	3	Day 194			08/03/2011		Gastrointestinal	Normal		
S119	107	Hepatitis B	Hepatitis B	3	Day 194			08/03/2011		Lymphatic	Normal		
S119	107	Hepatitis B	Hepatitis B	3	Day 194			08/03/2011		Neurological	Normal		
S119	107	Hepatitis B	Hepatitis B	3	Day 194			08/03/2011		Genitourinary	Normal		
S119	107	Hepatitis B	Hepatitis B	3	Day 194			08/03/2011		Dermatological	Normal		
S119	107	Hepatitis B	Hepatitis B	3	Day 208			22/03/2011		Head and Neck	Normal		
S119	107	Hepatitis B	Hepatitis B	3	Day 208			22/03/2011		Eyes	Normal		
S119	107	Hepatitis B	Hepatitis B	3	Day 208			22/03/2011		Ears, nose and throat	Normal		

Subject No.	Randomisation Number	Study Group	Dosage	Dose	Day	Not Done	Specify	Date of Assessment	*Was the assessment performed prior to vaccination?	Physical Examination Parameter	Result	Abnormality	Clinical Significance
S119	107	Hepatitis B	Hepatitis B	3	Day 208			22/03/2011		Cardiovascular	Normal		
S119	107	Hepatitis B	Hepatitis B	3	Day 208			22/03/2011		Respiratory	Normal		
S119	107	Hepatitis B	Hepatitis B	3	Day 208			22/03/2011		Musculoskeletal	Normal		
S119	107	Hepatitis B	Hepatitis B	3	Day 208			22/03/2011		Venous system	Normal		
S119	107	Hepatitis B	Hepatitis B	3	Day 208			22/03/2011		Gastrointestinal	Normal		
S119	107	Hepatitis B	Hepatitis B	3	Day 208			22/03/2011		Lymphatic	Normal		
S119	107	Hepatitis B	Hepatitis B	3	Day 208			22/03/2011		Neurological	Normal		
S119	107	Hepatitis B	Hepatitis B	3	Day 208			22/03/2011		Genitourinary	Normal		
S119	107	Hepatitis B	Hepatitis B	3	Day 208			22/03/2011		Dermatological	Normal		
S119	107	Hepatitis B	Hepatitis B	3	Day 365			25/08/2011		Head and Neck	Normal		
S119	107	Hepatitis B	Hepatitis B	3	Day 365			25/08/2011		Eyes	Normal		
S119	107	Hepatitis B	Hepatitis B	3	Day 365			25/08/2011		Ears, nose and throat	Normal		
S119	107	Hepatitis B	Hepatitis B	3	Day 365			25/08/2011		Cardiovascular	Normal		
S119	107	Hepatitis B	Hepatitis B	3	Day 365			25/08/2011		Respiratory	Normal		
S119	107	Hepatitis B	Hepatitis B	3	Day 365			25/08/2011		Musculoskeletal	Normal		
S119	107	Hepatitis B	Hepatitis B	3	Day 365			25/08/2011		Venous system	Normal		
S119	107	Hepatitis B	Hepatitis B	3	Day 365			25/08/2011		Gastrointestinal	Normal		
S119	107	Hepatitis B	Hepatitis B	3	Day 365			25/08/2011		Lymphatic	Normal		
S119	107	Hepatitis B	Hepatitis B	3	Day 365			25/08/2011		Neurological	Normal		

Subject No.	Randomisation Number	Study Group	Dosage	Dose	Day	Not Done	Specify	Date of Assessment	*Was the assessment performed prior to vaccination?	Physical Examination Parameter	Result	Abnormality	Clinical Significance
S119	107	Hepatitis B	Hepatitis B	3	Day 365			25/08/2011		Genitourinary	Normal		
S119	107	Hepatitis B	Hepatitis B	3	Day 365			25/08/2011		Dermatological	Normal		
S120	104	JAIVAC-1	Dosage A		Day -14 to Day -1			18/08/2010		Head and neck	Normal		
S120	104	JAIVAC-1	Dosage A		Day -14 to Day -1			18/08/2010		Eyes	Normal		
S120	104	JAIVAC-1	Dosage A		Day -14 to Day -1			18/08/2010		Ears, nose and throat	Normal		
S120	104	JAIVAC-1	Dosage A		Day -14 to Day -1			18/08/2010		Cardiovascular	Normal		
S120	104	JAIVAC-1	Dosage A		Day -14 to Day -1			18/08/2010		Respiratory	Normal		
S120	104	JAIVAC-1	Dosage A		Day -14 to Day -1			18/08/2010		Musculoskeletal	Normal		
S120	104	JAIVAC-1	Dosage A		Day -14 to Day -1			18/08/2010		Venous system	Normal		
S120	104	JAIVAC-1	Dosage A		Day -14 to Day -1			18/08/2010		Gastrointestinal	Normal		
S120	104	JAIVAC-1	Dosage A		Day -14 to Day -1			18/08/2010		Lymphatic	Normal		
S120	104	JAIVAC-1	Dosage A		Day -14 to Day -1			18/08/2010		Neurological	Normal		
S120	104	JAIVAC-1	Dosage A		Day -14 to Day -1			18/08/2010		Genitourinary	Normal		
S120	104	JAIVAC-1	Dosage A		Day -14 to Day -1			18/08/2010		Dermatological	Abnormal	Pityriasis Versicolor	NCS
S120	104	JAIVAC-1	Dosage A	1	Day 0			26/08/2010	Yes	Head and neck	Normal		
S120	104	JAIVAC-1	Dosage A	1	Day 0			26/08/2010	Yes	Eyes	Normal		
S120	104	JAIVAC-1	Dosage A	1	Day 0			26/08/2010	Yes	Ears, nose and throat	Normal		

Subject No.	Randomisation Number	Study Group	Dosage	Dose	Day	Not Done	Specify	Date of Assessment	*Was the assessment performed prior to vaccination?	Physical Examination Parameter	Result	Abnormality	Clinical Significance
S120	104	JAIVAC-1	Dosage A	1	Day 0			26/08/2010	Yes	Cardiovascular	Normal		
S120	104	JAIVAC-1	Dosage A	1	Day 0			26/08/2010	Yes	Respiratory	Normal		
S120	104	JAIVAC-1	Dosage A	1	Day 0			26/08/2010	Yes	Musculoskeletal	Normal		
S120	104	JAIVAC-1	Dosage A	1	Day 0			26/08/2010	Yes	Venous system	Normal		
S120	104	JAIVAC-1	Dosage A	1	Day 0			26/08/2010	Yes	Gastrointestinal	Normal		
S120	104	JAIVAC-1	Dosage A	1	Day 0			26/08/2010	Yes	Lymphatic	Normal		
S120	104	JAIVAC-1	Dosage A	1	Day 0			26/08/2010	Yes	Neurological	Normal		
S120	104	JAIVAC-1	Dosage A	1	Day 0			26/08/2010	Yes	Genitourinary	Normal		
S120	104	JAIVAC-1	Dosage A	1	Day 0			26/08/2010	Yes	Dermatological	Abnormal	Ptyriasis Versicolor Over Abdomen And Upper Arms	NCS
S120	104	JAIVAC-1	Dosage A	1	Day 7			02/09/2010		Head and Neck	Normal		
S120	104	JAIVAC-1	Dosage A	1	Day 7			02/09/2010		Eyes	Normal		
S120	104	JAIVAC-1	Dosage A	1	Day 7			02/09/2010		Ears, nose and throat	Normal		
S120	104	JAIVAC-1	Dosage A	1	Day 7			02/09/2010		Cardiovascular	Normal		
S120	104	JAIVAC-1	Dosage A	1	Day 7			02/09/2010		Respiratory	Normal		
S120	104	JAIVAC-1	Dosage A	1	Day 7			02/09/2010		Musculoskeletal	Normal		
S120	104	JAIVAC-1	Dosage A	1	Day 7			02/09/2010		Venous system	Normal		
S120	104	JAIVAC-1	Dosage A	1	Day 7			02/09/2010		Gastrointestinal	Normal		

Subject No.	Randomisation Number	Study Group	Dosage	Dose	Day	Not Done	Specify	Date of Assessment	*Was the assessment performed prior to vaccination?	Physical Examination Parameter	Result	Abnormality	Clinical Significance
S120	104	JAIVAC-1	Dosage A	1	Day 7			02/09/2010		Lymphatic	Normal		
S120	104	JAIVAC-1	Dosage A	1	Day 7			02/09/2010		Neurological	Normal		
S120	104	JAIVAC-1	Dosage A	1	Day 7			02/09/2010		Genitourinary	Normal		
S120	104	JAIVAC-1	Dosage A	1	Day 7			02/09/2010		Dermatological	Abnormal	Ptyriasis Versicolar Over Abdomen And Upper Arms	NCS
S120	104	JAIVAC-1	Dosage A	1	Day 14			08/09/2010		Head and Neck	Normal		
S120	104	JAIVAC-1	Dosage A	1	Day 14			08/09/2010		Eyes	Normal		
S120	104	JAIVAC-1	Dosage A	1	Day 14			08/09/2010		Ears, nose and throat	Normal		
S120	104	JAIVAC-1	Dosage A	1	Day 14			08/09/2010		Cardiovascular	Normal		
S120	104	JAIVAC-1	Dosage A	1	Day 14			08/09/2010		Respiratory	Normal		
S120	104	JAIVAC-1	Dosage A	1	Day 14			08/09/2010		Musculoskeletal	Normal		
S120	104	JAIVAC-1	Dosage A	1	Day 14			08/09/2010		Venous system	Normal		
S120	104	JAIVAC-1	Dosage A	1	Day 14			08/09/2010		Gastrointestinal	Normal		
S120	104	JAIVAC-1	Dosage A	1	Day 14			08/09/2010		Lymphatic	Normal		
S120	104	JAIVAC-1	Dosage A	1	Day 14			08/09/2010		Neurological	Normal		
S120	104	JAIVAC-1	Dosage A	1	Day 14			08/09/2010		Genitourinary	Normal		
S120	104	JAIVAC-1	Dosage A	1	Day 14			08/09/2010		Dermatological	Abnormal	Ptyriasis Versicolar Over Abdomen And Upper Arms	NCS

Subject No.	Randomisation Number	Study Group	Dosage	Dose	Day	Not Done	Specify	Date of Assessment	*Was the assessment performed prior to vaccination?	Physical Examination Parameter	Result	Abnormality	Clinical Significance
S120	104	JAIVAC-1	Dosage A	2	Day 28			23/09/2010	Yes	Head and Neck	Normal		
S120	104	JAIVAC-1	Dosage A	2	Day 28			23/09/2010	Yes	Eyes	Normal		
S120	104	JAIVAC-1	Dosage A	2	Day 28			23/09/2010	Yes	Ears, nose and throat	Normal		
S120	104	JAIVAC-1	Dosage A	2	Day 28			23/09/2010	Yes	Cardiovascular	Normal		
S120	104	JAIVAC-1	Dosage A	2	Day 28			23/09/2010	Yes	Respiratory	Normal		
S120	104	JAIVAC-1	Dosage A	2	Day 28			23/09/2010	Yes	Musculoskeletal	Normal		
S120	104	JAIVAC-1	Dosage A	2	Day 28			23/09/2010	Yes	Venous system	Normal		
S120	104	JAIVAC-1	Dosage A	2	Day 28			23/09/2010	Yes	Gastrointestinal	Normal		
S120	104	JAIVAC-1	Dosage A	2	Day 28			23/09/2010	Yes	Lymphatic	Normal		
S120	104	JAIVAC-1	Dosage A	2	Day 28			23/09/2010	Yes	Neurological	Normal		
S120	104	JAIVAC-1	Dosage A	2	Day 28			23/09/2010	Yes	Genitourinary	Normal		
S120	104	JAIVAC-1	Dosage A	2	Day 28			23/09/2010	Yes	Dermatological	Abnormal	Ptyriasis Versicolor Over Abdomen And Upper Arms	NCS
S120	104	JAIVAC-1	Dosage A	2	Day 35			30/09/2010		Head and Neck	Normal		
S120	104	JAIVAC-1	Dosage A	2	Day 35			30/09/2010		Eyes	Normal		
S120	104	JAIVAC-1	Dosage A	2	Day 35			30/09/2010		Ears, nose and throat	Normal		
S120	104	JAIVAC-1	Dosage A	2	Day 35			30/09/2010		Cardiovascular	Normal		
S120	104	JAIVAC-1	Dosage A	2	Day 35			30/09/2010		Respiratory	Normal		

Subject No.	Randomisation Number	Study Group	Dosage	Dose	Day	Not Done	Specify	Date of Assessment	*Was the assessment performed prior to vaccination?	Physical Examination Parameter	Result	Abnormality	Clinical Significance
S120	104	JAIVAC-1	Dosage A	2	Day 35			30/09/2010		Musculoskeletal	Normal		
S120	104	JAIVAC-1	Dosage A	2	Day 35			30/09/2010		Venous system	Normal		
S120	104	JAIVAC-1	Dosage A	2	Day 35			30/09/2010		Gastrointestinal	Normal		
S120	104	JAIVAC-1	Dosage A	2	Day 35			30/09/2010		Lymphatic	Normal		
S120	104	JAIVAC-1	Dosage A	2	Day 35			30/09/2010		Neurological	Normal		
S120	104	JAIVAC-1	Dosage A	2	Day 35			30/09/2010		Genitourinary	Normal		
S120	104	JAIVAC-1	Dosage A	2	Day 35			30/09/2010		Dermatological	Abnormal	Ptyriasis Versicolor Over Abdomen And Upper Arms	NCS
S120	104	JAIVAC-1	Dosage A	2	Day 42			07/10/2010		Head and Neck	Normal		
S120	104	JAIVAC-1	Dosage A	2	Day 42			07/10/2010		Eyes	Normal		
S120	104	JAIVAC-1	Dosage A	2	Day 42			07/10/2010		Ears, nose and throat	Normal		
S120	104	JAIVAC-1	Dosage A	2	Day 42			07/10/2010		Cardiovascular	Normal		
S120	104	JAIVAC-1	Dosage A	2	Day 42			07/10/2010		Respiratory	Normal		
S120	104	JAIVAC-1	Dosage A	2	Day 42			07/10/2010		Musculoskeletal	Normal		
S120	104	JAIVAC-1	Dosage A	2	Day 42			07/10/2010		Venous system	Normal		
S120	104	JAIVAC-1	Dosage A	2	Day 42			07/10/2010		Gastrointestinal	Normal		
S120	104	JAIVAC-1	Dosage A	2	Day 42			07/10/2010		Lymphatic	Normal		
S120	104	JAIVAC-1	Dosage A	2	Day 42			07/10/2010		Neurological	Normal		

Subject No.	Randomisation Number	Study Group	Dosage	Dose	Day	Not Done	Specify	Date of Assessment	*Was the assessment performed prior to vaccination?	Physical Examination Parameter	Result	Abnormality	Clinical Significance
S120	104	JAIVAC-1	Dosage A	2	Day 42			07/10/2010		Genitourinary	Normal		
S120	104	JAIVAC-1	Dosage A	2	Day 42			07/10/2010		Dermatological	Abnormal	Ptyriasis Versicolor Over Abdomen And Upper Arms	NCS
S120	104	JAIVAC-1	Dosage A	2	Day 56			21/10/2010		Head and Neck	Normal		
S120	104	JAIVAC-1	Dosage A	2	Day 56			21/10/2010		Eyes	Normal		
S120	104	JAIVAC-1	Dosage A	2	Day 56			21/10/2010		Ears, nose and throat	Normal		
S120	104	JAIVAC-1	Dosage A	2	Day 56			21/10/2010		Cardiovascular	Normal		
S120	104	JAIVAC-1	Dosage A	2	Day 56			21/10/2010		Respiratory	Normal		
S120	104	JAIVAC-1	Dosage A	2	Day 56			21/10/2010		Musculoskeletal	Normal		
S120	104	JAIVAC-1	Dosage A	2	Day 56			21/10/2010		Venous system	Normal		
S120	104	JAIVAC-1	Dosage A	2	Day 56			21/10/2010		Gastrointestinal	Normal		
S120	104	JAIVAC-1	Dosage A	2	Day 56			21/10/2010		Lymphatic	Normal		
S120	104	JAIVAC-1	Dosage A	2	Day 56			21/10/2010		Neurological	Normal		
S120	104	JAIVAC-1	Dosage A	2	Day 56			21/10/2010		Genitourinary	Normal		
S120	104	JAIVAC-1	Dosage A	2	Day 56			21/10/2010		Dermatological	Abnormal	Ptyriasis Versicolor Over Abdomen And Upper Arms	NCS
S120	104	JAIVAC-1	Dosage A	3	Day 180			22/02/2011	Yes	Head and Neck	Normal		

Subject No.	Randomisation Number	Study Group	Dosage	Dose	Day	Not Done	Specify	Date of Assessment	*Was the assessment performed prior to vaccination?	Physical Examination Parameter	Result	Abnormality	Clinical Significance
S120	104	JAIVAC-1	Dosage A	3	Day 180			22/02/2011	Yes	Eyes	Normal		
S120	104	JAIVAC-1	Dosage A	3	Day 180			22/02/2011	Yes	Ears, nose and throat	Normal		
S120	104	JAIVAC-1	Dosage A	3	Day 180			22/02/2011	Yes	Cardiovascular	Normal		
S120	104	JAIVAC-1	Dosage A	3	Day 180			22/02/2011	Yes	Respiratory	Normal		
S120	104	JAIVAC-1	Dosage A	3	Day 180			22/02/2011	Yes	Musculoskeletal	Normal		
S120	104	JAIVAC-1	Dosage A	3	Day 180			22/02/2011	Yes	Venous system	Normal		
S120	104	JAIVAC-1	Dosage A	3	Day 180			22/02/2011	Yes	Gastrointestinal	Normal		
S120	104	JAIVAC-1	Dosage A	3	Day 180			22/02/2011	Yes	Lymphatic	Normal		
S120	104	JAIVAC-1	Dosage A	3	Day 180			22/02/2011	Yes	Neurological	Normal		
S120	104	JAIVAC-1	Dosage A	3	Day 180			22/02/2011	Yes	Genitourinary	Normal		
S120	104	JAIVAC-1	Dosage A	3	Day 180			22/02/2011	Yes	Dermatological	Abnormal	Ptyriasis Versicolor Over Abdomen And Upper Arms	NCS
S120	104	JAIVAC-1	Dosage A	3	Day 187			01/03/2011		Head and Neck	Normal		
S120	104	JAIVAC-1	Dosage A	3	Day 187			01/03/2011		Eyes	Normal		
S120	104	JAIVAC-1	Dosage A	3	Day 187			01/03/2011		Ears, nose and throat	Normal		
S120	104	JAIVAC-1	Dosage A	3	Day 187			01/03/2011		Cardiovascular	Normal		
S120	104	JAIVAC-1	Dosage A	3	Day 187			01/03/2011		Respiratory	Normal		
S120	104	JAIVAC-1	Dosage A	3	Day 187			01/03/2011		Musculoskeletal	Normal		

Subject No.	Randomisation Number	Study Group	Dosage	Dose	Day	Not Done	Specify	Date of Assessment	*Was the assessment performed prior to vaccination?	Physical Examination Parameter	Result	Abnormality	Clinical Significance
S120	104	JAIVAC-1	Dosage A	3	Day 187			01/03/2011		Venous system	Normal		
S120	104	JAIVAC-1	Dosage A	3	Day 187			01/03/2011		Gastrointestinal	Normal		
S120	104	JAIVAC-1	Dosage A	3	Day 187			01/03/2011		Lymphatic	Normal		
S120	104	JAIVAC-1	Dosage A	3	Day 187			01/03/2011		Neurological	Normal		
S120	104	JAIVAC-1	Dosage A	3	Day 187			01/03/2011		Genitourinary	Normal		
S120	104	JAIVAC-1	Dosage A	3	Day 187			01/03/2011		Dermatological	Abnormal	Ptyriasis Versicolor Over Abdomen And Upper Arms	NCS
S120	104	JAIVAC-1	Dosage A	3	Day 194			08/03/2011		Head and Neck	Normal		
S120	104	JAIVAC-1	Dosage A	3	Day 194			08/03/2011		Eyes	Normal		
S120	104	JAIVAC-1	Dosage A	3	Day 194			08/03/2011		Ears, nose and throat	Normal		
S120	104	JAIVAC-1	Dosage A	3	Day 194			08/03/2011		Cardiovascular	Normal		
S120	104	JAIVAC-1	Dosage A	3	Day 194			08/03/2011		Respiratory	Normal		
S120	104	JAIVAC-1	Dosage A	3	Day 194			08/03/2011		Musculoskeletal	Normal		
S120	104	JAIVAC-1	Dosage A	3	Day 194			08/03/2011		Venous system	Normal		
S120	104	JAIVAC-1	Dosage A	3	Day 194			08/03/2011		Gastrointestinal	Normal		
S120	104	JAIVAC-1	Dosage A	3	Day 194			08/03/2011		Lymphatic	Normal		
S120	104	JAIVAC-1	Dosage A	3	Day 194			08/03/2011		Neurological	Normal		
S120	104	JAIVAC-1	Dosage A	3	Day 194			08/03/2011		Genitourinary	Normal		

Subject No.	Randomisation Number	Study Group	Dosage	Dose	Day	Not Done	Specify	Date of Assessment	*Was the assessment performed prior to vaccination?	Physical Examination Parameter	Result	Abnormality	Clinical Significance
S120	104	JAIVAC-1	Dosage A	3	Day 194			08/03/2011		Dermatological	Abnormal	Ptyriasis Versicolor Over Abdomen And Upper Arms	NCS
S120	104	JAIVAC-1	Dosage A	3	Day 208			22/03/2011		Head and Neck	Normal		
S120	104	JAIVAC-1	Dosage A	3	Day 208			22/03/2011		Eyes	Normal		
S120	104	JAIVAC-1	Dosage A	3	Day 208			22/03/2011		Ears, nose and throat	Normal		
S120	104	JAIVAC-1	Dosage A	3	Day 208			22/03/2011		Cardiovascular	Normal		
S120	104	JAIVAC-1	Dosage A	3	Day 208			22/03/2011		Respiratory	Normal		
S120	104	JAIVAC-1	Dosage A	3	Day 208			22/03/2011		Musculoskeletal	Normal		
S120	104	JAIVAC-1	Dosage A	3	Day 208			22/03/2011		Venous system	Normal		
S120	104	JAIVAC-1	Dosage A	3	Day 208			22/03/2011		Gastrointestinal	Normal		
S120	104	JAIVAC-1	Dosage A	3	Day 208			22/03/2011		Lymphatic	Normal		
S120	104	JAIVAC-1	Dosage A	3	Day 208			22/03/2011		Neurological	Normal		
S120	104	JAIVAC-1	Dosage A	3	Day 208			22/03/2011		Genitourinary	Normal		
S120	104	JAIVAC-1	Dosage A	3	Day 208			22/03/2011		Dermatological	Abnormal	Ptyriasis Versicolor Over Abdomen And Upper Arms	NCS
S120	104	JAIVAC-1	Dosage A	3	Day 365			25/08/2011		Head and Neck	Normal		
S120	104	JAIVAC-1	Dosage A	3	Day 365			25/08/2011		Eyes	Normal		

Subject No.	Randomisation Number	Study Group	Dosage	Dose	Day	Not Done	Specify	Date of Assessment	*Was the assessment performed prior to vaccination?	Physical Examination Parameter	Result	Abnormality	Clinical Significance
S120	104	JAIVAC-1	Dosage A	3	Day 365			25/08/2011		Ears, nose and throat	Normal		
S120	104	JAIVAC-1	Dosage A	3	Day 365			25/08/2011		Cardiovascular	Normal		
S120	104	JAIVAC-1	Dosage A	3	Day 365			25/08/2011		Respiratory	Normal		
S120	104	JAIVAC-1	Dosage A	3	Day 365			25/08/2011		Musculoskeletal	Normal		
S120	104	JAIVAC-1	Dosage A	3	Day 365			25/08/2011		Venous system	Normal		
S120	104	JAIVAC-1	Dosage A	3	Day 365			25/08/2011		Gastrointestinal	Normal		
S120	104	JAIVAC-1	Dosage A	3	Day 365			25/08/2011		Lymphatic	Normal		
S120	104	JAIVAC-1	Dosage A	3	Day 365			25/08/2011		Neurological	Normal		
S120	104	JAIVAC-1	Dosage A	3	Day 365			25/08/2011		Genitourinary	Normal		
S120	104	JAIVAC-1	Dosage A	3	Day 365			25/08/2011		Dermatological	Abnormal	Ptyriasis Versicolor Abdomen & Upper Arms	NCS
S120	104	JAIVAC-1	Dosage A		Unscheduled Visit	Yes	Laboratory Repeat At Local Lab	.		Head and Neck			
S120	104	JAIVAC-1	Dosage A		Unscheduled Visit	Yes	Laboratory Repeat At Local Lab	.		Eyes			
S120	104	JAIVAC-1	Dosage A		Unscheduled Visit	Yes	Laboratory Repeat At Local Lab	.		Ears, nose and throat			
S120	104	JAIVAC-1	Dosage A		Unscheduled Visit	Yes	Laboratory Repeat At Local Lab	.		Cardiovascular			
S120	104	JAIVAC-1	Dosage A		Unscheduled Visit	Yes	Laboratory Repeat At Local Lab	.		Respiratory			

Subject No.	Randomisation Number	Study Group	Dosage	Dose	Day	Not Done	Specify	Date of Assessment	*Was the assessment performed prior to vaccination?	Physical Examination Parameter	Result	Abnormality	Clinical Significance
S120	104	JAIVAC-1	Dosage A		Unscheduled Visit	Yes	Laboratory Repeat At Local Lab	.		Musculoskeletal			
S120	104	JAIVAC-1	Dosage A		Unscheduled Visit	Yes	Laboratory Repeat At Local Lab	.		Venous system			
S120	104	JAIVAC-1	Dosage A		Unscheduled Visit	Yes	Laboratory Repeat At Local Lab	.		Gastrointestinal			
S120	104	JAIVAC-1	Dosage A		Unscheduled Visit	Yes	Laboratory Repeat At Local Lab	.		Lymphatic			
S120	104	JAIVAC-1	Dosage A		Unscheduled Visit	Yes	Laboratory Repeat At Local Lab	.		Neurological			
S120	104	JAIVAC-1	Dosage A		Unscheduled Visit	Yes	Laboratory Repeat At Local Lab	.		Genitourinary			
S120	104	JAIVAC-1	Dosage A		Unscheduled Visit	Yes	Laboratory Repeat At Local Lab	.		Dermatological			
S124	106	Hepatitis B	Hepatitis B		Day -14 to Day -1			18/08/2010		Head and neck	Normal		
S124	106	Hepatitis B	Hepatitis B		Day -14 to Day -1			18/08/2010		Eyes	Normal		
S124	106	Hepatitis B	Hepatitis B		Day -14 to Day -1			18/08/2010		Ears, nose and throat	Normal		
S124	106	Hepatitis B	Hepatitis B		Day -14 to Day -1			18/08/2010		Cardiovascular	Normal		
S124	106	Hepatitis B	Hepatitis B		Day -14 to Day -1			18/08/2010		Respiratory	Normal		
S124	106	Hepatitis B	Hepatitis B		Day -14 to Day -1			18/08/2010		Musculoskeletal	Normal		
S124	106	Hepatitis B	Hepatitis B		Day -14 to Day -1			18/08/2010		Venous system	Normal		

Subject No.	Randomisation Number	Study Group	Dosage	Dose	Day	Not Done	Specify	Date of Assessment	*Was the assessment performed prior to vaccination?	Physical Examination Parameter	Result	Abnormality	Clinical Significance
S124	106	Hepatitis B	Hepatitis B		Day -14 to Day -1			18/08/2010		Gastrointestinal	Normal		
S124	106	Hepatitis B	Hepatitis B		Day -14 to Day -1			18/08/2010		Lymphatic	Normal		
S124	106	Hepatitis B	Hepatitis B		Day -14 to Day -1			18/08/2010		Neurological	Normal		
S124	106	Hepatitis B	Hepatitis B		Day -14 to Day -1			18/08/2010		Genitourinary	Normal		
S124	106	Hepatitis B	Hepatitis B		Day -14 to Day -1			18/08/2010		Dermatological	Normal		
S124	106	Hepatitis B	Hepatitis B	1	Day 0			26/08/2010	Yes	Head and neck	Normal		
S124	106	Hepatitis B	Hepatitis B	1	Day 0			26/08/2010	Yes	Eyes	Normal		
S124	106	Hepatitis B	Hepatitis B	1	Day 0			26/08/2010	Yes	Ears, nose and throat	Normal		
S124	106	Hepatitis B	Hepatitis B	1	Day 0			26/08/2010	Yes	Cardiovascular	Normal		
S124	106	Hepatitis B	Hepatitis B	1	Day 0			26/08/2010	Yes	Respiratory	Normal		
S124	106	Hepatitis B	Hepatitis B	1	Day 0			26/08/2010	Yes	Musculoskeletal	Normal		
S124	106	Hepatitis B	Hepatitis B	1	Day 0			26/08/2010	Yes	Venous system	Normal		
S124	106	Hepatitis B	Hepatitis B	1	Day 0			26/08/2010	Yes	Gastrointestinal	Normal		
S124	106	Hepatitis B	Hepatitis B	1	Day 0			26/08/2010	Yes	Lymphatic	Normal		
S124	106	Hepatitis B	Hepatitis B	1	Day 0			26/08/2010	Yes	Neurological	Normal		
S124	106	Hepatitis B	Hepatitis B	1	Day 0			26/08/2010	Yes	Genitourinary	Normal		
S124	106	Hepatitis B	Hepatitis B	1	Day 0			26/08/2010	Yes	Dermatological	Normal		
S124	106	Hepatitis B	Hepatitis B	1	Day 7			02/09/2010		Head and Neck	Normal		

Subject No.	Randomisation Number	Study Group	Dosage	Dose	Day	Not Done	Specify	Date of Assessment	*Was the assessment performed prior to vaccination?	Physical Examination Parameter	Result	Abnormality	Clinical Significance
S124	106	Hepatitis B	Hepatitis B	1	Day 7			02/09/2010		Eyes	Normal		
S124	106	Hepatitis B	Hepatitis B	1	Day 7			02/09/2010		Ears, nose and throat	Normal		
S124	106	Hepatitis B	Hepatitis B	1	Day 7			02/09/2010		Cardiovascular	Normal		
S124	106	Hepatitis B	Hepatitis B	1	Day 7			02/09/2010		Respiratory	Normal		
S124	106	Hepatitis B	Hepatitis B	1	Day 7			02/09/2010		Musculoskeletal	Normal		
S124	106	Hepatitis B	Hepatitis B	1	Day 7			02/09/2010		Venous system	Normal		
S124	106	Hepatitis B	Hepatitis B	1	Day 7			02/09/2010		Gastrointestinal	Normal		
S124	106	Hepatitis B	Hepatitis B	1	Day 7			02/09/2010		Lymphatic	Normal		
S124	106	Hepatitis B	Hepatitis B	1	Day 7			02/09/2010		Neurological	Normal		
S124	106	Hepatitis B	Hepatitis B	1	Day 7			02/09/2010		Genitourinary	Normal		
S124	106	Hepatitis B	Hepatitis B	1	Day 7			02/09/2010		Dermatological	Normal		
S124	106	Hepatitis B	Hepatitis B	1	Day 14			08/09/2010		Head and Neck	Normal		
S124	106	Hepatitis B	Hepatitis B	1	Day 14			08/09/2010		Eyes	Normal		
S124	106	Hepatitis B	Hepatitis B	1	Day 14			08/09/2010		Ears, nose and throat	Normal		
S124	106	Hepatitis B	Hepatitis B	1	Day 14			08/09/2010		Cardiovascular	Normal		
S124	106	Hepatitis B	Hepatitis B	1	Day 14			08/09/2010		Respiratory	Normal		
S124	106	Hepatitis B	Hepatitis B	1	Day 14			08/09/2010		Musculoskeletal	Normal		
S124	106	Hepatitis B	Hepatitis B	1	Day 14			08/09/2010		Venous system	Normal		
S124	106	Hepatitis B	Hepatitis B	1	Day 14			08/09/2010		Gastrointestinal	Normal		

Subject No.	Randomisation Number	Study Group	Dosage	Dose	Day	Not Done	Specify	Date of Assessment	*Was the assessment performed prior to vaccination?	Physical Examination Parameter	Result	Abnormality	Clinical Significance
S124	106	Hepatitis B	Hepatitis B	1	Day 14			08/09/2010		Lymphatic	Normal		
S124	106	Hepatitis B	Hepatitis B	1	Day 14			08/09/2010		Neurological	Normal		
S124	106	Hepatitis B	Hepatitis B	1	Day 14			08/09/2010		Genitourinary	Normal		
S124	106	Hepatitis B	Hepatitis B	1	Day 14			08/09/2010		Dermatological	Normal		
S124	106	Hepatitis B	Hepatitis B	2	Day 28			23/09/2010	Yes	Head and Neck	Normal		
S124	106	Hepatitis B	Hepatitis B	2	Day 28			23/09/2010	Yes	Eyes	Normal		
S124	106	Hepatitis B	Hepatitis B	2	Day 28			23/09/2010	Yes	Ears, nose and throat	Normal		
S124	106	Hepatitis B	Hepatitis B	2	Day 28			23/09/2010	Yes	Cardiovascular	Normal		
S124	106	Hepatitis B	Hepatitis B	2	Day 28			23/09/2010	Yes	Respiratory	Normal		
S124	106	Hepatitis B	Hepatitis B	2	Day 28			23/09/2010	Yes	Musculoskeletal	Normal		
S124	106	Hepatitis B	Hepatitis B	2	Day 28			23/09/2010	Yes	Venous system	Normal		
S124	106	Hepatitis B	Hepatitis B	2	Day 28			23/09/2010	Yes	Gastrointestinal	Normal		
S124	106	Hepatitis B	Hepatitis B	2	Day 28			23/09/2010	Yes	Lymphatic	Normal		
S124	106	Hepatitis B	Hepatitis B	2	Day 28			23/09/2010	Yes	Neurological	Normal		
S124	106	Hepatitis B	Hepatitis B	2	Day 28			23/09/2010	Yes	Genitourinary	Normal		
S124	106	Hepatitis B	Hepatitis B	2	Day 28			23/09/2010	Yes	Dermatological	Normal		
S124	106	Hepatitis B	Hepatitis B	2	Day 35			29/09/2010		Head and Neck	Normal		
S124	106	Hepatitis B	Hepatitis B	2	Day 35			29/09/2010		Eyes	Normal		
S124	106	Hepatitis B	Hepatitis B	2	Day 35			29/09/2010		Ears, nose and throat	Normal		

Subject No.	Randomisation Number	Study Group	Dosage	Dose	Day	Not Done	Specify	Date of Assessment	*Was the assessment performed prior to vaccination?	Physical Examination Parameter	Result	Abnormality	Clinical Significance
S124	106	Hepatitis B	Hepatitis B	2	Day 35			29/09/2010		Cardiovascular	Normal		
S124	106	Hepatitis B	Hepatitis B	2	Day 35			29/09/2010		Respiratory	Normal		
S124	106	Hepatitis B	Hepatitis B	2	Day 35			29/09/2010		Musculoskeletal	Normal		
S124	106	Hepatitis B	Hepatitis B	2	Day 35			29/09/2010		Venous system	Normal		
S124	106	Hepatitis B	Hepatitis B	2	Day 35			29/09/2010		Gastrointestinal	Normal		
S124	106	Hepatitis B	Hepatitis B	2	Day 35			29/09/2010		Lymphatic	Normal		
S124	106	Hepatitis B	Hepatitis B	2	Day 35			29/09/2010		Neurological	Normal		
S124	106	Hepatitis B	Hepatitis B	2	Day 35			29/09/2010		Genitourinary	Normal		
S124	106	Hepatitis B	Hepatitis B	2	Day 35			29/09/2010		Dermatological	Normal		
S124	106	Hepatitis B	Hepatitis B	2	Day 42			07/10/2010		Head and Neck	Normal		
S124	106	Hepatitis B	Hepatitis B	2	Day 42			07/10/2010		Eyes	Normal		
S124	106	Hepatitis B	Hepatitis B	2	Day 42			07/10/2010		Ears, nose and throat	Normal		
S124	106	Hepatitis B	Hepatitis B	2	Day 42			07/10/2010		Cardiovascular	Normal		
S124	106	Hepatitis B	Hepatitis B	2	Day 42			07/10/2010		Respiratory	Normal		
S124	106	Hepatitis B	Hepatitis B	2	Day 42			07/10/2010		Musculoskeletal	Normal		
S124	106	Hepatitis B	Hepatitis B	2	Day 42			07/10/2010		Venous system	Normal		
S124	106	Hepatitis B	Hepatitis B	2	Day 42			07/10/2010		Gastrointestinal	Normal		
S124	106	Hepatitis B	Hepatitis B	2	Day 42			07/10/2010		Lymphatic	Normal		
S124	106	Hepatitis B	Hepatitis B	2	Day 42			07/10/2010		Neurological	Normal		

Subject No.	Randomisation Number	Study Group	Dosage	Dose	Day	Not Done	Specify	Date of Assessment	*Was the assessment performed prior to vaccination?	Physical Examination Parameter	Result	Abnormality	Clinical Significance
S124	106	Hepatitis B	Hepatitis B	2	Day 42			07/10/2010		Genitourinary	Normal		
S124	106	Hepatitis B	Hepatitis B	2	Day 42			07/10/2010		Dermatological	Normal		
S124	106	Hepatitis B	Hepatitis B	2	Day 56			21/10/2010		Head and Neck	Normal		
S124	106	Hepatitis B	Hepatitis B	2	Day 56			21/10/2010		Eyes	Normal		
S124	106	Hepatitis B	Hepatitis B	2	Day 56			21/10/2010		Ears, nose and throat	Normal		
S124	106	Hepatitis B	Hepatitis B	2	Day 56			21/10/2010		Cardiovascular	Normal		
S124	106	Hepatitis B	Hepatitis B	2	Day 56			21/10/2010		Respiratory	Normal		
S124	106	Hepatitis B	Hepatitis B	2	Day 56			21/10/2010		Musculoskeletal	Normal		
S124	106	Hepatitis B	Hepatitis B	2	Day 56			21/10/2010		Venous system	Normal		
S124	106	Hepatitis B	Hepatitis B	2	Day 56			21/10/2010		Gastrointestinal	Normal		
S124	106	Hepatitis B	Hepatitis B	2	Day 56			21/10/2010		Lymphatic	Normal		
S124	106	Hepatitis B	Hepatitis B	2	Day 56			21/10/2010		Neurological	Normal		
S124	106	Hepatitis B	Hepatitis B	2	Day 56			21/10/2010		Genitourinary	Normal		
S124	106	Hepatitis B	Hepatitis B	2	Day 56			21/10/2010		Dermatological	Normal		
S124	106	Hepatitis B	Hepatitis B	3	Day 180			22/02/2011	Yes	Head and Neck	Normal		
S124	106	Hepatitis B	Hepatitis B	3	Day 180			22/02/2011	Yes	Eyes	Normal		
S124	106	Hepatitis B	Hepatitis B	3	Day 180			22/02/2011	Yes	Ears, nose and throat	Normal		
S124	106	Hepatitis B	Hepatitis B	3	Day 180			22/02/2011	Yes	Cardiovascular	Normal		
S124	106	Hepatitis B	Hepatitis B	3	Day 180			22/02/2011	Yes	Respiratory	Normal		

Subject No.	Randomisation Number	Study Group	Dosage	Dose	Day	Not Done	Specify	Date of Assessment	*Was the assessment performed prior to vaccination?	Physical Examination Parameter	Result	Abnormality	Clinical Significance
S124	106	Hepatitis B	Hepatitis B	3	Day 180			22/02/2011	Yes	Musculoskeletal	Normal		
S124	106	Hepatitis B	Hepatitis B	3	Day 180			22/02/2011	Yes	Venous system	Normal		
S124	106	Hepatitis B	Hepatitis B	3	Day 180			22/02/2011	Yes	Gastrointestinal	Normal		
S124	106	Hepatitis B	Hepatitis B	3	Day 180			22/02/2011	Yes	Lymphatic	Normal		
S124	106	Hepatitis B	Hepatitis B	3	Day 180			22/02/2011	Yes	Neurological	Normal		
S124	106	Hepatitis B	Hepatitis B	3	Day 180			22/02/2011	Yes	Genitourinary	Normal		
S124	106	Hepatitis B	Hepatitis B	3	Day 180			22/02/2011	Yes	Dermatological	Normal		
S124	106	Hepatitis B	Hepatitis B	3	Day 187			01/03/2011		Head and Neck	Normal		
S124	106	Hepatitis B	Hepatitis B	3	Day 187			01/03/2011		Eyes	Normal		
S124	106	Hepatitis B	Hepatitis B	3	Day 187			01/03/2011		Ears, nose and throat	Normal		
S124	106	Hepatitis B	Hepatitis B	3	Day 187			01/03/2011		Cardiovascular	Normal		
S124	106	Hepatitis B	Hepatitis B	3	Day 187			01/03/2011		Respiratory	Normal		
S124	106	Hepatitis B	Hepatitis B	3	Day 187			01/03/2011		Musculoskeletal	Normal		
S124	106	Hepatitis B	Hepatitis B	3	Day 187			01/03/2011		Venous system	Normal		
S124	106	Hepatitis B	Hepatitis B	3	Day 187			01/03/2011		Gastrointestinal	Normal		
S124	106	Hepatitis B	Hepatitis B	3	Day 187			01/03/2011		Lymphatic	Normal		
S124	106	Hepatitis B	Hepatitis B	3	Day 187			01/03/2011		Neurological	Normal		
S124	106	Hepatitis B	Hepatitis B	3	Day 187			01/03/2011		Genitourinary	Normal		
S124	106	Hepatitis B	Hepatitis B	3	Day 187			01/03/2011		Dermatological	Normal		

Subject No.	Randomisation Number	Study Group	Dosage	Dose	Day	Not Done	Specify	Date of Assessment	*Was the assessment performed prior to vaccination?	Physical Examination Parameter	Result	Abnormality	Clinical Significance
S124	106	Hepatitis B	Hepatitis B	3	Day 194			08/03/2011		Head and Neck	Normal		
S124	106	Hepatitis B	Hepatitis B	3	Day 194			08/03/2011		Eyes	Normal		
S124	106	Hepatitis B	Hepatitis B	3	Day 194			08/03/2011		Ears, nose and throat	Normal		
S124	106	Hepatitis B	Hepatitis B	3	Day 194			08/03/2011		Cardiovascular	Normal		
S124	106	Hepatitis B	Hepatitis B	3	Day 194			08/03/2011		Respiratory	Normal		
S124	106	Hepatitis B	Hepatitis B	3	Day 194			08/03/2011		Musculoskeletal	Normal		
S124	106	Hepatitis B	Hepatitis B	3	Day 194			08/03/2011		Venous system	Normal		
S124	106	Hepatitis B	Hepatitis B	3	Day 194			08/03/2011		Gastrointestinal	Normal		
S124	106	Hepatitis B	Hepatitis B	3	Day 194			08/03/2011		Lymphatic	Normal		
S124	106	Hepatitis B	Hepatitis B	3	Day 194			08/03/2011		Neurological	Normal		
S124	106	Hepatitis B	Hepatitis B	3	Day 194			08/03/2011		Genitourinary	Normal		
S124	106	Hepatitis B	Hepatitis B	3	Day 194			08/03/2011		Dermatological	Normal		
S124	106	Hepatitis B	Hepatitis B	3	Day 208			22/03/2011		Head and Neck	Normal		
S124	106	Hepatitis B	Hepatitis B	3	Day 208			22/03/2011		Eyes	Normal		
S124	106	Hepatitis B	Hepatitis B	3	Day 208			22/03/2011		Ears, nose and throat	Normal		
S124	106	Hepatitis B	Hepatitis B	3	Day 208			22/03/2011		Cardiovascular	Normal		
S124	106	Hepatitis B	Hepatitis B	3	Day 208			22/03/2011		Respiratory	Normal		
S124	106	Hepatitis B	Hepatitis B	3	Day 208			22/03/2011		Musculoskeletal	Normal		
S124	106	Hepatitis B	Hepatitis B	3	Day 208			22/03/2011		Venous system	Normal		

Subject No.	Randomisation Number	Study Group	Dosage	Dose	Day	Not Done	Specify	Date of Assessment	*Was the assessment performed prior to vaccination?	Physical Examination Parameter	Result	Abnormality	Clinical Significance
S124	106	Hepatitis B	Hepatitis B	3	Day 208			22/03/2011		Gastrointestinal	Normal		
S124	106	Hepatitis B	Hepatitis B	3	Day 208			22/03/2011		Lymphatic	Normal		
S124	106	Hepatitis B	Hepatitis B	3	Day 208			22/03/2011		Neurological	Normal		
S124	106	Hepatitis B	Hepatitis B	3	Day 208			22/03/2011		Genitourinary	Normal		
S124	106	Hepatitis B	Hepatitis B	3	Day 208			22/03/2011		Dermatological	Normal		
S124	106	Hepatitis B	Hepatitis B	3	Day 365			25/08/2011		Head and Neck	Normal		
S124	106	Hepatitis B	Hepatitis B	3	Day 365			25/08/2011		Eyes	Normal		
S124	106	Hepatitis B	Hepatitis B	3	Day 365			25/08/2011		Ears, nose and throat	Normal		
S124	106	Hepatitis B	Hepatitis B	3	Day 365			25/08/2011		Cardiovascular	Normal		
S124	106	Hepatitis B	Hepatitis B	3	Day 365			25/08/2011		Respiratory	Normal		
S124	106	Hepatitis B	Hepatitis B	3	Day 365			25/08/2011		Musculoskeletal	Normal		
S124	106	Hepatitis B	Hepatitis B	3	Day 365			25/08/2011		Venous system	Normal		
S124	106	Hepatitis B	Hepatitis B	3	Day 365			25/08/2011		Gastrointestinal	Normal		
S124	106	Hepatitis B	Hepatitis B	3	Day 365			25/08/2011		Lymphatic	Normal		
S124	106	Hepatitis B	Hepatitis B	3	Day 365			25/08/2011		Neurological	Normal		
S124	106	Hepatitis B	Hepatitis B	3	Day 365			25/08/2011		Genitourinary	Normal		
S124	106	Hepatitis B	Hepatitis B	3	Day 365			25/08/2011		Dermatological	Normal		
S124	106	Hepatitis B	Hepatitis B		Unscheduled Visit	Yes	Only Local Lab Parameters Done	.		Head and Neck			

Subject No.	Randomisation Number	Study Group	Dosage	Dose	Day	Not Done	Specify	Date of Assessment	*Was the assessment performed prior to vaccination?	Physical Examination Parameter	Result	Abnormality	Clinical Significance
S124	106	Hepatitis B	Hepatitis B		Unscheduled Visit	Yes	Only Local Lab Parameters Done	.		Eyes			
S124	106	Hepatitis B	Hepatitis B		Unscheduled Visit	Yes	Only Local Lab Parameters Done	.		Ears, nose and throat			
S124	106	Hepatitis B	Hepatitis B		Unscheduled Visit	Yes	Only Local Lab Parameters Done	.		Cardiovascular			
S124	106	Hepatitis B	Hepatitis B		Unscheduled Visit	Yes	Only Local Lab Parameters Done	.		Respiratory			
S124	106	Hepatitis B	Hepatitis B		Unscheduled Visit	Yes	Only Local Lab Parameters Done	.		Musculoskeletal			
S124	106	Hepatitis B	Hepatitis B		Unscheduled Visit	Yes	Only Local Lab Parameters Done	.		Venous system			
S124	106	Hepatitis B	Hepatitis B		Unscheduled Visit	Yes	Only Local Lab Parameters Done	.		Gastrointestinal			
S124	106	Hepatitis B	Hepatitis B		Unscheduled Visit	Yes	Only Local Lab Parameters Done	.		Lymphatic			
S124	106	Hepatitis B	Hepatitis B		Unscheduled Visit	Yes	Only Local Lab Parameters Done	.		Neurological			

Subject No.	Randomisation Number	Study Group	Dosage	Dose	Day	Not Done	Specify	Date of Assessment	*Was the assessment performed prior to vaccination?	Physical Examination Parameter	Result	Abnormality	Clinical Significance
S124	106	Hepatitis B	Hepatitis B		Unscheduled Visit	Yes	Only Local Lab Parameters Done	.		Genitourinary			
S124	106	Hepatitis B	Hepatitis B		Unscheduled Visit	Yes	Only Local Lab Parameters Done	.		Dermatological			
S124	106	Hepatitis B	Hepatitis B		Unscheduled Visit	Yes	Prescribing Medication For The Ae	.		Head and Neck			
S124	106	Hepatitis B	Hepatitis B		Unscheduled Visit	Yes	Prescribing Medication For The Ae	.		Eyes			
S124	106	Hepatitis B	Hepatitis B		Unscheduled Visit	Yes	Prescribing Medication For The Ae	.		Ears, nose and throat			
S124	106	Hepatitis B	Hepatitis B		Unscheduled Visit	Yes	Prescribing Medication For The Ae	.		Cardiovascular			
S124	106	Hepatitis B	Hepatitis B		Unscheduled Visit	Yes	Prescribing Medication For The Ae	.		Respiratory			
S124	106	Hepatitis B	Hepatitis B		Unscheduled Visit	Yes	Prescribing Medication For The Ae	.		Musculoskeletal			
S124	106	Hepatitis B	Hepatitis B		Unscheduled Visit	Yes	Prescribing Medication For The Ae	.		Venous system			
S124	106	Hepatitis B	Hepatitis B		Unscheduled Visit	Yes	Prescribing Medication For The Ae	.		Gastrointestinal			
S124	106	Hepatitis B	Hepatitis B		Unscheduled Visit	Yes	Prescribing Medication For The Ae	.		Lymphatic			

Subject No.	Randomisation Number	Study Group	Dosage	Dose	Day	Not Done	Specify	Date of Assessment	*Was the assessment performed prior to vaccination?	Physical Examination Parameter	Result	Abnormality	Clinical Significance
S124	106	Hepatitis B	Hepatitis B		Unscheduled Visit	Yes	Prescribing Medication For The Ae	.		Neurological			
S124	106	Hepatitis B	Hepatitis B		Unscheduled Visit	Yes	Prescribing Medication For The Ae	.		Genitourinary			
S124	106	Hepatitis B	Hepatitis B		Unscheduled Visit	Yes	Prescribing Medication For The Ae	.		Dermatological			
S124	106	Hepatitis B	Hepatitis B		Unscheduled Visit	Yes	To Repeat Laboratory Parameters In Local Lab	.		Head and Neck			
S124	106	Hepatitis B	Hepatitis B		Unscheduled Visit	Yes	To Repeat Laboratory Parameters In Local Lab	.		Eyes			
S124	106	Hepatitis B	Hepatitis B		Unscheduled Visit	Yes	To Repeat Laboratory Parameters In Local Lab	.		Ears, nose and throat			
S124	106	Hepatitis B	Hepatitis B		Unscheduled Visit	Yes	To Repeat Laboratory Parameters In Local Lab	.		Cardiovascular			
S124	106	Hepatitis B	Hepatitis B		Unscheduled Visit	Yes	To Repeat Laboratory Parameters In Local Lab	.		Respiratory			
S124	106	Hepatitis B	Hepatitis B		Unscheduled Visit	Yes	To Repeat Laboratory Parameters In Local Lab	.		Musculoskeletal			
S124	106	Hepatitis B	Hepatitis B		Unscheduled Visit	Yes	To Repeat Laboratory Parameters In Local Lab	.		Venous system			

Subject No.	Randomisation Number	Study Group	Dosage	Dose	Day	Not Done	Specify	Date of Assessment	*Was the assessment performed prior to vaccination ?	Physical Examination Parameter	Result	Abnormality	Clinical Significance
S124	106	Hepatitis B	Hepatitis B		Unscheduled Visit	Yes	To Repeat Laboratory Parameters In Local Lab	.		Gastrointestinal			
S124	106	Hepatitis B	Hepatitis B		Unscheduled Visit	Yes	To Repeat Laboratory Parameters In Local Lab	.		Lymphatic			
S124	106	Hepatitis B	Hepatitis B		Unscheduled Visit	Yes	To Repeat Laboratory Parameters In Local Lab	.		Neurological			
S124	106	Hepatitis B	Hepatitis B		Unscheduled Visit	Yes	To Repeat Laboratory Parameters In Local Lab	.		Genitourinary			
S124	106	Hepatitis B	Hepatitis B		Unscheduled Visit	Yes	To Repeat Laboratory Parameters In Local Lab	.		Dermatological			
S125	102	JAIVAC-1	Dosage A		Day -14 to Day -1			18/08/2010		Head and neck	Normal		
S125	102	JAIVAC-1	Dosage A		Day -14 to Day -1			18/08/2010		Eyes	Normal		
S125	102	JAIVAC-1	Dosage A		Day -14 to Day -1			18/08/2010		Ears, nose and throat	Normal		
S125	102	JAIVAC-1	Dosage A		Day -14 to Day -1			18/08/2010		Cardiovascular	Normal		
S125	102	JAIVAC-1	Dosage A		Day -14 to Day -1			18/08/2010		Respiratory	Normal		
S125	102	JAIVAC-1	Dosage A		Day -14 to Day -1			18/08/2010		Musculoskeletal	Normal		
S125	102	JAIVAC-1	Dosage A		Day -14 to Day -1			18/08/2010		Venous system	Normal		
S125	102	JAIVAC-1	Dosage A		Day -14 to Day -1			18/08/2010		Gastrointestinal	Normal		

Subject No.	Randomisation Number	Study Group	Dosage	Dose	Day	Not Done	Specify	Date of Assessment	*Was the assessment performed prior to vaccination?	Physical Examination Parameter	Result	Abnormality	Clinical Significance
S125	102	JAIVAC-1	Dosage A		Day -14 to Day -1			18/08/2010		Lymphatic	Normal		
S125	102	JAIVAC-1	Dosage A		Day -14 to Day -1			18/08/2010		Neurological	Normal		
S125	102	JAIVAC-1	Dosage A		Day -14 to Day -1			18/08/2010		Genitourinary	Normal		
S125	102	JAIVAC-1	Dosage A		Day -14 to Day -1			18/08/2010		Dermatological	Normal		
S125	102	JAIVAC-1	Dosage A	1	Day 0			24/08/2010	Yes	Head and neck	Normal		
S125	102	JAIVAC-1	Dosage A	1	Day 0			24/08/2010	Yes	Eyes	Normal		
S125	102	JAIVAC-1	Dosage A	1	Day 0			24/08/2010	Yes	Ears, nose and throat	Normal		
S125	102	JAIVAC-1	Dosage A	1	Day 0			24/08/2010	Yes	Cardiovascular	Normal		
S125	102	JAIVAC-1	Dosage A	1	Day 0			24/08/2010	Yes	Respiratory	Normal		
S125	102	JAIVAC-1	Dosage A	1	Day 0			24/08/2010	Yes	Musculoskeletal	Normal		
S125	102	JAIVAC-1	Dosage A	1	Day 0			24/08/2010	Yes	Venous system	Normal		
S125	102	JAIVAC-1	Dosage A	1	Day 0			24/08/2010	Yes	Gastrointestinal	Normal		
S125	102	JAIVAC-1	Dosage A	1	Day 0			24/08/2010	Yes	Lymphatic	Normal		
S125	102	JAIVAC-1	Dosage A	1	Day 0			24/08/2010	Yes	Neurological	Normal		
S125	102	JAIVAC-1	Dosage A	1	Day 0			24/08/2010	Yes	Genitourinary	Normal		
S125	102	JAIVAC-1	Dosage A	1	Day 0			24/08/2010	Yes	Dermatological	Normal		
S125	102	JAIVAC-1	Dosage A	1	Day 7			31/08/2010		Head and Neck	Normal		
S125	102	JAIVAC-1	Dosage A	1	Day 7			31/08/2010		Eyes	Normal		

Subject No.	Randomisation Number	Study Group	Dosage	Dose	Day	Not Done	Specify	Date of Assessment	*Was the assessment performed prior to vaccination?	Physical Examination Parameter	Result	Abnormality	Clinical Significance
S125	102	JAIVAC-1	Dosage A	1	Day 7			31/08/2010		Ears, nose and throat	Normal		
S125	102	JAIVAC-1	Dosage A	1	Day 7			31/08/2010		Cardiovascular	Normal		
S125	102	JAIVAC-1	Dosage A	1	Day 7			31/08/2010		Respiratory	Normal		
S125	102	JAIVAC-1	Dosage A	1	Day 7			31/08/2010		Musculoskeletal	Normal		
S125	102	JAIVAC-1	Dosage A	1	Day 7			31/08/2010		Venous system	Normal		
S125	102	JAIVAC-1	Dosage A	1	Day 7			31/08/2010		Gastrointestinal	Normal		
S125	102	JAIVAC-1	Dosage A	1	Day 7			31/08/2010		Lymphatic	Normal		
S125	102	JAIVAC-1	Dosage A	1	Day 7			31/08/2010		Neurological	Normal		
S125	102	JAIVAC-1	Dosage A	1	Day 7			31/08/2010		Genitourinary	Normal		
S125	102	JAIVAC-1	Dosage A	1	Day 7			31/08/2010		Dermatological	Normal		
S125	102	JAIVAC-1	Dosage A	1	Day 14			07/09/2010		Head and Neck	Normal		
S125	102	JAIVAC-1	Dosage A	1	Day 14			07/09/2010		Eyes	Normal		
S125	102	JAIVAC-1	Dosage A	1	Day 14			07/09/2010		Ears, nose and throat	Normal		
S125	102	JAIVAC-1	Dosage A	1	Day 14			07/09/2010		Cardiovascular	Normal		
S125	102	JAIVAC-1	Dosage A	1	Day 14			07/09/2010		Respiratory	Normal		
S125	102	JAIVAC-1	Dosage A	1	Day 14			07/09/2010		Musculoskeletal	Normal		
S125	102	JAIVAC-1	Dosage A	1	Day 14			07/09/2010		Venous system	Normal		
S125	102	JAIVAC-1	Dosage A	1	Day 14			07/09/2010		Gastrointestinal	Normal		
S125	102	JAIVAC-1	Dosage A	1	Day 14			07/09/2010		Lymphatic	Normal		

Subject No.	Randomisation Number	Study Group	Dosage	Dose	Day	Not Done	Specify	Date of Assessment	*Was the assessment performed prior to vaccination?	Physical Examination Parameter	Result	Abnormality	Clinical Significance
S125	102	JAIVAC-1	Dosage A	1	Day 14			07/09/2010		Neurological	Normal		
S125	102	JAIVAC-1	Dosage A	1	Day 14			07/09/2010		Genitourinary	Normal		
S125	102	JAIVAC-1	Dosage A	1	Day 14			07/09/2010		Dermatological	Normal		
S125	102	JAIVAC-1	Dosage A	2	Day 28			21/09/2010	Yes	Head and Neck	Normal		
S125	102	JAIVAC-1	Dosage A	2	Day 28			21/09/2010	Yes	Eyes	Normal		
S125	102	JAIVAC-1	Dosage A	2	Day 28			21/09/2010	Yes	Ears, nose and throat	Normal		
S125	102	JAIVAC-1	Dosage A	2	Day 28			21/09/2010	Yes	Cardiovascular	Normal		
S125	102	JAIVAC-1	Dosage A	2	Day 28			21/09/2010	Yes	Respiratory	Normal		
S125	102	JAIVAC-1	Dosage A	2	Day 28			21/09/2010	Yes	Musculoskeletal	Normal		
S125	102	JAIVAC-1	Dosage A	2	Day 28			21/09/2010	Yes	Venous system	Normal		
S125	102	JAIVAC-1	Dosage A	2	Day 28			21/09/2010	Yes	Gastrointestinal	Normal		
S125	102	JAIVAC-1	Dosage A	2	Day 28			21/09/2010	Yes	Lymphatic	Normal		
S125	102	JAIVAC-1	Dosage A	2	Day 28			21/09/2010	Yes	Neurological	Normal		
S125	102	JAIVAC-1	Dosage A	2	Day 28			21/09/2010	Yes	Genitourinary	Normal		
S125	102	JAIVAC-1	Dosage A	2	Day 28			21/09/2010	Yes	Dermatological	Normal		
S125	102	JAIVAC-1	Dosage A	2	Day 35			28/09/2010		Head and Neck	Normal		
S125	102	JAIVAC-1	Dosage A	2	Day 35			28/09/2010		Eyes	Normal		
S125	102	JAIVAC-1	Dosage A	2	Day 35			28/09/2010		Ears, nose and throat	Normal		
S125	102	JAIVAC-1	Dosage A	2	Day 35			28/09/2010		Cardiovascular	Normal		

Subject No.	Randomisation Number	Study Group	Dosage	Dose	Day	Not Done	Specify	Date of Assessment	*Was the assessment performed prior to vaccination?	Physical Examination Parameter	Result	Abnormality	Clinical Significance
S125	102	JAIVAC-1	Dosage A	2	Day 35			28/09/2010		Respiratory	Normal		
S125	102	JAIVAC-1	Dosage A	2	Day 35			28/09/2010		Musculoskeletal	Normal		
S125	102	JAIVAC-1	Dosage A	2	Day 35			28/09/2010		Venous system	Normal		
S125	102	JAIVAC-1	Dosage A	2	Day 35			28/09/2010		Gastrointestinal	Normal		
S125	102	JAIVAC-1	Dosage A	2	Day 35			28/09/2010		Lymphatic	Normal		
S125	102	JAIVAC-1	Dosage A	2	Day 35			28/09/2010		Neurological	Normal		
S125	102	JAIVAC-1	Dosage A	2	Day 35			28/09/2010		Genitourinary	Normal		
S125	102	JAIVAC-1	Dosage A	2	Day 35			28/09/2010		Dermatological	Normal		
S125	102	JAIVAC-1	Dosage A	2	Day 42			05/10/2010		Head and Neck	Normal		
S125	102	JAIVAC-1	Dosage A	2	Day 42			05/10/2010		Eyes	Normal		
S125	102	JAIVAC-1	Dosage A	2	Day 42			05/10/2010		Ears, nose and throat	Normal		
S125	102	JAIVAC-1	Dosage A	2	Day 42			05/10/2010		Cardiovascular	Normal		
S125	102	JAIVAC-1	Dosage A	2	Day 42			05/10/2010		Respiratory	Normal		
S125	102	JAIVAC-1	Dosage A	2	Day 42			05/10/2010		Musculoskeletal	Normal		
S125	102	JAIVAC-1	Dosage A	2	Day 42			05/10/2010		Venous system	Normal		
S125	102	JAIVAC-1	Dosage A	2	Day 42			05/10/2010		Gastrointestinal	Normal		
S125	102	JAIVAC-1	Dosage A	2	Day 42			05/10/2010		Lymphatic	Normal		
S125	102	JAIVAC-1	Dosage A	2	Day 42			05/10/2010		Neurological	Normal		
S125	102	JAIVAC-1	Dosage A	2	Day 42			05/10/2010		Genitourinary	Normal		

Subject No.	Randomisation Number	Study Group	Dosage	Dose	Day	Not Done	Specify	Date of Assessment	*Was the assessment performed prior to vaccination?	Physical Examination Parameter	Result	Abnormality	Clinical Significance
S125	102	JAIVAC-1	Dosage A	2	Day 42			05/10/2010		Dermatological	Normal		
S125	102	JAIVAC-1	Dosage A	2	Day 56			19/10/2010		Head and Neck	Normal		
S125	102	JAIVAC-1	Dosage A	2	Day 56			19/10/2010		Eyes	Normal		
S125	102	JAIVAC-1	Dosage A	2	Day 56			19/10/2010		Ears, nose and throat	Normal		
S125	102	JAIVAC-1	Dosage A	2	Day 56			19/10/2010		Cardiovascular	Normal		
S125	102	JAIVAC-1	Dosage A	2	Day 56			19/10/2010		Respiratory	Normal		
S125	102	JAIVAC-1	Dosage A	2	Day 56			19/10/2010		Musculoskeletal	Normal		
S125	102	JAIVAC-1	Dosage A	2	Day 56			19/10/2010		Venous system	Normal		
S125	102	JAIVAC-1	Dosage A	2	Day 56			19/10/2010		Gastrointestinal	Normal		
S125	102	JAIVAC-1	Dosage A	2	Day 56			19/10/2010		Lymphatic	Normal		
S125	102	JAIVAC-1	Dosage A	2	Day 56			19/10/2010		Neurological	Normal		
S125	102	JAIVAC-1	Dosage A	2	Day 56			19/10/2010		Genitourinary	Normal		
S125	102	JAIVAC-1	Dosage A	2	Day 56			19/10/2010		Dermatological	Normal		
S125	102	JAIVAC-1	Dosage A	3	Day 180			21/02/2011	Yes	Head and Neck	Normal		
S125	102	JAIVAC-1	Dosage A	3	Day 180			21/02/2011	Yes	Eyes	Normal		
S125	102	JAIVAC-1	Dosage A	3	Day 180			21/02/2011	Yes	Ears, nose and throat	Normal		
S125	102	JAIVAC-1	Dosage A	3	Day 180			21/02/2011	Yes	Cardiovascular	Normal		
S125	102	JAIVAC-1	Dosage A	3	Day 180			21/02/2011	Yes	Respiratory	Normal		
S125	102	JAIVAC-1	Dosage A	3	Day 180			21/02/2011	Yes	Musculoskeletal	Normal		

Subject No.	Randomisation Number	Study Group	Dosage	Dose	Day	Not Done	Specify	Date of Assessment	*Was the assessment performed prior to vaccination?	Physical Examination Parameter	Result	Abnormality	Clinical Significance
S125	102	JAIVAC-1	Dosage A	3	Day 180			21/02/2011	Yes	Venous system	Normal		
S125	102	JAIVAC-1	Dosage A	3	Day 180			21/02/2011	Yes	Gastrointestinal	Normal		
S125	102	JAIVAC-1	Dosage A	3	Day 180			21/02/2011	Yes	Lymphatic	Normal		
S125	102	JAIVAC-1	Dosage A	3	Day 180			21/02/2011	Yes	Neurological	Normal		
S125	102	JAIVAC-1	Dosage A	3	Day 180			21/02/2011	Yes	Genitourinary	Normal		
S125	102	JAIVAC-1	Dosage A	3	Day 180			21/02/2011	Yes	Dermatological	Normal		
S125	102	JAIVAC-1	Dosage A	3	Day 187			28/02/2011		Head and Neck	Normal		
S125	102	JAIVAC-1	Dosage A	3	Day 187			28/02/2011		Eyes	Normal		
S125	102	JAIVAC-1	Dosage A	3	Day 187			28/02/2011		Ears, nose and throat	Normal		
S125	102	JAIVAC-1	Dosage A	3	Day 187			28/02/2011		Cardiovascular	Normal		
S125	102	JAIVAC-1	Dosage A	3	Day 187			28/02/2011		Respiratory	Normal		
S125	102	JAIVAC-1	Dosage A	3	Day 187			28/02/2011		Musculoskeletal	Normal		
S125	102	JAIVAC-1	Dosage A	3	Day 187			28/02/2011		Venous system	Normal		
S125	102	JAIVAC-1	Dosage A	3	Day 187			28/02/2011		Gastrointestinal	Normal		
S125	102	JAIVAC-1	Dosage A	3	Day 187			28/02/2011		Lymphatic	Normal		
S125	102	JAIVAC-1	Dosage A	3	Day 187			28/02/2011		Neurological	Normal		
S125	102	JAIVAC-1	Dosage A	3	Day 187			28/02/2011		Genitourinary	Normal		
S125	102	JAIVAC-1	Dosage A	3	Day 187			28/02/2011		Dermatological	Normal		
S125	102	JAIVAC-1	Dosage A	3	Day 194			07/03/2011		Head and Neck	Normal		

Subject No.	Randomisation Number	Study Group	Dosage	Dose	Day	Not Done	Specify	Date of Assessment	*Was the assessment performed prior to vaccination?	Physical Examination Parameter	Result	Abnormality	Clinical Significance
S125	102	JAIVAC-1	Dosage A	3	Day 194			07/03/2011		Eyes	Normal		
S125	102	JAIVAC-1	Dosage A	3	Day 194			07/03/2011		Ears, nose and throat	Normal		
S125	102	JAIVAC-1	Dosage A	3	Day 194			07/03/2011		Cardiovascular	Normal		
S125	102	JAIVAC-1	Dosage A	3	Day 194			07/03/2011		Respiratory	Normal		
S125	102	JAIVAC-1	Dosage A	3	Day 194			07/03/2011		Musculoskeletal	Normal		
S125	102	JAIVAC-1	Dosage A	3	Day 194			07/03/2011		Venous system	Normal		
S125	102	JAIVAC-1	Dosage A	3	Day 194			07/03/2011		Gastrointestinal	Normal		
S125	102	JAIVAC-1	Dosage A	3	Day 194			07/03/2011		Lymphatic	Normal		
S125	102	JAIVAC-1	Dosage A	3	Day 194			07/03/2011		Neurological	Normal		
S125	102	JAIVAC-1	Dosage A	3	Day 194			07/03/2011		Genitourinary	Normal		
S125	102	JAIVAC-1	Dosage A	3	Day 194			07/03/2011		Dermatological	Normal		
S125	102	JAIVAC-1	Dosage A	3	Day 208			21/03/2011		Head and Neck	Normal		
S125	102	JAIVAC-1	Dosage A	3	Day 208			21/03/2011		Eyes	Normal		
S125	102	JAIVAC-1	Dosage A	3	Day 208			21/03/2011		Ears, nose and throat	Normal		
S125	102	JAIVAC-1	Dosage A	3	Day 208			21/03/2011		Cardiovascular	Normal		
S125	102	JAIVAC-1	Dosage A	3	Day 208			21/03/2011		Respiratory	Normal		
S125	102	JAIVAC-1	Dosage A	3	Day 208			21/03/2011		Musculoskeletal	Normal		
S125	102	JAIVAC-1	Dosage A	3	Day 208			21/03/2011		Venous system	Normal		
S125	102	JAIVAC-1	Dosage A	3	Day 208			21/03/2011		Gastrointestinal	Normal		

Subject No.	Randomisation Number	Study Group	Dosage	Dose	Day	Not Done	Specify	Date of Assessment	*Was the assessment performed prior to vaccination?	Physical Examination Parameter	Result	Abnormality	Clinical Significance
S125	102	JAIVAC-1	Dosage A	3	Day 208			21/03/2011		Lymphatic	Normal		
S125	102	JAIVAC-1	Dosage A	3	Day 208			21/03/2011		Neurological	Normal		
S125	102	JAIVAC-1	Dosage A	3	Day 208			21/03/2011		Genitourinary	Normal		
S125	102	JAIVAC-1	Dosage A	3	Day 208			21/03/2011		Dermatological	Normal		
S125	102	JAIVAC-1	Dosage A	3	Day 365			26/08/2011		Head and Neck	Normal		
S125	102	JAIVAC-1	Dosage A	3	Day 365			26/08/2011		Eyes	Normal		
S125	102	JAIVAC-1	Dosage A	3	Day 365			26/08/2011		Ears, nose and throat	Normal		
S125	102	JAIVAC-1	Dosage A	3	Day 365			26/08/2011		Cardiovascular	Normal		
S125	102	JAIVAC-1	Dosage A	3	Day 365			26/08/2011		Respiratory	Normal		
S125	102	JAIVAC-1	Dosage A	3	Day 365			26/08/2011		Musculoskeletal	Normal		
S125	102	JAIVAC-1	Dosage A	3	Day 365			26/08/2011		Venous system	Normal		
S125	102	JAIVAC-1	Dosage A	3	Day 365			26/08/2011		Gastrointestinal	Normal		
S125	102	JAIVAC-1	Dosage A	3	Day 365			26/08/2011		Lymphatic	Normal		
S125	102	JAIVAC-1	Dosage A	3	Day 365			26/08/2011		Neurological	Normal		
S125	102	JAIVAC-1	Dosage A	3	Day 365			26/08/2011		Genitourinary	Normal		
S125	102	JAIVAC-1	Dosage A	3	Day 365			26/08/2011		Dermatological	Normal		
S125	102	JAIVAC-1	Dosage A		Unscheduled Visit	Yes	Laboratory Repeat	.		Head and Neck			
S125	102	JAIVAC-1	Dosage A		Unscheduled Visit	Yes	Laboratory Repeat	.		Eyes			
S125	102	JAIVAC-1	Dosage A		Unscheduled Visit	Yes	Laboratory Repeat	.		Ears, nose and throat			

Subject No.	Randomisation Number	Study Group	Dosage	Dose	Day	Not Done	Specify	Date of Assessment	*Was the assessment performed prior to vaccination?	Physical Examination Parameter	Result	Abnormality	Clinical Significance
S125	102	JAIVAC-1	Dosage A		Unscheduled Visit	Yes	Laboratory Repeat	.		Cardiovascular			
S125	102	JAIVAC-1	Dosage A		Unscheduled Visit	Yes	Laboratory Repeat	.		Respiratory			
S125	102	JAIVAC-1	Dosage A		Unscheduled Visit	Yes	Laboratory Repeat	.		Musculoskeletal			
S125	102	JAIVAC-1	Dosage A		Unscheduled Visit	Yes	Laboratory Repeat	.		Venous system			
S125	102	JAIVAC-1	Dosage A		Unscheduled Visit	Yes	Laboratory Repeat	.		Gastrointestinal			
S125	102	JAIVAC-1	Dosage A		Unscheduled Visit	Yes	Laboratory Repeat	.		Lymphatic			
S125	102	JAIVAC-1	Dosage A		Unscheduled Visit	Yes	Laboratory Repeat	.		Neurological			
S125	102	JAIVAC-1	Dosage A		Unscheduled Visit	Yes	Laboratory Repeat	.		Genitourinary			
S125	102	JAIVAC-1	Dosage A		Unscheduled Visit	Yes	Laboratory Repeat	.		Dermatological			
S125	102	JAIVAC-1	Dosage A		Unscheduled Visit	Yes	Laboratory Repeat At Local Lab	.		Head and Neck			
S125	102	JAIVAC-1	Dosage A		Unscheduled Visit	Yes	Laboratory Repeat At Local Lab	.		Eyes			
S125	102	JAIVAC-1	Dosage A		Unscheduled Visit	Yes	Laboratory Repeat At Local Lab	.		Ears, nose and throat			
S125	102	JAIVAC-1	Dosage A		Unscheduled Visit	Yes	Laboratory Repeat At Local Lab	.		Cardiovascular			
S125	102	JAIVAC-1	Dosage A		Unscheduled Visit	Yes	Laboratory Repeat At Local Lab	.		Respiratory			

Subject No.	Randomisation Number	Study Group	Dosage	Dose	Day	Not Done	Specify	Date of Assessment	*Was the assessment performed prior to vaccination?	Physical Examination Parameter	Result	Abnormality	Clinical Significance
S125	102	JAIVAC-1	Dosage A		Unscheduled Visit	Yes	Laboratory Repeat At Local Lab	.		Musculoskeletal			
S125	102	JAIVAC-1	Dosage A		Unscheduled Visit	Yes	Laboratory Repeat At Local Lab	.		Venous system			
S125	102	JAIVAC-1	Dosage A		Unscheduled Visit	Yes	Laboratory Repeat At Local Lab	.		Gastrointestinal			
S125	102	JAIVAC-1	Dosage A		Unscheduled Visit	Yes	Laboratory Repeat At Local Lab	.		Lymphatic			
S125	102	JAIVAC-1	Dosage A		Unscheduled Visit	Yes	Laboratory Repeat At Local Lab	.		Neurological			
S125	102	JAIVAC-1	Dosage A		Unscheduled Visit	Yes	Laboratory Repeat At Local Lab	.		Genitourinary			
S125	102	JAIVAC-1	Dosage A		Unscheduled Visit	Yes	Laboratory Repeat At Local Lab	.		Dermatological			
S130	105	JAIVAC-1	Dosage A		Day -14 to Day -1			17/08/2010		Head and neck	Normal		
S130	105	JAIVAC-1	Dosage A		Day -14 to Day -1			17/08/2010		Eyes	Normal		
S130	105	JAIVAC-1	Dosage A		Day -14 to Day -1			17/08/2010		Ears, nose and throat	Normal		
S130	105	JAIVAC-1	Dosage A		Day -14 to Day -1			17/08/2010		Cardiovascular	Normal		
S130	105	JAIVAC-1	Dosage A		Day -14 to Day -1			17/08/2010		Respiratory	Normal		
S130	105	JAIVAC-1	Dosage A		Day -14 to Day -1			17/08/2010		Musculoskeletal	Normal		
S130	105	JAIVAC-1	Dosage A		Day -14 to Day -1			17/08/2010		Venous system	Normal		

Subject No.	Randomisation Number	Study Group	Dosage	Dose	Day	Not Done	Specify	Date of Assessment	*Was the assessment performed prior to vaccination?	Physical Examination Parameter	Result	Abnormality	Clinical Significance
S130	105	JAIVAC-1	Dosage A		Day -14 to Day -1			17/08/2010		Gastrointestinal	Normal		
S130	105	JAIVAC-1	Dosage A		Day -14 to Day -1			17/08/2010		Lymphatic	Normal		
S130	105	JAIVAC-1	Dosage A		Day -14 to Day -1			17/08/2010		Neurological	Normal		
S130	105	JAIVAC-1	Dosage A		Day -14 to Day -1			17/08/2010		Genitourinary	Normal		
S130	105	JAIVAC-1	Dosage A		Day -14 to Day -1			17/08/2010		Dermatological	Normal		
S130	105	JAIVAC-1	Dosage A	1	Day 0			26/08/2010	Yes	Head and neck	Normal		
S130	105	JAIVAC-1	Dosage A	1	Day 0			26/08/2010	Yes	Eyes	Normal		
S130	105	JAIVAC-1	Dosage A	1	Day 0			26/08/2010	Yes	Ears, nose and throat	Normal		
S130	105	JAIVAC-1	Dosage A	1	Day 0			26/08/2010	Yes	Cardiovascular	Normal		
S130	105	JAIVAC-1	Dosage A	1	Day 0			26/08/2010	Yes	Respiratory	Normal		
S130	105	JAIVAC-1	Dosage A	1	Day 0			26/08/2010	Yes	Musculoskeletal	Normal		
S130	105	JAIVAC-1	Dosage A	1	Day 0			26/08/2010	Yes	Venous system	Normal		
S130	105	JAIVAC-1	Dosage A	1	Day 0			26/08/2010	Yes	Gastrointestinal	Normal		
S130	105	JAIVAC-1	Dosage A	1	Day 0			26/08/2010	Yes	Lymphatic	Normal		
S130	105	JAIVAC-1	Dosage A	1	Day 0			26/08/2010	Yes	Neurological	Normal		
S130	105	JAIVAC-1	Dosage A	1	Day 0			26/08/2010	Yes	Genitourinary	Normal		
S130	105	JAIVAC-1	Dosage A	1	Day 0			26/08/2010	Yes	Dermatological	Normal		
S130	105	JAIVAC-1	Dosage A	1	Day 7			02/09/2010		Head and Neck	Normal		

Subject No.	Randomisation Number	Study Group	Dosage	Dose	Day	Not Done	Specify	Date of Assessment	*Was the assessment performed prior to vaccination?	Physical Examination Parameter	Result	Abnormality	Clinical Significance
S130	105	JAIVAC-1	Dosage A	1	Day 7			02/09/2010		Eyes	Normal		
S130	105	JAIVAC-1	Dosage A	1	Day 7			02/09/2010		Ears, nose and throat	Normal		
S130	105	JAIVAC-1	Dosage A	1	Day 7			02/09/2010		Cardiovascular	Normal		
S130	105	JAIVAC-1	Dosage A	1	Day 7			02/09/2010		Respiratory	Normal		
S130	105	JAIVAC-1	Dosage A	1	Day 7			02/09/2010		Musculoskeletal	Normal		
S130	105	JAIVAC-1	Dosage A	1	Day 7			02/09/2010		Venous system	Normal		
S130	105	JAIVAC-1	Dosage A	1	Day 7			02/09/2010		Gastrointestinal	Normal		
S130	105	JAIVAC-1	Dosage A	1	Day 7			02/09/2010		Lymphatic	Normal		
S130	105	JAIVAC-1	Dosage A	1	Day 7			02/09/2010		Neurological	Normal		
S130	105	JAIVAC-1	Dosage A	1	Day 7			02/09/2010		Genitourinary	Normal		
S130	105	JAIVAC-1	Dosage A	1	Day 7			02/09/2010		Dermatological	Normal		
S130	105	JAIVAC-1	Dosage A	1	Day 14			08/09/2010		Head and Neck	Normal		
S130	105	JAIVAC-1	Dosage A	1	Day 14			08/09/2010		Eyes	Normal		
S130	105	JAIVAC-1	Dosage A	1	Day 14			08/09/2010		Ears, nose and throat	Normal		
S130	105	JAIVAC-1	Dosage A	1	Day 14			08/09/2010		Cardiovascular	Normal		
S130	105	JAIVAC-1	Dosage A	1	Day 14			08/09/2010		Respiratory	Normal		
S130	105	JAIVAC-1	Dosage A	1	Day 14			08/09/2010		Musculoskeletal	Normal		
S130	105	JAIVAC-1	Dosage A	1	Day 14			08/09/2010		Venous system	Normal		
S130	105	JAIVAC-1	Dosage A	1	Day 14			08/09/2010		Gastrointestinal	Normal		

Subject No.	Randomisation Number	Study Group	Dosage	Dose	Day	Not Done	Specify	Date of Assessment	*Was the assessment performed prior to vaccination?	Physical Examination Parameter	Result	Abnormality	Clinical Significance
S130	105	JAIVAC-1	Dosage A	1	Day 14			08/09/2010		Lymphatic	Normal		
S130	105	JAIVAC-1	Dosage A	1	Day 14			08/09/2010		Neurological	Normal		
S130	105	JAIVAC-1	Dosage A	1	Day 14			08/09/2010		Genitourinary	Normal		
S130	105	JAIVAC-1	Dosage A	1	Day 14			08/09/2010		Dermatological	Normal		
S130	105	JAIVAC-1	Dosage A	2	Day 28			23/09/2010	Yes	Head and Neck	Normal		
S130	105	JAIVAC-1	Dosage A	2	Day 28			23/09/2010	Yes	Eyes	Normal		
S130	105	JAIVAC-1	Dosage A	2	Day 28			23/09/2010	Yes	Ears, nose and throat	Normal		
S130	105	JAIVAC-1	Dosage A	2	Day 28			23/09/2010	Yes	Cardiovascular	Normal		
S130	105	JAIVAC-1	Dosage A	2	Day 28			23/09/2010	Yes	Respiratory	Normal		
S130	105	JAIVAC-1	Dosage A	2	Day 28			23/09/2010	Yes	Musculoskeletal	Normal		
S130	105	JAIVAC-1	Dosage A	2	Day 28			23/09/2010	Yes	Venous system	Normal		
S130	105	JAIVAC-1	Dosage A	2	Day 28			23/09/2010	Yes	Gastrointestinal	Normal		
S130	105	JAIVAC-1	Dosage A	2	Day 28			23/09/2010	Yes	Lymphatic	Normal		
S130	105	JAIVAC-1	Dosage A	2	Day 28			23/09/2010	Yes	Neurological	Normal		
S130	105	JAIVAC-1	Dosage A	2	Day 28			23/09/2010	Yes	Genitourinary	Normal		
S130	105	JAIVAC-1	Dosage A	2	Day 28			23/09/2010	Yes	Dermatological	Normal		
S130	105	JAIVAC-1	Dosage A	2	Day 35			30/09/2010		Head and Neck	Normal		
S130	105	JAIVAC-1	Dosage A	2	Day 35			30/09/2010		Eyes	Normal		
S130	105	JAIVAC-1	Dosage A	2	Day 35			30/09/2010		Ears, nose and throat	Normal		

Subject No.	Randomisation Number	Study Group	Dosage	Dose	Day	Not Done	Specify	Date of Assessment	*Was the assessment performed prior to vaccination?	Physical Examination Parameter	Result	Abnormality	Clinical Significance
S130	105	JAIVAC-1	Dosage A	2	Day 35			30/09/2010		Cardiovascular	Normal		
S130	105	JAIVAC-1	Dosage A	2	Day 35			30/09/2010		Respiratory	Normal		
S130	105	JAIVAC-1	Dosage A	2	Day 35			30/09/2010		Musculoskeletal	Normal		
S130	105	JAIVAC-1	Dosage A	2	Day 35			30/09/2010		Venous system	Normal		
S130	105	JAIVAC-1	Dosage A	2	Day 35			30/09/2010		Gastrointestinal	Normal		
S130	105	JAIVAC-1	Dosage A	2	Day 35			30/09/2010		Lymphatic	Normal		
S130	105	JAIVAC-1	Dosage A	2	Day 35			30/09/2010		Neurological	Normal		
S130	105	JAIVAC-1	Dosage A	2	Day 35			30/09/2010		Genitourinary	Normal		
S130	105	JAIVAC-1	Dosage A	2	Day 35			30/09/2010		Dermatological	Normal		
S130	105	JAIVAC-1	Dosage A	2	Day 42			07/10/2010		Head and Neck	Normal		
S130	105	JAIVAC-1	Dosage A	2	Day 42			07/10/2010		Eyes	Normal		
S130	105	JAIVAC-1	Dosage A	2	Day 42			07/10/2010		Ears, nose and throat	Normal		
S130	105	JAIVAC-1	Dosage A	2	Day 42			07/10/2010		Cardiovascular	Normal		
S130	105	JAIVAC-1	Dosage A	2	Day 42			07/10/2010		Respiratory	Normal		
S130	105	JAIVAC-1	Dosage A	2	Day 42			07/10/2010		Musculoskeletal	Normal		
S130	105	JAIVAC-1	Dosage A	2	Day 42			07/10/2010		Venous system	Normal		
S130	105	JAIVAC-1	Dosage A	2	Day 42			07/10/2010		Gastrointestinal	Normal		
S130	105	JAIVAC-1	Dosage A	2	Day 42			07/10/2010		Lymphatic	Normal		
S130	105	JAIVAC-1	Dosage A	2	Day 42			07/10/2010		Neurological	Normal		

Subject No.	Randomisation Number	Study Group	Dosage	Dose	Day	Not Done	Specify	Date of Assessment	*Was the assessment performed prior to vaccination?	Physical Examination Parameter	Result	Abnormality	Clinical Significance
S130	105	JAIVAC-1	Dosage A	2	Day 42			07/10/2010		Genitourinary	Normal		
S130	105	JAIVAC-1	Dosage A	2	Day 42			07/10/2010		Dermatological	Normal		
S130	105	JAIVAC-1	Dosage A	2	Day 56			21/10/2010		Head and Neck	Normal		
S130	105	JAIVAC-1	Dosage A	2	Day 56			21/10/2010		Eyes	Normal		
S130	105	JAIVAC-1	Dosage A	2	Day 56			21/10/2010		Ears, nose and throat	Normal		
S130	105	JAIVAC-1	Dosage A	2	Day 56			21/10/2010		Cardiovascular	Normal		
S130	105	JAIVAC-1	Dosage A	2	Day 56			21/10/2010		Respiratory	Normal		
S130	105	JAIVAC-1	Dosage A	2	Day 56			21/10/2010		Musculoskeletal	Normal		
S130	105	JAIVAC-1	Dosage A	2	Day 56			21/10/2010		Venous system	Normal		
S130	105	JAIVAC-1	Dosage A	2	Day 56			21/10/2010		Gastrointestinal	Normal		
S130	105	JAIVAC-1	Dosage A	2	Day 56			21/10/2010		Lymphatic	Normal		
S130	105	JAIVAC-1	Dosage A	2	Day 56			21/10/2010		Neurological	Normal		
S130	105	JAIVAC-1	Dosage A	2	Day 56			21/10/2010		Genitourinary	Normal		
S130	105	JAIVAC-1	Dosage A	2	Day 56			21/10/2010		Dermatological	Normal		
S130	105	JAIVAC-1	Dosage A	3	Day 180			22/02/2011	Yes	Head and Neck	Normal		
S130	105	JAIVAC-1	Dosage A	3	Day 180			22/02/2011	Yes	Eyes	Normal		
S130	105	JAIVAC-1	Dosage A	3	Day 180			22/02/2011	Yes	Ears, nose and throat	Normal		
S130	105	JAIVAC-1	Dosage A	3	Day 180			22/02/2011	Yes	Cardiovascular	Normal		
S130	105	JAIVAC-1	Dosage A	3	Day 180			22/02/2011	Yes	Respiratory	Normal		

Subject No.	Randomisation Number	Study Group	Dosage	Dose	Day	Not Done	Specify	Date of Assessment	*Was the assessment performed prior to vaccination?	Physical Examination Parameter	Result	Abnormality	Clinical Significance
S130	105	JAIVAC-1	Dosage A	3	Day 180			22/02/2011	Yes	Musculoskeletal	Normal		
S130	105	JAIVAC-1	Dosage A	3	Day 180			22/02/2011	Yes	Venous system	Normal		
S130	105	JAIVAC-1	Dosage A	3	Day 180			22/02/2011	Yes	Gastrointestinal	Normal		
S130	105	JAIVAC-1	Dosage A	3	Day 180			22/02/2011	Yes	Lymphatic	Normal		
S130	105	JAIVAC-1	Dosage A	3	Day 180			22/02/2011	Yes	Neurological	Normal		
S130	105	JAIVAC-1	Dosage A	3	Day 180			22/02/2011	Yes	Genitourinary	Normal		
S130	105	JAIVAC-1	Dosage A	3	Day 180			22/02/2011	Yes	Dermatological	Normal		
S130	105	JAIVAC-1	Dosage A	3	Day 187			01/03/2011		Head and Neck	Normal		
S130	105	JAIVAC-1	Dosage A	3	Day 187			01/03/2011		Eyes	Normal		
S130	105	JAIVAC-1	Dosage A	3	Day 187			01/03/2011		Ears, nose and throat	Normal		
S130	105	JAIVAC-1	Dosage A	3	Day 187			01/03/2011		Cardiovascular	Normal		
S130	105	JAIVAC-1	Dosage A	3	Day 187			01/03/2011		Respiratory	Normal		
S130	105	JAIVAC-1	Dosage A	3	Day 187			01/03/2011		Musculoskeletal	Normal		
S130	105	JAIVAC-1	Dosage A	3	Day 187			01/03/2011		Venous system	Normal		
S130	105	JAIVAC-1	Dosage A	3	Day 187			01/03/2011		Gastrointestinal	Normal		
S130	105	JAIVAC-1	Dosage A	3	Day 187			01/03/2011		Lymphatic	Normal		
S130	105	JAIVAC-1	Dosage A	3	Day 187			01/03/2011		Neurological	Normal		
S130	105	JAIVAC-1	Dosage A	3	Day 187			01/03/2011		Genitourinary	Normal		
S130	105	JAIVAC-1	Dosage A	3	Day 187			01/03/2011		Dermatological	Normal		

Subject No.	Randomisation Number	Study Group	Dosage	Dose	Day	Not Done	Specify	Date of Assessment	*Was the assessment performed prior to vaccination?	Physical Examination Parameter	Result	Abnormality	Clinical Significance
S130	105	JAIVAC-1	Dosage A	3	Day 194			08/03/2011		Head and Neck	Normal		
S130	105	JAIVAC-1	Dosage A	3	Day 194			08/03/2011		Eyes	Normal		
S130	105	JAIVAC-1	Dosage A	3	Day 194			08/03/2011		Ears, nose and throat	Normal		
S130	105	JAIVAC-1	Dosage A	3	Day 194			08/03/2011		Cardiovascular	Normal		
S130	105	JAIVAC-1	Dosage A	3	Day 194			08/03/2011		Respiratory	Normal		
S130	105	JAIVAC-1	Dosage A	3	Day 194			08/03/2011		Musculoskeletal	Normal		
S130	105	JAIVAC-1	Dosage A	3	Day 194			08/03/2011		Venous system	Normal		
S130	105	JAIVAC-1	Dosage A	3	Day 194			08/03/2011		Gastrointestinal	Normal		
S130	105	JAIVAC-1	Dosage A	3	Day 194			08/03/2011		Lymphatic	Normal		
S130	105	JAIVAC-1	Dosage A	3	Day 194			08/03/2011		Neurological	Normal		
S130	105	JAIVAC-1	Dosage A	3	Day 194			08/03/2011		Genitourinary	Normal		
S130	105	JAIVAC-1	Dosage A	3	Day 194			08/03/2011		Dermatological	Normal		
S130	105	JAIVAC-1	Dosage A	3	Day 208			22/03/2011		Head and Neck	Normal		
S130	105	JAIVAC-1	Dosage A	3	Day 208			22/03/2011		Eyes	Normal		
S130	105	JAIVAC-1	Dosage A	3	Day 208			22/03/2011		Ears, nose and throat	Normal		
S130	105	JAIVAC-1	Dosage A	3	Day 208			22/03/2011		Cardiovascular	Normal		
S130	105	JAIVAC-1	Dosage A	3	Day 208			22/03/2011		Respiratory	Normal		
S130	105	JAIVAC-1	Dosage A	3	Day 208			22/03/2011		Musculoskeletal	Normal		
S130	105	JAIVAC-1	Dosage A	3	Day 208			22/03/2011		Venous system	Normal		

Subject No.	Randomisation Number	Study Group	Dosage	Dose	Day	Not Done	Specify	Date of Assessment	*Was the assessment performed prior to vaccination?	Physical Examination Parameter	Result	Abnormality	Clinical Significance
S130	105	JAIVAC-1	Dosage A	3	Day 208			22/03/2011		Gastrointestinal	Normal		
S130	105	JAIVAC-1	Dosage A	3	Day 208			22/03/2011		Lymphatic	Normal		
S130	105	JAIVAC-1	Dosage A	3	Day 208			22/03/2011		Neurological	Normal		
S130	105	JAIVAC-1	Dosage A	3	Day 208			22/03/2011		Genitourinary	Normal		
S130	105	JAIVAC-1	Dosage A	3	Day 208			22/03/2011		Dermatological	Normal		
S130	105	JAIVAC-1	Dosage A	3	Day 365			25/08/2011		Head and Neck	Normal		
S130	105	JAIVAC-1	Dosage A	3	Day 365			25/08/2011		Eyes	Normal		
S130	105	JAIVAC-1	Dosage A	3	Day 365			25/08/2011		Ears, nose and throat	Normal		
S130	105	JAIVAC-1	Dosage A	3	Day 365			25/08/2011		Cardiovascular	Normal		
S130	105	JAIVAC-1	Dosage A	3	Day 365			25/08/2011		Respiratory	Normal		
S130	105	JAIVAC-1	Dosage A	3	Day 365			25/08/2011		Musculoskeletal	Normal		
S130	105	JAIVAC-1	Dosage A	3	Day 365			25/08/2011		Venous system	Normal		
S130	105	JAIVAC-1	Dosage A	3	Day 365			25/08/2011		Gastrointestinal	Normal		
S130	105	JAIVAC-1	Dosage A	3	Day 365			25/08/2011		Lymphatic	Normal		
S130	105	JAIVAC-1	Dosage A	3	Day 365			25/08/2011		Neurological	Normal		
S130	105	JAIVAC-1	Dosage A	3	Day 365			25/08/2011		Genitourinary	Normal		
S130	105	JAIVAC-1	Dosage A	3	Day 365			25/08/2011		Dermatological	Normal		
S130	105	JAIVAC-1	Dosage A		Unscheduled Visit	Yes	Laboratory Repeat At Local Lab	.		Head and Neck			

Subject No.	Randomisation Number	Study Group	Dosage	Dose	Day	Not Done	Specify	Date of Assessment	*Was the assessment performed prior to vaccination?	Physical Examination Parameter	Result	Abnormality	Clinical Significance
S130	105	JAIVAC-1	Dosage A		Unscheduled Visit	Yes	Laboratory Repeat At Local Lab	.		Eyes			
S130	105	JAIVAC-1	Dosage A		Unscheduled Visit	Yes	Laboratory Repeat At Local Lab	.		Ears, nose and throat			
S130	105	JAIVAC-1	Dosage A		Unscheduled Visit	Yes	Laboratory Repeat At Local Lab	.		Cardiovascular			
S130	105	JAIVAC-1	Dosage A		Unscheduled Visit	Yes	Laboratory Repeat At Local Lab	.		Respiratory			
S130	105	JAIVAC-1	Dosage A		Unscheduled Visit	Yes	Laboratory Repeat At Local Lab	.		Musculoskeletal			
S130	105	JAIVAC-1	Dosage A		Unscheduled Visit	Yes	Laboratory Repeat At Local Lab	.		Venous system			
S130	105	JAIVAC-1	Dosage A		Unscheduled Visit	Yes	Laboratory Repeat At Local Lab	.		Gastrointestinal			
S130	105	JAIVAC-1	Dosage A		Unscheduled Visit	Yes	Laboratory Repeat At Local Lab	.		Lymphatic			
S130	105	JAIVAC-1	Dosage A		Unscheduled Visit	Yes	Laboratory Repeat At Local Lab	.		Neurological			
S130	105	JAIVAC-1	Dosage A		Unscheduled Visit	Yes	Laboratory Repeat At Local Lab	.		Genitourinary			
S130	105	JAIVAC-1	Dosage A		Unscheduled Visit	Yes	Laboratory Repeat At Local Lab	.		Dermatological			
S132	108	Hepatitis B	Hepatitis B		Day -14 to Day -1			18/08/2010		Head and neck	Normal		

Subject No.	Randomisation Number	Study Group	Dosage	Dose	Day	Not Done	Specify	Date of Assessment	*Was the assessment performed prior to vaccination?	Physical Examination Parameter	Result	Abnormality	Clinical Significance
S132	108	Hepatitis B	Hepatitis B		Day -14 to Day -1			18/08/2010		Eyes	Normal		
S132	108	Hepatitis B	Hepatitis B		Day -14 to Day -1			18/08/2010		Ears, nose and throat	Normal		
S132	108	Hepatitis B	Hepatitis B		Day -14 to Day -1			18/08/2010		Cardiovascular	Normal		
S132	108	Hepatitis B	Hepatitis B		Day -14 to Day -1			18/08/2010		Respiratory	Abnormal	Pectus Excavatum(Normal Variant)	NCS
S132	108	Hepatitis B	Hepatitis B		Day -14 to Day -1			18/08/2010		Musculoskeletal	Normal		
S132	108	Hepatitis B	Hepatitis B		Day -14 to Day -1			18/08/2010		Venous system	Normal		
S132	108	Hepatitis B	Hepatitis B		Day -14 to Day -1			18/08/2010		Gastrointestinal	Normal		
S132	108	Hepatitis B	Hepatitis B		Day -14 to Day -1			18/08/2010		Lymphatic	Normal		
S132	108	Hepatitis B	Hepatitis B		Day -14 to Day -1			18/08/2010		Neurological	Normal		
S132	108	Hepatitis B	Hepatitis B		Day -14 to Day -1			18/08/2010		Genitourinary	Normal		
S132	108	Hepatitis B	Hepatitis B		Day -14 to Day -1			18/08/2010		Dermatological	Normal		
S132	108	Hepatitis B	Hepatitis B	1	Day 0			26/08/2010	Yes	Head and neck	Normal		
S132	108	Hepatitis B	Hepatitis B	1	Day 0			26/08/2010	Yes	Eyes	Normal		
S132	108	Hepatitis B	Hepatitis B	1	Day 0			26/08/2010	Yes	Ears, nose and throat	Normal		
S132	108	Hepatitis B	Hepatitis B	1	Day 0			26/08/2010	Yes	Cardiovascular	Normal		
S132	108	Hepatitis B	Hepatitis B	1	Day 0			26/08/2010	Yes	Respiratory	Abnormal	Pectus Excavatum	NCS

Subject No.	Randomisation Number	Study Group	Dosage	Dose	Day	Not Done	Specify	Date of Assessment	*Was the assessment performed prior to vaccination?	Physical Examination Parameter	Result	Abnormality	Clinical Significance
S132	108	Hepatitis B	Hepatitis B	1	Day 0			26/08/2010	Yes	Musculoskeletal	Normal		
S132	108	Hepatitis B	Hepatitis B	1	Day 0			26/08/2010	Yes	Venous system	Normal		
S132	108	Hepatitis B	Hepatitis B	1	Day 0			26/08/2010	Yes	Gastrointestinal	Normal		
S132	108	Hepatitis B	Hepatitis B	1	Day 0			26/08/2010	Yes	Lymphatic	Normal		
S132	108	Hepatitis B	Hepatitis B	1	Day 0			26/08/2010	Yes	Neurological	Normal		
S132	108	Hepatitis B	Hepatitis B	1	Day 0			26/08/2010	Yes	Genitourinary	Normal		
S132	108	Hepatitis B	Hepatitis B	1	Day 0			26/08/2010	Yes	Dermatological	Normal		
S132	108	Hepatitis B	Hepatitis B	1	Day 7			02/09/2010		Head and Neck	Normal		
S132	108	Hepatitis B	Hepatitis B	1	Day 7			02/09/2010		Eyes	Normal		
S132	108	Hepatitis B	Hepatitis B	1	Day 7			02/09/2010		Ears, nose and throat	Normal		
S132	108	Hepatitis B	Hepatitis B	1	Day 7			02/09/2010		Cardiovascular	Normal		
S132	108	Hepatitis B	Hepatitis B	1	Day 7			02/09/2010		Respiratory	Abnormal	Pectus Excavatum	NCS
S132	108	Hepatitis B	Hepatitis B	1	Day 7			02/09/2010		Musculoskeletal	Normal		
S132	108	Hepatitis B	Hepatitis B	1	Day 7			02/09/2010		Venous system	Normal		
S132	108	Hepatitis B	Hepatitis B	1	Day 7			02/09/2010		Gastrointestinal	Normal		
S132	108	Hepatitis B	Hepatitis B	1	Day 7			02/09/2010		Lymphatic	Normal		
S132	108	Hepatitis B	Hepatitis B	1	Day 7			02/09/2010		Neurological	Normal		
S132	108	Hepatitis B	Hepatitis B	1	Day 7			02/09/2010		Genitourinary	Normal		

Subject No.	Randomisation Number	Study Group	Dosage	Dose	Day	Not Done	Specify	Date of Assessment	*Was the assessment performed prior to vaccination?	Physical Examination Parameter	Result	Abnormality	Clinical Significance
S132	108	Hepatitis B	Hepatitis B	1	Day 7			02/09/2010		Dermatological	Normal		
S132	108	Hepatitis B	Hepatitis B	1	Day 14			09/09/2010		Head and Neck	Normal		
S132	108	Hepatitis B	Hepatitis B	1	Day 14			09/09/2010		Eyes	Normal		
S132	108	Hepatitis B	Hepatitis B	1	Day 14			09/09/2010		Ears, nose and throat	Normal		
S132	108	Hepatitis B	Hepatitis B	1	Day 14			09/09/2010		Cardiovascular	Normal		
S132	108	Hepatitis B	Hepatitis B	1	Day 14			09/09/2010		Respiratory	Abnormal	Pectus Excavatum	NCS
S132	108	Hepatitis B	Hepatitis B	1	Day 14			09/09/2010		Musculoskeletal	Normal		
S132	108	Hepatitis B	Hepatitis B	1	Day 14			09/09/2010		Venous system	Normal		
S132	108	Hepatitis B	Hepatitis B	1	Day 14			09/09/2010		Gastrointestinal	Normal		
S132	108	Hepatitis B	Hepatitis B	1	Day 14			09/09/2010		Lymphatic	Normal		
S132	108	Hepatitis B	Hepatitis B	1	Day 14			09/09/2010		Neurological	Normal		
S132	108	Hepatitis B	Hepatitis B	1	Day 14			09/09/2010		Genitourinary	Normal		
S132	108	Hepatitis B	Hepatitis B	1	Day 14			09/09/2010		Dermatological	Normal		
S132	108	Hepatitis B	Hepatitis B	2	Day 28			23/09/2010	Yes	Head and Neck	Normal		
S132	108	Hepatitis B	Hepatitis B	2	Day 28			23/09/2010	Yes	Eyes	Normal		
S132	108	Hepatitis B	Hepatitis B	2	Day 28			23/09/2010	Yes	Ears, nose and throat	Normal		
S132	108	Hepatitis B	Hepatitis B	2	Day 28			23/09/2010	Yes	Cardiovascular	Normal		
S132	108	Hepatitis B	Hepatitis B	2	Day 28			23/09/2010	Yes	Respiratory	Abnormal	Pectus Excavatum	NCS

Subject No.	Randomisation Number	Study Group	Dosage	Dose	Day	Not Done	Specify	Date of Assessment	*Was the assessment performed prior to vaccination?	Physical Examination Parameter	Result	Abnormality	Clinical Significance
S132	108	Hepatitis B	Hepatitis B	2	Day 28			23/09/2010	Yes	Musculoskeletal	Normal		
S132	108	Hepatitis B	Hepatitis B	2	Day 28			23/09/2010	Yes	Venous system	Normal		
S132	108	Hepatitis B	Hepatitis B	2	Day 28			23/09/2010	Yes	Gastrointestinal	Normal		
S132	108	Hepatitis B	Hepatitis B	2	Day 28			23/09/2010	Yes	Lymphatic	Normal		
S132	108	Hepatitis B	Hepatitis B	2	Day 28			23/09/2010	Yes	Neurological	Normal		
S132	108	Hepatitis B	Hepatitis B	2	Day 28			23/09/2010	Yes	Genitourinary	Normal		
S132	108	Hepatitis B	Hepatitis B	2	Day 28			23/09/2010	Yes	Dermatological	Normal		
S132	108	Hepatitis B	Hepatitis B	2	Day 35			30/09/2010		Head and Neck	Normal		
S132	108	Hepatitis B	Hepatitis B	2	Day 35			30/09/2010		Eyes	Normal		
S132	108	Hepatitis B	Hepatitis B	2	Day 35			30/09/2010		Ears, nose and throat	Normal		
S132	108	Hepatitis B	Hepatitis B	2	Day 35			30/09/2010		Cardiovascular	Normal		
S132	108	Hepatitis B	Hepatitis B	2	Day 35			30/09/2010		Respiratory	Abnormal	Pectus Excavatum	NCS
S132	108	Hepatitis B	Hepatitis B	2	Day 35			30/09/2010		Musculoskeletal	Normal		
S132	108	Hepatitis B	Hepatitis B	2	Day 35			30/09/2010		Venous system	Normal		
S132	108	Hepatitis B	Hepatitis B	2	Day 35			30/09/2010		Gastrointestinal	Normal		
S132	108	Hepatitis B	Hepatitis B	2	Day 35			30/09/2010		Lymphatic	Normal		
S132	108	Hepatitis B	Hepatitis B	2	Day 35			30/09/2010		Neurological	Normal		
S132	108	Hepatitis B	Hepatitis B	2	Day 35			30/09/2010		Genitourinary	Normal		

Subject No.	Randomisation Number	Study Group	Dosage	Dose	Day	Not Done	Specify	Date of Assessment	*Was the assessment performed prior to vaccination?	Physical Examination Parameter	Result	Abnormality	Clinical Significance
S132	108	Hepatitis B	Hepatitis B	2	Day 35			30/09/2010		Dermatological	Normal		
S132	108	Hepatitis B	Hepatitis B	2	Day 42			07/10/2010		Head and Neck	Normal		
S132	108	Hepatitis B	Hepatitis B	2	Day 42			07/10/2010		Eyes	Normal		
S132	108	Hepatitis B	Hepatitis B	2	Day 42			07/10/2010		Ears, nose and throat	Normal		
S132	108	Hepatitis B	Hepatitis B	2	Day 42			07/10/2010		Cardiovascular	Normal		
S132	108	Hepatitis B	Hepatitis B	2	Day 42			07/10/2010		Respiratory	Abnormal	Pectus Excavatum	NCS
S132	108	Hepatitis B	Hepatitis B	2	Day 42			07/10/2010		Musculoskeletal	Normal		
S132	108	Hepatitis B	Hepatitis B	2	Day 42			07/10/2010		Venous system	Normal		
S132	108	Hepatitis B	Hepatitis B	2	Day 42			07/10/2010		Gastrointestinal	Normal		
S132	108	Hepatitis B	Hepatitis B	2	Day 42			07/10/2010		Lymphatic	Normal		
S132	108	Hepatitis B	Hepatitis B	2	Day 42			07/10/2010		Neurological	Normal		
S132	108	Hepatitis B	Hepatitis B	2	Day 42			07/10/2010		Genitourinary	Normal		
S132	108	Hepatitis B	Hepatitis B	2	Day 42			07/10/2010		Dermatological	Normal		
S132	108	Hepatitis B	Hepatitis B	2	Day 56			21/10/2010		Head and Neck	Normal		
S132	108	Hepatitis B	Hepatitis B	2	Day 56			21/10/2010		Eyes	Normal		
S132	108	Hepatitis B	Hepatitis B	2	Day 56			21/10/2010		Ears, nose and throat	Normal		
S132	108	Hepatitis B	Hepatitis B	2	Day 56			21/10/2010		Cardiovascular	Normal		
S132	108	Hepatitis B	Hepatitis B	2	Day 56			21/10/2010		Respiratory	Abnormal	Pectus Excavatum	NCS

Subject No.	Randomisation Number	Study Group	Dosage	Dose	Day	Not Done	Specify	Date of Assessment	*Was the assessment performed prior to vaccination?	Physical Examination Parameter	Result	Abnormality	Clinical Significance
S132	108	Hepatitis B	Hepatitis B	2	Day 56			21/10/2010		Musculoskeletal	Normal		
S132	108	Hepatitis B	Hepatitis B	2	Day 56			21/10/2010		Venous system	Normal		
S132	108	Hepatitis B	Hepatitis B	2	Day 56			21/10/2010		Gastrointestinal	Normal		
S132	108	Hepatitis B	Hepatitis B	2	Day 56			21/10/2010		Lymphatic	Normal		
S132	108	Hepatitis B	Hepatitis B	2	Day 56			21/10/2010		Neurological	Normal		
S132	108	Hepatitis B	Hepatitis B	2	Day 56			21/10/2010		Genitourinary	Normal		
S132	108	Hepatitis B	Hepatitis B	2	Day 56			21/10/2010		Dermatological	Normal		
S132	108	Hepatitis B	Hepatitis B	3	Day 180			22/02/2011	Yes	Head and Neck	Normal		
S132	108	Hepatitis B	Hepatitis B	3	Day 180			22/02/2011	Yes	Eyes	Normal		
S132	108	Hepatitis B	Hepatitis B	3	Day 180			22/02/2011	Yes	Ears, nose and throat	Normal		
S132	108	Hepatitis B	Hepatitis B	3	Day 180			22/02/2011	Yes	Cardiovascular	Normal		
S132	108	Hepatitis B	Hepatitis B	3	Day 180			22/02/2011	Yes	Respiratory	Abnormal	Pectus Excavatum	NCS
S132	108	Hepatitis B	Hepatitis B	3	Day 180			22/02/2011	Yes	Musculoskeletal	Normal		
S132	108	Hepatitis B	Hepatitis B	3	Day 180			22/02/2011	Yes	Venous system	Normal		
S132	108	Hepatitis B	Hepatitis B	3	Day 180			22/02/2011	Yes	Gastrointestinal	Normal		
S132	108	Hepatitis B	Hepatitis B	3	Day 180			22/02/2011	Yes	Lymphatic	Normal		
S132	108	Hepatitis B	Hepatitis B	3	Day 180			22/02/2011	Yes	Neurological	Normal		
S132	108	Hepatitis B	Hepatitis B	3	Day 180			22/02/2011	Yes	Genitourinary	Normal		

Subject No.	Randomisation Number	Study Group	Dosage	Dose	Day	Not Done	Specify	Date of Assessment	*Was the assessment performed prior to vaccination?	Physical Examination Parameter	Result	Abnormality	Clinical Significance
S132	108	Hepatitis B	Hepatitis B	3	Day 180			22/02/2011	Yes	Dermatological	Normal		
S132	108	Hepatitis B	Hepatitis B	3	Day 187			01/03/2011		Head and Neck	Normal		
S132	108	Hepatitis B	Hepatitis B	3	Day 187			01/03/2011		Eyes	Normal		
S132	108	Hepatitis B	Hepatitis B	3	Day 187			01/03/2011		Ears, nose and throat	Normal		
S132	108	Hepatitis B	Hepatitis B	3	Day 187			01/03/2011		Cardiovascular	Normal		
S132	108	Hepatitis B	Hepatitis B	3	Day 187			01/03/2011		Respiratory	Abnormal	Pectus Excavatum	NCS
S132	108	Hepatitis B	Hepatitis B	3	Day 187			01/03/2011		Musculoskeletal	Normal		
S132	108	Hepatitis B	Hepatitis B	3	Day 187			01/03/2011		Venous system	Normal		
S132	108	Hepatitis B	Hepatitis B	3	Day 187			01/03/2011		Gastrointestinal	Normal		
S132	108	Hepatitis B	Hepatitis B	3	Day 187			01/03/2011		Lymphatic	Normal		
S132	108	Hepatitis B	Hepatitis B	3	Day 187			01/03/2011		Neurological	Normal		
S132	108	Hepatitis B	Hepatitis B	3	Day 187			01/03/2011		Genitourinary	Normal		
S132	108	Hepatitis B	Hepatitis B	3	Day 187			01/03/2011		Dermatological	Normal		
S132	108	Hepatitis B	Hepatitis B	3	Day 194			08/03/2011		Head and Neck	Normal		
S132	108	Hepatitis B	Hepatitis B	3	Day 194			08/03/2011		Eyes	Normal		
S132	108	Hepatitis B	Hepatitis B	3	Day 194			08/03/2011		Ears, nose and throat	Normal		
S132	108	Hepatitis B	Hepatitis B	3	Day 194			08/03/2011		Cardiovascular	Normal		
S132	108	Hepatitis B	Hepatitis B	3	Day 194			08/03/2011		Respiratory	Abnormal	Pectus Excavatum	NCS

Subject No.	Randomisation Number	Study Group	Dosage	Dose	Day	Not Done	Specify	Date of Assessment	*Was the assessment performed prior to vaccination?	Physical Examination Parameter	Result	Abnormality	Clinical Significance
S132	108	Hepatitis B	Hepatitis B	3	Day 194			08/03/2011		Musculoskeletal	Normal		
S132	108	Hepatitis B	Hepatitis B	3	Day 194			08/03/2011		Venous system	Normal		
S132	108	Hepatitis B	Hepatitis B	3	Day 194			08/03/2011		Gastrointestinal	Normal		
S132	108	Hepatitis B	Hepatitis B	3	Day 194			08/03/2011		Lymphatic	Normal		
S132	108	Hepatitis B	Hepatitis B	3	Day 194			08/03/2011		Neurological	Normal		
S132	108	Hepatitis B	Hepatitis B	3	Day 194			08/03/2011		Genitourinary	Normal		
S132	108	Hepatitis B	Hepatitis B	3	Day 194			08/03/2011		Dermatological	Normal		
S132	108	Hepatitis B	Hepatitis B	3	Day 208			22/03/2011		Head and Neck	Normal		
S132	108	Hepatitis B	Hepatitis B	3	Day 208			22/03/2011		Eyes	Normal		
S132	108	Hepatitis B	Hepatitis B	3	Day 208			22/03/2011		Ears, nose and throat	Normal		
S132	108	Hepatitis B	Hepatitis B	3	Day 208			22/03/2011		Cardiovascular	Normal		
S132	108	Hepatitis B	Hepatitis B	3	Day 208			22/03/2011		Respiratory	Abnormal	Pectus Excavatum	NCS
S132	108	Hepatitis B	Hepatitis B	3	Day 208			22/03/2011		Musculoskeletal	Normal		
S132	108	Hepatitis B	Hepatitis B	3	Day 208			22/03/2011		Venous system	Normal		
S132	108	Hepatitis B	Hepatitis B	3	Day 208			22/03/2011		Gastrointestinal	Normal		
S132	108	Hepatitis B	Hepatitis B	3	Day 208			22/03/2011		Lymphatic	Normal		
S132	108	Hepatitis B	Hepatitis B	3	Day 208			22/03/2011		Neurological	Normal		
S132	108	Hepatitis B	Hepatitis B	3	Day 208			22/03/2011		Genitourinary	Normal		

Subject No.	Randomisation Number	Study Group	Dosage	Dose	Day	Not Done	Specify	Date of Assessment	*Was the assessment performed prior to vaccination?	Physical Examination Parameter	Result	Abnormality	Clinical Significance
S132	108	Hepatitis B	Hepatitis B	3	Day 208			22/03/2011		Dermatological	Normal		
S132	108	Hepatitis B	Hepatitis B	3	Day 365			25/08/2011		Head and Neck	Normal		
S132	108	Hepatitis B	Hepatitis B	3	Day 365			25/08/2011		Eyes	Normal		
S132	108	Hepatitis B	Hepatitis B	3	Day 365			25/08/2011		Ears, nose and throat	Normal		
S132	108	Hepatitis B	Hepatitis B	3	Day 365			25/08/2011		Cardiovascular	Normal		
S132	108	Hepatitis B	Hepatitis B	3	Day 365			25/08/2011		Respiratory	Abnormal	Pectus Excavation	NCS
S132	108	Hepatitis B	Hepatitis B	3	Day 365			25/08/2011		Musculoskeletal	Normal		
S132	108	Hepatitis B	Hepatitis B	3	Day 365			25/08/2011		Venous system	Normal		
S132	108	Hepatitis B	Hepatitis B	3	Day 365			25/08/2011		Gastrointestinal	Normal		
S132	108	Hepatitis B	Hepatitis B	3	Day 365			25/08/2011		Lymphatic	Normal		
S132	108	Hepatitis B	Hepatitis B	3	Day 365			25/08/2011		Neurological	Normal		
S132	108	Hepatitis B	Hepatitis B	3	Day 365			25/08/2011		Genitourinary	Normal		
S132	108	Hepatitis B	Hepatitis B	3	Day 365			25/08/2011		Dermatological	Normal		
S136	109	JAIVAC-1	Dosage A		Day -14 to Day -1			19/08/2010		Head and neck	Normal		
S136	109	JAIVAC-1	Dosage A		Day -14 to Day -1			19/08/2010		Eyes	Normal		
S136	109	JAIVAC-1	Dosage A		Day -14 to Day -1			19/08/2010		Ears, nose and throat	Normal		
S136	109	JAIVAC-1	Dosage A		Day -14 to Day -1			19/08/2010		Cardiovascular	Normal		
S136	109	JAIVAC-1	Dosage A		Day -14 to Day -1			19/08/2010		Respiratory	Normal		

Subject No.	Randomisation Number	Study Group	Dosage	Dose	Day	Not Done	Specify	Date of Assessment	*Was the assessment performed prior to vaccination?	Physical Examination Parameter	Result	Abnormality	Clinical Significance
S136	109	JAIVAC-1	Dosage A		Day -14 to Day -1			19/08/2010		Musculoskeletal	Normal		
S136	109	JAIVAC-1	Dosage A		Day -14 to Day -1			19/08/2010		Venous system	Normal		
S136	109	JAIVAC-1	Dosage A		Day -14 to Day -1			19/08/2010		Gastrointestinal	Normal		
S136	109	JAIVAC-1	Dosage A		Day -14 to Day -1			19/08/2010		Lymphatic	Normal		
S136	109	JAIVAC-1	Dosage A		Day -14 to Day -1			19/08/2010		Neurological	Normal		
S136	109	JAIVAC-1	Dosage A		Day -14 to Day -1			19/08/2010		Genitourinary	Normal		
S136	109	JAIVAC-1	Dosage A		Day -14 to Day -1			19/08/2010		Dermatological	Normal		
S136	109	JAIVAC-1	Dosage A	1	Day 0			26/08/2010	Yes	Head and neck	Normal		
S136	109	JAIVAC-1	Dosage A	1	Day 0			26/08/2010	Yes	Eyes	Normal		
S136	109	JAIVAC-1	Dosage A	1	Day 0			26/08/2010	Yes	Ears, nose and throat	Normal		
S136	109	JAIVAC-1	Dosage A	1	Day 0			26/08/2010	Yes	Cardiovascular	Normal		
S136	109	JAIVAC-1	Dosage A	1	Day 0			26/08/2010	Yes	Respiratory	Normal		
S136	109	JAIVAC-1	Dosage A	1	Day 0			26/08/2010	Yes	Musculoskeletal	Normal		
S136	109	JAIVAC-1	Dosage A	1	Day 0			26/08/2010	Yes	Venous system	Normal		
S136	109	JAIVAC-1	Dosage A	1	Day 0			26/08/2010	Yes	Gastrointestinal	Normal		
S136	109	JAIVAC-1	Dosage A	1	Day 0			26/08/2010	Yes	Lymphatic	Normal		
S136	109	JAIVAC-1	Dosage A	1	Day 0			26/08/2010	Yes	Neurological	Normal		
S136	109	JAIVAC-1	Dosage A	1	Day 0			26/08/2010	Yes	Genitourinary	Normal		

Subject No.	Randomisation Number	Study Group	Dosage	Dose	Day	Not Done	Specify	Date of Assessment	*Was the assessment performed prior to vaccination?	Physical Examination Parameter	Result	Abnormality	Clinical Significance
S136	109	JAIVAC-1	Dosage A	1	Day 0			26/08/2010	Yes	Dermatological	Normal		
S136	109	JAIVAC-1	Dosage A	1	Day 7			02/09/2010		Head and Neck	Normal		
S136	109	JAIVAC-1	Dosage A	1	Day 7			02/09/2010		Eyes	Normal		
S136	109	JAIVAC-1	Dosage A	1	Day 7			02/09/2010		Ears, nose and throat	Normal		
S136	109	JAIVAC-1	Dosage A	1	Day 7			02/09/2010		Cardiovascular	Normal		
S136	109	JAIVAC-1	Dosage A	1	Day 7			02/09/2010		Respiratory	Normal		
S136	109	JAIVAC-1	Dosage A	1	Day 7			02/09/2010		Musculoskeletal	Normal		
S136	109	JAIVAC-1	Dosage A	1	Day 7			02/09/2010		Venous system	Normal		
S136	109	JAIVAC-1	Dosage A	1	Day 7			02/09/2010		Gastrointestinal	Normal		
S136	109	JAIVAC-1	Dosage A	1	Day 7			02/09/2010		Lymphatic	Normal		
S136	109	JAIVAC-1	Dosage A	1	Day 7			02/09/2010		Neurological	Normal		
S136	109	JAIVAC-1	Dosage A	1	Day 7			02/09/2010		Genitourinary	Normal		
S136	109	JAIVAC-1	Dosage A	1	Day 7			02/09/2010		Dermatological	Normal		
S136	109	JAIVAC-1	Dosage A	1	Day 14			09/09/2010		Head and Neck	Normal		
S136	109	JAIVAC-1	Dosage A	1	Day 14			09/09/2010		Eyes	Normal		
S136	109	JAIVAC-1	Dosage A	1	Day 14			09/09/2010		Ears, nose and throat	Normal		
S136	109	JAIVAC-1	Dosage A	1	Day 14			09/09/2010		Cardiovascular	Normal		
S136	109	JAIVAC-1	Dosage A	1	Day 14			09/09/2010		Respiratory	Normal		
S136	109	JAIVAC-1	Dosage A	1	Day 14			09/09/2010		Musculoskeletal	Normal		

Subject No.	Randomisation Number	Study Group	Dosage	Dose	Day	Not Done	Specify	Date of Assessment	*Was the assessment performed prior to vaccination?	Physical Examination Parameter	Result	Abnormality	Clinical Significance
S136	109	JAIVAC-1	Dosage A	1	Day 14			09/09/2010		Venous system	Normal		
S136	109	JAIVAC-1	Dosage A	1	Day 14			09/09/2010		Gastrointestinal	Normal		
S136	109	JAIVAC-1	Dosage A	1	Day 14			09/09/2010		Lymphatic	Normal		
S136	109	JAIVAC-1	Dosage A	1	Day 14			09/09/2010		Neurological	Normal		
S136	109	JAIVAC-1	Dosage A	1	Day 14			09/09/2010		Genitourinary	Normal		
S136	109	JAIVAC-1	Dosage A	1	Day 14			09/09/2010		Dermatological	Normal		
S136	109	JAIVAC-1	Dosage A	2	Day 28			23/09/2010	Yes	Head and Neck	Normal		
S136	109	JAIVAC-1	Dosage A	2	Day 28			23/09/2010	Yes	Eyes	Normal		
S136	109	JAIVAC-1	Dosage A	2	Day 28			23/09/2010	Yes	Ears, nose and throat	Normal		
S136	109	JAIVAC-1	Dosage A	2	Day 28			23/09/2010	Yes	Cardiovascular	Normal		
S136	109	JAIVAC-1	Dosage A	2	Day 28			23/09/2010	Yes	Respiratory	Normal		
S136	109	JAIVAC-1	Dosage A	2	Day 28			23/09/2010	Yes	Musculoskeletal	Normal		
S136	109	JAIVAC-1	Dosage A	2	Day 28			23/09/2010	Yes	Venous system	Normal		
S136	109	JAIVAC-1	Dosage A	2	Day 28			23/09/2010	Yes	Gastrointestinal	Normal		
S136	109	JAIVAC-1	Dosage A	2	Day 28			23/09/2010	Yes	Lymphatic	Normal		
S136	109	JAIVAC-1	Dosage A	2	Day 28			23/09/2010	Yes	Neurological	Normal		
S136	109	JAIVAC-1	Dosage A	2	Day 28			23/09/2010	Yes	Genitourinary	Normal		
S136	109	JAIVAC-1	Dosage A	2	Day 28			23/09/2010	Yes	Dermatological	Normal		
S136	109	JAIVAC-1	Dosage A	2	Day 35			30/09/2010		Head and Neck	Normal		

Subject No.	Randomisation Number	Study Group	Dosage	Dose	Day	Not Done	Specify	Date of Assessment	*Was the assessment performed prior to vaccination?	Physical Examination Parameter	Result	Abnormality	Clinical Significance
S136	109	JAIVAC-1	Dosage A	2	Day 35			30/09/2010		Eyes	Normal		
S136	109	JAIVAC-1	Dosage A	2	Day 35			30/09/2010		Ears, nose and throat	Normal		
S136	109	JAIVAC-1	Dosage A	2	Day 35			30/09/2010		Cardiovascular	Normal		
S136	109	JAIVAC-1	Dosage A	2	Day 35			30/09/2010		Respiratory	Normal		
S136	109	JAIVAC-1	Dosage A	2	Day 35			30/09/2010		Musculoskeletal	Normal		
S136	109	JAIVAC-1	Dosage A	2	Day 35			30/09/2010		Venous system	Normal		
S136	109	JAIVAC-1	Dosage A	2	Day 35			30/09/2010		Gastrointestinal	Normal		
S136	109	JAIVAC-1	Dosage A	2	Day 35			30/09/2010		Lymphatic	Normal		
S136	109	JAIVAC-1	Dosage A	2	Day 35			30/09/2010		Neurological	Normal		
S136	109	JAIVAC-1	Dosage A	2	Day 35			30/09/2010		Genitourinary	Normal		
S136	109	JAIVAC-1	Dosage A	2	Day 35			30/09/2010		Dermatological	Normal		
S136	109	JAIVAC-1	Dosage A	2	Day 42			07/10/2010		Head and Neck	Normal		
S136	109	JAIVAC-1	Dosage A	2	Day 42			07/10/2010		Eyes	Normal		
S136	109	JAIVAC-1	Dosage A	2	Day 42			07/10/2010		Ears, nose and throat	Normal		
S136	109	JAIVAC-1	Dosage A	2	Day 42			07/10/2010		Cardiovascular	Normal		
S136	109	JAIVAC-1	Dosage A	2	Day 42			07/10/2010		Respiratory	Normal		
S136	109	JAIVAC-1	Dosage A	2	Day 42			07/10/2010		Musculoskeletal	Normal		
S136	109	JAIVAC-1	Dosage A	2	Day 42			07/10/2010		Venous system	Normal		
S136	109	JAIVAC-1	Dosage A	2	Day 42			07/10/2010		Gastrointestinal	Normal		

Subject No.	Randomisation Number	Study Group	Dosage	Dose	Day	Not Done	Specify	Date of Assessment	*Was the assessment performed prior to vaccination?	Physical Examination Parameter	Result	Abnormality	Clinical Significance
S136	109	JAIVAC-1	Dosage A	2	Day 42			07/10/2010		Lymphatic	Normal		
S136	109	JAIVAC-1	Dosage A	2	Day 42			07/10/2010		Neurological	Normal		
S136	109	JAIVAC-1	Dosage A	2	Day 42			07/10/2010		Genitourinary	Normal		
S136	109	JAIVAC-1	Dosage A	2	Day 42			07/10/2010		Dermatological	Normal		
S136	109	JAIVAC-1	Dosage A	2	Day 56			21/10/2010		Head and Neck	Normal		
S136	109	JAIVAC-1	Dosage A	2	Day 56			21/10/2010		Eyes	Normal		
S136	109	JAIVAC-1	Dosage A	2	Day 56			21/10/2010		Ears, nose and throat	Normal		
S136	109	JAIVAC-1	Dosage A	2	Day 56			21/10/2010		Cardiovascular	Normal		
S136	109	JAIVAC-1	Dosage A	2	Day 56			21/10/2010		Respiratory	Normal		
S136	109	JAIVAC-1	Dosage A	2	Day 56			21/10/2010		Musculoskeletal	Normal		
S136	109	JAIVAC-1	Dosage A	2	Day 56			21/10/2010		Venous system	Normal		
S136	109	JAIVAC-1	Dosage A	2	Day 56			21/10/2010		Gastrointestinal	Normal		
S136	109	JAIVAC-1	Dosage A	2	Day 56			21/10/2010		Lymphatic	Normal		
S136	109	JAIVAC-1	Dosage A	2	Day 56			21/10/2010		Neurological	Normal		
S136	109	JAIVAC-1	Dosage A	2	Day 56			21/10/2010		Genitourinary	Normal		
S136	109	JAIVAC-1	Dosage A	2	Day 56			21/10/2010		Dermatological	Normal		
S136	109	JAIVAC-1	Dosage A	3	Day 180			22/02/2011	Yes	Head and Neck	Normal		
S136	109	JAIVAC-1	Dosage A	3	Day 180			22/02/2011	Yes	Eyes	Normal		
S136	109	JAIVAC-1	Dosage A	3	Day 180			22/02/2011	Yes	Ears, nose and throat	Normal		

Subject No.	Randomisation Number	Study Group	Dosage	Dose	Day	Not Done	Specify	Date of Assessment	*Was the assessment performed prior to vaccination?	Physical Examination Parameter	Result	Abnormality	Clinical Significance
S136	109	JAIVAC-1	Dosage A	3	Day 180			22/02/2011	Yes	Cardiovascular	Normal		
S136	109	JAIVAC-1	Dosage A	3	Day 180			22/02/2011	Yes	Respiratory	Normal		
S136	109	JAIVAC-1	Dosage A	3	Day 180			22/02/2011	Yes	Musculoskeletal	Normal		
S136	109	JAIVAC-1	Dosage A	3	Day 180			22/02/2011	Yes	Venous system	Normal		
S136	109	JAIVAC-1	Dosage A	3	Day 180			22/02/2011	Yes	Gastrointestinal	Normal		
S136	109	JAIVAC-1	Dosage A	3	Day 180			22/02/2011	Yes	Lymphatic	Normal		
S136	109	JAIVAC-1	Dosage A	3	Day 180			22/02/2011	Yes	Neurological	Normal		
S136	109	JAIVAC-1	Dosage A	3	Day 180			22/02/2011	Yes	Genitourinary	Normal		
S136	109	JAIVAC-1	Dosage A	3	Day 180			22/02/2011	Yes	Dermatological	Normal		
S136	109	JAIVAC-1	Dosage A	3	Day 187			01/03/2011		Head and Neck	Normal		
S136	109	JAIVAC-1	Dosage A	3	Day 187			01/03/2011		Eyes	Normal		
S136	109	JAIVAC-1	Dosage A	3	Day 187			01/03/2011		Ears, nose and throat	Normal		
S136	109	JAIVAC-1	Dosage A	3	Day 187			01/03/2011		Cardiovascular	Normal		
S136	109	JAIVAC-1	Dosage A	3	Day 187			01/03/2011		Respiratory	Normal		
S136	109	JAIVAC-1	Dosage A	3	Day 187			01/03/2011		Musculoskeletal	Normal		
S136	109	JAIVAC-1	Dosage A	3	Day 187			01/03/2011		Venous system	Normal		
S136	109	JAIVAC-1	Dosage A	3	Day 187			01/03/2011		Gastrointestinal	Normal		
S136	109	JAIVAC-1	Dosage A	3	Day 187			01/03/2011		Lymphatic	Normal		
S136	109	JAIVAC-1	Dosage A	3	Day 187			01/03/2011		Neurological	Normal		

Subject No.	Randomisation Number	Study Group	Dosage	Dose	Day	Not Done	Specify	Date of Assessment	*Was the assessment performed prior to vaccination?	Physical Examination Parameter	Result	Abnormality	Clinical Significance
S136	109	JAIVAC-1	Dosage A	3	Day 187			01/03/2011		Genitourinary	Normal		
S136	109	JAIVAC-1	Dosage A	3	Day 187			01/03/2011		Dermatological	Normal		
S136	109	JAIVAC-1	Dosage A	3	Day 194			08/03/2011		Head and Neck	Normal		
S136	109	JAIVAC-1	Dosage A	3	Day 194			08/03/2011		Eyes	Normal		
S136	109	JAIVAC-1	Dosage A	3	Day 194			08/03/2011		Ears, nose and throat	Normal		
S136	109	JAIVAC-1	Dosage A	3	Day 194			08/03/2011		Cardiovascular	Normal		
S136	109	JAIVAC-1	Dosage A	3	Day 194			08/03/2011		Respiratory	Normal		
S136	109	JAIVAC-1	Dosage A	3	Day 194			08/03/2011		Musculoskeletal	Normal		
S136	109	JAIVAC-1	Dosage A	3	Day 194			08/03/2011		Venous system	Normal		
S136	109	JAIVAC-1	Dosage A	3	Day 194			08/03/2011		Gastrointestinal	Normal		
S136	109	JAIVAC-1	Dosage A	3	Day 194			08/03/2011		Lymphatic	Normal		
S136	109	JAIVAC-1	Dosage A	3	Day 194			08/03/2011		Neurological	Normal		
S136	109	JAIVAC-1	Dosage A	3	Day 194			08/03/2011		Genitourinary	Normal		
S136	109	JAIVAC-1	Dosage A	3	Day 194			08/03/2011		Dermatological	Normal		
S136	109	JAIVAC-1	Dosage A	3	Day 208			22/03/2011		Head and Neck	Normal		
S136	109	JAIVAC-1	Dosage A	3	Day 208			22/03/2011		Eyes	Normal		
S136	109	JAIVAC-1	Dosage A	3	Day 208			22/03/2011		Ears, nose and throat	Normal		
S136	109	JAIVAC-1	Dosage A	3	Day 208			22/03/2011		Cardiovascular	Normal		
S136	109	JAIVAC-1	Dosage A	3	Day 208			22/03/2011		Respiratory	Normal		

Subject No.	Randomisation Number	Study Group	Dosage	Dose	Day	Not Done	Specify	Date of Assessment	*Was the assessment performed prior to vaccination?	Physical Examination Parameter	Result	Abnormality	Clinical Significance
S136	109	JAIVAC-1	Dosage A	3	Day 208			22/03/2011		Musculoskeletal	Normal		
S136	109	JAIVAC-1	Dosage A	3	Day 208			22/03/2011		Venous system	Normal		
S136	109	JAIVAC-1	Dosage A	3	Day 208			22/03/2011		Gastrointestinal	Normal		
S136	109	JAIVAC-1	Dosage A	3	Day 208			22/03/2011		Lymphatic	Normal		
S136	109	JAIVAC-1	Dosage A	3	Day 208			22/03/2011		Neurological	Normal		
S136	109	JAIVAC-1	Dosage A	3	Day 208			22/03/2011		Genitourinary	Normal		
S136	109	JAIVAC-1	Dosage A	3	Day 208			22/03/2011		Dermatological	Normal		
S136	109	JAIVAC-1	Dosage A	3	Day 365			25/08/2011		Head and Neck	Normal		
S136	109	JAIVAC-1	Dosage A	3	Day 365			25/08/2011		Eyes	Normal		
S136	109	JAIVAC-1	Dosage A	3	Day 365			25/08/2011		Ears, nose and throat	Normal		
S136	109	JAIVAC-1	Dosage A	3	Day 365			25/08/2011		Cardiovascular	Normal		
S136	109	JAIVAC-1	Dosage A	3	Day 365			25/08/2011		Respiratory	Normal		
S136	109	JAIVAC-1	Dosage A	3	Day 365			25/08/2011		Musculoskeletal	Normal		
S136	109	JAIVAC-1	Dosage A	3	Day 365			25/08/2011		Venous system	Normal		
S136	109	JAIVAC-1	Dosage A	3	Day 365			25/08/2011		Gastrointestinal	Normal		
S136	109	JAIVAC-1	Dosage A	3	Day 365			25/08/2011		Lymphatic	Normal		
S136	109	JAIVAC-1	Dosage A	3	Day 365			25/08/2011		Neurological	Normal		
S136	109	JAIVAC-1	Dosage A	3	Day 365			25/08/2011		Genitourinary	Normal		
S136	109	JAIVAC-1	Dosage A	3	Day 365			25/08/2011		Dermatological	Normal		

Subject No.	Randomisation Number	Study Group	Dosage	Dose	Day	Not Done	Specify	Date of Assessment	*Was the assessment performed prior to vaccination?	Physical Examination Parameter	Result	Abnormality	Clinical Significance
S143	101	JAIVAC-1	Dosage A		Day -14 to Day -1			18/08/2010		Head and neck	Normal		
S143	101	JAIVAC-1	Dosage A		Day -14 to Day -1			18/08/2010		Eyes	Normal		
S143	101	JAIVAC-1	Dosage A		Day -14 to Day -1			18/08/2010		Ears, nose and throat	Normal		
S143	101	JAIVAC-1	Dosage A		Day -14 to Day -1			18/08/2010		Cardiovascular	Normal		
S143	101	JAIVAC-1	Dosage A		Day -14 to Day -1			18/08/2010		Respiratory	Normal		
S143	101	JAIVAC-1	Dosage A		Day -14 to Day -1			18/08/2010		Musculoskeletal	Normal		
S143	101	JAIVAC-1	Dosage A		Day -14 to Day -1			18/08/2010		Venous system	Normal		
S143	101	JAIVAC-1	Dosage A		Day -14 to Day -1			18/08/2010		Gastrointestinal	Normal		
S143	101	JAIVAC-1	Dosage A		Day -14 to Day -1			18/08/2010		Lymphatic	Normal		
S143	101	JAIVAC-1	Dosage A		Day -14 to Day -1			18/08/2010		Neurological	Normal		
S143	101	JAIVAC-1	Dosage A		Day -14 to Day -1			18/08/2010		Genitourinary	Normal		
S143	101	JAIVAC-1	Dosage A		Day -14 to Day -1			18/08/2010		Dermatological	Normal		
S143	101	JAIVAC-1	Dosage A	1	Day 0			24/08/2010	Yes	Head and neck	Normal		
S143	101	JAIVAC-1	Dosage A	1	Day 0			24/08/2010	Yes	Eyes	Normal		
S143	101	JAIVAC-1	Dosage A	1	Day 0			24/08/2010	Yes	Ears, nose and throat	Normal		
S143	101	JAIVAC-1	Dosage A	1	Day 0			24/08/2010	Yes	Cardiovascular	Normal		
S143	101	JAIVAC-1	Dosage A	1	Day 0			24/08/2010	Yes	Respiratory	Normal		

Subject No.	Randomisation Number	Study Group	Dosage	Dose	Day	Not Done	Specify	Date of Assessment	*Was the assessment performed prior to vaccination?	Physical Examination Parameter	Result	Abnormality	Clinical Significance
S143	101	JAIVAC-1	Dosage A	1	Day 0			24/08/2010	Yes	Musculoskeletal	Normal		
S143	101	JAIVAC-1	Dosage A	1	Day 0			24/08/2010	Yes	Venous system	Normal		
S143	101	JAIVAC-1	Dosage A	1	Day 0			24/08/2010	Yes	Gastrointestinal	Normal		
S143	101	JAIVAC-1	Dosage A	1	Day 0			24/08/2010	Yes	Lymphatic	Normal		
S143	101	JAIVAC-1	Dosage A	1	Day 0			24/08/2010	Yes	Neurological	Normal		
S143	101	JAIVAC-1	Dosage A	1	Day 0			24/08/2010	Yes	Genitourinary	Normal		
S143	101	JAIVAC-1	Dosage A	1	Day 0			24/08/2010	Yes	Dermatological	Normal		
S143	101	JAIVAC-1	Dosage A	1	Day 7			31/08/2010		Head and Neck	Normal		
S143	101	JAIVAC-1	Dosage A	1	Day 7			31/08/2010		Eyes	Normal		
S143	101	JAIVAC-1	Dosage A	1	Day 7			31/08/2010		Ears, nose and throat	Normal		
S143	101	JAIVAC-1	Dosage A	1	Day 7			31/08/2010		Cardiovascular	Normal		
S143	101	JAIVAC-1	Dosage A	1	Day 7			31/08/2010		Respiratory	Normal		
S143	101	JAIVAC-1	Dosage A	1	Day 7			31/08/2010		Musculoskeletal	Normal		
S143	101	JAIVAC-1	Dosage A	1	Day 7			31/08/2010		Venous system	Normal		
S143	101	JAIVAC-1	Dosage A	1	Day 7			31/08/2010		Gastrointestinal	Normal		
S143	101	JAIVAC-1	Dosage A	1	Day 7			31/08/2010		Lymphatic	Normal		
S143	101	JAIVAC-1	Dosage A	1	Day 7			31/08/2010		Neurological	Normal		
S143	101	JAIVAC-1	Dosage A	1	Day 7			31/08/2010		Genitourinary	Normal		
S143	101	JAIVAC-1	Dosage A	1	Day 7			31/08/2010		Dermatological	Normal		

Subject No.	Randomisation Number	Study Group	Dosage	Dose	Day	Not Done	Specify	Date of Assessment	*Was the assessment performed prior to vaccination?	Physical Examination Parameter	Result	Abnormality	Clinical Significance
S143	101	JAIVAC-1	Dosage A	1	Day 14			07/09/2010		Head and Neck	Normal		
S143	101	JAIVAC-1	Dosage A	1	Day 14			07/09/2010		Eyes	Normal		
S143	101	JAIVAC-1	Dosage A	1	Day 14			07/09/2010		Ears, nose and throat	Normal		
S143	101	JAIVAC-1	Dosage A	1	Day 14			07/09/2010		Cardiovascular	Normal		
S143	101	JAIVAC-1	Dosage A	1	Day 14			07/09/2010		Respiratory	Normal		
S143	101	JAIVAC-1	Dosage A	1	Day 14			07/09/2010		Musculoskeletal	Normal		
S143	101	JAIVAC-1	Dosage A	1	Day 14			07/09/2010		Venous system	Normal		
S143	101	JAIVAC-1	Dosage A	1	Day 14			07/09/2010		Gastrointestinal	Normal		
S143	101	JAIVAC-1	Dosage A	1	Day 14			07/09/2010		Lymphatic	Normal		
S143	101	JAIVAC-1	Dosage A	1	Day 14			07/09/2010		Neurological	Normal		
S143	101	JAIVAC-1	Dosage A	1	Day 14			07/09/2010		Genitourinary	Normal		
S143	101	JAIVAC-1	Dosage A	1	Day 14			07/09/2010		Dermatological	Normal		
S143	101	JAIVAC-1	Dosage A	2	Day 28			21/09/2010	Yes	Head and Neck	Normal		
S143	101	JAIVAC-1	Dosage A	2	Day 28			21/09/2010	Yes	Eyes	Normal		
S143	101	JAIVAC-1	Dosage A	2	Day 28			21/09/2010	Yes	Ears, nose and throat	Normal		
S143	101	JAIVAC-1	Dosage A	2	Day 28			21/09/2010	Yes	Cardiovascular	Normal		
S143	101	JAIVAC-1	Dosage A	2	Day 28			21/09/2010	Yes	Respiratory	Normal		
S143	101	JAIVAC-1	Dosage A	2	Day 28			21/09/2010	Yes	Musculoskeletal	Normal		
S143	101	JAIVAC-1	Dosage A	2	Day 28			21/09/2010	Yes	Venous system	Normal		

Subject No.	Randomisation Number	Study Group	Dosage	Dose	Day	Not Done	Specify	Date of Assessment	*Was the assessment performed prior to vaccination?	Physical Examination Parameter	Result	Abnormality	Clinical Significance
S143	101	JAIVAC-1	Dosage A	2	Day 28			21/09/2010	Yes	Gastrointestinal	Normal		
S143	101	JAIVAC-1	Dosage A	2	Day 28			21/09/2010	Yes	Lymphatic	Normal		
S143	101	JAIVAC-1	Dosage A	2	Day 28			21/09/2010	Yes	Neurological	Normal		
S143	101	JAIVAC-1	Dosage A	2	Day 28			21/09/2010	Yes	Genitourinary	Normal		
S143	101	JAIVAC-1	Dosage A	2	Day 28			21/09/2010	Yes	Dermatological	Normal		
S143	101	JAIVAC-1	Dosage A	2	Day 35			28/09/2010		Head and Neck	Normal		
S143	101	JAIVAC-1	Dosage A	2	Day 35			28/09/2010		Eyes	Normal		
S143	101	JAIVAC-1	Dosage A	2	Day 35			28/09/2010		Ears, nose and throat	Normal		
S143	101	JAIVAC-1	Dosage A	2	Day 35			28/09/2010		Cardiovascular	Normal		
S143	101	JAIVAC-1	Dosage A	2	Day 35			28/09/2010		Respiratory	Normal		
S143	101	JAIVAC-1	Dosage A	2	Day 35			28/09/2010		Musculoskeletal	Normal		
S143	101	JAIVAC-1	Dosage A	2	Day 35			28/09/2010		Venous system	Normal		
S143	101	JAIVAC-1	Dosage A	2	Day 35			28/09/2010		Gastrointestinal	Normal		
S143	101	JAIVAC-1	Dosage A	2	Day 35			28/09/2010		Lymphatic	Normal		
S143	101	JAIVAC-1	Dosage A	2	Day 35			28/09/2010		Neurological	Normal		
S143	101	JAIVAC-1	Dosage A	2	Day 35			28/09/2010		Genitourinary	Normal		
S143	101	JAIVAC-1	Dosage A	2	Day 35			28/09/2010		Dermatological	Normal		
S143	101	JAIVAC-1	Dosage A	2	Day 42			05/10/2010		Head and Neck	Normal		
S143	101	JAIVAC-1	Dosage A	2	Day 42			05/10/2010		Eyes	Normal		

Subject No.	Randomisation Number	Study Group	Dosage	Dose	Day	Not Done	Specify	Date of Assessment	*Was the assessment performed prior to vaccination?	Physical Examination Parameter	Result	Abnormality	Clinical Significance
S143	101	JAIVAC-1	Dosage A	2	Day 42			05/10/2010		Ears, nose and throat	Normal		
S143	101	JAIVAC-1	Dosage A	2	Day 42			05/10/2010		Cardiovascular	Normal		
S143	101	JAIVAC-1	Dosage A	2	Day 42			05/10/2010		Respiratory	Normal		
S143	101	JAIVAC-1	Dosage A	2	Day 42			05/10/2010		Musculoskeletal	Normal		
S143	101	JAIVAC-1	Dosage A	2	Day 42			05/10/2010		Venous system	Normal		
S143	101	JAIVAC-1	Dosage A	2	Day 42			05/10/2010		Gastrointestinal	Normal		
S143	101	JAIVAC-1	Dosage A	2	Day 42			05/10/2010		Lymphatic	Normal		
S143	101	JAIVAC-1	Dosage A	2	Day 42			05/10/2010		Neurological	Normal		
S143	101	JAIVAC-1	Dosage A	2	Day 42			05/10/2010		Genitourinary	Normal		
S143	101	JAIVAC-1	Dosage A	2	Day 42			05/10/2010		Dermatological	Normal		
S143	101	JAIVAC-1	Dosage A	2	Day 56			19/10/2010		Head and Neck	Normal		
S143	101	JAIVAC-1	Dosage A	2	Day 56			19/10/2010		Eyes	Normal		
S143	101	JAIVAC-1	Dosage A	2	Day 56			19/10/2010		Ears, nose and throat	Normal		
S143	101	JAIVAC-1	Dosage A	2	Day 56			19/10/2010		Cardiovascular	Normal		
S143	101	JAIVAC-1	Dosage A	2	Day 56			19/10/2010		Respiratory	Normal		
S143	101	JAIVAC-1	Dosage A	2	Day 56			19/10/2010		Musculoskeletal	Normal		
S143	101	JAIVAC-1	Dosage A	2	Day 56			19/10/2010		Venous system	Normal		
S143	101	JAIVAC-1	Dosage A	2	Day 56			19/10/2010		Gastrointestinal	Normal		
S143	101	JAIVAC-1	Dosage A	2	Day 56			19/10/2010		Lymphatic	Normal		

Subject No.	Randomisation Number	Study Group	Dosage	Dose	Day	Not Done	Specify	Date of Assessment	*Was the assessment performed prior to vaccination?	Physical Examination Parameter	Result	Abnormality	Clinical Significance
S143	101	JAIVAC-1	Dosage A	2	Day 56			19/10/2010		Neurological	Normal		
S143	101	JAIVAC-1	Dosage A	2	Day 56			19/10/2010		Genitourinary	Normal		
S143	101	JAIVAC-1	Dosage A	2	Day 56			19/10/2010		Dermatological	Normal		
S143	101	JAIVAC-1	Dosage A	3	Day 180			21/02/2011	Yes	Head and Neck	Normal		
S143	101	JAIVAC-1	Dosage A	3	Day 180			21/02/2011	Yes	Eyes	Normal		
S143	101	JAIVAC-1	Dosage A	3	Day 180			21/02/2011	Yes	Ears, nose and throat	Normal		
S143	101	JAIVAC-1	Dosage A	3	Day 180			21/02/2011	Yes	Cardiovascular	Normal		
S143	101	JAIVAC-1	Dosage A	3	Day 180			21/02/2011	Yes	Respiratory	Normal		
S143	101	JAIVAC-1	Dosage A	3	Day 180			21/02/2011	Yes	Musculoskeletal	Normal		
S143	101	JAIVAC-1	Dosage A	3	Day 180			21/02/2011	Yes	Venous system	Normal		
S143	101	JAIVAC-1	Dosage A	3	Day 180			21/02/2011	Yes	Gastrointestinal	Normal		
S143	101	JAIVAC-1	Dosage A	3	Day 180			21/02/2011	Yes	Lymphatic	Normal		
S143	101	JAIVAC-1	Dosage A	3	Day 180			21/02/2011	Yes	Neurological	Normal		
S143	101	JAIVAC-1	Dosage A	3	Day 180			21/02/2011	Yes	Genitourinary	Normal		
S143	101	JAIVAC-1	Dosage A	3	Day 180			21/02/2011	Yes	Dermatological	Normal		
S143	101	JAIVAC-1	Dosage A	3	Day 187			28/02/2011		Head and Neck	Normal		
S143	101	JAIVAC-1	Dosage A	3	Day 187			28/02/2011		Eyes	Normal		
S143	101	JAIVAC-1	Dosage A	3	Day 187			28/02/2011		Ears, nose and throat	Normal		
S143	101	JAIVAC-1	Dosage A	3	Day 187			28/02/2011		Cardiovascular	Normal		

Subject No.	Randomisation Number	Study Group	Dosage	Dose	Day	Not Done	Specify	Date of Assessment	*Was the assessment performed prior to vaccination?	Physical Examination Parameter	Result	Abnormality	Clinical Significance
S143	101	JAIVAC-1	Dosage A	3	Day 187			28/02/2011		Respiratory	Normal		
S143	101	JAIVAC-1	Dosage A	3	Day 187			28/02/2011		Musculoskeletal	Normal		
S143	101	JAIVAC-1	Dosage A	3	Day 187			28/02/2011		Venous system	Normal		
S143	101	JAIVAC-1	Dosage A	3	Day 187			28/02/2011		Gastrointestinal	Normal		
S143	101	JAIVAC-1	Dosage A	3	Day 187			28/02/2011		Lymphatic	Normal		
S143	101	JAIVAC-1	Dosage A	3	Day 187			28/02/2011		Neurological	Normal		
S143	101	JAIVAC-1	Dosage A	3	Day 187			28/02/2011		Genitourinary	Normal		
S143	101	JAIVAC-1	Dosage A	3	Day 187			28/02/2011		Dermatological	Normal		
S143	101	JAIVAC-1	Dosage A	3	Day 194			07/03/2011		Head and Neck	Normal		
S143	101	JAIVAC-1	Dosage A	3	Day 194			07/03/2011		Eyes	Normal		
S143	101	JAIVAC-1	Dosage A	3	Day 194			07/03/2011		Ears, nose and throat	Normal		
S143	101	JAIVAC-1	Dosage A	3	Day 194			07/03/2011		Cardiovascular	Normal		
S143	101	JAIVAC-1	Dosage A	3	Day 194			07/03/2011		Respiratory	Normal		
S143	101	JAIVAC-1	Dosage A	3	Day 194			07/03/2011		Musculoskeletal	Normal		
S143	101	JAIVAC-1	Dosage A	3	Day 194			07/03/2011		Venous system	Normal		
S143	101	JAIVAC-1	Dosage A	3	Day 194			07/03/2011		Gastrointestinal	Normal		
S143	101	JAIVAC-1	Dosage A	3	Day 194			07/03/2011		Lymphatic	Normal		
S143	101	JAIVAC-1	Dosage A	3	Day 194			07/03/2011		Neurological	Normal		
S143	101	JAIVAC-1	Dosage A	3	Day 194			07/03/2011		Genitourinary	Normal		

Subject No.	Randomisation Number	Study Group	Dosage	Dose	Day	Not Done	Specify	Date of Assessment	*Was the assessment performed prior to vaccination?	Physical Examination Parameter	Result	Abnormality	Clinical Significance
S143	101	JAIVAC-1	Dosage A	3	Day 194			07/03/2011		Dermatological	Normal		
S143	101	JAIVAC-1	Dosage A	3	Day 208			21/03/2011		Head and Neck	Normal		
S143	101	JAIVAC-1	Dosage A	3	Day 208			21/03/2011		Eyes	Normal		
S143	101	JAIVAC-1	Dosage A	3	Day 208			21/03/2011		Ears, nose and throat	Normal		
S143	101	JAIVAC-1	Dosage A	3	Day 208			21/03/2011		Cardiovascular	Normal		
S143	101	JAIVAC-1	Dosage A	3	Day 208			21/03/2011		Respiratory	Normal		
S143	101	JAIVAC-1	Dosage A	3	Day 208			21/03/2011		Musculoskeletal	Normal		
S143	101	JAIVAC-1	Dosage A	3	Day 208			21/03/2011		Venous system	Normal		
S143	101	JAIVAC-1	Dosage A	3	Day 208			21/03/2011		Gastrointestinal	Normal		
S143	101	JAIVAC-1	Dosage A	3	Day 208			21/03/2011		Lymphatic	Normal		
S143	101	JAIVAC-1	Dosage A	3	Day 208			21/03/2011		Neurological	Normal		
S143	101	JAIVAC-1	Dosage A	3	Day 208			21/03/2011		Genitourinary	Normal		
S143	101	JAIVAC-1	Dosage A	3	Day 208			21/03/2011		Dermatological	Normal		
S143	101	JAIVAC-1	Dosage A	3	Day 365			26/08/2011		Head and Neck	Normal		
S143	101	JAIVAC-1	Dosage A	3	Day 365			26/08/2011		Eyes	Normal		
S143	101	JAIVAC-1	Dosage A	3	Day 365			26/08/2011		Ears, nose and throat	Normal		
S143	101	JAIVAC-1	Dosage A	3	Day 365			26/08/2011		Cardiovascular	Normal		
S143	101	JAIVAC-1	Dosage A	3	Day 365			26/08/2011		Respiratory	Normal		
S143	101	JAIVAC-1	Dosage A	3	Day 365			26/08/2011		Musculoskeletal	Normal		

Subject No.	Randomisation Number	Study Group	Dosage	Dose	Day	Not Done	Specify	Date of Assessment	*Was the assessment performed prior to vaccination?	Physical Examination Parameter	Result	Abnormality	Clinical Significance
S143	101	JAIVAC-1	Dosage A	3	Day 365			26/08/2011		Venous system	Normal		
S143	101	JAIVAC-1	Dosage A	3	Day 365			26/08/2011		Gastrointestinal	Normal		
S143	101	JAIVAC-1	Dosage A	3	Day 365			26/08/2011		Lymphatic	Normal		
S143	101	JAIVAC-1	Dosage A	3	Day 365			26/08/2011		Neurological	Normal		
S143	101	JAIVAC-1	Dosage A	3	Day 365			26/08/2011		Genitourinary	Normal		
S143	101	JAIVAC-1	Dosage A	3	Day 365			26/08/2011		Dermatological	Normal		
S144	103	Hepatitis B	Hepatitis B		Day -14 to Day -1			19/08/2010		Head and neck	Normal		
S144	103	Hepatitis B	Hepatitis B		Day -14 to Day -1			19/08/2010		Eyes	Normal		
S144	103	Hepatitis B	Hepatitis B		Day -14 to Day -1			19/08/2010		Ears, nose and throat	Normal		
S144	103	Hepatitis B	Hepatitis B		Day -14 to Day -1			19/08/2010		Cardiovascular	Normal		
S144	103	Hepatitis B	Hepatitis B		Day -14 to Day -1			19/08/2010		Respiratory	Normal		
S144	103	Hepatitis B	Hepatitis B		Day -14 to Day -1			19/08/2010		Musculoskeletal	Normal		
S144	103	Hepatitis B	Hepatitis B		Day -14 to Day -1			19/08/2010		Venous system	Normal		
S144	103	Hepatitis B	Hepatitis B		Day -14 to Day -1			19/08/2010		Gastrointestinal	Normal		
S144	103	Hepatitis B	Hepatitis B		Day -14 to Day -1			19/08/2010		Lymphatic	Normal		
S144	103	Hepatitis B	Hepatitis B		Day -14 to Day -1			19/08/2010		Neurological	Normal		
S144	103	Hepatitis B	Hepatitis B		Day -14 to Day -1			19/08/2010		Genitourinary	Normal		

Subject No.	Randomisation Number	Study Group	Dosage	Dose	Day	Not Done	Specify	Date of Assessment	*Was the assessment performed prior to vaccination?	Physical Examination Parameter	Result	Abnormality	Clinical Significance
S144	103	Hepatitis B	Hepatitis B		Day -14 to Day -1			19/08/2010		Dermatological	Normal		
S144	103	Hepatitis B	Hepatitis B	1	Day 0			24/08/2010	Yes	Head and neck	Normal		
S144	103	Hepatitis B	Hepatitis B	1	Day 0			24/08/2010	Yes	Eyes	Normal		
S144	103	Hepatitis B	Hepatitis B	1	Day 0			24/08/2010	Yes	Ears, nose and throat	Normal		
S144	103	Hepatitis B	Hepatitis B	1	Day 0			24/08/2010	Yes	Cardiovascular	Normal		
S144	103	Hepatitis B	Hepatitis B	1	Day 0			24/08/2010	Yes	Respiratory	Normal		
S144	103	Hepatitis B	Hepatitis B	1	Day 0			24/08/2010	Yes	Musculoskeletal	Normal		
S144	103	Hepatitis B	Hepatitis B	1	Day 0			24/08/2010	Yes	Venous system	Normal		
S144	103	Hepatitis B	Hepatitis B	1	Day 0			24/08/2010	Yes	Gastrointestinal	Normal		
S144	103	Hepatitis B	Hepatitis B	1	Day 0			24/08/2010	Yes	Lymphatic	Normal		
S144	103	Hepatitis B	Hepatitis B	1	Day 0			24/08/2010	Yes	Neurological	Normal		
S144	103	Hepatitis B	Hepatitis B	1	Day 0			24/08/2010	Yes	Genitourinary	Normal		
S144	103	Hepatitis B	Hepatitis B	1	Day 0			24/08/2010	Yes	Dermatological	Normal		
S144	103	Hepatitis B	Hepatitis B	1	Day 7			31/08/2010		Head and Neck	Normal		
S144	103	Hepatitis B	Hepatitis B	1	Day 7			31/08/2010		Eyes	Normal		
S144	103	Hepatitis B	Hepatitis B	1	Day 7			31/08/2010		Ears, nose and throat	Normal		
S144	103	Hepatitis B	Hepatitis B	1	Day 7			31/08/2010		Cardiovascular	Normal		
S144	103	Hepatitis B	Hepatitis B	1	Day 7			31/08/2010		Respiratory	Normal		
S144	103	Hepatitis B	Hepatitis B	1	Day 7			31/08/2010		Musculoskeletal	Normal		

Subject No.	Randomisation Number	Study Group	Dosage	Dose	Day	Not Done	Specify	Date of Assessment	*Was the assessment performed prior to vaccination?	Physical Examination Parameter	Result	Abnormality	Clinical Significance
S144	103	Hepatitis B	Hepatitis B	1	Day 7			31/08/2010		Venous system	Normal		
S144	103	Hepatitis B	Hepatitis B	1	Day 7			31/08/2010		Gastrointestinal	Normal		
S144	103	Hepatitis B	Hepatitis B	1	Day 7			31/08/2010		Lymphatic	Normal		
S144	103	Hepatitis B	Hepatitis B	1	Day 7			31/08/2010		Neurological	Normal		
S144	103	Hepatitis B	Hepatitis B	1	Day 7			31/08/2010		Genitourinary	Normal		
S144	103	Hepatitis B	Hepatitis B	1	Day 7			31/08/2010		Dermatological	Normal		
S144	103	Hepatitis B	Hepatitis B	1	Day 14			07/09/2010		Head and Neck	Normal		
S144	103	Hepatitis B	Hepatitis B	1	Day 14			07/09/2010		Eyes	Normal		
S144	103	Hepatitis B	Hepatitis B	1	Day 14			07/09/2010		Ears, nose and throat	Normal		
S144	103	Hepatitis B	Hepatitis B	1	Day 14			07/09/2010		Cardiovascular	Normal		
S144	103	Hepatitis B	Hepatitis B	1	Day 14			07/09/2010		Respiratory	Normal		
S144	103	Hepatitis B	Hepatitis B	1	Day 14			07/09/2010		Musculoskeletal	Normal		
S144	103	Hepatitis B	Hepatitis B	1	Day 14			07/09/2010		Venous system	Normal		
S144	103	Hepatitis B	Hepatitis B	1	Day 14			07/09/2010		Gastrointestinal	Normal		
S144	103	Hepatitis B	Hepatitis B	1	Day 14			07/09/2010		Lymphatic	Normal		
S144	103	Hepatitis B	Hepatitis B	1	Day 14			07/09/2010		Neurological	Normal		
S144	103	Hepatitis B	Hepatitis B	1	Day 14			07/09/2010		Genitourinary	Normal		
S144	103	Hepatitis B	Hepatitis B	1	Day 14			07/09/2010		Dermatological	Normal		
S144	103	Hepatitis B	Hepatitis B	2	Day 28			21/09/2010	Yes	Head and Neck	Normal		

Subject No.	Randomisation Number	Study Group	Dosage	Dose	Day	Not Done	Specify	Date of Assessment	*Was the assessment performed prior to vaccination?	Physical Examination Parameter	Result	Abnormality	Clinical Significance
S144	103	Hepatitis B	Hepatitis B	2	Day 28			21/09/2010	Yes	Eyes	Normal		
S144	103	Hepatitis B	Hepatitis B	2	Day 28			21/09/2010	Yes	Ears, nose and throat	Normal		
S144	103	Hepatitis B	Hepatitis B	2	Day 28			21/09/2010	Yes	Cardiovascular	Normal		
S144	103	Hepatitis B	Hepatitis B	2	Day 28			21/09/2010	Yes	Respiratory	Normal		
S144	103	Hepatitis B	Hepatitis B	2	Day 28			21/09/2010	Yes	Musculoskeletal	Normal		
S144	103	Hepatitis B	Hepatitis B	2	Day 28			21/09/2010	Yes	Venous system	Normal		
S144	103	Hepatitis B	Hepatitis B	2	Day 28			21/09/2010	Yes	Gastrointestinal	Normal		
S144	103	Hepatitis B	Hepatitis B	2	Day 28			21/09/2010	Yes	Lymphatic	Normal		
S144	103	Hepatitis B	Hepatitis B	2	Day 28			21/09/2010	Yes	Neurological	Normal		
S144	103	Hepatitis B	Hepatitis B	2	Day 28			21/09/2010	Yes	Genitourinary	Normal		
S144	103	Hepatitis B	Hepatitis B	2	Day 28			21/09/2010	Yes	Dermatological	Normal		
S144	103	Hepatitis B	Hepatitis B	2	Day 35			28/09/2010		Head and Neck	Normal		
S144	103	Hepatitis B	Hepatitis B	2	Day 35			28/09/2010		Eyes	Normal		
S144	103	Hepatitis B	Hepatitis B	2	Day 35			28/09/2010		Ears, nose and throat	Normal		
S144	103	Hepatitis B	Hepatitis B	2	Day 35			28/09/2010		Cardiovascular	Normal		
S144	103	Hepatitis B	Hepatitis B	2	Day 35			28/09/2010		Respiratory	Normal		
S144	103	Hepatitis B	Hepatitis B	2	Day 35			28/09/2010		Musculoskeletal	Normal		
S144	103	Hepatitis B	Hepatitis B	2	Day 35			28/09/2010		Venous system	Normal		
S144	103	Hepatitis B	Hepatitis B	2	Day 35			28/09/2010		Gastrointestinal	Normal		

Subject No.	Randomisation Number	Study Group	Dosage	Dose	Day	Not Done	Specify	Date of Assessment	*Was the assessment performed prior to vaccination?	Physical Examination Parameter	Result	Abnormality	Clinical Significance
S144	103	Hepatitis B	Hepatitis B	2	Day 35			28/09/2010		Lymphatic	Normal		
S144	103	Hepatitis B	Hepatitis B	2	Day 35			28/09/2010		Neurological	Normal		
S144	103	Hepatitis B	Hepatitis B	2	Day 35			28/09/2010		Genitourinary	Normal		
S144	103	Hepatitis B	Hepatitis B	2	Day 35			28/09/2010		Dermatological	Normal		
S144	103	Hepatitis B	Hepatitis B	2	Day 42			05/10/2010		Head and Neck	Normal		
S144	103	Hepatitis B	Hepatitis B	2	Day 42			05/10/2010		Eyes	Normal		
S144	103	Hepatitis B	Hepatitis B	2	Day 42			05/10/2010		Ears, nose and throat	Normal		
S144	103	Hepatitis B	Hepatitis B	2	Day 42			05/10/2010		Cardiovascular	Normal		
S144	103	Hepatitis B	Hepatitis B	2	Day 42			05/10/2010		Respiratory	Normal		
S144	103	Hepatitis B	Hepatitis B	2	Day 42			05/10/2010		Musculoskeletal	Normal		
S144	103	Hepatitis B	Hepatitis B	2	Day 42			05/10/2010		Venous system	Normal		
S144	103	Hepatitis B	Hepatitis B	2	Day 42			05/10/2010		Gastrointestinal	Normal		
S144	103	Hepatitis B	Hepatitis B	2	Day 42			05/10/2010		Lymphatic	Normal		
S144	103	Hepatitis B	Hepatitis B	2	Day 42			05/10/2010		Neurological	Normal		
S144	103	Hepatitis B	Hepatitis B	2	Day 42			05/10/2010		Genitourinary	Normal		
S144	103	Hepatitis B	Hepatitis B	2	Day 42			05/10/2010		Dermatological	Normal		
S144	103	Hepatitis B	Hepatitis B	2	Day 56			19/10/2010		Head and Neck	Normal		
S144	103	Hepatitis B	Hepatitis B	2	Day 56			19/10/2010		Eyes	Normal		
S144	103	Hepatitis B	Hepatitis B	2	Day 56			19/10/2010		Ears, nose and throat	Normal		

Subject No.	Randomisation Number	Study Group	Dosage	Dose	Day	Not Done	Specify	Date of Assessment	*Was the assessment performed prior to vaccination?	Physical Examination Parameter	Result	Abnormality	Clinical Significance
S144	103	Hepatitis B	Hepatitis B	2	Day 56			19/10/2010		Cardiovascular	Normal		
S144	103	Hepatitis B	Hepatitis B	2	Day 56			19/10/2010		Respiratory	Normal		
S144	103	Hepatitis B	Hepatitis B	2	Day 56			19/10/2010		Musculoskeletal	Normal		
S144	103	Hepatitis B	Hepatitis B	2	Day 56			19/10/2010		Venous system	Normal		
S144	103	Hepatitis B	Hepatitis B	2	Day 56			19/10/2010		Gastrointestinal	Normal		
S144	103	Hepatitis B	Hepatitis B	2	Day 56			19/10/2010		Lymphatic	Normal		
S144	103	Hepatitis B	Hepatitis B	2	Day 56			19/10/2010		Neurological	Normal		
S144	103	Hepatitis B	Hepatitis B	2	Day 56			19/10/2010		Genitourinary	Normal		
S144	103	Hepatitis B	Hepatitis B	2	Day 56			19/10/2010		Dermatological	Normal		
S144	103	Hepatitis B	Hepatitis B	3	Day 180			21/02/2011	Yes	Head and Neck	Normal		
S144	103	Hepatitis B	Hepatitis B	3	Day 180			21/02/2011	Yes	Eyes	Normal		
S144	103	Hepatitis B	Hepatitis B	3	Day 180			21/02/2011	Yes	Ears, nose and throat	Normal		
S144	103	Hepatitis B	Hepatitis B	3	Day 180			21/02/2011	Yes	Cardiovascular	Normal		
S144	103	Hepatitis B	Hepatitis B	3	Day 180			21/02/2011	Yes	Respiratory	Normal		
S144	103	Hepatitis B	Hepatitis B	3	Day 180			21/02/2011	Yes	Musculoskeletal	Normal		
S144	103	Hepatitis B	Hepatitis B	3	Day 180			21/02/2011	Yes	Venous system	Normal		
S144	103	Hepatitis B	Hepatitis B	3	Day 180			21/02/2011	Yes	Gastrointestinal	Normal		
S144	103	Hepatitis B	Hepatitis B	3	Day 180			21/02/2011	Yes	Lymphatic	Normal		
S144	103	Hepatitis B	Hepatitis B	3	Day 180			21/02/2011	Yes	Neurological	Normal		

Subject No.	Randomisation Number	Study Group	Dosage	Dose	Day	Not Done	Specify	Date of Assessment	*Was the assessment performed prior to vaccination?	Physical Examination Parameter	Result	Abnormality	Clinical Significance
S144	103	Hepatitis B	Hepatitis B	3	Day 180			21/02/2011	Yes	Genitourinary	Normal		
S144	103	Hepatitis B	Hepatitis B	3	Day 180			21/02/2011	Yes	Dermatological	Normal		
S144	103	Hepatitis B	Hepatitis B	3	Day 187			28/02/2011		Head and Neck	Normal		
S144	103	Hepatitis B	Hepatitis B	3	Day 187			28/02/2011		Eyes	Normal		
S144	103	Hepatitis B	Hepatitis B	3	Day 187			28/02/2011		Ears, nose and throat	Normal		
S144	103	Hepatitis B	Hepatitis B	3	Day 187			28/02/2011		Cardiovascular	Normal		
S144	103	Hepatitis B	Hepatitis B	3	Day 187			28/02/2011		Respiratory	Normal		
S144	103	Hepatitis B	Hepatitis B	3	Day 187			28/02/2011		Musculoskeletal	Normal		
S144	103	Hepatitis B	Hepatitis B	3	Day 187			28/02/2011		Venous system	Normal		
S144	103	Hepatitis B	Hepatitis B	3	Day 187			28/02/2011		Gastrointestinal	Normal		
S144	103	Hepatitis B	Hepatitis B	3	Day 187			28/02/2011		Lymphatic	Normal		
S144	103	Hepatitis B	Hepatitis B	3	Day 187			28/02/2011		Neurological	Normal		
S144	103	Hepatitis B	Hepatitis B	3	Day 187			28/02/2011		Genitourinary	Normal		
S144	103	Hepatitis B	Hepatitis B	3	Day 187			28/02/2011		Dermatological	Normal		
S144	103	Hepatitis B	Hepatitis B	3	Day 194			07/03/2011		Head and Neck	Normal		
S144	103	Hepatitis B	Hepatitis B	3	Day 194			07/03/2011		Eyes	Normal		
S144	103	Hepatitis B	Hepatitis B	3	Day 194			07/03/2011		Ears, nose and throat	Normal		
S144	103	Hepatitis B	Hepatitis B	3	Day 194			07/03/2011		Cardiovascular	Normal		
S144	103	Hepatitis B	Hepatitis B	3	Day 194			07/03/2011		Respiratory	Normal		

Subject No.	Randomisation Number	Study Group	Dosage	Dose	Day	Not Done	Specify	Date of Assessment	*Was the assessment performed prior to vaccination?	Physical Examination Parameter	Result	Abnormality	Clinical Significance
S144	103	Hepatitis B	Hepatitis B	3	Day 194			07/03/2011		Musculoskeletal	Normal		
S144	103	Hepatitis B	Hepatitis B	3	Day 194			07/03/2011		Venous system	Normal		
S144	103	Hepatitis B	Hepatitis B	3	Day 194			07/03/2011		Gastrointestinal	Normal		
S144	103	Hepatitis B	Hepatitis B	3	Day 194			07/03/2011		Lymphatic	Normal		
S144	103	Hepatitis B	Hepatitis B	3	Day 194			07/03/2011		Neurological	Normal		
S144	103	Hepatitis B	Hepatitis B	3	Day 194			07/03/2011		Genitourinary	Normal		
S144	103	Hepatitis B	Hepatitis B	3	Day 194			07/03/2011		Dermatological	Normal		
S144	103	Hepatitis B	Hepatitis B	3	Day 208			21/03/2011		Head and Neck	Normal		
S144	103	Hepatitis B	Hepatitis B	3	Day 208			21/03/2011		Eyes	Normal		
S144	103	Hepatitis B	Hepatitis B	3	Day 208			21/03/2011		Ears, nose and throat	Normal		
S144	103	Hepatitis B	Hepatitis B	3	Day 208			21/03/2011		Cardiovascular	Normal		
S144	103	Hepatitis B	Hepatitis B	3	Day 208			21/03/2011		Respiratory	Normal		
S144	103	Hepatitis B	Hepatitis B	3	Day 208			21/03/2011		Musculoskeletal	Normal		
S144	103	Hepatitis B	Hepatitis B	3	Day 208			21/03/2011		Venous system	Normal		
S144	103	Hepatitis B	Hepatitis B	3	Day 208			21/03/2011		Gastrointestinal	Normal		
S144	103	Hepatitis B	Hepatitis B	3	Day 208			21/03/2011		Lymphatic	Normal		
S144	103	Hepatitis B	Hepatitis B	3	Day 208			21/03/2011		Neurological	Normal		
S144	103	Hepatitis B	Hepatitis B	3	Day 208			21/03/2011		Genitourinary	Normal		
S144	103	Hepatitis B	Hepatitis B	3	Day 208			21/03/2011		Dermatological	Normal		

Subject No.	Randomisation Number	Study Group	Dosage	Dose	Day	Not Done	Specify	Date of Assessment	*Was the assessment performed prior to vaccination?	Physical Examination Parameter	Result	Abnormality	Clinical Significance
S144	103	Hepatitis B	Hepatitis B	3	Day 365			26/08/2011		Head and Neck	Normal		
S144	103	Hepatitis B	Hepatitis B	3	Day 365			26/08/2011		Eyes	Normal		
S144	103	Hepatitis B	Hepatitis B	3	Day 365			26/08/2011		Ears, nose and throat	Normal		
S144	103	Hepatitis B	Hepatitis B	3	Day 365			26/08/2011		Cardiovascular	Normal		
S144	103	Hepatitis B	Hepatitis B	3	Day 365			26/08/2011		Respiratory	Normal		
S144	103	Hepatitis B	Hepatitis B	3	Day 365			26/08/2011		Musculoskeletal	Normal		
S144	103	Hepatitis B	Hepatitis B	3	Day 365			26/08/2011		Venous system	Normal		
S144	103	Hepatitis B	Hepatitis B	3	Day 365			26/08/2011		Gastrointestinal	Normal		
S144	103	Hepatitis B	Hepatitis B	3	Day 365			26/08/2011		Lymphatic	Normal		
S144	103	Hepatitis B	Hepatitis B	3	Day 365			26/08/2011		Neurological	Normal		
S144	103	Hepatitis B	Hepatitis B	3	Day 365			26/08/2011		Genitourinary	Normal		
S144	103	Hepatitis B	Hepatitis B	3	Day 365			26/08/2011		Dermatological	Normal		
S145	112	JAIVAC-1	Dosage A		Day -14 to Day -1			19/08/2010		Head and neck	Normal		
S145	112	JAIVAC-1	Dosage A		Day -14 to Day -1			19/08/2010		Eyes	Normal		
S145	112	JAIVAC-1	Dosage A		Day -14 to Day -1			19/08/2010		Ears, nose and throat	Normal		
S145	112	JAIVAC-1	Dosage A		Day -14 to Day -1			19/08/2010		Cardiovascular	Normal		
S145	112	JAIVAC-1	Dosage A		Day -14 to Day -1			19/08/2010		Respiratory	Normal		
S145	112	JAIVAC-1	Dosage A		Day -14 to Day -1			19/08/2010		Musculoskeletal	Normal		

Subject No.	Randomisation Number	Study Group	Dosage	Dose	Day	Not Done	Specify	Date of Assessment	*Was the assessment performed prior to vaccination?	Physical Examination Parameter	Result	Abnormality	Clinical Significance
S145	112	JAIVAC-1	Dosage A		Day -14 to Day -1			19/08/2010		Venous system	Normal		
S145	112	JAIVAC-1	Dosage A		Day -14 to Day -1			19/08/2010		Gastrointestinal	Normal		
S145	112	JAIVAC-1	Dosage A		Day -14 to Day -1			19/08/2010		Lymphatic	Normal		
S145	112	JAIVAC-1	Dosage A		Day -14 to Day -1			19/08/2010		Neurological	Normal		
S145	112	JAIVAC-1	Dosage A		Day -14 to Day -1			19/08/2010		Genitourinary	Normal		
S145	112	JAIVAC-1	Dosage A		Day -14 to Day -1			19/08/2010		Dermatological	Normal		
S145	112	JAIVAC-1	Dosage A	1	Day 0			27/08/2010	Yes	Head and neck	Normal		
S145	112	JAIVAC-1	Dosage A	1	Day 0			27/08/2010	Yes	Eyes	Normal		
S145	112	JAIVAC-1	Dosage A	1	Day 0			27/08/2010	Yes	Ears, nose and throat	Normal		
S145	112	JAIVAC-1	Dosage A	1	Day 0			27/08/2010	Yes	Cardiovascular	Normal		
S145	112	JAIVAC-1	Dosage A	1	Day 0			27/08/2010	Yes	Respiratory	Normal		
S145	112	JAIVAC-1	Dosage A	1	Day 0			27/08/2010	Yes	Musculoskeletal	Normal		
S145	112	JAIVAC-1	Dosage A	1	Day 0			27/08/2010	Yes	Venous system	Normal		
S145	112	JAIVAC-1	Dosage A	1	Day 0			27/08/2010	Yes	Gastrointestinal	Normal		
S145	112	JAIVAC-1	Dosage A	1	Day 0			27/08/2010	Yes	Lymphatic	Normal		
S145	112	JAIVAC-1	Dosage A	1	Day 0			27/08/2010	Yes	Neurological	Normal		
S145	112	JAIVAC-1	Dosage A	1	Day 0			27/08/2010	Yes	Genitourinary	Normal		
S145	112	JAIVAC-1	Dosage A	1	Day 0			27/08/2010	Yes	Dermatological	Normal		

Subject No.	Randomisation Number	Study Group	Dosage	Dose	Day	Not Done	Specify	Date of Assessment	*Was the assessment performed prior to vaccination?	Physical Examination Parameter	Result	Abnormality	Clinical Significance
S145	112	JAIVAC-1	Dosage A	1	Day 7			04/09/2010		Head and Neck	Normal		
S145	112	JAIVAC-1	Dosage A	1	Day 7			04/09/2010		Eyes	Normal		
S145	112	JAIVAC-1	Dosage A	1	Day 7			04/09/2010		Ears, nose and throat	Normal		
S145	112	JAIVAC-1	Dosage A	1	Day 7			04/09/2010		Cardiovascular	Normal		
S145	112	JAIVAC-1	Dosage A	1	Day 7			04/09/2010		Respiratory	Normal		
S145	112	JAIVAC-1	Dosage A	1	Day 7			04/09/2010		Musculoskeletal	Normal		
S145	112	JAIVAC-1	Dosage A	1	Day 7			04/09/2010		Venous system	Normal		
S145	112	JAIVAC-1	Dosage A	1	Day 7			04/09/2010		Gastrointestinal	Normal		
S145	112	JAIVAC-1	Dosage A	1	Day 7			04/09/2010		Lymphatic	Normal		
S145	112	JAIVAC-1	Dosage A	1	Day 7			04/09/2010		Neurological	Normal		
S145	112	JAIVAC-1	Dosage A	1	Day 7			04/09/2010		Genitourinary	Normal		
S145	112	JAIVAC-1	Dosage A	1	Day 7			04/09/2010		Dermatological	Normal		
S145	112	JAIVAC-1	Dosage A	1	Day 14			10/09/2010		Head and Neck	Normal		
S145	112	JAIVAC-1	Dosage A	1	Day 14			10/09/2010		Eyes	Normal		
S145	112	JAIVAC-1	Dosage A	1	Day 14			10/09/2010		Ears, nose and throat	Normal		
S145	112	JAIVAC-1	Dosage A	1	Day 14			10/09/2010		Cardiovascular	Normal		
S145	112	JAIVAC-1	Dosage A	1	Day 14			10/09/2010		Respiratory	Normal		
S145	112	JAIVAC-1	Dosage A	1	Day 14			10/09/2010		Musculoskeletal	Normal		
S145	112	JAIVAC-1	Dosage A	1	Day 14			10/09/2010		Venous system	Normal		

Subject No.	Randomisation Number	Study Group	Dosage	Dose	Day	Not Done	Specify	Date of Assessment	*Was the assessment performed prior to vaccination?	Physical Examination Parameter	Result	Abnormality	Clinical Significance
S145	112	JAIVAC-1	Dosage A	1	Day 14			10/09/2010		Gastrointestinal	Normal		
S145	112	JAIVAC-1	Dosage A	1	Day 14			10/09/2010		Lymphatic	Normal		
S145	112	JAIVAC-1	Dosage A	1	Day 14			10/09/2010		Neurological	Normal		
S145	112	JAIVAC-1	Dosage A	1	Day 14			10/09/2010		Genitourinary	Normal		
S145	112	JAIVAC-1	Dosage A	1	Day 14			10/09/2010		Dermatological	Normal		
S145	112	JAIVAC-1	Dosage A	2	Day 28			24/09/2010	Yes	Head and Neck	Normal		
S145	112	JAIVAC-1	Dosage A	2	Day 28			24/09/2010	Yes	Eyes	Normal		
S145	112	JAIVAC-1	Dosage A	2	Day 28			24/09/2010	Yes	Ears, nose and throat	Normal		
S145	112	JAIVAC-1	Dosage A	2	Day 28			24/09/2010	Yes	Cardiovascular	Normal		
S145	112	JAIVAC-1	Dosage A	2	Day 28			24/09/2010	Yes	Respiratory	Normal		
S145	112	JAIVAC-1	Dosage A	2	Day 28			24/09/2010	Yes	Musculoskeletal	Normal		
S145	112	JAIVAC-1	Dosage A	2	Day 28			24/09/2010	Yes	Venous system	Normal		
S145	112	JAIVAC-1	Dosage A	2	Day 28			24/09/2010	Yes	Gastrointestinal	Normal		
S145	112	JAIVAC-1	Dosage A	2	Day 28			24/09/2010	Yes	Lymphatic	Normal		
S145	112	JAIVAC-1	Dosage A	2	Day 28			24/09/2010	Yes	Neurological	Normal		
S145	112	JAIVAC-1	Dosage A	2	Day 28			24/09/2010	Yes	Genitourinary	Normal		
S145	112	JAIVAC-1	Dosage A	2	Day 28			24/09/2010	Yes	Dermatological	Normal		
S145	112	JAIVAC-1	Dosage A	2	Day 35			30/09/2010		Head and Neck	Normal		
S145	112	JAIVAC-1	Dosage A	2	Day 35			30/09/2010		Eyes	Normal		

Subject No.	Randomisation Number	Study Group	Dosage	Dose	Day	Not Done	Specify	Date of Assessment	*Was the assessment performed prior to vaccination?	Physical Examination Parameter	Result	Abnormality	Clinical Significance
S145	112	JAIVAC-1	Dosage A	2	Day 35			30/09/2010		Ears, nose and throat	Normal		
S145	112	JAIVAC-1	Dosage A	2	Day 35			30/09/2010		Cardiovascular	Normal		
S145	112	JAIVAC-1	Dosage A	2	Day 35			30/09/2010		Respiratory	Normal		
S145	112	JAIVAC-1	Dosage A	2	Day 35			30/09/2010		Musculoskeletal	Normal		
S145	112	JAIVAC-1	Dosage A	2	Day 35			30/09/2010		Venous system	Normal		
S145	112	JAIVAC-1	Dosage A	2	Day 35			30/09/2010		Gastrointestinal	Normal		
S145	112	JAIVAC-1	Dosage A	2	Day 35			30/09/2010		Lymphatic	Normal		
S145	112	JAIVAC-1	Dosage A	2	Day 35			30/09/2010		Neurological	Normal		
S145	112	JAIVAC-1	Dosage A	2	Day 35			30/09/2010		Genitourinary	Normal		
S145	112	JAIVAC-1	Dosage A	2	Day 35			30/09/2010		Dermatological	Normal		
S145	112	JAIVAC-1	Dosage A	2	Day 42			08/10/2010		Head and Neck	Normal		
S145	112	JAIVAC-1	Dosage A	2	Day 42			08/10/2010		Eyes	Normal		
S145	112	JAIVAC-1	Dosage A	2	Day 42			08/10/2010		Ears, nose and throat	Normal		
S145	112	JAIVAC-1	Dosage A	2	Day 42			08/10/2010		Cardiovascular	Normal		
S145	112	JAIVAC-1	Dosage A	2	Day 42			08/10/2010		Respiratory	Normal		
S145	112	JAIVAC-1	Dosage A	2	Day 42			08/10/2010		Musculoskeletal	Normal		
S145	112	JAIVAC-1	Dosage A	2	Day 42			08/10/2010		Venous system	Normal		
S145	112	JAIVAC-1	Dosage A	2	Day 42			08/10/2010		Gastrointestinal	Normal		
S145	112	JAIVAC-1	Dosage A	2	Day 42			08/10/2010		Lymphatic	Normal		

Subject No.	Randomisation Number	Study Group	Dosage	Dose	Day	Not Done	Specify	Date of Assessment	*Was the assessment performed prior to vaccination?	Physical Examination Parameter	Result	Abnormality	Clinical Significance
S145	112	JAIVAC-1	Dosage A	2	Day 42			08/10/2010		Neurological	Normal		
S145	112	JAIVAC-1	Dosage A	2	Day 42			08/10/2010		Genitourinary	Normal		
S145	112	JAIVAC-1	Dosage A	2	Day 42			08/10/2010		Dermatological	Normal		
S145	112	JAIVAC-1	Dosage A	2	Day 56			22/10/2010		Head and Neck	Normal		
S145	112	JAIVAC-1	Dosage A	2	Day 56			22/10/2010		Eyes	Normal		
S145	112	JAIVAC-1	Dosage A	2	Day 56			22/10/2010		Ears, nose and throat	Normal		
S145	112	JAIVAC-1	Dosage A	2	Day 56			22/10/2010		Cardiovascular	Normal		
S145	112	JAIVAC-1	Dosage A	2	Day 56			22/10/2010		Respiratory	Normal		
S145	112	JAIVAC-1	Dosage A	2	Day 56			22/10/2010		Musculoskeletal	Normal		
S145	112	JAIVAC-1	Dosage A	2	Day 56			22/10/2010		Venous system	Normal		
S145	112	JAIVAC-1	Dosage A	2	Day 56			22/10/2010		Gastrointestinal	Normal		
S145	112	JAIVAC-1	Dosage A	2	Day 56			22/10/2010		Lymphatic	Normal		
S145	112	JAIVAC-1	Dosage A	2	Day 56			22/10/2010		Neurological	Normal		
S145	112	JAIVAC-1	Dosage A	2	Day 56			22/10/2010		Genitourinary	Normal		
S145	112	JAIVAC-1	Dosage A	2	Day 56			22/10/2010		Dermatological	Normal		
S145	112	JAIVAC-1	Dosage A	3	Day 180			23/02/2011	Yes	Head and Neck	Normal		
S145	112	JAIVAC-1	Dosage A	3	Day 180			23/02/2011	Yes	Eyes	Normal		
S145	112	JAIVAC-1	Dosage A	3	Day 180			23/02/2011	Yes	Ears, nose and throat	Normal		
S145	112	JAIVAC-1	Dosage A	3	Day 180			23/02/2011	Yes	Cardiovascular	Normal		

Subject No.	Randomisation Number	Study Group	Dosage	Dose	Day	Not Done	Specify	Date of Assessment	*Was the assessment performed prior to vaccination?	Physical Examination Parameter	Result	Abnormality	Clinical Significance
S145	112	JAIVAC-1	Dosage A	3	Day 180			23/02/2011	Yes	Respiratory	Normal		
S145	112	JAIVAC-1	Dosage A	3	Day 180			23/02/2011	Yes	Musculoskeletal	Normal		
S145	112	JAIVAC-1	Dosage A	3	Day 180			23/02/2011	Yes	Venous system	Normal		
S145	112	JAIVAC-1	Dosage A	3	Day 180			23/02/2011	Yes	Gastrointestinal	Normal		
S145	112	JAIVAC-1	Dosage A	3	Day 180			23/02/2011	Yes	Lymphatic	Normal		
S145	112	JAIVAC-1	Dosage A	3	Day 180			23/02/2011	Yes	Neurological	Normal		
S145	112	JAIVAC-1	Dosage A	3	Day 180			23/02/2011	Yes	Genitourinary	Normal		
S145	112	JAIVAC-1	Dosage A	3	Day 180			23/02/2011	Yes	Dermatological	Normal		
S145	112	JAIVAC-1	Dosage A	3	Day 187			02/03/2011		Head and Neck	Normal		
S145	112	JAIVAC-1	Dosage A	3	Day 187			02/03/2011		Eyes	Normal		
S145	112	JAIVAC-1	Dosage A	3	Day 187			02/03/2011		Ears, nose and throat	Normal		
S145	112	JAIVAC-1	Dosage A	3	Day 187			02/03/2011		Cardiovascular	Normal		
S145	112	JAIVAC-1	Dosage A	3	Day 187			02/03/2011		Respiratory	Normal		
S145	112	JAIVAC-1	Dosage A	3	Day 187			02/03/2011		Musculoskeletal	Normal		
S145	112	JAIVAC-1	Dosage A	3	Day 187			02/03/2011		Venous system	Normal		
S145	112	JAIVAC-1	Dosage A	3	Day 187			02/03/2011		Gastrointestinal	Normal		
S145	112	JAIVAC-1	Dosage A	3	Day 187			02/03/2011		Lymphatic	Normal		
S145	112	JAIVAC-1	Dosage A	3	Day 187			02/03/2011		Neurological	Normal		
S145	112	JAIVAC-1	Dosage A	3	Day 187			02/03/2011		Genitourinary	Normal		

Subject No.	Randomisation Number	Study Group	Dosage	Dose	Day	Not Done	Specify	Date of Assessment	*Was the assessment performed prior to vaccination?	Physical Examination Parameter	Result	Abnormality	Clinical Significance
S145	112	JAIVAC-1	Dosage A	3	Day 187			02/03/2011		Dermatological	Normal		
S145	112	JAIVAC-1	Dosage A	3	Day 194			09/03/2011		Head and Neck	Normal		
S145	112	JAIVAC-1	Dosage A	3	Day 194			09/03/2011		Eyes	Normal		
S145	112	JAIVAC-1	Dosage A	3	Day 194			09/03/2011		Ears, nose and throat	Normal		
S145	112	JAIVAC-1	Dosage A	3	Day 194			09/03/2011		Cardiovascular	Normal		
S145	112	JAIVAC-1	Dosage A	3	Day 194			09/03/2011		Respiratory	Normal		
S145	112	JAIVAC-1	Dosage A	3	Day 194			09/03/2011		Musculoskeletal	Normal		
S145	112	JAIVAC-1	Dosage A	3	Day 194			09/03/2011		Venous system	Normal		
S145	112	JAIVAC-1	Dosage A	3	Day 194			09/03/2011		Gastrointestinal	Normal		
S145	112	JAIVAC-1	Dosage A	3	Day 194			09/03/2011		Lymphatic	Normal		
S145	112	JAIVAC-1	Dosage A	3	Day 194			09/03/2011		Neurological	Normal		
S145	112	JAIVAC-1	Dosage A	3	Day 194			09/03/2011		Genitourinary	Normal		
S145	112	JAIVAC-1	Dosage A	3	Day 194			09/03/2011		Dermatological	Normal		
S145	112	JAIVAC-1	Dosage A	3	Day 208			23/03/2011		Head and Neck	Normal		
S145	112	JAIVAC-1	Dosage A	3	Day 208			23/03/2011		Eyes	Normal		
S145	112	JAIVAC-1	Dosage A	3	Day 208			23/03/2011		Ears, nose and throat	Normal		
S145	112	JAIVAC-1	Dosage A	3	Day 208			23/03/2011		Cardiovascular	Normal		
S145	112	JAIVAC-1	Dosage A	3	Day 208			23/03/2011		Respiratory	Normal		
S145	112	JAIVAC-1	Dosage A	3	Day 208			23/03/2011		Musculoskeletal	Normal		

Subject No.	Randomisation Number	Study Group	Dosage	Dose	Day	Not Done	Specify	Date of Assessment	*Was the assessment performed prior to vaccination?	Physical Examination Parameter	Result	Abnormality	Clinical Significance
S145	112	JAIVAC-1	Dosage A	3	Day 208			23/03/2011		Venous system	Normal		
S145	112	JAIVAC-1	Dosage A	3	Day 208			23/03/2011		Gastrointestinal	Normal		
S145	112	JAIVAC-1	Dosage A	3	Day 208			23/03/2011		Lymphatic	Normal		
S145	112	JAIVAC-1	Dosage A	3	Day 208			23/03/2011		Neurological	Normal		
S145	112	JAIVAC-1	Dosage A	3	Day 208			23/03/2011		Genitourinary	Normal		
S145	112	JAIVAC-1	Dosage A	3	Day 208			23/03/2011		Dermatological	Normal		
S145	112	JAIVAC-1	Dosage A	3	Day 365			26/08/2011		Head and Neck	Normal		
S145	112	JAIVAC-1	Dosage A	3	Day 365			26/08/2011		Eyes	Normal		
S145	112	JAIVAC-1	Dosage A	3	Day 365			26/08/2011		Ears, nose and throat	Normal		
S145	112	JAIVAC-1	Dosage A	3	Day 365			26/08/2011		Cardiovascular	Normal		
S145	112	JAIVAC-1	Dosage A	3	Day 365			26/08/2011		Respiratory	Normal		
S145	112	JAIVAC-1	Dosage A	3	Day 365			26/08/2011		Musculoskeletal	Normal		
S145	112	JAIVAC-1	Dosage A	3	Day 365			26/08/2011		Venous system	Normal		
S145	112	JAIVAC-1	Dosage A	3	Day 365			26/08/2011		Gastrointestinal	Normal		
S145	112	JAIVAC-1	Dosage A	3	Day 365			26/08/2011		Lymphatic	Normal		
S145	112	JAIVAC-1	Dosage A	3	Day 365			26/08/2011		Neurological	Normal		
S145	112	JAIVAC-1	Dosage A	3	Day 365			26/08/2011		Genitourinary	Normal		
S145	112	JAIVAC-1	Dosage A	3	Day 365			26/08/2011		Dermatological	Normal		
S148	111	JAIVAC-1	Dosage A		Day -14 to Day -1			20/08/2010		Head and neck	Normal		

Subject No.	Randomisation Number	Study Group	Dosage	Dose	Day	Not Done	Specify	Date of Assessment	*Was the assessment performed prior to vaccination?	Physical Examination Parameter	Result	Abnormality	Clinical Significance
S148	111	JAIVAC-1	Dosage A		Day -14 to Day -1			20/08/2010		Eyes	Normal		
S148	111	JAIVAC-1	Dosage A		Day -14 to Day -1			20/08/2010		Ears, nose and throat	Normal		
S148	111	JAIVAC-1	Dosage A		Day -14 to Day -1			20/08/2010		Cardiovascular	Normal		
S148	111	JAIVAC-1	Dosage A		Day -14 to Day -1			20/08/2010		Respiratory	Normal		
S148	111	JAIVAC-1	Dosage A		Day -14 to Day -1			20/08/2010		Musculoskeletal	Normal		
S148	111	JAIVAC-1	Dosage A		Day -14 to Day -1			20/08/2010		Venous system	Normal		
S148	111	JAIVAC-1	Dosage A		Day -14 to Day -1			20/08/2010		Gastrointestinal	Normal		
S148	111	JAIVAC-1	Dosage A		Day -14 to Day -1			20/08/2010		Lymphatic	Normal		
S148	111	JAIVAC-1	Dosage A		Day -14 to Day -1			20/08/2010		Neurological	Normal		
S148	111	JAIVAC-1	Dosage A		Day -14 to Day -1			20/08/2010		Genitourinary	Normal		
S148	111	JAIVAC-1	Dosage A		Day -14 to Day -1			20/08/2010		Dermatological	Normal		
S148	111	JAIVAC-1	Dosage A	1	Day 0			27/08/2010	Yes	Head and neck	Normal		
S148	111	JAIVAC-1	Dosage A	1	Day 0			27/08/2010	Yes	Eyes	Normal		
S148	111	JAIVAC-1	Dosage A	1	Day 0			27/08/2010	Yes	Ears, nose and throat	Normal		
S148	111	JAIVAC-1	Dosage A	1	Day 0			27/08/2010	Yes	Cardiovascular	Normal		
S148	111	JAIVAC-1	Dosage A	1	Day 0			27/08/2010	Yes	Respiratory	Normal		
S148	111	JAIVAC-1	Dosage A	1	Day 0			27/08/2010	Yes	Musculoskeletal	Normal		

Subject No.	Randomisation Number	Study Group	Dosage	Dose	Day	Not Done	Specify	Date of Assessment	*Was the assessment performed prior to vaccination?	Physical Examination Parameter	Result	Abnormality	Clinical Significance
S148	111	JAIVAC-1	Dosage A	1	Day 0			27/08/2010	Yes	Venous system	Normal		
S148	111	JAIVAC-1	Dosage A	1	Day 0			27/08/2010	Yes	Gastrointestinal	Normal		
S148	111	JAIVAC-1	Dosage A	1	Day 0			27/08/2010	Yes	Lymphatic	Normal		
S148	111	JAIVAC-1	Dosage A	1	Day 0			27/08/2010	Yes	Neurological	Normal		
S148	111	JAIVAC-1	Dosage A	1	Day 0			27/08/2010	Yes	Genitourinary	Normal		
S148	111	JAIVAC-1	Dosage A	1	Day 0			27/08/2010	Yes	Dermatological	Normal		
S148	111	JAIVAC-1	Dosage A	1	Day 7			03/09/2010		Head and Neck	Normal		
S148	111	JAIVAC-1	Dosage A	1	Day 7			03/09/2010		Eyes	Normal		
S148	111	JAIVAC-1	Dosage A	1	Day 7			03/09/2010		Ears, nose and throat	Normal		
S148	111	JAIVAC-1	Dosage A	1	Day 7			03/09/2010		Cardiovascular	Normal		
S148	111	JAIVAC-1	Dosage A	1	Day 7			03/09/2010		Respiratory	Normal		
S148	111	JAIVAC-1	Dosage A	1	Day 7			03/09/2010		Musculoskeletal	Normal		
S148	111	JAIVAC-1	Dosage A	1	Day 7			03/09/2010		Venous system	Normal		
S148	111	JAIVAC-1	Dosage A	1	Day 7			03/09/2010		Gastrointestinal	Normal		
S148	111	JAIVAC-1	Dosage A	1	Day 7			03/09/2010		Lymphatic	Normal		
S148	111	JAIVAC-1	Dosage A	1	Day 7			03/09/2010		Neurological	Normal		
S148	111	JAIVAC-1	Dosage A	1	Day 7			03/09/2010		Genitourinary	Normal		
S148	111	JAIVAC-1	Dosage A	1	Day 7			03/09/2010		Dermatological	Normal		
S148	111	JAIVAC-1	Dosage A	1	Day 14			10/09/2010		Head and Neck	Normal		

Subject No.	Randomisation Number	Study Group	Dosage	Dose	Day	Not Done	Specify	Date of Assessment	*Was the assessment performed prior to vaccination?	Physical Examination Parameter	Result	Abnormality	Clinical Significance
S148	111	JAIVAC-1	Dosage A	1	Day 14			10/09/2010		Eyes	Normal		
S148	111	JAIVAC-1	Dosage A	1	Day 14			10/09/2010		Ears, nose and throat	Normal		
S148	111	JAIVAC-1	Dosage A	1	Day 14			10/09/2010		Cardiovascular	Normal		
S148	111	JAIVAC-1	Dosage A	1	Day 14			10/09/2010		Respiratory	Normal		
S148	111	JAIVAC-1	Dosage A	1	Day 14			10/09/2010		Musculoskeletal	Abnormal	Mild Tenderness Over Left Lower Forearm	CS
S148	111	JAIVAC-1	Dosage A	1	Day 14			10/09/2010		Venous system	Normal		
S148	111	JAIVAC-1	Dosage A	1	Day 14			10/09/2010		Gastrointestinal	Normal		
S148	111	JAIVAC-1	Dosage A	1	Day 14			10/09/2010		Lymphatic	Normal		
S148	111	JAIVAC-1	Dosage A	1	Day 14			10/09/2010		Neurological	Normal		
S148	111	JAIVAC-1	Dosage A	1	Day 14			10/09/2010		Genitourinary	Normal		
S148	111	JAIVAC-1	Dosage A	1	Day 14			10/09/2010		Dermatological	Normal		
S148	111	JAIVAC-1	Dosage A	2	Day 28			24/09/2010	Yes	Head and Neck	Normal		
S148	111	JAIVAC-1	Dosage A	2	Day 28			24/09/2010	Yes	Eyes	Normal		
S148	111	JAIVAC-1	Dosage A	2	Day 28			24/09/2010	Yes	Ears, nose and throat	Normal		
S148	111	JAIVAC-1	Dosage A	2	Day 28			24/09/2010	Yes	Cardiovascular	Normal		
S148	111	JAIVAC-1	Dosage A	2	Day 28			24/09/2010	Yes	Respiratory	Normal		
S148	111	JAIVAC-1	Dosage A	2	Day 28			24/09/2010	Yes	Musculoskeletal	Normal		
S148	111	JAIVAC-1	Dosage A	2	Day 28			24/09/2010	Yes	Venous system	Normal		

Subject No.	Randomisation Number	Study Group	Dosage	Dose	Day	Not Done	Specify	Date of Assessment	*Was the assessment performed prior to vaccination?	Physical Examination Parameter	Result	Abnormality	Clinical Significance
S148	111	JAIVAC-1	Dosage A	2	Day 28			24/09/2010	Yes	Gastrointestinal	Normal		
S148	111	JAIVAC-1	Dosage A	2	Day 28			24/09/2010	Yes	Lymphatic	Normal		
S148	111	JAIVAC-1	Dosage A	2	Day 28			24/09/2010	Yes	Neurological	Normal		
S148	111	JAIVAC-1	Dosage A	2	Day 28			24/09/2010	Yes	Genitourinary	Normal		
S148	111	JAIVAC-1	Dosage A	2	Day 28			24/09/2010	Yes	Dermatological	Normal		
S148	111	JAIVAC-1	Dosage A	2	Day 35			30/09/2010		Head and Neck	Normal		
S148	111	JAIVAC-1	Dosage A	2	Day 35			30/09/2010		Eyes	Normal		
S148	111	JAIVAC-1	Dosage A	2	Day 35			30/09/2010		Ears, nose and throat	Normal		
S148	111	JAIVAC-1	Dosage A	2	Day 35			30/09/2010		Cardiovascular	Normal		
S148	111	JAIVAC-1	Dosage A	2	Day 35			30/09/2010		Respiratory	Normal		
S148	111	JAIVAC-1	Dosage A	2	Day 35			30/09/2010		Musculoskeletal	Normal		
S148	111	JAIVAC-1	Dosage A	2	Day 35			30/09/2010		Venous system	Normal		
S148	111	JAIVAC-1	Dosage A	2	Day 35			30/09/2010		Gastrointestinal	Normal		
S148	111	JAIVAC-1	Dosage A	2	Day 35			30/09/2010		Lymphatic	Normal		
S148	111	JAIVAC-1	Dosage A	2	Day 35			30/09/2010		Neurological	Normal		
S148	111	JAIVAC-1	Dosage A	2	Day 35			30/09/2010		Genitourinary	Normal		
S148	111	JAIVAC-1	Dosage A	2	Day 35			30/09/2010		Dermatological	Normal		
S148	111	JAIVAC-1	Dosage A	2	Day 42			08/10/2010		Head and Neck	Normal		
S148	111	JAIVAC-1	Dosage A	2	Day 42			08/10/2010		Eyes	Normal		

Subject No.	Randomisation Number	Study Group	Dosage	Dose	Day	Not Done	Specify	Date of Assessment	*Was the assessment performed prior to vaccination?	Physical Examination Parameter	Result	Abnormality	Clinical Significance
S148	111	JAIVAC-1	Dosage A	2	Day 42			08/10/2010		Ears, nose and throat	Normal		
S148	111	JAIVAC-1	Dosage A	2	Day 42			08/10/2010		Cardiovascular	Normal		
S148	111	JAIVAC-1	Dosage A	2	Day 42			08/10/2010		Respiratory	Normal		
S148	111	JAIVAC-1	Dosage A	2	Day 42			08/10/2010		Musculoskeletal	Normal		
S148	111	JAIVAC-1	Dosage A	2	Day 42			08/10/2010		Venous system	Normal		
S148	111	JAIVAC-1	Dosage A	2	Day 42			08/10/2010		Gastrointestinal	Normal		
S148	111	JAIVAC-1	Dosage A	2	Day 42			08/10/2010		Lymphatic	Normal		
S148	111	JAIVAC-1	Dosage A	2	Day 42			08/10/2010		Neurological	Normal		
S148	111	JAIVAC-1	Dosage A	2	Day 42			08/10/2010		Genitourinary	Normal		
S148	111	JAIVAC-1	Dosage A	2	Day 42			08/10/2010		Dermatological	Normal		
S148	111	JAIVAC-1	Dosage A	2	Day 56			22/10/2010		Head and Neck	Normal		
S148	111	JAIVAC-1	Dosage A	2	Day 56			22/10/2010		Eyes	Normal		
S148	111	JAIVAC-1	Dosage A	2	Day 56			22/10/2010		Ears, nose and throat	Normal		
S148	111	JAIVAC-1	Dosage A	2	Day 56			22/10/2010		Cardiovascular	Normal		
S148	111	JAIVAC-1	Dosage A	2	Day 56			22/10/2010		Respiratory	Normal		
S148	111	JAIVAC-1	Dosage A	2	Day 56			22/10/2010		Musculoskeletal	Normal		
S148	111	JAIVAC-1	Dosage A	2	Day 56			22/10/2010		Venous system	Normal		
S148	111	JAIVAC-1	Dosage A	2	Day 56			22/10/2010		Gastrointestinal	Normal		
S148	111	JAIVAC-1	Dosage A	2	Day 56			22/10/2010		Lymphatic	Normal		

Subject No.	Randomisation Number	Study Group	Dosage	Dose	Day	Not Done	Specify	Date of Assessment	*Was the assessment performed prior to vaccination?	Physical Examination Parameter	Result	Abnormality	Clinical Significance
S148	111	JAIVAC-1	Dosage A	2	Day 56			22/10/2010		Neurological	Normal		
S148	111	JAIVAC-1	Dosage A	2	Day 56			22/10/2010		Genitourinary	Normal		
S148	111	JAIVAC-1	Dosage A	2	Day 56			22/10/2010		Dermatological	Normal		
S148	111	JAIVAC-1	Dosage A	3	Day 180			23/02/2011	Yes	Head and Neck	Normal		
S148	111	JAIVAC-1	Dosage A	3	Day 180			23/02/2011	Yes	Eyes	Normal		
S148	111	JAIVAC-1	Dosage A	3	Day 180			23/02/2011	Yes	Ears, nose and throat	Normal		
S148	111	JAIVAC-1	Dosage A	3	Day 180			23/02/2011	Yes	Cardiovascular	Normal		
S148	111	JAIVAC-1	Dosage A	3	Day 180			23/02/2011	Yes	Respiratory	Normal		
S148	111	JAIVAC-1	Dosage A	3	Day 180			23/02/2011	Yes	Musculoskeletal	Normal		
S148	111	JAIVAC-1	Dosage A	3	Day 180			23/02/2011	Yes	Venous system	Normal		
S148	111	JAIVAC-1	Dosage A	3	Day 180			23/02/2011	Yes	Gastrointestinal	Normal		
S148	111	JAIVAC-1	Dosage A	3	Day 180			23/02/2011	Yes	Lymphatic	Normal		
S148	111	JAIVAC-1	Dosage A	3	Day 180			23/02/2011	Yes	Neurological	Normal		
S148	111	JAIVAC-1	Dosage A	3	Day 180			23/02/2011	Yes	Genitourinary	Normal		
S148	111	JAIVAC-1	Dosage A	3	Day 180			23/02/2011	Yes	Dermatological	Normal		
S148	111	JAIVAC-1	Dosage A	3	Day 187			03/03/2011		Head and Neck	Normal		
S148	111	JAIVAC-1	Dosage A	3	Day 187			03/03/2011		Eyes	Normal		
S148	111	JAIVAC-1	Dosage A	3	Day 187			03/03/2011		Ears, nose and throat	Normal		
S148	111	JAIVAC-1	Dosage A	3	Day 187			03/03/2011		Cardiovascular	Normal		

Subject No.	Randomisation Number	Study Group	Dosage	Dose	Day	Not Done	Specify	Date of Assessment	*Was the assessment performed prior to vaccination?	Physical Examination Parameter	Result	Abnormality	Clinical Significance
S148	111	JAIVAC-1	Dosage A	3	Day 187			03/03/2011		Respiratory	Normal		
S148	111	JAIVAC-1	Dosage A	3	Day 187			03/03/2011		Musculoskeletal	Normal		
S148	111	JAIVAC-1	Dosage A	3	Day 187			03/03/2011		Venous system	Normal		
S148	111	JAIVAC-1	Dosage A	3	Day 187			03/03/2011		Gastrointestinal	Normal		
S148	111	JAIVAC-1	Dosage A	3	Day 187			03/03/2011		Lymphatic	Normal		
S148	111	JAIVAC-1	Dosage A	3	Day 187			03/03/2011		Neurological	Normal		
S148	111	JAIVAC-1	Dosage A	3	Day 187			03/03/2011		Genitourinary	Normal		
S148	111	JAIVAC-1	Dosage A	3	Day 187			03/03/2011		Dermatological	Normal		
S148	111	JAIVAC-1	Dosage A	3	Day 194			09/03/2011		Head and Neck	Normal		
S148	111	JAIVAC-1	Dosage A	3	Day 194			09/03/2011		Eyes	Normal		
S148	111	JAIVAC-1	Dosage A	3	Day 194			09/03/2011		Ears, nose and throat	Normal		
S148	111	JAIVAC-1	Dosage A	3	Day 194			09/03/2011		Cardiovascular	Normal		
S148	111	JAIVAC-1	Dosage A	3	Day 194			09/03/2011		Respiratory	Normal		
S148	111	JAIVAC-1	Dosage A	3	Day 194			09/03/2011		Musculoskeletal	Normal		
S148	111	JAIVAC-1	Dosage A	3	Day 194			09/03/2011		Venous system	Normal		
S148	111	JAIVAC-1	Dosage A	3	Day 194			09/03/2011		Gastrointestinal	Normal		
S148	111	JAIVAC-1	Dosage A	3	Day 194			09/03/2011		Lymphatic	Normal		
S148	111	JAIVAC-1	Dosage A	3	Day 194			09/03/2011		Neurological	Normal		
S148	111	JAIVAC-1	Dosage A	3	Day 194			09/03/2011		Genitourinary	Normal		

Subject No.	Randomisation Number	Study Group	Dosage	Dose	Day	Not Done	Specify	Date of Assessment	*Was the assessment performed prior to vaccination ?	Physical Examination Parameter	Result	Abnormality	Clinical Significance
S148	111	JAIVAC-1	Dosage A	3	Day 194			09/03/2011		Dermatological	Normal		
S148	111	JAIVAC-1	Dosage A	3	Day 208			23/03/2011		Head and Neck	Normal		
S148	111	JAIVAC-1	Dosage A	3	Day 208			23/03/2011		Eyes	Normal		
S148	111	JAIVAC-1	Dosage A	3	Day 208			23/03/2011		Ears, nose and throat	Normal		
S148	111	JAIVAC-1	Dosage A	3	Day 208			23/03/2011		Cardiovascular	Normal		
S148	111	JAIVAC-1	Dosage A	3	Day 208			23/03/2011		Respiratory	Normal		
S148	111	JAIVAC-1	Dosage A	3	Day 208			23/03/2011		Musculoskeletal	Normal		
S148	111	JAIVAC-1	Dosage A	3	Day 208			23/03/2011		Venous system	Normal		
S148	111	JAIVAC-1	Dosage A	3	Day 208			23/03/2011		Gastrointestinal	Normal		
S148	111	JAIVAC-1	Dosage A	3	Day 208			23/03/2011		Lymphatic	Normal		
S148	111	JAIVAC-1	Dosage A	3	Day 208			23/03/2011		Neurological	Normal		
S148	111	JAIVAC-1	Dosage A	3	Day 208			23/03/2011		Genitourinary	Normal		
S148	111	JAIVAC-1	Dosage A	3	Day 208			23/03/2011		Dermatological	Normal		
S148	111	JAIVAC-1	Dosage A	3	Day 365			26/08/2011		Head and Neck	Normal		
S148	111	JAIVAC-1	Dosage A	3	Day 365			26/08/2011		Eyes	Normal		
S148	111	JAIVAC-1	Dosage A	3	Day 365			26/08/2011		Ears, nose and throat	Normal		
S148	111	JAIVAC-1	Dosage A	3	Day 365			26/08/2011		Cardiovascular	Normal		
S148	111	JAIVAC-1	Dosage A	3	Day 365			26/08/2011		Respiratory	Normal		
S148	111	JAIVAC-1	Dosage A	3	Day 365			26/08/2011		Musculoskeletal	Normal		

Subject No.	Randomisation Number	Study Group	Dosage	Dose	Day	Not Done	Specify	Date of Assessment	*Was the assessment performed prior to vaccination?	Physical Examination Parameter	Result	Abnormality	Clinical Significance
S148	111	JAIVAC-1	Dosage A	3	Day 365			26/08/2011		Venous system	Normal		
S148	111	JAIVAC-1	Dosage A	3	Day 365			26/08/2011		Gastrointestinal	Normal		
S148	111	JAIVAC-1	Dosage A	3	Day 365			26/08/2011		Lymphatic	Normal		
S148	111	JAIVAC-1	Dosage A	3	Day 365			26/08/2011		Neurological	Normal		
S148	111	JAIVAC-1	Dosage A	3	Day 365			26/08/2011		Genitourinary	Normal		
S148	111	JAIVAC-1	Dosage A	3	Day 365			26/08/2011		Dermatological	Normal		
S154	113	JAIVAC-1	Dosage A		Day -14 to Day -1			19/08/2010		Head and neck	Normal		
S154	113	JAIVAC-1	Dosage A		Day -14 to Day -1			19/08/2010		Eyes	Normal		
S154	113	JAIVAC-1	Dosage A		Day -14 to Day -1			19/08/2010		Ears, nose and throat	Normal		
S154	113	JAIVAC-1	Dosage A		Day -14 to Day -1			19/08/2010		Cardiovascular	Normal		
S154	113	JAIVAC-1	Dosage A		Day -14 to Day -1			19/08/2010		Respiratory	Normal		
S154	113	JAIVAC-1	Dosage A		Day -14 to Day -1			19/08/2010		Musculoskeletal	Normal		
S154	113	JAIVAC-1	Dosage A		Day -14 to Day -1			19/08/2010		Venous system	Normal		
S154	113	JAIVAC-1	Dosage A		Day -14 to Day -1			19/08/2010		Gastrointestinal	Normal		
S154	113	JAIVAC-1	Dosage A		Day -14 to Day -1			19/08/2010		Lymphatic	Normal		
S154	113	JAIVAC-1	Dosage A		Day -14 to Day -1			19/08/2010		Neurological	Normal		
S154	113	JAIVAC-1	Dosage A		Day -14 to Day -1			19/08/2010		Genitourinary	Normal		

Subject No.	Randomisation Number	Study Group	Dosage	Dose	Day	Not Done	Specify	Date of Assessment	*Was the assessment performed prior to vaccination?	Physical Examination Parameter	Result	Abnormality	Clinical Significance
S154	113	JAIVAC-1	Dosage A		Day -14 to Day -1			19/08/2010		Dermatological	Normal		
S154	113	JAIVAC-1	Dosage A	1	Day 0			27/08/2010	Yes	Head and neck	Normal		
S154	113	JAIVAC-1	Dosage A	1	Day 0			27/08/2010	Yes	Eyes	Normal		
S154	113	JAIVAC-1	Dosage A	1	Day 0			27/08/2010	Yes	Ears, nose and throat	Normal		
S154	113	JAIVAC-1	Dosage A	1	Day 0			27/08/2010	Yes	Cardiovascular	Normal		
S154	113	JAIVAC-1	Dosage A	1	Day 0			27/08/2010	Yes	Respiratory	Normal		
S154	113	JAIVAC-1	Dosage A	1	Day 0			27/08/2010	Yes	Musculoskeletal	Normal		
S154	113	JAIVAC-1	Dosage A	1	Day 0			27/08/2010	Yes	Venous system	Normal		
S154	113	JAIVAC-1	Dosage A	1	Day 0			27/08/2010	Yes	Gastrointestinal	Normal		
S154	113	JAIVAC-1	Dosage A	1	Day 0			27/08/2010	Yes	Lymphatic	Normal		
S154	113	JAIVAC-1	Dosage A	1	Day 0			27/08/2010	Yes	Neurological	Normal		
S154	113	JAIVAC-1	Dosage A	1	Day 0			27/08/2010	Yes	Genitourinary	Normal		
S154	113	JAIVAC-1	Dosage A	1	Day 0			27/08/2010	Yes	Dermatological	Normal		
S154	113	JAIVAC-1	Dosage A	1	Day 7			03/09/2010		Head and Neck	Normal		
S154	113	JAIVAC-1	Dosage A	1	Day 7			03/09/2010		Eyes	Normal		
S154	113	JAIVAC-1	Dosage A	1	Day 7			03/09/2010		Ears, nose and throat	Normal		
S154	113	JAIVAC-1	Dosage A	1	Day 7			03/09/2010		Cardiovascular	Normal		
S154	113	JAIVAC-1	Dosage A	1	Day 7			03/09/2010		Respiratory	Normal		
S154	113	JAIVAC-1	Dosage A	1	Day 7			03/09/2010		Musculoskeletal	Normal		

Subject No.	Randomisation Number	Study Group	Dosage	Dose	Day	Not Done	Specify	Date of Assessment	*Was the assessment performed prior to vaccination?	Physical Examination Parameter	Result	Abnormality	Clinical Significance
S154	113	JAIVAC-1	Dosage A	1	Day 7			03/09/2010		Venous system	Normal		
S154	113	JAIVAC-1	Dosage A	1	Day 7			03/09/2010		Gastrointestinal	Normal		
S154	113	JAIVAC-1	Dosage A	1	Day 7			03/09/2010		Lymphatic	Normal		
S154	113	JAIVAC-1	Dosage A	1	Day 7			03/09/2010		Neurological	Normal		
S154	113	JAIVAC-1	Dosage A	1	Day 7			03/09/2010		Genitourinary	Normal		
S154	113	JAIVAC-1	Dosage A	1	Day 7			03/09/2010		Dermatological	Normal		
S154	113	JAIVAC-1	Dosage A	1	Day 14			09/09/2010		Head and Neck	Normal		
S154	113	JAIVAC-1	Dosage A	1	Day 14			09/09/2010		Eyes	Normal		
S154	113	JAIVAC-1	Dosage A	1	Day 14			09/09/2010		Ears, nose and throat	Normal		
S154	113	JAIVAC-1	Dosage A	1	Day 14			09/09/2010		Cardiovascular	Normal		
S154	113	JAIVAC-1	Dosage A	1	Day 14			09/09/2010		Respiratory	Normal		
S154	113	JAIVAC-1	Dosage A	1	Day 14			09/09/2010		Musculoskeletal	Normal		
S154	113	JAIVAC-1	Dosage A	1	Day 14			09/09/2010		Venous system	Normal		
S154	113	JAIVAC-1	Dosage A	1	Day 14			09/09/2010		Gastrointestinal	Normal		
S154	113	JAIVAC-1	Dosage A	1	Day 14			09/09/2010		Lymphatic	Normal		
S154	113	JAIVAC-1	Dosage A	1	Day 14			09/09/2010		Neurological	Normal		
S154	113	JAIVAC-1	Dosage A	1	Day 14			09/09/2010		Genitourinary	Normal		
S154	113	JAIVAC-1	Dosage A	1	Day 14			09/09/2010		Dermatological	Normal		
S154	113	JAIVAC-1	Dosage A	2	Day 28			24/09/2010	Yes	Head and Neck	Normal		

Subject No.	Randomisation Number	Study Group	Dosage	Dose	Day	Not Done	Specify	Date of Assessment	*Was the assessment performed prior to vaccination?	Physical Examination Parameter	Result	Abnormality	Clinical Significance
S154	113	JAIVAC-1	Dosage A	2	Day 28			24/09/2010	Yes	Eyes	Normal		
S154	113	JAIVAC-1	Dosage A	2	Day 28			24/09/2010	Yes	Ears, nose and throat	Normal		
S154	113	JAIVAC-1	Dosage A	2	Day 28			24/09/2010	Yes	Cardiovascular	Normal		
S154	113	JAIVAC-1	Dosage A	2	Day 28			24/09/2010	Yes	Respiratory	Normal		
S154	113	JAIVAC-1	Dosage A	2	Day 28			24/09/2010	Yes	Musculoskeletal	Normal		
S154	113	JAIVAC-1	Dosage A	2	Day 28			24/09/2010	Yes	Venous system	Normal		
S154	113	JAIVAC-1	Dosage A	2	Day 28			24/09/2010	Yes	Gastrointestinal	Normal		
S154	113	JAIVAC-1	Dosage A	2	Day 28			24/09/2010	Yes	Lymphatic	Normal		
S154	113	JAIVAC-1	Dosage A	2	Day 28			24/09/2010	Yes	Neurological	Normal		
S154	113	JAIVAC-1	Dosage A	2	Day 28			24/09/2010	Yes	Genitourinary	Normal		
S154	113	JAIVAC-1	Dosage A	2	Day 28			24/09/2010	Yes	Dermatological	Normal		
S154	113	JAIVAC-1	Dosage A	2	Day 35			30/09/2010		Head and Neck	Normal		
S154	113	JAIVAC-1	Dosage A	2	Day 35			30/09/2010		Eyes	Normal		
S154	113	JAIVAC-1	Dosage A	2	Day 35			30/09/2010		Ears, nose and throat	Normal		
S154	113	JAIVAC-1	Dosage A	2	Day 35			30/09/2010		Cardiovascular	Normal		
S154	113	JAIVAC-1	Dosage A	2	Day 35			30/09/2010		Respiratory	Normal		
S154	113	JAIVAC-1	Dosage A	2	Day 35			30/09/2010		Musculoskeletal	Normal		
S154	113	JAIVAC-1	Dosage A	2	Day 35			30/09/2010		Venous system	Normal		
S154	113	JAIVAC-1	Dosage A	2	Day 35			30/09/2010		Gastrointestinal	Normal		

Subject No.	Randomisation Number	Study Group	Dosage	Dose	Day	Not Done	Specify	Date of Assessment	*Was the assessment performed prior to vaccination?	Physical Examination Parameter	Result	Abnormality	Clinical Significance
S154	113	JAIVAC-1	Dosage A	2	Day 35			30/09/2010		Lymphatic	Normal		
S154	113	JAIVAC-1	Dosage A	2	Day 35			30/09/2010		Neurological	Normal		
S154	113	JAIVAC-1	Dosage A	2	Day 35			30/09/2010		Genitourinary	Normal		
S154	113	JAIVAC-1	Dosage A	2	Day 35			30/09/2010		Dermatological	Normal		
S154	113	JAIVAC-1	Dosage A	2	Day 42			08/10/2010		Head and Neck	Normal		
S154	113	JAIVAC-1	Dosage A	2	Day 42			08/10/2010		Eyes	Normal		
S154	113	JAIVAC-1	Dosage A	2	Day 42			08/10/2010		Ears, nose and throat	Normal		
S154	113	JAIVAC-1	Dosage A	2	Day 42			08/10/2010		Cardiovascular	Normal		
S154	113	JAIVAC-1	Dosage A	2	Day 42			08/10/2010		Respiratory	Normal		
S154	113	JAIVAC-1	Dosage A	2	Day 42			08/10/2010		Musculoskeletal	Normal		
S154	113	JAIVAC-1	Dosage A	2	Day 42			08/10/2010		Venous system	Normal		
S154	113	JAIVAC-1	Dosage A	2	Day 42			08/10/2010		Gastrointestinal	Normal		
S154	113	JAIVAC-1	Dosage A	2	Day 42			08/10/2010		Lymphatic	Normal		
S154	113	JAIVAC-1	Dosage A	2	Day 42			08/10/2010		Neurological	Normal		
S154	113	JAIVAC-1	Dosage A	2	Day 42			08/10/2010		Genitourinary	Normal		
S154	113	JAIVAC-1	Dosage A	2	Day 42			08/10/2010		Dermatological	Normal		
S154	113	JAIVAC-1	Dosage A	2	Day 56			22/10/2010		Head and Neck	Normal		
S154	113	JAIVAC-1	Dosage A	2	Day 56			22/10/2010		Eyes	Normal		
S154	113	JAIVAC-1	Dosage A	2	Day 56			22/10/2010		Ears, nose and throat	Normal		

Subject No.	Randomisation Number	Study Group	Dosage	Dose	Day	Not Done	Specify	Date of Assessment	*Was the assessment performed prior to vaccination?	Physical Examination Parameter	Result	Abnormality	Clinical Significance
S154	113	JAIVAC-1	Dosage A	2	Day 56			22/10/2010		Cardiovascular	Normal		
S154	113	JAIVAC-1	Dosage A	2	Day 56			22/10/2010		Respiratory	Normal		
S154	113	JAIVAC-1	Dosage A	2	Day 56			22/10/2010		Musculoskeletal	Normal		
S154	113	JAIVAC-1	Dosage A	2	Day 56			22/10/2010		Venous system	Normal		
S154	113	JAIVAC-1	Dosage A	2	Day 56			22/10/2010		Gastrointestinal	Normal		
S154	113	JAIVAC-1	Dosage A	2	Day 56			22/10/2010		Lymphatic	Normal		
S154	113	JAIVAC-1	Dosage A	2	Day 56			22/10/2010		Neurological	Normal		
S154	113	JAIVAC-1	Dosage A	2	Day 56			22/10/2010		Genitourinary	Normal		
S154	113	JAIVAC-1	Dosage A	2	Day 56			22/10/2010		Dermatological	Normal		
S154	113	JAIVAC-1	Dosage A	3	Day 180			23/02/2011	Yes	Head and Neck	Normal		
S154	113	JAIVAC-1	Dosage A	3	Day 180			23/02/2011	Yes	Eyes	Normal		
S154	113	JAIVAC-1	Dosage A	3	Day 180			23/02/2011	Yes	Ears, nose and throat	Normal		
S154	113	JAIVAC-1	Dosage A	3	Day 180			23/02/2011	Yes	Cardiovascular	Normal		
S154	113	JAIVAC-1	Dosage A	3	Day 180			23/02/2011	Yes	Respiratory	Normal		
S154	113	JAIVAC-1	Dosage A	3	Day 180			23/02/2011	Yes	Musculoskeletal	Normal		
S154	113	JAIVAC-1	Dosage A	3	Day 180			23/02/2011	Yes	Venous system	Normal		
S154	113	JAIVAC-1	Dosage A	3	Day 180			23/02/2011	Yes	Gastrointestinal	Normal		
S154	113	JAIVAC-1	Dosage A	3	Day 180			23/02/2011	Yes	Lymphatic	Normal		
S154	113	JAIVAC-1	Dosage A	3	Day 180			23/02/2011	Yes	Neurological	Normal		

Subject No.	Randomisation Number	Study Group	Dosage	Dose	Day	Not Done	Specify	Date of Assessment	*Was the assessment performed prior to vaccination?	Physical Examination Parameter	Result	Abnormality	Clinical Significance
S154	113	JAIVAC-1	Dosage A	3	Day 180			23/02/2011	Yes	Genitourinary	Normal		
S154	113	JAIVAC-1	Dosage A	3	Day 180			23/02/2011	Yes	Dermatological	Normal		
S154	113	JAIVAC-1	Dosage A	3	Day 187			01/03/2011		Head and Neck	Normal		
S154	113	JAIVAC-1	Dosage A	3	Day 187			01/03/2011		Eyes	Normal		
S154	113	JAIVAC-1	Dosage A	3	Day 187			01/03/2011		Ears, nose and throat	Normal		
S154	113	JAIVAC-1	Dosage A	3	Day 187			01/03/2011		Cardiovascular	Normal		
S154	113	JAIVAC-1	Dosage A	3	Day 187			01/03/2011		Respiratory	Normal		
S154	113	JAIVAC-1	Dosage A	3	Day 187			01/03/2011		Musculoskeletal	Normal		
S154	113	JAIVAC-1	Dosage A	3	Day 187			01/03/2011		Venous system	Normal		
S154	113	JAIVAC-1	Dosage A	3	Day 187			01/03/2011		Gastrointestinal	Normal		
S154	113	JAIVAC-1	Dosage A	3	Day 187			01/03/2011		Lymphatic	Normal		
S154	113	JAIVAC-1	Dosage A	3	Day 187			01/03/2011		Neurological	Normal		
S154	113	JAIVAC-1	Dosage A	3	Day 187			01/03/2011		Genitourinary	Normal		
S154	113	JAIVAC-1	Dosage A	3	Day 187			01/03/2011		Dermatological	Normal		
S154	113	JAIVAC-1	Dosage A	3	Day 194			09/03/2011		Head and Neck	Normal		
S154	113	JAIVAC-1	Dosage A	3	Day 194			09/03/2011		Eyes	Normal		
S154	113	JAIVAC-1	Dosage A	3	Day 194			09/03/2011		Ears, nose and throat	Normal		
S154	113	JAIVAC-1	Dosage A	3	Day 194			09/03/2011		Cardiovascular	Normal		
S154	113	JAIVAC-1	Dosage A	3	Day 194			09/03/2011		Respiratory	Normal		

Subject No.	Randomisation Number	Study Group	Dosage	Dose	Day	Not Done	Specify	Date of Assessment	*Was the assessment performed prior to vaccination?	Physical Examination Parameter	Result	Abnormality	Clinical Significance
S154	113	JAIVAC-1	Dosage A	3	Day 194			09/03/2011		Musculoskeletal	Normal		
S154	113	JAIVAC-1	Dosage A	3	Day 194			09/03/2011		Venous system	Normal		
S154	113	JAIVAC-1	Dosage A	3	Day 194			09/03/2011		Gastrointestinal	Normal		
S154	113	JAIVAC-1	Dosage A	3	Day 194			09/03/2011		Lymphatic	Normal		
S154	113	JAIVAC-1	Dosage A	3	Day 194			09/03/2011		Neurological	Normal		
S154	113	JAIVAC-1	Dosage A	3	Day 194			09/03/2011		Genitourinary	Normal		
S154	113	JAIVAC-1	Dosage A	3	Day 194			09/03/2011		Dermatological	Normal		
S154	113	JAIVAC-1	Dosage A	3	Day 208			23/03/2011		Head and Neck	Normal		
S154	113	JAIVAC-1	Dosage A	3	Day 208			23/03/2011		Eyes	Normal		
S154	113	JAIVAC-1	Dosage A	3	Day 208			23/03/2011		Ears, nose and throat	Normal		
S154	113	JAIVAC-1	Dosage A	3	Day 208			23/03/2011		Cardiovascular	Normal		
S154	113	JAIVAC-1	Dosage A	3	Day 208			23/03/2011		Respiratory	Normal		
S154	113	JAIVAC-1	Dosage A	3	Day 208			23/03/2011		Musculoskeletal	Normal		
S154	113	JAIVAC-1	Dosage A	3	Day 208			23/03/2011		Venous system	Normal		
S154	113	JAIVAC-1	Dosage A	3	Day 208			23/03/2011		Gastrointestinal	Normal		
S154	113	JAIVAC-1	Dosage A	3	Day 208			23/03/2011		Lymphatic	Normal		
S154	113	JAIVAC-1	Dosage A	3	Day 208			23/03/2011		Neurological	Normal		
S154	113	JAIVAC-1	Dosage A	3	Day 208			23/03/2011		Genitourinary	Normal		
S154	113	JAIVAC-1	Dosage A	3	Day 208			23/03/2011		Dermatological	Normal		

Subject No.	Randomisation Number	Study Group	Dosage	Dose	Day	Not Done	Specify	Date of Assessment	*Was the assessment performed prior to vaccination?	Physical Examination Parameter	Result	Abnormality	Clinical Significance
S154	113	JAIVAC-1	Dosage A	3	Day 365			26/08/2011		Head and Neck	Normal		
S154	113	JAIVAC-1	Dosage A	3	Day 365			26/08/2011		Eyes	Normal		
S154	113	JAIVAC-1	Dosage A	3	Day 365			26/08/2011		Ears, nose and throat	Normal		
S154	113	JAIVAC-1	Dosage A	3	Day 365			26/08/2011		Cardiovascular	Normal		
S154	113	JAIVAC-1	Dosage A	3	Day 365			26/08/2011		Respiratory	Normal		
S154	113	JAIVAC-1	Dosage A	3	Day 365			26/08/2011		Musculoskeletal	Normal		
S154	113	JAIVAC-1	Dosage A	3	Day 365			26/08/2011		Venous system	Normal		
S154	113	JAIVAC-1	Dosage A	3	Day 365			26/08/2011		Gastrointestinal	Normal		
S154	113	JAIVAC-1	Dosage A	3	Day 365			26/08/2011		Lymphatic	Normal		
S154	113	JAIVAC-1	Dosage A	3	Day 365			26/08/2011		Neurological	Normal		
S154	113	JAIVAC-1	Dosage A	3	Day 365			26/08/2011		Genitourinary	Normal		
S154	113	JAIVAC-1	Dosage A	3	Day 365			26/08/2011		Dermatological	Normal		
S156	110	JAIVAC-1	Dosage A		Day -14 to Day -1			19/08/2010		Head and neck	Normal		
S156	110	JAIVAC-1	Dosage A		Day -14 to Day -1			19/08/2010		Eyes	Normal		
S156	110	JAIVAC-1	Dosage A		Day -14 to Day -1			19/08/2010		Ears, nose and throat	Normal		
S156	110	JAIVAC-1	Dosage A		Day -14 to Day -1			19/08/2010		Cardiovascular	Normal		
S156	110	JAIVAC-1	Dosage A		Day -14 to Day -1			19/08/2010		Respiratory	Normal		
S156	110	JAIVAC-1	Dosage A		Day -14 to Day -1			19/08/2010		Musculoskeletal	Normal		

Subject No.	Randomisation Number	Study Group	Dosage	Dose	Day	Not Done	Specify	Date of Assessment	*Was the assessment performed prior to vaccination?	Physical Examination Parameter	Result	Abnormality	Clinical Significance
S156	110	JAIVAC-1	Dosage A		Day -14 to Day -1			19/08/2010		Venous system	Normal		
S156	110	JAIVAC-1	Dosage A		Day -14 to Day -1			19/08/2010		Gastrointestinal	Normal		
S156	110	JAIVAC-1	Dosage A		Day -14 to Day -1			19/08/2010		Lymphatic	Normal		
S156	110	JAIVAC-1	Dosage A		Day -14 to Day -1			19/08/2010		Neurological	Normal		
S156	110	JAIVAC-1	Dosage A		Day -14 to Day -1			19/08/2010		Genitourinary	Normal		
S156	110	JAIVAC-1	Dosage A		Day -14 to Day -1			19/08/2010		Dermatological	Normal		
S156	110	JAIVAC-1	Dosage A	1	Day 0			27/08/2010	Yes	Head and neck	Normal		
S156	110	JAIVAC-1	Dosage A	1	Day 0			27/08/2010	Yes	Eyes	Normal		
S156	110	JAIVAC-1	Dosage A	1	Day 0			27/08/2010	Yes	Ears, nose and throat	Normal		
S156	110	JAIVAC-1	Dosage A	1	Day 0			27/08/2010	Yes	Cardiovascular	Normal		
S156	110	JAIVAC-1	Dosage A	1	Day 0			27/08/2010	Yes	Respiratory	Normal		
S156	110	JAIVAC-1	Dosage A	1	Day 0			27/08/2010	Yes	Musculoskeletal	Normal		
S156	110	JAIVAC-1	Dosage A	1	Day 0			27/08/2010	Yes	Venous system	Normal		
S156	110	JAIVAC-1	Dosage A	1	Day 0			27/08/2010	Yes	Gastrointestinal	Normal		
S156	110	JAIVAC-1	Dosage A	1	Day 0			27/08/2010	Yes	Lymphatic	Normal		
S156	110	JAIVAC-1	Dosage A	1	Day 0			27/08/2010	Yes	Neurological	Normal		
S156	110	JAIVAC-1	Dosage A	1	Day 0			27/08/2010	Yes	Genitourinary	Normal		
S156	110	JAIVAC-1	Dosage A	1	Day 0			27/08/2010	Yes	Dermatological	Normal		

Subject No.	Randomisation Number	Study Group	Dosage	Dose	Day	Not Done	Specify	Date of Assessment	*Was the assessment performed prior to vaccination?	Physical Examination Parameter	Result	Abnormality	Clinical Significance
S156	110	JAIVAC-1	Dosage A	1	Day 7			03/09/2010		Head and Neck	Normal		
S156	110	JAIVAC-1	Dosage A	1	Day 7			03/09/2010		Eyes	Normal		
S156	110	JAIVAC-1	Dosage A	1	Day 7			03/09/2010		Ears, nose and throat	Normal		
S156	110	JAIVAC-1	Dosage A	1	Day 7			03/09/2010		Cardiovascular	Normal		
S156	110	JAIVAC-1	Dosage A	1	Day 7			03/09/2010		Respiratory	Normal		
S156	110	JAIVAC-1	Dosage A	1	Day 7			03/09/2010		Musculoskeletal	Normal		
S156	110	JAIVAC-1	Dosage A	1	Day 7			03/09/2010		Venous system	Normal		
S156	110	JAIVAC-1	Dosage A	1	Day 7			03/09/2010		Gastrointestinal	Normal		
S156	110	JAIVAC-1	Dosage A	1	Day 7			03/09/2010		Lymphatic	Normal		
S156	110	JAIVAC-1	Dosage A	1	Day 7			03/09/2010		Neurological	Normal		
S156	110	JAIVAC-1	Dosage A	1	Day 7			03/09/2010		Genitourinary	Normal		
S156	110	JAIVAC-1	Dosage A	1	Day 7			03/09/2010		Dermatological	Normal		
S156	110	JAIVAC-1	Dosage A	1	Day 14			09/09/2010		Head and Neck	Normal		
S156	110	JAIVAC-1	Dosage A	1	Day 14			09/09/2010		Eyes	Normal		
S156	110	JAIVAC-1	Dosage A	1	Day 14			09/09/2010		Ears, nose and throat	Normal		
S156	110	JAIVAC-1	Dosage A	1	Day 14			09/09/2010		Cardiovascular	Normal		
S156	110	JAIVAC-1	Dosage A	1	Day 14			09/09/2010		Respiratory	Normal		
S156	110	JAIVAC-1	Dosage A	1	Day 14			09/09/2010		Musculoskeletal	Normal		
S156	110	JAIVAC-1	Dosage A	1	Day 14			09/09/2010		Venous system	Normal		

Subject No.	Randomisation Number	Study Group	Dosage	Dose	Day	Not Done	Specify	Date of Assessment	*Was the assessment performed prior to vaccination?	Physical Examination Parameter	Result	Abnormality	Clinical Significance
S156	110	JAIVAC-1	Dosage A	1	Day 14			09/09/2010		Gastrointestinal	Normal		
S156	110	JAIVAC-1	Dosage A	1	Day 14			09/09/2010		Lymphatic	Normal		
S156	110	JAIVAC-1	Dosage A	1	Day 14			09/09/2010		Neurological	Normal		
S156	110	JAIVAC-1	Dosage A	1	Day 14			09/09/2010		Genitourinary	Normal		
S156	110	JAIVAC-1	Dosage A	1	Day 14			09/09/2010		Dermatological	Normal		
S156	110	JAIVAC-1	Dosage A	2	Day 28			24/09/2010	Yes	Head and Neck	Normal		
S156	110	JAIVAC-1	Dosage A	2	Day 28			24/09/2010	Yes	Eyes	Normal		
S156	110	JAIVAC-1	Dosage A	2	Day 28			24/09/2010	Yes	Ears, nose and throat	Normal		
S156	110	JAIVAC-1	Dosage A	2	Day 28			24/09/2010	Yes	Cardiovascular	Normal		
S156	110	JAIVAC-1	Dosage A	2	Day 28			24/09/2010	Yes	Respiratory	Normal		
S156	110	JAIVAC-1	Dosage A	2	Day 28			24/09/2010	Yes	Musculoskeletal	Normal		
S156	110	JAIVAC-1	Dosage A	2	Day 28			24/09/2010	Yes	Venous system	Normal		
S156	110	JAIVAC-1	Dosage A	2	Day 28			24/09/2010	Yes	Gastrointestinal	Normal		
S156	110	JAIVAC-1	Dosage A	2	Day 28			24/09/2010	Yes	Lymphatic	Normal		
S156	110	JAIVAC-1	Dosage A	2	Day 28			24/09/2010	Yes	Neurological	Normal		
S156	110	JAIVAC-1	Dosage A	2	Day 28			24/09/2010	Yes	Genitourinary	Normal		
S156	110	JAIVAC-1	Dosage A	2	Day 28			24/09/2010	Yes	Dermatological	Normal		
S156	110	JAIVAC-1	Dosage A	2	Day 35			01/10/2010		Head and Neck	Normal		
S156	110	JAIVAC-1	Dosage A	2	Day 35			01/10/2010		Eyes	Normal		

Subject No.	Randomisation Number	Study Group	Dosage	Dose	Day	Not Done	Specify	Date of Assessment	*Was the assessment performed prior to vaccination?	Physical Examination Parameter	Result	Abnormality	Clinical Significance
S156	110	JAIVAC-1	Dosage A	2	Day 35			01/10/2010		Ears, nose and throat	Normal		
S156	110	JAIVAC-1	Dosage A	2	Day 35			01/10/2010		Cardiovascular	Normal		
S156	110	JAIVAC-1	Dosage A	2	Day 35			01/10/2010		Respiratory	Normal		
S156	110	JAIVAC-1	Dosage A	2	Day 35			01/10/2010		Musculoskeletal	Normal		
S156	110	JAIVAC-1	Dosage A	2	Day 35			01/10/2010		Venous system	Normal		
S156	110	JAIVAC-1	Dosage A	2	Day 35			01/10/2010		Gastrointestinal	Normal		
S156	110	JAIVAC-1	Dosage A	2	Day 35			01/10/2010		Lymphatic	Normal		
S156	110	JAIVAC-1	Dosage A	2	Day 35			01/10/2010		Neurological	Normal		
S156	110	JAIVAC-1	Dosage A	2	Day 35			01/10/2010		Genitourinary	Normal		
S156	110	JAIVAC-1	Dosage A	2	Day 35			01/10/2010		Dermatological	Normal		
S156	110	JAIVAC-1	Dosage A	2	Day 42			08/10/2010		Head and Neck	Normal		
S156	110	JAIVAC-1	Dosage A	2	Day 42			08/10/2010		Eyes	Normal		
S156	110	JAIVAC-1	Dosage A	2	Day 42			08/10/2010		Ears, nose and throat	Normal		
S156	110	JAIVAC-1	Dosage A	2	Day 42			08/10/2010		Cardiovascular	Normal		
S156	110	JAIVAC-1	Dosage A	2	Day 42			08/10/2010		Respiratory	Normal		
S156	110	JAIVAC-1	Dosage A	2	Day 42			08/10/2010		Musculoskeletal	Normal		
S156	110	JAIVAC-1	Dosage A	2	Day 42			08/10/2010		Venous system	Normal		
S156	110	JAIVAC-1	Dosage A	2	Day 42			08/10/2010		Gastrointestinal	Normal		
S156	110	JAIVAC-1	Dosage A	2	Day 42			08/10/2010		Lymphatic	Normal		

Subject No.	Randomisation Number	Study Group	Dosage	Dose	Day	Not Done	Specify	Date of Assessment	*Was the assessment performed prior to vaccination?	Physical Examination Parameter	Result	Abnormality	Clinical Significance
S156	110	JAIVAC-1	Dosage A	2	Day 42			08/10/2010		Neurological	Normal		
S156	110	JAIVAC-1	Dosage A	2	Day 42			08/10/2010		Genitourinary	Normal		
S156	110	JAIVAC-1	Dosage A	2	Day 42			08/10/2010		Dermatological	Normal		
S156	110	JAIVAC-1	Dosage A	2	Day 56			22/10/2010		Head and Neck	Normal		
S156	110	JAIVAC-1	Dosage A	2	Day 56			22/10/2010		Eyes	Normal		
S156	110	JAIVAC-1	Dosage A	2	Day 56			22/10/2010		Ears, nose and throat	Normal		
S156	110	JAIVAC-1	Dosage A	2	Day 56			22/10/2010		Cardiovascular	Normal		
S156	110	JAIVAC-1	Dosage A	2	Day 56			22/10/2010		Respiratory	Normal		
S156	110	JAIVAC-1	Dosage A	2	Day 56			22/10/2010		Musculoskeletal	Normal		
S156	110	JAIVAC-1	Dosage A	2	Day 56			22/10/2010		Venous system	Normal		
S156	110	JAIVAC-1	Dosage A	2	Day 56			22/10/2010		Gastrointestinal	Normal		
S156	110	JAIVAC-1	Dosage A	2	Day 56			22/10/2010		Lymphatic	Normal		
S156	110	JAIVAC-1	Dosage A	2	Day 56			22/10/2010		Neurological	Normal		
S156	110	JAIVAC-1	Dosage A	2	Day 56			22/10/2010		Genitourinary	Normal		
S156	110	JAIVAC-1	Dosage A	2	Day 56			22/10/2010		Dermatological	Normal		
S156	110	JAIVAC-1	Dosage A	3	Day 180			23/02/2011	Yes	Head and Neck	Normal		
S156	110	JAIVAC-1	Dosage A	3	Day 180			23/02/2011	Yes	Eyes	Normal		
S156	110	JAIVAC-1	Dosage A	3	Day 180			23/02/2011	Yes	Ears, nose and throat	Normal		
S156	110	JAIVAC-1	Dosage A	3	Day 180			23/02/2011	Yes	Cardiovascular	Normal		

Subject No.	Randomisation Number	Study Group	Dosage	Dose	Day	Not Done	Specify	Date of Assessment	*Was the assessment performed prior to vaccination?	Physical Examination Parameter	Result	Abnormality	Clinical Significance
S156	110	JAIVAC-1	Dosage A	3	Day 180			23/02/2011	Yes	Respiratory	Normal		
S156	110	JAIVAC-1	Dosage A	3	Day 180			23/02/2011	Yes	Musculoskeletal	Normal		
S156	110	JAIVAC-1	Dosage A	3	Day 180			23/02/2011	Yes	Venous system	Normal		
S156	110	JAIVAC-1	Dosage A	3	Day 180			23/02/2011	Yes	Gastrointestinal	Normal		
S156	110	JAIVAC-1	Dosage A	3	Day 180			23/02/2011	Yes	Lymphatic	Normal		
S156	110	JAIVAC-1	Dosage A	3	Day 180			23/02/2011	Yes	Neurological	Normal		
S156	110	JAIVAC-1	Dosage A	3	Day 180			23/02/2011	Yes	Genitourinary	Normal		
S156	110	JAIVAC-1	Dosage A	3	Day 180			23/02/2011	Yes	Dermatological	Normal		
S156	110	JAIVAC-1	Dosage A	3	Day 187			02/03/2011		Head and Neck	Normal		
S156	110	JAIVAC-1	Dosage A	3	Day 187			02/03/2011		Eyes	Normal		
S156	110	JAIVAC-1	Dosage A	3	Day 187			02/03/2011		Ears, nose and throat	Normal		
S156	110	JAIVAC-1	Dosage A	3	Day 187			02/03/2011		Cardiovascular	Normal		
S156	110	JAIVAC-1	Dosage A	3	Day 187			02/03/2011		Respiratory	Normal		
S156	110	JAIVAC-1	Dosage A	3	Day 187			02/03/2011		Musculoskeletal	Normal		
S156	110	JAIVAC-1	Dosage A	3	Day 187			02/03/2011		Venous system	Normal		
S156	110	JAIVAC-1	Dosage A	3	Day 187			02/03/2011		Gastrointestinal	Normal		
S156	110	JAIVAC-1	Dosage A	3	Day 187			02/03/2011		Lymphatic	Normal		
S156	110	JAIVAC-1	Dosage A	3	Day 187			02/03/2011		Neurological	Normal		
S156	110	JAIVAC-1	Dosage A	3	Day 187			02/03/2011		Genitourinary	Normal		

Subject No.	Randomisation Number	Study Group	Dosage	Dose	Day	Not Done	Specify	Date of Assessment	*Was the assessment performed prior to vaccination?	Physical Examination Parameter	Result	Abnormality	Clinical Significance
S156	110	JAIVAC-1	Dosage A	3	Day 187			02/03/2011		Dermatological	Normal		
S156	110	JAIVAC-1	Dosage A	3	Day 194			09/03/2011		Head and Neck	Normal		
S156	110	JAIVAC-1	Dosage A	3	Day 194			09/03/2011		Eyes	Normal		
S156	110	JAIVAC-1	Dosage A	3	Day 194			09/03/2011		Ears, nose and throat	Normal		
S156	110	JAIVAC-1	Dosage A	3	Day 194			09/03/2011		Cardiovascular	Normal		
S156	110	JAIVAC-1	Dosage A	3	Day 194			09/03/2011		Respiratory	Normal		
S156	110	JAIVAC-1	Dosage A	3	Day 194			09/03/2011		Musculoskeletal	Normal		
S156	110	JAIVAC-1	Dosage A	3	Day 194			09/03/2011		Venous system	Normal		
S156	110	JAIVAC-1	Dosage A	3	Day 194			09/03/2011		Gastrointestinal	Normal		
S156	110	JAIVAC-1	Dosage A	3	Day 194			09/03/2011		Lymphatic	Normal		
S156	110	JAIVAC-1	Dosage A	3	Day 194			09/03/2011		Neurological	Normal		
S156	110	JAIVAC-1	Dosage A	3	Day 194			09/03/2011		Genitourinary	Normal		
S156	110	JAIVAC-1	Dosage A	3	Day 194			09/03/2011		Dermatological	Normal		
S156	110	JAIVAC-1	Dosage A	3	Day 208			23/03/2011		Head and Neck	Normal		
S156	110	JAIVAC-1	Dosage A	3	Day 208			23/03/2011		Eyes	Normal		
S156	110	JAIVAC-1	Dosage A	3	Day 208			23/03/2011		Ears, nose and throat	Normal		
S156	110	JAIVAC-1	Dosage A	3	Day 208			23/03/2011		Cardiovascular	Normal		
S156	110	JAIVAC-1	Dosage A	3	Day 208			23/03/2011		Respiratory	Normal		
S156	110	JAIVAC-1	Dosage A	3	Day 208			23/03/2011		Musculoskeletal	Normal		

Subject No.	Randomisation Number	Study Group	Dosage	Dose	Day	Not Done	Specify	Date of Assessment	*Was the assessment performed prior to vaccination?	Physical Examination Parameter	Result	Abnormality	Clinical Significance
S156	110	JAIVAC-1	Dosage A	3	Day 208			23/03/2011		Venous system	Normal		
S156	110	JAIVAC-1	Dosage A	3	Day 208			23/03/2011		Gastrointestinal	Normal		
S156	110	JAIVAC-1	Dosage A	3	Day 208			23/03/2011		Lymphatic	Normal		
S156	110	JAIVAC-1	Dosage A	3	Day 208			23/03/2011		Neurological	Normal		
S156	110	JAIVAC-1	Dosage A	3	Day 208			23/03/2011		Genitourinary	Normal		
S156	110	JAIVAC-1	Dosage A	3	Day 208			23/03/2011		Dermatological	Normal		
S156	110	JAIVAC-1	Dosage A	3	Day 365			26/08/2011		Head and Neck	Normal		
S156	110	JAIVAC-1	Dosage A	3	Day 365			26/08/2011		Eyes	Normal		
S156	110	JAIVAC-1	Dosage A	3	Day 365			26/08/2011		Ears, nose and throat	Normal		
S156	110	JAIVAC-1	Dosage A	3	Day 365			26/08/2011		Cardiovascular	Normal		
S156	110	JAIVAC-1	Dosage A	3	Day 365			26/08/2011		Respiratory	Normal		
S156	110	JAIVAC-1	Dosage A	3	Day 365			26/08/2011		Musculoskeletal	Normal		
S156	110	JAIVAC-1	Dosage A	3	Day 365			26/08/2011		Venous system	Normal		
S156	110	JAIVAC-1	Dosage A	3	Day 365			26/08/2011		Gastrointestinal	Normal		
S156	110	JAIVAC-1	Dosage A	3	Day 365			26/08/2011		Lymphatic	Normal		
S156	110	JAIVAC-1	Dosage A	3	Day 365			26/08/2011		Neurological	Normal		
S156	110	JAIVAC-1	Dosage A	3	Day 365			26/08/2011		Genitourinary	Normal		
S156	110	JAIVAC-1	Dosage A	3	Day 365			26/08/2011		Dermatological	Normal		
S170	204	Hepatitis B	Hepatitis B		Day -14 to Day -1			21/09/2010		Head and neck	Normal		

Subject No.	Randomisation Number	Study Group	Dosage	Dose	Day	Not Done	Specify	Date of Assessment	*Was the assessment performed prior to vaccination?	Physical Examination Parameter	Result	Abnormality	Clinical Significance
S170	204	Hepatitis B	Hepatitis B		Day -14 to Day -1			21/09/2010		Eyes	Normal		
S170	204	Hepatitis B	Hepatitis B		Day -14 to Day -1			21/09/2010		Ears, nose and throat	Normal		
S170	204	Hepatitis B	Hepatitis B		Day -14 to Day -1			21/09/2010		Cardiovascular	Normal		
S170	204	Hepatitis B	Hepatitis B		Day -14 to Day -1			21/09/2010		Respiratory	Normal		
S170	204	Hepatitis B	Hepatitis B		Day -14 to Day -1			21/09/2010		Musculoskeletal	Normal		
S170	204	Hepatitis B	Hepatitis B		Day -14 to Day -1			21/09/2010		Venous system	Normal		
S170	204	Hepatitis B	Hepatitis B		Day -14 to Day -1			21/09/2010		Gastrointestinal	Normal		
S170	204	Hepatitis B	Hepatitis B		Day -14 to Day -1			21/09/2010		Lymphatic	Normal		
S170	204	Hepatitis B	Hepatitis B		Day -14 to Day -1			21/09/2010		Neurological	Normal		
S170	204	Hepatitis B	Hepatitis B		Day -14 to Day -1			21/09/2010		Genitourinary	Normal		
S170	204	Hepatitis B	Hepatitis B		Day -14 to Day -1			21/09/2010		Dermatological	Normal		
S170	204	Hepatitis B	Hepatitis B	1	Day 0			29/09/2010	Yes	Head and neck	Normal		
S170	204	Hepatitis B	Hepatitis B	1	Day 0			29/09/2010	Yes	Eyes	Normal		
S170	204	Hepatitis B	Hepatitis B	1	Day 0			29/09/2010	Yes	Ears, nose and throat	Normal		
S170	204	Hepatitis B	Hepatitis B	1	Day 0			29/09/2010	Yes	Cardiovascular	Normal		
S170	204	Hepatitis B	Hepatitis B	1	Day 0			29/09/2010	Yes	Respiratory	Normal		
S170	204	Hepatitis B	Hepatitis B	1	Day 0			29/09/2010	Yes	Musculoskeletal	Normal		

Subject No.	Randomisation Number	Study Group	Dosage	Dose	Day	Not Done	Specify	Date of Assessment	*Was the assessment performed prior to vaccination?	Physical Examination Parameter	Result	Abnormality	Clinical Significance
S170	204	Hepatitis B	Hepatitis B	1	Day 0			29/09/2010	Yes	Venous system	Normal		
S170	204	Hepatitis B	Hepatitis B	1	Day 0			29/09/2010	Yes	Gastrointestinal	Normal		
S170	204	Hepatitis B	Hepatitis B	1	Day 0			29/09/2010	Yes	Lymphatic	Normal		
S170	204	Hepatitis B	Hepatitis B	1	Day 0			29/09/2010	Yes	Neurological	Normal		
S170	204	Hepatitis B	Hepatitis B	1	Day 0			29/09/2010	Yes	Genitourinary	Normal		
S170	204	Hepatitis B	Hepatitis B	1	Day 0			29/09/2010	Yes	Dermatological	Normal		
S170	204	Hepatitis B	Hepatitis B	1	Day 7			06/10/2010		Head and Neck	Normal		
S170	204	Hepatitis B	Hepatitis B	1	Day 7			06/10/2010		Eyes	Normal		
S170	204	Hepatitis B	Hepatitis B	1	Day 7			06/10/2010		Ears, nose and throat	Normal		
S170	204	Hepatitis B	Hepatitis B	1	Day 7			06/10/2010		Cardiovascular	Normal		
S170	204	Hepatitis B	Hepatitis B	1	Day 7			06/10/2010		Respiratory	Normal		
S170	204	Hepatitis B	Hepatitis B	1	Day 7			06/10/2010		Musculoskeletal	Normal		
S170	204	Hepatitis B	Hepatitis B	1	Day 7			06/10/2010		Venous system	Normal		
S170	204	Hepatitis B	Hepatitis B	1	Day 7			06/10/2010		Gastrointestinal	Normal		
S170	204	Hepatitis B	Hepatitis B	1	Day 7			06/10/2010		Lymphatic	Normal		
S170	204	Hepatitis B	Hepatitis B	1	Day 7			06/10/2010		Neurological	Normal		
S170	204	Hepatitis B	Hepatitis B	1	Day 7			06/10/2010		Genitourinary	Normal		
S170	204	Hepatitis B	Hepatitis B	1	Day 7			06/10/2010		Dermatological	Normal		
S170	204	Hepatitis B	Hepatitis B	1	Day 14			13/10/2010		Head and Neck	Normal		

Subject No.	Randomisation Number	Study Group	Dosage	Dose	Day	Not Done	Specify	Date of Assessment	*Was the assessment performed prior to vaccination?	Physical Examination Parameter	Result	Abnormality	Clinical Significance
S170	204	Hepatitis B	Hepatitis B	1	Day 14			13/10/2010		Eyes	Normal		
S170	204	Hepatitis B	Hepatitis B	1	Day 14			13/10/2010		Ears, nose and throat	Normal		
S170	204	Hepatitis B	Hepatitis B	1	Day 14			13/10/2010		Cardiovascular	Normal		
S170	204	Hepatitis B	Hepatitis B	1	Day 14			13/10/2010		Respiratory	Normal		
S170	204	Hepatitis B	Hepatitis B	1	Day 14			13/10/2010		Musculoskeletal	Normal		
S170	204	Hepatitis B	Hepatitis B	1	Day 14			13/10/2010		Venous system	Normal		
S170	204	Hepatitis B	Hepatitis B	1	Day 14			13/10/2010		Gastrointestinal	Normal		
S170	204	Hepatitis B	Hepatitis B	1	Day 14			13/10/2010		Lymphatic	Normal		
S170	204	Hepatitis B	Hepatitis B	1	Day 14			13/10/2010		Neurological	Normal		
S170	204	Hepatitis B	Hepatitis B	1	Day 14			13/10/2010		Genitourinary	Normal		
S170	204	Hepatitis B	Hepatitis B	1	Day 14			13/10/2010		Dermatological	Normal		
S170	204	Hepatitis B	Hepatitis B	2	Day 28			27/10/2010	Yes	Head and Neck	Normal		
S170	204	Hepatitis B	Hepatitis B	2	Day 28			27/10/2010	Yes	Eyes	Normal		
S170	204	Hepatitis B	Hepatitis B	2	Day 28			27/10/2010	Yes	Ears, nose and throat	Normal		
S170	204	Hepatitis B	Hepatitis B	2	Day 28			27/10/2010	Yes	Cardiovascular	Normal		
S170	204	Hepatitis B	Hepatitis B	2	Day 28			27/10/2010	Yes	Respiratory	Normal		
S170	204	Hepatitis B	Hepatitis B	2	Day 28			27/10/2010	Yes	Musculoskeletal	Normal		
S170	204	Hepatitis B	Hepatitis B	2	Day 28			27/10/2010	Yes	Venous system	Normal		
S170	204	Hepatitis B	Hepatitis B	2	Day 28			27/10/2010	Yes	Gastrointestinal	Normal		

Subject No.	Randomisation Number	Study Group	Dosage	Dose	Day	Not Done	Specify	Date of Assessment	*Was the assessment performed prior to vaccination?	Physical Examination Parameter	Result	Abnormality	Clinical Significance
S170	204	Hepatitis B	Hepatitis B	2	Day 28			27/10/2010	Yes	Lymphatic	Normal		
S170	204	Hepatitis B	Hepatitis B	2	Day 28			27/10/2010	Yes	Neurological	Normal		
S170	204	Hepatitis B	Hepatitis B	2	Day 28			27/10/2010	Yes	Genitourinary	Normal		
S170	204	Hepatitis B	Hepatitis B	2	Day 28			27/10/2010	Yes	Dermatological	Normal		
S170	204	Hepatitis B	Hepatitis B	2	Day 35			03/11/2010		Head and Neck	Normal		
S170	204	Hepatitis B	Hepatitis B	2	Day 35			03/11/2010		Eyes	Normal		
S170	204	Hepatitis B	Hepatitis B	2	Day 35			03/11/2010		Ears, nose and throat	Normal		
S170	204	Hepatitis B	Hepatitis B	2	Day 35			03/11/2010		Cardiovascular	Normal		
S170	204	Hepatitis B	Hepatitis B	2	Day 35			03/11/2010		Respiratory	Normal		
S170	204	Hepatitis B	Hepatitis B	2	Day 35			03/11/2010		Musculoskeletal	Normal		
S170	204	Hepatitis B	Hepatitis B	2	Day 35			03/11/2010		Venous system	Normal		
S170	204	Hepatitis B	Hepatitis B	2	Day 35			03/11/2010		Gastrointestinal	Normal		
S170	204	Hepatitis B	Hepatitis B	2	Day 35			03/11/2010		Lymphatic	Normal		
S170	204	Hepatitis B	Hepatitis B	2	Day 35			03/11/2010		Neurological	Normal		
S170	204	Hepatitis B	Hepatitis B	2	Day 35			03/11/2010		Genitourinary	Normal		
S170	204	Hepatitis B	Hepatitis B	2	Day 35			03/11/2010		Dermatological	Normal		
S170	204	Hepatitis B	Hepatitis B	2	Day 42			10/11/2010		Head and Neck	Normal		
S170	204	Hepatitis B	Hepatitis B	2	Day 42			10/11/2010		Eyes	Normal		
S170	204	Hepatitis B	Hepatitis B	2	Day 42			10/11/2010		Ears, nose and throat	Normal		

Subject No.	Randomisation Number	Study Group	Dosage	Dose	Day	Not Done	Specify	Date of Assessment	*Was the assessment performed prior to vaccination?	Physical Examination Parameter	Result	Abnormality	Clinical Significance
S170	204	Hepatitis B	Hepatitis B	2	Day 42			10/11/2010		Cardiovascular	Normal		
S170	204	Hepatitis B	Hepatitis B	2	Day 42			10/11/2010		Respiratory	Normal		
S170	204	Hepatitis B	Hepatitis B	2	Day 42			10/11/2010		Musculoskeletal	Normal		
S170	204	Hepatitis B	Hepatitis B	2	Day 42			10/11/2010		Venous system	Normal		
S170	204	Hepatitis B	Hepatitis B	2	Day 42			10/11/2010		Gastrointestinal	Normal		
S170	204	Hepatitis B	Hepatitis B	2	Day 42			10/11/2010		Lymphatic	Normal		
S170	204	Hepatitis B	Hepatitis B	2	Day 42			10/11/2010		Neurological	Normal		
S170	204	Hepatitis B	Hepatitis B	2	Day 42			10/11/2010		Genitourinary	Normal		
S170	204	Hepatitis B	Hepatitis B	2	Day 42			10/11/2010		Dermatological	Normal		
S170	204	Hepatitis B	Hepatitis B	2	Day 56			25/11/2010		Head and Neck	Normal		
S170	204	Hepatitis B	Hepatitis B	2	Day 56			25/11/2010		Eyes	Normal		
S170	204	Hepatitis B	Hepatitis B	2	Day 56			25/11/2010		Ears, nose and throat	Normal		
S170	204	Hepatitis B	Hepatitis B	2	Day 56			25/11/2010		Cardiovascular	Normal		
S170	204	Hepatitis B	Hepatitis B	2	Day 56			25/11/2010		Respiratory	Normal		
S170	204	Hepatitis B	Hepatitis B	2	Day 56			25/11/2010		Musculoskeletal	Normal		
S170	204	Hepatitis B	Hepatitis B	2	Day 56			25/11/2010		Venous system	Normal		
S170	204	Hepatitis B	Hepatitis B	2	Day 56			25/11/2010		Gastrointestinal	Normal		
S170	204	Hepatitis B	Hepatitis B	2	Day 56			25/11/2010		Lymphatic	Normal		
S170	204	Hepatitis B	Hepatitis B	2	Day 56			25/11/2010		Neurological	Normal		

Subject No.	Randomisation Number	Study Group	Dosage	Dose	Day	Not Done	Specify	Date of Assessment	*Was the assessment performed prior to vaccination?	Physical Examination Parameter	Result	Abnormality	Clinical Significance
S170	204	Hepatitis B	Hepatitis B	2	Day 56			25/11/2010		Genitourinary	Normal		
S170	204	Hepatitis B	Hepatitis B	2	Day 56			25/11/2010		Dermatological	Normal		
S170	204	Hepatitis B	Hepatitis B	3	Day 180			28/03/2011	Yes	Head and Neck	Normal		
S170	204	Hepatitis B	Hepatitis B	3	Day 180			28/03/2011	Yes	Eyes	Normal		
S170	204	Hepatitis B	Hepatitis B	3	Day 180			28/03/2011	Yes	Ears, nose and throat	Normal		
S170	204	Hepatitis B	Hepatitis B	3	Day 180			28/03/2011	Yes	Cardiovascular	Normal		
S170	204	Hepatitis B	Hepatitis B	3	Day 180			28/03/2011	Yes	Respiratory	Normal		
S170	204	Hepatitis B	Hepatitis B	3	Day 180			28/03/2011	Yes	Musculoskeletal	Normal		
S170	204	Hepatitis B	Hepatitis B	3	Day 180			28/03/2011	Yes	Venous system	Normal		
S170	204	Hepatitis B	Hepatitis B	3	Day 180			28/03/2011	Yes	Gastrointestinal	Normal		
S170	204	Hepatitis B	Hepatitis B	3	Day 180			28/03/2011	Yes	Lymphatic	Normal		
S170	204	Hepatitis B	Hepatitis B	3	Day 180			28/03/2011	Yes	Neurological	Normal		
S170	204	Hepatitis B	Hepatitis B	3	Day 180			28/03/2011	Yes	Genitourinary	Normal		
S170	204	Hepatitis B	Hepatitis B	3	Day 180			28/03/2011	Yes	Dermatological	Normal		
S170	204	Hepatitis B	Hepatitis B	3	Day 187			06/04/2011		Head and Neck	Normal		
S170	204	Hepatitis B	Hepatitis B	3	Day 187			06/04/2011		Eyes	Normal		
S170	204	Hepatitis B	Hepatitis B	3	Day 187			06/04/2011		Ears, nose and throat	Normal		
S170	204	Hepatitis B	Hepatitis B	3	Day 187			06/04/2011		Cardiovascular	Normal		
S170	204	Hepatitis B	Hepatitis B	3	Day 187			06/04/2011		Respiratory	Normal		

Subject No.	Randomisation Number	Study Group	Dosage	Dose	Day	Not Done	Specify	Date of Assessment	*Was the assessment performed prior to vaccination?	Physical Examination Parameter	Result	Abnormality	Clinical Significance
S170	204	Hepatitis B	Hepatitis B	3	Day 187			06/04/2011		Musculoskeletal	Normal		
S170	204	Hepatitis B	Hepatitis B	3	Day 187			06/04/2011		Venous system	Normal		
S170	204	Hepatitis B	Hepatitis B	3	Day 187			06/04/2011		Gastrointestinal	Normal		
S170	204	Hepatitis B	Hepatitis B	3	Day 187			06/04/2011		Lymphatic	Normal		
S170	204	Hepatitis B	Hepatitis B	3	Day 187			06/04/2011		Neurological	Normal		
S170	204	Hepatitis B	Hepatitis B	3	Day 187			06/04/2011		Genitourinary	Normal		
S170	204	Hepatitis B	Hepatitis B	3	Day 187			06/04/2011		Dermatological	Normal		
S170	204	Hepatitis B	Hepatitis B	3	Day 194			11/04/2011		Head and Neck	Normal		
S170	204	Hepatitis B	Hepatitis B	3	Day 194			11/04/2011		Eyes	Normal		
S170	204	Hepatitis B	Hepatitis B	3	Day 194			11/04/2011		Ears, nose and throat	Normal		
S170	204	Hepatitis B	Hepatitis B	3	Day 194			11/04/2011		Cardiovascular	Normal		
S170	204	Hepatitis B	Hepatitis B	3	Day 194			11/04/2011		Respiratory	Normal		
S170	204	Hepatitis B	Hepatitis B	3	Day 194			11/04/2011		Musculoskeletal	Normal		
S170	204	Hepatitis B	Hepatitis B	3	Day 194			11/04/2011		Venous system	Normal		
S170	204	Hepatitis B	Hepatitis B	3	Day 194			11/04/2011		Gastrointestinal	Normal		
S170	204	Hepatitis B	Hepatitis B	3	Day 194			11/04/2011		Lymphatic	Normal		
S170	204	Hepatitis B	Hepatitis B	3	Day 194			11/04/2011		Neurological	Normal		
S170	204	Hepatitis B	Hepatitis B	3	Day 194			11/04/2011		Genitourinary	Normal		
S170	204	Hepatitis B	Hepatitis B	3	Day 194			11/04/2011		Dermatological	Normal		

Subject No.	Randomisation Number	Study Group	Dosage	Dose	Day	Not Done	Specify	Date of Assessment	*Was the assessment performed prior to vaccination?	Physical Examination Parameter	Result	Abnormality	Clinical Significance
S170	204	Hepatitis B	Hepatitis B	3	Day 208			25/04/2011		Head and Neck	Normal		
S170	204	Hepatitis B	Hepatitis B	3	Day 208			25/04/2011		Eyes	Normal		
S170	204	Hepatitis B	Hepatitis B	3	Day 208			25/04/2011		Ears, nose and throat	Normal		
S170	204	Hepatitis B	Hepatitis B	3	Day 208			25/04/2011		Cardiovascular	Normal		
S170	204	Hepatitis B	Hepatitis B	3	Day 208			25/04/2011		Respiratory	Normal		
S170	204	Hepatitis B	Hepatitis B	3	Day 208			25/04/2011		Musculoskeletal	Normal		
S170	204	Hepatitis B	Hepatitis B	3	Day 208			25/04/2011		Venous system	Normal		
S170	204	Hepatitis B	Hepatitis B	3	Day 208			25/04/2011		Gastrointestinal	Normal		
S170	204	Hepatitis B	Hepatitis B	3	Day 208			25/04/2011		Lymphatic	Normal		
S170	204	Hepatitis B	Hepatitis B	3	Day 208			25/04/2011		Neurological	Normal		
S170	204	Hepatitis B	Hepatitis B	3	Day 208			25/04/2011		Genitourinary	Normal		
S170	204	Hepatitis B	Hepatitis B	3	Day 208			25/04/2011		Dermatological	Normal		
S170	204	Hepatitis B	Hepatitis B	3	Day 365			28/09/2011		Head and Neck	Normal		
S170	204	Hepatitis B	Hepatitis B	3	Day 365			28/09/2011		Eyes	Normal		
S170	204	Hepatitis B	Hepatitis B	3	Day 365			28/09/2011		Ears, nose and throat	Normal		
S170	204	Hepatitis B	Hepatitis B	3	Day 365			28/09/2011		Cardiovascular	Normal		
S170	204	Hepatitis B	Hepatitis B	3	Day 365			28/09/2011		Respiratory	Normal		
S170	204	Hepatitis B	Hepatitis B	3	Day 365			28/09/2011		Musculoskeletal	Normal		
S170	204	Hepatitis B	Hepatitis B	3	Day 365			28/09/2011		Venous system	Normal		

Subject No.	Randomisation Number	Study Group	Dosage	Dose	Day	Not Done	Specify	Date of Assessment	*Was the assessment performed prior to vaccination?	Physical Examination Parameter	Result	Abnormality	Clinical Significance
S170	204	Hepatitis B	Hepatitis B	3	Day 365			28/09/2011		Gastrointestinal	Normal		
S170	204	Hepatitis B	Hepatitis B	3	Day 365			28/09/2011		Lymphatic	Normal		
S170	204	Hepatitis B	Hepatitis B	3	Day 365			28/09/2011		Neurological	Normal		
S170	204	Hepatitis B	Hepatitis B	3	Day 365			28/09/2011		Genitourinary	Normal		
S170	204	Hepatitis B	Hepatitis B	3	Day 365			28/09/2011		Dermatological	Normal		
S175	209	JAIVAC-1	Dosage B		Day -14 to Day -1			21/09/2010		Head and neck	Normal		
S175	209	JAIVAC-1	Dosage B		Day -14 to Day -1			21/09/2010		Eyes	Normal		
S175	209	JAIVAC-1	Dosage B		Day -14 to Day -1			21/09/2010		Ears, nose and throat	Normal		
S175	209	JAIVAC-1	Dosage B		Day -14 to Day -1			21/09/2010		Cardiovascular	Normal		
S175	209	JAIVAC-1	Dosage B		Day -14 to Day -1			21/09/2010		Respiratory	Normal		
S175	209	JAIVAC-1	Dosage B		Day -14 to Day -1			21/09/2010		Musculoskeletal	Normal		
S175	209	JAIVAC-1	Dosage B		Day -14 to Day -1			21/09/2010		Venous system	Normal		
S175	209	JAIVAC-1	Dosage B		Day -14 to Day -1			21/09/2010		Gastrointestinal	Normal		
S175	209	JAIVAC-1	Dosage B		Day -14 to Day -1			21/09/2010		Lymphatic	Normal		
S175	209	JAIVAC-1	Dosage B		Day -14 to Day -1			21/09/2010		Neurological	Normal		
S175	209	JAIVAC-1	Dosage B		Day -14 to Day -1			21/09/2010		Genitourinary	Normal		
S175	209	JAIVAC-1	Dosage B		Day -14 to Day -1			21/09/2010		Dermatological	Normal		

Subject No.	Randomisation Number	Study Group	Dosage	Dose	Day	Not Done	Specify	Date of Assessment	*Was the assessment performed prior to vaccination?	Physical Examination Parameter	Result	Abnormality	Clinical Significance
S175	209	JAIVAC-1	Dosage B	1	Day 0			29/09/2010	Yes	Head and neck	Normal		
S175	209	JAIVAC-1	Dosage B	1	Day 0			29/09/2010	Yes	Eyes	Normal		
S175	209	JAIVAC-1	Dosage B	1	Day 0			29/09/2010	Yes	Ears, nose and throat	Normal		
S175	209	JAIVAC-1	Dosage B	1	Day 0			29/09/2010	Yes	Cardiovascular	Normal		
S175	209	JAIVAC-1	Dosage B	1	Day 0			29/09/2010	Yes	Respiratory	Normal		
S175	209	JAIVAC-1	Dosage B	1	Day 0			29/09/2010	Yes	Musculoskeletal	Normal		
S175	209	JAIVAC-1	Dosage B	1	Day 0			29/09/2010	Yes	Venous system	Normal		
S175	209	JAIVAC-1	Dosage B	1	Day 0			29/09/2010	Yes	Gastrointestinal	Normal		
S175	209	JAIVAC-1	Dosage B	1	Day 0			29/09/2010	Yes	Lymphatic	Normal		
S175	209	JAIVAC-1	Dosage B	1	Day 0			29/09/2010	Yes	Neurological	Normal		
S175	209	JAIVAC-1	Dosage B	1	Day 0			29/09/2010	Yes	Genitourinary	Normal		
S175	209	JAIVAC-1	Dosage B	1	Day 0			29/09/2010	Yes	Dermatological	Normal		
S175	209	JAIVAC-1	Dosage B	1	Day 7			06/10/2010		Head and Neck	Normal		
S175	209	JAIVAC-1	Dosage B	1	Day 7			06/10/2010		Eyes	Normal		
S175	209	JAIVAC-1	Dosage B	1	Day 7			06/10/2010		Ears, nose and throat	Normal		
S175	209	JAIVAC-1	Dosage B	1	Day 7			06/10/2010		Cardiovascular	Normal		
S175	209	JAIVAC-1	Dosage B	1	Day 7			06/10/2010		Respiratory	Normal		
S175	209	JAIVAC-1	Dosage B	1	Day 7			06/10/2010		Musculoskeletal	Normal		
S175	209	JAIVAC-1	Dosage B	1	Day 7			06/10/2010		Venous system	Normal		

Subject No.	Randomisation Number	Study Group	Dosage	Dose	Day	Not Done	Specify	Date of Assessment	*Was the assessment performed prior to vaccination?	Physical Examination Parameter	Result	Abnormality	Clinical Significance
S175	209	JAIVAC-1	Dosage B	1	Day 7			06/10/2010		Gastrointestinal	Normal		
S175	209	JAIVAC-1	Dosage B	1	Day 7			06/10/2010		Lymphatic	Normal		
S175	209	JAIVAC-1	Dosage B	1	Day 7			06/10/2010		Neurological	Normal		
S175	209	JAIVAC-1	Dosage B	1	Day 7			06/10/2010		Genitourinary	Normal		
S175	209	JAIVAC-1	Dosage B	1	Day 7			06/10/2010		Dermatological	Normal		
S175	209	JAIVAC-1	Dosage B	1	Day 14			13/10/2010		Head and Neck	Normal		
S175	209	JAIVAC-1	Dosage B	1	Day 14			13/10/2010		Eyes	Normal		
S175	209	JAIVAC-1	Dosage B	1	Day 14			13/10/2010		Ears, nose and throat	Normal		
S175	209	JAIVAC-1	Dosage B	1	Day 14			13/10/2010		Cardiovascular	Normal		
S175	209	JAIVAC-1	Dosage B	1	Day 14			13/10/2010		Respiratory	Normal		
S175	209	JAIVAC-1	Dosage B	1	Day 14			13/10/2010		Musculoskeletal	Normal		
S175	209	JAIVAC-1	Dosage B	1	Day 14			13/10/2010		Venous system	Normal		
S175	209	JAIVAC-1	Dosage B	1	Day 14			13/10/2010		Gastrointestinal	Normal		
S175	209	JAIVAC-1	Dosage B	1	Day 14			13/10/2010		Lymphatic	Normal		
S175	209	JAIVAC-1	Dosage B	1	Day 14			13/10/2010		Neurological	Normal		
S175	209	JAIVAC-1	Dosage B	1	Day 14			13/10/2010		Genitourinary	Normal		
S175	209	JAIVAC-1	Dosage B	1	Day 14			13/10/2010		Dermatological	Normal		
S175	209	JAIVAC-1	Dosage B	2	Day 28			27/10/2010	Yes	Head and Neck	Normal		
S175	209	JAIVAC-1	Dosage B	2	Day 28			27/10/2010	Yes	Eyes	Normal		

Subject No.	Randomisation Number	Study Group	Dosage	Dose	Day	Not Done	Specify	Date of Assessment	*Was the assessment performed prior to vaccination?	Physical Examination Parameter	Result	Abnormality	Clinical Significance
S175	209	JAIVAC-1	Dosage B	2	Day 28			27/10/2010	Yes	Ears, nose and throat	Normal		
S175	209	JAIVAC-1	Dosage B	2	Day 28			27/10/2010	Yes	Cardiovascular	Normal		
S175	209	JAIVAC-1	Dosage B	2	Day 28			27/10/2010	Yes	Respiratory	Normal		
S175	209	JAIVAC-1	Dosage B	2	Day 28			27/10/2010	Yes	Musculoskeletal	Normal		
S175	209	JAIVAC-1	Dosage B	2	Day 28			27/10/2010	Yes	Venous system	Normal		
S175	209	JAIVAC-1	Dosage B	2	Day 28			27/10/2010	Yes	Gastrointestinal	Normal		
S175	209	JAIVAC-1	Dosage B	2	Day 28			27/10/2010	Yes	Lymphatic	Normal		
S175	209	JAIVAC-1	Dosage B	2	Day 28			27/10/2010	Yes	Neurological	Normal		
S175	209	JAIVAC-1	Dosage B	2	Day 28			27/10/2010	Yes	Genitourinary	Normal		
S175	209	JAIVAC-1	Dosage B	2	Day 28			27/10/2010	Yes	Dermatological	Normal		
S175	209	JAIVAC-1	Dosage B	2	Day 35			03/11/2010		Head and Neck	Normal		
S175	209	JAIVAC-1	Dosage B	2	Day 35			03/11/2010		Eyes	Normal		
S175	209	JAIVAC-1	Dosage B	2	Day 35			03/11/2010		Ears, nose and throat	Normal		
S175	209	JAIVAC-1	Dosage B	2	Day 35			03/11/2010		Cardiovascular	Normal		
S175	209	JAIVAC-1	Dosage B	2	Day 35			03/11/2010		Respiratory	Normal		
S175	209	JAIVAC-1	Dosage B	2	Day 35			03/11/2010		Musculoskeletal	Normal		
S175	209	JAIVAC-1	Dosage B	2	Day 35			03/11/2010		Venous system	Normal		
S175	209	JAIVAC-1	Dosage B	2	Day 35			03/11/2010		Gastrointestinal	Normal		
S175	209	JAIVAC-1	Dosage B	2	Day 35			03/11/2010		Lymphatic	Normal		

Subject No.	Randomisation Number	Study Group	Dosage	Dose	Day	Not Done	Specify	Date of Assessment	*Was the assessment performed prior to vaccination?	Physical Examination Parameter	Result	Abnormality	Clinical Significance
S175	209	JAIVAC-1	Dosage B	2	Day 35			03/11/2010		Neurological	Normal		
S175	209	JAIVAC-1	Dosage B	2	Day 35			03/11/2010		Genitourinary	Normal		
S175	209	JAIVAC-1	Dosage B	2	Day 35			03/11/2010		Dermatological	Normal		
S175	209	JAIVAC-1	Dosage B	2	Day 42			10/11/2010		Head and Neck	Normal		
S175	209	JAIVAC-1	Dosage B	2	Day 42			10/11/2010		Eyes	Normal		
S175	209	JAIVAC-1	Dosage B	2	Day 42			10/11/2010		Ears, nose and throat	Normal		
S175	209	JAIVAC-1	Dosage B	2	Day 42			10/11/2010		Cardiovascular	Normal		
S175	209	JAIVAC-1	Dosage B	2	Day 42			10/11/2010		Respiratory	Normal		
S175	209	JAIVAC-1	Dosage B	2	Day 42			10/11/2010		Musculoskeletal	Normal		
S175	209	JAIVAC-1	Dosage B	2	Day 42			10/11/2010		Venous system	Normal		
S175	209	JAIVAC-1	Dosage B	2	Day 42			10/11/2010		Gastrointestinal	Normal		
S175	209	JAIVAC-1	Dosage B	2	Day 42			10/11/2010		Lymphatic	Normal		
S175	209	JAIVAC-1	Dosage B	2	Day 42			10/11/2010		Neurological	Normal		
S175	209	JAIVAC-1	Dosage B	2	Day 42			10/11/2010		Genitourinary	Normal		
S175	209	JAIVAC-1	Dosage B	2	Day 42			10/11/2010		Dermatological	Normal		
S175	209	JAIVAC-1	Dosage B	2	Day 56			24/11/2010		Head and Neck	Normal		
S175	209	JAIVAC-1	Dosage B	2	Day 56			24/11/2010		Eyes	Normal		
S175	209	JAIVAC-1	Dosage B	2	Day 56			24/11/2010		Ears, nose and throat	Normal		
S175	209	JAIVAC-1	Dosage B	2	Day 56			24/11/2010		Cardiovascular	Normal		

Subject No.	Randomisation Number	Study Group	Dosage	Dose	Day	Not Done	Specify	Date of Assessment	*Was the assessment performed prior to vaccination?	Physical Examination Parameter	Result	Abnormality	Clinical Significance
S175	209	JAIVAC-1	Dosage B	2	Day 56			24/11/2010		Respiratory	Normal		
S175	209	JAIVAC-1	Dosage B	2	Day 56			24/11/2010		Musculoskeletal	Normal		
S175	209	JAIVAC-1	Dosage B	2	Day 56			24/11/2010		Venous system	Normal		
S175	209	JAIVAC-1	Dosage B	2	Day 56			24/11/2010		Gastrointestinal	Normal		
S175	209	JAIVAC-1	Dosage B	2	Day 56			24/11/2010		Lymphatic	Normal		
S175	209	JAIVAC-1	Dosage B	2	Day 56			24/11/2010		Neurological	Normal		
S175	209	JAIVAC-1	Dosage B	2	Day 56			24/11/2010		Genitourinary	Normal		
S175	209	JAIVAC-1	Dosage B	2	Day 56			24/11/2010		Dermatological	Normal		
S175	209	JAIVAC-1	Dosage B	3	Day 180			28/03/2011	Yes	Head and Neck	Normal		
S175	209	JAIVAC-1	Dosage B	3	Day 180			28/03/2011	Yes	Eyes	Normal		
S175	209	JAIVAC-1	Dosage B	3	Day 180			28/03/2011	Yes	Ears, nose and throat	Normal		
S175	209	JAIVAC-1	Dosage B	3	Day 180			28/03/2011	Yes	Cardiovascular	Normal		
S175	209	JAIVAC-1	Dosage B	3	Day 180			28/03/2011	Yes	Respiratory	Normal		
S175	209	JAIVAC-1	Dosage B	3	Day 180			28/03/2011	Yes	Musculoskeletal	Normal		
S175	209	JAIVAC-1	Dosage B	3	Day 180			28/03/2011	Yes	Venous system	Normal		
S175	209	JAIVAC-1	Dosage B	3	Day 180			28/03/2011	Yes	Gastrointestinal	Normal		
S175	209	JAIVAC-1	Dosage B	3	Day 180			28/03/2011	Yes	Lymphatic	Normal		
S175	209	JAIVAC-1	Dosage B	3	Day 180			28/03/2011	Yes	Neurological	Normal		
S175	209	JAIVAC-1	Dosage B	3	Day 180			28/03/2011	Yes	Genitourinary	Normal		

Subject No.	Randomisation Number	Study Group	Dosage	Dose	Day	Not Done	Specify	Date of Assessment	*Was the assessment performed prior to vaccination?	Physical Examination Parameter	Result	Abnormality	Clinical Significance
S175	209	JAIVAC-1	Dosage B	3	Day 180			28/03/2011	Yes	Dermatological	Normal		
S175	209	JAIVAC-1	Dosage B	3	Day 187			05/04/2011		Head and Neck	Normal		
S175	209	JAIVAC-1	Dosage B	3	Day 187			05/04/2011		Eyes	Normal		
S175	209	JAIVAC-1	Dosage B	3	Day 187			05/04/2011		Ears, nose and throat	Normal		
S175	209	JAIVAC-1	Dosage B	3	Day 187			05/04/2011		Cardiovascular	Normal		
S175	209	JAIVAC-1	Dosage B	3	Day 187			05/04/2011		Respiratory	Normal		
S175	209	JAIVAC-1	Dosage B	3	Day 187			05/04/2011		Musculoskeletal	Normal		
S175	209	JAIVAC-1	Dosage B	3	Day 187			05/04/2011		Venous system	Normal		
S175	209	JAIVAC-1	Dosage B	3	Day 187			05/04/2011		Gastrointestinal	Normal		
S175	209	JAIVAC-1	Dosage B	3	Day 187			05/04/2011		Lymphatic	Normal		
S175	209	JAIVAC-1	Dosage B	3	Day 187			05/04/2011		Neurological	Normal		
S175	209	JAIVAC-1	Dosage B	3	Day 187			05/04/2011		Genitourinary	Normal		
S175	209	JAIVAC-1	Dosage B	3	Day 187			05/04/2011		Dermatological	Normal		
S175	209	JAIVAC-1	Dosage B	3	Day 194			11/04/2011		Head and Neck	Normal		
S175	209	JAIVAC-1	Dosage B	3	Day 194			11/04/2011		Eyes	Normal		
S175	209	JAIVAC-1	Dosage B	3	Day 194			11/04/2011		Ears, nose and throat	Normal		
S175	209	JAIVAC-1	Dosage B	3	Day 194			11/04/2011		Cardiovascular	Normal		
S175	209	JAIVAC-1	Dosage B	3	Day 194			11/04/2011		Respiratory	Normal		
S175	209	JAIVAC-1	Dosage B	3	Day 194			11/04/2011		Musculoskeletal	Normal		

Subject No.	Randomisation Number	Study Group	Dosage	Dose	Day	Not Done	Specify	Date of Assessment	*Was the assessment performed prior to vaccination?	Physical Examination Parameter	Result	Abnormality	Clinical Significance
S175	209	JAIVAC-1	Dosage B	3	Day 194			11/04/2011		Venous system	Normal		
S175	209	JAIVAC-1	Dosage B	3	Day 194			11/04/2011		Gastrointestinal	Normal		
S175	209	JAIVAC-1	Dosage B	3	Day 194			11/04/2011		Lymphatic	Normal		
S175	209	JAIVAC-1	Dosage B	3	Day 194			11/04/2011		Neurological	Normal		
S175	209	JAIVAC-1	Dosage B	3	Day 194			11/04/2011		Genitourinary	Normal		
S175	209	JAIVAC-1	Dosage B	3	Day 194			11/04/2011		Dermatological	Normal		
S175	209	JAIVAC-1	Dosage B	3	Day 208			25/04/2011		Head and Neck	Normal		
S175	209	JAIVAC-1	Dosage B	3	Day 208			25/04/2011		Eyes	Normal		
S175	209	JAIVAC-1	Dosage B	3	Day 208			25/04/2011		Ears, nose and throat	Normal		
S175	209	JAIVAC-1	Dosage B	3	Day 208			25/04/2011		Cardiovascular	Normal		
S175	209	JAIVAC-1	Dosage B	3	Day 208			25/04/2011		Respiratory	Normal		
S175	209	JAIVAC-1	Dosage B	3	Day 208			25/04/2011		Musculoskeletal	Normal		
S175	209	JAIVAC-1	Dosage B	3	Day 208			25/04/2011		Venous system	Normal		
S175	209	JAIVAC-1	Dosage B	3	Day 208			25/04/2011		Gastrointestinal	Normal		
S175	209	JAIVAC-1	Dosage B	3	Day 208			25/04/2011		Lymphatic	Normal		
S175	209	JAIVAC-1	Dosage B	3	Day 208			25/04/2011		Neurological	Normal		
S175	209	JAIVAC-1	Dosage B	3	Day 208			25/04/2011		Genitourinary	Normal		
S175	209	JAIVAC-1	Dosage B	3	Day 208			25/04/2011		Dermatological	Normal		
S175	209	JAIVAC-1	Dosage B	3	Day 365			28/09/2011		Head and Neck	Normal		

Subject No.	Randomisation Number	Study Group	Dosage	Dose	Day	Not Done	Specify	Date of Assessment	*Was the assessment performed prior to vaccination?	Physical Examination Parameter	Result	Abnormality	Clinical Significance
S175	209	JAIVAC-1	Dosage B	3	Day 365			28/09/2011		Eyes	Normal		
S175	209	JAIVAC-1	Dosage B	3	Day 365			28/09/2011		Ears, nose and throat	Normal		
S175	209	JAIVAC-1	Dosage B	3	Day 365			28/09/2011		Cardiovascular	Normal		
S175	209	JAIVAC-1	Dosage B	3	Day 365			28/09/2011		Respiratory	Normal		
S175	209	JAIVAC-1	Dosage B	3	Day 365			28/09/2011		Musculoskeletal	Normal		
S175	209	JAIVAC-1	Dosage B	3	Day 365			28/09/2011		Venous system	Normal		
S175	209	JAIVAC-1	Dosage B	3	Day 365			28/09/2011		Gastrointestinal	Normal		
S175	209	JAIVAC-1	Dosage B	3	Day 365			28/09/2011		Lymphatic	Normal		
S175	209	JAIVAC-1	Dosage B	3	Day 365			28/09/2011		Neurological	Normal		
S175	209	JAIVAC-1	Dosage B	3	Day 365			28/09/2011		Genitourinary	Normal		
S175	209	JAIVAC-1	Dosage B	3	Day 365			28/09/2011		Dermatological	Normal		
S176	205	Hepatitis B	Hepatitis B		Day -14 to Day -1			22/09/2010		Head and neck	Normal		
S176	205	Hepatitis B	Hepatitis B		Day -14 to Day -1			22/09/2010		Eyes	Normal		
S176	205	Hepatitis B	Hepatitis B		Day -14 to Day -1			22/09/2010		Ears, nose and throat	Normal		
S176	205	Hepatitis B	Hepatitis B		Day -14 to Day -1			22/09/2010		Cardiovascular	Normal		
S176	205	Hepatitis B	Hepatitis B		Day -14 to Day -1			22/09/2010		Respiratory	Normal		
S176	205	Hepatitis B	Hepatitis B		Day -14 to Day -1			22/09/2010		Musculoskeletal	Normal		
S176	205	Hepatitis B	Hepatitis B		Day -14 to Day -1			22/09/2010		Venous system	Normal		

Subject No.	Randomisation Number	Study Group	Dosage	Dose	Day	Not Done	Specify	Date of Assessment	*Was the assessment performed prior to vaccination?	Physical Examination Parameter	Result	Abnormality	Clinical Significance
S176	205	Hepatitis B	Hepatitis B		Day -14 to Day -1			22/09/2010		Gastrointestinal	Normal		
S176	205	Hepatitis B	Hepatitis B		Day -14 to Day -1			22/09/2010		Lymphatic	Normal		
S176	205	Hepatitis B	Hepatitis B		Day -14 to Day -1			22/09/2010		Neurological	Normal		
S176	205	Hepatitis B	Hepatitis B		Day -14 to Day -1			22/09/2010		Genitourinary	Normal		
S176	205	Hepatitis B	Hepatitis B		Day -14 to Day -1			22/09/2010		Dermatological	Normal		
S176	205	Hepatitis B	Hepatitis B	1	Day 0			29/09/2010	Yes	Head and neck	Normal		
S176	205	Hepatitis B	Hepatitis B	1	Day 0			29/09/2010	Yes	Eyes	Normal		
S176	205	Hepatitis B	Hepatitis B	1	Day 0			29/09/2010	Yes	Ears, nose and throat	Normal		
S176	205	Hepatitis B	Hepatitis B	1	Day 0			29/09/2010	Yes	Cardiovascular	Normal		
S176	205	Hepatitis B	Hepatitis B	1	Day 0			29/09/2010	Yes	Respiratory	Normal		
S176	205	Hepatitis B	Hepatitis B	1	Day 0			29/09/2010	Yes	Musculoskeletal	Normal		
S176	205	Hepatitis B	Hepatitis B	1	Day 0			29/09/2010	Yes	Venous system	Normal		
S176	205	Hepatitis B	Hepatitis B	1	Day 0			29/09/2010	Yes	Gastrointestinal	Normal		
S176	205	Hepatitis B	Hepatitis B	1	Day 0			29/09/2010	Yes	Lymphatic	Normal		
S176	205	Hepatitis B	Hepatitis B	1	Day 0			29/09/2010	Yes	Neurological	Normal		
S176	205	Hepatitis B	Hepatitis B	1	Day 0			29/09/2010	Yes	Genitourinary	Normal		
S176	205	Hepatitis B	Hepatitis B	1	Day 0			29/09/2010	Yes	Dermatological	Normal		
S176	205	Hepatitis B	Hepatitis B	1	Day 7			06/10/2010		Head and Neck	Normal		

Subject No.	Randomisation Number	Study Group	Dosage	Dose	Day	Not Done	Specify	Date of Assessment	*Was the assessment performed prior to vaccination?	Physical Examination Parameter	Result	Abnormality	Clinical Significance
S176	205	Hepatitis B	Hepatitis B	1	Day 7			06/10/2010		Eyes	Normal		
S176	205	Hepatitis B	Hepatitis B	1	Day 7			06/10/2010		Ears, nose and throat	Normal		
S176	205	Hepatitis B	Hepatitis B	1	Day 7			06/10/2010		Cardiovascular	Normal		
S176	205	Hepatitis B	Hepatitis B	1	Day 7			06/10/2010		Respiratory	Normal		
S176	205	Hepatitis B	Hepatitis B	1	Day 7			06/10/2010		Musculoskeletal	Normal		
S176	205	Hepatitis B	Hepatitis B	1	Day 7			06/10/2010		Venous system	Normal		
S176	205	Hepatitis B	Hepatitis B	1	Day 7			06/10/2010		Gastrointestinal	Normal		
S176	205	Hepatitis B	Hepatitis B	1	Day 7			06/10/2010		Lymphatic	Normal		
S176	205	Hepatitis B	Hepatitis B	1	Day 7			06/10/2010		Neurological	Normal		
S176	205	Hepatitis B	Hepatitis B	1	Day 7			06/10/2010		Genitourinary	Normal		
S176	205	Hepatitis B	Hepatitis B	1	Day 7			06/10/2010		Dermatological	Normal		
S176	205	Hepatitis B	Hepatitis B	1	Day 14			13/10/2010		Head and Neck	Normal		
S176	205	Hepatitis B	Hepatitis B	1	Day 14			13/10/2010		Eyes	Normal		
S176	205	Hepatitis B	Hepatitis B	1	Day 14			13/10/2010		Ears, nose and throat	Normal		
S176	205	Hepatitis B	Hepatitis B	1	Day 14			13/10/2010		Cardiovascular	Normal		
S176	205	Hepatitis B	Hepatitis B	1	Day 14			13/10/2010		Respiratory	Normal		
S176	205	Hepatitis B	Hepatitis B	1	Day 14			13/10/2010		Musculoskeletal	Normal		
S176	205	Hepatitis B	Hepatitis B	1	Day 14			13/10/2010		Venous system	Normal		
S176	205	Hepatitis B	Hepatitis B	1	Day 14			13/10/2010		Gastrointestinal	Normal		

Subject No.	Randomisation Number	Study Group	Dosage	Dose	Day	Not Done	Specify	Date of Assessment	*Was the assessment performed prior to vaccination?	Physical Examination Parameter	Result	Abnormality	Clinical Significance
S176	205	Hepatitis B	Hepatitis B	1	Day 14			13/10/2010		Lymphatic	Normal		
S176	205	Hepatitis B	Hepatitis B	1	Day 14			13/10/2010		Neurological	Normal		
S176	205	Hepatitis B	Hepatitis B	1	Day 14			13/10/2010		Genitourinary	Normal		
S176	205	Hepatitis B	Hepatitis B	1	Day 14			13/10/2010		Dermatological	Normal		
S176	205	Hepatitis B	Hepatitis B	2	Day 28			27/10/2010	Yes	Head and Neck	Normal		
S176	205	Hepatitis B	Hepatitis B	2	Day 28			27/10/2010	Yes	Eyes	Normal		
S176	205	Hepatitis B	Hepatitis B	2	Day 28			27/10/2010	Yes	Ears, nose and throat	Normal		
S176	205	Hepatitis B	Hepatitis B	2	Day 28			27/10/2010	Yes	Cardiovascular	Normal		
S176	205	Hepatitis B	Hepatitis B	2	Day 28			27/10/2010	Yes	Respiratory	Normal		
S176	205	Hepatitis B	Hepatitis B	2	Day 28			27/10/2010	Yes	Musculoskeletal	Normal		
S176	205	Hepatitis B	Hepatitis B	2	Day 28			27/10/2010	Yes	Venous system	Normal		
S176	205	Hepatitis B	Hepatitis B	2	Day 28			27/10/2010	Yes	Gastrointestinal	Normal		
S176	205	Hepatitis B	Hepatitis B	2	Day 28			27/10/2010	Yes	Lymphatic	Normal		
S176	205	Hepatitis B	Hepatitis B	2	Day 28			27/10/2010	Yes	Neurological	Normal		
S176	205	Hepatitis B	Hepatitis B	2	Day 28			27/10/2010	Yes	Genitourinary	Normal		
S176	205	Hepatitis B	Hepatitis B	2	Day 28			27/10/2010	Yes	Dermatological	Normal		
S176	205	Hepatitis B	Hepatitis B	2	Day 35			03/11/2010		Head and Neck	Normal		
S176	205	Hepatitis B	Hepatitis B	2	Day 35			03/11/2010		Eyes	Normal		
S176	205	Hepatitis B	Hepatitis B	2	Day 35			03/11/2010		Ears, nose and throat	Normal		

Subject No.	Randomisation Number	Study Group	Dosage	Dose	Day	Not Done	Specify	Date of Assessment	*Was the assessment performed prior to vaccination?	Physical Examination Parameter	Result	Abnormality	Clinical Significance
S176	205	Hepatitis B	Hepatitis B	2	Day 35			03/11/2010		Cardiovascular	Normal		
S176	205	Hepatitis B	Hepatitis B	2	Day 35			03/11/2010		Respiratory	Normal		
S176	205	Hepatitis B	Hepatitis B	2	Day 35			03/11/2010		Musculoskeletal	Normal		
S176	205	Hepatitis B	Hepatitis B	2	Day 35			03/11/2010		Venous system	Normal		
S176	205	Hepatitis B	Hepatitis B	2	Day 35			03/11/2010		Gastrointestinal	Normal		
S176	205	Hepatitis B	Hepatitis B	2	Day 35			03/11/2010		Lymphatic	Normal		
S176	205	Hepatitis B	Hepatitis B	2	Day 35			03/11/2010		Neurological	Normal		
S176	205	Hepatitis B	Hepatitis B	2	Day 35			03/11/2010		Genitourinary	Normal		
S176	205	Hepatitis B	Hepatitis B	2	Day 35			03/11/2010		Dermatological	Normal		
S176	205	Hepatitis B	Hepatitis B	2	Day 42			10/11/2010		Head and Neck	Normal		
S176	205	Hepatitis B	Hepatitis B	2	Day 42			10/11/2010		Eyes	Normal		
S176	205	Hepatitis B	Hepatitis B	2	Day 42			10/11/2010		Ears, nose and throat	Normal		
S176	205	Hepatitis B	Hepatitis B	2	Day 42			10/11/2010		Cardiovascular	Normal		
S176	205	Hepatitis B	Hepatitis B	2	Day 42			10/11/2010		Respiratory	Normal		
S176	205	Hepatitis B	Hepatitis B	2	Day 42			10/11/2010		Musculoskeletal	Normal		
S176	205	Hepatitis B	Hepatitis B	2	Day 42			10/11/2010		Venous system	Normal		
S176	205	Hepatitis B	Hepatitis B	2	Day 42			10/11/2010		Gastrointestinal	Normal		
S176	205	Hepatitis B	Hepatitis B	2	Day 42			10/11/2010		Lymphatic	Normal		
S176	205	Hepatitis B	Hepatitis B	2	Day 42			10/11/2010		Neurological	Normal		

Subject No.	Randomisation Number	Study Group	Dosage	Dose	Day	Not Done	Specify	Date of Assessment	*Was the assessment performed prior to vaccination?	Physical Examination Parameter	Result	Abnormality	Clinical Significance
S176	205	Hepatitis B	Hepatitis B	2	Day 42			10/11/2010		Genitourinary	Normal		
S176	205	Hepatitis B	Hepatitis B	2	Day 42			10/11/2010		Dermatological	Normal		
S176	205	Hepatitis B	Hepatitis B	2	Day 56			24/11/2010		Head and Neck	Normal		
S176	205	Hepatitis B	Hepatitis B	2	Day 56			24/11/2010		Eyes	Normal		
S176	205	Hepatitis B	Hepatitis B	2	Day 56			24/11/2010		Ears, nose and throat	Normal		
S176	205	Hepatitis B	Hepatitis B	2	Day 56			24/11/2010		Cardiovascular	Normal		
S176	205	Hepatitis B	Hepatitis B	2	Day 56			24/11/2010		Respiratory	Normal		
S176	205	Hepatitis B	Hepatitis B	2	Day 56			24/11/2010		Musculoskeletal	Normal		
S176	205	Hepatitis B	Hepatitis B	2	Day 56			24/11/2010		Venous system	Normal		
S176	205	Hepatitis B	Hepatitis B	2	Day 56			24/11/2010		Gastrointestinal	Normal		
S176	205	Hepatitis B	Hepatitis B	2	Day 56			24/11/2010		Lymphatic	Normal		
S176	205	Hepatitis B	Hepatitis B	2	Day 56			24/11/2010		Neurological	Normal		
S176	205	Hepatitis B	Hepatitis B	2	Day 56			24/11/2010		Genitourinary	Normal		
S176	205	Hepatitis B	Hepatitis B	2	Day 56			24/11/2010		Dermatological	Normal		
S176	205	Hepatitis B	Hepatitis B	3	Day 180			28/03/2011	Yes	Head and Neck	Normal		
S176	205	Hepatitis B	Hepatitis B	3	Day 180			28/03/2011	Yes	Eyes	Normal		
S176	205	Hepatitis B	Hepatitis B	3	Day 180			28/03/2011	Yes	Ears, nose and throat	Normal		
S176	205	Hepatitis B	Hepatitis B	3	Day 180			28/03/2011	Yes	Cardiovascular	Normal		
S176	205	Hepatitis B	Hepatitis B	3	Day 180			28/03/2011	Yes	Respiratory	Normal		

Subject No.	Randomisation Number	Study Group	Dosage	Dose	Day	Not Done	Specify	Date of Assessment	*Was the assessment performed prior to vaccination?	Physical Examination Parameter	Result	Abnormality	Clinical Significance
S176	205	Hepatitis B	Hepatitis B	3	Day 180			28/03/2011	Yes	Musculoskeletal	Normal		
S176	205	Hepatitis B	Hepatitis B	3	Day 180			28/03/2011	Yes	Venous system	Normal		
S176	205	Hepatitis B	Hepatitis B	3	Day 180			28/03/2011	Yes	Gastrointestinal	Normal		
S176	205	Hepatitis B	Hepatitis B	3	Day 180			28/03/2011	Yes	Lymphatic	Normal		
S176	205	Hepatitis B	Hepatitis B	3	Day 180			28/03/2011	Yes	Neurological	Normal		
S176	205	Hepatitis B	Hepatitis B	3	Day 180			28/03/2011	Yes	Genitourinary	Normal		
S176	205	Hepatitis B	Hepatitis B	3	Day 180			28/03/2011	Yes	Dermatological	Normal		
S176	205	Hepatitis B	Hepatitis B	3	Day 187			05/04/2011		Head and Neck	Normal		
S176	205	Hepatitis B	Hepatitis B	3	Day 187			05/04/2011		Eyes	Normal		
S176	205	Hepatitis B	Hepatitis B	3	Day 187			05/04/2011		Ears, nose and throat	Normal		
S176	205	Hepatitis B	Hepatitis B	3	Day 187			05/04/2011		Cardiovascular	Normal		
S176	205	Hepatitis B	Hepatitis B	3	Day 187			05/04/2011		Respiratory	Normal		
S176	205	Hepatitis B	Hepatitis B	3	Day 187			05/04/2011		Musculoskeletal	Normal		
S176	205	Hepatitis B	Hepatitis B	3	Day 187			05/04/2011		Venous system	Normal		
S176	205	Hepatitis B	Hepatitis B	3	Day 187			05/04/2011		Gastrointestinal	Normal		
S176	205	Hepatitis B	Hepatitis B	3	Day 187			05/04/2011		Lymphatic	Normal		
S176	205	Hepatitis B	Hepatitis B	3	Day 187			05/04/2011		Neurological	Normal		
S176	205	Hepatitis B	Hepatitis B	3	Day 187			05/04/2011		Genitourinary	Normal		
S176	205	Hepatitis B	Hepatitis B	3	Day 187			05/04/2011		Dermatological	Normal		

Subject No.	Randomisation Number	Study Group	Dosage	Dose	Day	Not Done	Specify	Date of Assessment	*Was the assessment performed prior to vaccination?	Physical Examination Parameter	Result	Abnormality	Clinical Significance
S176	205	Hepatitis B	Hepatitis B	3	Day 194			11/04/2011		Head and Neck	Normal		
S176	205	Hepatitis B	Hepatitis B	3	Day 194			11/04/2011		Eyes	Normal		
S176	205	Hepatitis B	Hepatitis B	3	Day 194			11/04/2011		Ears, nose and throat	Normal		
S176	205	Hepatitis B	Hepatitis B	3	Day 194			11/04/2011		Cardiovascular	Normal		
S176	205	Hepatitis B	Hepatitis B	3	Day 194			11/04/2011		Respiratory	Normal		
S176	205	Hepatitis B	Hepatitis B	3	Day 194			11/04/2011		Musculoskeletal	Normal		
S176	205	Hepatitis B	Hepatitis B	3	Day 194			11/04/2011		Venous system	Normal		
S176	205	Hepatitis B	Hepatitis B	3	Day 194			11/04/2011		Gastrointestinal	Normal		
S176	205	Hepatitis B	Hepatitis B	3	Day 194			11/04/2011		Lymphatic	Normal		
S176	205	Hepatitis B	Hepatitis B	3	Day 194			11/04/2011		Neurological	Normal		
S176	205	Hepatitis B	Hepatitis B	3	Day 194			11/04/2011		Genitourinary	Normal		
S176	205	Hepatitis B	Hepatitis B	3	Day 194			11/04/2011		Dermatological	Normal		
S176	205	Hepatitis B	Hepatitis B	3	Day 208			25/04/2011		Head and Neck	Normal		
S176	205	Hepatitis B	Hepatitis B	3	Day 208			25/04/2011		Eyes	Normal		
S176	205	Hepatitis B	Hepatitis B	3	Day 208			25/04/2011		Ears, nose and throat	Normal		
S176	205	Hepatitis B	Hepatitis B	3	Day 208			25/04/2011		Cardiovascular	Normal		
S176	205	Hepatitis B	Hepatitis B	3	Day 208			25/04/2011		Respiratory	Normal		
S176	205	Hepatitis B	Hepatitis B	3	Day 208			25/04/2011		Musculoskeletal	Normal		
S176	205	Hepatitis B	Hepatitis B	3	Day 208			25/04/2011		Venous system	Normal		

Subject No.	Randomisation Number	Study Group	Dosage	Dose	Day	Not Done	Specify	Date of Assessment	*Was the assessment performed prior to vaccination?	Physical Examination Parameter	Result	Abnormality	Clinical Significance
S176	205	Hepatitis B	Hepatitis B	3	Day 208			25/04/2011		Gastrointestinal	Normal		
S176	205	Hepatitis B	Hepatitis B	3	Day 208			25/04/2011		Lymphatic	Normal		
S176	205	Hepatitis B	Hepatitis B	3	Day 208			25/04/2011		Neurological	Normal		
S176	205	Hepatitis B	Hepatitis B	3	Day 208			25/04/2011		Genitourinary	Normal		
S176	205	Hepatitis B	Hepatitis B	3	Day 208			25/04/2011		Dermatological	Normal		
S176	205	Hepatitis B	Hepatitis B	3	Day 365			28/09/2011		Head and Neck	Normal		
S176	205	Hepatitis B	Hepatitis B	3	Day 365			28/09/2011		Eyes	Normal		
S176	205	Hepatitis B	Hepatitis B	3	Day 365			28/09/2011		Ears, nose and throat	Normal		
S176	205	Hepatitis B	Hepatitis B	3	Day 365			28/09/2011		Cardiovascular	Normal		
S176	205	Hepatitis B	Hepatitis B	3	Day 365			28/09/2011		Respiratory	Normal		
S176	205	Hepatitis B	Hepatitis B	3	Day 365			28/09/2011		Musculoskeletal	Normal		
S176	205	Hepatitis B	Hepatitis B	3	Day 365			28/09/2011		Venous system	Normal		
S176	205	Hepatitis B	Hepatitis B	3	Day 365			28/09/2011		Gastrointestinal	Normal		
S176	205	Hepatitis B	Hepatitis B	3	Day 365			28/09/2011		Lymphatic	Normal		
S176	205	Hepatitis B	Hepatitis B	3	Day 365			28/09/2011		Neurological	Normal		
S176	205	Hepatitis B	Hepatitis B	3	Day 365			28/09/2011		Genitourinary	Normal		
S176	205	Hepatitis B	Hepatitis B	3	Day 365			28/09/2011		Dermatological	Normal		
S176	205	Hepatitis B	Hepatitis B		Unscheduled Visit	Yes	Laboratory Repeat At Local Lab	.		Head and Neck			

Subject No.	Randomisation Number	Study Group	Dosage	Dose	Day	Not Done	Specify	Date of Assessment	*Was the assessment performed prior to vaccination?	Physical Examination Parameter	Result	Abnormality	Clinical Significance
S176	205	Hepatitis B	Hepatitis B		Unscheduled Visit	Yes	Laboratory Repeat At Local Lab	.		Eyes			
S176	205	Hepatitis B	Hepatitis B		Unscheduled Visit	Yes	Laboratory Repeat At Local Lab	.		Ears, nose and throat			
S176	205	Hepatitis B	Hepatitis B		Unscheduled Visit	Yes	Laboratory Repeat At Local Lab	.		Cardiovascular			
S176	205	Hepatitis B	Hepatitis B		Unscheduled Visit	Yes	Laboratory Repeat At Local Lab	.		Respiratory			
S176	205	Hepatitis B	Hepatitis B		Unscheduled Visit	Yes	Laboratory Repeat At Local Lab	.		Musculoskeletal			
S176	205	Hepatitis B	Hepatitis B		Unscheduled Visit	Yes	Laboratory Repeat At Local Lab	.		Venous system			
S176	205	Hepatitis B	Hepatitis B		Unscheduled Visit	Yes	Laboratory Repeat At Local Lab	.		Gastrointestinal			
S176	205	Hepatitis B	Hepatitis B		Unscheduled Visit	Yes	Laboratory Repeat At Local Lab	.		Lymphatic			
S176	205	Hepatitis B	Hepatitis B		Unscheduled Visit	Yes	Laboratory Repeat At Local Lab	.		Neurological			
S176	205	Hepatitis B	Hepatitis B		Unscheduled Visit	Yes	Laboratory Repeat At Local Lab	.		Genitourinary			
S176	205	Hepatitis B	Hepatitis B		Unscheduled Visit	Yes	Laboratory Repeat At Local Lab	.		Dermatological			
S179	207	Hepatitis B	Hepatitis B		Day -14 to Day -1			22/09/2010		Head and neck	Normal		

Subject No.	Randomisation Number	Study Group	Dosage	Dose	Day	Not Done	Specify	Date of Assessment	*Was the assessment performed prior to vaccination?	Physical Examination Parameter	Result	Abnormality	Clinical Significance
S179	207	Hepatitis B	Hepatitis B		Day -14 to Day -1			22/09/2010		Eyes	Normal		
S179	207	Hepatitis B	Hepatitis B		Day -14 to Day -1			22/09/2010		Ears, nose and throat	Normal		
S179	207	Hepatitis B	Hepatitis B		Day -14 to Day -1			22/09/2010		Cardiovascular	Normal		
S179	207	Hepatitis B	Hepatitis B		Day -14 to Day -1			22/09/2010		Respiratory	Normal		
S179	207	Hepatitis B	Hepatitis B		Day -14 to Day -1			22/09/2010		Musculoskeletal	Normal		
S179	207	Hepatitis B	Hepatitis B		Day -14 to Day -1			22/09/2010		Venous system	Normal		
S179	207	Hepatitis B	Hepatitis B		Day -14 to Day -1			22/09/2010		Gastrointestinal	Normal		
S179	207	Hepatitis B	Hepatitis B		Day -14 to Day -1			22/09/2010		Lymphatic	Normal		
S179	207	Hepatitis B	Hepatitis B		Day -14 to Day -1			22/09/2010		Neurological	Normal		
S179	207	Hepatitis B	Hepatitis B		Day -14 to Day -1			22/09/2010		Genitourinary	Normal		
S179	207	Hepatitis B	Hepatitis B		Day -14 to Day -1			22/09/2010		Dermatological	Normal		
S179	207	Hepatitis B	Hepatitis B	1	Day 0			29/09/2010	Yes	Head and neck	Normal		
S179	207	Hepatitis B	Hepatitis B	1	Day 0			29/09/2010	Yes	Eyes	Normal		
S179	207	Hepatitis B	Hepatitis B	1	Day 0			29/09/2010	Yes	Ears, nose and throat	Normal		
S179	207	Hepatitis B	Hepatitis B	1	Day 0			29/09/2010	Yes	Cardiovascular	Normal		
S179	207	Hepatitis B	Hepatitis B	1	Day 0			29/09/2010	Yes	Respiratory	Normal		
S179	207	Hepatitis B	Hepatitis B	1	Day 0			29/09/2010	Yes	Musculoskeletal	Normal		

Subject No.	Randomisation Number	Study Group	Dosage	Dose	Day	Not Done	Specify	Date of Assessment	*Was the assessment performed prior to vaccination?	Physical Examination Parameter	Result	Abnormality	Clinical Significance
S179	207	Hepatitis B	Hepatitis B	1	Day 0			29/09/2010	Yes	Venous system	Normal		
S179	207	Hepatitis B	Hepatitis B	1	Day 0			29/09/2010	Yes	Gastrointestinal	Normal		
S179	207	Hepatitis B	Hepatitis B	1	Day 0			29/09/2010	Yes	Lymphatic	Normal		
S179	207	Hepatitis B	Hepatitis B	1	Day 0			29/09/2010	Yes	Neurological	Normal		
S179	207	Hepatitis B	Hepatitis B	1	Day 0			29/09/2010	Yes	Genitourinary	Normal		
S179	207	Hepatitis B	Hepatitis B	1	Day 0			29/09/2010	Yes	Dermatological	Normal		
S179	207	Hepatitis B	Hepatitis B	1	Day 7			06/10/2010		Head and Neck	Normal		
S179	207	Hepatitis B	Hepatitis B	1	Day 7			06/10/2010		Eyes	Normal		
S179	207	Hepatitis B	Hepatitis B	1	Day 7			06/10/2010		Ears, nose and throat	Normal		
S179	207	Hepatitis B	Hepatitis B	1	Day 7			06/10/2010		Cardiovascular	Normal		
S179	207	Hepatitis B	Hepatitis B	1	Day 7			06/10/2010		Respiratory	Normal		
S179	207	Hepatitis B	Hepatitis B	1	Day 7			06/10/2010		Musculoskeletal	Normal		
S179	207	Hepatitis B	Hepatitis B	1	Day 7			06/10/2010		Venous system	Normal		
S179	207	Hepatitis B	Hepatitis B	1	Day 7			06/10/2010		Gastrointestinal	Normal		
S179	207	Hepatitis B	Hepatitis B	1	Day 7			06/10/2010		Lymphatic	Normal		
S179	207	Hepatitis B	Hepatitis B	1	Day 7			06/10/2010		Neurological	Normal		
S179	207	Hepatitis B	Hepatitis B	1	Day 7			06/10/2010		Genitourinary	Normal		
S179	207	Hepatitis B	Hepatitis B	1	Day 7			06/10/2010		Dermatological	Normal		
S179	207	Hepatitis B	Hepatitis B	1	Day 14			13/10/2010		Head and Neck	Normal		

Subject No.	Randomisation Number	Study Group	Dosage	Dose	Day	Not Done	Specify	Date of Assessment	*Was the assessment performed prior to vaccination?	Physical Examination Parameter	Result	Abnormality	Clinical Significance
S179	207	Hepatitis B	Hepatitis B	1	Day 14			13/10/2010		Eyes	Normal		
S179	207	Hepatitis B	Hepatitis B	1	Day 14			13/10/2010		Ears, nose and throat	Normal		
S179	207	Hepatitis B	Hepatitis B	1	Day 14			13/10/2010		Cardiovascular	Normal		
S179	207	Hepatitis B	Hepatitis B	1	Day 14			13/10/2010		Respiratory	Normal		
S179	207	Hepatitis B	Hepatitis B	1	Day 14			13/10/2010		Musculoskeletal	Normal		
S179	207	Hepatitis B	Hepatitis B	1	Day 14			13/10/2010		Venous system	Normal		
S179	207	Hepatitis B	Hepatitis B	1	Day 14			13/10/2010		Gastrointestinal	Normal		
S179	207	Hepatitis B	Hepatitis B	1	Day 14			13/10/2010		Lymphatic	Normal		
S179	207	Hepatitis B	Hepatitis B	1	Day 14			13/10/2010		Neurological	Normal		
S179	207	Hepatitis B	Hepatitis B	1	Day 14			13/10/2010		Genitourinary	Normal		
S179	207	Hepatitis B	Hepatitis B	1	Day 14			13/10/2010		Dermatological	Normal		
S179	207	Hepatitis B	Hepatitis B	2	Day 28			27/10/2010	Yes	Head and Neck	Normal		
S179	207	Hepatitis B	Hepatitis B	2	Day 28			27/10/2010	Yes	Eyes	Normal		
S179	207	Hepatitis B	Hepatitis B	2	Day 28			27/10/2010	Yes	Ears, nose and throat	Normal		
S179	207	Hepatitis B	Hepatitis B	2	Day 28			27/10/2010	Yes	Cardiovascular	Normal		
S179	207	Hepatitis B	Hepatitis B	2	Day 28			27/10/2010	Yes	Respiratory	Normal		
S179	207	Hepatitis B	Hepatitis B	2	Day 28			27/10/2010	Yes	Musculoskeletal	Normal		
S179	207	Hepatitis B	Hepatitis B	2	Day 28			27/10/2010	Yes	Venous system	Normal		
S179	207	Hepatitis B	Hepatitis B	2	Day 28			27/10/2010	Yes	Gastrointestinal	Normal		

Subject No.	Randomisation Number	Study Group	Dosage	Dose	Day	Not Done	Specify	Date of Assessment	*Was the assessment performed prior to vaccination?	Physical Examination Parameter	Result	Abnormality	Clinical Significance
S179	207	Hepatitis B	Hepatitis B	2	Day 28			27/10/2010	Yes	Lymphatic	Normal		
S179	207	Hepatitis B	Hepatitis B	2	Day 28			27/10/2010	Yes	Neurological	Normal		
S179	207	Hepatitis B	Hepatitis B	2	Day 28			27/10/2010	Yes	Genitourinary	Normal		
S179	207	Hepatitis B	Hepatitis B	2	Day 28			27/10/2010	Yes	Dermatological	Normal		
S179	207	Hepatitis B	Hepatitis B	2	Day 35			03/11/2010		Head and Neck	Normal		
S179	207	Hepatitis B	Hepatitis B	2	Day 35			03/11/2010		Eyes	Normal		
S179	207	Hepatitis B	Hepatitis B	2	Day 35			03/11/2010		Ears, nose and throat	Normal		
S179	207	Hepatitis B	Hepatitis B	2	Day 35			03/11/2010		Cardiovascular	Normal		
S179	207	Hepatitis B	Hepatitis B	2	Day 35			03/11/2010		Respiratory	Normal		
S179	207	Hepatitis B	Hepatitis B	2	Day 35			03/11/2010		Musculoskeletal	Normal		
S179	207	Hepatitis B	Hepatitis B	2	Day 35			03/11/2010		Venous system	Normal		
S179	207	Hepatitis B	Hepatitis B	2	Day 35			03/11/2010		Gastrointestinal	Normal		
S179	207	Hepatitis B	Hepatitis B	2	Day 35			03/11/2010		Lymphatic	Normal		
S179	207	Hepatitis B	Hepatitis B	2	Day 35			03/11/2010		Neurological	Normal		
S179	207	Hepatitis B	Hepatitis B	2	Day 35			03/11/2010		Genitourinary	Normal		
S179	207	Hepatitis B	Hepatitis B	2	Day 35			03/11/2010		Dermatological	Normal		
S179	207	Hepatitis B	Hepatitis B	2	Day 42			10/11/2010		Head and Neck	Normal		
S179	207	Hepatitis B	Hepatitis B	2	Day 42			10/11/2010		Eyes	Normal		
S179	207	Hepatitis B	Hepatitis B	2	Day 42			10/11/2010		Ears, nose and throat	Normal		

Subject No.	Randomisation Number	Study Group	Dosage	Dose	Day	Not Done	Specify	Date of Assessment	*Was the assessment performed prior to vaccination ?	Physical Examination Parameter	Result	Abnormality	Clinical Significance
S179	207	Hepatitis B	Hepatitis B	2	Day 42			10/11/2010		Cardiovascular	Normal		
S179	207	Hepatitis B	Hepatitis B	2	Day 42			10/11/2010		Respiratory	Normal		
S179	207	Hepatitis B	Hepatitis B	2	Day 42			10/11/2010		Musculoskeletal	Normal		
S179	207	Hepatitis B	Hepatitis B	2	Day 42			10/11/2010		Venous system	Normal		
S179	207	Hepatitis B	Hepatitis B	2	Day 42			10/11/2010		Gastrointestinal	Normal		
S179	207	Hepatitis B	Hepatitis B	2	Day 42			10/11/2010		Lymphatic	Normal		
S179	207	Hepatitis B	Hepatitis B	2	Day 42			10/11/2010		Neurological	Normal		
S179	207	Hepatitis B	Hepatitis B	2	Day 42			10/11/2010		Genitourinary	Normal		
S179	207	Hepatitis B	Hepatitis B	2	Day 42			10/11/2010		Dermatological	Normal		
S179	207	Hepatitis B	Hepatitis B	2	Day 56			24/11/2010		Head and Neck	Normal		
S179	207	Hepatitis B	Hepatitis B	2	Day 56			24/11/2010		Eyes	Normal		
S179	207	Hepatitis B	Hepatitis B	2	Day 56			24/11/2010		Ears, nose and throat	Normal		
S179	207	Hepatitis B	Hepatitis B	2	Day 56			24/11/2010		Cardiovascular	Normal		
S179	207	Hepatitis B	Hepatitis B	2	Day 56			24/11/2010		Respiratory	Normal		
S179	207	Hepatitis B	Hepatitis B	2	Day 56			24/11/2010		Musculoskeletal	Normal		
S179	207	Hepatitis B	Hepatitis B	2	Day 56			24/11/2010		Venous system	Normal		
S179	207	Hepatitis B	Hepatitis B	2	Day 56			24/11/2010		Gastrointestinal	Normal		
S179	207	Hepatitis B	Hepatitis B	2	Day 56			24/11/2010		Lymphatic	Normal		
S179	207	Hepatitis B	Hepatitis B	2	Day 56			24/11/2010		Neurological	Normal		

Subject No.	Randomisation Number	Study Group	Dosage	Dose	Day	Not Done	Specify	Date of Assessment	*Was the assessment performed prior to vaccination?	Physical Examination Parameter	Result	Abnormality	Clinical Significance
S179	207	Hepatitis B	Hepatitis B	2	Day 56			24/11/2010		Genitourinary	Normal		
S179	207	Hepatitis B	Hepatitis B	2	Day 56			24/11/2010		Dermatological	Normal		
S179	207	Hepatitis B	Hepatitis B	3	Day 180			28/03/2011	Yes	Head and Neck	Normal		
S179	207	Hepatitis B	Hepatitis B	3	Day 180			28/03/2011	Yes	Eyes	Normal		
S179	207	Hepatitis B	Hepatitis B	3	Day 180			28/03/2011	Yes	Ears, nose and throat	Normal		
S179	207	Hepatitis B	Hepatitis B	3	Day 180			28/03/2011	Yes	Cardiovascular	Normal		
S179	207	Hepatitis B	Hepatitis B	3	Day 180			28/03/2011	Yes	Respiratory	Normal		
S179	207	Hepatitis B	Hepatitis B	3	Day 180			28/03/2011	Yes	Musculoskeletal	Normal		
S179	207	Hepatitis B	Hepatitis B	3	Day 180			28/03/2011	Yes	Venous system	Normal		
S179	207	Hepatitis B	Hepatitis B	3	Day 180			28/03/2011	Yes	Gastrointestinal	Normal		
S179	207	Hepatitis B	Hepatitis B	3	Day 180			28/03/2011	Yes	Lymphatic	Normal		
S179	207	Hepatitis B	Hepatitis B	3	Day 180			28/03/2011	Yes	Neurological	Normal		
S179	207	Hepatitis B	Hepatitis B	3	Day 180			28/03/2011	Yes	Genitourinary	Normal		
S179	207	Hepatitis B	Hepatitis B	3	Day 180			28/03/2011	Yes	Dermatological	Normal		
S179	207	Hepatitis B	Hepatitis B	3	Day 187			06/04/2011		Head and Neck	Normal		
S179	207	Hepatitis B	Hepatitis B	3	Day 187			06/04/2011		Eyes	Normal		
S179	207	Hepatitis B	Hepatitis B	3	Day 187			06/04/2011		Ears, nose and throat	Normal		
S179	207	Hepatitis B	Hepatitis B	3	Day 187			06/04/2011		Cardiovascular	Normal		
S179	207	Hepatitis B	Hepatitis B	3	Day 187			06/04/2011		Respiratory	Normal		

Subject No.	Randomisation Number	Study Group	Dosage	Dose	Day	Not Done	Specify	Date of Assessment	*Was the assessment performed prior to vaccination?	Physical Examination Parameter	Result	Abnormality	Clinical Significance
S179	207	Hepatitis B	Hepatitis B	3	Day 187			06/04/2011		Musculoskeletal	Normal		
S179	207	Hepatitis B	Hepatitis B	3	Day 187			06/04/2011		Venous system	Normal		
S179	207	Hepatitis B	Hepatitis B	3	Day 187			06/04/2011		Gastrointestinal	Normal		
S179	207	Hepatitis B	Hepatitis B	3	Day 187			06/04/2011		Lymphatic	Normal		
S179	207	Hepatitis B	Hepatitis B	3	Day 187			06/04/2011		Neurological	Normal		
S179	207	Hepatitis B	Hepatitis B	3	Day 187			06/04/2011		Genitourinary	Normal		
S179	207	Hepatitis B	Hepatitis B	3	Day 187			06/04/2011		Dermatological	Normal		
S179	207	Hepatitis B	Hepatitis B	3	Day 194			11/04/2011		Head and Neck	Normal		
S179	207	Hepatitis B	Hepatitis B	3	Day 194			11/04/2011		Eyes	Normal		
S179	207	Hepatitis B	Hepatitis B	3	Day 194			11/04/2011		Ears, nose and throat	Normal		
S179	207	Hepatitis B	Hepatitis B	3	Day 194			11/04/2011		Cardiovascular	Normal		
S179	207	Hepatitis B	Hepatitis B	3	Day 194			11/04/2011		Respiratory	Normal		
S179	207	Hepatitis B	Hepatitis B	3	Day 194			11/04/2011		Musculoskeletal	Normal		
S179	207	Hepatitis B	Hepatitis B	3	Day 194			11/04/2011		Venous system	Normal		
S179	207	Hepatitis B	Hepatitis B	3	Day 194			11/04/2011		Gastrointestinal	Normal		
S179	207	Hepatitis B	Hepatitis B	3	Day 194			11/04/2011		Lymphatic	Normal		
S179	207	Hepatitis B	Hepatitis B	3	Day 194			11/04/2011		Neurological	Normal		
S179	207	Hepatitis B	Hepatitis B	3	Day 194			11/04/2011		Genitourinary	Normal		
S179	207	Hepatitis B	Hepatitis B	3	Day 194			11/04/2011		Dermatological	Normal		

Subject No.	Randomisation Number	Study Group	Dosage	Dose	Day	Not Done	Specify	Date of Assessment	*Was the assessment performed prior to vaccination?	Physical Examination Parameter	Result	Abnormality	Clinical Significance
S179	207	Hepatitis B	Hepatitis B	3	Day 208			25/04/2011		Head and Neck	Normal		
S179	207	Hepatitis B	Hepatitis B	3	Day 208			25/04/2011		Eyes	Normal		
S179	207	Hepatitis B	Hepatitis B	3	Day 208			25/04/2011		Ears, nose and throat	Normal		
S179	207	Hepatitis B	Hepatitis B	3	Day 208			25/04/2011		Cardiovascular	Normal		
S179	207	Hepatitis B	Hepatitis B	3	Day 208			25/04/2011		Respiratory	Normal		
S179	207	Hepatitis B	Hepatitis B	3	Day 208			25/04/2011		Musculoskeletal	Normal		
S179	207	Hepatitis B	Hepatitis B	3	Day 208			25/04/2011		Venous system	Normal		
S179	207	Hepatitis B	Hepatitis B	3	Day 208			25/04/2011		Gastrointestinal	Normal		
S179	207	Hepatitis B	Hepatitis B	3	Day 208			25/04/2011		Lymphatic	Normal		
S179	207	Hepatitis B	Hepatitis B	3	Day 208			25/04/2011		Neurological	Normal		
S179	207	Hepatitis B	Hepatitis B	3	Day 208			25/04/2011		Genitourinary	Normal		
S179	207	Hepatitis B	Hepatitis B	3	Day 208			25/04/2011		Dermatological	Normal		
S179	207	Hepatitis B	Hepatitis B	3	Day 365			28/09/2011		Head and Neck	Normal		
S179	207	Hepatitis B	Hepatitis B	3	Day 365			28/09/2011		Eyes	Normal		
S179	207	Hepatitis B	Hepatitis B	3	Day 365			28/09/2011		Ears, nose and throat	Normal		
S179	207	Hepatitis B	Hepatitis B	3	Day 365			28/09/2011		Cardiovascular	Normal		
S179	207	Hepatitis B	Hepatitis B	3	Day 365			28/09/2011		Respiratory	Normal		
S179	207	Hepatitis B	Hepatitis B	3	Day 365			28/09/2011		Musculoskeletal	Normal		
S179	207	Hepatitis B	Hepatitis B	3	Day 365			28/09/2011		Venous system	Normal		

Subject No.	Randomisation Number	Study Group	Dosage	Dose	Day	Not Done	Specify	Date of Assessment	*Was the assessment performed prior to vaccination?	Physical Examination Parameter	Result	Abnormality	Clinical Significance
S179	207	Hepatitis B	Hepatitis B	3	Day 365			28/09/2011		Gastrointestinal	Normal		
S179	207	Hepatitis B	Hepatitis B	3	Day 365			28/09/2011		Lymphatic	Normal		
S179	207	Hepatitis B	Hepatitis B	3	Day 365			28/09/2011		Neurological	Normal		
S179	207	Hepatitis B	Hepatitis B	3	Day 365			28/09/2011		Genitourinary	Normal		
S179	207	Hepatitis B	Hepatitis B	3	Day 365			28/09/2011		Dermatological	Normal		
S182	208	JAIVAC-1	Dosage B		Day -14 to Day -1			21/09/2010		Head and neck	Normal		
S182	208	JAIVAC-1	Dosage B		Day -14 to Day -1			21/09/2010		Eyes	Normal		
S182	208	JAIVAC-1	Dosage B		Day -14 to Day -1			21/09/2010		Ears, nose and throat	Normal		
S182	208	JAIVAC-1	Dosage B		Day -14 to Day -1			21/09/2010		Cardiovascular	Normal		
S182	208	JAIVAC-1	Dosage B		Day -14 to Day -1			21/09/2010		Respiratory	Normal		
S182	208	JAIVAC-1	Dosage B		Day -14 to Day -1			21/09/2010		Musculoskeletal	Normal		
S182	208	JAIVAC-1	Dosage B		Day -14 to Day -1			21/09/2010		Venous system	Normal		
S182	208	JAIVAC-1	Dosage B		Day -14 to Day -1			21/09/2010		Gastrointestinal	Normal		
S182	208	JAIVAC-1	Dosage B		Day -14 to Day -1			21/09/2010		Lymphatic	Normal		
S182	208	JAIVAC-1	Dosage B		Day -14 to Day -1			21/09/2010		Neurological	Normal		
S182	208	JAIVAC-1	Dosage B		Day -14 to Day -1			21/09/2010		Genitourinary	Normal		
S182	208	JAIVAC-1	Dosage B		Day -14 to Day -1			21/09/2010		Dermatological	Normal		

Subject No.	Randomisation Number	Study Group	Dosage	Dose	Day	Not Done	Specify	Date of Assessment	*Was the assessment performed prior to vaccination?	Physical Examination Parameter	Result	Abnormality	Clinical Significance
S182	208	JAIVAC-1	Dosage B	1	Day 0			29/09/2010	Yes	Head and neck	Normal		
S182	208	JAIVAC-1	Dosage B	1	Day 0			29/09/2010	Yes	Eyes	Normal		
S182	208	JAIVAC-1	Dosage B	1	Day 0			29/09/2010	Yes	Ears, nose and throat	Normal		
S182	208	JAIVAC-1	Dosage B	1	Day 0			29/09/2010	Yes	Cardiovascular	Normal		
S182	208	JAIVAC-1	Dosage B	1	Day 0			29/09/2010	Yes	Respiratory	Normal		
S182	208	JAIVAC-1	Dosage B	1	Day 0			29/09/2010	Yes	Musculoskeletal	Normal		
S182	208	JAIVAC-1	Dosage B	1	Day 0			29/09/2010	Yes	Venous system	Normal		
S182	208	JAIVAC-1	Dosage B	1	Day 0			29/09/2010	Yes	Gastrointestinal	Normal		
S182	208	JAIVAC-1	Dosage B	1	Day 0			29/09/2010	Yes	Lymphatic	Normal		
S182	208	JAIVAC-1	Dosage B	1	Day 0			29/09/2010	Yes	Neurological	Normal		
S182	208	JAIVAC-1	Dosage B	1	Day 0			29/09/2010	Yes	Genitourinary	Normal		
S182	208	JAIVAC-1	Dosage B	1	Day 0			29/09/2010	Yes	Dermatological	Normal		
S182	208	JAIVAC-1	Dosage B	1	Day 7			06/10/2010		Head and Neck	Normal		
S182	208	JAIVAC-1	Dosage B	1	Day 7			06/10/2010		Eyes	Normal		
S182	208	JAIVAC-1	Dosage B	1	Day 7			06/10/2010		Ears, nose and throat	Normal		
S182	208	JAIVAC-1	Dosage B	1	Day 7			06/10/2010		Cardiovascular	Normal		
S182	208	JAIVAC-1	Dosage B	1	Day 7			06/10/2010		Respiratory	Normal		
S182	208	JAIVAC-1	Dosage B	1	Day 7			06/10/2010		Musculoskeletal	Normal		
S182	208	JAIVAC-1	Dosage B	1	Day 7			06/10/2010		Venous system	Normal		

Subject No.	Randomisation Number	Study Group	Dosage	Dose	Day	Not Done	Specify	Date of Assessment	*Was the assessment performed prior to vaccination?	Physical Examination Parameter	Result	Abnormality	Clinical Significance
S182	208	JAIVAC-1	Dosage B	1	Day 7			06/10/2010		Gastrointestinal	Normal		
S182	208	JAIVAC-1	Dosage B	1	Day 7			06/10/2010		Lymphatic	Normal		
S182	208	JAIVAC-1	Dosage B	1	Day 7			06/10/2010		Neurological	Normal		
S182	208	JAIVAC-1	Dosage B	1	Day 7			06/10/2010		Genitourinary	Normal		
S182	208	JAIVAC-1	Dosage B	1	Day 7			06/10/2010		Dermatological	Normal		
S182	208	JAIVAC-1	Dosage B	1	Day 14			13/10/2010		Head and Neck	Normal		
S182	208	JAIVAC-1	Dosage B	1	Day 14			13/10/2010		Eyes	Normal		
S182	208	JAIVAC-1	Dosage B	1	Day 14			13/10/2010		Ears, nose and throat	Normal		
S182	208	JAIVAC-1	Dosage B	1	Day 14			13/10/2010		Cardiovascular	Normal		
S182	208	JAIVAC-1	Dosage B	1	Day 14			13/10/2010		Respiratory	Normal		
S182	208	JAIVAC-1	Dosage B	1	Day 14			13/10/2010		Musculoskeletal	Normal		
S182	208	JAIVAC-1	Dosage B	1	Day 14			13/10/2010		Venous system	Normal		
S182	208	JAIVAC-1	Dosage B	1	Day 14			13/10/2010		Gastrointestinal	Normal		
S182	208	JAIVAC-1	Dosage B	1	Day 14			13/10/2010		Lymphatic	Normal		
S182	208	JAIVAC-1	Dosage B	1	Day 14			13/10/2010		Neurological	Normal		
S182	208	JAIVAC-1	Dosage B	1	Day 14			13/10/2010		Genitourinary	Normal		
S182	208	JAIVAC-1	Dosage B	1	Day 14			13/10/2010		Dermatological	Normal		
S182	208	JAIVAC-1	Dosage B	2	Day 28			27/10/2010	Yes	Head and Neck	Normal		
S182	208	JAIVAC-1	Dosage B	2	Day 28			27/10/2010	Yes	Eyes	Normal		

Subject No.	Randomisation Number	Study Group	Dosage	Dose	Day	Not Done	Specify	Date of Assessment	*Was the assessment performed prior to vaccination?	Physical Examination Parameter	Result	Abnormality	Clinical Significance
S182	208	JAIVAC-1	Dosage B	2	Day 28			27/10/2010	Yes	Ears, nose and throat	Normal		
S182	208	JAIVAC-1	Dosage B	2	Day 28			27/10/2010	Yes	Cardiovascular	Normal		
S182	208	JAIVAC-1	Dosage B	2	Day 28			27/10/2010	Yes	Respiratory	Normal		
S182	208	JAIVAC-1	Dosage B	2	Day 28			27/10/2010	Yes	Musculoskeletal	Normal		
S182	208	JAIVAC-1	Dosage B	2	Day 28			27/10/2010	Yes	Venous system	Normal		
S182	208	JAIVAC-1	Dosage B	2	Day 28			27/10/2010	Yes	Gastrointestinal	Normal		
S182	208	JAIVAC-1	Dosage B	2	Day 28			27/10/2010	Yes	Lymphatic	Normal		
S182	208	JAIVAC-1	Dosage B	2	Day 28			27/10/2010	Yes	Neurological	Normal		
S182	208	JAIVAC-1	Dosage B	2	Day 28			27/10/2010	Yes	Genitourinary	Normal		
S182	208	JAIVAC-1	Dosage B	2	Day 28			27/10/2010	Yes	Dermatological	Normal		
S182	208	JAIVAC-1	Dosage B	2	Day 35			03/11/2010		Head and Neck	Normal		
S182	208	JAIVAC-1	Dosage B	2	Day 35			03/11/2010		Eyes	Abnormal	Reddish Discoloration And Watering Of{l} Eye	CS
S182	208	JAIVAC-1	Dosage B	2	Day 35			03/11/2010		Ears, nose and throat	Normal		
S182	208	JAIVAC-1	Dosage B	2	Day 35			03/11/2010		Cardiovascular	Normal		
S182	208	JAIVAC-1	Dosage B	2	Day 35			03/11/2010		Respiratory	Normal		
S182	208	JAIVAC-1	Dosage B	2	Day 35			03/11/2010		Musculoskeletal	Normal		
S182	208	JAIVAC-1	Dosage B	2	Day 35			03/11/2010		Venous system	Normal		

Subject No.	Randomisation Number	Study Group	Dosage	Dose	Day	Not Done	Specify	Date of Assessment	*Was the assessment performed prior to vaccination?	Physical Examination Parameter	Result	Abnormality	Clinical Significance
S182	208	JAIVAC-1	Dosage B	2	Day 35			03/11/2010		Gastrointestinal	Normal		
S182	208	JAIVAC-1	Dosage B	2	Day 35			03/11/2010		Lymphatic	Normal		
S182	208	JAIVAC-1	Dosage B	2	Day 35			03/11/2010		Neurological	Normal		
S182	208	JAIVAC-1	Dosage B	2	Day 35			03/11/2010		Genitourinary	Normal		
S182	208	JAIVAC-1	Dosage B	2	Day 35			03/11/2010		Dermatological	Normal		
S182	208	JAIVAC-1	Dosage B	2	Day 42			10/11/2010		Head and Neck	Normal		
S182	208	JAIVAC-1	Dosage B	2	Day 42			10/11/2010		Eyes	Normal		
S182	208	JAIVAC-1	Dosage B	2	Day 42			10/11/2010		Ears, nose and throat	Normal		
S182	208	JAIVAC-1	Dosage B	2	Day 42			10/11/2010		Cardiovascular	Normal		
S182	208	JAIVAC-1	Dosage B	2	Day 42			10/11/2010		Respiratory	Normal		
S182	208	JAIVAC-1	Dosage B	2	Day 42			10/11/2010		Musculoskeletal	Normal		
S182	208	JAIVAC-1	Dosage B	2	Day 42			10/11/2010		Venous system	Normal		
S182	208	JAIVAC-1	Dosage B	2	Day 42			10/11/2010		Gastrointestinal	Normal		
S182	208	JAIVAC-1	Dosage B	2	Day 42			10/11/2010		Lymphatic	Normal		
S182	208	JAIVAC-1	Dosage B	2	Day 42			10/11/2010		Neurological	Normal		
S182	208	JAIVAC-1	Dosage B	2	Day 42			10/11/2010		Genitourinary	Normal		
S182	208	JAIVAC-1	Dosage B	2	Day 42			10/11/2010		Dermatological	Normal		
S182	208	JAIVAC-1	Dosage B	2	Day 56			24/11/2010		Head and Neck	Normal		
S182	208	JAIVAC-1	Dosage B	2	Day 56			24/11/2010		Eyes	Normal		

Subject No.	Randomisation Number	Study Group	Dosage	Dose	Day	Not Done	Specify	Date of Assessment	*Was the assessment performed prior to vaccination?	Physical Examination Parameter	Result	Abnormality	Clinical Significance
S182	208	JAIVAC-1	Dosage B	2	Day 56			24/11/2010		Ears, nose and throat	Normal		
S182	208	JAIVAC-1	Dosage B	2	Day 56			24/11/2010		Cardiovascular	Normal		
S182	208	JAIVAC-1	Dosage B	2	Day 56			24/11/2010		Respiratory	Normal		
S182	208	JAIVAC-1	Dosage B	2	Day 56			24/11/2010		Musculoskeletal	Normal		
S182	208	JAIVAC-1	Dosage B	2	Day 56			24/11/2010		Venous system	Normal		
S182	208	JAIVAC-1	Dosage B	2	Day 56			24/11/2010		Gastrointestinal	Normal		
S182	208	JAIVAC-1	Dosage B	2	Day 56			24/11/2010		Lymphatic	Normal		
S182	208	JAIVAC-1	Dosage B	2	Day 56			24/11/2010		Neurological	Normal		
S182	208	JAIVAC-1	Dosage B	2	Day 56			24/11/2010		Genitourinary	Normal		
S182	208	JAIVAC-1	Dosage B	2	Day 56			24/11/2010		Dermatological	Normal		
S182	208	JAIVAC-1	Dosage B	3	Day 180			28/03/2011	Yes	Head and Neck	Normal		
S182	208	JAIVAC-1	Dosage B	3	Day 180			28/03/2011	Yes	Eyes	Normal		
S182	208	JAIVAC-1	Dosage B	3	Day 180			28/03/2011	Yes	Ears, nose and throat	Normal		
S182	208	JAIVAC-1	Dosage B	3	Day 180			28/03/2011	Yes	Cardiovascular	Normal		
S182	208	JAIVAC-1	Dosage B	3	Day 180			28/03/2011	Yes	Respiratory	Normal		
S182	208	JAIVAC-1	Dosage B	3	Day 180			28/03/2011	Yes	Musculoskeletal	Normal		
S182	208	JAIVAC-1	Dosage B	3	Day 180			28/03/2011	Yes	Venous system	Normal		
S182	208	JAIVAC-1	Dosage B	3	Day 180			28/03/2011	Yes	Gastrointestinal	Normal		
S182	208	JAIVAC-1	Dosage B	3	Day 180			28/03/2011	Yes	Lymphatic	Normal		

Subject No.	Randomisation Number	Study Group	Dosage	Dose	Day	Not Done	Specify	Date of Assessment	*Was the assessment performed prior to vaccination?	Physical Examination Parameter	Result	Abnormality	Clinical Significance
S182	208	JAIVAC-1	Dosage B	3	Day 180			28/03/2011	Yes	Neurological	Normal		
S182	208	JAIVAC-1	Dosage B	3	Day 180			28/03/2011	Yes	Genitourinary	Normal		
S182	208	JAIVAC-1	Dosage B	3	Day 180			28/03/2011	Yes	Dermatological	Normal		
S182	208	JAIVAC-1	Dosage B	3	Day 187			05/04/2011		Head and Neck	Normal		
S182	208	JAIVAC-1	Dosage B	3	Day 187			05/04/2011		Eyes	Normal		
S182	208	JAIVAC-1	Dosage B	3	Day 187			05/04/2011		Ears, nose and throat	Normal		
S182	208	JAIVAC-1	Dosage B	3	Day 187			05/04/2011		Cardiovascular	Normal		
S182	208	JAIVAC-1	Dosage B	3	Day 187			05/04/2011		Respiratory	Normal		
S182	208	JAIVAC-1	Dosage B	3	Day 187			05/04/2011		Musculoskeletal	Normal		
S182	208	JAIVAC-1	Dosage B	3	Day 187			05/04/2011		Venous system	Normal		
S182	208	JAIVAC-1	Dosage B	3	Day 187			05/04/2011		Gastrointestinal	Normal		
S182	208	JAIVAC-1	Dosage B	3	Day 187			05/04/2011		Lymphatic	Normal		
S182	208	JAIVAC-1	Dosage B	3	Day 187			05/04/2011		Neurological	Normal		
S182	208	JAIVAC-1	Dosage B	3	Day 187			05/04/2011		Genitourinary	Normal		
S182	208	JAIVAC-1	Dosage B	3	Day 187			05/04/2011		Dermatological	Normal		
S182	208	JAIVAC-1	Dosage B	3	Day 194			11/04/2011		Head and Neck	Normal		
S182	208	JAIVAC-1	Dosage B	3	Day 194			11/04/2011		Eyes	Normal		
S182	208	JAIVAC-1	Dosage B	3	Day 194			11/04/2011		Ears, nose and throat	Normal		
S182	208	JAIVAC-1	Dosage B	3	Day 194			11/04/2011		Cardiovascular	Normal		

Subject No.	Randomisation Number	Study Group	Dosage	Dose	Day	Not Done	Specify	Date of Assessment	*Was the assessment performed prior to vaccination?	Physical Examination Parameter	Result	Abnormality	Clinical Significance
S182	208	JAIVAC-1	Dosage B	3	Day 194			11/04/2011		Respiratory	Normal		
S182	208	JAIVAC-1	Dosage B	3	Day 194			11/04/2011		Musculoskeletal	Normal		
S182	208	JAIVAC-1	Dosage B	3	Day 194			11/04/2011		Venous system	Normal		
S182	208	JAIVAC-1	Dosage B	3	Day 194			11/04/2011		Gastrointestinal	Normal		
S182	208	JAIVAC-1	Dosage B	3	Day 194			11/04/2011		Lymphatic	Normal		
S182	208	JAIVAC-1	Dosage B	3	Day 194			11/04/2011		Neurological	Normal		
S182	208	JAIVAC-1	Dosage B	3	Day 194			11/04/2011		Genitourinary	Normal		
S182	208	JAIVAC-1	Dosage B	3	Day 194			11/04/2011		Dermatological	Normal		
S182	208	JAIVAC-1	Dosage B	3	Day 208			25/04/2011		Head and Neck	Normal		
S182	208	JAIVAC-1	Dosage B	3	Day 208			25/04/2011		Eyes	Normal		
S182	208	JAIVAC-1	Dosage B	3	Day 208			25/04/2011		Ears, nose and throat	Normal		
S182	208	JAIVAC-1	Dosage B	3	Day 208			25/04/2011		Cardiovascular	Normal		
S182	208	JAIVAC-1	Dosage B	3	Day 208			25/04/2011		Respiratory	Normal		
S182	208	JAIVAC-1	Dosage B	3	Day 208			25/04/2011		Musculoskeletal	Normal		
S182	208	JAIVAC-1	Dosage B	3	Day 208			25/04/2011		Venous system	Normal		
S182	208	JAIVAC-1	Dosage B	3	Day 208			25/04/2011		Gastrointestinal	Normal		
S182	208	JAIVAC-1	Dosage B	3	Day 208			25/04/2011		Lymphatic	Normal		
S182	208	JAIVAC-1	Dosage B	3	Day 208			25/04/2011		Neurological	Normal		
S182	208	JAIVAC-1	Dosage B	3	Day 208			25/04/2011		Genitourinary	Normal		

Subject No.	Randomisation Number	Study Group	Dosage	Dose	Day	Not Done	Specify	Date of Assessment	*Was the assessment performed prior to vaccination?	Physical Examination Parameter	Result	Abnormality	Clinical Significance
S182	208	JAIVAC-1	Dosage B	3	Day 208			25/04/2011		Dermatological	Normal		
S182	208	JAIVAC-1	Dosage B	3	Day 365			29/09/2011		Head and Neck	Normal		
S182	208	JAIVAC-1	Dosage B	3	Day 365			29/09/2011		Eyes	Normal		
S182	208	JAIVAC-1	Dosage B	3	Day 365			29/09/2011		Ears, nose and throat	Normal		
S182	208	JAIVAC-1	Dosage B	3	Day 365			29/09/2011		Cardiovascular	Normal		
S182	208	JAIVAC-1	Dosage B	3	Day 365			29/09/2011		Respiratory	Normal		
S182	208	JAIVAC-1	Dosage B	3	Day 365			29/09/2011		Musculoskeletal	Normal		
S182	208	JAIVAC-1	Dosage B	3	Day 365			29/09/2011		Venous system	Normal		
S182	208	JAIVAC-1	Dosage B	3	Day 365			29/09/2011		Gastrointestinal	Normal		
S182	208	JAIVAC-1	Dosage B	3	Day 365			29/09/2011		Lymphatic	Normal		
S182	208	JAIVAC-1	Dosage B	3	Day 365			29/09/2011		Neurological	Normal		
S182	208	JAIVAC-1	Dosage B	3	Day 365			29/09/2011		Genitourinary	Normal		
S182	208	JAIVAC-1	Dosage B	3	Day 365			29/09/2011		Dermatological	Normal		
S182	208	JAIVAC-1	Dosage B		Unscheduled Visit	Yes	Laboratory Repeat At Local Lab	.		Head and Neck			
S182	208	JAIVAC-1	Dosage B		Unscheduled Visit	Yes	Laboratory Repeat At Local Lab	.		Eyes			
S182	208	JAIVAC-1	Dosage B		Unscheduled Visit	Yes	Laboratory Repeat At Local Lab	.		Ears, nose and throat			

Subject No.	Randomisation Number	Study Group	Dosage	Dose	Day	Not Done	Specify	Date of Assessment	*Was the assessment performed prior to vaccination?	Physical Examination Parameter	Result	Abnormality	Clinical Significance
S182	208	JAIVAC-1	Dosage B		Unscheduled Visit	Yes	Laboratory Repeat At Local Lab	.		Cardiovascular			
S182	208	JAIVAC-1	Dosage B		Unscheduled Visit	Yes	Laboratory Repeat At Local Lab	.		Respiratory			
S182	208	JAIVAC-1	Dosage B		Unscheduled Visit	Yes	Laboratory Repeat At Local Lab	.		Musculoskeletal			
S182	208	JAIVAC-1	Dosage B		Unscheduled Visit	Yes	Laboratory Repeat At Local Lab	.		Venous system			
S182	208	JAIVAC-1	Dosage B		Unscheduled Visit	Yes	Laboratory Repeat At Local Lab	.		Gastrointestinal			
S182	208	JAIVAC-1	Dosage B		Unscheduled Visit	Yes	Laboratory Repeat At Local Lab	.		Lymphatic			
S182	208	JAIVAC-1	Dosage B		Unscheduled Visit	Yes	Laboratory Repeat At Local Lab	.		Neurological			
S182	208	JAIVAC-1	Dosage B		Unscheduled Visit	Yes	Laboratory Repeat At Local Lab	.		Genitourinary			
S182	208	JAIVAC-1	Dosage B		Unscheduled Visit	Yes	Laboratory Repeat At Local Lab	.		Dermatological			
S185	202	JAIVAC-1	Dosage B		Day -14 to Day -1			21/09/2010		Head and neck	Normal		
S185	202	JAIVAC-1	Dosage B		Day -14 to Day -1			21/09/2010		Eyes	Normal		
S185	202	JAIVAC-1	Dosage B		Day -14 to Day -1			21/09/2010		Ears, nose and throat	Normal		
S185	202	JAIVAC-1	Dosage B		Day -14 to Day -1			21/09/2010		Cardiovascular	Normal		

Subject No.	Randomisation Number	Study Group	Dosage	Dose	Day	Not Done	Specify	Date of Assessment	*Was the assessment performed prior to vaccination?	Physical Examination Parameter	Result	Abnormality	Clinical Significance
S185	202	JAIVAC-1	Dosage B		Day -14 to Day -1			21/09/2010		Respiratory	Normal		
S185	202	JAIVAC-1	Dosage B		Day -14 to Day -1			21/09/2010		Musculoskeletal	Normal		
S185	202	JAIVAC-1	Dosage B		Day -14 to Day -1			21/09/2010		Venous system	Normal		
S185	202	JAIVAC-1	Dosage B		Day -14 to Day -1			21/09/2010		Gastrointestinal	Normal		
S185	202	JAIVAC-1	Dosage B		Day -14 to Day -1			21/09/2010		Lymphatic	Normal		
S185	202	JAIVAC-1	Dosage B		Day -14 to Day -1			21/09/2010		Neurological	Normal		
S185	202	JAIVAC-1	Dosage B		Day -14 to Day -1			21/09/2010		Genitourinary	Normal		
S185	202	JAIVAC-1	Dosage B		Day -14 to Day -1			21/09/2010		Dermatological	Normal		
S185	202	JAIVAC-1	Dosage B	1	Day 0			28/09/2010	Yes	Head and neck	Normal		
S185	202	JAIVAC-1	Dosage B	1	Day 0			28/09/2010	Yes	Eyes	Normal		
S185	202	JAIVAC-1	Dosage B	1	Day 0			28/09/2010	Yes	Ears, nose and throat	Normal		
S185	202	JAIVAC-1	Dosage B	1	Day 0			28/09/2010	Yes	Cardiovascular	Normal		
S185	202	JAIVAC-1	Dosage B	1	Day 0			28/09/2010	Yes	Respiratory	Normal		
S185	202	JAIVAC-1	Dosage B	1	Day 0			28/09/2010	Yes	Musculoskeletal	Normal		
S185	202	JAIVAC-1	Dosage B	1	Day 0			28/09/2010	Yes	Venous system	Normal		
S185	202	JAIVAC-1	Dosage B	1	Day 0			28/09/2010	Yes	Gastrointestinal	Normal		
S185	202	JAIVAC-1	Dosage B	1	Day 0			28/09/2010	Yes	Lymphatic	Normal		
S185	202	JAIVAC-1	Dosage B	1	Day 0			28/09/2010	Yes	Neurological	Normal		

Subject No.	Randomisation Number	Study Group	Dosage	Dose	Day	Not Done	Specify	Date of Assessment	*Was the assessment performed prior to vaccination?	Physical Examination Parameter	Result	Abnormality	Clinical Significance
S185	202	JAIVAC-1	Dosage B	1	Day 0			28/09/2010	Yes	Genitourinary	Normal		
S185	202	JAIVAC-1	Dosage B	1	Day 0			28/09/2010	Yes	Dermatological	Normal		
S185	202	JAIVAC-1	Dosage B	1	Day 7			05/10/2010		Head and Neck	Normal		
S185	202	JAIVAC-1	Dosage B	1	Day 7			05/10/2010		Eyes	Normal		
S185	202	JAIVAC-1	Dosage B	1	Day 7			05/10/2010		Ears, nose and throat	Normal		
S185	202	JAIVAC-1	Dosage B	1	Day 7			05/10/2010		Cardiovascular	Normal		
S185	202	JAIVAC-1	Dosage B	1	Day 7			05/10/2010		Respiratory	Normal		
S185	202	JAIVAC-1	Dosage B	1	Day 7			05/10/2010		Musculoskeletal	Normal		
S185	202	JAIVAC-1	Dosage B	1	Day 7			05/10/2010		Venous system	Normal		
S185	202	JAIVAC-1	Dosage B	1	Day 7			05/10/2010		Gastrointestinal	Normal		
S185	202	JAIVAC-1	Dosage B	1	Day 7			05/10/2010		Lymphatic	Normal		
S185	202	JAIVAC-1	Dosage B	1	Day 7			05/10/2010		Neurological	Normal		
S185	202	JAIVAC-1	Dosage B	1	Day 7			05/10/2010		Genitourinary	Normal		
S185	202	JAIVAC-1	Dosage B	1	Day 7			05/10/2010		Dermatological	Normal		
S185	202	JAIVAC-1	Dosage B	1	Day 14			13/10/2010		Head and Neck	Normal		
S185	202	JAIVAC-1	Dosage B	1	Day 14			13/10/2010		Eyes	Normal		
S185	202	JAIVAC-1	Dosage B	1	Day 14			13/10/2010		Ears, nose and throat	Normal		
S185	202	JAIVAC-1	Dosage B	1	Day 14			13/10/2010		Cardiovascular	Normal		
S185	202	JAIVAC-1	Dosage B	1	Day 14			13/10/2010		Respiratory	Normal		

Subject No.	Randomisation Number	Study Group	Dosage	Dose	Day	Not Done	Specify	Date of Assessment	*Was the assessment performed prior to vaccination?	Physical Examination Parameter	Result	Abnormality	Clinical Significance
S185	202	JAIVAC-1	Dosage B	1	Day 14			13/10/2010		Musculoskeletal	Normal		
S185	202	JAIVAC-1	Dosage B	1	Day 14			13/10/2010		Venous system	Normal		
S185	202	JAIVAC-1	Dosage B	1	Day 14			13/10/2010		Gastrointestinal	Normal		
S185	202	JAIVAC-1	Dosage B	1	Day 14			13/10/2010		Lymphatic	Normal		
S185	202	JAIVAC-1	Dosage B	1	Day 14			13/10/2010		Neurological	Normal		
S185	202	JAIVAC-1	Dosage B	1	Day 14			13/10/2010		Genitourinary	Normal		
S185	202	JAIVAC-1	Dosage B	1	Day 14			13/10/2010		Dermatological	Normal		
S185	202	JAIVAC-1	Dosage B	2	Day 28			26/10/2010	Yes	Head and Neck	Normal		
S185	202	JAIVAC-1	Dosage B	2	Day 28			26/10/2010	Yes	Eyes	Normal		
S185	202	JAIVAC-1	Dosage B	2	Day 28			26/10/2010	Yes	Ears, nose and throat	Normal		
S185	202	JAIVAC-1	Dosage B	2	Day 28			26/10/2010	Yes	Cardiovascular	Normal		
S185	202	JAIVAC-1	Dosage B	2	Day 28			26/10/2010	Yes	Respiratory	Normal		
S185	202	JAIVAC-1	Dosage B	2	Day 28			26/10/2010	Yes	Musculoskeletal	Normal		
S185	202	JAIVAC-1	Dosage B	2	Day 28			26/10/2010	Yes	Venous system	Normal		
S185	202	JAIVAC-1	Dosage B	2	Day 28			26/10/2010	Yes	Gastrointestinal	Normal		
S185	202	JAIVAC-1	Dosage B	2	Day 28			26/10/2010	Yes	Lymphatic	Normal		
S185	202	JAIVAC-1	Dosage B	2	Day 28			26/10/2010	Yes	Neurological	Normal		
S185	202	JAIVAC-1	Dosage B	2	Day 28			26/10/2010	Yes	Genitourinary	Normal		
S185	202	JAIVAC-1	Dosage B	2	Day 28			26/10/2010	Yes	Dermatological	Normal		

Subject No.	Randomisation Number	Study Group	Dosage	Dose	Day	Not Done	Specify	Date of Assessment	*Was the assessment performed prior to vaccination?	Physical Examination Parameter	Result	Abnormality	Clinical Significance
S185	202	JAIVAC-1	Dosage B	2	Day 35			02/11/2010		Head and Neck	Normal		
S185	202	JAIVAC-1	Dosage B	2	Day 35			02/11/2010		Eyes	Normal		
S185	202	JAIVAC-1	Dosage B	2	Day 35			02/11/2010		Ears, nose and throat	Normal		
S185	202	JAIVAC-1	Dosage B	2	Day 35			02/11/2010		Cardiovascular	Normal		
S185	202	JAIVAC-1	Dosage B	2	Day 35			02/11/2010		Respiratory	Normal		
S185	202	JAIVAC-1	Dosage B	2	Day 35			02/11/2010		Musculoskeletal	Normal		
S185	202	JAIVAC-1	Dosage B	2	Day 35			02/11/2010		Venous system	Normal		
S185	202	JAIVAC-1	Dosage B	2	Day 35			02/11/2010		Gastrointestinal	Normal		
S185	202	JAIVAC-1	Dosage B	2	Day 35			02/11/2010		Lymphatic	Normal		
S185	202	JAIVAC-1	Dosage B	2	Day 35			02/11/2010		Neurological	Normal		
S185	202	JAIVAC-1	Dosage B	2	Day 35			02/11/2010		Genitourinary	Normal		
S185	202	JAIVAC-1	Dosage B	2	Day 35			02/11/2010		Dermatological	Normal		
S185	202	JAIVAC-1	Dosage B	2	Day 42			10/11/2010		Head and Neck	Normal		
S185	202	JAIVAC-1	Dosage B	2	Day 42			10/11/2010		Eyes	Normal		
S185	202	JAIVAC-1	Dosage B	2	Day 42			10/11/2010		Ears, nose and throat	Normal		
S185	202	JAIVAC-1	Dosage B	2	Day 42			10/11/2010		Cardiovascular	Normal		
S185	202	JAIVAC-1	Dosage B	2	Day 42			10/11/2010		Respiratory	Normal		
S185	202	JAIVAC-1	Dosage B	2	Day 42			10/11/2010		Musculoskeletal	Normal		
S185	202	JAIVAC-1	Dosage B	2	Day 42			10/11/2010		Venous system	Normal		

Subject No.	Randomisation Number	Study Group	Dosage	Dose	Day	Not Done	Specify	Date of Assessment	*Was the assessment performed prior to vaccination?	Physical Examination Parameter	Result	Abnormality	Clinical Significance
S185	202	JAIVAC-1	Dosage B	2	Day 42			10/11/2010		Gastrointestinal	Normal		
S185	202	JAIVAC-1	Dosage B	2	Day 42			10/11/2010		Lymphatic	Normal		
S185	202	JAIVAC-1	Dosage B	2	Day 42			10/11/2010		Neurological	Normal		
S185	202	JAIVAC-1	Dosage B	2	Day 42			10/11/2010		Genitourinary	Normal		
S185	202	JAIVAC-1	Dosage B	2	Day 42			10/11/2010		Dermatological	Normal		
S185	202	JAIVAC-1	Dosage B	2	Day 56			23/11/2010		Head and Neck	Normal		
S185	202	JAIVAC-1	Dosage B	2	Day 56			23/11/2010		Eyes	Normal		
S185	202	JAIVAC-1	Dosage B	2	Day 56			23/11/2010		Ears, nose and throat	Normal		
S185	202	JAIVAC-1	Dosage B	2	Day 56			23/11/2010		Cardiovascular	Normal		
S185	202	JAIVAC-1	Dosage B	2	Day 56			23/11/2010		Respiratory	Normal		
S185	202	JAIVAC-1	Dosage B	2	Day 56			23/11/2010		Musculoskeletal	Normal		
S185	202	JAIVAC-1	Dosage B	2	Day 56			23/11/2010		Venous system	Normal		
S185	202	JAIVAC-1	Dosage B	2	Day 56			23/11/2010		Gastrointestinal	Normal		
S185	202	JAIVAC-1	Dosage B	2	Day 56			23/11/2010		Lymphatic	Normal		
S185	202	JAIVAC-1	Dosage B	2	Day 56			23/11/2010		Neurological	Normal		
S185	202	JAIVAC-1	Dosage B	2	Day 56			23/11/2010		Genitourinary	Normal		
S185	202	JAIVAC-1	Dosage B	2	Day 56			23/11/2010		Dermatological	Normal		
S185	202	JAIVAC-1	Dosage B	3	Day 180			28/03/2011	Yes	Head and Neck	Normal		
S185	202	JAIVAC-1	Dosage B	3	Day 180			28/03/2011	Yes	Eyes	Normal		

Subject No.	Randomisation Number	Study Group	Dosage	Dose	Day	Not Done	Specify	Date of Assessment	*Was the assessment performed prior to vaccination?	Physical Examination Parameter	Result	Abnormality	Clinical Significance
S185	202	JAIVAC-1	Dosage B	3	Day 180			28/03/2011	Yes	Ears, nose and throat	Normal		
S185	202	JAIVAC-1	Dosage B	3	Day 180			28/03/2011	Yes	Cardiovascular	Normal		
S185	202	JAIVAC-1	Dosage B	3	Day 180			28/03/2011	Yes	Respiratory	Normal		
S185	202	JAIVAC-1	Dosage B	3	Day 180			28/03/2011	Yes	Musculoskeletal	Normal		
S185	202	JAIVAC-1	Dosage B	3	Day 180			28/03/2011	Yes	Venous system	Normal		
S185	202	JAIVAC-1	Dosage B	3	Day 180			28/03/2011	Yes	Gastrointestinal	Normal		
S185	202	JAIVAC-1	Dosage B	3	Day 180			28/03/2011	Yes	Lymphatic	Normal		
S185	202	JAIVAC-1	Dosage B	3	Day 180			28/03/2011	Yes	Neurological	Normal		
S185	202	JAIVAC-1	Dosage B	3	Day 180			28/03/2011	Yes	Genitourinary	Normal		
S185	202	JAIVAC-1	Dosage B	3	Day 180			28/03/2011	Yes	Dermatological	Normal		
S185	202	JAIVAC-1	Dosage B	3	Day 187			02/04/2011		Head and Neck	Normal		
S185	202	JAIVAC-1	Dosage B	3	Day 187			02/04/2011		Eyes	Normal		
S185	202	JAIVAC-1	Dosage B	3	Day 187			02/04/2011		Ears, nose and throat	Normal		
S185	202	JAIVAC-1	Dosage B	3	Day 187			02/04/2011		Cardiovascular	Normal		
S185	202	JAIVAC-1	Dosage B	3	Day 187			02/04/2011		Respiratory	Normal		
S185	202	JAIVAC-1	Dosage B	3	Day 187			02/04/2011		Musculoskeletal	Normal		
S185	202	JAIVAC-1	Dosage B	3	Day 187			02/04/2011		Venous system	Normal		
S185	202	JAIVAC-1	Dosage B	3	Day 187			02/04/2011		Gastrointestinal	Normal		
S185	202	JAIVAC-1	Dosage B	3	Day 187			02/04/2011		Lymphatic	Normal		

Subject No.	Randomisation Number	Study Group	Dosage	Dose	Day	Not Done	Specify	Date of Assessment	*Was the assessment performed prior to vaccination?	Physical Examination Parameter	Result	Abnormality	Clinical Significance
S185	202	JAIVAC-1	Dosage B	3	Day 187			02/04/2011		Neurological	Normal		
S185	202	JAIVAC-1	Dosage B	3	Day 187			02/04/2011		Genitourinary	Normal		
S185	202	JAIVAC-1	Dosage B	3	Day 187			02/04/2011		Dermatological	Normal		
S185	202	JAIVAC-1	Dosage B	3	Day 194			11/04/2011		Head and Neck	Normal		
S185	202	JAIVAC-1	Dosage B	3	Day 194			11/04/2011		Eyes	Normal		
S185	202	JAIVAC-1	Dosage B	3	Day 194			11/04/2011		Ears, nose and throat	Normal		
S185	202	JAIVAC-1	Dosage B	3	Day 194			11/04/2011		Cardiovascular	Normal		
S185	202	JAIVAC-1	Dosage B	3	Day 194			11/04/2011		Respiratory	Normal		
S185	202	JAIVAC-1	Dosage B	3	Day 194			11/04/2011		Musculoskeletal	Normal		
S185	202	JAIVAC-1	Dosage B	3	Day 194			11/04/2011		Venous system	Normal		
S185	202	JAIVAC-1	Dosage B	3	Day 194			11/04/2011		Gastrointestinal	Normal		
S185	202	JAIVAC-1	Dosage B	3	Day 194			11/04/2011		Lymphatic	Normal		
S185	202	JAIVAC-1	Dosage B	3	Day 194			11/04/2011		Neurological	Normal		
S185	202	JAIVAC-1	Dosage B	3	Day 194			11/04/2011		Genitourinary	Normal		
S185	202	JAIVAC-1	Dosage B	3	Day 194			11/04/2011		Dermatological	Normal		
S185	202	JAIVAC-1	Dosage B	3	Day 208			25/04/2011		Head and Neck	Normal		
S185	202	JAIVAC-1	Dosage B	3	Day 208			25/04/2011		Eyes	Normal		
S185	202	JAIVAC-1	Dosage B	3	Day 208			25/04/2011		Ears, nose and throat	Normal		
S185	202	JAIVAC-1	Dosage B	3	Day 208			25/04/2011		Cardiovascular	Normal		

Subject No.	Randomisation Number	Study Group	Dosage	Dose	Day	Not Done	Specify	Date of Assessment	*Was the assessment performed prior to vaccination?	Physical Examination Parameter	Result	Abnormality	Clinical Significance
S185	202	JAIVAC-1	Dosage B	3	Day 208			25/04/2011		Respiratory	Normal		
S185	202	JAIVAC-1	Dosage B	3	Day 208			25/04/2011		Musculoskeletal	Normal		
S185	202	JAIVAC-1	Dosage B	3	Day 208			25/04/2011		Venous system	Normal		
S185	202	JAIVAC-1	Dosage B	3	Day 208			25/04/2011		Gastrointestinal	Normal		
S185	202	JAIVAC-1	Dosage B	3	Day 208			25/04/2011		Lymphatic	Normal		
S185	202	JAIVAC-1	Dosage B	3	Day 208			25/04/2011		Neurological	Normal		
S185	202	JAIVAC-1	Dosage B	3	Day 208			25/04/2011		Genitourinary	Normal		
S185	202	JAIVAC-1	Dosage B	3	Day 208			25/04/2011		Dermatological	Normal		
S185	202	JAIVAC-1	Dosage B	3	Day 365			28/09/2011		Head and Neck	Normal		
S185	202	JAIVAC-1	Dosage B	3	Day 365			28/09/2011		Eyes	Normal		
S185	202	JAIVAC-1	Dosage B	3	Day 365			28/09/2011		Ears, nose and throat	Normal		
S185	202	JAIVAC-1	Dosage B	3	Day 365			28/09/2011		Cardiovascular	Normal		
S185	202	JAIVAC-1	Dosage B	3	Day 365			28/09/2011		Respiratory	Normal		
S185	202	JAIVAC-1	Dosage B	3	Day 365			28/09/2011		Musculoskeletal	Normal		
S185	202	JAIVAC-1	Dosage B	3	Day 365			28/09/2011		Venous system	Normal		
S185	202	JAIVAC-1	Dosage B	3	Day 365			28/09/2011		Gastrointestinal	Normal		
S185	202	JAIVAC-1	Dosage B	3	Day 365			28/09/2011		Lymphatic	Normal		
S185	202	JAIVAC-1	Dosage B	3	Day 365			28/09/2011		Neurological	Normal		
S185	202	JAIVAC-1	Dosage B	3	Day 365			28/09/2011		Genitourinary	Normal		

Subject No.	Randomisation Number	Study Group	Dosage	Dose	Day	Not Done	Specify	Date of Assessment	*Was the assessment performed prior to vaccination?	Physical Examination Parameter	Result	Abnormality	Clinical Significance
S185	202	JAIVAC-1	Dosage B	3	Day 365			28/09/2011		Dermatological	Normal		
S186	201	JAIVAC-1	Dosage B		Day -14 to Day -1			21/09/2010		Head and neck	Normal		
S186	201	JAIVAC-1	Dosage B		Day -14 to Day -1			21/09/2010		Eyes	Normal		
S186	201	JAIVAC-1	Dosage B		Day -14 to Day -1			21/09/2010		Ears, nose and throat	Normal		
S186	201	JAIVAC-1	Dosage B		Day -14 to Day -1			21/09/2010		Cardiovascular	Normal		
S186	201	JAIVAC-1	Dosage B		Day -14 to Day -1			21/09/2010		Respiratory	Normal		
S186	201	JAIVAC-1	Dosage B		Day -14 to Day -1			21/09/2010		Musculoskeletal	Normal		
S186	201	JAIVAC-1	Dosage B		Day -14 to Day -1			21/09/2010		Venous system	Normal		
S186	201	JAIVAC-1	Dosage B		Day -14 to Day -1			21/09/2010		Gastrointestinal	Normal		
S186	201	JAIVAC-1	Dosage B		Day -14 to Day -1			21/09/2010		Lymphatic	Normal		
S186	201	JAIVAC-1	Dosage B		Day -14 to Day -1			21/09/2010		Neurological	Normal		
S186	201	JAIVAC-1	Dosage B		Day -14 to Day -1			21/09/2010		Genitourinary	Normal		
S186	201	JAIVAC-1	Dosage B		Day -14 to Day -1			21/09/2010		Dermatological	Normal		
S186	201	JAIVAC-1	Dosage B	1	Day 0			28/09/2010	Yes	Head and neck	Normal		
S186	201	JAIVAC-1	Dosage B	1	Day 0			28/09/2010	Yes	Eyes	Normal		
S186	201	JAIVAC-1	Dosage B	1	Day 0			28/09/2010	Yes	Ears, nose and throat	Normal		
S186	201	JAIVAC-1	Dosage B	1	Day 0			28/09/2010	Yes	Cardiovascular	Normal		

Subject No.	Randomisation Number	Study Group	Dosage	Dose	Day	Not Done	Specify	Date of Assessment	*Was the assessment performed prior to vaccination?	Physical Examination Parameter	Result	Abnormality	Clinical Significance
S186	201	JAIVAC-1	Dosage B	1	Day 0			28/09/2010	Yes	Respiratory	Normal		
S186	201	JAIVAC-1	Dosage B	1	Day 0			28/09/2010	Yes	Musculoskeletal	Normal		
S186	201	JAIVAC-1	Dosage B	1	Day 0			28/09/2010	Yes	Venous system	Normal		
S186	201	JAIVAC-1	Dosage B	1	Day 0			28/09/2010	Yes	Gastrointestinal	Normal		
S186	201	JAIVAC-1	Dosage B	1	Day 0			28/09/2010	Yes	Lymphatic	Normal		
S186	201	JAIVAC-1	Dosage B	1	Day 0			28/09/2010	Yes	Neurological	Normal		
S186	201	JAIVAC-1	Dosage B	1	Day 0			28/09/2010	Yes	Genitourinary	Normal		
S186	201	JAIVAC-1	Dosage B	1	Day 0			28/09/2010	Yes	Dermatological	Normal		
S186	201	JAIVAC-1	Dosage B	1	Day 7			05/10/2010		Head and Neck	Normal		
S186	201	JAIVAC-1	Dosage B	1	Day 7			05/10/2010		Eyes	Normal		
S186	201	JAIVAC-1	Dosage B	1	Day 7			05/10/2010		Ears, nose and throat	Normal		
S186	201	JAIVAC-1	Dosage B	1	Day 7			05/10/2010		Cardiovascular	Normal		
S186	201	JAIVAC-1	Dosage B	1	Day 7			05/10/2010		Respiratory	Normal		
S186	201	JAIVAC-1	Dosage B	1	Day 7			05/10/2010		Musculoskeletal	Normal		
S186	201	JAIVAC-1	Dosage B	1	Day 7			05/10/2010		Venous system	Normal		
S186	201	JAIVAC-1	Dosage B	1	Day 7			05/10/2010		Gastrointestinal	Normal		
S186	201	JAIVAC-1	Dosage B	1	Day 7			05/10/2010		Lymphatic	Normal		
S186	201	JAIVAC-1	Dosage B	1	Day 7			05/10/2010		Neurological	Normal		
S186	201	JAIVAC-1	Dosage B	1	Day 7			05/10/2010		Genitourinary	Normal		

Subject No.	Randomisation Number	Study Group	Dosage	Dose	Day	Not Done	Specify	Date of Assessment	*Was the assessment performed prior to vaccination?	Physical Examination Parameter	Result	Abnormality	Clinical Significance
S186	201	JAIVAC-1	Dosage B	1	Day 7			05/10/2010		Dermatological	Normal		
S186	201	JAIVAC-1	Dosage B	1	Day 14			12/10/2010		Head and Neck	Normal		
S186	201	JAIVAC-1	Dosage B	1	Day 14			12/10/2010		Eyes	Normal		
S186	201	JAIVAC-1	Dosage B	1	Day 14			12/10/2010		Ears, nose and throat	Normal		
S186	201	JAIVAC-1	Dosage B	1	Day 14			12/10/2010		Cardiovascular	Normal		
S186	201	JAIVAC-1	Dosage B	1	Day 14			12/10/2010		Respiratory	Normal		
S186	201	JAIVAC-1	Dosage B	1	Day 14			12/10/2010		Musculoskeletal	Normal		
S186	201	JAIVAC-1	Dosage B	1	Day 14			12/10/2010		Venous system	Normal		
S186	201	JAIVAC-1	Dosage B	1	Day 14			12/10/2010		Gastrointestinal	Normal		
S186	201	JAIVAC-1	Dosage B	1	Day 14			12/10/2010		Lymphatic	Normal		
S186	201	JAIVAC-1	Dosage B	1	Day 14			12/10/2010		Neurological	Normal		
S186	201	JAIVAC-1	Dosage B	1	Day 14			12/10/2010		Genitourinary	Normal		
S186	201	JAIVAC-1	Dosage B	1	Day 14			12/10/2010		Dermatological	Normal		
S186	201	JAIVAC-1	Dosage B	2	Day 28			26/10/2010	Yes	Head and Neck	Normal		
S186	201	JAIVAC-1	Dosage B	2	Day 28			26/10/2010	Yes	Eyes	Normal		
S186	201	JAIVAC-1	Dosage B	2	Day 28			26/10/2010	Yes	Ears, nose and throat	Normal		
S186	201	JAIVAC-1	Dosage B	2	Day 28			26/10/2010	Yes	Cardiovascular	Normal		
S186	201	JAIVAC-1	Dosage B	2	Day 28			26/10/2010	Yes	Respiratory	Normal		
S186	201	JAIVAC-1	Dosage B	2	Day 28			26/10/2010	Yes	Musculoskeletal	Normal		

Subject No.	Randomisation Number	Study Group	Dosage	Dose	Day	Not Done	Specify	Date of Assessment	*Was the assessment performed prior to vaccination?	Physical Examination Parameter	Result	Abnormality	Clinical Significance
S186	201	JAIVAC-1	Dosage B	2	Day 28			26/10/2010	Yes	Venous system	Normal		
S186	201	JAIVAC-1	Dosage B	2	Day 28			26/10/2010	Yes	Gastrointestinal	Normal		
S186	201	JAIVAC-1	Dosage B	2	Day 28			26/10/2010	Yes	Lymphatic	Normal		
S186	201	JAIVAC-1	Dosage B	2	Day 28			26/10/2010	Yes	Neurological	Normal		
S186	201	JAIVAC-1	Dosage B	2	Day 28			26/10/2010	Yes	Genitourinary	Normal		
S186	201	JAIVAC-1	Dosage B	2	Day 28			26/10/2010	Yes	Dermatological	Normal		
S186	201	JAIVAC-1	Dosage B	2	Day 35			02/11/2010		Head and Neck	Normal		
S186	201	JAIVAC-1	Dosage B	2	Day 35			02/11/2010		Eyes	Normal		
S186	201	JAIVAC-1	Dosage B	2	Day 35			02/11/2010		Ears, nose and throat	Normal		
S186	201	JAIVAC-1	Dosage B	2	Day 35			02/11/2010		Cardiovascular	Normal		
S186	201	JAIVAC-1	Dosage B	2	Day 35			02/11/2010		Respiratory	Normal		
S186	201	JAIVAC-1	Dosage B	2	Day 35			02/11/2010		Musculoskeletal	Normal		
S186	201	JAIVAC-1	Dosage B	2	Day 35			02/11/2010		Venous system	Normal		
S186	201	JAIVAC-1	Dosage B	2	Day 35			02/11/2010		Gastrointestinal	Normal		
S186	201	JAIVAC-1	Dosage B	2	Day 35			02/11/2010		Lymphatic	Normal		
S186	201	JAIVAC-1	Dosage B	2	Day 35			02/11/2010		Neurological	Normal		
S186	201	JAIVAC-1	Dosage B	2	Day 35			02/11/2010		Genitourinary	Normal		
S186	201	JAIVAC-1	Dosage B	2	Day 35			02/11/2010		Dermatological	Normal		
S186	201	JAIVAC-1	Dosage B	2	Day 42			09/11/2010		Head and Neck	Normal		

Subject No.	Randomisation Number	Study Group	Dosage	Dose	Day	Not Done	Specify	Date of Assessment	*Was the assessment performed prior to vaccination?	Physical Examination Parameter	Result	Abnormality	Clinical Significance
S186	201	JAIVAC-1	Dosage B	2	Day 42			09/11/2010		Eyes	Normal		
S186	201	JAIVAC-1	Dosage B	2	Day 42			09/11/2010		Ears, nose and throat	Normal		
S186	201	JAIVAC-1	Dosage B	2	Day 42			09/11/2010		Cardiovascular	Normal		
S186	201	JAIVAC-1	Dosage B	2	Day 42			09/11/2010		Respiratory	Normal		
S186	201	JAIVAC-1	Dosage B	2	Day 42			09/11/2010		Musculoskeletal	Normal		
S186	201	JAIVAC-1	Dosage B	2	Day 42			09/11/2010		Venous system	Normal		
S186	201	JAIVAC-1	Dosage B	2	Day 42			09/11/2010		Gastrointestinal	Normal		
S186	201	JAIVAC-1	Dosage B	2	Day 42			09/11/2010		Lymphatic	Normal		
S186	201	JAIVAC-1	Dosage B	2	Day 42			09/11/2010		Neurological	Normal		
S186	201	JAIVAC-1	Dosage B	2	Day 42			09/11/2010		Genitourinary	Normal		
S186	201	JAIVAC-1	Dosage B	2	Day 42			09/11/2010		Dermatological	Normal		
S186	201	JAIVAC-1	Dosage B	2	Day 56			23/11/2010		Head and Neck	Normal		
S186	201	JAIVAC-1	Dosage B	2	Day 56			23/11/2010		Eyes	Normal		
S186	201	JAIVAC-1	Dosage B	2	Day 56			23/11/2010		Ears, nose and throat	Normal		
S186	201	JAIVAC-1	Dosage B	2	Day 56			23/11/2010		Cardiovascular	Normal		
S186	201	JAIVAC-1	Dosage B	2	Day 56			23/11/2010		Respiratory	Normal		
S186	201	JAIVAC-1	Dosage B	2	Day 56			23/11/2010		Musculoskeletal	Normal		
S186	201	JAIVAC-1	Dosage B	2	Day 56			23/11/2010		Venous system	Normal		
S186	201	JAIVAC-1	Dosage B	2	Day 56			23/11/2010		Gastrointestinal	Normal		

Subject No.	Randomisation Number	Study Group	Dosage	Dose	Day	Not Done	Specify	Date of Assessment	*Was the assessment performed prior to vaccination?	Physical Examination Parameter	Result	Abnormality	Clinical Significance
S186	201	JAIVAC-1	Dosage B	2	Day 56			23/11/2010		Lymphatic	Normal		
S186	201	JAIVAC-1	Dosage B	2	Day 56			23/11/2010		Neurological	Normal		
S186	201	JAIVAC-1	Dosage B	2	Day 56			23/11/2010		Genitourinary	Normal		
S186	201	JAIVAC-1	Dosage B	2	Day 56			23/11/2010		Dermatological	Normal		
S186	201	JAIVAC-1	Dosage B	3	Day 180			28/03/2011	Yes	Head and Neck	Normal		
S186	201	JAIVAC-1	Dosage B	3	Day 180			28/03/2011	Yes	Eyes	Normal		
S186	201	JAIVAC-1	Dosage B	3	Day 180			28/03/2011	Yes	Ears, nose and throat	Normal		
S186	201	JAIVAC-1	Dosage B	3	Day 180			28/03/2011	Yes	Cardiovascular	Normal		
S186	201	JAIVAC-1	Dosage B	3	Day 180			28/03/2011	Yes	Respiratory	Normal		
S186	201	JAIVAC-1	Dosage B	3	Day 180			28/03/2011	Yes	Musculoskeletal	Normal		
S186	201	JAIVAC-1	Dosage B	3	Day 180			28/03/2011	Yes	Venous system	Normal		
S186	201	JAIVAC-1	Dosage B	3	Day 180			28/03/2011	Yes	Gastrointestinal	Normal		
S186	201	JAIVAC-1	Dosage B	3	Day 180			28/03/2011	Yes	Lymphatic	Normal		
S186	201	JAIVAC-1	Dosage B	3	Day 180			28/03/2011	Yes	Neurological	Normal		
S186	201	JAIVAC-1	Dosage B	3	Day 180			28/03/2011	Yes	Genitourinary	Normal		
S186	201	JAIVAC-1	Dosage B	3	Day 180			28/03/2011	Yes	Dermatological	Normal		
S186	201	JAIVAC-1	Dosage B	3	Day 187			02/04/2011		Head and Neck	Normal		
S186	201	JAIVAC-1	Dosage B	3	Day 187			02/04/2011		Eyes	Normal		
S186	201	JAIVAC-1	Dosage B	3	Day 187			02/04/2011		Ears, nose and throat	Normal		

Subject No.	Randomisation Number	Study Group	Dosage	Dose	Day	Not Done	Specify	Date of Assessment	*Was the assessment performed prior to vaccination?	Physical Examination Parameter	Result	Abnormality	Clinical Significance
S186	201	JAIVAC-1	Dosage B	3	Day 187			02/04/2011		Cardiovascular	Normal		
S186	201	JAIVAC-1	Dosage B	3	Day 187			02/04/2011		Respiratory	Normal		
S186	201	JAIVAC-1	Dosage B	3	Day 187			02/04/2011		Musculoskeletal	Normal		
S186	201	JAIVAC-1	Dosage B	3	Day 187			02/04/2011		Venous system	Normal		
S186	201	JAIVAC-1	Dosage B	3	Day 187			02/04/2011		Gastrointestinal	Normal		
S186	201	JAIVAC-1	Dosage B	3	Day 187			02/04/2011		Lymphatic	Normal		
S186	201	JAIVAC-1	Dosage B	3	Day 187			02/04/2011		Neurological	Normal		
S186	201	JAIVAC-1	Dosage B	3	Day 187			02/04/2011		Genitourinary	Normal		
S186	201	JAIVAC-1	Dosage B	3	Day 187			02/04/2011		Dermatological	Normal		
S186	201	JAIVAC-1	Dosage B	3	Day 194			11/04/2011		Head and Neck	Normal		
S186	201	JAIVAC-1	Dosage B	3	Day 194			11/04/2011		Eyes	Normal		
S186	201	JAIVAC-1	Dosage B	3	Day 194			11/04/2011		Ears, nose and throat	Normal		
S186	201	JAIVAC-1	Dosage B	3	Day 194			11/04/2011		Cardiovascular	Normal		
S186	201	JAIVAC-1	Dosage B	3	Day 194			11/04/2011		Respiratory	Normal		
S186	201	JAIVAC-1	Dosage B	3	Day 194			11/04/2011		Musculoskeletal	Normal		
S186	201	JAIVAC-1	Dosage B	3	Day 194			11/04/2011		Venous system	Normal		
S186	201	JAIVAC-1	Dosage B	3	Day 194			11/04/2011		Gastrointestinal	Normal		
S186	201	JAIVAC-1	Dosage B	3	Day 194			11/04/2011		Lymphatic	Normal		
S186	201	JAIVAC-1	Dosage B	3	Day 194			11/04/2011		Neurological	Normal		

Subject No.	Randomisation Number	Study Group	Dosage	Dose	Day	Not Done	Specify	Date of Assessment	*Was the assessment performed prior to vaccination?	Physical Examination Parameter	Result	Abnormality	Clinical Significance
S186	201	JAIVAC-1	Dosage B	3	Day 194			11/04/2011		Genitourinary	Normal		
S186	201	JAIVAC-1	Dosage B	3	Day 194			11/04/2011		Dermatological	Normal		
S186	201	JAIVAC-1	Dosage B	3	Day 208			25/04/2011		Head and Neck	Normal		
S186	201	JAIVAC-1	Dosage B	3	Day 208			25/04/2011		Eyes	Normal		
S186	201	JAIVAC-1	Dosage B	3	Day 208			25/04/2011		Ears, nose and throat	Normal		
S186	201	JAIVAC-1	Dosage B	3	Day 208			25/04/2011		Cardiovascular	Normal		
S186	201	JAIVAC-1	Dosage B	3	Day 208			25/04/2011		Respiratory	Normal		
S186	201	JAIVAC-1	Dosage B	3	Day 208			25/04/2011		Musculoskeletal	Normal		
S186	201	JAIVAC-1	Dosage B	3	Day 208			25/04/2011		Venous system	Normal		
S186	201	JAIVAC-1	Dosage B	3	Day 208			25/04/2011		Gastrointestinal	Normal		
S186	201	JAIVAC-1	Dosage B	3	Day 208			25/04/2011		Lymphatic	Normal		
S186	201	JAIVAC-1	Dosage B	3	Day 208			25/04/2011		Neurological	Normal		
S186	201	JAIVAC-1	Dosage B	3	Day 208			25/04/2011		Genitourinary	Normal		
S186	201	JAIVAC-1	Dosage B	3	Day 208			25/04/2011		Dermatological	Normal		
S186	201	JAIVAC-1	Dosage B	3	Day 365			28/09/2011		Head and Neck	Normal		
S186	201	JAIVAC-1	Dosage B	3	Day 365			28/09/2011		Eyes	Normal		
S186	201	JAIVAC-1	Dosage B	3	Day 365			28/09/2011		Ears, nose and throat	Normal		
S186	201	JAIVAC-1	Dosage B	3	Day 365			28/09/2011		Cardiovascular	Normal		
S186	201	JAIVAC-1	Dosage B	3	Day 365			28/09/2011		Respiratory	Normal		

Subject No.	Randomisation Number	Study Group	Dosage	Dose	Day	Not Done	Specify	Date of Assessment	*Was the assessment performed prior to vaccination?	Physical Examination Parameter	Result	Abnormality	Clinical Significance
S186	201	JAIVAC-1	Dosage B	3	Day 365			28/09/2011		Musculoskeletal	Normal		
S186	201	JAIVAC-1	Dosage B	3	Day 365			28/09/2011		Venous system	Normal		
S186	201	JAIVAC-1	Dosage B	3	Day 365			28/09/2011		Gastrointestinal	Normal		
S186	201	JAIVAC-1	Dosage B	3	Day 365			28/09/2011		Lymphatic	Normal		
S186	201	JAIVAC-1	Dosage B	3	Day 365			28/09/2011		Neurological	Normal		
S186	201	JAIVAC-1	Dosage B	3	Day 365			28/09/2011		Genitourinary	Normal		
S186	201	JAIVAC-1	Dosage B	3	Day 365			28/09/2011		Dermatological	Normal		
S192	203	JAIVAC-1	Dosage B		Day -14 to Day -1			23/09/2010		Head and neck	Normal		
S192	203	JAIVAC-1	Dosage B		Day -14 to Day -1			23/09/2010		Eyes	Normal		
S192	203	JAIVAC-1	Dosage B		Day -14 to Day -1			23/09/2010		Ears, nose and throat	Normal		
S192	203	JAIVAC-1	Dosage B		Day -14 to Day -1			23/09/2010		Cardiovascular	Normal		
S192	203	JAIVAC-1	Dosage B		Day -14 to Day -1			23/09/2010		Respiratory	Normal		
S192	203	JAIVAC-1	Dosage B		Day -14 to Day -1			23/09/2010		Musculoskeletal	Normal		
S192	203	JAIVAC-1	Dosage B		Day -14 to Day -1			23/09/2010		Venous system	Normal		
S192	203	JAIVAC-1	Dosage B		Day -14 to Day -1			23/09/2010		Gastrointestinal	Normal		
S192	203	JAIVAC-1	Dosage B		Day -14 to Day -1			23/09/2010		Lymphatic	Normal		
S192	203	JAIVAC-1	Dosage B		Day -14 to Day -1			23/09/2010		Neurological	Normal		

Subject No.	Randomisation Number	Study Group	Dosage	Dose	Day	Not Done	Specify	Date of Assessment	*Was the assessment performed prior to vaccination?	Physical Examination Parameter	Result	Abnormality	Clinical Significance
S192	203	JAIVAC-1	Dosage B		Day -14 to Day -1			23/09/2010		Genitourinary	Normal		
S192	203	JAIVAC-1	Dosage B		Day -14 to Day -1			23/09/2010		Dermatological	Normal		
S192	203	JAIVAC-1	Dosage B	1	Day 0			28/09/2010	Yes	Head and neck	Normal		
S192	203	JAIVAC-1	Dosage B	1	Day 0			28/09/2010	Yes	Eyes	Normal		
S192	203	JAIVAC-1	Dosage B	1	Day 0			28/09/2010	Yes	Ears, nose and throat	Normal		
S192	203	JAIVAC-1	Dosage B	1	Day 0			28/09/2010	Yes	Cardiovascular	Normal		
S192	203	JAIVAC-1	Dosage B	1	Day 0			28/09/2010	Yes	Respiratory	Normal		
S192	203	JAIVAC-1	Dosage B	1	Day 0			28/09/2010	Yes	Musculoskeletal	Normal		
S192	203	JAIVAC-1	Dosage B	1	Day 0			28/09/2010	Yes	Venous system	Normal		
S192	203	JAIVAC-1	Dosage B	1	Day 0			28/09/2010	Yes	Gastrointestinal	Normal		
S192	203	JAIVAC-1	Dosage B	1	Day 0			28/09/2010	Yes	Lymphatic	Normal		
S192	203	JAIVAC-1	Dosage B	1	Day 0			28/09/2010	Yes	Neurological	Normal		
S192	203	JAIVAC-1	Dosage B	1	Day 0			28/09/2010	Yes	Genitourinary	Normal		
S192	203	JAIVAC-1	Dosage B	1	Day 0			28/09/2010	Yes	Dermatological	Normal		
S192	203	JAIVAC-1	Dosage B	1	Day 7			05/10/2010		Head and Neck	Normal		
S192	203	JAIVAC-1	Dosage B	1	Day 7			05/10/2010		Eyes	Normal		
S192	203	JAIVAC-1	Dosage B	1	Day 7			05/10/2010		Ears, nose and throat	Normal		
S192	203	JAIVAC-1	Dosage B	1	Day 7			05/10/2010		Cardiovascular	Normal		
S192	203	JAIVAC-1	Dosage B	1	Day 7			05/10/2010		Respiratory	Normal		

Subject No.	Randomisation Number	Study Group	Dosage	Dose	Day	Not Done	Specify	Date of Assessment	*Was the assessment performed prior to vaccination?	Physical Examination Parameter	Result	Abnormality	Clinical Significance
S192	203	JAIVAC-1	Dosage B	1	Day 7			05/10/2010		Musculoskeletal	Normal		
S192	203	JAIVAC-1	Dosage B	1	Day 7			05/10/2010		Venous system	Normal		
S192	203	JAIVAC-1	Dosage B	1	Day 7			05/10/2010		Gastrointestinal	Normal		
S192	203	JAIVAC-1	Dosage B	1	Day 7			05/10/2010		Lymphatic	Normal		
S192	203	JAIVAC-1	Dosage B	1	Day 7			05/10/2010		Neurological	Normal		
S192	203	JAIVAC-1	Dosage B	1	Day 7			05/10/2010		Genitourinary	Normal		
S192	203	JAIVAC-1	Dosage B	1	Day 7			05/10/2010		Dermatological	Normal		
S192	203	JAIVAC-1	Dosage B	1	Day 14			12/10/2010		Head and Neck	Normal		
S192	203	JAIVAC-1	Dosage B	1	Day 14			12/10/2010		Eyes	Normal		
S192	203	JAIVAC-1	Dosage B	1	Day 14			12/10/2010		Ears, nose and throat	Normal		
S192	203	JAIVAC-1	Dosage B	1	Day 14			12/10/2010		Cardiovascular	Normal		
S192	203	JAIVAC-1	Dosage B	1	Day 14			12/10/2010		Respiratory	Normal		
S192	203	JAIVAC-1	Dosage B	1	Day 14			12/10/2010		Musculoskeletal	Normal		
S192	203	JAIVAC-1	Dosage B	1	Day 14			12/10/2010		Venous system	Normal		
S192	203	JAIVAC-1	Dosage B	1	Day 14			12/10/2010		Gastrointestinal	Normal		
S192	203	JAIVAC-1	Dosage B	1	Day 14			12/10/2010		Lymphatic	Normal		
S192	203	JAIVAC-1	Dosage B	1	Day 14			12/10/2010		Neurological	Normal		
S192	203	JAIVAC-1	Dosage B	1	Day 14			12/10/2010		Genitourinary	Normal		
S192	203	JAIVAC-1	Dosage B	1	Day 14			12/10/2010		Dermatological	Normal		

Subject No.	Randomisation Number	Study Group	Dosage	Dose	Day	Not Done	Specify	Date of Assessment	*Was the assessment performed prior to vaccination ?	Physical Examination Parameter	Result	Abnormality	Clinical Significance
S192	203	JAIVAC-1	Dosage B	2	Day 28			26/10/2010	Yes	Head and Neck	Normal		
S192	203	JAIVAC-1	Dosage B	2	Day 28			26/10/2010	Yes	Eyes	Normal		
S192	203	JAIVAC-1	Dosage B	2	Day 28			26/10/2010	Yes	Ears, nose and throat	Normal		
S192	203	JAIVAC-1	Dosage B	2	Day 28			26/10/2010	Yes	Cardiovascular	Normal		
S192	203	JAIVAC-1	Dosage B	2	Day 28			26/10/2010	Yes	Respiratory	Normal		
S192	203	JAIVAC-1	Dosage B	2	Day 28			26/10/2010	Yes	Musculoskeletal	Normal		
S192	203	JAIVAC-1	Dosage B	2	Day 28			26/10/2010	Yes	Venous system	Normal		
S192	203	JAIVAC-1	Dosage B	2	Day 28			26/10/2010	Yes	Gastrointestinal	Normal		
S192	203	JAIVAC-1	Dosage B	2	Day 28			26/10/2010	Yes	Lymphatic	Normal		
S192	203	JAIVAC-1	Dosage B	2	Day 28			26/10/2010	Yes	Neurological	Normal		
S192	203	JAIVAC-1	Dosage B	2	Day 28			26/10/2010	Yes	Genitourinary	Normal		
S192	203	JAIVAC-1	Dosage B	2	Day 28			26/10/2010	Yes	Dermatological	Normal		
S192	203	JAIVAC-1	Dosage B	2	Day 35			02/11/2010		Head and Neck	Normal		
S192	203	JAIVAC-1	Dosage B	2	Day 35			02/11/2010		Eyes	Normal		
S192	203	JAIVAC-1	Dosage B	2	Day 35			02/11/2010		Ears, nose and throat	Normal		
S192	203	JAIVAC-1	Dosage B	2	Day 35			02/11/2010		Cardiovascular	Normal		
S192	203	JAIVAC-1	Dosage B	2	Day 35			02/11/2010		Respiratory	Normal		
S192	203	JAIVAC-1	Dosage B	2	Day 35			02/11/2010		Musculoskeletal	Normal		
S192	203	JAIVAC-1	Dosage B	2	Day 35			02/11/2010		Venous system	Normal		

Subject No.	Randomisation Number	Study Group	Dosage	Dose	Day	Not Done	Specify	Date of Assessment	*Was the assessment performed prior to vaccination?	Physical Examination Parameter	Result	Abnormality	Clinical Significance
S192	203	JAIVAC-1	Dosage B	2	Day 35			02/11/2010		Gastrointestinal	Normal		
S192	203	JAIVAC-1	Dosage B	2	Day 35			02/11/2010		Lymphatic	Normal		
S192	203	JAIVAC-1	Dosage B	2	Day 35			02/11/2010		Neurological	Normal		
S192	203	JAIVAC-1	Dosage B	2	Day 35			02/11/2010		Genitourinary	Normal		
S192	203	JAIVAC-1	Dosage B	2	Day 35			02/11/2010		Dermatological	Normal		
S192	203	JAIVAC-1	Dosage B	2	Day 42			09/11/2010		Head and Neck	Normal		
S192	203	JAIVAC-1	Dosage B	2	Day 42			09/11/2010		Eyes	Normal		
S192	203	JAIVAC-1	Dosage B	2	Day 42			09/11/2010		Ears, nose and throat	Normal		
S192	203	JAIVAC-1	Dosage B	2	Day 42			09/11/2010		Cardiovascular	Normal		
S192	203	JAIVAC-1	Dosage B	2	Day 42			09/11/2010		Respiratory	Normal		
S192	203	JAIVAC-1	Dosage B	2	Day 42			09/11/2010		Musculoskeletal	Normal		
S192	203	JAIVAC-1	Dosage B	2	Day 42			09/11/2010		Venous system	Normal		
S192	203	JAIVAC-1	Dosage B	2	Day 42			09/11/2010		Gastrointestinal	Normal		
S192	203	JAIVAC-1	Dosage B	2	Day 42			09/11/2010		Lymphatic	Normal		
S192	203	JAIVAC-1	Dosage B	2	Day 42			09/11/2010		Neurological	Normal		
S192	203	JAIVAC-1	Dosage B	2	Day 42			09/11/2010		Genitourinary	Normal		
S192	203	JAIVAC-1	Dosage B	2	Day 42			09/11/2010		Dermatological	Normal		
S192	203	JAIVAC-1	Dosage B	2	Day 56			23/11/2010		Head and Neck	Normal		
S192	203	JAIVAC-1	Dosage B	2	Day 56			23/11/2010		Eyes	Normal		

Subject No.	Randomisation Number	Study Group	Dosage	Dose	Day	Not Done	Specify	Date of Assessment	*Was the assessment performed prior to vaccination?	Physical Examination Parameter	Result	Abnormality	Clinical Significance
S192	203	JAIVAC-1	Dosage B	2	Day 56			23/11/2010		Ears, nose and throat	Normal		
S192	203	JAIVAC-1	Dosage B	2	Day 56			23/11/2010		Cardiovascular	Normal		
S192	203	JAIVAC-1	Dosage B	2	Day 56			23/11/2010		Respiratory	Normal		
S192	203	JAIVAC-1	Dosage B	2	Day 56			23/11/2010		Musculoskeletal	Normal		
S192	203	JAIVAC-1	Dosage B	2	Day 56			23/11/2010		Venous system	Normal		
S192	203	JAIVAC-1	Dosage B	2	Day 56			23/11/2010		Gastrointestinal	Normal		
S192	203	JAIVAC-1	Dosage B	2	Day 56			23/11/2010		Lymphatic	Normal		
S192	203	JAIVAC-1	Dosage B	2	Day 56			23/11/2010		Neurological	Normal		
S192	203	JAIVAC-1	Dosage B	2	Day 56			23/11/2010		Genitourinary	Normal		
S192	203	JAIVAC-1	Dosage B	2	Day 56			23/11/2010		Dermatological	Normal		
S192	203	JAIVAC-1	Dosage B	3	Day 180			28/03/2011	Yes	Head and Neck	Normal		
S192	203	JAIVAC-1	Dosage B	3	Day 180			28/03/2011	Yes	Eyes	Normal		
S192	203	JAIVAC-1	Dosage B	3	Day 180			28/03/2011	Yes	Ears, nose and throat	Normal		
S192	203	JAIVAC-1	Dosage B	3	Day 180			28/03/2011	Yes	Cardiovascular	Normal		
S192	203	JAIVAC-1	Dosage B	3	Day 180			28/03/2011	Yes	Respiratory	Normal		
S192	203	JAIVAC-1	Dosage B	3	Day 180			28/03/2011	Yes	Musculoskeletal	Normal		
S192	203	JAIVAC-1	Dosage B	3	Day 180			28/03/2011	Yes	Venous system	Normal		
S192	203	JAIVAC-1	Dosage B	3	Day 180			28/03/2011	Yes	Gastrointestinal	Normal		
S192	203	JAIVAC-1	Dosage B	3	Day 180			28/03/2011	Yes	Lymphatic	Normal		

Subject No.	Randomisation Number	Study Group	Dosage	Dose	Day	Not Done	Specify	Date of Assessment	*Was the assessment performed prior to vaccination?	Physical Examination Parameter	Result	Abnormality	Clinical Significance
S192	203	JAIVAC-1	Dosage B	3	Day 180			28/03/2011	Yes	Neurological	Normal		
S192	203	JAIVAC-1	Dosage B	3	Day 180			28/03/2011	Yes	Genitourinary	Normal		
S192	203	JAIVAC-1	Dosage B	3	Day 180			28/03/2011	Yes	Dermatological	Normal		
S192	203	JAIVAC-1	Dosage B	3	Day 187			02/04/2011		Head and Neck	Normal		
S192	203	JAIVAC-1	Dosage B	3	Day 187			02/04/2011		Eyes	Normal		
S192	203	JAIVAC-1	Dosage B	3	Day 187			02/04/2011		Ears, nose and throat	Normal		
S192	203	JAIVAC-1	Dosage B	3	Day 187			02/04/2011		Cardiovascular	Normal		
S192	203	JAIVAC-1	Dosage B	3	Day 187			02/04/2011		Respiratory	Normal		
S192	203	JAIVAC-1	Dosage B	3	Day 187			02/04/2011		Musculoskeletal	Normal		
S192	203	JAIVAC-1	Dosage B	3	Day 187			02/04/2011		Venous system	Normal		
S192	203	JAIVAC-1	Dosage B	3	Day 187			02/04/2011		Gastrointestinal	Normal		
S192	203	JAIVAC-1	Dosage B	3	Day 187			02/04/2011		Lymphatic	Normal		
S192	203	JAIVAC-1	Dosage B	3	Day 187			02/04/2011		Neurological	Normal		
S192	203	JAIVAC-1	Dosage B	3	Day 187			02/04/2011		Genitourinary	Normal		
S192	203	JAIVAC-1	Dosage B	3	Day 187			02/04/2011		Dermatological	Normal		
S192	203	JAIVAC-1	Dosage B	3	Day 194			11/04/2011		Head and Neck	Normal		
S192	203	JAIVAC-1	Dosage B	3	Day 194			11/04/2011		Eyes	Normal		
S192	203	JAIVAC-1	Dosage B	3	Day 194			11/04/2011		Ears, nose and throat	Normal		
S192	203	JAIVAC-1	Dosage B	3	Day 194			11/04/2011		Cardiovascular	Normal		

Subject No.	Randomisation Number	Study Group	Dosage	Dose	Day	Not Done	Specify	Date of Assessment	*Was the assessment performed prior to vaccination?	Physical Examination Parameter	Result	Abnormality	Clinical Significance
S192	203	JAIVAC-1	Dosage B	3	Day 194			11/04/2011		Respiratory	Normal		
S192	203	JAIVAC-1	Dosage B	3	Day 194			11/04/2011		Musculoskeletal	Normal		
S192	203	JAIVAC-1	Dosage B	3	Day 194			11/04/2011		Venous system	Normal		
S192	203	JAIVAC-1	Dosage B	3	Day 194			11/04/2011		Gastrointestinal	Normal		
S192	203	JAIVAC-1	Dosage B	3	Day 194			11/04/2011		Lymphatic	Normal		
S192	203	JAIVAC-1	Dosage B	3	Day 194			11/04/2011		Neurological	Normal		
S192	203	JAIVAC-1	Dosage B	3	Day 194			11/04/2011		Genitourinary	Normal		
S192	203	JAIVAC-1	Dosage B	3	Day 194			11/04/2011		Dermatological	Normal		
S192	203	JAIVAC-1	Dosage B	3	Day 208			25/04/2011		Head and Neck	Normal		
S192	203	JAIVAC-1	Dosage B	3	Day 208			25/04/2011		Eyes	Normal		
S192	203	JAIVAC-1	Dosage B	3	Day 208			25/04/2011		Ears, nose and throat	Normal		
S192	203	JAIVAC-1	Dosage B	3	Day 208			25/04/2011		Cardiovascular	Normal		
S192	203	JAIVAC-1	Dosage B	3	Day 208			25/04/2011		Respiratory	Normal		
S192	203	JAIVAC-1	Dosage B	3	Day 208			25/04/2011		Musculoskeletal	Normal		
S192	203	JAIVAC-1	Dosage B	3	Day 208			25/04/2011		Venous system	Normal		
S192	203	JAIVAC-1	Dosage B	3	Day 208			25/04/2011		Gastrointestinal	Normal		
S192	203	JAIVAC-1	Dosage B	3	Day 208			25/04/2011		Lymphatic	Normal		
S192	203	JAIVAC-1	Dosage B	3	Day 208			25/04/2011		Neurological	Normal		
S192	203	JAIVAC-1	Dosage B	3	Day 208			25/04/2011		Genitourinary	Normal		

Subject No.	Randomisation Number	Study Group	Dosage	Dose	Day	Not Done	Specify	Date of Assessment	*Was the assessment performed prior to vaccination?	Physical Examination Parameter	Result	Abnormality	Clinical Significance
S192	203	JAIVAC-1	Dosage B	3	Day 208			25/04/2011		Dermatological	Normal		
S192	203	JAIVAC-1	Dosage B	3	Day 365			28/09/2011		Head and Neck	Normal		
S192	203	JAIVAC-1	Dosage B	3	Day 365			28/09/2011		Eyes	Normal		
S192	203	JAIVAC-1	Dosage B	3	Day 365			28/09/2011		Ears, nose and throat	Normal		
S192	203	JAIVAC-1	Dosage B	3	Day 365			28/09/2011		Cardiovascular	Normal		
S192	203	JAIVAC-1	Dosage B	3	Day 365			28/09/2011		Respiratory	Normal		
S192	203	JAIVAC-1	Dosage B	3	Day 365			28/09/2011		Musculoskeletal	Normal		
S192	203	JAIVAC-1	Dosage B	3	Day 365			28/09/2011		Venous system	Normal		
S192	203	JAIVAC-1	Dosage B	3	Day 365			28/09/2011		Gastrointestinal	Normal		
S192	203	JAIVAC-1	Dosage B	3	Day 365			28/09/2011		Lymphatic	Normal		
S192	203	JAIVAC-1	Dosage B	3	Day 365			28/09/2011		Neurological	Normal		
S192	203	JAIVAC-1	Dosage B	3	Day 365			28/09/2011		Genitourinary	Normal		
S192	203	JAIVAC-1	Dosage B	3	Day 365			28/09/2011		Dermatological	Normal		
S193	215	JAIVAC-1	Dosage B		Day -14 to Day -1			24/09/2010		Head and neck	Normal		
S193	215	JAIVAC-1	Dosage B		Day -14 to Day -1			24/09/2010		Eyes	Normal		
S193	215	JAIVAC-1	Dosage B		Day -14 to Day -1			24/09/2010		Ears, nose and throat	Normal		
S193	215	JAIVAC-1	Dosage B		Day -14 to Day -1			24/09/2010		Cardiovascular	Normal		
S193	215	JAIVAC-1	Dosage B		Day -14 to Day -1			24/09/2010		Respiratory	Normal		

Subject No.	Randomisation Number	Study Group	Dosage	Dose	Day	Not Done	Specify	Date of Assessment	*Was the assessment performed prior to vaccination?	Physical Examination Parameter	Result	Abnormality	Clinical Significance
S193	215	JAIVAC-1	Dosage B		Day -14 to Day -1			24/09/2010		Musculoskeletal	Normal		
S193	215	JAIVAC-1	Dosage B		Day -14 to Day -1			24/09/2010		Venous system	Normal		
S193	215	JAIVAC-1	Dosage B		Day -14 to Day -1			24/09/2010		Gastrointestinal	Normal		
S193	215	JAIVAC-1	Dosage B		Day -14 to Day -1			24/09/2010		Lymphatic	Normal		
S193	215	JAIVAC-1	Dosage B		Day -14 to Day -1			24/09/2010		Neurological	Normal		
S193	215	JAIVAC-1	Dosage B		Day -14 to Day -1			24/09/2010		Genitourinary	Normal		
S193	215	JAIVAC-1	Dosage B		Day -14 to Day -1			24/09/2010		Dermatological	Normal		
S193	215	JAIVAC-1	Dosage B	1	Day 0			30/09/2010	Yes	Head and neck	Normal		
S193	215	JAIVAC-1	Dosage B	1	Day 0			30/09/2010	Yes	Eyes	Normal		
S193	215	JAIVAC-1	Dosage B	1	Day 0			30/09/2010	Yes	Ears, nose and throat	Normal		
S193	215	JAIVAC-1	Dosage B	1	Day 0			30/09/2010	Yes	Cardiovascular	Normal		
S193	215	JAIVAC-1	Dosage B	1	Day 0			30/09/2010	Yes	Respiratory	Normal		
S193	215	JAIVAC-1	Dosage B	1	Day 0			30/09/2010	Yes	Musculoskeletal	Normal		
S193	215	JAIVAC-1	Dosage B	1	Day 0			30/09/2010	Yes	Venous system	Normal		
S193	215	JAIVAC-1	Dosage B	1	Day 0			30/09/2010	Yes	Gastrointestinal	Normal		
S193	215	JAIVAC-1	Dosage B	1	Day 0			30/09/2010	Yes	Lymphatic	Normal		
S193	215	JAIVAC-1	Dosage B	1	Day 0			30/09/2010	Yes	Neurological	Normal		
S193	215	JAIVAC-1	Dosage B	1	Day 0			30/09/2010	Yes	Genitourinary	Normal		

Subject No.	Randomisation Number	Study Group	Dosage	Dose	Day	Not Done	Specify	Date of Assessment	*Was the assessment performed prior to vaccination?	Physical Examination Parameter	Result	Abnormality	Clinical Significance
S193	215	JAIVAC-1	Dosage B	1	Day 0			30/09/2010	Yes	Dermatological	Normal		
S193	215	JAIVAC-1	Dosage B	1	Day 7			07/10/2010		Head and Neck	Normal		
S193	215	JAIVAC-1	Dosage B	1	Day 7			07/10/2010		Eyes	Normal		
S193	215	JAIVAC-1	Dosage B	1	Day 7			07/10/2010		Ears, nose and throat	Normal		
S193	215	JAIVAC-1	Dosage B	1	Day 7			07/10/2010		Cardiovascular	Normal		
S193	215	JAIVAC-1	Dosage B	1	Day 7			07/10/2010		Respiratory	Normal		
S193	215	JAIVAC-1	Dosage B	1	Day 7			07/10/2010		Musculoskeletal	Normal		
S193	215	JAIVAC-1	Dosage B	1	Day 7			07/10/2010		Venous system	Normal		
S193	215	JAIVAC-1	Dosage B	1	Day 7			07/10/2010		Gastrointestinal	Normal		
S193	215	JAIVAC-1	Dosage B	1	Day 7			07/10/2010		Lymphatic	Normal		
S193	215	JAIVAC-1	Dosage B	1	Day 7			07/10/2010		Neurological	Normal		
S193	215	JAIVAC-1	Dosage B	1	Day 7			07/10/2010		Genitourinary	Normal		
S193	215	JAIVAC-1	Dosage B	1	Day 7			07/10/2010		Dermatological	Normal		
S193	215	JAIVAC-1	Dosage B	1	Day 14			14/10/2010		Head and Neck	Normal		
S193	215	JAIVAC-1	Dosage B	1	Day 14			14/10/2010		Eyes	Normal		
S193	215	JAIVAC-1	Dosage B	1	Day 14			14/10/2010		Ears, nose and throat	Normal		
S193	215	JAIVAC-1	Dosage B	1	Day 14			14/10/2010		Cardiovascular	Normal		
S193	215	JAIVAC-1	Dosage B	1	Day 14			14/10/2010		Respiratory	Normal		
S193	215	JAIVAC-1	Dosage B	1	Day 14			14/10/2010		Musculoskeletal	Normal		

Subject No.	Randomisation Number	Study Group	Dosage	Dose	Day	Not Done	Specify	Date of Assessment	*Was the assessment performed prior to vaccination?	Physical Examination Parameter	Result	Abnormality	Clinical Significance
S193	215	JAIVAC-1	Dosage B	1	Day 14			14/10/2010		Venous system	Normal		
S193	215	JAIVAC-1	Dosage B	1	Day 14			14/10/2010		Gastrointestinal	Normal		
S193	215	JAIVAC-1	Dosage B	1	Day 14			14/10/2010		Lymphatic	Normal		
S193	215	JAIVAC-1	Dosage B	1	Day 14			14/10/2010		Neurological	Normal		
S193	215	JAIVAC-1	Dosage B	1	Day 14			14/10/2010		Genitourinary	Normal		
S193	215	JAIVAC-1	Dosage B	1	Day 14			14/10/2010		Dermatological	Normal		
S193	215	JAIVAC-1	Dosage B	2	Day 28			28/10/2010	Yes	Head and Neck	Normal		
S193	215	JAIVAC-1	Dosage B	2	Day 28			28/10/2010	Yes	Eyes	Normal		
S193	215	JAIVAC-1	Dosage B	2	Day 28			28/10/2010	Yes	Ears, nose and throat	Normal		
S193	215	JAIVAC-1	Dosage B	2	Day 28			28/10/2010	Yes	Cardiovascular	Normal		
S193	215	JAIVAC-1	Dosage B	2	Day 28			28/10/2010	Yes	Respiratory	Normal		
S193	215	JAIVAC-1	Dosage B	2	Day 28			28/10/2010	Yes	Musculoskeletal	Normal		
S193	215	JAIVAC-1	Dosage B	2	Day 28			28/10/2010	Yes	Venous system	Normal		
S193	215	JAIVAC-1	Dosage B	2	Day 28			28/10/2010	Yes	Gastrointestinal	Normal		
S193	215	JAIVAC-1	Dosage B	2	Day 28			28/10/2010	Yes	Lymphatic	Normal		
S193	215	JAIVAC-1	Dosage B	2	Day 28			28/10/2010	Yes	Neurological	Normal		
S193	215	JAIVAC-1	Dosage B	2	Day 28			28/10/2010	Yes	Genitourinary	Normal		
S193	215	JAIVAC-1	Dosage B	2	Day 28			28/10/2010	Yes	Dermatological	Normal		
S193	215	JAIVAC-1	Dosage B	2	Day 35			04/11/2010		Head and Neck	Normal		

Subject No.	Randomisation Number	Study Group	Dosage	Dose	Day	Not Done	Specify	Date of Assessment	*Was the assessment performed prior to vaccination ?	Physical Examination Parameter	Result	Abnormality	Clinical Significance
S193	215	JAIVAC-1	Dosage B	2	Day 35			04/11/2010		Eyes	Normal		
S193	215	JAIVAC-1	Dosage B	2	Day 35			04/11/2010		Ears, nose and throat	Normal		
S193	215	JAIVAC-1	Dosage B	2	Day 35			04/11/2010		Cardiovascular	Normal		
S193	215	JAIVAC-1	Dosage B	2	Day 35			04/11/2010		Respiratory	Normal		
S193	215	JAIVAC-1	Dosage B	2	Day 35			04/11/2010		Musculoskeletal	Normal		
S193	215	JAIVAC-1	Dosage B	2	Day 35			04/11/2010		Venous system	Normal		
S193	215	JAIVAC-1	Dosage B	2	Day 35			04/11/2010		Gastrointestinal	Normal		
S193	215	JAIVAC-1	Dosage B	2	Day 35			04/11/2010		Lymphatic	Normal		
S193	215	JAIVAC-1	Dosage B	2	Day 35			04/11/2010		Neurological	Normal		
S193	215	JAIVAC-1	Dosage B	2	Day 35			04/11/2010		Genitourinary	Normal		
S193	215	JAIVAC-1	Dosage B	2	Day 35			04/11/2010		Dermatological	Normal		
S193	215	JAIVAC-1	Dosage B	2	Day 42			11/11/2010		Head and Neck	Normal		
S193	215	JAIVAC-1	Dosage B	2	Day 42			11/11/2010		Eyes	Normal		
S193	215	JAIVAC-1	Dosage B	2	Day 42			11/11/2010		Ears, nose and throat	Normal		
S193	215	JAIVAC-1	Dosage B	2	Day 42			11/11/2010		Cardiovascular	Normal		
S193	215	JAIVAC-1	Dosage B	2	Day 42			11/11/2010		Respiratory	Normal		
S193	215	JAIVAC-1	Dosage B	2	Day 42			11/11/2010		Musculoskeletal	Normal		
S193	215	JAIVAC-1	Dosage B	2	Day 42			11/11/2010		Venous system	Normal		
S193	215	JAIVAC-1	Dosage B	2	Day 42			11/11/2010		Gastrointestinal	Normal		

Subject No.	Randomisation Number	Study Group	Dosage	Dose	Day	Not Done	Specify	Date of Assessment	*Was the assessment performed prior to vaccination?	Physical Examination Parameter	Result	Abnormality	Clinical Significance
S193	215	JAIVAC-1	Dosage B	2	Day 42			11/11/2010		Lymphatic	Normal		
S193	215	JAIVAC-1	Dosage B	2	Day 42			11/11/2010		Neurological	Normal		
S193	215	JAIVAC-1	Dosage B	2	Day 42			11/11/2010		Genitourinary	Normal		
S193	215	JAIVAC-1	Dosage B	2	Day 42			11/11/2010		Dermatological	Normal		
S193	215	JAIVAC-1	Dosage B	2	Day 56			25/11/2010		Head and Neck	Normal		
S193	215	JAIVAC-1	Dosage B	2	Day 56			25/11/2010		Eyes	Normal		
S193	215	JAIVAC-1	Dosage B	2	Day 56			25/11/2010		Ears, nose and throat	Normal		
S193	215	JAIVAC-1	Dosage B	2	Day 56			25/11/2010		Cardiovascular	Normal		
S193	215	JAIVAC-1	Dosage B	2	Day 56			25/11/2010		Respiratory	Normal		
S193	215	JAIVAC-1	Dosage B	2	Day 56			25/11/2010		Musculoskeletal	Normal		
S193	215	JAIVAC-1	Dosage B	2	Day 56			25/11/2010		Venous system	Normal		
S193	215	JAIVAC-1	Dosage B	2	Day 56			25/11/2010		Gastrointestinal	Normal		
S193	215	JAIVAC-1	Dosage B	2	Day 56			25/11/2010		Lymphatic	Normal		
S193	215	JAIVAC-1	Dosage B	2	Day 56			25/11/2010		Neurological	Normal		
S193	215	JAIVAC-1	Dosage B	2	Day 56			25/11/2010		Genitourinary	Normal		
S193	215	JAIVAC-1	Dosage B	2	Day 56			25/11/2010		Dermatological	Normal		
S193	215	JAIVAC-1	Dosage B	3	Day 180			29/03/2011	Yes	Head and Neck	Normal		
S193	215	JAIVAC-1	Dosage B	3	Day 180			29/03/2011	Yes	Eyes	Normal		
S193	215	JAIVAC-1	Dosage B	3	Day 180			29/03/2011	Yes	Ears, nose and throat	Normal		

Subject No.	Randomisation Number	Study Group	Dosage	Dose	Day	Not Done	Specify	Date of Assessment	*Was the assessment performed prior to vaccination?	Physical Examination Parameter	Result	Abnormality	Clinical Significance
S193	215	JAIVAC-1	Dosage B	3	Day 180			29/03/2011	Yes	Cardiovascular	Normal		
S193	215	JAIVAC-1	Dosage B	3	Day 180			29/03/2011	Yes	Respiratory	Normal		
S193	215	JAIVAC-1	Dosage B	3	Day 180			29/03/2011	Yes	Musculoskeletal	Normal		
S193	215	JAIVAC-1	Dosage B	3	Day 180			29/03/2011	Yes	Venous system	Normal		
S193	215	JAIVAC-1	Dosage B	3	Day 180			29/03/2011	Yes	Gastrointestinal	Normal		
S193	215	JAIVAC-1	Dosage B	3	Day 180			29/03/2011	Yes	Lymphatic	Normal		
S193	215	JAIVAC-1	Dosage B	3	Day 180			29/03/2011	Yes	Neurological	Normal		
S193	215	JAIVAC-1	Dosage B	3	Day 180			29/03/2011	Yes	Genitourinary	Normal		
S193	215	JAIVAC-1	Dosage B	3	Day 180			29/03/2011	Yes	Dermatological	Normal		
S193	215	JAIVAC-1	Dosage B	3	Day 187			05/04/2011		Head and Neck	Normal		
S193	215	JAIVAC-1	Dosage B	3	Day 187			05/04/2011		Eyes	Normal		
S193	215	JAIVAC-1	Dosage B	3	Day 187			05/04/2011		Ears, nose and throat	Normal		
S193	215	JAIVAC-1	Dosage B	3	Day 187			05/04/2011		Cardiovascular	Normal		
S193	215	JAIVAC-1	Dosage B	3	Day 187			05/04/2011		Respiratory	Normal		
S193	215	JAIVAC-1	Dosage B	3	Day 187			05/04/2011		Musculoskeletal	Normal		
S193	215	JAIVAC-1	Dosage B	3	Day 187			05/04/2011		Venous system	Normal		
S193	215	JAIVAC-1	Dosage B	3	Day 187			05/04/2011		Gastrointestinal	Normal		
S193	215	JAIVAC-1	Dosage B	3	Day 187			05/04/2011		Lymphatic	Normal		
S193	215	JAIVAC-1	Dosage B	3	Day 187			05/04/2011		Neurological	Normal		

Subject No.	Randomisation Number	Study Group	Dosage	Dose	Day	Not Done	Specify	Date of Assessment	*Was the assessment performed prior to vaccination?	Physical Examination Parameter	Result	Abnormality	Clinical Significance
S193	215	JAIVAC-1	Dosage B	3	Day 187			05/04/2011		Genitourinary	Normal		
S193	215	JAIVAC-1	Dosage B	3	Day 187			05/04/2011		Dermatological	Normal		
S193	215	JAIVAC-1	Dosage B	3	Day 194			12/04/2011		Head and Neck	Normal		
S193	215	JAIVAC-1	Dosage B	3	Day 194			12/04/2011		Eyes	Normal		
S193	215	JAIVAC-1	Dosage B	3	Day 194			12/04/2011		Ears, nose and throat	Normal		
S193	215	JAIVAC-1	Dosage B	3	Day 194			12/04/2011		Cardiovascular	Normal		
S193	215	JAIVAC-1	Dosage B	3	Day 194			12/04/2011		Respiratory	Normal		
S193	215	JAIVAC-1	Dosage B	3	Day 194			12/04/2011		Musculoskeletal	Normal		
S193	215	JAIVAC-1	Dosage B	3	Day 194			12/04/2011		Venous system	Normal		
S193	215	JAIVAC-1	Dosage B	3	Day 194			12/04/2011		Gastrointestinal	Normal		
S193	215	JAIVAC-1	Dosage B	3	Day 194			12/04/2011		Lymphatic	Normal		
S193	215	JAIVAC-1	Dosage B	3	Day 194			12/04/2011		Neurological	Normal		
S193	215	JAIVAC-1	Dosage B	3	Day 194			12/04/2011		Genitourinary	Normal		
S193	215	JAIVAC-1	Dosage B	3	Day 194			12/04/2011		Dermatological	Normal		
S193	215	JAIVAC-1	Dosage B	3	Day 208			26/04/2011		Head and Neck	Normal		
S193	215	JAIVAC-1	Dosage B	3	Day 208			26/04/2011		Eyes	Normal		
S193	215	JAIVAC-1	Dosage B	3	Day 208			26/04/2011		Ears, nose and throat	Normal		
S193	215	JAIVAC-1	Dosage B	3	Day 208			26/04/2011		Cardiovascular	Normal		
S193	215	JAIVAC-1	Dosage B	3	Day 208			26/04/2011		Respiratory	Normal		

Subject No.	Randomisation Number	Study Group	Dosage	Dose	Day	Not Done	Specify	Date of Assessment	*Was the assessment performed prior to vaccination?	Physical Examination Parameter	Result	Abnormality	Clinical Significance
S193	215	JAIVAC-1	Dosage B	3	Day 208			26/04/2011		Musculoskeletal	Normal		
S193	215	JAIVAC-1	Dosage B	3	Day 208			26/04/2011		Venous system	Normal		
S193	215	JAIVAC-1	Dosage B	3	Day 208			26/04/2011		Gastrointestinal	Normal		
S193	215	JAIVAC-1	Dosage B	3	Day 208			26/04/2011		Lymphatic	Normal		
S193	215	JAIVAC-1	Dosage B	3	Day 208			26/04/2011		Neurological	Normal		
S193	215	JAIVAC-1	Dosage B	3	Day 208			26/04/2011		Genitourinary	Normal		
S193	215	JAIVAC-1	Dosage B	3	Day 208			26/04/2011		Dermatological	Normal		
S193	215	JAIVAC-1	Dosage B	3	Day 365			29/09/2011		Head and Neck	Normal		
S193	215	JAIVAC-1	Dosage B	3	Day 365			29/09/2011		Eyes	Normal		
S193	215	JAIVAC-1	Dosage B	3	Day 365			29/09/2011		Ears, nose and throat	Normal		
S193	215	JAIVAC-1	Dosage B	3	Day 365			29/09/2011		Cardiovascular	Normal		
S193	215	JAIVAC-1	Dosage B	3	Day 365			29/09/2011		Respiratory	Normal		
S193	215	JAIVAC-1	Dosage B	3	Day 365			29/09/2011		Musculoskeletal	Normal		
S193	215	JAIVAC-1	Dosage B	3	Day 365			29/09/2011		Venous system	Normal		
S193	215	JAIVAC-1	Dosage B	3	Day 365			29/09/2011		Gastrointestinal	Normal		
S193	215	JAIVAC-1	Dosage B	3	Day 365			29/09/2011		Lymphatic	Normal		
S193	215	JAIVAC-1	Dosage B	3	Day 365			29/09/2011		Neurological	Normal		
S193	215	JAIVAC-1	Dosage B	3	Day 365			29/09/2011		Genitourinary	Normal		
S193	215	JAIVAC-1	Dosage B	3	Day 365			29/09/2011		Dermatological	Normal		

Subject No.	Randomisation Number	Study Group	Dosage	Dose	Day	Not Done	Specify	Date of Assessment	*Was the assessment performed prior to vaccination?	Physical Examination Parameter	Result	Abnormality	Clinical Significance
S195	214	JAIVAC-1	Dosage B		Day -14 to Day -1			23/09/2010		Head and neck	Normal		
S195	214	JAIVAC-1	Dosage B		Day -14 to Day -1			23/09/2010		Eyes	Normal		
S195	214	JAIVAC-1	Dosage B		Day -14 to Day -1			23/09/2010		Ears, nose and throat	Normal		
S195	214	JAIVAC-1	Dosage B		Day -14 to Day -1			23/09/2010		Cardiovascular	Normal		
S195	214	JAIVAC-1	Dosage B		Day -14 to Day -1			23/09/2010		Respiratory	Normal		
S195	214	JAIVAC-1	Dosage B		Day -14 to Day -1			23/09/2010		Musculoskeletal	Normal		
S195	214	JAIVAC-1	Dosage B		Day -14 to Day -1			23/09/2010		Venous system	Normal		
S195	214	JAIVAC-1	Dosage B		Day -14 to Day -1			23/09/2010		Gastrointestinal	Normal		
S195	214	JAIVAC-1	Dosage B		Day -14 to Day -1			23/09/2010		Lymphatic	Normal		
S195	214	JAIVAC-1	Dosage B		Day -14 to Day -1			23/09/2010		Neurological	Normal		
S195	214	JAIVAC-1	Dosage B		Day -14 to Day -1			23/09/2010		Genitourinary	Normal		
S195	214	JAIVAC-1	Dosage B		Day -14 to Day -1			23/09/2010		Dermatological	Normal		
S195	214	JAIVAC-1	Dosage B	1	Day 0			30/09/2010	Yes	Head and neck	Normal		
S195	214	JAIVAC-1	Dosage B	1	Day 0			30/09/2010	Yes	Eyes	Normal		
S195	214	JAIVAC-1	Dosage B	1	Day 0			30/09/2010	Yes	Ears, nose and throat	Normal		
S195	214	JAIVAC-1	Dosage B	1	Day 0			30/09/2010	Yes	Cardiovascular	Normal		
S195	214	JAIVAC-1	Dosage B	1	Day 0			30/09/2010	Yes	Respiratory	Normal		

Subject No.	Randomisation Number	Study Group	Dosage	Dose	Day	Not Done	Specify	Date of Assessment	*Was the assessment performed prior to vaccination?	Physical Examination Parameter	Result	Abnormality	Clinical Significance
S195	214	JAIVAC-1	Dosage B	1	Day 0			30/09/2010	Yes	Musculoskeletal	Normal		
S195	214	JAIVAC-1	Dosage B	1	Day 0			30/09/2010	Yes	Venous system	Normal		
S195	214	JAIVAC-1	Dosage B	1	Day 0			30/09/2010	Yes	Gastrointestinal	Normal		
S195	214	JAIVAC-1	Dosage B	1	Day 0			30/09/2010	Yes	Lymphatic	Normal		
S195	214	JAIVAC-1	Dosage B	1	Day 0			30/09/2010	Yes	Neurological	Normal		
S195	214	JAIVAC-1	Dosage B	1	Day 0			30/09/2010	Yes	Genitourinary	Normal		
S195	214	JAIVAC-1	Dosage B	1	Day 0			30/09/2010	Yes	Dermatological	Normal		
S195	214	JAIVAC-1	Dosage B	1	Day 7			07/10/2010		Head and Neck	Normal		
S195	214	JAIVAC-1	Dosage B	1	Day 7			07/10/2010		Eyes	Normal		
S195	214	JAIVAC-1	Dosage B	1	Day 7			07/10/2010		Ears, nose and throat	Normal		
S195	214	JAIVAC-1	Dosage B	1	Day 7			07/10/2010		Cardiovascular	Normal		
S195	214	JAIVAC-1	Dosage B	1	Day 7			07/10/2010		Respiratory	Normal		
S195	214	JAIVAC-1	Dosage B	1	Day 7			07/10/2010		Musculoskeletal	Normal		
S195	214	JAIVAC-1	Dosage B	1	Day 7			07/10/2010		Venous system	Normal		
S195	214	JAIVAC-1	Dosage B	1	Day 7			07/10/2010		Gastrointestinal	Normal		
S195	214	JAIVAC-1	Dosage B	1	Day 7			07/10/2010		Lymphatic	Normal		
S195	214	JAIVAC-1	Dosage B	1	Day 7			07/10/2010		Neurological	Normal		
S195	214	JAIVAC-1	Dosage B	1	Day 7			07/10/2010		Genitourinary	Normal		
S195	214	JAIVAC-1	Dosage B	1	Day 7			07/10/2010		Dermatological	Normal		

Subject No.	Randomisation Number	Study Group	Dosage	Dose	Day	Not Done	Specify	Date of Assessment	*Was the assessment performed prior to vaccination?	Physical Examination Parameter	Result	Abnormality	Clinical Significance
S195	214	JAIVAC-1	Dosage B	1	Day 14			14/10/2010		Head and Neck	Normal		
S195	214	JAIVAC-1	Dosage B	1	Day 14			14/10/2010		Eyes	Normal		
S195	214	JAIVAC-1	Dosage B	1	Day 14			14/10/2010		Ears, nose and throat	Normal		
S195	214	JAIVAC-1	Dosage B	1	Day 14			14/10/2010		Cardiovascular	Normal		
S195	214	JAIVAC-1	Dosage B	1	Day 14			14/10/2010		Respiratory	Normal		
S195	214	JAIVAC-1	Dosage B	1	Day 14			14/10/2010		Musculoskeletal	Normal		
S195	214	JAIVAC-1	Dosage B	1	Day 14			14/10/2010		Venous system	Normal		
S195	214	JAIVAC-1	Dosage B	1	Day 14			14/10/2010		Gastrointestinal	Normal		
S195	214	JAIVAC-1	Dosage B	1	Day 14			14/10/2010		Lymphatic	Normal		
S195	214	JAIVAC-1	Dosage B	1	Day 14			14/10/2010		Neurological	Normal		
S195	214	JAIVAC-1	Dosage B	1	Day 14			14/10/2010		Genitourinary	Normal		
S195	214	JAIVAC-1	Dosage B	1	Day 14			14/10/2010		Dermatological	Normal		
S195	214	JAIVAC-1	Dosage B	2	Day 28			28/10/2010	Yes	Head and Neck	Normal		
S195	214	JAIVAC-1	Dosage B	2	Day 28			28/10/2010	Yes	Eyes	Normal		
S195	214	JAIVAC-1	Dosage B	2	Day 28			28/10/2010	Yes	Ears, nose and throat	Normal		
S195	214	JAIVAC-1	Dosage B	2	Day 28			28/10/2010	Yes	Cardiovascular	Normal		
S195	214	JAIVAC-1	Dosage B	2	Day 28			28/10/2010	Yes	Respiratory	Normal		
S195	214	JAIVAC-1	Dosage B	2	Day 28			28/10/2010	Yes	Musculoskeletal	Normal		
S195	214	JAIVAC-1	Dosage B	2	Day 28			28/10/2010	Yes	Venous system	Normal		

Subject No.	Randomisation Number	Study Group	Dosage	Dose	Day	Not Done	Specify	Date of Assessment	*Was the assessment performed prior to vaccination?	Physical Examination Parameter	Result	Abnormality	Clinical Significance
S195	214	JAIVAC-1	Dosage B	2	Day 28			28/10/2010	Yes	Gastrointestinal	Normal		
S195	214	JAIVAC-1	Dosage B	2	Day 28			28/10/2010	Yes	Lymphatic	Normal		
S195	214	JAIVAC-1	Dosage B	2	Day 28			28/10/2010	Yes	Neurological	Normal		
S195	214	JAIVAC-1	Dosage B	2	Day 28			28/10/2010	Yes	Genitourinary	Normal		
S195	214	JAIVAC-1	Dosage B	2	Day 28			28/10/2010	Yes	Dermatological	Normal		
S195	214	JAIVAC-1	Dosage B	2	Day 35			04/11/2010		Head and Neck	Normal		
S195	214	JAIVAC-1	Dosage B	2	Day 35			04/11/2010		Eyes	Normal		
S195	214	JAIVAC-1	Dosage B	2	Day 35			04/11/2010		Ears, nose and throat	Normal		
S195	214	JAIVAC-1	Dosage B	2	Day 35			04/11/2010		Cardiovascular	Normal		
S195	214	JAIVAC-1	Dosage B	2	Day 35			04/11/2010		Respiratory	Normal		
S195	214	JAIVAC-1	Dosage B	2	Day 35			04/11/2010		Musculoskeletal	Normal		
S195	214	JAIVAC-1	Dosage B	2	Day 35			04/11/2010		Venous system	Normal		
S195	214	JAIVAC-1	Dosage B	2	Day 35			04/11/2010		Gastrointestinal	Normal		
S195	214	JAIVAC-1	Dosage B	2	Day 35			04/11/2010		Lymphatic	Normal		
S195	214	JAIVAC-1	Dosage B	2	Day 35			04/11/2010		Neurological	Normal		
S195	214	JAIVAC-1	Dosage B	2	Day 35			04/11/2010		Genitourinary	Normal		
S195	214	JAIVAC-1	Dosage B	2	Day 35			04/11/2010		Dermatological	Normal		
S195	214	JAIVAC-1	Dosage B	2	Day 42			11/11/2010		Head and Neck	Normal		
S195	214	JAIVAC-1	Dosage B	2	Day 42			11/11/2010		Eyes	Normal		

Subject No.	Randomisation Number	Study Group	Dosage	Dose	Day	Not Done	Specify	Date of Assessment	*Was the assessment performed prior to vaccination?	Physical Examination Parameter	Result	Abnormality	Clinical Significance
S195	214	JAIVAC-1	Dosage B	2	Day 42			11/11/2010		Ears, nose and throat	Normal		
S195	214	JAIVAC-1	Dosage B	2	Day 42			11/11/2010		Cardiovascular	Normal		
S195	214	JAIVAC-1	Dosage B	2	Day 42			11/11/2010		Respiratory	Normal		
S195	214	JAIVAC-1	Dosage B	2	Day 42			11/11/2010		Musculoskeletal	Normal		
S195	214	JAIVAC-1	Dosage B	2	Day 42			11/11/2010		Venous system	Normal		
S195	214	JAIVAC-1	Dosage B	2	Day 42			11/11/2010		Gastrointestinal	Normal		
S195	214	JAIVAC-1	Dosage B	2	Day 42			11/11/2010		Lymphatic	Normal		
S195	214	JAIVAC-1	Dosage B	2	Day 42			11/11/2010		Neurological	Normal		
S195	214	JAIVAC-1	Dosage B	2	Day 42			11/11/2010		Genitourinary	Normal		
S195	214	JAIVAC-1	Dosage B	2	Day 42			11/11/2010		Dermatological	Normal		
S195	214	JAIVAC-1	Dosage B	2	Day 56			24/11/2010		Head and Neck	Normal		
S195	214	JAIVAC-1	Dosage B	2	Day 56			24/11/2010		Eyes	Normal		
S195	214	JAIVAC-1	Dosage B	2	Day 56			24/11/2010		Ears, nose and throat	Normal		
S195	214	JAIVAC-1	Dosage B	2	Day 56			24/11/2010		Cardiovascular	Normal		
S195	214	JAIVAC-1	Dosage B	2	Day 56			24/11/2010		Respiratory	Normal		
S195	214	JAIVAC-1	Dosage B	2	Day 56			24/11/2010		Musculoskeletal	Normal		
S195	214	JAIVAC-1	Dosage B	2	Day 56			24/11/2010		Venous system	Normal		
S195	214	JAIVAC-1	Dosage B	2	Day 56			24/11/2010		Gastrointestinal	Normal		
S195	214	JAIVAC-1	Dosage B	2	Day 56			24/11/2010		Lymphatic	Normal		

Subject No.	Randomisation Number	Study Group	Dosage	Dose	Day	Not Done	Specify	Date of Assessment	*Was the assessment performed prior to vaccination?	Physical Examination Parameter	Result	Abnormality	Clinical Significance
S195	214	JAIVAC-1	Dosage B	2	Day 56			24/11/2010		Neurological	Normal		
S195	214	JAIVAC-1	Dosage B	2	Day 56			24/11/2010		Genitourinary	Normal		
S195	214	JAIVAC-1	Dosage B	2	Day 56			24/11/2010		Dermatological	Normal		
S195	214	JAIVAC-1	Dosage B	3	Day 180			29/03/2011	Yes	Head and Neck	Normal		
S195	214	JAIVAC-1	Dosage B	3	Day 180			29/03/2011	Yes	Eyes	Normal		
S195	214	JAIVAC-1	Dosage B	3	Day 180			29/03/2011	Yes	Ears, nose and throat	Normal		
S195	214	JAIVAC-1	Dosage B	3	Day 180			29/03/2011	Yes	Cardiovascular	Normal		
S195	214	JAIVAC-1	Dosage B	3	Day 180			29/03/2011	Yes	Respiratory	Normal		
S195	214	JAIVAC-1	Dosage B	3	Day 180			29/03/2011	Yes	Musculoskeletal	Normal		
S195	214	JAIVAC-1	Dosage B	3	Day 180			29/03/2011	Yes	Venous system	Normal		
S195	214	JAIVAC-1	Dosage B	3	Day 180			29/03/2011	Yes	Gastrointestinal	Normal		
S195	214	JAIVAC-1	Dosage B	3	Day 180			29/03/2011	Yes	Lymphatic	Normal		
S195	214	JAIVAC-1	Dosage B	3	Day 180			29/03/2011	Yes	Neurological	Normal		
S195	214	JAIVAC-1	Dosage B	3	Day 180			29/03/2011	Yes	Genitourinary	Normal		
S195	214	JAIVAC-1	Dosage B	3	Day 180			29/03/2011	Yes	Dermatological	Normal		
S195	214	JAIVAC-1	Dosage B	3	Day 187			05/04/2011		Head and Neck	Normal		
S195	214	JAIVAC-1	Dosage B	3	Day 187			05/04/2011		Eyes	Normal		
S195	214	JAIVAC-1	Dosage B	3	Day 187			05/04/2011		Ears, nose and throat	Normal		
S195	214	JAIVAC-1	Dosage B	3	Day 187			05/04/2011		Cardiovascular	Normal		

Subject No.	Randomisation Number	Study Group	Dosage	Dose	Day	Not Done	Specify	Date of Assessment	*Was the assessment performed prior to vaccination?	Physical Examination Parameter	Result	Abnormality	Clinical Significance
S195	214	JAIVAC-1	Dosage B	3	Day 187			05/04/2011		Respiratory	Normal		
S195	214	JAIVAC-1	Dosage B	3	Day 187			05/04/2011		Musculoskeletal	Normal		
S195	214	JAIVAC-1	Dosage B	3	Day 187			05/04/2011		Venous system	Normal		
S195	214	JAIVAC-1	Dosage B	3	Day 187			05/04/2011		Gastrointestinal	Normal		
S195	214	JAIVAC-1	Dosage B	3	Day 187			05/04/2011		Lymphatic	Normal		
S195	214	JAIVAC-1	Dosage B	3	Day 187			05/04/2011		Neurological	Normal		
S195	214	JAIVAC-1	Dosage B	3	Day 187			05/04/2011		Genitourinary	Normal		
S195	214	JAIVAC-1	Dosage B	3	Day 187			05/04/2011		Dermatological	Normal		
S195	214	JAIVAC-1	Dosage B	3	Day 194			12/04/2011		Head and Neck	Normal		
S195	214	JAIVAC-1	Dosage B	3	Day 194			12/04/2011		Eyes	Normal		
S195	214	JAIVAC-1	Dosage B	3	Day 194			12/04/2011		Ears, nose and throat	Normal		
S195	214	JAIVAC-1	Dosage B	3	Day 194			12/04/2011		Cardiovascular	Normal		
S195	214	JAIVAC-1	Dosage B	3	Day 194			12/04/2011		Respiratory	Normal		
S195	214	JAIVAC-1	Dosage B	3	Day 194			12/04/2011		Musculoskeletal	Normal		
S195	214	JAIVAC-1	Dosage B	3	Day 194			12/04/2011		Venous system	Normal		
S195	214	JAIVAC-1	Dosage B	3	Day 194			12/04/2011		Gastrointestinal	Normal		
S195	214	JAIVAC-1	Dosage B	3	Day 194			12/04/2011		Lymphatic	Normal		
S195	214	JAIVAC-1	Dosage B	3	Day 194			12/04/2011		Neurological	Normal		
S195	214	JAIVAC-1	Dosage B	3	Day 194			12/04/2011		Genitourinary	Normal		

Subject No.	Randomisation Number	Study Group	Dosage	Dose	Day	Not Done	Specify	Date of Assessment	*Was the assessment performed prior to vaccination?	Physical Examination Parameter	Result	Abnormality	Clinical Significance
S195	214	JAIVAC-1	Dosage B	3	Day 194			12/04/2011		Dermatological	Normal		
S195	214	JAIVAC-1	Dosage B	3	Day 208			26/04/2011		Head and Neck	Normal		
S195	214	JAIVAC-1	Dosage B	3	Day 208			26/04/2011		Eyes	Normal		
S195	214	JAIVAC-1	Dosage B	3	Day 208			26/04/2011		Ears, nose and throat	Normal		
S195	214	JAIVAC-1	Dosage B	3	Day 208			26/04/2011		Cardiovascular	Normal		
S195	214	JAIVAC-1	Dosage B	3	Day 208			26/04/2011		Respiratory	Normal		
S195	214	JAIVAC-1	Dosage B	3	Day 208			26/04/2011		Musculoskeletal	Normal		
S195	214	JAIVAC-1	Dosage B	3	Day 208			26/04/2011		Venous system	Normal		
S195	214	JAIVAC-1	Dosage B	3	Day 208			26/04/2011		Gastrointestinal	Normal		
S195	214	JAIVAC-1	Dosage B	3	Day 208			26/04/2011		Lymphatic	Normal		
S195	214	JAIVAC-1	Dosage B	3	Day 208			26/04/2011		Neurological	Normal		
S195	214	JAIVAC-1	Dosage B	3	Day 208			26/04/2011		Genitourinary	Normal		
S195	214	JAIVAC-1	Dosage B	3	Day 208			26/04/2011		Dermatological	Normal		
S195	214	JAIVAC-1	Dosage B	3	Day 365			29/09/2011		Head and Neck	Normal		
S195	214	JAIVAC-1	Dosage B	3	Day 365			29/09/2011		Eyes	Normal		
S195	214	JAIVAC-1	Dosage B	3	Day 365			29/09/2011		Ears, nose and throat	Normal		
S195	214	JAIVAC-1	Dosage B	3	Day 365			29/09/2011		Cardiovascular	Normal		
S195	214	JAIVAC-1	Dosage B	3	Day 365			29/09/2011		Respiratory	Normal		
S195	214	JAIVAC-1	Dosage B	3	Day 365			29/09/2011		Musculoskeletal	Normal		

Subject No.	Randomisation Number	Study Group	Dosage	Dose	Day	Not Done	Specify	Date of Assessment	*Was the assessment performed prior to vaccination?	Physical Examination Parameter	Result	Abnormality	Clinical Significance
S195	214	JAIVAC-1	Dosage B	3	Day 365			29/09/2011		Venous system	Normal		
S195	214	JAIVAC-1	Dosage B	3	Day 365			29/09/2011		Gastrointestinal	Normal		
S195	214	JAIVAC-1	Dosage B	3	Day 365			29/09/2011		Lymphatic	Normal		
S195	214	JAIVAC-1	Dosage B	3	Day 365			29/09/2011		Neurological	Normal		
S195	214	JAIVAC-1	Dosage B	3	Day 365			29/09/2011		Genitourinary	Normal		
S195	214	JAIVAC-1	Dosage B	3	Day 365			29/09/2011		Dermatological	Normal		
S202	206	JAIVAC-1	Dosage B		Day -14 to Day -1			23/09/2010		Head and neck	Normal		
S202	206	JAIVAC-1	Dosage B		Day -14 to Day -1			23/09/2010		Eyes	Normal		
S202	206	JAIVAC-1	Dosage B		Day -14 to Day -1			23/09/2010		Ears, nose and throat	Normal		
S202	206	JAIVAC-1	Dosage B		Day -14 to Day -1			23/09/2010		Cardiovascular	Normal		
S202	206	JAIVAC-1	Dosage B		Day -14 to Day -1			23/09/2010		Respiratory	Normal		
S202	206	JAIVAC-1	Dosage B		Day -14 to Day -1			23/09/2010		Musculoskeletal	Normal		
S202	206	JAIVAC-1	Dosage B		Day -14 to Day -1			23/09/2010		Venous system	Normal		
S202	206	JAIVAC-1	Dosage B		Day -14 to Day -1			23/09/2010		Gastrointestinal	Normal		
S202	206	JAIVAC-1	Dosage B		Day -14 to Day -1			23/09/2010		Lymphatic	Normal		
S202	206	JAIVAC-1	Dosage B		Day -14 to Day -1			23/09/2010		Neurological	Normal		
S202	206	JAIVAC-1	Dosage B		Day -14 to Day -1			23/09/2010		Genitourinary	Normal		

Subject No.	Randomisation Number	Study Group	Dosage	Dose	Day	Not Done	Specify	Date of Assessment	*Was the assessment performed prior to vaccination?	Physical Examination Parameter	Result	Abnormality	Clinical Significance
S202	206	JAIVAC-1	Dosage B		Day -14 to Day -1			23/09/2010		Dermatological	Normal		
S202	206	JAIVAC-1	Dosage B	1	Day 0			29/09/2010	Yes	Head and neck	Normal		
S202	206	JAIVAC-1	Dosage B	1	Day 0			29/09/2010	Yes	Eyes	Normal		
S202	206	JAIVAC-1	Dosage B	1	Day 0			29/09/2010	Yes	Ears, nose and throat	Normal		
S202	206	JAIVAC-1	Dosage B	1	Day 0			29/09/2010	Yes	Cardiovascular	Normal		
S202	206	JAIVAC-1	Dosage B	1	Day 0			29/09/2010	Yes	Respiratory	Normal		
S202	206	JAIVAC-1	Dosage B	1	Day 0			29/09/2010	Yes	Musculoskeletal	Normal		
S202	206	JAIVAC-1	Dosage B	1	Day 0			29/09/2010	Yes	Venous system	Normal		
S202	206	JAIVAC-1	Dosage B	1	Day 0			29/09/2010	Yes	Gastrointestinal	Normal		
S202	206	JAIVAC-1	Dosage B	1	Day 0			29/09/2010	Yes	Lymphatic	Normal		
S202	206	JAIVAC-1	Dosage B	1	Day 0			29/09/2010	Yes	Neurological	Normal		
S202	206	JAIVAC-1	Dosage B	1	Day 0			29/09/2010	Yes	Genitourinary	Normal		
S202	206	JAIVAC-1	Dosage B	1	Day 0			29/09/2010	Yes	Dermatological	Normal		
S202	206	JAIVAC-1	Dosage B	1	Day 7			07/10/2010		Head and Neck	Normal		
S202	206	JAIVAC-1	Dosage B	1	Day 7			07/10/2010		Eyes	Normal		
S202	206	JAIVAC-1	Dosage B	1	Day 7			07/10/2010		Ears, nose and throat	Normal		
S202	206	JAIVAC-1	Dosage B	1	Day 7			07/10/2010		Cardiovascular	Normal		
S202	206	JAIVAC-1	Dosage B	1	Day 7			07/10/2010		Respiratory	Normal		
S202	206	JAIVAC-1	Dosage B	1	Day 7			07/10/2010		Musculoskeletal	Normal		

Subject No.	Randomisation Number	Study Group	Dosage	Dose	Day	Not Done	Specify	Date of Assessment	*Was the assessment performed prior to vaccination?	Physical Examination Parameter	Result	Abnormality	Clinical Significance
S202	206	JAIVAC-1	Dosage B	1	Day 7			07/10/2010		Venous system	Normal		
S202	206	JAIVAC-1	Dosage B	1	Day 7			07/10/2010		Gastrointestinal	Normal		
S202	206	JAIVAC-1	Dosage B	1	Day 7			07/10/2010		Lymphatic	Normal		
S202	206	JAIVAC-1	Dosage B	1	Day 7			07/10/2010		Neurological	Normal		
S202	206	JAIVAC-1	Dosage B	1	Day 7			07/10/2010		Genitourinary	Normal		
S202	206	JAIVAC-1	Dosage B	1	Day 7			07/10/2010		Dermatological	Normal		
S202	206	JAIVAC-1	Dosage B	1	Day 14			13/10/2010		Head and Neck	Normal		
S202	206	JAIVAC-1	Dosage B	1	Day 14			13/10/2010		Eyes	Normal		
S202	206	JAIVAC-1	Dosage B	1	Day 14			13/10/2010		Ears, nose and throat	Normal		
S202	206	JAIVAC-1	Dosage B	1	Day 14			13/10/2010		Cardiovascular	Normal		
S202	206	JAIVAC-1	Dosage B	1	Day 14			13/10/2010		Respiratory	Normal		
S202	206	JAIVAC-1	Dosage B	1	Day 14			13/10/2010		Musculoskeletal	Normal		
S202	206	JAIVAC-1	Dosage B	1	Day 14			13/10/2010		Venous system	Normal		
S202	206	JAIVAC-1	Dosage B	1	Day 14			13/10/2010		Gastrointestinal	Normal		
S202	206	JAIVAC-1	Dosage B	1	Day 14			13/10/2010		Lymphatic	Normal		
S202	206	JAIVAC-1	Dosage B	1	Day 14			13/10/2010		Neurological	Normal		
S202	206	JAIVAC-1	Dosage B	1	Day 14			13/10/2010		Genitourinary	Normal		
S202	206	JAIVAC-1	Dosage B	1	Day 14			13/10/2010		Dermatological	Normal		
S202	206	JAIVAC-1	Dosage B	2	Day 28			27/10/2010	Yes	Head and Neck	Normal		

Subject No.	Randomisation Number	Study Group	Dosage	Dose	Day	Not Done	Specify	Date of Assessment	*Was the assessment performed prior to vaccination?	Physical Examination Parameter	Result	Abnormality	Clinical Significance
S202	206	JAIVAC-1	Dosage B	2	Day 28			27/10/2010	Yes	Eyes	Normal		
S202	206	JAIVAC-1	Dosage B	2	Day 28			27/10/2010	Yes	Ears, nose and throat	Normal		
S202	206	JAIVAC-1	Dosage B	2	Day 28			27/10/2010	Yes	Cardiovascular	Normal		
S202	206	JAIVAC-1	Dosage B	2	Day 28			27/10/2010	Yes	Respiratory	Normal		
S202	206	JAIVAC-1	Dosage B	2	Day 28			27/10/2010	Yes	Musculoskeletal	Normal		
S202	206	JAIVAC-1	Dosage B	2	Day 28			27/10/2010	Yes	Venous system	Normal		
S202	206	JAIVAC-1	Dosage B	2	Day 28			27/10/2010	Yes	Gastrointestinal	Normal		
S202	206	JAIVAC-1	Dosage B	2	Day 28			27/10/2010	Yes	Lymphatic	Normal		
S202	206	JAIVAC-1	Dosage B	2	Day 28			27/10/2010	Yes	Neurological	Normal		
S202	206	JAIVAC-1	Dosage B	2	Day 28			27/10/2010	Yes	Genitourinary	Normal		
S202	206	JAIVAC-1	Dosage B	2	Day 28			27/10/2010	Yes	Dermatological	Normal		
S202	206	JAIVAC-1	Dosage B	2	Day 35			03/11/2010		Head and Neck	Normal		
S202	206	JAIVAC-1	Dosage B	2	Day 35			03/11/2010		Eyes	Normal		
S202	206	JAIVAC-1	Dosage B	2	Day 35			03/11/2010		Ears, nose and throat	Normal		
S202	206	JAIVAC-1	Dosage B	2	Day 35			03/11/2010		Cardiovascular	Normal		
S202	206	JAIVAC-1	Dosage B	2	Day 35			03/11/2010		Respiratory	Normal		
S202	206	JAIVAC-1	Dosage B	2	Day 35			03/11/2010		Musculoskeletal	Normal		
S202	206	JAIVAC-1	Dosage B	2	Day 35			03/11/2010		Venous system	Normal		
S202	206	JAIVAC-1	Dosage B	2	Day 35			03/11/2010		Gastrointestinal	Normal		

Subject No.	Randomisation Number	Study Group	Dosage	Dose	Day	Not Done	Specify	Date of Assessment	*Was the assessment performed prior to vaccination?	Physical Examination Parameter	Result	Abnormality	Clinical Significance
S202	206	JAIVAC-1	Dosage B	2	Day 35			03/11/2010		Lymphatic	Normal		
S202	206	JAIVAC-1	Dosage B	2	Day 35			03/11/2010		Neurological	Normal		
S202	206	JAIVAC-1	Dosage B	2	Day 35			03/11/2010		Genitourinary	Normal		
S202	206	JAIVAC-1	Dosage B	2	Day 35			03/11/2010		Dermatological	Normal		
S202	206	JAIVAC-1	Dosage B	2	Day 42			10/11/2010		Head and Neck	Normal		
S202	206	JAIVAC-1	Dosage B	2	Day 42			10/11/2010		Eyes	Normal		
S202	206	JAIVAC-1	Dosage B	2	Day 42			10/11/2010		Ears, nose and throat	Normal		
S202	206	JAIVAC-1	Dosage B	2	Day 42			10/11/2010		Cardiovascular	Normal		
S202	206	JAIVAC-1	Dosage B	2	Day 42			10/11/2010		Respiratory	Normal		
S202	206	JAIVAC-1	Dosage B	2	Day 42			10/11/2010		Musculoskeletal	Normal		
S202	206	JAIVAC-1	Dosage B	2	Day 42			10/11/2010		Venous system	Normal		
S202	206	JAIVAC-1	Dosage B	2	Day 42			10/11/2010		Gastrointestinal	Normal		
S202	206	JAIVAC-1	Dosage B	2	Day 42			10/11/2010		Lymphatic	Normal		
S202	206	JAIVAC-1	Dosage B	2	Day 42			10/11/2010		Neurological	Normal		
S202	206	JAIVAC-1	Dosage B	2	Day 42			10/11/2010		Genitourinary	Normal		
S202	206	JAIVAC-1	Dosage B	2	Day 42			10/11/2010		Dermatological	Normal		
S202	206	JAIVAC-1	Dosage B	2	Day 56			24/11/2010		Head and Neck	Normal		
S202	206	JAIVAC-1	Dosage B	2	Day 56			24/11/2010		Eyes	Normal		
S202	206	JAIVAC-1	Dosage B	2	Day 56			24/11/2010		Ears, nose and throat	Normal		

Subject No.	Randomisation Number	Study Group	Dosage	Dose	Day	Not Done	Specify	Date of Assessment	*Was the assessment performed prior to vaccination?	Physical Examination Parameter	Result	Abnormality	Clinical Significance
S202	206	JAIVAC-1	Dosage B	2	Day 56			24/11/2010		Cardiovascular	Normal		
S202	206	JAIVAC-1	Dosage B	2	Day 56			24/11/2010		Respiratory	Normal		
S202	206	JAIVAC-1	Dosage B	2	Day 56			24/11/2010		Musculoskeletal	Normal		
S202	206	JAIVAC-1	Dosage B	2	Day 56			24/11/2010		Venous system	Normal		
S202	206	JAIVAC-1	Dosage B	2	Day 56			24/11/2010		Gastrointestinal	Normal		
S202	206	JAIVAC-1	Dosage B	2	Day 56			24/11/2010		Lymphatic	Normal		
S202	206	JAIVAC-1	Dosage B	2	Day 56			24/11/2010		Neurological	Normal		
S202	206	JAIVAC-1	Dosage B	2	Day 56			24/11/2010		Genitourinary	Normal		
S202	206	JAIVAC-1	Dosage B	2	Day 56			24/11/2010		Dermatological	Normal		
S202	206	JAIVAC-1	Dosage B	3	Day 180			28/03/2011	Yes	Head and Neck	Normal		
S202	206	JAIVAC-1	Dosage B	3	Day 180			28/03/2011	Yes	Eyes	Normal		
S202	206	JAIVAC-1	Dosage B	3	Day 180			28/03/2011	Yes	Ears, nose and throat	Normal		
S202	206	JAIVAC-1	Dosage B	3	Day 180			28/03/2011	Yes	Cardiovascular	Normal		
S202	206	JAIVAC-1	Dosage B	3	Day 180			28/03/2011	Yes	Respiratory	Normal		
S202	206	JAIVAC-1	Dosage B	3	Day 180			28/03/2011	Yes	Musculoskeletal	Normal		
S202	206	JAIVAC-1	Dosage B	3	Day 180			28/03/2011	Yes	Venous system	Normal		
S202	206	JAIVAC-1	Dosage B	3	Day 180			28/03/2011	Yes	Gastrointestinal	Normal		
S202	206	JAIVAC-1	Dosage B	3	Day 180			28/03/2011	Yes	Lymphatic	Normal		
S202	206	JAIVAC-1	Dosage B	3	Day 180			28/03/2011	Yes	Neurological	Normal		

Subject No.	Randomisation Number	Study Group	Dosage	Dose	Day	Not Done	Specify	Date of Assessment	*Was the assessment performed prior to vaccination?	Physical Examination Parameter	Result	Abnormality	Clinical Significance
S202	206	JAIVAC-1	Dosage B	3	Day 180			28/03/2011	Yes	Genitourinary	Normal		
S202	206	JAIVAC-1	Dosage B	3	Day 180			28/03/2011	Yes	Dermatological	Normal		
S202	206	JAIVAC-1	Dosage B	3	Day 187			05/04/2011		Head and Neck	Normal		
S202	206	JAIVAC-1	Dosage B	3	Day 187			05/04/2011		Eyes	Normal		
S202	206	JAIVAC-1	Dosage B	3	Day 187			05/04/2011		Ears, nose and throat	Normal		
S202	206	JAIVAC-1	Dosage B	3	Day 187			05/04/2011		Cardiovascular	Normal		
S202	206	JAIVAC-1	Dosage B	3	Day 187			05/04/2011		Respiratory	Normal		
S202	206	JAIVAC-1	Dosage B	3	Day 187			05/04/2011		Musculoskeletal	Normal		
S202	206	JAIVAC-1	Dosage B	3	Day 187			05/04/2011		Venous system	Normal		
S202	206	JAIVAC-1	Dosage B	3	Day 187			05/04/2011		Gastrointestinal	Normal		
S202	206	JAIVAC-1	Dosage B	3	Day 187			05/04/2011		Lymphatic	Normal		
S202	206	JAIVAC-1	Dosage B	3	Day 187			05/04/2011		Neurological	Normal		
S202	206	JAIVAC-1	Dosage B	3	Day 187			05/04/2011		Genitourinary	Normal		
S202	206	JAIVAC-1	Dosage B	3	Day 187			05/04/2011		Dermatological	Normal		
S202	206	JAIVAC-1	Dosage B	3	Day 194			11/04/2011		Head and Neck	Normal		
S202	206	JAIVAC-1	Dosage B	3	Day 194			11/04/2011		Eyes	Normal		
S202	206	JAIVAC-1	Dosage B	3	Day 194			11/04/2011		Ears, nose and throat	Normal		
S202	206	JAIVAC-1	Dosage B	3	Day 194			11/04/2011		Cardiovascular	Normal		
S202	206	JAIVAC-1	Dosage B	3	Day 194			11/04/2011		Respiratory	Normal		

Subject No.	Randomisation Number	Study Group	Dosage	Dose	Day	Not Done	Specify	Date of Assessment	*Was the assessment performed prior to vaccination?	Physical Examination Parameter	Result	Abnormality	Clinical Significance
S202	206	JAIVAC-1	Dosage B	3	Day 194			11/04/2011		Musculoskeletal	Normal		
S202	206	JAIVAC-1	Dosage B	3	Day 194			11/04/2011		Venous system	Normal		
S202	206	JAIVAC-1	Dosage B	3	Day 194			11/04/2011		Gastrointestinal	Normal		
S202	206	JAIVAC-1	Dosage B	3	Day 194			11/04/2011		Lymphatic	Normal		
S202	206	JAIVAC-1	Dosage B	3	Day 194			11/04/2011		Neurological	Normal		
S202	206	JAIVAC-1	Dosage B	3	Day 194			11/04/2011		Genitourinary	Normal		
S202	206	JAIVAC-1	Dosage B	3	Day 194			11/04/2011		Dermatological	Normal		
S202	206	JAIVAC-1	Dosage B	3	Day 208			25/04/2011		Head and Neck	Normal		
S202	206	JAIVAC-1	Dosage B	3	Day 208			25/04/2011		Eyes	Normal		
S202	206	JAIVAC-1	Dosage B	3	Day 208			25/04/2011		Ears, nose and throat	Normal		
S202	206	JAIVAC-1	Dosage B	3	Day 208			25/04/2011		Cardiovascular	Normal		
S202	206	JAIVAC-1	Dosage B	3	Day 208			25/04/2011		Respiratory	Normal		
S202	206	JAIVAC-1	Dosage B	3	Day 208			25/04/2011		Musculoskeletal	Normal		
S202	206	JAIVAC-1	Dosage B	3	Day 208			25/04/2011		Venous system	Normal		
S202	206	JAIVAC-1	Dosage B	3	Day 208			25/04/2011		Gastrointestinal	Normal		
S202	206	JAIVAC-1	Dosage B	3	Day 208			25/04/2011		Lymphatic	Normal		
S202	206	JAIVAC-1	Dosage B	3	Day 208			25/04/2011		Neurological	Normal		
S202	206	JAIVAC-1	Dosage B	3	Day 208			25/04/2011		Genitourinary	Normal		
S202	206	JAIVAC-1	Dosage B	3	Day 208			25/04/2011		Dermatological	Normal		

Subject No.	Randomisation Number	Study Group	Dosage	Dose	Day	Not Done	Specify	Date of Assessment	*Was the assessment performed prior to vaccination?	Physical Examination Parameter	Result	Abnormality	Clinical Significance
S202	206	JAIVAC-1	Dosage B	3	Day 365			29/09/2011		Head and Neck	Normal		
S202	206	JAIVAC-1	Dosage B	3	Day 365			29/09/2011		Eyes	Normal		
S202	206	JAIVAC-1	Dosage B	3	Day 365			29/09/2011		Ears, nose and throat	Normal		
S202	206	JAIVAC-1	Dosage B	3	Day 365			29/09/2011		Cardiovascular	Normal		
S202	206	JAIVAC-1	Dosage B	3	Day 365			29/09/2011		Respiratory	Normal		
S202	206	JAIVAC-1	Dosage B	3	Day 365			29/09/2011		Musculoskeletal	Normal		
S202	206	JAIVAC-1	Dosage B	3	Day 365			29/09/2011		Venous system	Normal		
S202	206	JAIVAC-1	Dosage B	3	Day 365			29/09/2011		Gastrointestinal	Normal		
S202	206	JAIVAC-1	Dosage B	3	Day 365			29/09/2011		Lymphatic	Normal		
S202	206	JAIVAC-1	Dosage B	3	Day 365			29/09/2011		Neurological	Normal		
S202	206	JAIVAC-1	Dosage B	3	Day 365			29/09/2011		Genitourinary	Normal		
S202	206	JAIVAC-1	Dosage B	3	Day 365			29/09/2011		Dermatological	Normal		
S203	211	Hepatitis B	Hepatitis B		Day -14 to Day -1			24/09/2010		Head and neck	Normal		
S203	211	Hepatitis B	Hepatitis B		Day -14 to Day -1			24/09/2010		Eyes	Normal		
S203	211	Hepatitis B	Hepatitis B		Day -14 to Day -1			24/09/2010		Ears, nose and throat	Normal		
S203	211	Hepatitis B	Hepatitis B		Day -14 to Day -1			24/09/2010		Cardiovascular	Normal		
S203	211	Hepatitis B	Hepatitis B		Day -14 to Day -1			24/09/2010		Respiratory	Normal		
S203	211	Hepatitis B	Hepatitis B		Day -14 to Day -1			24/09/2010		Musculoskeletal	Normal		

Subject No.	Randomisation Number	Study Group	Dosage	Dose	Day	Not Done	Specify	Date of Assessment	*Was the assessment performed prior to vaccination?	Physical Examination Parameter	Result	Abnormality	Clinical Significance
S203	211	Hepatitis B	Hepatitis B		Day -14 to Day -1			24/09/2010		Venous system	Normal		
S203	211	Hepatitis B	Hepatitis B		Day -14 to Day -1			24/09/2010		Gastrointestinal	Normal		
S203	211	Hepatitis B	Hepatitis B		Day -14 to Day -1			24/09/2010		Lymphatic	Normal		
S203	211	Hepatitis B	Hepatitis B		Day -14 to Day -1			24/09/2010		Neurological	Normal		
S203	211	Hepatitis B	Hepatitis B		Day -14 to Day -1			24/09/2010		Genitourinary	Normal		
S203	211	Hepatitis B	Hepatitis B		Day -14 to Day -1			24/09/2010		Dermatological	Normal		
S203	211	Hepatitis B	Hepatitis B	1	Day 0			30/09/2010	Yes	Head and neck	Normal		
S203	211	Hepatitis B	Hepatitis B	1	Day 0			30/09/2010	Yes	Eyes	Normal		
S203	211	Hepatitis B	Hepatitis B	1	Day 0			30/09/2010	Yes	Ears, nose and throat	Normal		
S203	211	Hepatitis B	Hepatitis B	1	Day 0			30/09/2010	Yes	Cardiovascular	Normal		
S203	211	Hepatitis B	Hepatitis B	1	Day 0			30/09/2010	Yes	Respiratory	Normal		
S203	211	Hepatitis B	Hepatitis B	1	Day 0			30/09/2010	Yes	Musculoskeletal	Normal		
S203	211	Hepatitis B	Hepatitis B	1	Day 0			30/09/2010	Yes	Venous system	Normal		
S203	211	Hepatitis B	Hepatitis B	1	Day 0			30/09/2010	Yes	Gastrointestinal	Normal		
S203	211	Hepatitis B	Hepatitis B	1	Day 0			30/09/2010	Yes	Lymphatic	Normal		
S203	211	Hepatitis B	Hepatitis B	1	Day 0			30/09/2010	Yes	Neurological	Normal		
S203	211	Hepatitis B	Hepatitis B	1	Day 0			30/09/2010	Yes	Genitourinary	Normal		
S203	211	Hepatitis B	Hepatitis B	1	Day 0			30/09/2010	Yes	Dermatological	Normal		

Subject No.	Randomisation Number	Study Group	Dosage	Dose	Day	Not Done	Specify	Date of Assessment	*Was the assessment performed prior to vaccination?	Physical Examination Parameter	Result	Abnormality	Clinical Significance
S203	211	Hepatitis B	Hepatitis B	1	Day 7			07/10/2010		Head and Neck	Normal		
S203	211	Hepatitis B	Hepatitis B	1	Day 7			07/10/2010		Eyes	Normal		
S203	211	Hepatitis B	Hepatitis B	1	Day 7			07/10/2010		Ears, nose and throat	Normal		
S203	211	Hepatitis B	Hepatitis B	1	Day 7			07/10/2010		Cardiovascular	Normal		
S203	211	Hepatitis B	Hepatitis B	1	Day 7			07/10/2010		Respiratory	Normal		
S203	211	Hepatitis B	Hepatitis B	1	Day 7			07/10/2010		Musculoskeletal	Normal		
S203	211	Hepatitis B	Hepatitis B	1	Day 7			07/10/2010		Venous system	Normal		
S203	211	Hepatitis B	Hepatitis B	1	Day 7			07/10/2010		Gastrointestinal	Normal		
S203	211	Hepatitis B	Hepatitis B	1	Day 7			07/10/2010		Lymphatic	Normal		
S203	211	Hepatitis B	Hepatitis B	1	Day 7			07/10/2010		Neurological	Normal		
S203	211	Hepatitis B	Hepatitis B	1	Day 7			07/10/2010		Genitourinary	Normal		
S203	211	Hepatitis B	Hepatitis B	1	Day 7			07/10/2010		Dermatological	Normal		
S203	211	Hepatitis B	Hepatitis B	1	Day 14			14/10/2010		Head and Neck	Normal		
S203	211	Hepatitis B	Hepatitis B	1	Day 14			14/10/2010		Eyes	Normal		
S203	211	Hepatitis B	Hepatitis B	1	Day 14			14/10/2010		Ears, nose and throat	Normal		
S203	211	Hepatitis B	Hepatitis B	1	Day 14			14/10/2010		Cardiovascular	Normal		
S203	211	Hepatitis B	Hepatitis B	1	Day 14			14/10/2010		Respiratory	Normal		
S203	211	Hepatitis B	Hepatitis B	1	Day 14			14/10/2010		Musculoskeletal	Normal		
S203	211	Hepatitis B	Hepatitis B	1	Day 14			14/10/2010		Venous system	Normal		

Subject No.	Randomisation Number	Study Group	Dosage	Dose	Day	Not Done	Specify	Date of Assessment	*Was the assessment performed prior to vaccination?	Physical Examination Parameter	Result	Abnormality	Clinical Significance
S203	211	Hepatitis B	Hepatitis B	1	Day 14			14/10/2010		Gastrointestinal	Normal		
S203	211	Hepatitis B	Hepatitis B	1	Day 14			14/10/2010		Lymphatic	Normal		
S203	211	Hepatitis B	Hepatitis B	1	Day 14			14/10/2010		Neurological	Normal		
S203	211	Hepatitis B	Hepatitis B	1	Day 14			14/10/2010		Genitourinary	Normal		
S203	211	Hepatitis B	Hepatitis B	1	Day 14			14/10/2010		Dermatological	Normal		
S203	211	Hepatitis B	Hepatitis B	2	Day 28			28/10/2010	Yes	Head and Neck	Normal		
S203	211	Hepatitis B	Hepatitis B	2	Day 28			28/10/2010	Yes	Eyes	Normal		
S203	211	Hepatitis B	Hepatitis B	2	Day 28			28/10/2010	Yes	Ears, nose and throat	Normal		
S203	211	Hepatitis B	Hepatitis B	2	Day 28			28/10/2010	Yes	Cardiovascular	Normal		
S203	211	Hepatitis B	Hepatitis B	2	Day 28			28/10/2010	Yes	Respiratory	Normal		
S203	211	Hepatitis B	Hepatitis B	2	Day 28			28/10/2010	Yes	Musculoskeletal	Normal		
S203	211	Hepatitis B	Hepatitis B	2	Day 28			28/10/2010	Yes	Venous system	Normal		
S203	211	Hepatitis B	Hepatitis B	2	Day 28			28/10/2010	Yes	Gastrointestinal	Normal		
S203	211	Hepatitis B	Hepatitis B	2	Day 28			28/10/2010	Yes	Lymphatic	Normal		
S203	211	Hepatitis B	Hepatitis B	2	Day 28			28/10/2010	Yes	Neurological	Normal		
S203	211	Hepatitis B	Hepatitis B	2	Day 28			28/10/2010	Yes	Genitourinary	Normal		
S203	211	Hepatitis B	Hepatitis B	2	Day 28			28/10/2010	Yes	Dermatological	Normal		
S203	211	Hepatitis B	Hepatitis B	2	Day 35			04/11/2010		Head and Neck	Normal		
S203	211	Hepatitis B	Hepatitis B	2	Day 35			04/11/2010		Eyes	Normal		

Subject No.	Randomisation Number	Study Group	Dosage	Dose	Day	Not Done	Specify	Date of Assessment	*Was the assessment performed prior to vaccination?	Physical Examination Parameter	Result	Abnormality	Clinical Significance
S203	211	Hepatitis B	Hepatitis B	2	Day 35			04/11/2010		Ears, nose and throat	Normal		
S203	211	Hepatitis B	Hepatitis B	2	Day 35			04/11/2010		Cardiovascular	Normal		
S203	211	Hepatitis B	Hepatitis B	2	Day 35			04/11/2010		Respiratory	Normal		
S203	211	Hepatitis B	Hepatitis B	2	Day 35			04/11/2010		Musculoskeletal	Normal		
S203	211	Hepatitis B	Hepatitis B	2	Day 35			04/11/2010		Venous system	Normal		
S203	211	Hepatitis B	Hepatitis B	2	Day 35			04/11/2010		Gastrointestinal	Normal		
S203	211	Hepatitis B	Hepatitis B	2	Day 35			04/11/2010		Lymphatic	Normal		
S203	211	Hepatitis B	Hepatitis B	2	Day 35			04/11/2010		Neurological	Normal		
S203	211	Hepatitis B	Hepatitis B	2	Day 35			04/11/2010		Genitourinary	Normal		
S203	211	Hepatitis B	Hepatitis B	2	Day 35			04/11/2010		Dermatological	Normal		
S203	211	Hepatitis B	Hepatitis B	2	Day 42			11/11/2010		Head and Neck	Normal		
S203	211	Hepatitis B	Hepatitis B	2	Day 42			11/11/2010		Eyes	Normal		
S203	211	Hepatitis B	Hepatitis B	2	Day 42			11/11/2010		Ears, nose and throat	Normal		
S203	211	Hepatitis B	Hepatitis B	2	Day 42			11/11/2010		Cardiovascular	Normal		
S203	211	Hepatitis B	Hepatitis B	2	Day 42			11/11/2010		Respiratory	Normal		
S203	211	Hepatitis B	Hepatitis B	2	Day 42			11/11/2010		Musculoskeletal	Normal		
S203	211	Hepatitis B	Hepatitis B	2	Day 42			11/11/2010		Venous system	Normal		
S203	211	Hepatitis B	Hepatitis B	2	Day 42			11/11/2010		Gastrointestinal	Normal		
S203	211	Hepatitis B	Hepatitis B	2	Day 42			11/11/2010		Lymphatic	Normal		

Subject No.	Randomisation Number	Study Group	Dosage	Dose	Day	Not Done	Specify	Date of Assessment	*Was the assessment performed prior to vaccination?	Physical Examination Parameter	Result	Abnormality	Clinical Significance
S203	211	Hepatitis B	Hepatitis B	2	Day 42			11/11/2010		Neurological	Normal		
S203	211	Hepatitis B	Hepatitis B	2	Day 42			11/11/2010		Genitourinary	Normal		
S203	211	Hepatitis B	Hepatitis B	2	Day 42			11/11/2010		Dermatological	Normal		
S203	211	Hepatitis B	Hepatitis B	2	Day 56			25/11/2010		Head and Neck	Normal		
S203	211	Hepatitis B	Hepatitis B	2	Day 56			25/11/2010		Eyes	Normal		
S203	211	Hepatitis B	Hepatitis B	2	Day 56			25/11/2010		Ears, nose and throat	Normal		
S203	211	Hepatitis B	Hepatitis B	2	Day 56			25/11/2010		Cardiovascular	Normal		
S203	211	Hepatitis B	Hepatitis B	2	Day 56			25/11/2010		Respiratory	Normal		
S203	211	Hepatitis B	Hepatitis B	2	Day 56			25/11/2010		Musculoskeletal	Normal		
S203	211	Hepatitis B	Hepatitis B	2	Day 56			25/11/2010		Venous system	Normal		
S203	211	Hepatitis B	Hepatitis B	2	Day 56			25/11/2010		Gastrointestinal	Normal		
S203	211	Hepatitis B	Hepatitis B	2	Day 56			25/11/2010		Lymphatic	Normal		
S203	211	Hepatitis B	Hepatitis B	2	Day 56			25/11/2010		Neurological	Normal		
S203	211	Hepatitis B	Hepatitis B	2	Day 56			25/11/2010		Genitourinary	Normal		
S203	211	Hepatitis B	Hepatitis B	2	Day 56			25/11/2010		Dermatological	Normal		
S203	211	Hepatitis B	Hepatitis B	3	Day 180			29/03/2011	Yes	Head and Neck	Normal		
S203	211	Hepatitis B	Hepatitis B	3	Day 180			29/03/2011	Yes	Eyes	Normal		
S203	211	Hepatitis B	Hepatitis B	3	Day 180			29/03/2011	Yes	Ears, nose and throat	Normal		
S203	211	Hepatitis B	Hepatitis B	3	Day 180			29/03/2011	Yes	Cardiovascular	Normal		

Subject No.	Randomisation Number	Study Group	Dosage	Dose	Day	Not Done	Specify	Date of Assessment	*Was the assessment performed prior to vaccination?	Physical Examination Parameter	Result	Abnormality	Clinical Significance
S203	211	Hepatitis B	Hepatitis B	3	Day 180			29/03/2011	Yes	Respiratory	Normal		
S203	211	Hepatitis B	Hepatitis B	3	Day 180			29/03/2011	Yes	Musculoskeletal	Normal		
S203	211	Hepatitis B	Hepatitis B	3	Day 180			29/03/2011	Yes	Venous system	Normal		
S203	211	Hepatitis B	Hepatitis B	3	Day 180			29/03/2011	Yes	Gastrointestinal	Normal		
S203	211	Hepatitis B	Hepatitis B	3	Day 180			29/03/2011	Yes	Lymphatic	Normal		
S203	211	Hepatitis B	Hepatitis B	3	Day 180			29/03/2011	Yes	Neurological	Normal		
S203	211	Hepatitis B	Hepatitis B	3	Day 180			29/03/2011	Yes	Genitourinary	Normal		
S203	211	Hepatitis B	Hepatitis B	3	Day 180			29/03/2011	Yes	Dermatological	Normal		
S203	211	Hepatitis B	Hepatitis B	3	Day 187			05/04/2011		Head and Neck	Normal		
S203	211	Hepatitis B	Hepatitis B	3	Day 187			05/04/2011		Eyes	Normal		
S203	211	Hepatitis B	Hepatitis B	3	Day 187			05/04/2011		Ears, nose and throat	Normal		
S203	211	Hepatitis B	Hepatitis B	3	Day 187			05/04/2011		Cardiovascular	Normal		
S203	211	Hepatitis B	Hepatitis B	3	Day 187			05/04/2011		Respiratory	Normal		
S203	211	Hepatitis B	Hepatitis B	3	Day 187			05/04/2011		Musculoskeletal	Normal		
S203	211	Hepatitis B	Hepatitis B	3	Day 187			05/04/2011		Venous system	Normal		
S203	211	Hepatitis B	Hepatitis B	3	Day 187			05/04/2011		Gastrointestinal	Normal		
S203	211	Hepatitis B	Hepatitis B	3	Day 187			05/04/2011		Lymphatic	Normal		
S203	211	Hepatitis B	Hepatitis B	3	Day 187			05/04/2011		Neurological	Normal		
S203	211	Hepatitis B	Hepatitis B	3	Day 187			05/04/2011		Genitourinary	Normal		

Subject No.	Randomisation Number	Study Group	Dosage	Dose	Day	Not Done	Specify	Date of Assessment	*Was the assessment performed prior to vaccination?	Physical Examination Parameter	Result	Abnormality	Clinical Significance
S203	211	Hepatitis B	Hepatitis B	3	Day 187			05/04/2011		Dermatological	Normal		
S203	211	Hepatitis B	Hepatitis B	3	Day 194			12/04/2011		Head and Neck	Normal		
S203	211	Hepatitis B	Hepatitis B	3	Day 194			12/04/2011		Eyes	Normal		
S203	211	Hepatitis B	Hepatitis B	3	Day 194			12/04/2011		Ears, nose and throat	Normal		
S203	211	Hepatitis B	Hepatitis B	3	Day 194			12/04/2011		Cardiovascular	Normal		
S203	211	Hepatitis B	Hepatitis B	3	Day 194			12/04/2011		Respiratory	Normal		
S203	211	Hepatitis B	Hepatitis B	3	Day 194			12/04/2011		Musculoskeletal	Normal		
S203	211	Hepatitis B	Hepatitis B	3	Day 194			12/04/2011		Venous system	Normal		
S203	211	Hepatitis B	Hepatitis B	3	Day 194			12/04/2011		Gastrointestinal	Normal		
S203	211	Hepatitis B	Hepatitis B	3	Day 194			12/04/2011		Lymphatic	Normal		
S203	211	Hepatitis B	Hepatitis B	3	Day 194			12/04/2011		Neurological	Normal		
S203	211	Hepatitis B	Hepatitis B	3	Day 194			12/04/2011		Genitourinary	Normal		
S203	211	Hepatitis B	Hepatitis B	3	Day 194			12/04/2011		Dermatological	Normal		
S203	211	Hepatitis B	Hepatitis B	3	Day 208			28/04/2011		Head and Neck	Normal		
S203	211	Hepatitis B	Hepatitis B	3	Day 208			28/04/2011		Eyes	Normal		
S203	211	Hepatitis B	Hepatitis B	3	Day 208			28/04/2011		Ears, nose and throat	Normal		
S203	211	Hepatitis B	Hepatitis B	3	Day 208			28/04/2011		Cardiovascular	Normal		
S203	211	Hepatitis B	Hepatitis B	3	Day 208			28/04/2011		Respiratory	Normal		
S203	211	Hepatitis B	Hepatitis B	3	Day 208			28/04/2011		Musculoskeletal	Normal		

Subject No.	Randomisation Number	Study Group	Dosage	Dose	Day	Not Done	Specify	Date of Assessment	*Was the assessment performed prior to vaccination?	Physical Examination Parameter	Result	Abnormality	Clinical Significance
S203	211	Hepatitis B	Hepatitis B	3	Day 208			28/04/2011		Venous system	Normal		
S203	211	Hepatitis B	Hepatitis B	3	Day 208			28/04/2011		Gastrointestinal	Normal		
S203	211	Hepatitis B	Hepatitis B	3	Day 208			28/04/2011		Lymphatic	Normal		
S203	211	Hepatitis B	Hepatitis B	3	Day 208			28/04/2011		Neurological	Normal		
S203	211	Hepatitis B	Hepatitis B	3	Day 208			28/04/2011		Genitourinary	Normal		
S203	211	Hepatitis B	Hepatitis B	3	Day 208			28/04/2011		Dermatological	Normal		
S203	211	Hepatitis B	Hepatitis B	3	Day 365			29/09/2011		Head and Neck	Normal		
S203	211	Hepatitis B	Hepatitis B	3	Day 365			29/09/2011		Eyes	Normal		
S203	211	Hepatitis B	Hepatitis B	3	Day 365			29/09/2011		Ears, nose and throat	Normal		
S203	211	Hepatitis B	Hepatitis B	3	Day 365			29/09/2011		Cardiovascular	Normal		
S203	211	Hepatitis B	Hepatitis B	3	Day 365			29/09/2011		Respiratory	Normal		
S203	211	Hepatitis B	Hepatitis B	3	Day 365			29/09/2011		Musculoskeletal	Normal		
S203	211	Hepatitis B	Hepatitis B	3	Day 365			29/09/2011		Venous system	Normal		
S203	211	Hepatitis B	Hepatitis B	3	Day 365			29/09/2011		Gastrointestinal	Normal		
S203	211	Hepatitis B	Hepatitis B	3	Day 365			29/09/2011		Lymphatic	Normal		
S203	211	Hepatitis B	Hepatitis B	3	Day 365			29/09/2011		Neurological	Normal		
S203	211	Hepatitis B	Hepatitis B	3	Day 365			29/09/2011		Genitourinary	Normal		
S203	211	Hepatitis B	Hepatitis B	3	Day 365			29/09/2011		Dermatological	Normal		

Subject No.	Randomisation Number	Study Group	Dosage	Dose	Day	Not Done	Specify	Date of Assessment	*Was the assessment performed prior to vaccination?	Physical Examination Parameter	Result	Abnormality	Clinical Significance
S203	211	Hepatitis B	Hepatitis B		Unscheduled Visit	Yes	Repeat For Laboratory Parameters	.		Head and Neck			
S203	211	Hepatitis B	Hepatitis B		Unscheduled Visit	Yes	Repeat For Laboratory Parameters	.		Eyes			
S203	211	Hepatitis B	Hepatitis B		Unscheduled Visit	Yes	Repeat For Laboratory Parameters	.		Ears, nose and throat			
S203	211	Hepatitis B	Hepatitis B		Unscheduled Visit	Yes	Repeat For Laboratory Parameters	.		Cardiovascular			
S203	211	Hepatitis B	Hepatitis B		Unscheduled Visit	Yes	Repeat For Laboratory Parameters	.		Respiratory			
S203	211	Hepatitis B	Hepatitis B		Unscheduled Visit	Yes	Repeat For Laboratory Parameters	.		Musculoskeletal			
S203	211	Hepatitis B	Hepatitis B		Unscheduled Visit	Yes	Repeat For Laboratory Parameters	.		Venous system			
S203	211	Hepatitis B	Hepatitis B		Unscheduled Visit	Yes	Repeat For Laboratory Parameters	.		Gastrointestinal			
S203	211	Hepatitis B	Hepatitis B		Unscheduled Visit	Yes	Repeat For Laboratory Parameters	.		Lymphatic			
S203	211	Hepatitis B	Hepatitis B		Unscheduled Visit	Yes	Repeat For Laboratory Parameters	.		Neurological			
S203	211	Hepatitis B	Hepatitis B		Unscheduled Visit	Yes	Repeat For Laboratory Parameters	.		Genitourinary			
S203	211	Hepatitis B	Hepatitis B		Unscheduled Visit	Yes	Repeat For Laboratory Parameters	.		Dermatological			

Subject No.	Randomisation Number	Study Group	Dosage	Dose	Day	Not Done	Specify	Date of Assessment	*Was the assessment performed prior to vaccination?	Physical Examination Parameter	Result	Abnormality	Clinical Significance
S203	211	Hepatitis B	Hepatitis B		Unscheduled Visit	Yes	Repeat For Laboratory Parameter	.		Head and Neck			
S203	211	Hepatitis B	Hepatitis B		Unscheduled Visit	Yes	Repeat For Laboratory Parameter	.		Eyes			
S203	211	Hepatitis B	Hepatitis B		Unscheduled Visit	Yes	Repeat For Laboratory Parameter	.		Ears, nose and throat			
S203	211	Hepatitis B	Hepatitis B		Unscheduled Visit	Yes	Repeat For Laboratory Parameter	.		Cardiovascular			
S203	211	Hepatitis B	Hepatitis B		Unscheduled Visit	Yes	Repeat For Laboratory Parameter	.		Respiratory			
S203	211	Hepatitis B	Hepatitis B		Unscheduled Visit	Yes	Repeat For Laboratory Parameter	.		Musculoskeletal			
S203	211	Hepatitis B	Hepatitis B		Unscheduled Visit	Yes	Repeat For Laboratory Parameter	.		Venous system			
S203	211	Hepatitis B	Hepatitis B		Unscheduled Visit	Yes	Repeat For Laboratory Parameter	.		Gastrointestinal			
S203	211	Hepatitis B	Hepatitis B		Unscheduled Visit	Yes	Repeat For Laboratory Parameter	.		Lymphatic			
S203	211	Hepatitis B	Hepatitis B		Unscheduled Visit	Yes	Repeat For Laboratory Parameter	.		Neurological			
S203	211	Hepatitis B	Hepatitis B		Unscheduled Visit	Yes	Repeat For Laboratory Parameter	.		Genitourinary			
S203	211	Hepatitis B	Hepatitis B		Unscheduled Visit	Yes	Repeat For Laboratory Parameter	.		Dermatological			

Subject No.	Randomisation Number	Study Group	Dosage	Dose	Day	Not Done	Specify	Date of Assessment	*Was the assessment performed prior to vaccination?	Physical Examination Parameter	Result	Abnormality	Clinical Significance
S203	211	Hepatitis B	Hepatitis B		Unscheduled Visit	Yes	Laboratory Repeat(Local)	.		Head and Neck			
S203	211	Hepatitis B	Hepatitis B		Unscheduled Visit	Yes	Laboratory Repeat(Local)	.		Eyes			
S203	211	Hepatitis B	Hepatitis B		Unscheduled Visit	Yes	Laboratory Repeat(Local)	.		Ears, nose and throat			
S203	211	Hepatitis B	Hepatitis B		Unscheduled Visit	Yes	Laboratory Repeat(Local)	.		Cardiovascular			
S203	211	Hepatitis B	Hepatitis B		Unscheduled Visit	Yes	Laboratory Repeat(Local)	.		Respiratory			
S203	211	Hepatitis B	Hepatitis B		Unscheduled Visit	Yes	Laboratory Repeat(Local)	.		Musculoskeletal			
S203	211	Hepatitis B	Hepatitis B		Unscheduled Visit	Yes	Laboratory Repeat(Local)	.		Venous system			
S203	211	Hepatitis B	Hepatitis B		Unscheduled Visit	Yes	Laboratory Repeat(Local)	.		Gastrointestinal			
S203	211	Hepatitis B	Hepatitis B		Unscheduled Visit	Yes	Laboratory Repeat(Local)	.		Lymphatic			
S203	211	Hepatitis B	Hepatitis B		Unscheduled Visit	Yes	Laboratory Repeat(Local)	.		Neurological			
S203	211	Hepatitis B	Hepatitis B		Unscheduled Visit	Yes	Laboratory Repeat(Local)	.		Genitourinary			
S203	211	Hepatitis B	Hepatitis B		Unscheduled Visit	Yes	Laboratory Repeat(Local)	.		Dermatological			

Subject No.	Randomisation Number	Study Group	Dosage	Dose	Day	Not Done	Specify	Date of Assessment	*Was the assessment performed prior to vaccination?	Physical Examination Parameter	Result	Abnormality	Clinical Significance
S212	212	JAIVAC-1	Dosage B		Day -14 to Day -1			23/09/2010		Head and neck	Normal		
S212	212	JAIVAC-1	Dosage B		Day -14 to Day -1			23/09/2010		Eyes	Normal		
S212	212	JAIVAC-1	Dosage B		Day -14 to Day -1			23/09/2010		Ears, nose and throat	Normal		
S212	212	JAIVAC-1	Dosage B		Day -14 to Day -1			23/09/2010		Cardiovascular	Normal		
S212	212	JAIVAC-1	Dosage B		Day -14 to Day -1			23/09/2010		Respiratory	Normal		
S212	212	JAIVAC-1	Dosage B		Day -14 to Day -1			23/09/2010		Musculoskeletal	Normal		
S212	212	JAIVAC-1	Dosage B		Day -14 to Day -1			23/09/2010		Venous system	Normal		
S212	212	JAIVAC-1	Dosage B		Day -14 to Day -1			23/09/2010		Gastrointestinal	Normal		
S212	212	JAIVAC-1	Dosage B		Day -14 to Day -1			23/09/2010		Lymphatic	Normal		
S212	212	JAIVAC-1	Dosage B		Day -14 to Day -1			23/09/2010		Neurological	Normal		
S212	212	JAIVAC-1	Dosage B		Day -14 to Day -1			23/09/2010		Genitourinary	Normal		
S212	212	JAIVAC-1	Dosage B		Day -14 to Day -1			23/09/2010		Dermatological	Normal		
S212	212	JAIVAC-1	Dosage B	1	Day 0			30/09/2010	Yes	Head and neck	Normal		
S212	212	JAIVAC-1	Dosage B	1	Day 0			30/09/2010	Yes	Eyes	Normal		
S212	212	JAIVAC-1	Dosage B	1	Day 0			30/09/2010	Yes	Ears, nose and throat	Normal		
S212	212	JAIVAC-1	Dosage B	1	Day 0			30/09/2010	Yes	Cardiovascular	Normal		
S212	212	JAIVAC-1	Dosage B	1	Day 0			30/09/2010	Yes	Respiratory	Normal		

Subject No.	Randomisation Number	Study Group	Dosage	Dose	Day	Not Done	Specify	Date of Assessment	*Was the assessment performed prior to vaccination?	Physical Examination Parameter	Result	Abnormality	Clinical Significance
S212	212	JAIVAC-1	Dosage B	1	Day 0			30/09/2010	Yes	Musculoskeletal	Normal		
S212	212	JAIVAC-1	Dosage B	1	Day 0			30/09/2010	Yes	Venous system	Normal		
S212	212	JAIVAC-1	Dosage B	1	Day 0			30/09/2010	Yes	Gastrointestinal	Normal		
S212	212	JAIVAC-1	Dosage B	1	Day 0			30/09/2010	Yes	Lymphatic	Normal		
S212	212	JAIVAC-1	Dosage B	1	Day 0			30/09/2010	Yes	Neurological	Normal		
S212	212	JAIVAC-1	Dosage B	1	Day 0			30/09/2010	Yes	Genitourinary	Normal		
S212	212	JAIVAC-1	Dosage B	1	Day 0			30/09/2010	Yes	Dermatological	Normal		
S212	212	JAIVAC-1	Dosage B	1	Day 7			07/10/2010		Head and Neck	Normal		
S212	212	JAIVAC-1	Dosage B	1	Day 7			07/10/2010		Eyes	Normal		
S212	212	JAIVAC-1	Dosage B	1	Day 7			07/10/2010		Ears, nose and throat	Normal		
S212	212	JAIVAC-1	Dosage B	1	Day 7			07/10/2010		Cardiovascular	Normal		
S212	212	JAIVAC-1	Dosage B	1	Day 7			07/10/2010		Respiratory	Normal		
S212	212	JAIVAC-1	Dosage B	1	Day 7			07/10/2010		Musculoskeletal	Normal		
S212	212	JAIVAC-1	Dosage B	1	Day 7			07/10/2010		Venous system	Normal		
S212	212	JAIVAC-1	Dosage B	1	Day 7			07/10/2010		Gastrointestinal	Normal		
S212	212	JAIVAC-1	Dosage B	1	Day 7			07/10/2010		Lymphatic	Normal		
S212	212	JAIVAC-1	Dosage B	1	Day 7			07/10/2010		Neurological	Normal		
S212	212	JAIVAC-1	Dosage B	1	Day 7			07/10/2010		Genitourinary	Normal		
S212	212	JAIVAC-1	Dosage B	1	Day 7			07/10/2010		Dermatological	Normal		

Subject No.	Randomisation Number	Study Group	Dosage	Dose	Day	Not Done	Specify	Date of Assessment	*Was the assessment performed prior to vaccination?	Physical Examination Parameter	Result	Abnormality	Clinical Significance
S212	212	JAIVAC-1	Dosage B	1	Day 14			14/10/2010		Head and Neck	Normal		
S212	212	JAIVAC-1	Dosage B	1	Day 14			14/10/2010		Eyes	Normal		
S212	212	JAIVAC-1	Dosage B	1	Day 14			14/10/2010		Ears, nose and throat	Normal		
S212	212	JAIVAC-1	Dosage B	1	Day 14			14/10/2010		Cardiovascular	Normal		
S212	212	JAIVAC-1	Dosage B	1	Day 14			14/10/2010		Respiratory	Normal		
S212	212	JAIVAC-1	Dosage B	1	Day 14			14/10/2010		Musculoskeletal	Normal		
S212	212	JAIVAC-1	Dosage B	1	Day 14			14/10/2010		Venous system	Normal		
S212	212	JAIVAC-1	Dosage B	1	Day 14			14/10/2010		Gastrointestinal	Normal		
S212	212	JAIVAC-1	Dosage B	1	Day 14			14/10/2010		Lymphatic	Normal		
S212	212	JAIVAC-1	Dosage B	1	Day 14			14/10/2010		Neurological	Normal		
S212	212	JAIVAC-1	Dosage B	1	Day 14			14/10/2010		Genitourinary	Normal		
S212	212	JAIVAC-1	Dosage B	1	Day 14			14/10/2010		Dermatological	Normal		
S212	212	JAIVAC-1	Dosage B	2	Day 28			28/10/2010	Yes	Head and Neck	Normal		
S212	212	JAIVAC-1	Dosage B	2	Day 28			28/10/2010	Yes	Eyes	Normal		
S212	212	JAIVAC-1	Dosage B	2	Day 28			28/10/2010	Yes	Ears, nose and throat	Normal		
S212	212	JAIVAC-1	Dosage B	2	Day 28			28/10/2010	Yes	Cardiovascular	Normal		
S212	212	JAIVAC-1	Dosage B	2	Day 28			28/10/2010	Yes	Respiratory	Normal		
S212	212	JAIVAC-1	Dosage B	2	Day 28			28/10/2010	Yes	Musculoskeletal	Normal		
S212	212	JAIVAC-1	Dosage B	2	Day 28			28/10/2010	Yes	Venous system	Normal		

Subject No.	Randomisation Number	Study Group	Dosage	Dose	Day	Not Done	Specify	Date of Assessment	*Was the assessment performed prior to vaccination?	Physical Examination Parameter	Result	Abnormality	Clinical Significance
S212	212	JAIVAC-1	Dosage B	2	Day 28			28/10/2010	Yes	Gastrointestinal	Normal		
S212	212	JAIVAC-1	Dosage B	2	Day 28			28/10/2010	Yes	Lymphatic	Normal		
S212	212	JAIVAC-1	Dosage B	2	Day 28			28/10/2010	Yes	Neurological	Normal		
S212	212	JAIVAC-1	Dosage B	2	Day 28			28/10/2010	Yes	Genitourinary	Normal		
S212	212	JAIVAC-1	Dosage B	2	Day 28			28/10/2010	Yes	Dermatological	Normal		
S212	212	JAIVAC-1	Dosage B	2	Day 35			04/11/2010		Head and Neck	Normal		
S212	212	JAIVAC-1	Dosage B	2	Day 35			04/11/2010		Eyes	Normal		
S212	212	JAIVAC-1	Dosage B	2	Day 35			04/11/2010		Ears, nose and throat	Normal		
S212	212	JAIVAC-1	Dosage B	2	Day 35			04/11/2010		Cardiovascular	Normal		
S212	212	JAIVAC-1	Dosage B	2	Day 35			04/11/2010		Respiratory	Normal		
S212	212	JAIVAC-1	Dosage B	2	Day 35			04/11/2010		Musculoskeletal	Normal		
S212	212	JAIVAC-1	Dosage B	2	Day 35			04/11/2010		Venous system	Normal		
S212	212	JAIVAC-1	Dosage B	2	Day 35			04/11/2010		Gastrointestinal	Normal		
S212	212	JAIVAC-1	Dosage B	2	Day 35			04/11/2010		Lymphatic	Normal		
S212	212	JAIVAC-1	Dosage B	2	Day 35			04/11/2010		Neurological	Normal		
S212	212	JAIVAC-1	Dosage B	2	Day 35			04/11/2010		Genitourinary	Normal		
S212	212	JAIVAC-1	Dosage B	2	Day 35			04/11/2010		Dermatological	Normal		
S212	212	JAIVAC-1	Dosage B	2	Day 42			11/11/2010		Head and Neck	Normal		
S212	212	JAIVAC-1	Dosage B	2	Day 42			11/11/2010		Eyes	Normal		

Subject No.	Randomisation Number	Study Group	Dosage	Dose	Day	Not Done	Specify	Date of Assessment	*Was the assessment performed prior to vaccination?	Physical Examination Parameter	Result	Abnormality	Clinical Significance
S212	212	JAIVAC-1	Dosage B	2	Day 42			11/11/2010		Ears, nose and throat	Normal		
S212	212	JAIVAC-1	Dosage B	2	Day 42			11/11/2010		Cardiovascular	Normal		
S212	212	JAIVAC-1	Dosage B	2	Day 42			11/11/2010		Respiratory	Normal		
S212	212	JAIVAC-1	Dosage B	2	Day 42			11/11/2010		Musculoskeletal	Normal		
S212	212	JAIVAC-1	Dosage B	2	Day 42			11/11/2010		Venous system	Normal		
S212	212	JAIVAC-1	Dosage B	2	Day 42			11/11/2010		Gastrointestinal	Normal		
S212	212	JAIVAC-1	Dosage B	2	Day 42			11/11/2010		Lymphatic	Normal		
S212	212	JAIVAC-1	Dosage B	2	Day 42			11/11/2010		Neurological	Normal		
S212	212	JAIVAC-1	Dosage B	2	Day 42			11/11/2010		Genitourinary	Normal		
S212	212	JAIVAC-1	Dosage B	2	Day 42			11/11/2010		Dermatological	Normal		
S212	212	JAIVAC-1	Dosage B	2	Day 56			25/11/2010		Head and Neck	Normal		
S212	212	JAIVAC-1	Dosage B	2	Day 56			25/11/2010		Eyes	Normal		
S212	212	JAIVAC-1	Dosage B	2	Day 56			25/11/2010		Ears, nose and throat	Normal		
S212	212	JAIVAC-1	Dosage B	2	Day 56			25/11/2010		Cardiovascular	Normal		
S212	212	JAIVAC-1	Dosage B	2	Day 56			25/11/2010		Respiratory	Normal		
S212	212	JAIVAC-1	Dosage B	2	Day 56			25/11/2010		Musculoskeletal	Normal		
S212	212	JAIVAC-1	Dosage B	2	Day 56			25/11/2010		Venous system	Normal		
S212	212	JAIVAC-1	Dosage B	2	Day 56			25/11/2010		Gastrointestinal	Normal		
S212	212	JAIVAC-1	Dosage B	2	Day 56			25/11/2010		Lymphatic	Normal		

Subject No.	Randomisation Number	Study Group	Dosage	Dose	Day	Not Done	Specify	Date of Assessment	*Was the assessment performed prior to vaccination?	Physical Examination Parameter	Result	Abnormality	Clinical Significance
S212	212	JAIVAC-1	Dosage B	2	Day 56			25/11/2010		Neurological	Normal		
S212	212	JAIVAC-1	Dosage B	2	Day 56			25/11/2010		Genitourinary	Normal		
S212	212	JAIVAC-1	Dosage B	2	Day 56			25/11/2010		Dermatological	Normal		
S212	212	JAIVAC-1	Dosage B	3	Day 180			29/03/2011	Yes	Head and Neck	Normal		
S212	212	JAIVAC-1	Dosage B	3	Day 180			29/03/2011	Yes	Eyes	Normal		
S212	212	JAIVAC-1	Dosage B	3	Day 180			29/03/2011	Yes	Ears, nose and throat	Normal		
S212	212	JAIVAC-1	Dosage B	3	Day 180			29/03/2011	Yes	Cardiovascular	Normal		
S212	212	JAIVAC-1	Dosage B	3	Day 180			29/03/2011	Yes	Respiratory	Normal		
S212	212	JAIVAC-1	Dosage B	3	Day 180			29/03/2011	Yes	Musculoskeletal	Normal		
S212	212	JAIVAC-1	Dosage B	3	Day 180			29/03/2011	Yes	Venous system	Normal		
S212	212	JAIVAC-1	Dosage B	3	Day 180			29/03/2011	Yes	Gastrointestinal	Normal		
S212	212	JAIVAC-1	Dosage B	3	Day 180			29/03/2011	Yes	Lymphatic	Normal		
S212	212	JAIVAC-1	Dosage B	3	Day 180			29/03/2011	Yes	Neurological	Normal		
S212	212	JAIVAC-1	Dosage B	3	Day 180			29/03/2011	Yes	Genitourinary	Normal		
S212	212	JAIVAC-1	Dosage B	3	Day 180			29/03/2011	Yes	Dermatological	Normal		
S212	212	JAIVAC-1	Dosage B	3	Day 187			05/04/2011		Head and Neck	Normal		
S212	212	JAIVAC-1	Dosage B	3	Day 187			05/04/2011		Eyes	Normal		
S212	212	JAIVAC-1	Dosage B	3	Day 187			05/04/2011		Ears, nose and throat	Normal		
S212	212	JAIVAC-1	Dosage B	3	Day 187			05/04/2011		Cardiovascular	Normal		

Subject No.	Randomisation Number	Study Group	Dosage	Dose	Day	Not Done	Specify	Date of Assessment	*Was the assessment performed prior to vaccination?	Physical Examination Parameter	Result	Abnormality	Clinical Significance
S212	212	JAIVAC-1	Dosage B	3	Day 187			05/04/2011		Respiratory	Normal		
S212	212	JAIVAC-1	Dosage B	3	Day 187			05/04/2011		Musculoskeletal	Normal		
S212	212	JAIVAC-1	Dosage B	3	Day 187			05/04/2011		Venous system	Normal		
S212	212	JAIVAC-1	Dosage B	3	Day 187			05/04/2011		Gastrointestinal	Normal		
S212	212	JAIVAC-1	Dosage B	3	Day 187			05/04/2011		Lymphatic	Normal		
S212	212	JAIVAC-1	Dosage B	3	Day 187			05/04/2011		Neurological	Normal		
S212	212	JAIVAC-1	Dosage B	3	Day 187			05/04/2011		Genitourinary	Normal		
S212	212	JAIVAC-1	Dosage B	3	Day 187			05/04/2011		Dermatological	Normal		
S212	212	JAIVAC-1	Dosage B	3	Day 194			12/04/2011		Head and Neck	Normal		
S212	212	JAIVAC-1	Dosage B	3	Day 194			12/04/2011		Eyes	Normal		
S212	212	JAIVAC-1	Dosage B	3	Day 194			12/04/2011		Ears, nose and throat	Normal		
S212	212	JAIVAC-1	Dosage B	3	Day 194			12/04/2011		Cardiovascular	Normal		
S212	212	JAIVAC-1	Dosage B	3	Day 194			12/04/2011		Respiratory	Normal		
S212	212	JAIVAC-1	Dosage B	3	Day 194			12/04/2011		Musculoskeletal	Normal		
S212	212	JAIVAC-1	Dosage B	3	Day 194			12/04/2011		Venous system	Normal		
S212	212	JAIVAC-1	Dosage B	3	Day 194			12/04/2011		Gastrointestinal	Normal		
S212	212	JAIVAC-1	Dosage B	3	Day 194			12/04/2011		Lymphatic	Normal		
S212	212	JAIVAC-1	Dosage B	3	Day 194			12/04/2011		Neurological	Normal		
S212	212	JAIVAC-1	Dosage B	3	Day 194			12/04/2011		Genitourinary	Normal		

Subject No.	Randomisation Number	Study Group	Dosage	Dose	Day	Not Done	Specify	Date of Assessment	*Was the assessment performed prior to vaccination?	Physical Examination Parameter	Result	Abnormality	Clinical Significance
S212	212	JAIVAC-1	Dosage B	3	Day 194			12/04/2011		Dermatological	Normal		
S212	212	JAIVAC-1	Dosage B	3	Day 208			19/04/2011		Head and Neck	Normal		
S212	212	JAIVAC-1	Dosage B	3	Day 208			19/04/2011		Eyes	Normal		
S212	212	JAIVAC-1	Dosage B	3	Day 208			19/04/2011		Ears, nose and throat	Normal		
S212	212	JAIVAC-1	Dosage B	3	Day 208			19/04/2011		Cardiovascular	Normal		
S212	212	JAIVAC-1	Dosage B	3	Day 208			19/04/2011		Respiratory	Normal		
S212	212	JAIVAC-1	Dosage B	3	Day 208			19/04/2011		Musculoskeletal	Normal		
S212	212	JAIVAC-1	Dosage B	3	Day 208			19/04/2011		Venous system	Normal		
S212	212	JAIVAC-1	Dosage B	3	Day 208			19/04/2011		Gastrointestinal	Normal		
S212	212	JAIVAC-1	Dosage B	3	Day 208			19/04/2011		Lymphatic	Normal		
S212	212	JAIVAC-1	Dosage B	3	Day 208			19/04/2011		Neurological	Normal		
S212	212	JAIVAC-1	Dosage B	3	Day 208			19/04/2011		Genitourinary	Normal		
S212	212	JAIVAC-1	Dosage B	3	Day 208			19/04/2011		Dermatological	Normal		
S212	212	JAIVAC-1	Dosage B	3	Day 365			29/09/2011		Head and Neck	Normal		
S212	212	JAIVAC-1	Dosage B	3	Day 365			29/09/2011		Eyes	Normal		
S212	212	JAIVAC-1	Dosage B	3	Day 365			29/09/2011		Ears, nose and throat	Normal		
S212	212	JAIVAC-1	Dosage B	3	Day 365			29/09/2011		Cardiovascular	Normal		
S212	212	JAIVAC-1	Dosage B	3	Day 365			29/09/2011		Respiratory	Normal		
S212	212	JAIVAC-1	Dosage B	3	Day 365			29/09/2011		Musculoskeletal	Normal		

Subject No.	Randomisation Number	Study Group	Dosage	Dose	Day	Not Done	Specify	Date of Assessment	*Was the assessment performed prior to vaccination?	Physical Examination Parameter	Result	Abnormality	Clinical Significance
S212	212	JAIVAC-1	Dosage B	3	Day 365			29/09/2011		Venous system	Normal		
S212	212	JAIVAC-1	Dosage B	3	Day 365			29/09/2011		Gastrointestinal	Normal		
S212	212	JAIVAC-1	Dosage B	3	Day 365			29/09/2011		Lymphatic	Normal		
S212	212	JAIVAC-1	Dosage B	3	Day 365			29/09/2011		Neurological	Normal		
S212	212	JAIVAC-1	Dosage B	3	Day 365			29/09/2011		Genitourinary	Normal		
S212	212	JAIVAC-1	Dosage B	3	Day 365			29/09/2011		Dermatological	Normal		
S215	213	Hepatitis B	Hepatitis B		Day -14 to Day -1			24/09/2010		Head and neck	Normal		
S215	213	Hepatitis B	Hepatitis B		Day -14 to Day -1			24/09/2010		Eyes	Normal		
S215	213	Hepatitis B	Hepatitis B		Day -14 to Day -1			24/09/2010		Ears, nose and throat	Normal		
S215	213	Hepatitis B	Hepatitis B		Day -14 to Day -1			24/09/2010		Cardiovascular	Normal		
S215	213	Hepatitis B	Hepatitis B		Day -14 to Day -1			24/09/2010		Respiratory	Normal		
S215	213	Hepatitis B	Hepatitis B		Day -14 to Day -1			24/09/2010		Musculoskeletal	Normal		
S215	213	Hepatitis B	Hepatitis B		Day -14 to Day -1			24/09/2010		Venous system	Normal		
S215	213	Hepatitis B	Hepatitis B		Day -14 to Day -1			24/09/2010		Gastrointestinal	Normal		
S215	213	Hepatitis B	Hepatitis B		Day -14 to Day -1			24/09/2010		Lymphatic	Normal		
S215	213	Hepatitis B	Hepatitis B		Day -14 to Day -1			24/09/2010		Neurological	Normal		
S215	213	Hepatitis B	Hepatitis B		Day -14 to Day -1			24/09/2010		Genitourinary	Normal		

Subject No.	Randomisation Number	Study Group	Dosage	Dose	Day	Not Done	Specify	Date of Assessment	*Was the assessment performed prior to vaccination?	Physical Examination Parameter	Result	Abnormality	Clinical Significance
S215	213	Hepatitis B	Hepatitis B		Day -14 to Day -1			24/09/2010		Dermatological	Normal		
S215	213	Hepatitis B	Hepatitis B	1	Day 0			30/09/2010	Yes	Head and neck	Normal		
S215	213	Hepatitis B	Hepatitis B	1	Day 0			30/09/2010	Yes	Eyes	Normal		
S215	213	Hepatitis B	Hepatitis B	1	Day 0			30/09/2010	Yes	Ears, nose and throat	Normal		
S215	213	Hepatitis B	Hepatitis B	1	Day 0			30/09/2010	Yes	Cardiovascular	Normal		
S215	213	Hepatitis B	Hepatitis B	1	Day 0			30/09/2010	Yes	Respiratory	Normal		
S215	213	Hepatitis B	Hepatitis B	1	Day 0			30/09/2010	Yes	Musculoskeletal	Normal		
S215	213	Hepatitis B	Hepatitis B	1	Day 0			30/09/2010	Yes	Venous system	Normal		
S215	213	Hepatitis B	Hepatitis B	1	Day 0			30/09/2010	Yes	Gastrointestinal	Normal		
S215	213	Hepatitis B	Hepatitis B	1	Day 0			30/09/2010	Yes	Lymphatic	Normal		
S215	213	Hepatitis B	Hepatitis B	1	Day 0			30/09/2010	Yes	Neurological	Normal		
S215	213	Hepatitis B	Hepatitis B	1	Day 0			30/09/2010	Yes	Genitourinary	Normal		
S215	213	Hepatitis B	Hepatitis B	1	Day 0			30/09/2010	Yes	Dermatological	Normal		
S215	213	Hepatitis B	Hepatitis B	1	Day 7			07/10/2010		Head and Neck	Normal		
S215	213	Hepatitis B	Hepatitis B	1	Day 7			07/10/2010		Eyes	Normal		
S215	213	Hepatitis B	Hepatitis B	1	Day 7			07/10/2010		Ears, nose and throat	Normal		
S215	213	Hepatitis B	Hepatitis B	1	Day 7			07/10/2010		Cardiovascular	Normal		
S215	213	Hepatitis B	Hepatitis B	1	Day 7			07/10/2010		Respiratory	Normal		
S215	213	Hepatitis B	Hepatitis B	1	Day 7			07/10/2010		Musculoskeletal	Normal		

Subject No.	Randomisation Number	Study Group	Dosage	Dose	Day	Not Done	Specify	Date of Assessment	*Was the assessment performed prior to vaccination?	Physical Examination Parameter	Result	Abnormality	Clinical Significance
S215	213	Hepatitis B	Hepatitis B	1	Day 7			07/10/2010		Venous system	Normal		
S215	213	Hepatitis B	Hepatitis B	1	Day 7			07/10/2010		Gastrointestinal	Normal		
S215	213	Hepatitis B	Hepatitis B	1	Day 7			07/10/2010		Lymphatic	Normal		
S215	213	Hepatitis B	Hepatitis B	1	Day 7			07/10/2010		Neurological	Normal		
S215	213	Hepatitis B	Hepatitis B	1	Day 7			07/10/2010		Genitourinary	Normal		
S215	213	Hepatitis B	Hepatitis B	1	Day 7			07/10/2010		Dermatological	Normal		
S215	213	Hepatitis B	Hepatitis B	1	Day 14			14/10/2010		Head and Neck	Normal		
S215	213	Hepatitis B	Hepatitis B	1	Day 14			14/10/2010		Eyes	Normal		
S215	213	Hepatitis B	Hepatitis B	1	Day 14			14/10/2010		Ears, nose and throat	Normal		
S215	213	Hepatitis B	Hepatitis B	1	Day 14			14/10/2010		Cardiovascular	Normal		
S215	213	Hepatitis B	Hepatitis B	1	Day 14			14/10/2010		Respiratory	Normal		
S215	213	Hepatitis B	Hepatitis B	1	Day 14			14/10/2010		Musculoskeletal	Normal		
S215	213	Hepatitis B	Hepatitis B	1	Day 14			14/10/2010		Venous system	Normal		
S215	213	Hepatitis B	Hepatitis B	1	Day 14			14/10/2010		Gastrointestinal	Normal		
S215	213	Hepatitis B	Hepatitis B	1	Day 14			14/10/2010		Lymphatic	Normal		
S215	213	Hepatitis B	Hepatitis B	1	Day 14			14/10/2010		Neurological	Normal		
S215	213	Hepatitis B	Hepatitis B	1	Day 14			14/10/2010		Genitourinary	Normal		
S215	213	Hepatitis B	Hepatitis B	1	Day 14			14/10/2010		Dermatological	Normal		
S215	213	Hepatitis B	Hepatitis B	2	Day 28			28/10/2010	Yes	Head and Neck	Normal		

Subject No.	Randomisation Number	Study Group	Dosage	Dose	Day	Not Done	Specify	Date of Assessment	*Was the assessment performed prior to vaccination?	Physical Examination Parameter	Result	Abnormality	Clinical Significance
S215	213	Hepatitis B	Hepatitis B	2	Day 28			28/10/2010	Yes	Eyes	Normal		
S215	213	Hepatitis B	Hepatitis B	2	Day 28			28/10/2010	Yes	Ears, nose and throat	Normal		
S215	213	Hepatitis B	Hepatitis B	2	Day 28			28/10/2010	Yes	Cardiovascular	Normal		
S215	213	Hepatitis B	Hepatitis B	2	Day 28			28/10/2010	Yes	Respiratory	Normal		
S215	213	Hepatitis B	Hepatitis B	2	Day 28			28/10/2010	Yes	Musculoskeletal	Normal		
S215	213	Hepatitis B	Hepatitis B	2	Day 28			28/10/2010	Yes	Venous system	Normal		
S215	213	Hepatitis B	Hepatitis B	2	Day 28			28/10/2010	Yes	Gastrointestinal	Normal		
S215	213	Hepatitis B	Hepatitis B	2	Day 28			28/10/2010	Yes	Lymphatic	Normal		
S215	213	Hepatitis B	Hepatitis B	2	Day 28			28/10/2010	Yes	Neurological	Normal		
S215	213	Hepatitis B	Hepatitis B	2	Day 28			28/10/2010	Yes	Genitourinary	Normal		
S215	213	Hepatitis B	Hepatitis B	2	Day 28			28/10/2010	Yes	Dermatological	Normal		
S215	213	Hepatitis B	Hepatitis B	2	Day 35			04/11/2010		Head and Neck	Normal		
S215	213	Hepatitis B	Hepatitis B	2	Day 35			04/11/2010		Eyes	Normal		
S215	213	Hepatitis B	Hepatitis B	2	Day 35			04/11/2010		Ears, nose and throat	Normal		
S215	213	Hepatitis B	Hepatitis B	2	Day 35			04/11/2010		Cardiovascular	Normal		
S215	213	Hepatitis B	Hepatitis B	2	Day 35			04/11/2010		Respiratory	Normal		
S215	213	Hepatitis B	Hepatitis B	2	Day 35			04/11/2010		Musculoskeletal	Normal		
S215	213	Hepatitis B	Hepatitis B	2	Day 35			04/11/2010		Venous system	Normal		
S215	213	Hepatitis B	Hepatitis B	2	Day 35			04/11/2010		Gastrointestinal	Normal		

Subject No.	Randomisation Number	Study Group	Dosage	Dose	Day	Not Done	Specify	Date of Assessment	*Was the assessment performed prior to vaccination?	Physical Examination Parameter	Result	Abnormality	Clinical Significance
S215	213	Hepatitis B	Hepatitis B	2	Day 35			04/11/2010		Lymphatic	Normal		
S215	213	Hepatitis B	Hepatitis B	2	Day 35			04/11/2010		Neurological	Normal		
S215	213	Hepatitis B	Hepatitis B	2	Day 35			04/11/2010		Genitourinary	Normal		
S215	213	Hepatitis B	Hepatitis B	2	Day 35			04/11/2010		Dermatological	Normal		
S215	213	Hepatitis B	Hepatitis B	2	Day 42			11/11/2010		Head and Neck	Normal		
S215	213	Hepatitis B	Hepatitis B	2	Day 42			11/11/2010		Eyes	Normal		
S215	213	Hepatitis B	Hepatitis B	2	Day 42			11/11/2010		Ears, nose and throat	Normal		
S215	213	Hepatitis B	Hepatitis B	2	Day 42			11/11/2010		Cardiovascular	Normal		
S215	213	Hepatitis B	Hepatitis B	2	Day 42			11/11/2010		Respiratory	Normal		
S215	213	Hepatitis B	Hepatitis B	2	Day 42			11/11/2010		Musculoskeletal	Normal		
S215	213	Hepatitis B	Hepatitis B	2	Day 42			11/11/2010		Venous system	Normal		
S215	213	Hepatitis B	Hepatitis B	2	Day 42			11/11/2010		Gastrointestinal	Normal		
S215	213	Hepatitis B	Hepatitis B	2	Day 42			11/11/2010		Lymphatic	Normal		
S215	213	Hepatitis B	Hepatitis B	2	Day 42			11/11/2010		Neurological	Normal		
S215	213	Hepatitis B	Hepatitis B	2	Day 42			11/11/2010		Genitourinary	Normal		
S215	213	Hepatitis B	Hepatitis B	2	Day 42			11/11/2010		Dermatological	Normal		
S215	213	Hepatitis B	Hepatitis B	2	Day 56			25/11/2010		Head and Neck	Normal		
S215	213	Hepatitis B	Hepatitis B	2	Day 56			25/11/2010		Eyes	Normal		
S215	213	Hepatitis B	Hepatitis B	2	Day 56			25/11/2010		Ears, nose and throat	Normal		

Subject No.	Randomisation Number	Study Group	Dosage	Dose	Day	Not Done	Specify	Date of Assessment	*Was the assessment performed prior to vaccination?	Physical Examination Parameter	Result	Abnormality	Clinical Significance
S215	213	Hepatitis B	Hepatitis B	2	Day 56			25/11/2010		Cardiovascular	Normal		
S215	213	Hepatitis B	Hepatitis B	2	Day 56			25/11/2010		Respiratory	Normal		
S215	213	Hepatitis B	Hepatitis B	2	Day 56			25/11/2010		Musculoskeletal	Normal		
S215	213	Hepatitis B	Hepatitis B	2	Day 56			25/11/2010		Venous system	Normal		
S215	213	Hepatitis B	Hepatitis B	2	Day 56			25/11/2010		Gastrointestinal	Normal		
S215	213	Hepatitis B	Hepatitis B	2	Day 56			25/11/2010		Lymphatic	Normal		
S215	213	Hepatitis B	Hepatitis B	2	Day 56			25/11/2010		Neurological	Normal		
S215	213	Hepatitis B	Hepatitis B	2	Day 56			25/11/2010		Genitourinary	Normal		
S215	213	Hepatitis B	Hepatitis B	2	Day 56			25/11/2010		Dermatological	Normal		
S215	213	Hepatitis B	Hepatitis B	3	Day 180			29/03/2011	Yes	Head and Neck	Normal		
S215	213	Hepatitis B	Hepatitis B	3	Day 180			29/03/2011	Yes	Eyes	Normal		
S215	213	Hepatitis B	Hepatitis B	3	Day 180			29/03/2011	Yes	Ears, nose and throat	Normal		
S215	213	Hepatitis B	Hepatitis B	3	Day 180			29/03/2011	Yes	Cardiovascular	Normal		
S215	213	Hepatitis B	Hepatitis B	3	Day 180			29/03/2011	Yes	Respiratory	Normal		
S215	213	Hepatitis B	Hepatitis B	3	Day 180			29/03/2011	Yes	Musculoskeletal	Normal		
S215	213	Hepatitis B	Hepatitis B	3	Day 180			29/03/2011	Yes	Venous system	Normal		
S215	213	Hepatitis B	Hepatitis B	3	Day 180			29/03/2011	Yes	Gastrointestinal	Normal		
S215	213	Hepatitis B	Hepatitis B	3	Day 180			29/03/2011	Yes	Lymphatic	Normal		
S215	213	Hepatitis B	Hepatitis B	3	Day 180			29/03/2011	Yes	Neurological	Normal		

Subject No.	Randomisation Number	Study Group	Dosage	Dose	Day	Not Done	Specify	Date of Assessment	*Was the assessment performed prior to vaccination?	Physical Examination Parameter	Result	Abnormality	Clinical Significance
S215	213	Hepatitis B	Hepatitis B	3	Day 180			29/03/2011	Yes	Genitourinary	Normal		
S215	213	Hepatitis B	Hepatitis B	3	Day 180			29/03/2011	Yes	Dermatological	Normal		
S215	213	Hepatitis B	Hepatitis B	3	Day 187			06/04/2011		Head and Neck	Normal		
S215	213	Hepatitis B	Hepatitis B	3	Day 187			06/04/2011		Eyes	Normal		
S215	213	Hepatitis B	Hepatitis B	3	Day 187			06/04/2011		Ears, nose and throat	Normal		
S215	213	Hepatitis B	Hepatitis B	3	Day 187			06/04/2011		Cardiovascular	Normal		
S215	213	Hepatitis B	Hepatitis B	3	Day 187			06/04/2011		Respiratory	Normal		
S215	213	Hepatitis B	Hepatitis B	3	Day 187			06/04/2011		Musculoskeletal	Normal		
S215	213	Hepatitis B	Hepatitis B	3	Day 187			06/04/2011		Venous system	Normal		
S215	213	Hepatitis B	Hepatitis B	3	Day 187			06/04/2011		Gastrointestinal	Normal		
S215	213	Hepatitis B	Hepatitis B	3	Day 187			06/04/2011		Lymphatic	Normal		
S215	213	Hepatitis B	Hepatitis B	3	Day 187			06/04/2011		Neurological	Normal		
S215	213	Hepatitis B	Hepatitis B	3	Day 187			06/04/2011		Genitourinary	Normal		
S215	213	Hepatitis B	Hepatitis B	3	Day 187			06/04/2011		Dermatological	Normal		
S215	213	Hepatitis B	Hepatitis B	3	Day 194			12/04/2011		Head and Neck	Normal		
S215	213	Hepatitis B	Hepatitis B	3	Day 194			12/04/2011		Eyes	Normal		
S215	213	Hepatitis B	Hepatitis B	3	Day 194			12/04/2011		Ears, nose and throat	Normal		
S215	213	Hepatitis B	Hepatitis B	3	Day 194			12/04/2011		Cardiovascular	Normal		
S215	213	Hepatitis B	Hepatitis B	3	Day 194			12/04/2011		Respiratory	Normal		

Subject No.	Randomisation Number	Study Group	Dosage	Dose	Day	Not Done	Specify	Date of Assessment	*Was the assessment performed prior to vaccination?	Physical Examination Parameter	Result	Abnormality	Clinical Significance
S215	213	Hepatitis B	Hepatitis B	3	Day 194			12/04/2011		Musculoskeletal	Normal		
S215	213	Hepatitis B	Hepatitis B	3	Day 194			12/04/2011		Venous system	Normal		
S215	213	Hepatitis B	Hepatitis B	3	Day 194			12/04/2011		Gastrointestinal	Normal		
S215	213	Hepatitis B	Hepatitis B	3	Day 194			12/04/2011		Lymphatic	Normal		
S215	213	Hepatitis B	Hepatitis B	3	Day 194			12/04/2011		Neurological	Normal		
S215	213	Hepatitis B	Hepatitis B	3	Day 194			12/04/2011		Genitourinary	Normal		
S215	213	Hepatitis B	Hepatitis B	3	Day 194			12/04/2011		Dermatological	Normal		
S215	213	Hepatitis B	Hepatitis B	3	Day 208			26/04/2011		Head and Neck	Normal		
S215	213	Hepatitis B	Hepatitis B	3	Day 208			26/04/2011		Eyes	Normal		
S215	213	Hepatitis B	Hepatitis B	3	Day 208			26/04/2011		Ears, nose and throat	Normal		
S215	213	Hepatitis B	Hepatitis B	3	Day 208			26/04/2011		Cardiovascular	Normal		
S215	213	Hepatitis B	Hepatitis B	3	Day 208			26/04/2011		Respiratory	Normal		
S215	213	Hepatitis B	Hepatitis B	3	Day 208			26/04/2011		Musculoskeletal	Normal		
S215	213	Hepatitis B	Hepatitis B	3	Day 208			26/04/2011		Venous system	Normal		
S215	213	Hepatitis B	Hepatitis B	3	Day 208			26/04/2011		Gastrointestinal	Normal		
S215	213	Hepatitis B	Hepatitis B	3	Day 208			26/04/2011		Lymphatic	Normal		
S215	213	Hepatitis B	Hepatitis B	3	Day 208			26/04/2011		Neurological	Normal		
S215	213	Hepatitis B	Hepatitis B	3	Day 208			26/04/2011		Genitourinary	Normal		
S215	213	Hepatitis B	Hepatitis B	3	Day 208			26/04/2011		Dermatological	Normal		

Subject No.	Randomisation Number	Study Group	Dosage	Dose	Day	Not Done	Specify	Date of Assessment	*Was the assessment performed prior to vaccination?	Physical Examination Parameter	Result	Abnormality	Clinical Significance
S215	213	Hepatitis B	Hepatitis B	3	Day 365			29/09/2011		Head and Neck	Normal		
S215	213	Hepatitis B	Hepatitis B	3	Day 365			29/09/2011		Eyes	Normal		
S215	213	Hepatitis B	Hepatitis B	3	Day 365			29/09/2011		Ears, nose and throat	Normal		
S215	213	Hepatitis B	Hepatitis B	3	Day 365			29/09/2011		Cardiovascular	Normal		
S215	213	Hepatitis B	Hepatitis B	3	Day 365			29/09/2011		Respiratory	Normal		
S215	213	Hepatitis B	Hepatitis B	3	Day 365			29/09/2011		Musculoskeletal	Normal		
S215	213	Hepatitis B	Hepatitis B	3	Day 365			29/09/2011		Venous system	Normal		
S215	213	Hepatitis B	Hepatitis B	3	Day 365			29/09/2011		Gastrointestinal	Normal		
S215	213	Hepatitis B	Hepatitis B	3	Day 365			29/09/2011		Lymphatic	Normal		
S215	213	Hepatitis B	Hepatitis B	3	Day 365			29/09/2011		Neurological	Normal		
S215	213	Hepatitis B	Hepatitis B	3	Day 365			29/09/2011		Genitourinary	Normal		
S215	213	Hepatitis B	Hepatitis B	3	Day 365			29/09/2011		Dermatological	Normal		
S233	210	JAIVAC-1	Dosage B		Day -14 to Day -1			24/09/2010		Head and neck	Normal		
S233	210	JAIVAC-1	Dosage B		Day -14 to Day -1			24/09/2010		Eyes	Normal		
S233	210	JAIVAC-1	Dosage B		Day -14 to Day -1			24/09/2010		Ears, nose and throat	Normal		
S233	210	JAIVAC-1	Dosage B		Day -14 to Day -1			24/09/2010		Cardiovascular	Normal		
S233	210	JAIVAC-1	Dosage B		Day -14 to Day -1			24/09/2010		Respiratory	Normal		
S233	210	JAIVAC-1	Dosage B		Day -14 to Day -1			24/09/2010		Musculoskeletal	Normal		

Subject No.	Randomisation Number	Study Group	Dosage	Dose	Day	Not Done	Specify	Date of Assessment	*Was the assessment performed prior to vaccination?	Physical Examination Parameter	Result	Abnormality	Clinical Significance
S233	210	JAIVAC-1	Dosage B		Day -14 to Day -1			24/09/2010		Venous system	Normal		
S233	210	JAIVAC-1	Dosage B		Day -14 to Day -1			24/09/2010		Gastrointestinal	Normal		
S233	210	JAIVAC-1	Dosage B		Day -14 to Day -1			24/09/2010		Lymphatic	Normal		
S233	210	JAIVAC-1	Dosage B		Day -14 to Day -1			24/09/2010		Neurological	Normal		
S233	210	JAIVAC-1	Dosage B		Day -14 to Day -1			24/09/2010		Genitourinary	Normal		
S233	210	JAIVAC-1	Dosage B		Day -14 to Day -1			24/09/2010		Dermatological	Normal		
S233	210	JAIVAC-1	Dosage B	1	Day 0			30/09/2010	Yes	Head and neck	Normal		
S233	210	JAIVAC-1	Dosage B	1	Day 0			30/09/2010	Yes	Eyes	Normal		
S233	210	JAIVAC-1	Dosage B	1	Day 0			30/09/2010	Yes	Ears, nose and throat	Normal		
S233	210	JAIVAC-1	Dosage B	1	Day 0			30/09/2010	Yes	Cardiovascular	Normal		
S233	210	JAIVAC-1	Dosage B	1	Day 0			30/09/2010	Yes	Respiratory	Normal		
S233	210	JAIVAC-1	Dosage B	1	Day 0			30/09/2010	Yes	Musculoskeletal	Normal		
S233	210	JAIVAC-1	Dosage B	1	Day 0			30/09/2010	Yes	Venous system	Normal		
S233	210	JAIVAC-1	Dosage B	1	Day 0			30/09/2010	Yes	Gastrointestinal	Normal		
S233	210	JAIVAC-1	Dosage B	1	Day 0			30/09/2010	Yes	Lymphatic	Normal		
S233	210	JAIVAC-1	Dosage B	1	Day 0			30/09/2010	Yes	Neurological	Normal		
S233	210	JAIVAC-1	Dosage B	1	Day 0			30/09/2010	Yes	Genitourinary	Normal		
S233	210	JAIVAC-1	Dosage B	1	Day 0			30/09/2010	Yes	Dermatological	Normal		

Subject No.	Randomisation Number	Study Group	Dosage	Dose	Day	Not Done	Specify	Date of Assessment	*Was the assessment performed prior to vaccination?	Physical Examination Parameter	Result	Abnormality	Clinical Significance
S233	210	JAIVAC-1	Dosage B	1	Day 7			07/10/2010		Head and Neck	Normal		
S233	210	JAIVAC-1	Dosage B	1	Day 7			07/10/2010		Eyes	Normal		
S233	210	JAIVAC-1	Dosage B	1	Day 7			07/10/2010		Ears, nose and throat	Normal		
S233	210	JAIVAC-1	Dosage B	1	Day 7			07/10/2010		Cardiovascular	Normal		
S233	210	JAIVAC-1	Dosage B	1	Day 7			07/10/2010		Respiratory	Normal		
S233	210	JAIVAC-1	Dosage B	1	Day 7			07/10/2010		Musculoskeletal	Normal		
S233	210	JAIVAC-1	Dosage B	1	Day 7			07/10/2010		Venous system	Normal		
S233	210	JAIVAC-1	Dosage B	1	Day 7			07/10/2010		Gastrointestinal	Normal		
S233	210	JAIVAC-1	Dosage B	1	Day 7			07/10/2010		Lymphatic	Normal		
S233	210	JAIVAC-1	Dosage B	1	Day 7			07/10/2010		Neurological	Normal		
S233	210	JAIVAC-1	Dosage B	1	Day 7			07/10/2010		Genitourinary	Normal		
S233	210	JAIVAC-1	Dosage B	1	Day 7			07/10/2010		Dermatological	Normal		
S233	210	JAIVAC-1	Dosage B	1	Day 14			14/10/2010		Head and Neck	Normal		
S233	210	JAIVAC-1	Dosage B	1	Day 14			14/10/2010		Eyes	Normal		
S233	210	JAIVAC-1	Dosage B	1	Day 14			14/10/2010		Ears, nose and throat	Normal		
S233	210	JAIVAC-1	Dosage B	1	Day 14			14/10/2010		Cardiovascular	Normal		
S233	210	JAIVAC-1	Dosage B	1	Day 14			14/10/2010		Respiratory	Normal		
S233	210	JAIVAC-1	Dosage B	1	Day 14			14/10/2010		Musculoskeletal	Normal		
S233	210	JAIVAC-1	Dosage B	1	Day 14			14/10/2010		Venous system	Normal		

Subject No.	Randomisation Number	Study Group	Dosage	Dose	Day	Not Done	Specify	Date of Assessment	*Was the assessment performed prior to vaccination?	Physical Examination Parameter	Result	Abnormality	Clinical Significance
S233	210	JAIVAC-1	Dosage B	1	Day 14			14/10/2010		Gastrointestinal	Normal		
S233	210	JAIVAC-1	Dosage B	1	Day 14			14/10/2010		Lymphatic	Normal		
S233	210	JAIVAC-1	Dosage B	1	Day 14			14/10/2010		Neurological	Normal		
S233	210	JAIVAC-1	Dosage B	1	Day 14			14/10/2010		Genitourinary	Normal		
S233	210	JAIVAC-1	Dosage B	1	Day 14			14/10/2010		Dermatological	Normal		
S233	210	JAIVAC-1	Dosage B	2	Day 28			28/10/2010	Yes	Head and Neck	Normal		
S233	210	JAIVAC-1	Dosage B	2	Day 28			28/10/2010	Yes	Eyes	Normal		
S233	210	JAIVAC-1	Dosage B	2	Day 28			28/10/2010	Yes	Ears, nose and throat	Normal		
S233	210	JAIVAC-1	Dosage B	2	Day 28			28/10/2010	Yes	Cardiovascular	Normal		
S233	210	JAIVAC-1	Dosage B	2	Day 28			28/10/2010	Yes	Respiratory	Normal		
S233	210	JAIVAC-1	Dosage B	2	Day 28			28/10/2010	Yes	Musculoskeletal	Normal		
S233	210	JAIVAC-1	Dosage B	2	Day 28			28/10/2010	Yes	Venous system	Normal		
S233	210	JAIVAC-1	Dosage B	2	Day 28			28/10/2010	Yes	Gastrointestinal	Normal		
S233	210	JAIVAC-1	Dosage B	2	Day 28			28/10/2010	Yes	Lymphatic	Normal		
S233	210	JAIVAC-1	Dosage B	2	Day 28			28/10/2010	Yes	Neurological	Normal		
S233	210	JAIVAC-1	Dosage B	2	Day 28			28/10/2010	Yes	Genitourinary	Normal		
S233	210	JAIVAC-1	Dosage B	2	Day 28			28/10/2010	Yes	Dermatological	Normal		
S233	210	JAIVAC-1	Dosage B	2	Day 35			04/11/2010		Head and Neck	Normal		
S233	210	JAIVAC-1	Dosage B	2	Day 35			04/11/2010		Eyes	Normal		

Subject No.	Randomisation Number	Study Group	Dosage	Dose	Day	Not Done	Specify	Date of Assessment	*Was the assessment performed prior to vaccination?	Physical Examination Parameter	Result	Abnormality	Clinical Significance
S233	210	JAIVAC-1	Dosage B	2	Day 35			04/11/2010		Ears, nose and throat	Normal		
S233	210	JAIVAC-1	Dosage B	2	Day 35			04/11/2010		Cardiovascular	Normal		
S233	210	JAIVAC-1	Dosage B	2	Day 35			04/11/2010		Respiratory	Normal		
S233	210	JAIVAC-1	Dosage B	2	Day 35			04/11/2010		Musculoskeletal	Normal		
S233	210	JAIVAC-1	Dosage B	2	Day 35			04/11/2010		Venous system	Normal		
S233	210	JAIVAC-1	Dosage B	2	Day 35			04/11/2010		Gastrointestinal	Normal		
S233	210	JAIVAC-1	Dosage B	2	Day 35			04/11/2010		Lymphatic	Normal		
S233	210	JAIVAC-1	Dosage B	2	Day 35			04/11/2010		Neurological	Normal		
S233	210	JAIVAC-1	Dosage B	2	Day 35			04/11/2010		Genitourinary	Normal		
S233	210	JAIVAC-1	Dosage B	2	Day 35			04/11/2010		Dermatological	Normal		
S233	210	JAIVAC-1	Dosage B	2	Day 42			11/11/2010		Head and Neck	Normal		
S233	210	JAIVAC-1	Dosage B	2	Day 42			11/11/2010		Eyes	Normal		
S233	210	JAIVAC-1	Dosage B	2	Day 42			11/11/2010		Ears, nose and throat	Normal		
S233	210	JAIVAC-1	Dosage B	2	Day 42			11/11/2010		Cardiovascular	Normal		
S233	210	JAIVAC-1	Dosage B	2	Day 42			11/11/2010		Respiratory	Normal		
S233	210	JAIVAC-1	Dosage B	2	Day 42			11/11/2010		Musculoskeletal	Normal		
S233	210	JAIVAC-1	Dosage B	2	Day 42			11/11/2010		Venous system	Normal		
S233	210	JAIVAC-1	Dosage B	2	Day 42			11/11/2010		Gastrointestinal	Normal		
S233	210	JAIVAC-1	Dosage B	2	Day 42			11/11/2010		Lymphatic	Normal		

Subject No.	Randomisation Number	Study Group	Dosage	Dose	Day	Not Done	Specify	Date of Assessment	*Was the assessment performed prior to vaccination?	Physical Examination Parameter	Result	Abnormality	Clinical Significance
S233	210	JAIVAC-1	Dosage B	2	Day 42			11/11/2010		Neurological	Normal		
S233	210	JAIVAC-1	Dosage B	2	Day 42			11/11/2010		Genitourinary	Normal		
S233	210	JAIVAC-1	Dosage B	2	Day 42			11/11/2010		Dermatological	Normal		
S233	210	JAIVAC-1	Dosage B	2	Day 56			25/11/2010		Head and Neck	Normal		
S233	210	JAIVAC-1	Dosage B	2	Day 56			25/11/2010		Eyes	Normal		
S233	210	JAIVAC-1	Dosage B	2	Day 56			25/11/2010		Ears, nose and throat	Normal		
S233	210	JAIVAC-1	Dosage B	2	Day 56			25/11/2010		Cardiovascular	Normal		
S233	210	JAIVAC-1	Dosage B	2	Day 56			25/11/2010		Respiratory	Normal		
S233	210	JAIVAC-1	Dosage B	2	Day 56			25/11/2010		Musculoskeletal	Normal		
S233	210	JAIVAC-1	Dosage B	2	Day 56			25/11/2010		Venous system	Normal		
S233	210	JAIVAC-1	Dosage B	2	Day 56			25/11/2010		Gastrointestinal	Normal		
S233	210	JAIVAC-1	Dosage B	2	Day 56			25/11/2010		Lymphatic	Normal		
S233	210	JAIVAC-1	Dosage B	2	Day 56			25/11/2010		Neurological	Normal		
S233	210	JAIVAC-1	Dosage B	2	Day 56			25/11/2010		Genitourinary	Normal		
S233	210	JAIVAC-1	Dosage B	2	Day 56			25/11/2010		Dermatological	Normal		
S233	210	JAIVAC-1	Dosage B	3	Day 180			29/03/2011	Yes	Head and Neck	Normal		
S233	210	JAIVAC-1	Dosage B	3	Day 180			29/03/2011	Yes	Eyes	Normal		
S233	210	JAIVAC-1	Dosage B	3	Day 180			29/03/2011	Yes	Ears, nose and throat	Normal		
S233	210	JAIVAC-1	Dosage B	3	Day 180			29/03/2011	Yes	Cardiovascular	Normal		

Subject No.	Randomisation Number	Study Group	Dosage	Dose	Day	Not Done	Specify	Date of Assessment	*Was the assessment performed prior to vaccination?	Physical Examination Parameter	Result	Abnormality	Clinical Significance
S233	210	JAIVAC-1	Dosage B	3	Day 180			29/03/2011	Yes	Respiratory	Normal		
S233	210	JAIVAC-1	Dosage B	3	Day 180			29/03/2011	Yes	Musculoskeletal	Normal		
S233	210	JAIVAC-1	Dosage B	3	Day 180			29/03/2011	Yes	Venous system	Normal		
S233	210	JAIVAC-1	Dosage B	3	Day 180			29/03/2011	Yes	Gastrointestinal	Normal		
S233	210	JAIVAC-1	Dosage B	3	Day 180			29/03/2011	Yes	Lymphatic	Normal		
S233	210	JAIVAC-1	Dosage B	3	Day 180			29/03/2011	Yes	Neurological	Normal		
S233	210	JAIVAC-1	Dosage B	3	Day 180			29/03/2011	Yes	Genitourinary	Normal		
S233	210	JAIVAC-1	Dosage B	3	Day 180			29/03/2011	Yes	Dermatological	Normal		
S233	210	JAIVAC-1	Dosage B	3	Day 187			05/04/2011		Head and Neck	Normal		
S233	210	JAIVAC-1	Dosage B	3	Day 187			05/04/2011		Eyes	Normal		
S233	210	JAIVAC-1	Dosage B	3	Day 187			05/04/2011		Ears, nose and throat	Normal		
S233	210	JAIVAC-1	Dosage B	3	Day 187			05/04/2011		Cardiovascular	Normal		
S233	210	JAIVAC-1	Dosage B	3	Day 187			05/04/2011		Respiratory	Normal		
S233	210	JAIVAC-1	Dosage B	3	Day 187			05/04/2011		Musculoskeletal	Normal		
S233	210	JAIVAC-1	Dosage B	3	Day 187			05/04/2011		Venous system	Normal		
S233	210	JAIVAC-1	Dosage B	3	Day 187			05/04/2011		Gastrointestinal	Normal		
S233	210	JAIVAC-1	Dosage B	3	Day 187			05/04/2011		Lymphatic	Normal		
S233	210	JAIVAC-1	Dosage B	3	Day 187			05/04/2011		Neurological	Normal		
S233	210	JAIVAC-1	Dosage B	3	Day 187			05/04/2011		Genitourinary	Normal		

Subject No.	Randomisation Number	Study Group	Dosage	Dose	Day	Not Done	Specify	Date of Assessment	*Was the assessment performed prior to vaccination?	Physical Examination Parameter	Result	Abnormality	Clinical Significance
S233	210	JAIVAC-1	Dosage B	3	Day 187			05/04/2011		Dermatological	Normal		
S233	210	JAIVAC-1	Dosage B	3	Day 194			13/04/2011		Head and Neck	Normal		
S233	210	JAIVAC-1	Dosage B	3	Day 194			13/04/2011		Eyes	Normal		
S233	210	JAIVAC-1	Dosage B	3	Day 194			13/04/2011		Ears, nose and throat	Normal		
S233	210	JAIVAC-1	Dosage B	3	Day 194			13/04/2011		Cardiovascular	Normal		
S233	210	JAIVAC-1	Dosage B	3	Day 194			13/04/2011		Respiratory	Normal		
S233	210	JAIVAC-1	Dosage B	3	Day 194			13/04/2011		Musculoskeletal	Normal		
S233	210	JAIVAC-1	Dosage B	3	Day 194			13/04/2011		Venous system	Normal		
S233	210	JAIVAC-1	Dosage B	3	Day 194			13/04/2011		Gastrointestinal	Normal		
S233	210	JAIVAC-1	Dosage B	3	Day 194			13/04/2011		Lymphatic	Normal		
S233	210	JAIVAC-1	Dosage B	3	Day 194			13/04/2011		Neurological	Normal		
S233	210	JAIVAC-1	Dosage B	3	Day 194			13/04/2011		Genitourinary	Normal		
S233	210	JAIVAC-1	Dosage B	3	Day 194			13/04/2011		Dermatological	Normal		
S233	210	JAIVAC-1	Dosage B	3	Day 208			27/04/2011		Head and Neck	Normal		
S233	210	JAIVAC-1	Dosage B	3	Day 208			27/04/2011		Eyes	Normal		
S233	210	JAIVAC-1	Dosage B	3	Day 208			27/04/2011		Ears, nose and throat	Normal		
S233	210	JAIVAC-1	Dosage B	3	Day 208			27/04/2011		Cardiovascular	Normal		
S233	210	JAIVAC-1	Dosage B	3	Day 208			27/04/2011		Respiratory	Normal		
S233	210	JAIVAC-1	Dosage B	3	Day 208			27/04/2011		Musculoskeletal	Normal		

Subject No.	Randomisation Number	Study Group	Dosage	Dose	Day	Not Done	Specify	Date of Assessment	*Was the assessment performed prior to vaccination?	Physical Examination Parameter	Result	Abnormality	Clinical Significance
S233	210	JAIVAC-1	Dosage B	3	Day 208			27/04/2011		Venous system	Normal		
S233	210	JAIVAC-1	Dosage B	3	Day 208			27/04/2011		Gastrointestinal	Normal		
S233	210	JAIVAC-1	Dosage B	3	Day 208			27/04/2011		Lymphatic	Normal		
S233	210	JAIVAC-1	Dosage B	3	Day 208			27/04/2011		Neurological	Normal		
S233	210	JAIVAC-1	Dosage B	3	Day 208			27/04/2011		Genitourinary	Normal		
S233	210	JAIVAC-1	Dosage B	3	Day 208			27/04/2011		Dermatological	Normal		
S233	210	JAIVAC-1	Dosage B	3	Day 365			29/09/2011		Head and Neck	Normal		
S233	210	JAIVAC-1	Dosage B	3	Day 365			29/09/2011		Eyes	Normal		
S233	210	JAIVAC-1	Dosage B	3	Day 365			29/09/2011		Ears, nose and throat	Normal		
S233	210	JAIVAC-1	Dosage B	3	Day 365			29/09/2011		Cardiovascular	Normal		
S233	210	JAIVAC-1	Dosage B	3	Day 365			29/09/2011		Respiratory	Normal		
S233	210	JAIVAC-1	Dosage B	3	Day 365			29/09/2011		Musculoskeletal	Normal		
S233	210	JAIVAC-1	Dosage B	3	Day 365			29/09/2011		Venous system	Normal		
S233	210	JAIVAC-1	Dosage B	3	Day 365			29/09/2011		Gastrointestinal	Normal		
S233	210	JAIVAC-1	Dosage B	3	Day 365			29/09/2011		Lymphatic	Normal		
S233	210	JAIVAC-1	Dosage B	3	Day 365			29/09/2011		Neurological	Normal		
S233	210	JAIVAC-1	Dosage B	3	Day 365			29/09/2011		Genitourinary	Normal		
S233	210	JAIVAC-1	Dosage B	3	Day 365			29/09/2011		Dermatological	Normal		
S241	303	Hepatitis B	Hepatitis B		Day -14 to Day -1			03/11/2010		Head and neck	Normal		

Subject No.	Randomisation Number	Study Group	Dosage	Dose	Day	Not Done	Specify	Date of Assessment	*Was the assessment performed prior to vaccination?	Physical Examination Parameter	Result	Abnormality	Clinical Significance
S241	303	Hepatitis B	Hepatitis B		Day -14 to Day -1			03/11/2010		Eyes	Normal		
S241	303	Hepatitis B	Hepatitis B		Day -14 to Day -1			03/11/2010		Ears, nose and throat	Normal		
S241	303	Hepatitis B	Hepatitis B		Day -14 to Day -1			03/11/2010		Cardiovascular	Normal		
S241	303	Hepatitis B	Hepatitis B		Day -14 to Day -1			03/11/2010		Respiratory	Normal		
S241	303	Hepatitis B	Hepatitis B		Day -14 to Day -1			03/11/2010		Musculoskeletal	Normal		
S241	303	Hepatitis B	Hepatitis B		Day -14 to Day -1			03/11/2010		Venous system	Normal		
S241	303	Hepatitis B	Hepatitis B		Day -14 to Day -1			03/11/2010		Gastrointestinal	Normal		
S241	303	Hepatitis B	Hepatitis B		Day -14 to Day -1			03/11/2010		Lymphatic	Normal		
S241	303	Hepatitis B	Hepatitis B		Day -14 to Day -1			03/11/2010		Neurological	Normal		
S241	303	Hepatitis B	Hepatitis B		Day -14 to Day -1			03/11/2010		Genitourinary	Normal		
S241	303	Hepatitis B	Hepatitis B		Day -14 to Day -1			03/11/2010		Dermatological	Normal		
S241	303	Hepatitis B	Hepatitis B	1	Day 0			10/11/2010	Yes	Head and neck	Normal		
S241	303	Hepatitis B	Hepatitis B	1	Day 0			10/11/2010	Yes	Eyes	Normal		
S241	303	Hepatitis B	Hepatitis B	1	Day 0			10/11/2010	Yes	Ears, nose and throat	Normal		
S241	303	Hepatitis B	Hepatitis B	1	Day 0			10/11/2010	Yes	Cardiovascular	Normal		
S241	303	Hepatitis B	Hepatitis B	1	Day 0			10/11/2010	Yes	Respiratory	Normal		
S241	303	Hepatitis B	Hepatitis B	1	Day 0			10/11/2010	Yes	Musculoskeletal	Normal		

Subject No.	Randomisation Number	Study Group	Dosage	Dose	Day	Not Done	Specify	Date of Assessment	*Was the assessment performed prior to vaccination?	Physical Examination Parameter	Result	Abnormality	Clinical Significance
S241	303	Hepatitis B	Hepatitis B	1	Day 0			10/11/2010	Yes	Venous system	Normal		
S241	303	Hepatitis B	Hepatitis B	1	Day 0			10/11/2010	Yes	Gastrointestinal	Normal		
S241	303	Hepatitis B	Hepatitis B	1	Day 0			10/11/2010	Yes	Lymphatic	Normal		
S241	303	Hepatitis B	Hepatitis B	1	Day 0			10/11/2010	Yes	Neurological	Normal		
S241	303	Hepatitis B	Hepatitis B	1	Day 0			10/11/2010	Yes	Genitourinary	Normal		
S241	303	Hepatitis B	Hepatitis B	1	Day 0			10/11/2010	Yes	Dermatological	Normal		
S241	303	Hepatitis B	Hepatitis B	1	Day 7			17/11/2010		Head and Neck	Normal		
S241	303	Hepatitis B	Hepatitis B	1	Day 7			17/11/2010		Eyes	Normal		
S241	303	Hepatitis B	Hepatitis B	1	Day 7			17/11/2010		Ears, nose and throat	Normal		
S241	303	Hepatitis B	Hepatitis B	1	Day 7			17/11/2010		Cardiovascular	Normal		
S241	303	Hepatitis B	Hepatitis B	1	Day 7			17/11/2010		Respiratory	Normal		
S241	303	Hepatitis B	Hepatitis B	1	Day 7			17/11/2010		Musculoskeletal	Normal		
S241	303	Hepatitis B	Hepatitis B	1	Day 7			17/11/2010		Venous system	Normal		
S241	303	Hepatitis B	Hepatitis B	1	Day 7			17/11/2010		Gastrointestinal	Normal		
S241	303	Hepatitis B	Hepatitis B	1	Day 7			17/11/2010		Lymphatic	Normal		
S241	303	Hepatitis B	Hepatitis B	1	Day 7			17/11/2010		Neurological	Normal		
S241	303	Hepatitis B	Hepatitis B	1	Day 7			17/11/2010		Genitourinary	Normal		
S241	303	Hepatitis B	Hepatitis B	1	Day 7			17/11/2010		Dermatological	Normal		
S241	303	Hepatitis B	Hepatitis B	1	Day 14			24/11/2010		Head and Neck	Normal		

Subject No.	Randomisation Number	Study Group	Dosage	Dose	Day	Not Done	Specify	Date of Assessment	*Was the assessment performed prior to vaccination?	Physical Examination Parameter	Result	Abnormality	Clinical Significance
S241	303	Hepatitis B	Hepatitis B	1	Day 14			24/11/2010		Eyes	Normal		
S241	303	Hepatitis B	Hepatitis B	1	Day 14			24/11/2010		Ears, nose and throat	Normal		
S241	303	Hepatitis B	Hepatitis B	1	Day 14			24/11/2010		Cardiovascular	Normal		
S241	303	Hepatitis B	Hepatitis B	1	Day 14			24/11/2010		Respiratory	Normal		
S241	303	Hepatitis B	Hepatitis B	1	Day 14			24/11/2010		Musculoskeletal	Normal		
S241	303	Hepatitis B	Hepatitis B	1	Day 14			24/11/2010		Venous system	Normal		
S241	303	Hepatitis B	Hepatitis B	1	Day 14			24/11/2010		Gastrointestinal	Normal		
S241	303	Hepatitis B	Hepatitis B	1	Day 14			24/11/2010		Lymphatic	Normal		
S241	303	Hepatitis B	Hepatitis B	1	Day 14			24/11/2010		Neurological	Normal		
S241	303	Hepatitis B	Hepatitis B	1	Day 14			24/11/2010		Genitourinary	Normal		
S241	303	Hepatitis B	Hepatitis B	1	Day 14			24/11/2010		Dermatological	Normal		
S241	303	Hepatitis B	Hepatitis B	2	Day 28			08/12/2010	Yes	Head and Neck	Normal		
S241	303	Hepatitis B	Hepatitis B	2	Day 28			08/12/2010	Yes	Eyes	Normal		
S241	303	Hepatitis B	Hepatitis B	2	Day 28			08/12/2010	Yes	Ears, nose and throat	Normal		
S241	303	Hepatitis B	Hepatitis B	2	Day 28			08/12/2010	Yes	Cardiovascular	Normal		
S241	303	Hepatitis B	Hepatitis B	2	Day 28			08/12/2010	Yes	Respiratory	Normal		
S241	303	Hepatitis B	Hepatitis B	2	Day 28			08/12/2010	Yes	Musculoskeletal	Normal		
S241	303	Hepatitis B	Hepatitis B	2	Day 28			08/12/2010	Yes	Venous system	Normal		
S241	303	Hepatitis B	Hepatitis B	2	Day 28			08/12/2010	Yes	Gastrointestinal	Normal		

Subject No.	Randomisation Number	Study Group	Dosage	Dose	Day	Not Done	Specify	Date of Assessment	*Was the assessment performed prior to vaccination?	Physical Examination Parameter	Result	Abnormality	Clinical Significance
S241	303	Hepatitis B	Hepatitis B	2	Day 28			08/12/2010	Yes	Lymphatic	Normal		
S241	303	Hepatitis B	Hepatitis B	2	Day 28			08/12/2010	Yes	Neurological	Normal		
S241	303	Hepatitis B	Hepatitis B	2	Day 28			08/12/2010	Yes	Genitourinary	Normal		
S241	303	Hepatitis B	Hepatitis B	2	Day 28			08/12/2010	Yes	Dermatological	Normal		
S241	303	Hepatitis B	Hepatitis B	2	Day 35			15/12/2010		Head and Neck	Normal		
S241	303	Hepatitis B	Hepatitis B	2	Day 35			15/12/2010		Eyes	Normal		
S241	303	Hepatitis B	Hepatitis B	2	Day 35			15/12/2010		Ears, nose and throat	Normal		
S241	303	Hepatitis B	Hepatitis B	2	Day 35			15/12/2010		Cardiovascular	Normal		
S241	303	Hepatitis B	Hepatitis B	2	Day 35			15/12/2010		Respiratory	Normal		
S241	303	Hepatitis B	Hepatitis B	2	Day 35			15/12/2010		Musculoskeletal	Normal		
S241	303	Hepatitis B	Hepatitis B	2	Day 35			15/12/2010		Venous system	Normal		
S241	303	Hepatitis B	Hepatitis B	2	Day 35			15/12/2010		Gastrointestinal	Normal		
S241	303	Hepatitis B	Hepatitis B	2	Day 35			15/12/2010		Lymphatic	Normal		
S241	303	Hepatitis B	Hepatitis B	2	Day 35			15/12/2010		Neurological	Normal		
S241	303	Hepatitis B	Hepatitis B	2	Day 35			15/12/2010		Genitourinary	Normal		
S241	303	Hepatitis B	Hepatitis B	2	Day 35			15/12/2010		Dermatological	Normal		
S241	303	Hepatitis B	Hepatitis B	2	Day 42			22/12/2010		Head and Neck	Normal		
S241	303	Hepatitis B	Hepatitis B	2	Day 42			22/12/2010		Eyes	Normal		
S241	303	Hepatitis B	Hepatitis B	2	Day 42			22/12/2010		Ears, nose and throat	Normal		

Subject No.	Randomisation Number	Study Group	Dosage	Dose	Day	Not Done	Specify	Date of Assessment	*Was the assessment performed prior to vaccination?	Physical Examination Parameter	Result	Abnormality	Clinical Significance
S241	303	Hepatitis B	Hepatitis B	2	Day 42			22/12/2010		Cardiovascular	Normal		
S241	303	Hepatitis B	Hepatitis B	2	Day 42			22/12/2010		Respiratory	Normal		
S241	303	Hepatitis B	Hepatitis B	2	Day 42			22/12/2010		Musculoskeletal	Normal		
S241	303	Hepatitis B	Hepatitis B	2	Day 42			22/12/2010		Venous system	Normal		
S241	303	Hepatitis B	Hepatitis B	2	Day 42			22/12/2010		Gastrointestinal	Normal		
S241	303	Hepatitis B	Hepatitis B	2	Day 42			22/12/2010		Lymphatic	Normal		
S241	303	Hepatitis B	Hepatitis B	2	Day 42			22/12/2010		Neurological	Normal		
S241	303	Hepatitis B	Hepatitis B	2	Day 42			22/12/2010		Genitourinary	Normal		
S241	303	Hepatitis B	Hepatitis B	2	Day 42			22/12/2010		Dermatological	Normal		
S241	303	Hepatitis B	Hepatitis B	2	Day 56			05/01/2011		Head and Neck	Normal		
S241	303	Hepatitis B	Hepatitis B	2	Day 56			05/01/2011		Eyes	Normal		
S241	303	Hepatitis B	Hepatitis B	2	Day 56			05/01/2011		Ears, nose and throat	Normal		
S241	303	Hepatitis B	Hepatitis B	2	Day 56			05/01/2011		Cardiovascular	Normal		
S241	303	Hepatitis B	Hepatitis B	2	Day 56			05/01/2011		Respiratory	Normal		
S241	303	Hepatitis B	Hepatitis B	2	Day 56			05/01/2011		Musculoskeletal	Normal		
S241	303	Hepatitis B	Hepatitis B	2	Day 56			05/01/2011		Venous system	Normal		
S241	303	Hepatitis B	Hepatitis B	2	Day 56			05/01/2011		Gastrointestinal	Normal		
S241	303	Hepatitis B	Hepatitis B	2	Day 56			05/01/2011		Lymphatic	Normal		
S241	303	Hepatitis B	Hepatitis B	2	Day 56			05/01/2011		Neurological	Normal		

Subject No.	Randomisation Number	Study Group	Dosage	Dose	Day	Not Done	Specify	Date of Assessment	*Was the assessment performed prior to vaccination?	Physical Examination Parameter	Result	Abnormality	Clinical Significance
S241	303	Hepatitis B	Hepatitis B	2	Day 56			05/01/2011		Genitourinary	Normal		
S241	303	Hepatitis B	Hepatitis B	2	Day 56			05/01/2011		Dermatological	Normal		
S241	303	Hepatitis B	Hepatitis B	3	Day 180			09/05/2011	Yes	Head and Neck	Normal		
S241	303	Hepatitis B	Hepatitis B	3	Day 180			09/05/2011	Yes	Eyes	Normal		
S241	303	Hepatitis B	Hepatitis B	3	Day 180			09/05/2011	Yes	Ears, nose and throat	Normal		
S241	303	Hepatitis B	Hepatitis B	3	Day 180			09/05/2011	Yes	Cardiovascular	Normal		
S241	303	Hepatitis B	Hepatitis B	3	Day 180			09/05/2011	Yes	Respiratory	Normal		
S241	303	Hepatitis B	Hepatitis B	3	Day 180			09/05/2011	Yes	Musculoskeletal	Normal		
S241	303	Hepatitis B	Hepatitis B	3	Day 180			09/05/2011	Yes	Venous system	Normal		
S241	303	Hepatitis B	Hepatitis B	3	Day 180			09/05/2011	Yes	Gastrointestinal	Normal		
S241	303	Hepatitis B	Hepatitis B	3	Day 180			09/05/2011	Yes	Lymphatic	Normal		
S241	303	Hepatitis B	Hepatitis B	3	Day 180			09/05/2011	Yes	Neurological	Normal		
S241	303	Hepatitis B	Hepatitis B	3	Day 180			09/05/2011	Yes	Genitourinary	Normal		
S241	303	Hepatitis B	Hepatitis B	3	Day 180			09/05/2011	Yes	Dermatological	Normal		
S241	303	Hepatitis B	Hepatitis B	3	Day 187			16/05/2011		Head and Neck	Normal		
S241	303	Hepatitis B	Hepatitis B	3	Day 187			16/05/2011		Eyes	Normal		
S241	303	Hepatitis B	Hepatitis B	3	Day 187			16/05/2011		Ears, nose and throat	Normal		
S241	303	Hepatitis B	Hepatitis B	3	Day 187			16/05/2011		Cardiovascular	Normal		
S241	303	Hepatitis B	Hepatitis B	3	Day 187			16/05/2011		Respiratory	Normal		

Subject No.	Randomisation Number	Study Group	Dosage	Dose	Day	Not Done	Specify	Date of Assessment	*Was the assessment performed prior to vaccination?	Physical Examination Parameter	Result	Abnormality	Clinical Significance
S241	303	Hepatitis B	Hepatitis B	3	Day 187			16/05/2011		Musculoskeletal	Normal		
S241	303	Hepatitis B	Hepatitis B	3	Day 187			16/05/2011		Venous system	Normal		
S241	303	Hepatitis B	Hepatitis B	3	Day 187			16/05/2011		Gastrointestinal	Normal		
S241	303	Hepatitis B	Hepatitis B	3	Day 187			16/05/2011		Lymphatic	Normal		
S241	303	Hepatitis B	Hepatitis B	3	Day 187			16/05/2011		Neurological	Normal		
S241	303	Hepatitis B	Hepatitis B	3	Day 187			16/05/2011		Genitourinary	Normal		
S241	303	Hepatitis B	Hepatitis B	3	Day 187			16/05/2011		Dermatological	Normal		
S241	303	Hepatitis B	Hepatitis B	3	Day 194			23/05/2011		Head and Neck	Normal		
S241	303	Hepatitis B	Hepatitis B	3	Day 194			23/05/2011		Eyes	Normal		
S241	303	Hepatitis B	Hepatitis B	3	Day 194			23/05/2011		Ears, nose and throat	Normal		
S241	303	Hepatitis B	Hepatitis B	3	Day 194			23/05/2011		Cardiovascular	Normal		
S241	303	Hepatitis B	Hepatitis B	3	Day 194			23/05/2011		Respiratory	Normal		
S241	303	Hepatitis B	Hepatitis B	3	Day 194			23/05/2011		Musculoskeletal	Normal		
S241	303	Hepatitis B	Hepatitis B	3	Day 194			23/05/2011		Venous system	Normal		
S241	303	Hepatitis B	Hepatitis B	3	Day 194			23/05/2011		Gastrointestinal	Normal		
S241	303	Hepatitis B	Hepatitis B	3	Day 194			23/05/2011		Lymphatic	Normal		
S241	303	Hepatitis B	Hepatitis B	3	Day 194			23/05/2011		Neurological	Normal		
S241	303	Hepatitis B	Hepatitis B	3	Day 194			23/05/2011		Genitourinary	Normal		
S241	303	Hepatitis B	Hepatitis B	3	Day 194			23/05/2011		Dermatological	Normal		

Subject No.	Randomisation Number	Study Group	Dosage	Dose	Day	Not Done	Specify	Date of Assessment	*Was the assessment performed prior to vaccination?	Physical Examination Parameter	Result	Abnormality	Clinical Significance
S241	303	Hepatitis B	Hepatitis B	3	Day 208			06/06/2011		Head and Neck	Normal		
S241	303	Hepatitis B	Hepatitis B	3	Day 208			06/06/2011		Eyes	Normal		
S241	303	Hepatitis B	Hepatitis B	3	Day 208			06/06/2011		Ears, nose and throat	Normal		
S241	303	Hepatitis B	Hepatitis B	3	Day 208			06/06/2011		Cardiovascular	Normal		
S241	303	Hepatitis B	Hepatitis B	3	Day 208			06/06/2011		Respiratory	Normal		
S241	303	Hepatitis B	Hepatitis B	3	Day 208			06/06/2011		Musculoskeletal	Normal		
S241	303	Hepatitis B	Hepatitis B	3	Day 208			06/06/2011		Venous system	Normal		
S241	303	Hepatitis B	Hepatitis B	3	Day 208			06/06/2011		Gastrointestinal	Normal		
S241	303	Hepatitis B	Hepatitis B	3	Day 208			06/06/2011		Lymphatic	Normal		
S241	303	Hepatitis B	Hepatitis B	3	Day 208			06/06/2011		Neurological	Normal		
S241	303	Hepatitis B	Hepatitis B	3	Day 208			06/06/2011		Genitourinary	Normal		
S241	303	Hepatitis B	Hepatitis B	3	Day 208			06/06/2011		Dermatological	Normal		
S241	303	Hepatitis B	Hepatitis B	3	Day 365			09/11/2011		Head and Neck	Normal		
S241	303	Hepatitis B	Hepatitis B	3	Day 365			09/11/2011		Eyes	Normal		
S241	303	Hepatitis B	Hepatitis B	3	Day 365			09/11/2011		Ears, nose and throat	Normal		
S241	303	Hepatitis B	Hepatitis B	3	Day 365			09/11/2011		Cardiovascular	Normal		
S241	303	Hepatitis B	Hepatitis B	3	Day 365			09/11/2011		Respiratory	Normal		
S241	303	Hepatitis B	Hepatitis B	3	Day 365			09/11/2011		Musculoskeletal	Normal		
S241	303	Hepatitis B	Hepatitis B	3	Day 365			09/11/2011		Venous system	Normal		

Subject No.	Randomisation Number	Study Group	Dosage	Dose	Day	Not Done	Specify	Date of Assessment	*Was the assessment performed prior to vaccination?	Physical Examination Parameter	Result	Abnormality	Clinical Significance
S241	303	Hepatitis B	Hepatitis B	3	Day 365			09/11/2011		Gastrointestinal	Normal		
S241	303	Hepatitis B	Hepatitis B	3	Day 365			09/11/2011		Lymphatic	Normal		
S241	303	Hepatitis B	Hepatitis B	3	Day 365			09/11/2011		Neurological	Normal		
S241	303	Hepatitis B	Hepatitis B	3	Day 365			09/11/2011		Genitourinary	Normal		
S241	303	Hepatitis B	Hepatitis B	3	Day 365			09/11/2011		Dermatological	Normal		
S250	311	JAIVAC-1	Dosage C		Day -14 to Day -1			03/11/2010		Head and neck	Normal		
S250	311	JAIVAC-1	Dosage C		Day -14 to Day -1			03/11/2010		Eyes	Normal		
S250	311	JAIVAC-1	Dosage C		Day -14 to Day -1			03/11/2010		Ears, nose and throat	Normal		
S250	311	JAIVAC-1	Dosage C		Day -14 to Day -1			03/11/2010		Cardiovascular	Normal		
S250	311	JAIVAC-1	Dosage C		Day -14 to Day -1			03/11/2010		Respiratory	Normal		
S250	311	JAIVAC-1	Dosage C		Day -14 to Day -1			03/11/2010		Musculoskeletal	Normal		
S250	311	JAIVAC-1	Dosage C		Day -14 to Day -1			03/11/2010		Venous system	Normal		
S250	311	JAIVAC-1	Dosage C		Day -14 to Day -1			03/11/2010		Gastrointestinal	Normal		
S250	311	JAIVAC-1	Dosage C		Day -14 to Day -1			03/11/2010		Lymphatic	Normal		
S250	311	JAIVAC-1	Dosage C		Day -14 to Day -1			03/11/2010		Neurological	Normal		
S250	311	JAIVAC-1	Dosage C		Day -14 to Day -1			03/11/2010		Genitourinary	Normal		
S250	311	JAIVAC-1	Dosage C		Day -14 to Day -1			03/11/2010		Dermatological	Normal		

Subject No.	Randomisation Number	Study Group	Dosage	Dose	Day	Not Done	Specify	Date of Assessment	*Was the assessment performed prior to vaccination?	Physical Examination Parameter	Result	Abnormality	Clinical Significance
S250	311	JAIVAC-1	Dosage C	1	Day 0			12/11/2010	Yes	Head and neck	Normal		
S250	311	JAIVAC-1	Dosage C	1	Day 0			12/11/2010	Yes	Eyes	Normal		
S250	311	JAIVAC-1	Dosage C	1	Day 0			12/11/2010	Yes	Ears, nose and throat	Normal		
S250	311	JAIVAC-1	Dosage C	1	Day 0			12/11/2010	Yes	Cardiovascular	Normal		
S250	311	JAIVAC-1	Dosage C	1	Day 0			12/11/2010	Yes	Respiratory	Normal		
S250	311	JAIVAC-1	Dosage C	1	Day 0			12/11/2010	Yes	Musculoskeletal	Normal		
S250	311	JAIVAC-1	Dosage C	1	Day 0			12/11/2010	Yes	Venous system	Normal		
S250	311	JAIVAC-1	Dosage C	1	Day 0			12/11/2010	Yes	Gastrointestinal	Normal		
S250	311	JAIVAC-1	Dosage C	1	Day 0			12/11/2010	Yes	Lymphatic	Normal		
S250	311	JAIVAC-1	Dosage C	1	Day 0			12/11/2010	Yes	Neurological	Normal		
S250	311	JAIVAC-1	Dosage C	1	Day 0			12/11/2010	Yes	Genitourinary	Normal		
S250	311	JAIVAC-1	Dosage C	1	Day 0			12/11/2010	Yes	Dermatological	Normal		
S250	311	JAIVAC-1	Dosage C	1	Day 7			19/11/2010		Head and Neck	Normal		
S250	311	JAIVAC-1	Dosage C	1	Day 7			19/11/2010		Eyes	Normal		
S250	311	JAIVAC-1	Dosage C	1	Day 7			19/11/2010		Ears, nose and throat	Normal		
S250	311	JAIVAC-1	Dosage C	1	Day 7			19/11/2010		Cardiovascular	Normal		
S250	311	JAIVAC-1	Dosage C	1	Day 7			19/11/2010		Respiratory	Normal		
S250	311	JAIVAC-1	Dosage C	1	Day 7			19/11/2010		Musculoskeletal	Normal		
S250	311	JAIVAC-1	Dosage C	1	Day 7			19/11/2010		Venous system	Normal		

Subject No.	Randomisation Number	Study Group	Dosage	Dose	Day	Not Done	Specify	Date of Assessment	*Was the assessment performed prior to vaccination?	Physical Examination Parameter	Result	Abnormality	Clinical Significance
S250	311	JAIVAC-1	Dosage C	1	Day 7			19/11/2010		Gastrointestinal	Normal		
S250	311	JAIVAC-1	Dosage C	1	Day 7			19/11/2010		Lymphatic	Normal		
S250	311	JAIVAC-1	Dosage C	1	Day 7			19/11/2010		Neurological	Normal		
S250	311	JAIVAC-1	Dosage C	1	Day 7			19/11/2010		Genitourinary	Normal		
S250	311	JAIVAC-1	Dosage C	1	Day 7			19/11/2010		Dermatological	Normal		
S250	311	JAIVAC-1	Dosage C	1	Day 14			26/11/2010		Head and Neck	Normal		
S250	311	JAIVAC-1	Dosage C	1	Day 14			26/11/2010		Eyes	Normal		
S250	311	JAIVAC-1	Dosage C	1	Day 14			26/11/2010		Ears, nose and throat	Normal		
S250	311	JAIVAC-1	Dosage C	1	Day 14			26/11/2010		Cardiovascular	Normal		
S250	311	JAIVAC-1	Dosage C	1	Day 14			26/11/2010		Respiratory	Normal		
S250	311	JAIVAC-1	Dosage C	1	Day 14			26/11/2010		Musculoskeletal	Normal		
S250	311	JAIVAC-1	Dosage C	1	Day 14			26/11/2010		Venous system	Normal		
S250	311	JAIVAC-1	Dosage C	1	Day 14			26/11/2010		Gastrointestinal	Normal		
S250	311	JAIVAC-1	Dosage C	1	Day 14			26/11/2010		Lymphatic	Normal		
S250	311	JAIVAC-1	Dosage C	1	Day 14			26/11/2010		Neurological	Normal		
S250	311	JAIVAC-1	Dosage C	1	Day 14			26/11/2010		Genitourinary	Normal		
S250	311	JAIVAC-1	Dosage C	1	Day 14			26/11/2010		Dermatological	Normal		
S250	311	JAIVAC-1	Dosage C	2	Day 28			10/12/2010	Yes	Head and Neck	Normal		
S250	311	JAIVAC-1	Dosage C	2	Day 28			10/12/2010	Yes	Eyes	Normal		

Subject No.	Randomisation Number	Study Group	Dosage	Dose	Day	Not Done	Specify	Date of Assessment	*Was the assessment performed prior to vaccination?	Physical Examination Parameter	Result	Abnormality	Clinical Significance
S250	311	JAIVAC-1	Dosage C	2	Day 28			10/12/2010	Yes	Ears, nose and throat	Normal		
S250	311	JAIVAC-1	Dosage C	2	Day 28			10/12/2010	Yes	Cardiovascular	Normal		
S250	311	JAIVAC-1	Dosage C	2	Day 28			10/12/2010	Yes	Respiratory	Normal		
S250	311	JAIVAC-1	Dosage C	2	Day 28			10/12/2010	Yes	Musculoskeletal	Normal		
S250	311	JAIVAC-1	Dosage C	2	Day 28			10/12/2010	Yes	Venous system	Normal		
S250	311	JAIVAC-1	Dosage C	2	Day 28			10/12/2010	Yes	Gastrointestinal	Normal		
S250	311	JAIVAC-1	Dosage C	2	Day 28			10/12/2010	Yes	Lymphatic	Normal		
S250	311	JAIVAC-1	Dosage C	2	Day 28			10/12/2010	Yes	Neurological	Normal		
S250	311	JAIVAC-1	Dosage C	2	Day 28			10/12/2010	Yes	Genitourinary	Normal		
S250	311	JAIVAC-1	Dosage C	2	Day 28			10/12/2010	Yes	Dermatological	Normal		
S250	311	JAIVAC-1	Dosage C	2	Day 35			17/12/2010		Head and Neck	Normal		
S250	311	JAIVAC-1	Dosage C	2	Day 35			17/12/2010		Eyes	Normal		
S250	311	JAIVAC-1	Dosage C	2	Day 35			17/12/2010		Ears, nose and throat	Normal		
S250	311	JAIVAC-1	Dosage C	2	Day 35			17/12/2010		Cardiovascular	Normal		
S250	311	JAIVAC-1	Dosage C	2	Day 35			17/12/2010		Respiratory	Normal		
S250	311	JAIVAC-1	Dosage C	2	Day 35			17/12/2010		Musculoskeletal	Normal		
S250	311	JAIVAC-1	Dosage C	2	Day 35			17/12/2010		Venous system	Normal		
S250	311	JAIVAC-1	Dosage C	2	Day 35			17/12/2010		Gastrointestinal	Normal		
S250	311	JAIVAC-1	Dosage C	2	Day 35			17/12/2010		Lymphatic	Normal		

Subject No.	Randomisation Number	Study Group	Dosage	Dose	Day	Not Done	Specify	Date of Assessment	*Was the assessment performed prior to vaccination?	Physical Examination Parameter	Result	Abnormality	Clinical Significance
S250	311	JAIVAC-1	Dosage C	2	Day 35			17/12/2010		Neurological	Normal		
S250	311	JAIVAC-1	Dosage C	2	Day 35			17/12/2010		Genitourinary	Normal		
S250	311	JAIVAC-1	Dosage C	2	Day 35			17/12/2010		Dermatological	Normal		
S250	311	JAIVAC-1	Dosage C	2	Day 42			24/12/2010		Head and Neck	Normal		
S250	311	JAIVAC-1	Dosage C	2	Day 42			24/12/2010		Eyes	Normal		
S250	311	JAIVAC-1	Dosage C	2	Day 42			24/12/2010		Ears, nose and throat	Normal		
S250	311	JAIVAC-1	Dosage C	2	Day 42			24/12/2010		Cardiovascular	Normal		
S250	311	JAIVAC-1	Dosage C	2	Day 42			24/12/2010		Respiratory	Normal		
S250	311	JAIVAC-1	Dosage C	2	Day 42			24/12/2010		Musculoskeletal	Normal		
S250	311	JAIVAC-1	Dosage C	2	Day 42			24/12/2010		Venous system	Normal		
S250	311	JAIVAC-1	Dosage C	2	Day 42			24/12/2010		Gastrointestinal	Normal		
S250	311	JAIVAC-1	Dosage C	2	Day 42			24/12/2010		Lymphatic	Normal		
S250	311	JAIVAC-1	Dosage C	2	Day 42			24/12/2010		Neurological	Normal		
S250	311	JAIVAC-1	Dosage C	2	Day 42			24/12/2010		Genitourinary	Normal		
S250	311	JAIVAC-1	Dosage C	2	Day 42			24/12/2010		Dermatological	Normal		
S250	311	JAIVAC-1	Dosage C	2	Day 56			07/01/2011		Head and Neck	Normal		
S250	311	JAIVAC-1	Dosage C	2	Day 56			07/01/2011		Eyes	Normal		
S250	311	JAIVAC-1	Dosage C	2	Day 56			07/01/2011		Ears, nose and throat	Normal		
S250	311	JAIVAC-1	Dosage C	2	Day 56			07/01/2011		Cardiovascular	Normal		

Subject No.	Randomisation Number	Study Group	Dosage	Dose	Day	Not Done	Specify	Date of Assessment	*Was the assessment performed prior to vaccination?	Physical Examination Parameter	Result	Abnormality	Clinical Significance
S250	311	JAIVAC-1	Dosage C	2	Day 56			07/01/2011		Respiratory	Normal		
S250	311	JAIVAC-1	Dosage C	2	Day 56			07/01/2011		Musculoskeletal	Normal		
S250	311	JAIVAC-1	Dosage C	2	Day 56			07/01/2011		Venous system	Normal		
S250	311	JAIVAC-1	Dosage C	2	Day 56			07/01/2011		Gastrointestinal	Normal		
S250	311	JAIVAC-1	Dosage C	2	Day 56			07/01/2011		Lymphatic	Normal		
S250	311	JAIVAC-1	Dosage C	2	Day 56			07/01/2011		Neurological	Normal		
S250	311	JAIVAC-1	Dosage C	2	Day 56			07/01/2011		Genitourinary	Normal		
S250	311	JAIVAC-1	Dosage C	2	Day 56			07/01/2011		Dermatological	Normal		
S250	311	JAIVAC-1	Dosage C	3	Day 180			11/05/2011	Yes	Head and Neck	Normal		
S250	311	JAIVAC-1	Dosage C	3	Day 180			11/05/2011	Yes	Eyes	Normal		
S250	311	JAIVAC-1	Dosage C	3	Day 180			11/05/2011	Yes	Ears, nose and throat	Normal		
S250	311	JAIVAC-1	Dosage C	3	Day 180			11/05/2011	Yes	Cardiovascular	Normal		
S250	311	JAIVAC-1	Dosage C	3	Day 180			11/05/2011	Yes	Respiratory	Normal		
S250	311	JAIVAC-1	Dosage C	3	Day 180			11/05/2011	Yes	Musculoskeletal	Normal		
S250	311	JAIVAC-1	Dosage C	3	Day 180			11/05/2011	Yes	Venous system	Normal		
S250	311	JAIVAC-1	Dosage C	3	Day 180			11/05/2011	Yes	Gastrointestinal	Normal		
S250	311	JAIVAC-1	Dosage C	3	Day 180			11/05/2011	Yes	Lymphatic	Normal		
S250	311	JAIVAC-1	Dosage C	3	Day 180			11/05/2011	Yes	Neurological	Normal		
S250	311	JAIVAC-1	Dosage C	3	Day 180			11/05/2011	Yes	Genitourinary	Normal		

Subject No.	Randomisation Number	Study Group	Dosage	Dose	Day	Not Done	Specify	Date of Assessment	*Was the assessment performed prior to vaccination?	Physical Examination Parameter	Result	Abnormality	Clinical Significance
S250	311	JAIVAC-1	Dosage C	3	Day 180			11/05/2011	Yes	Dermatological	Normal		
S250	311	JAIVAC-1	Dosage C	3	Day 187			18/05/2011		Head and Neck	Normal		
S250	311	JAIVAC-1	Dosage C	3	Day 187			18/05/2011		Eyes	Normal		
S250	311	JAIVAC-1	Dosage C	3	Day 187			18/05/2011		Ears, nose and throat	Normal		
S250	311	JAIVAC-1	Dosage C	3	Day 187			18/05/2011		Cardiovascular	Normal		
S250	311	JAIVAC-1	Dosage C	3	Day 187			18/05/2011		Respiratory	Normal		
S250	311	JAIVAC-1	Dosage C	3	Day 187			18/05/2011		Musculoskeletal	Normal		
S250	311	JAIVAC-1	Dosage C	3	Day 187			18/05/2011		Venous system	Normal		
S250	311	JAIVAC-1	Dosage C	3	Day 187			18/05/2011		Gastrointestinal	Normal		
S250	311	JAIVAC-1	Dosage C	3	Day 187			18/05/2011		Lymphatic	Normal		
S250	311	JAIVAC-1	Dosage C	3	Day 187			18/05/2011		Neurological	Normal		
S250	311	JAIVAC-1	Dosage C	3	Day 187			18/05/2011		Genitourinary	Normal		
S250	311	JAIVAC-1	Dosage C	3	Day 187			18/05/2011		Dermatological	Normal		
S250	311	JAIVAC-1	Dosage C	3	Day 194			24/05/2011		Head and Neck	Normal		
S250	311	JAIVAC-1	Dosage C	3	Day 194			24/05/2011		Eyes	Normal		
S250	311	JAIVAC-1	Dosage C	3	Day 194			24/05/2011		Ears, nose and throat	Normal		
S250	311	JAIVAC-1	Dosage C	3	Day 194			24/05/2011		Cardiovascular	Normal		
S250	311	JAIVAC-1	Dosage C	3	Day 194			24/05/2011		Respiratory	Normal		
S250	311	JAIVAC-1	Dosage C	3	Day 194			24/05/2011		Musculoskeletal	Normal		

Subject No.	Randomisation Number	Study Group	Dosage	Dose	Day	Not Done	Specify	Date of Assessment	*Was the assessment performed prior to vaccination?	Physical Examination Parameter	Result	Abnormality	Clinical Significance
S250	311	JAIVAC-1	Dosage C	3	Day 194			24/05/2011		Venous system	Normal		
S250	311	JAIVAC-1	Dosage C	3	Day 194			24/05/2011		Gastrointestinal	Normal		
S250	311	JAIVAC-1	Dosage C	3	Day 194			24/05/2011		Lymphatic	Normal		
S250	311	JAIVAC-1	Dosage C	3	Day 194			24/05/2011		Neurological	Normal		
S250	311	JAIVAC-1	Dosage C	3	Day 194			24/05/2011		Genitourinary	Normal		
S250	311	JAIVAC-1	Dosage C	3	Day 194			24/05/2011		Dermatological	Normal		
S250	311	JAIVAC-1	Dosage C	3	Day 208			08/06/2011		Head and Neck	Normal		
S250	311	JAIVAC-1	Dosage C	3	Day 208			08/06/2011		Eyes	Normal		
S250	311	JAIVAC-1	Dosage C	3	Day 208			08/06/2011		Ears, nose and throat	Normal		
S250	311	JAIVAC-1	Dosage C	3	Day 208			08/06/2011		Cardiovascular	Normal		
S250	311	JAIVAC-1	Dosage C	3	Day 208			08/06/2011		Respiratory	Normal		
S250	311	JAIVAC-1	Dosage C	3	Day 208			08/06/2011		Musculoskeletal	Normal		
S250	311	JAIVAC-1	Dosage C	3	Day 208			08/06/2011		Venous system	Normal		
S250	311	JAIVAC-1	Dosage C	3	Day 208			08/06/2011		Gastrointestinal	Normal		
S250	311	JAIVAC-1	Dosage C	3	Day 208			08/06/2011		Lymphatic	Normal		
S250	311	JAIVAC-1	Dosage C	3	Day 208			08/06/2011		Neurological	Normal		
S250	311	JAIVAC-1	Dosage C	3	Day 208			08/06/2011		Genitourinary	Normal		
S250	311	JAIVAC-1	Dosage C	3	Day 208			08/06/2011		Dermatological	Normal		
S250	311	JAIVAC-1	Dosage C	3	Day 365			10/11/2011		Head and Neck	Normal		

Subject No.	Randomisation Number	Study Group	Dosage	Dose	Day	Not Done	Specify	Date of Assessment	*Was the assessment performed prior to vaccination?	Physical Examination Parameter	Result	Abnormality	Clinical Significance
S250	311	JAIVAC-1	Dosage C	3	Day 365			10/11/2011		Eyes	Normal		
S250	311	JAIVAC-1	Dosage C	3	Day 365			10/11/2011		Ears, nose and throat	Normal		
S250	311	JAIVAC-1	Dosage C	3	Day 365			10/11/2011		Cardiovascular	Normal		
S250	311	JAIVAC-1	Dosage C	3	Day 365			10/11/2011		Respiratory	Normal		
S250	311	JAIVAC-1	Dosage C	3	Day 365			10/11/2011		Musculoskeletal	Normal		
S250	311	JAIVAC-1	Dosage C	3	Day 365			10/11/2011		Venous system	Normal		
S250	311	JAIVAC-1	Dosage C	3	Day 365			10/11/2011		Gastrointestinal	Normal		
S250	311	JAIVAC-1	Dosage C	3	Day 365			10/11/2011		Lymphatic	Normal		
S250	311	JAIVAC-1	Dosage C	3	Day 365			10/11/2011		Neurological	Normal		
S250	311	JAIVAC-1	Dosage C	3	Day 365			10/11/2011		Genitourinary	Normal		
S250	311	JAIVAC-1	Dosage C	3	Day 365			10/11/2011		Dermatological	Normal		
S263	313	Hepatitis B	Hepatitis B		Day -14 to Day -1			09/11/2010		Head and neck	Normal		
S263	313	Hepatitis B	Hepatitis B		Day -14 to Day -1			09/11/2010		Eyes	Normal		
S263	313	Hepatitis B	Hepatitis B		Day -14 to Day -1			09/11/2010		Ears, nose and throat	Normal		
S263	313	Hepatitis B	Hepatitis B		Day -14 to Day -1			09/11/2010		Cardiovascular	Normal		
S263	313	Hepatitis B	Hepatitis B		Day -14 to Day -1			09/11/2010		Respiratory	Normal		
S263	313	Hepatitis B	Hepatitis B		Day -14 to Day -1			09/11/2010		Musculoskeletal	Normal		
S263	313	Hepatitis B	Hepatitis B		Day -14 to Day -1			09/11/2010		Venous system	Normal		

Subject No.	Randomisation Number	Study Group	Dosage	Dose	Day	Not Done	Specify	Date of Assessment	*Was the assessment performed prior to vaccination?	Physical Examination Parameter	Result	Abnormality	Clinical Significance
S263	313	Hepatitis B	Hepatitis B		Day -14 to Day -1			09/11/2010		Gastrointestinal	Normal		
S263	313	Hepatitis B	Hepatitis B		Day -14 to Day -1			09/11/2010		Lymphatic	Normal		
S263	313	Hepatitis B	Hepatitis B		Day -14 to Day -1			09/11/2010		Neurological	Normal		
S263	313	Hepatitis B	Hepatitis B		Day -14 to Day -1			09/11/2010		Genitourinary	Normal		
S263	313	Hepatitis B	Hepatitis B		Day -14 to Day -1			09/11/2010		Dermatological	Normal		
S263	313	Hepatitis B	Hepatitis B	1	Day 0			12/11/2010	Yes	Head and neck	Normal		
S263	313	Hepatitis B	Hepatitis B	1	Day 0			12/11/2010	Yes	Eyes	Normal		
S263	313	Hepatitis B	Hepatitis B	1	Day 0			12/11/2010	Yes	Ears, nose and throat	Normal		
S263	313	Hepatitis B	Hepatitis B	1	Day 0			12/11/2010	Yes	Cardiovascular	Normal		
S263	313	Hepatitis B	Hepatitis B	1	Day 0			12/11/2010	Yes	Respiratory	Normal		
S263	313	Hepatitis B	Hepatitis B	1	Day 0			12/11/2010	Yes	Musculoskeletal	Normal		
S263	313	Hepatitis B	Hepatitis B	1	Day 0			12/11/2010	Yes	Venous system	Normal		
S263	313	Hepatitis B	Hepatitis B	1	Day 0			12/11/2010	Yes	Gastrointestinal	Normal		
S263	313	Hepatitis B	Hepatitis B	1	Day 0			12/11/2010	Yes	Lymphatic	Normal		
S263	313	Hepatitis B	Hepatitis B	1	Day 0			12/11/2010	Yes	Neurological	Normal		
S263	313	Hepatitis B	Hepatitis B	1	Day 0			12/11/2010	Yes	Genitourinary	Normal		
S263	313	Hepatitis B	Hepatitis B	1	Day 0			12/11/2010	Yes	Dermatological	Normal		
S263	313	Hepatitis B	Hepatitis B	1	Day 7			19/11/2010		Head and Neck	Normal		

Subject No.	Randomisation Number	Study Group	Dosage	Dose	Day	Not Done	Specify	Date of Assessment	*Was the assessment performed prior to vaccination?	Physical Examination Parameter	Result	Abnormality	Clinical Significance
S263	313	Hepatitis B	Hepatitis B	1	Day 7			19/11/2010		Eyes	Normal		
S263	313	Hepatitis B	Hepatitis B	1	Day 7			19/11/2010		Ears, nose and throat	Normal		
S263	313	Hepatitis B	Hepatitis B	1	Day 7			19/11/2010		Cardiovascular	Normal		
S263	313	Hepatitis B	Hepatitis B	1	Day 7			19/11/2010		Respiratory	Normal		
S263	313	Hepatitis B	Hepatitis B	1	Day 7			19/11/2010		Musculoskeletal	Normal		
S263	313	Hepatitis B	Hepatitis B	1	Day 7			19/11/2010		Venous system	Normal		
S263	313	Hepatitis B	Hepatitis B	1	Day 7			19/11/2010		Gastrointestinal	Normal		
S263	313	Hepatitis B	Hepatitis B	1	Day 7			19/11/2010		Lymphatic	Normal		
S263	313	Hepatitis B	Hepatitis B	1	Day 7			19/11/2010		Neurological	Normal		
S263	313	Hepatitis B	Hepatitis B	1	Day 7			19/11/2010		Genitourinary	Normal		
S263	313	Hepatitis B	Hepatitis B	1	Day 7			19/11/2010		Dermatological	Normal		
S263	313	Hepatitis B	Hepatitis B	1	Day 14			26/11/2010		Head and Neck	Normal		
S263	313	Hepatitis B	Hepatitis B	1	Day 14			26/11/2010		Eyes	Normal		
S263	313	Hepatitis B	Hepatitis B	1	Day 14			26/11/2010		Ears, nose and throat	Normal		
S263	313	Hepatitis B	Hepatitis B	1	Day 14			26/11/2010		Cardiovascular	Normal		
S263	313	Hepatitis B	Hepatitis B	1	Day 14			26/11/2010		Respiratory	Normal		
S263	313	Hepatitis B	Hepatitis B	1	Day 14			26/11/2010		Musculoskeletal	Normal		
S263	313	Hepatitis B	Hepatitis B	1	Day 14			26/11/2010		Venous system	Normal		
S263	313	Hepatitis B	Hepatitis B	1	Day 14			26/11/2010		Gastrointestinal	Normal		

Subject No.	Randomisation Number	Study Group	Dosage	Dose	Day	Not Done	Specify	Date of Assessment	*Was the assessment performed prior to vaccination?	Physical Examination Parameter	Result	Abnormality	Clinical Significance
S263	313	Hepatitis B	Hepatitis B	1	Day 14			26/11/2010		Lymphatic	Normal		
S263	313	Hepatitis B	Hepatitis B	1	Day 14			26/11/2010		Neurological	Normal		
S263	313	Hepatitis B	Hepatitis B	1	Day 14			26/11/2010		Genitourinary	Normal		
S263	313	Hepatitis B	Hepatitis B	1	Day 14			26/11/2010		Dermatological	Normal		
S263	313	Hepatitis B	Hepatitis B	2	Day 28			10/12/2010	Yes	Head and Neck	Normal		
S263	313	Hepatitis B	Hepatitis B	2	Day 28			10/12/2010	Yes	Eyes	Normal		
S263	313	Hepatitis B	Hepatitis B	2	Day 28			10/12/2010	Yes	Ears, nose and throat	Normal		
S263	313	Hepatitis B	Hepatitis B	2	Day 28			10/12/2010	Yes	Cardiovascular	Normal		
S263	313	Hepatitis B	Hepatitis B	2	Day 28			10/12/2010	Yes	Respiratory	Normal		
S263	313	Hepatitis B	Hepatitis B	2	Day 28			10/12/2010	Yes	Musculoskeletal	Normal		
S263	313	Hepatitis B	Hepatitis B	2	Day 28			10/12/2010	Yes	Venous system	Normal		
S263	313	Hepatitis B	Hepatitis B	2	Day 28			10/12/2010	Yes	Gastrointestinal	Normal		
S263	313	Hepatitis B	Hepatitis B	2	Day 28			10/12/2010	Yes	Lymphatic	Normal		
S263	313	Hepatitis B	Hepatitis B	2	Day 28			10/12/2010	Yes	Neurological	Normal		
S263	313	Hepatitis B	Hepatitis B	2	Day 28			10/12/2010	Yes	Genitourinary	Normal		
S263	313	Hepatitis B	Hepatitis B	2	Day 28			10/12/2010	Yes	Dermatological	Normal		
S263	313	Hepatitis B	Hepatitis B	2	Day 35			17/12/2010		Head and Neck	Normal		
S263	313	Hepatitis B	Hepatitis B	2	Day 35			17/12/2010		Eyes	Normal		
S263	313	Hepatitis B	Hepatitis B	2	Day 35			17/12/2010		Ears, nose and throat	Normal		

Subject No.	Randomisation Number	Study Group	Dosage	Dose	Day	Not Done	Specify	Date of Assessment	*Was the assessment performed prior to vaccination?	Physical Examination Parameter	Result	Abnormality	Clinical Significance
S263	313	Hepatitis B	Hepatitis B	2	Day 35			17/12/2010		Cardiovascular	Normal		
S263	313	Hepatitis B	Hepatitis B	2	Day 35			17/12/2010		Respiratory	Normal		
S263	313	Hepatitis B	Hepatitis B	2	Day 35			17/12/2010		Musculoskeletal	Normal		
S263	313	Hepatitis B	Hepatitis B	2	Day 35			17/12/2010		Venous system	Normal		
S263	313	Hepatitis B	Hepatitis B	2	Day 35			17/12/2010		Gastrointestinal	Normal		
S263	313	Hepatitis B	Hepatitis B	2	Day 35			17/12/2010		Lymphatic	Normal		
S263	313	Hepatitis B	Hepatitis B	2	Day 35			17/12/2010		Neurological	Normal		
S263	313	Hepatitis B	Hepatitis B	2	Day 35			17/12/2010		Genitourinary	Normal		
S263	313	Hepatitis B	Hepatitis B	2	Day 35			17/12/2010		Dermatological	Normal		
S263	313	Hepatitis B	Hepatitis B	2	Day 42			24/12/2010		Head and Neck	Normal		
S263	313	Hepatitis B	Hepatitis B	2	Day 42			24/12/2010		Eyes	Normal		
S263	313	Hepatitis B	Hepatitis B	2	Day 42			24/12/2010		Ears, nose and throat	Normal		
S263	313	Hepatitis B	Hepatitis B	2	Day 42			24/12/2010		Cardiovascular	Normal		
S263	313	Hepatitis B	Hepatitis B	2	Day 42			24/12/2010		Respiratory	Normal		
S263	313	Hepatitis B	Hepatitis B	2	Day 42			24/12/2010		Musculoskeletal	Normal		
S263	313	Hepatitis B	Hepatitis B	2	Day 42			24/12/2010		Venous system	Normal		
S263	313	Hepatitis B	Hepatitis B	2	Day 42			24/12/2010		Gastrointestinal	Normal		
S263	313	Hepatitis B	Hepatitis B	2	Day 42			24/12/2010		Lymphatic	Normal		
S263	313	Hepatitis B	Hepatitis B	2	Day 42			24/12/2010		Neurological	Normal		

Subject No.	Randomisation Number	Study Group	Dosage	Dose	Day	Not Done	Specify	Date of Assessment	*Was the assessment performed prior to vaccination?	Physical Examination Parameter	Result	Abnormality	Clinical Significance
S263	313	Hepatitis B	Hepatitis B	2	Day 42			24/12/2010		Genitourinary	Normal		
S263	313	Hepatitis B	Hepatitis B	2	Day 42			24/12/2010		Dermatological	Normal		
S263	313	Hepatitis B	Hepatitis B	2	Day 56			07/01/2011		Head and Neck	Normal		
S263	313	Hepatitis B	Hepatitis B	2	Day 56			07/01/2011		Eyes	Normal		
S263	313	Hepatitis B	Hepatitis B	2	Day 56			07/01/2011		Ears, nose and throat	Normal		
S263	313	Hepatitis B	Hepatitis B	2	Day 56			07/01/2011		Cardiovascular	Normal		
S263	313	Hepatitis B	Hepatitis B	2	Day 56			07/01/2011		Respiratory	Normal		
S263	313	Hepatitis B	Hepatitis B	2	Day 56			07/01/2011		Musculoskeletal	Normal		
S263	313	Hepatitis B	Hepatitis B	2	Day 56			07/01/2011		Venous system	Normal		
S263	313	Hepatitis B	Hepatitis B	2	Day 56			07/01/2011		Gastrointestinal	Normal		
S263	313	Hepatitis B	Hepatitis B	2	Day 56			07/01/2011		Lymphatic	Normal		
S263	313	Hepatitis B	Hepatitis B	2	Day 56			07/01/2011		Neurological	Normal		
S263	313	Hepatitis B	Hepatitis B	2	Day 56			07/01/2011		Genitourinary	Normal		
S263	313	Hepatitis B	Hepatitis B	2	Day 56			07/01/2011		Dermatological	Normal		
S263	313	Hepatitis B	Hepatitis B	3	Day 180			11/05/2011	Yes	Head and Neck	Normal		
S263	313	Hepatitis B	Hepatitis B	3	Day 180			11/05/2011	Yes	Eyes	Normal		
S263	313	Hepatitis B	Hepatitis B	3	Day 180			11/05/2011	Yes	Ears, nose and throat	Normal		
S263	313	Hepatitis B	Hepatitis B	3	Day 180			11/05/2011	Yes	Cardiovascular	Normal		
S263	313	Hepatitis B	Hepatitis B	3	Day 180			11/05/2011	Yes	Respiratory	Normal		

Subject No.	Randomisation Number	Study Group	Dosage	Dose	Day	Not Done	Specify	Date of Assessment	*Was the assessment performed prior to vaccination?	Physical Examination Parameter	Result	Abnormality	Clinical Significance
S263	313	Hepatitis B	Hepatitis B	3	Day 180			11/05/2011	Yes	Musculoskeletal	Normal		
S263	313	Hepatitis B	Hepatitis B	3	Day 180			11/05/2011	Yes	Venous system	Normal		
S263	313	Hepatitis B	Hepatitis B	3	Day 180			11/05/2011	Yes	Gastrointestinal	Normal		
S263	313	Hepatitis B	Hepatitis B	3	Day 180			11/05/2011	Yes	Lymphatic	Normal		
S263	313	Hepatitis B	Hepatitis B	3	Day 180			11/05/2011	Yes	Neurological	Normal		
S263	313	Hepatitis B	Hepatitis B	3	Day 180			11/05/2011	Yes	Genitourinary	Normal		
S263	313	Hepatitis B	Hepatitis B	3	Day 180			11/05/2011	Yes	Dermatological	Normal		
S263	313	Hepatitis B	Hepatitis B	3	Day 187			18/05/2011		Head and Neck	Normal		
S263	313	Hepatitis B	Hepatitis B	3	Day 187			18/05/2011		Eyes	Normal		
S263	313	Hepatitis B	Hepatitis B	3	Day 187			18/05/2011		Ears, nose and throat	Normal		
S263	313	Hepatitis B	Hepatitis B	3	Day 187			18/05/2011		Cardiovascular	Normal		
S263	313	Hepatitis B	Hepatitis B	3	Day 187			18/05/2011		Respiratory	Normal		
S263	313	Hepatitis B	Hepatitis B	3	Day 187			18/05/2011		Musculoskeletal	Normal		
S263	313	Hepatitis B	Hepatitis B	3	Day 187			18/05/2011		Venous system	Normal		
S263	313	Hepatitis B	Hepatitis B	3	Day 187			18/05/2011		Gastrointestinal	Normal		
S263	313	Hepatitis B	Hepatitis B	3	Day 187			18/05/2011		Lymphatic	Normal		
S263	313	Hepatitis B	Hepatitis B	3	Day 187			18/05/2011		Neurological	Normal		
S263	313	Hepatitis B	Hepatitis B	3	Day 187			18/05/2011		Genitourinary	Normal		
S263	313	Hepatitis B	Hepatitis B	3	Day 187			18/05/2011		Dermatological	Normal		

Subject No.	Randomisation Number	Study Group	Dosage	Dose	Day	Not Done	Specify	Date of Assessment	*Was the assessment performed prior to vaccination?	Physical Examination Parameter	Result	Abnormality	Clinical Significance
S263	313	Hepatitis B	Hepatitis B	3	Day 194			25/05/2011		Head and Neck	Normal		
S263	313	Hepatitis B	Hepatitis B	3	Day 194			25/05/2011		Eyes	Normal		
S263	313	Hepatitis B	Hepatitis B	3	Day 194			25/05/2011		Ears, nose and throat	Normal		
S263	313	Hepatitis B	Hepatitis B	3	Day 194			25/05/2011		Cardiovascular	Normal		
S263	313	Hepatitis B	Hepatitis B	3	Day 194			25/05/2011		Respiratory	Normal		
S263	313	Hepatitis B	Hepatitis B	3	Day 194			25/05/2011		Musculoskeletal	Normal		
S263	313	Hepatitis B	Hepatitis B	3	Day 194			25/05/2011		Venous system	Normal		
S263	313	Hepatitis B	Hepatitis B	3	Day 194			25/05/2011		Gastrointestinal	Normal		
S263	313	Hepatitis B	Hepatitis B	3	Day 194			25/05/2011		Lymphatic	Normal		
S263	313	Hepatitis B	Hepatitis B	3	Day 194			25/05/2011		Neurological	Normal		
S263	313	Hepatitis B	Hepatitis B	3	Day 194			25/05/2011		Genitourinary	Normal		
S263	313	Hepatitis B	Hepatitis B	3	Day 194			25/05/2011		Dermatological	Normal		
S263	313	Hepatitis B	Hepatitis B	3	Day 208			08/06/2011		Head and Neck	Normal		
S263	313	Hepatitis B	Hepatitis B	3	Day 208			08/06/2011		Eyes	Normal		
S263	313	Hepatitis B	Hepatitis B	3	Day 208			08/06/2011		Ears, nose and throat	Normal		
S263	313	Hepatitis B	Hepatitis B	3	Day 208			08/06/2011		Cardiovascular	Normal		
S263	313	Hepatitis B	Hepatitis B	3	Day 208			08/06/2011		Respiratory	Normal		
S263	313	Hepatitis B	Hepatitis B	3	Day 208			08/06/2011		Musculoskeletal	Normal		
S263	313	Hepatitis B	Hepatitis B	3	Day 208			08/06/2011		Venous system	Normal		

Subject No.	Randomisation Number	Study Group	Dosage	Dose	Day	Not Done	Specify	Date of Assessment	*Was the assessment performed prior to vaccination?	Physical Examination Parameter	Result	Abnormality	Clinical Significance
S263	313	Hepatitis B	Hepatitis B	3	Day 208			08/06/2011		Gastrointestinal	Normal		
S263	313	Hepatitis B	Hepatitis B	3	Day 208			08/06/2011		Lymphatic	Normal		
S263	313	Hepatitis B	Hepatitis B	3	Day 208			08/06/2011		Neurological	Normal		
S263	313	Hepatitis B	Hepatitis B	3	Day 208			08/06/2011		Genitourinary	Normal		
S263	313	Hepatitis B	Hepatitis B	3	Day 208			08/06/2011		Dermatological	Normal		
S263	313	Hepatitis B	Hepatitis B	3	Day 365			10/11/2011		Head and Neck	Normal		
S263	313	Hepatitis B	Hepatitis B	3	Day 365			10/11/2011		Eyes	Normal		
S263	313	Hepatitis B	Hepatitis B	3	Day 365			10/11/2011		Ears, nose and throat	Normal		
S263	313	Hepatitis B	Hepatitis B	3	Day 365			10/11/2011		Cardiovascular	Normal		
S263	313	Hepatitis B	Hepatitis B	3	Day 365			10/11/2011		Respiratory	Normal		
S263	313	Hepatitis B	Hepatitis B	3	Day 365			10/11/2011		Musculoskeletal	Normal		
S263	313	Hepatitis B	Hepatitis B	3	Day 365			10/11/2011		Venous system	Normal		
S263	313	Hepatitis B	Hepatitis B	3	Day 365			10/11/2011		Gastrointestinal	Normal		
S263	313	Hepatitis B	Hepatitis B	3	Day 365			10/11/2011		Lymphatic	Normal		
S263	313	Hepatitis B	Hepatitis B	3	Day 365			10/11/2011		Neurological	Normal		
S263	313	Hepatitis B	Hepatitis B	3	Day 365			10/11/2011		Genitourinary	Normal		
S263	313	Hepatitis B	Hepatitis B	3	Day 365			10/11/2011		Dermatological	Normal		
S263	313	Hepatitis B	Hepatitis B		Unscheduled Visit	Yes	Laboratory Test Done	.		Head and Neck			
S263	313	Hepatitis B	Hepatitis B		Unscheduled Visit	Yes	Laboratory Test Done	.		Eyes			

Subject No.	Randomisation Number	Study Group	Dosage	Dose	Day	Not Done	Specify	Date of Assessment	*Was the assessment performed prior to vaccination?	Physical Examination Parameter	Result	Abnormality	Clinical Significance
S263	313	Hepatitis B	Hepatitis B		Unscheduled Visit	Yes	Laboratory Test Done	.		Ears, nose and throat			
S263	313	Hepatitis B	Hepatitis B		Unscheduled Visit	Yes	Laboratory Test Done	.		Cardiovascular			
S263	313	Hepatitis B	Hepatitis B		Unscheduled Visit	Yes	Laboratory Test Done	.		Respiratory			
S263	313	Hepatitis B	Hepatitis B		Unscheduled Visit	Yes	Laboratory Test Done	.		Musculoskeletal			
S263	313	Hepatitis B	Hepatitis B		Unscheduled Visit	Yes	Laboratory Test Done	.		Venous system			
S263	313	Hepatitis B	Hepatitis B		Unscheduled Visit	Yes	Laboratory Test Done	.		Gastrointestinal			
S263	313	Hepatitis B	Hepatitis B		Unscheduled Visit	Yes	Laboratory Test Done	.		Lymphatic			
S263	313	Hepatitis B	Hepatitis B		Unscheduled Visit	Yes	Laboratory Test Done	.		Neurological			
S263	313	Hepatitis B	Hepatitis B		Unscheduled Visit	Yes	Laboratory Test Done	.		Genitourinary			
S263	313	Hepatitis B	Hepatitis B		Unscheduled Visit	Yes	Laboratory Test Done	.		Dermatological			
S264	306	JAIVAC-1	Dosage C		Day -14 to Day -1			03/11/2010		Head and neck	Normal		
S264	306	JAIVAC-1	Dosage C		Day -14 to Day -1			03/11/2010		Eyes	Normal		
S264	306	JAIVAC-1	Dosage C		Day -14 to Day -1			03/11/2010		Ears, nose and throat	Normal		
S264	306	JAIVAC-1	Dosage C		Day -14 to Day -1			03/11/2010		Cardiovascular	Normal		
S264	306	JAIVAC-1	Dosage C		Day -14 to Day -1			03/11/2010		Respiratory	Normal		
S264	306	JAIVAC-1	Dosage C		Day -14 to Day -1			03/11/2010		Musculoskeletal	Normal		

Subject No.	Randomisation Number	Study Group	Dosage	Dose	Day	Not Done	Specify	Date of Assessment	*Was the assessment performed prior to vaccination?	Physical Examination Parameter	Result	Abnormality	Clinical Significance
S264	306	JAIVAC-1	Dosage C		Day -14 to Day -1			03/11/2010		Venous system	Normal		
S264	306	JAIVAC-1	Dosage C		Day -14 to Day -1			03/11/2010		Gastrointestinal	Normal		
S264	306	JAIVAC-1	Dosage C		Day -14 to Day -1			03/11/2010		Lymphatic	Normal		
S264	306	JAIVAC-1	Dosage C		Day -14 to Day -1			03/11/2010		Neurological	Normal		
S264	306	JAIVAC-1	Dosage C		Day -14 to Day -1			03/11/2010		Genitourinary	Normal		
S264	306	JAIVAC-1	Dosage C		Day -14 to Day -1			03/11/2010		Dermatological	Normal		
S264	306	JAIVAC-1	Dosage C	1	Day 0			11/11/2010	Yes	Head and neck	Normal		
S264	306	JAIVAC-1	Dosage C	1	Day 0			11/11/2010	Yes	Eyes	Normal		
S264	306	JAIVAC-1	Dosage C	1	Day 0			11/11/2010	Yes	Ears, nose and throat	Normal		
S264	306	JAIVAC-1	Dosage C	1	Day 0			11/11/2010	Yes	Cardiovascular	Normal		
S264	306	JAIVAC-1	Dosage C	1	Day 0			11/11/2010	Yes	Respiratory	Normal		
S264	306	JAIVAC-1	Dosage C	1	Day 0			11/11/2010	Yes	Musculoskeletal	Normal		
S264	306	JAIVAC-1	Dosage C	1	Day 0			11/11/2010	Yes	Venous system	Normal		
S264	306	JAIVAC-1	Dosage C	1	Day 0			11/11/2010	Yes	Gastrointestinal	Normal		
S264	306	JAIVAC-1	Dosage C	1	Day 0			11/11/2010	Yes	Lymphatic	Normal		
S264	306	JAIVAC-1	Dosage C	1	Day 0			11/11/2010	Yes	Neurological	Normal		
S264	306	JAIVAC-1	Dosage C	1	Day 0			11/11/2010	Yes	Genitourinary	Normal		
S264	306	JAIVAC-1	Dosage C	1	Day 0			11/11/2010	Yes	Dermatological	Normal		

Subject No.	Randomisation Number	Study Group	Dosage	Dose	Day	Not Done	Specify	Date of Assessment	*Was the assessment performed prior to vaccination?	Physical Examination Parameter	Result	Abnormality	Clinical Significance
S264	306	JAIVAC-1	Dosage C	1	Day 7			18/11/2010		Head and Neck	Normal		
S264	306	JAIVAC-1	Dosage C	1	Day 7			18/11/2010		Eyes	Normal		
S264	306	JAIVAC-1	Dosage C	1	Day 7			18/11/2010		Ears, nose and throat	Normal		
S264	306	JAIVAC-1	Dosage C	1	Day 7			18/11/2010		Cardiovascular	Normal		
S264	306	JAIVAC-1	Dosage C	1	Day 7			18/11/2010		Respiratory	Normal		
S264	306	JAIVAC-1	Dosage C	1	Day 7			18/11/2010		Musculoskeletal	Normal		
S264	306	JAIVAC-1	Dosage C	1	Day 7			18/11/2010		Venous system	Normal		
S264	306	JAIVAC-1	Dosage C	1	Day 7			18/11/2010		Gastrointestinal	Normal		
S264	306	JAIVAC-1	Dosage C	1	Day 7			18/11/2010		Lymphatic	Normal		
S264	306	JAIVAC-1	Dosage C	1	Day 7			18/11/2010		Neurological	Normal		
S264	306	JAIVAC-1	Dosage C	1	Day 7			18/11/2010		Genitourinary	Normal		
S264	306	JAIVAC-1	Dosage C	1	Day 7			18/11/2010		Dermatological	Normal		
S264	306	JAIVAC-1	Dosage C	1	Day 14			25/11/2010		Head and Neck	Normal		
S264	306	JAIVAC-1	Dosage C	1	Day 14			25/11/2010		Eyes	Normal		
S264	306	JAIVAC-1	Dosage C	1	Day 14			25/11/2010		Ears, nose and throat	Normal		
S264	306	JAIVAC-1	Dosage C	1	Day 14			25/11/2010		Cardiovascular	Normal		
S264	306	JAIVAC-1	Dosage C	1	Day 14			25/11/2010		Respiratory	Normal		
S264	306	JAIVAC-1	Dosage C	1	Day 14			25/11/2010		Musculoskeletal	Normal		
S264	306	JAIVAC-1	Dosage C	1	Day 14			25/11/2010		Venous system	Normal		

Subject No.	Randomisation Number	Study Group	Dosage	Dose	Day	Not Done	Specify	Date of Assessment	*Was the assessment performed prior to vaccination?	Physical Examination Parameter	Result	Abnormality	Clinical Significance
S264	306	JAIVAC-1	Dosage C	1	Day 14			25/11/2010		Gastrointestinal	Normal		
S264	306	JAIVAC-1	Dosage C	1	Day 14			25/11/2010		Lymphatic	Normal		
S264	306	JAIVAC-1	Dosage C	1	Day 14			25/11/2010		Neurological	Normal		
S264	306	JAIVAC-1	Dosage C	1	Day 14			25/11/2010		Genitourinary	Normal		
S264	306	JAIVAC-1	Dosage C	1	Day 14			25/11/2010		Dermatological	Normal		
S264	306	JAIVAC-1	Dosage C	2	Day 28			09/12/2010	Yes	Head and Neck	Normal		
S264	306	JAIVAC-1	Dosage C	2	Day 28			09/12/2010	Yes	Eyes	Normal		
S264	306	JAIVAC-1	Dosage C	2	Day 28			09/12/2010	Yes	Ears, nose and throat	Normal		
S264	306	JAIVAC-1	Dosage C	2	Day 28			09/12/2010	Yes	Cardiovascular	Normal		
S264	306	JAIVAC-1	Dosage C	2	Day 28			09/12/2010	Yes	Respiratory	Normal		
S264	306	JAIVAC-1	Dosage C	2	Day 28			09/12/2010	Yes	Musculoskeletal	Normal		
S264	306	JAIVAC-1	Dosage C	2	Day 28			09/12/2010	Yes	Venous system	Normal		
S264	306	JAIVAC-1	Dosage C	2	Day 28			09/12/2010	Yes	Gastrointestinal	Normal		
S264	306	JAIVAC-1	Dosage C	2	Day 28			09/12/2010	Yes	Lymphatic	Normal		
S264	306	JAIVAC-1	Dosage C	2	Day 28			09/12/2010	Yes	Neurological	Normal		
S264	306	JAIVAC-1	Dosage C	2	Day 28			09/12/2010	Yes	Genitourinary	Normal		
S264	306	JAIVAC-1	Dosage C	2	Day 28			09/12/2010	Yes	Dermatological	Normal		
S264	306	JAIVAC-1	Dosage C	2	Day 35			16/12/2010		Head and Neck	Normal		
S264	306	JAIVAC-1	Dosage C	2	Day 35			16/12/2010		Eyes	Normal		

Subject No.	Randomisation Number	Study Group	Dosage	Dose	Day	Not Done	Specify	Date of Assessment	*Was the assessment performed prior to vaccination?	Physical Examination Parameter	Result	Abnormality	Clinical Significance
S264	306	JAIVAC-1	Dosage C	2	Day 35			16/12/2010		Ears, nose and throat	Normal		
S264	306	JAIVAC-1	Dosage C	2	Day 35			16/12/2010		Cardiovascular	Normal		
S264	306	JAIVAC-1	Dosage C	2	Day 35			16/12/2010		Respiratory	Normal		
S264	306	JAIVAC-1	Dosage C	2	Day 35			16/12/2010		Musculoskeletal	Normal		
S264	306	JAIVAC-1	Dosage C	2	Day 35			16/12/2010		Venous system	Normal		
S264	306	JAIVAC-1	Dosage C	2	Day 35			16/12/2010		Gastrointestinal	Normal		
S264	306	JAIVAC-1	Dosage C	2	Day 35			16/12/2010		Lymphatic	Normal		
S264	306	JAIVAC-1	Dosage C	2	Day 35			16/12/2010		Neurological	Normal		
S264	306	JAIVAC-1	Dosage C	2	Day 35			16/12/2010		Genitourinary	Normal		
S264	306	JAIVAC-1	Dosage C	2	Day 35			16/12/2010		Dermatological	Normal		
S264	306	JAIVAC-1	Dosage C	2	Day 42			23/12/2010		Head and Neck	Normal		
S264	306	JAIVAC-1	Dosage C	2	Day 42			23/12/2010		Eyes	Normal		
S264	306	JAIVAC-1	Dosage C	2	Day 42			23/12/2010		Ears, nose and throat	Normal		
S264	306	JAIVAC-1	Dosage C	2	Day 42			23/12/2010		Cardiovascular	Normal		
S264	306	JAIVAC-1	Dosage C	2	Day 42			23/12/2010		Respiratory	Normal		
S264	306	JAIVAC-1	Dosage C	2	Day 42			23/12/2010		Musculoskeletal	Normal		
S264	306	JAIVAC-1	Dosage C	2	Day 42			23/12/2010		Venous system	Normal		
S264	306	JAIVAC-1	Dosage C	2	Day 42			23/12/2010		Gastrointestinal	Normal		
S264	306	JAIVAC-1	Dosage C	2	Day 42			23/12/2010		Lymphatic	Normal		

Subject No.	Randomisation Number	Study Group	Dosage	Dose	Day	Not Done	Specify	Date of Assessment	*Was the assessment performed prior to vaccination?	Physical Examination Parameter	Result	Abnormality	Clinical Significance
S264	306	JAIVAC-1	Dosage C	2	Day 42			23/12/2010		Neurological	Normal		
S264	306	JAIVAC-1	Dosage C	2	Day 42			23/12/2010		Genitourinary	Normal		
S264	306	JAIVAC-1	Dosage C	2	Day 42			23/12/2010		Dermatological	Normal		
S264	306	JAIVAC-1	Dosage C	2	Day 56			06/01/2011		Head and Neck	Normal		
S264	306	JAIVAC-1	Dosage C	2	Day 56			06/01/2011		Eyes	Normal		
S264	306	JAIVAC-1	Dosage C	2	Day 56			06/01/2011		Ears, nose and throat	Normal		
S264	306	JAIVAC-1	Dosage C	2	Day 56			06/01/2011		Cardiovascular	Normal		
S264	306	JAIVAC-1	Dosage C	2	Day 56			06/01/2011		Respiratory	Normal		
S264	306	JAIVAC-1	Dosage C	2	Day 56			06/01/2011		Musculoskeletal	Normal		
S264	306	JAIVAC-1	Dosage C	2	Day 56			06/01/2011		Venous system	Normal		
S264	306	JAIVAC-1	Dosage C	2	Day 56			06/01/2011		Gastrointestinal	Normal		
S264	306	JAIVAC-1	Dosage C	2	Day 56			06/01/2011		Lymphatic	Normal		
S264	306	JAIVAC-1	Dosage C	2	Day 56			06/01/2011		Neurological	Normal		
S264	306	JAIVAC-1	Dosage C	2	Day 56			06/01/2011		Genitourinary	Normal		
S264	306	JAIVAC-1	Dosage C	2	Day 56			06/01/2011		Dermatological	Normal		
S264	306	JAIVAC-1	Dosage C	3	Day 180			10/05/2011	Yes	Head and Neck	Normal		
S264	306	JAIVAC-1	Dosage C	3	Day 180			10/05/2011	Yes	Eyes	Normal		
[truncated: 385,280 more chars]
